# Supplementary material for: Cohesin forms fountains at active enhancers in C. elegans
Source: Nat Commun. 2025 Dec 11;17:681. doi: 10.1038/s41467-025-67302-6 (PMC12820367; doi:10.1038/s41467-025-67302-6)
Supplement: Supplementary file 1 — Supplementary Information [file 41467_2025_67302_MOESM1_ESM.pdf]

Supplementary information

Supplementary tables

Supplementary Table 1: ***C. elegans*** **strains**

Strain name	Genotype
PMW366	*ubsSi20[hsp-16.2p::TEV::unc-54 3'UTR; Cbr-unc-119(+)] II; unc-119(ed3) III*
PMW828	*ubsSi20[hsp-16.2p::TEV] II; unc-119(?) III; coh-1(ubs26[1xFLAG TEVcs]) X*
PMW836	*scc-1(ubs19[1xFLAG TEVcs]) ubsSi20[hsp-16.2p::TEV] II; unc-119(?) III; wIs54[scm::gfp] V*
PMW844	*scc-1(ubs19[1xFLAG TEVcs]) ubsSi20[hsp-16.2p::TEV] II; unc-119(?) III; coh-1(ubs26[1xFLAG TEVcs]) X*
PMW1116	*coh-1(ubs26[TEVcs]) X;*<br>*ubsSi20[hsp-16.2p::TEV::unc-54 3'UTR; Cbunc-119] II*<br>*ujIs113 II; skn-1(hq82([skn-1::gfp]) IV*
PMW1117	*coh-1(ubs26[TEVcs]) X; ujIs113 II; skn-1(hq82([skn-1::gfp]) IV*
PMW1063	*ubsSi20[hsp-16.2p::TEV::unc-54 3'UTR; Cbunc-119]II; xeSi301[Peft-3::luc::gfp::unc-54 3'UTR, unc-119(+)]III; coh-1(ubs26[TEVcs])X*
PMW1064	*ubsSi20[hsp-16.2p::TEV::unc-54 3'UTR; Cbunc-119]II; xeSi301[Peft-3::luc::gfp::unc-54 3'UTR, unc-119(+)]III*
PMW1182	*ubsSi20[hsp-16.2p::TEV::unc-54 3'UTR; Cbunc-119] II;*<br>*skn-1(hq82(skn-1::gfp]) ubs62(bec-1 enhancer long deletion) IV;*<br>*coh-1(ubs26[TEVcs]) X.*
PMW1217	*ubsSi20[hsp-16.2p::TEV::unc-54 3'UTR; Cbunc-119] II; skn-1(hq82([skn-1::gfp]) nhr-46(ubs71[deletion]) IV; coh-1(ubs26[TEVcs]) X*

PMW1218	*ubsSi20[hsp-16.2p::TEV::unc-54 3'UTR; Cbunc-119] II; skn-1(hq82([skn-1::gfp]) clec-178(ubs72[deletion]) IV; coh-1(ubs26[TEVcs]) X*
PMW1219	*ubsSi20[hsp-16.2p::TEV::unc-54 3'UTR; Cbunc-119] II; skn-1(hq82([skn-1::gfp] ubs73[very large intron deletion]) IV ; coh-1(ubs26[TEVcs]) X*

All primers and guides were purchased from IDT.

Supplementary Table 2: *skn-1* enhancer deletion location (ce11)

Deletion	Chromosome number	Start	End
ubs62 deletion	chrIV	5665244	5667587
ubs71 deletion	chrIV	5747318	5766196
ubs72 deletion	chrIV	5654185	5655138
ubs73 deletion	chrIV	5655970	5659745

Supplementary Table 3: Guide RNAs/primers used for deletion

Deletion	Guide RNA 1	Guide RNA 2	Stitching primer
ubs62 deletion	ttaaaaaatgtataacagca	tAATCAATAAAACATTGTGG	ATGAATAACTAATGCACGTCTCTTGTCTGTACCCTCCTCAATTTGCACgCtgctgttatacattttttaattaaaaattaatgtcaaaatatagaa
ubs71 deletion	tttttGAAACGAAGTCACAC	ACTGGTTGTTTTTTATTACG	tttAGGGCTATGTTGCAAACTTAGTTAGCTTGCCAATTTTATCGAAACCCCGTAATAAAAAACAACCAGTTTTCAAATTATGAAGAACAAGATAAT
ubs72 deletion	tagacagggacgaactcttt	aaccctgctgcagatactgt	TGTACTTACGCAATAGGTATATTCCCTAAAGTAGGATAGGTTTTACTGTCAGAAGCTTCG
ubs73 deletion	TTAACAGGGTGGAAAAAGCA	TTCAAAGTAAATATGTTACG	AAACATTTTTTCTTATTTACACCCGGTTATAGATCATTTCTCAAAGACCGTGCTTTTTCCACCCTGTTAATATTATTTTCGATATTCCCAAAAATAATTC

Supplementary Table 4: Public datasets

Data set	Larval stage	GEO Accession code	Reference
RNAP II ChIP-seq	L3	GSE188851	[24]
TOP-1 ChIP-seq	L3	GSE188851	[24]
TOP-2 ChIP-seq	L3	GSE188851	[24]
COH-1 ChIP-seq	Young adult	modENCODE project	PMID 25164756
cohesin^{COH-1} cleavage Hi-C	L3	GSE199723	[9]
Cohesin^{SCC-1} cleavage Hi-C	L3	GSE199723	[9]
TIR1 control Hi-C	L3	GSE188851	[24]
TOP-1 Hi-C	L3	GSE188851	[24]
TOP-2 Hi-C	L3	GSE188851	[24]
cohesin^{COH-1} cleavage RNA-seq	L3	GSE199723	[9]
Cohesin^{SCC-1} cleavage RNA-seq	L3	GSE199723	[9]
Enhancers	L3	GSE114494	[6]
Enhancers	L3	GSE89608	[7]
HOT regions	Late embryo	GSE49870	[16]

Supplementary figures and legends

Lüthi et al.,
Figure S1

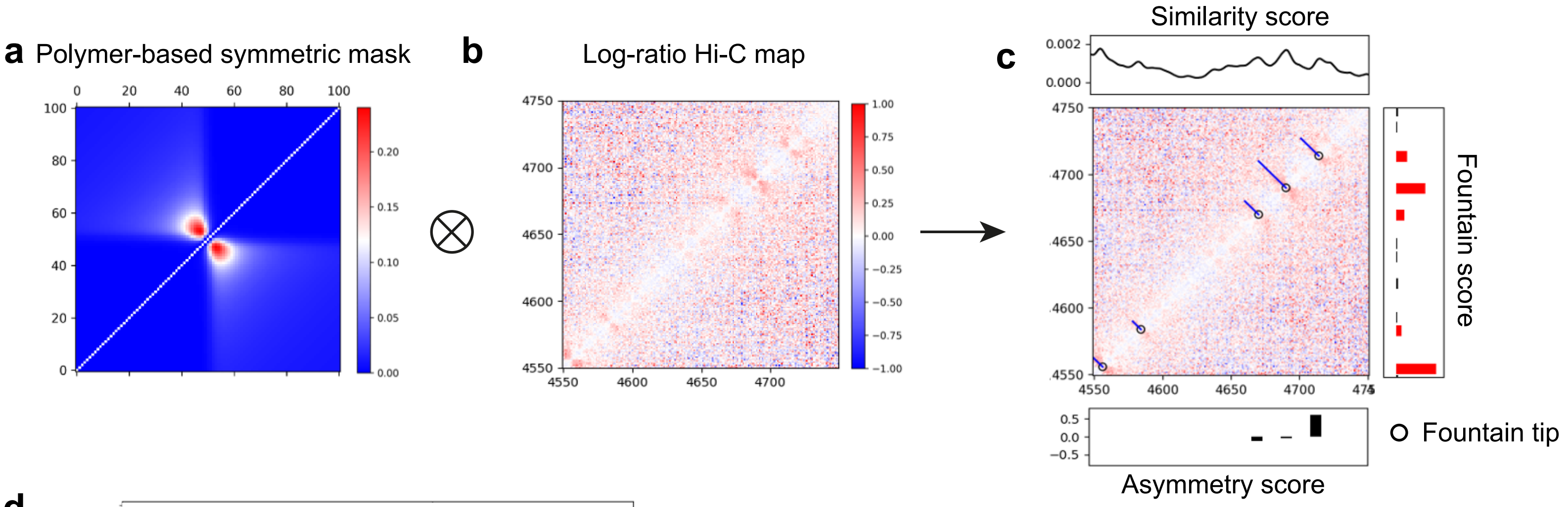

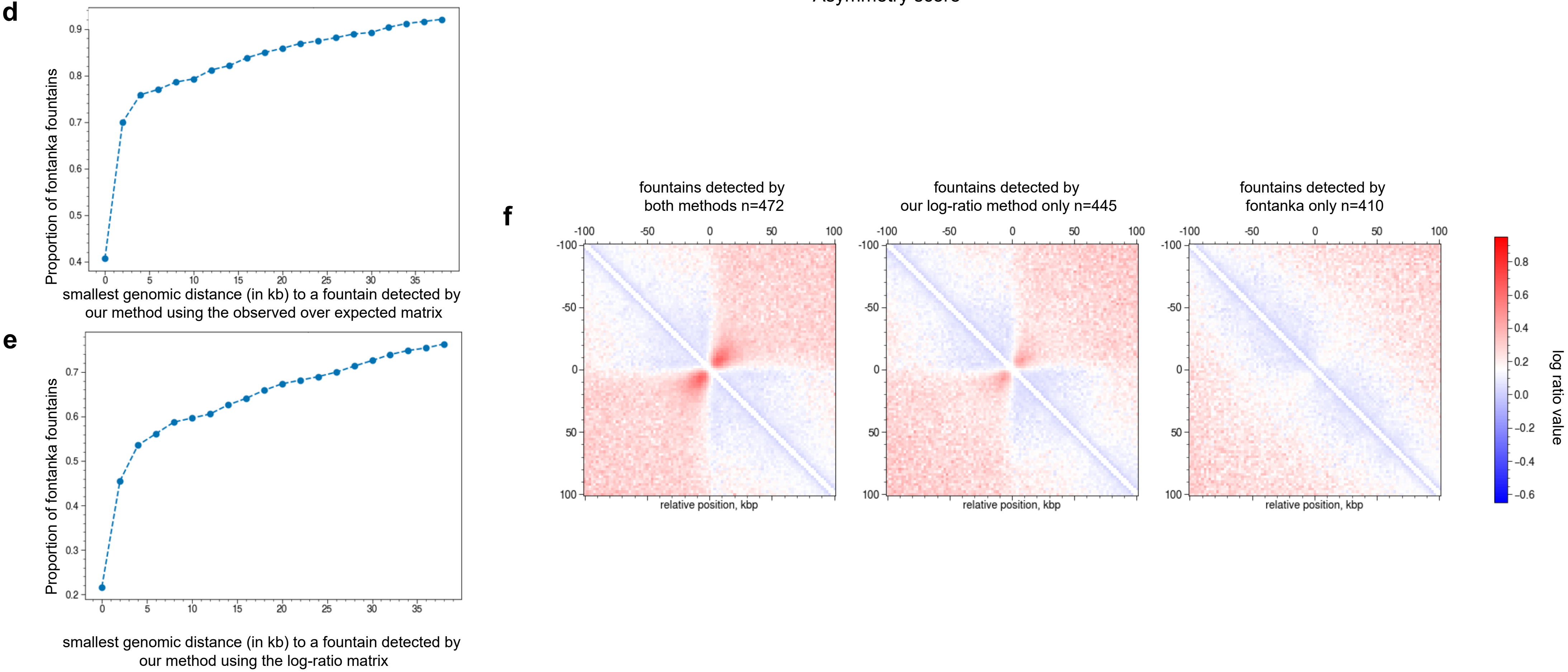

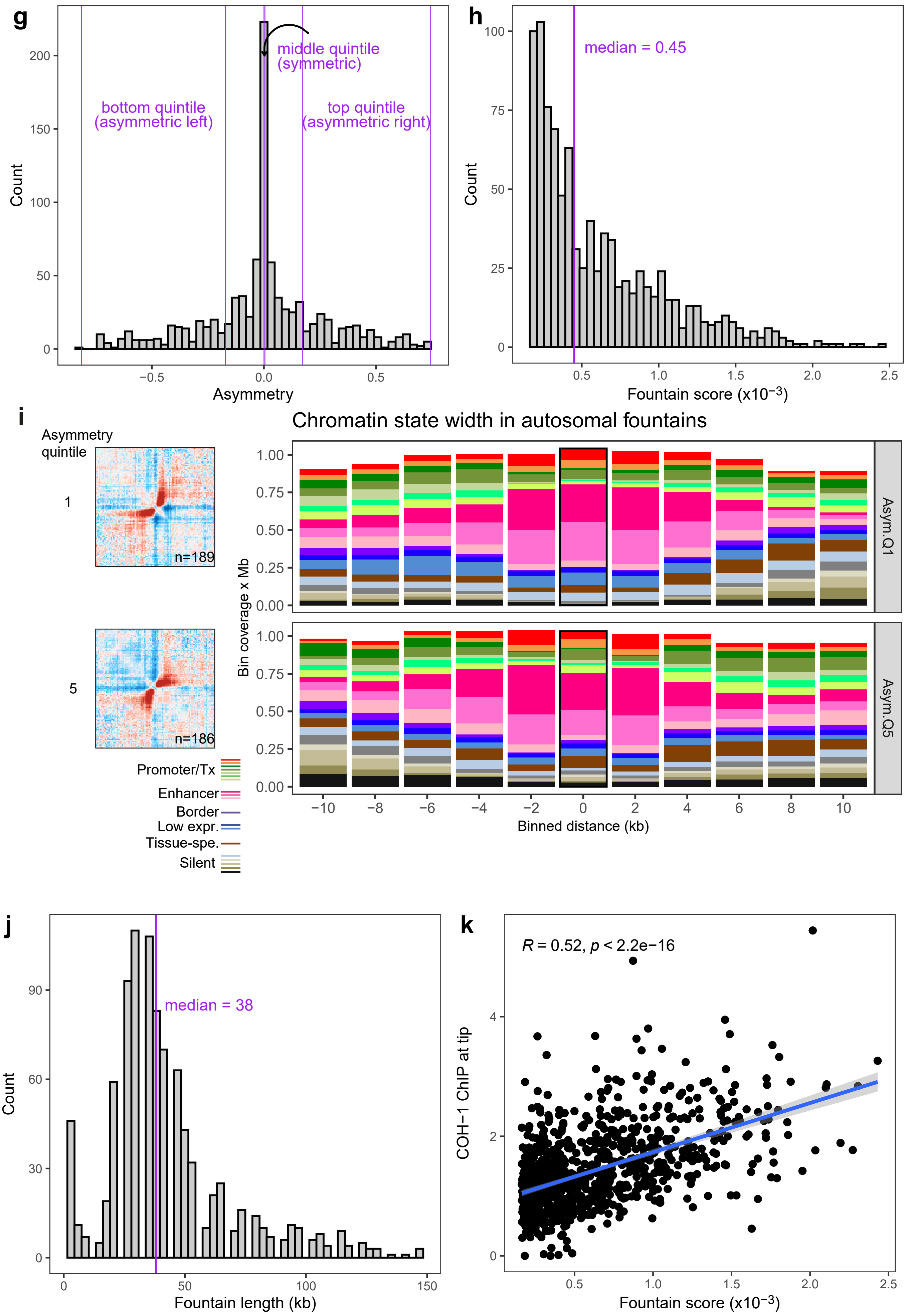
g
middle quintile
(symmetric)
bottom quintile
(asymmetric left)
top quintile
(asymmetric right)
Count
0
50
100
150
200
−0.5
0.0
0.5
Asymmetry
h
median = 0.45
Count
0
25
50
75
100
0.5
1.0
1.5
2.0
2.5
Fountain score (x10^{-3})
i
Chromatin state width in autosomal fountains
Asymmetry
quintile
1
n=189
5
n=186
Bin coverage x Mb
0.00
0.25
0.50
0.75
1.00
Asym.Q1
Asym.Q5
−10
−8
−6
−4
−2
0
2
4
6
8
10
Binned distance (kb)
Promoter/Tx
Enhancer
Border
Low expr.
Tissue-spe.
Silent
j
median = 38
Count
0
30
60
90
0
50
100
150
Fountain length (kb)
k
R = 0.52, p < 2.2e−16
COH−1 ChIP at tip
0
2
4
0.5
1.0
1.5
2.0
2.5
Fountain score (x10^{-3})

Supplementary Figure 1

Inferring fountain tip positions and asymmetries

a. A polymer-model-based mask of a symmetric fountain is convoluted along the diagonal of the **b.** log-ratio matrix (TEV control divided by cohesin-depleted) to compute a local fountain score. **c.** Maxima of the profile of fountain score (black line in top panel) along the genome with significant prominence (red bars in right panel) are assigned as fountain tips (black circles). Asymmetry in fountain shapes is measured using asymmetric binarized masks around the position of the fountain tip determined previously (<0: left-handed fountain, >0: right-handed fountain, top panel). Fountain lengths (blue segments) are estimated by considering the average value of the log-ratio matrix perpendicular to the main diagonal around fountain origin. **d.** Comparison between our fountain detection method and fontanka[15]. Fountains were detected using fontanka on the TEV control dataset using default parameters and the same symmetric polymer-model-based mask that we developed. Note that fontanka works on the observed-over-expected matrix of a single input Hi-C dataset and thus cannot be run over the log-ratio matrix (TEV control divided by cohesin^{COH-1} cleavage) that we are using to detect fountains. To allow fair comparison with our method, we therefore applied our strategy on the observed-over-expected matrix of the TEV control dataset and compared the inferred positions of fountains. **d.** Percentage of fountains detected by fontanka that are closer than the distance on the x axis from a fountain detected by our method. About 75% of fontanka-detected fountains are also detected (genomic distance less than 5 kbp) by applying our approach to the observed-over-expected TEV control Hi-C map. **e.** Comparison of fontanka-inferred fountains on observed-over-expected TEV control Hi-C map with the positions of the fountains detected by applying our method to the log-ratio matrix (TEV control over cohesin^{COH-1}-cleavage). About 55% of fontanka-detected fountains colocalized with log-ratio matrix-fountains. **f.** Average contact frequency maps of the log ratio matrix around fountains detected by both methods (left), only by our approach (center) and only by fontanka (right). Interestingly, fountains detected only by fontanka do not exhibit a clear signature over the log-ratio matrix (right) suggesting that they may represent cohesin-independent motifs, while fountains detected by our method only have a clear signature on the log-ratio matrix (center). **g.** Histogram of fountain asymmetry score. The score was divided into 5 equally sized groups with the lowest and highest quintiles corresponding to asymmetric fountains oriented left and right respectively, and the central quintile (values all around 0) representing symmetric fountains. **h.** Histogram of the fountain prominence score with the median score marked in purple. **i.** Fountain asymmetry is associated with asymmetric enhancer states. Fountain asymmetry scores were divided into five equally sized bins as in g. Left panel: average contact frequency maps in regions around

asymmetric fountains from the first and fifth quintiles. Right panel: width in bp of each chromatin states in fountain regions from the highest and lowest asymmetry quintiles (up to 10 kb upstream and downstream of fountain tips. **j.** Histogram of fountains length in kb with the median length marked in purple. **k.** Pearson correlation of the fountain prominence score with the COH-1 ChIP-seq signal measured at the 2 kb fountain tip bin.

Lüthi et al., Figure S2

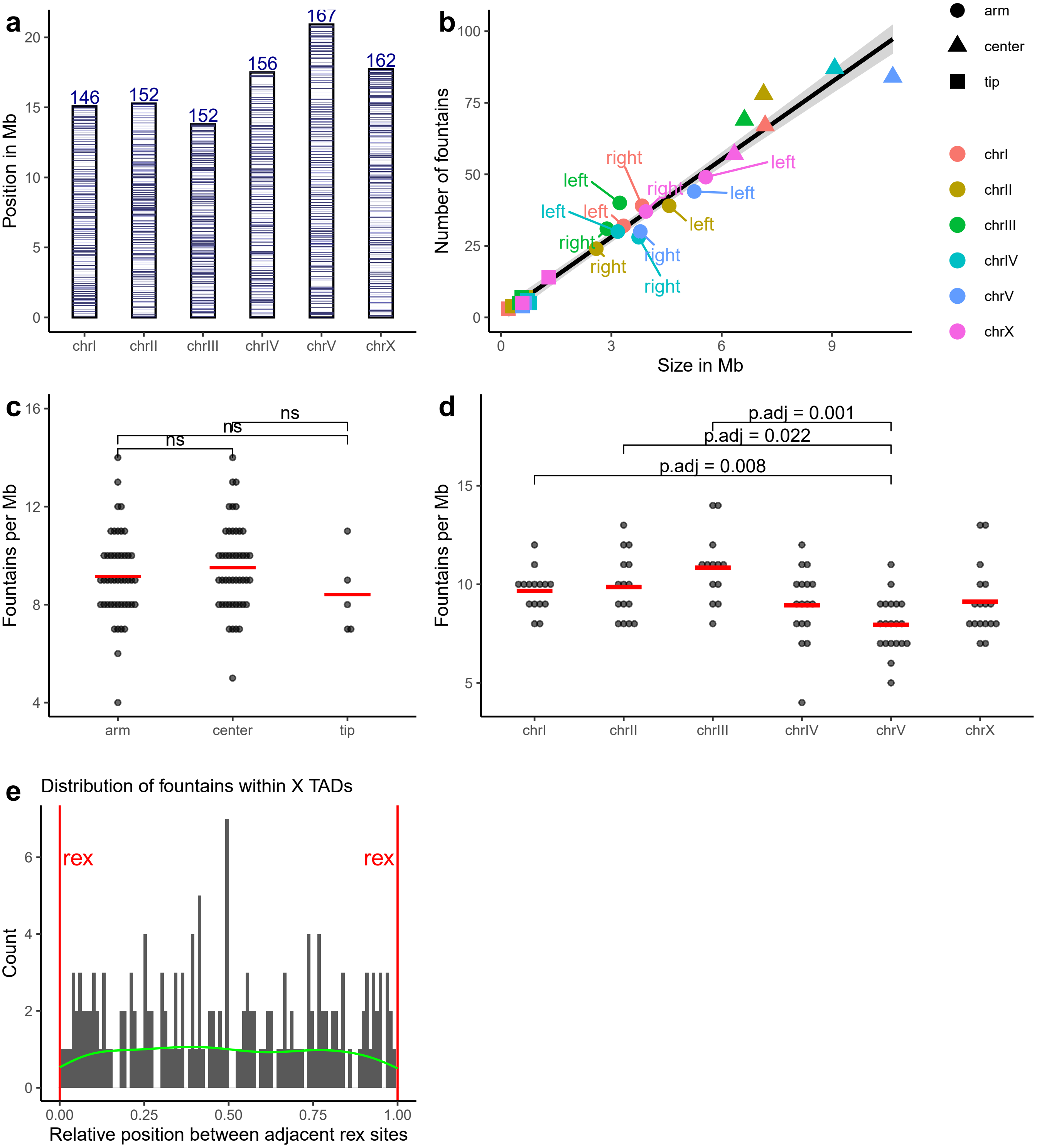

Supplementary Figure 2. Analysis of the genome-wide distribution of identified fountains.

a. Location of all the fountains plotted as horizontal blue lines (with transparency) on the chromosomes to provide a visual representation of their distribution. The total number of fountains per chromosome is indicated above the bars **b.** The number of fountains identified per chromosomal region (see PMID19283065) as a function of the region length. A fitted linear regression line is shown in black. **c. d.** Fountains per 1Mb bin plotted by region type or by chromosome. The mean number of fountains per Mb per group is shown with horizontal red lines. A two sided Wilcoxon rank sum test was used to compare the groups. For clarity, only statistically significant comparisons are indicated with their FDR adjusted p-values in d, while in c the differences were all non-significant (ns). **e.** Histogram of relative position of fountains in-between adjacent *rex* sites (red) shown in gray and their density as a green line.

Lüthi et al., Figure S3

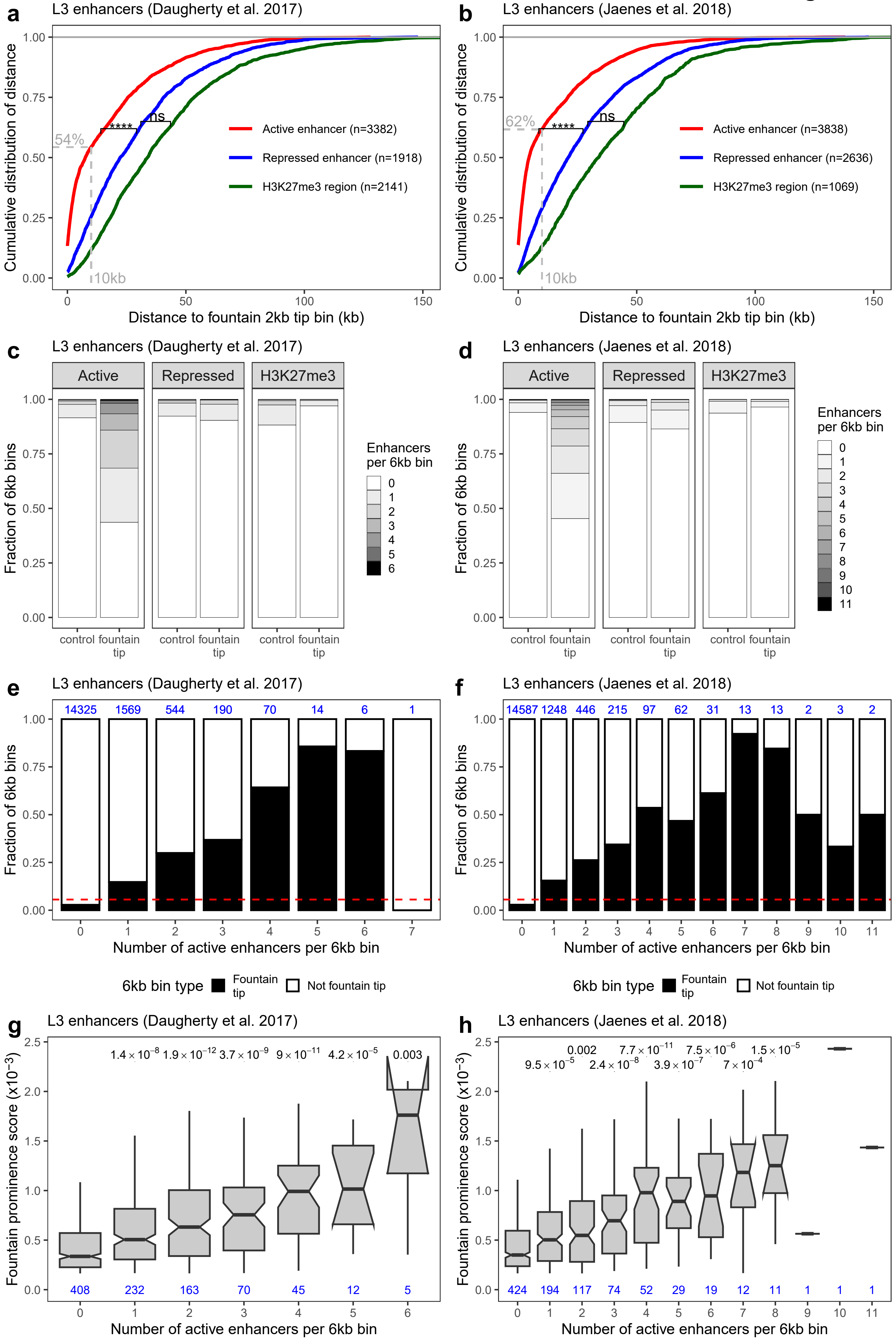

Supplementary Figure 3

Enhancer location and clustering at fountain tips

a,b. Cumulative distribution of the distance of enhancers from the 2 kb fountain tip bin for the different types of enhancers. The percentage of enhancers found within 10 kb of the fountain tip is shown in gray. A one-sided Kolmogorov-Smirnov test was used to compare the curves (**** p value < 0.0001, ns: not significant). **c,d.** Fountain tip bins were resized to 6 kb to include the neighboring bins and the number of enhancers of each type that overlapped these bins was counted. Six kilobase regions located midway between neighboring fountains were used as controls (Fig. 4c). **e,f.** Fountain tips are enriched for clustered active enhancers. The entire genome was divided into 6 kb bins and the number of active enhancers in each bin was counted. The number of 6 kb fountain tip bins containing a given number of active enhancers is shown as a fraction of all genomic 6 kb regions with the indicated number of enhancers (x axis). The total number of genomic 6 kb bins with a given number of enhancers is shown in blue on top. The red line depicts the ratio between the total number of fountain 6 kb bins and the total number of genomic 6kb bins. **g,h** Boxplots of the fountain prominence score of fountain-tip 6kb bins according to the number of enhancers present in that bin. FDR adjusted p-values from a two sided Wilcoxon rank sum test comparing each group to the fountains bins without any enhancers, are shown above the boxplots. **a,c,e,g** plots are for ref. [7] L3 enhancers and **b,d,f,h** plots are for ref. [6] L3 enhancers where the enhancer type was determined by finding the longest overlap with ChromHMM states from ref. [7].

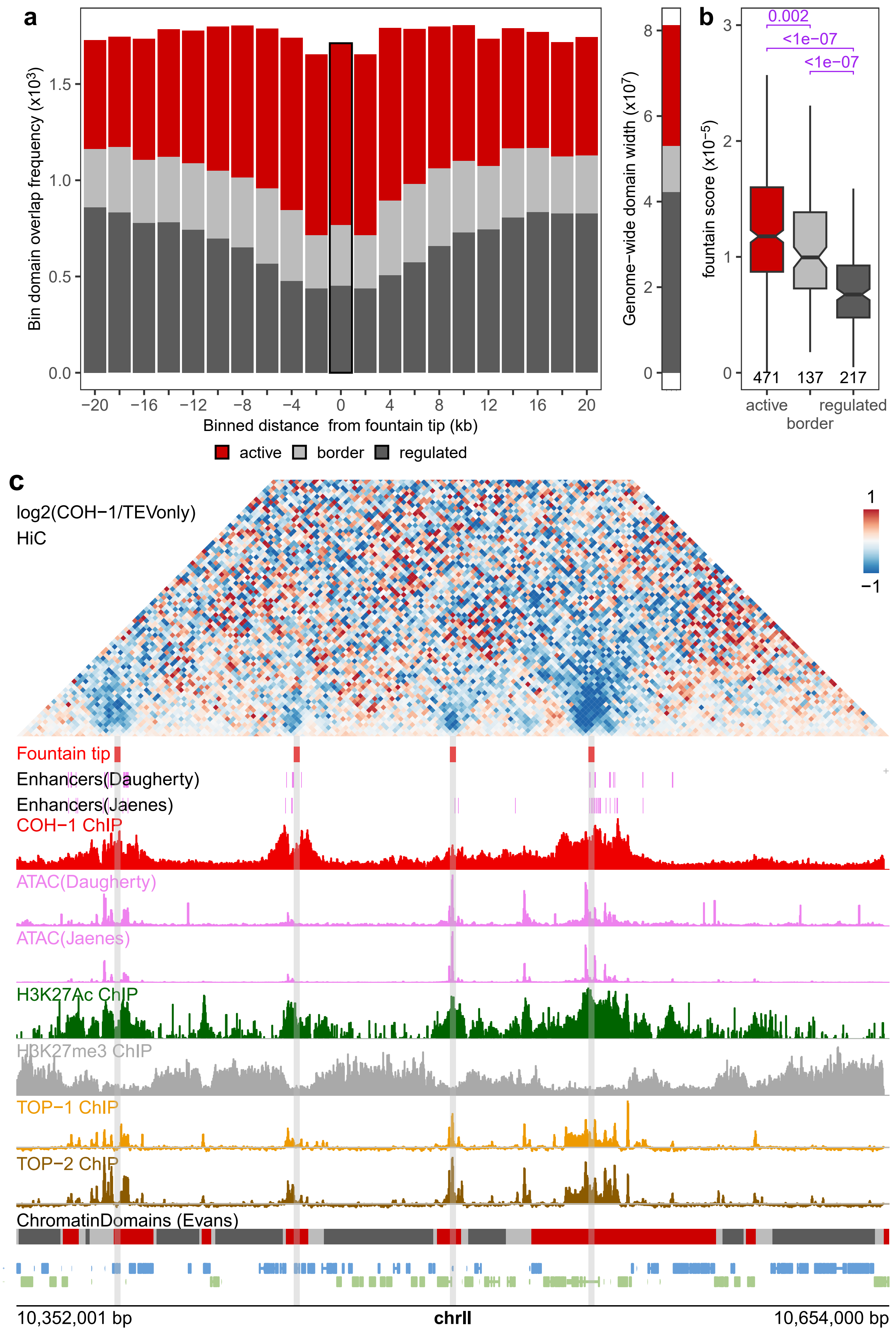
a
Bin domain overlap frequency (x10³)
Binned distance from fountain tip (kb)
active
border
regulated
Genome−wide domain width (x10⁷)
b
fountain score (x10⁻⁵)
0.002
<1e−07
<1e−07
471
137
217
active
border
regulated
c
log2(COH−1/TEVonly)
HiC
Fountain tip
Enhancers(Daugherty)
Enhancers(Jaenes)
COH−1 ChIP
ATAC(Daugherty)
ATAC(Jaenes)
H3K27Ac ChIP
H3K27me3 ChIP
TOP−1 ChIP
TOP−2 ChIP
ChromatinDomains (Evans)
10,352,001 bp
chrII
10,654,000 bp

Supplementary Figure 4

Fountain overlap with chromatin domain types

a. Frequency of overlap of different autosomal chromatin domain types[23] with fountain tip bins and 2 kb bins up to 20 kb upstream and downstream of the fountain tips (left panel), compared to the cumulative genome wide width of these domain types (right panel). **b.** Comparison of the fountain score for fountains that overlap different types of domains. The number of fountains in each group is indicated below the boxplots. Adjusted p-values from Wilcoxon rank sum test are shown in purple. **c.** Example of a 0.3 Mb region on chromosome II containing several fountains that fall within active chromatin domains. Only L3 active enhancers from [7] and [6] are shown in the enhancer tracks. Chromatin IP data: COH-1 in young adults (GSE50324), H3K27 acetylation (GSM624432) and H3K27me3 in L3 larvae (GSM1206310), TOP-1 (GSM5686806 & GSM5686807) and TOP-2 in L2-L3 larvae (GSM5686812 & GSM5686813), bottom track: gene locations.

Lüthi et al.,
Figure S5

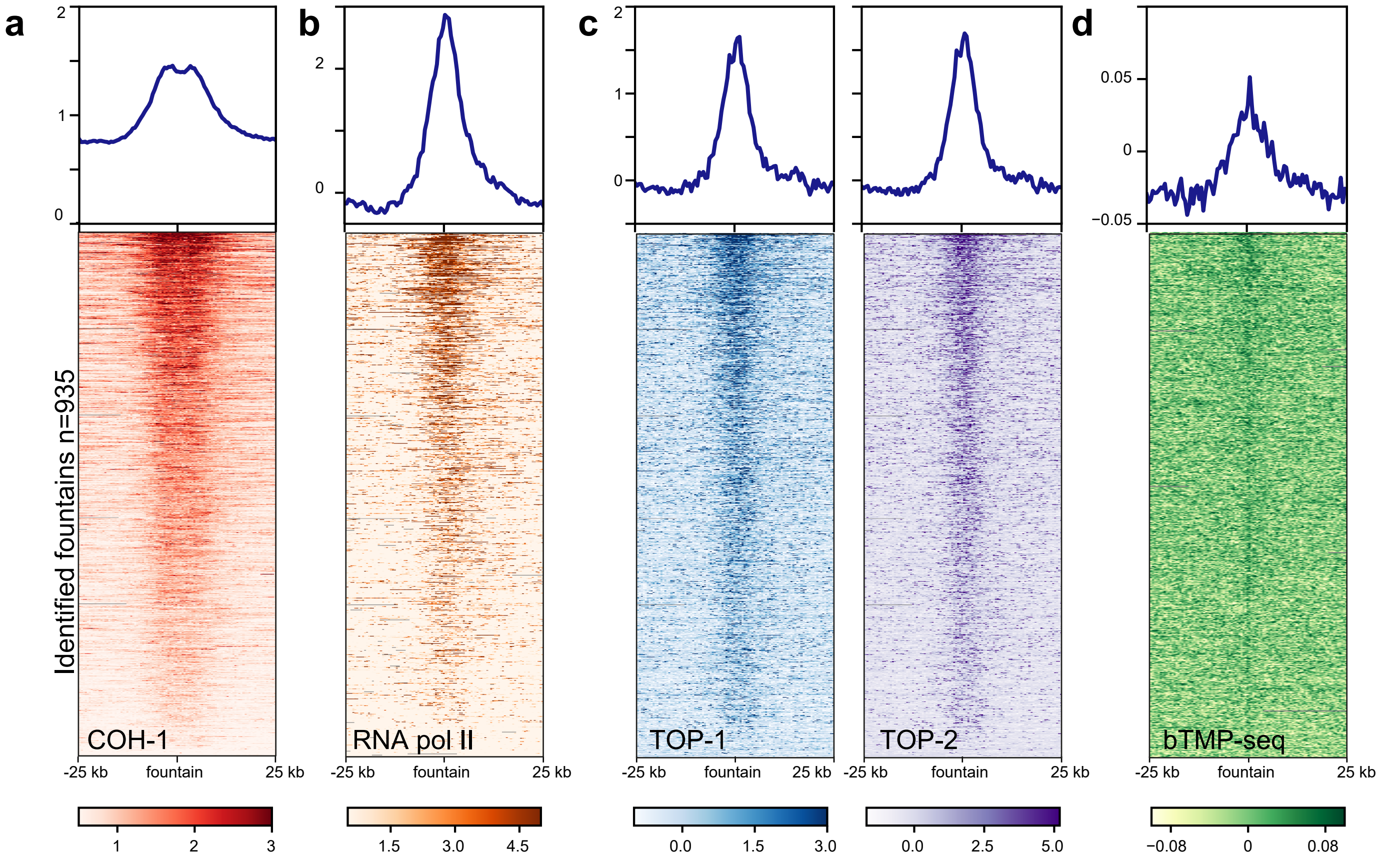

Supplementary Figure 5.

Fountain tips are enriched for COH-1/topoisomerases and bTMP, similar to active enhancers.

(top) Average ChIP-seq profiles at fountains as in Fig. 3a-d for **a.** COH-1 (young adults). **b.** RNA pol II (L3), **c.** (left) TOP-1 (L3) and (right) TOP-2 (L3) **d.** bTMP (L3). (bottom) Heatmap of **a.** COH-1, **b.** RNA pol II, **c.** (left) TOP-1 and (right) TOP-2 **d.** bTMP enrichment centered on fountain tips, sorted by COH-1 ChIP-seq enrichment.

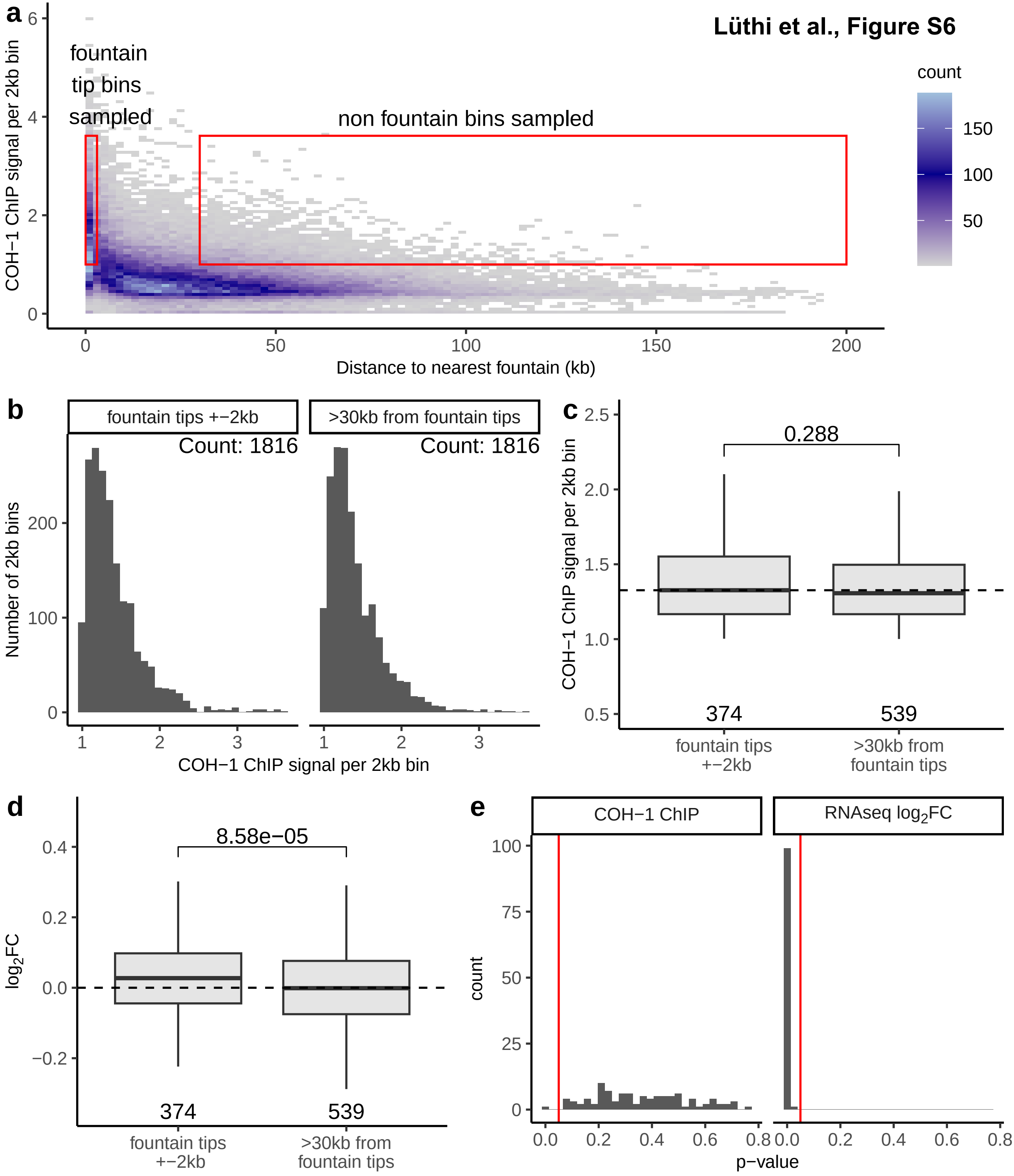

Lüthi et al., Figure S6
a
fountain tip bins sampled
non fountain bins sampled
count
150
100
50
COH−1 ChIP signal per 2kb bin
Distance to nearest fountain (kb)
0
50
100
150
200
0
2
4
6
b
fountain tips +−2kb
>30kb from fountain tips
Count: 1816
Count: 1816
Number of 2kb bins
0
100
200
1
2
3
COH−1 ChIP signal per 2kb bin
c
0.288
2.5
2.0
1.5
1.0
0.5
374
539
fountain tips +−2kb
>30kb from fountain tips
d
8.58e−05
log2FC
0.4
0.2
0.0
−0.2
374
539
fountain tips +−2kb
>30kb from fountain tips
e
COH−1 ChIP
RNAseq log2FC
count
100
75
50
25
0
0.0
0.2
0.4
0.6
0.8
p−value

Supplementary Figure 6

Gene upregulation is specific to fountains and not COH-1 enrichment.

a. Binned density map of COH-1 ChIP signal by distance from the nearest fountain for all 2kb bins in the genome. To identify bins with matched COH-1 levels, we sampled from fountain tip bins and their immediate neighbours and compared them to bins that were at least 30 kb from the nearest fountain as indicated by the regions enclosed in red boxes. **b.** Histograms of the ChIP signal from two examples COH-1-matched, equally-sized sets of bins from fountain tips and distal regions. The matched sets were created by sampling without replacement from quantiles of COH-1 ChIP signal of the bins indicated in panel a. **c.** Boxplot of the COH-1 ChIP signal of all bins from the matched sets in b, that overlapped a TSS of an expressed gene in the COH-1 cleavage RNAseq dataset. For c and d, the p-value from a one-sided Wilcoxon rank sum test is shown above the boxplots and the number of bins in each group are shown below. **d.** Boxplot of the log2 fold change in gene expression upon COH-1 cleavage of genes whose TSS overlaps bins from the COH-1 matched sets of 2kb bins shown in c. **e.** Histograms of the p-values from one-sided Wilcoxon rank sum tests comparing the COH-1 ChIP signal and RNAseq log2 fold change between fountain tips and regions at least 30 kb from fountains, obtained by repeating 100 times the sampling procedure from the regions shown in panel a, to create matched COH-1 subsets as shown for one example in panels b-d. The vertical red line shows the statistical significance threshold of 0.05.

Lüthi et al., Figure S7

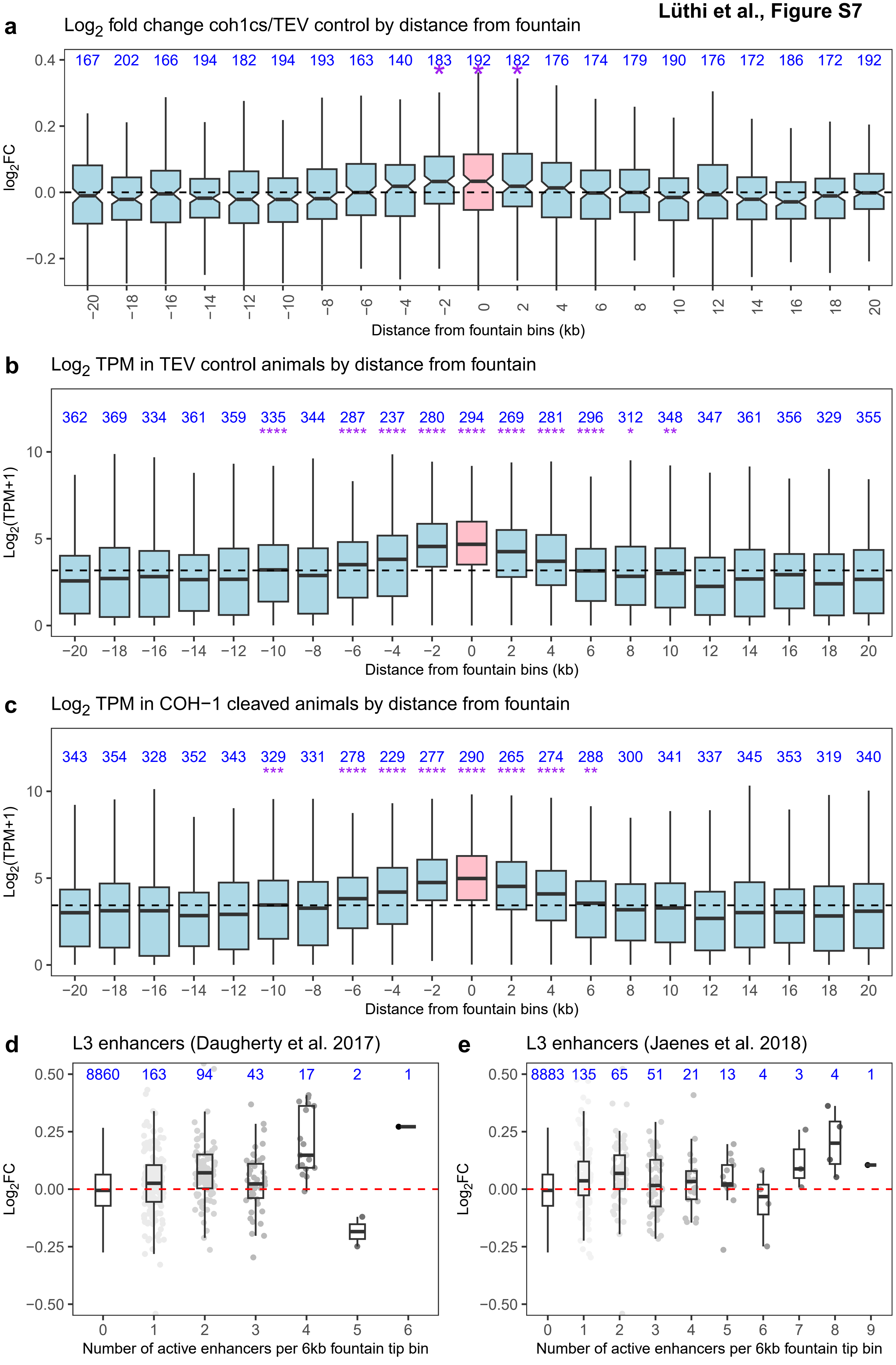

Supplementary Figure 7

Gene expression around fountain tips.

a-c 2 kb bins overlapping fountain tips (pink) and up to 20 kb upstream and downstream (light blue), were used to quantify log2 fold change (**a**) and transcript abundance in control (**b**) and upon cohesin^{COH-1} cleavage (**c**) for genes whose TSS overlaps them. **d,e.** Effect of active enhancer clustering on gene expression: Log2 fold change of genes whose TSS overlaps 6kb bins at the fountain tips with different numbers of active enhancers. The number of genes in each group is shown in blue. Individual data points are shaded in gray.

Lüthi et al., Figure S8

a

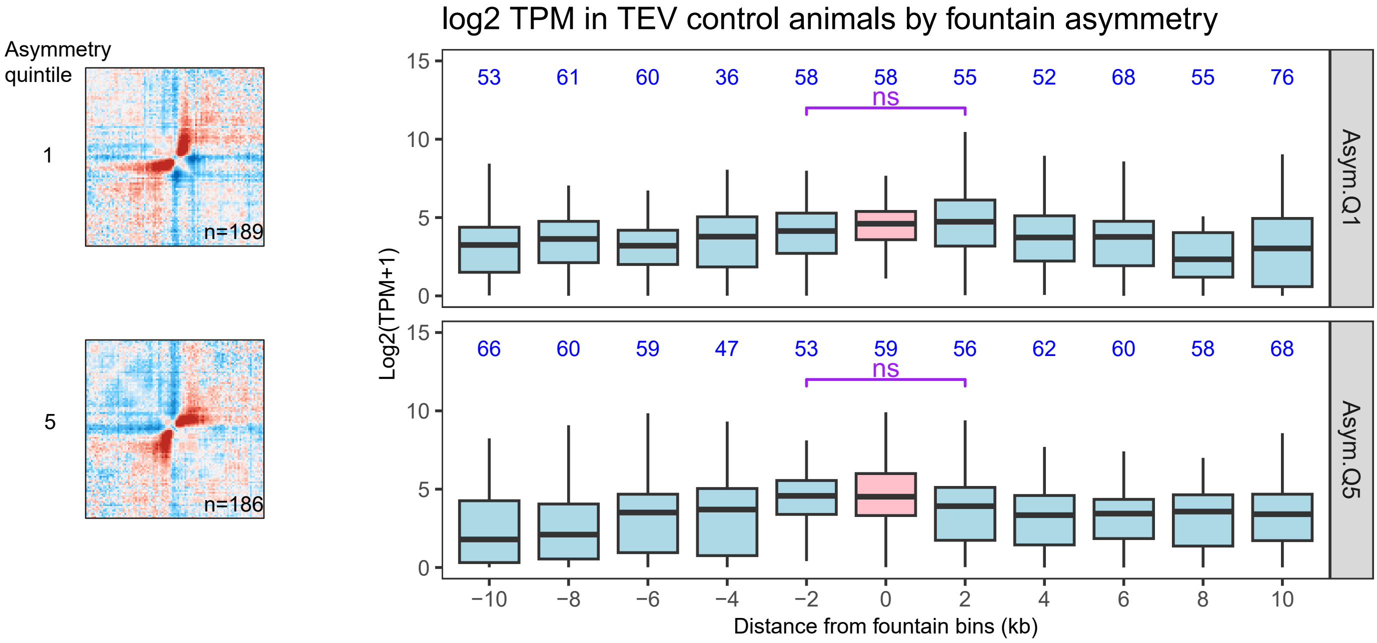

b

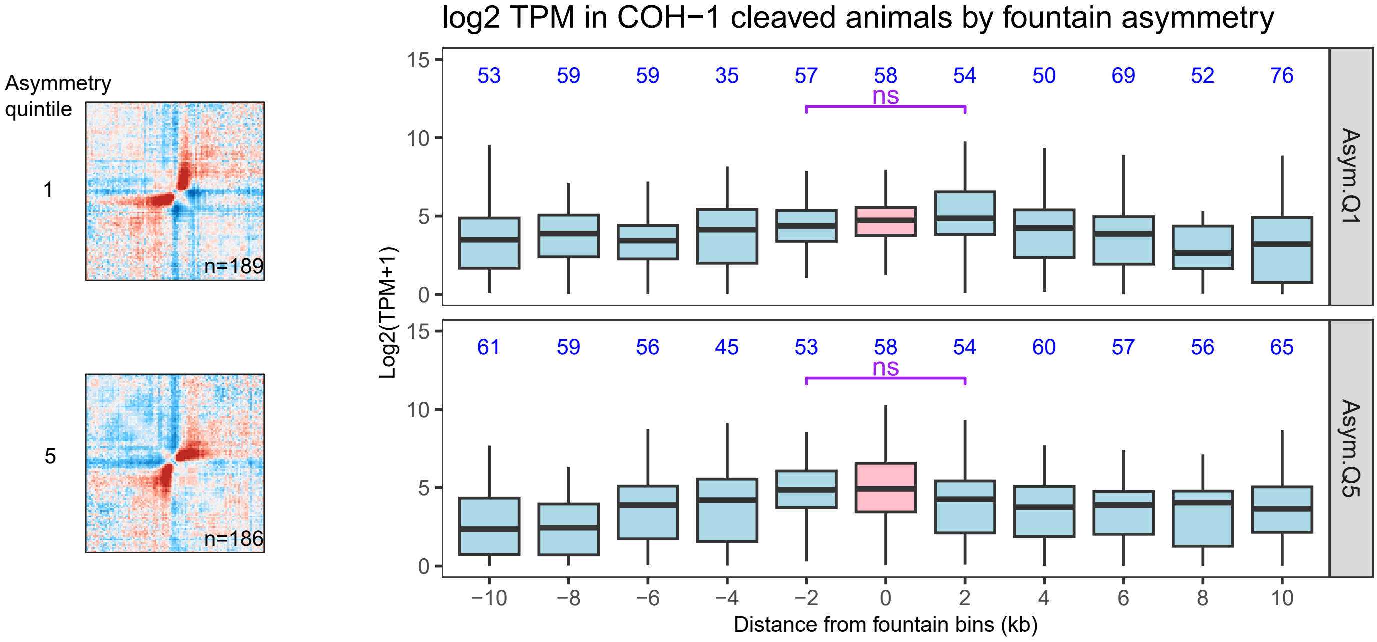

Supplementary Figure 8

Fountain asymmetry is associated with asymmetry in expression levels

Fountain asymmetry scores were divided into five equally sized bins (quintiles, see Fig S1g). 2 kb bins overlapping the fountain-tips (pink) from the highest and lowest quintiles, and up to 10 kb upstream and downstream (light blue), were used to quantify transcript abundance for genes whose TSS overlaps them. **a.** Left panel: average contact frequency maps in regions around asymmetric fountains from the first and fifth quintiles. Right panel: Boxplot of gene expression (TPM) in TEV-only control samples of genes whose TSSs overlap the respective 2 kb bins around fountain tips. **b.** Left panel as in a. Right panel: Boxplot of gene expression (TPM) in COH-1 cleavage samples of genes whose TSSs overlap the respective 2 kb bins around fountain tips. In both a and b the purple line indicates that a two sided Wilcoxon rank sum test was carried out to compare the expression of bins immediately upstream and downstream of the fountain tip (ns, not significant). The number of genes in each bin are indicated in blue.

Lüthi et al.
Figure S9

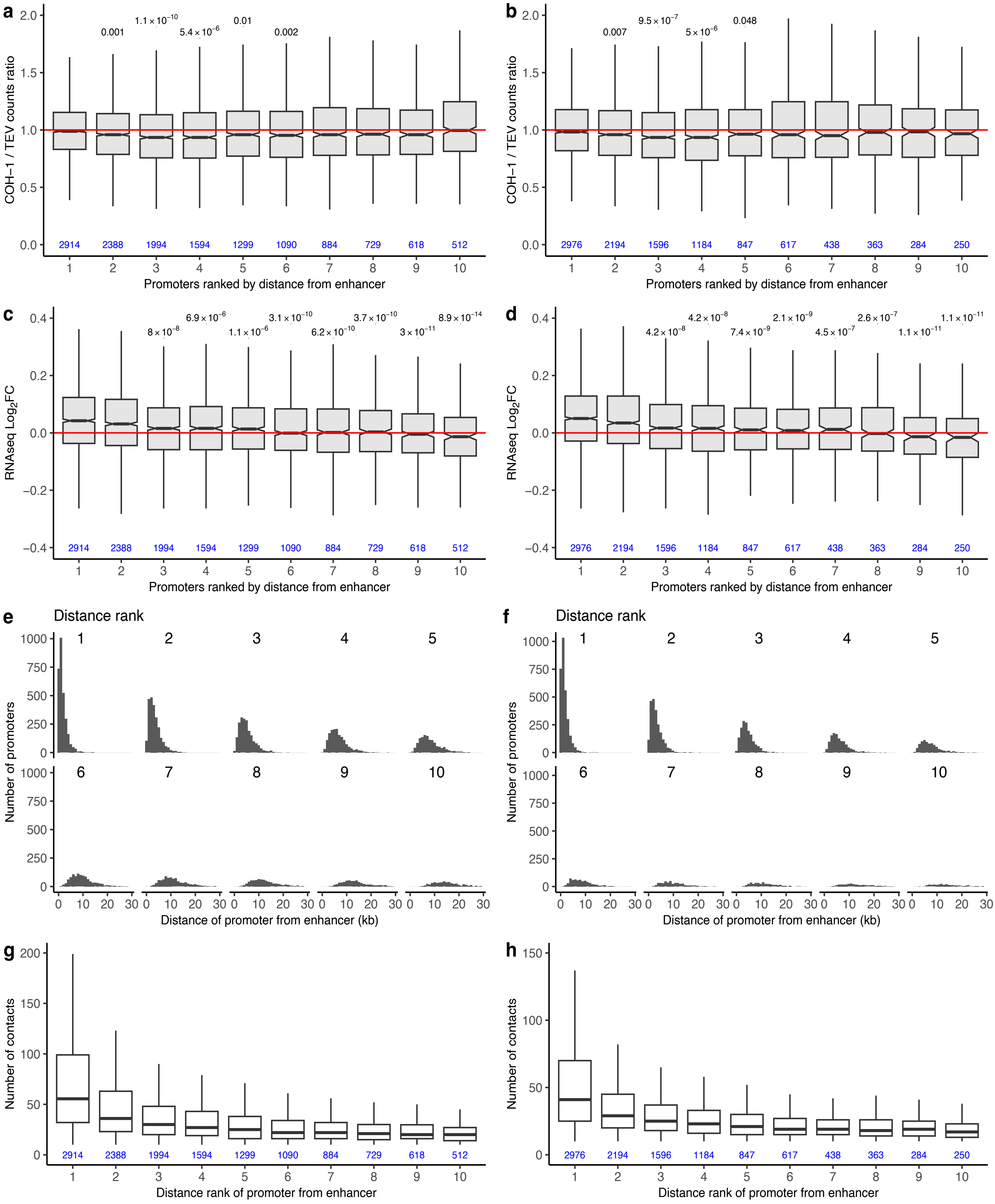

Supplementary Figure 9

Changes in active enhancer promoter contacts upon COH-1 cleavage and transcriptional modifications on target genes.

a, b. Ratio of Hi-C fragment contacts from COH-1 cleavage and TEV-only control data for each L3 active enhancer with its closest 10 transcript promoters up to a maximum distance of 30 kb. Promoters were ranked by the genomic distance from the active enhancer **c, d.** RNA-seq log2 fold change between COH-1 cleavage and TEV control for promoters ranked by their genomic distance from the active enhancer. **e, f.** Histogram of the genomic distances of the closest 10 promoters to the active enhancer grouped by distance rank . **g, h.** Boxplot of the number of active enhancer-promoter contacts grouped by the distance rank of the promoter from the enhancer in TEV control Hi-C data. For a-d, g & h the number of active enhancer-promoter pairs in each ranked group is shown in blue below the boxplot. For a-d the significant FDR adjusted p-values for a two-sided Wilcoxon rank sum test comparing each group to the first are shown at the top. a,c,e,g panels are for L3 active enhancers from Daugherty et al. (2017) and b,d,f,h panels are for L3 active enhancers from Jaenes et al. (2018).

Lüthi et al., Figure S10

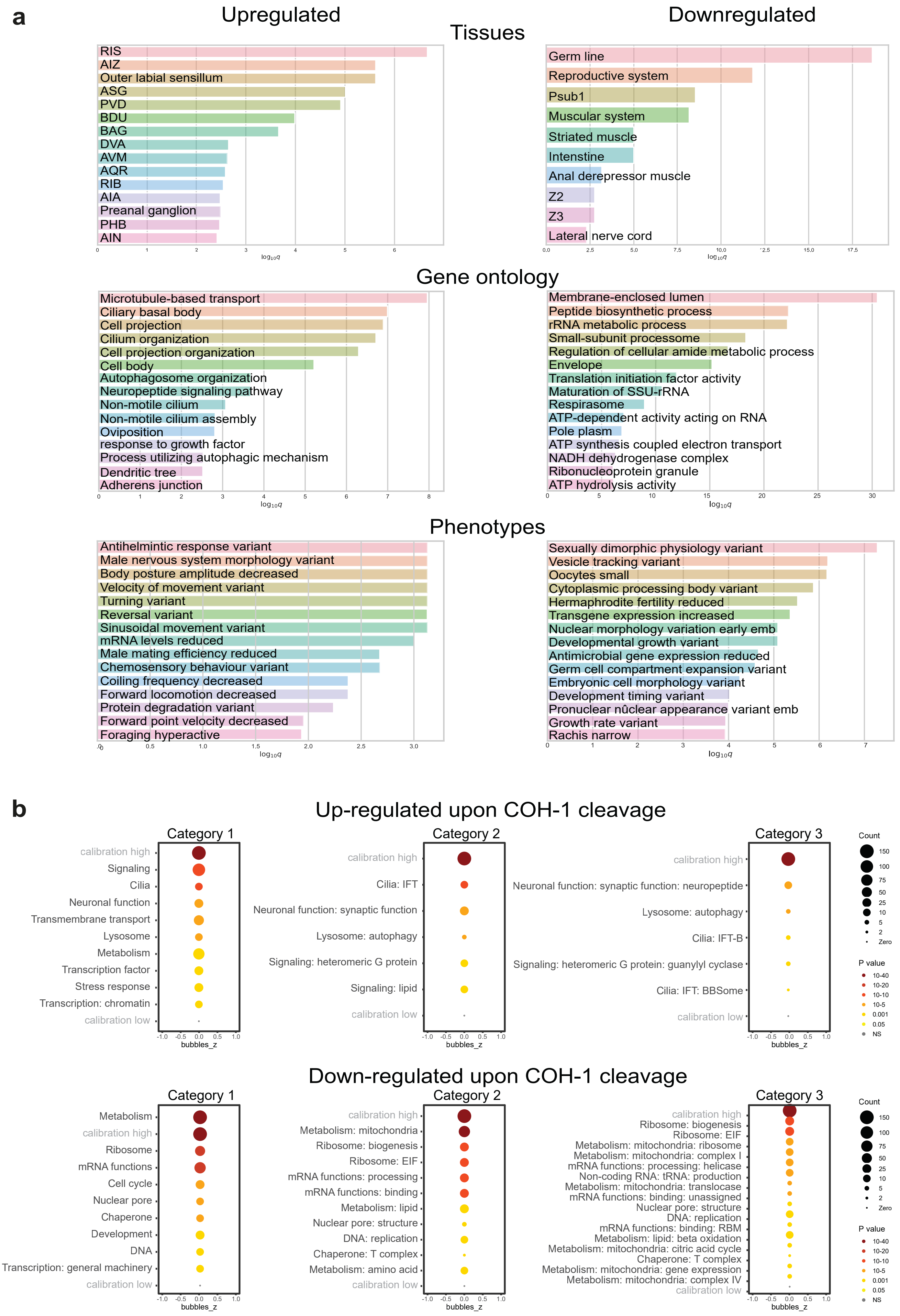

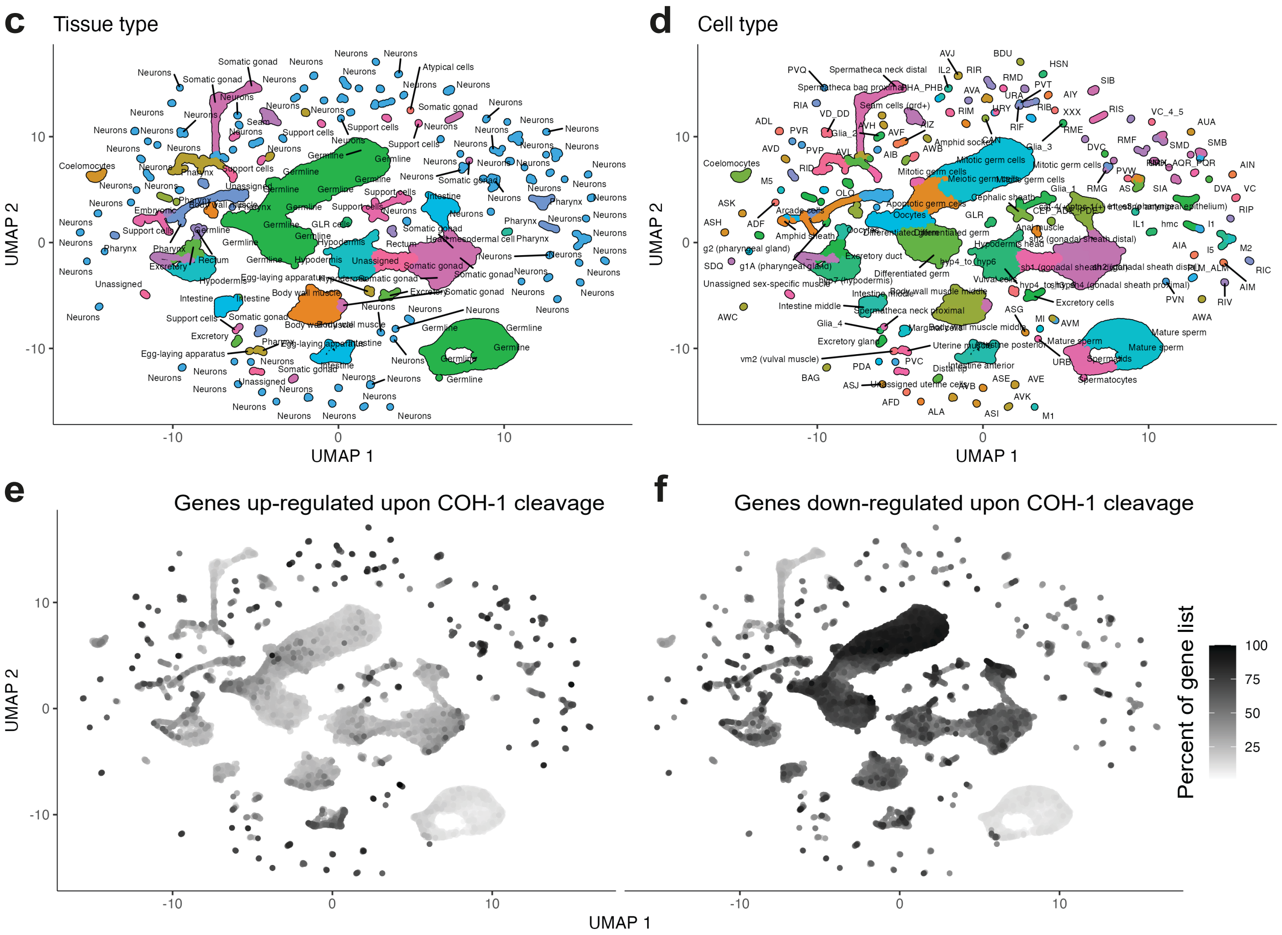
c
Tissue type
d
Cell type
UMAP 1
UMAP 2
e
Genes up-regulated upon COH-1 cleavage
f
Genes down-regulated upon COH-1 cleavage
Percent of gene list
100
75
50
25
UMAP 1
UMAP 2

Supplementary Figure 10. GO term, tissue, cell and phenotype enrichment for genes changing upon COH-1 cleavage.

a. Tissue Enrichment Analysis for genes significantly up and down regulated upon COH-1^{cs} cleavage (Padj<0.05), using tissues, Gene Ontology and Phenotypes annotations[29]. **b.** WormCat enrichment analysis for genes significantly up and down regulated upon COH-1^{cs} cleavage (adjP<0.05; ref. [66]). **c.** UMAP visualization of the 180 identified cell types as in ref. [30], annotated according to tissue type. **d.** Same map as in c with individual cell types annotated. **e.** Same map as in c with individual cells colored according to the percentage of genes up-regulated upon COH-1 cleavage expressed in those cells (scale in f). **f.** Same map as in c, with individual cells colored according to the percentage of genes down-regulated upon COH-1 cleavage expressed in those cells.

Lüthi et al., Figure S11

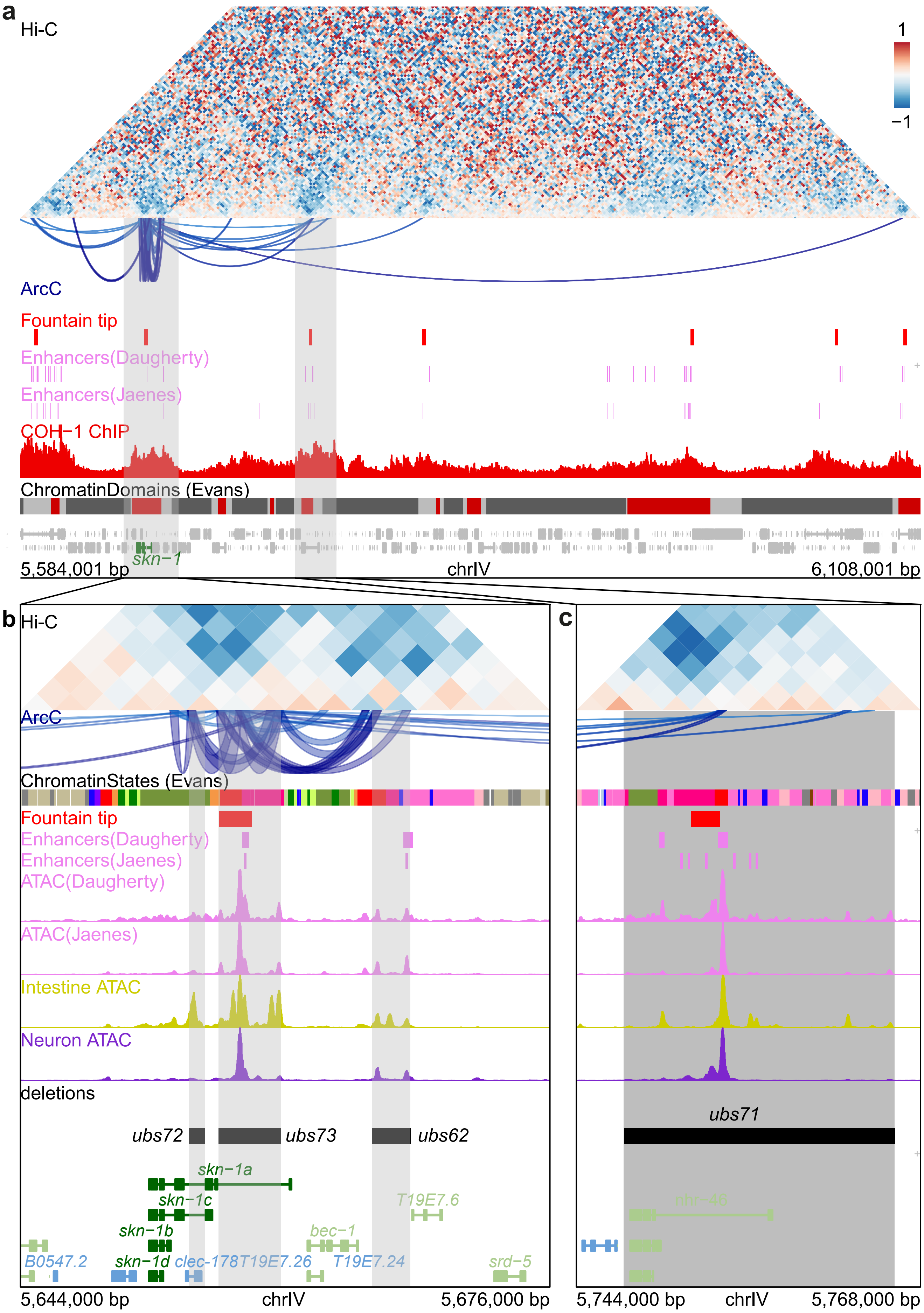

Supplementary Figure 11. Deletion of putative *skn-1* enhancers

a. View of a 524 kb region around the *skn-1* gene (highlighted in green in the gene track). The region was chosen by finding all significant ARC-C cis interactions[8] with at least one anchor in the *skn-1* gene and its flanking intergenic regions. Grey highlights in the top panel indicate regions shown in detail below: **b.** a 32 kb region around the *skn-1* and *bec-1* genes and **c.** a distal 24 kb region around the *nhr-46* gene. The regions highlighted in the bottom panels correspond to the regions targeted by the deletions. Hi-C: log2 of the ratio of cohesin COH-1 cleavage and TEV control Hi-C maps at 2 kb resolution. Fountain tip: 2 kb bin found at the tip of fountains. Enhancers(Daugherty): L3 active enhancers from [7]. Enhancers(Jaenes): L3 active enhancers from [6]. COH-1 ChIP: from young adults (GSE50324). ChromatinDomains (Evans): active (red), regulated (dark grey) and border (light grey) domains as per [23]. Chromatin states (Evans): see previous reference, red-pink colors indicate enhancer/promoter chromatin states. Intestinal and neuronal ATAC: tissue-specific ATAC in L2 larvae from [36]. Deletions: CRISPR deletions carried out in this study. The refgene transcript track labeled by gene name is shown on the bottom with transcripts coloured by strand (forward - blue, reverse - green) and *skn-1* transcripts highlighted in dark green.

Lüthi et al.
Figure S12

a

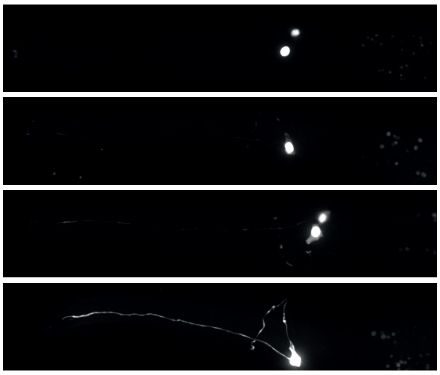

Score 1: nuclear staining only

Score 2: staining mostly missing from dendrites but very weak staining or puncta in the cell body near nucleus

Score 3: staining is weak or punctate in sensory dendrite

Score 4: strong staining in sensory dendrites and axons

b

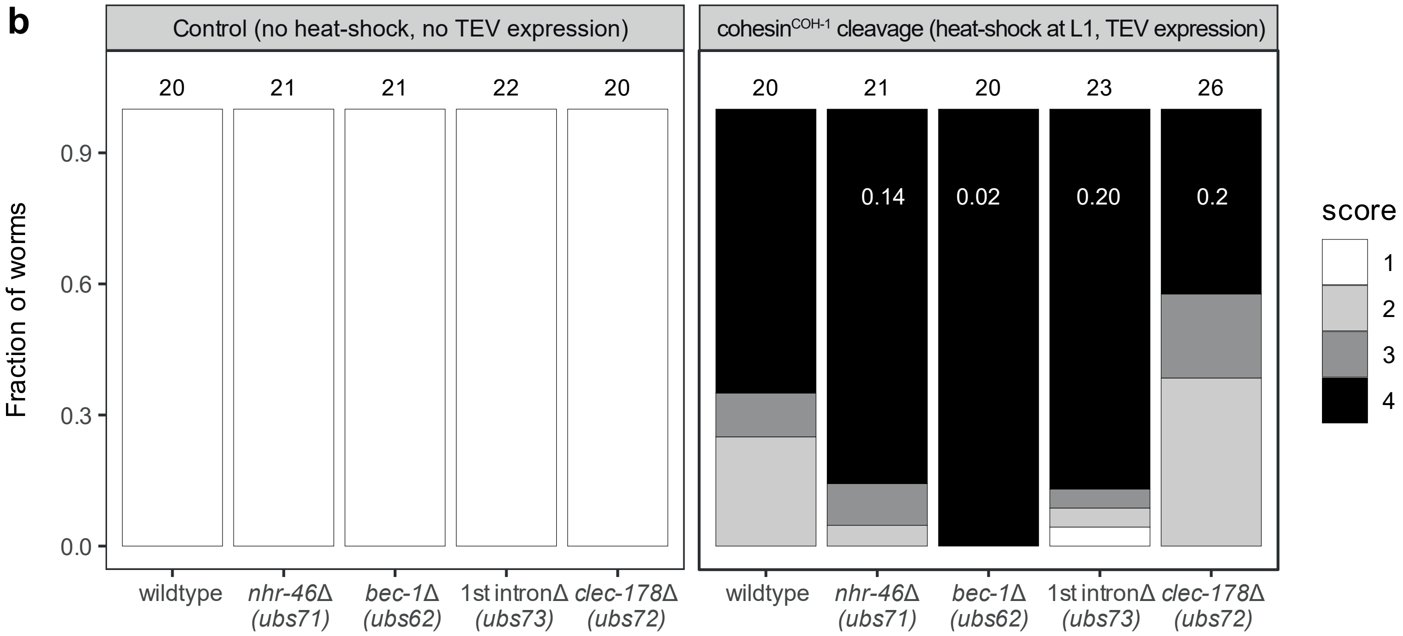

Supplementary Figure 12

The effect of enhancer deletions on *skn-1::GFP* expression in ASI

a. Scoring scheme used to evaluate ectopic expression of *skn-1::GFP* in ASI cells. Images were scored blindly by two separate individuals and then averaged. **b.** Images of the heads of control and COH-1 cleavage worms were scored blindly as described in a. The number of animals scored in each group is shown above the bars. FDR adjusted p-values from an extended Cochran–Armitage test comparing all COH-1 cleavage enhancer deletion strains to the COH-1 cleavage wildtype (without enhancer deletions) strain are shown inside the bars in white.

Lüthi et al.,
Figure S13

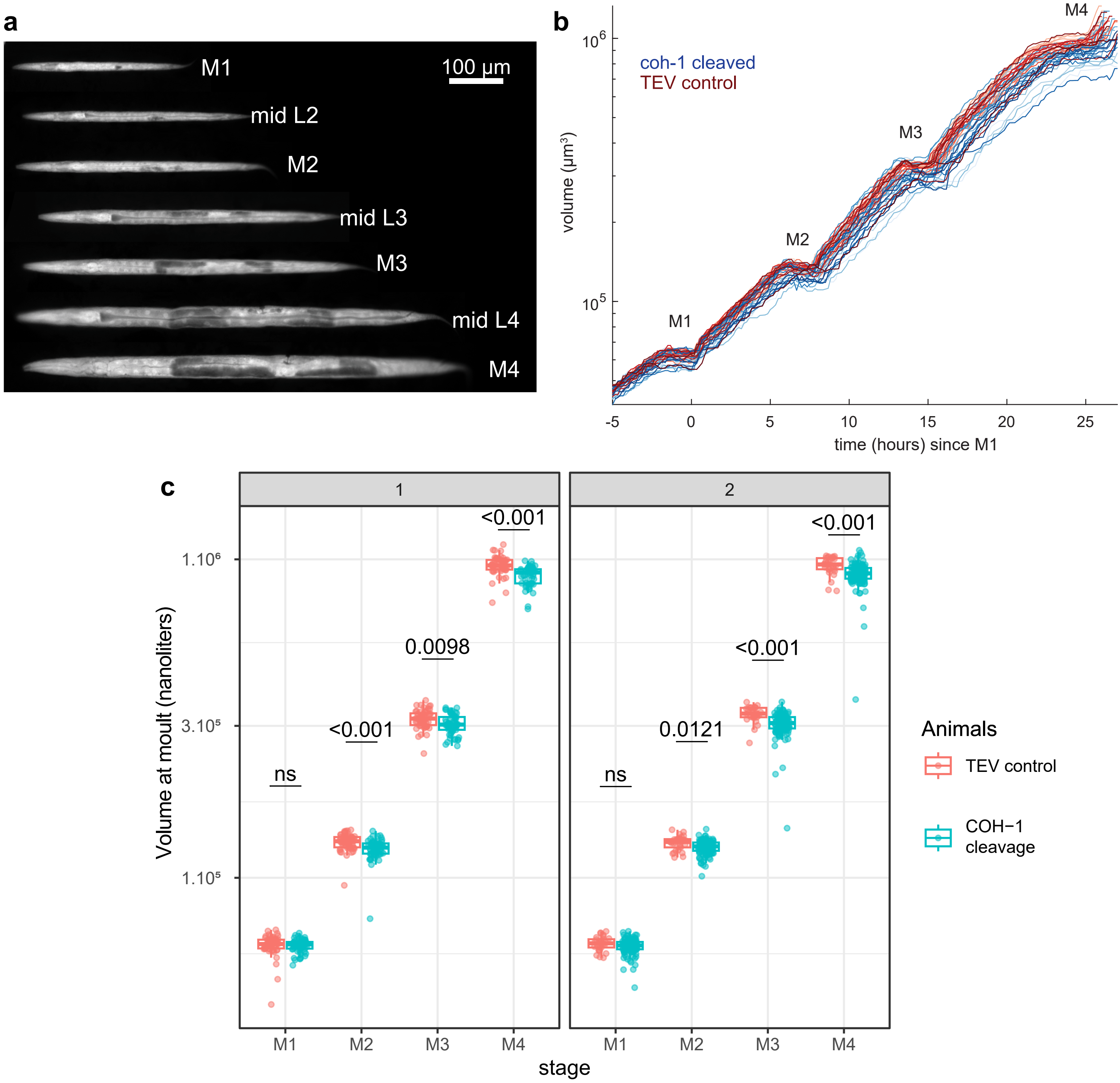

Supplementary Figure 13. Growth analysis of animals upon TEV control expression or COH-1 cleavage

a. An individual animal imaged in a micro chamber at indicated developmental milestones. Contrast was adjusted for each time point individually and the animal straightened computationally. Scale bar: 100 µm. **b.** Example growth curves for 10 control animals and 10 animals upon COH-1 cleavage, synchronized on the first molt. Molts are marked by M. **c.** Body volume at molts for two independent experiments in control animals and upon COH-1 cleavage, n=57;60 (first experiment), 29;122 (second experiment). Box represents 1st quartile, median and 3rd quartile of the data (bottom to top), with individual animals marked as dots. Values above samples are Wilcoxon rank sum test p-values.

Lüthi et al., Figure S14

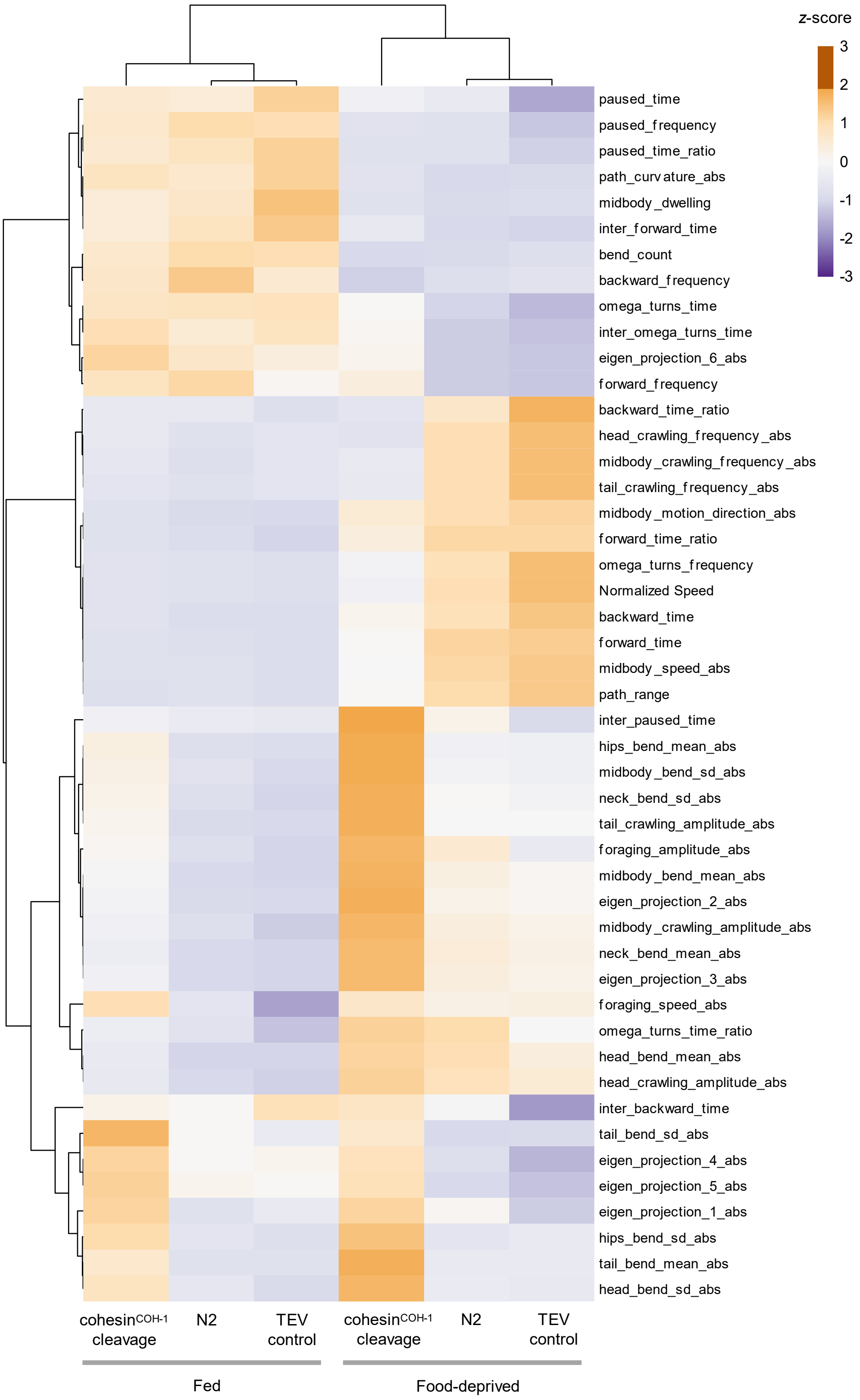

Supplementary Figure 14

COH-1 cleavage produces broad effects on animal posture and locomotion

Heat-map of behavioral parameters (as z-scores) across the indicated conditions (same treatment as in Fig. 6) and hierarchical clustering based on Euclidean distances (trees). Each data point represents the average value for 3-min recordings on n=15 independent replicates (each scoring ≥40 worms).

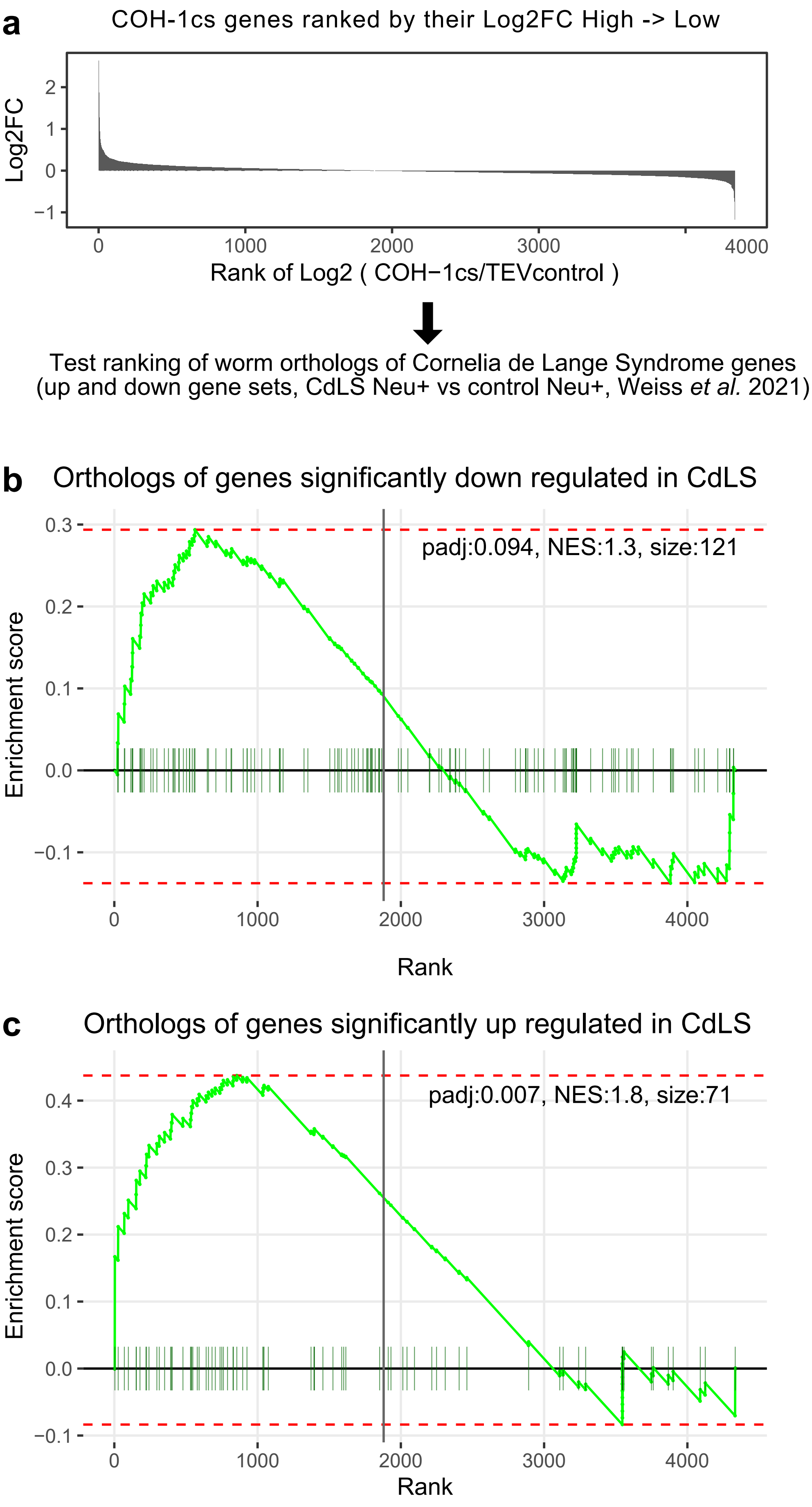
a
COH-1cs genes ranked by their Log2FC High -> Low
Log2FC
2
1
0
−1
0
1000
2000
3000
4000
Rank of Log2 (COH−1cs/TEVcontrol)
Test ranking of worm orthologs of Cornelia de Lange Syndrome genes
(up and down gene sets, CdLS Neu+ vs control Neu+, Weiss et al. 2021)
b
Orthologs of genes significantly down regulated in CdLS
padj:0.094, NES:1.3, size:121
Enrichment score
0.3
0.2
0.1
0.0
−0.1
0
1000
2000
3000
4000
Rank
c
Orthologs of genes significantly up regulated in CdLS
padj:0.007, NES:1.8, size:71
Enrichment score
0.4
0.3
0.2
0.1
0.0
−0.1
0
1000
2000
3000
4000
Rank

Supplementary Figure 15

Gene set enrichment analysis (GSEA) of Cornelia de Lange syndrome genes among their nematode orthologs ranked by their log2FC upon COH-1 cleavage.

a. Schematic of GSEA procedure: genes were ranked in descending order by their log2 fold change upon COH-1 cleavage. The ranks of *C. elegans* orthologs of genes significantly changing in Cornelia de Lange Syndrome (CdLS) patient neurons vs control neurons[64] were tested for enrichment. **b.** Enrichment of nematode orthologs of genes significantly down regulated in CdLS patient neurons. **c.** Enrichment of nematode orthologs of genes significantly up regulated in CdLS patient neurons. The adjusted p value (padj), normalized enrichment score (NES) and the size of the gene set is shown in each panel on the top right corner. Green ticks indicate the position of the orthologs of CdLS genes among the nematode genes ranked by their log2FC upon COH-1 cleavage. The light green line indicates the enrichment score: a running sum of the degree to which the CdLS genes are enriched at the beginning of the COH-1 cleavage ranked list (positive values) versus the end of the list (negative values).
