# Supplementary material for: Cohesin forms fountains at active enhancers in C. elegans
Source: Nat Commun. 2025 Dec 11;17:681. doi: 10.1038/s41467-025-67302-6 (PMC12820367; doi:10.1038/s41467-025-67302-6)
Supplement: Supplementary file 2 — Description of Additional Supplementary Files [file 41467_2025_67302_MOESM2_ESM.pdf]

Description of Additional Supplementary Files

Supplementary Data 1.

Python script for fountain detection on ratio maps. (ipynb format).

Supplementary Data 2.

Bed format file with detected fountains genome-wide (result of Supplementary Data 1).

Supplementary Data 3.

Gallery of the 935 identified fountains, one fountain per row. The first column shows the Hi-C contact map around the identified fountain, upper right: TEV control conditions; lower left: cohesin$^{COH\text{-}1}$ cleavage. The second column shows the log-ratio map between cohesin$^{COH\text{-}1}$ cleavage and TEV control. The third column shows the Hi-C contact map as in the first column, upper right: cohesin$^{SCC\text{-}1}$ cleavage; lower left: cohesin$^{COH\text{-}1}$ and cohesin$^{SCC\text{-}1}$ simultaneous cleavage. The last column is the log-ratio map between simultaneous cohesin$^{COH\text{-}1}$ and cohesin$^{SCC\text{-}1}$ cleavage and cohesin$^{SCC\text{-}1}$ cleavage. For each contact map, genes are indicated on the upper and left side (horizontal lines) as well as the identified active enhancers[7] (vertical ticks). The COH-1 ChIP-seq enrichment from modENCODE is depicted on the lowest track on the top of the Hi-C-map, as well as the right-most track on the left side.

Supplementary Movie 1.

TEV control animals (PMW366) crawling on an agar plate without bacterial food, 20 hours post TEV induction at L1 stage.

Supplementary Movie 2.

Animals in which COH-1 has been cleaved (PMW828), crawling on an agar plate without bacterial food, 20 hours post TEV induction at L1 stage.
