# Supplementary material for: Cohesin forms fountains at active enhancers in C. elegans
Source: Nat Commun. 2025 Dec 11;17:681. doi: 10.1038/s41467-025-67302-6 (PMC12820367; doi:10.1038/s41467-025-67302-6)

chrII:10451001-10651000

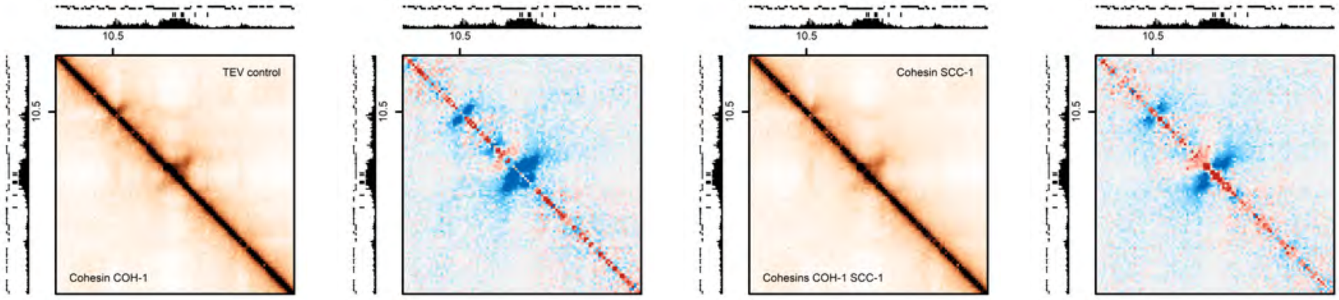

chrI:6983001-7183000

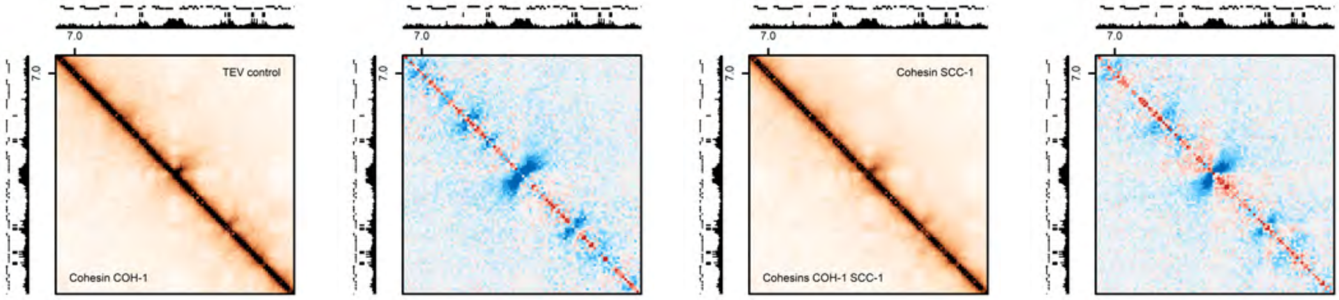

chrV:14035001-14235000

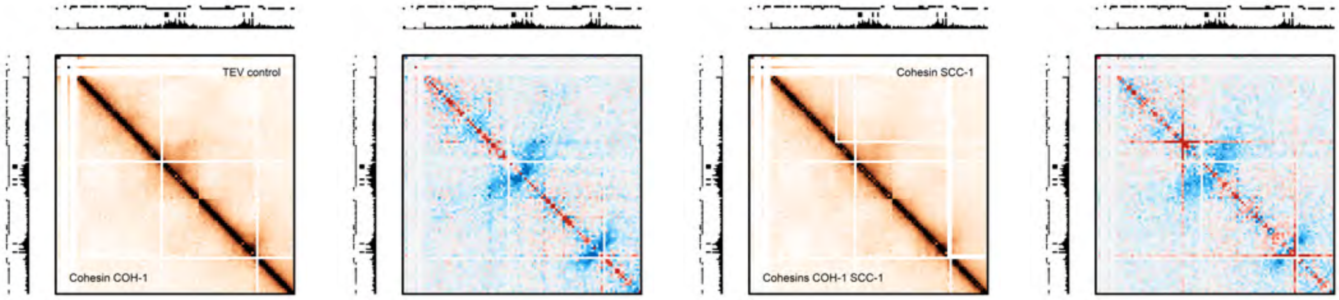

chrIII:5739001-5939000

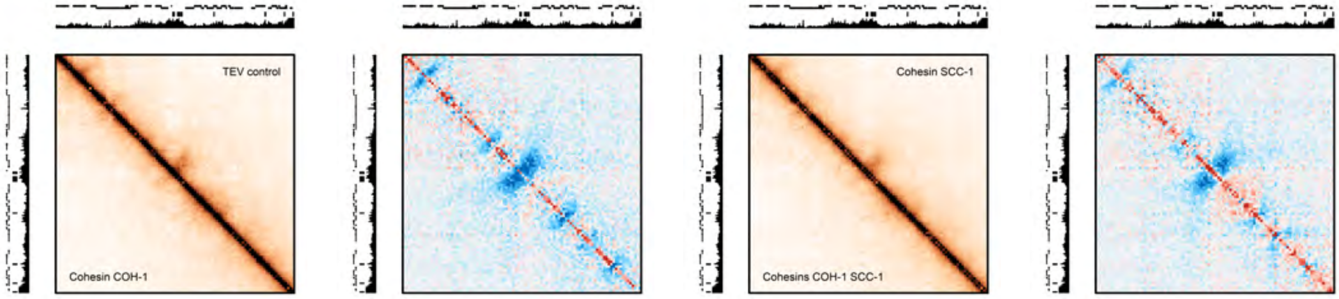

chrIII:6733001-6933000

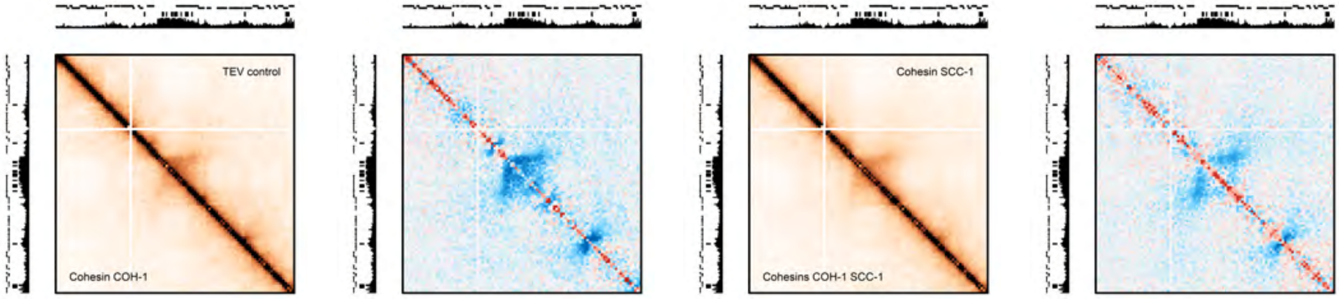

chrX:4043001-4243000

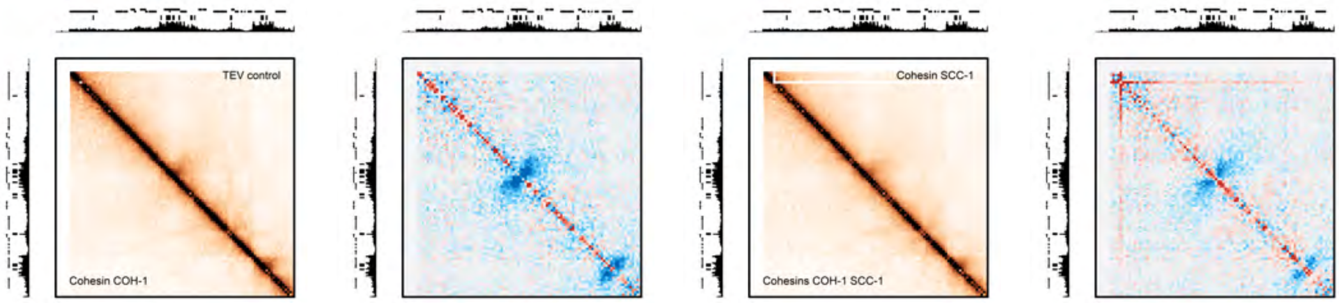

chrI:9013001-9213000

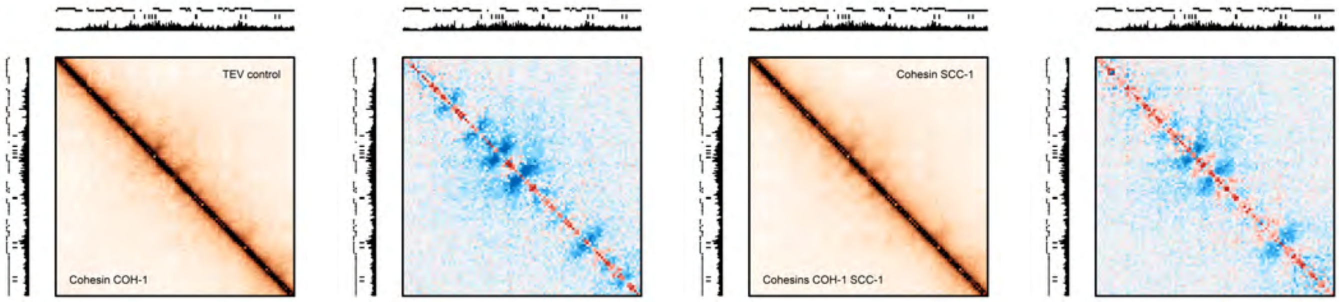

chrIII:12513001-12713000

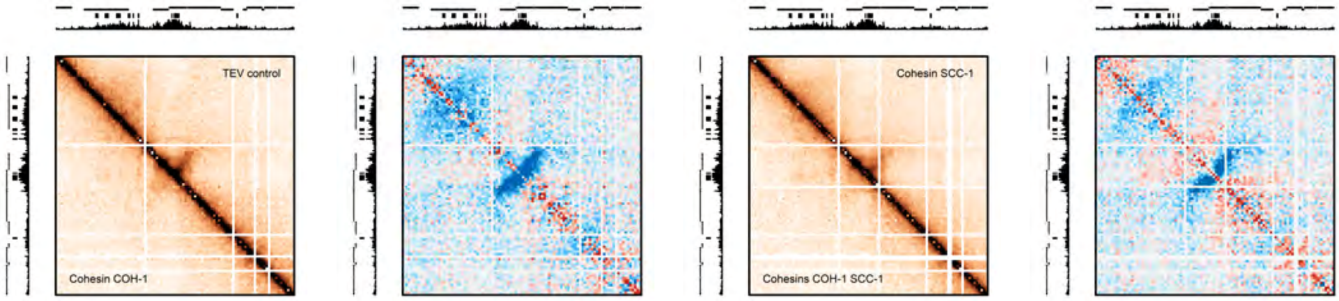

chrI:1597001-1797000

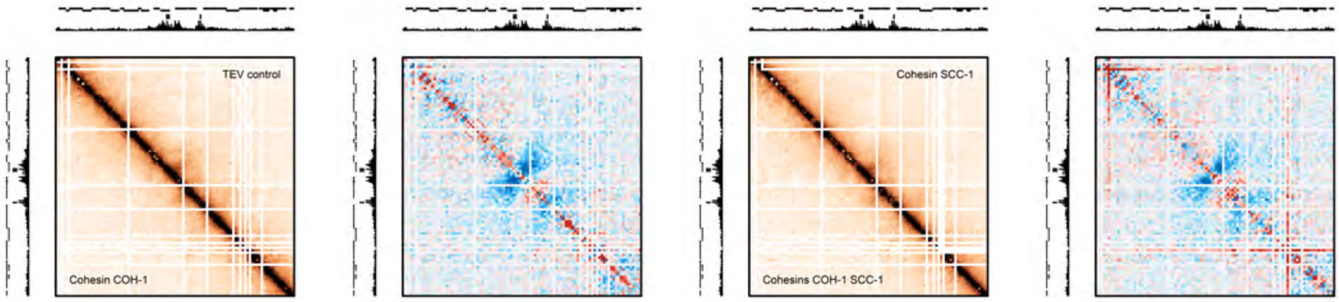

chrIV:4373001-4573000

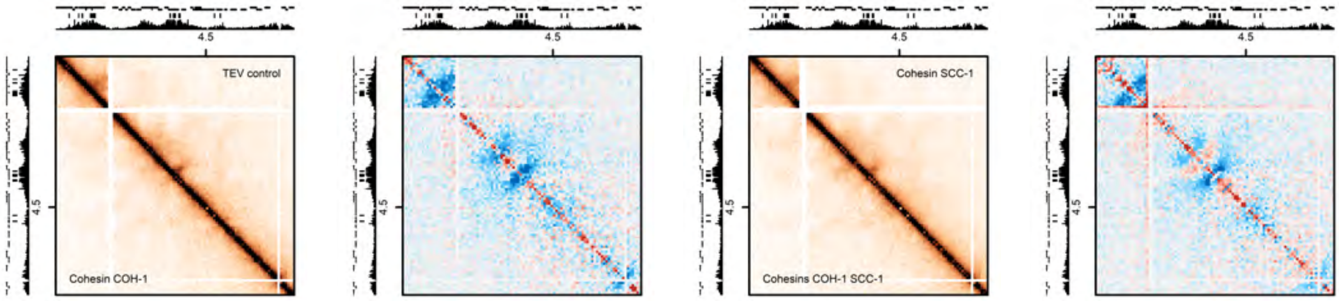

chrII:15109001-15309000

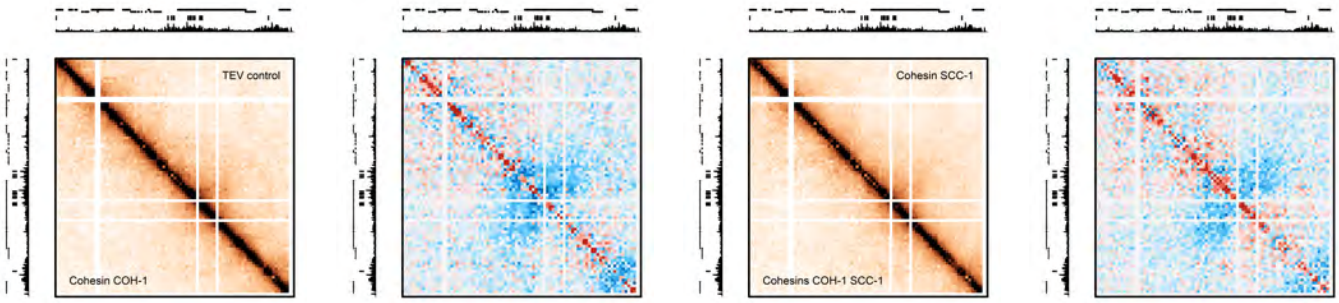

chrII:9181001-9381000

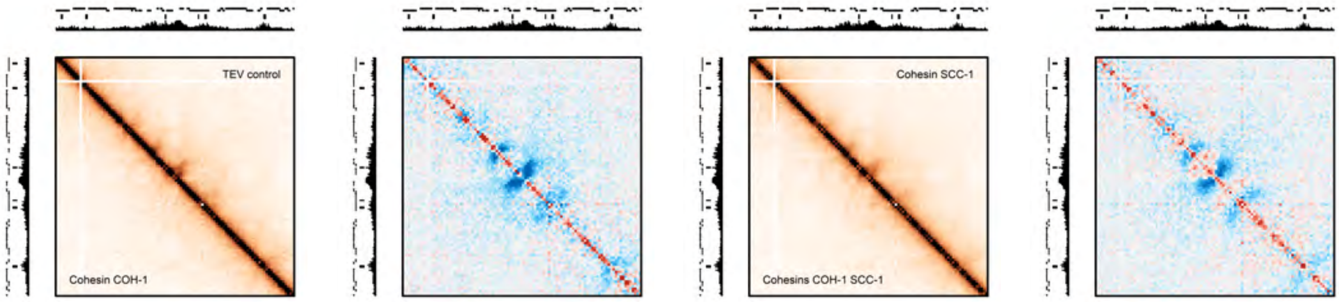

chrV:6127001-6327000

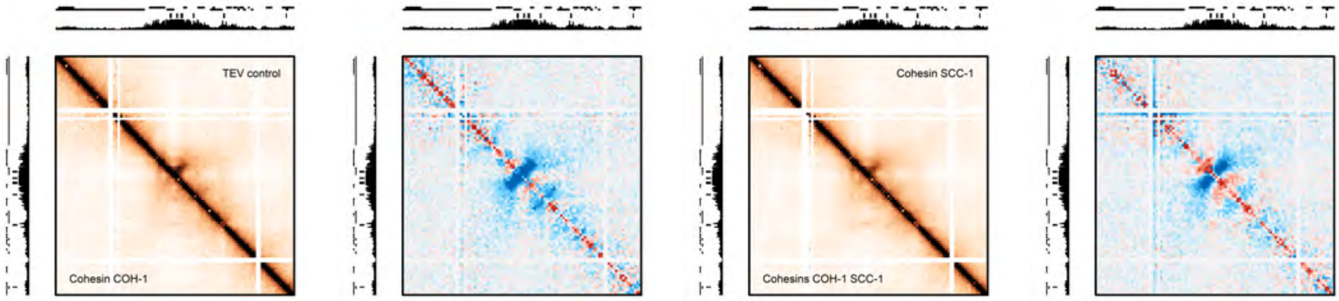

chrI:7393001-7593000

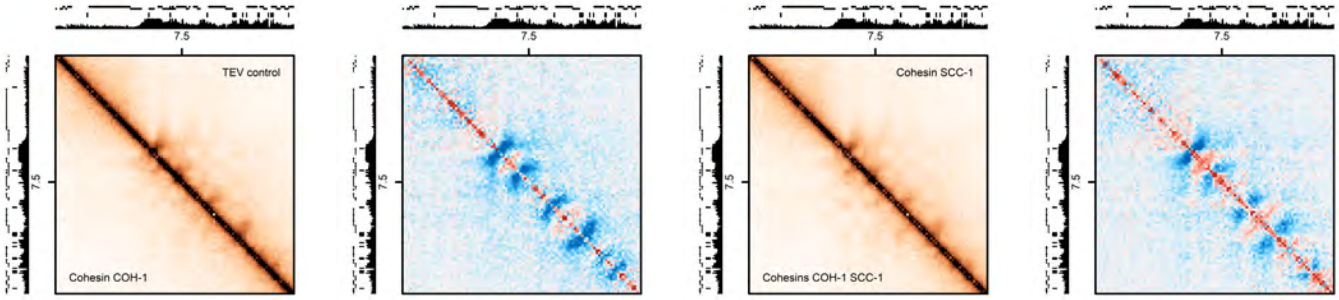

chrV:10769001-10969000

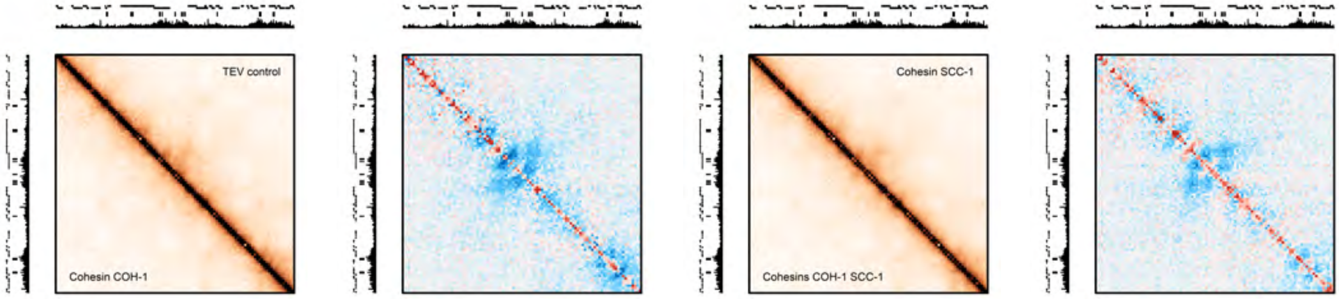

chrV:8837001-9037000

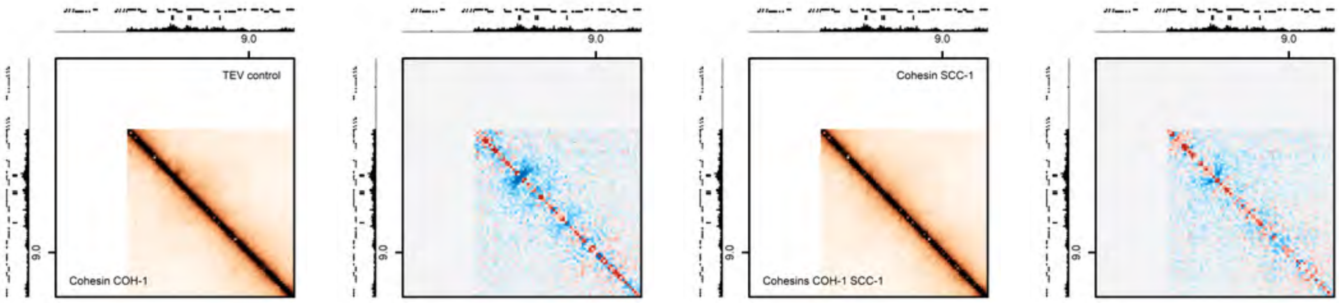

chrIII:8699001-8899000

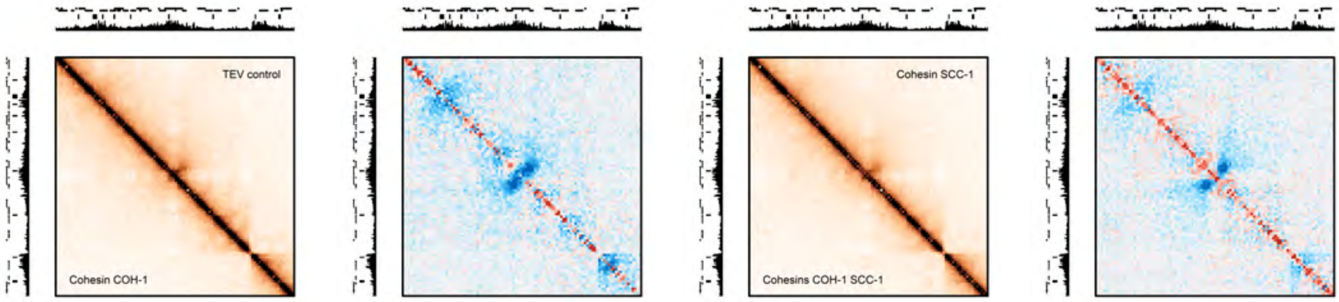

chrX:7919001-8119000

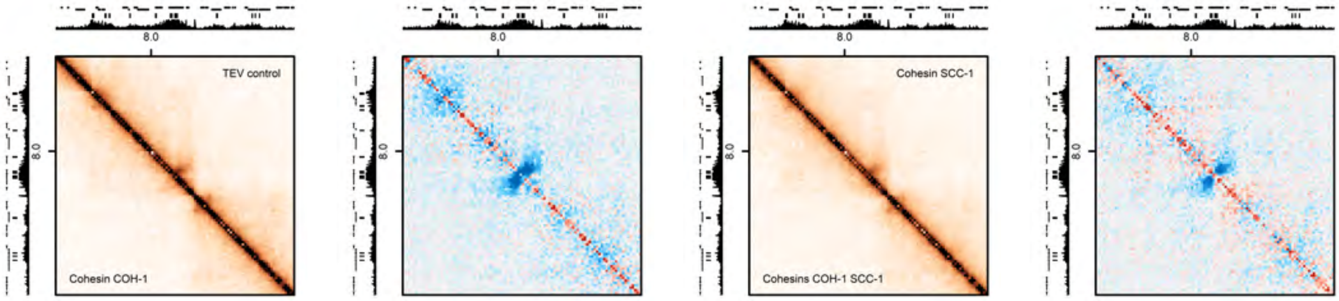

chrV:1053001-1253000

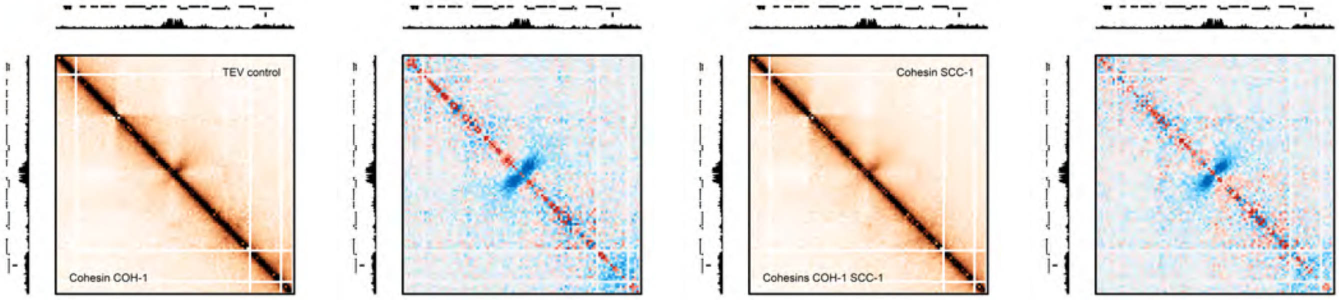

chrIV:3033001-3233000

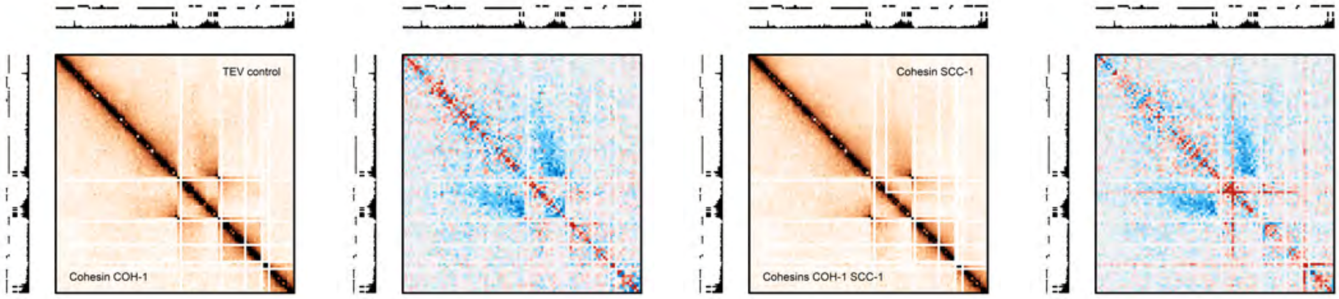

chrI:11943001-12143000

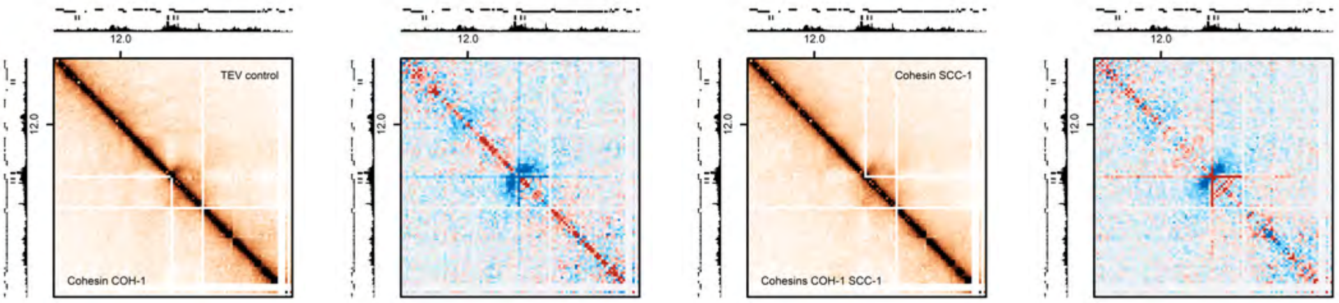

chrII:2875001-3075000

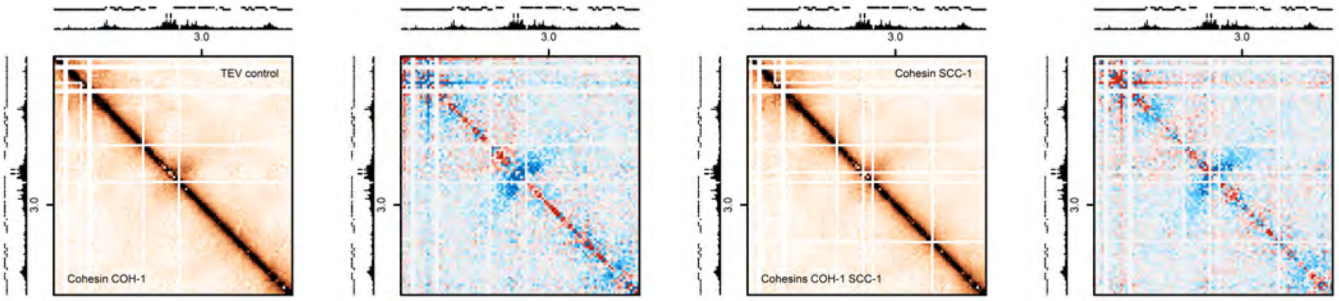

chrII:5233001-5433000

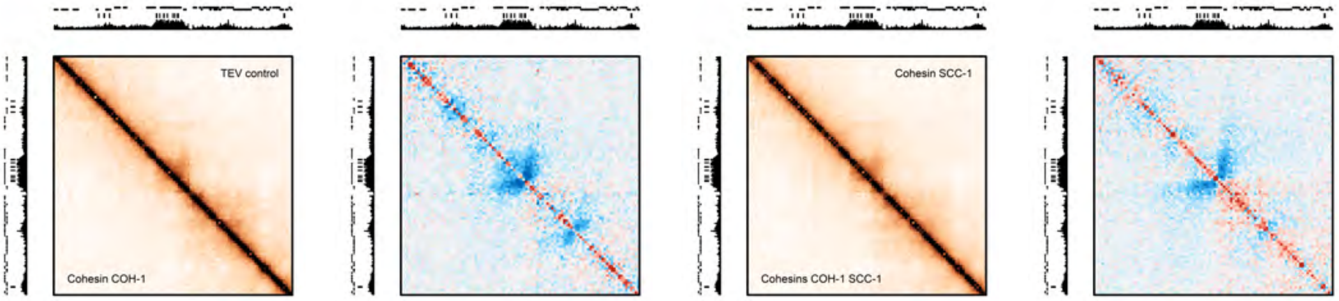

chrI:7767001-7967000

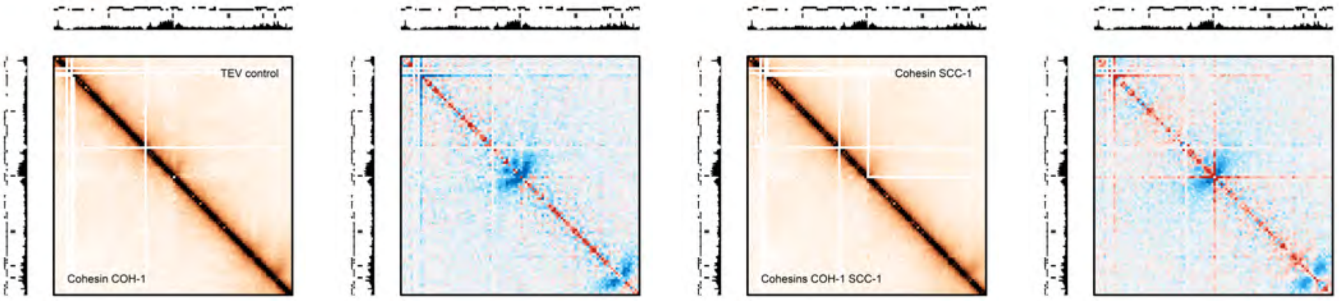

chrI:13271001-13471000

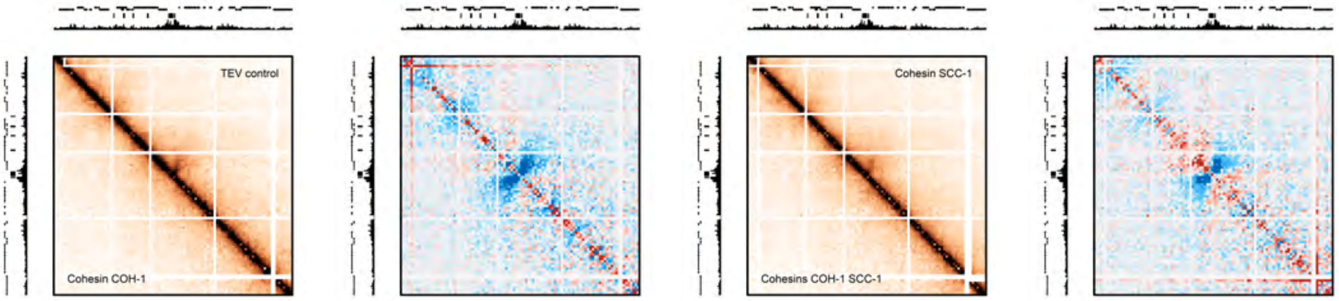

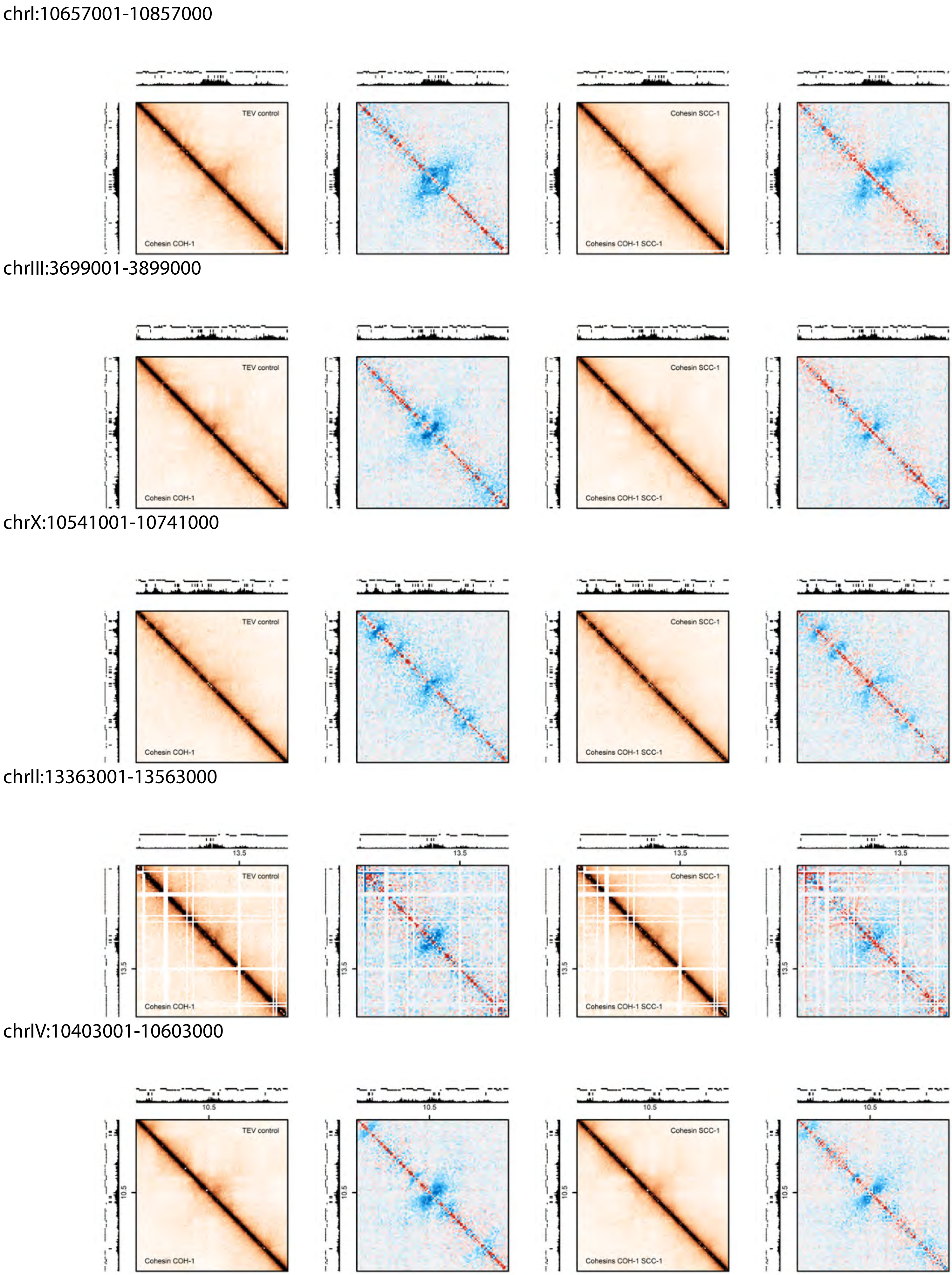
chrI:10657001-10857000
TEV control
Cohesin COH-1
Cohesin SCC-1
Cohesins COH-1 SCC-1
chrIII:3699001-3899000
TEV control
Cohesin COH-1
Cohesin SCC-1
Cohesins COH-1 SCC-1
chrX:10541001-10741000
TEV control
Cohesin COH-1
Cohesin SCC-1
Cohesins COH-1 SCC-1
chrII:13363001-13563000
13.5
TEV control
Cohesin COH-1
Cohesin SCC-1
Cohesins COH-1 SCC-1
chrIV:10403001-10603000
10.5
TEV control
Cohesin COH-1
Cohesin SCC-1
Cohesins COH-1 SCC-1

chrX:16833001-17033000

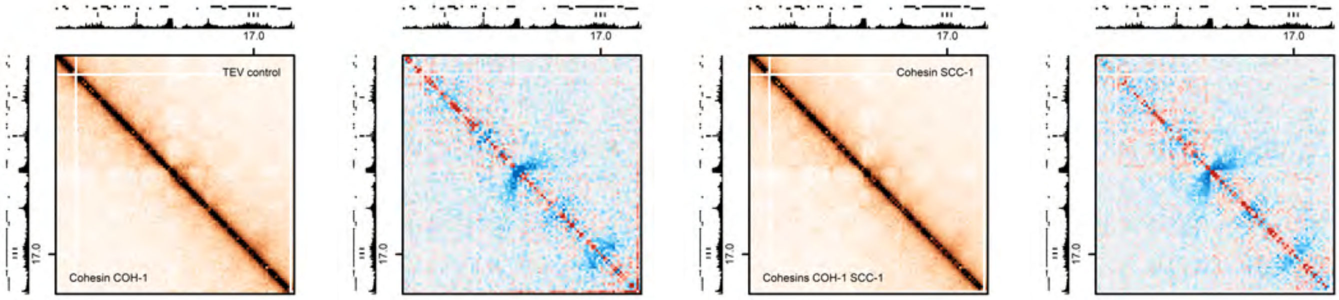

chrI:-62999-137000

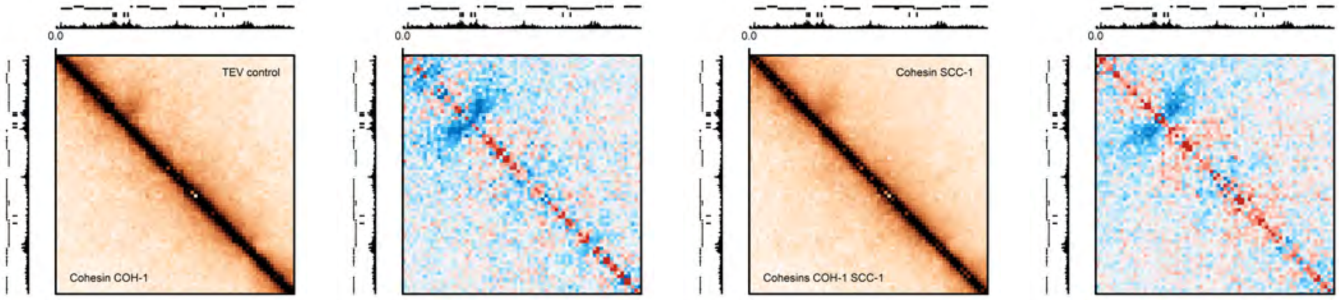

chrIII:7627001-7827000

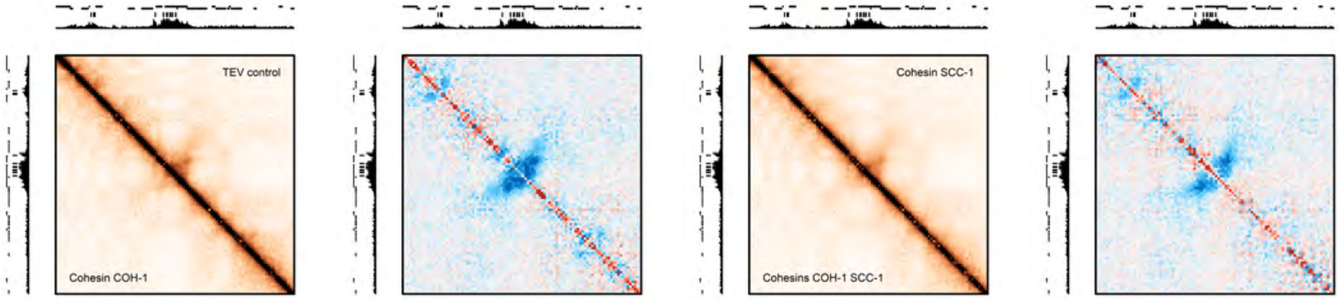

chrI:5281001-5481000

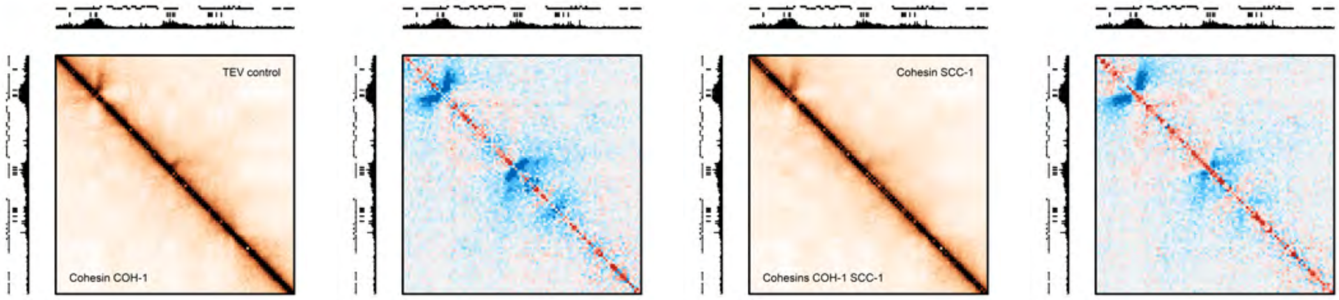

chrIII:9601001-9801000

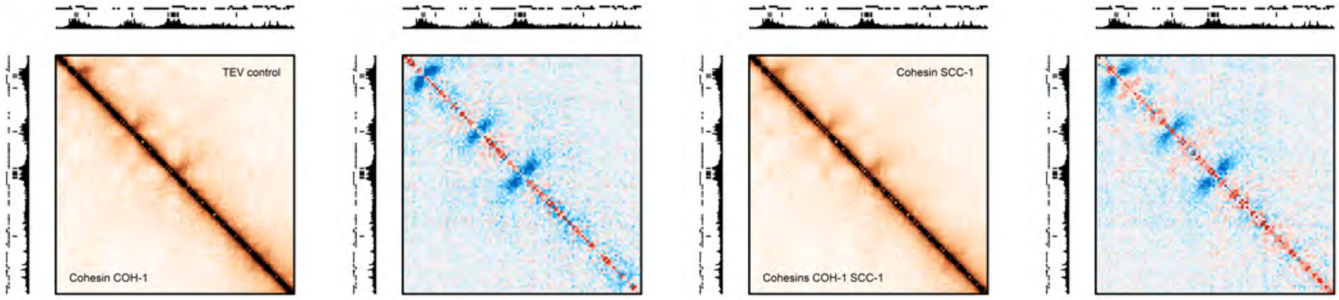

chrIII:6261001-6461000

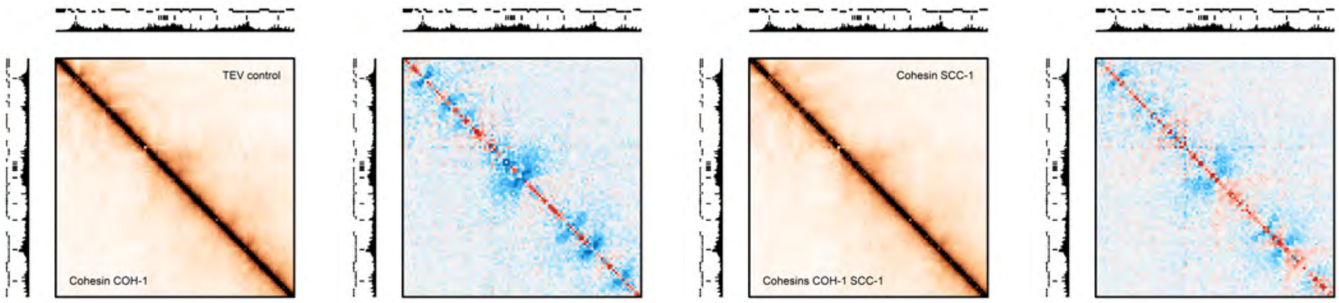

chrIV:2503001-2703000

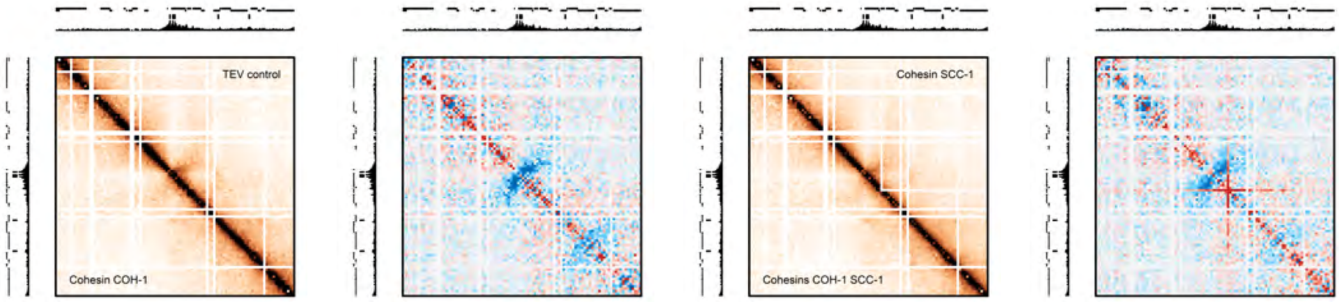

chrIII:251001-451000

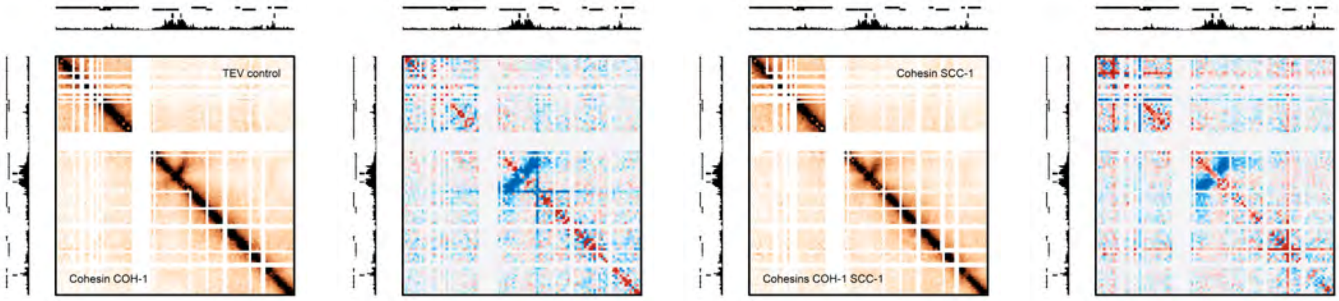

chrIV:1553001-1753000

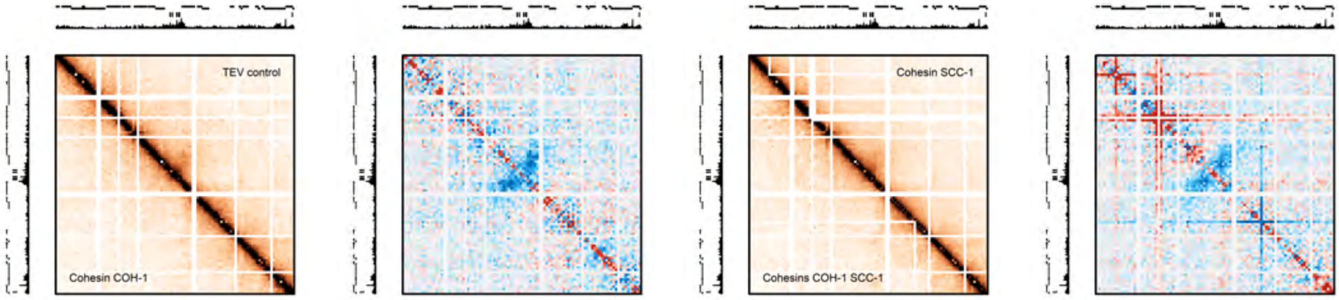

chrX:12413001-12613000

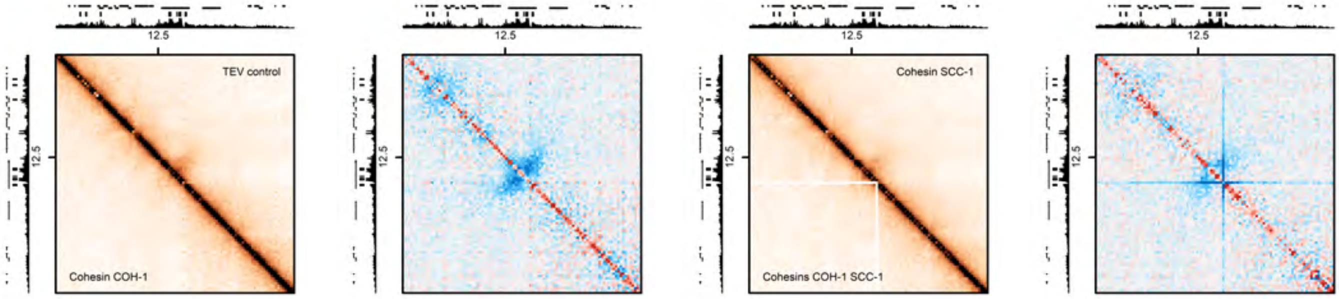

chrV:12987001-13187000

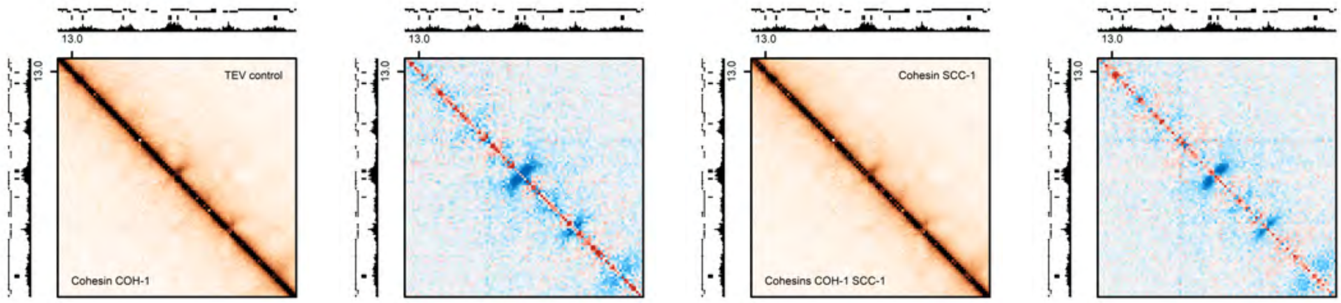

chrI:1737001-1937000

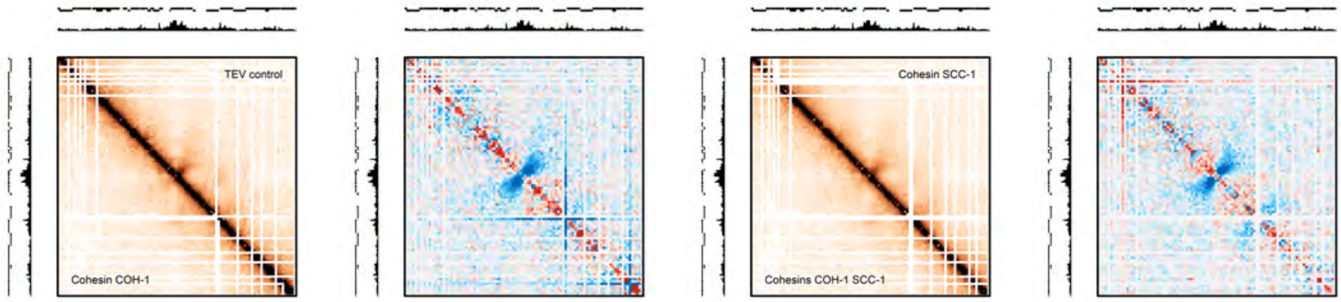

chrII:13943001-14143000

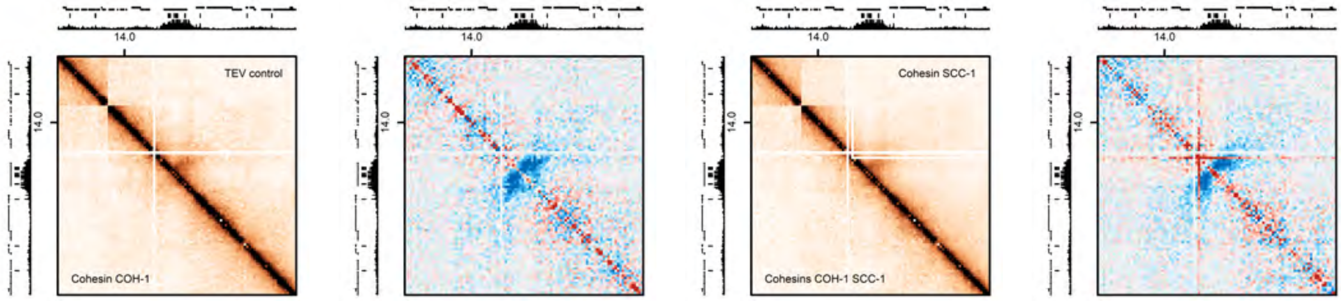

chrIV:7933001-8133000

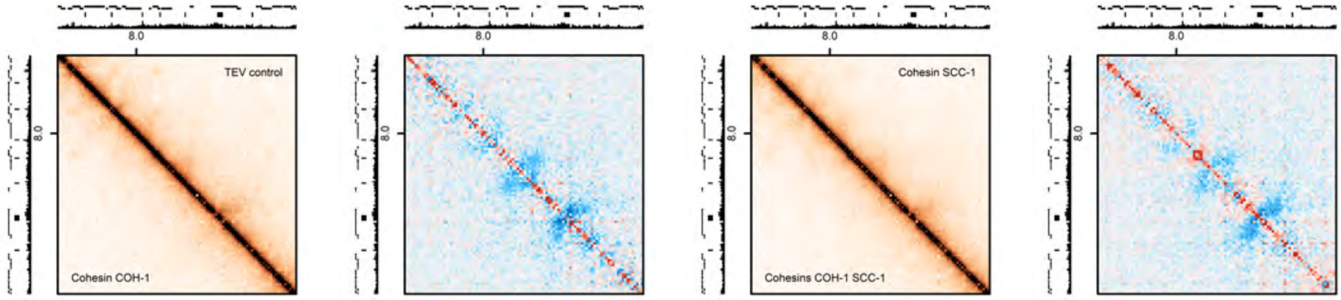

chrII:11533001-11733000

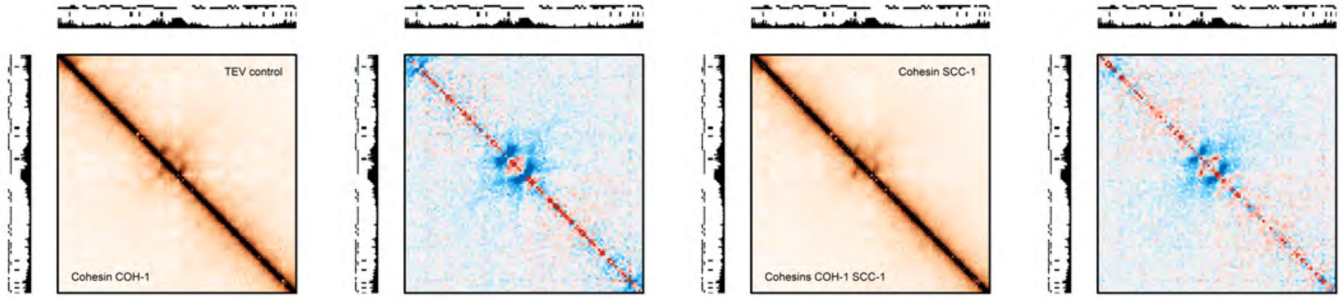

chrIV:14515001-14715000

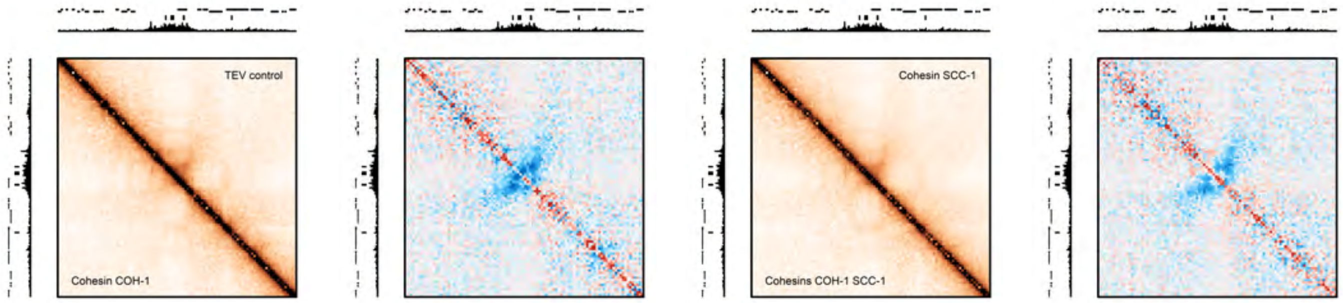

chrII:7793001-7993000

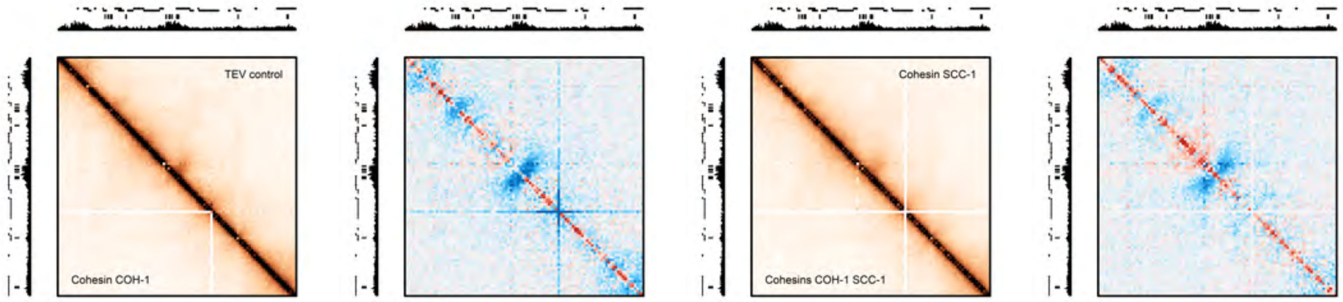

chrX:2495001-2695000

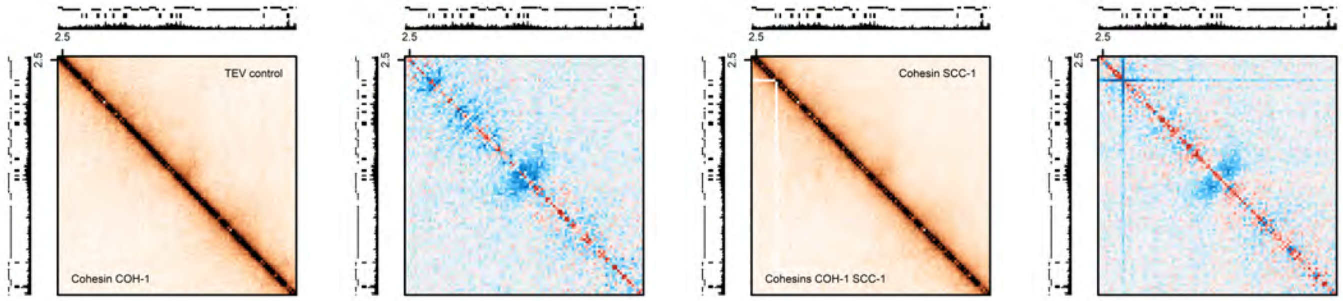

chrV:8325001-8525000

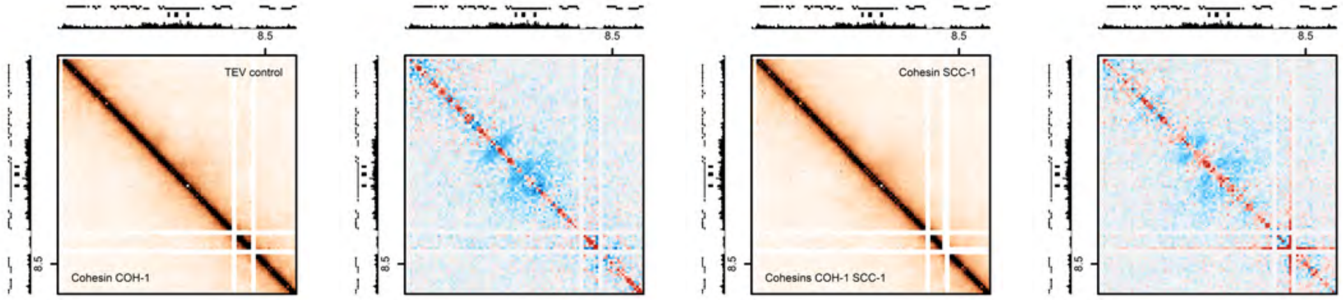

chrIV:3927001-4127000

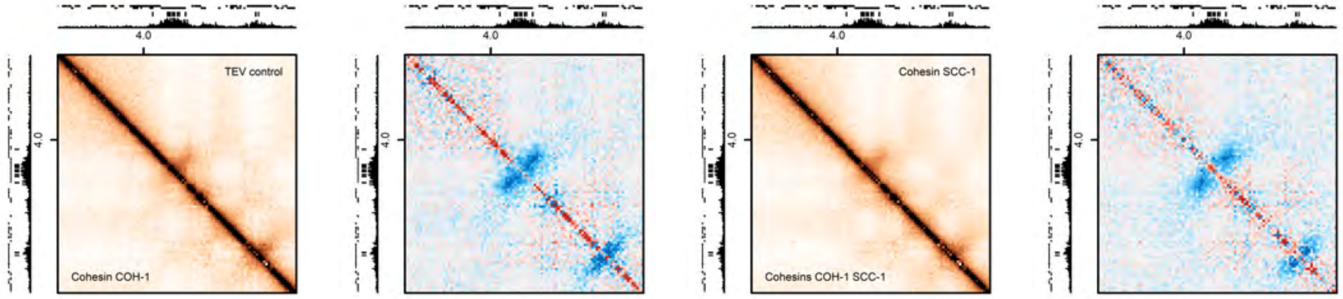

chrX:6153001-6353000

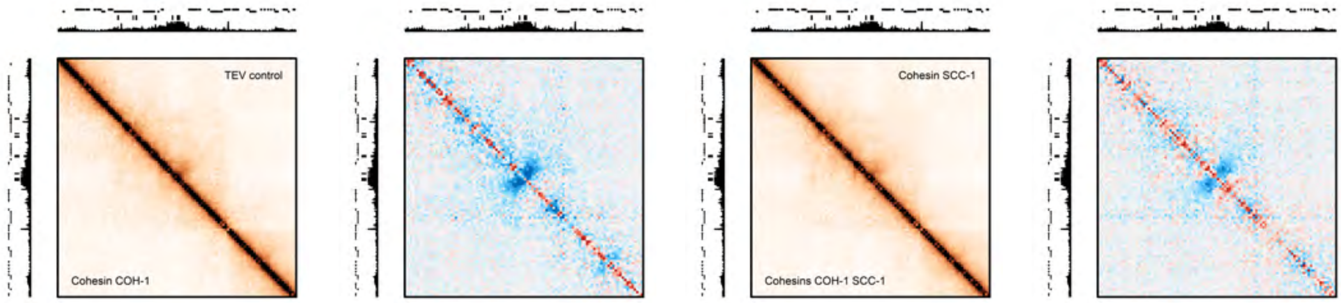

chrIII:1831001-2031000

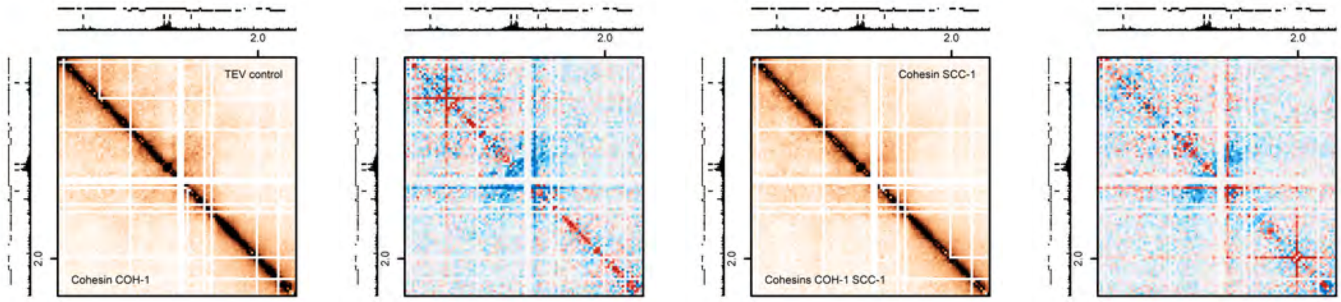

chrI:9281001-9481000

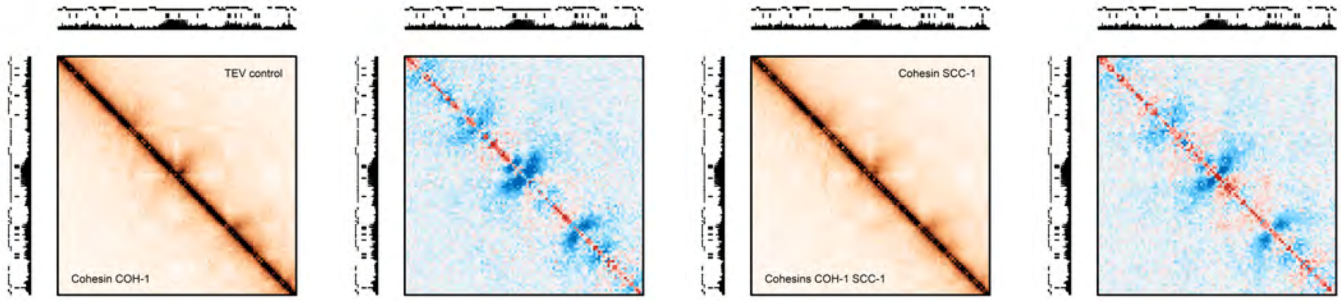

chrV:14749001-14949000

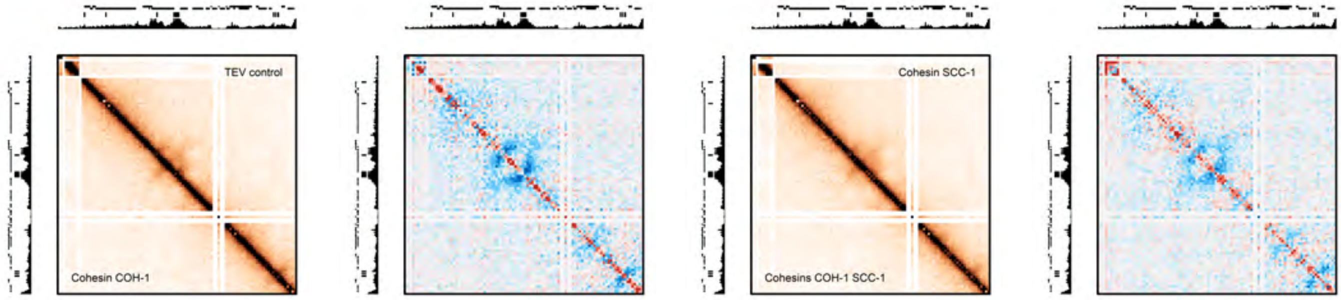

chrII:12697001-12897000

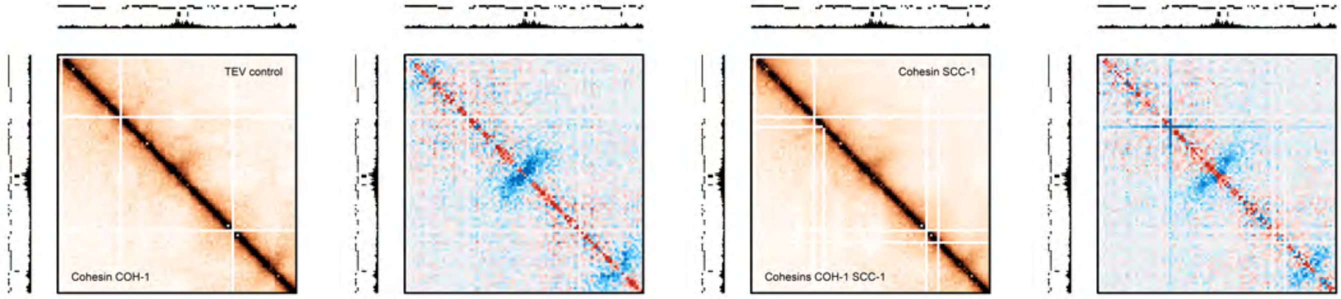

chrV:10369001-10569000

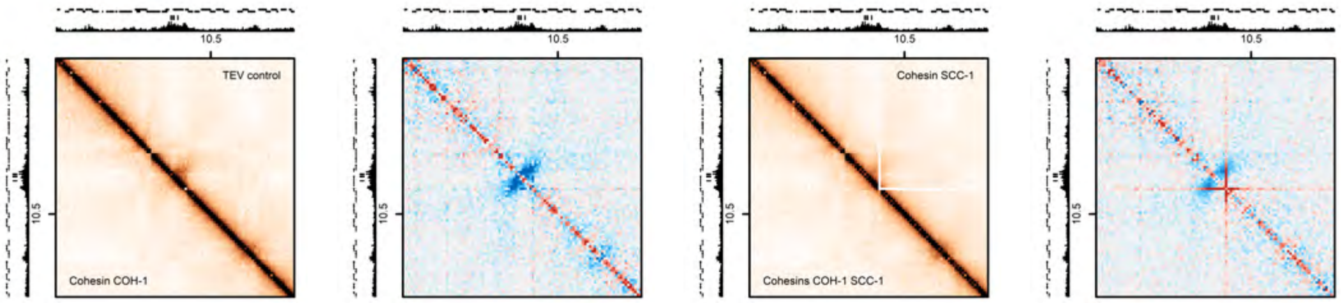

chrX:12077001-12277000

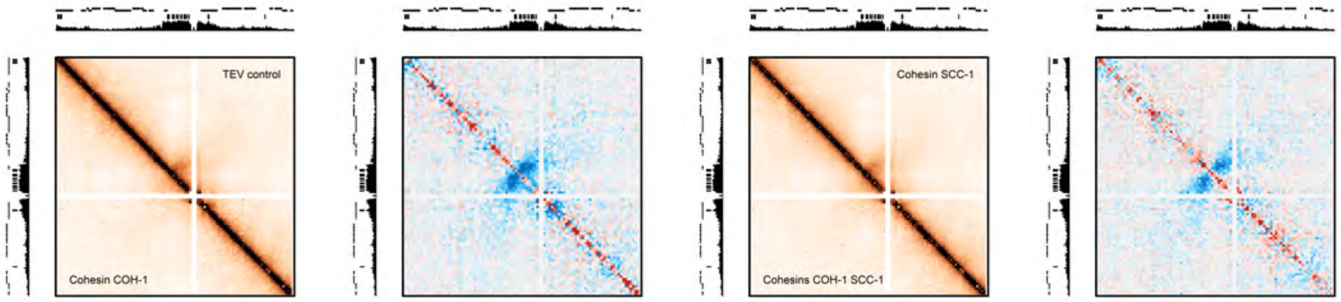

chrI:3621001-3821000

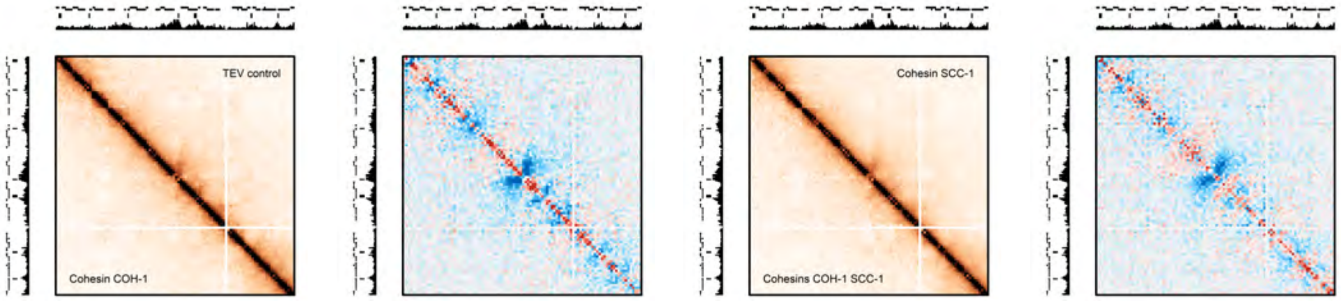

chrIII:4401001-4601000

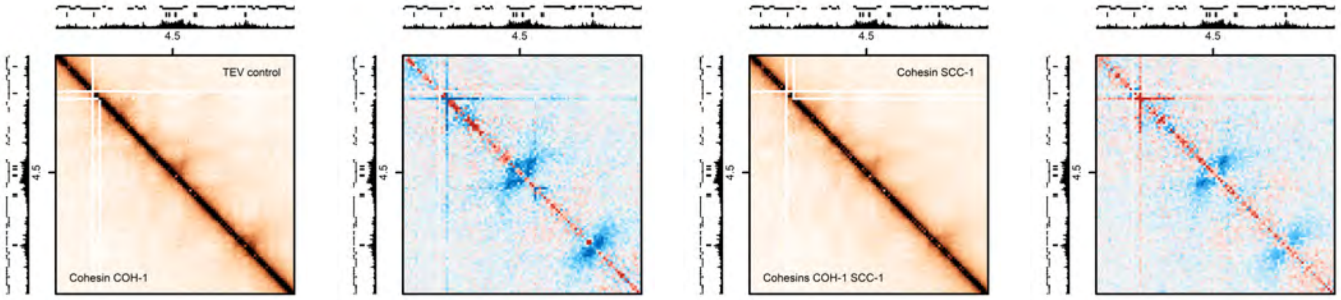

chrV:-84999-115000

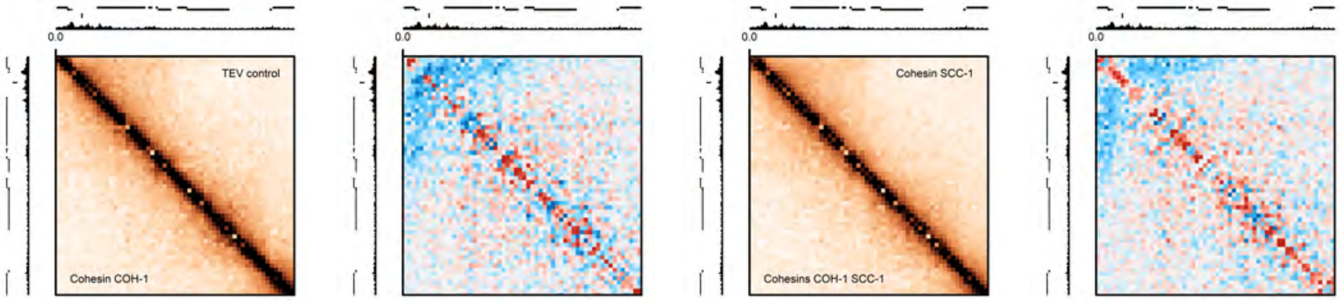

chrIV:16391001-16591000

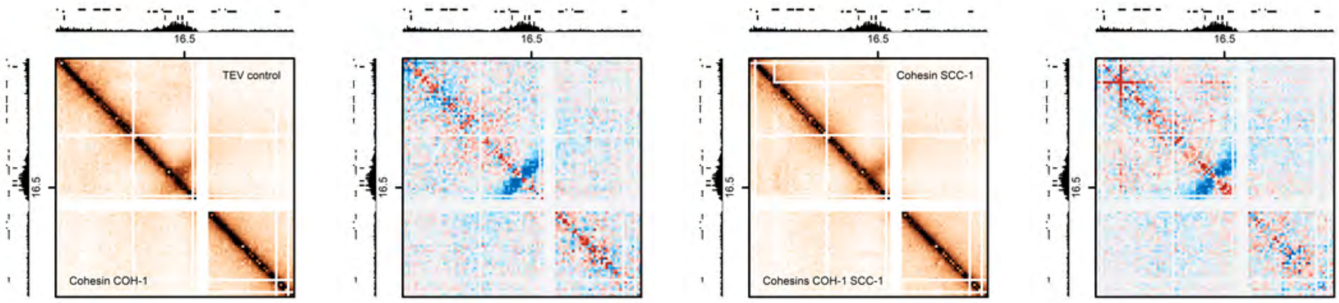

chrIII:7053001-7253000

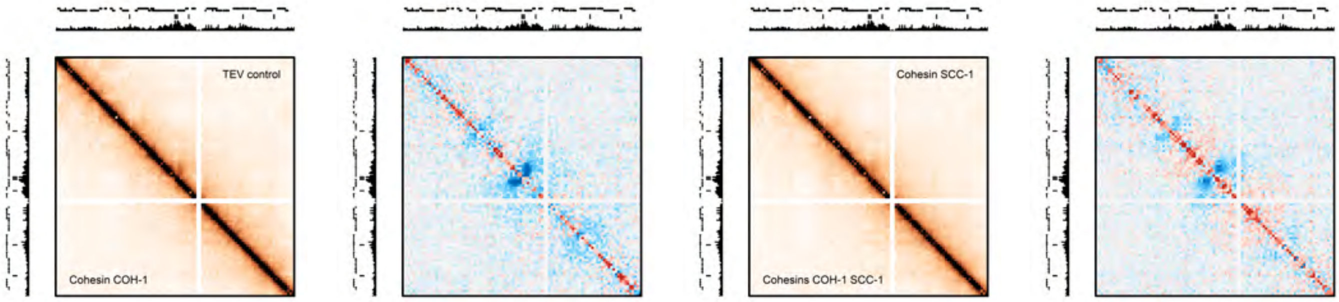

chrI:6479001-6679000

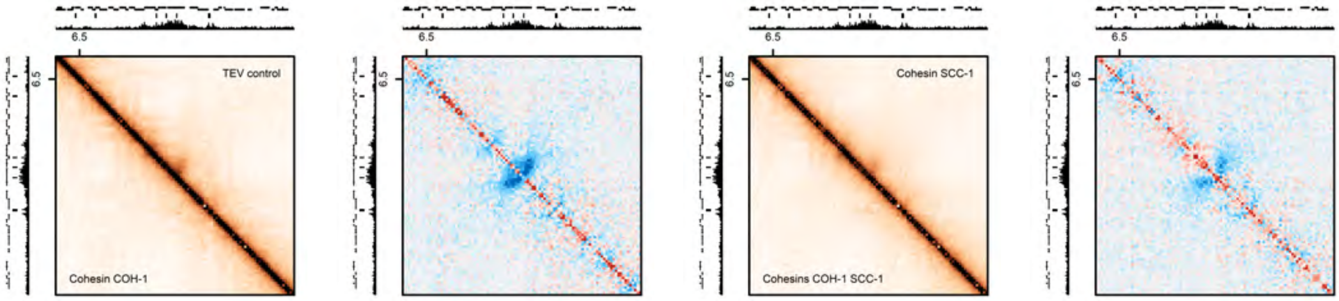

chrX:15177001-15377000

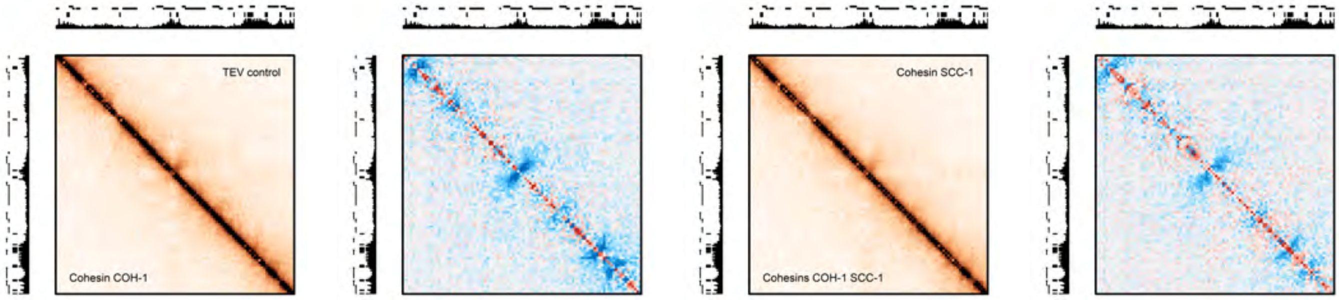

chrIV:12931001-13131000

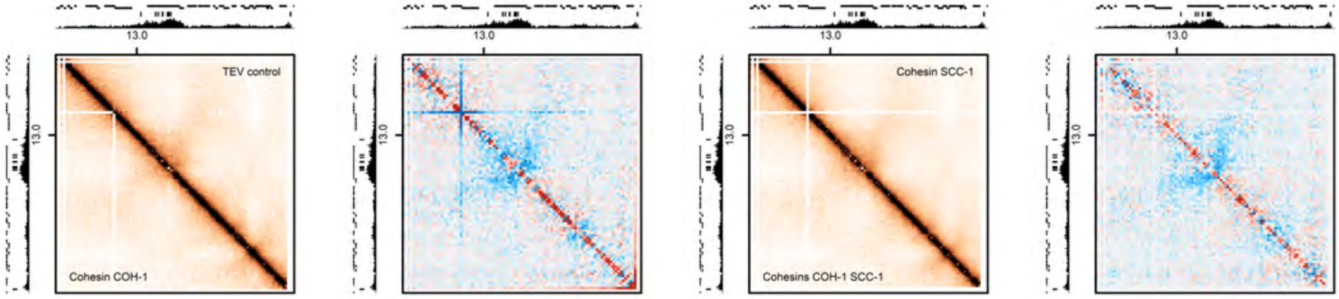

chrII:3859001-4059000

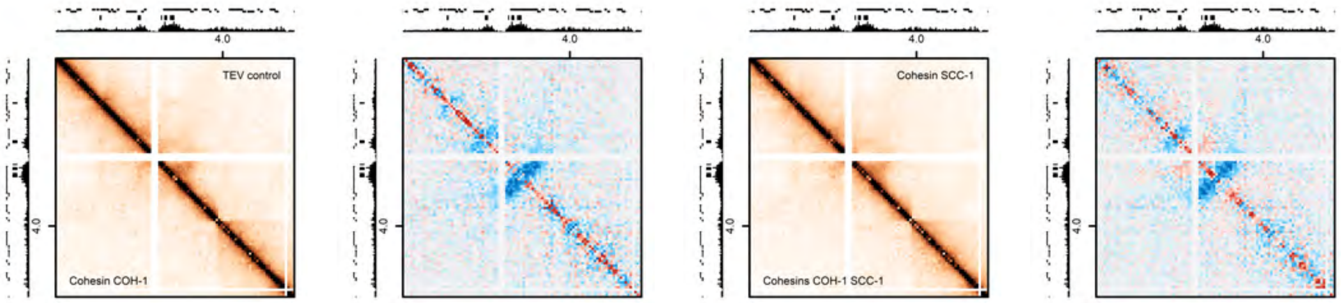

chrV:14403001-14603000

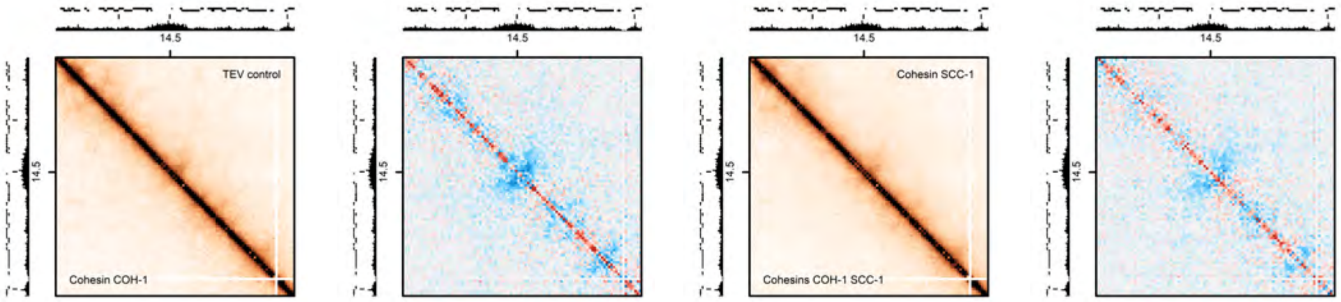

chrX:5717001-5917000

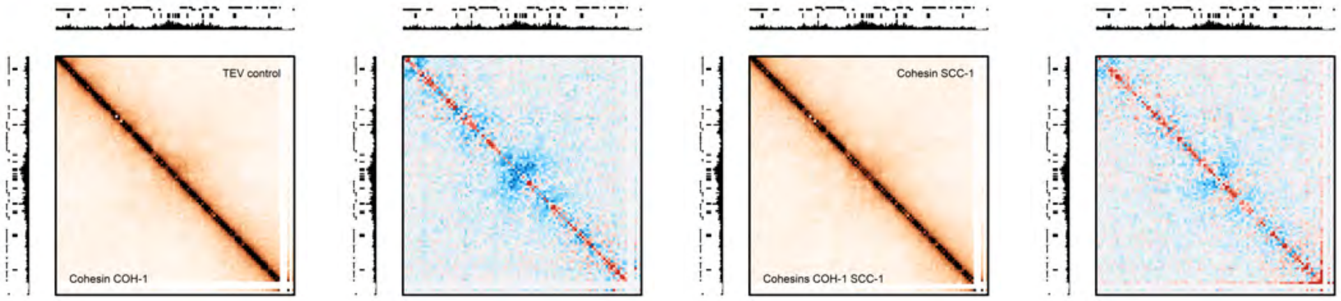

chrIII:11101001-11301000

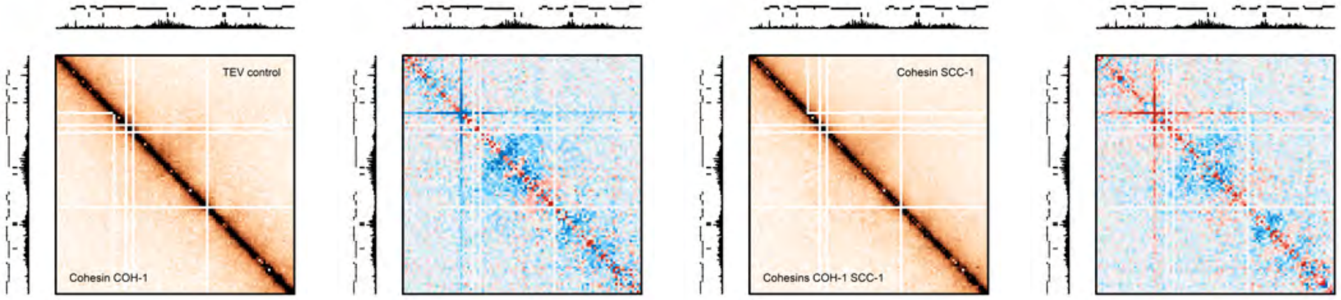

chrIV:13789001-13989000

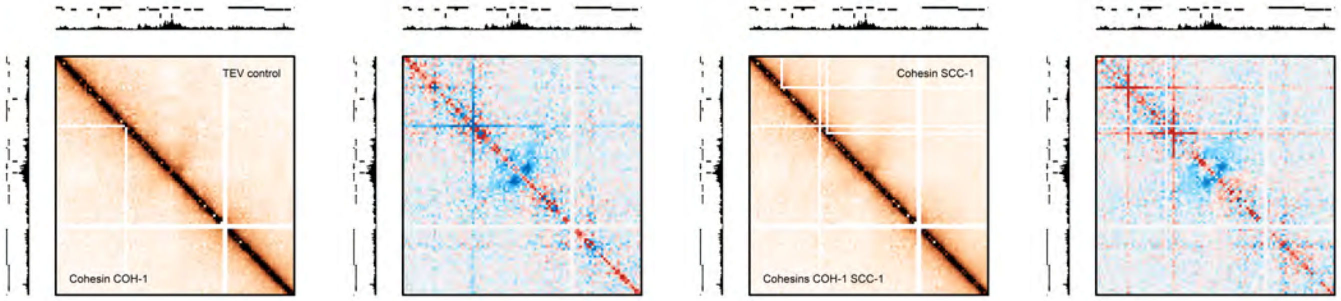

chrX:11375001-11575000

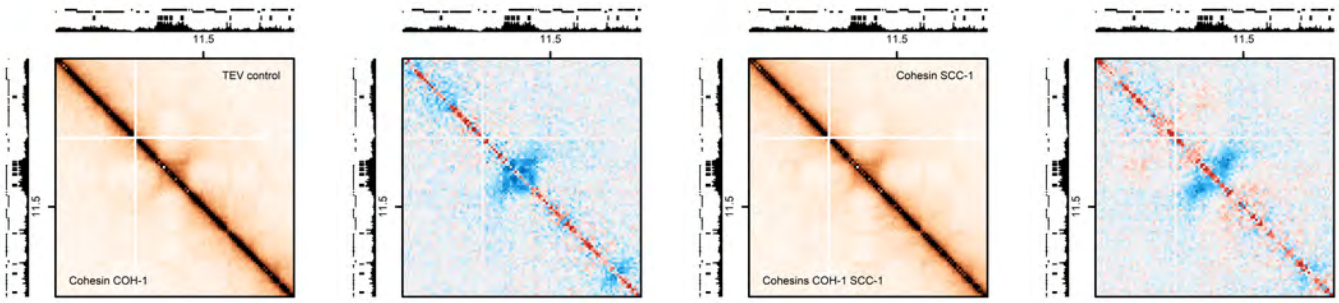

chrIII:10535001-10735000

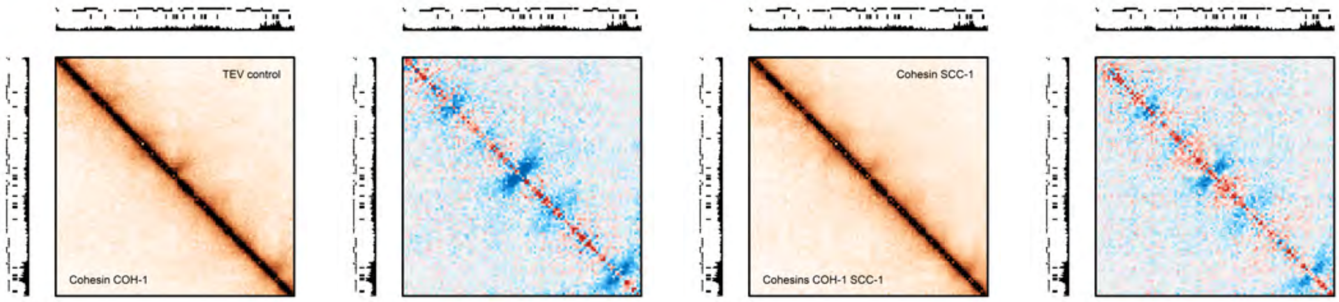

chrI:9625001-9825000

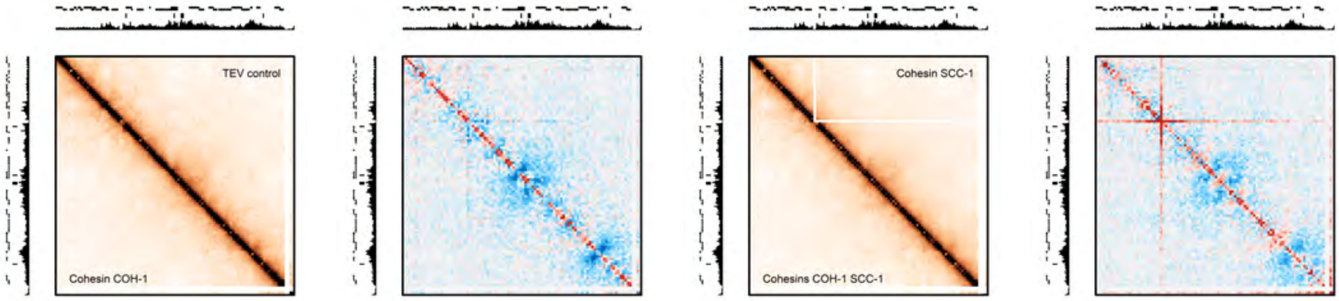

chrIV:5653001-5853000

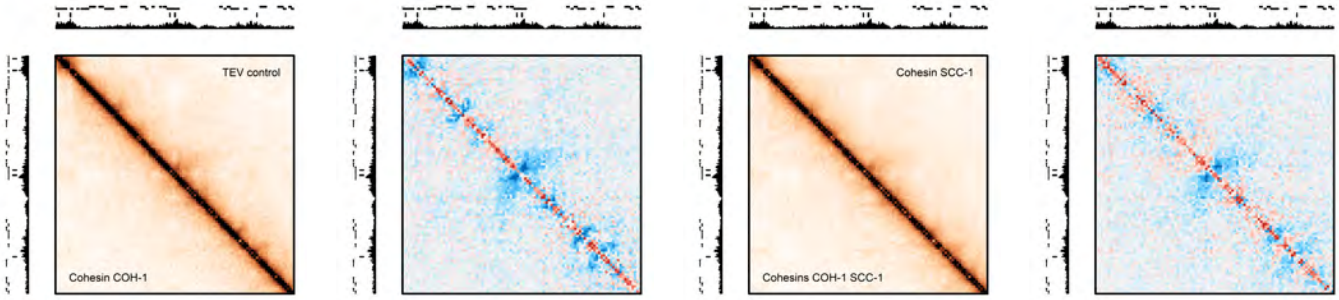

chrI:969001-1169000

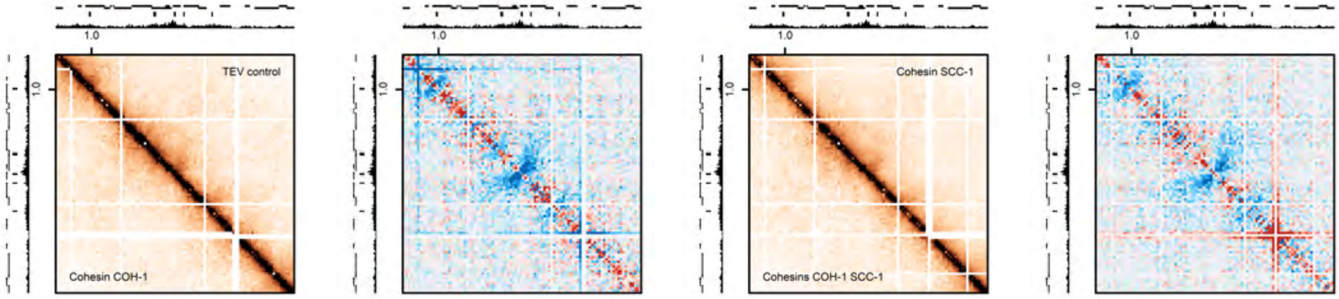

chrV:5745001-5945000

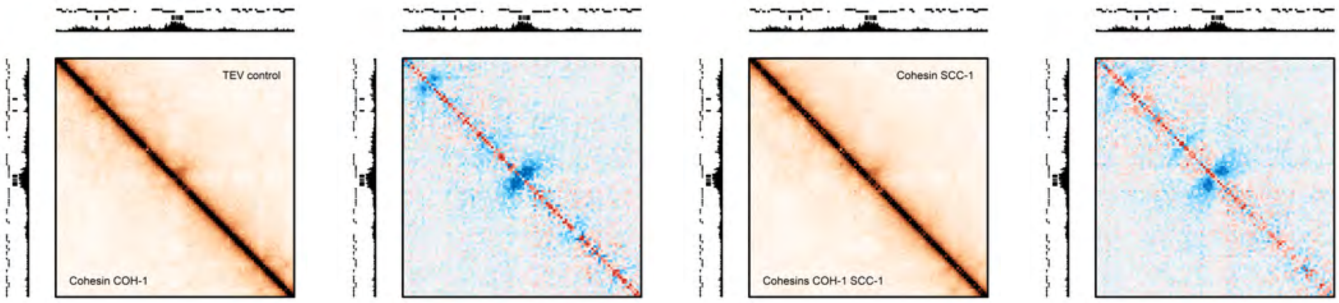

chrII:13191001-13391000

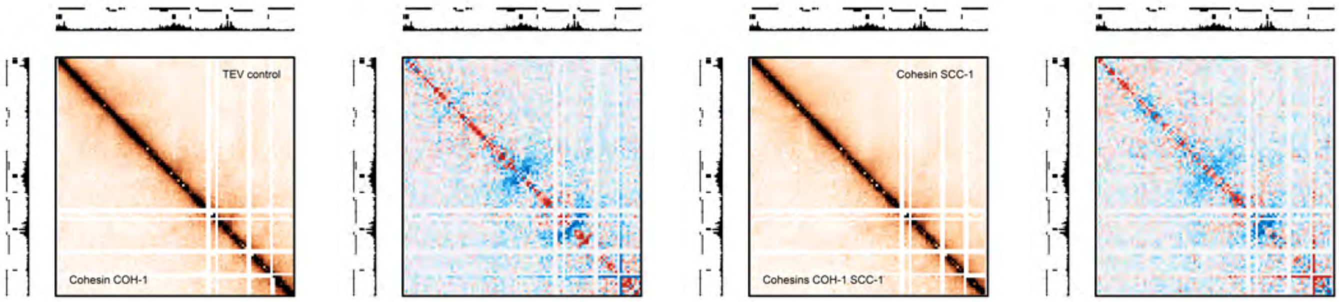

chrIII:11589001-11789000

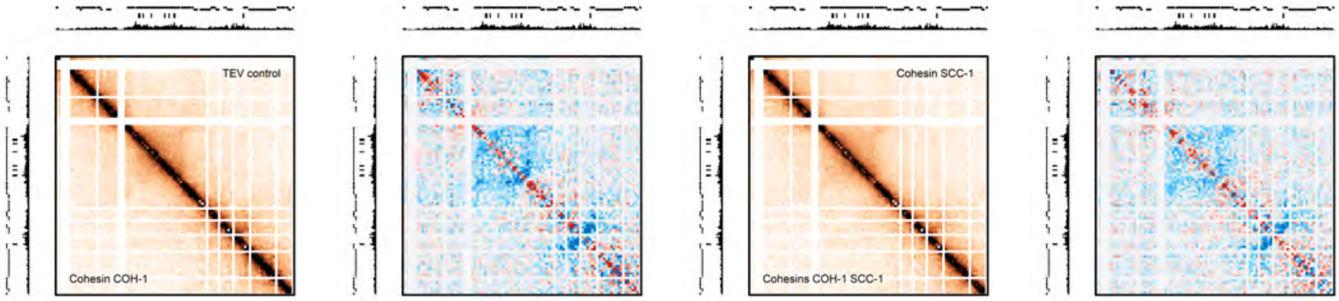

chrX:13065001-13265000

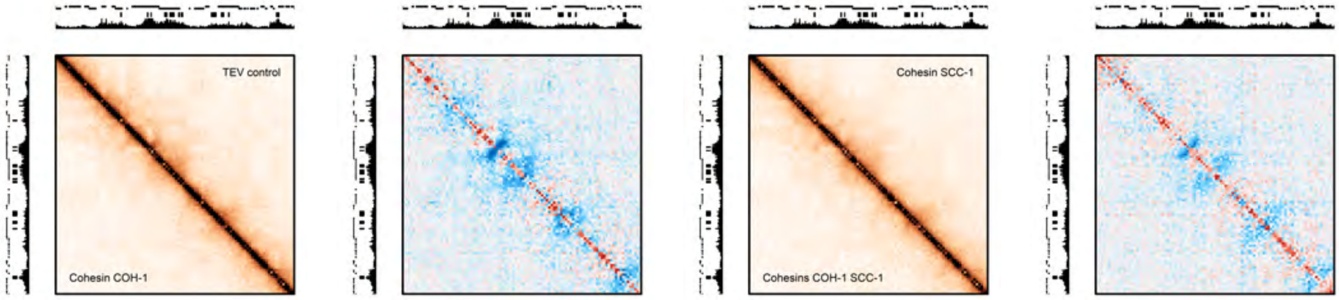

chrI:2487001-2687000

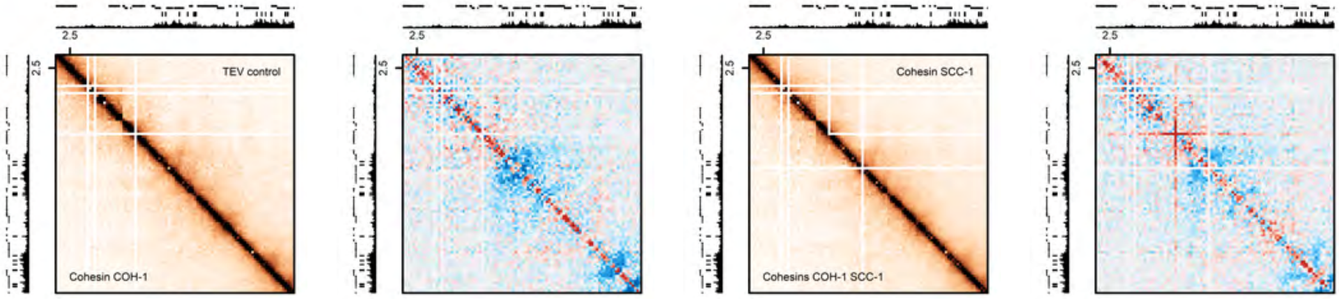

chrIII:8025001-8225000

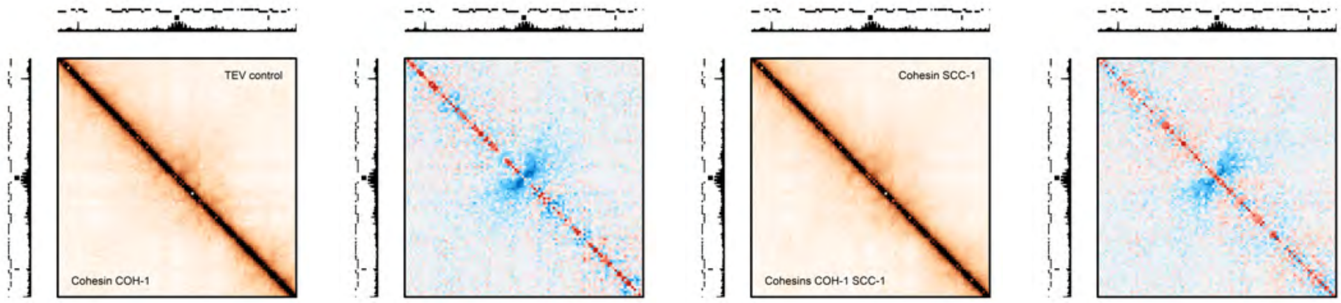

chrV:20679001-20879000

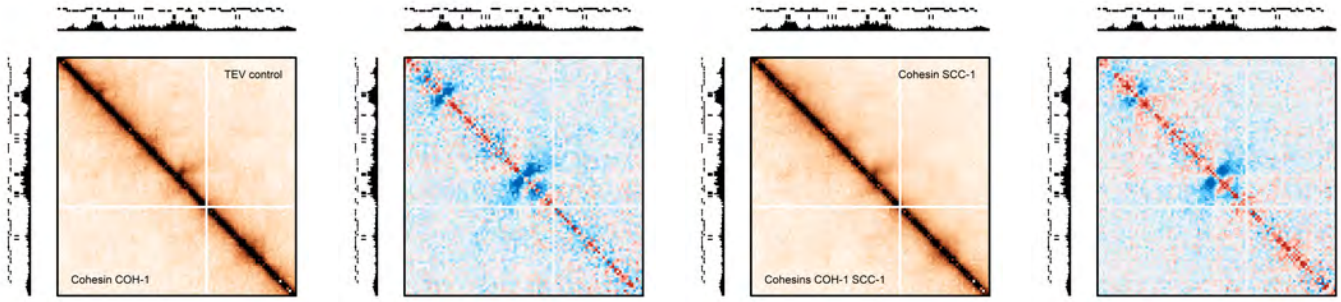

chrI:1921001-2121000

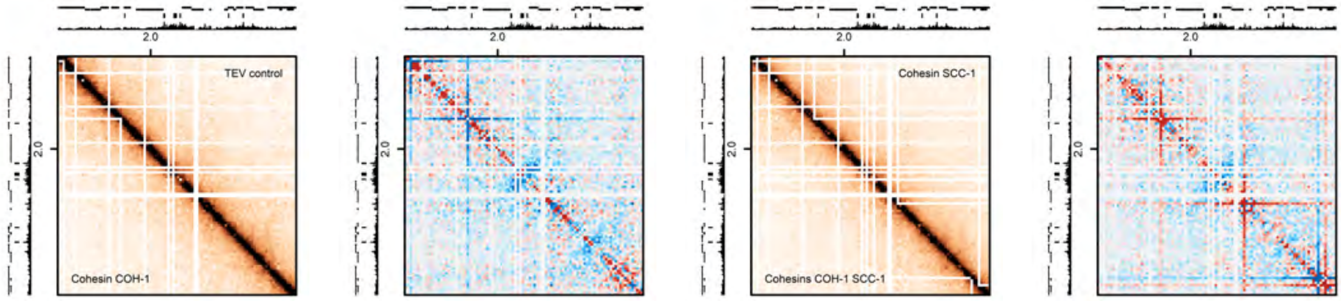

chrX:10087001-10287000

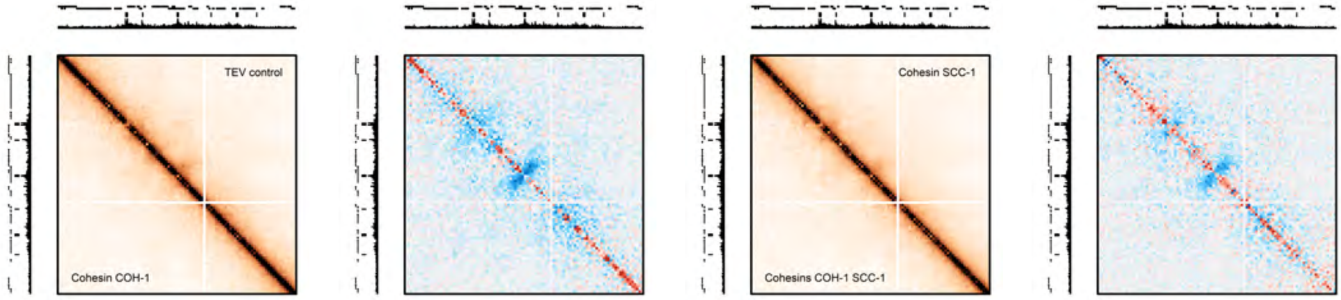

chrII:5835001-6035000

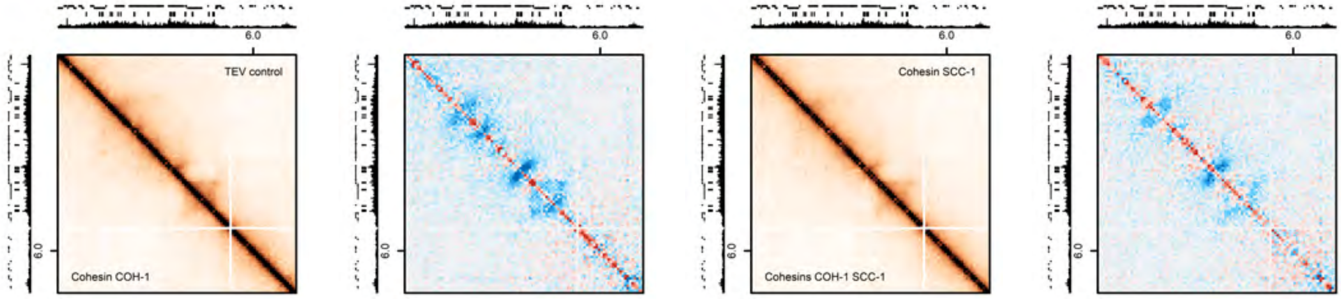

chrIII:6891001-7091000

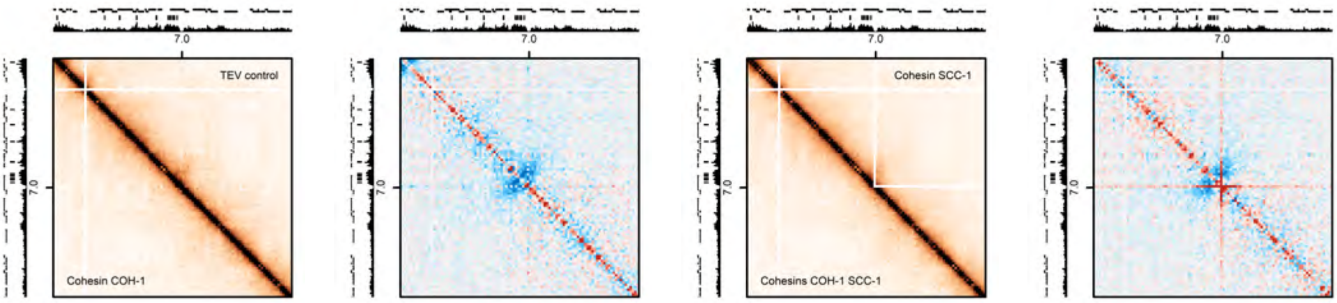

chrI:12719001-12919000

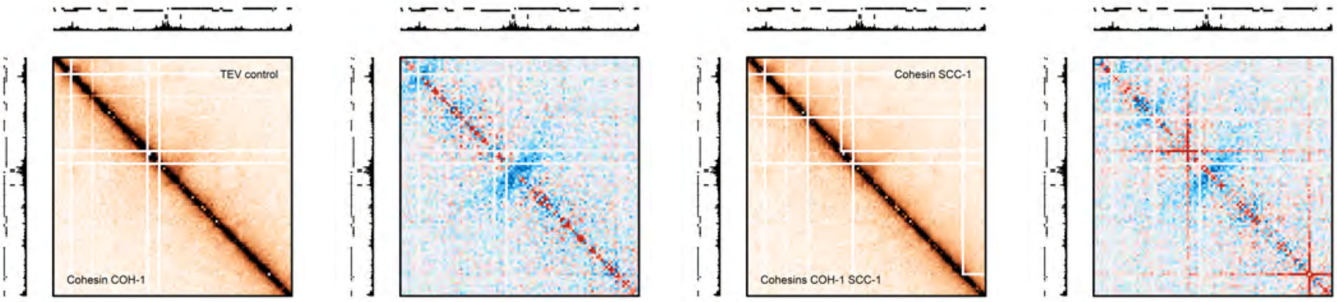

chrII:1139001-1339000

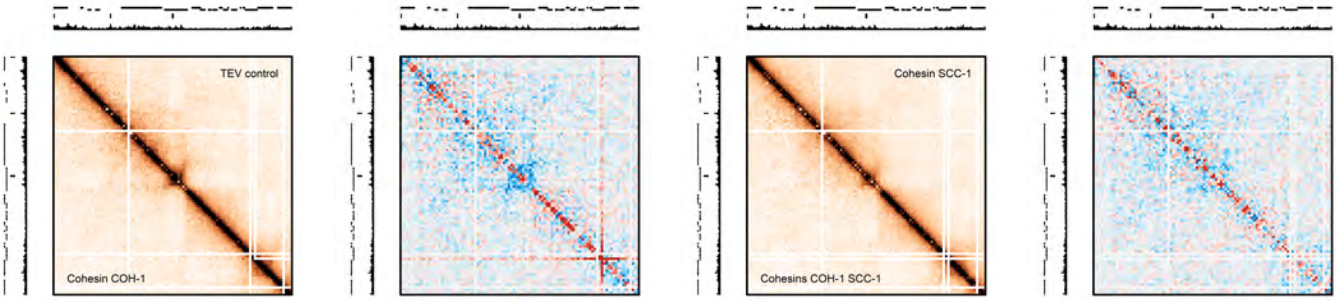

chrV:10619001-10819000

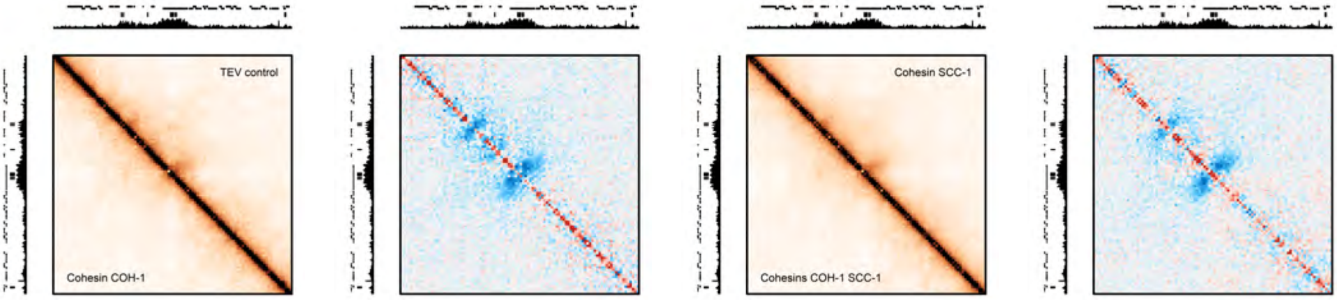

chrIII:6037001-6237000

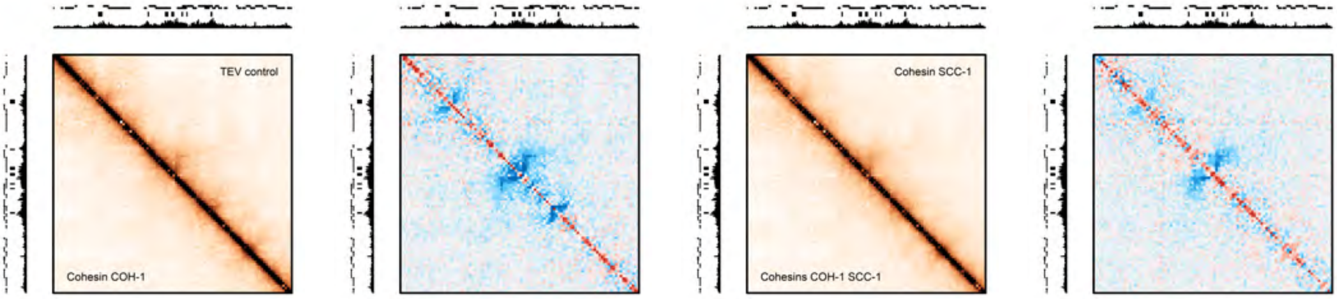

chrV:3595001-3795000

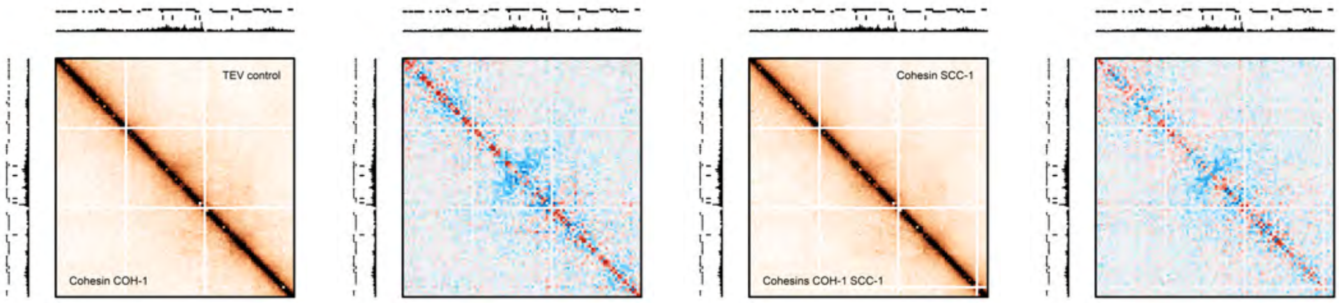

chrIV:2121001-2321000

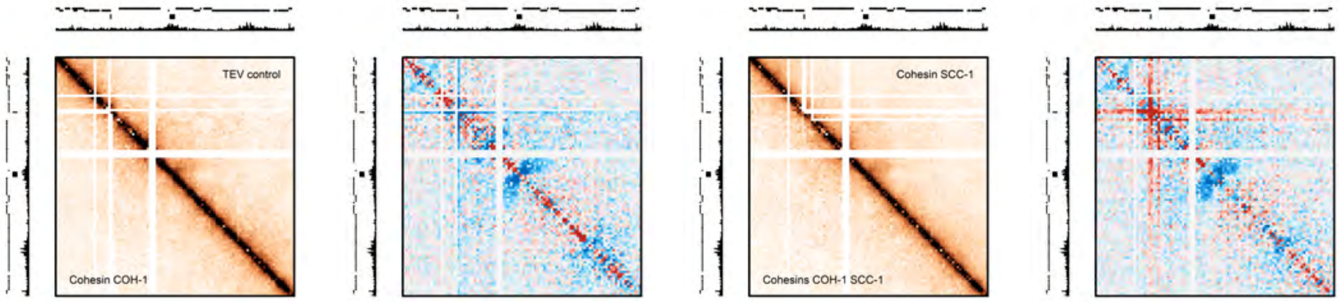

chrII:12925001-13125000

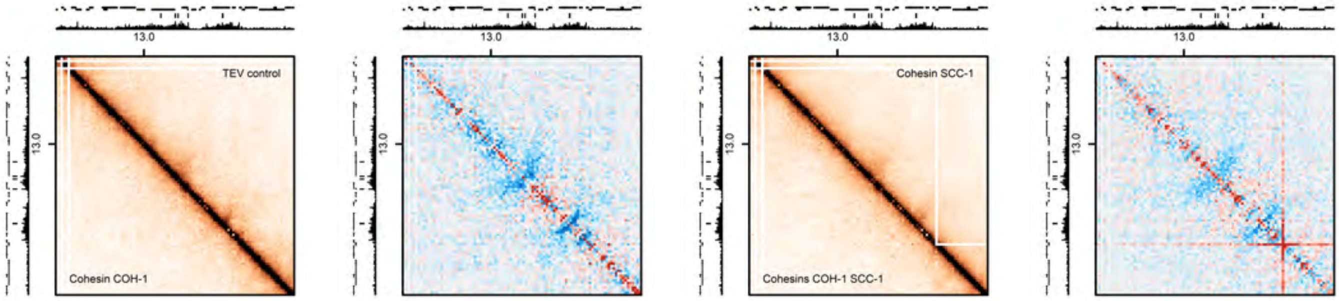

chrII:14503001-14703000

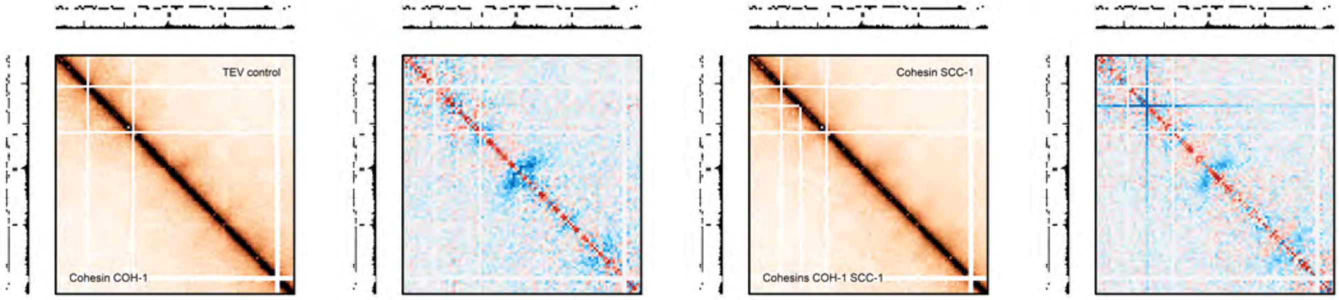

chrIV:10719001-10919000

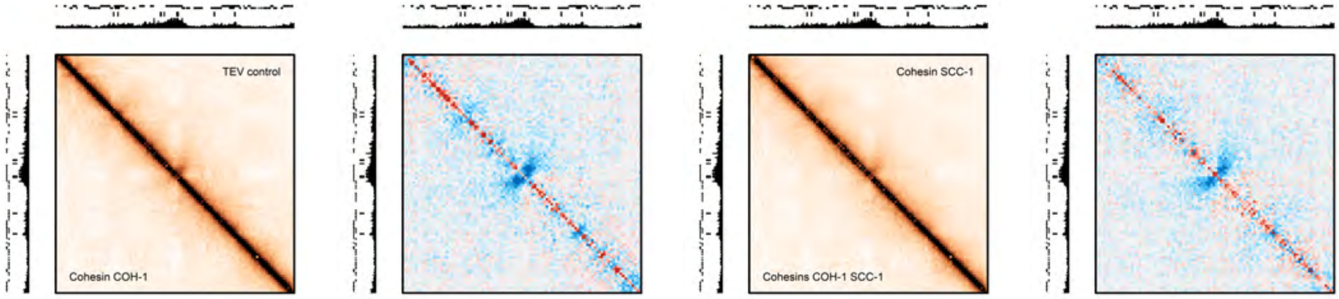

chrIV:2895001-3095000

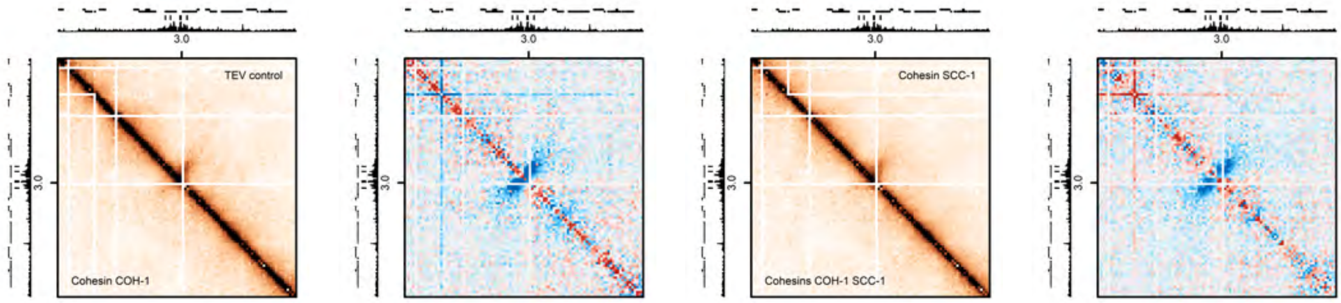

chrII:9505001-9705000

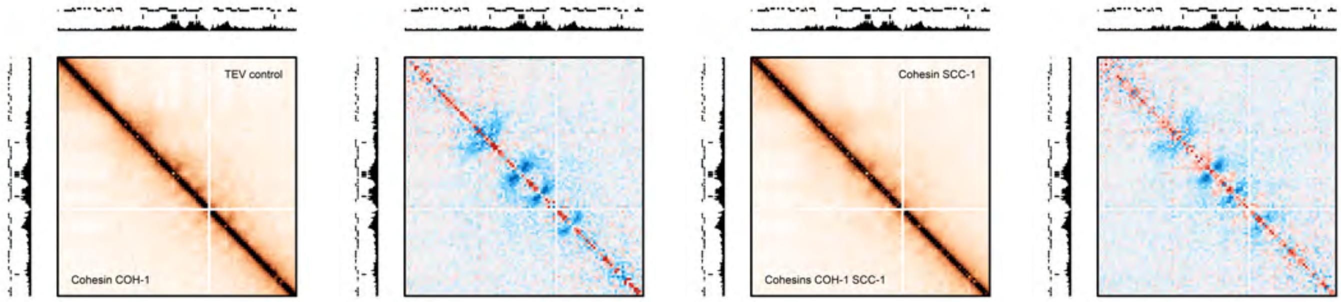

chrV:4405001-4605000

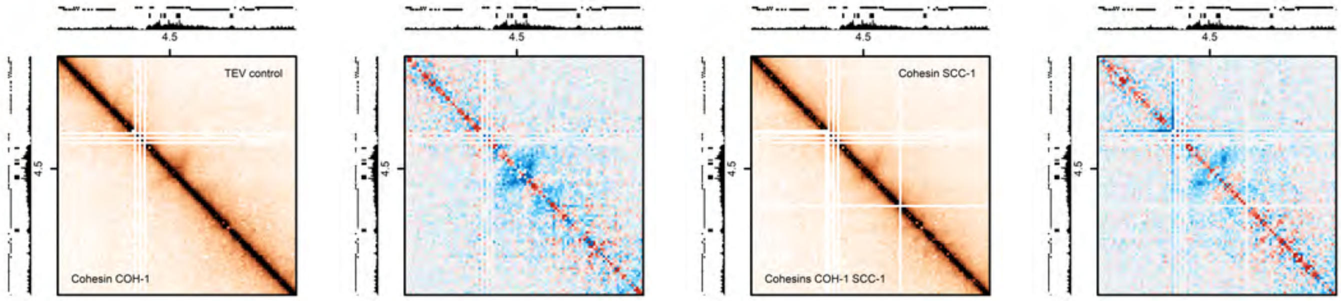

chrI:14837001-15037000

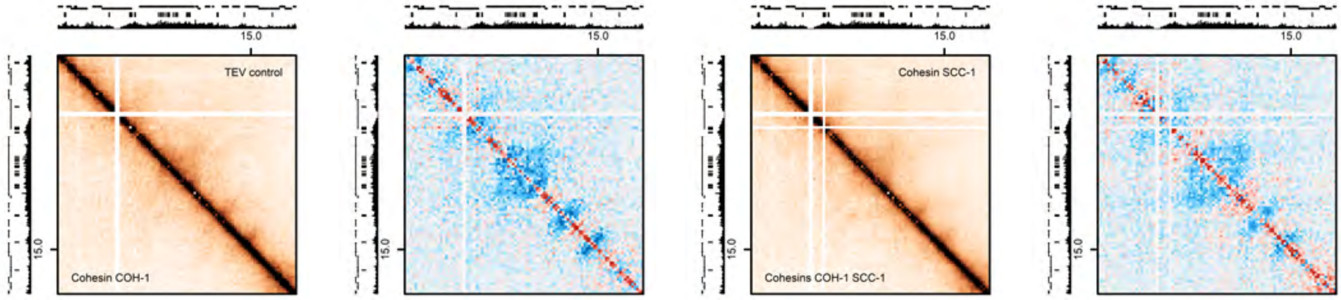

chrV:11723001-11923000

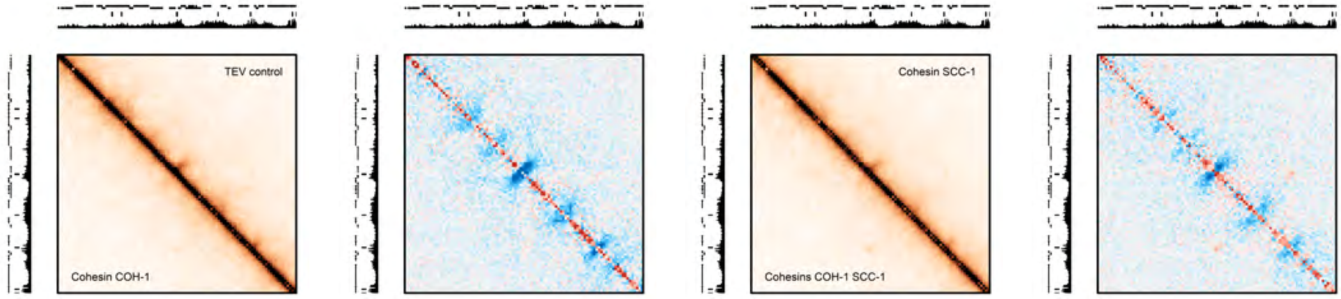

chrV:16255001-16455000

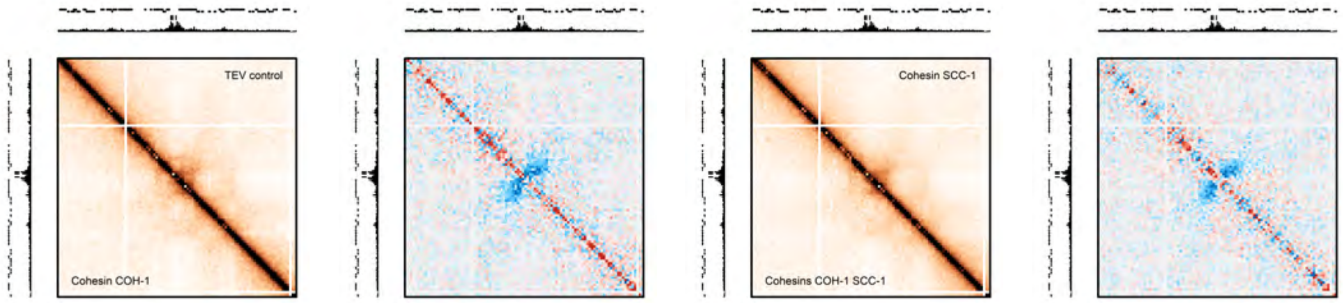

chrI:4815001-5015000

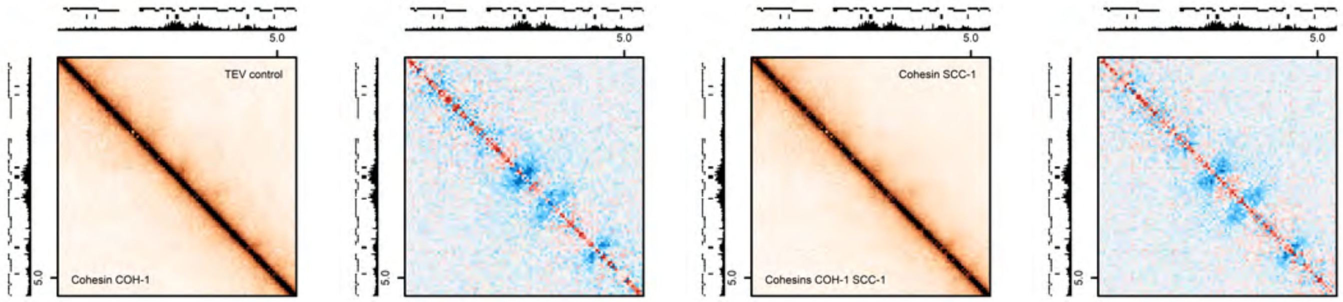

chrII:13637001-13837000

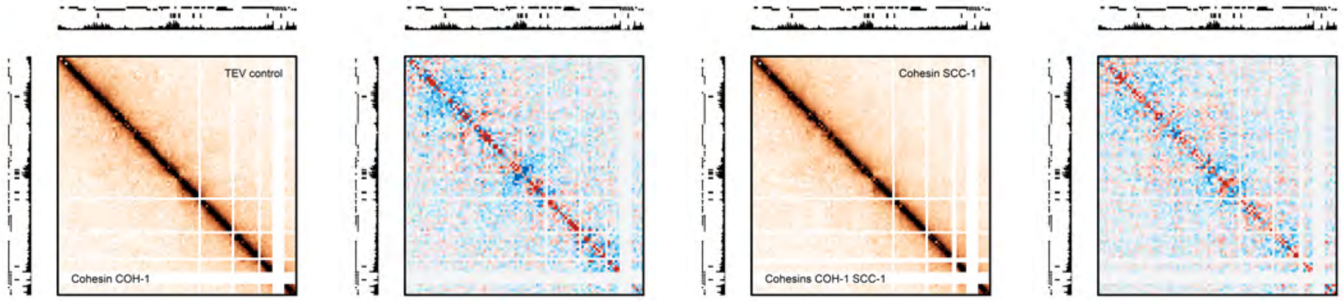

chrIV:9573001-9773000

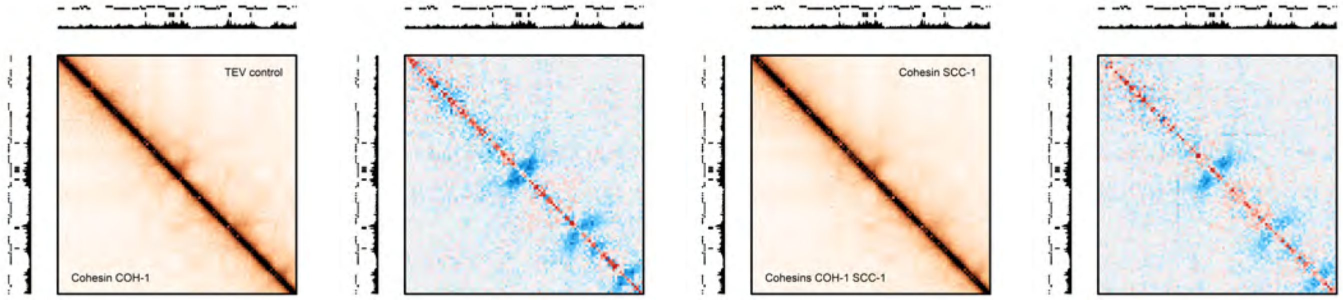

chrX:6643001-6843000

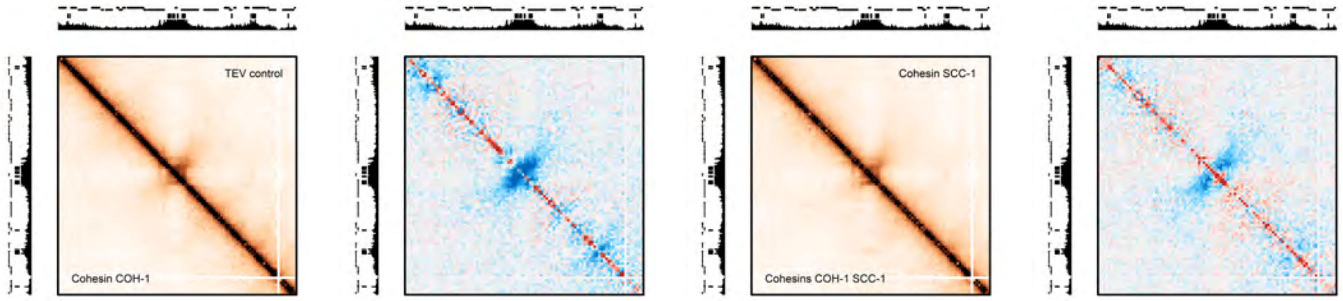

chrX:989001-1189000

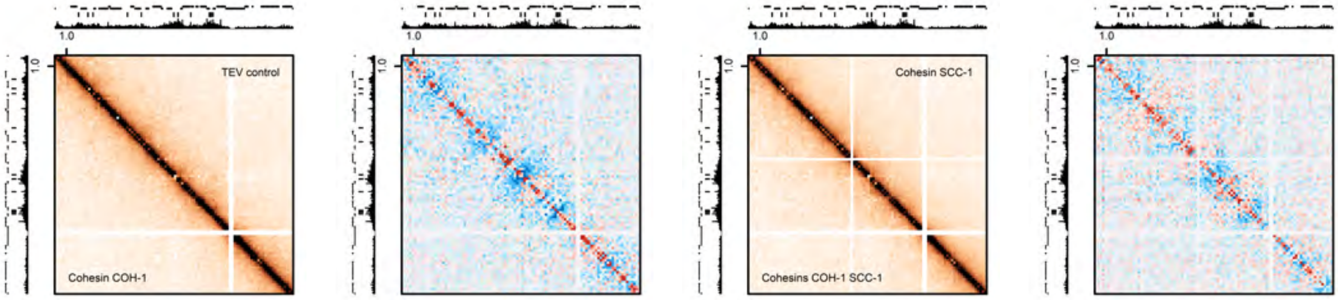

chrIII:13619001-13819000

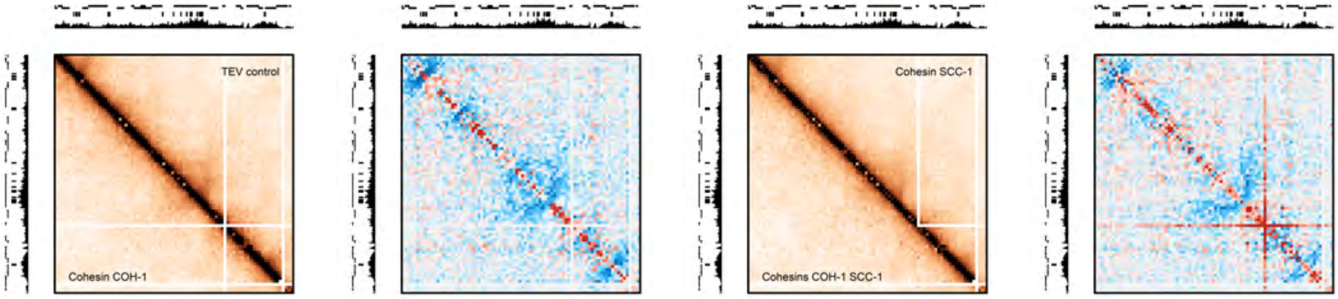

chrIV:6683001-6883000

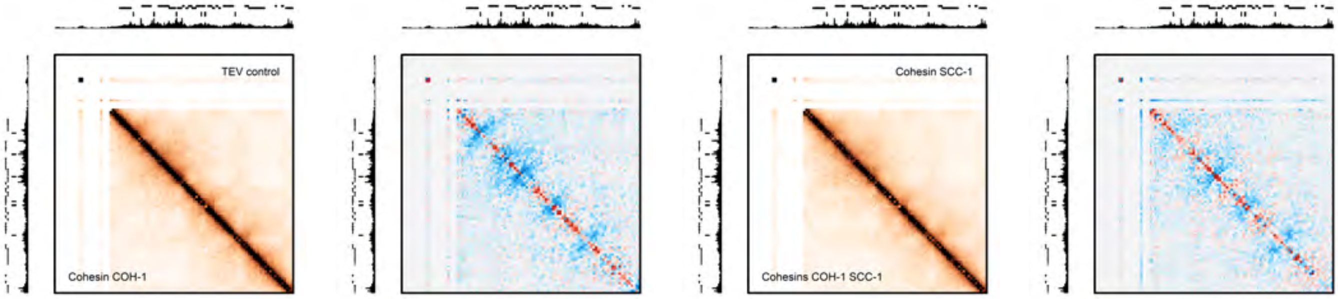

chrIV:11495001-11695000

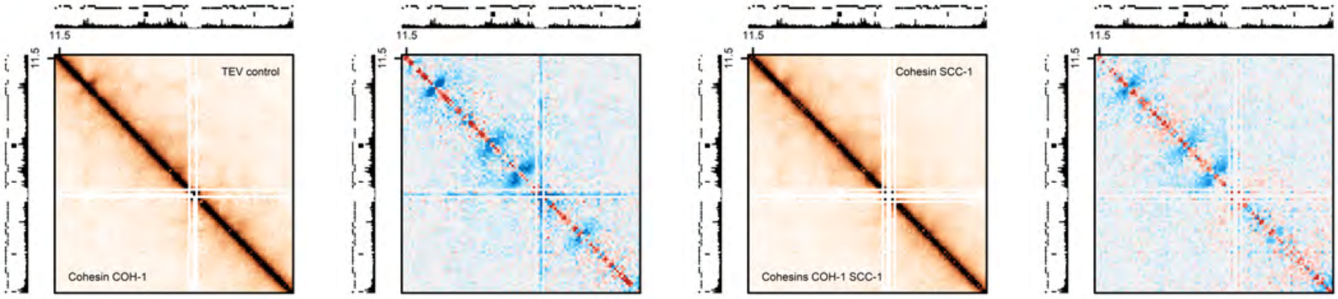

chrII:6245001-6445000

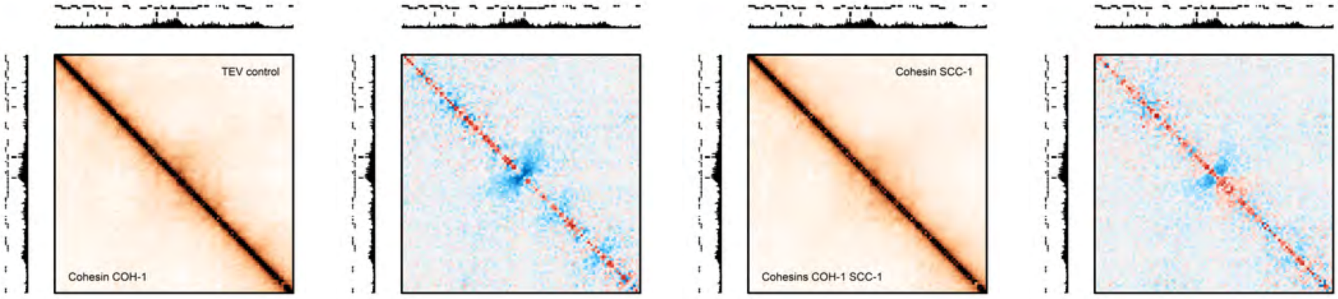

chrV:20317001-20517000

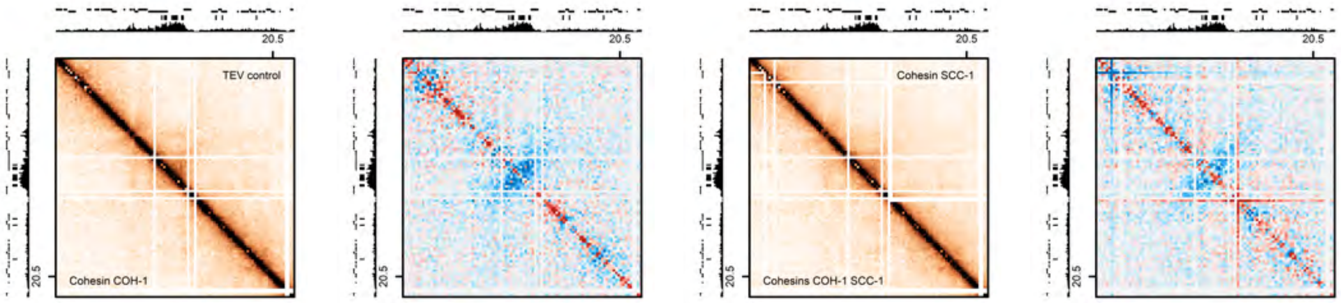

chrX:14721001-14921000

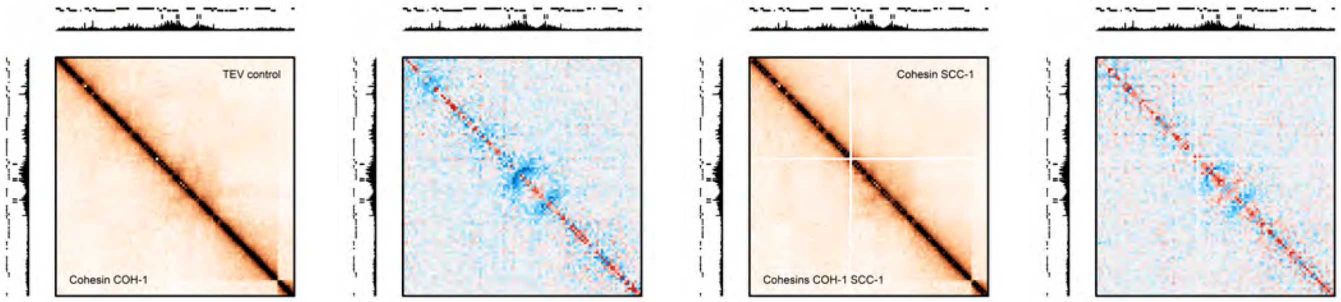

chrV:19425001-19625000

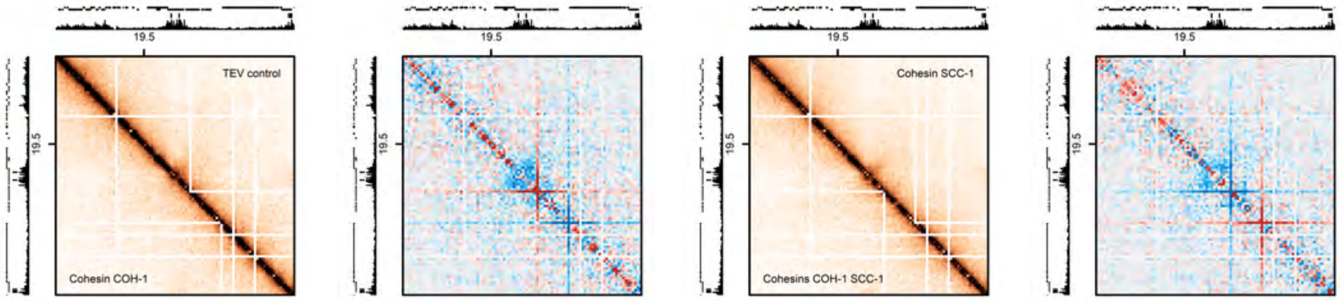

chrIV:16071001-16271000

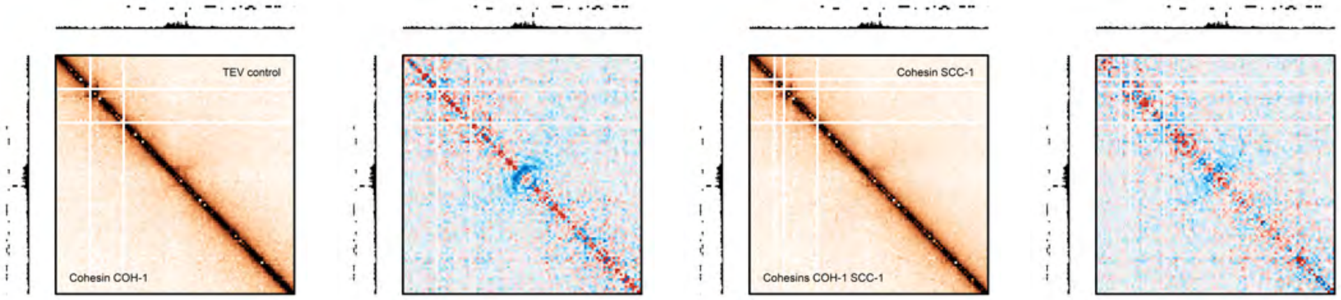

chrIV:12739001-12939000

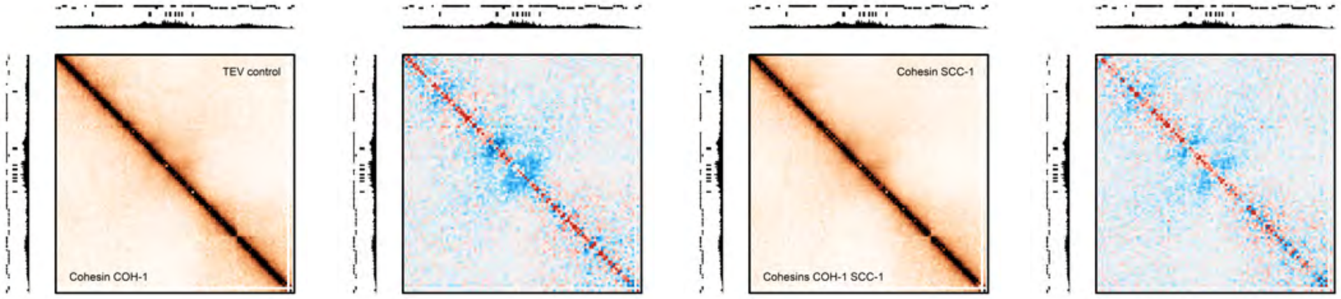

chrI:2761001-2961000

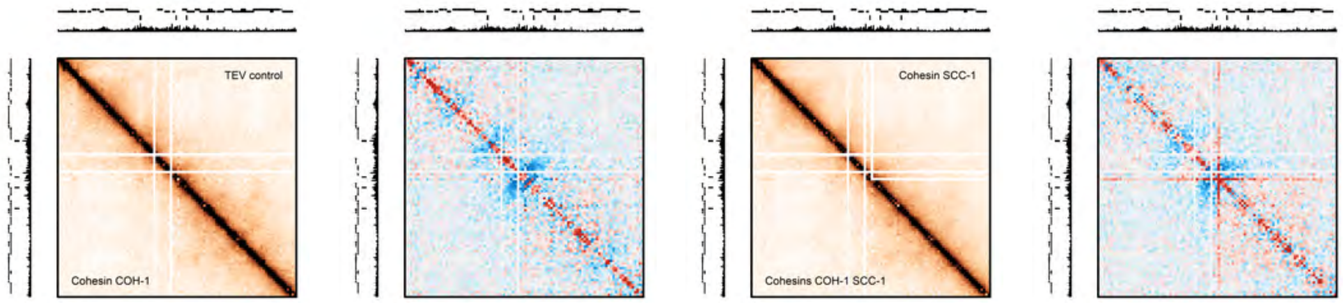

chrX:12809001-13009000

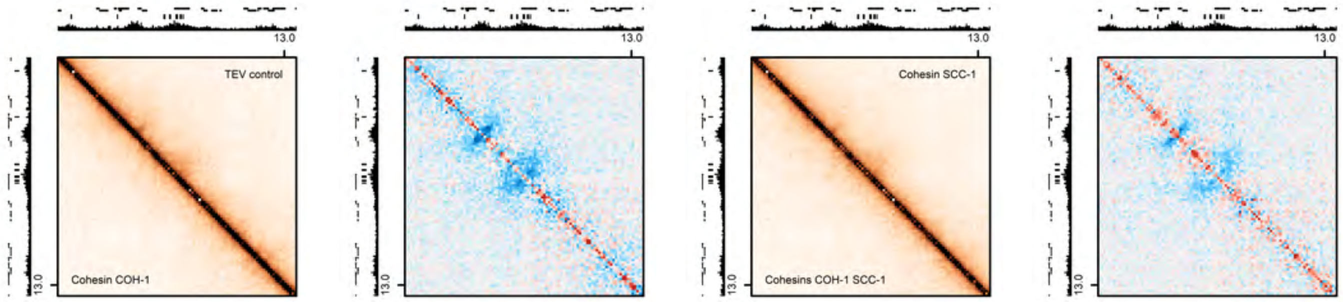

chrIII:4915001-5115000

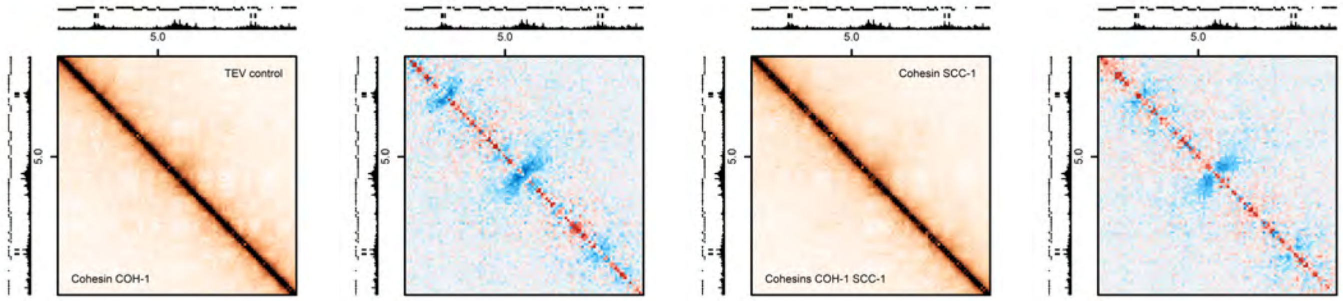

chrV:6781001-6981000

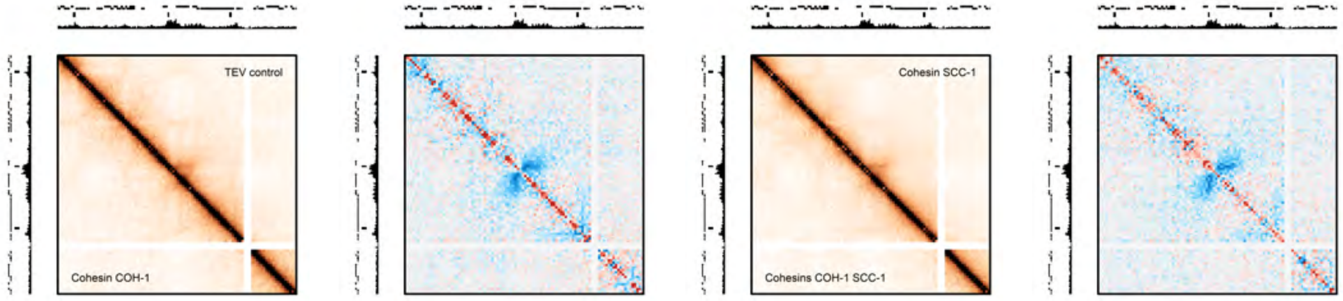

chrX:9451001-9651000

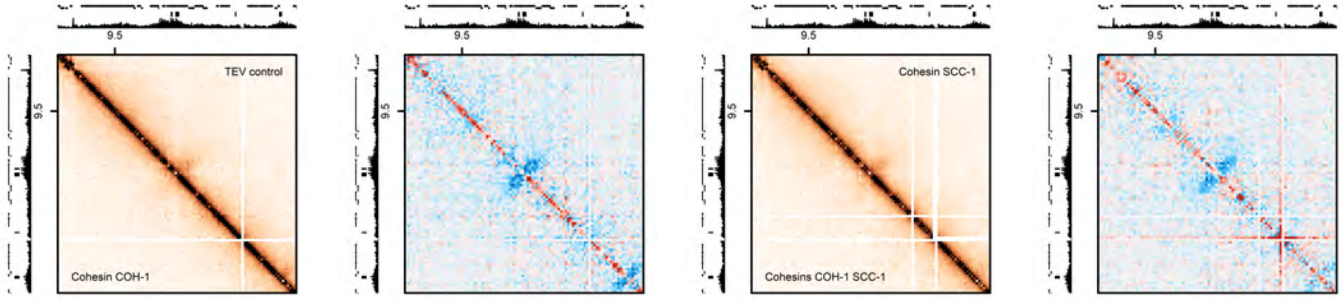

chrIII:4181001-4381000

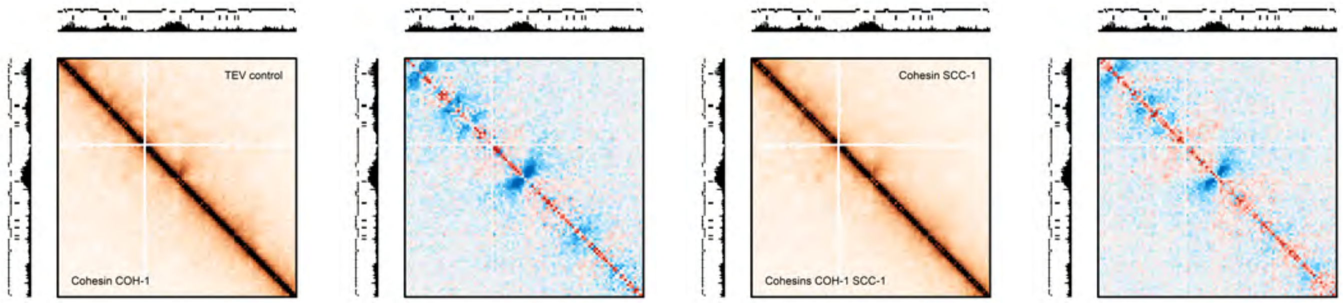

chrV:629001-829000

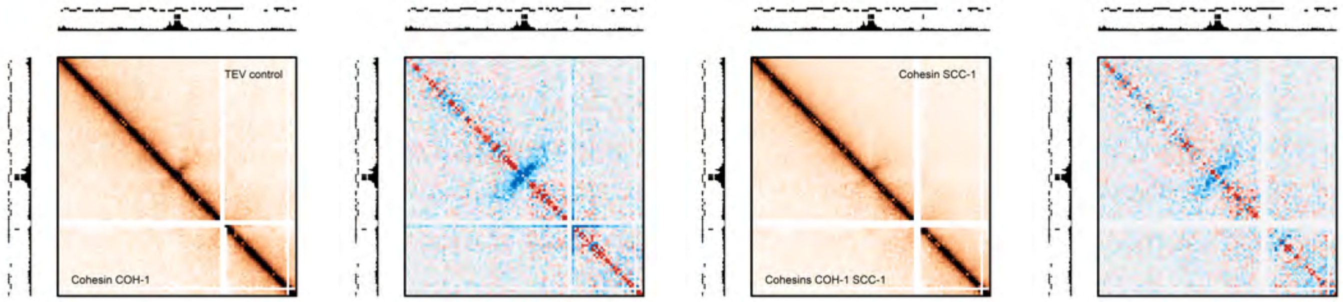

chrX:15509001-15709000

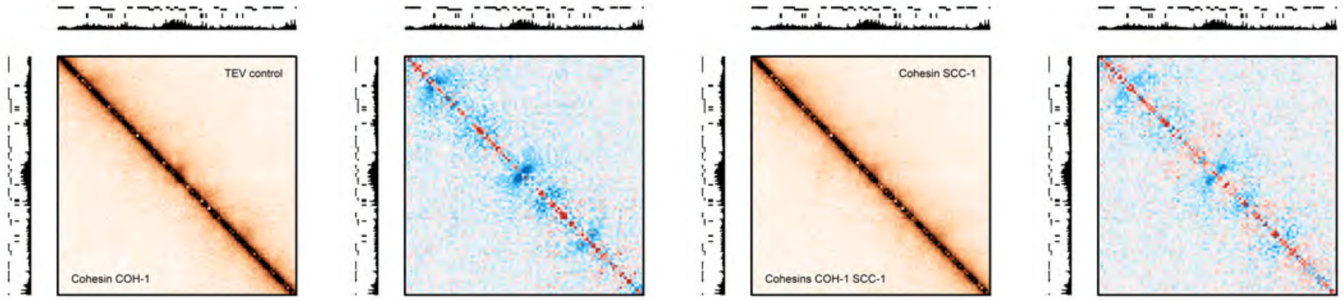

chrIV:15605001-15805000

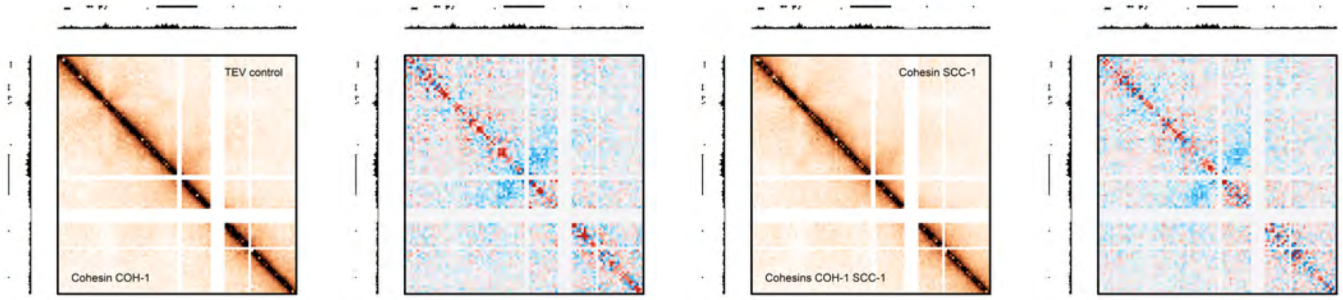

chrII:11647001-11847000

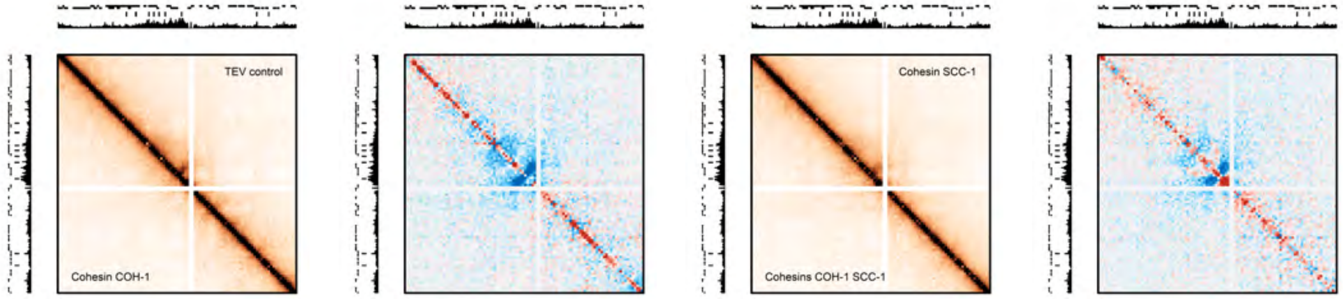

chrII:14717001-14917000

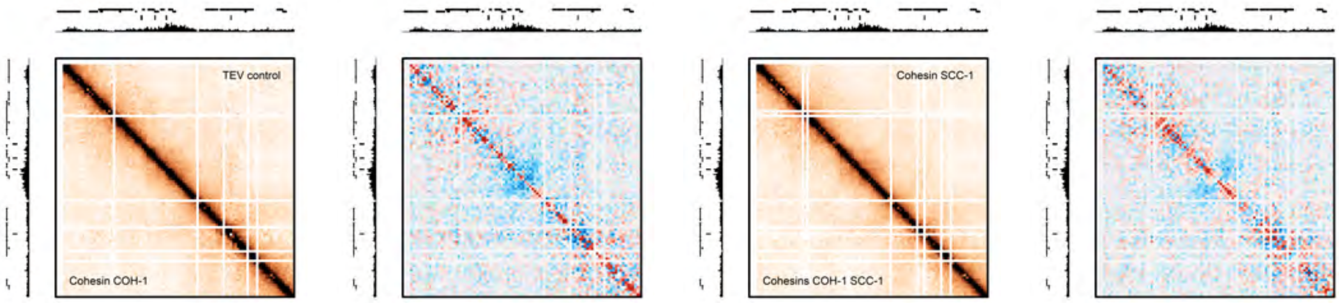

chrV:9941001-10141000

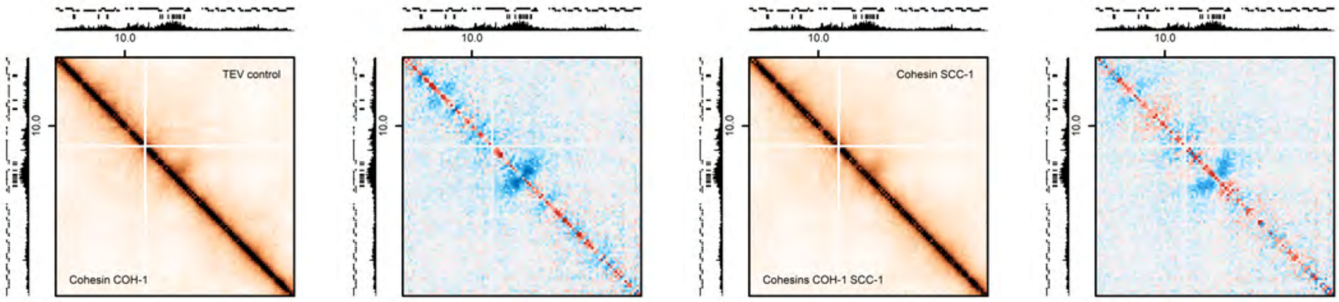

chrX:7135001-7335000

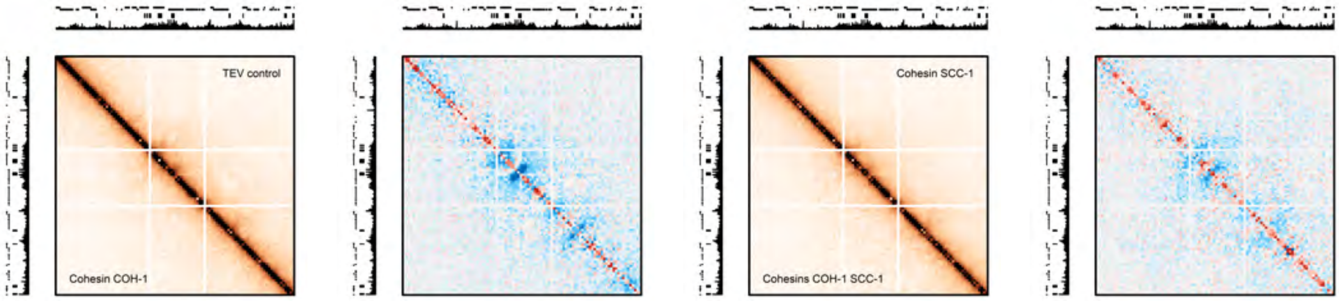

chrI:11101001-11301000

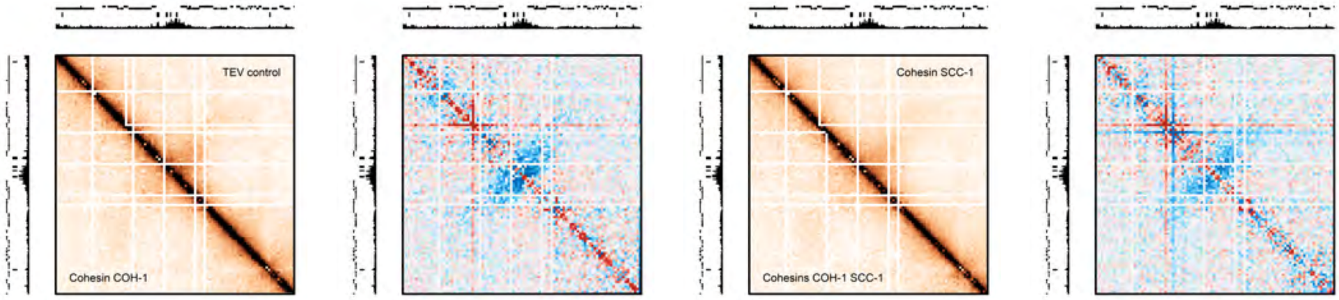

chrIV:3549001-3749000

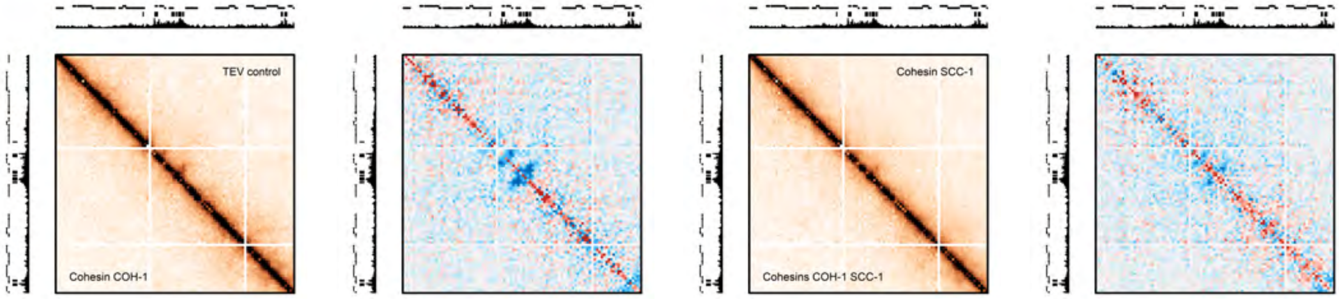

chrIV:9099001-9299000

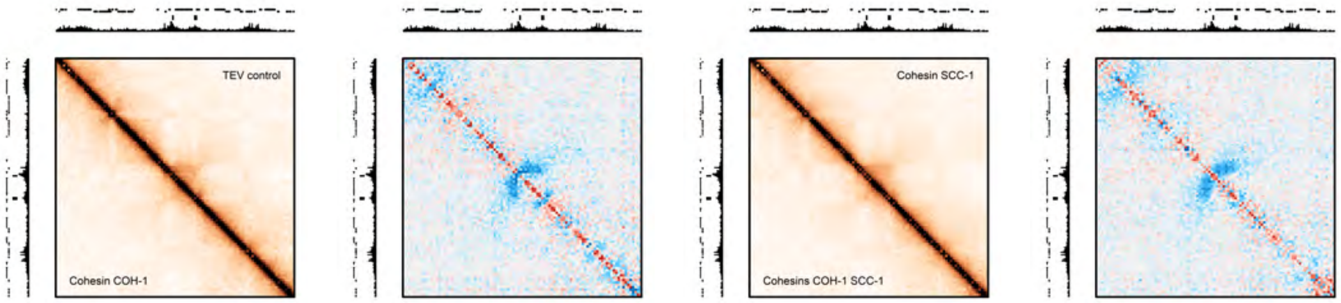

chrIV:13219001-13419000

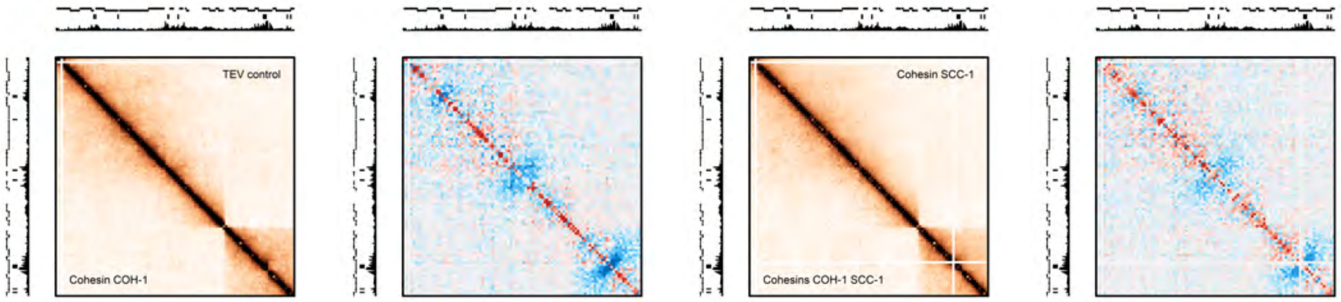

chrI:11571001-11771000

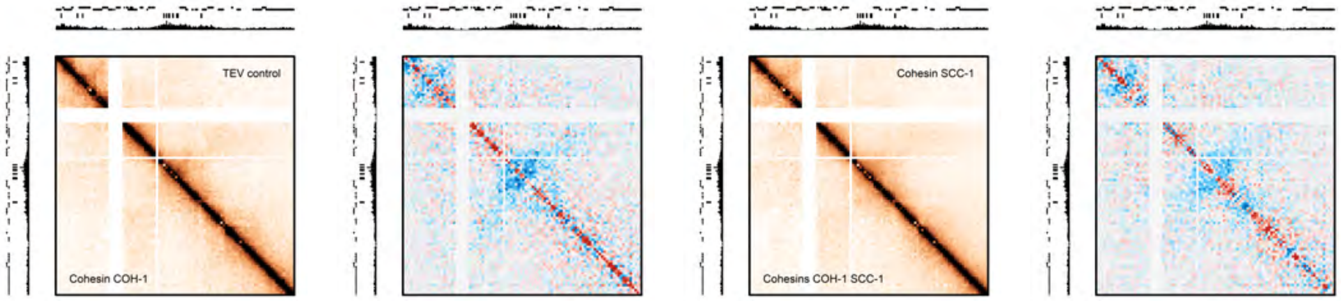

chrV:12437001-12637000

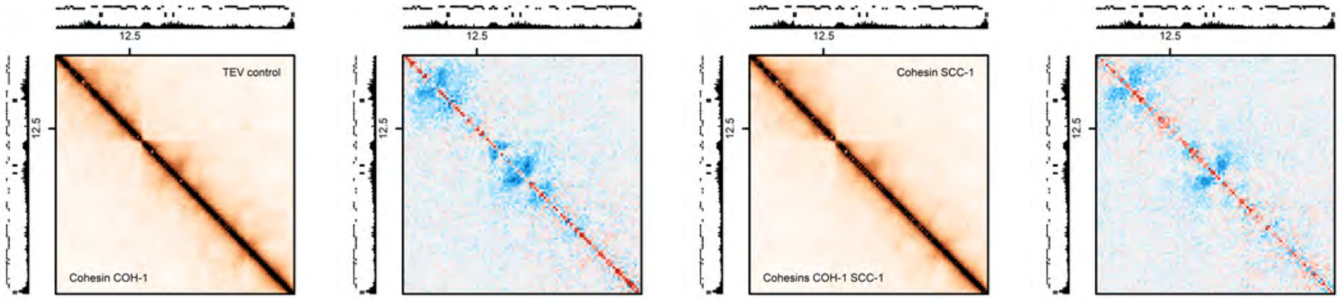

chrV:5903001-6103000

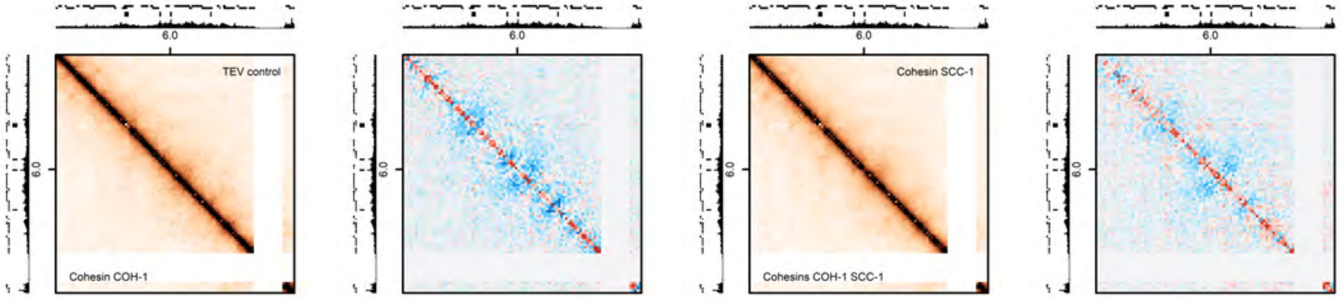

chrX:3725001-3925000

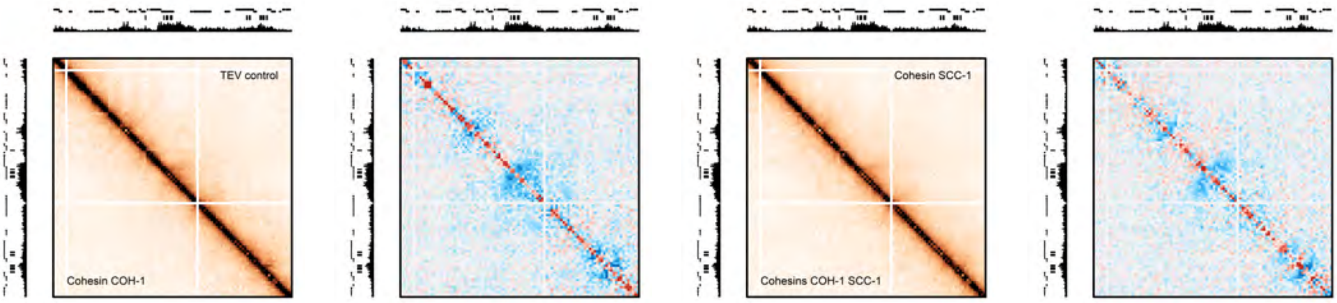

chrII:2355001-2555000

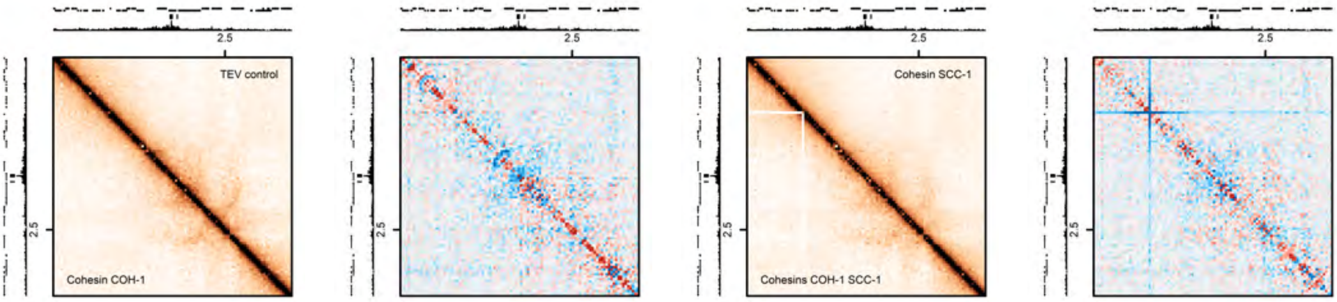

chrII:12123001-12323000

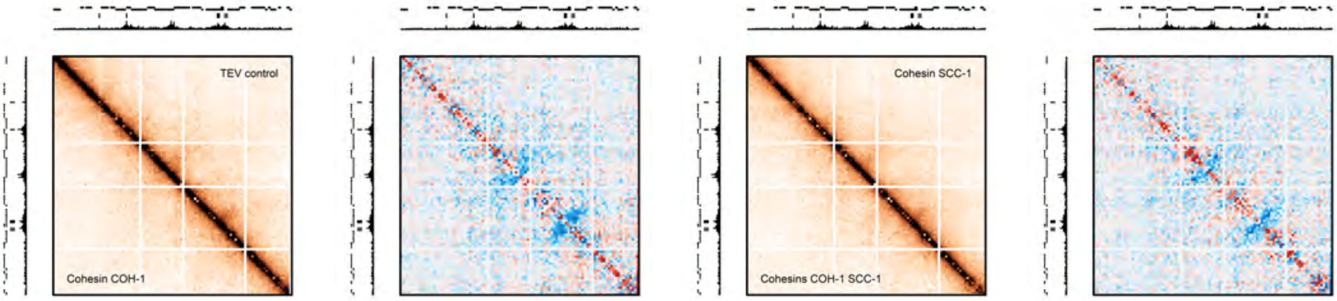

chrII:-78999-121000

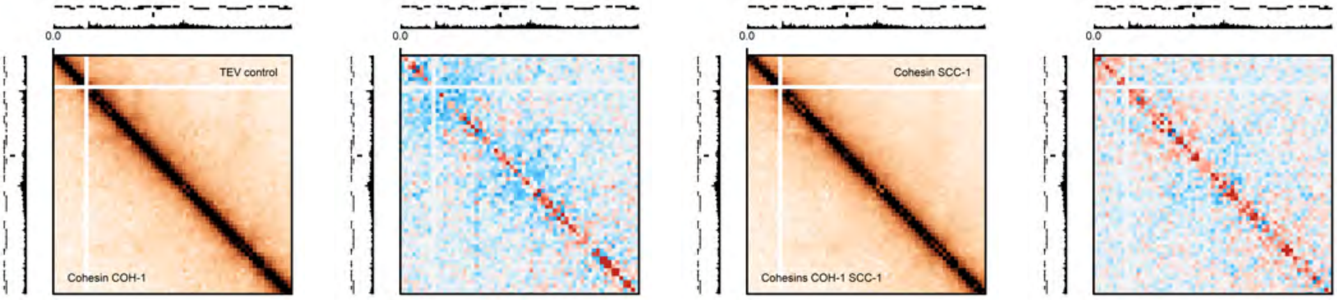

chrIII:11733001-11933000

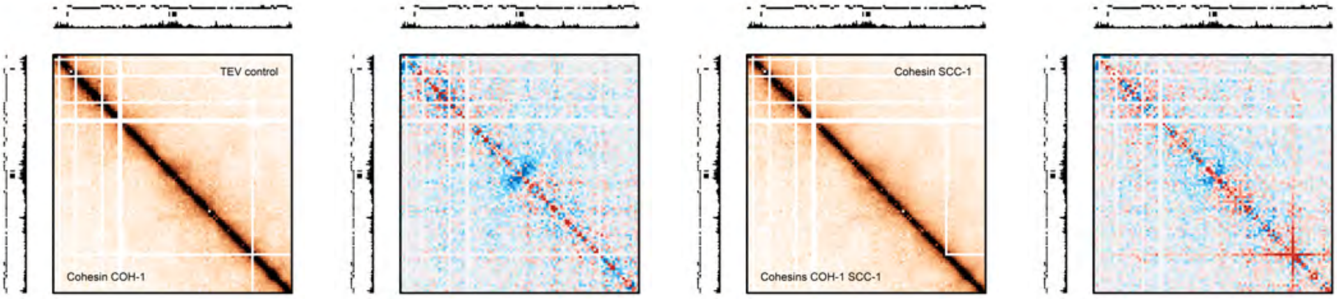

chrIV:3133001-3333000

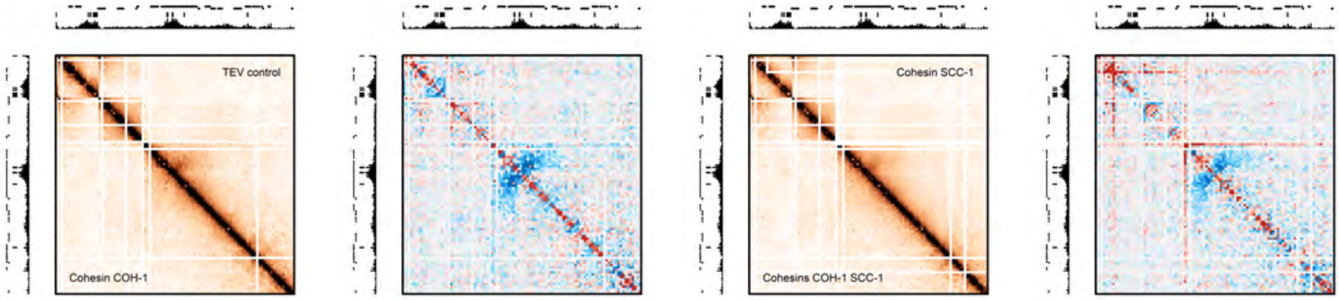

chrIV:5035001-5235000

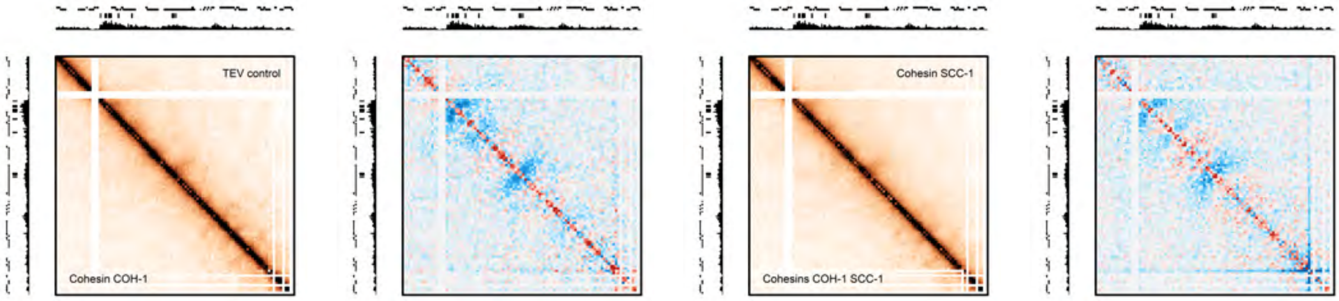

chrI:1203001-1403000

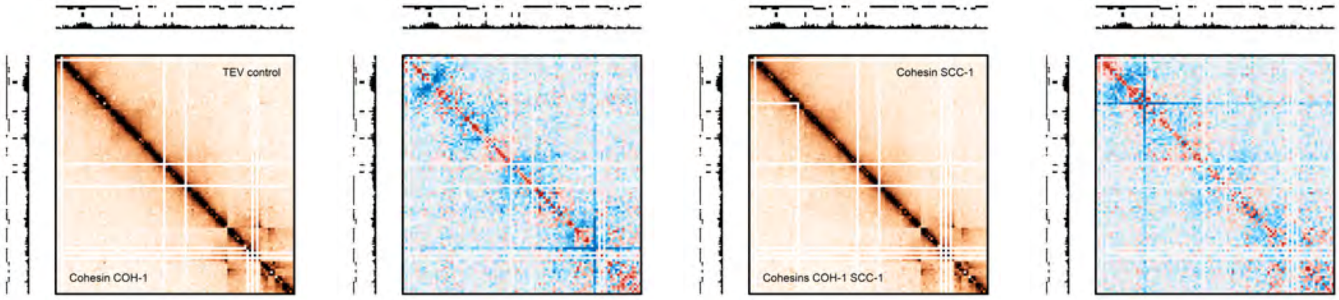

chrI:9999001-10199000

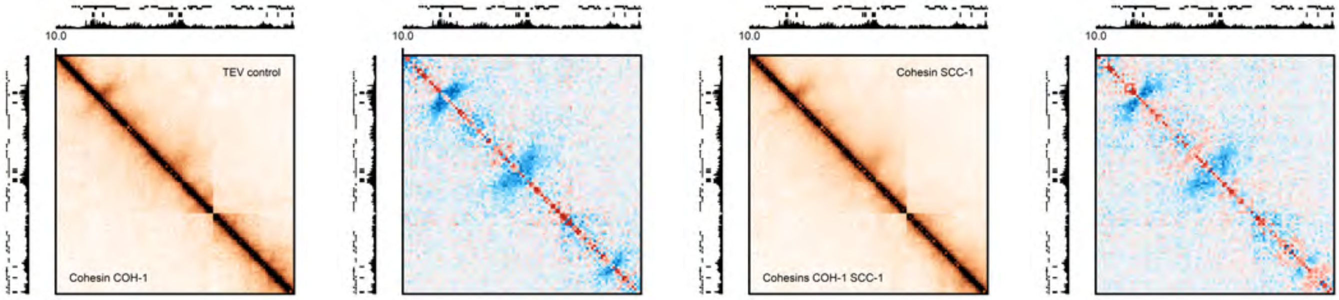

chrI:10993001-11193000

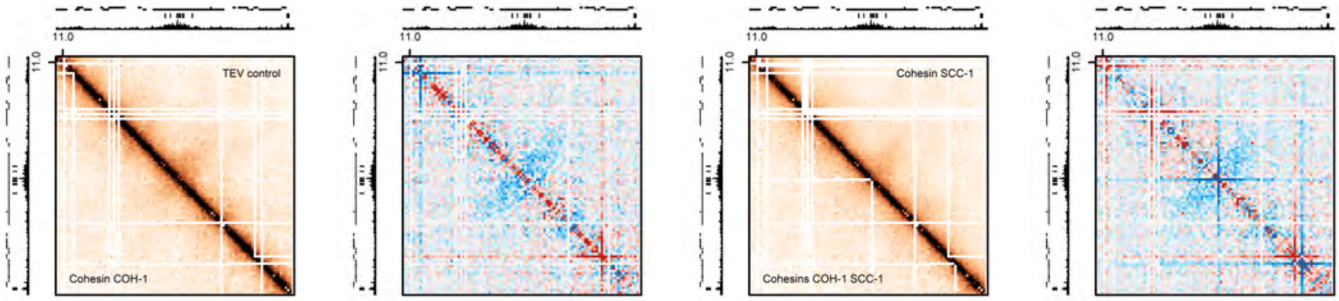

chrIV:-16999-183000

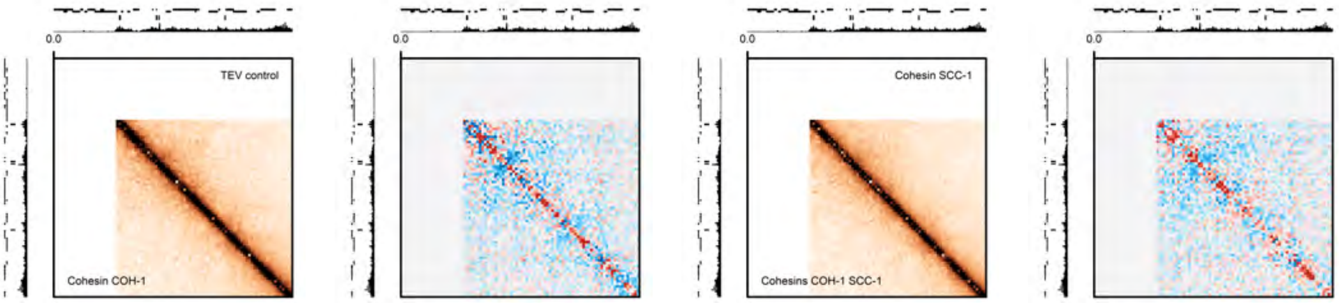

chrI:4599001-4799000

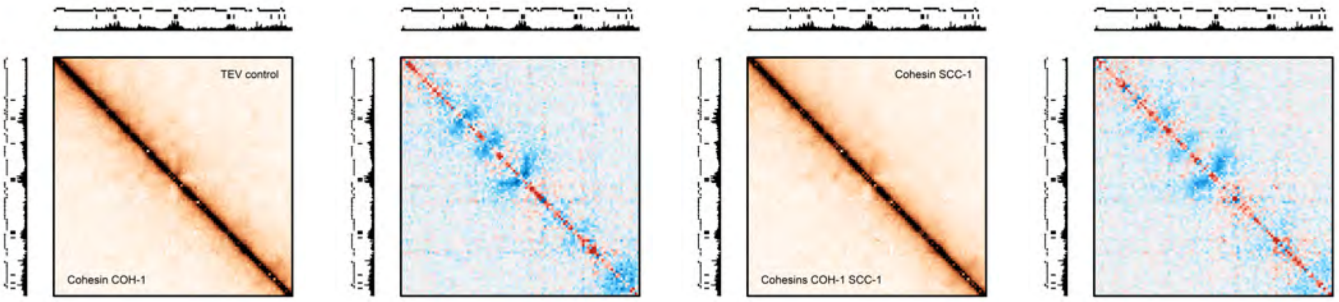

chrX:3015001-3215000

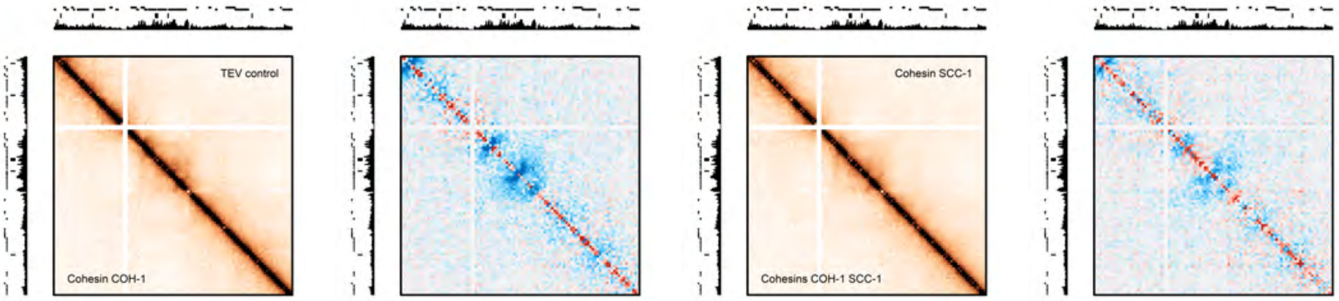

chrI:12473001-12673000

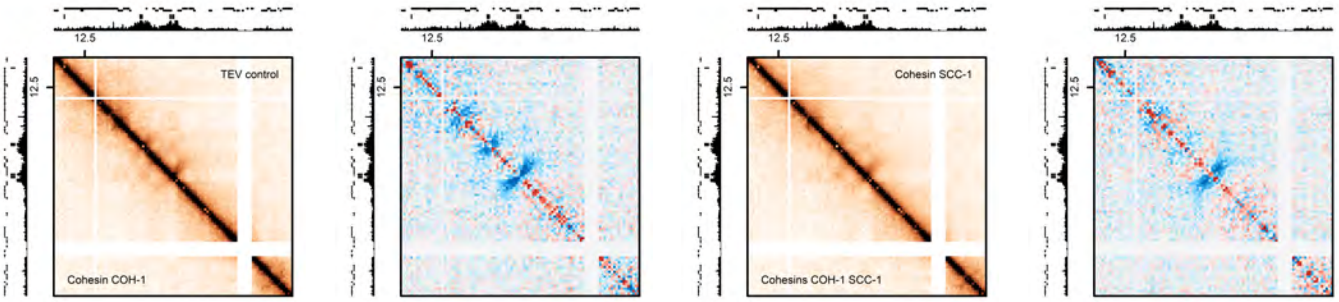

chrX:17419001-17619000

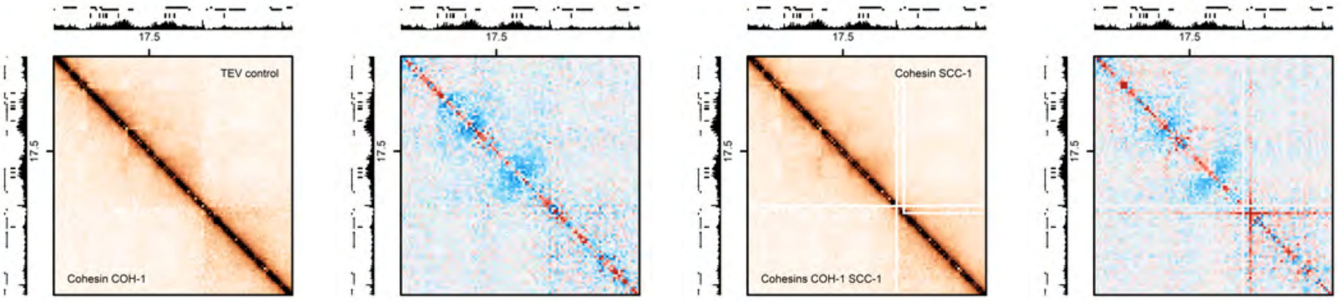

chrII:8029001-8229000

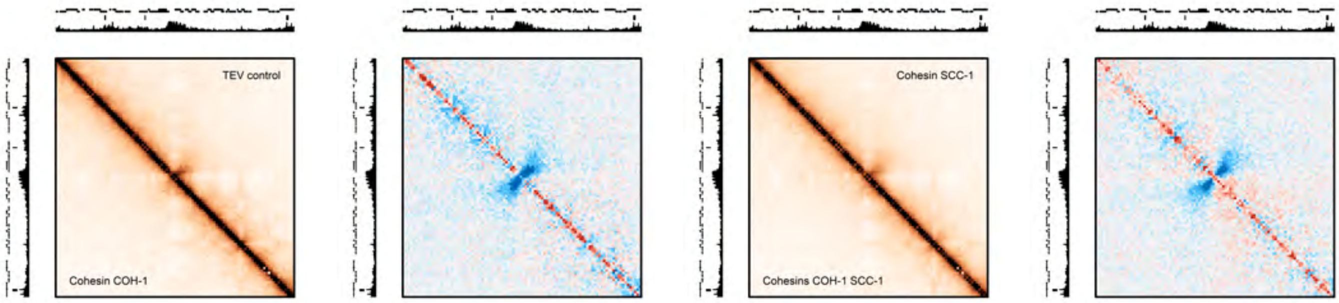

chrIV:1759001-1959000

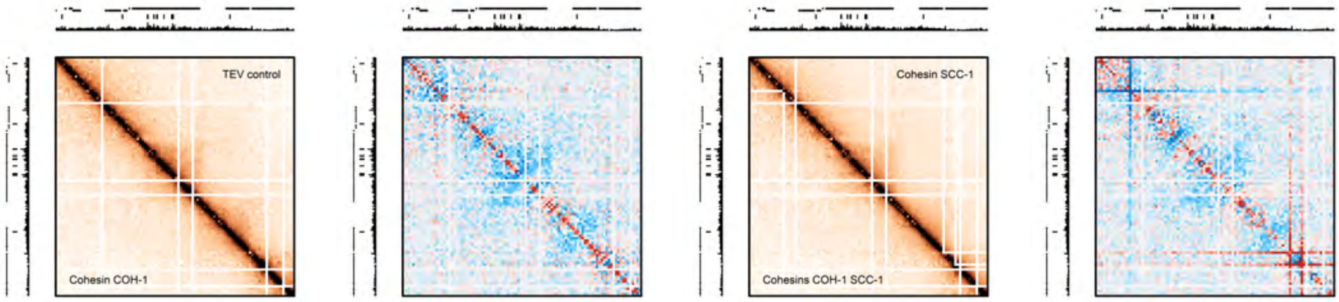

chrIV:4113001-4313000

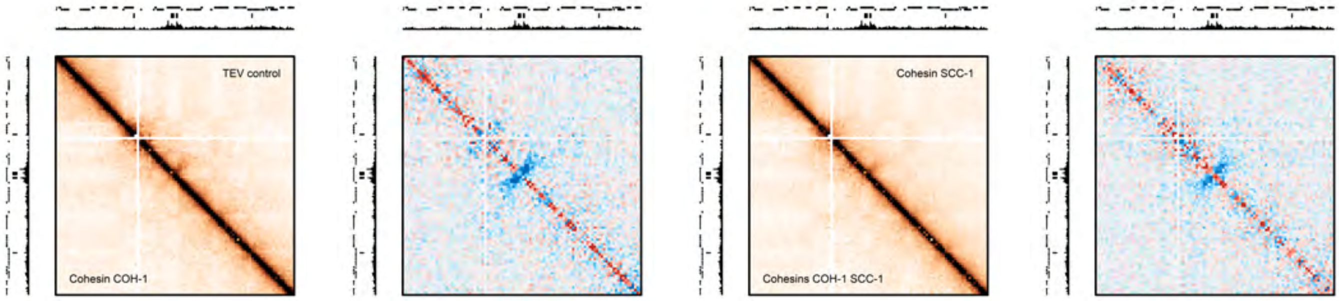

chrIV:5875001-6075000

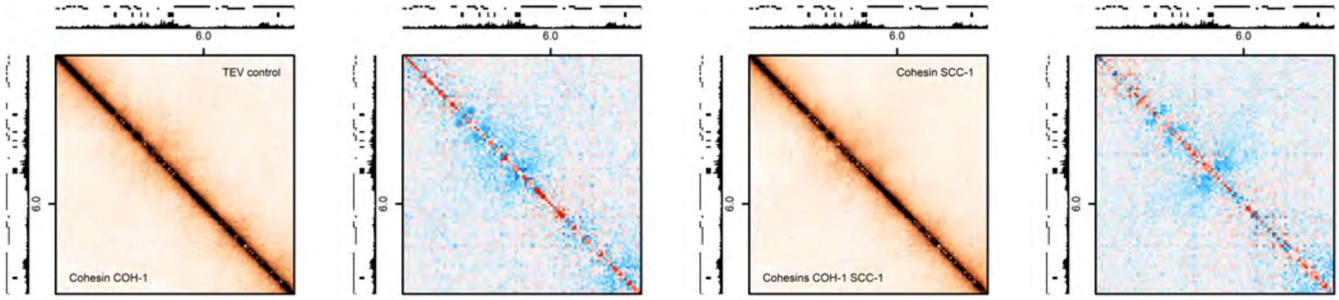

chrV:8687001-8887000

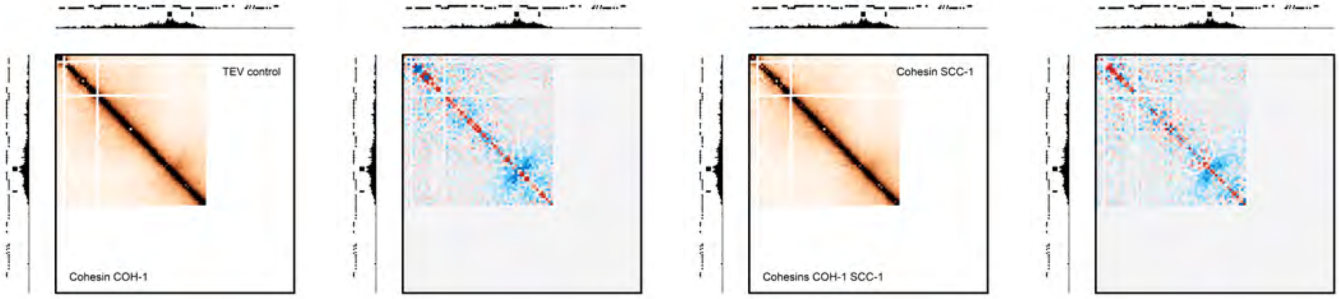

chrV:11433001-11633000

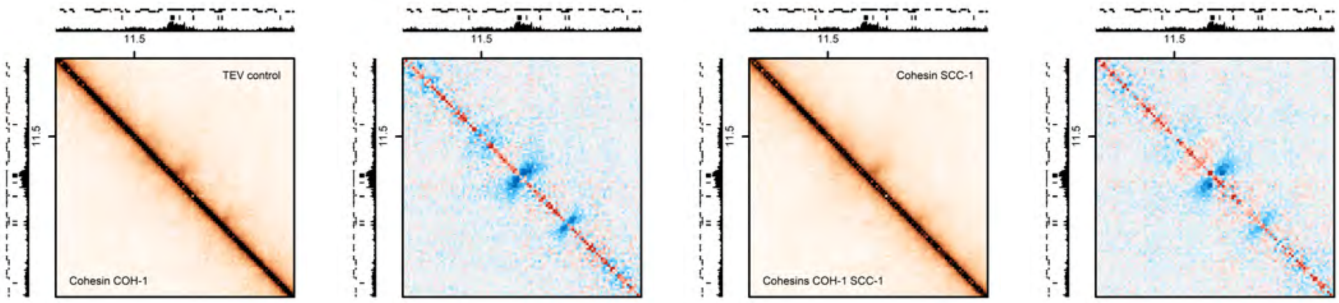

chrV:13557001-13757000

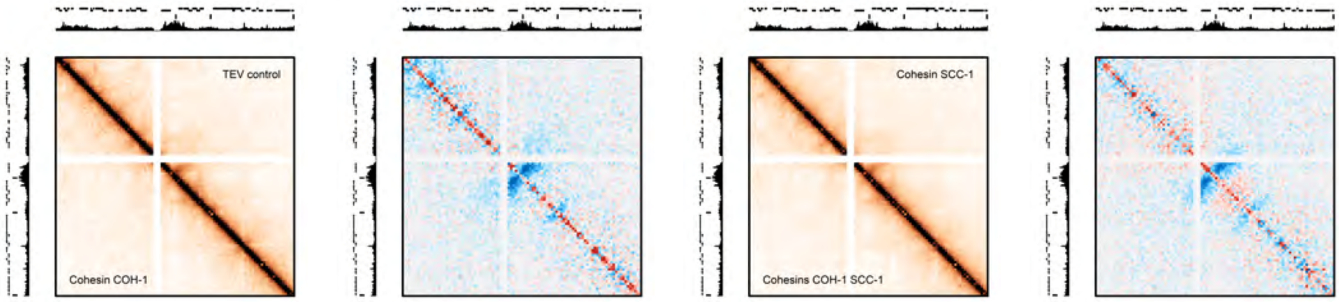

chrI:9757001-9957000

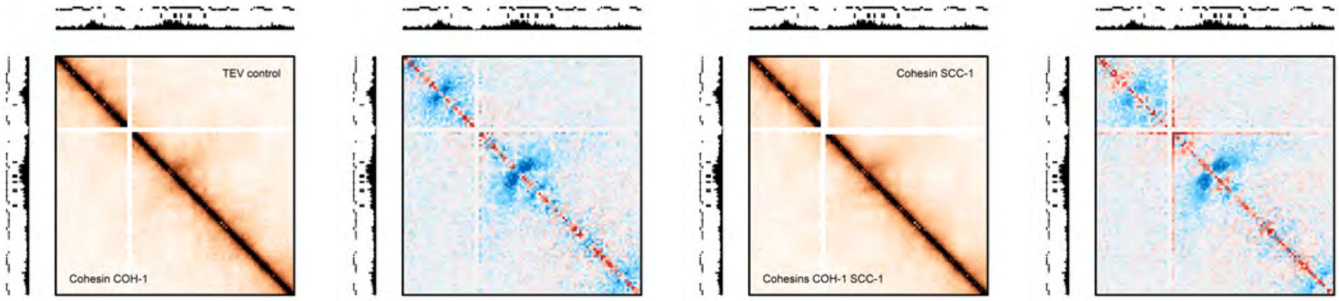

chrV:13689001-13889000

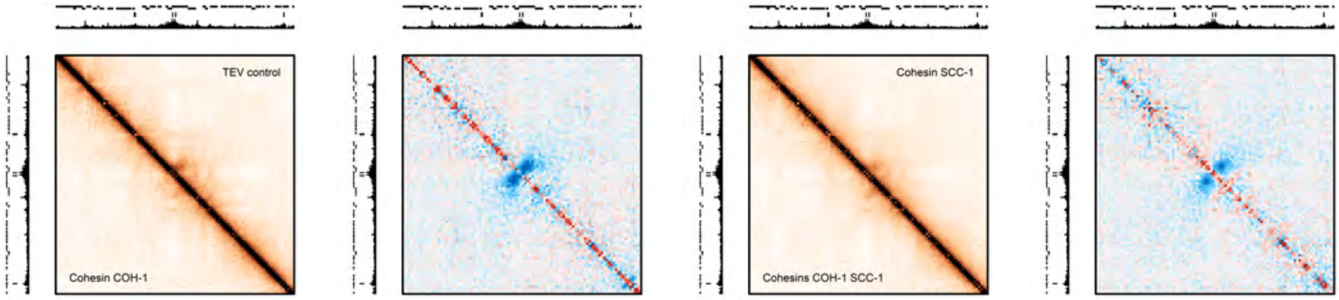

chrIV:14779001-14979000

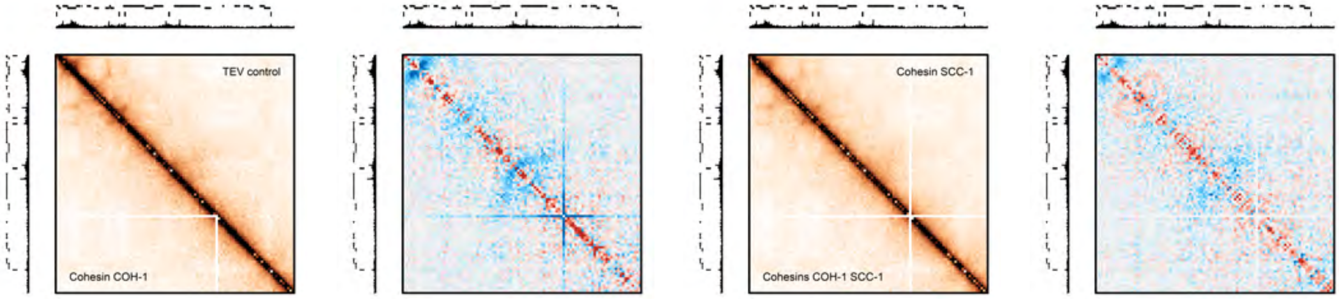

chrIII:735001-935000

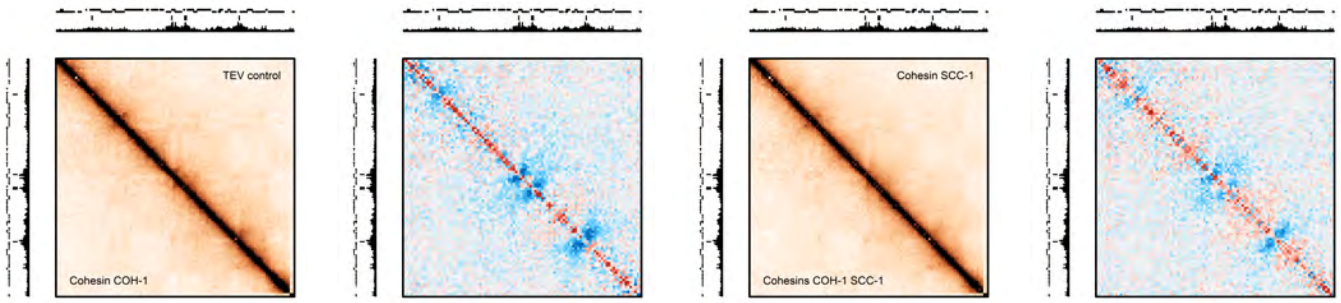

chrV:13903001-14103000

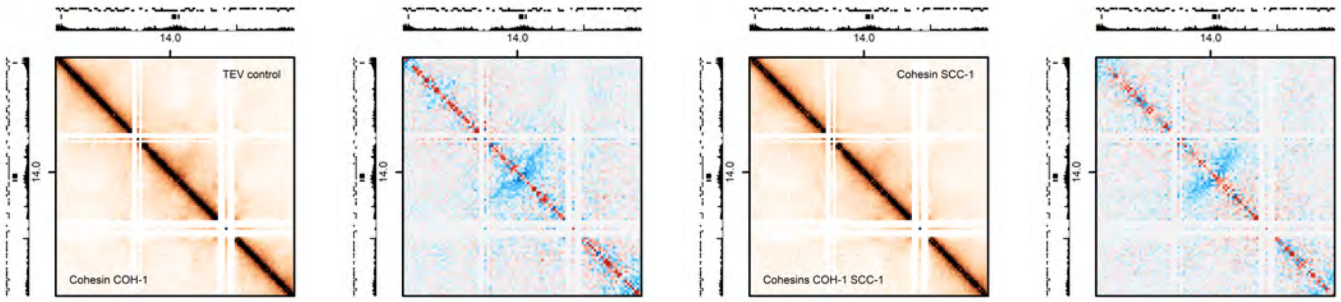

chrI:445001-645000

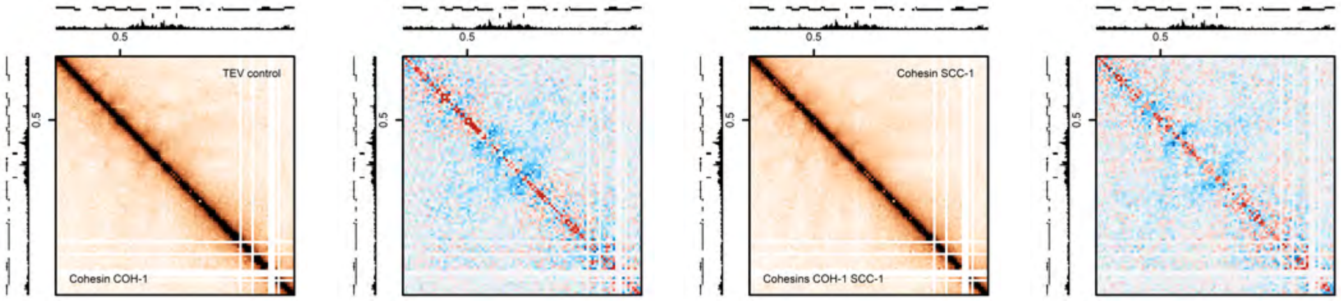

chrIV:13293001-13493000

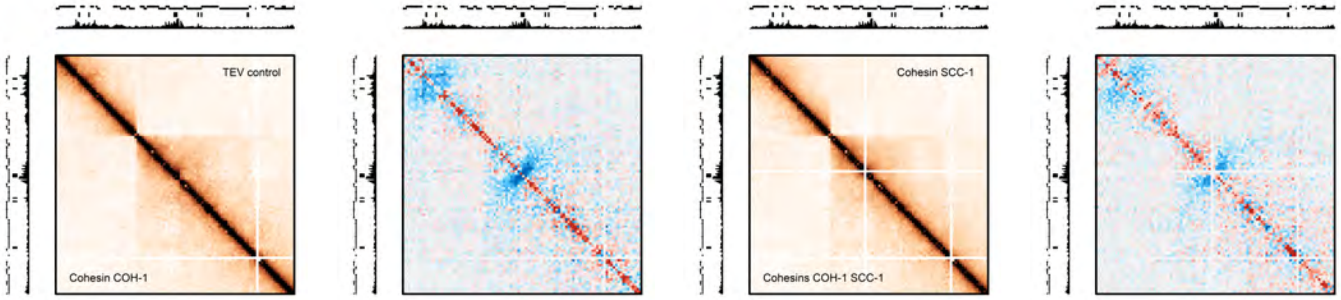

chrIV:8429001-8629000

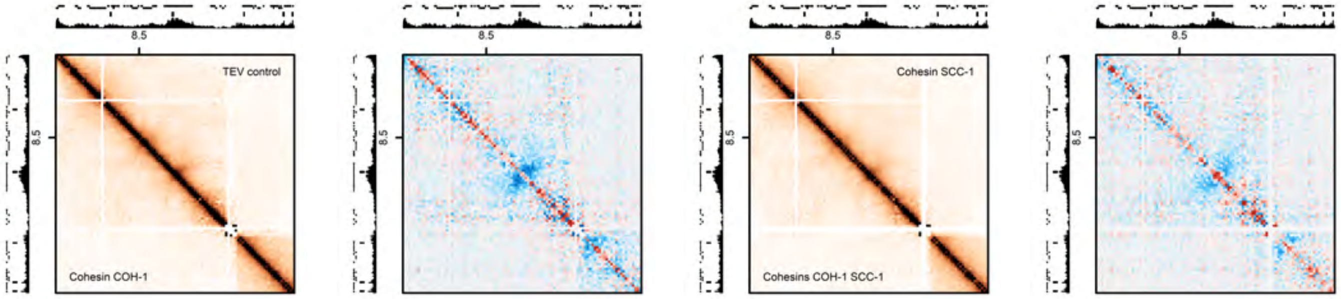

chrV:8561001-8761000

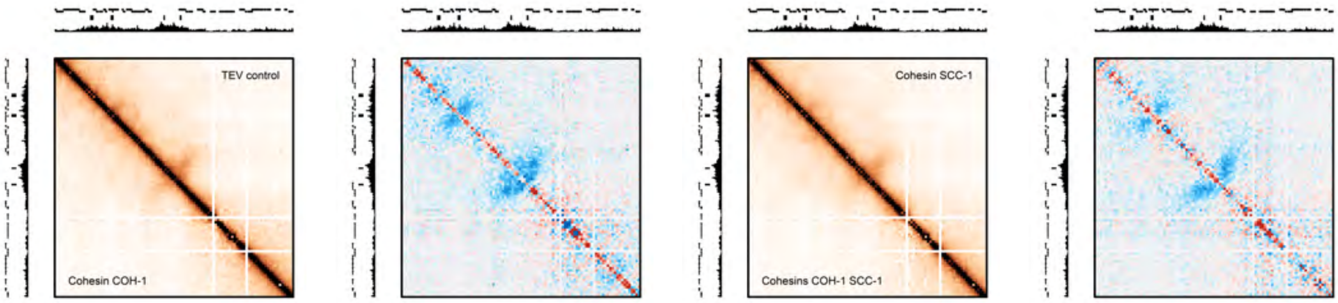

chrIII:12037001-12237000

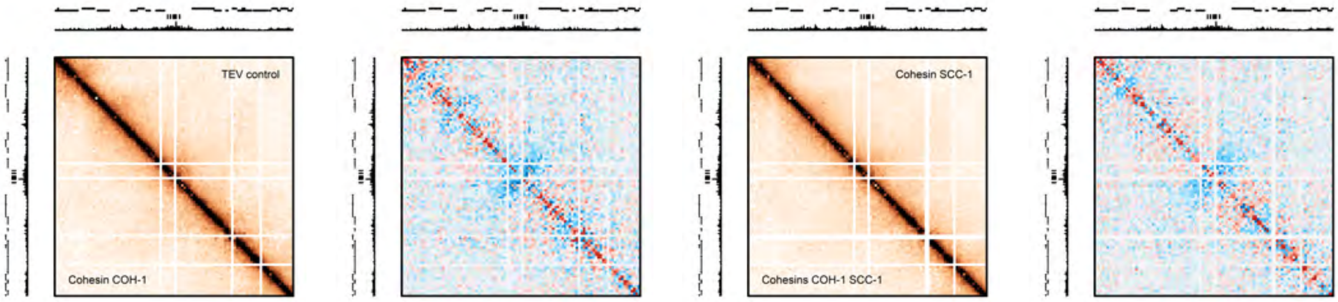

chrII:9889001-10089000

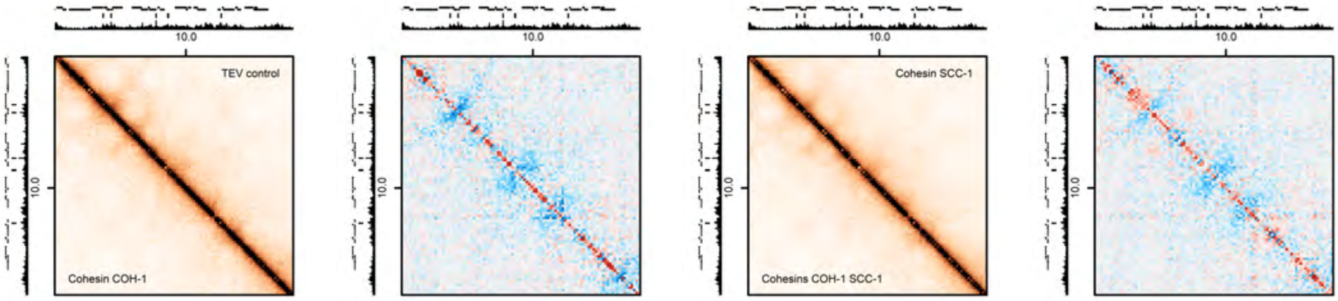

chrIII:1517001-1717000

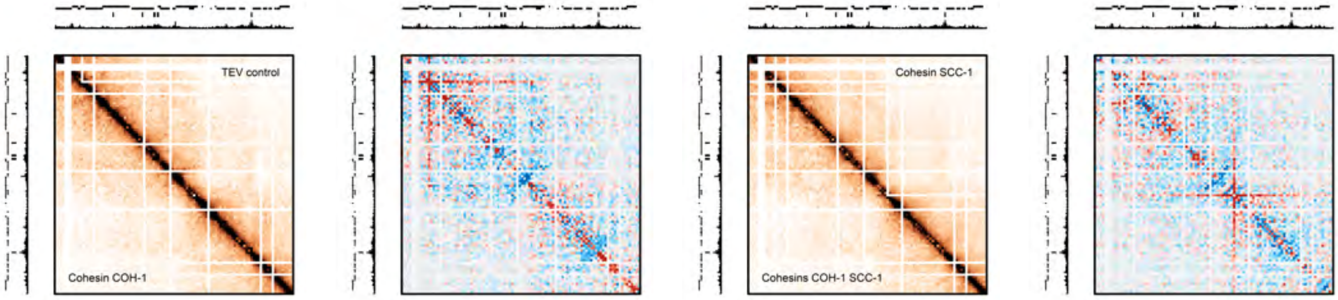

chrII:13093001-13293000

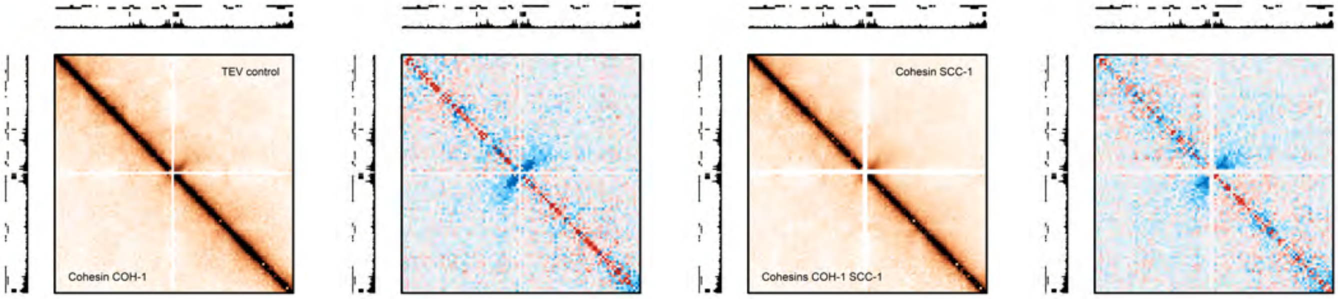

chrIII:12965001-13165000

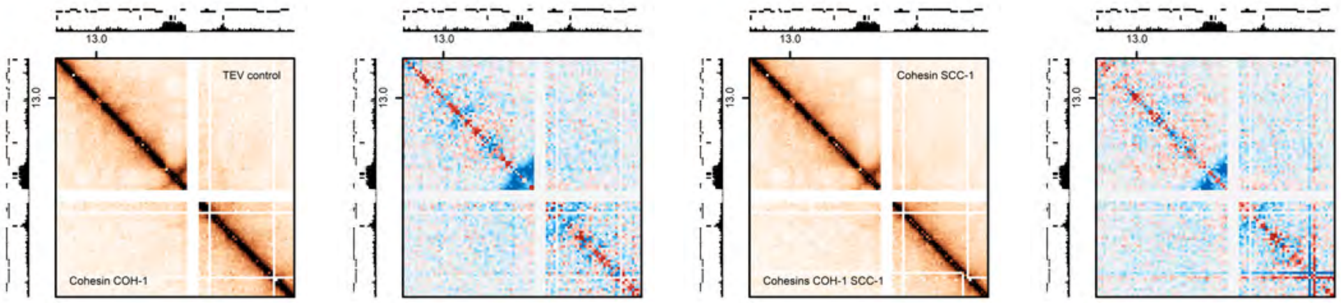

chrV:1787001-1987000

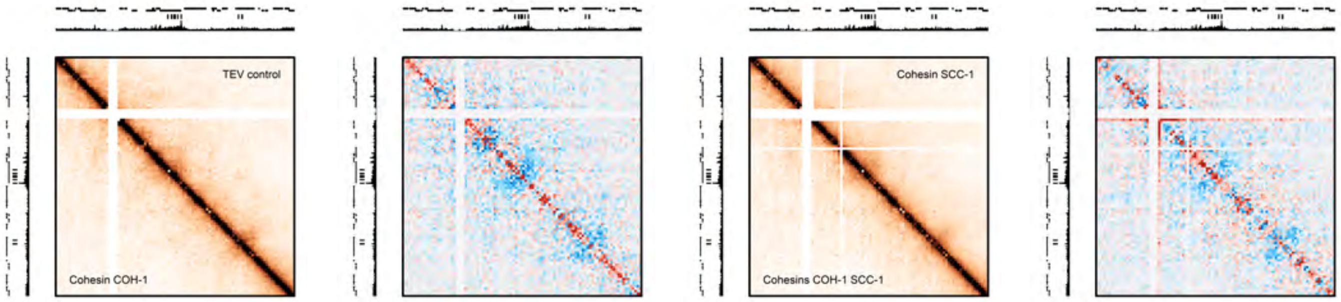

chrX:10669001-10869000

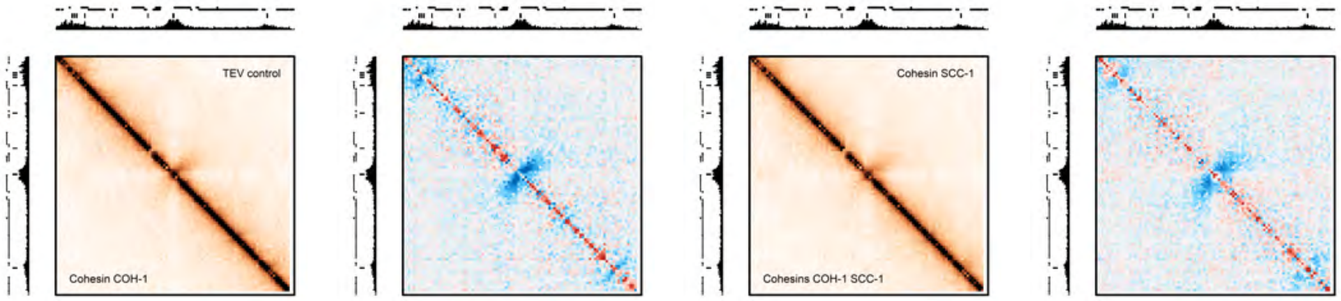

chrX:8155001-8355000

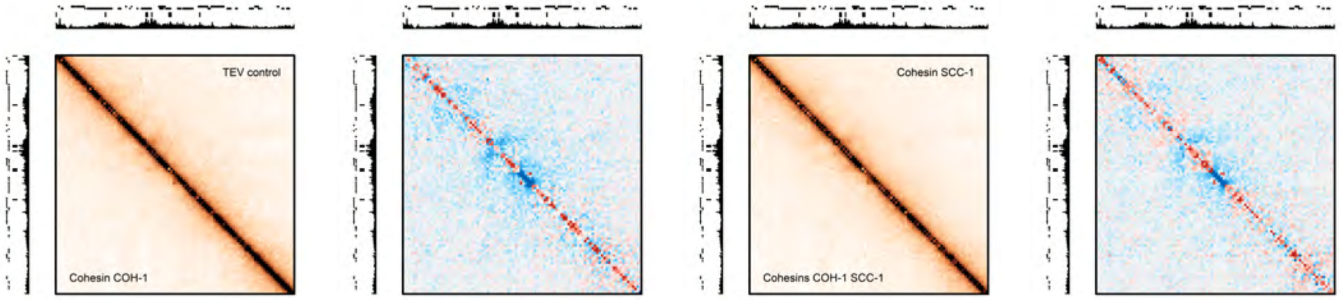

chrX:4425001-4625000

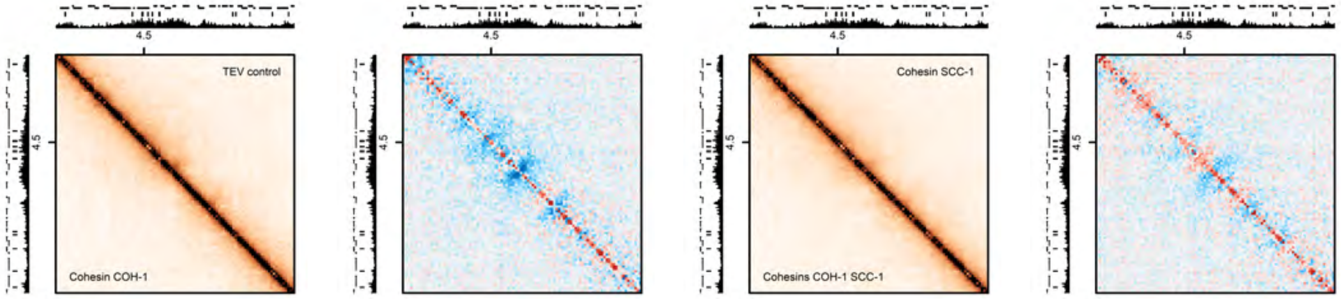

chrV:9171001-9371000

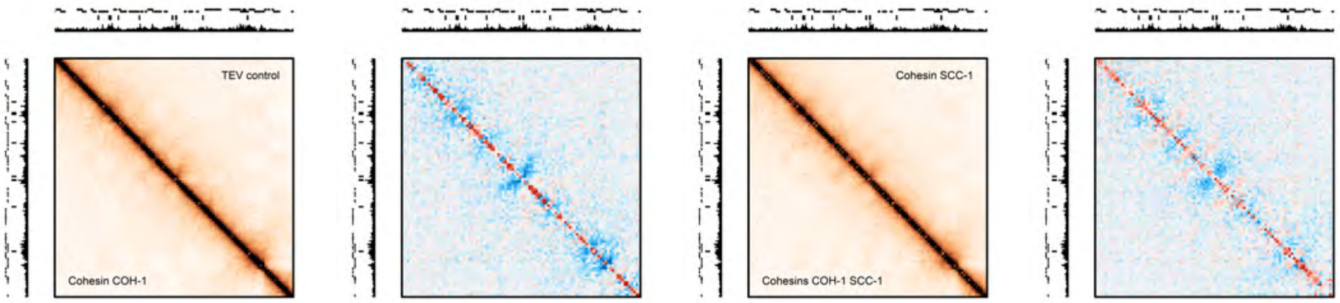

chrV:879001-1079000

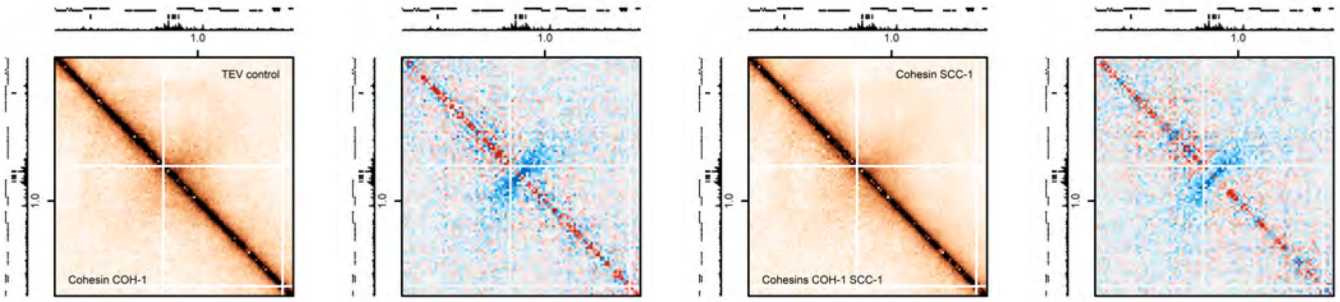

chrV:17851001-18051000

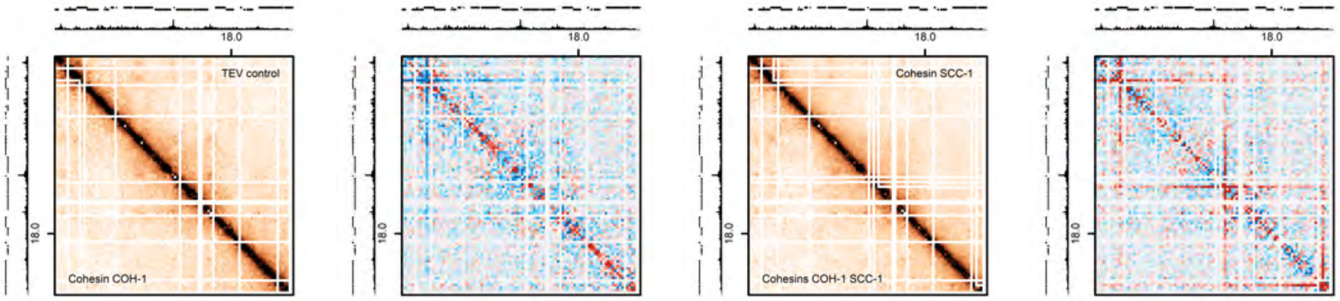

chrX:5019001-5219000

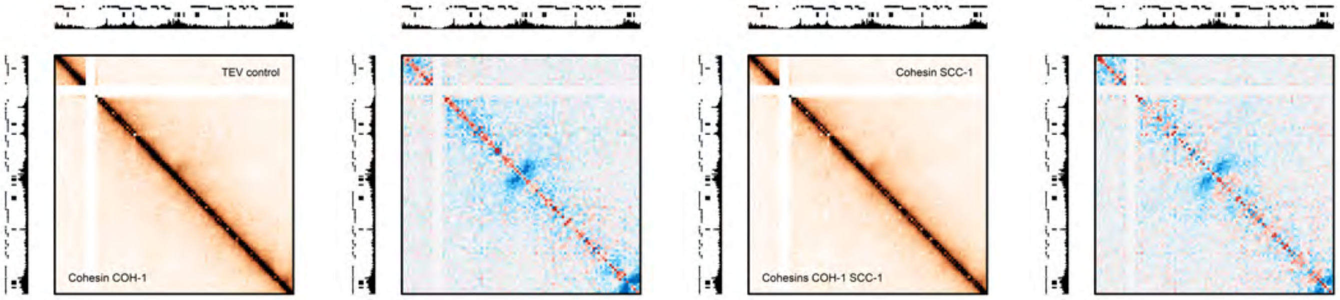

chrIII:2933001-3133000

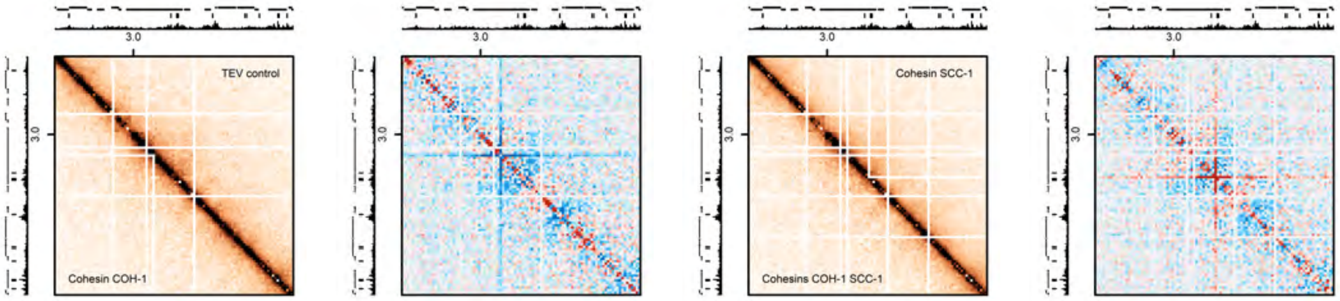

chrI:5213001-5413000

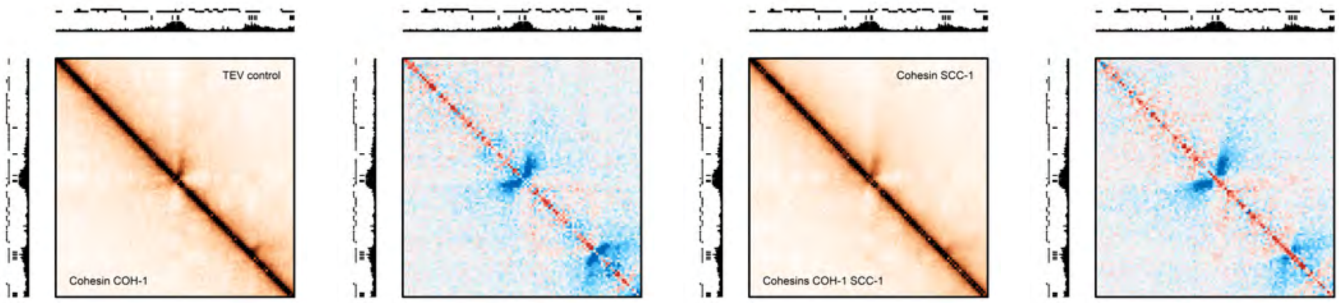

chrX:9001001-9201000

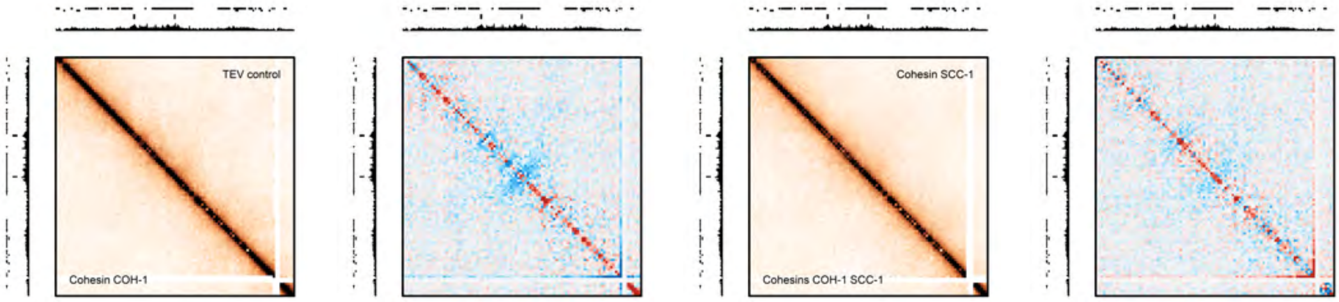

chrII:14147001-14347000

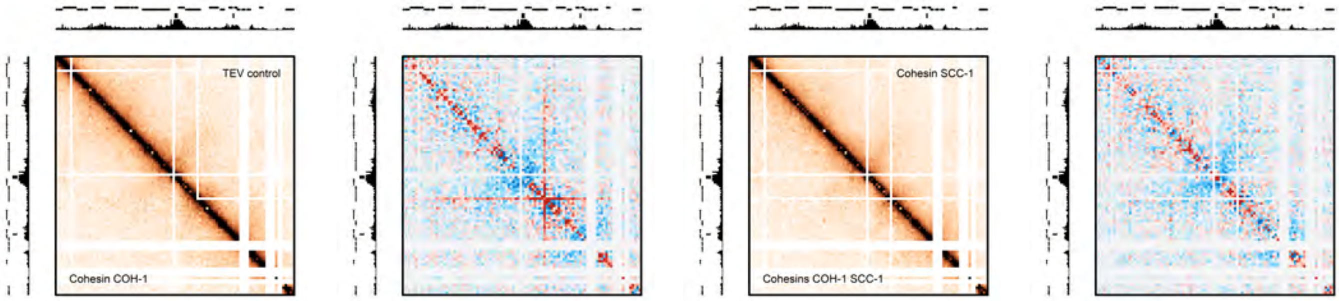

chrII:8963001-9163000

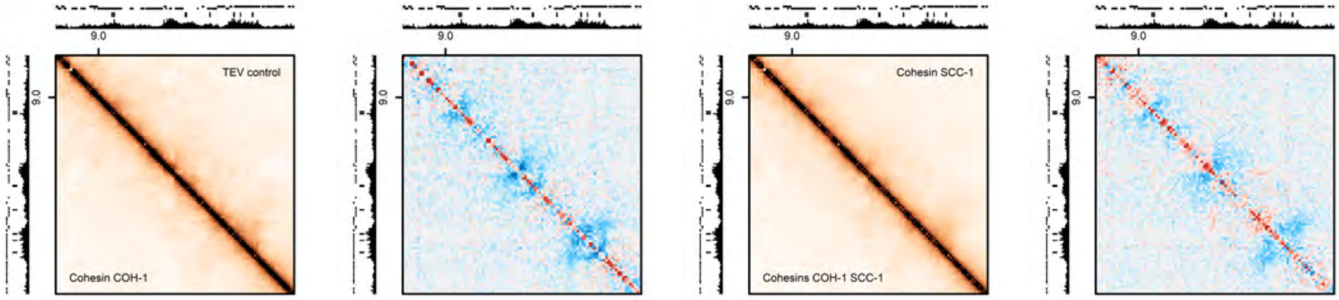

chrII:8383001-8583000

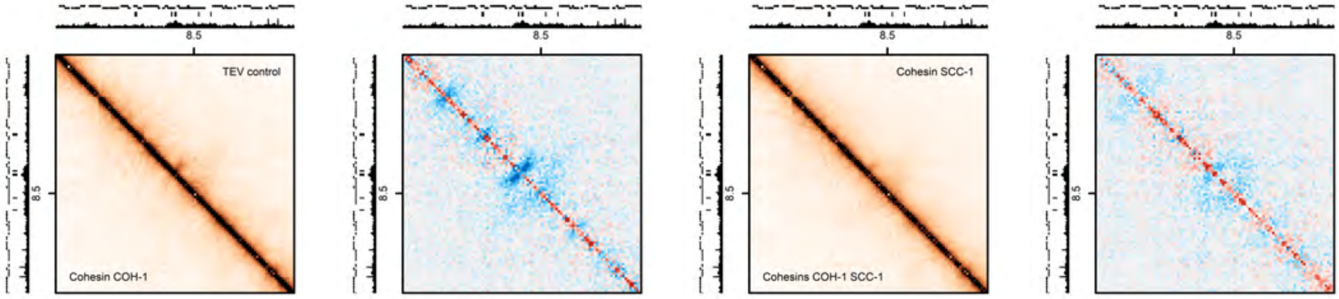

chrIII:5525001-5725000

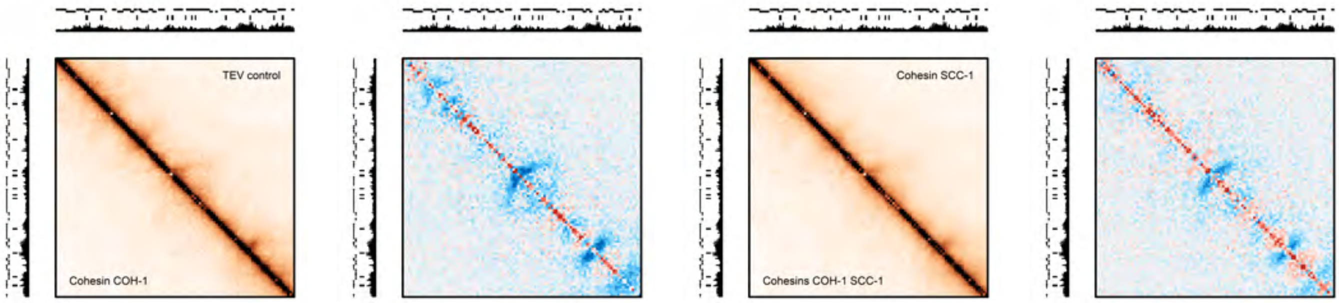

chrII:7241001-7441000

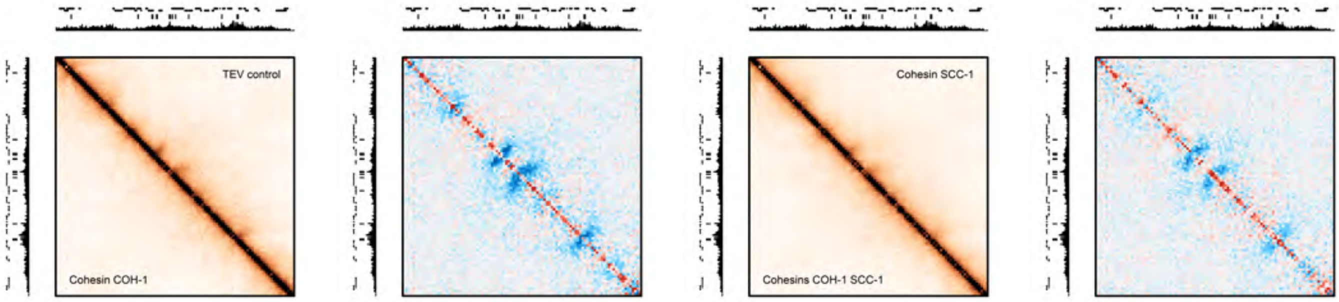

chrX:10935001-11135000

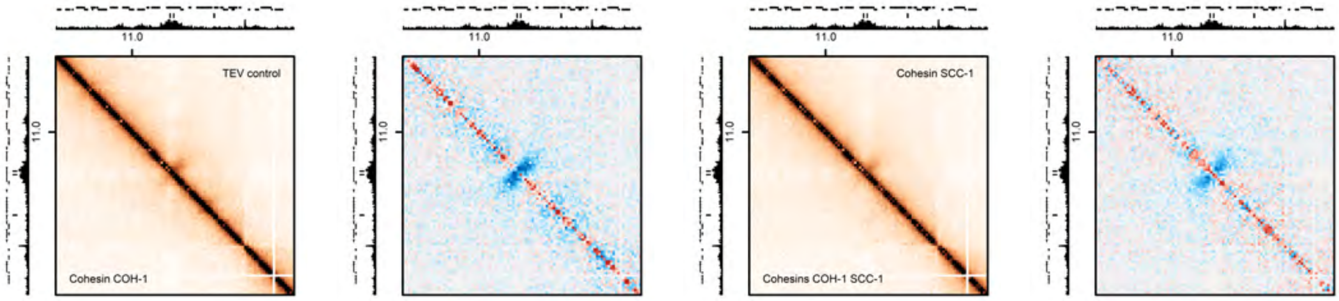

chrX:12569001-12769000

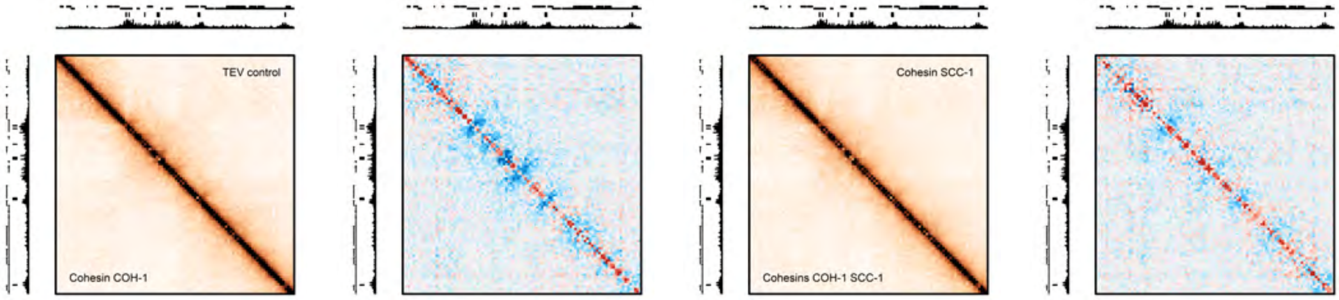

chrX:13645001-13845000

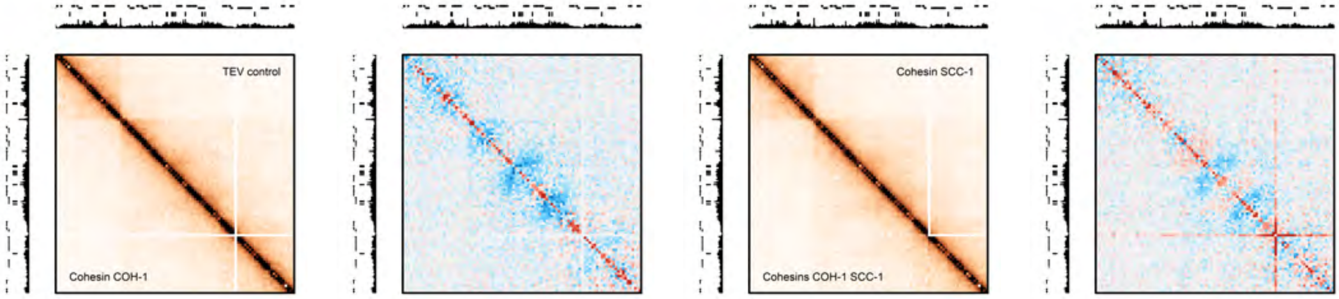

chrIV:6473001-6673000

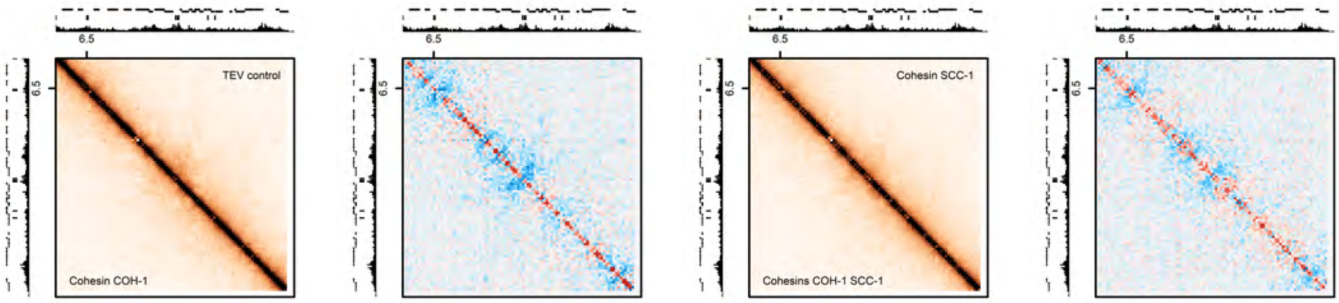

chrV:12201001-12401000

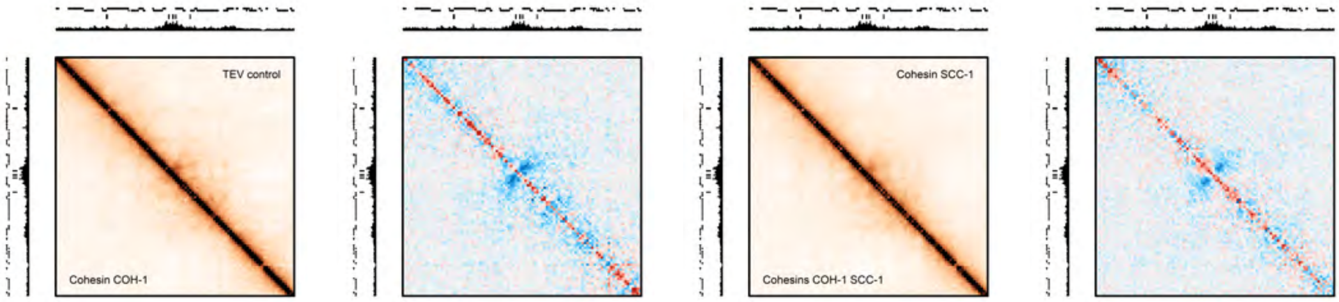

chrI:6021001-6221000

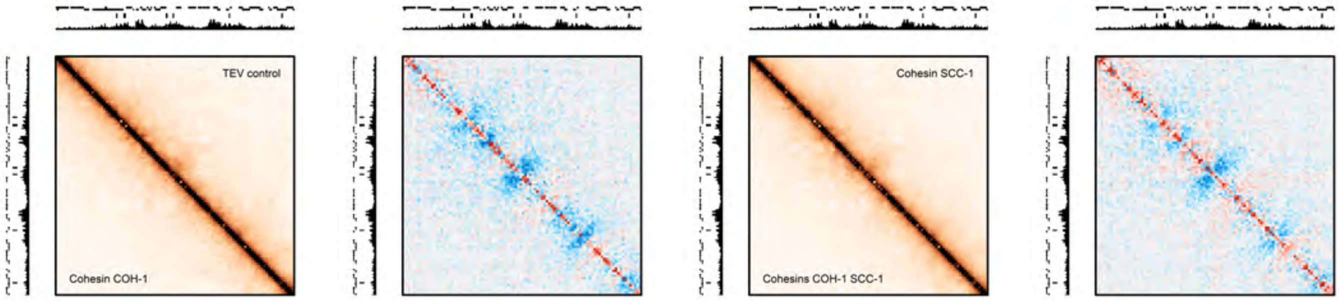

chrX:13255001-13455000

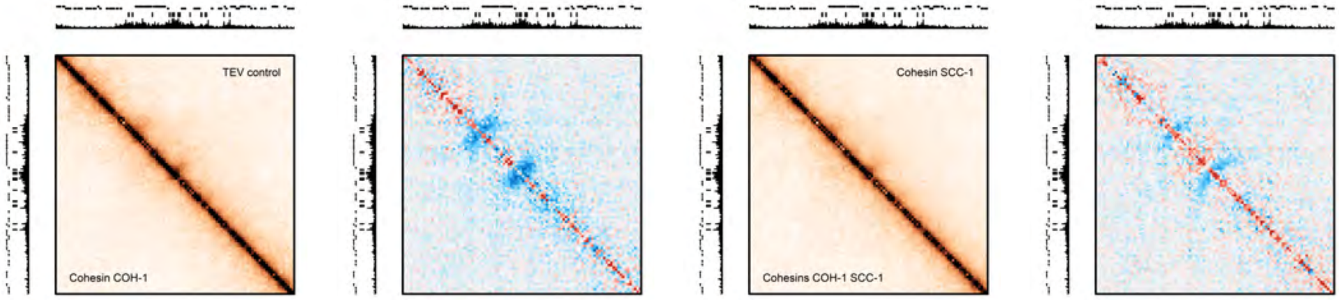

chrIII:1123001-1323000

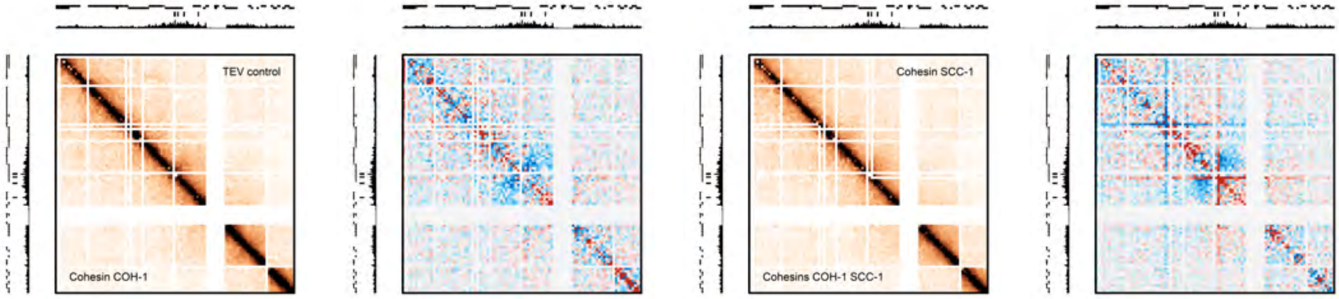

chrV:7747001-7947000

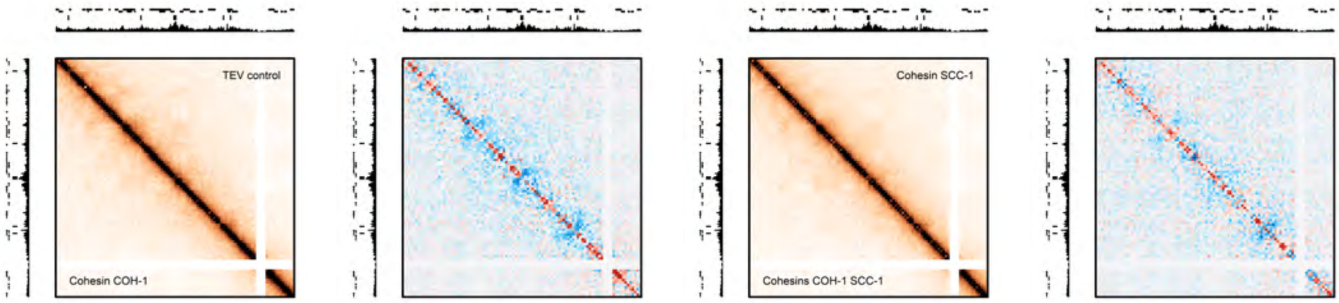

chrI:3209001-3409000

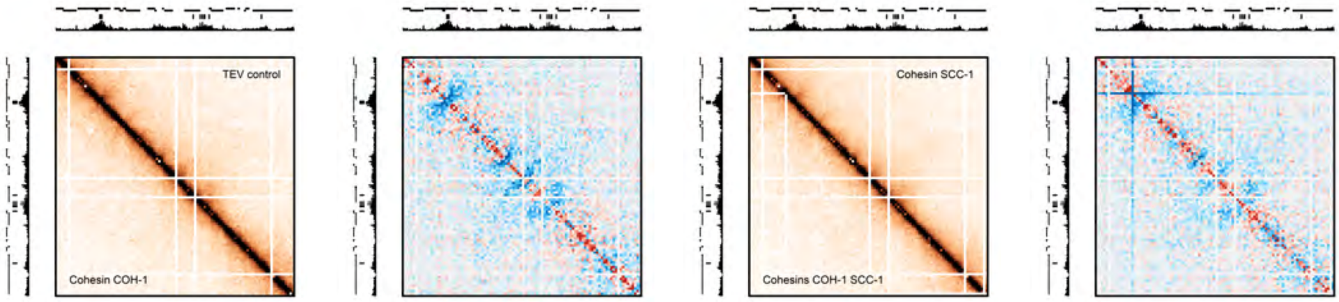

chrI:3727001-3927000

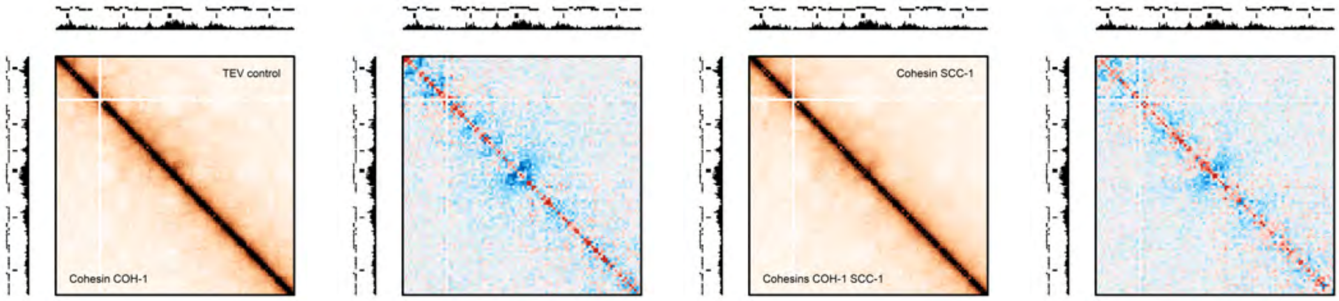

chrIV:731001-931000

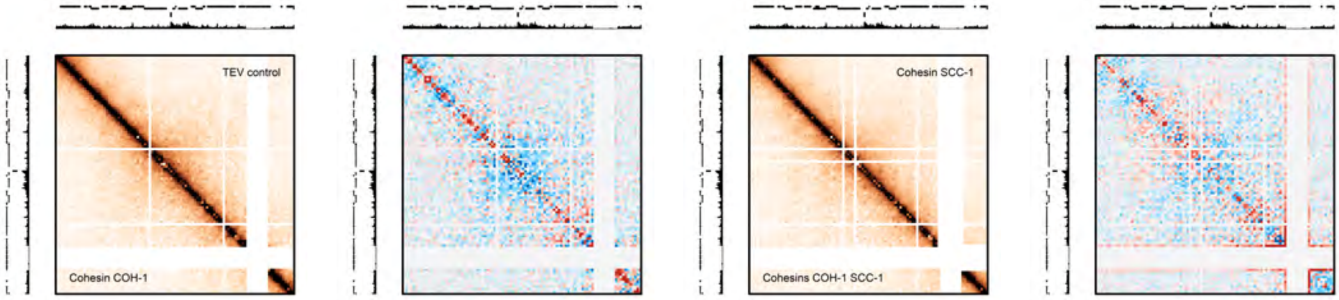

chrII:4807001-5007000

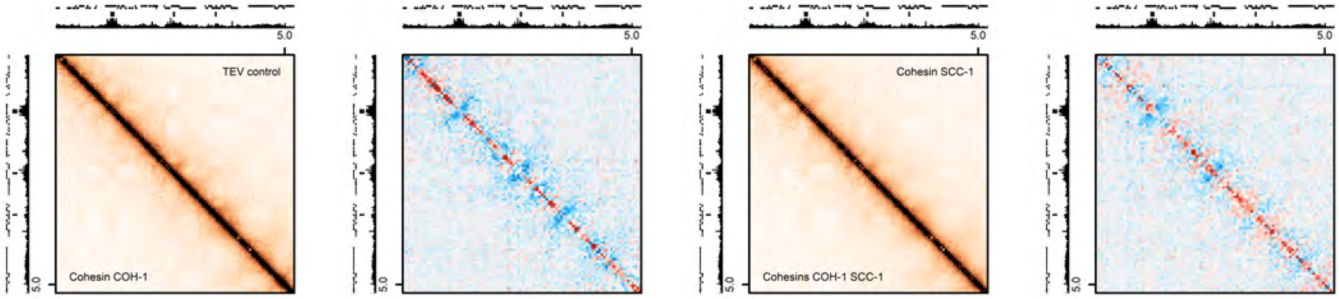

chrI:779001-979000

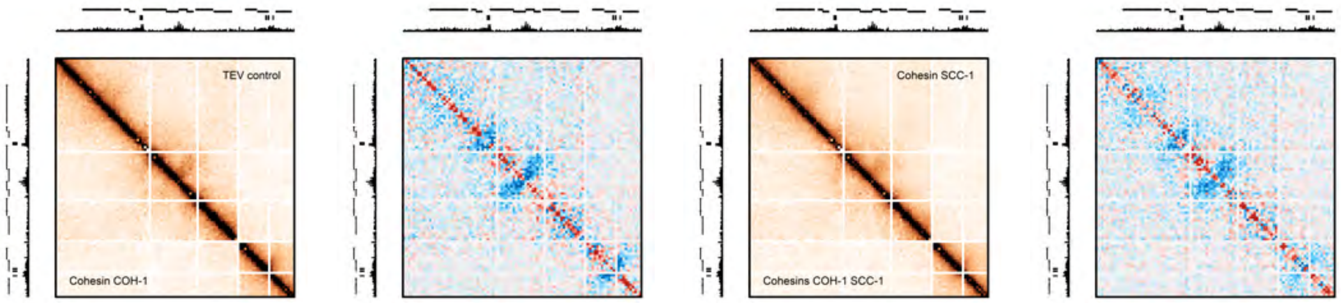

chrIII:6505001-6705000

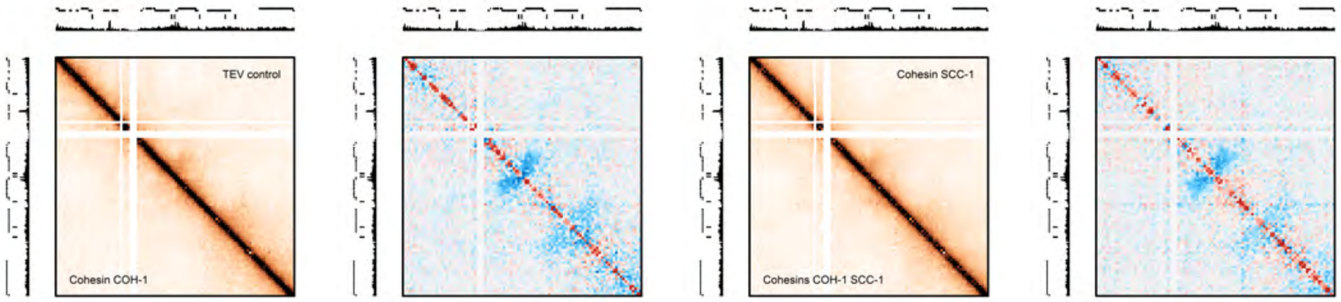

chrI:3909001-4109000

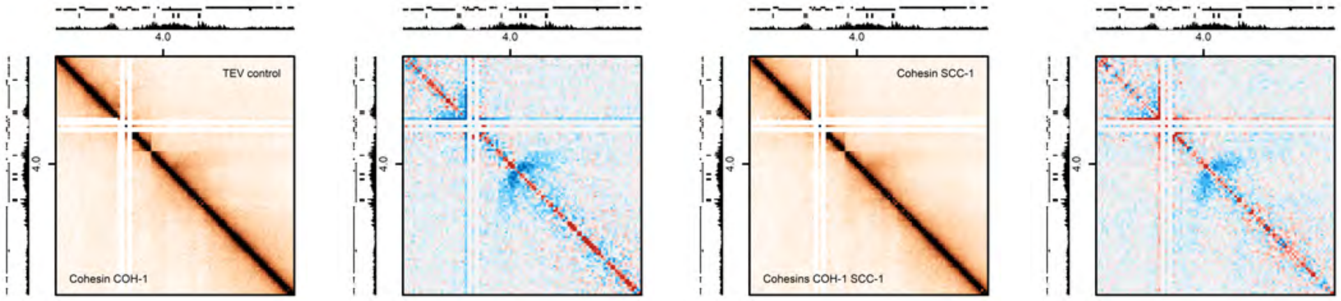

chrIII:-70999-129000

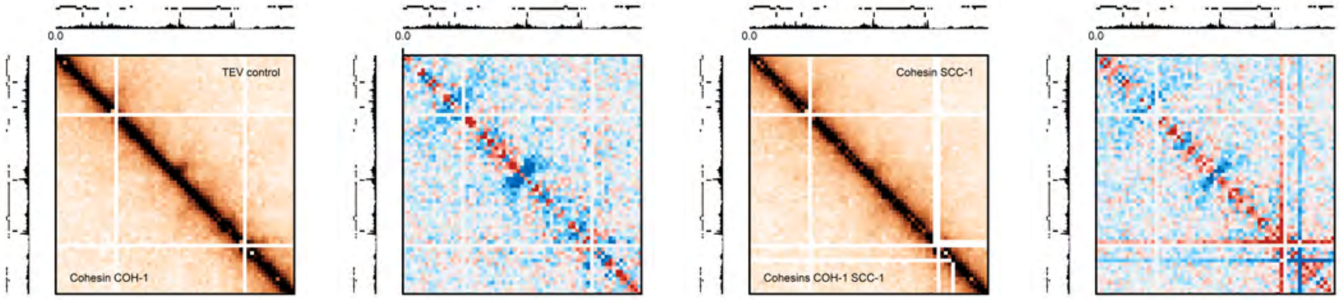

chrI:10463001-10663000

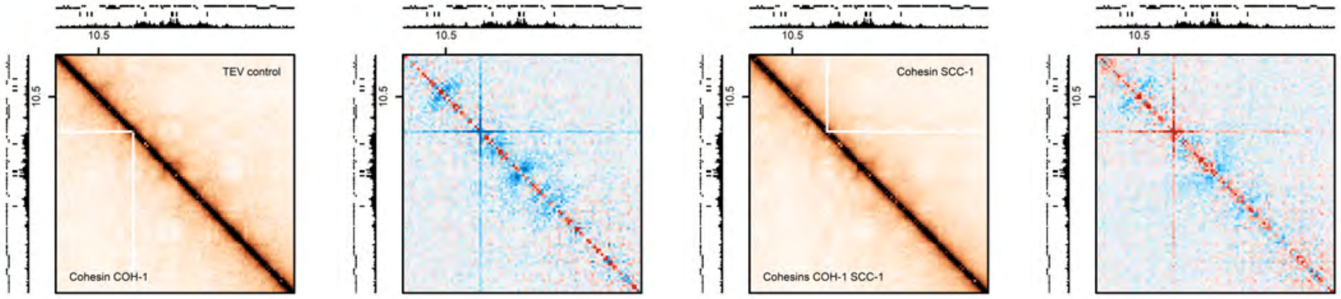

chrIII:7405001-7605000

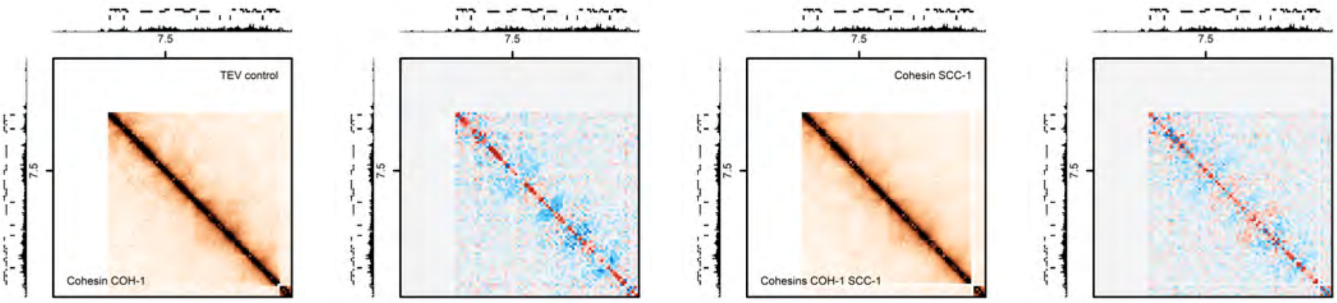

chrIV:8133001-8333000

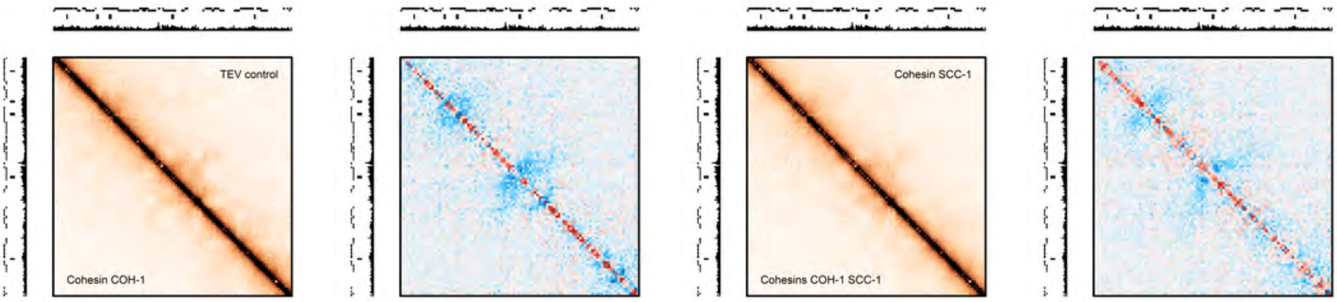

chrII:11941001-12141000

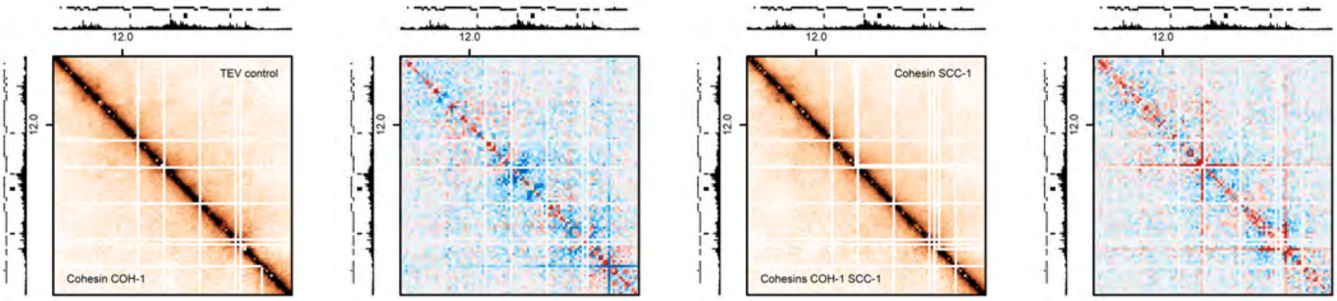

chrIV:13935001-14135000

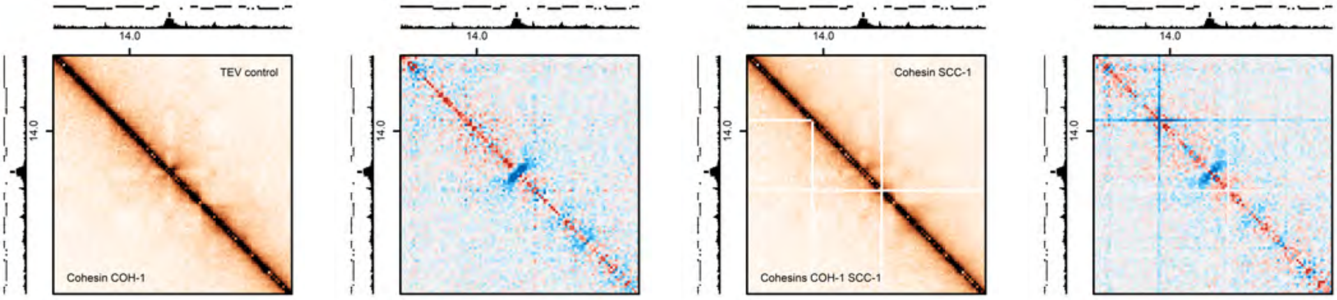

chrIII:3573001-3773000

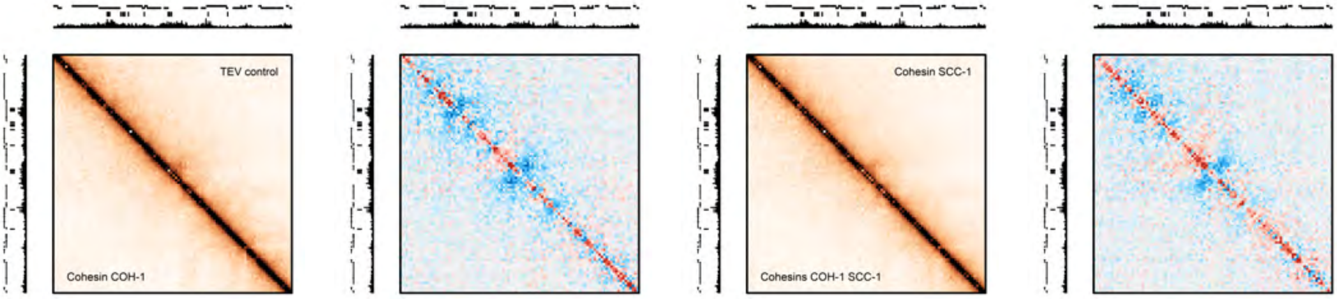

chrIV:12517001-12717000

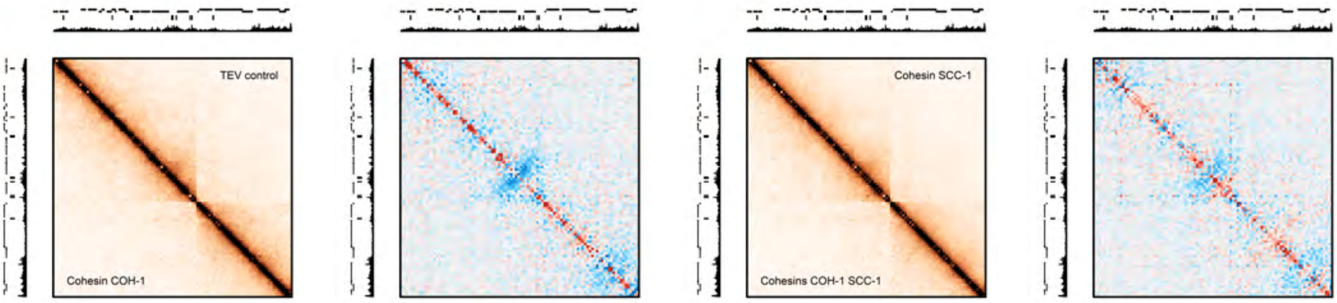

chrIV:9341001-9541000

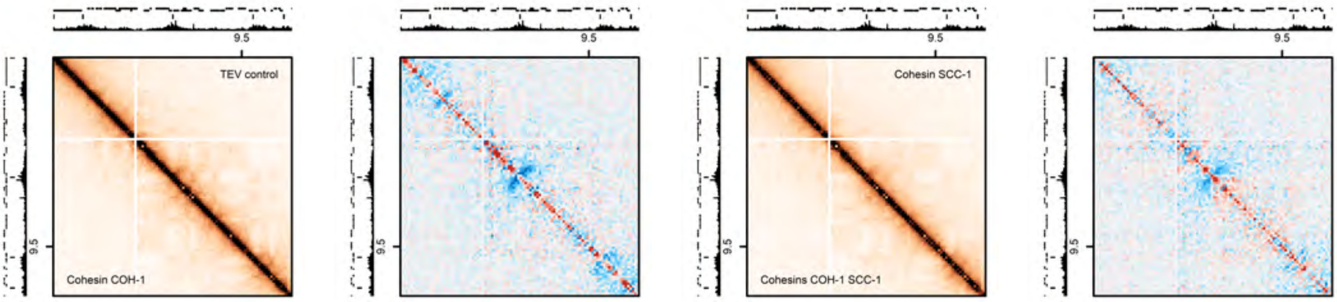

chrIV:2671001-2871000

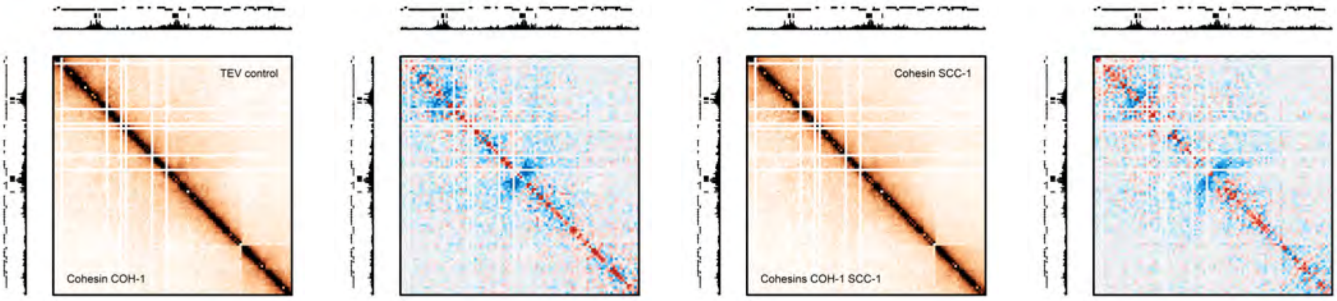

chrV:13291001-13491000

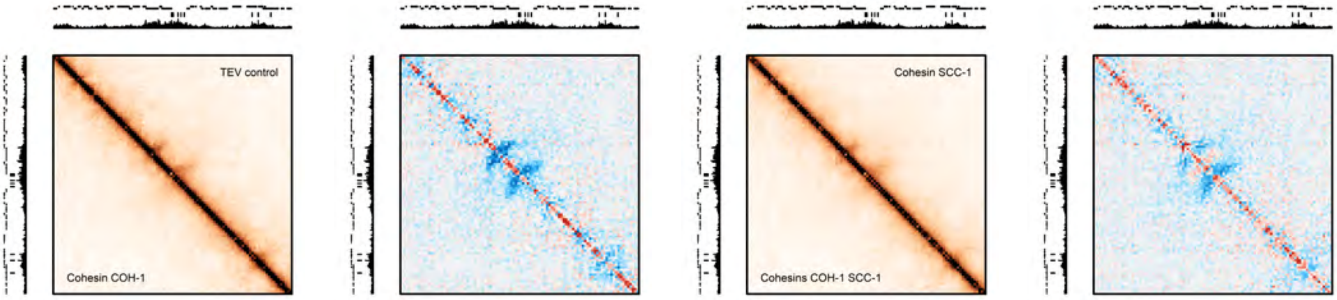

chrIV:7303001-7503000

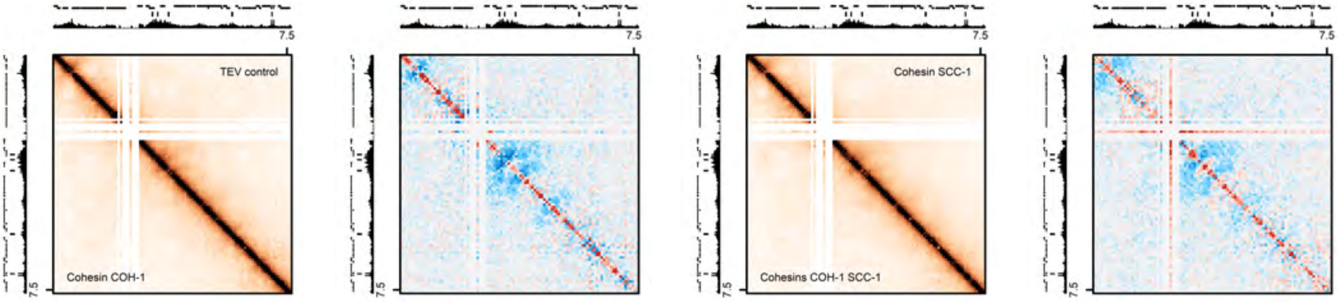

chrI:2583001-2783000

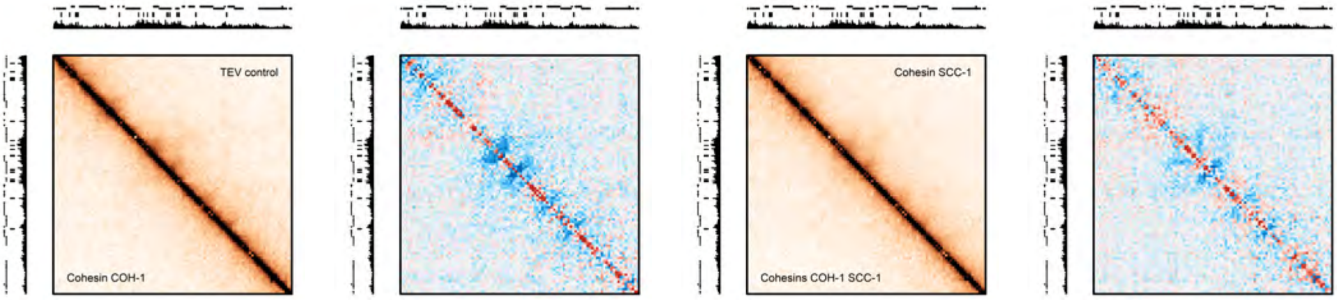

chrX:5941001-6141000

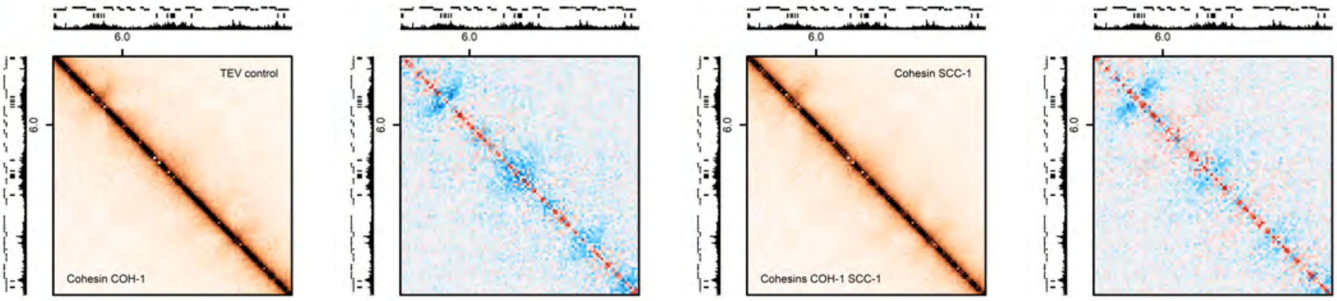

chrV:16497001-16697000

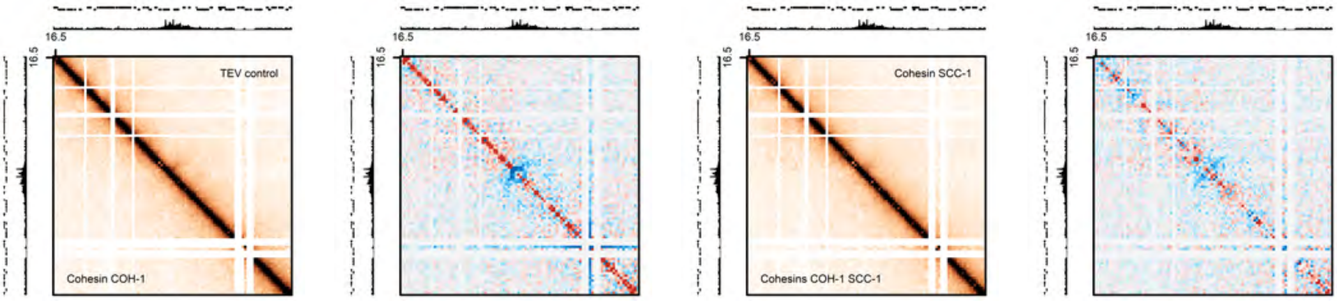

chrIII:3879001-4079000

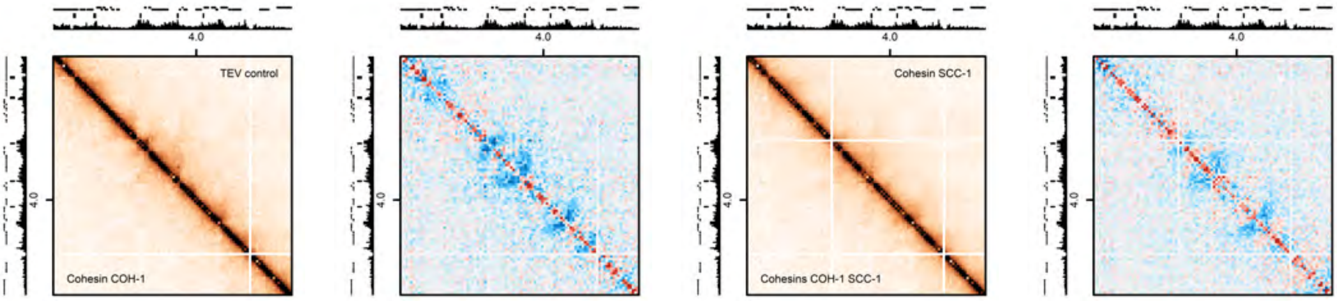

chrIV:5493001-5693000

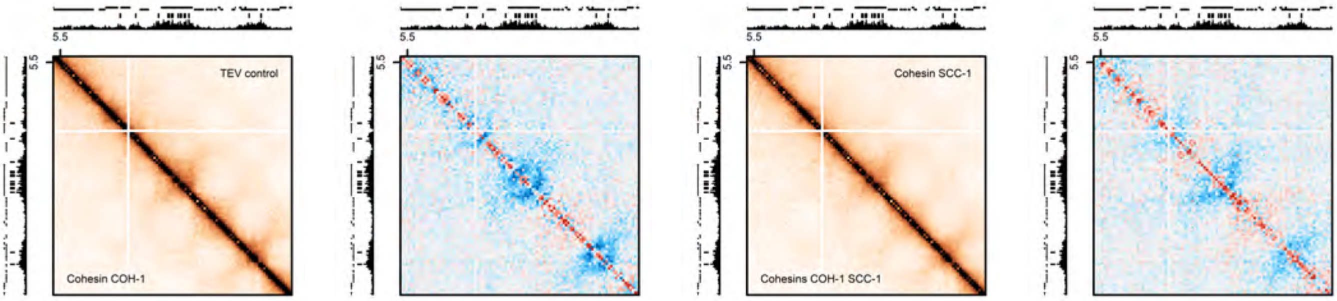

chrIV:8697001-8897000

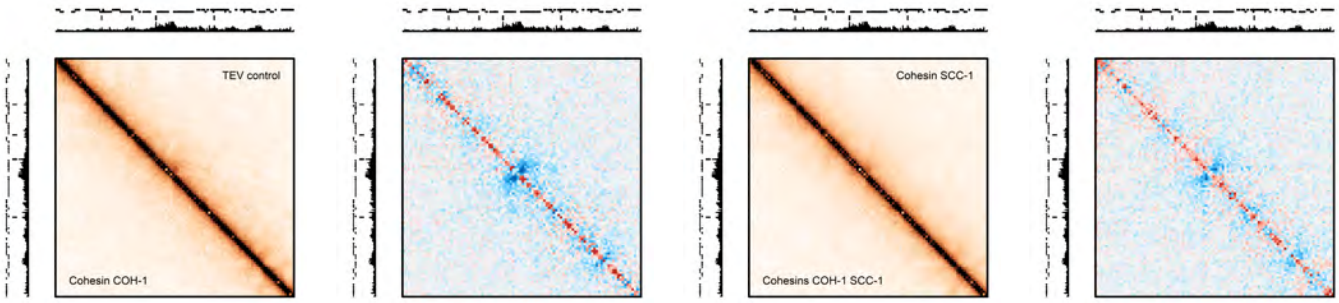

chrV:15561001-15761000

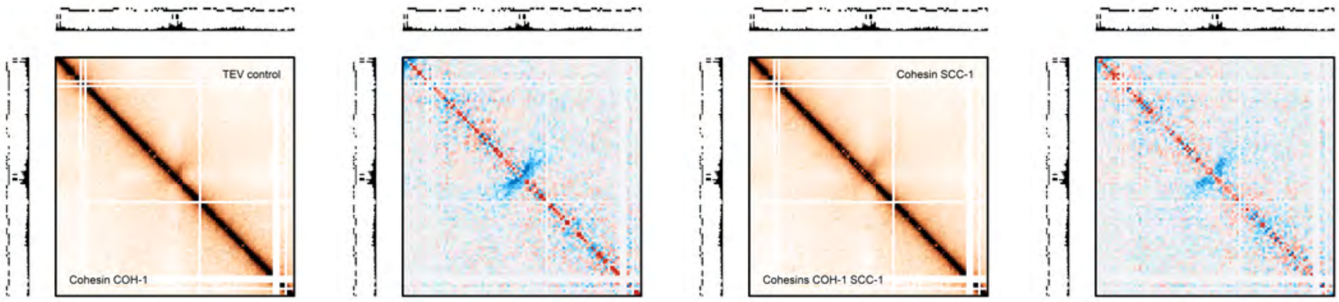

chrII:7539001-7739000

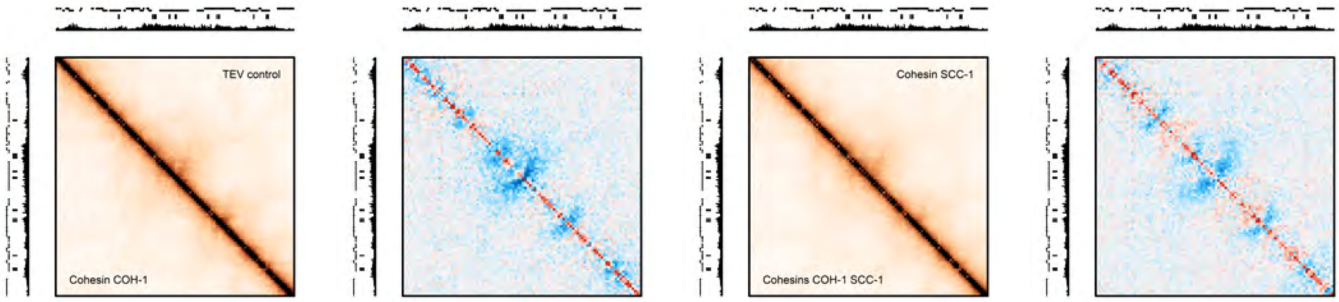

chrV:6415001-6615000

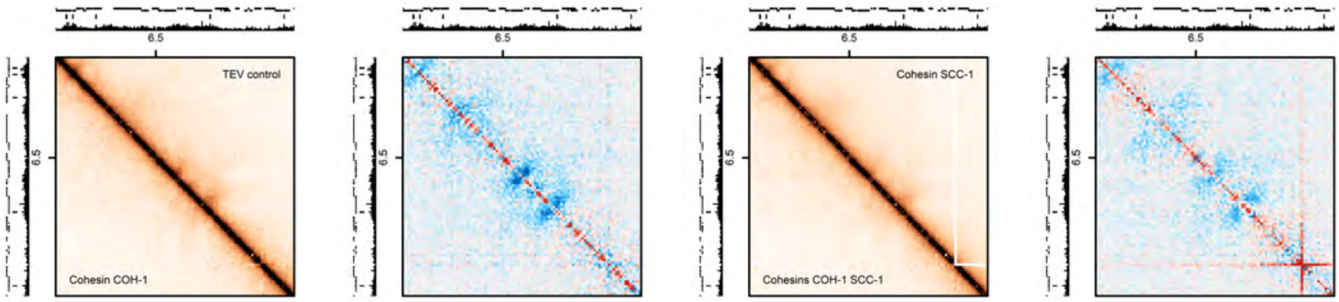

chrI:8205001-8405000

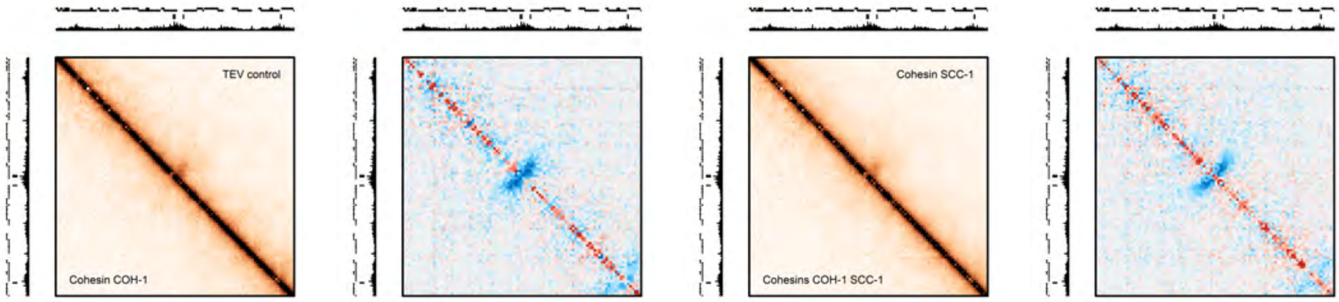

chrV:16681001-16881000

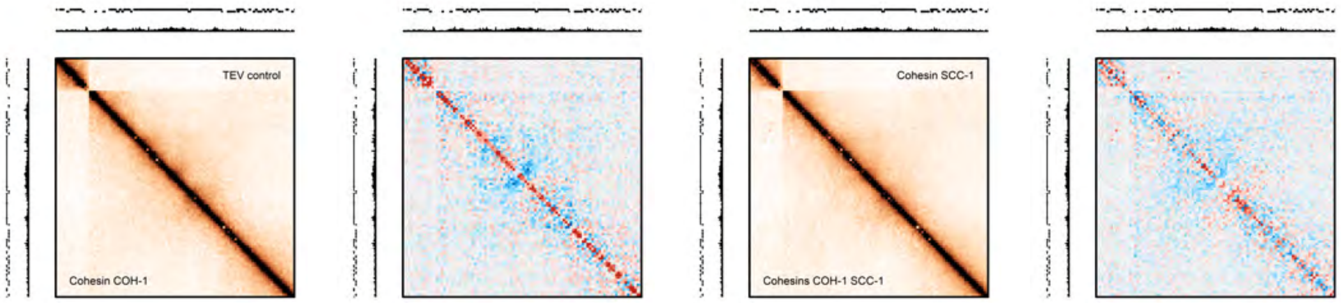

chrI:7561001-7761000

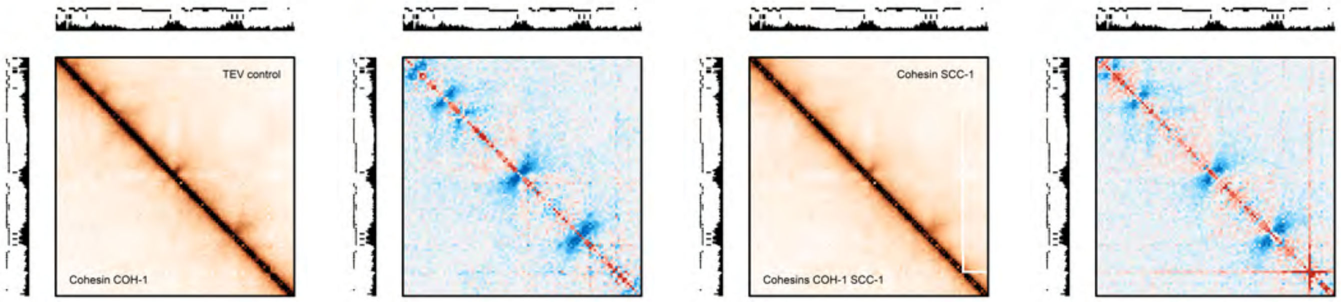

chrI:4393001-4593000

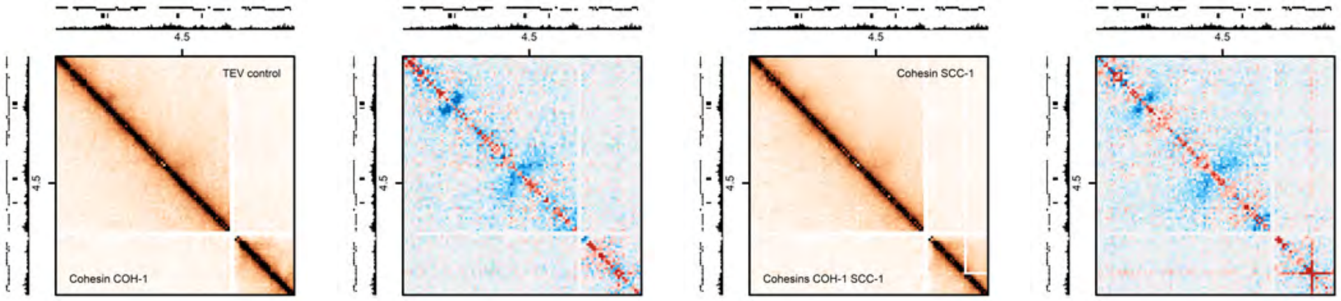

chrIII:11253001-11453000

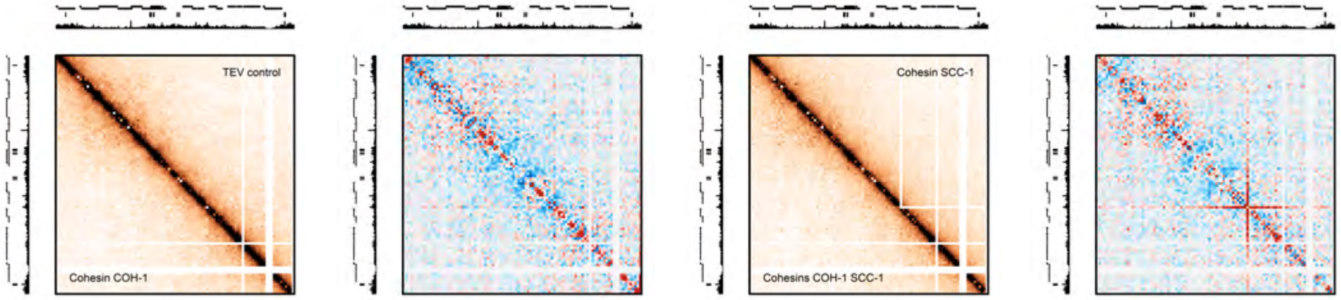

chrIV:6949001-7149000

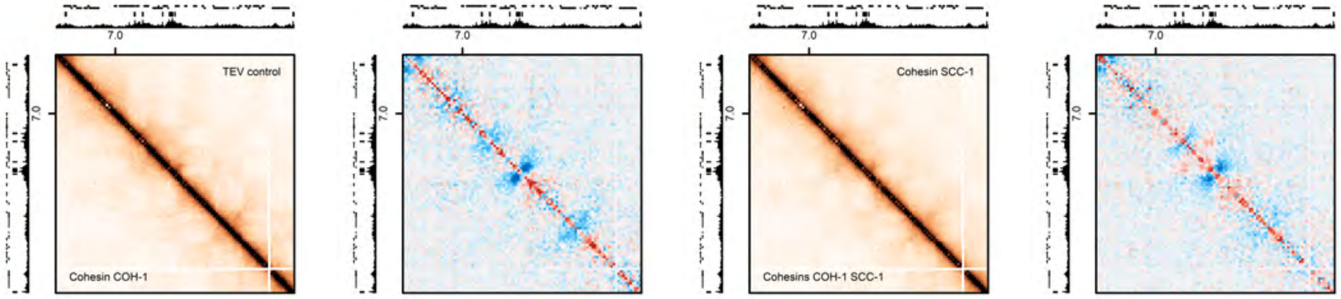

chrV:4539001-4739000

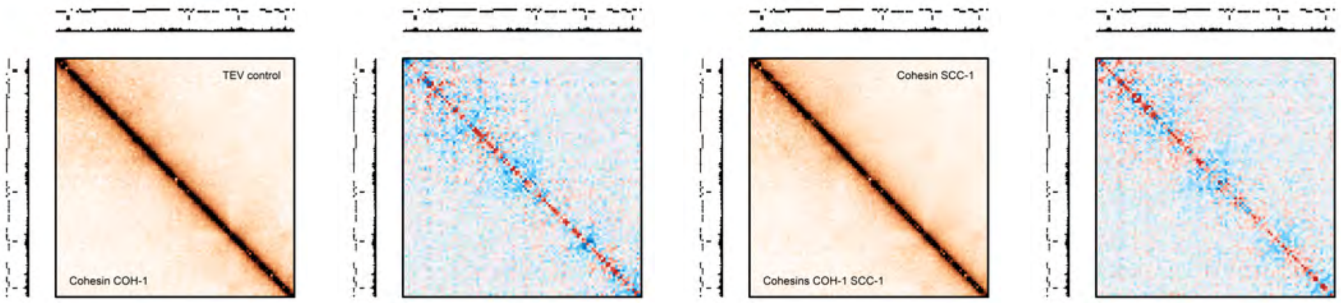

chrII:63001-263000

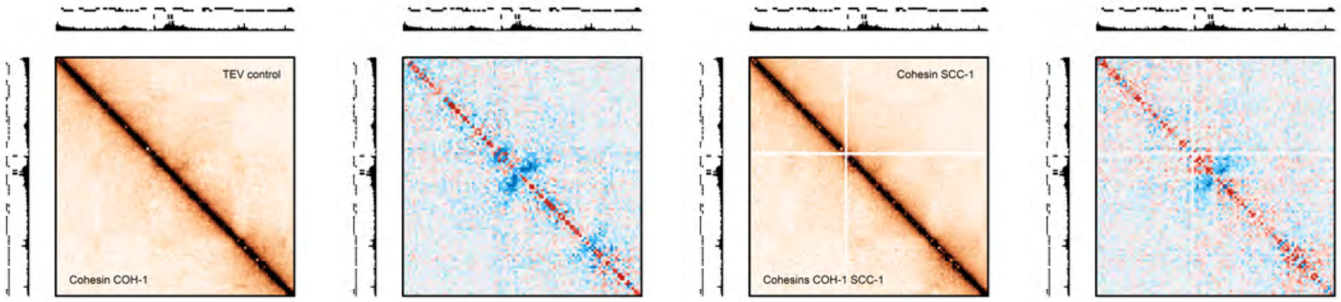

chrX:4119001-4319000

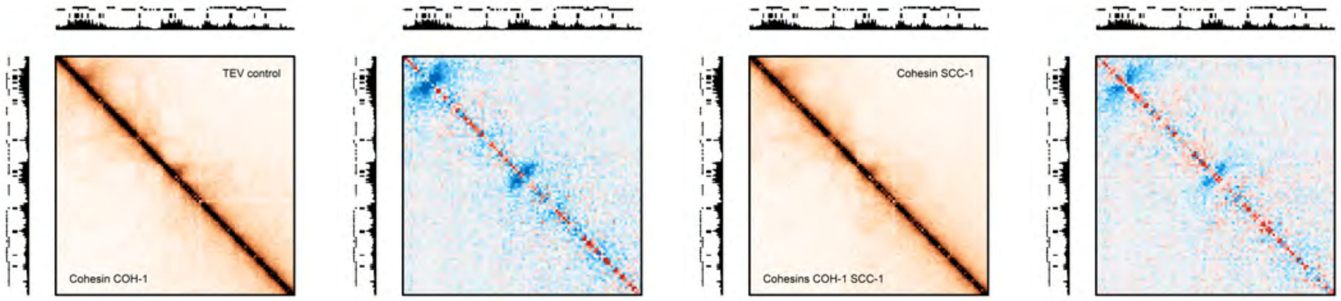

chrV:10977001-11177000

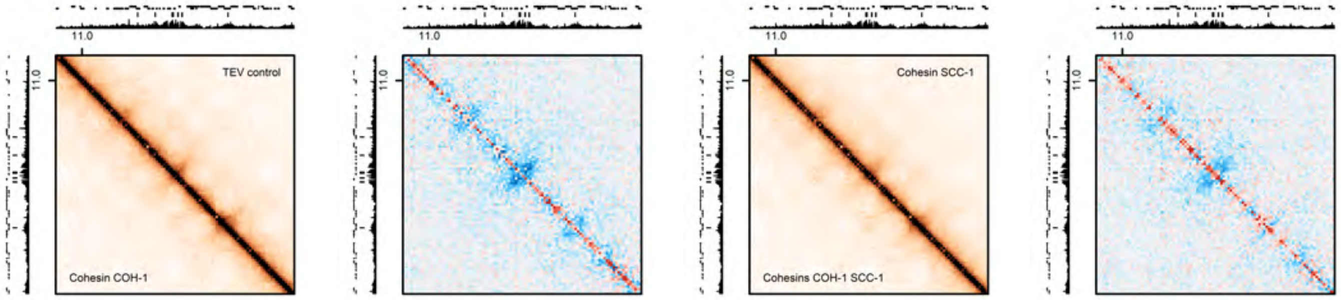

chrI:2193001-2393000

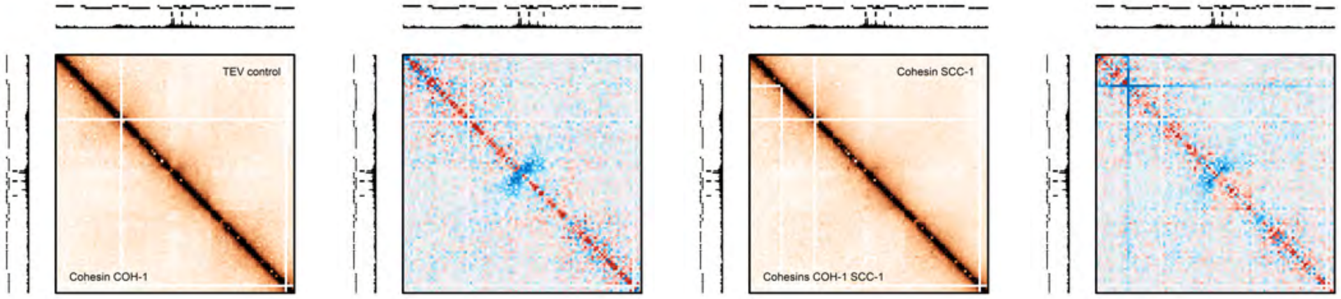

chrX:5111001-5311000

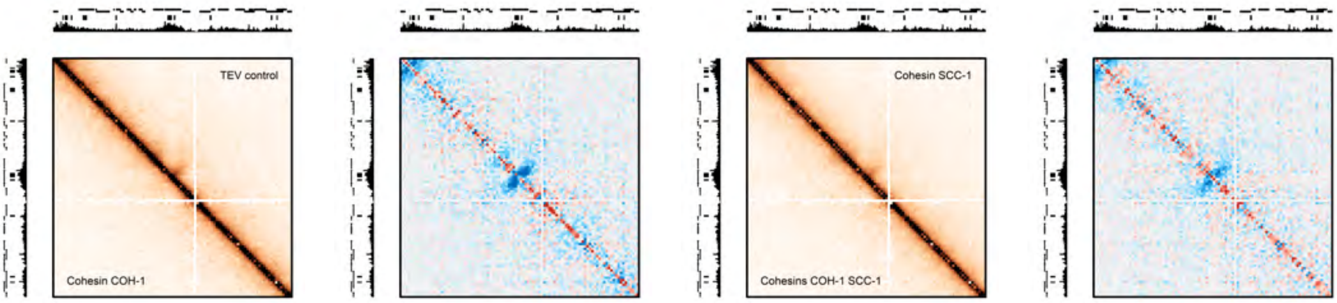

chrI:13971001-14171000

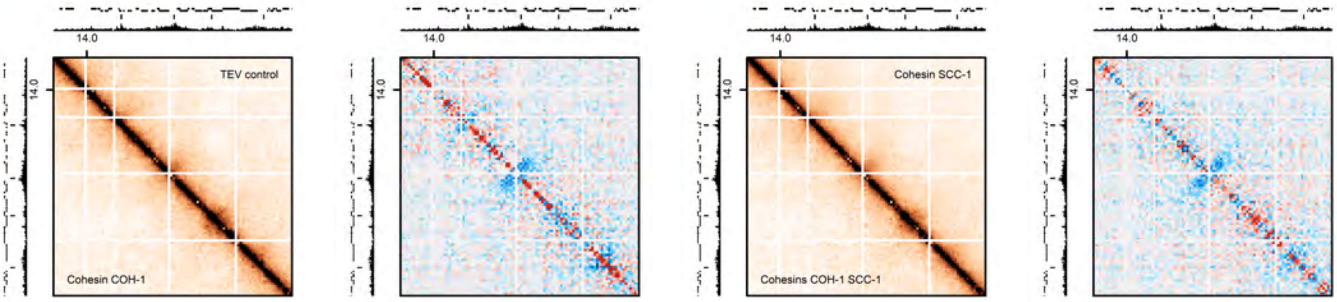

chrIII:10719001-10919000

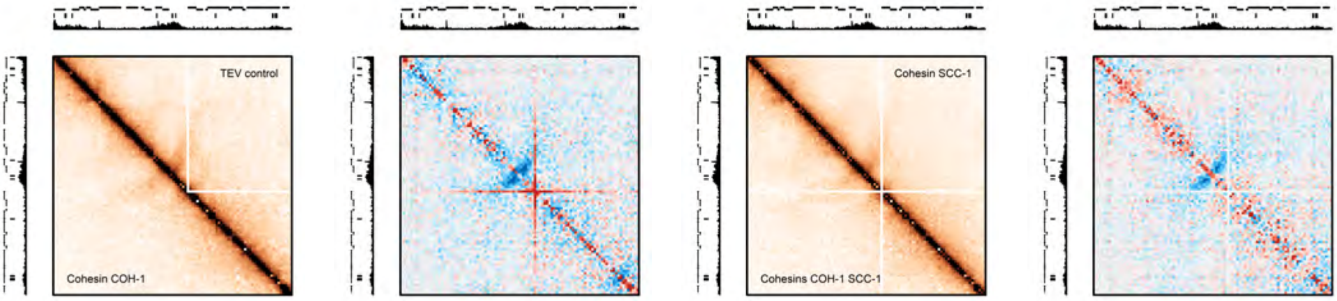

chrV:2577001-2777000

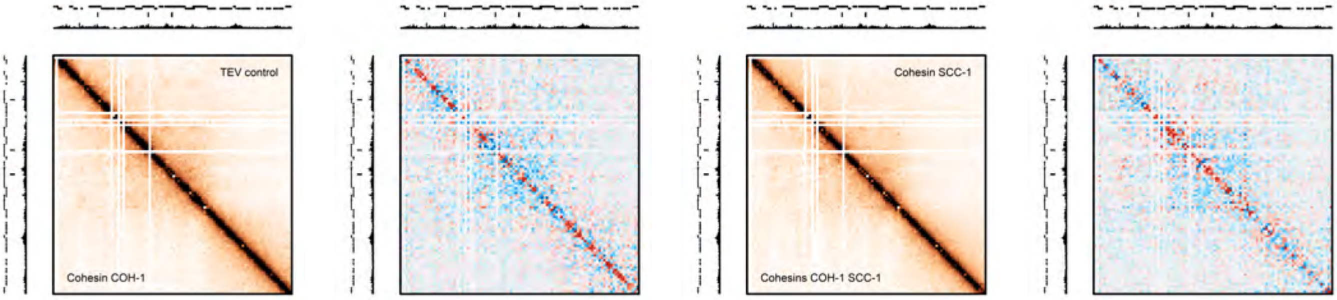

chrI:3001001-3201000

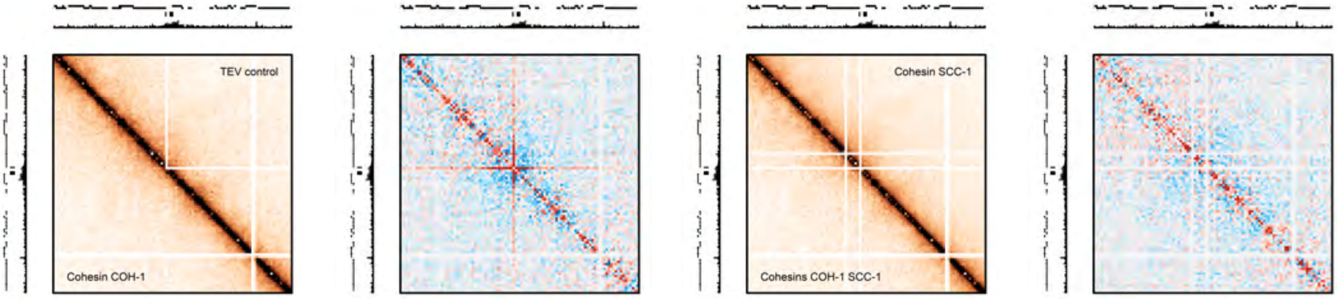

chrIV:17179001-17379000

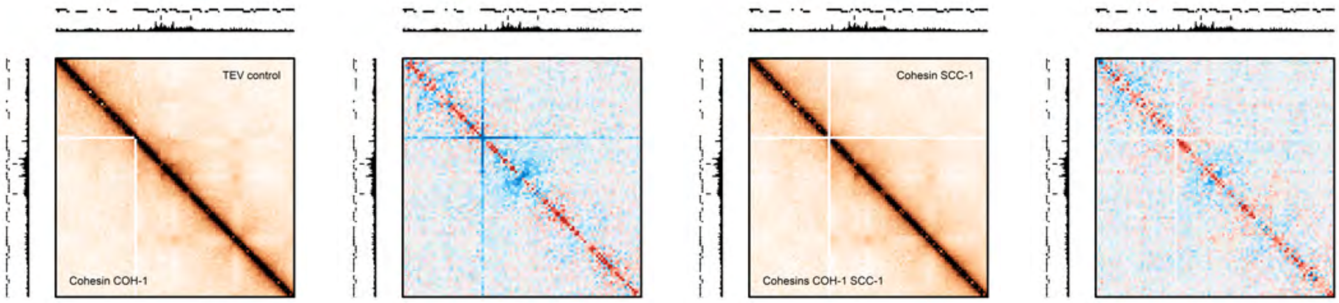

chrIII:7275001-7475000

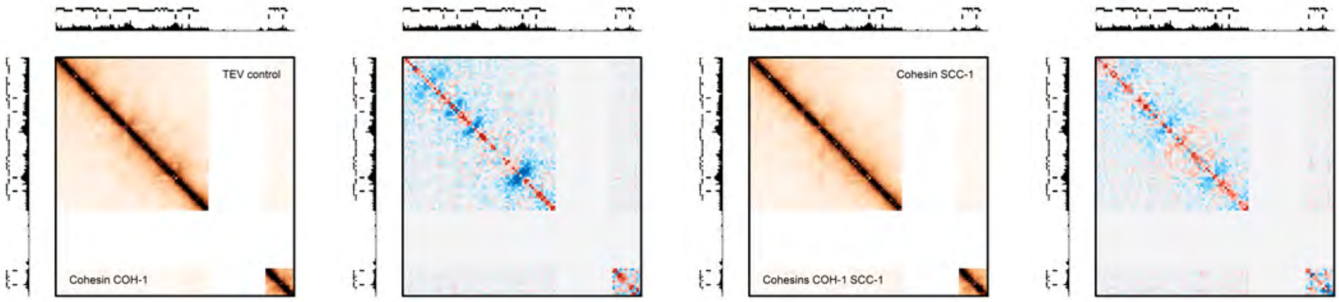

chrIV:967001-1167000

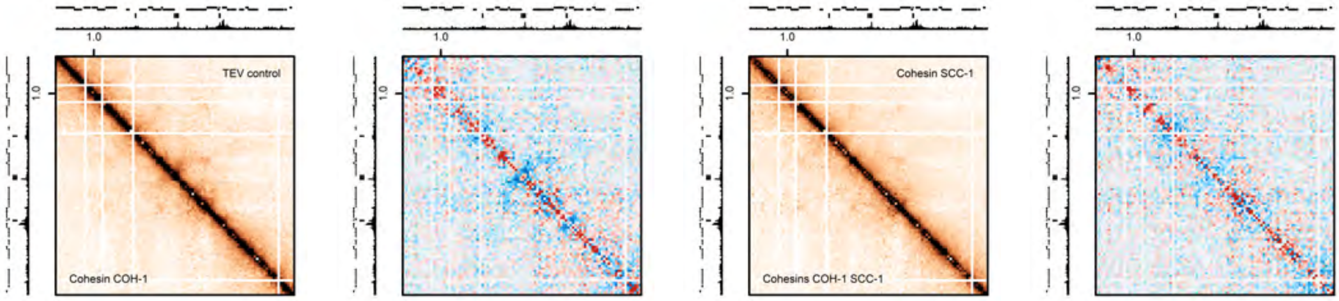

chrV:14267001-14467000

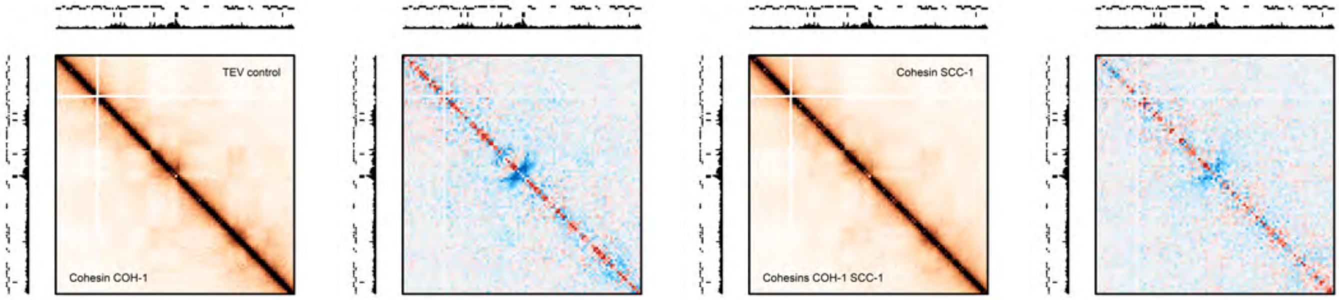

chrV:19905001-20105000

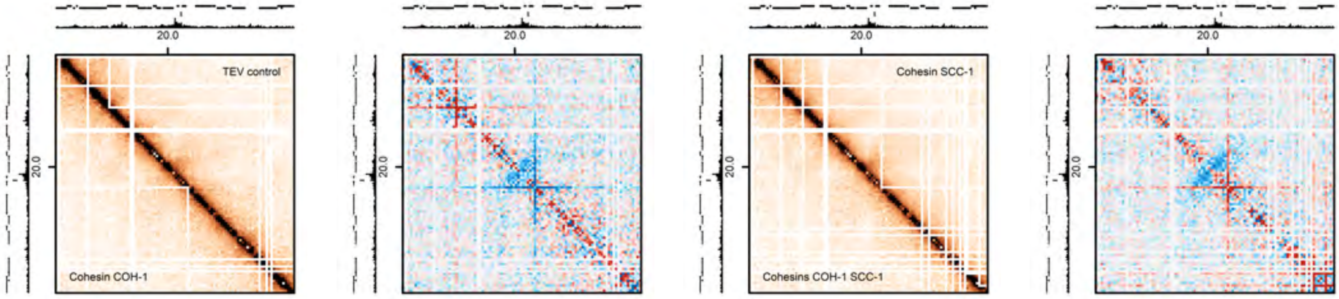

chrX:8377001-8577000

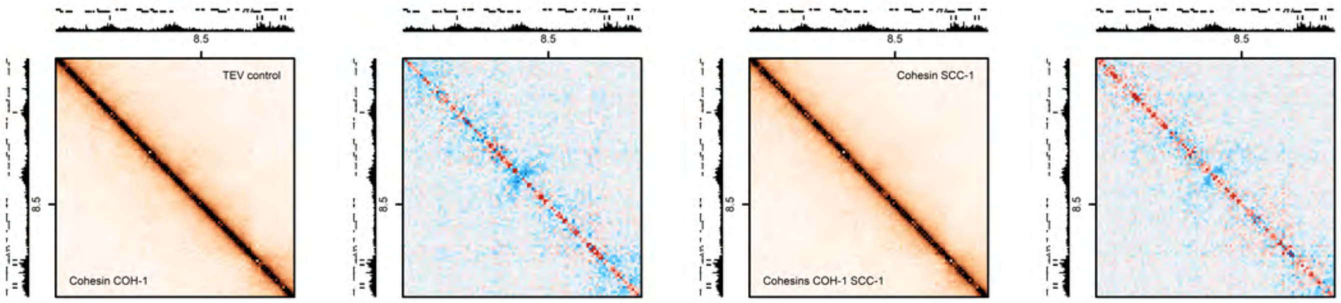

chrI:12935001-13135000

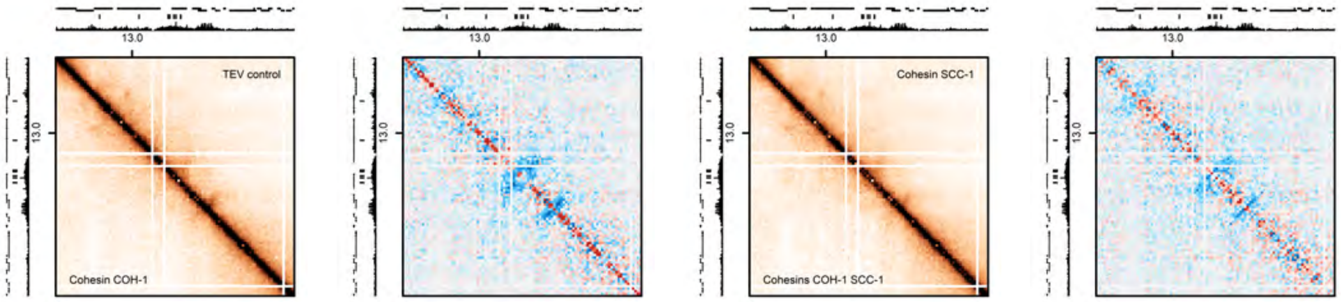

chrV:6993001-7193000

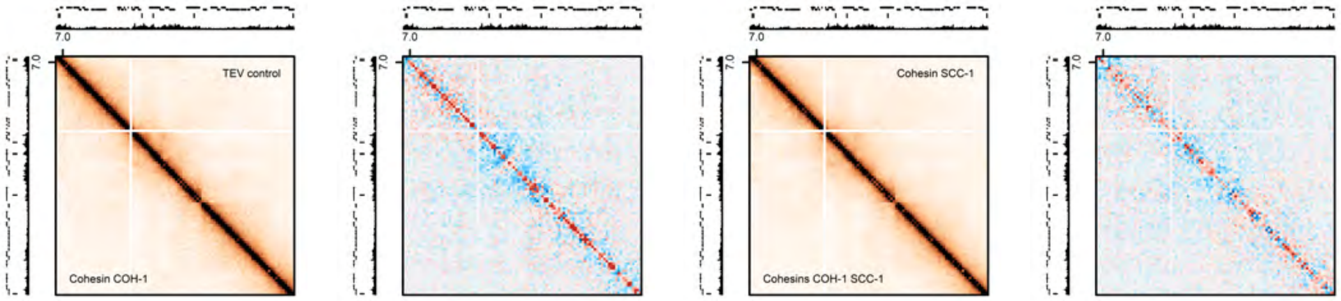

chrV:9631001-9831000

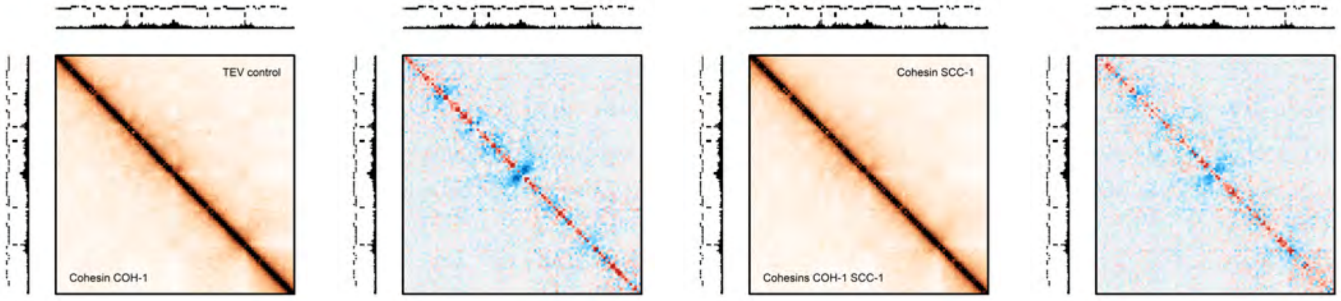

chrIII:465001-665000

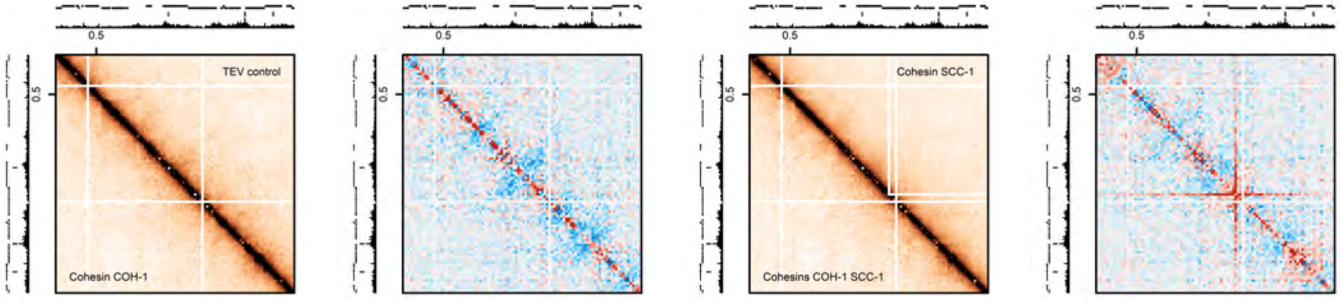

chrIII:5101001-5301000

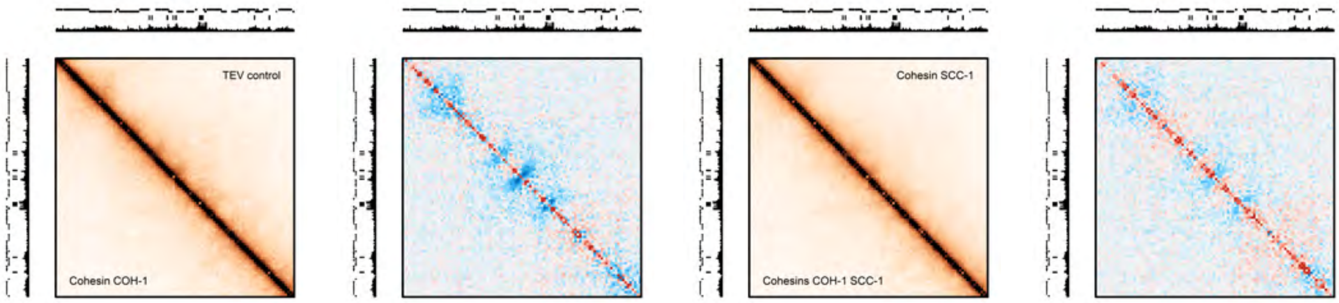

chrIII:7877001-8077000

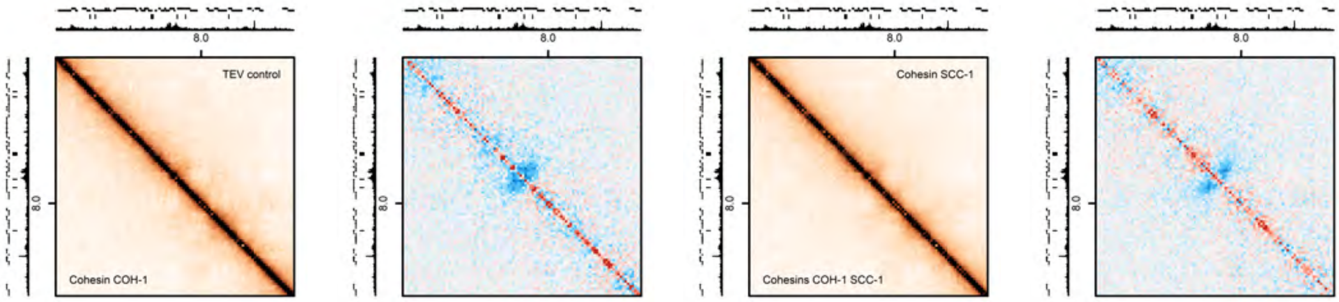

chrV:15465001-15665000

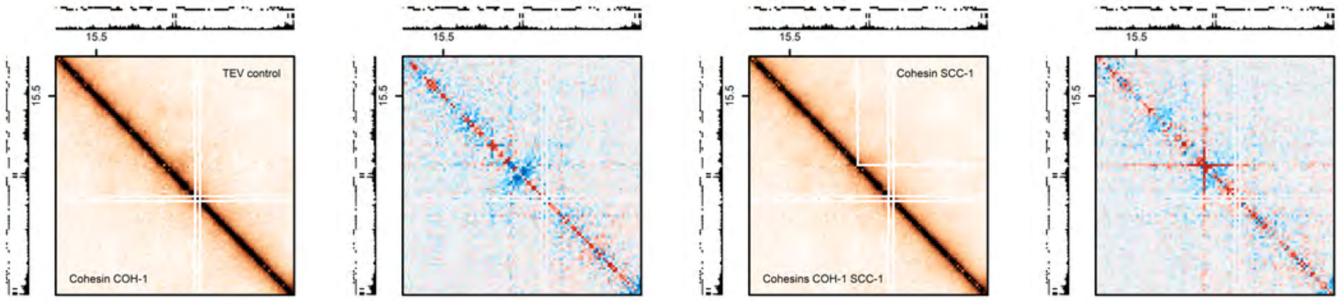

chrIV:10239001-10439000

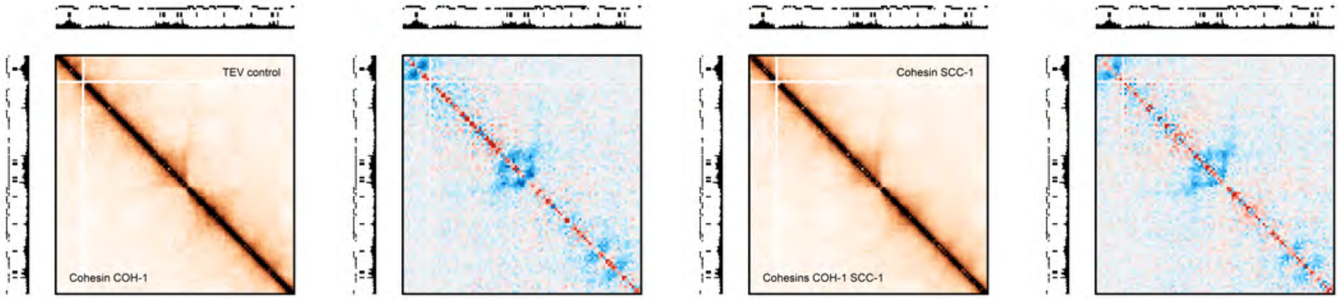

chrX:7561001-7761000

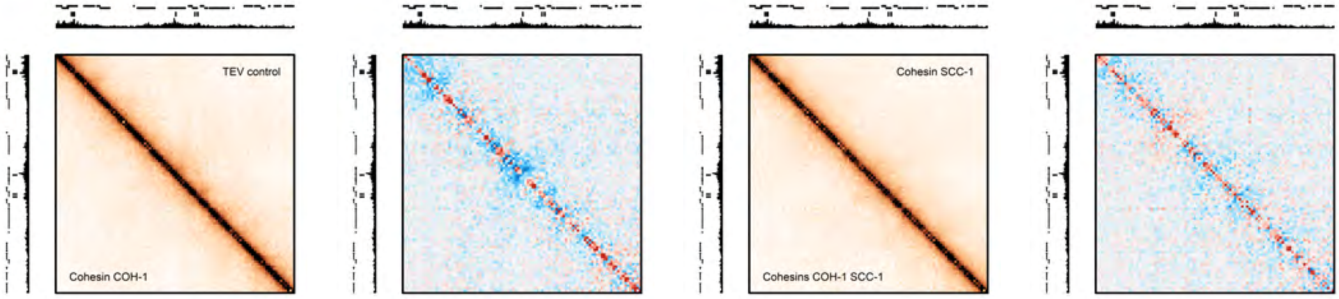

chrI:1463001-1663000

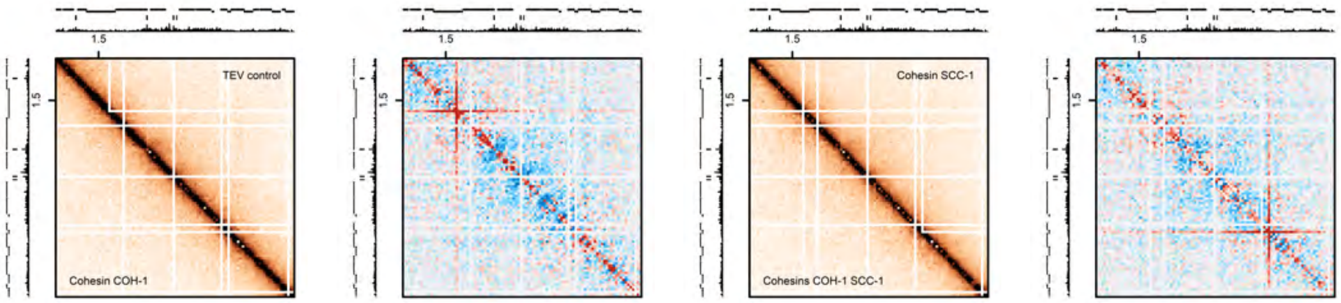

chrII:2703001-2903000

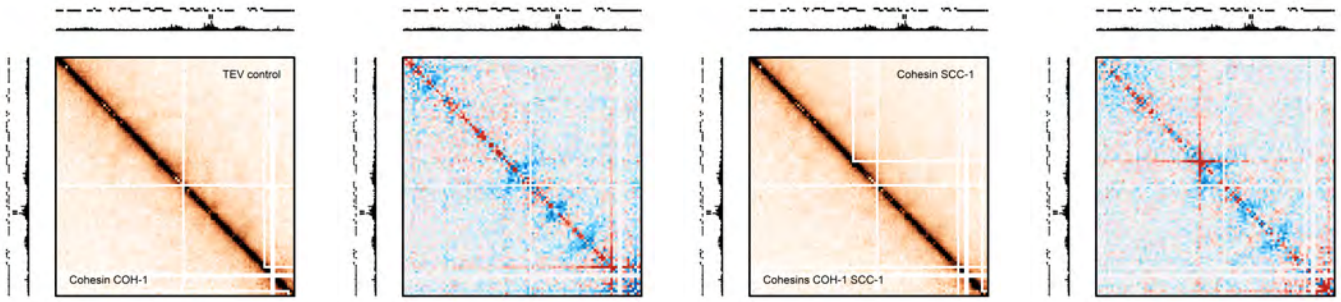

chrII:11059001-11259000

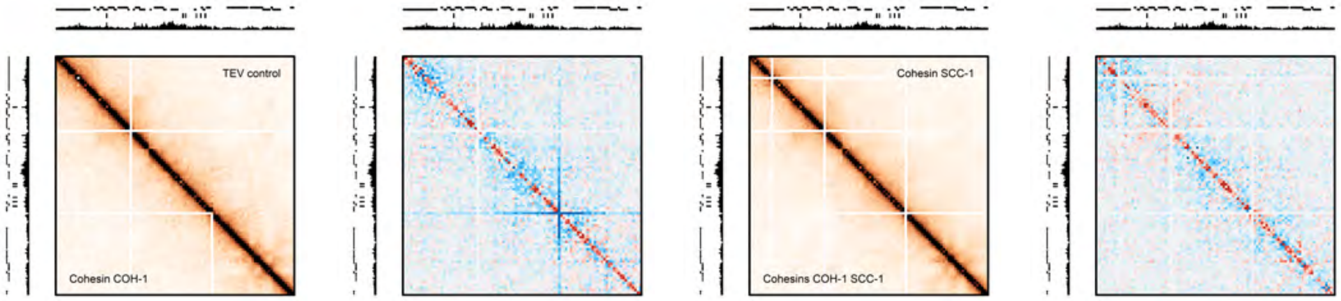

chrI:5779001-5979000

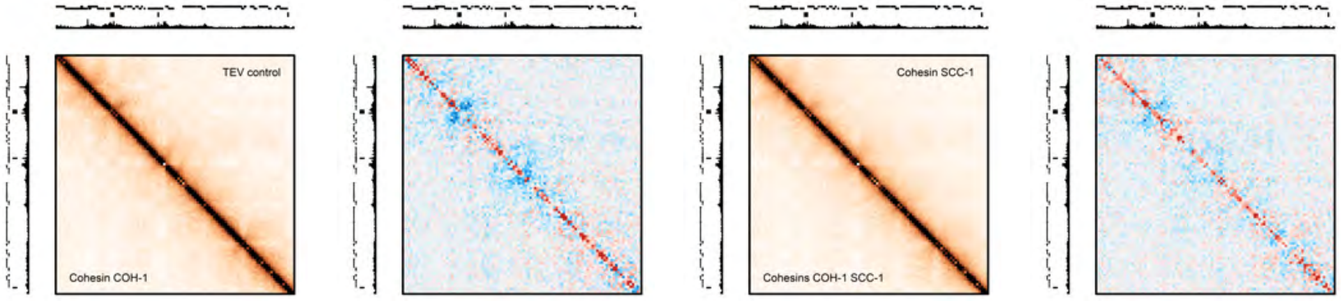

chrV:4087001-4287000

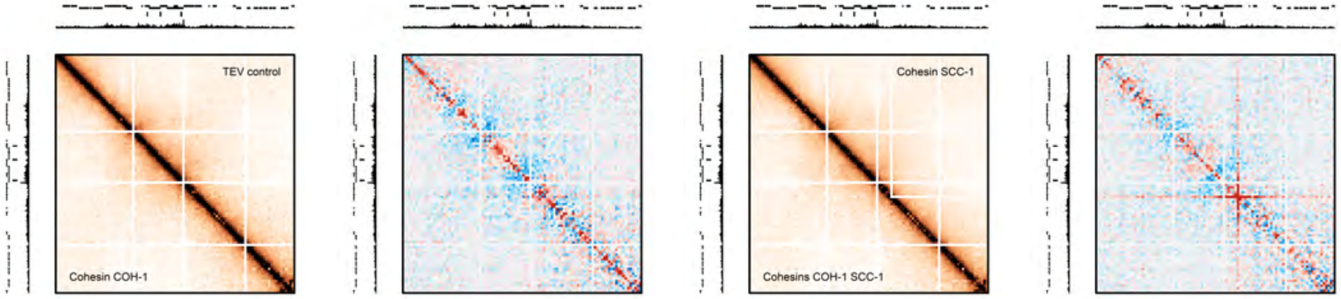

chrI:14509001-14709000

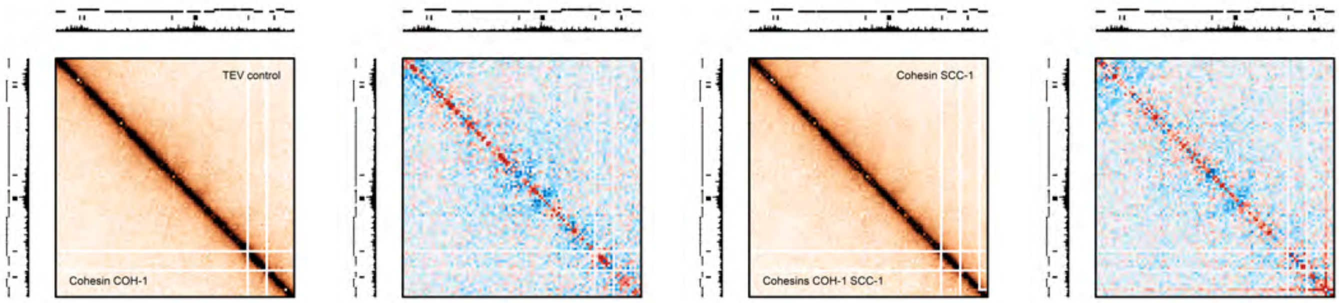

chrX:16697001-16897000

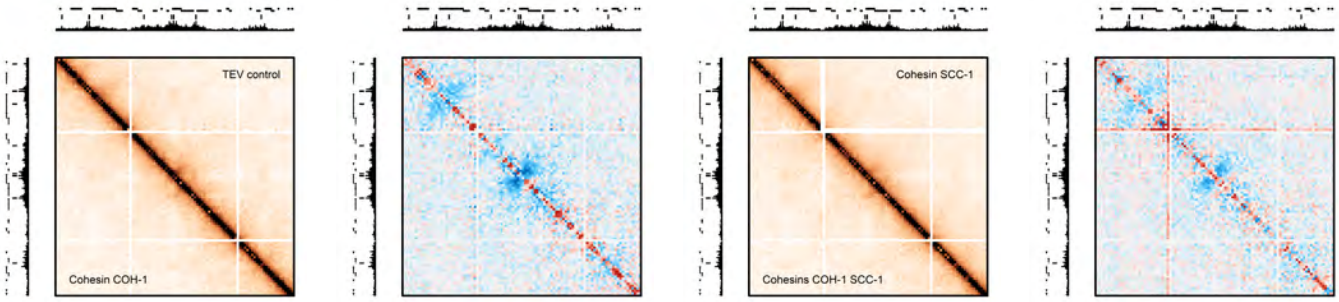

chrX:16513001-16713000

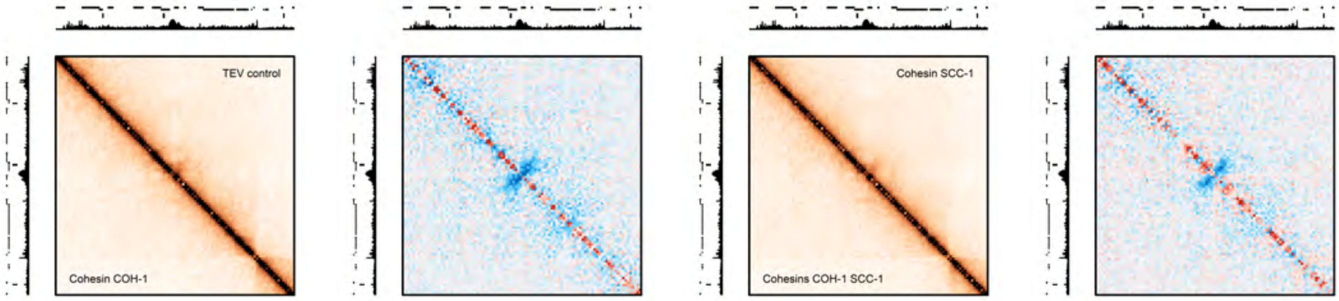

chrIII:8883001-9083000

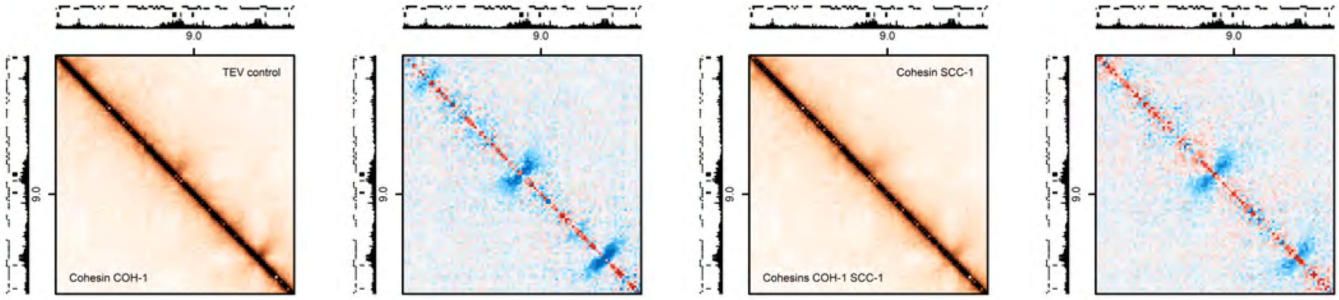

chrIII:11371001-11571000

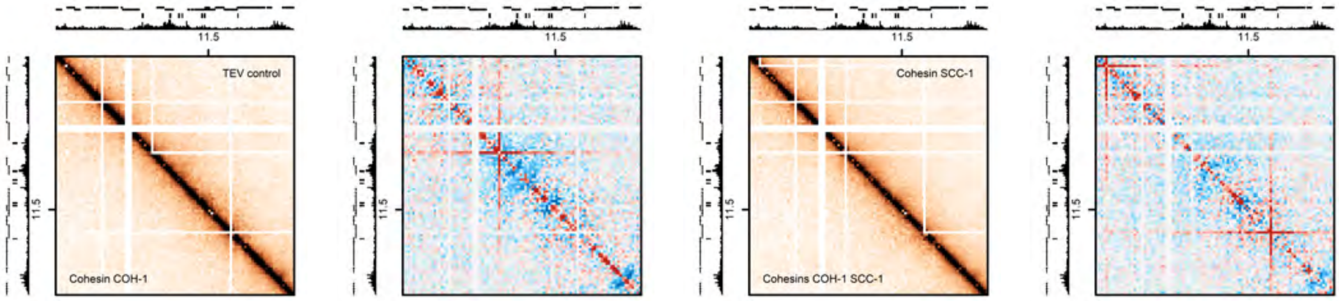

chrV:1973001-2173000

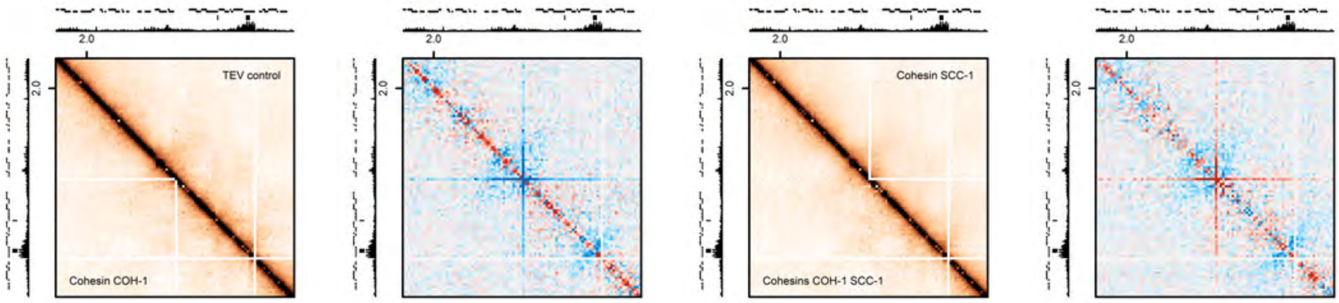

chrII:10287001-10487000

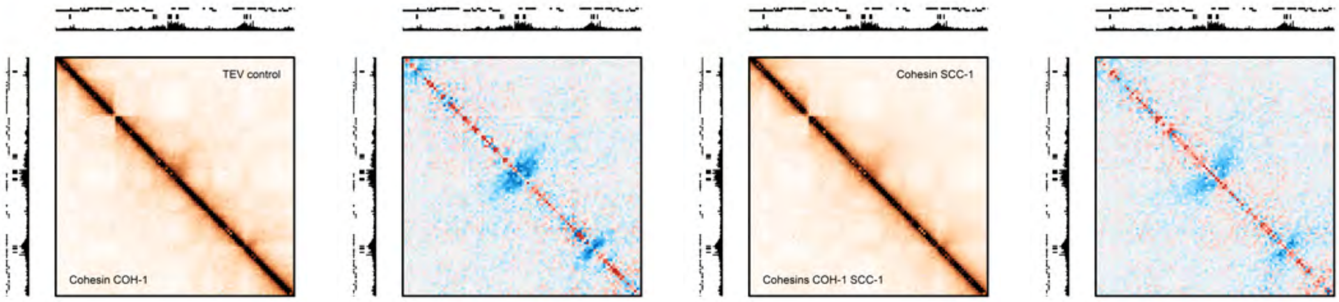

chrV:10149001-10349000

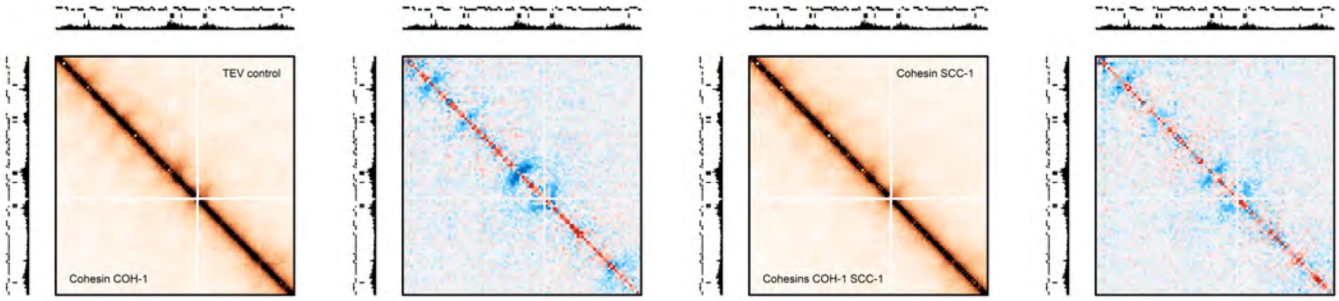

chrX:14405001-14605000

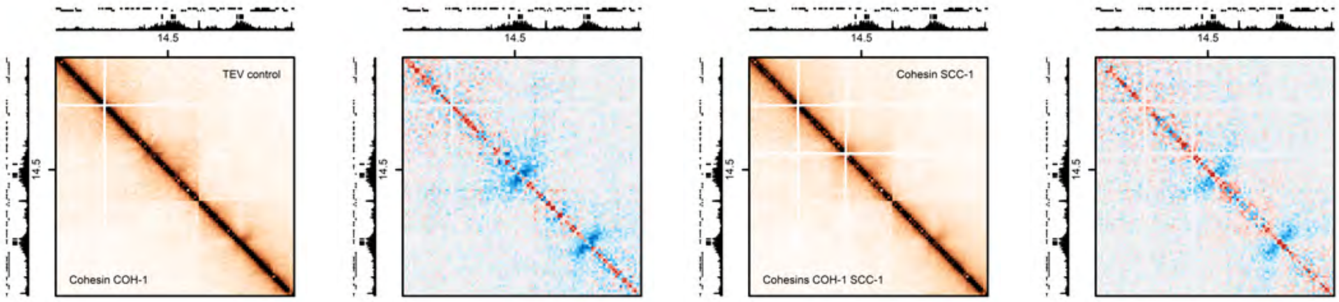

chrX:11865001-12065000

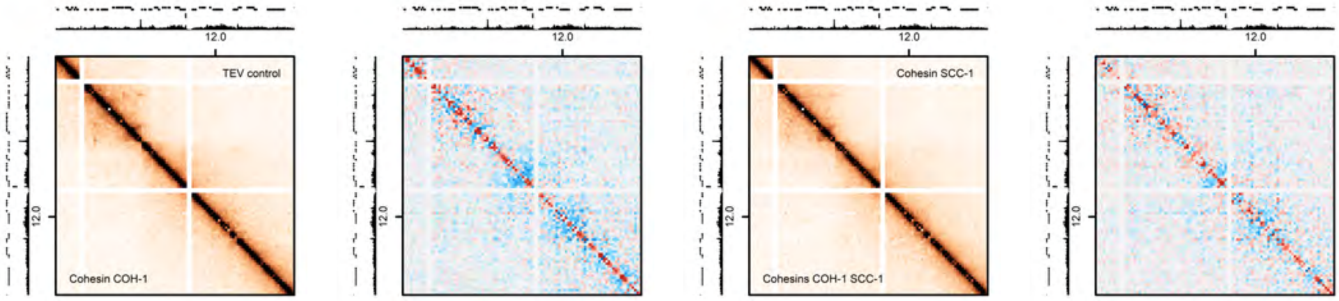

chrII:8645001-8845000

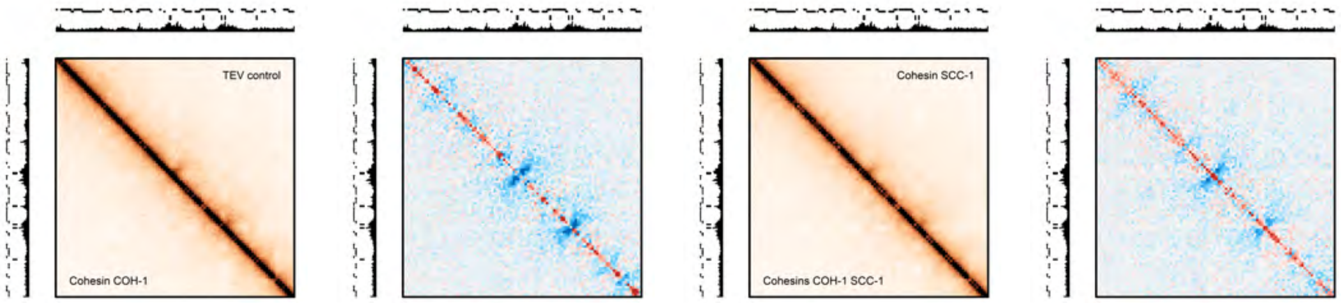

chrIII:12455001-12655000

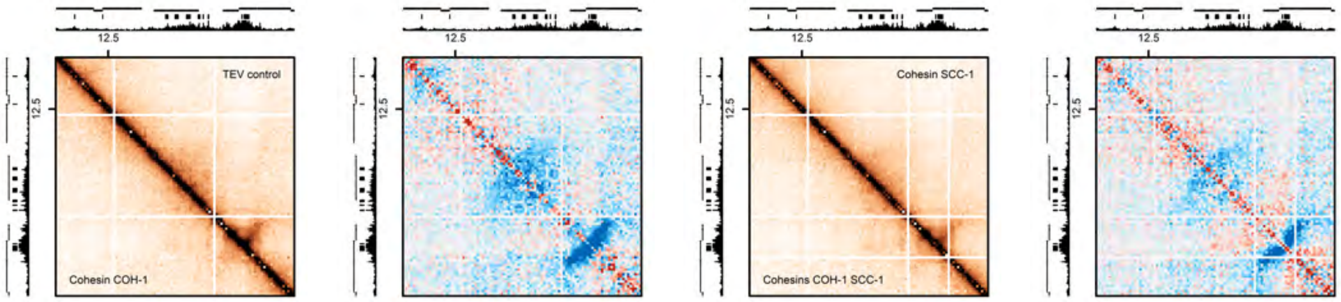

chrV:18683001-18883000

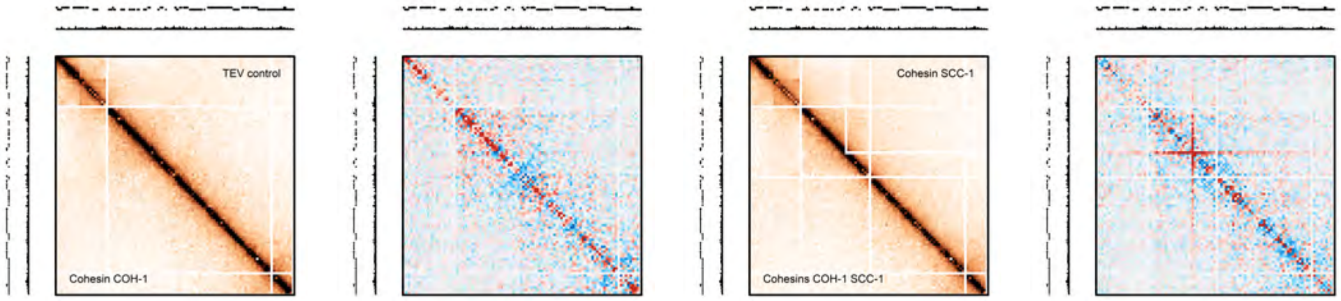

chrII:4193001-4393000

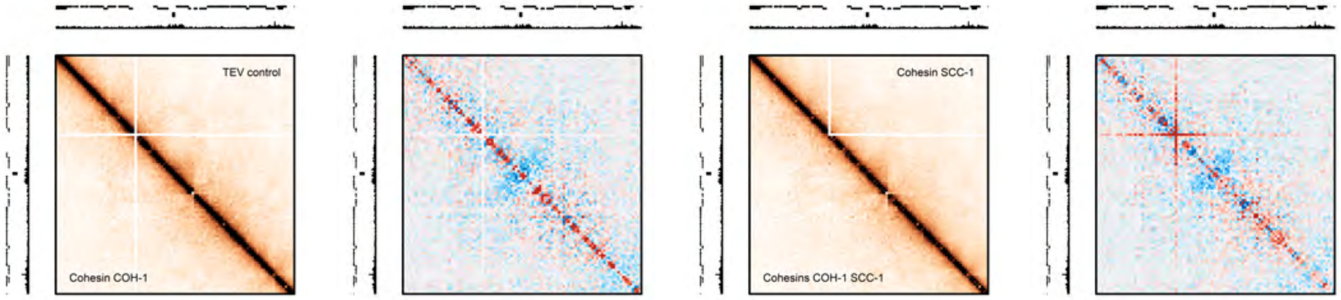

chrX:4863001-5063000

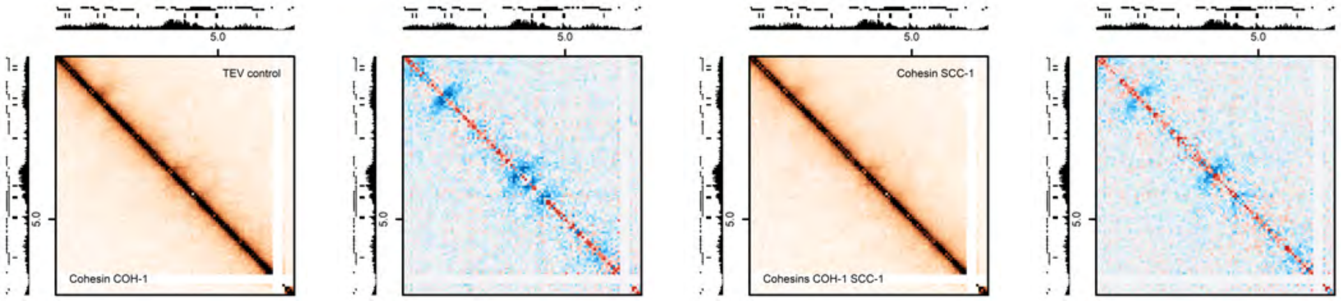

chrIII:8557001-8757000

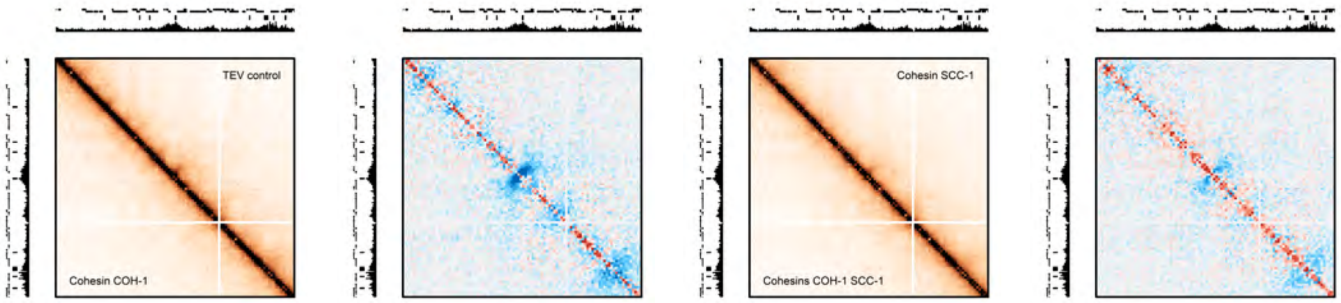

chrV:8987001-9187000

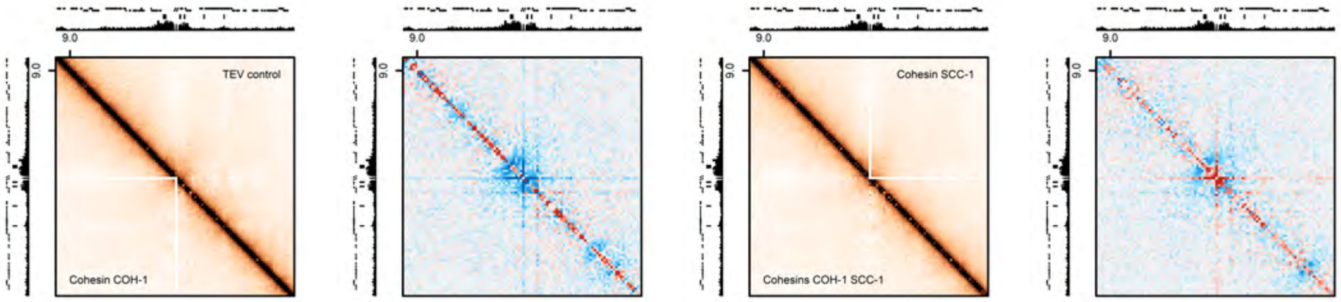

chrI:11477001-11677000

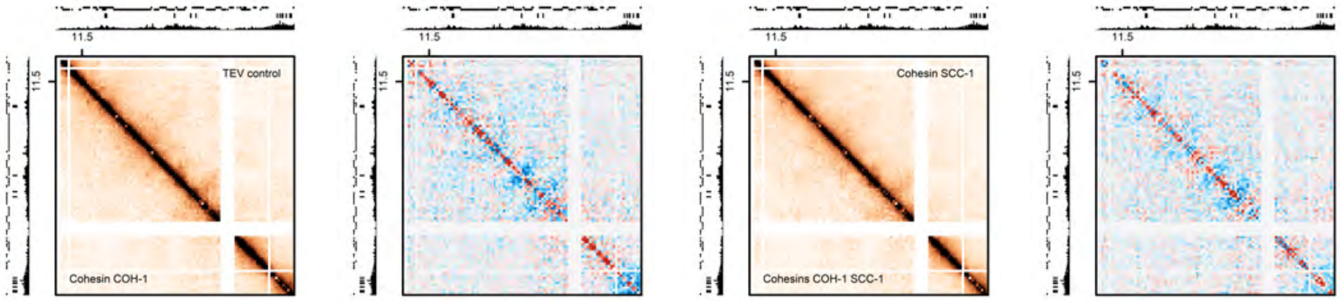

chrIV:8851001-9051000

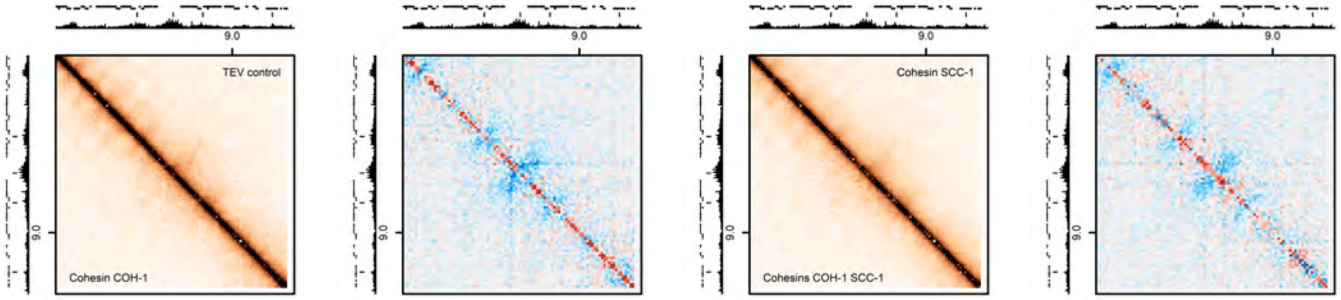

chrIII:9755001-9955000

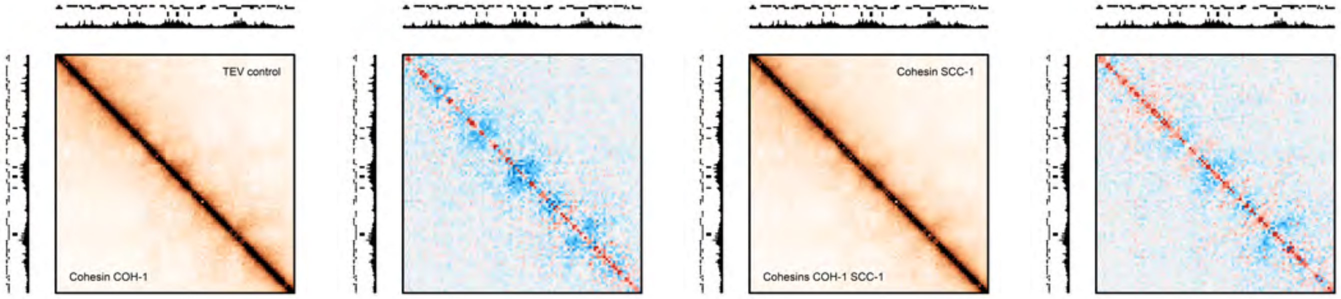

chrIII:8407001-8607000

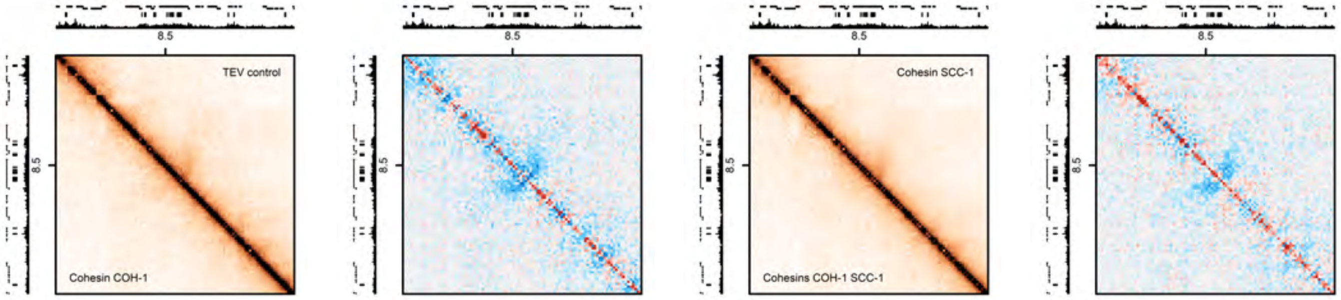

chrIII:8953001-9153000

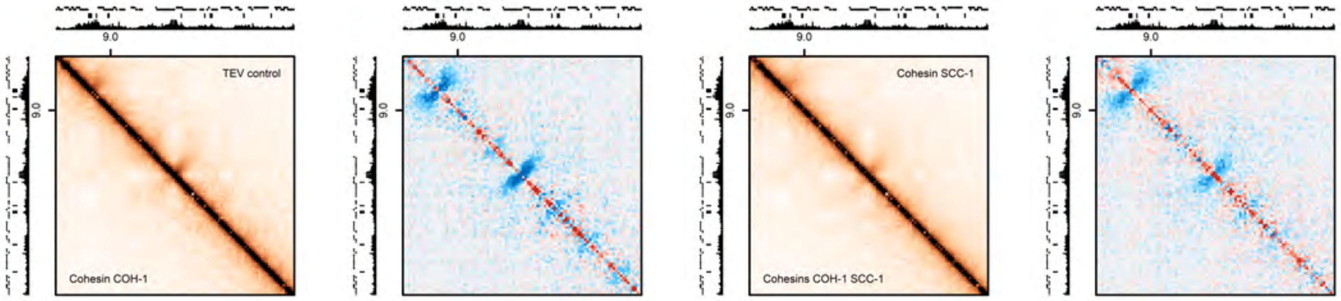

chrIV:16299001-16499000

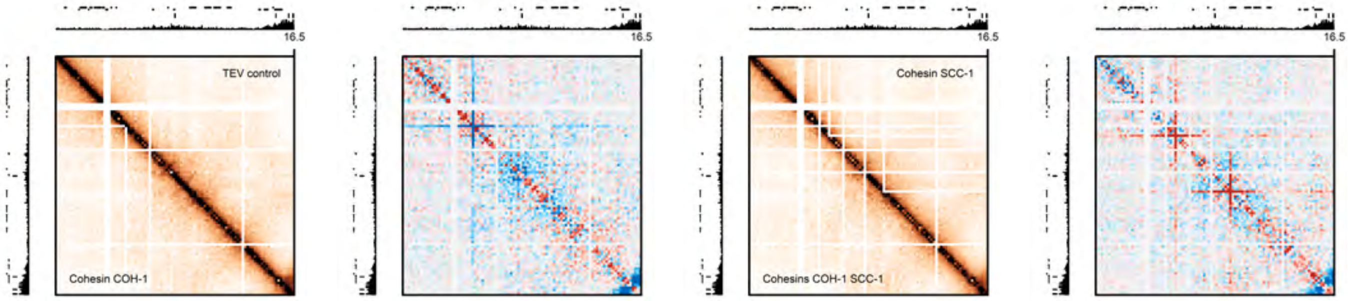

chrIII:10953001-11153000

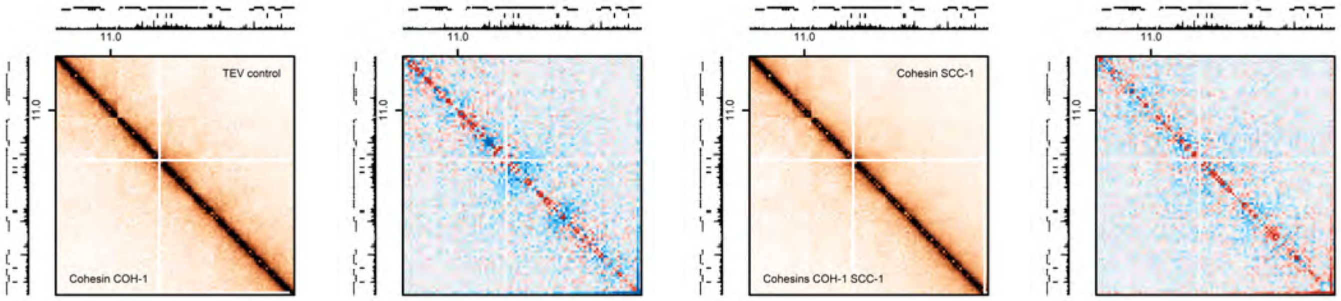

chrII:6515001-6715000

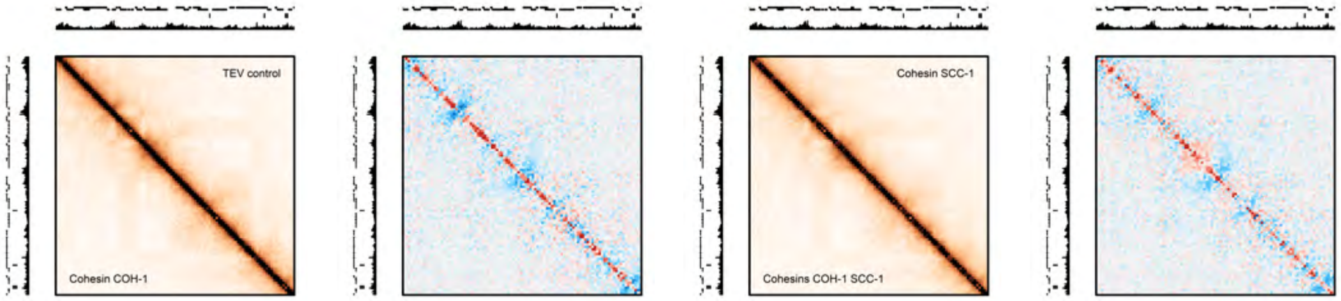

chrIII:4463001-4663000

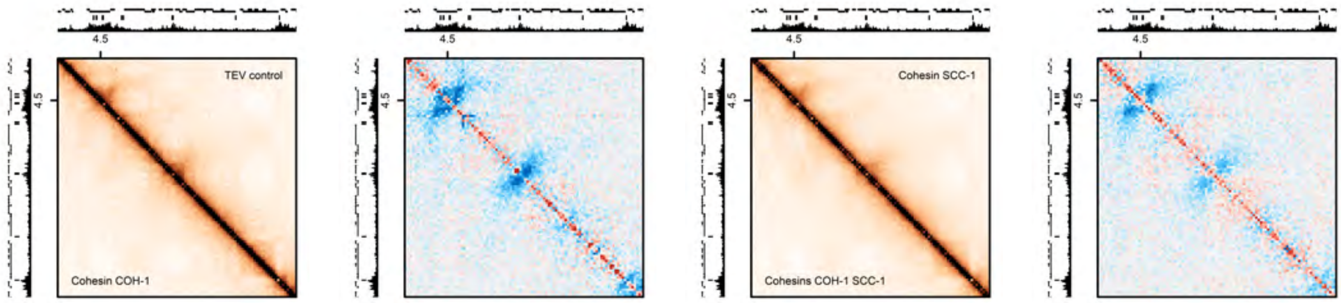

chrV:3293001-3493000

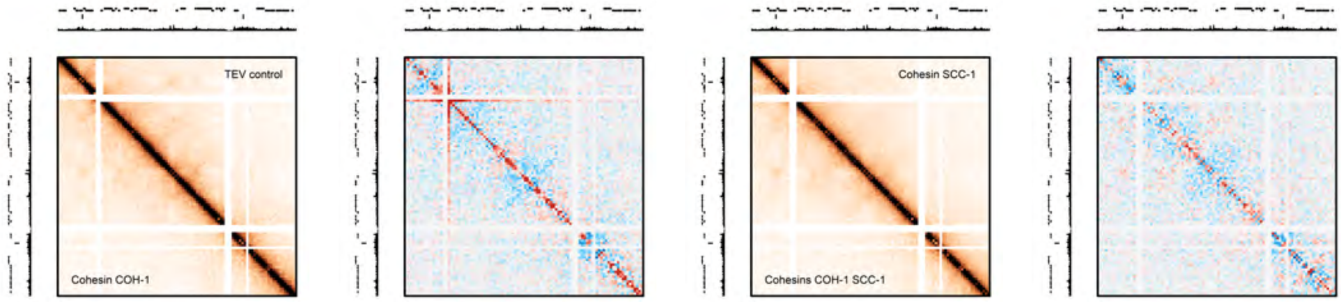

chrIII:2583001-2783000

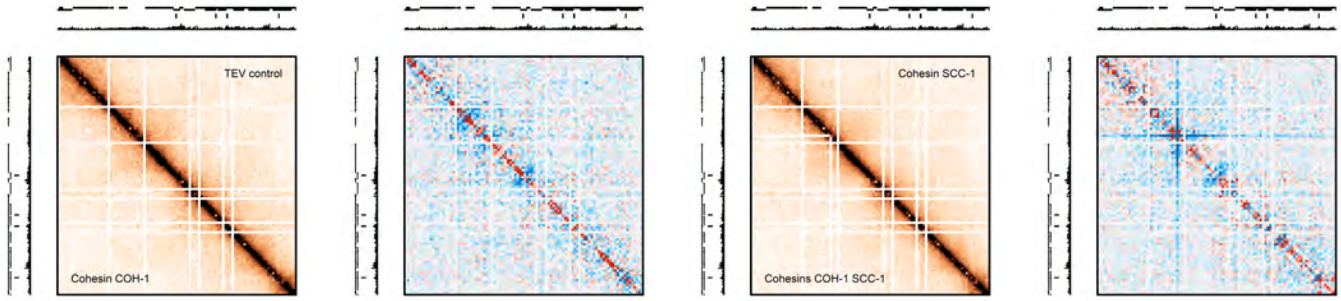

chrIV:2183001-2383000

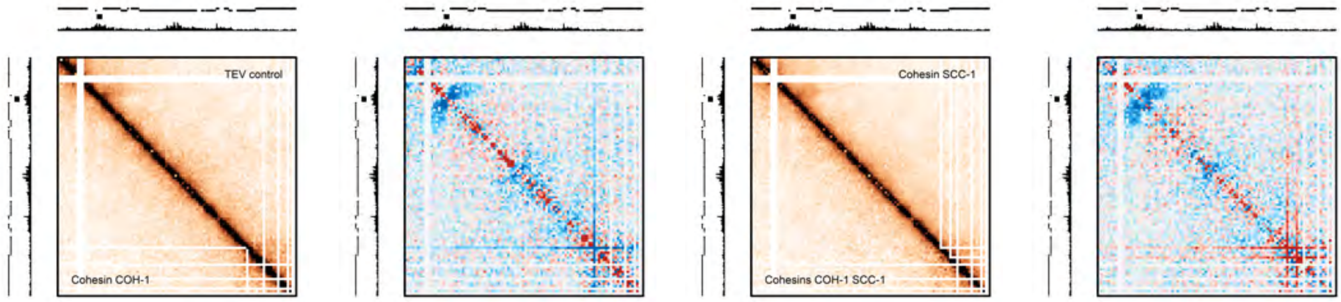

chrI:8687001-8887000

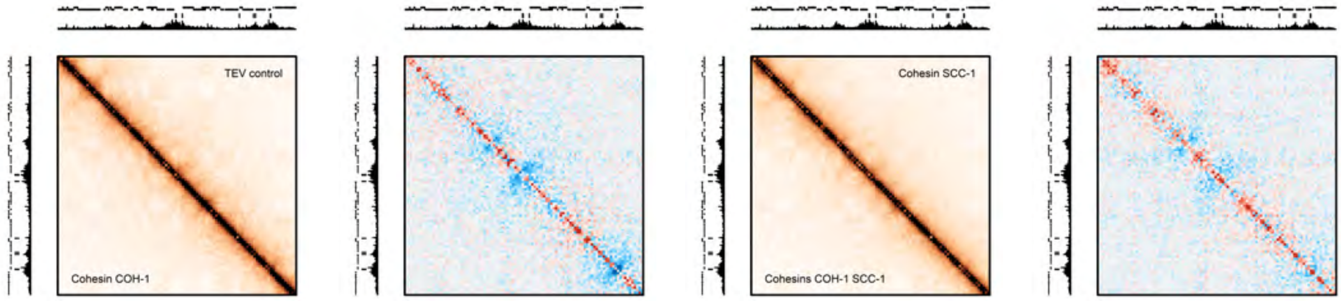

chrX:17533001-17733000

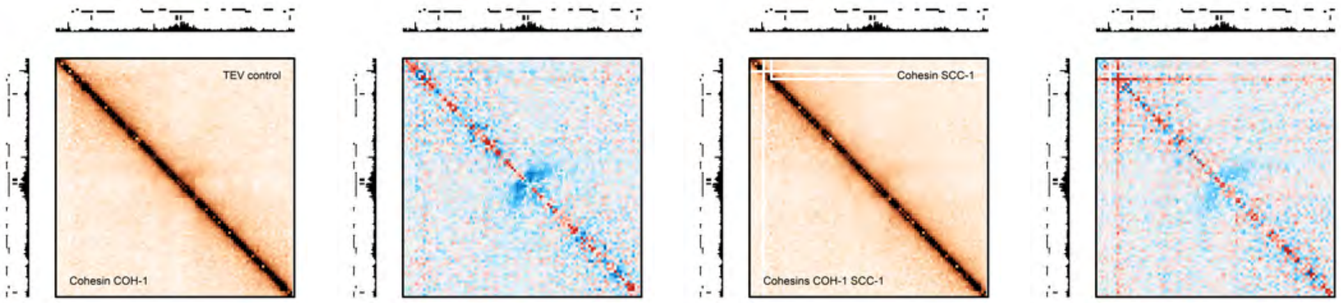

chrII:7917001-8117000

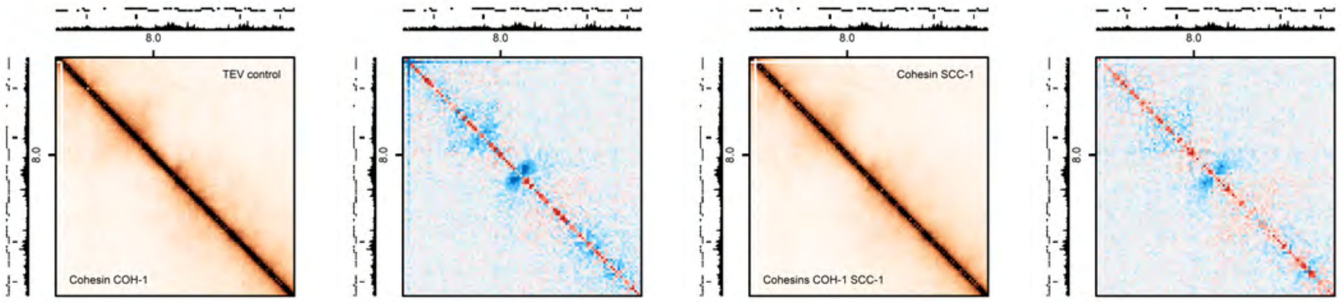

chrII:14305001-14505000

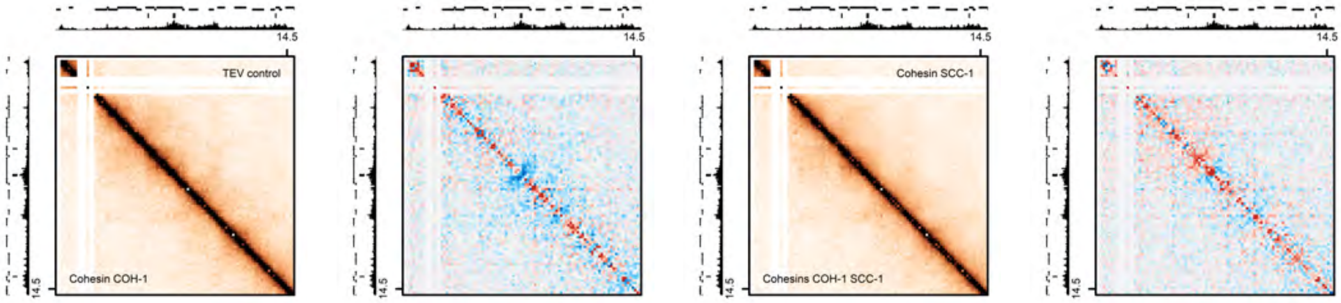

chrV:6251001-6451000

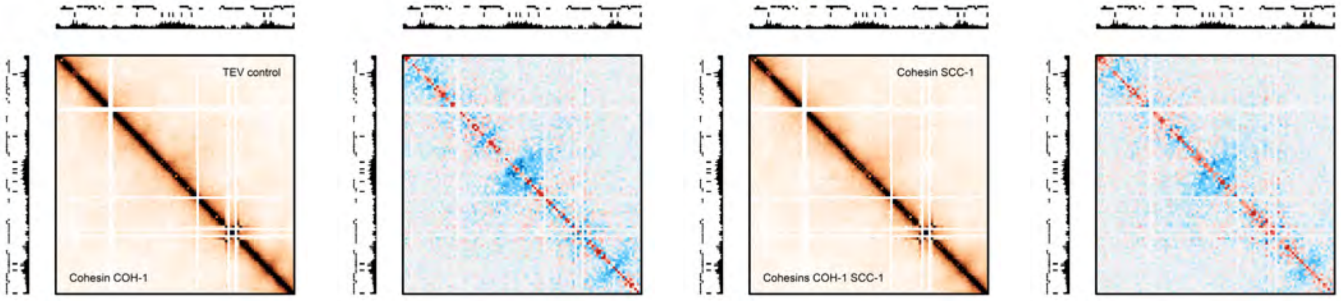

chrIV:15917001-16117000

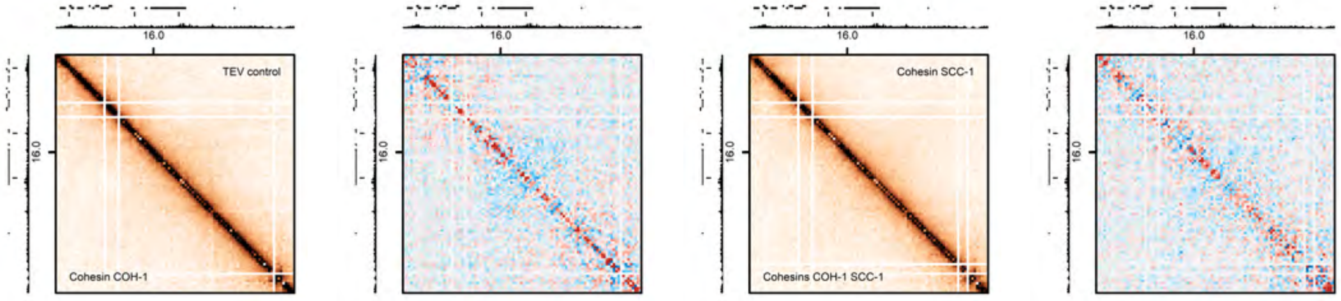

chrX:13849001-14049000

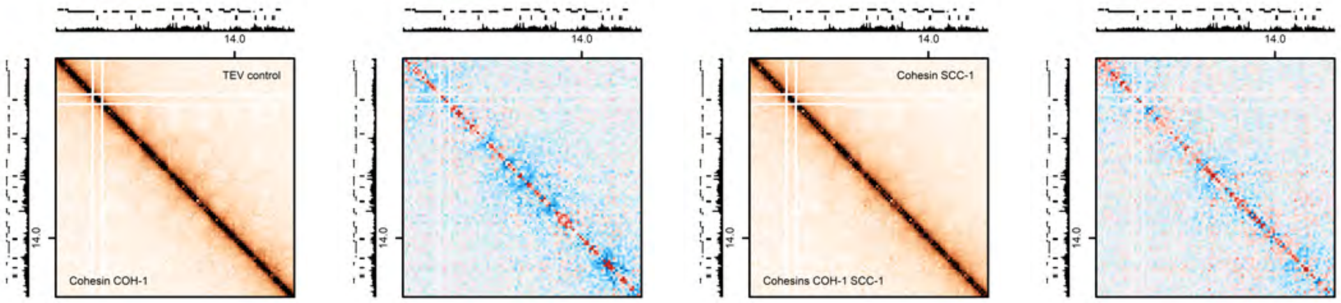

chrIII:5841001-6041000

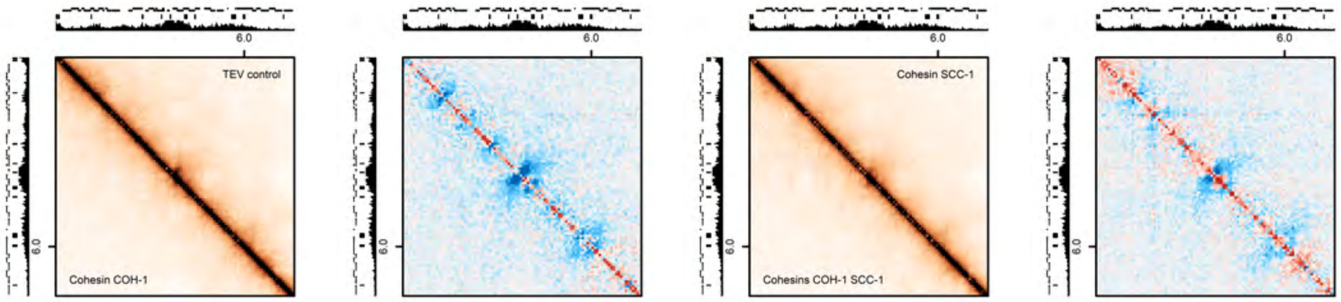

chrIV:7745001-7945000

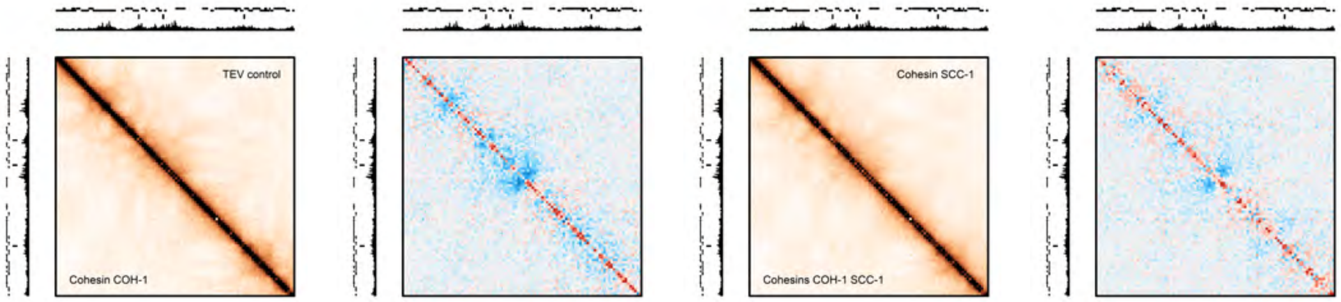

chrIV:201001-401000

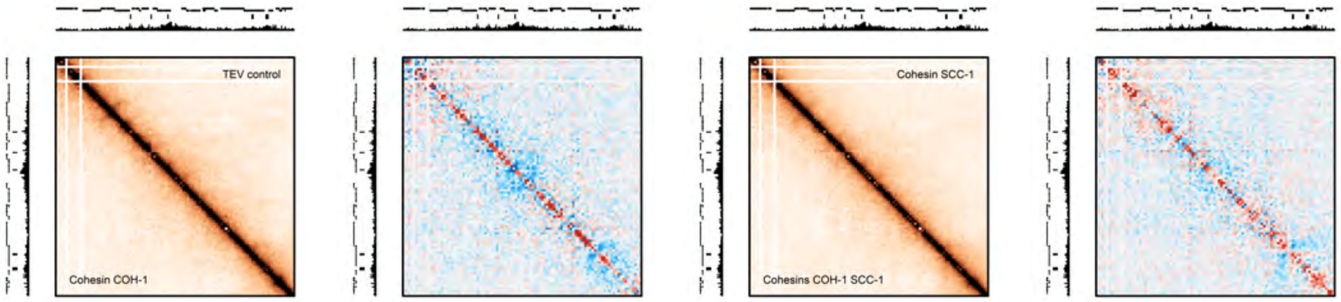

chrX:1355001-1555000

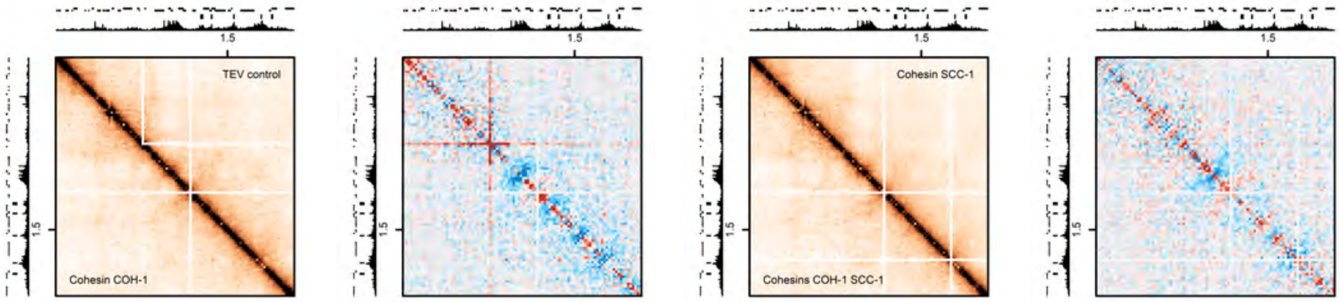

chrIII:8195001-8395000

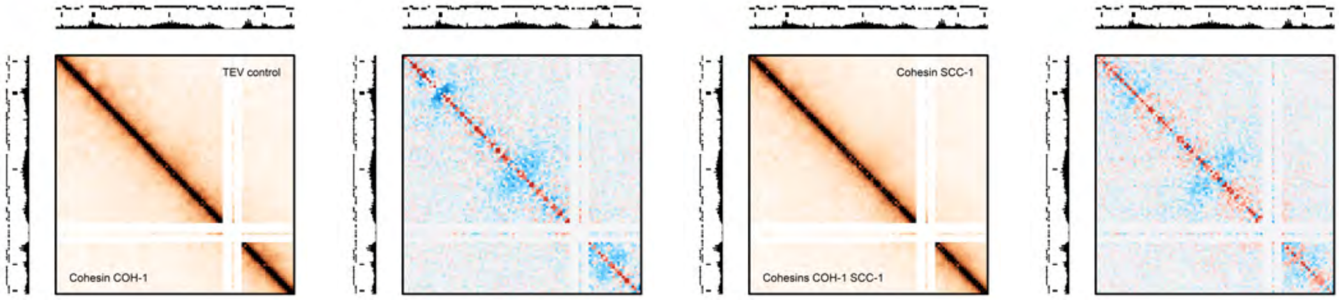

chrIII:12235001-12435000

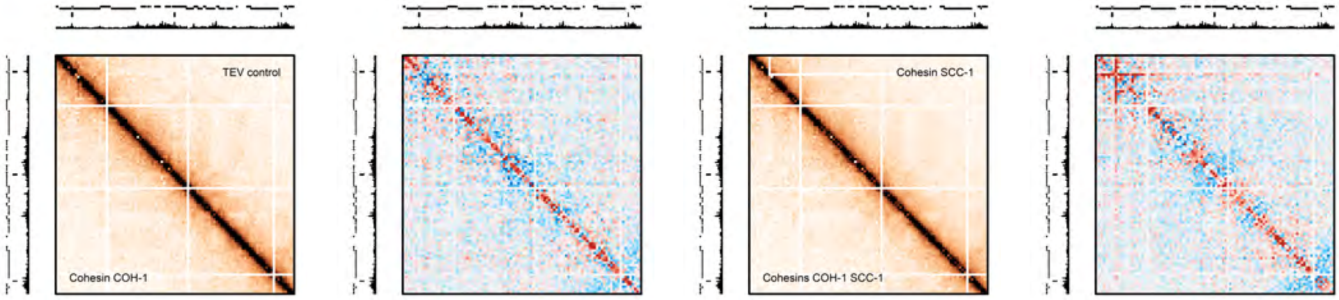

chrV:4961001-5161000

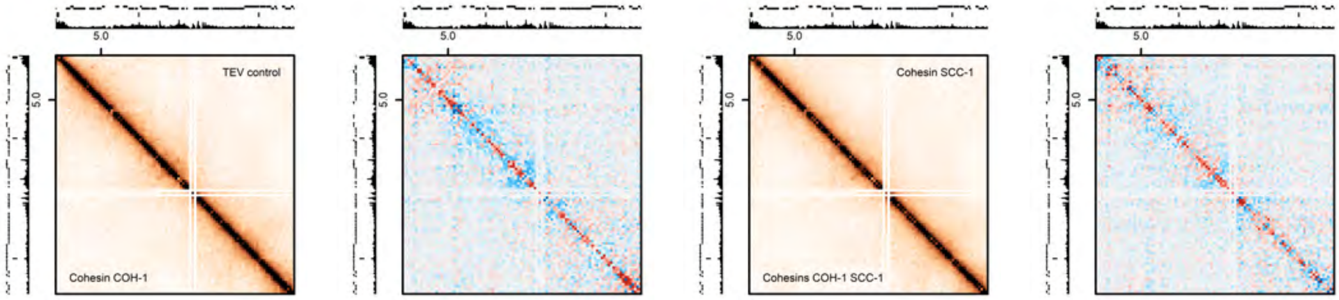

chrV:1373001-1573000

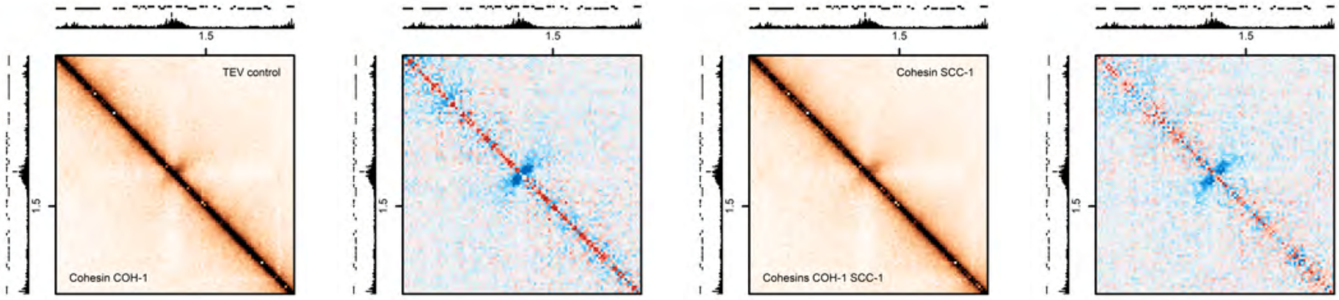

chrV:18477001-18677000

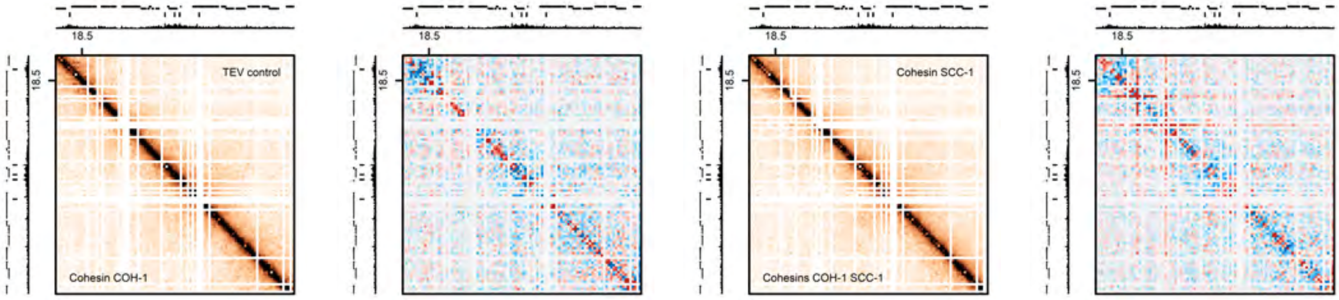

chrI:5591001-5791000

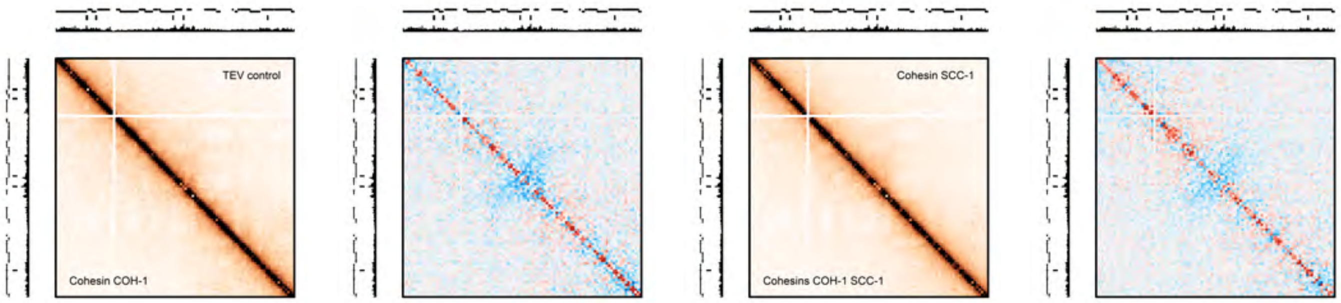

chrIV:8531001-8731000

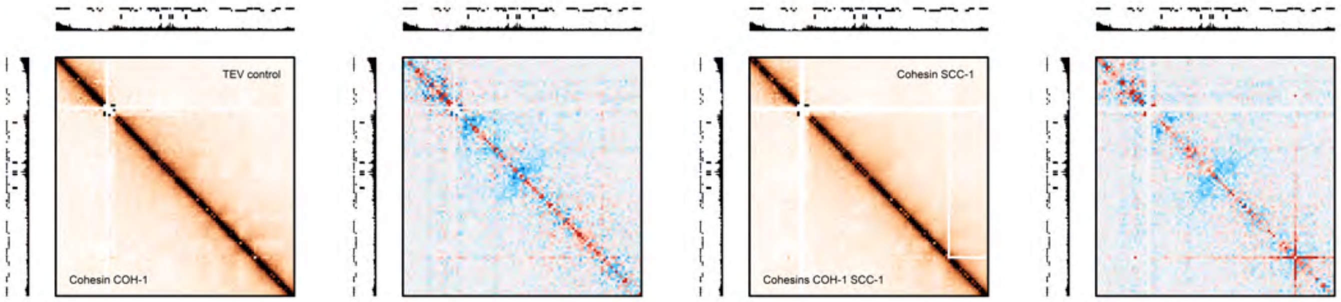

chrX:16051001-16251000

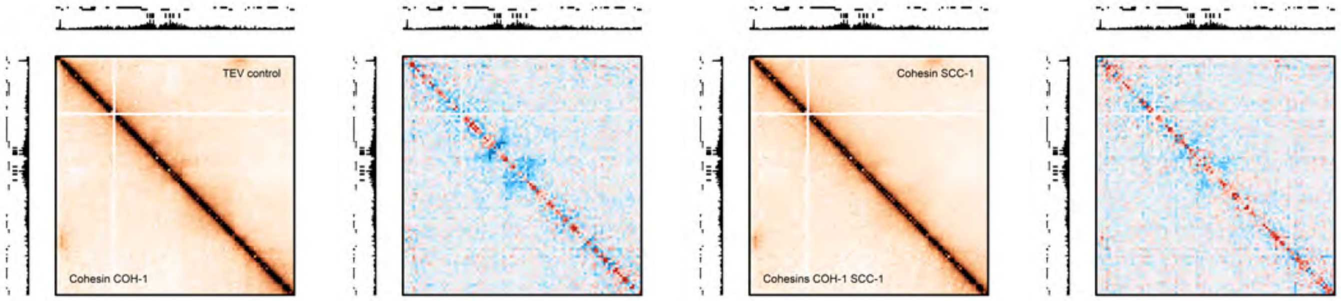

chrIV:8335001-8535000

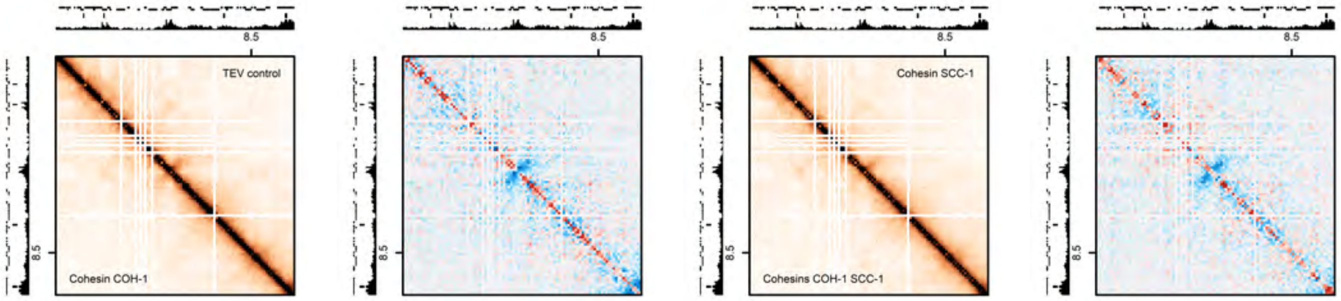

chrIII:13175001-13375000

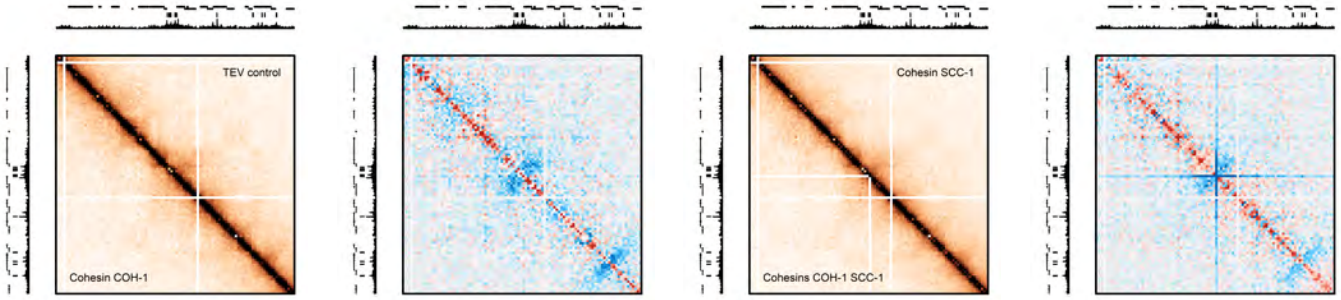

chrIV:9775001-9975000

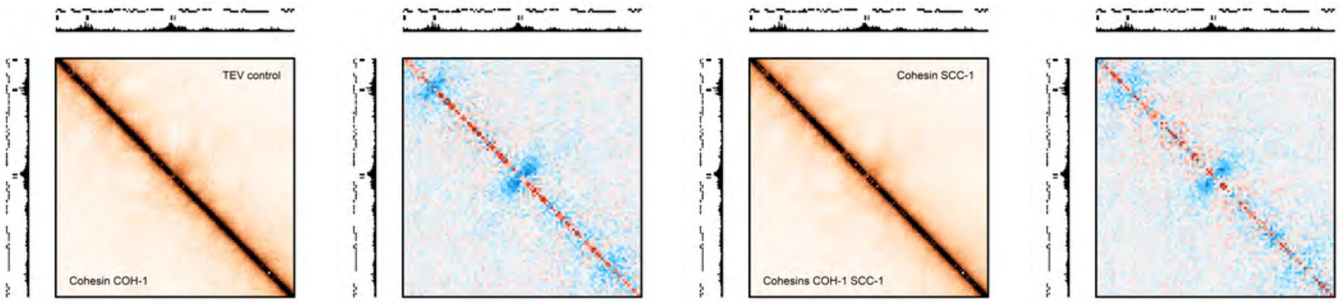

chrV:14973001-15173000

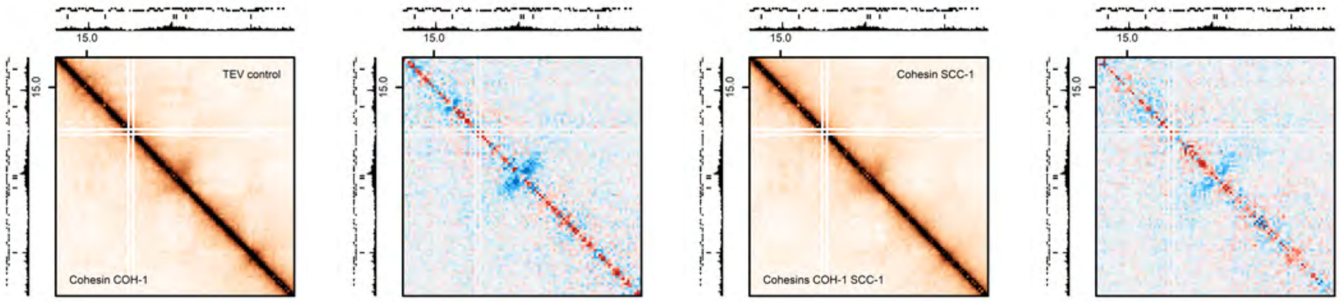

chrII:9389001-9589000

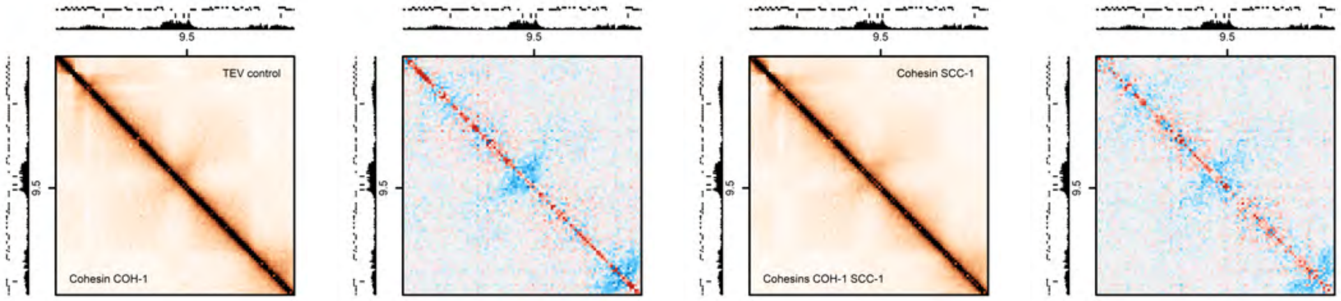

chrII:12565001-12765000

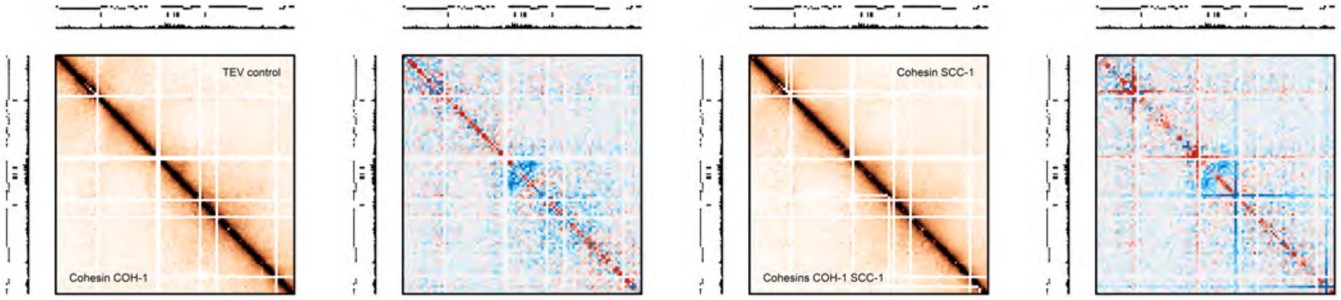

chrI:4215001-4415000

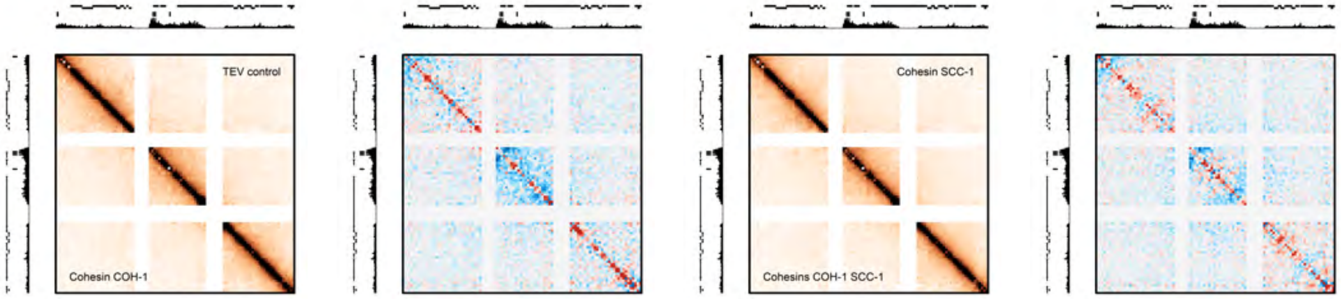

chrIV:4303001-4503000

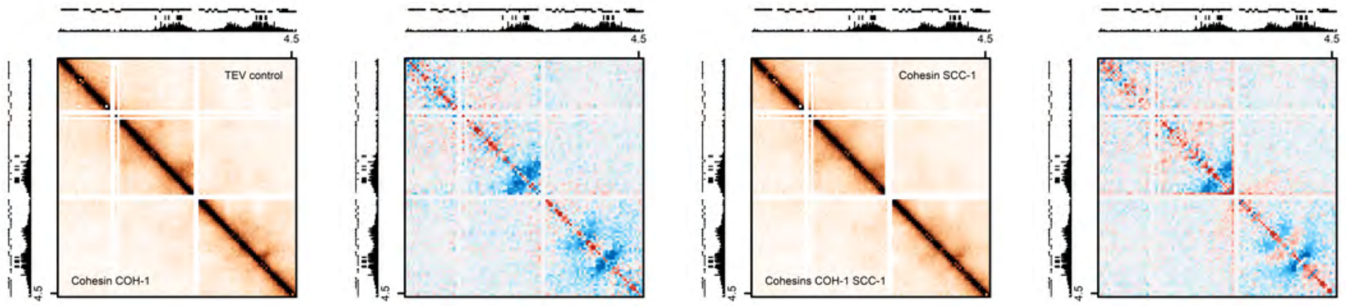

chrI:8765001-8965000

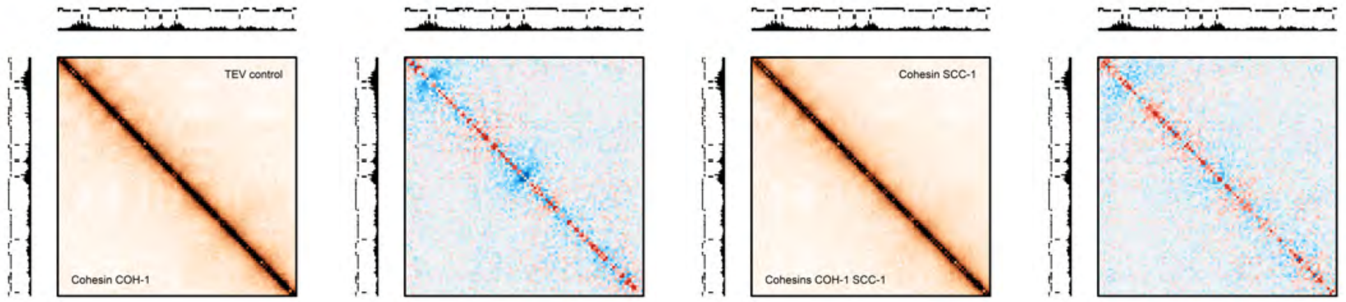

chrIV:12319001-12519000

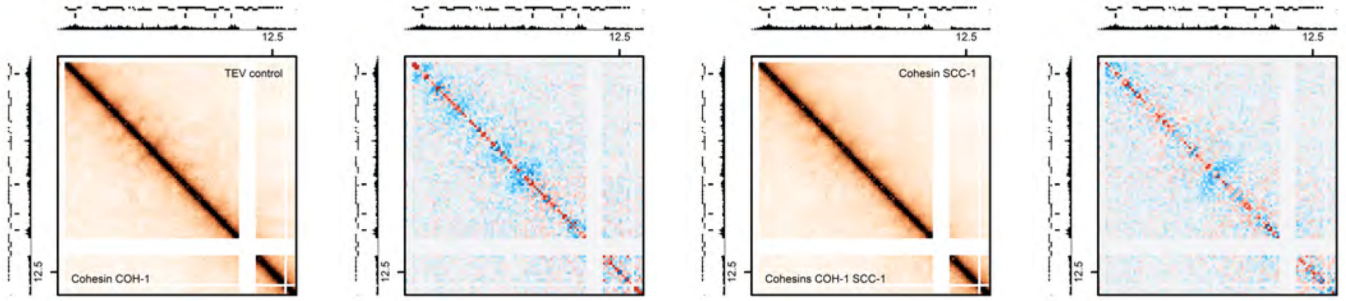

chrI:199001-399000

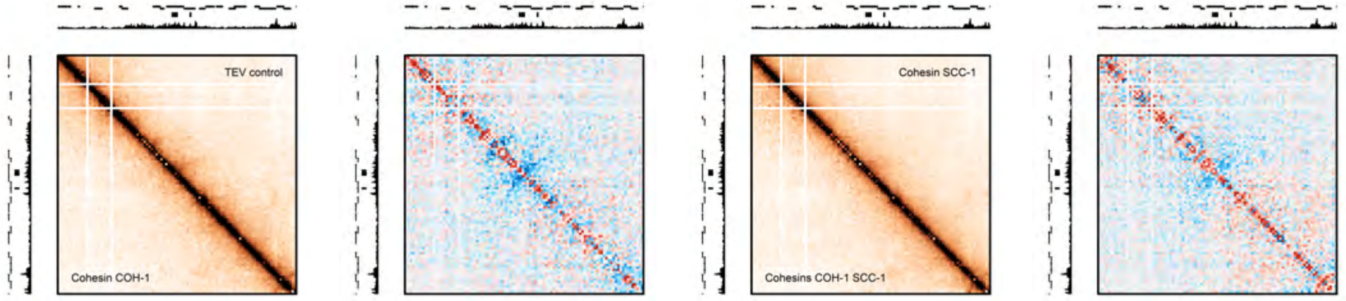

chrX:11143001-11343000

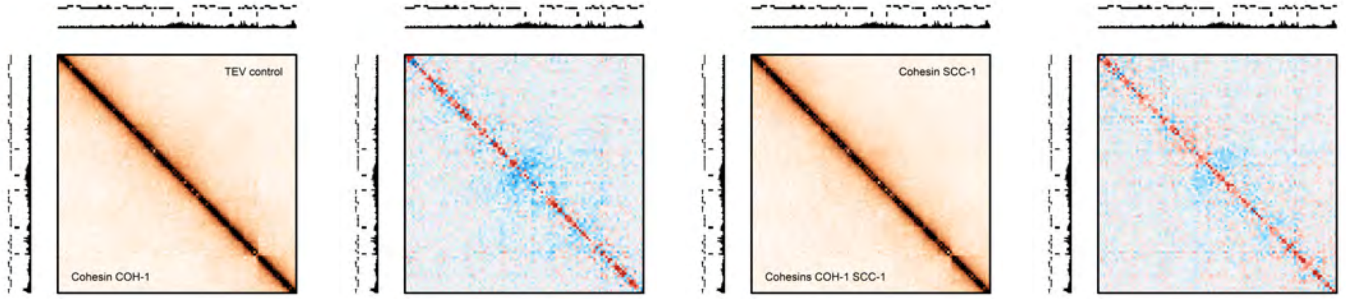

chrII:3169001-3369000

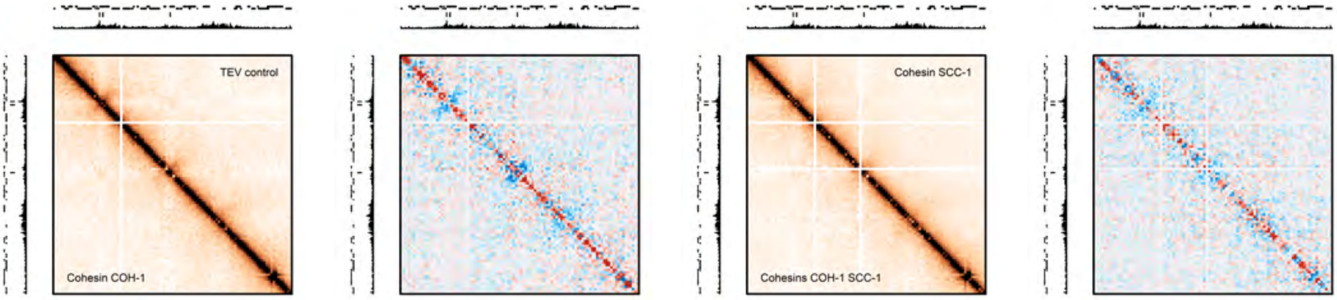

chrII:13571001-13771000

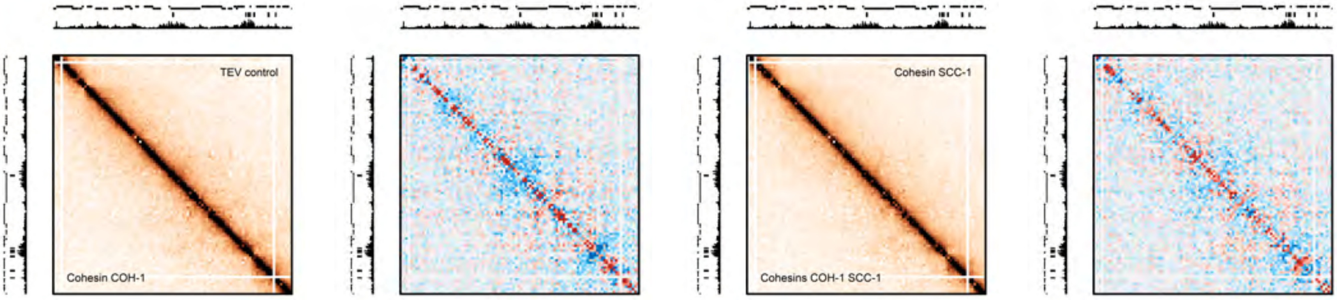

chrV:6561001-6761000

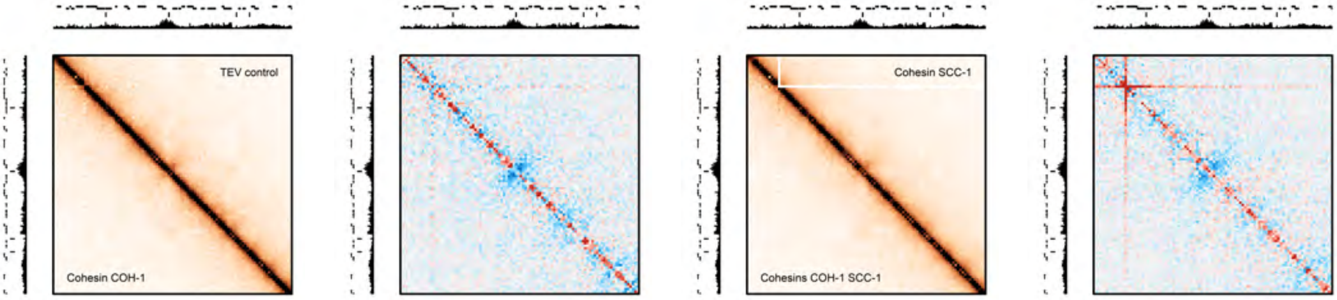

chrIV:15299001-15499000

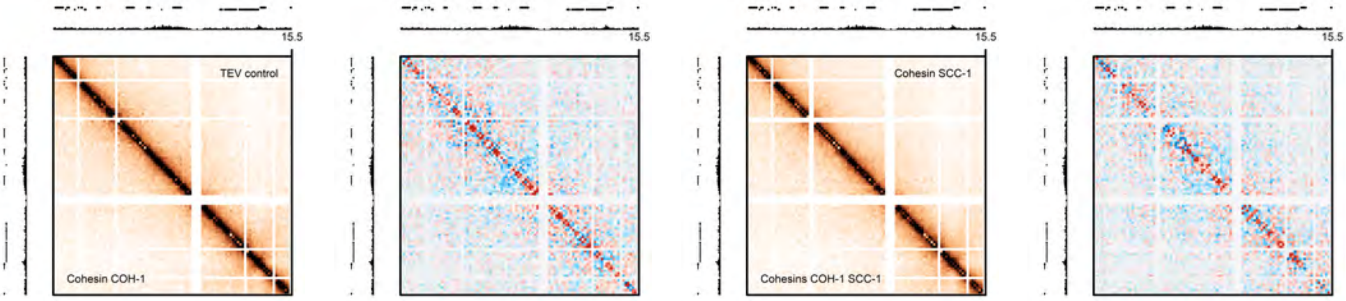

chrII:5603001-5803000

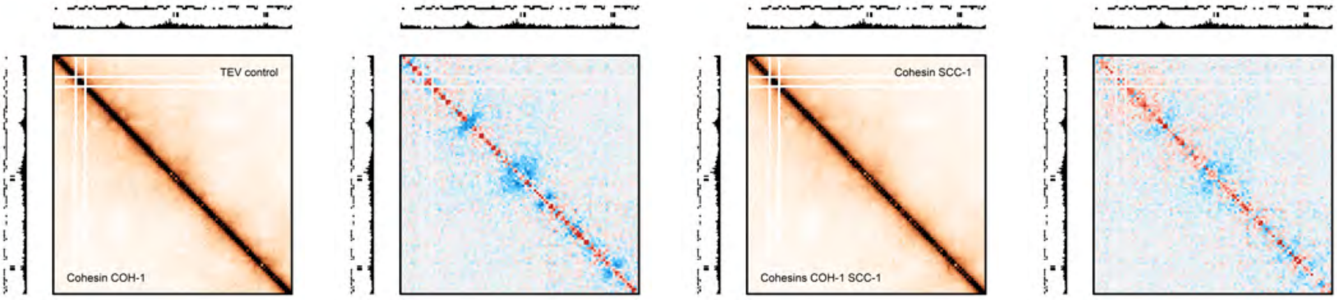

chrIII:11917001-12117000

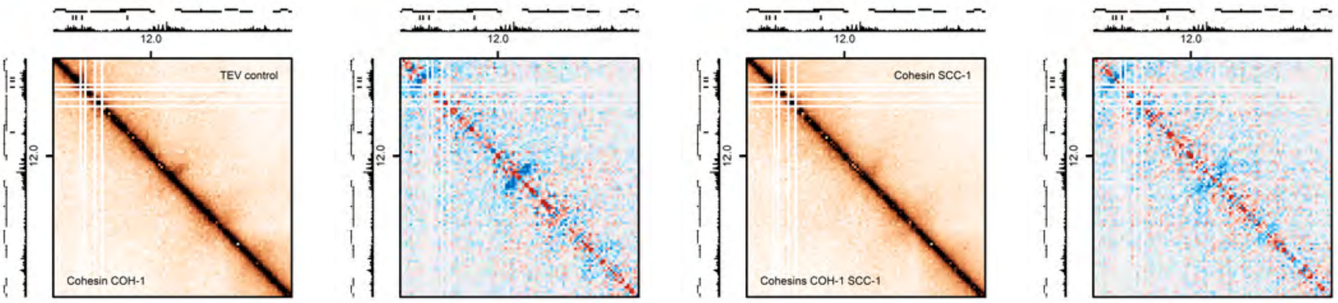

chrIV:1355001-1555000

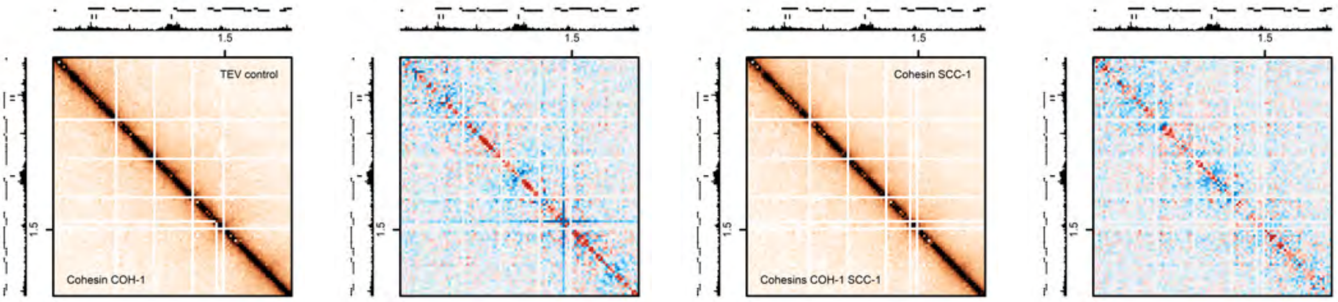

chrIV:10153001-10353000

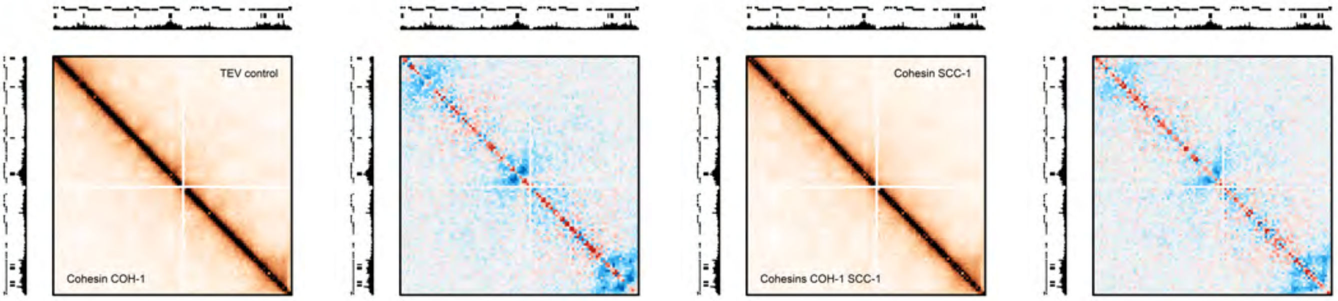

chrX:7715001-7915000

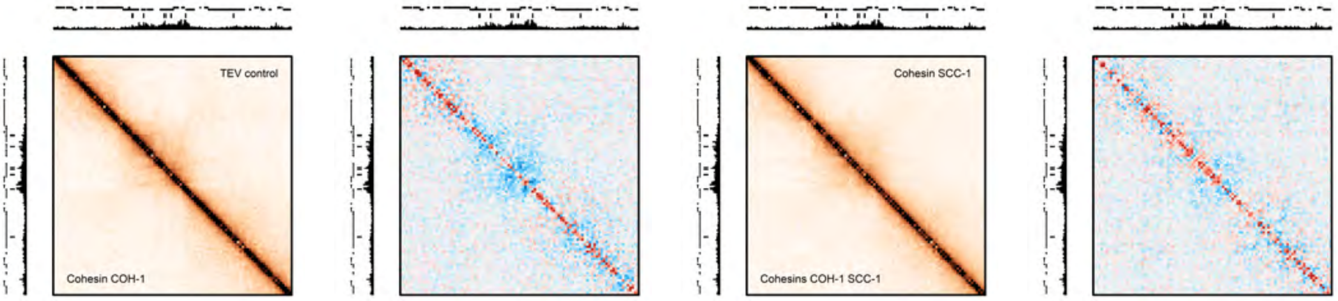

chrIV:16999001-17199000

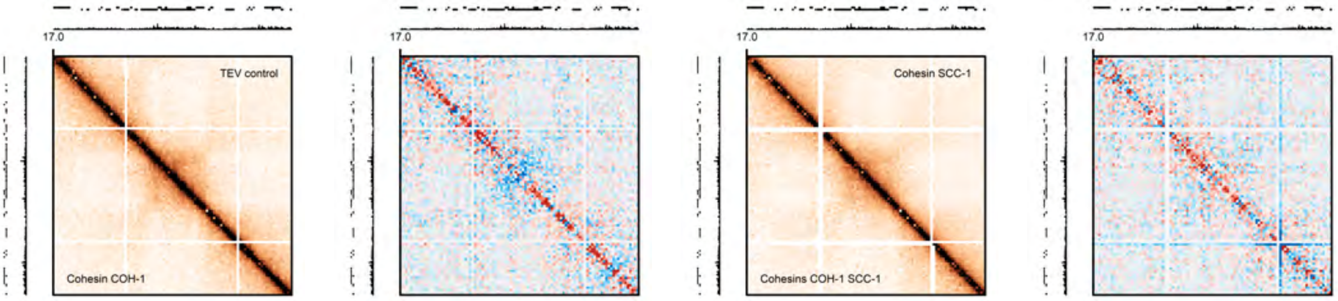

chrI:8031001-8231000

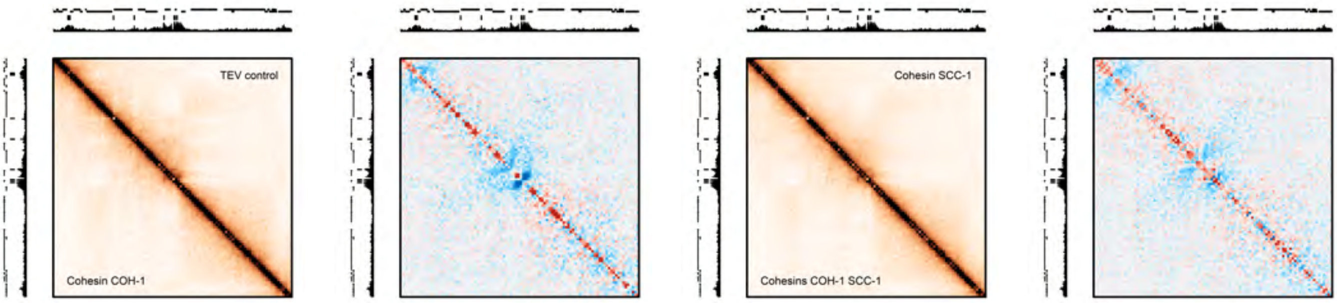

chrII:2095001-2295000

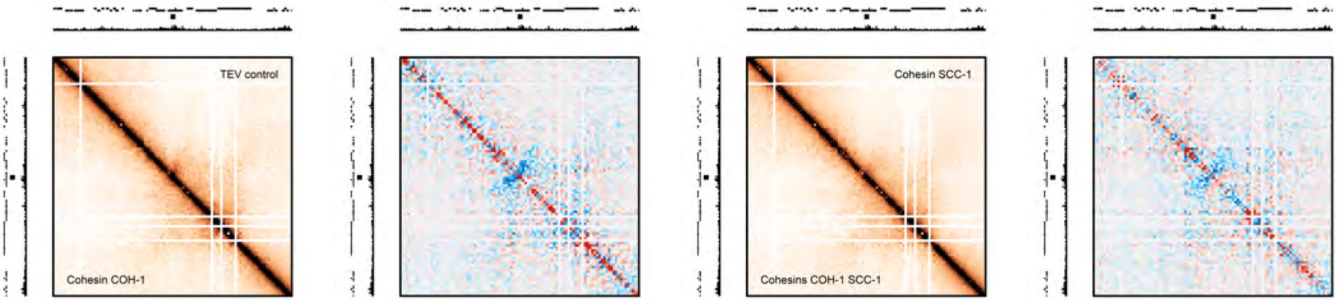

chrIII:10617001-10817000

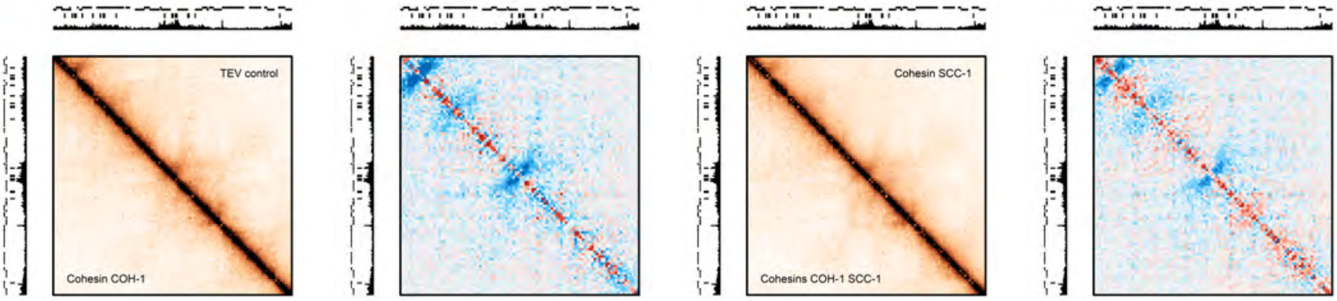

chrI:5899001-6099000

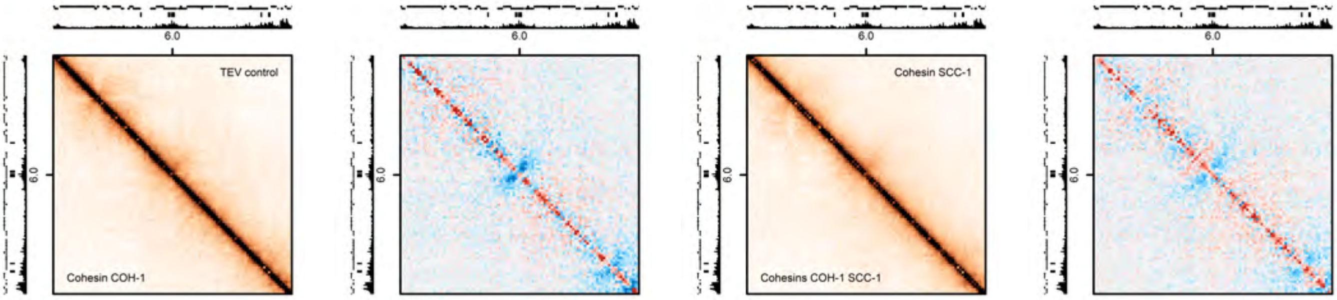

chrIII:3777001-3977000

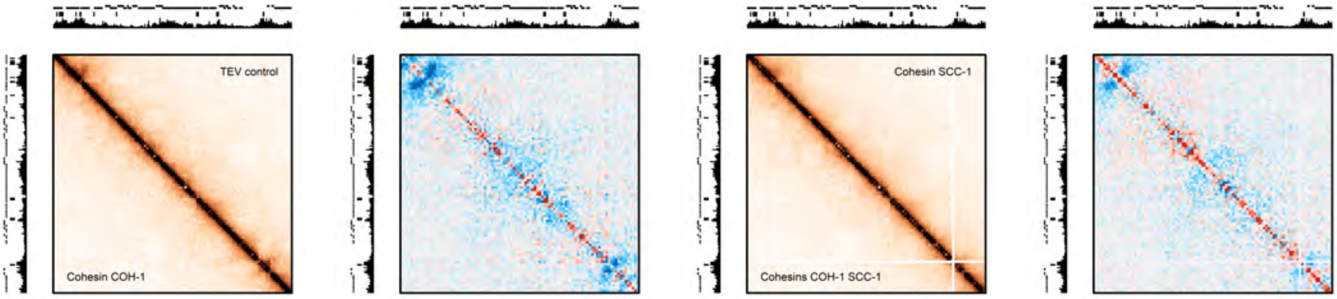

chrIV:3717001-3917000

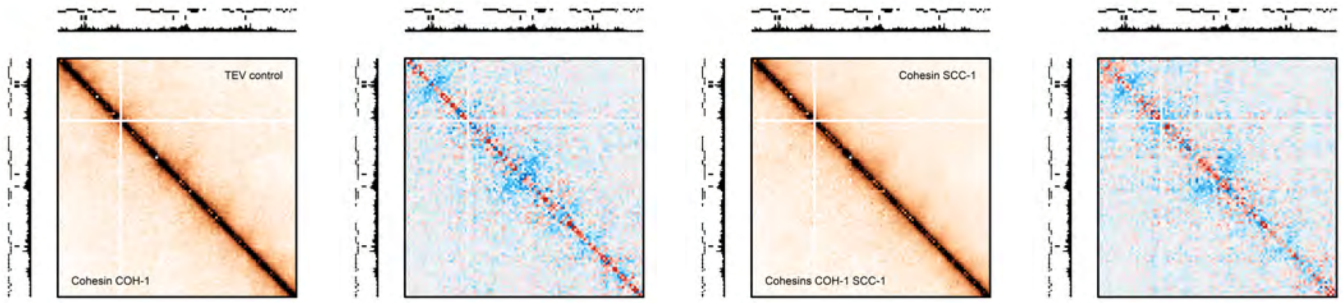

chrI:6245001-6445000

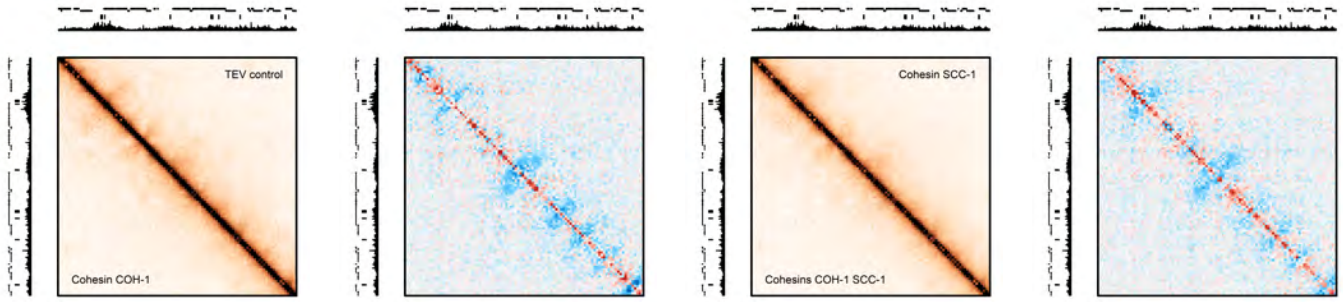

chrIV:1949001-2149000

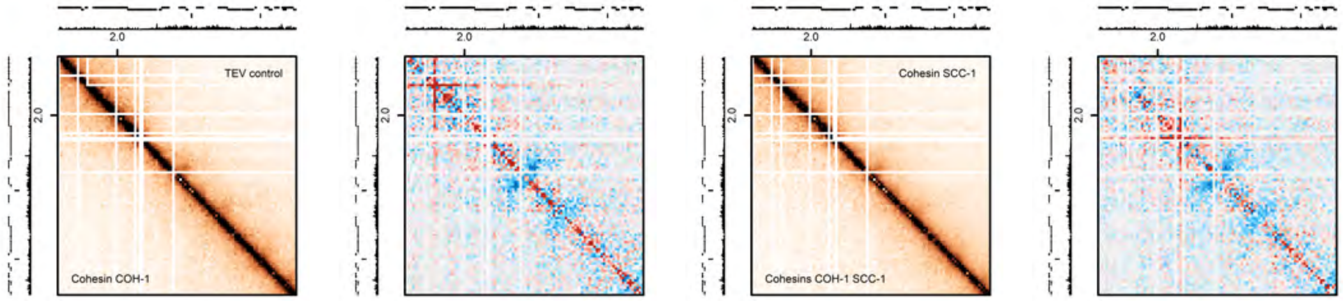

chrIV:11663001-11863000

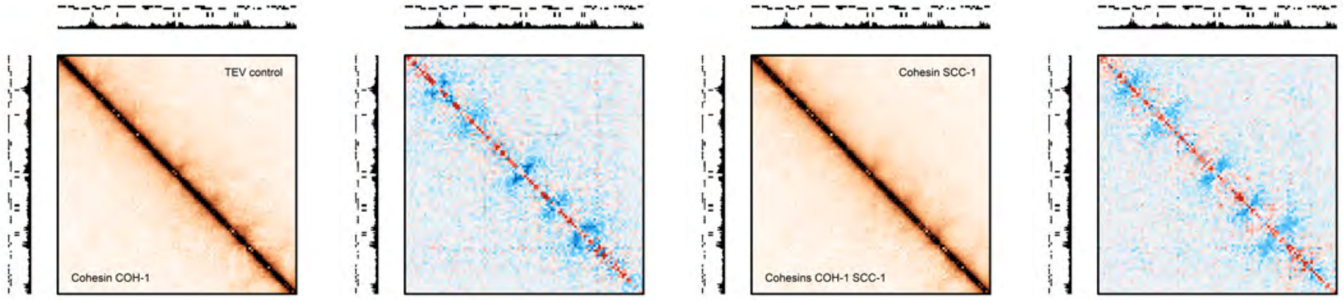

chrII:12251001-12451000

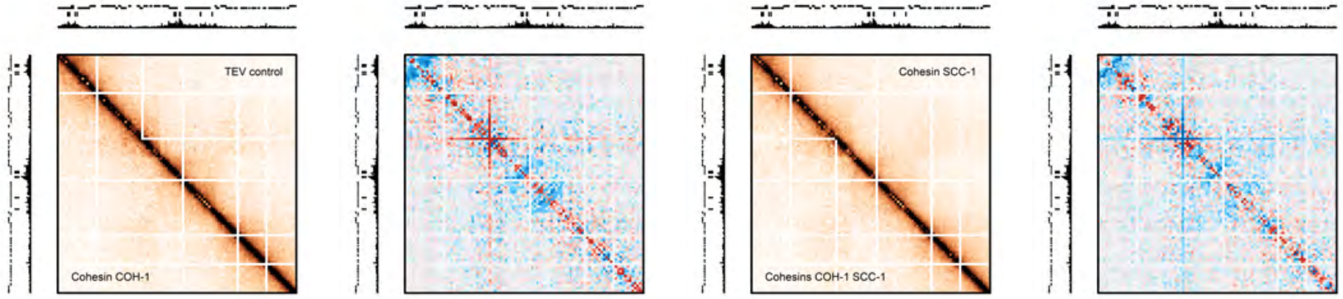

chrI:337001-537000

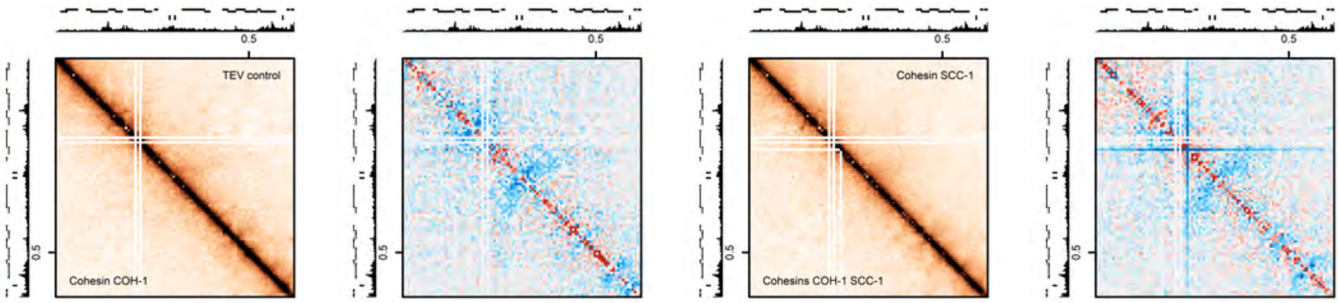

chrI:11773001-11973000

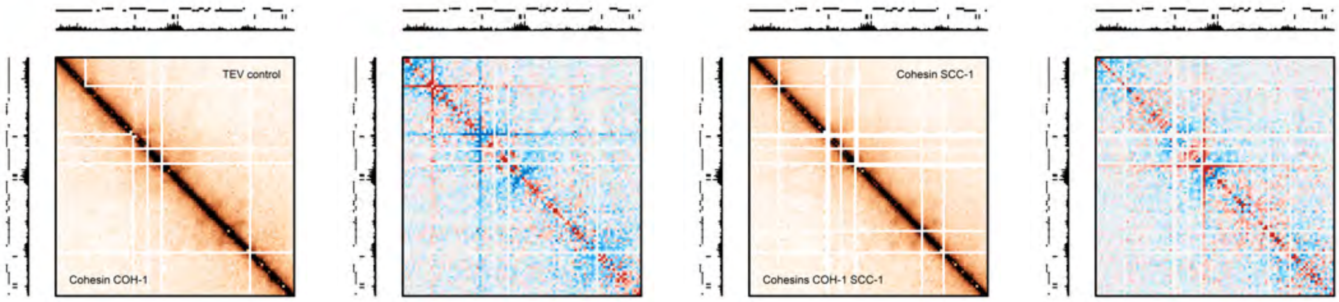

chrIII:11639001-11839000

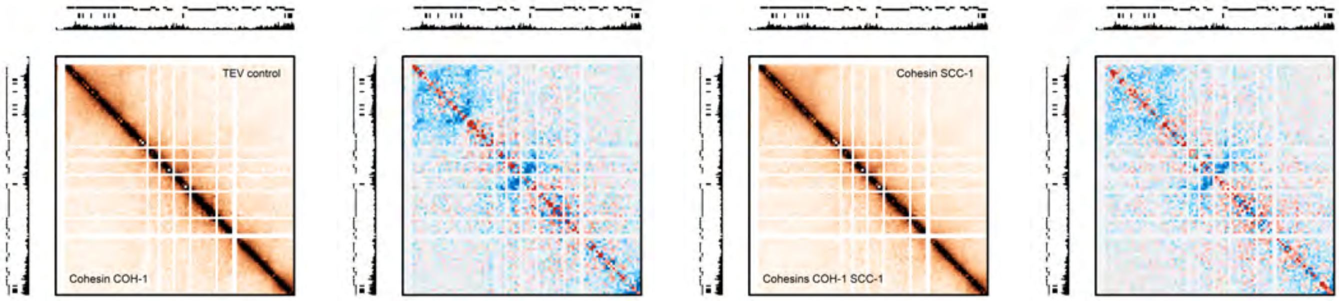

chrI:7915001-8115000

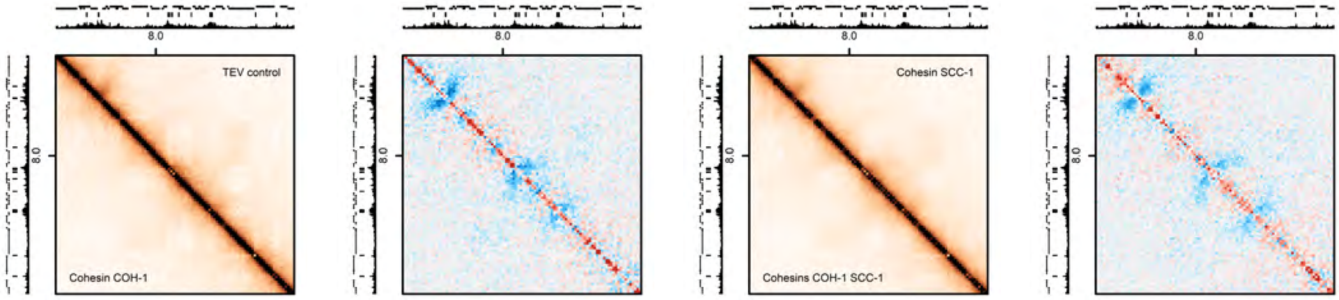

chrX:5379001-5579000

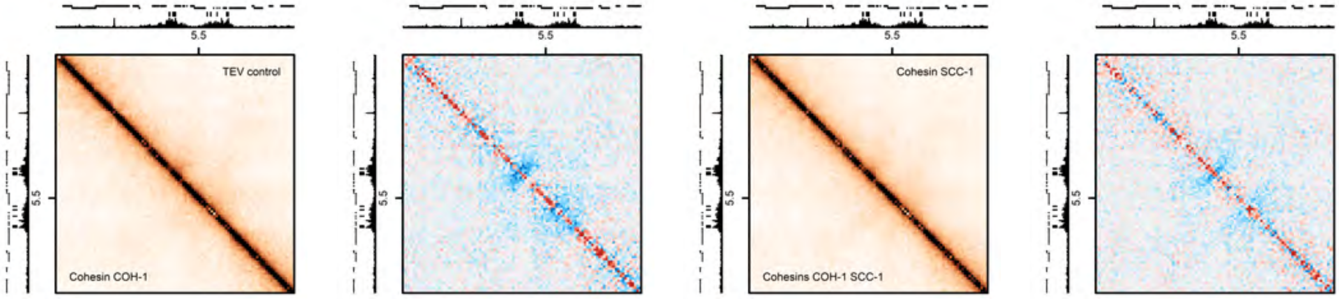

chrII:4477001-4677000

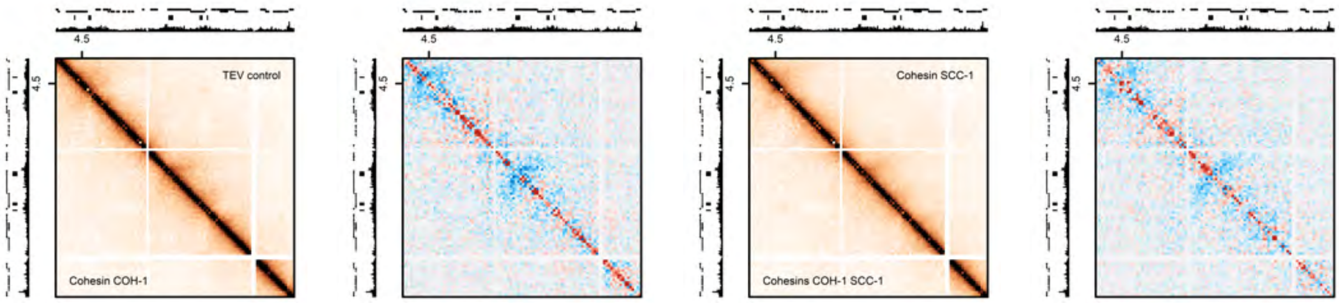

chrI:8311001-8511000

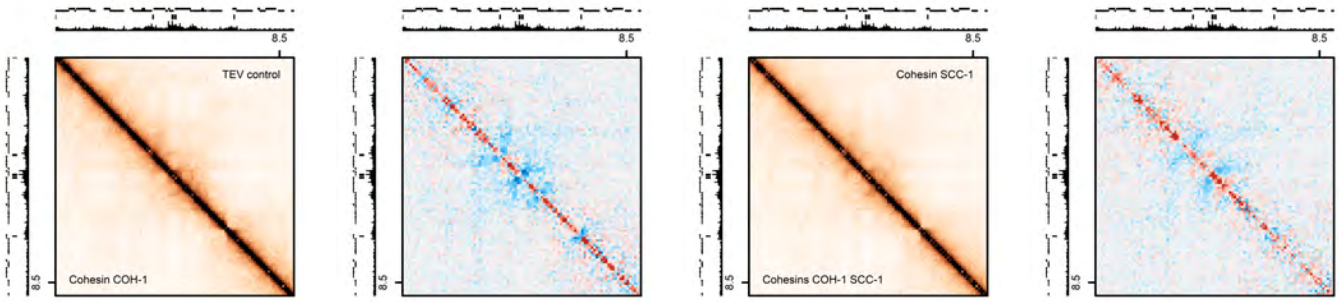

chrIV:10833001-11033000

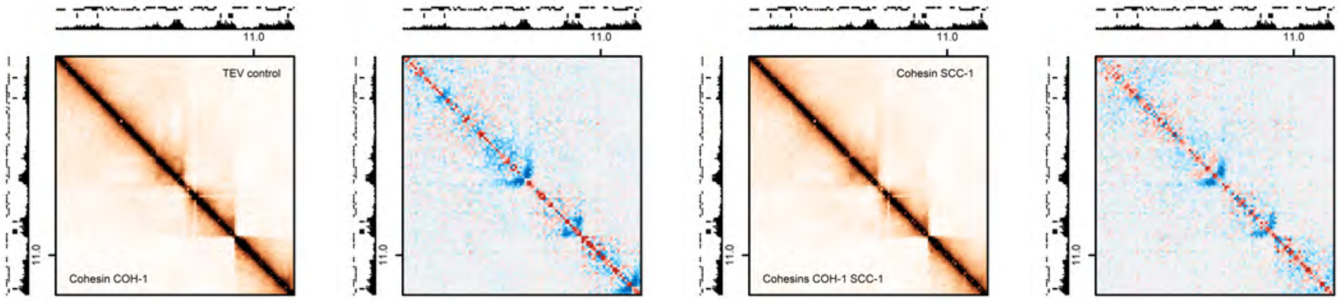

chrIV:16563001-16763000

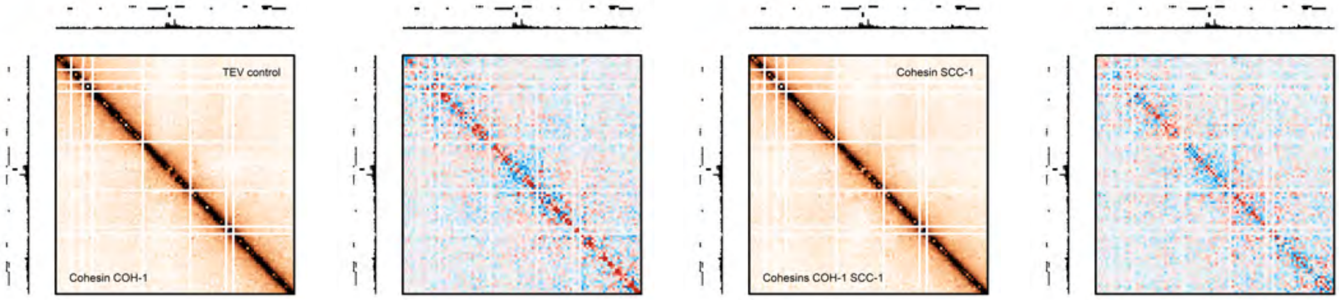

chrIII:13429001-13629000

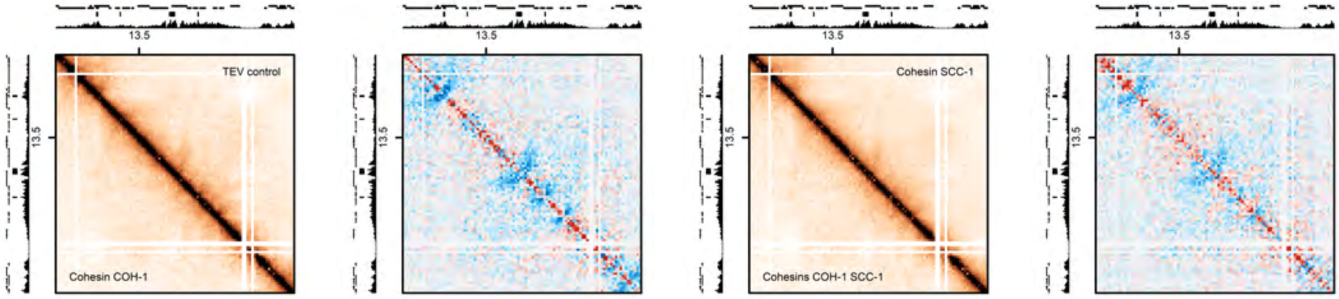

chrIII:3207001-3407000

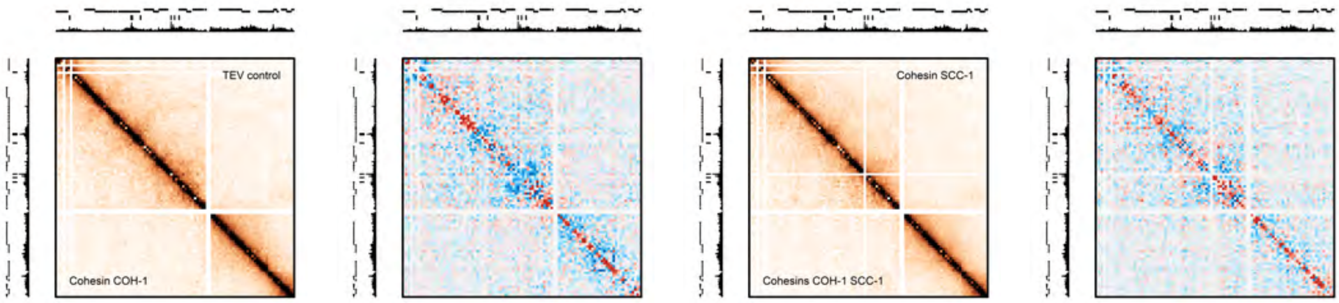

chrIII:6765001-6965000

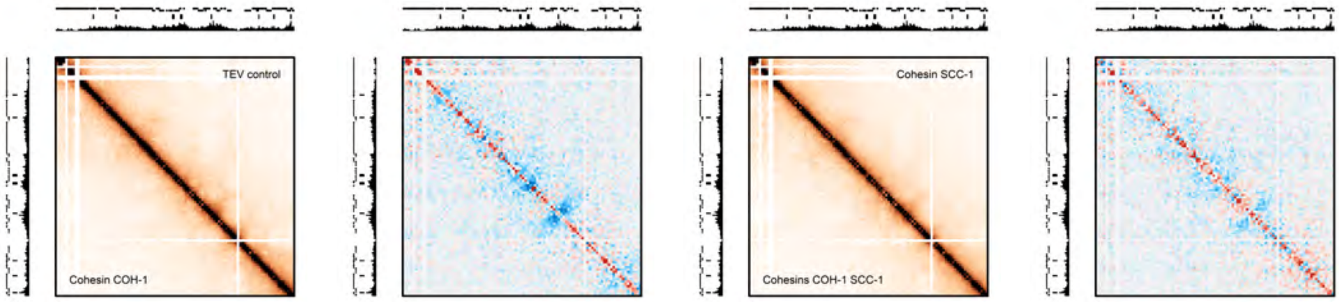

chrIII:2069001-2269000

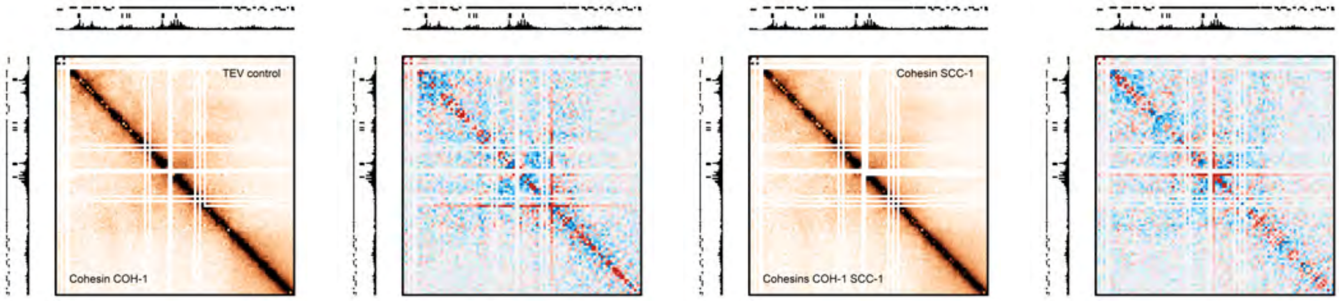

chrII:15161001-15361000

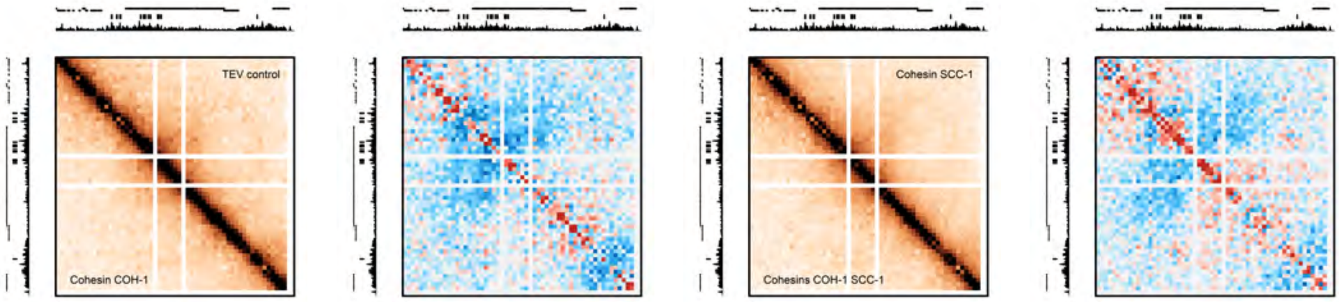

chrI:6785001-6985000

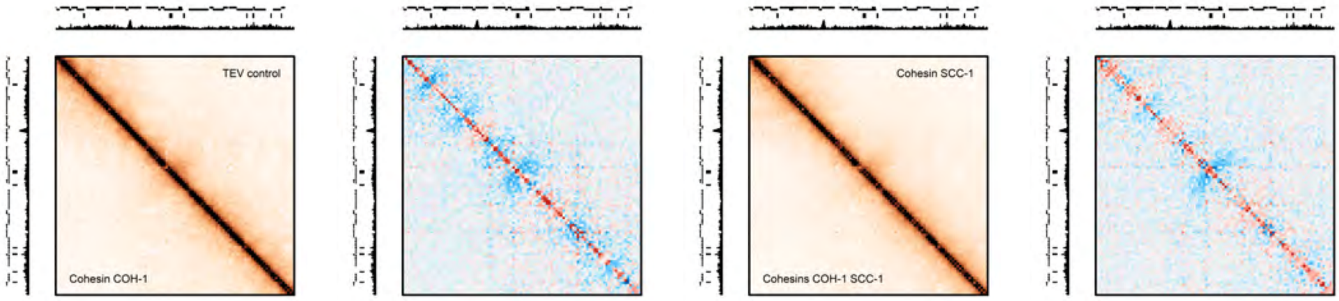

chrIV:6235001-6435000

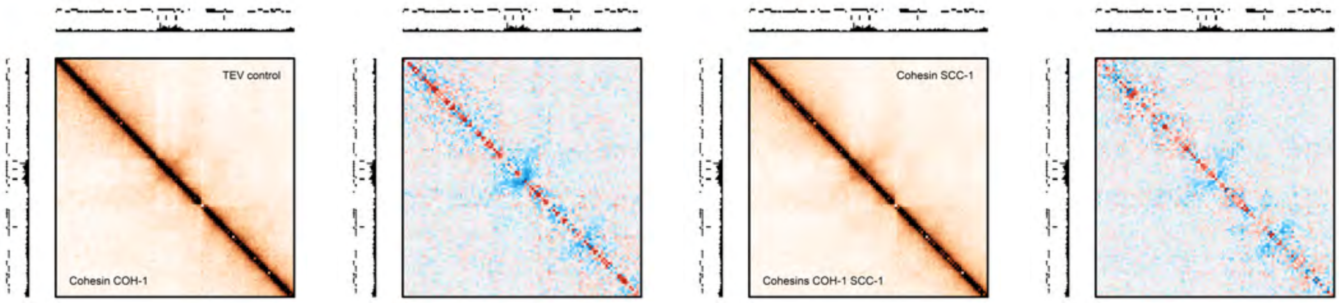

chrX:3845001-4045000

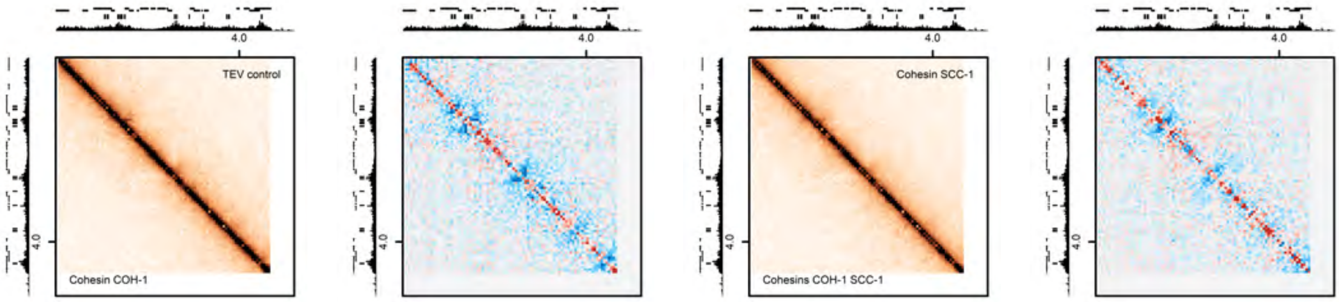

chrV:14099001-14299000

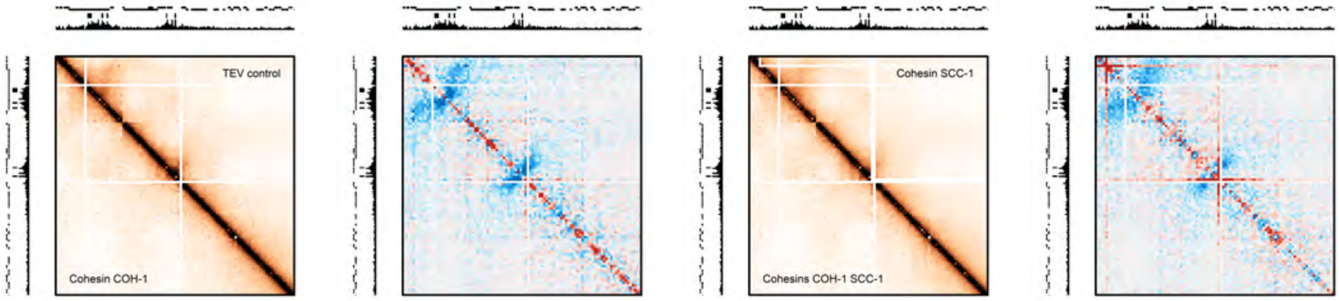

chrX:10331001-10531000

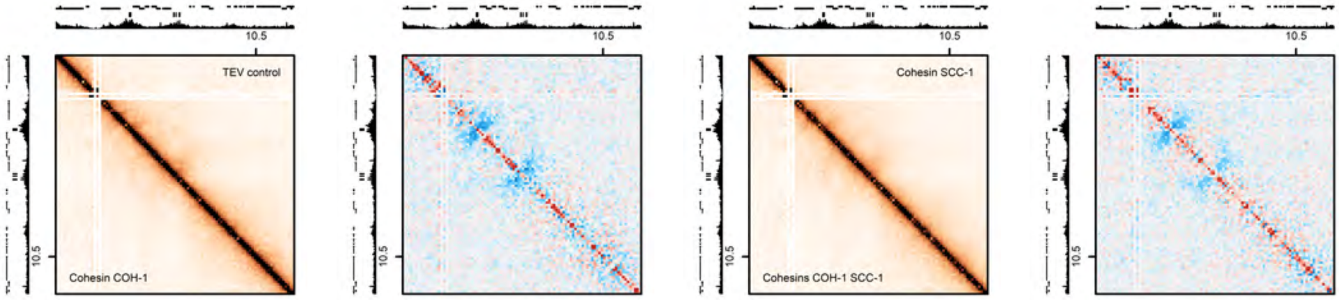

chrIII:10053001-10253000

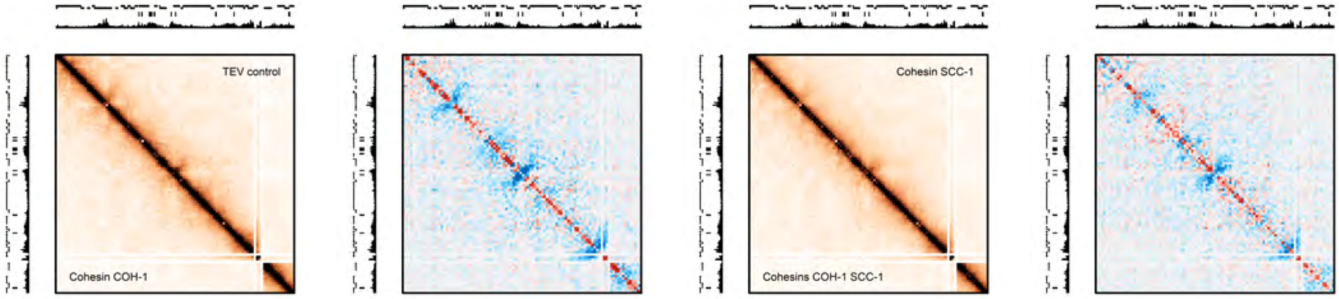

chrI:2029001-2229000

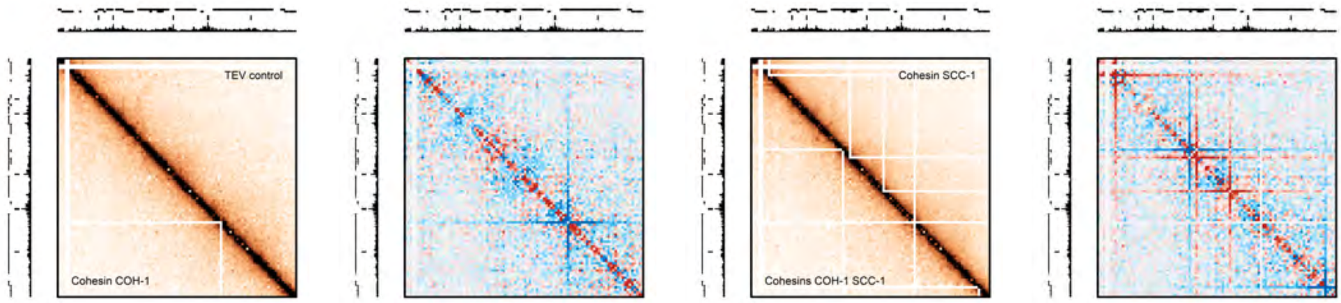

chrIV:5557001-5757000

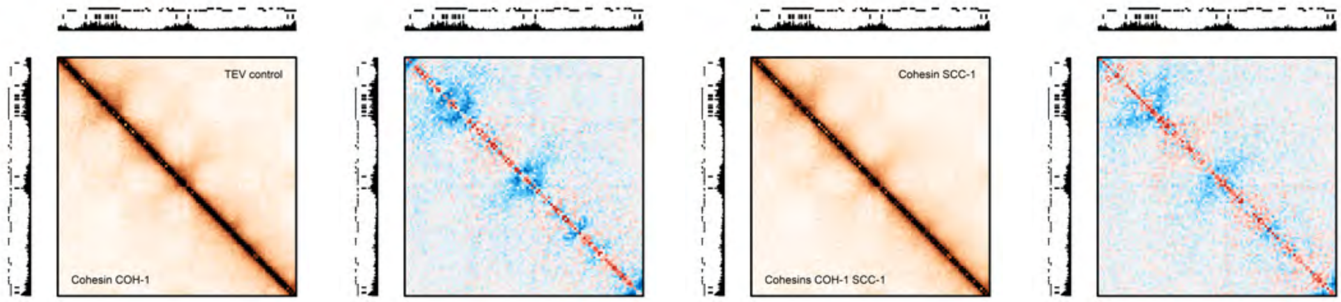

chrV:8113001-8313000

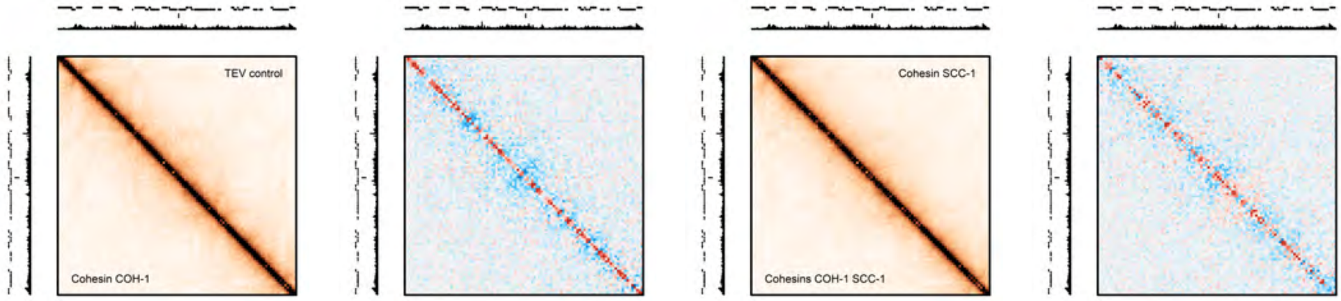

chrIV:10077001-10277000

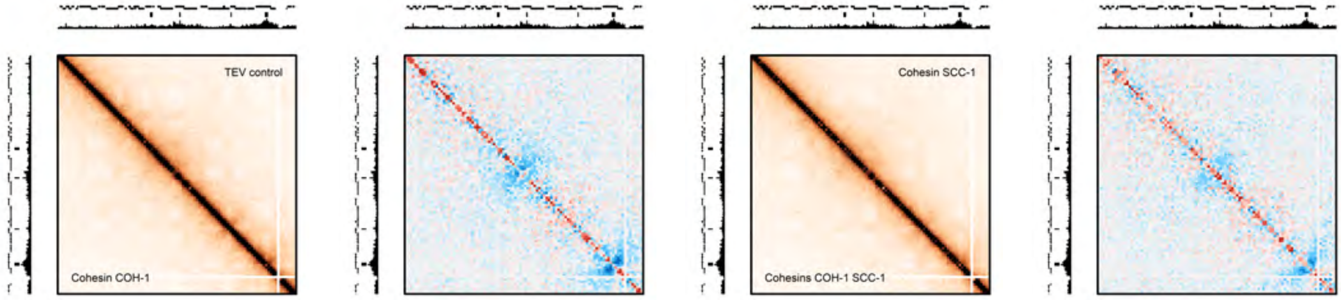

chrV:2141001-2341000

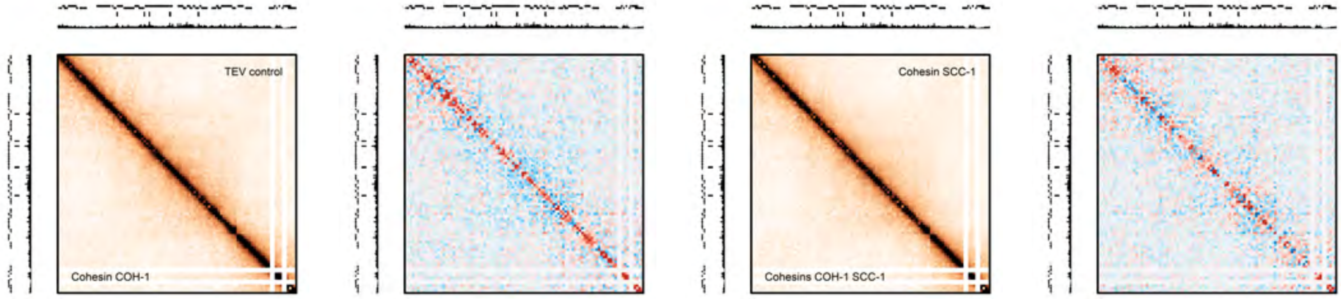

chrV:5121001-5321000

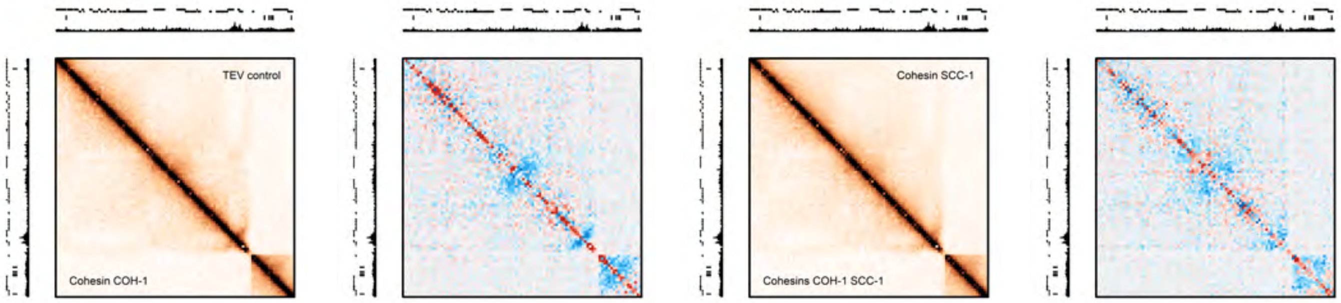

chrII:14841001-15041000

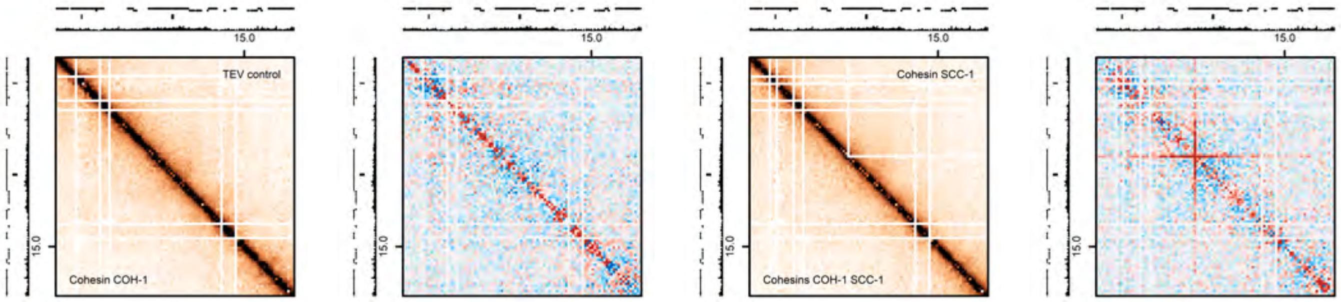

chrI:3303001-3503000

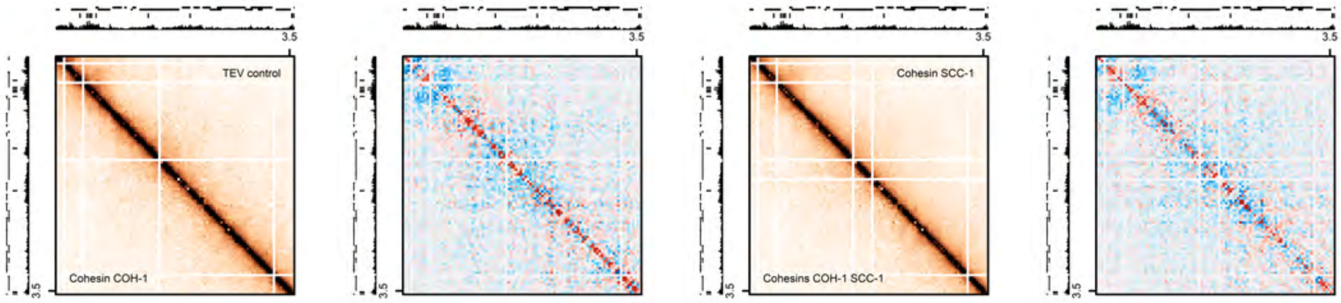

chrII:10067001-10267000

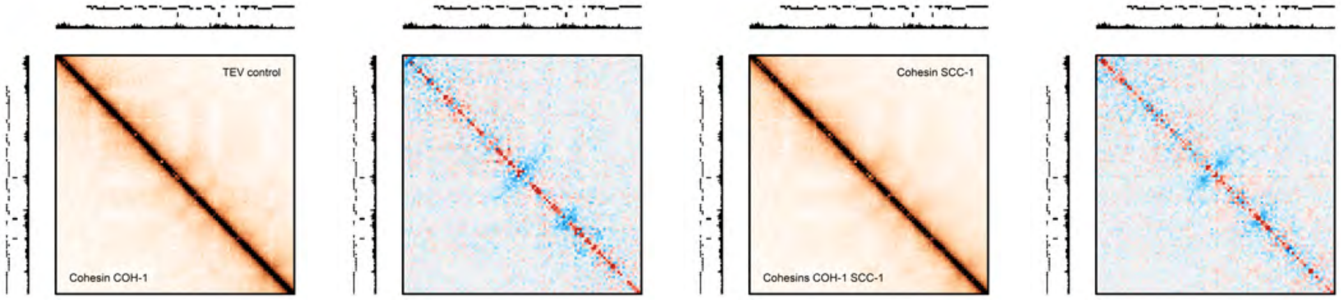

chrIV:11391001-11591000

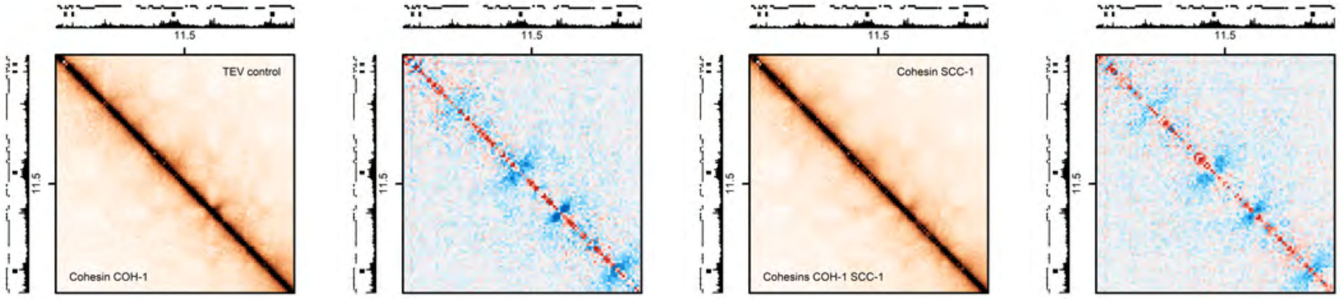

chrIV:5999001-6199000

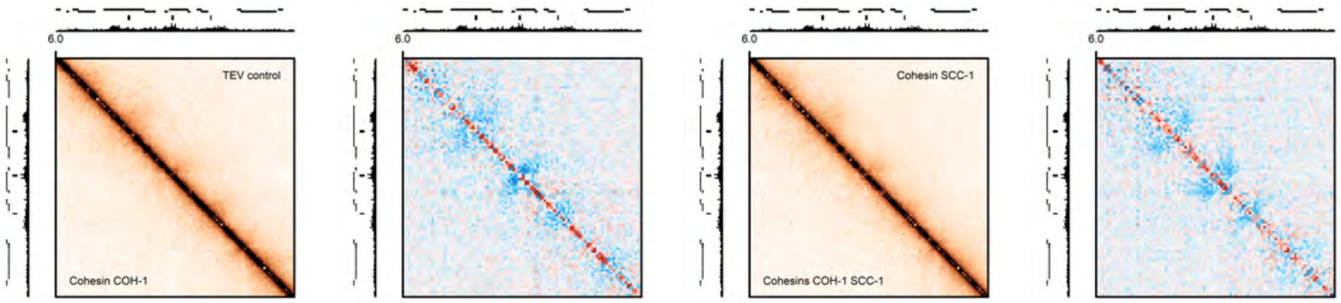

chrIII:4095001-4295000

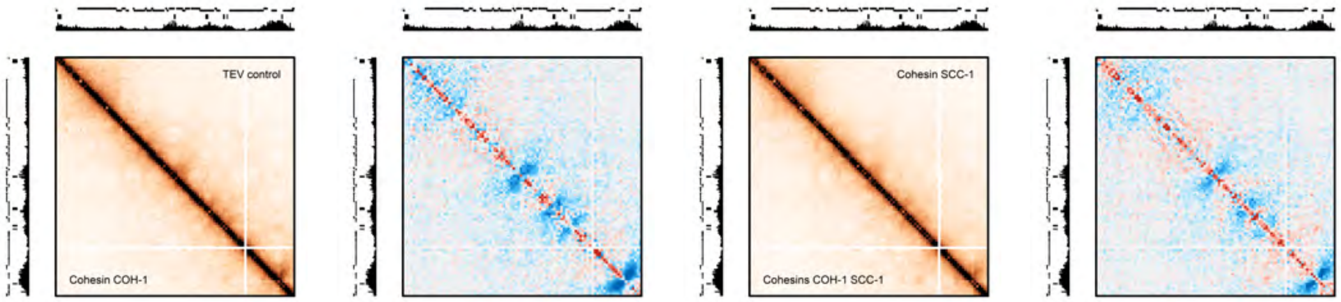

chrIII:9379001-9579000

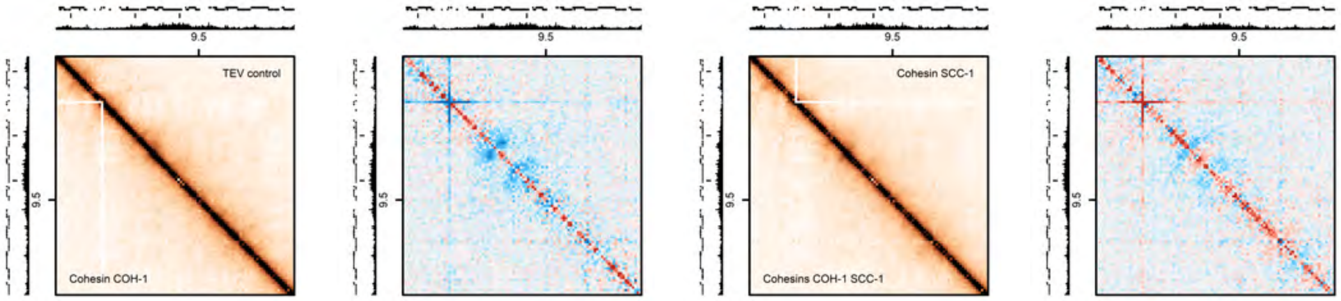

chrI:8479001-8679000

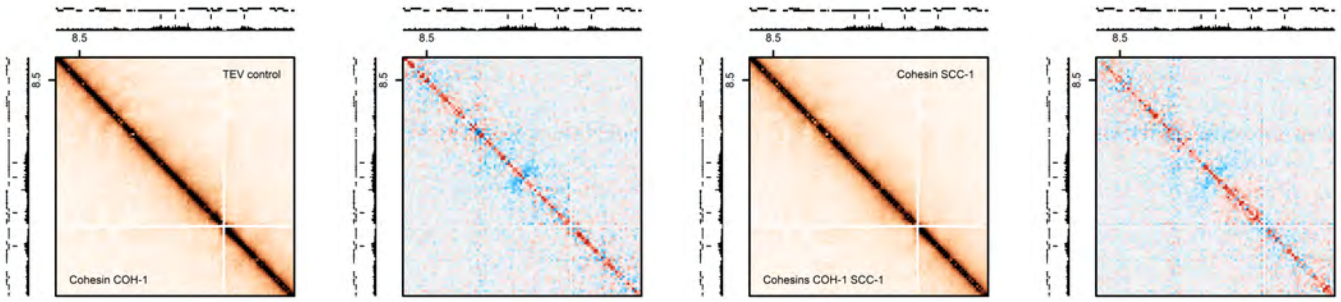

chrII:12777001-12977000

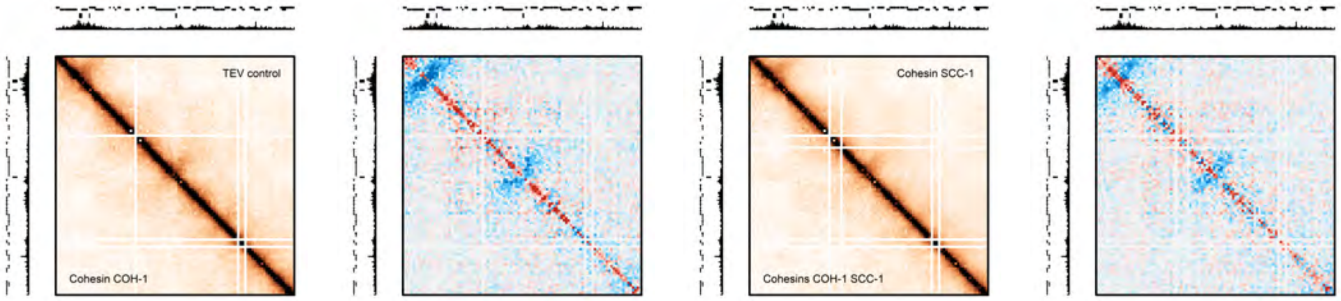

chrX:6563001-6763000

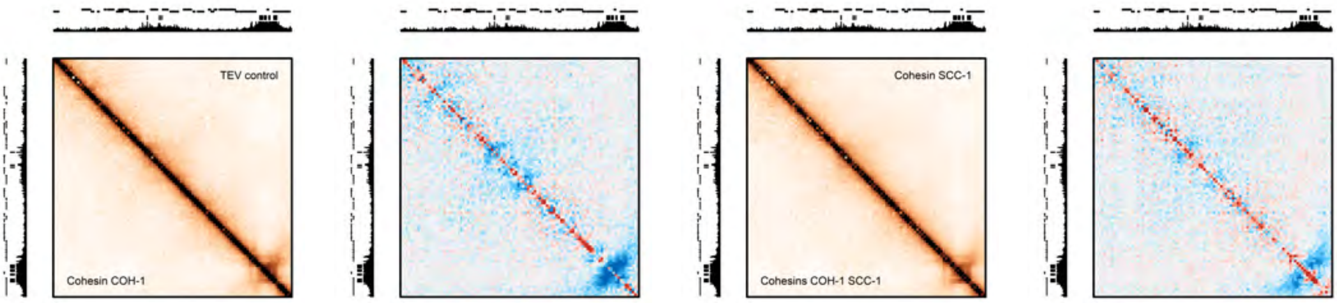

chrX:2871001-3071000

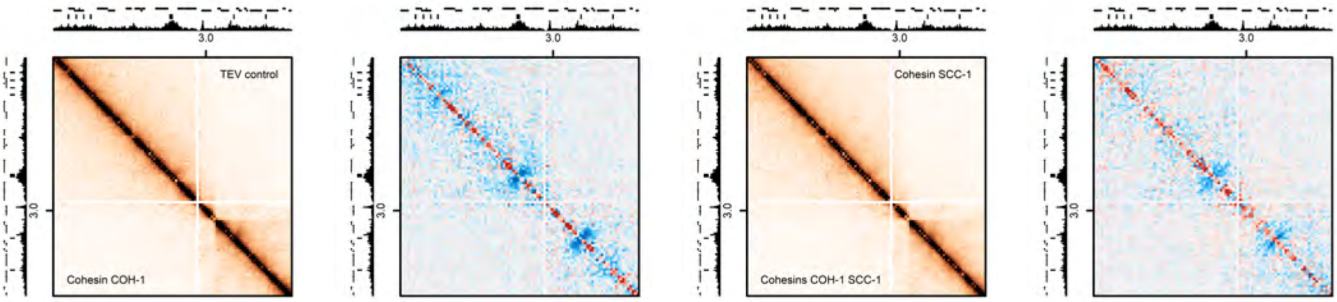

chrII:625001-825000

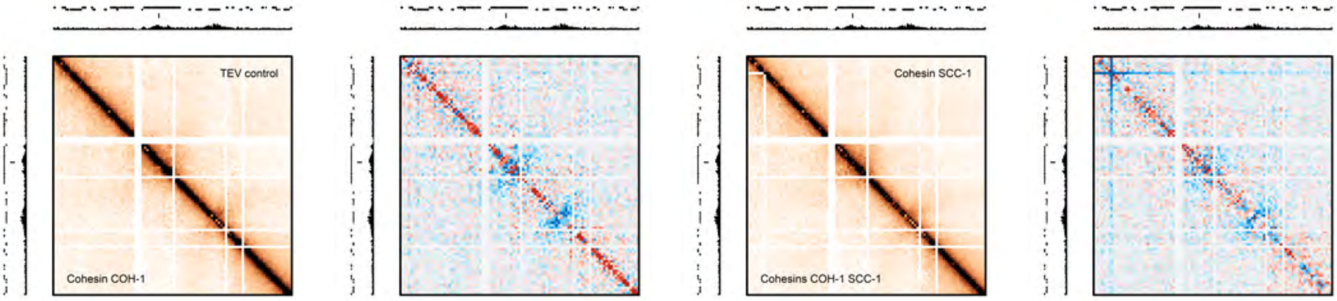

chrV:12367001-12567000

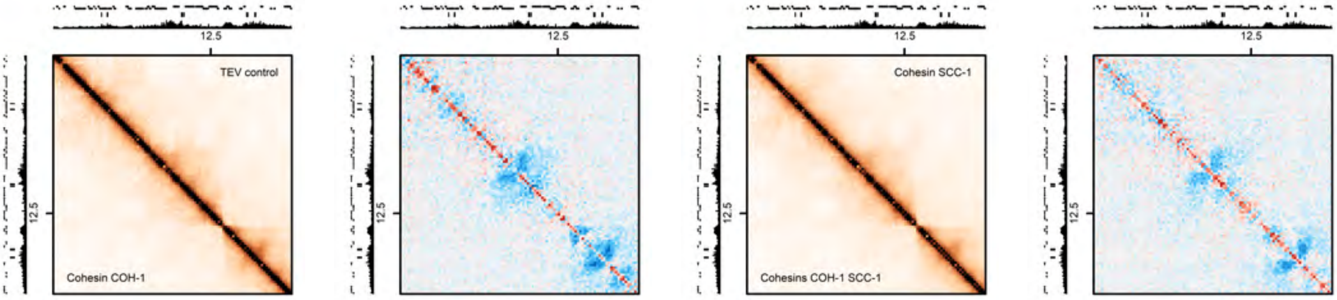

chrV:17561001-17761000

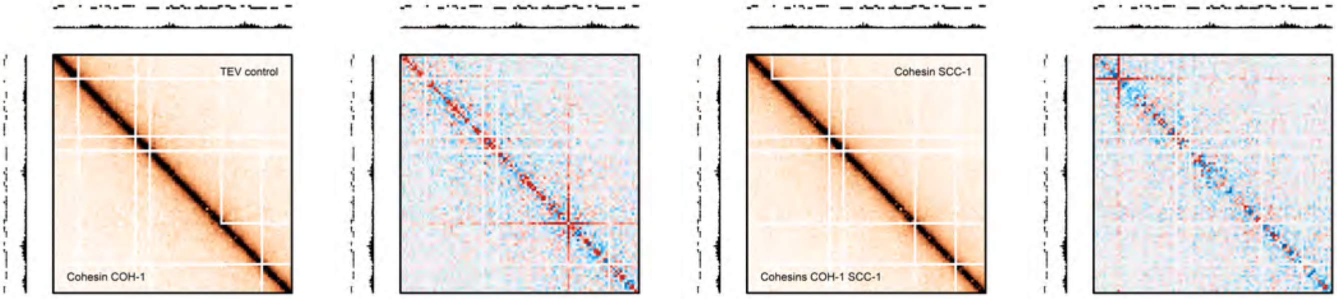

chrV:18131001-18331000

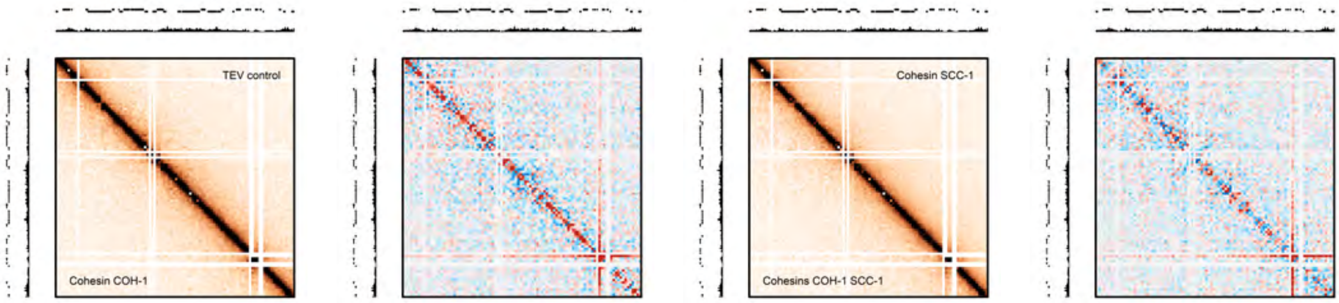

chrX:14461001-14661000

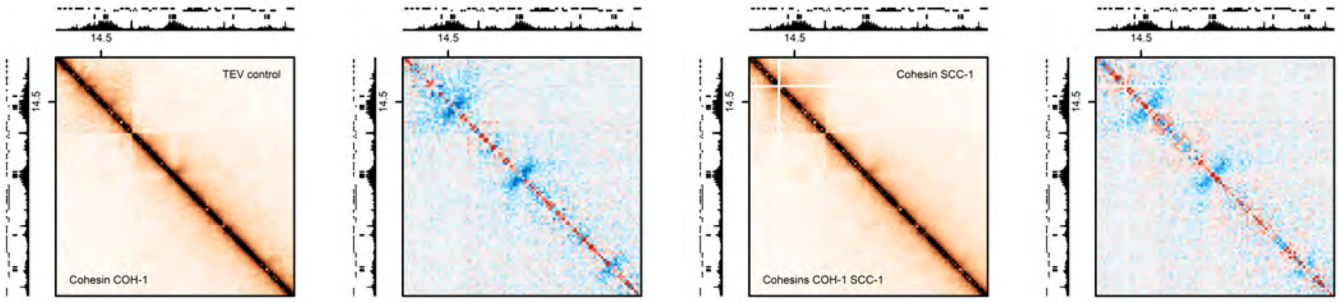

chrIV:3995001-4195000

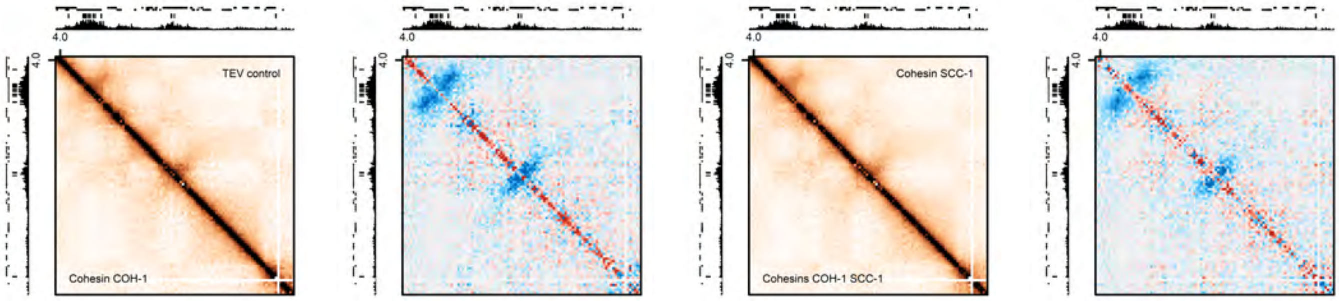

chrII:6045001-6245000

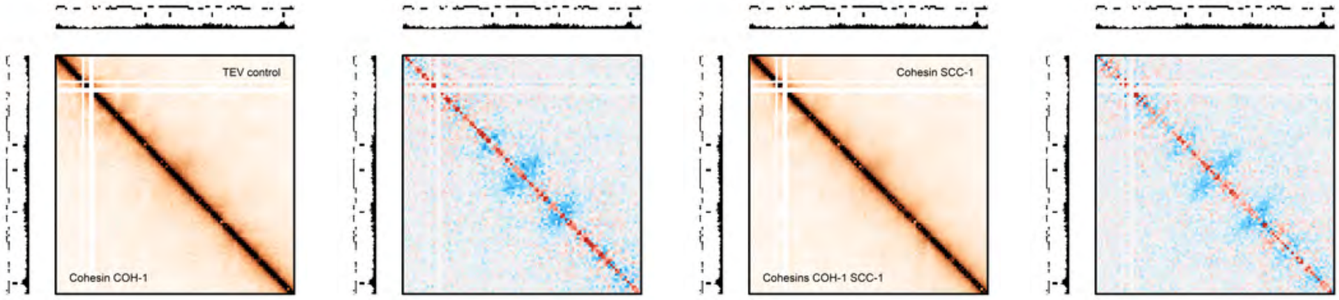

chrIV:9621001-9821000

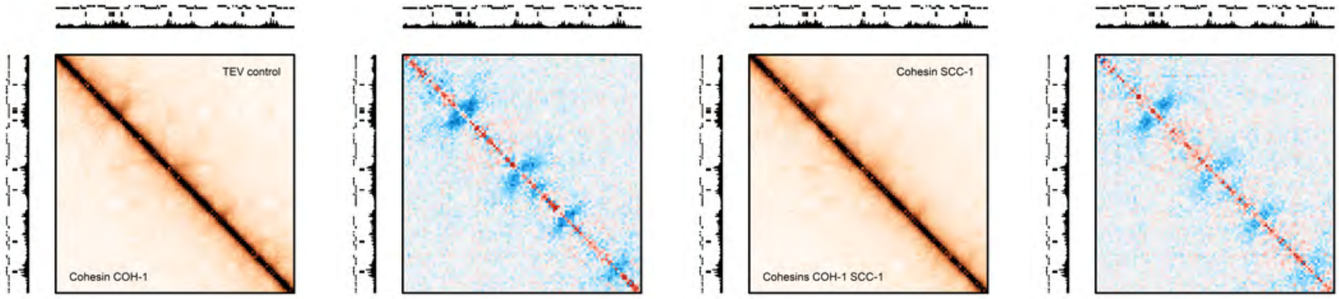

chrI:11683001-11883000

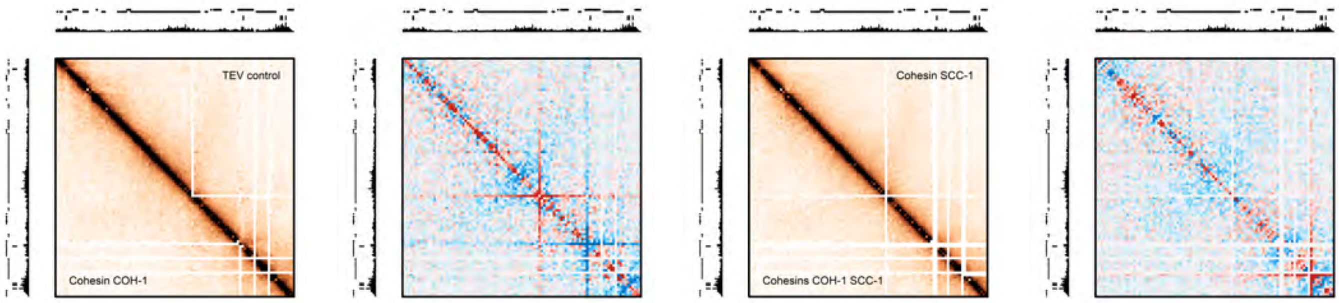

chrI:10185001-10385000

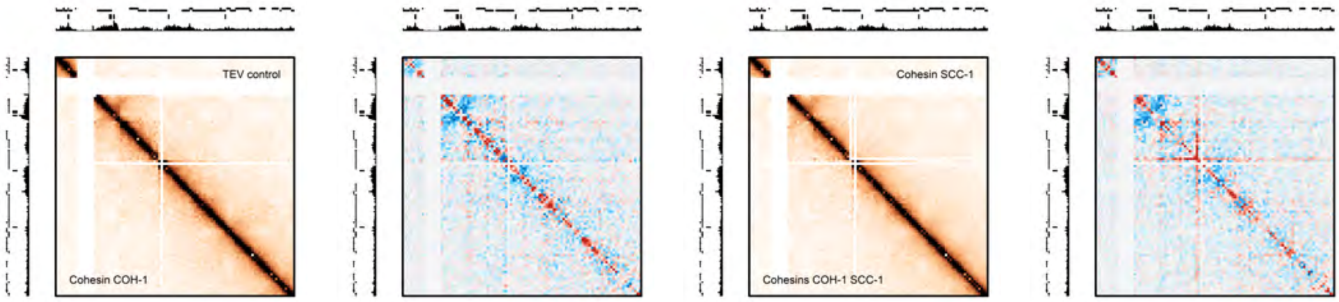

chrIII:1579001-1779000

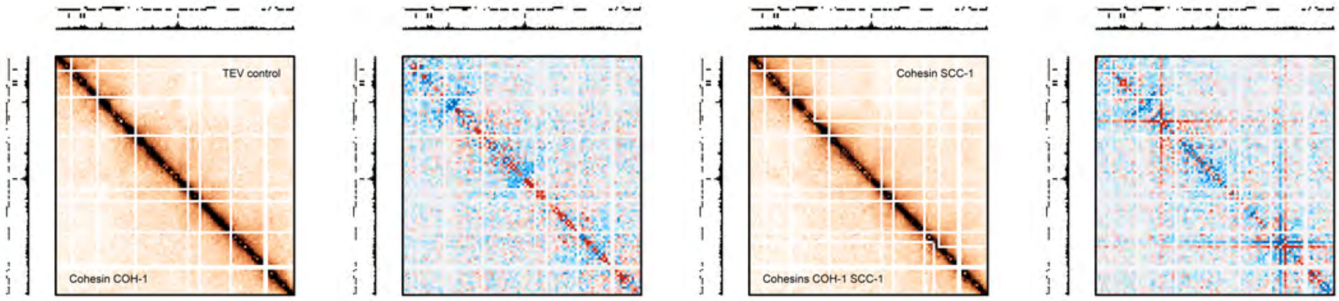

chrIV:11019001-11219000

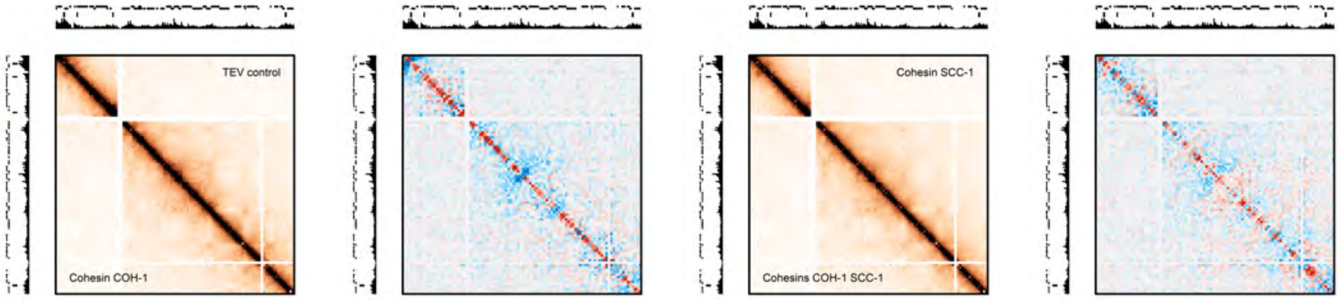

chrII:3427001-3627000

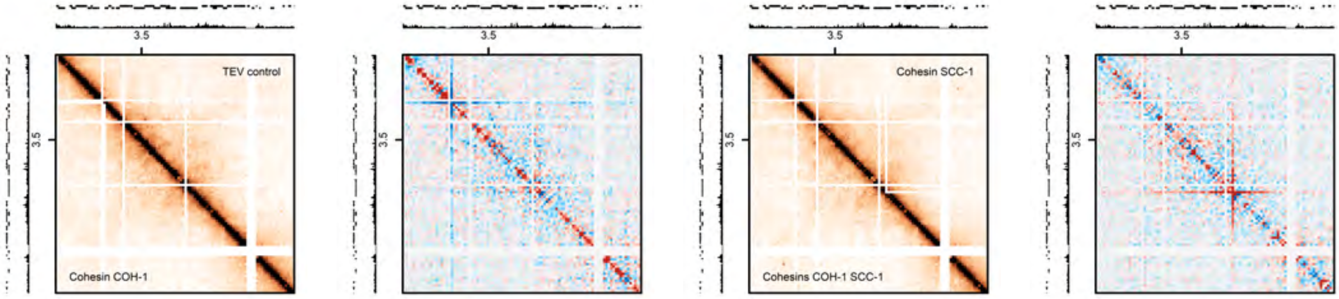

chrX:3367001-3567000

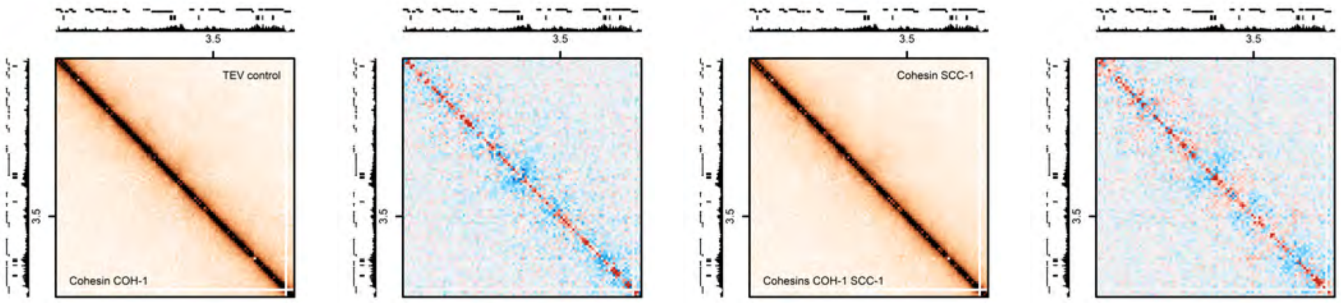

chrIII:5321001-5521000

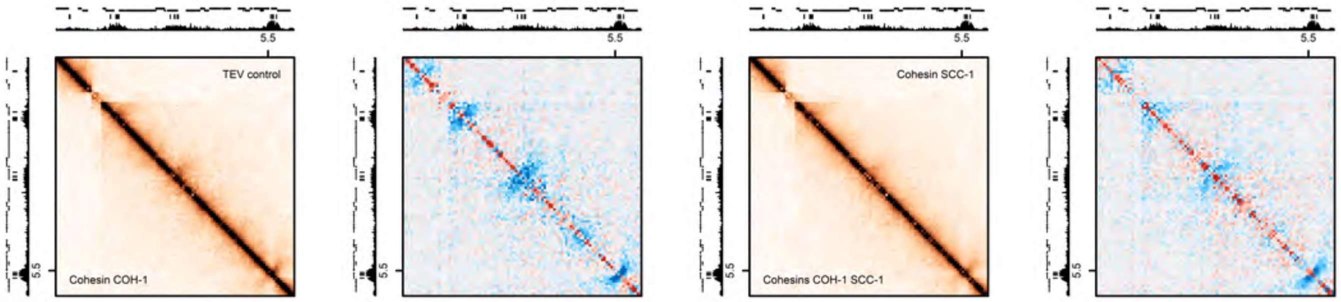

chrV:19105001-19305000

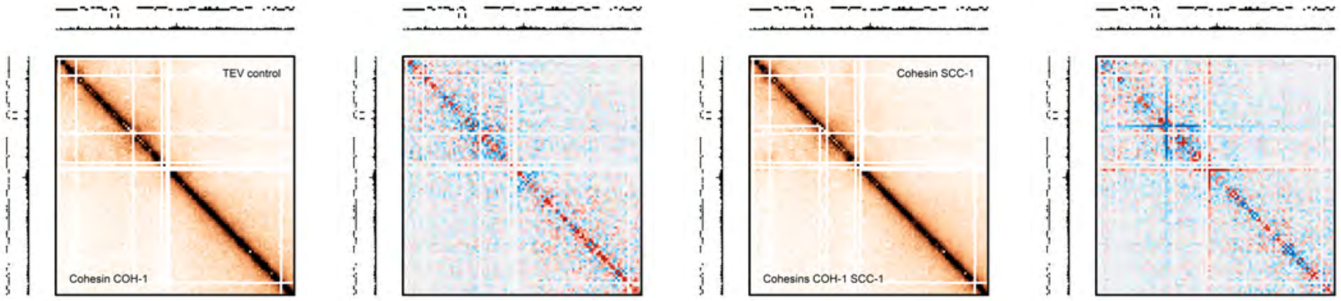

chrV:7335001-7535000

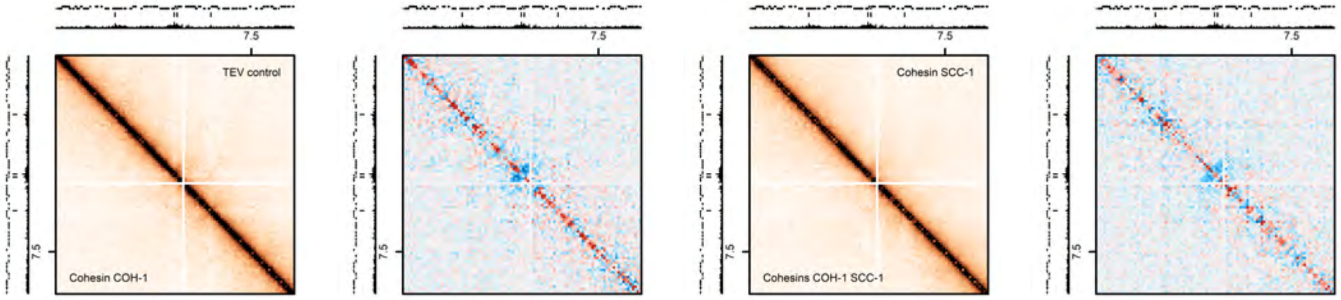

chrI:3147001-3347000

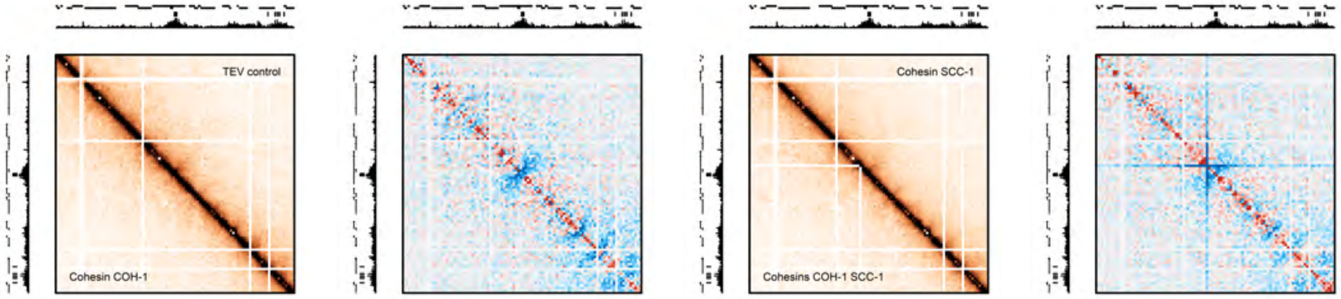

chrV:13181001-13381000

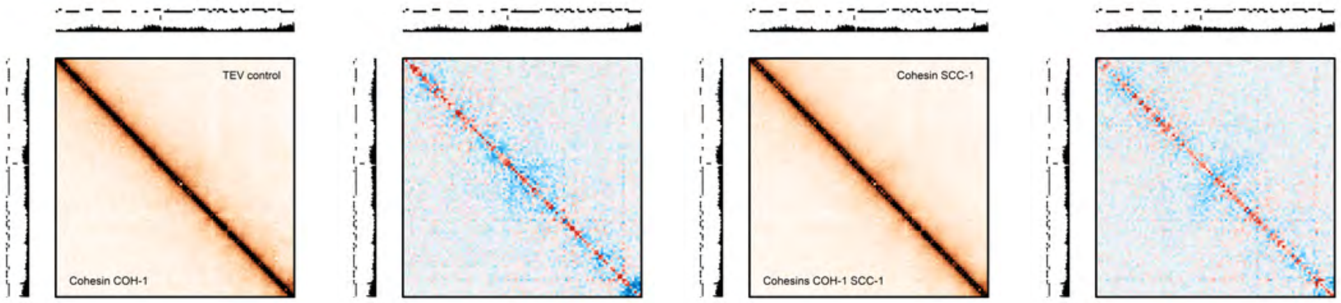

chrI:12075001-12275000

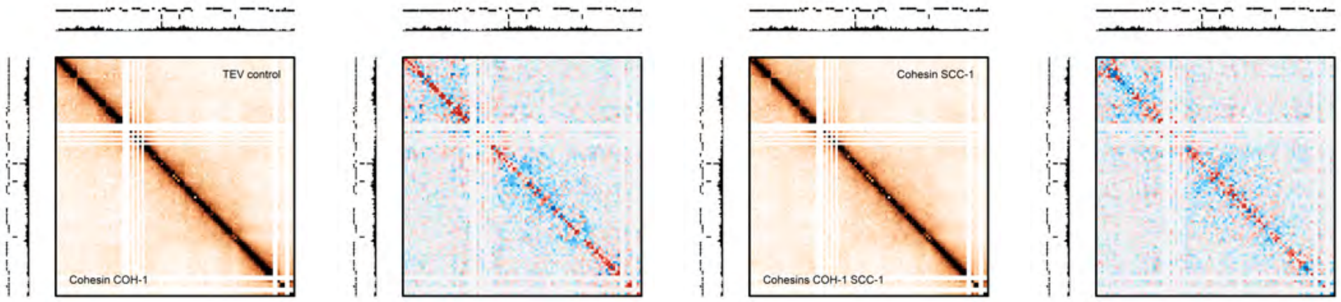

chrII:5365001-5565000

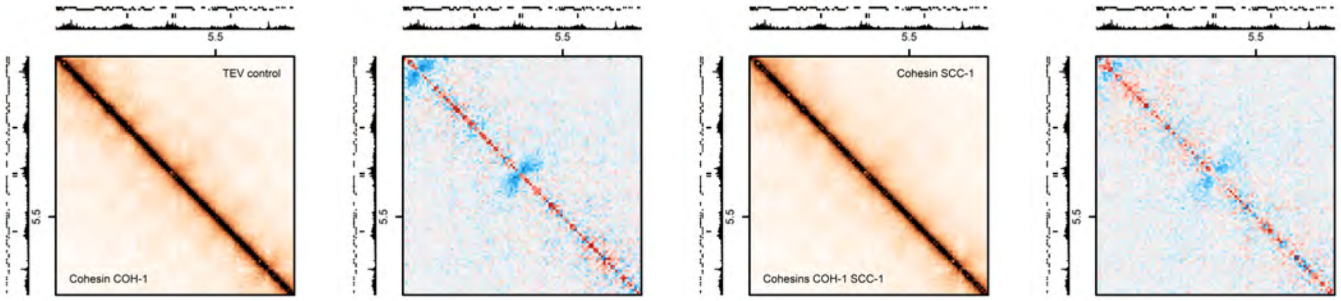

chrI:2317001-2517000

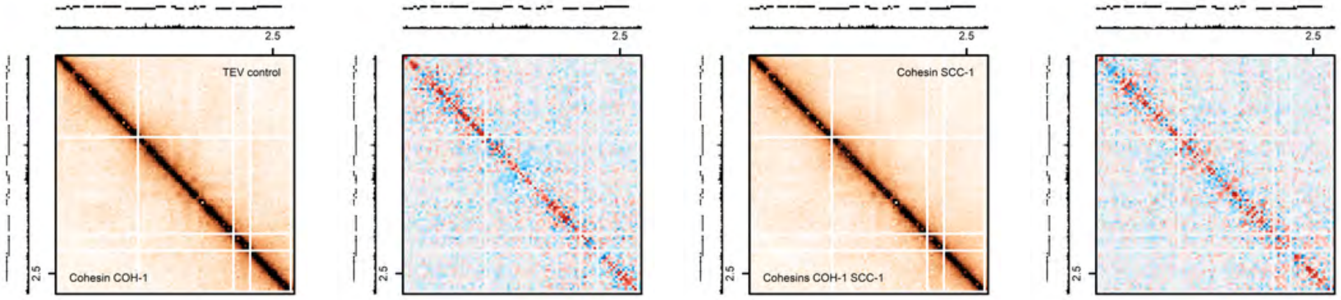

chrX:11505001-11705000

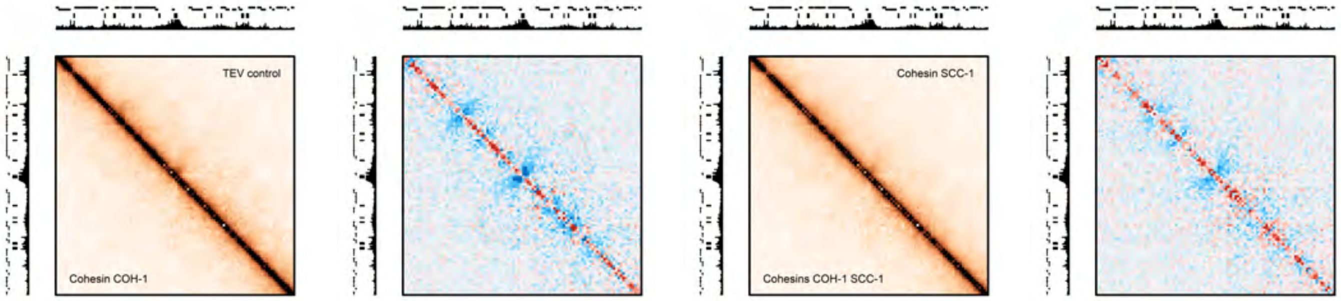

chrIV:9023001-9223000

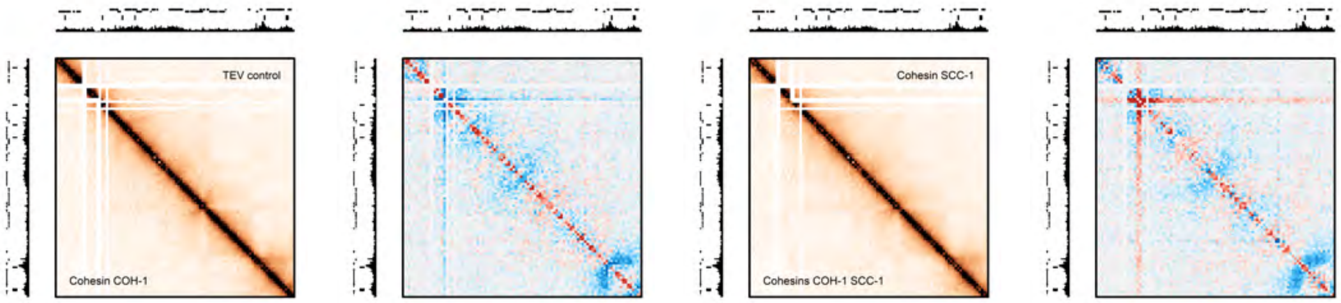

chrIII:13361001-13561000

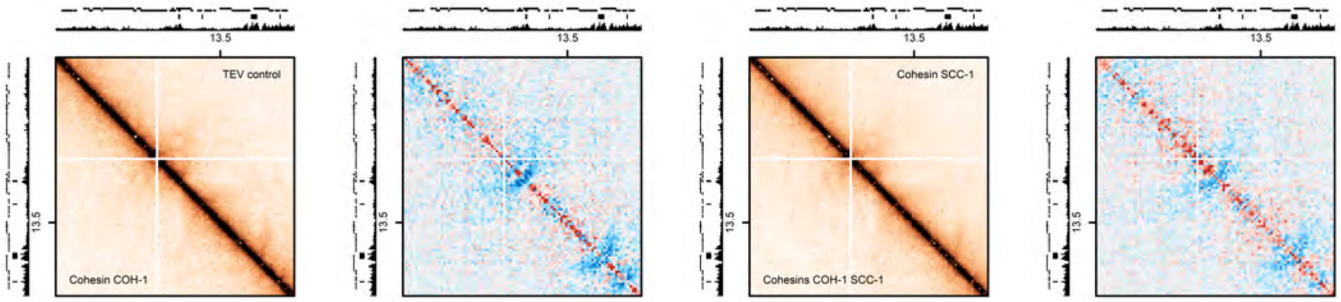

chrI:13735001-13935000

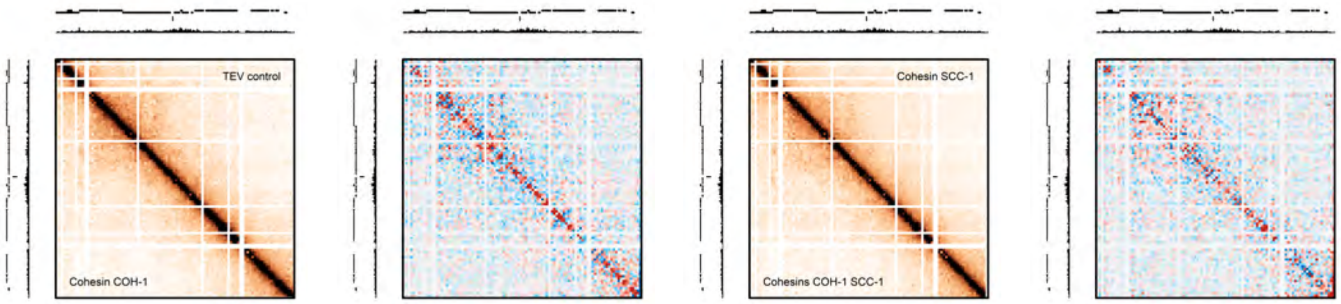

chrIV:1187001-1387000

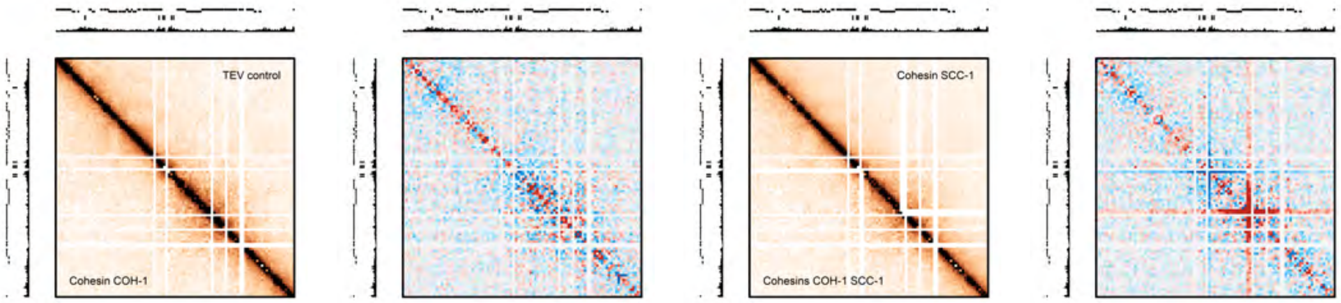

chrX:8703001-8903000

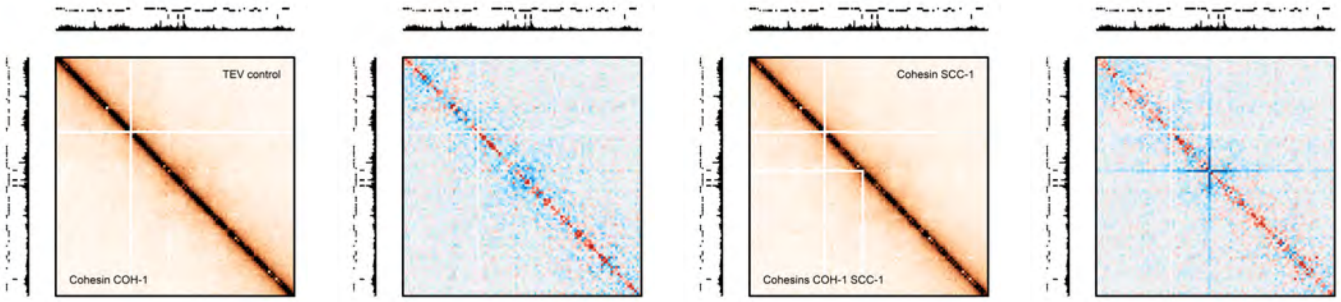

chrV:363001-563000

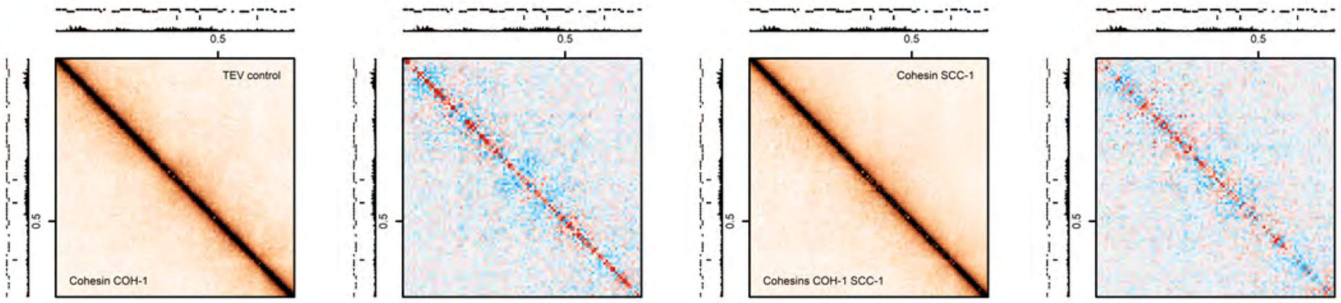

chrIII:10231001-10431000

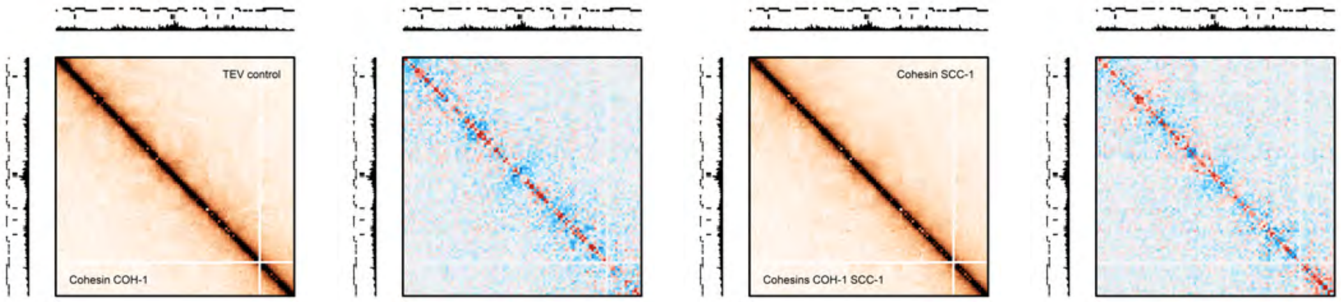

chrI:14077001-14277000

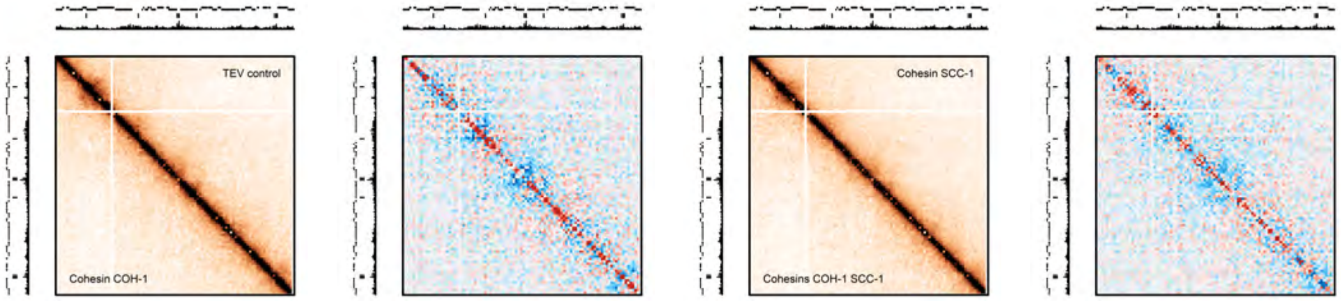

chrI:13395001-13595000

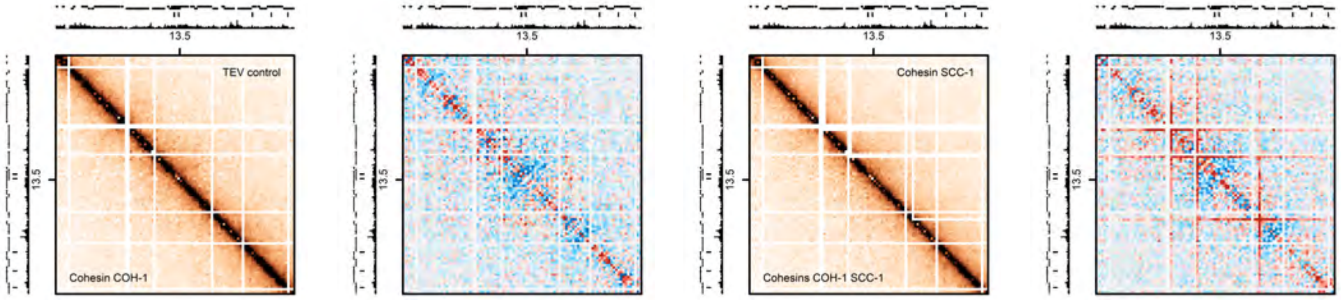

chrIII:5587001-5787000

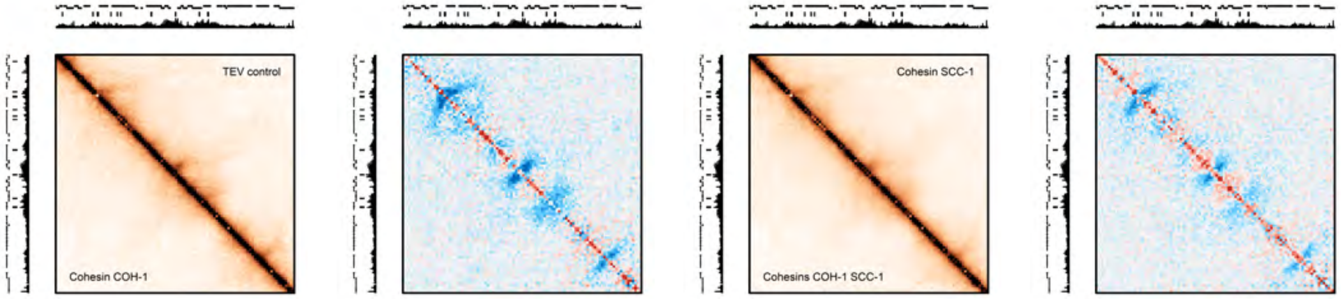

chrII:3337001-3537000

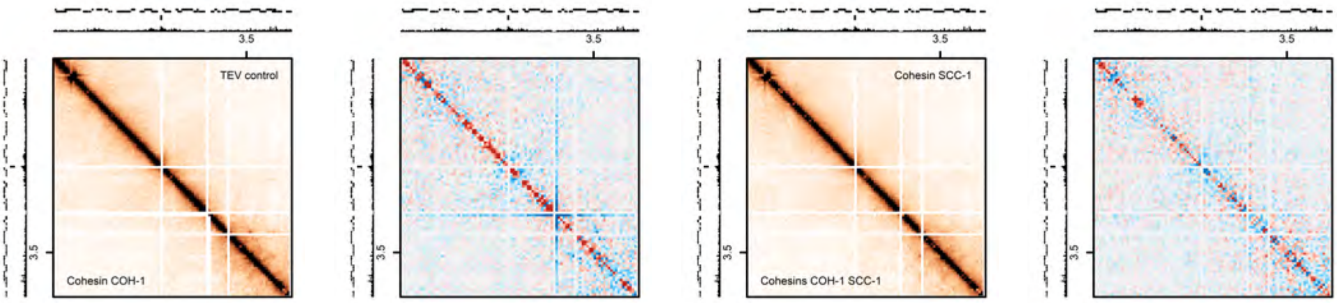

chrV:11537001-11737000

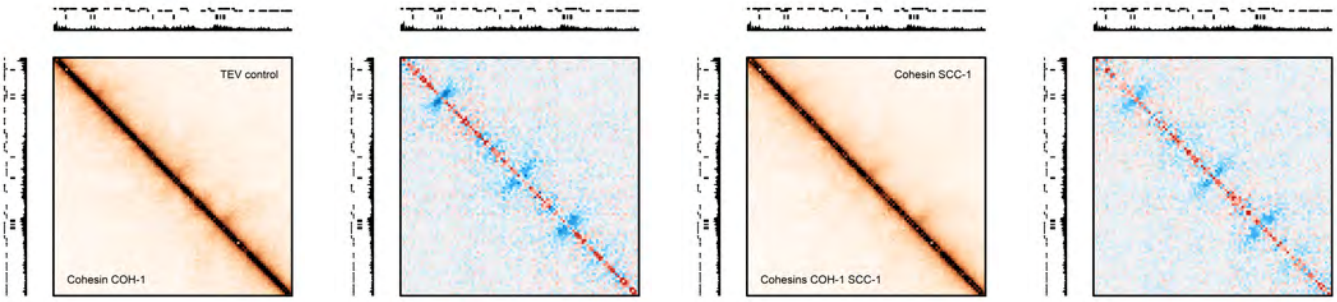

chrV:7919001-8119000

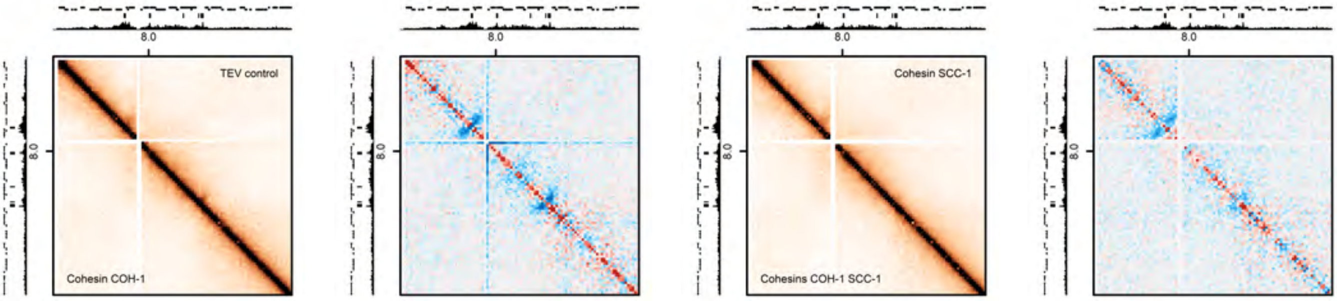

chrIII:2775001-2975000

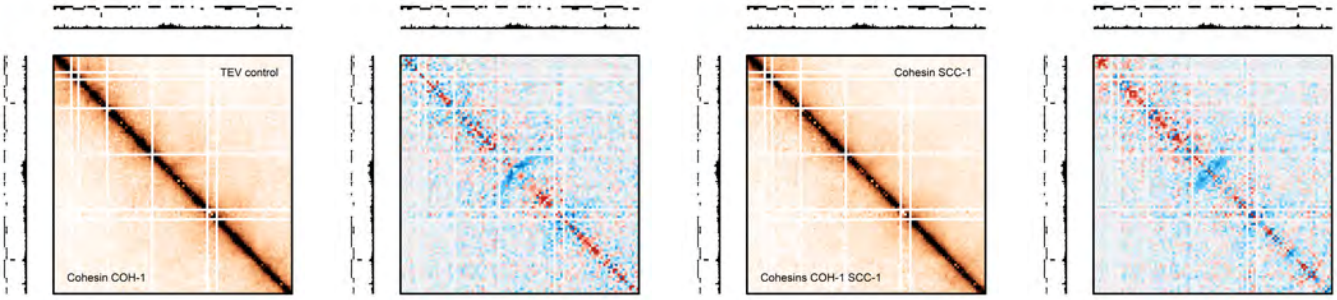

chrII:5121001-5321000

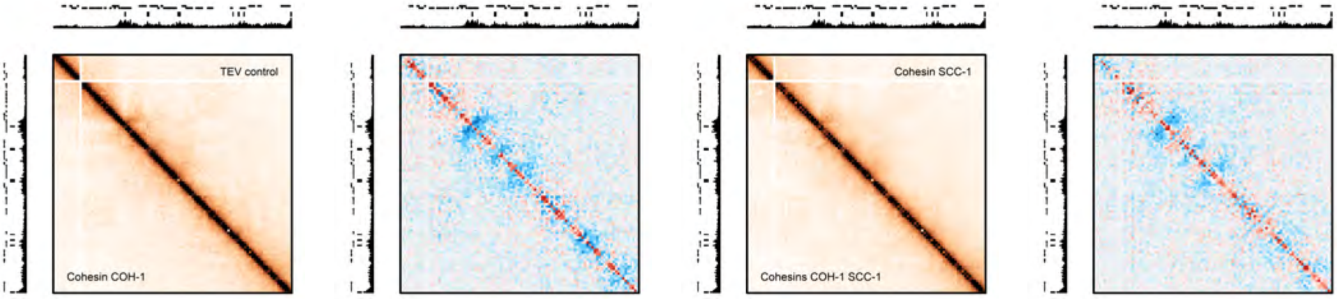

chrII:499001-699000

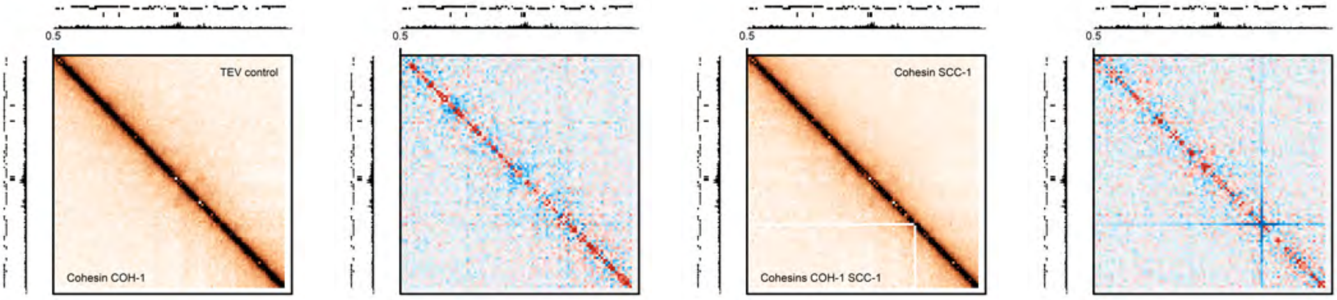

chrI:9329001-9529000

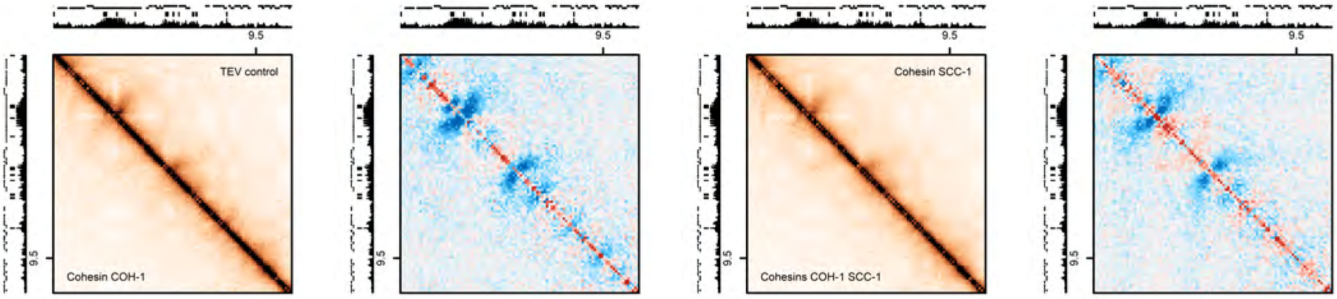

chrV:6897001-7097000

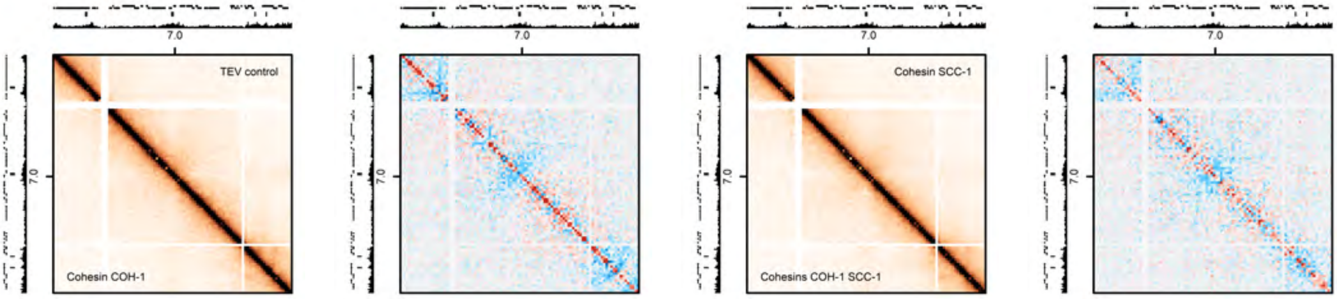

chrV:20779001-20979000

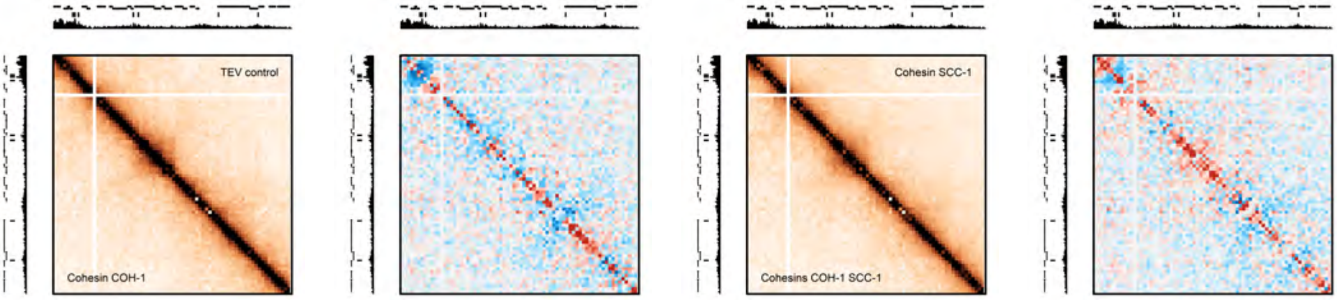

chrIII:1329001-1529000

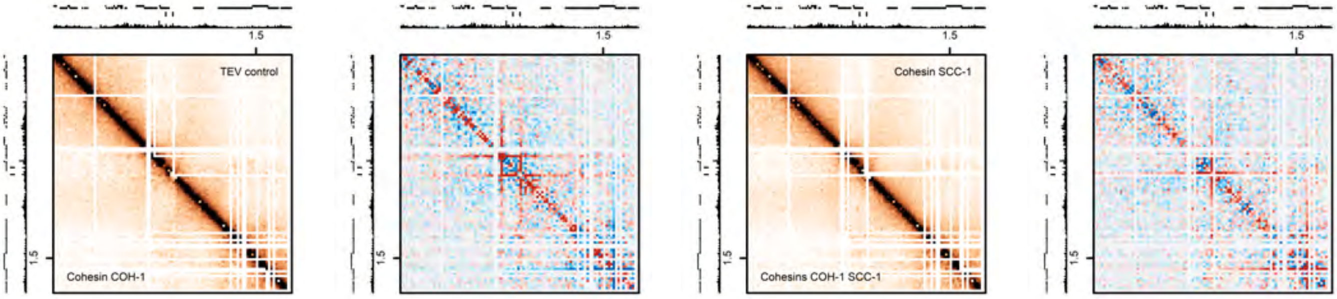

chrI:14245001-14445000

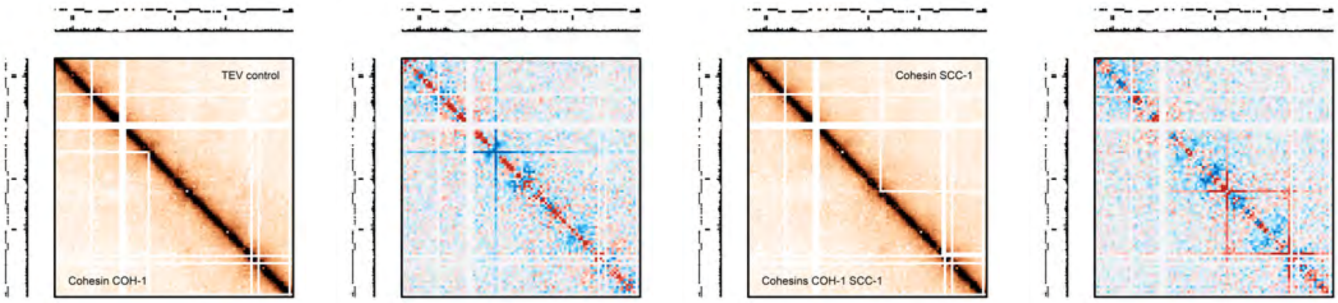

chrIV:11187001-11387000

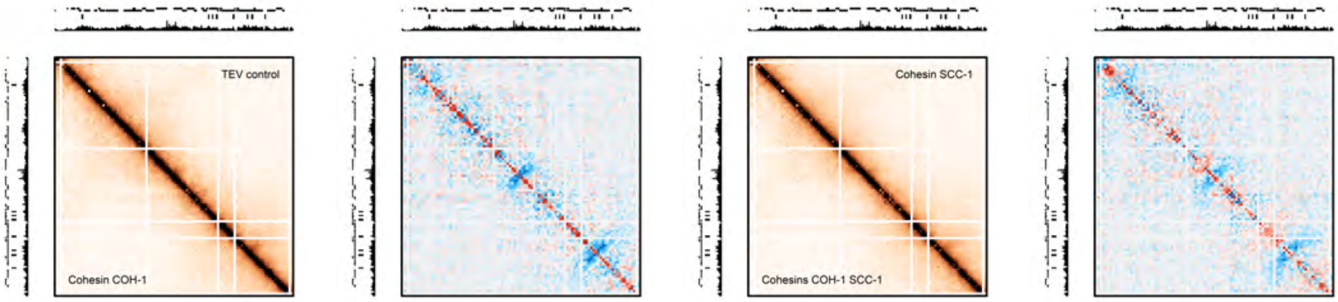

chrX:1217001-1417000

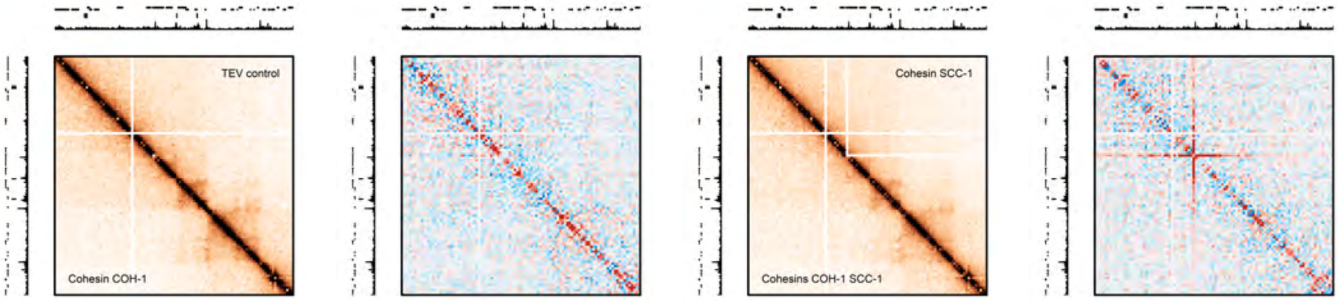

chrII:10761001-10961000

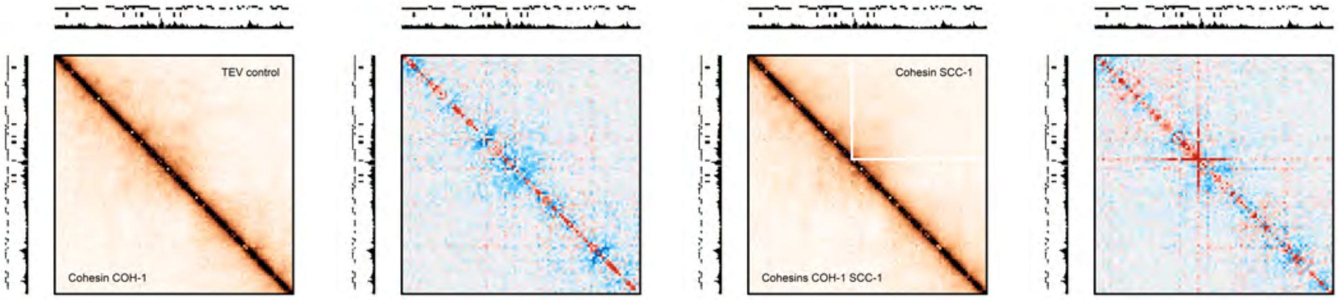

chrV:3885001-4085000

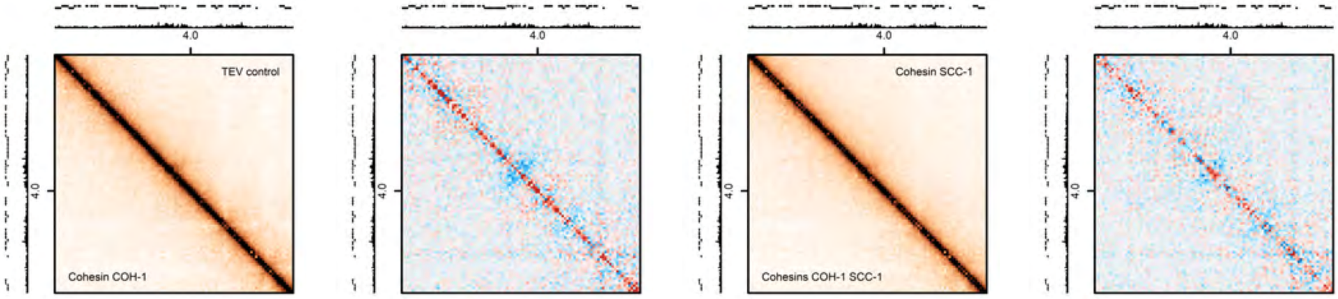

chrIII:9139001-9339000

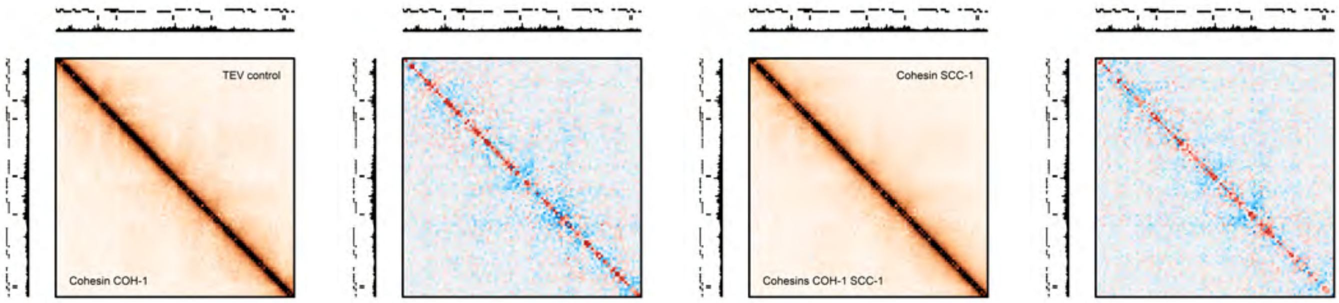

chrV:4723001-4923000

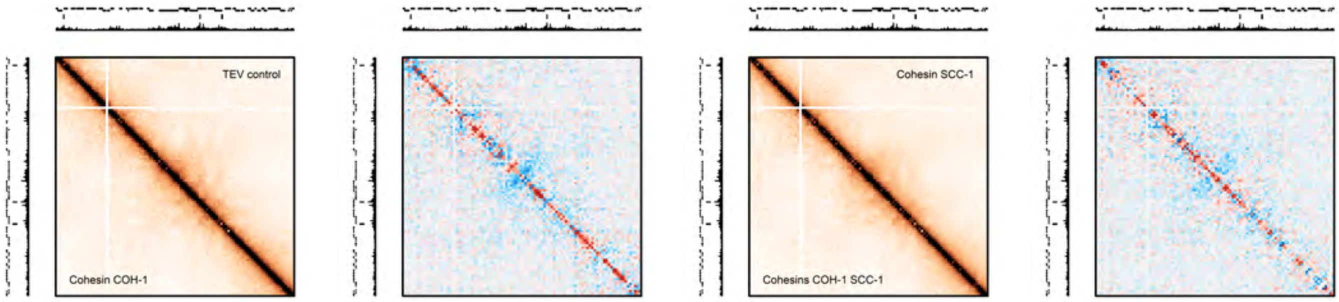

chrII:4399001-4599000

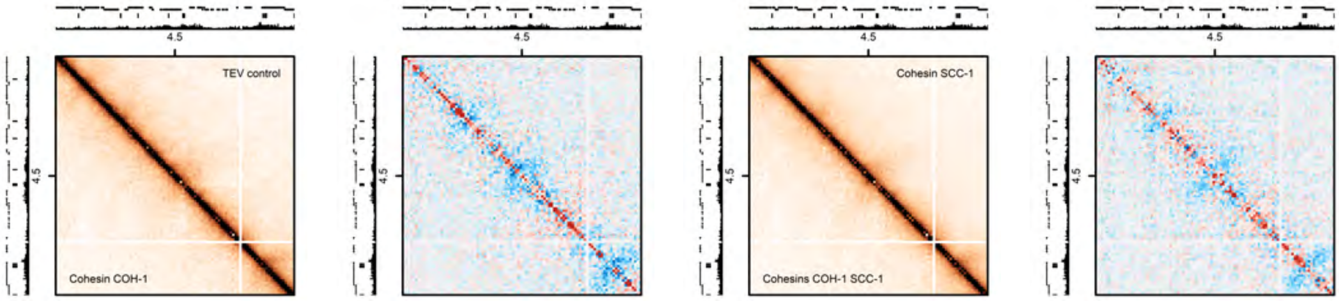

chrI:9691001-9891000

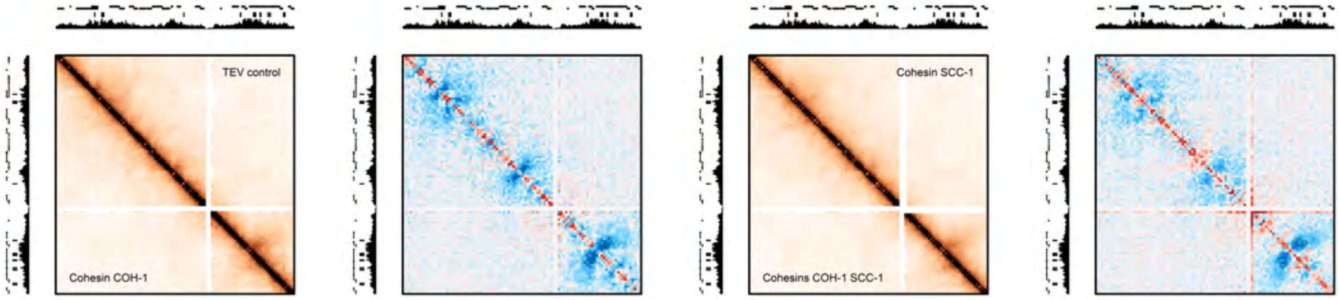

chrIV:10927001-11127000

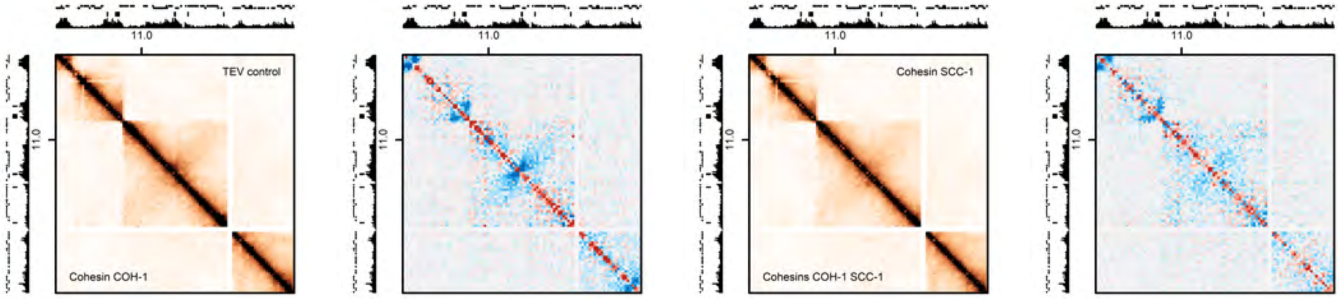

chrII:1403001-1603000

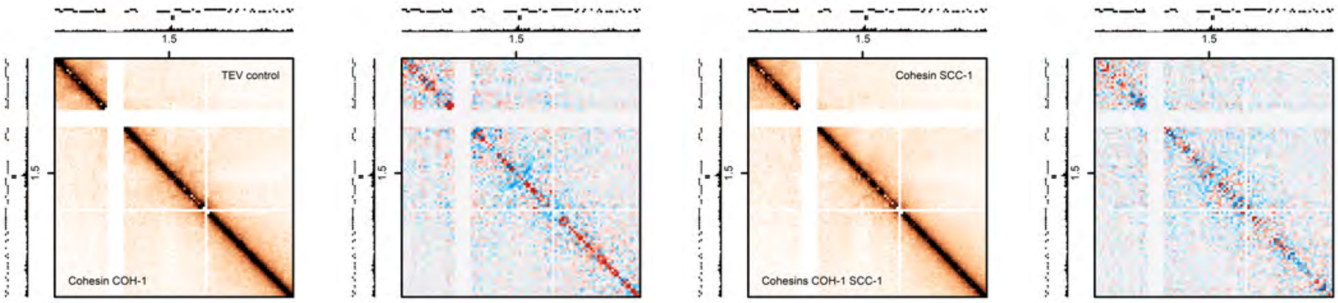

chrX:15243001-15443000

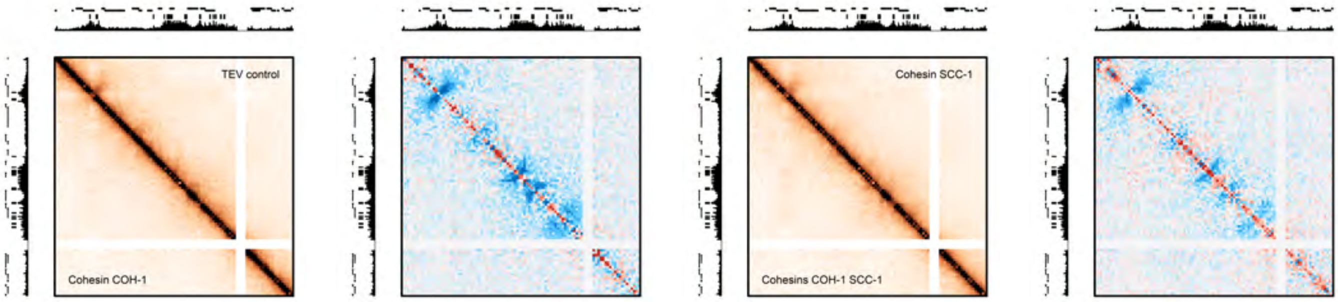

chrIV:7137001-7337000

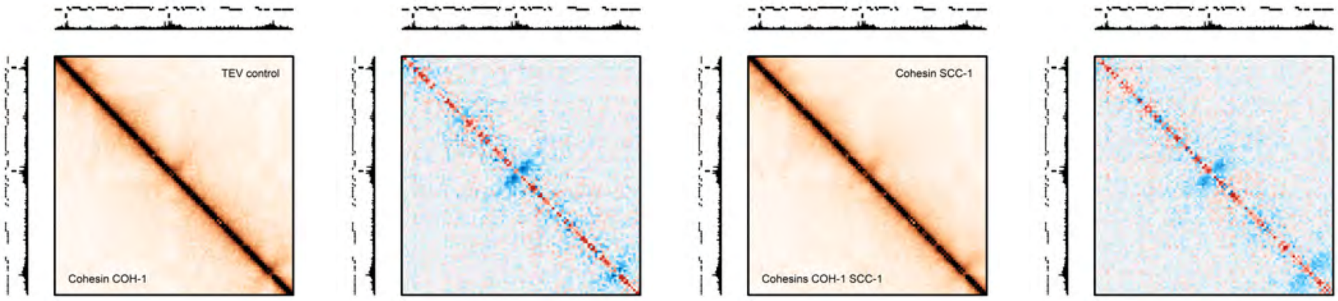

chrV:8509001-8709000

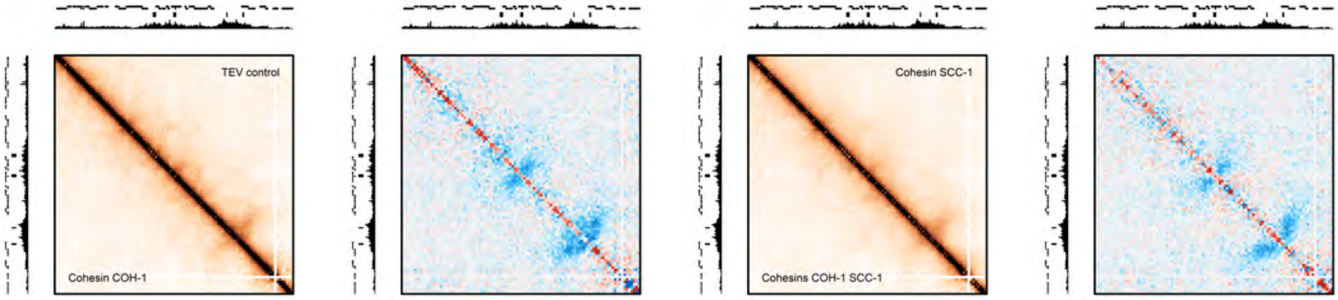

chrI:2861001-3061000

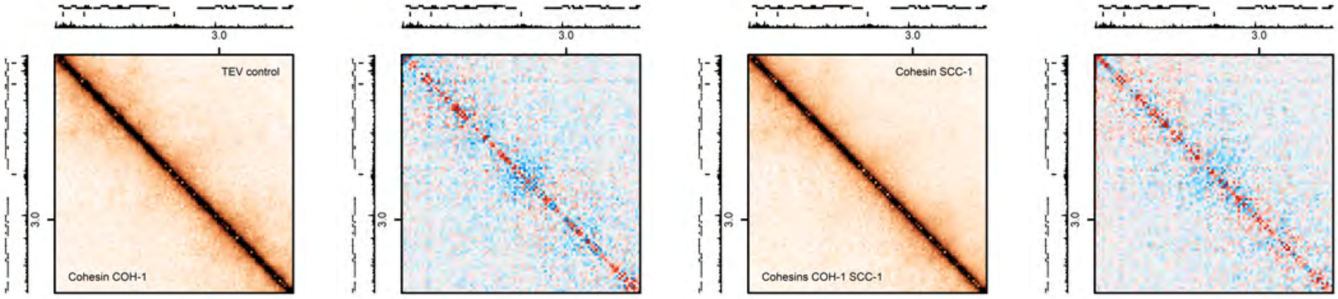

chrIV:2353001-2553000

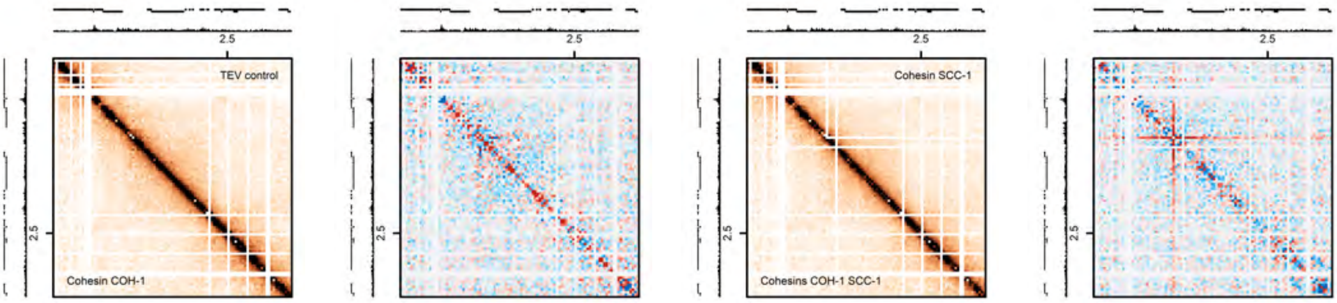

chrV:3481001-3681000

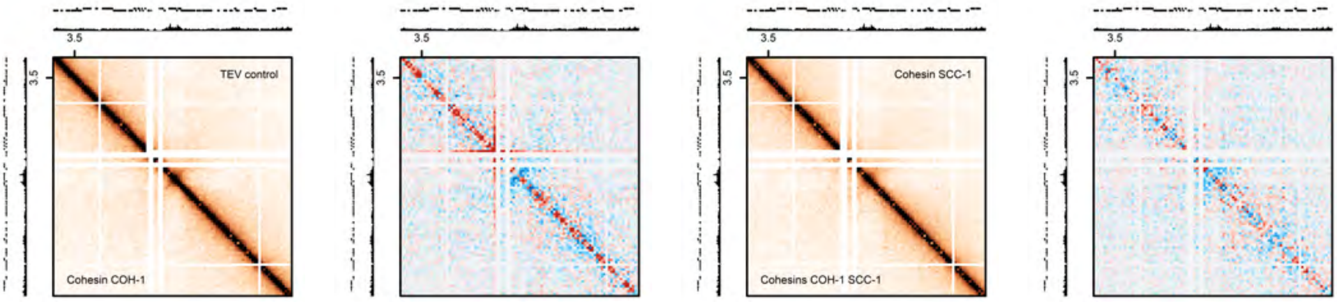

chrI:11837001-12037000

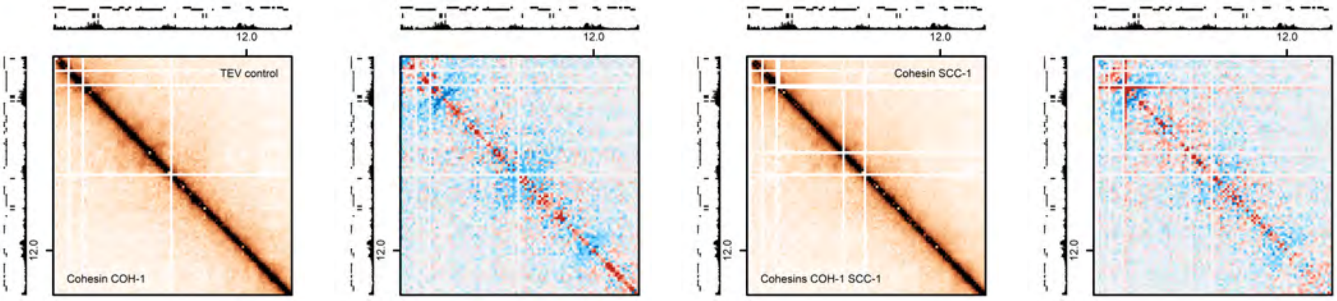

chrI:3413001-3613000

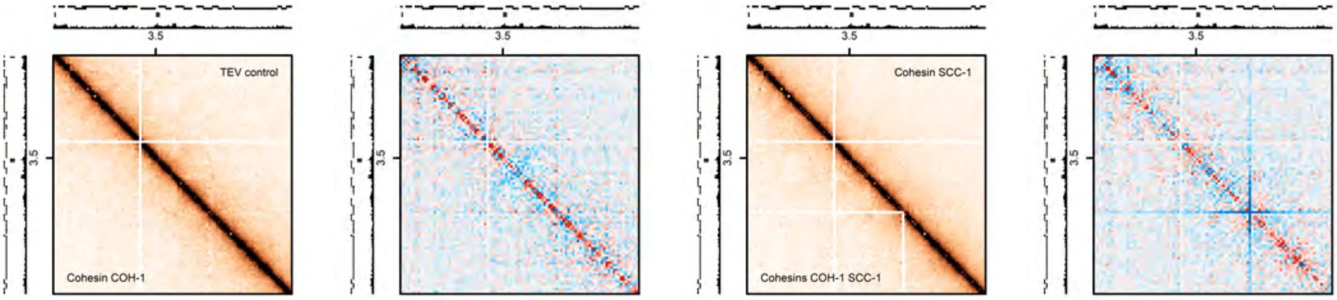

chrIV:563001-763000

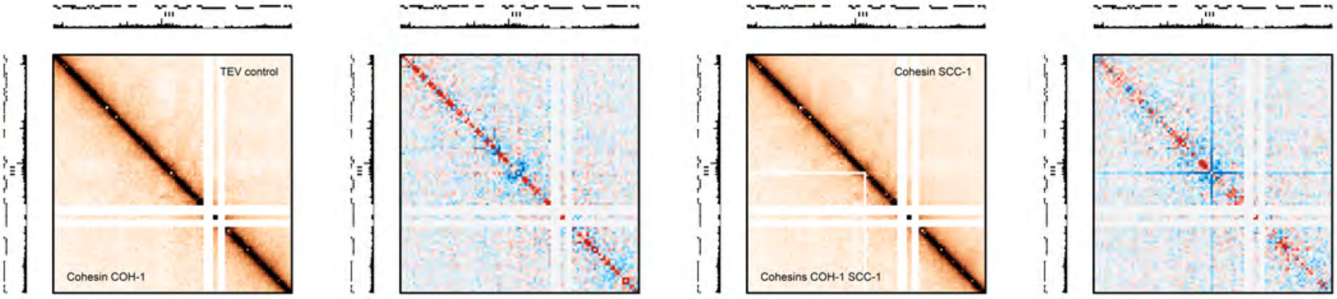

chrIV:4543001-4743000

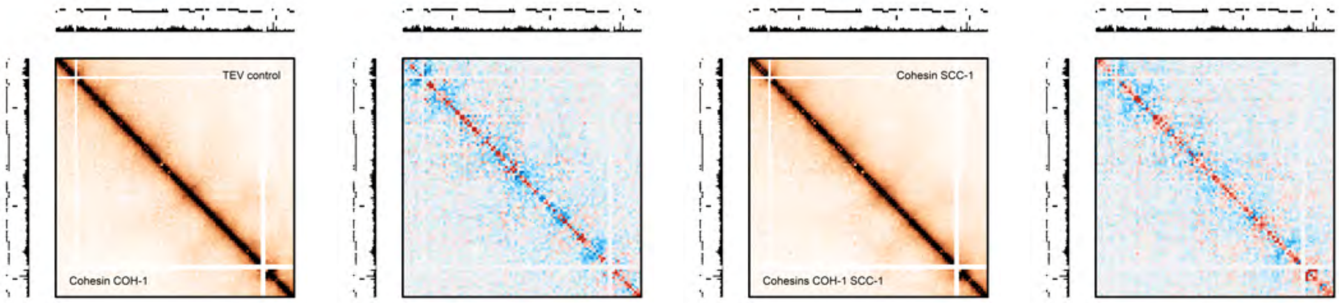

chrI:7611001-7811000

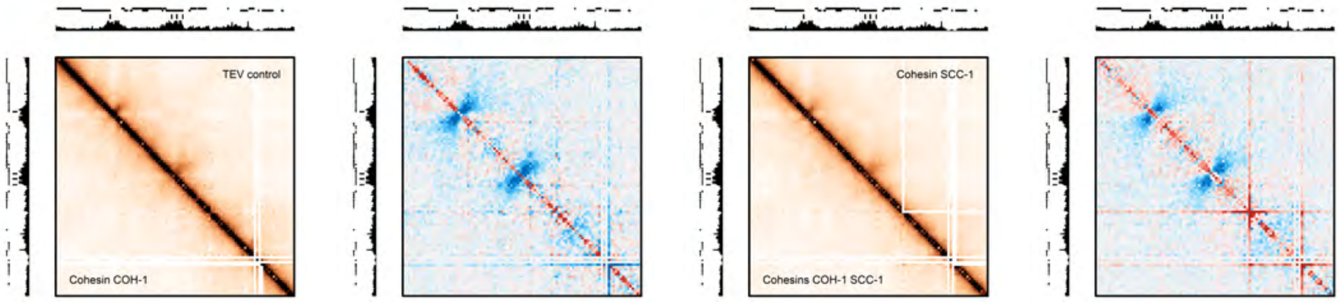

chrX:17117001-17317000

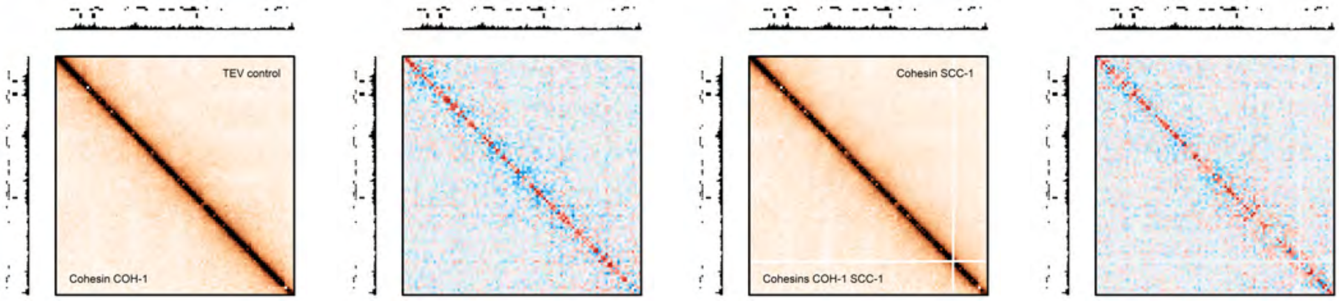

chrI:5493001-5693000

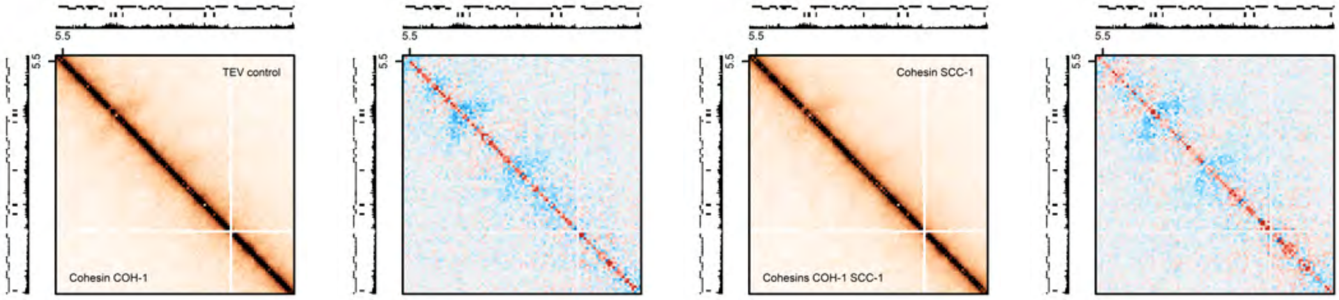

chrII:217001-417000

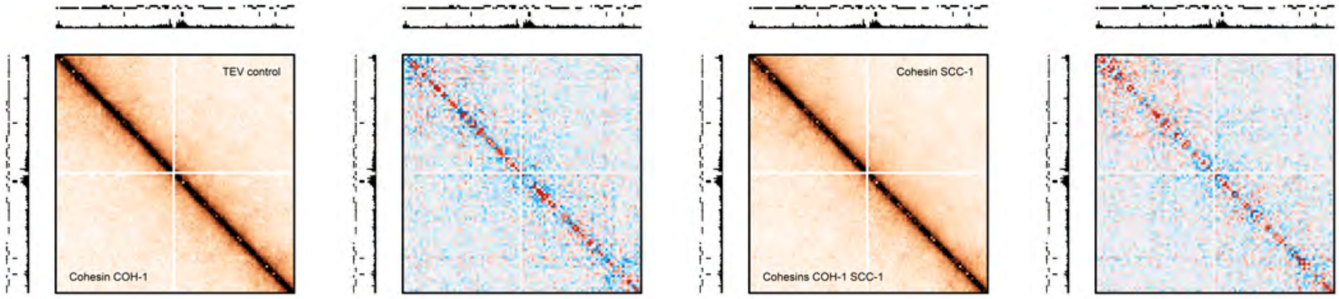

chrX:5879001-6079000

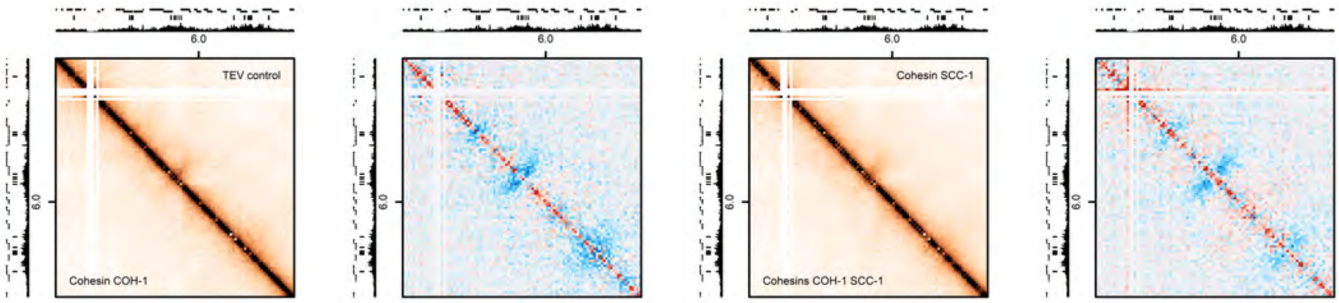

chrIII:3999001-4199000

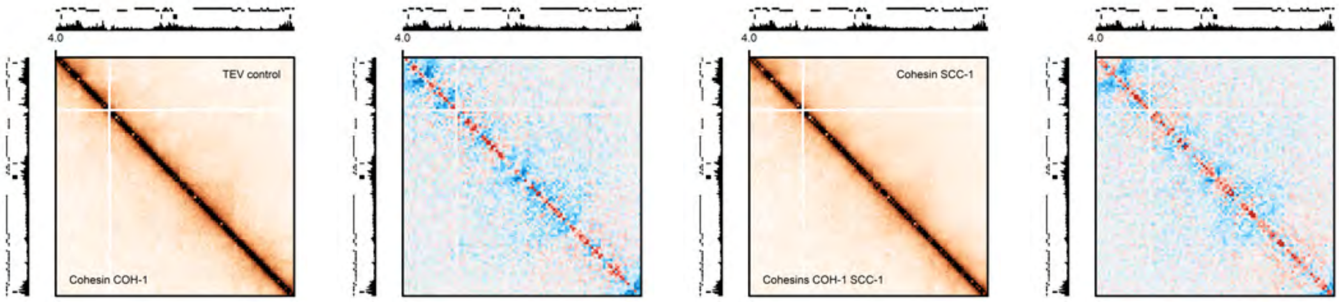

chrV:1673001-1873000

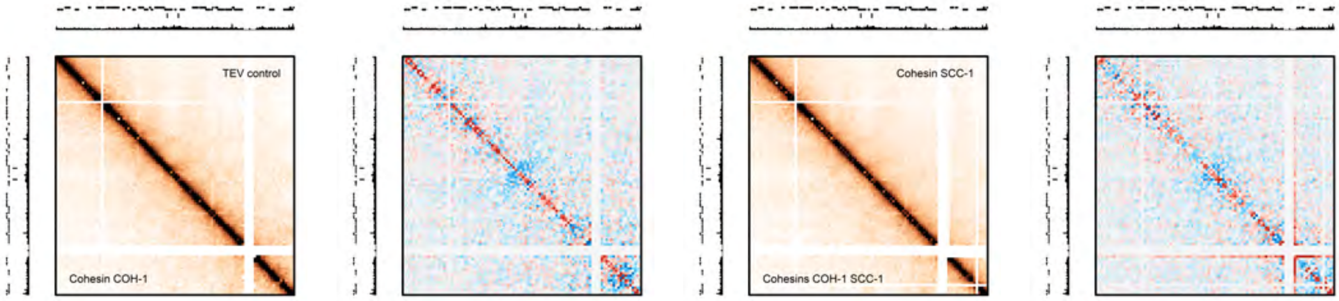

chrIII:4559001-4759000

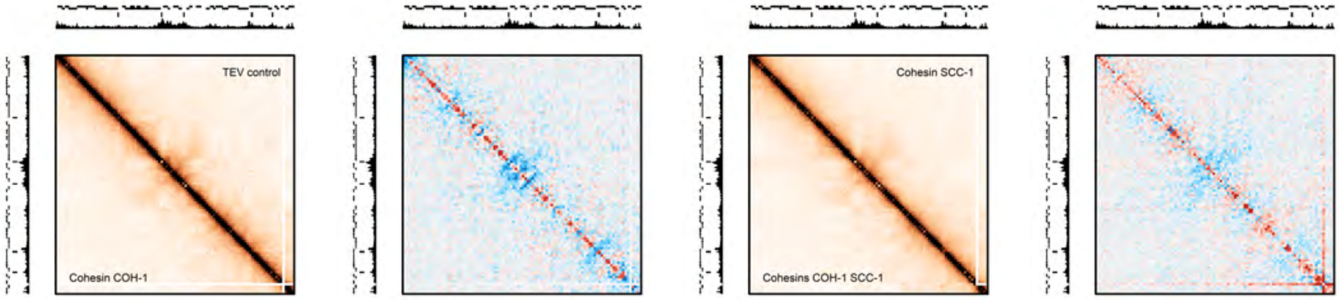

chrIV:5239001-5439000

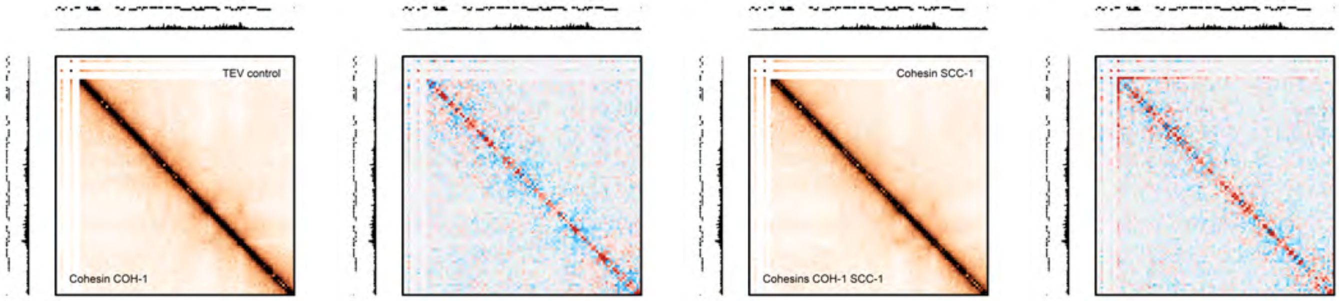

chrIV:855001-1055000

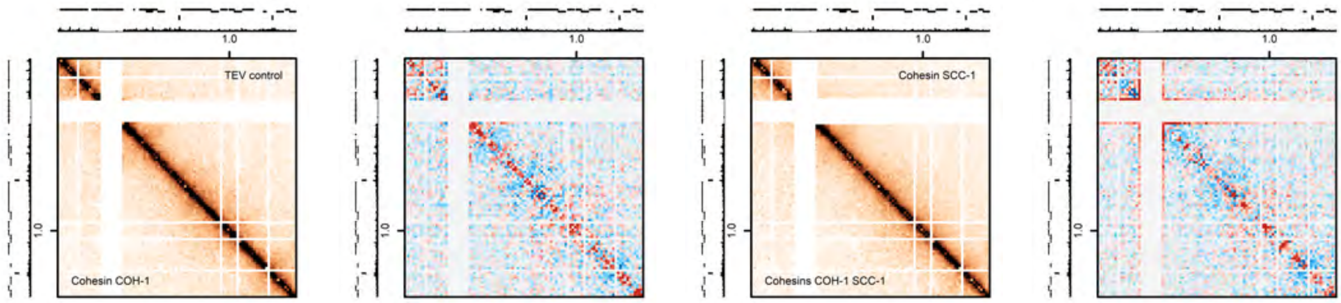

chrII:6611001-6811000

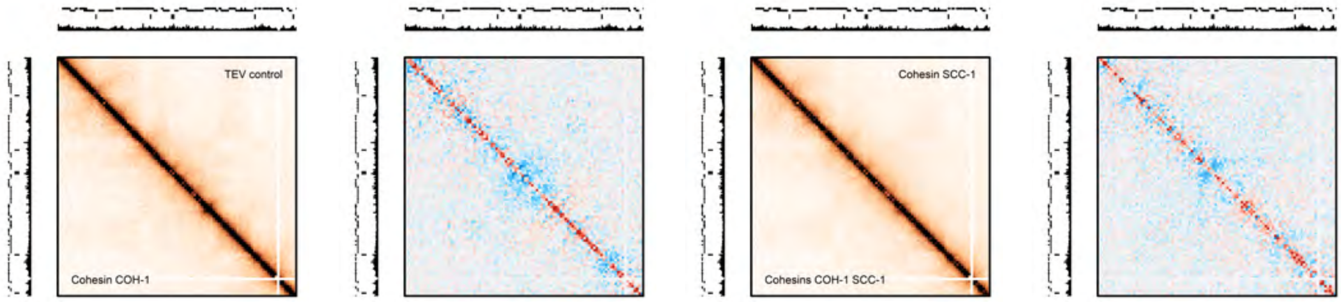

chrI:10307001-10507000

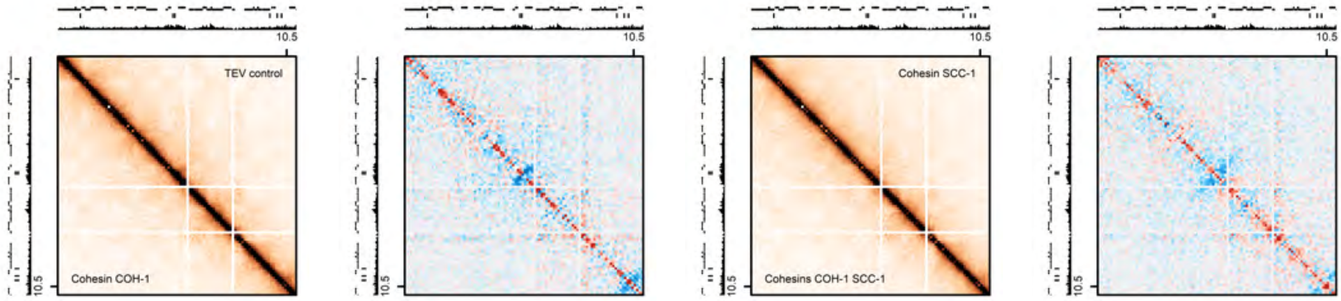

chrI:14425001-14625000

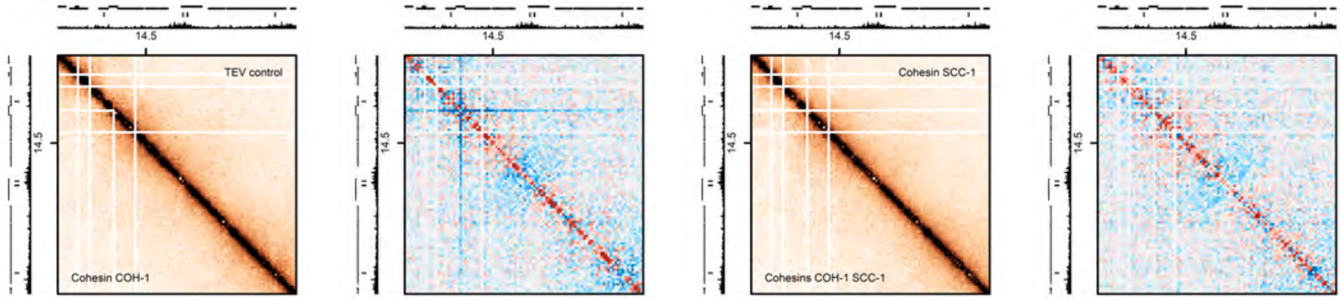

chrV:5457001-5657000

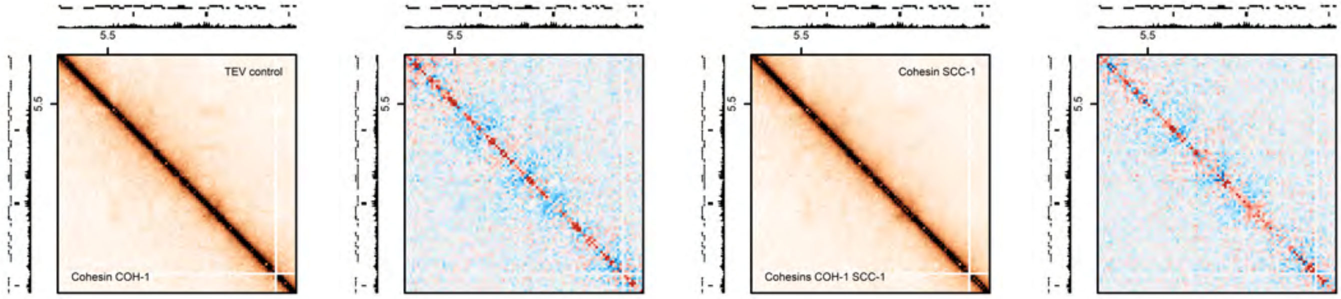

chrV:17023001-17223000

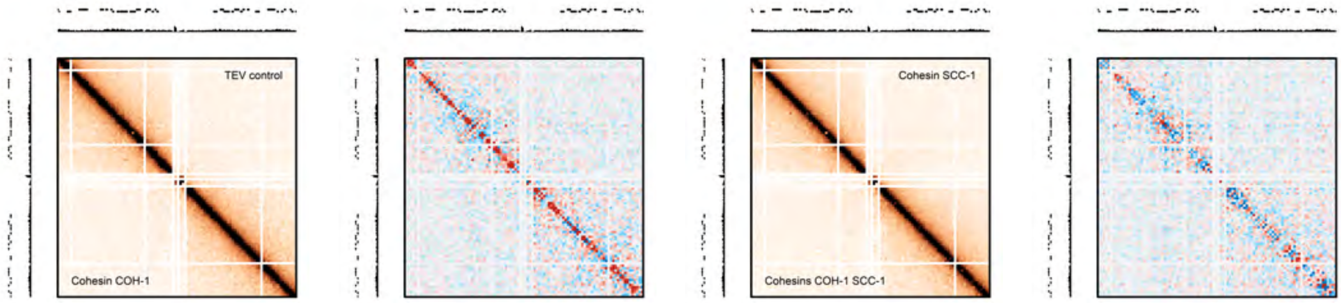

chrIII:12861001-13061000

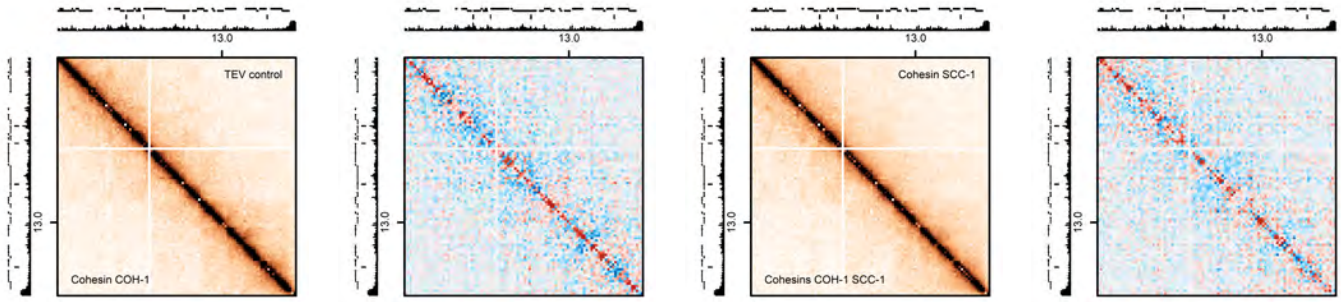

chrIV:1659001-1859000

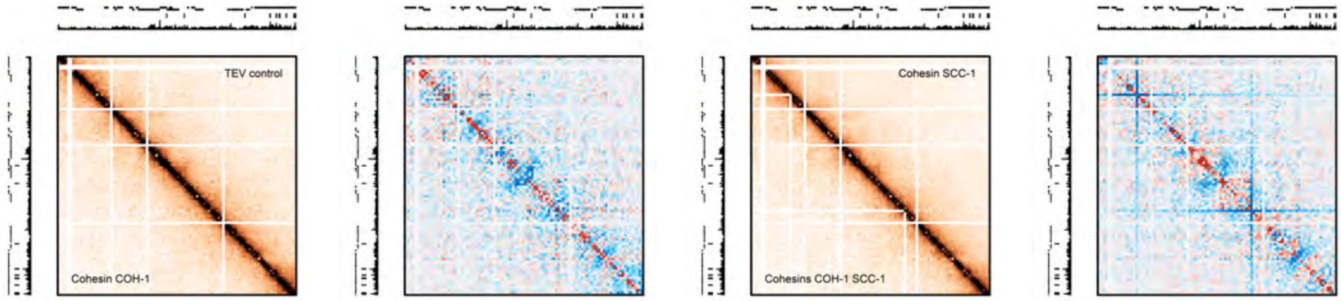

chrIV:3423001-3623000

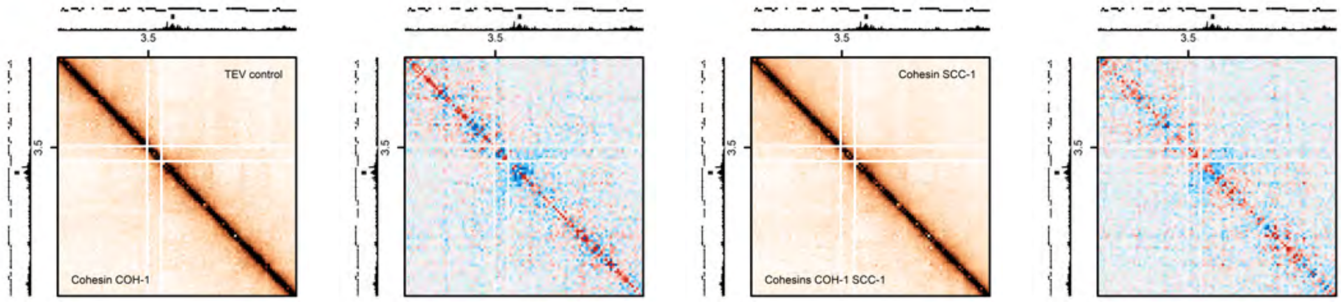

chrX:6707001-6907000

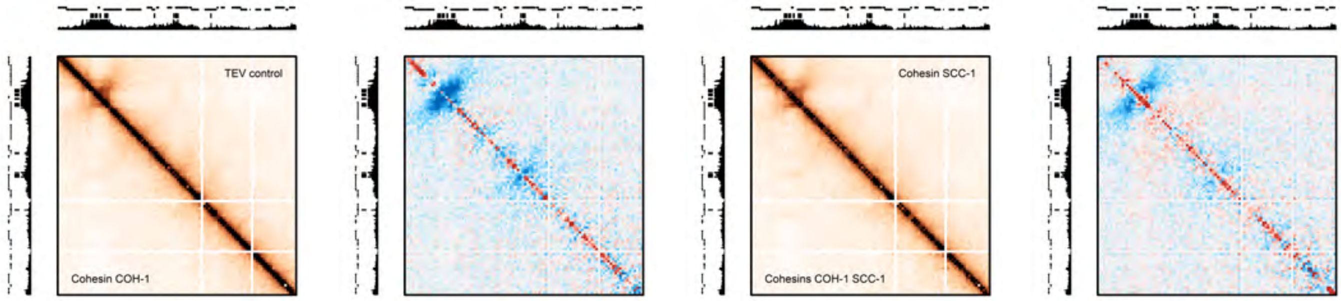

chrII:9017001-9217000

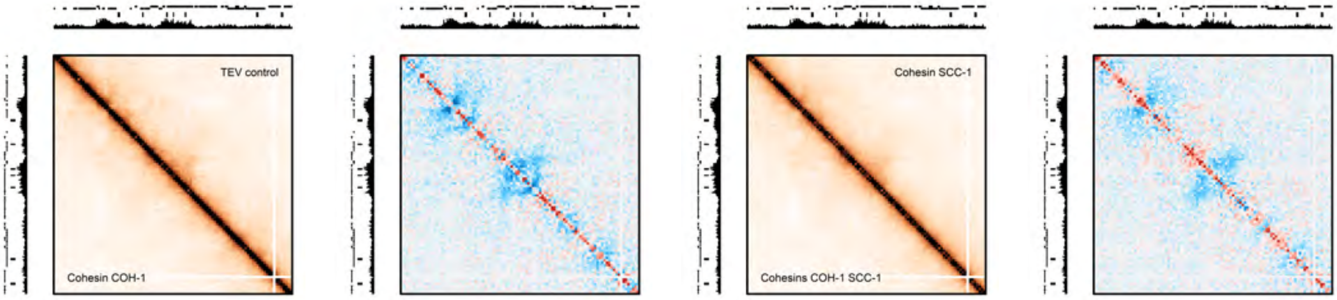

chrIV:12595001-12795000

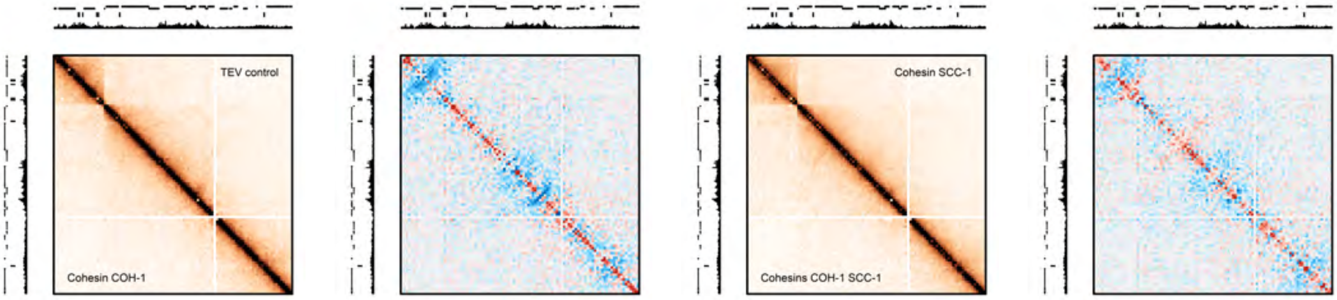

chrII:6841001-7041000

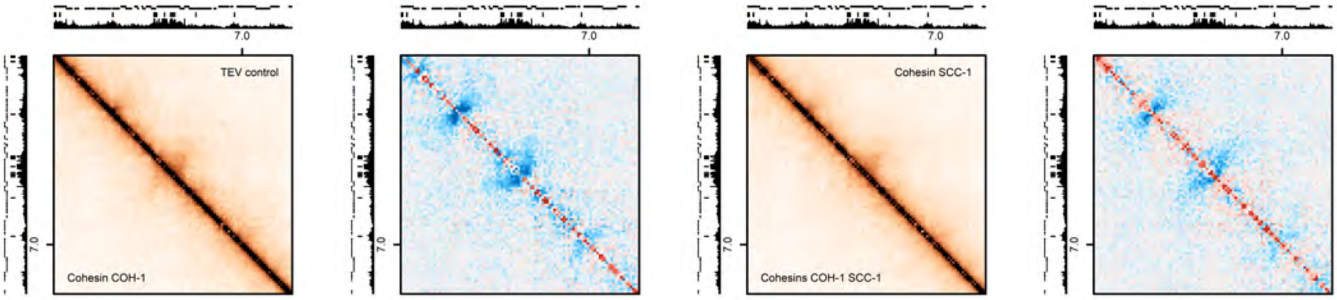

chrIV:3249001-3449000

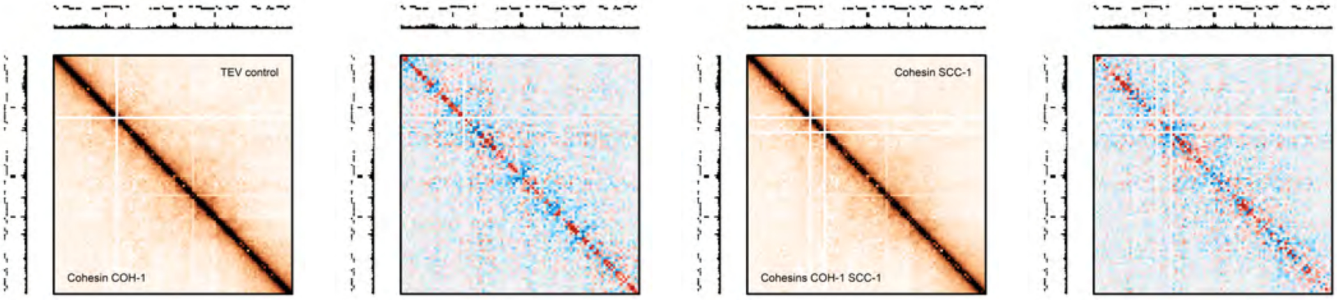

chrII:4053001-4253000

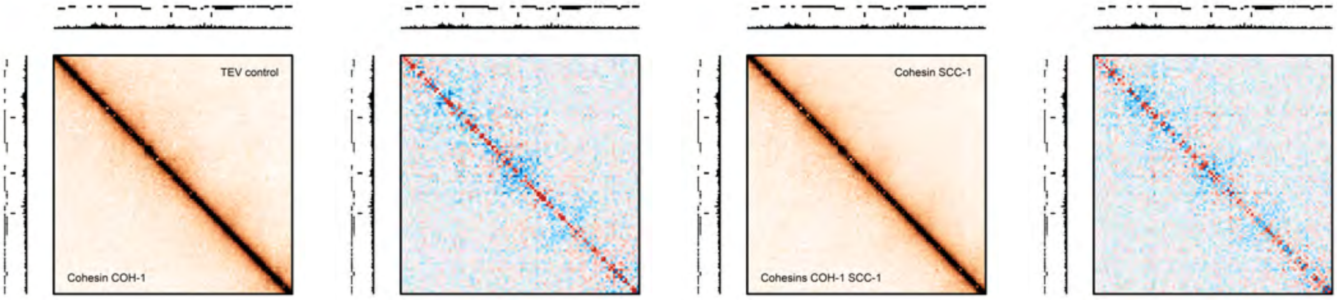

chrIV:13401001-13601000

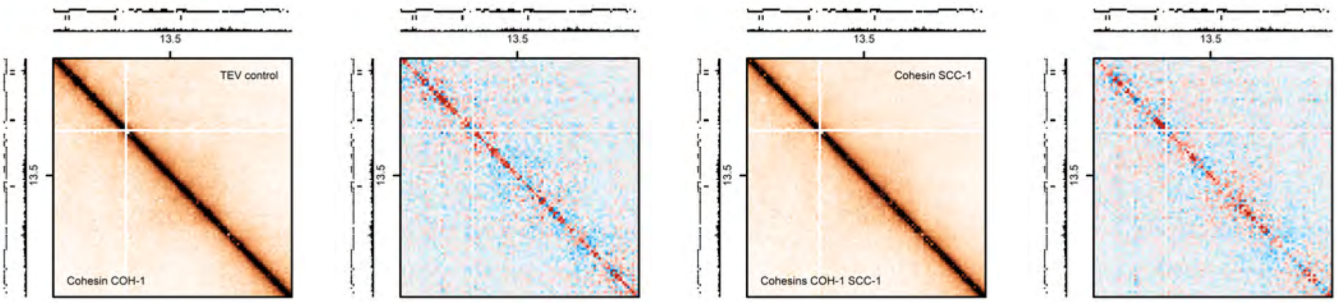

chrIII:12741001-12941000

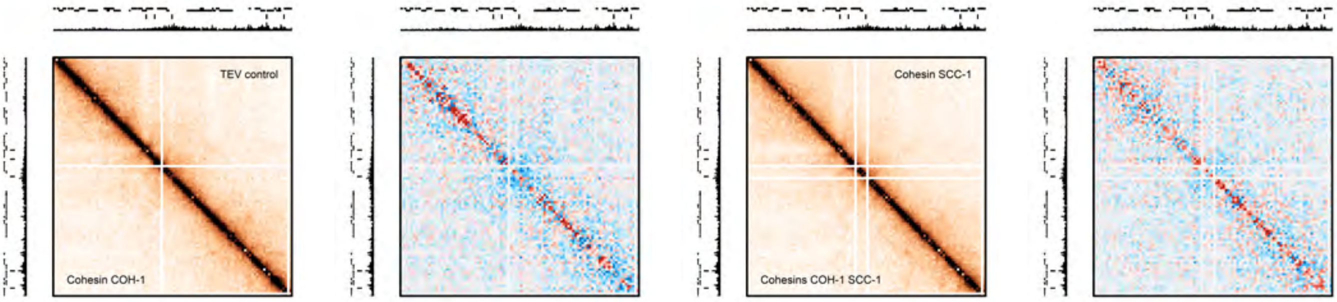

chrII:1851001-2051000

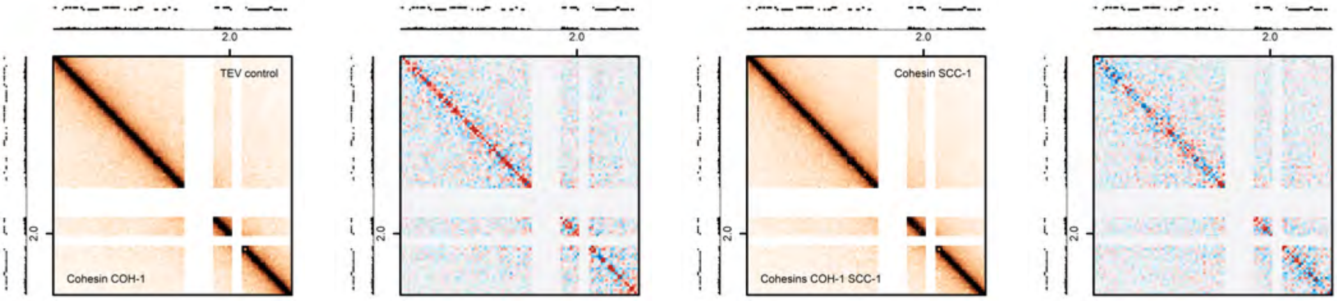

chrIV:14377001-14577000

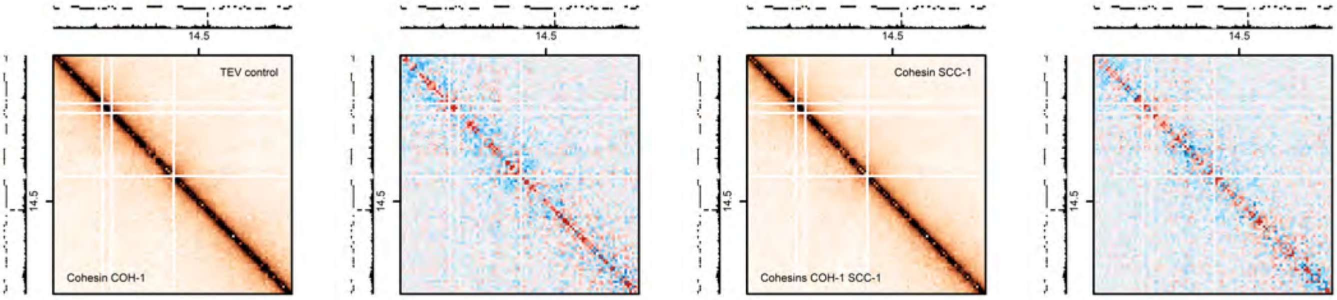

chrIV:11905001-12105000

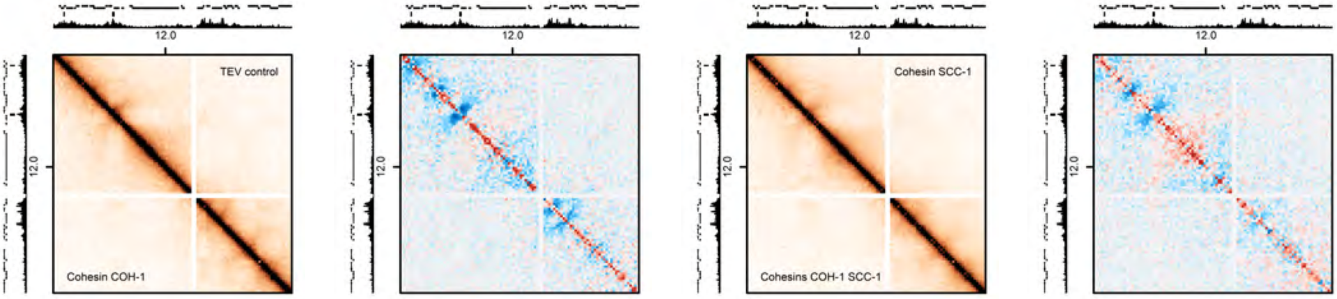

chrV:16813001-17013000

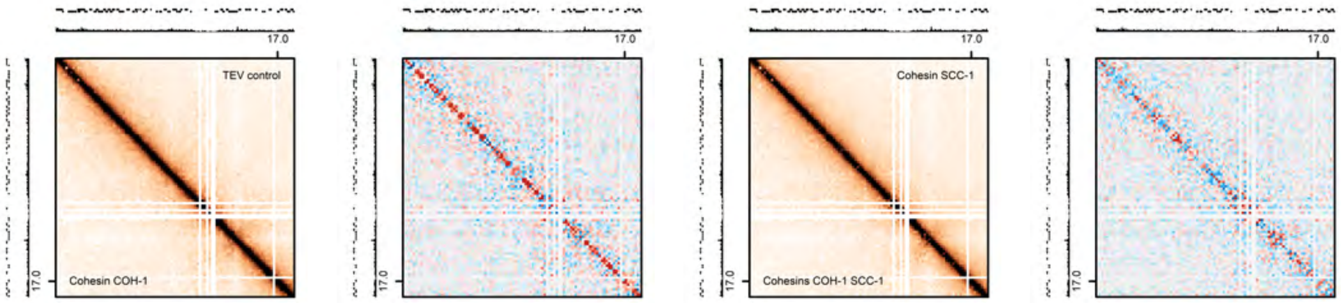

chrI:4335001-4535000

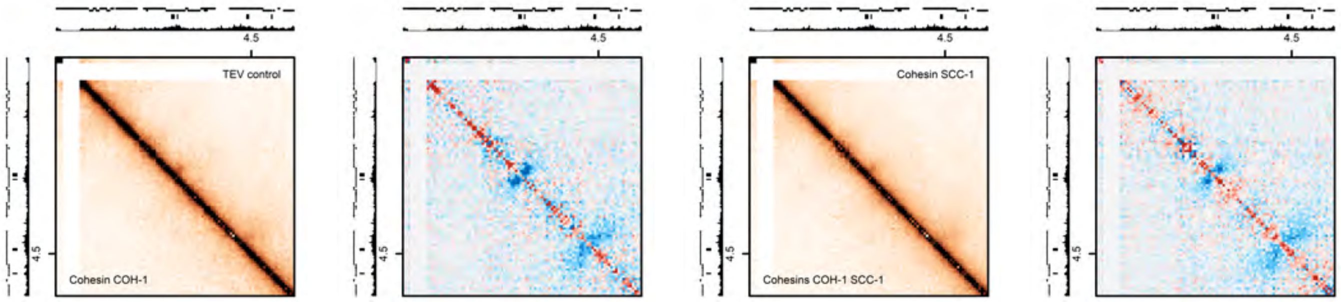

chrIII:8319001-8519000

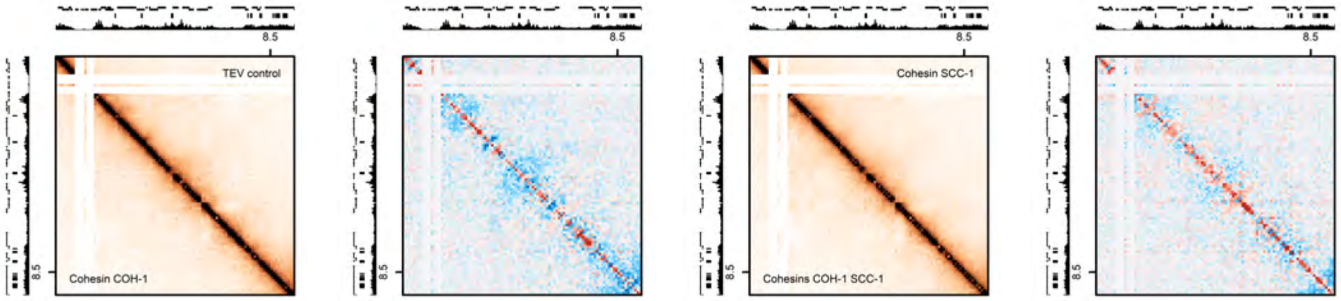

chrX:7487001-7687000

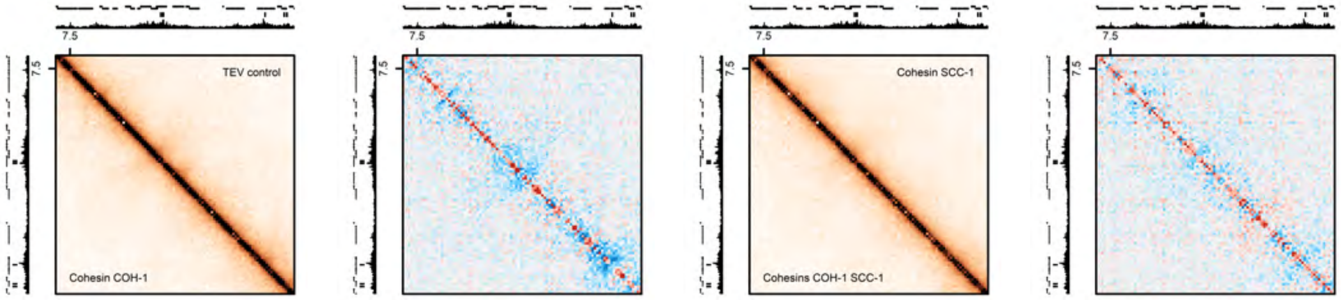

chrII:4921001-5121000

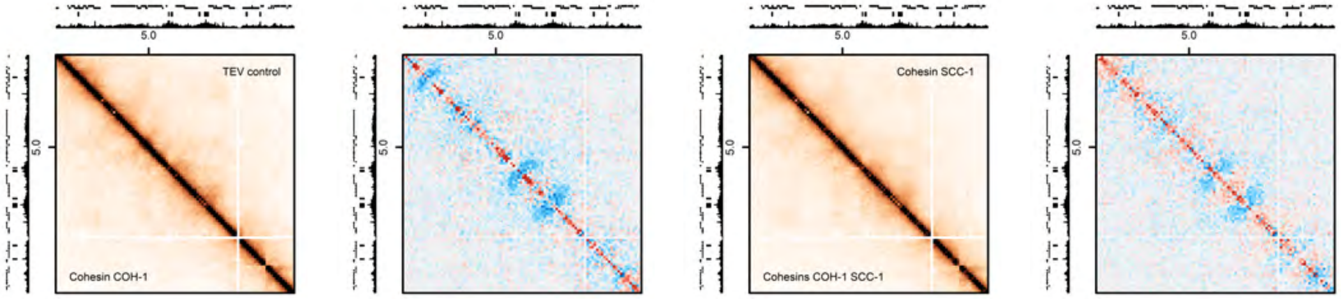

chrX:8835001-9035000

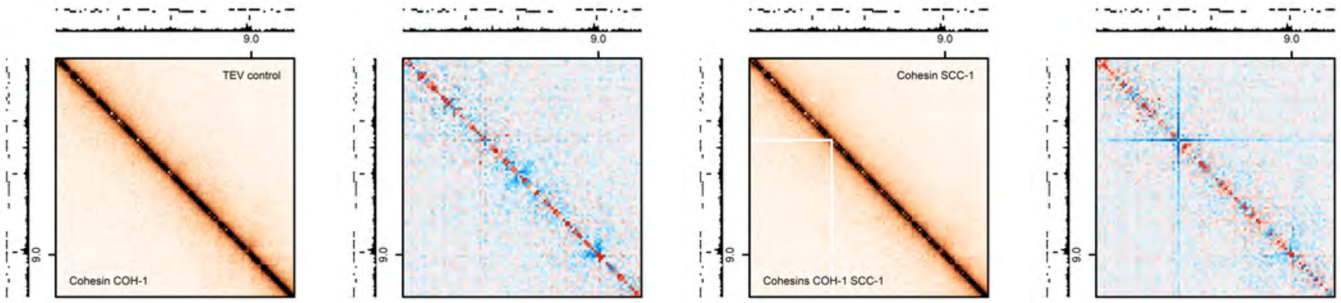

chrI:897001-1097000

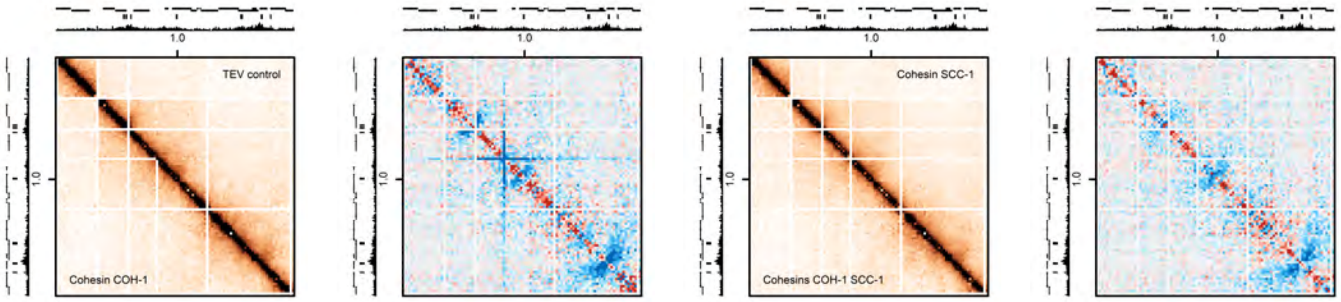

chrIV:11853001-12053000

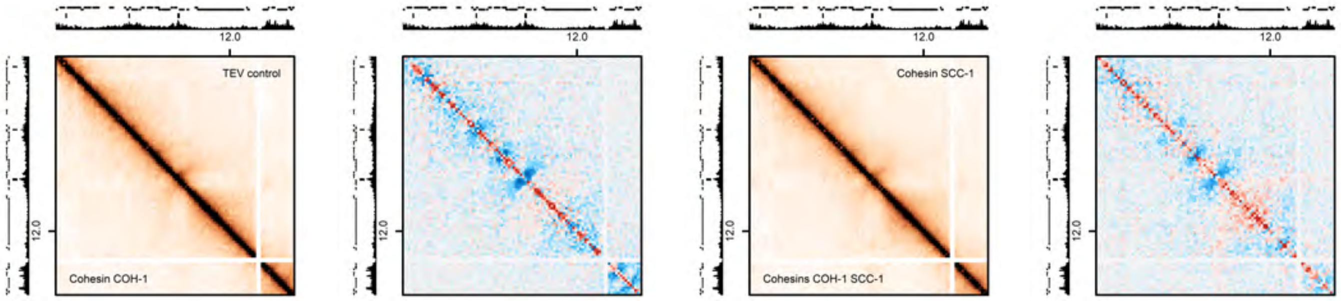

chrII:1243001-1443000

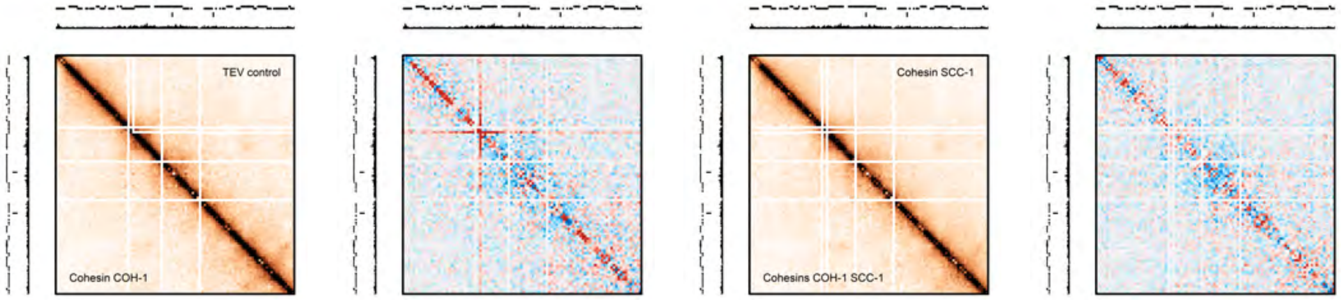

chrX:9903001-10103000

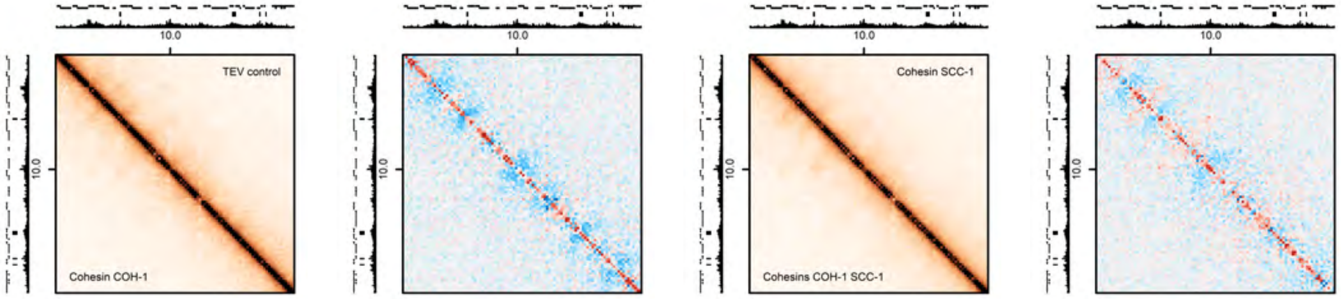

chrI:7445001-7645000

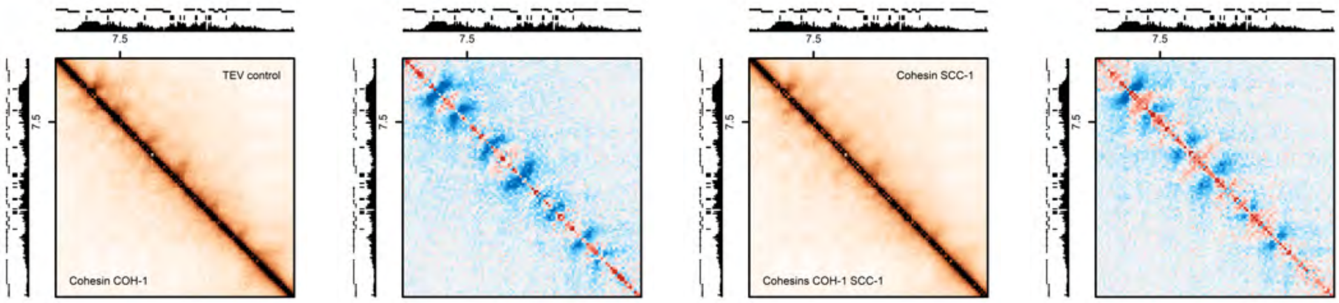

chrIII:9521001-9721000

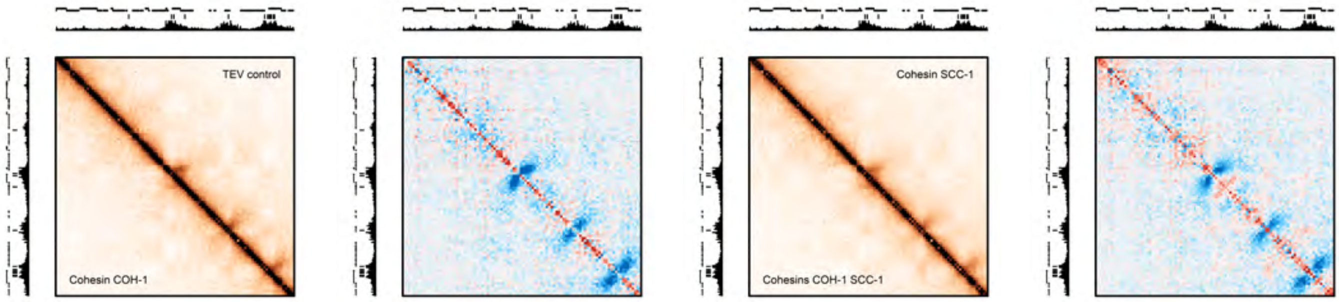

chrII:2241001-2441000

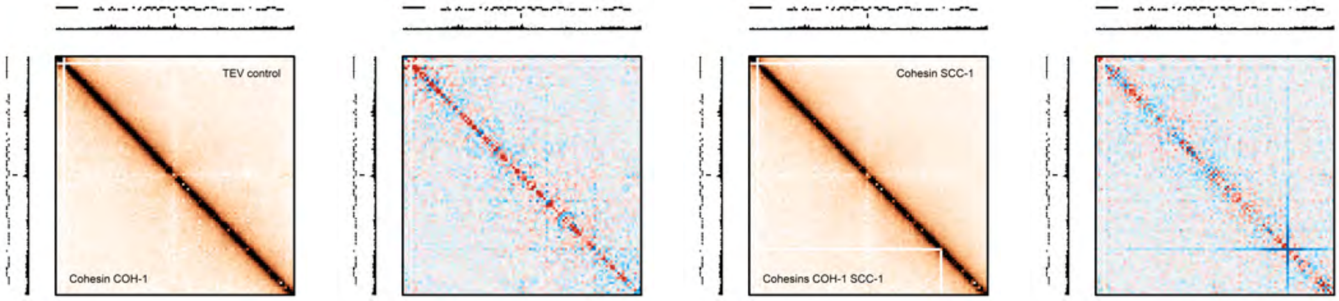

chrIV:14175001-14375000

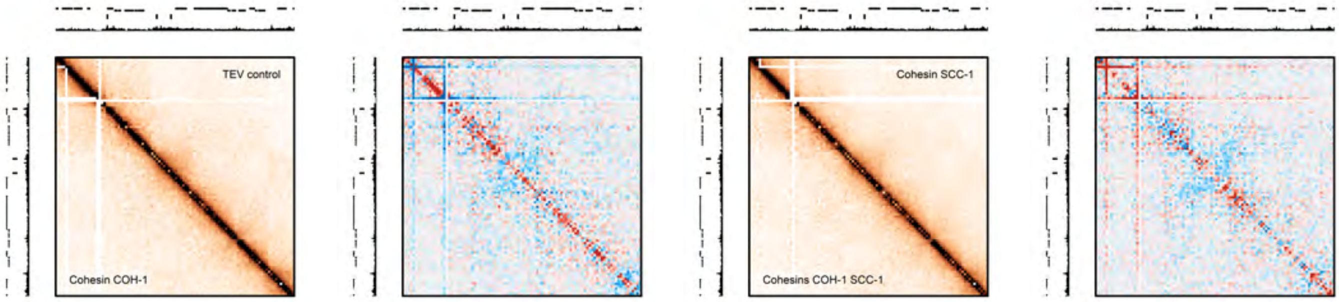

chrV:20169001-20369000

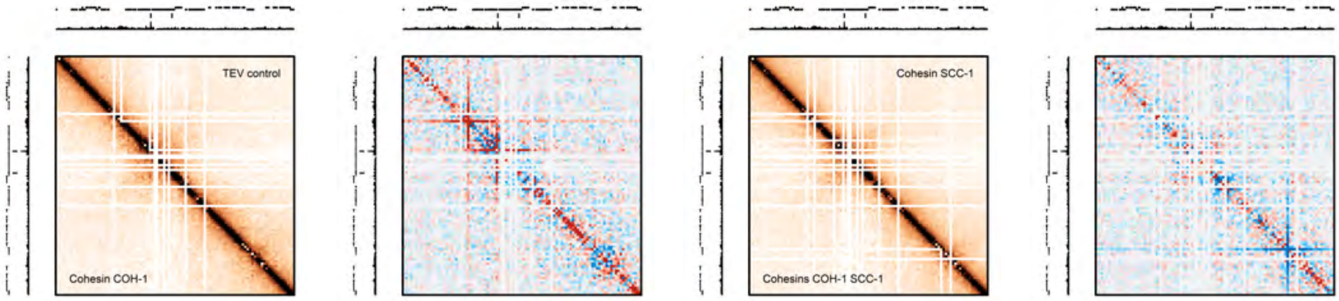

chrIII:13533001-13733000

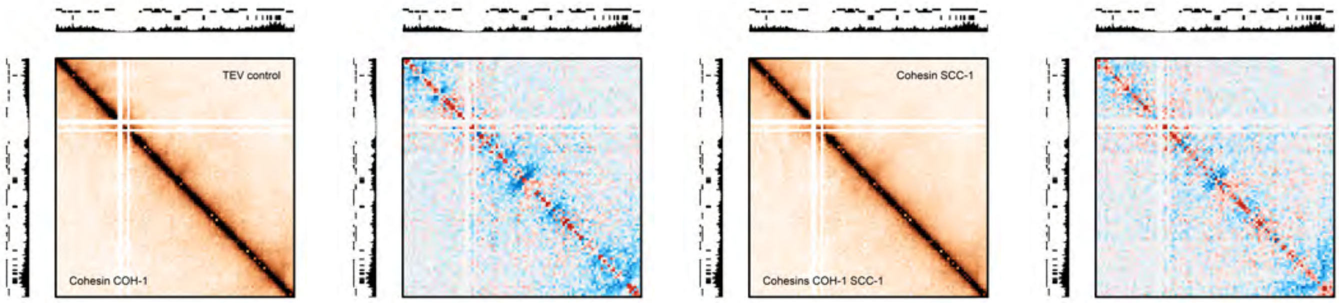

chrII:10197001-10397000

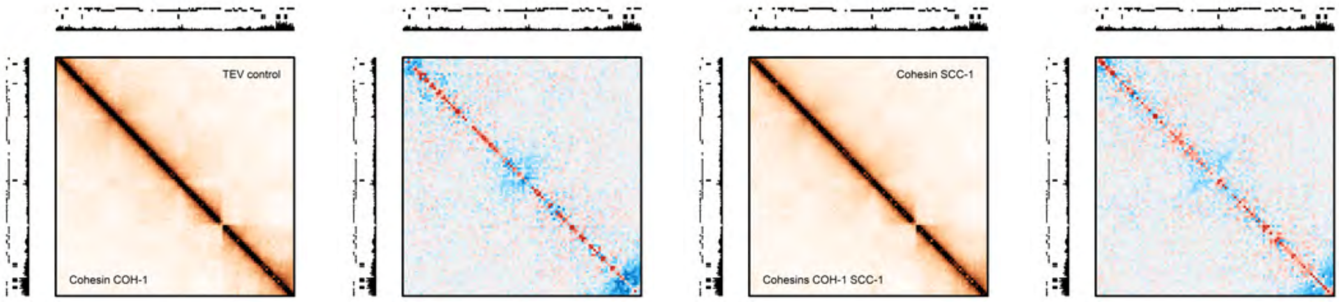

chrI:4079001-4279000

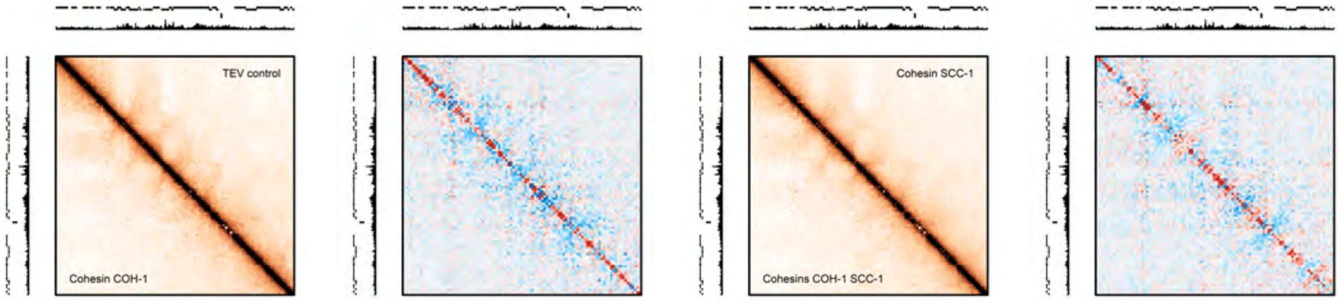

chrI:7117001-7317000

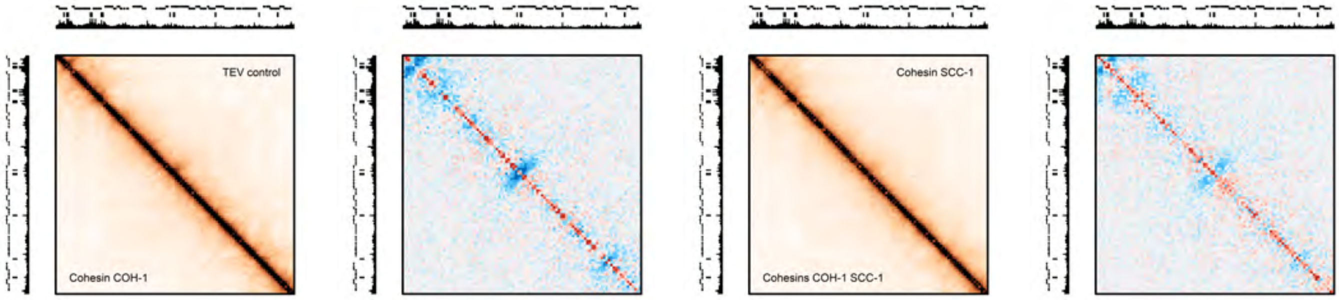

chrV:19251001-19451000

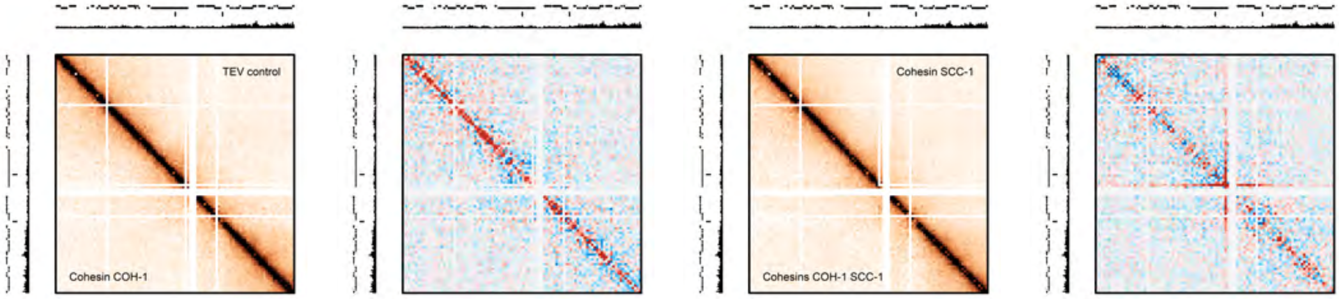

chrV:12537001-12737000

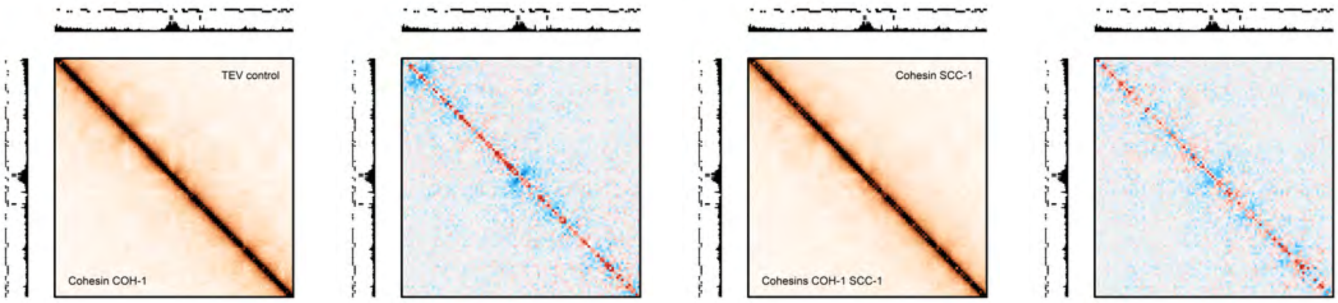

chrI:9937001-10137000

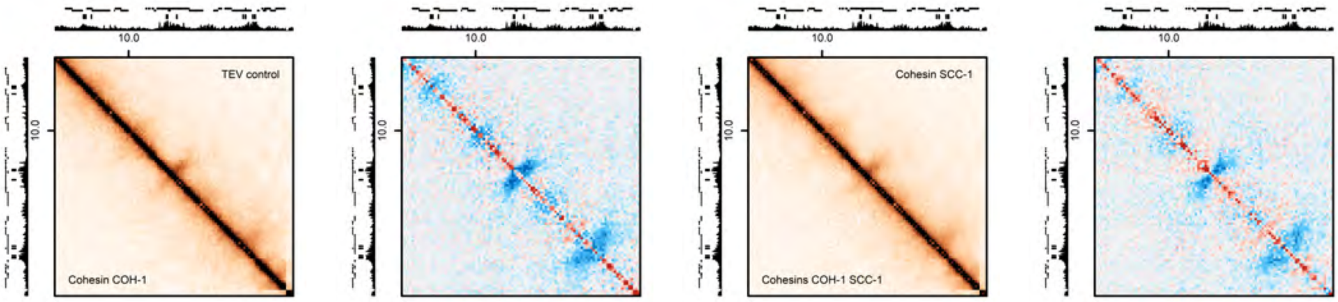

chrIV:16637001-16837000

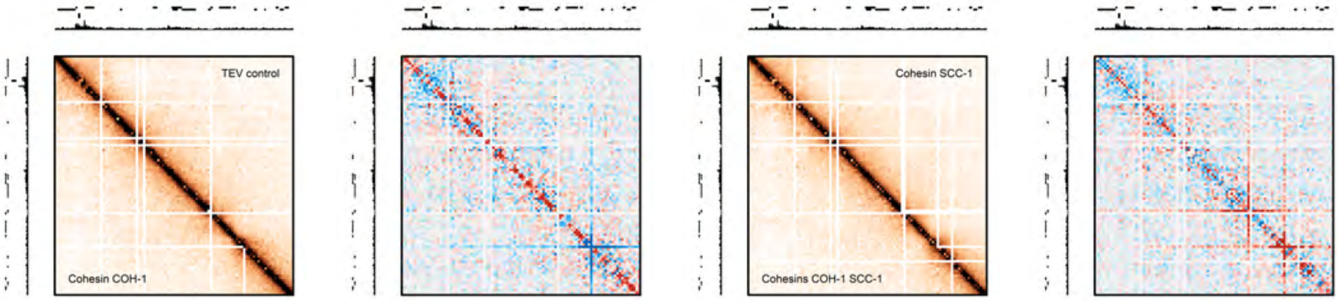

chrX:15941001-16141000

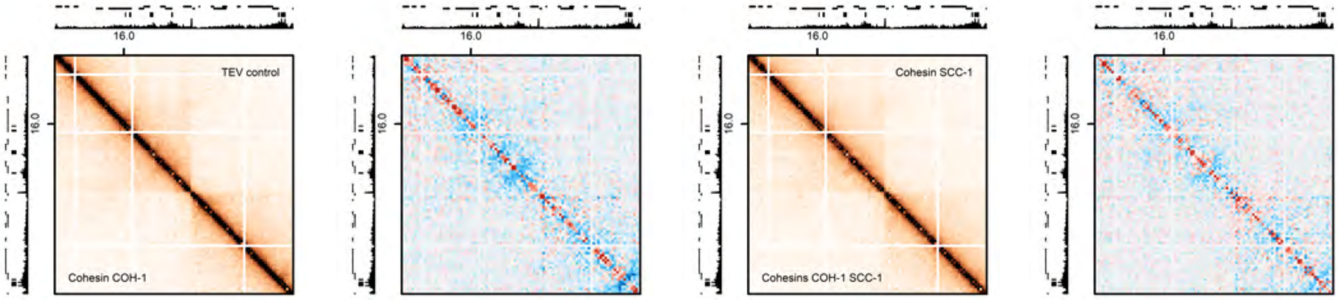

chrX:9539001-9739000

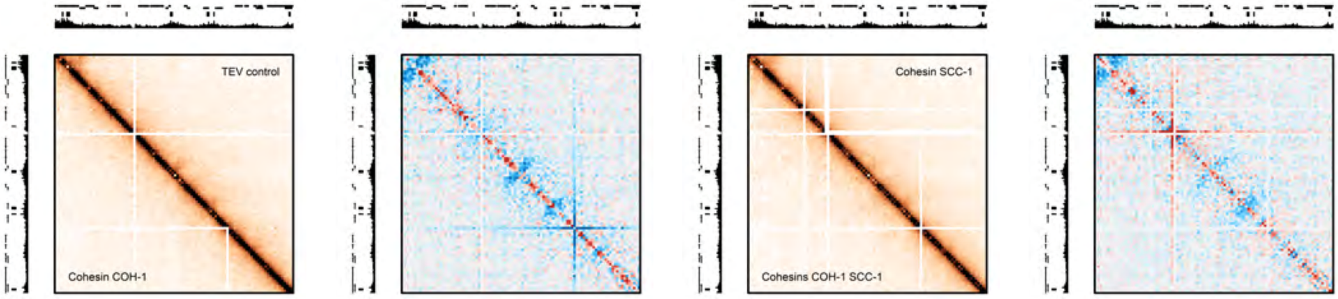

chrIII:6549001-6749000

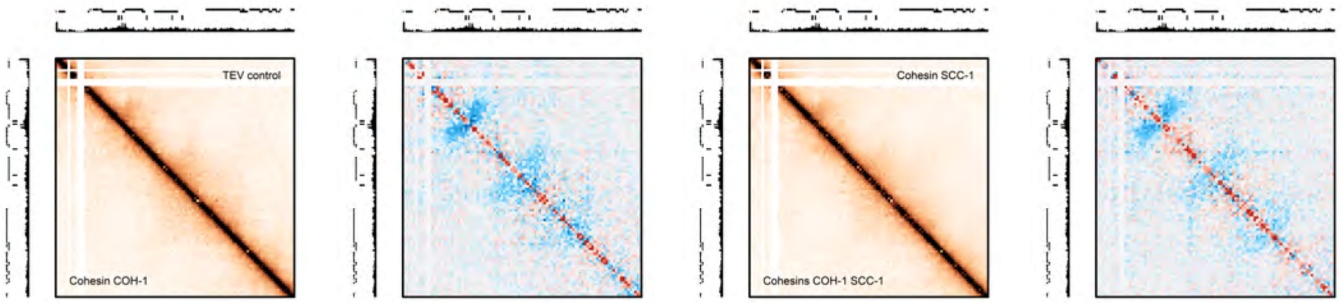

chrIV:7447001-7647000

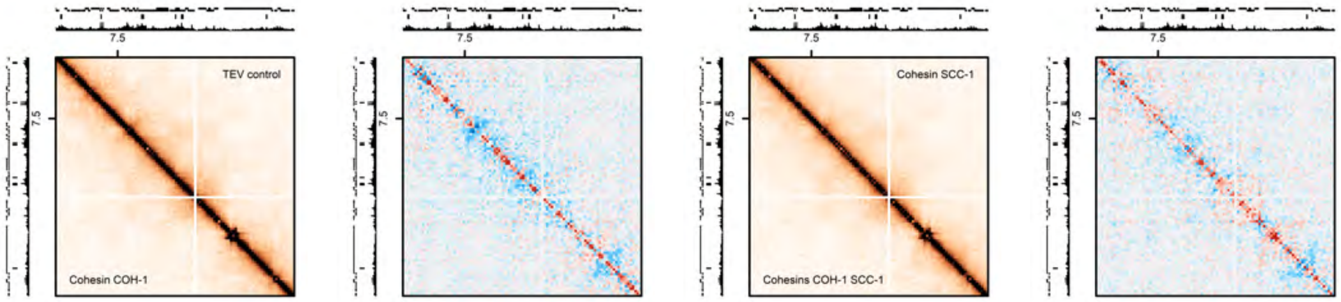

chrIII:955001-1155000

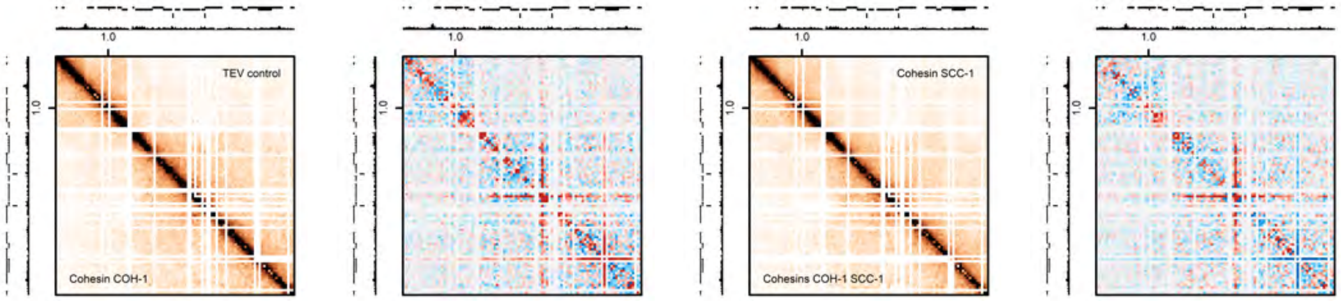

chrI:6631001-6831000

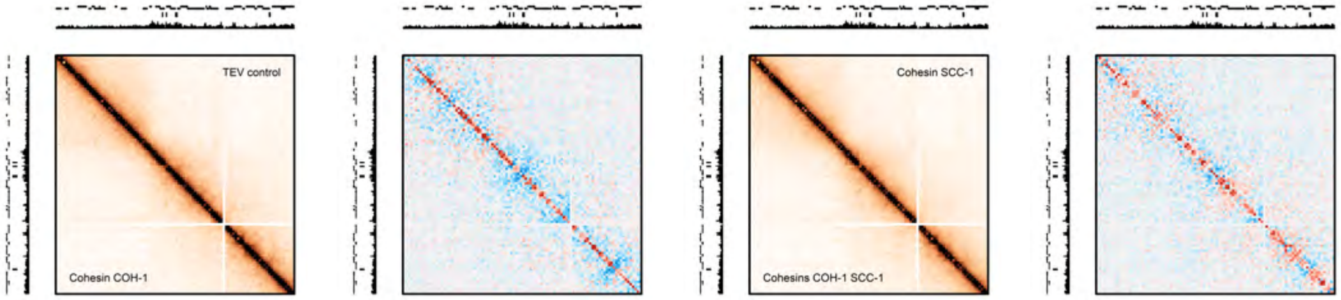

chrV:4865001-5065000

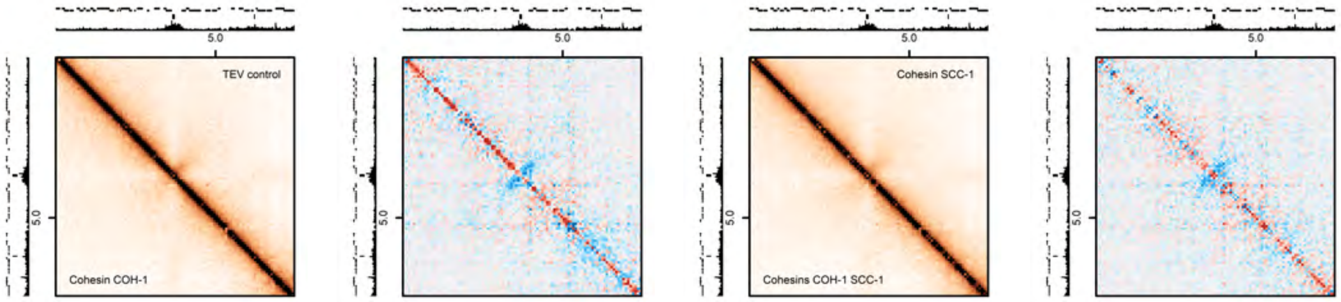

chrIV:17371001-17571000

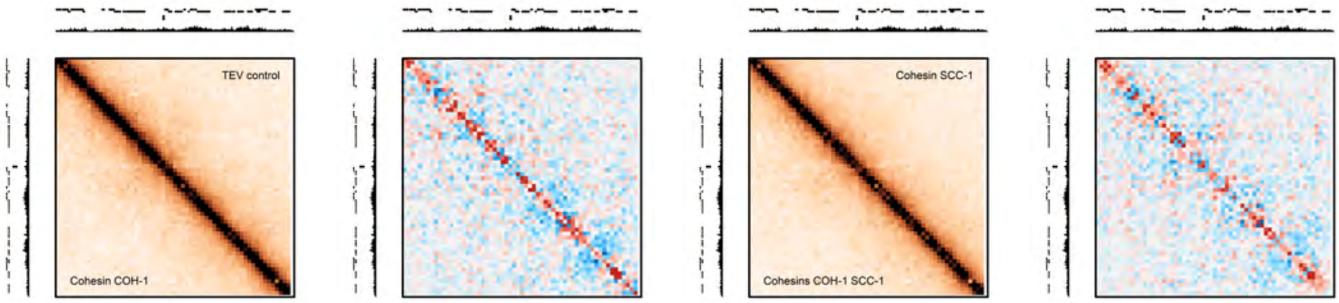

chrIII:3279001-3479000

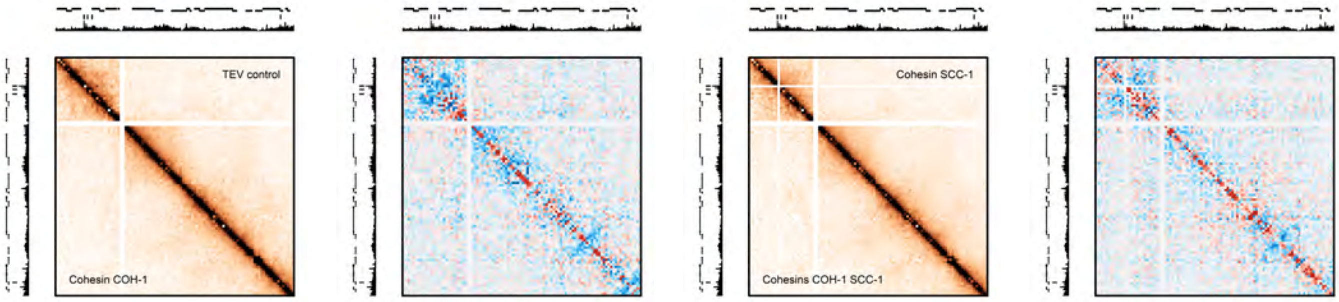

chrV:13807001-14007000

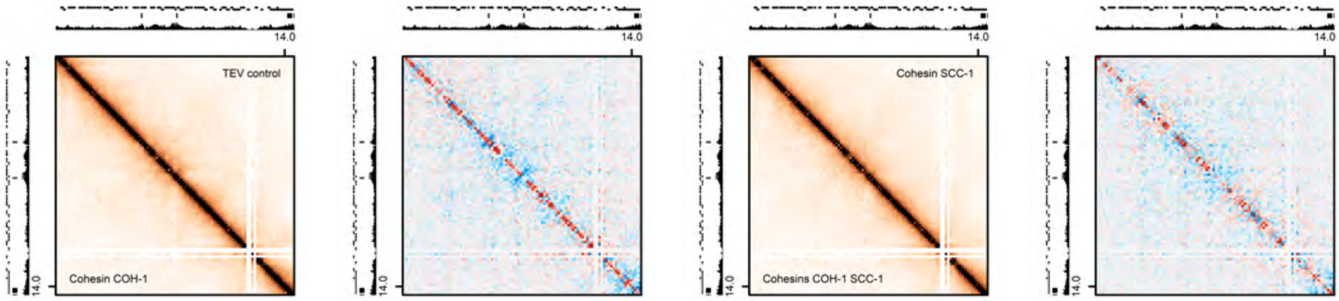

chrI:9241001-9441000

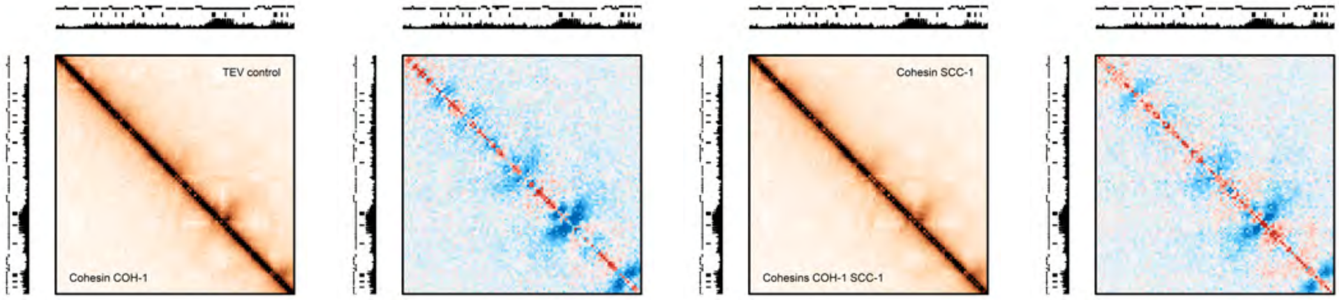

chrV:10843001-11043000

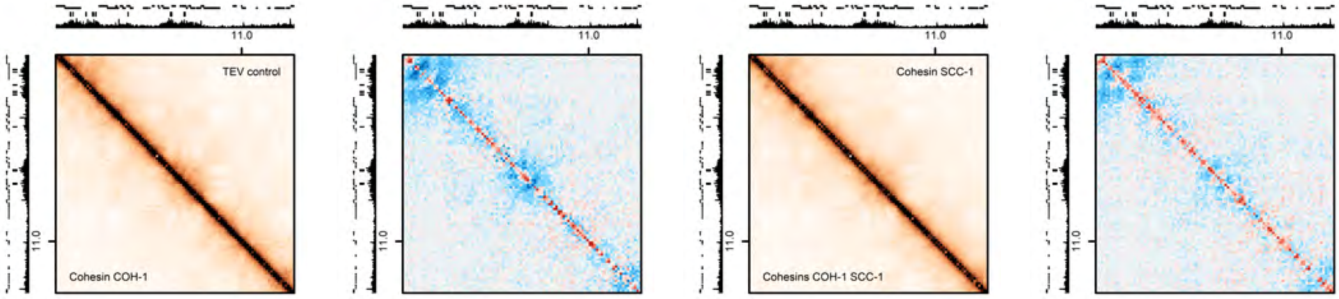

chrIII:3027001-3227000

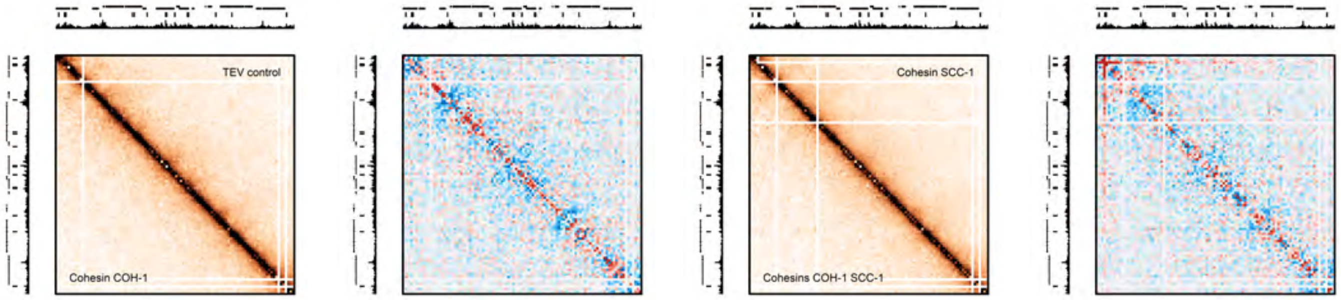

chrI:65001-265000

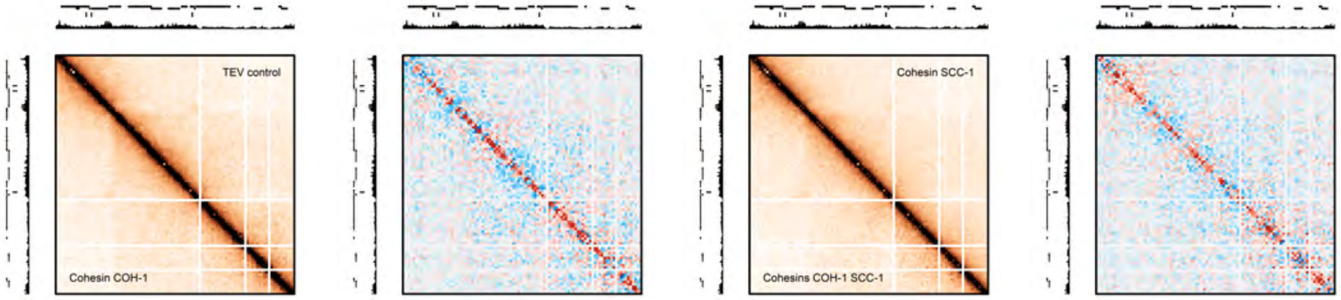

chrIV:1117001-1317000

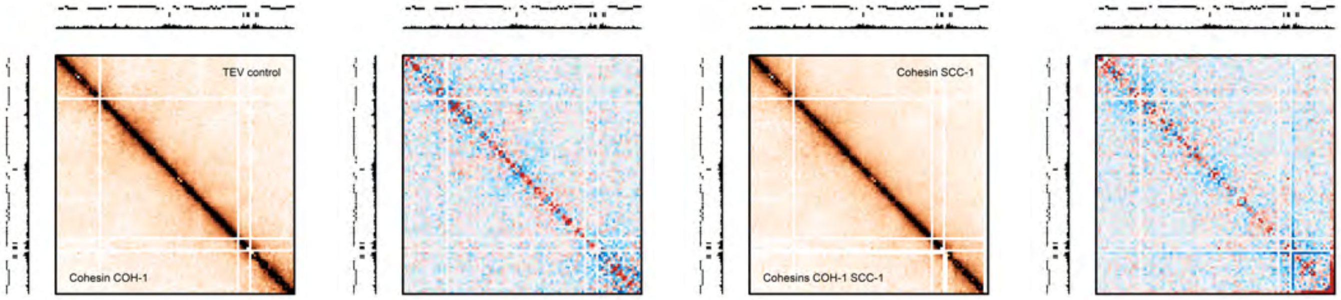

chrIII:787001-987000

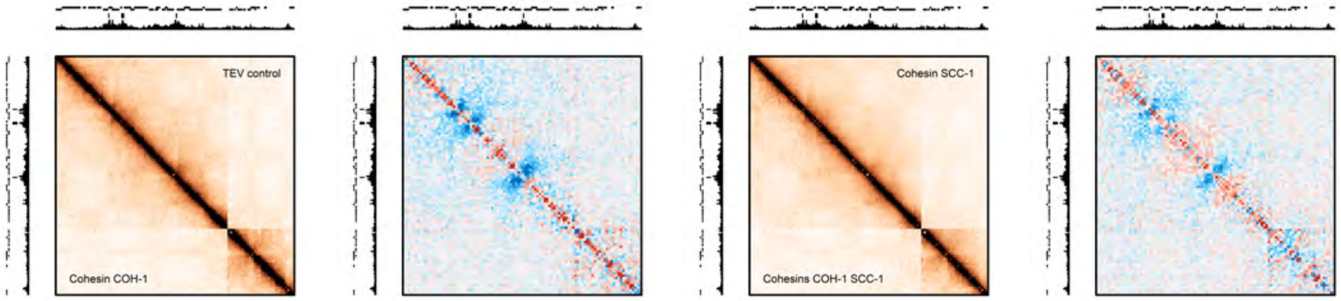

chrV:12797001-12997000

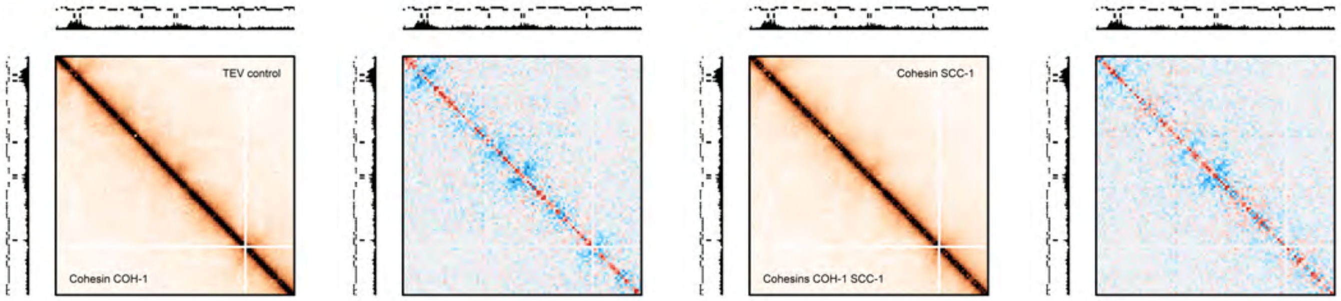

chrX:-56999-143000

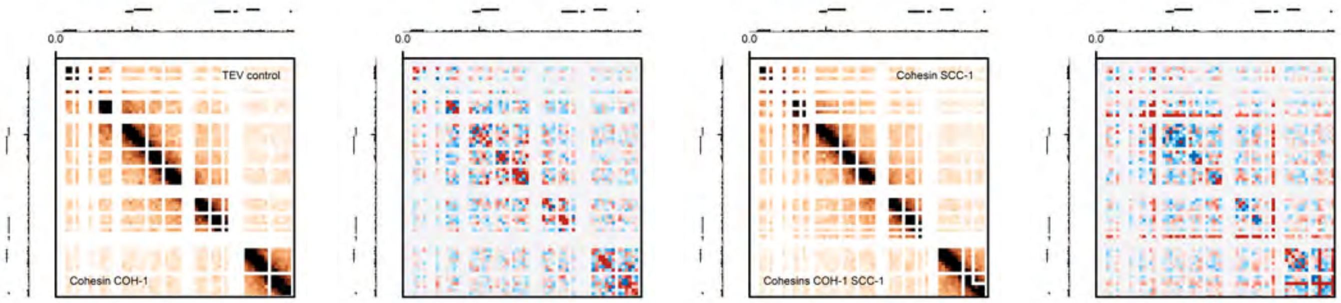

chrII:7677001-7877000

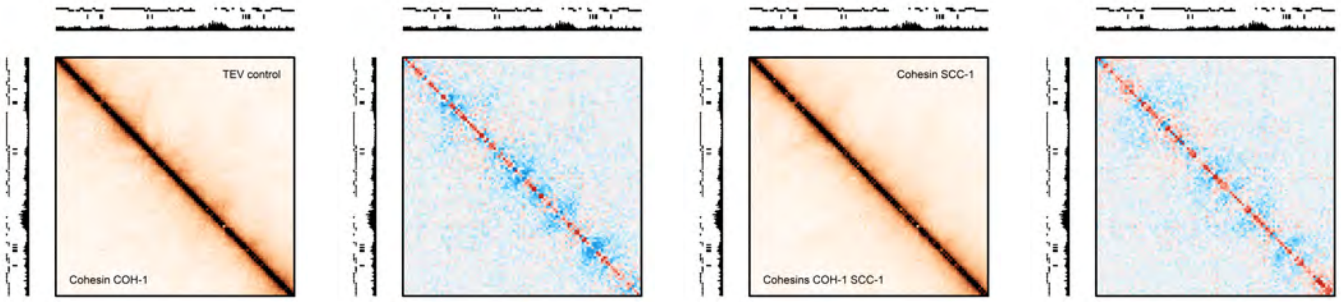

chrI:4685001-4885000

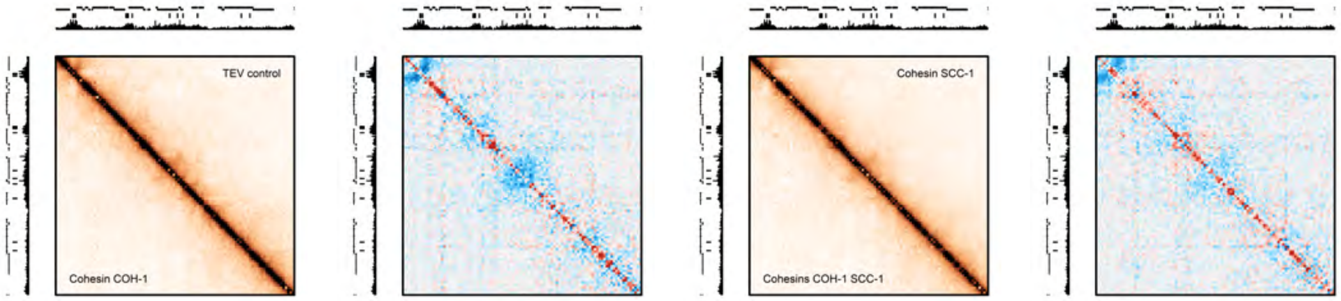

chrV:5595001-5795000

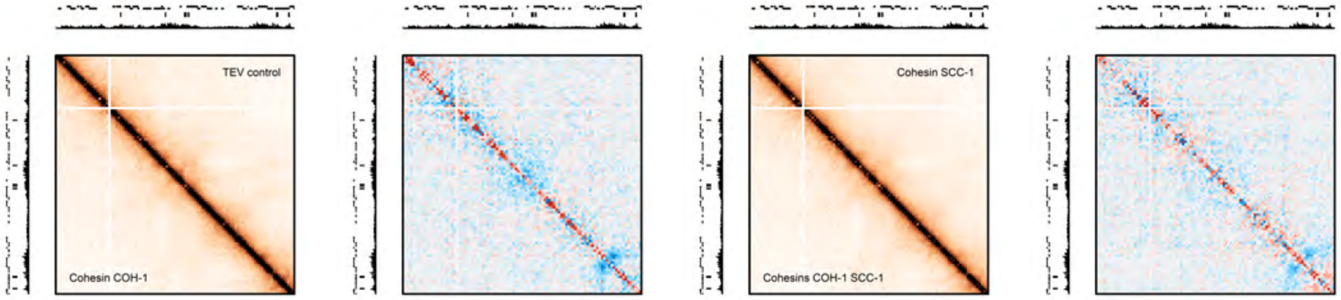

chrV:109001-309000

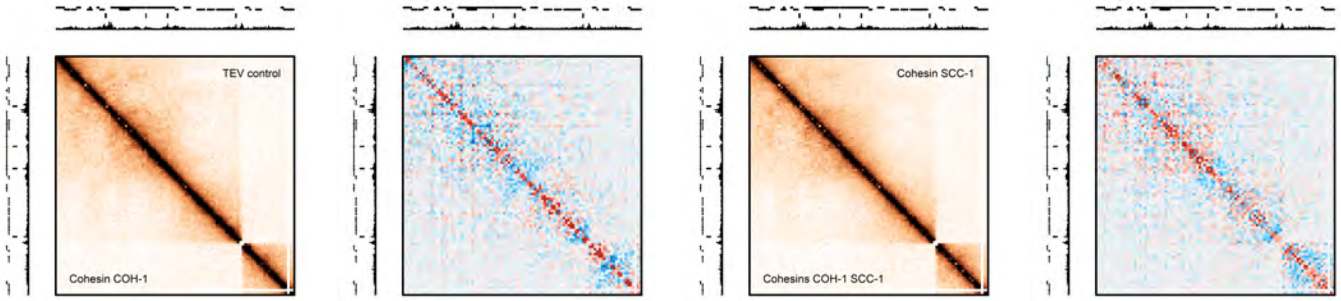

chrIV:2607001-2807000

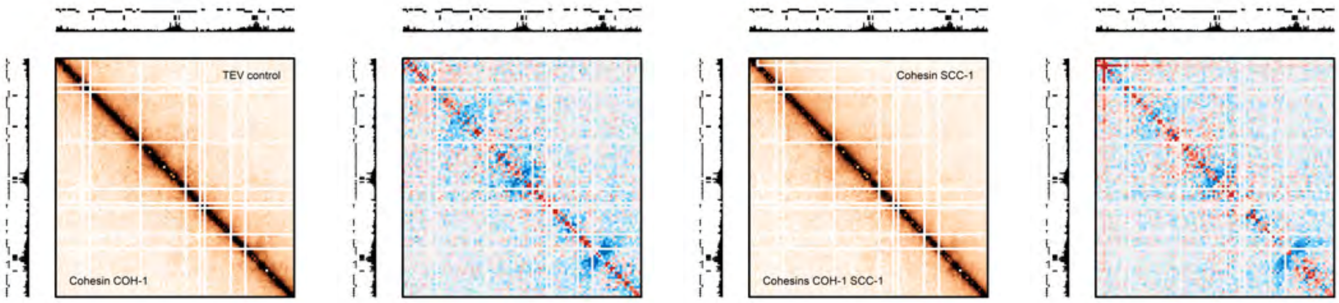

chrII:2627001-2827000

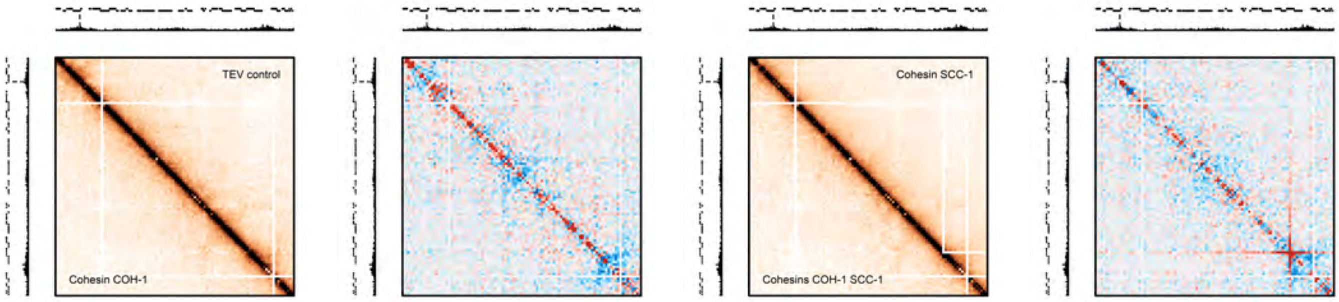

chrIII:3373001-3573000

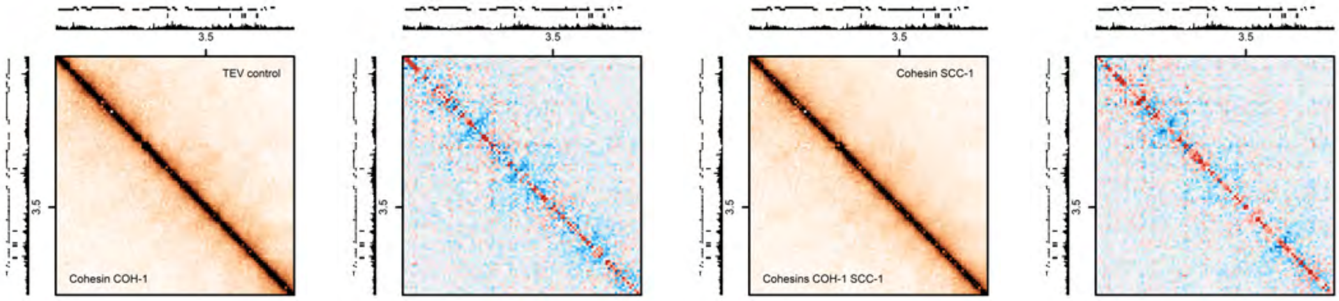

chrI:10077001-10277000

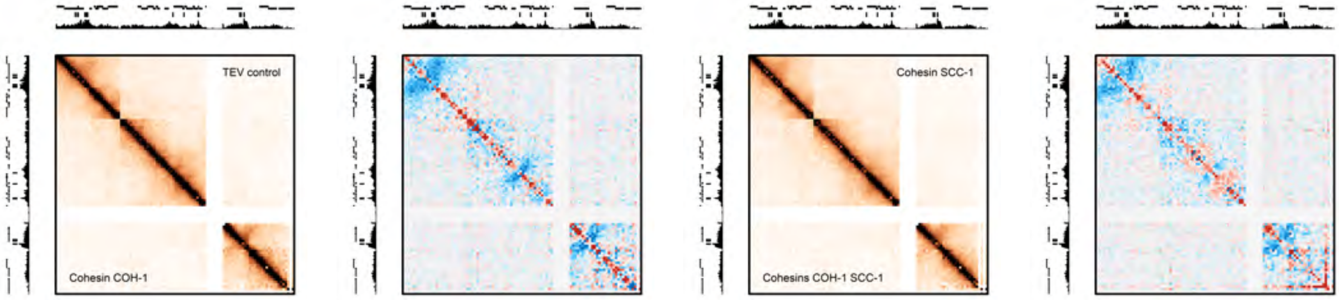

chrX:14049001-14249000

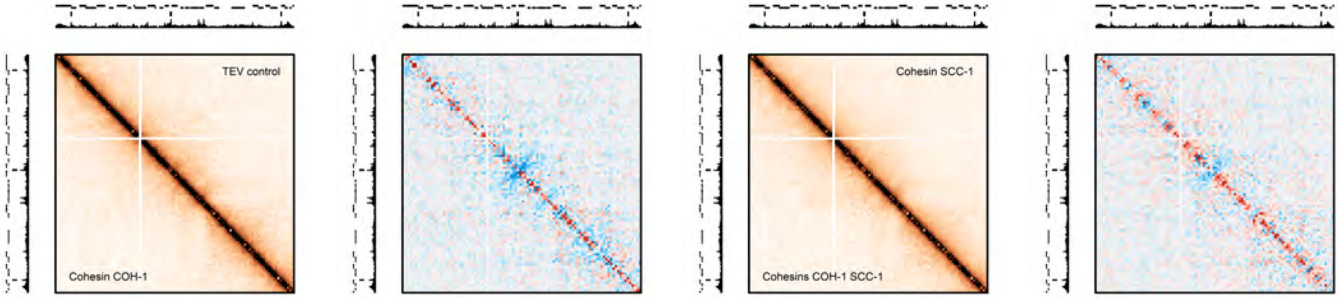

chrX:16633001-16833000

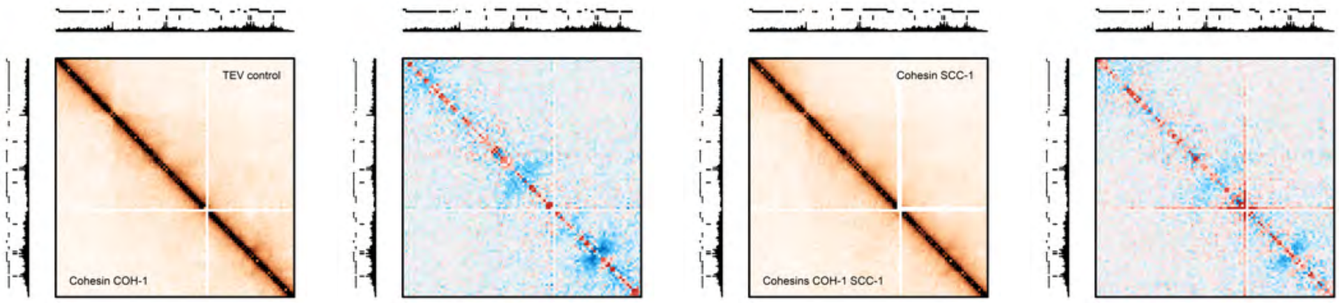

chrV:7115001-7315000

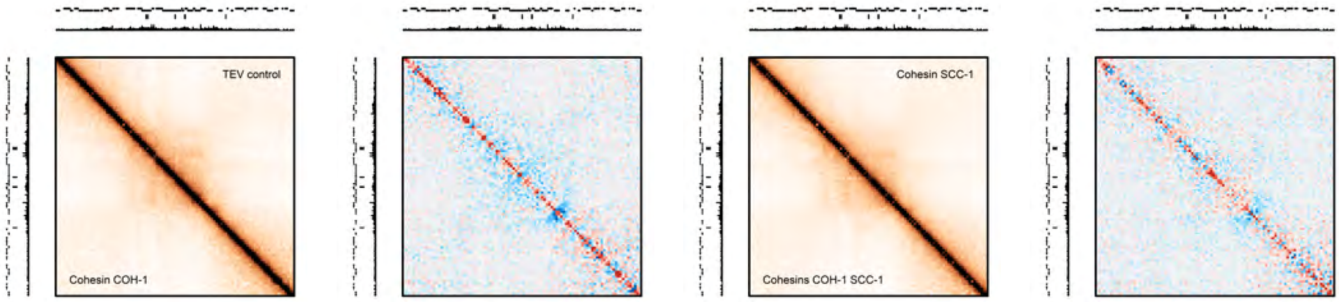

chrII:9631001-9831000

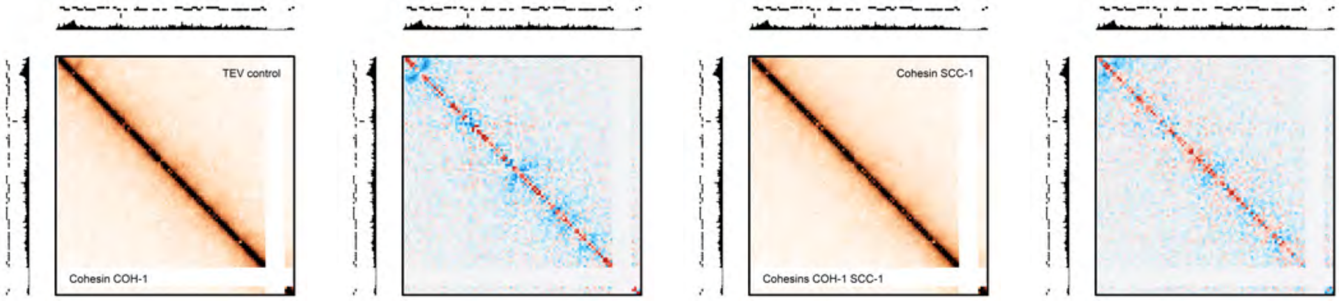

chrII:6789001-6989000

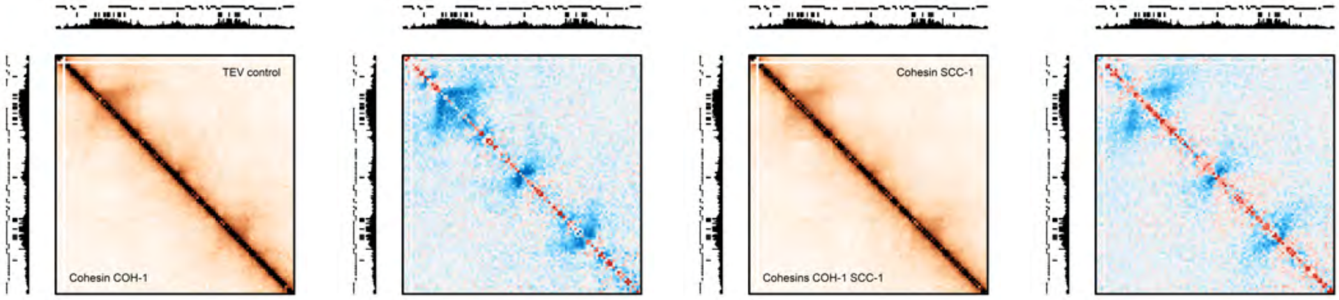

chrV:2805001-3005000

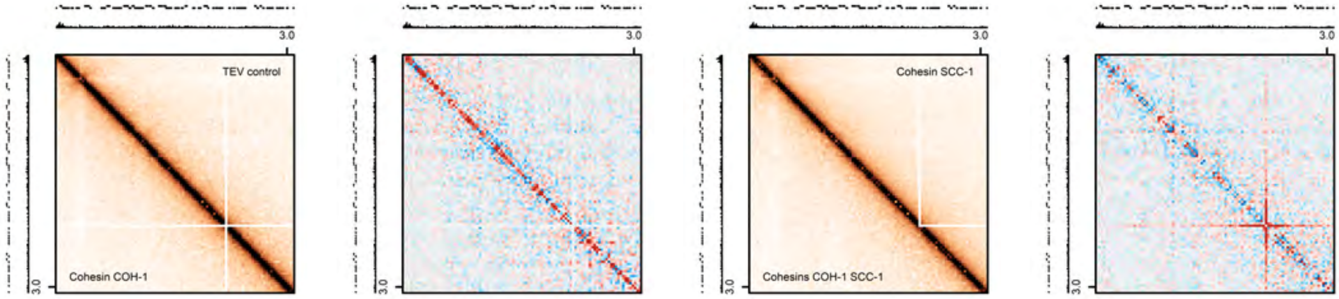

chrIII:5403001-5603000

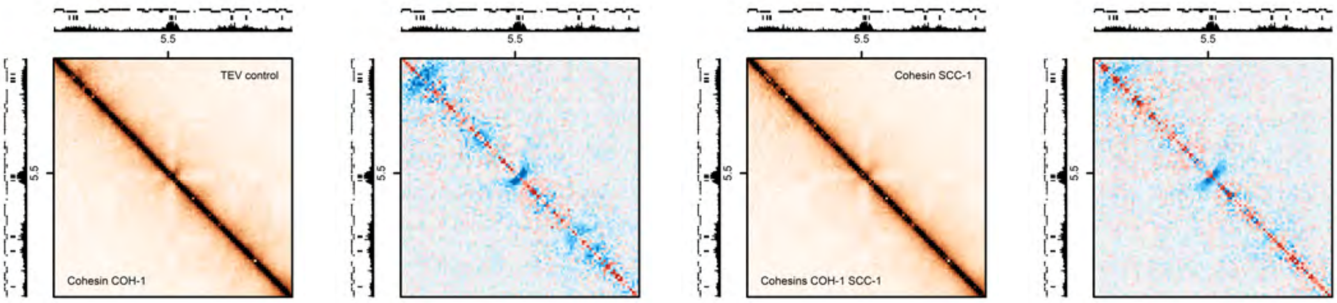

chrII:11875001-12075000

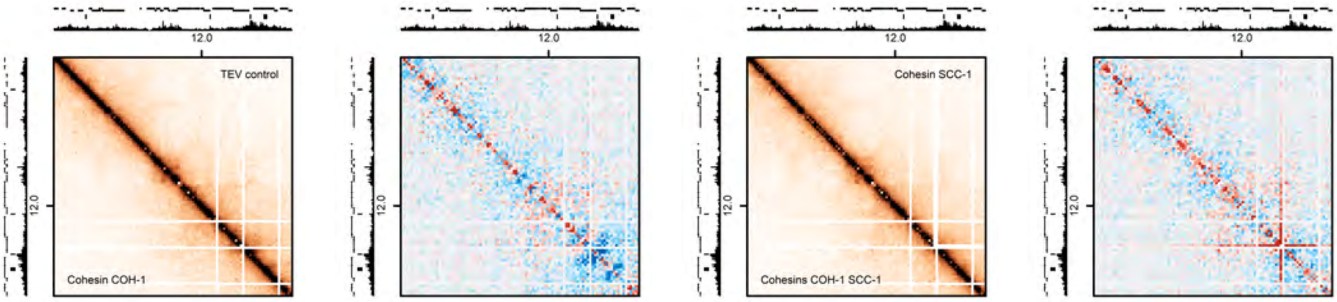

chrV:11111001-11311000

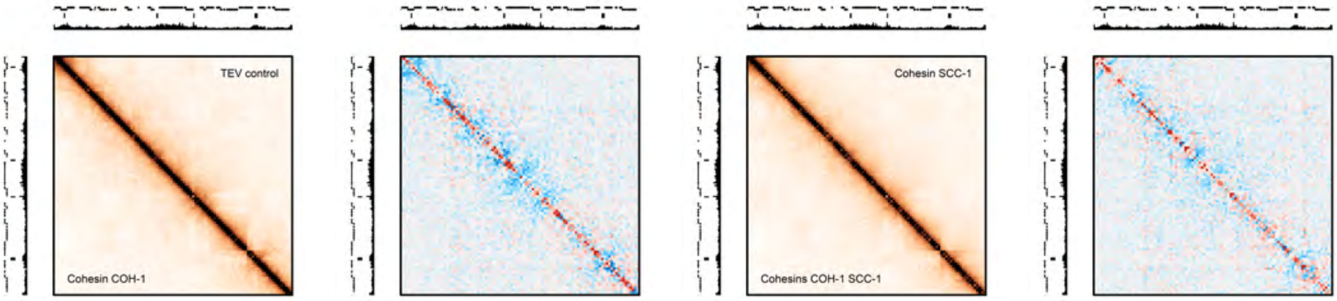

chrX:4801001-5001000

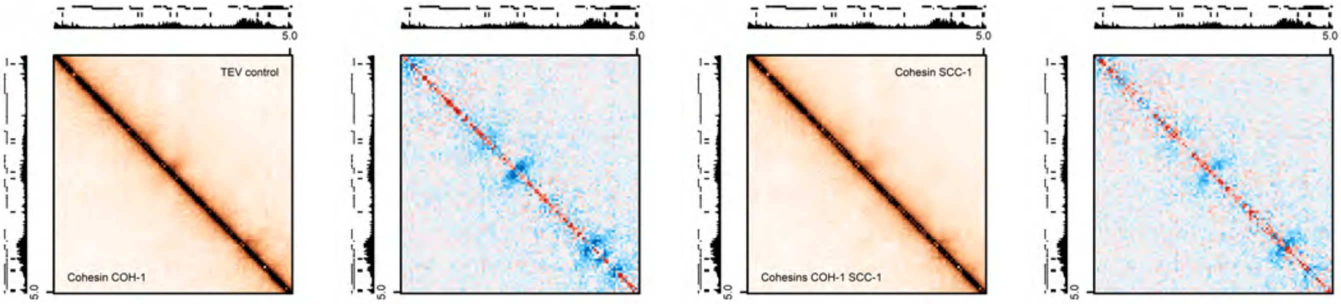

chrI:11353001-11553000

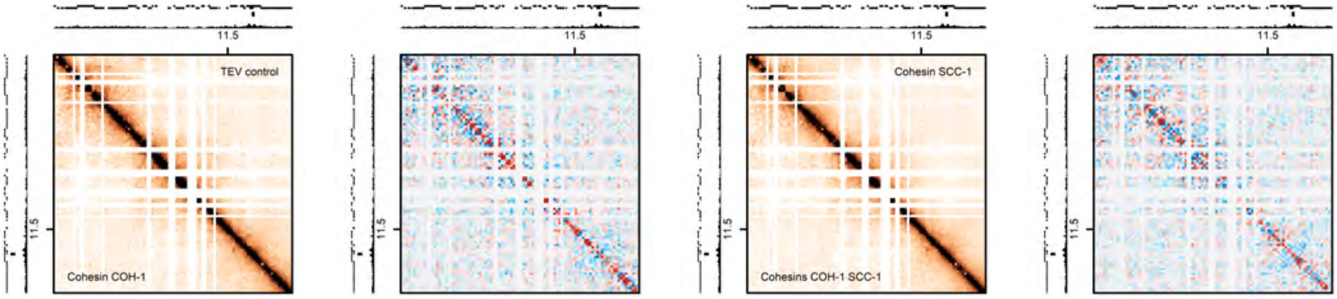

chrIII:5271001-5471000

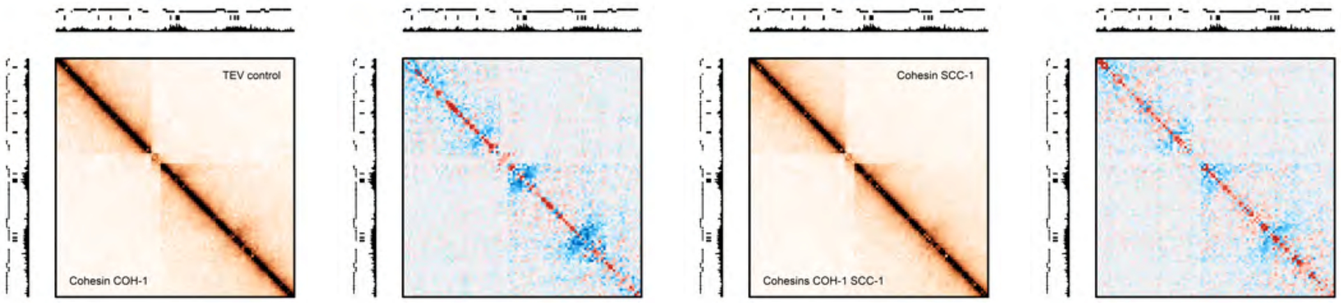

chrV:5667001-5867000

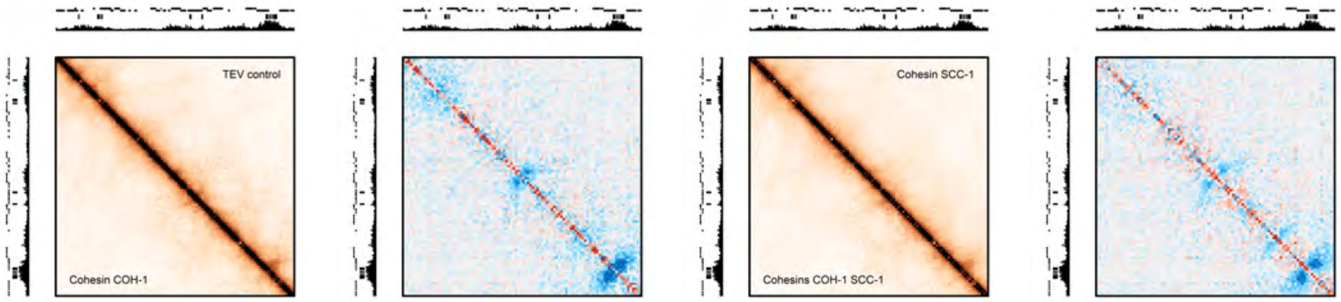

chrV:16001001-16201000

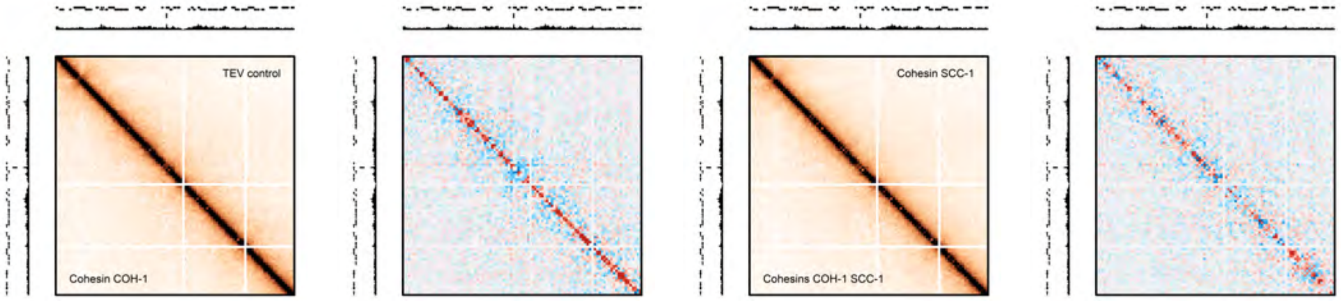

chrV:18393001-18593000

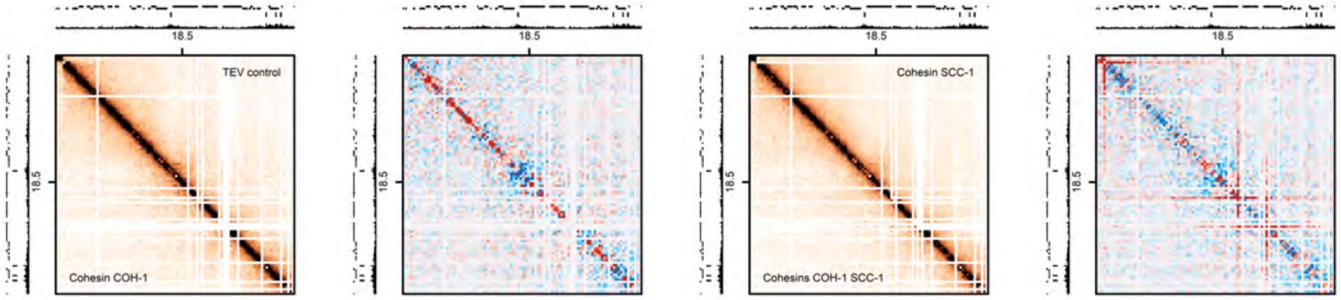

chrX:15745001-15945000

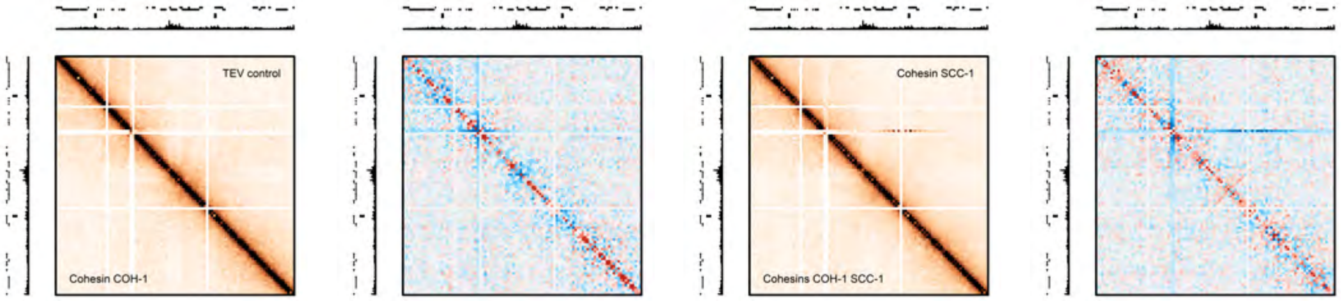

chrII:10349001-10549000

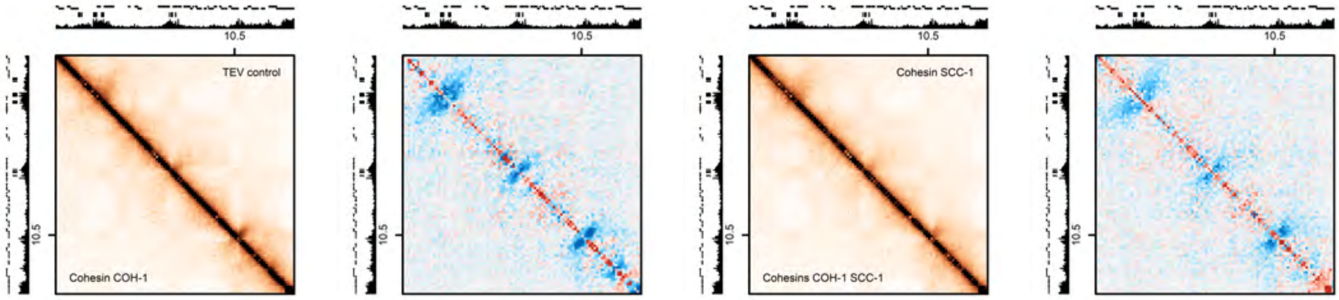

chrIII:1721001-1921000

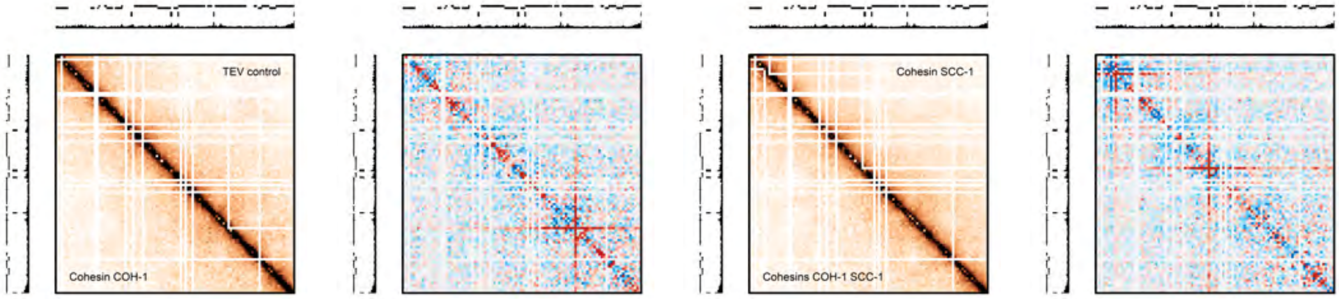

chrI:5035001-5235000

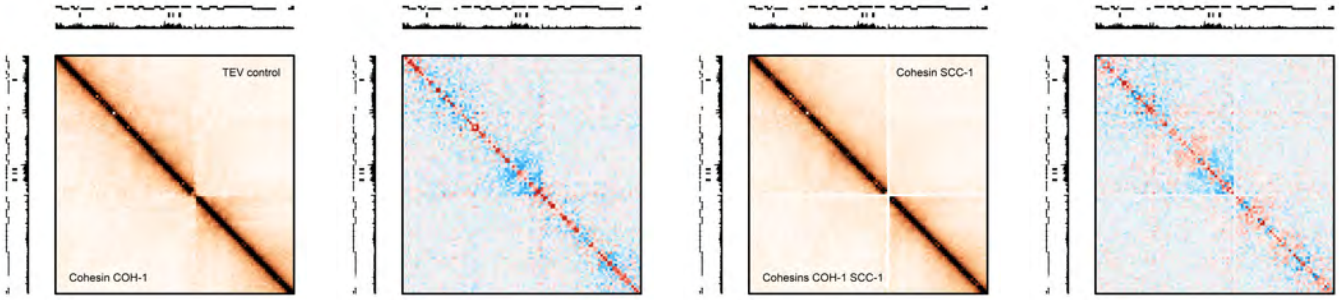

chrII:783001-983000

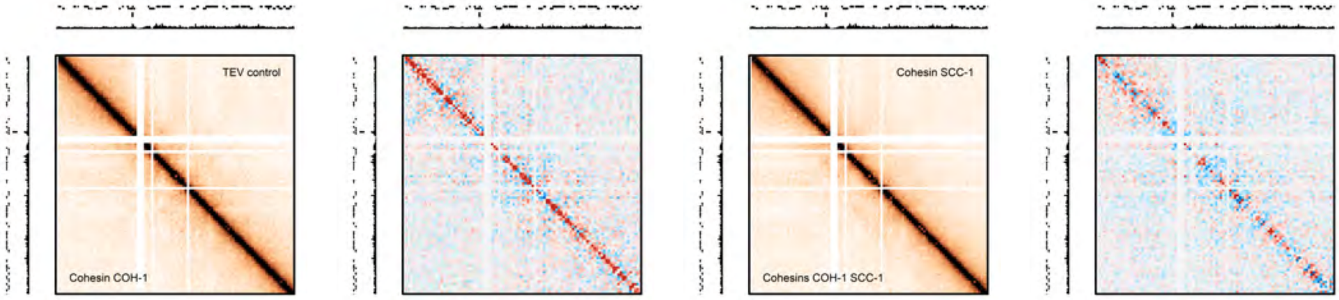

chrIII:3521001-3721000

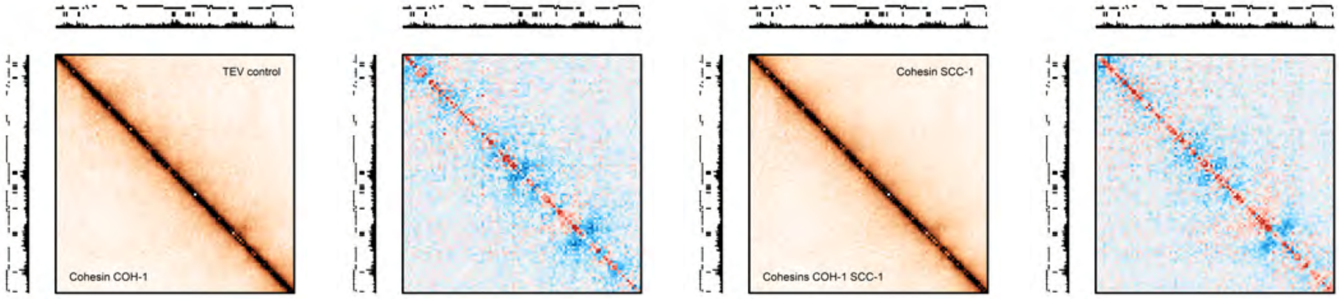

chrI:13447001-13647000

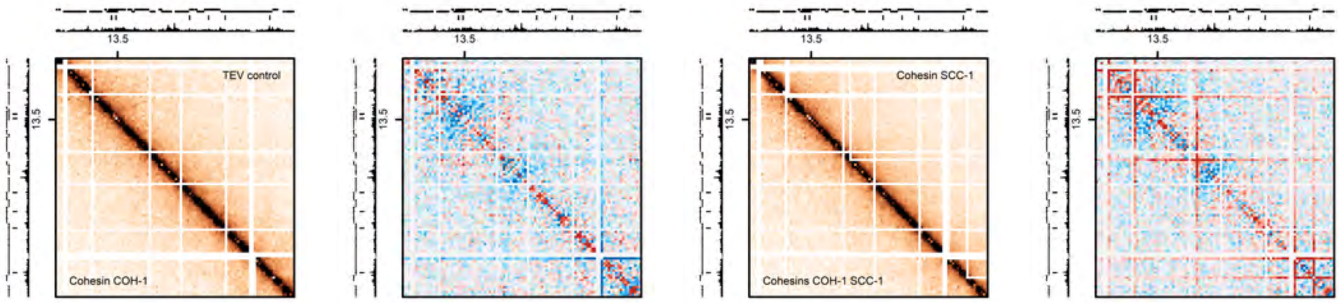

chrIII:7197001-7397000

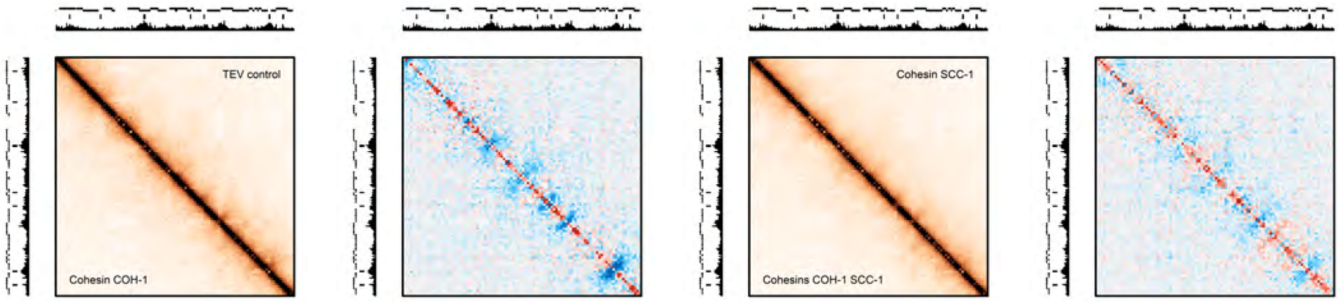

chrI:8883001-9083000

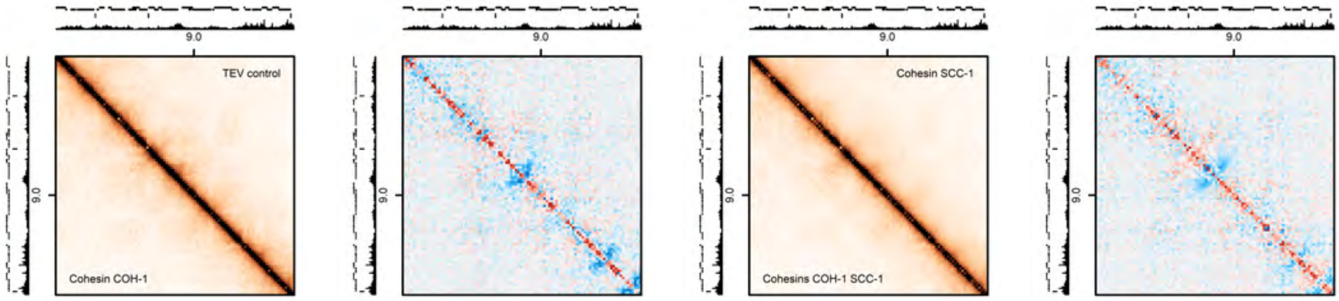

chrIV:6403001-6603000

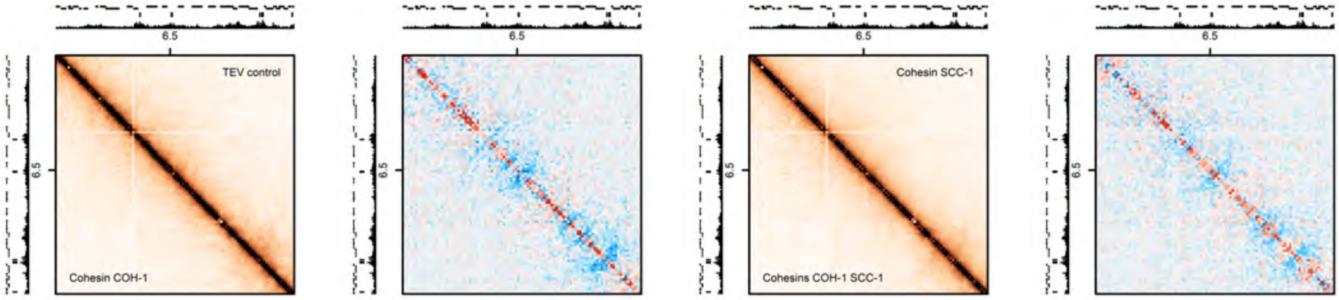

chrIII:1431001-1631000

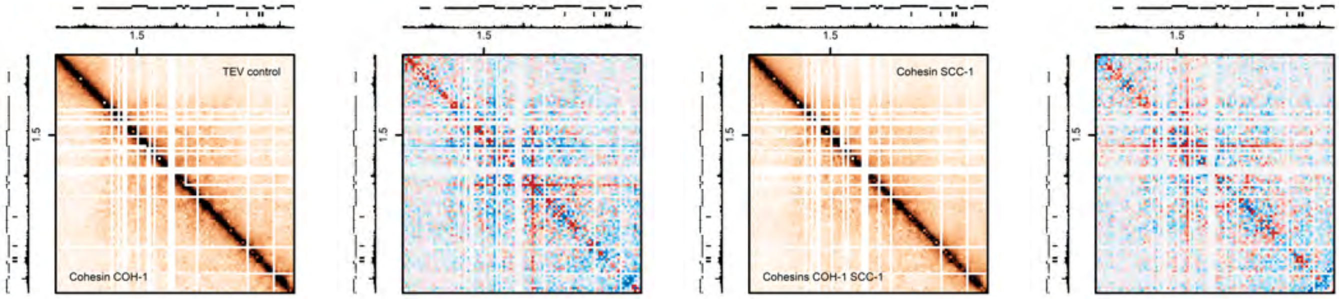

chrV:1135001-1335000

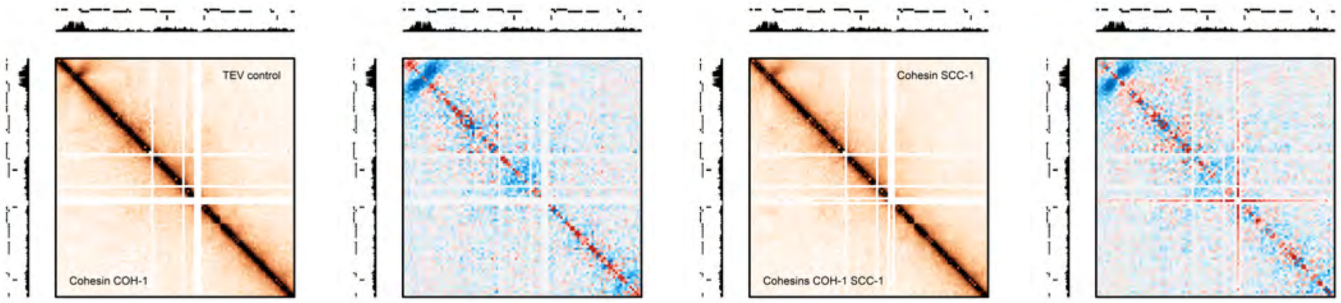

chrIV:6117001-6317000

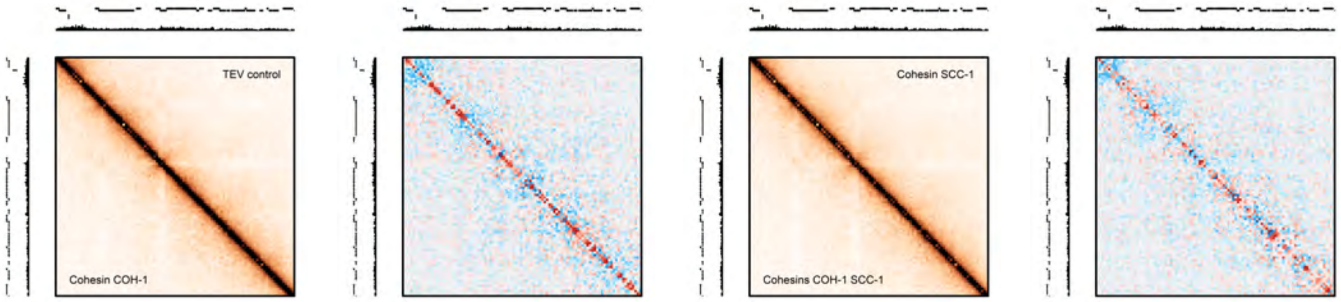

chrIV:2789001-2989000

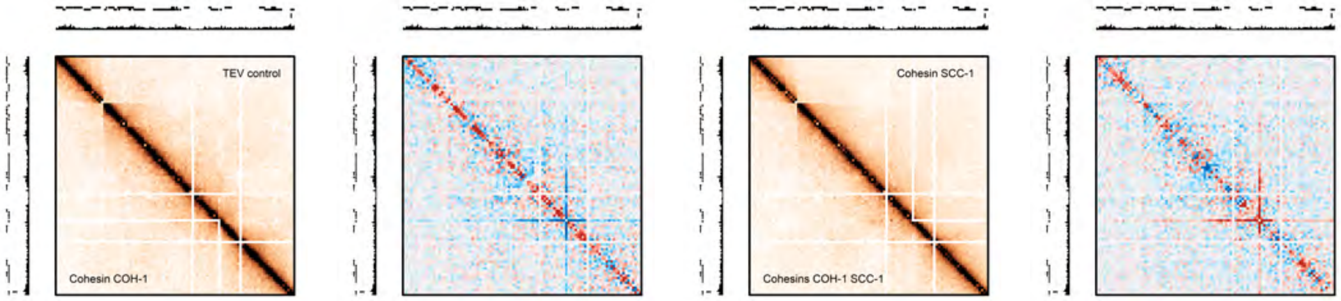

chrX:3581001-3781000

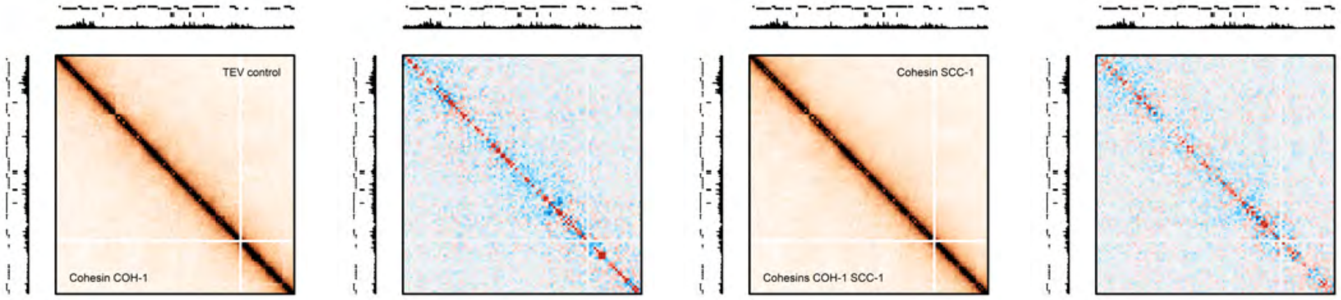

chrIV:9415001-9615000

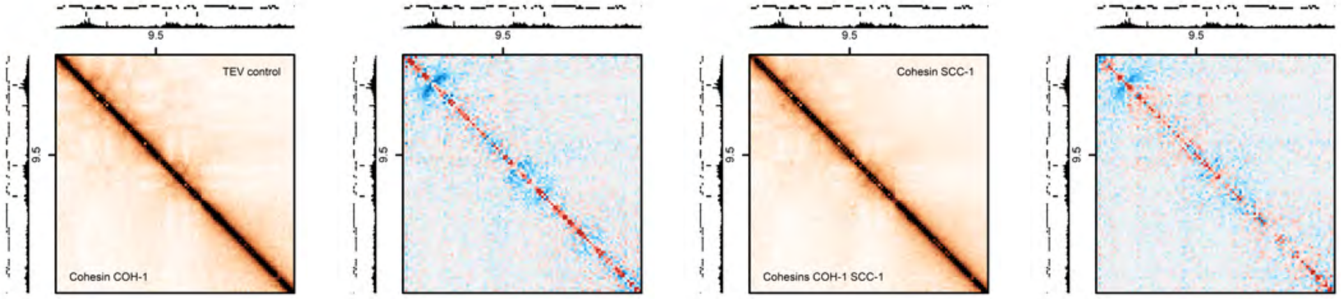

chrII:10981001-11181000

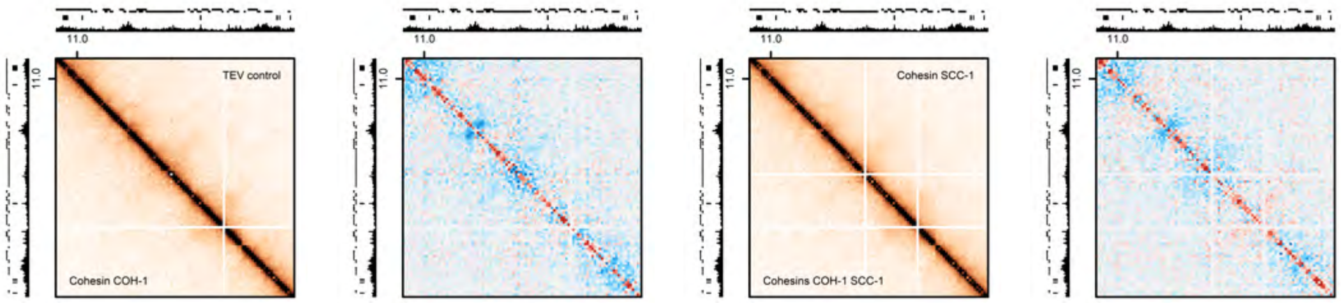

chrX:4527001-4727000

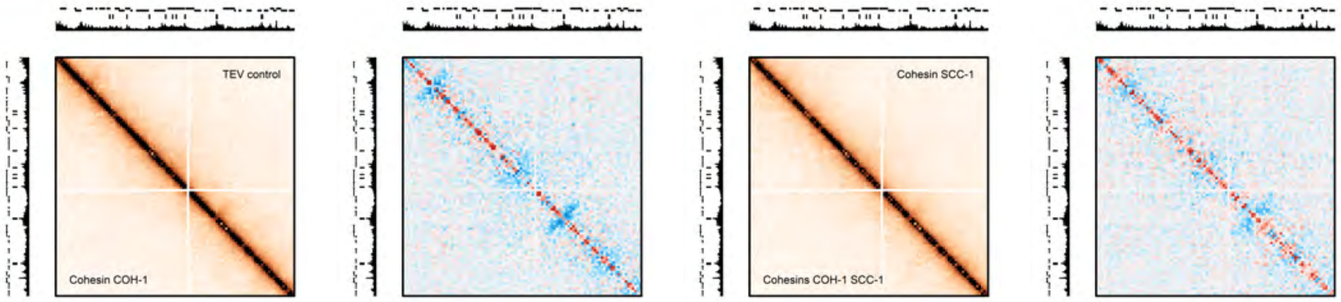

chrI:1247001-1447000

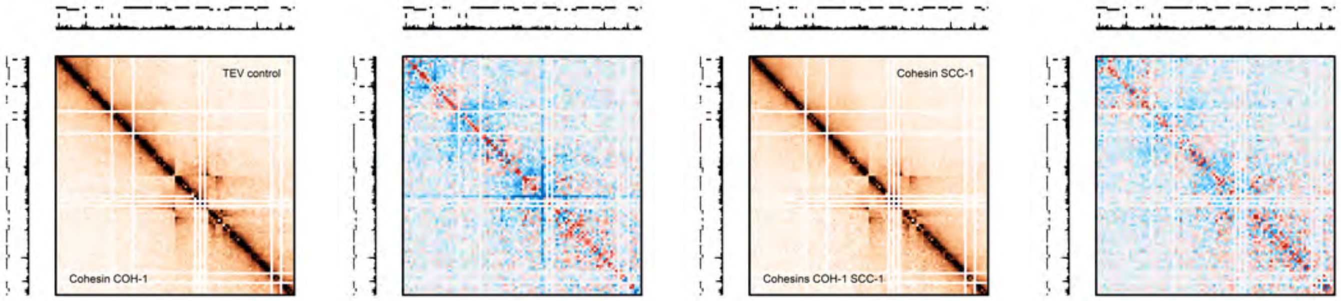

chrV:13477001-13677000

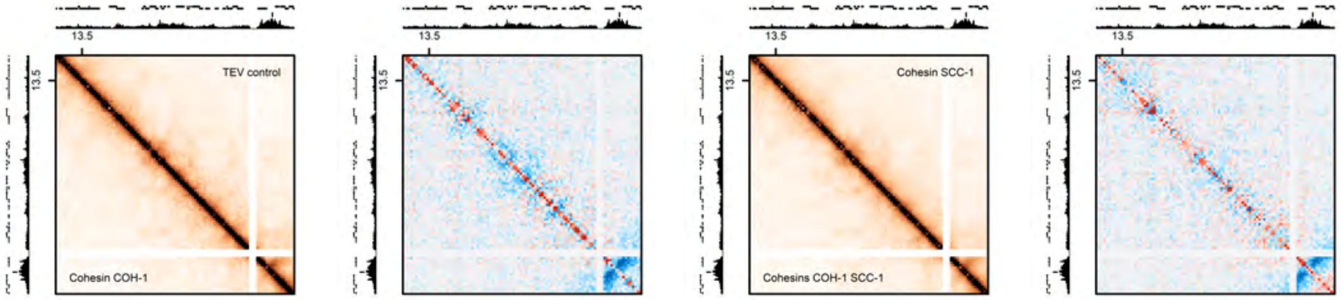

chrX:13427001-13627000

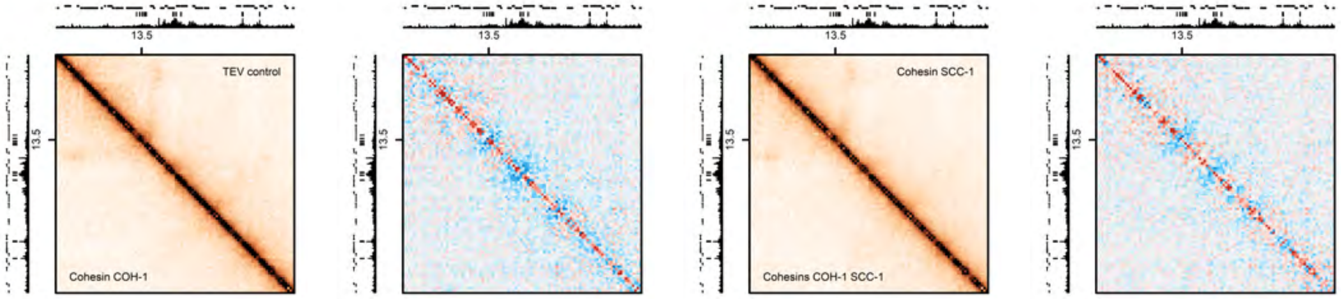

chrX:4285001-4485000

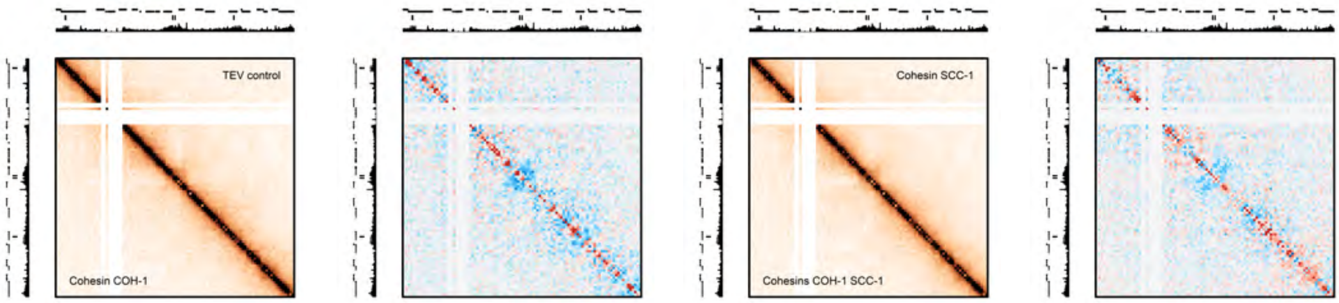

chrX:2333001-2533000

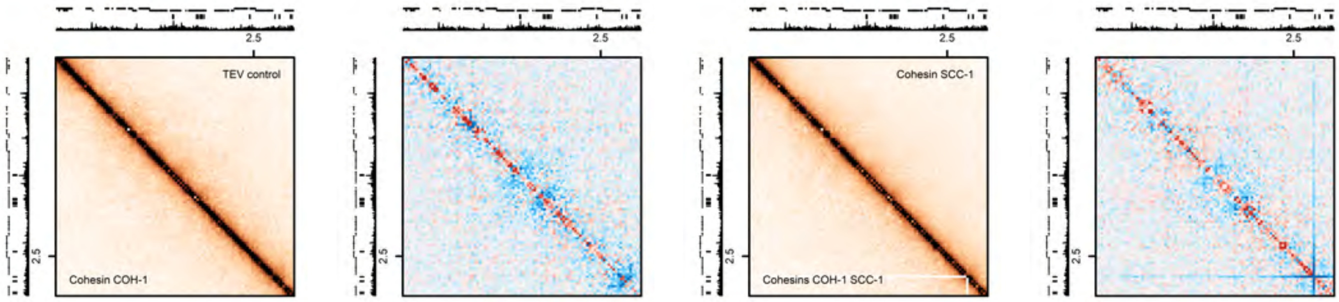

chrII:12967001-13167000

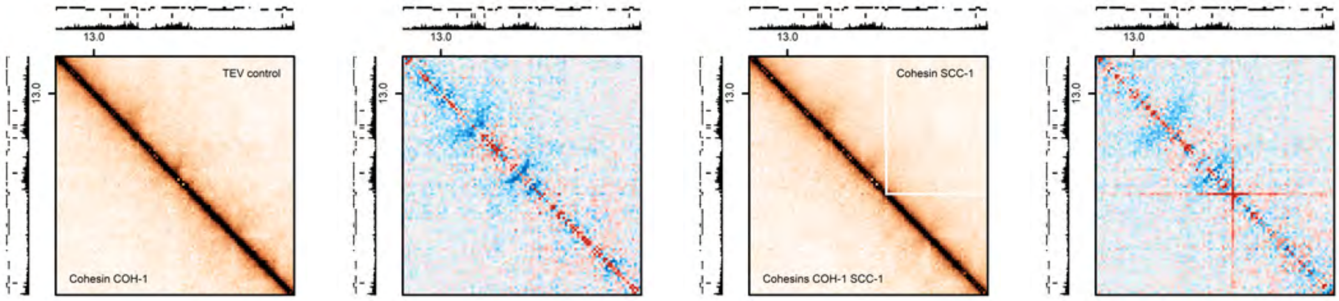

chrIV:14695001-14895000

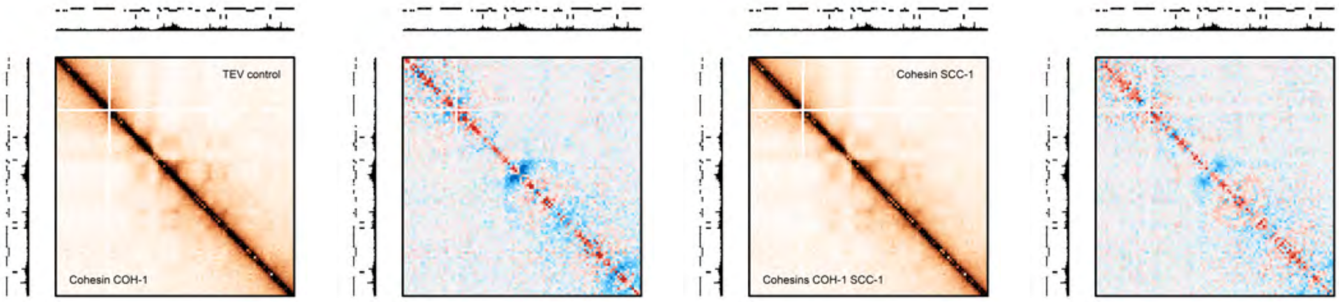

chrX:7359001-7559000

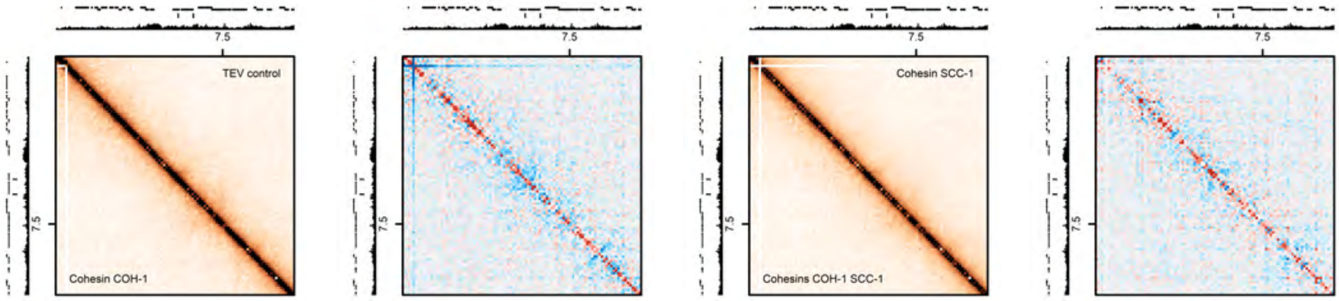

chrIV:15465001-15665000

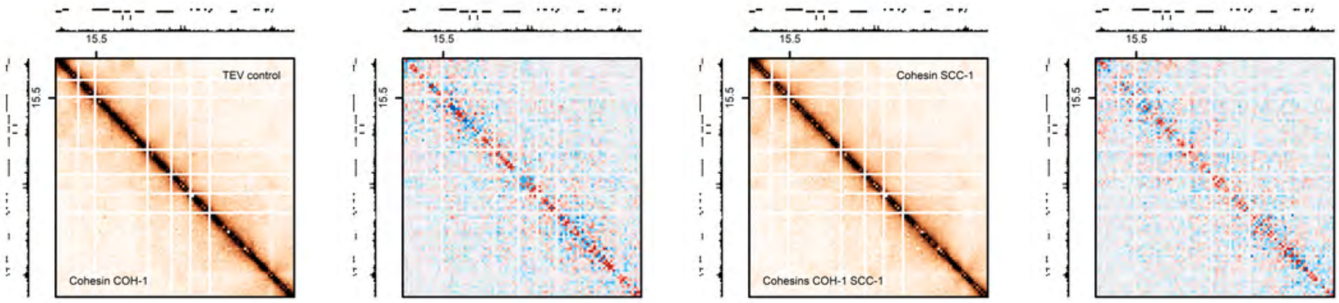

chrX:723001-923000

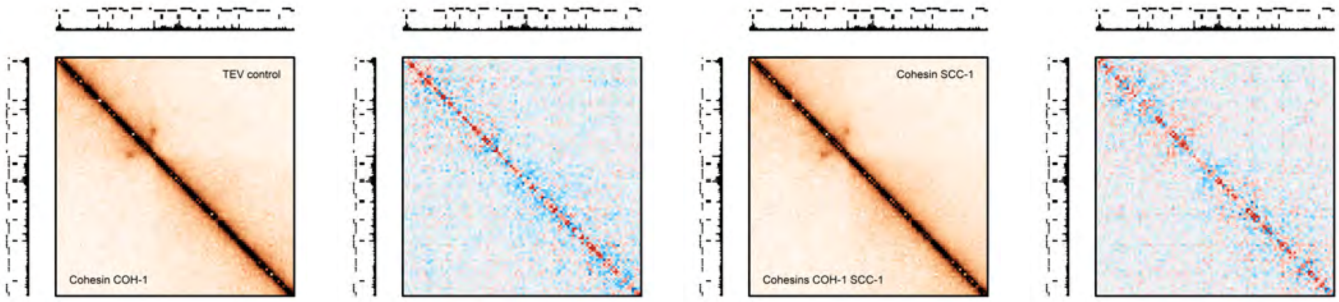

chrIV:9897001-10097000

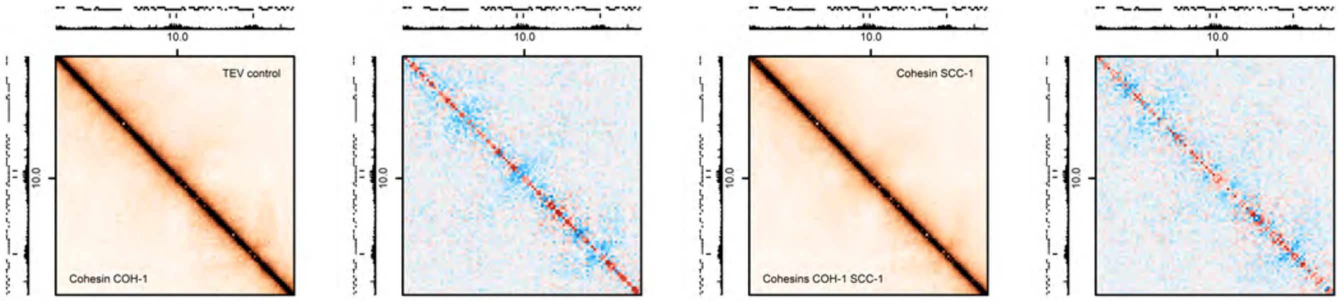

chrV:2515001-2715000

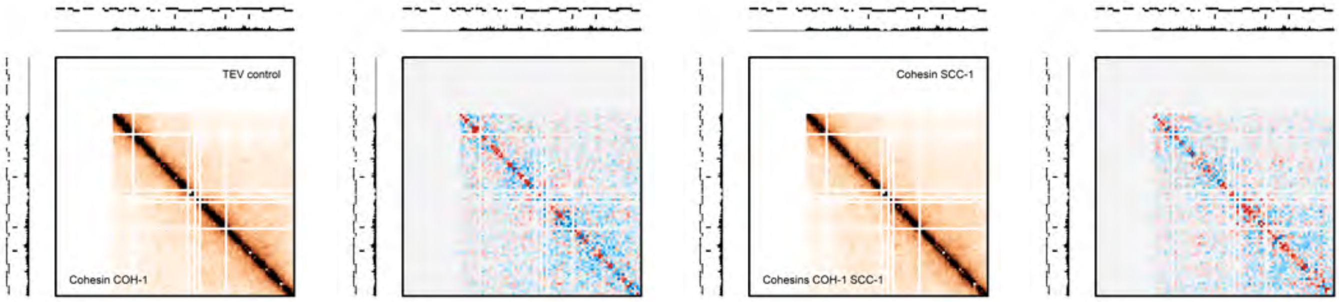

chrII:7293001-7493000

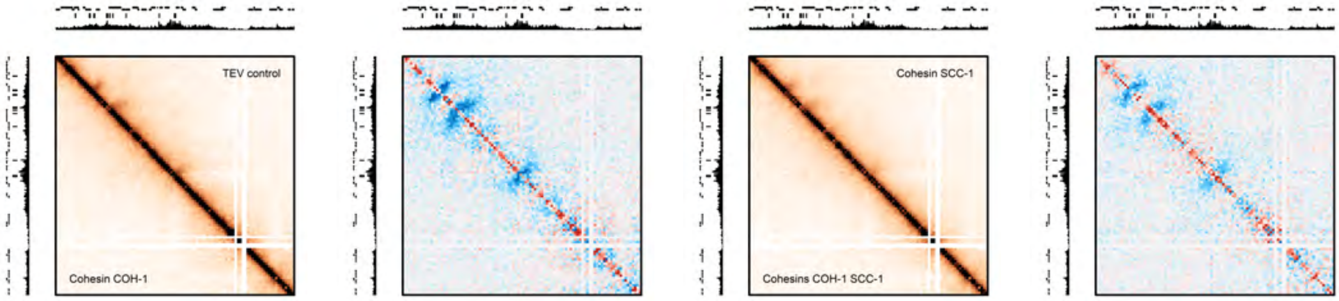

chrIV:7219001-7419000

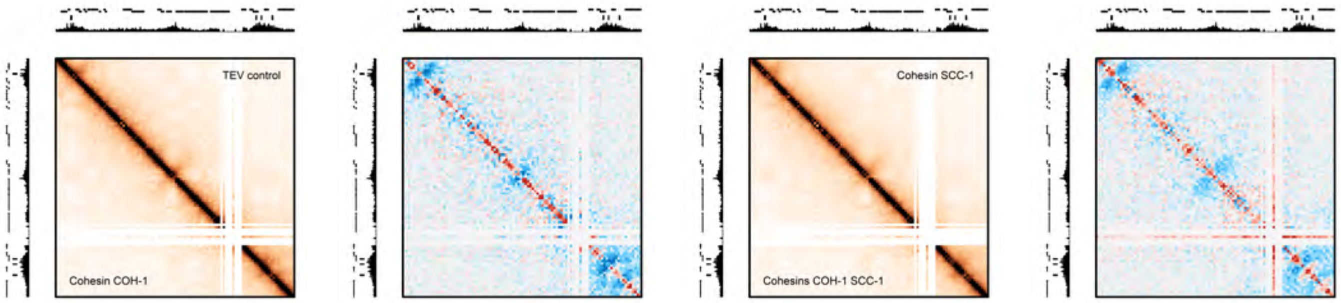

chrV:14521001-14721000

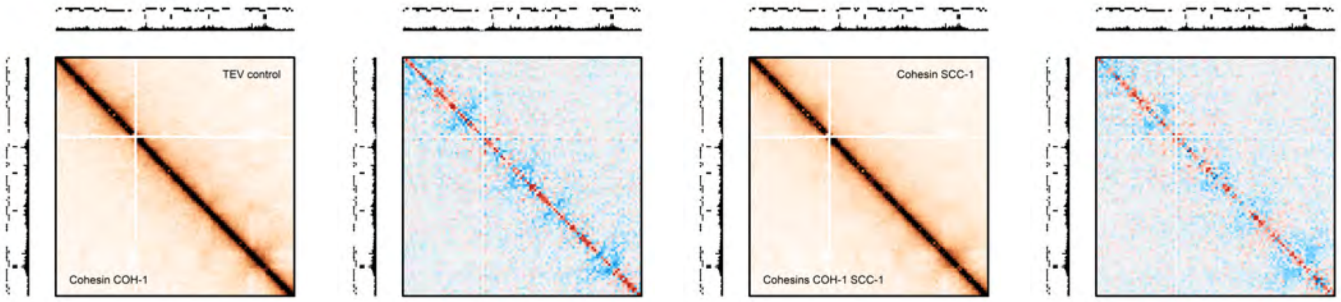

chrIII:13665001-13865000

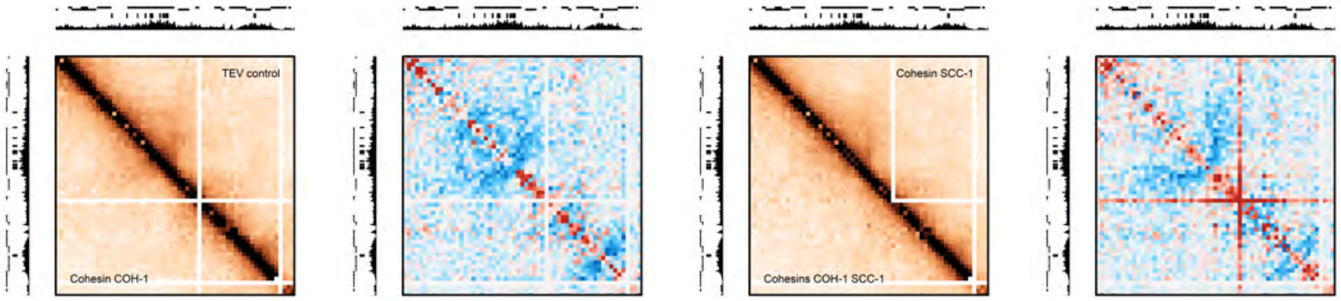

chrI:9843001-10043000

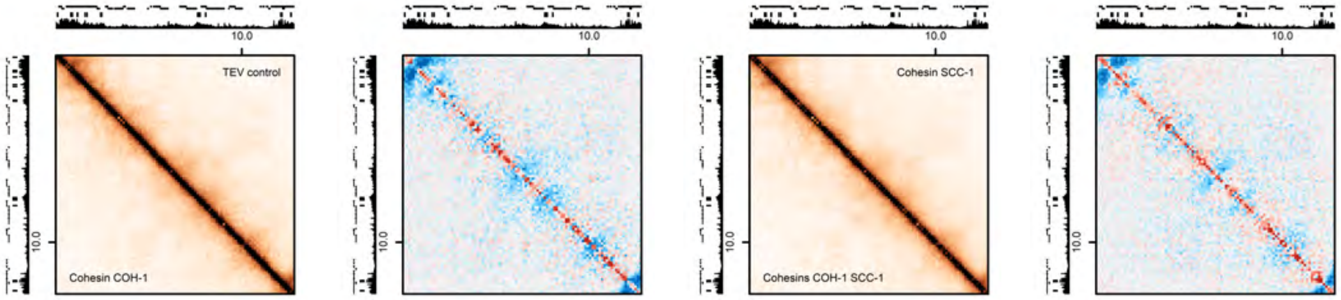

chrX:3799001-3999000

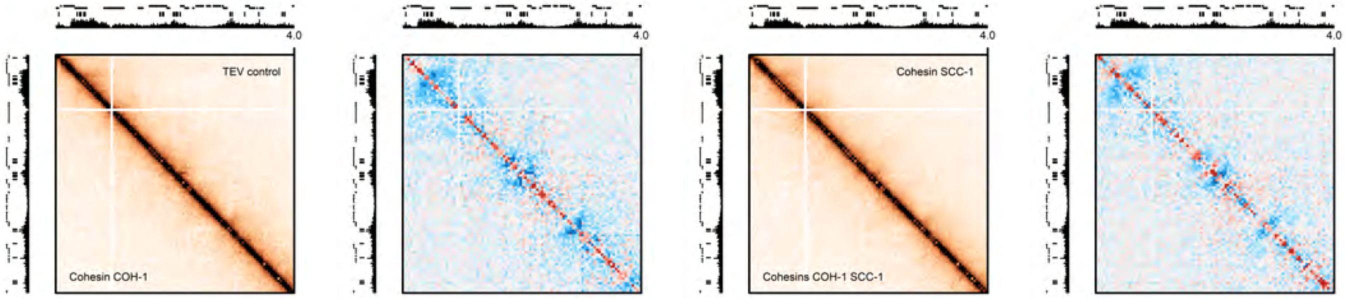

chrV:15781001-15981000

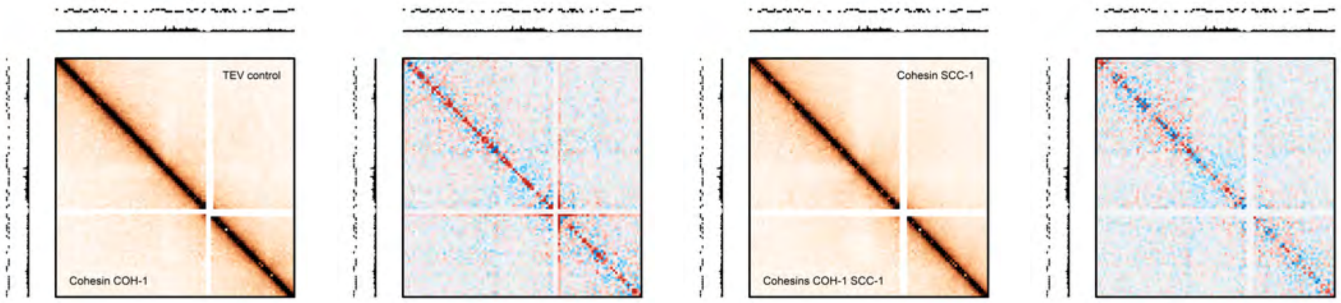

chrV:4257001-4457000

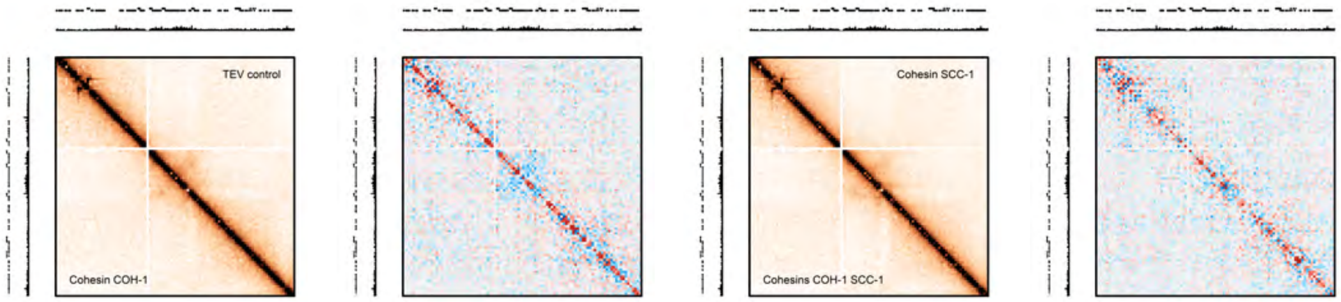

chrV:5307001-5507000

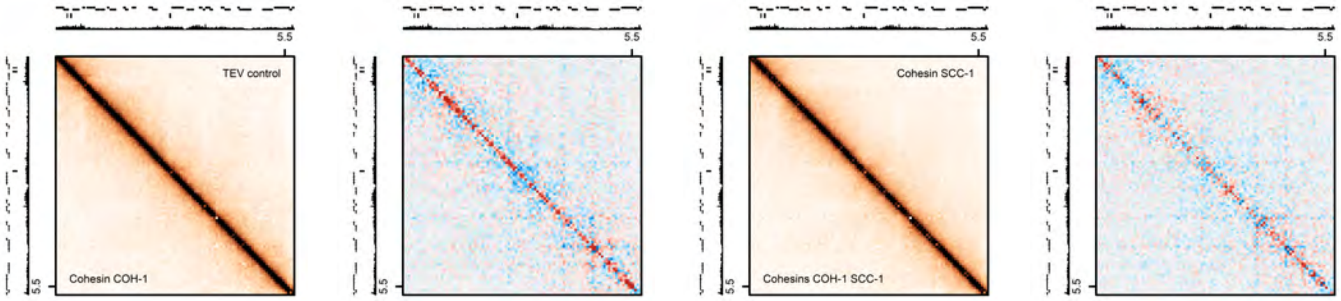

chrIII:2213001-2413000

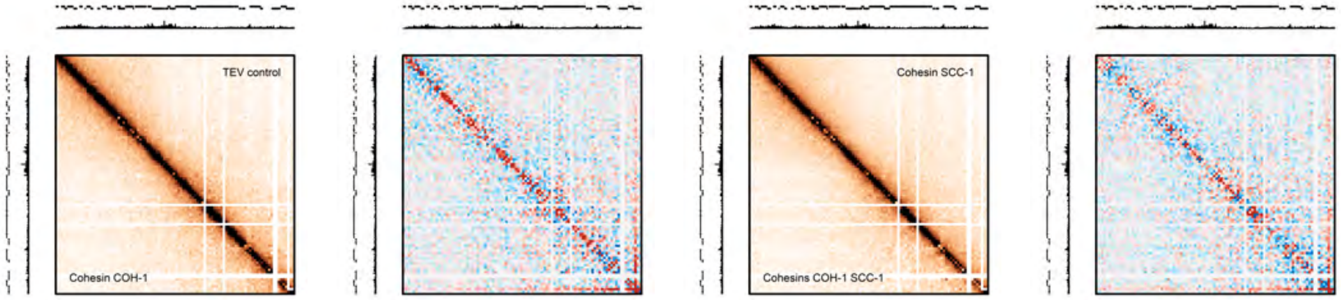

chrV:2701001-2901000

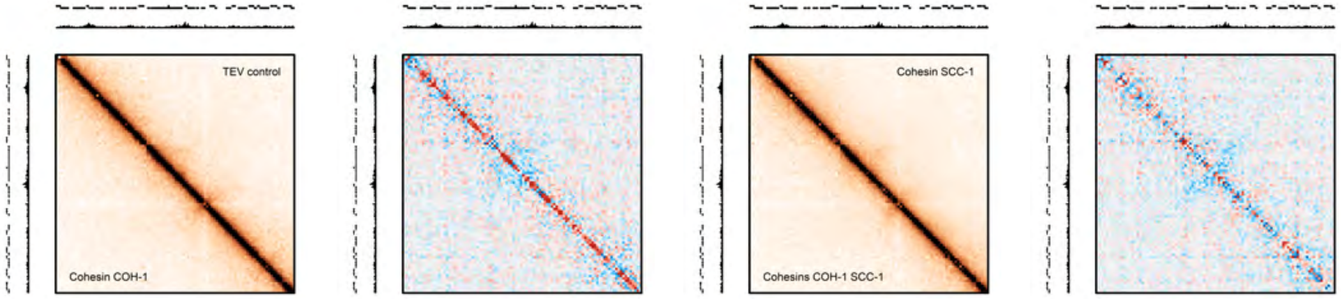

chrIII:13249001-13449000

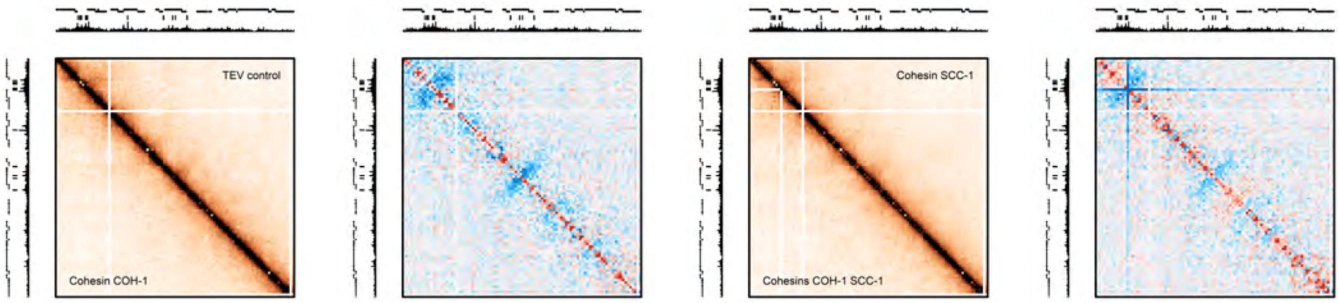

chrI:12637001-12837000

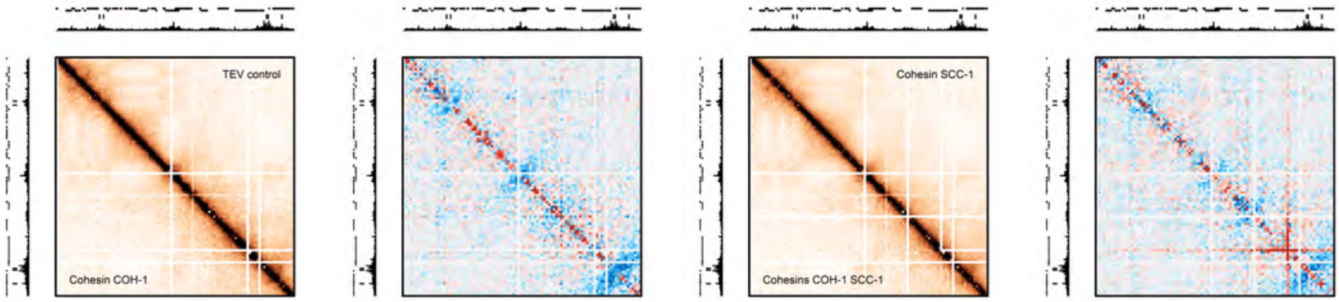

chrIV:7969001-8169000

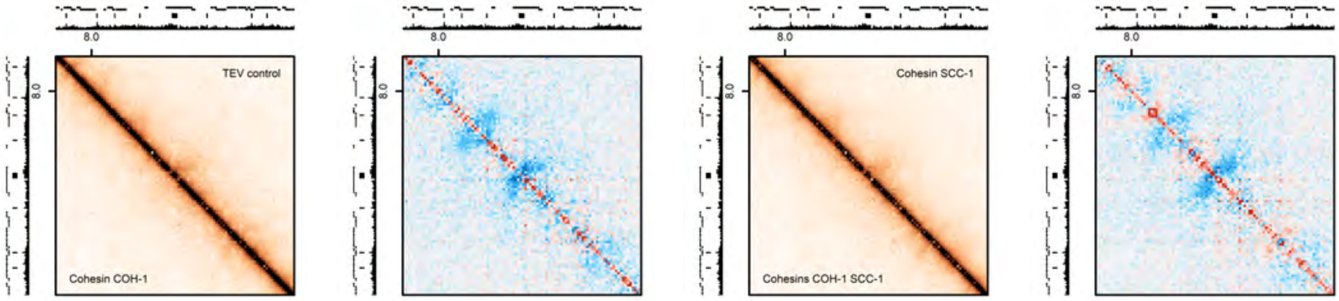

chrV:9231001-9431000

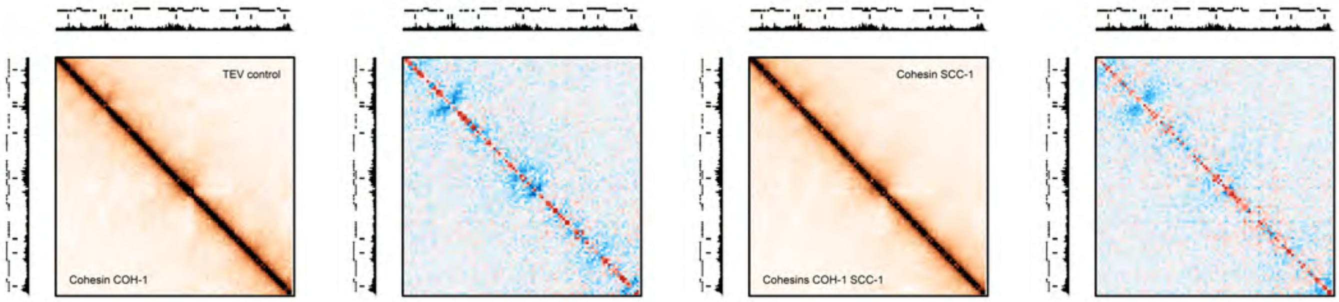

chrIII:327001-527000

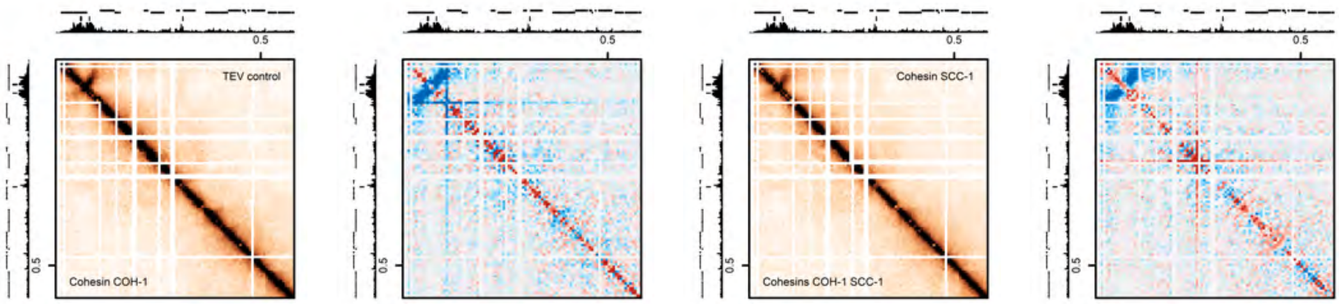

chrI:12579001-12779000

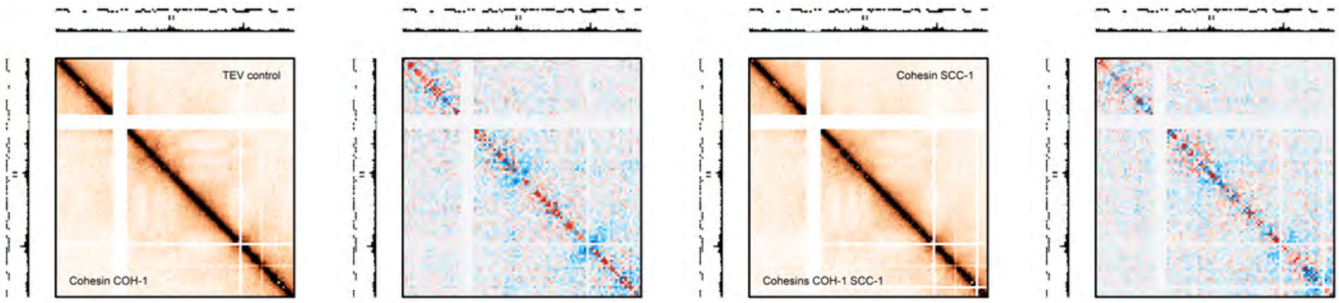

chrIII:12317001-12517000

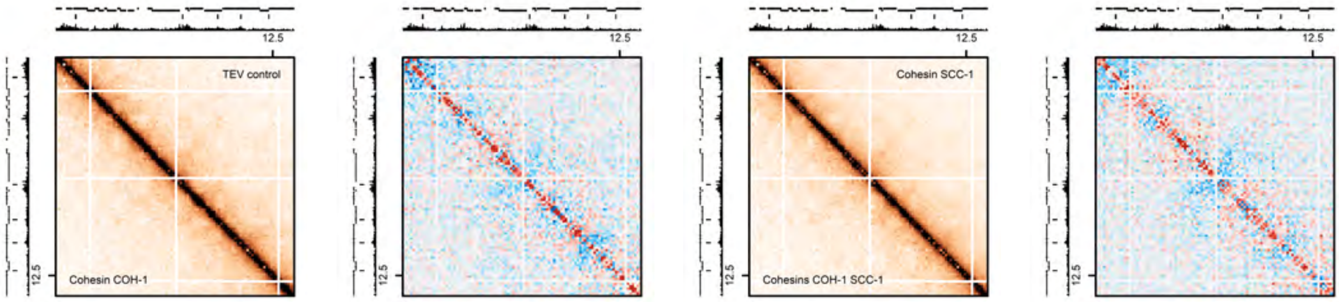

chrV:15231001-15431000

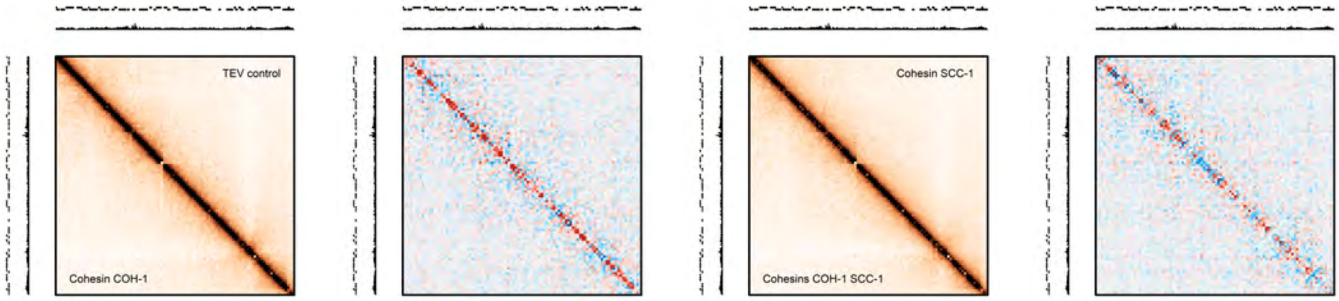

chrII:1043001-1243000

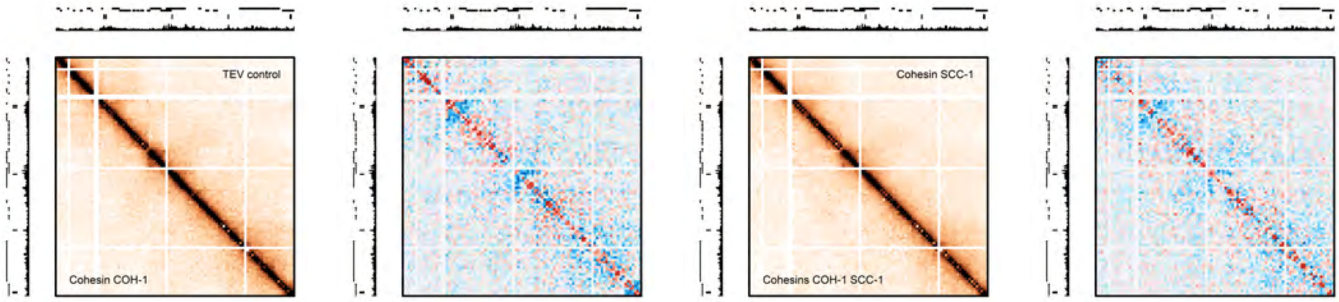

chrX:10227001-10427000

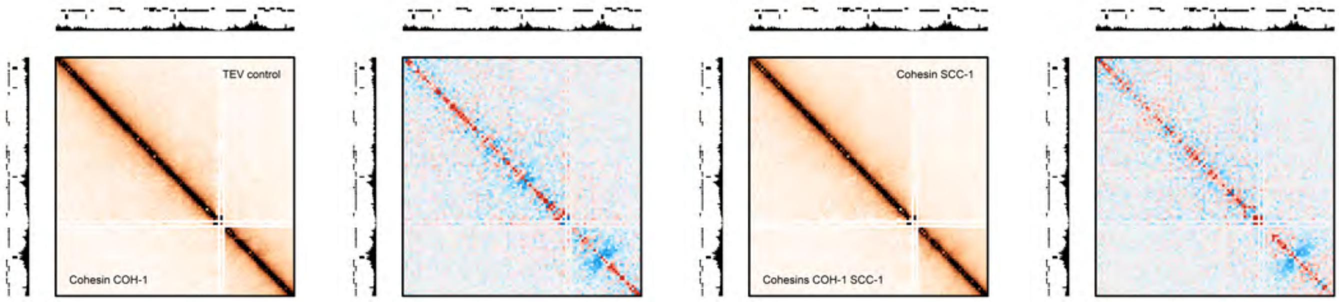

chrV:18953001-19153000

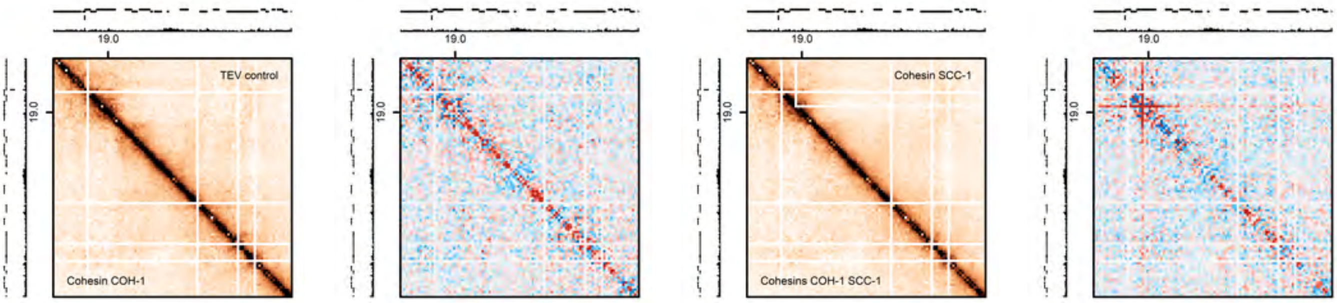

chrV:1479001-1679000

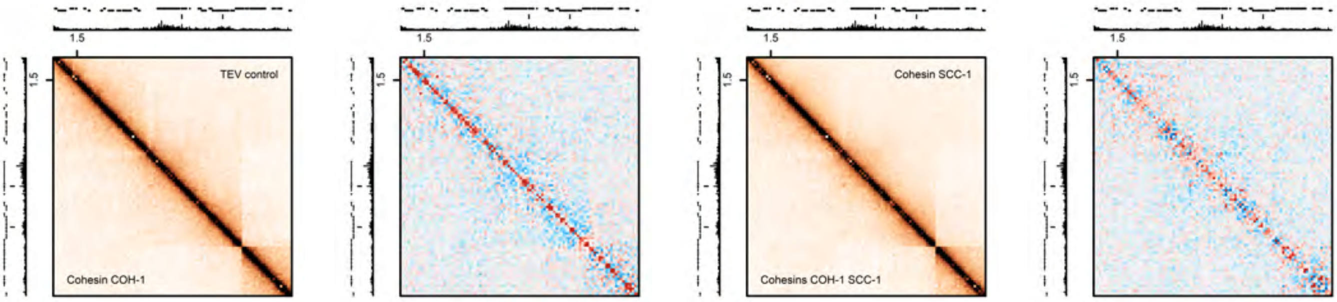

chrIV:11251001-11451000

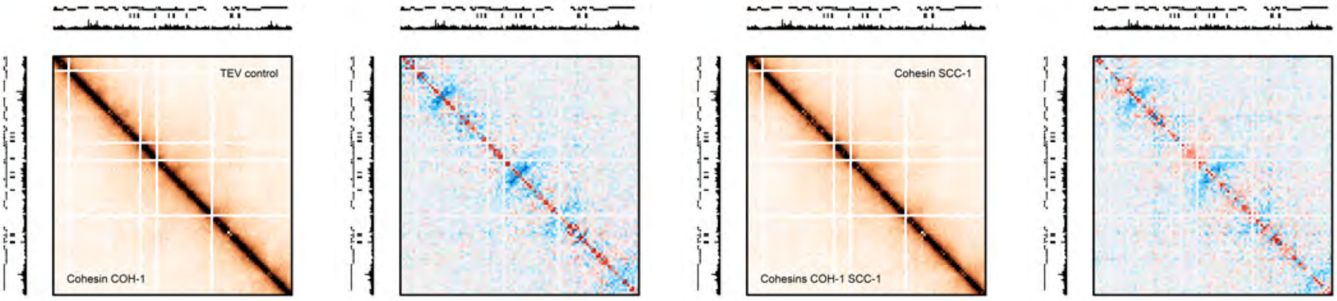

chrII:14203001-14403000

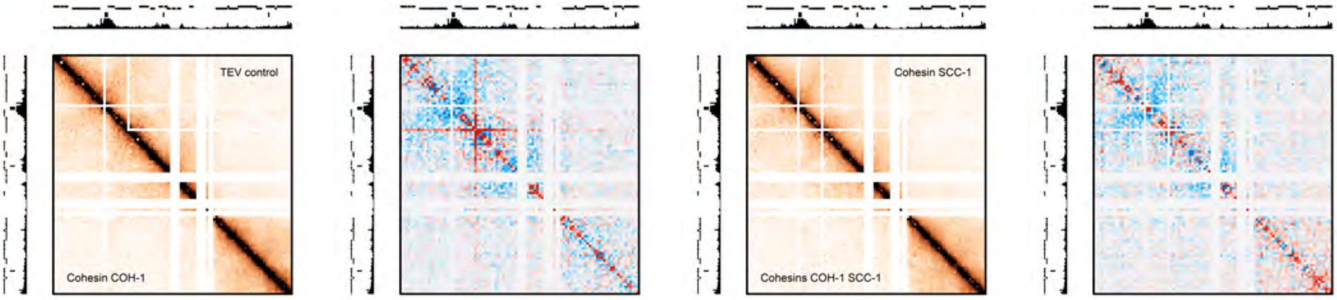

chrIV:15173001-15373000

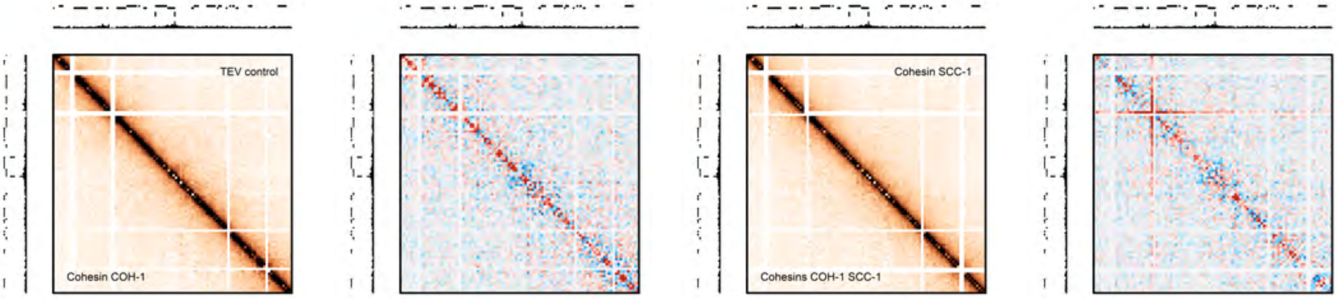

chrII:11445001-11645000

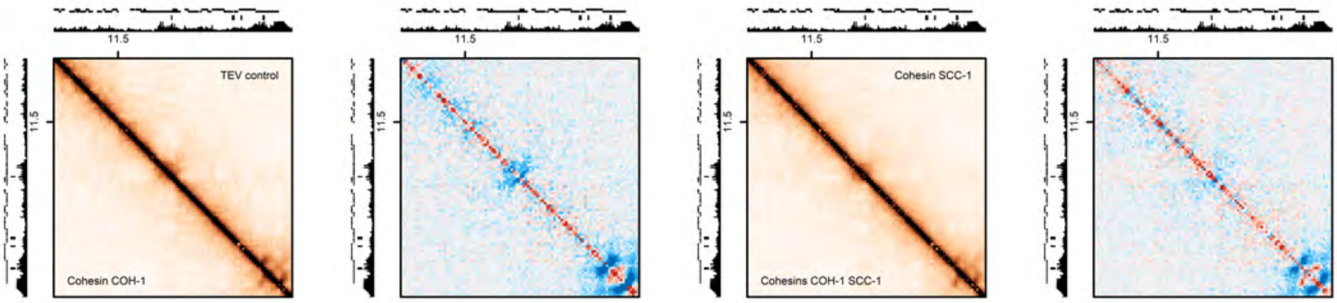

chrV:251001-451000

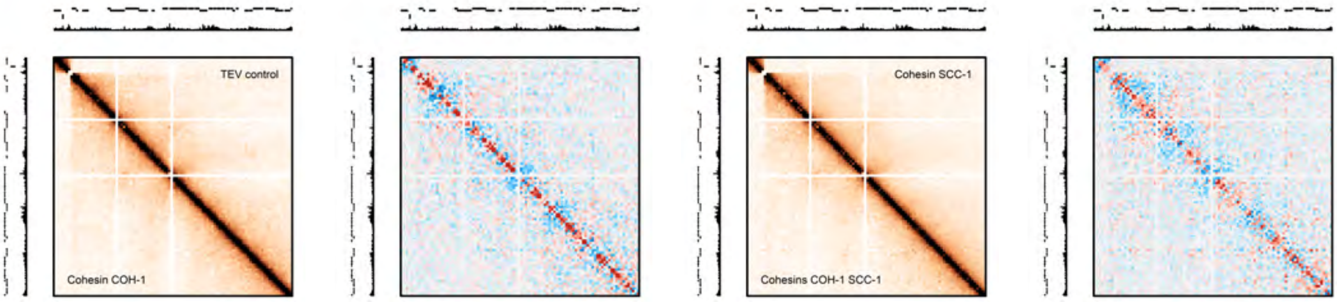

chrIV:9261001-9461000

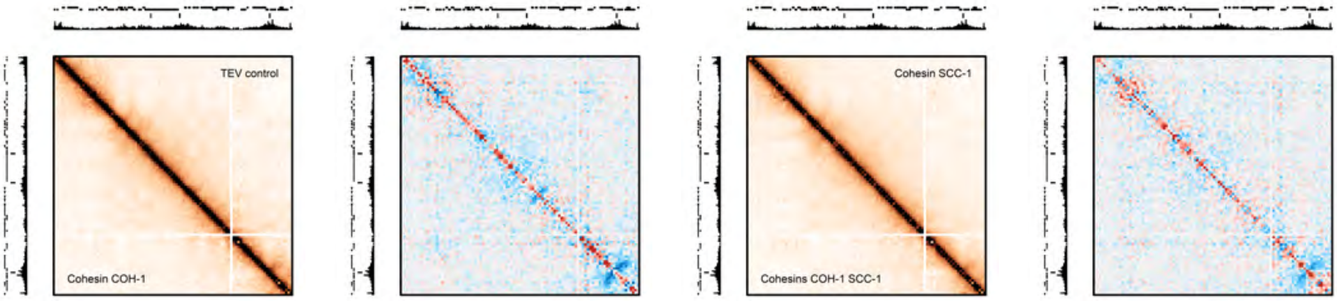

chrX:2115001-2315000

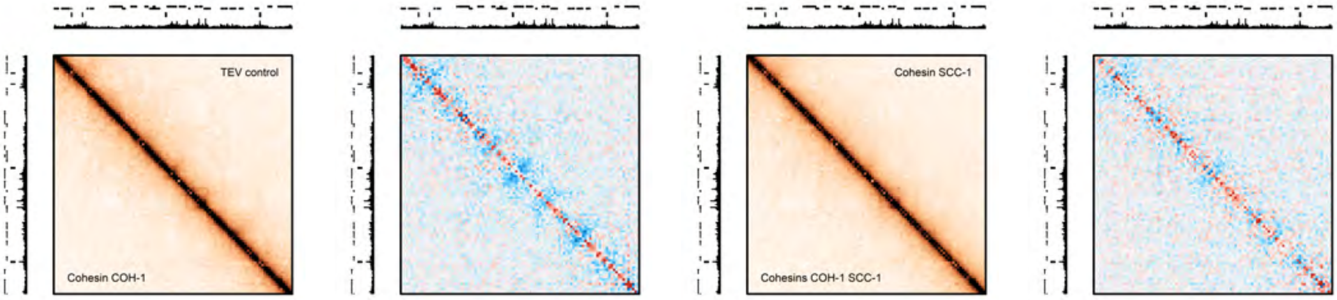

chrIV:2561001-2761000

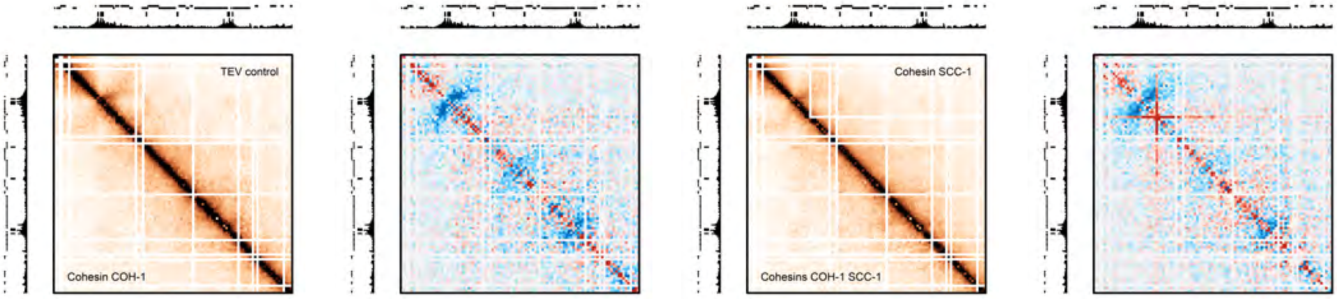

chrI:297001-497000

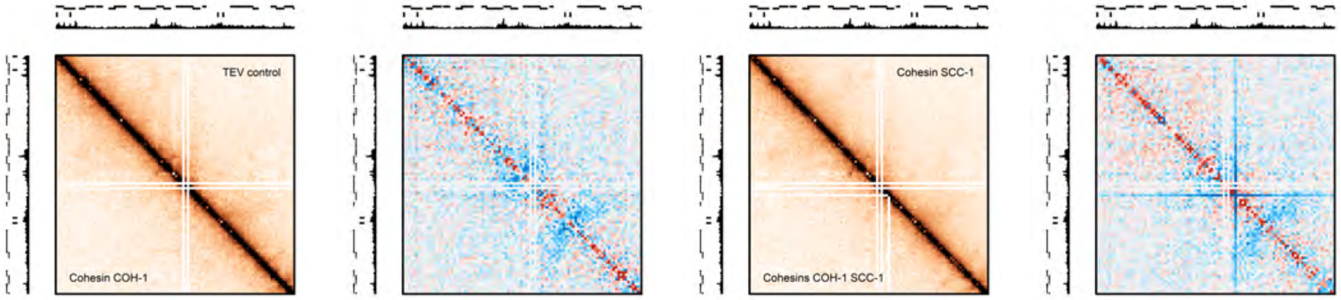

chrV:20021001-20221000

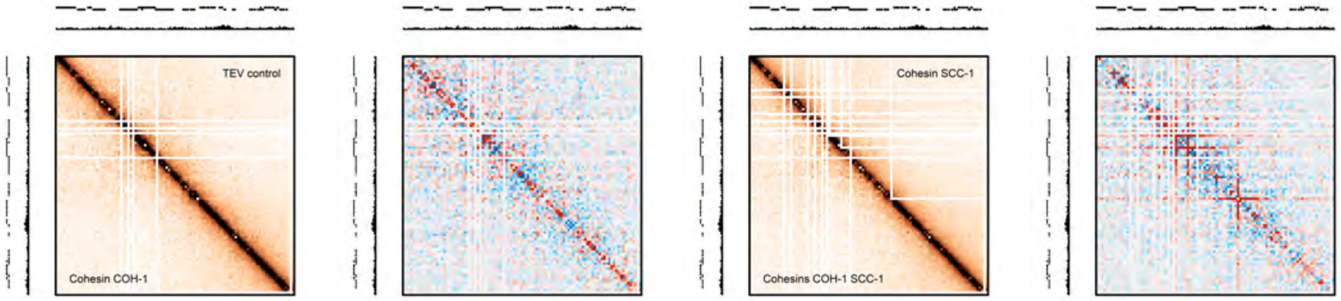

chrIII:10425001-10625000

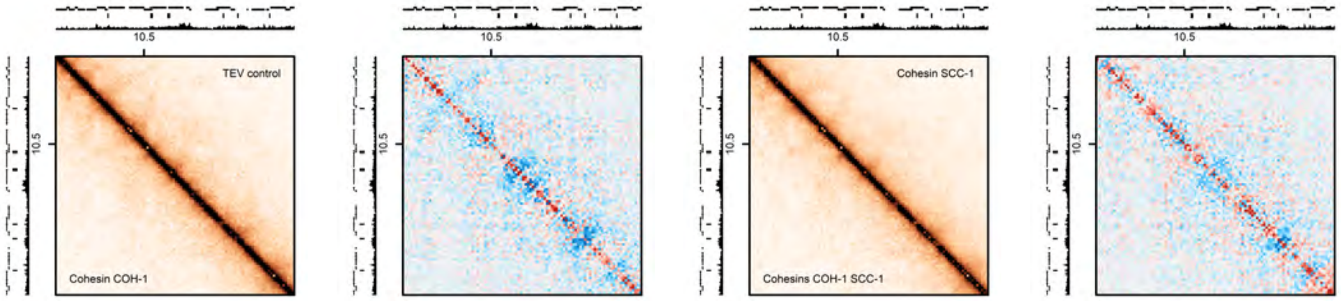

chrIV:12031001-12231000

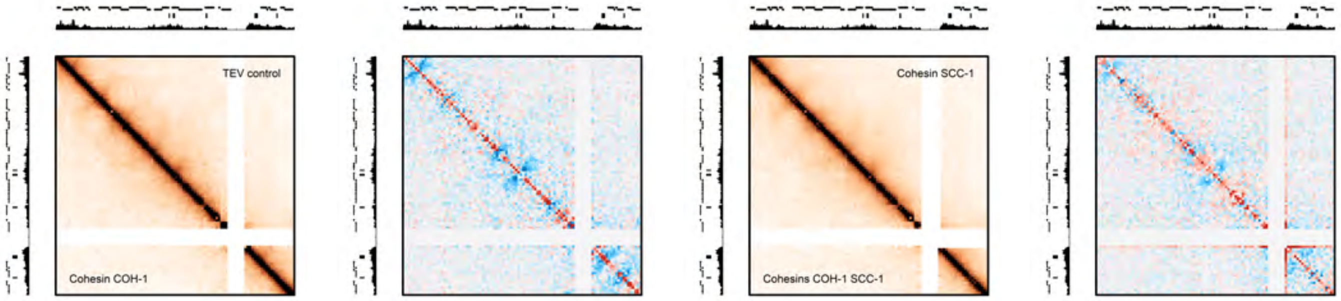

chrII:5945001-6145000

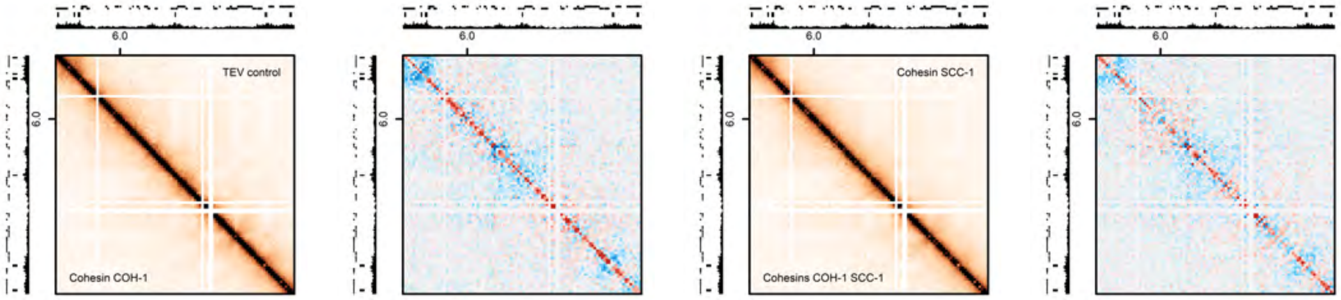

chrX:16899001-17099000

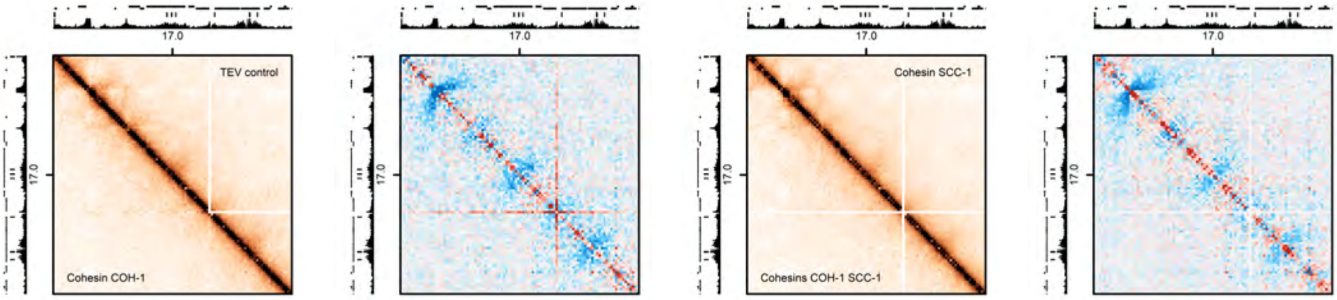

chrII:10657001-10857000

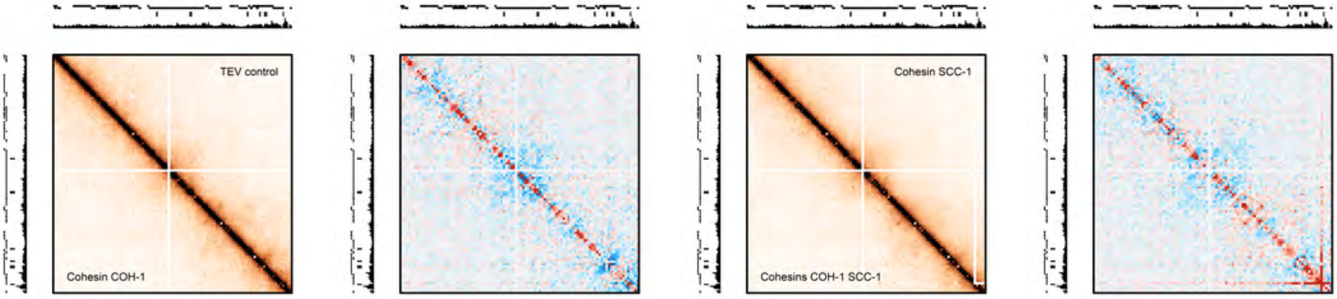

chrV:20613001-20813000

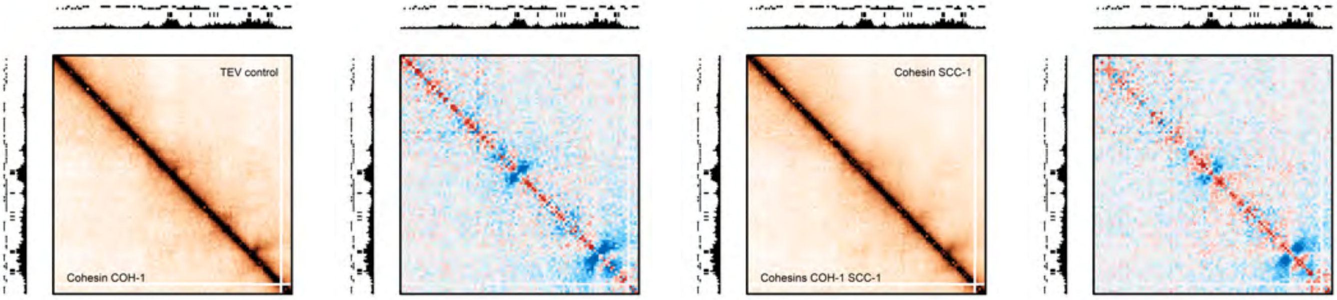

chrX:16959001-17159000

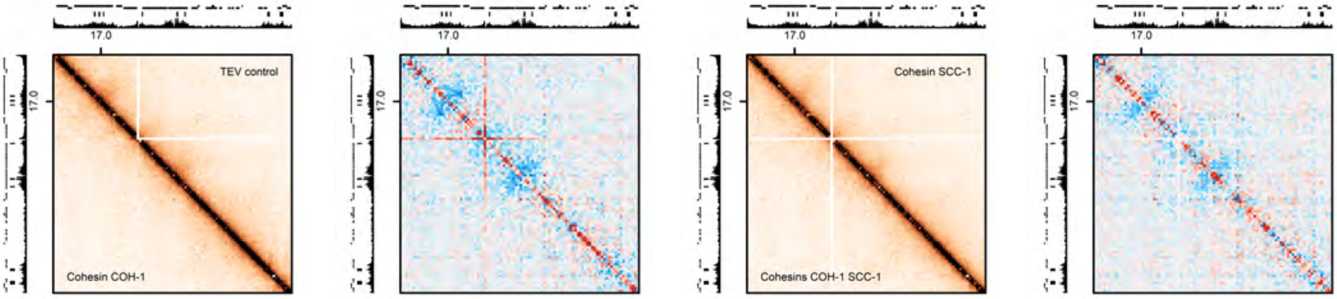

chrI:1385001-1585000

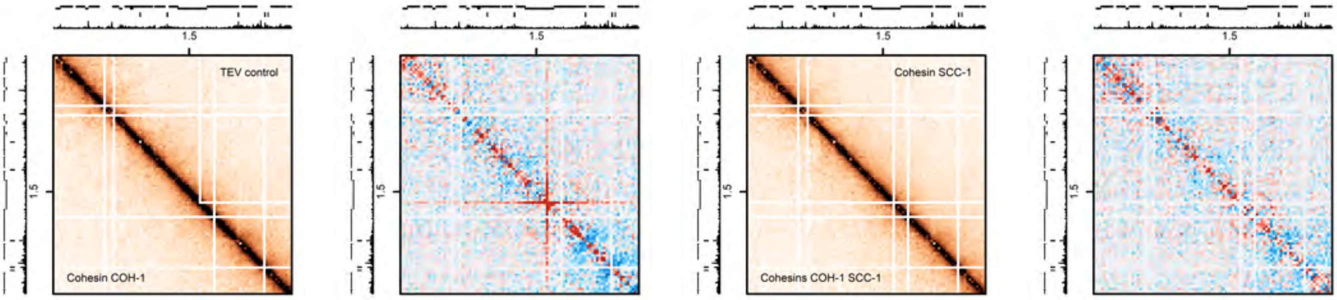

chrI:6071001-6271000

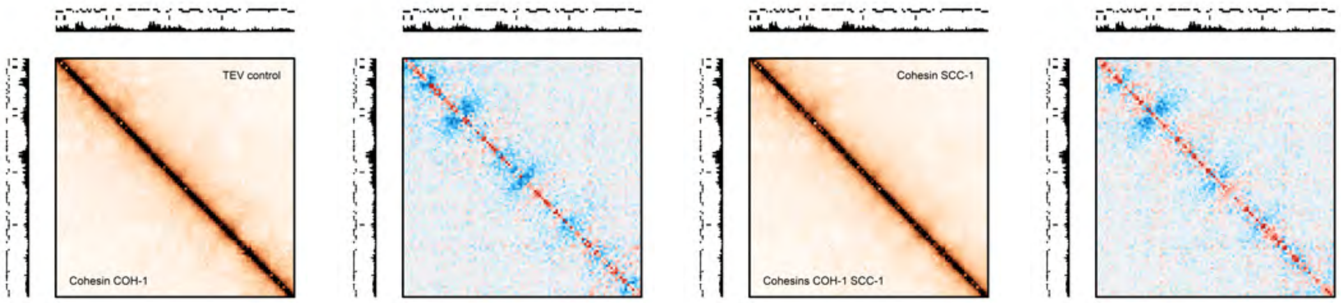

chrIV:3643001-3843000

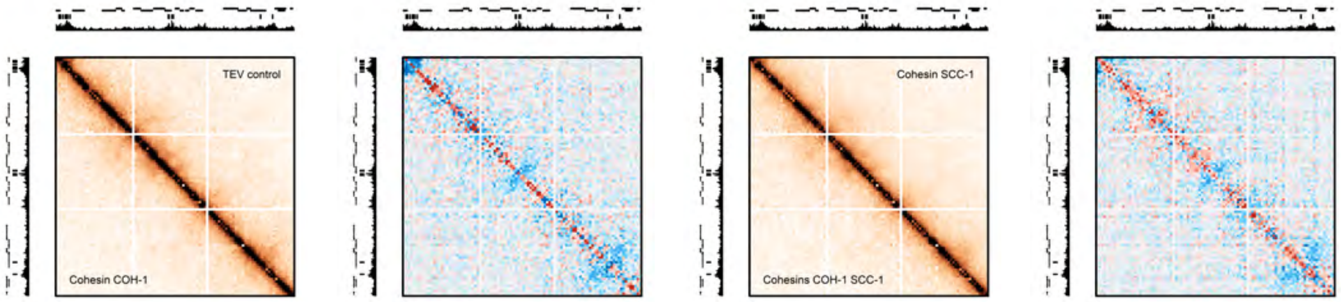

chrIII:13083001-13283000

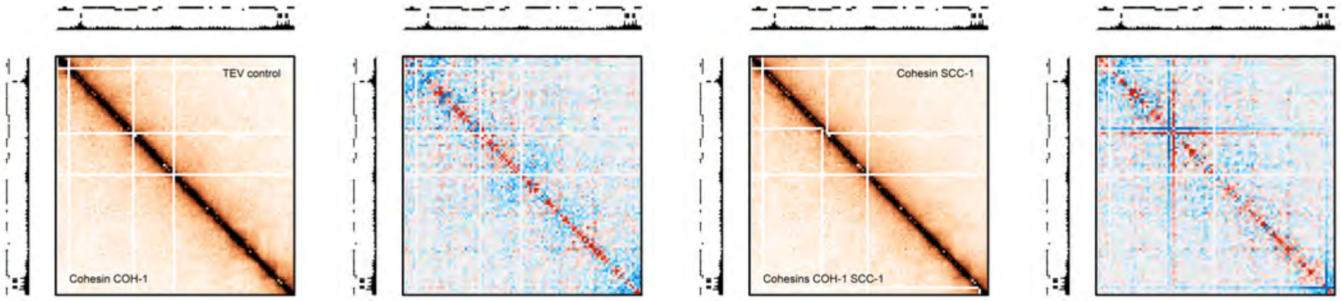

chrIII:9567001-9767000

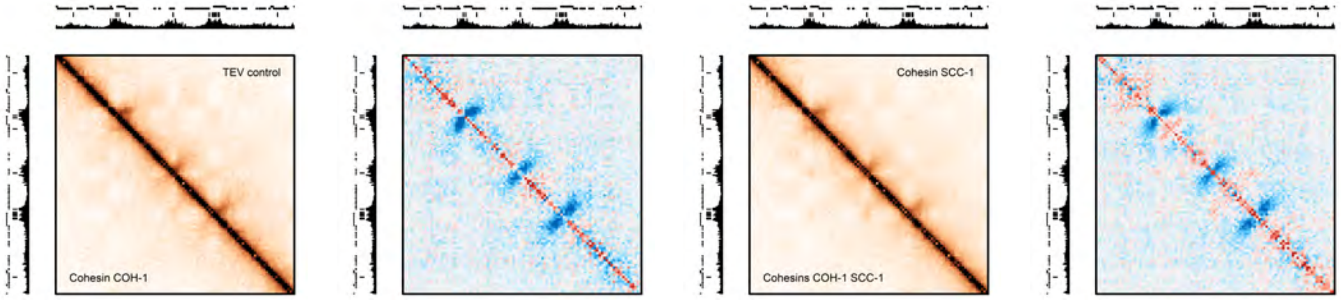

chrIII:2371001-2571000

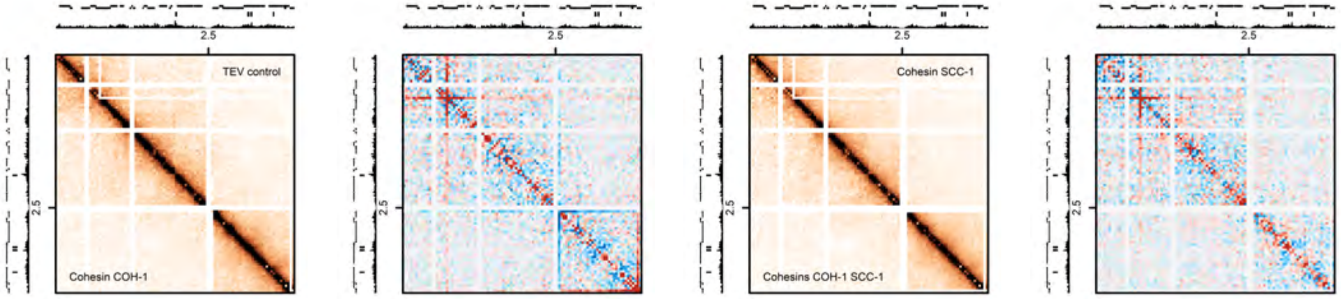

chrIV:14883001-15083000

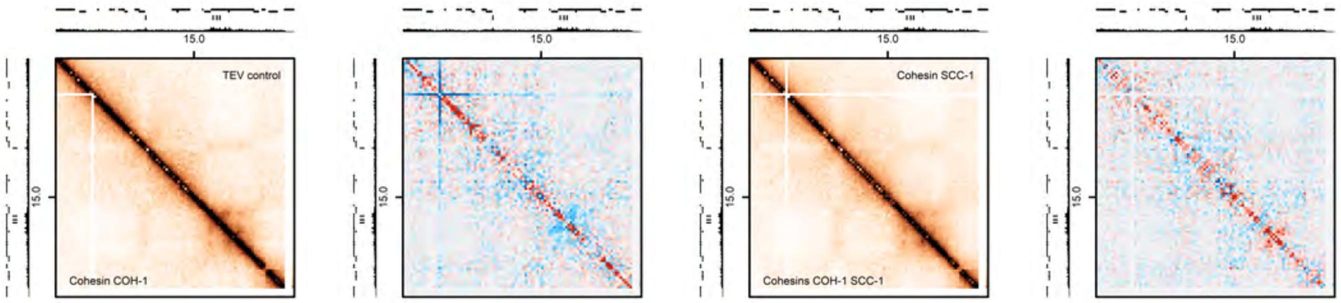

chrIV:7561001-7761000

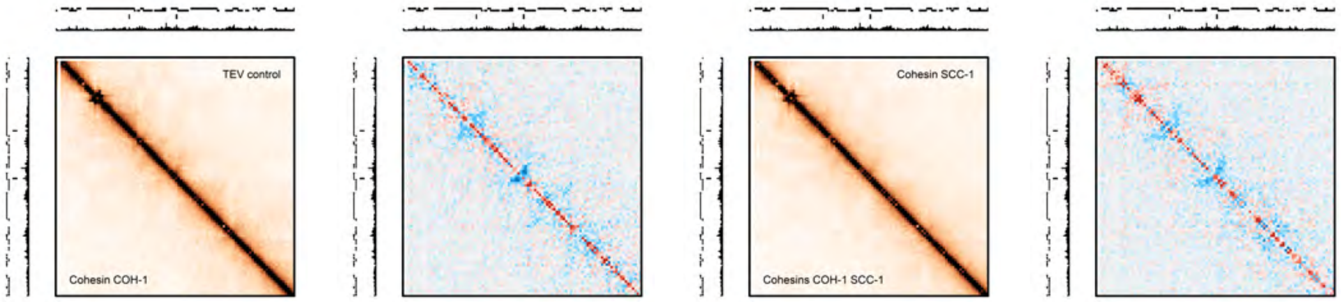

chrX:11311001-11511000

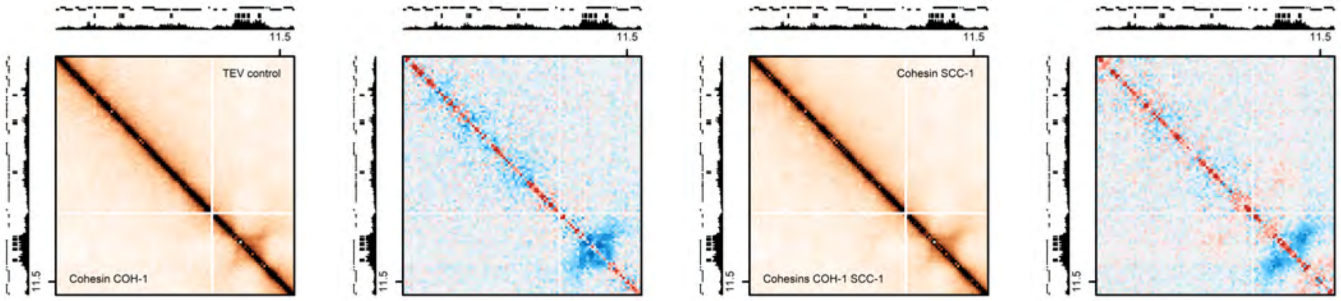

chrV:11923001-12123000

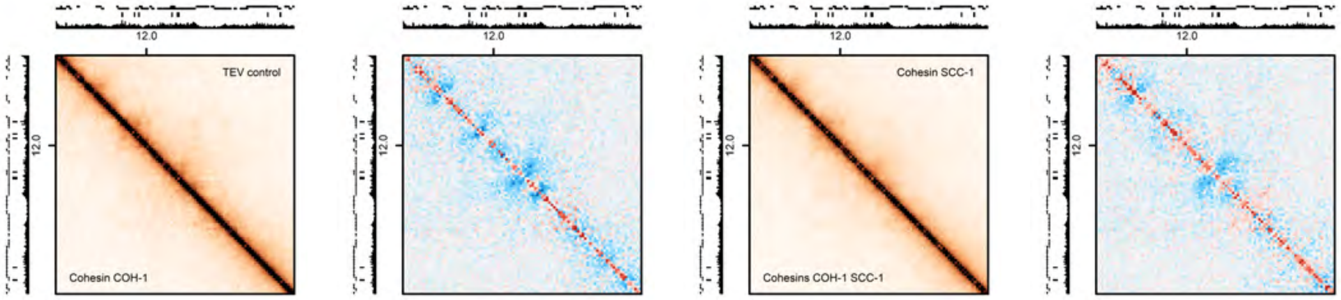

chrII:993001-1193000

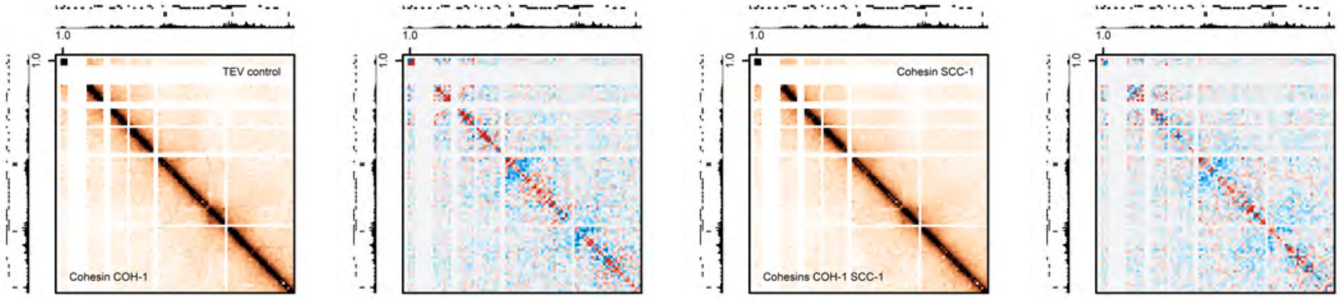

chrII:13233001-13433000

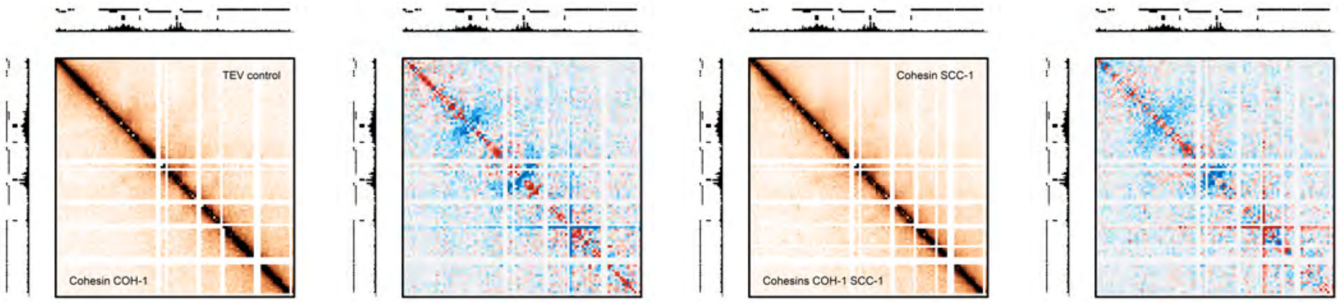

chrII:9975001-10175000

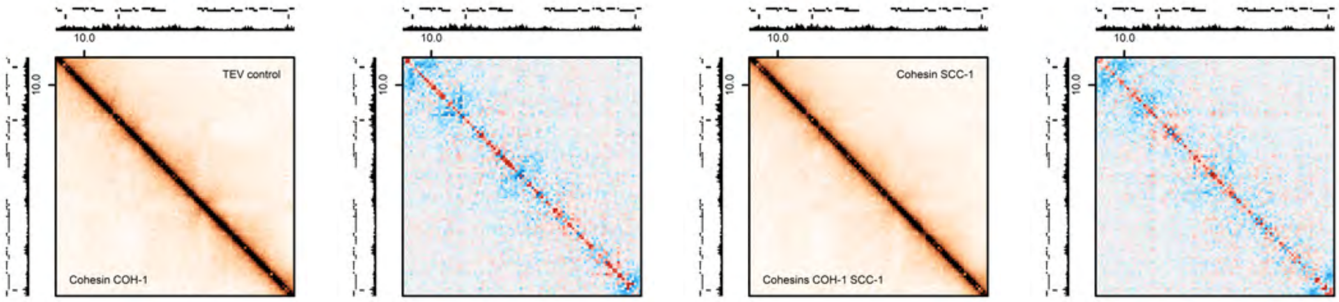

chrX:7855001-8055000

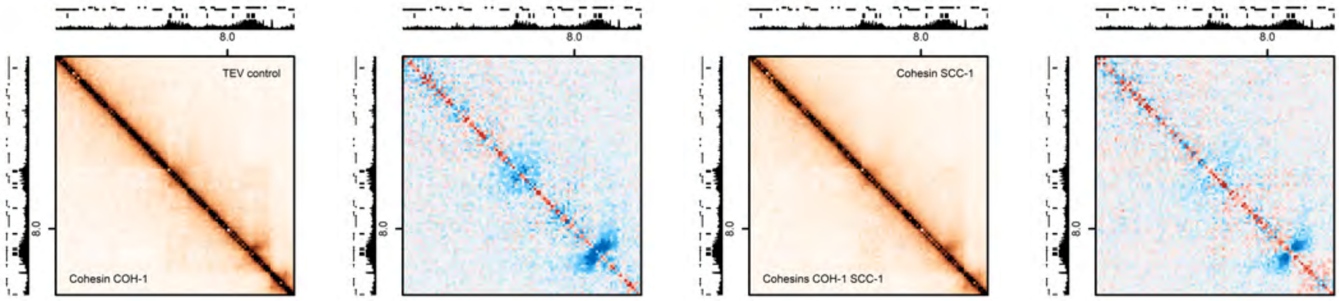

chrI:5445001-5645000

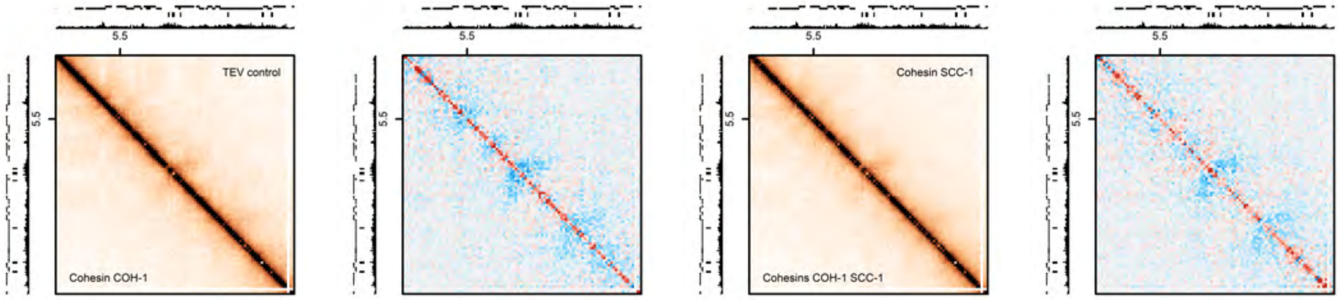

chrX:9121001-9321000

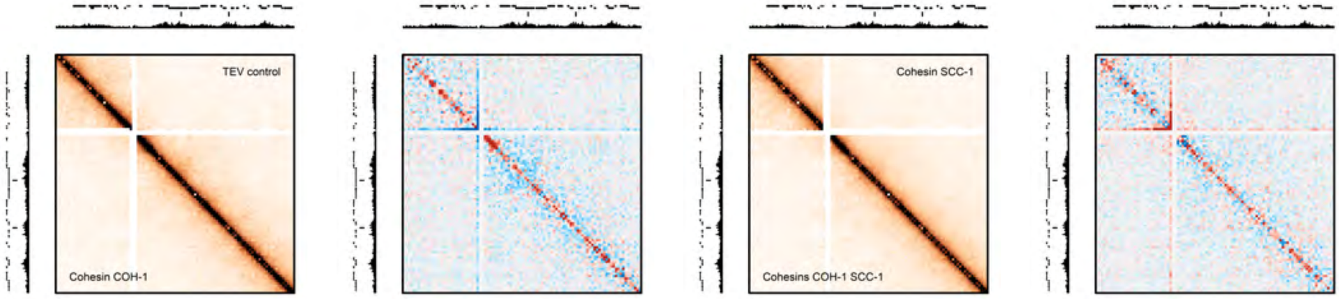

chrIII:3429001-3629000

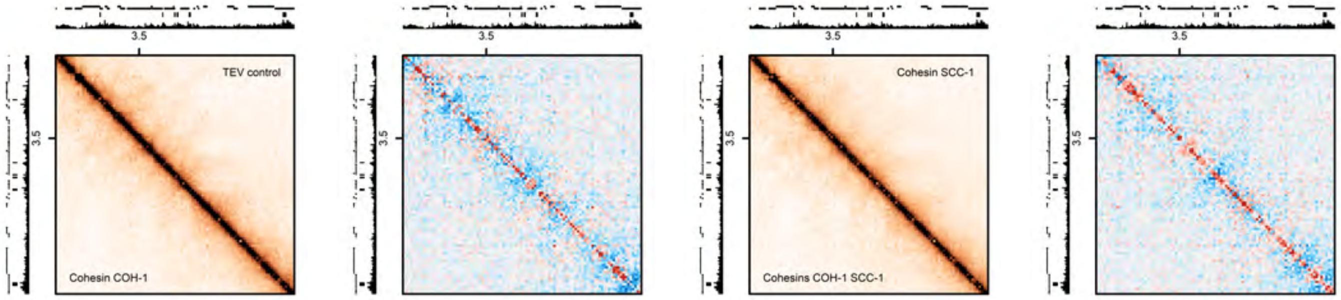

chrII:3715001-3915000

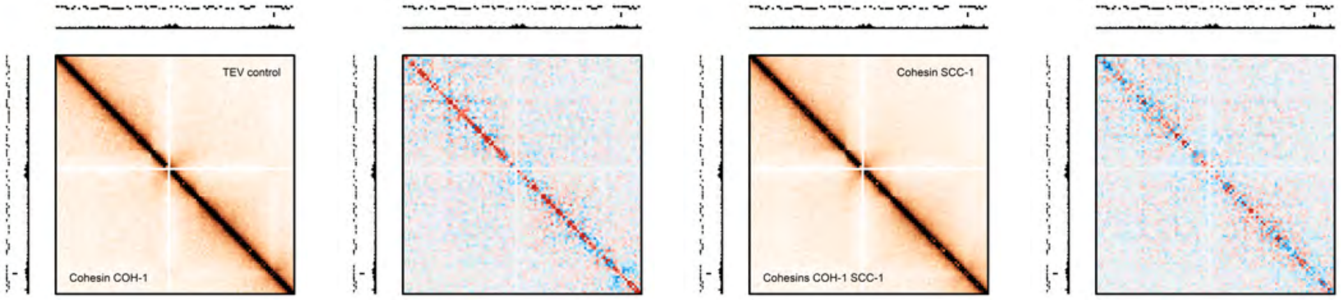

chrII:3993001-4193000

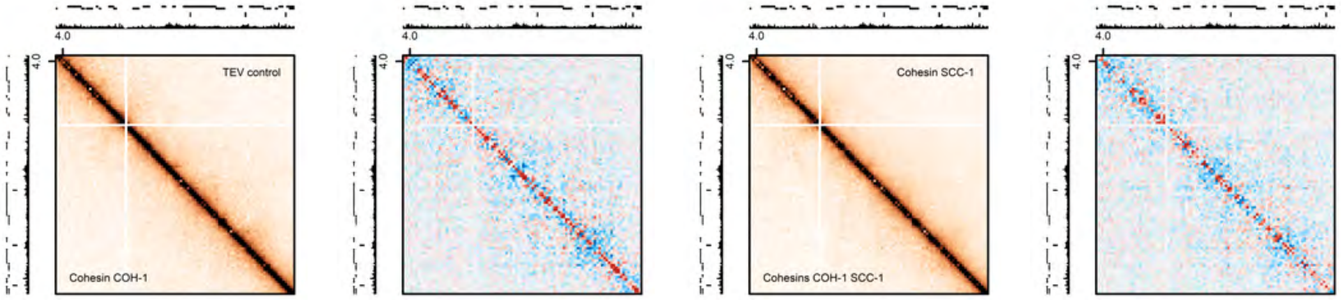

chrIV:4999001-5199000

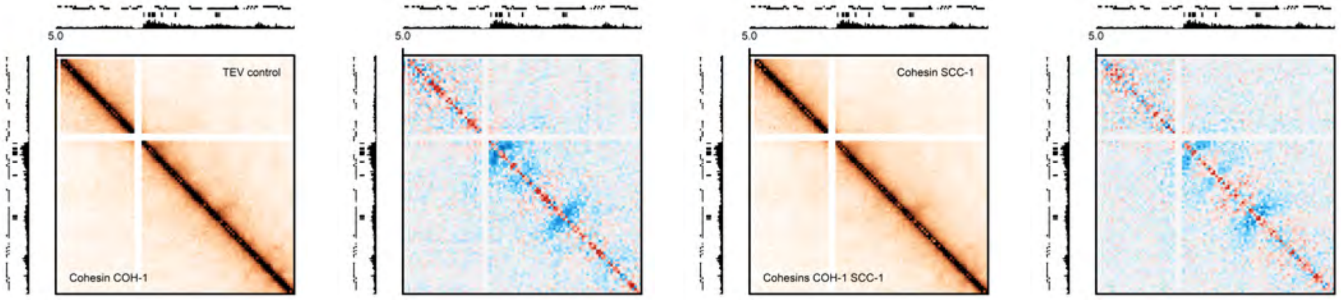

chrX:6859001-7059000

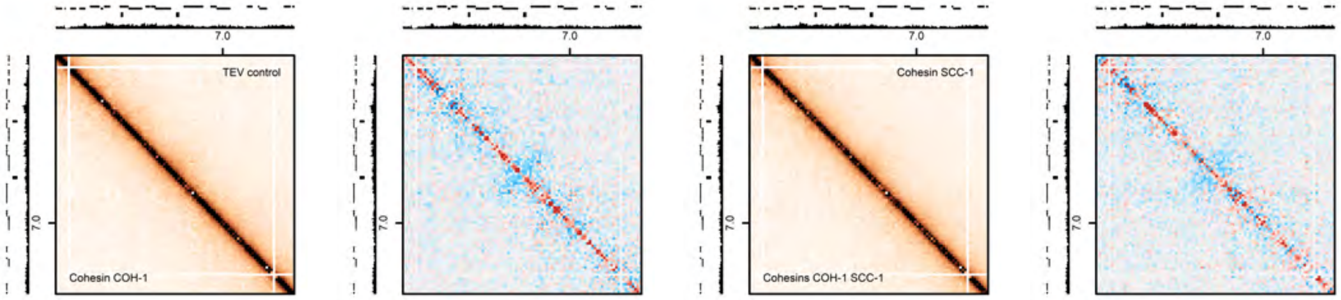

chrII:7183001-7383000

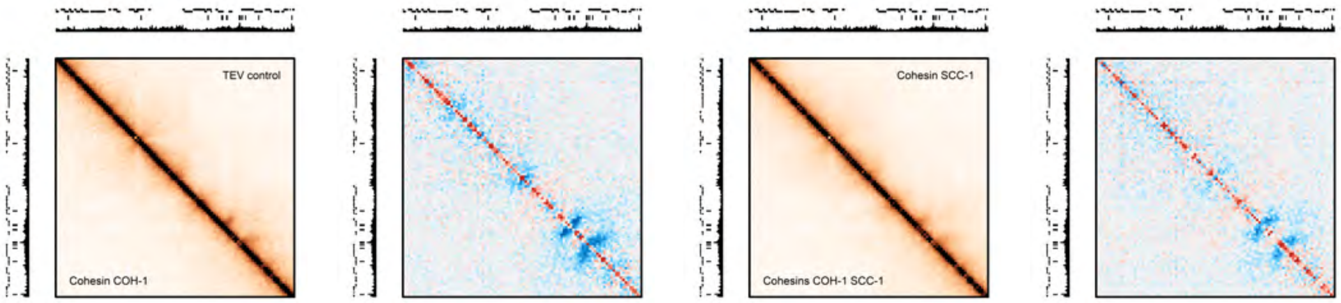

chrII:12163001-12363000

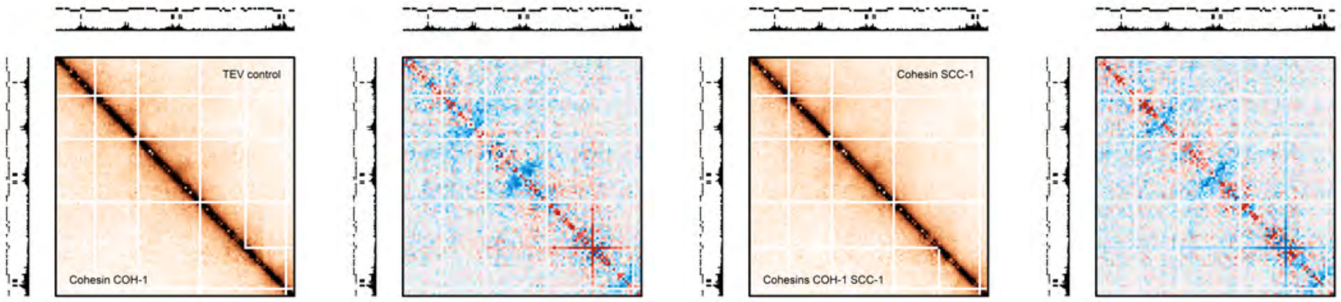

chrV:2031001-2231000

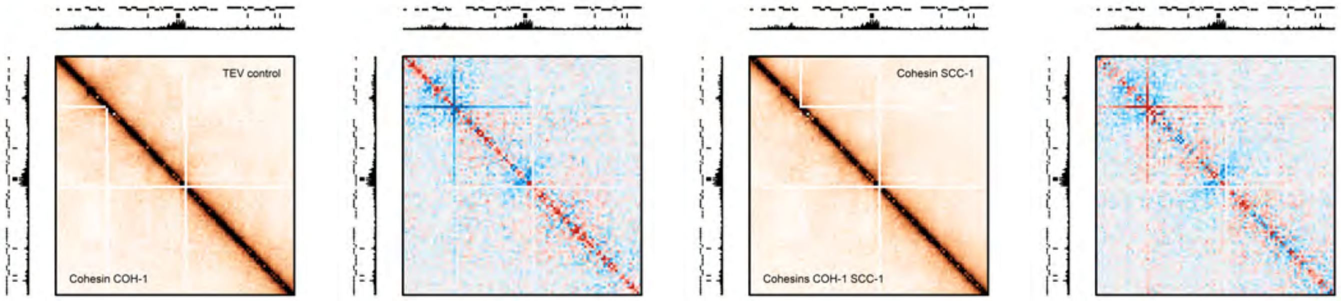

chrIII:5193001-5393000

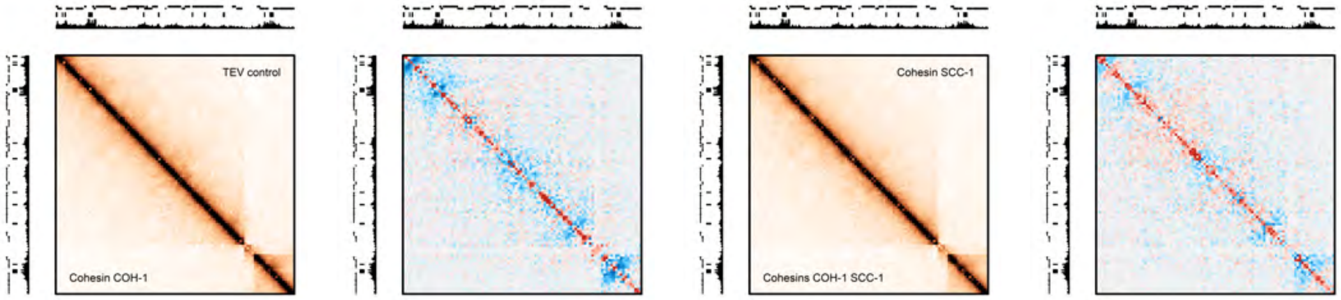

chrI:7499001-7699000

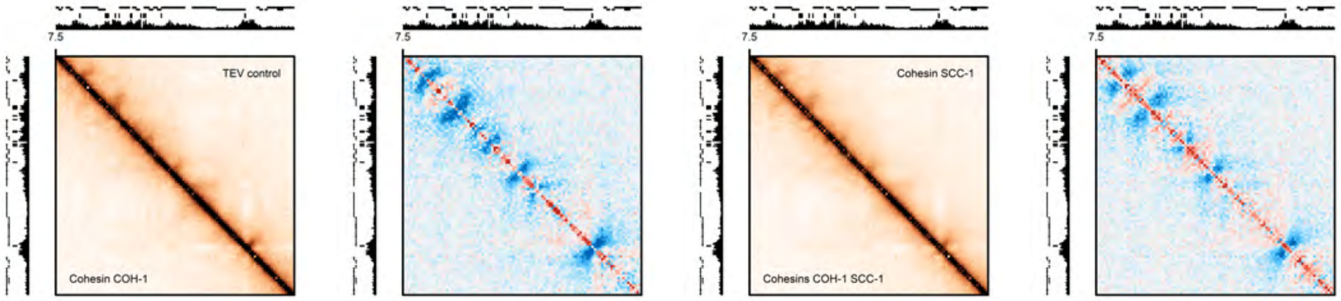

chrIV:11713001-11913000

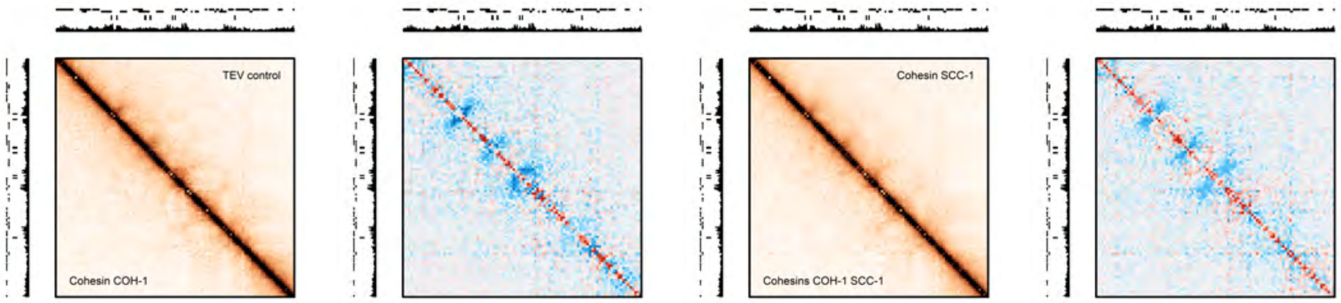

chrIV:13153001-13353000

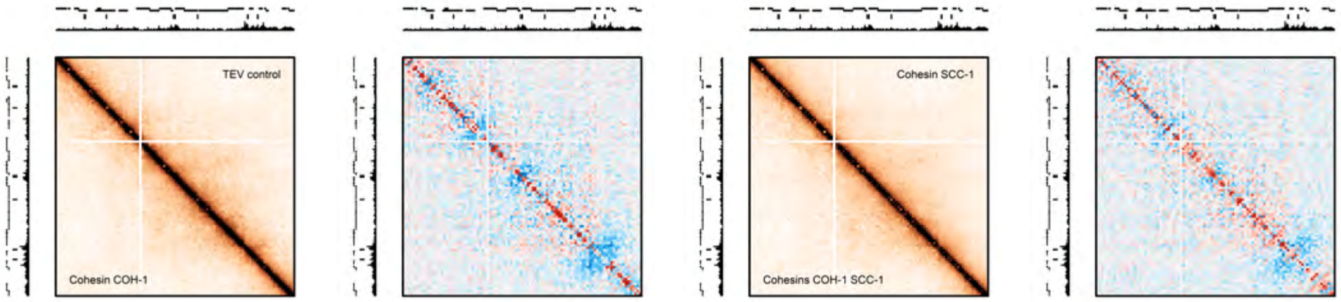

chrII:7003001-7203000

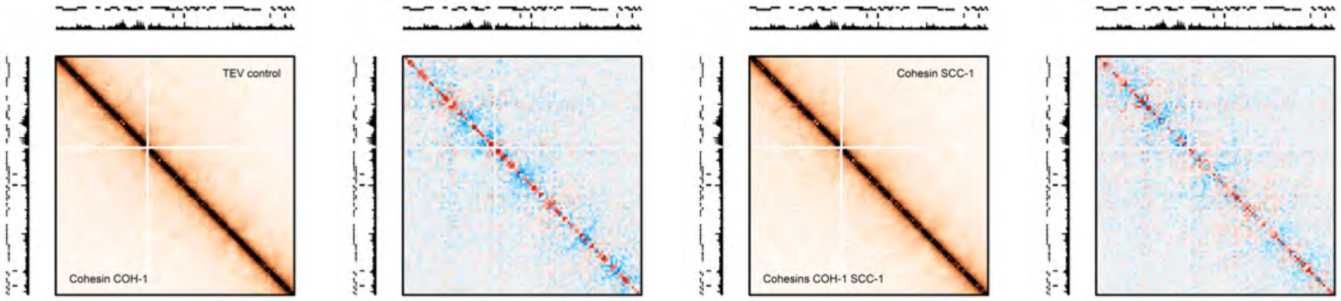

chrI:11275001-11475000

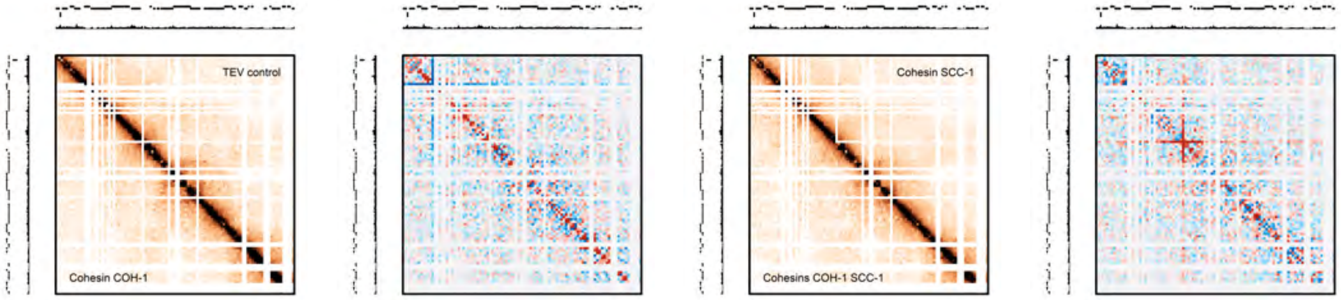

chrX:13925001-14125000

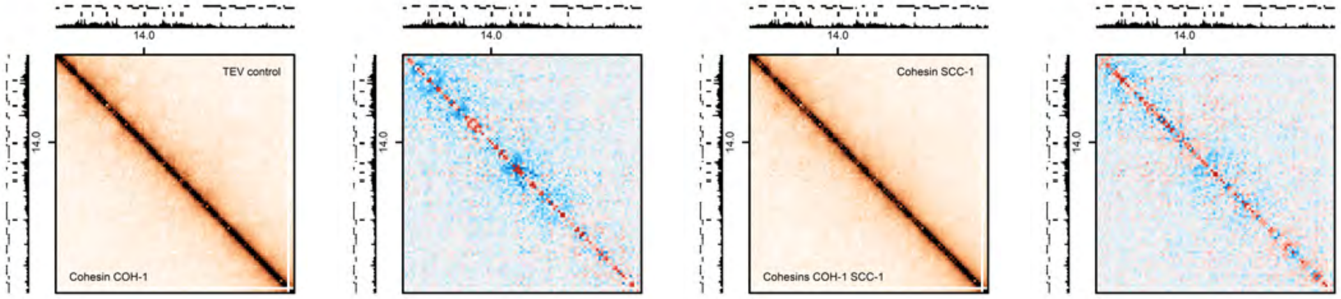

chrIII:8635001-8835000

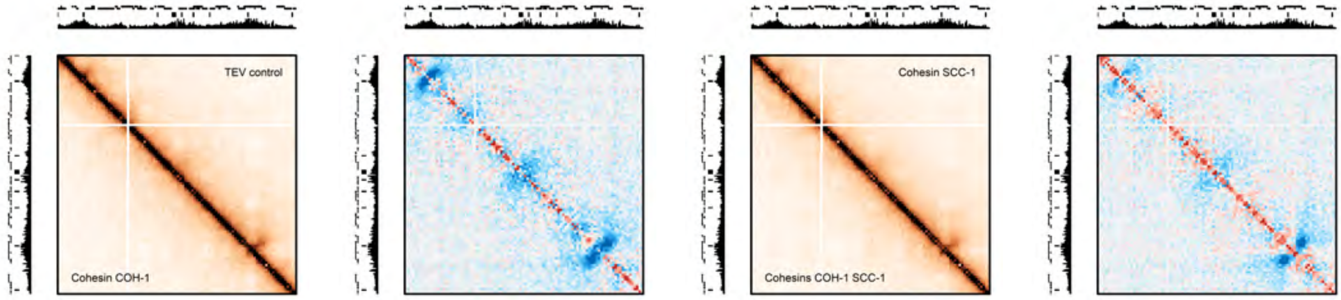

chrI:13543001-13743000

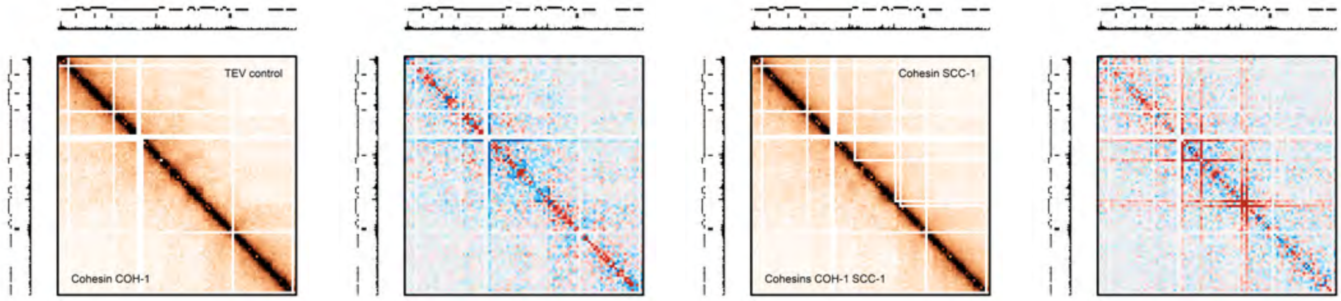

chrX:12907001-13107000

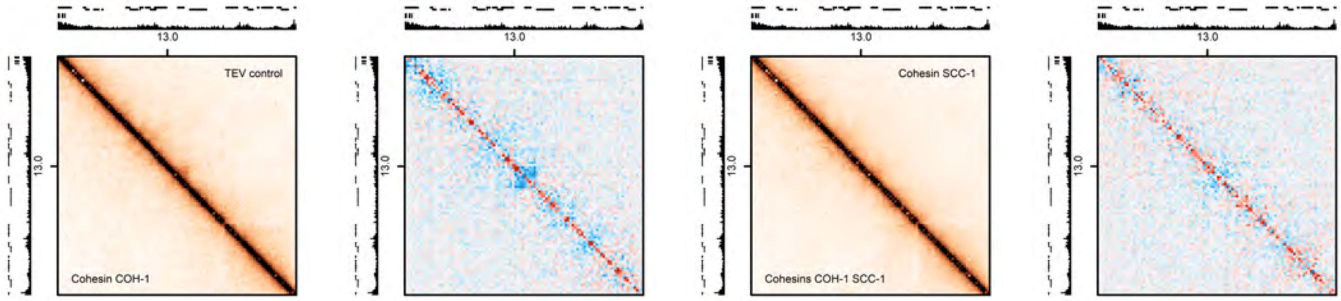

chrI:5727001-5927000

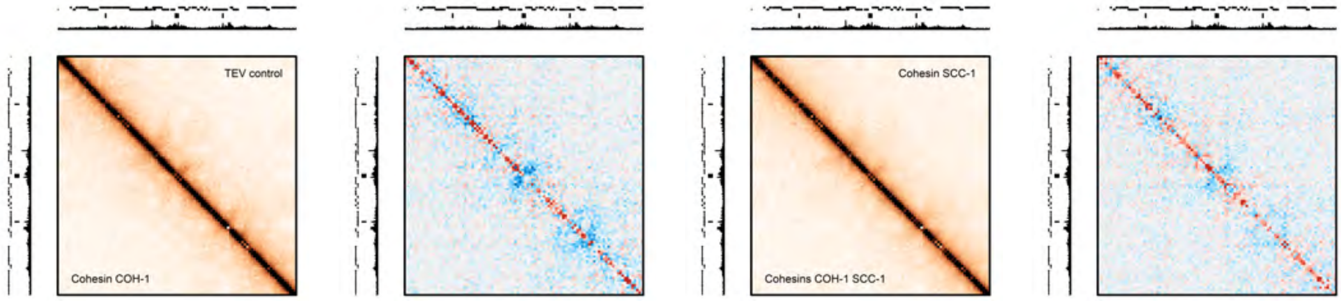

chrX:17325001-17525000

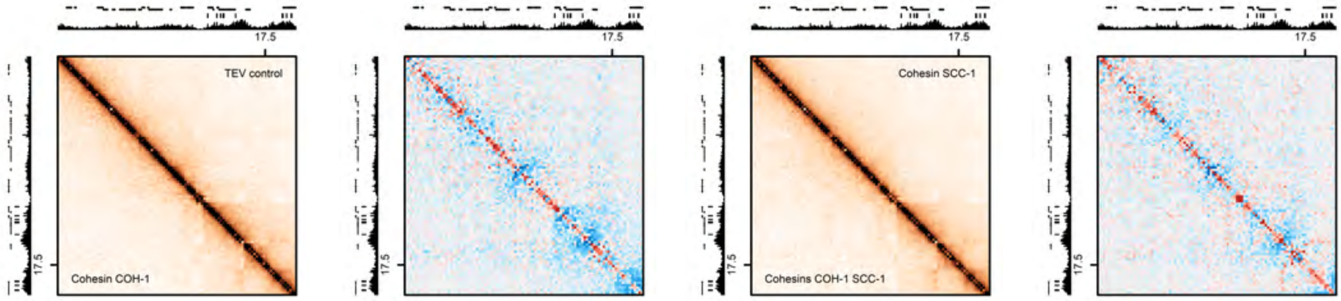

chrI:11897001-12097000

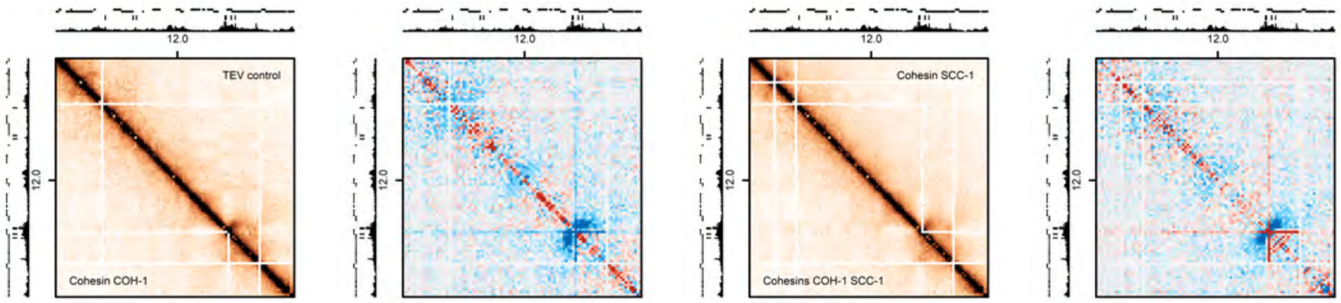

chrII:7739001-7939000

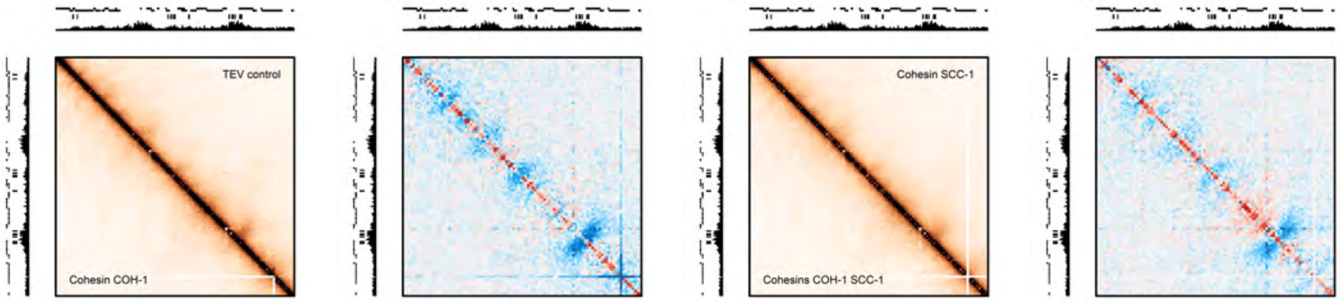

chrV:13067001-13267000

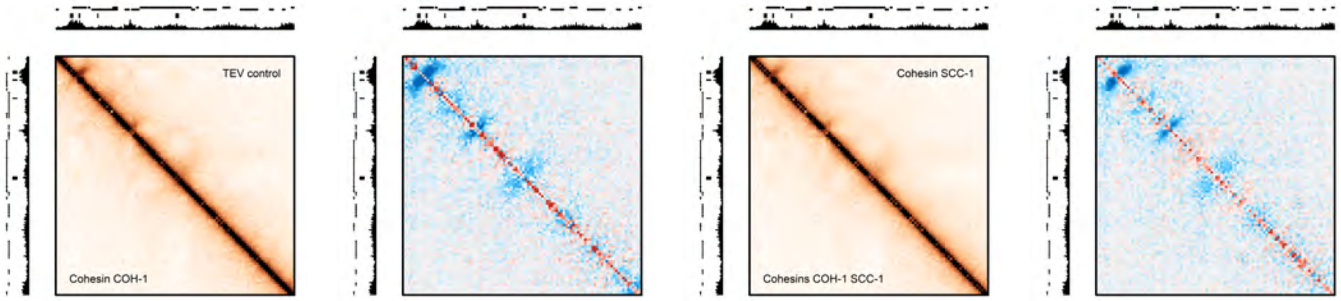

chrIII:1639001-1839000

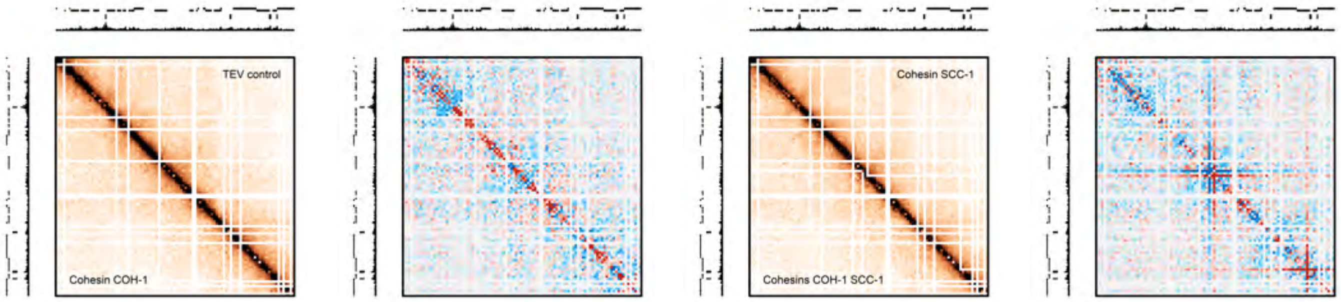

chrX:3499001-3699000

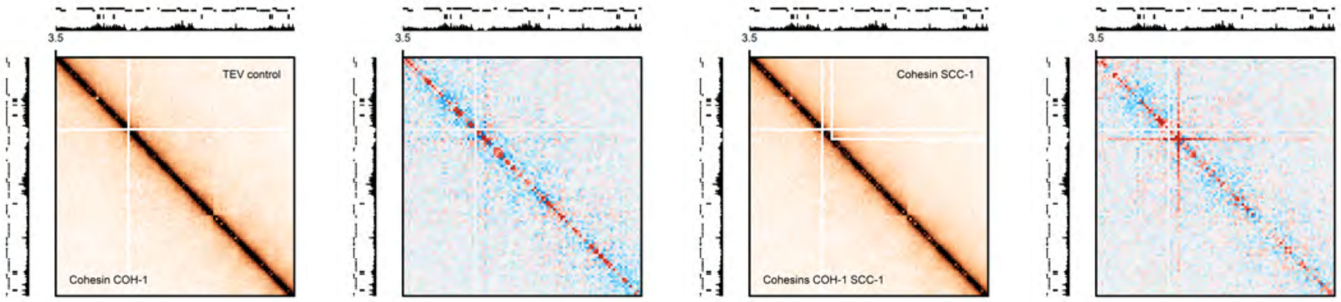

chrIII:4979001-5179000

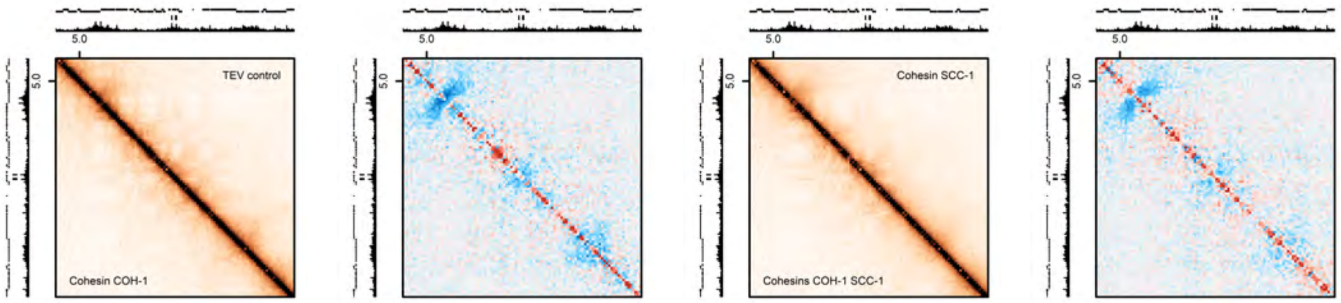

chrX:16305001-16505000

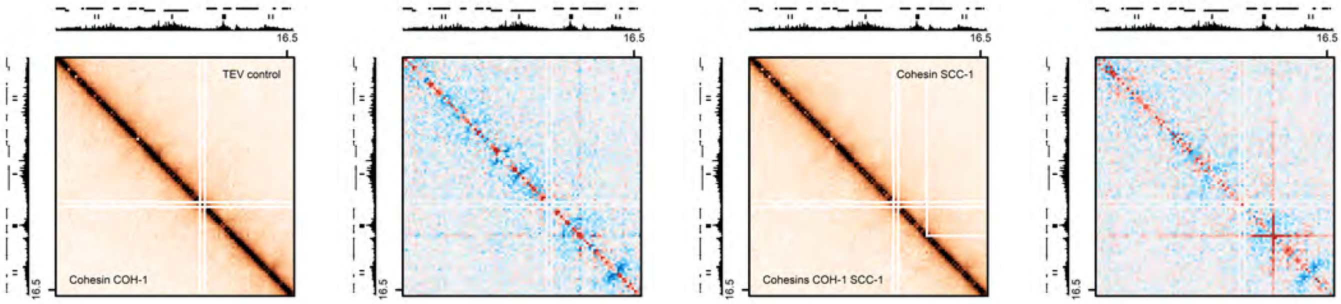

chrI:9507001-9707000

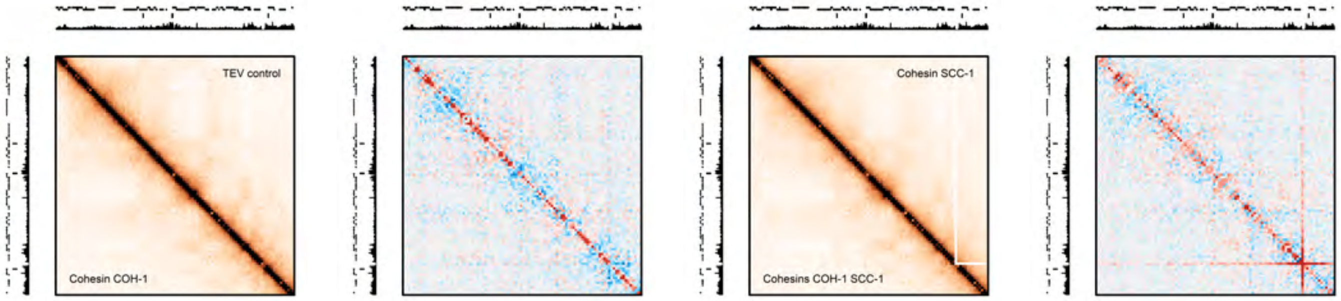

chrV:19521001-19721000

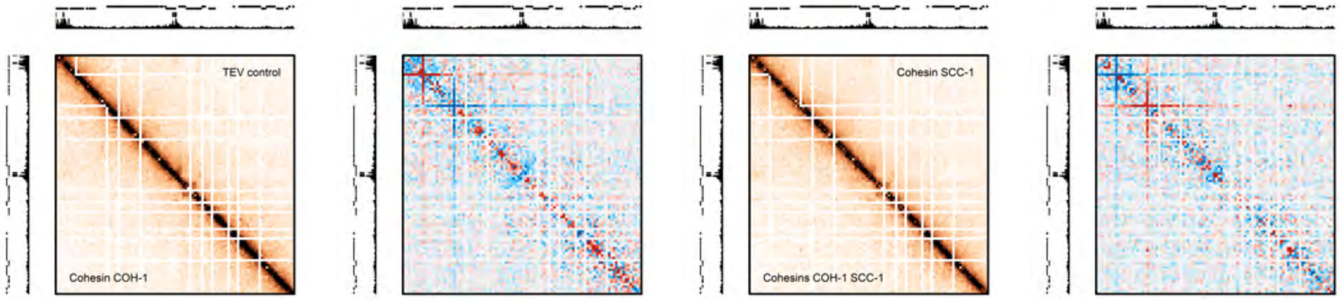

chrIII:571001-771000

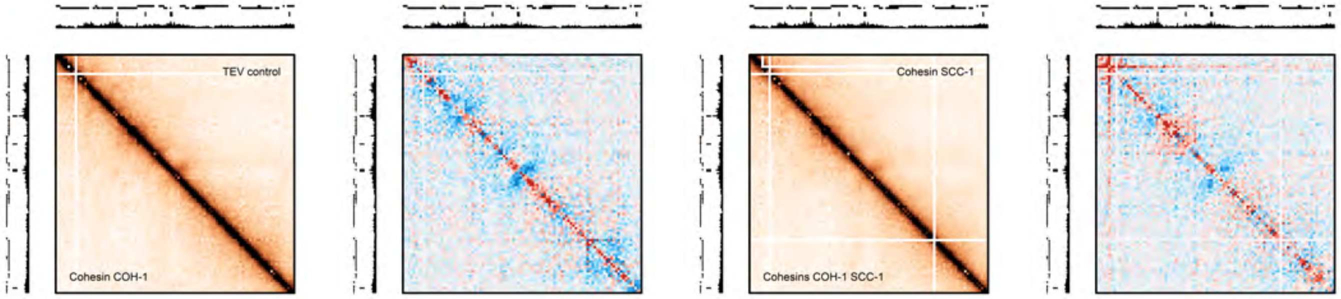

chrIV:7795001-7995000

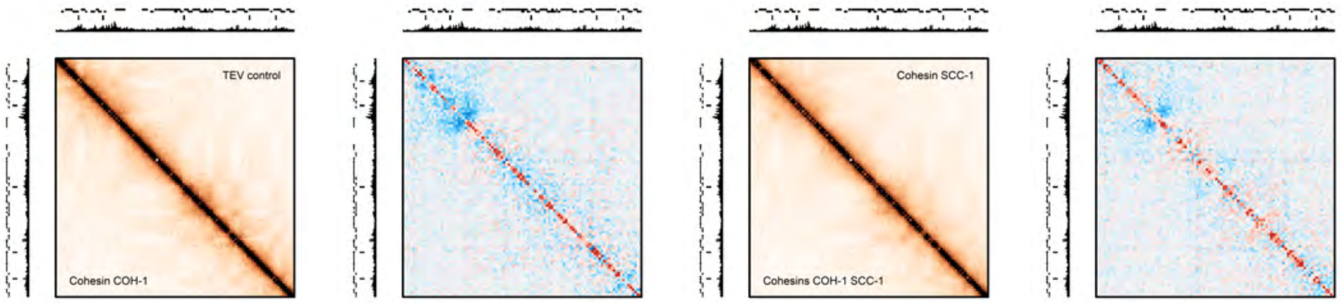

chrV:3179001-3379000

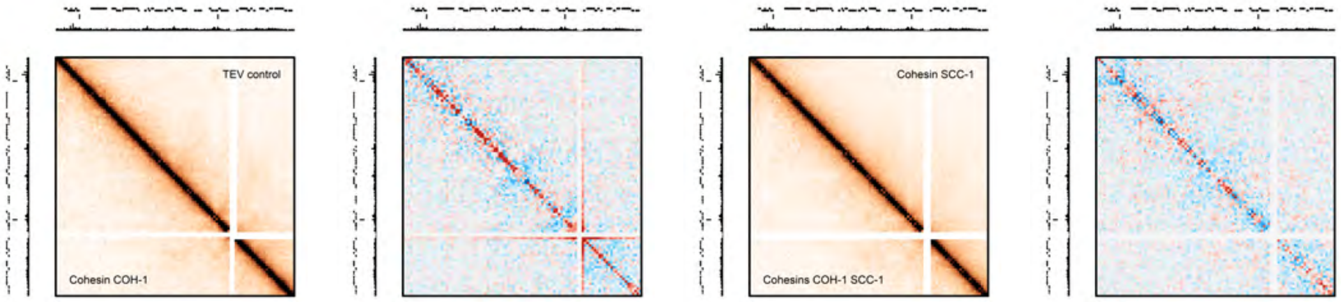

chrII:8831001-9031000

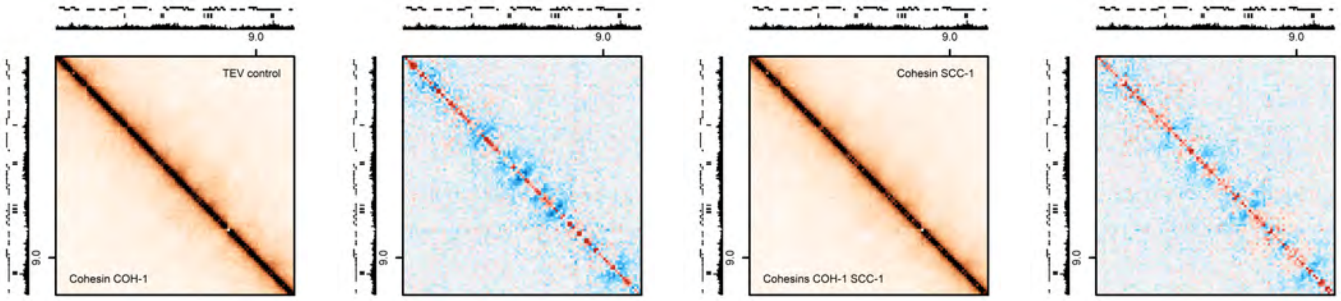

chrI:1129001-1329000

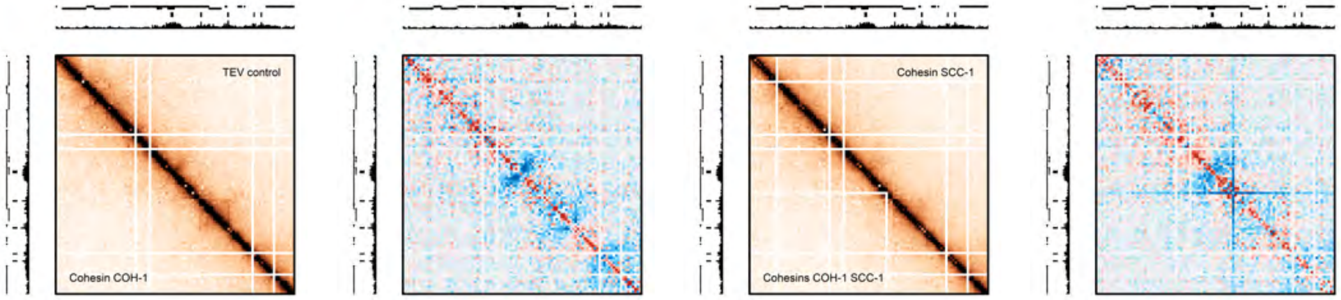

chrII:13723001-13923000

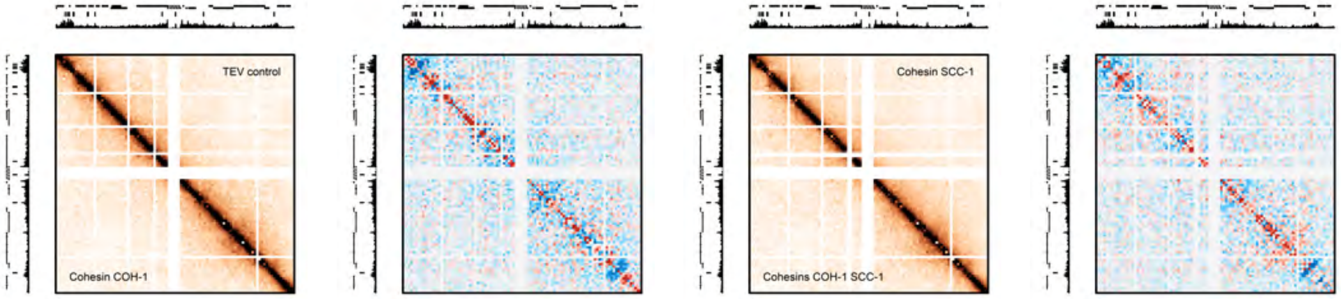

chrII:2823001-3023000

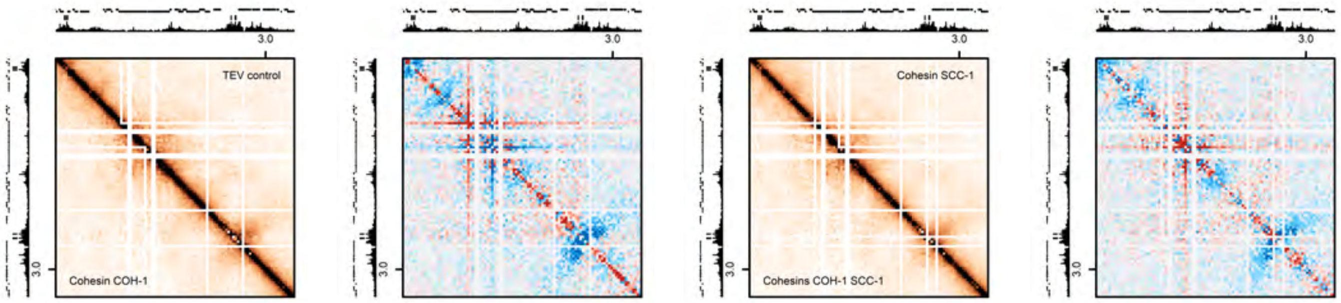

chrI:8583001-8783000

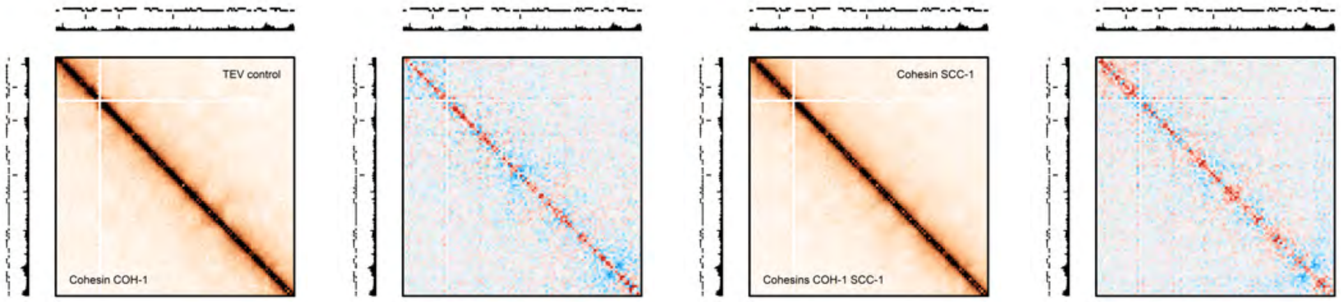

chrI:14943001-15143000

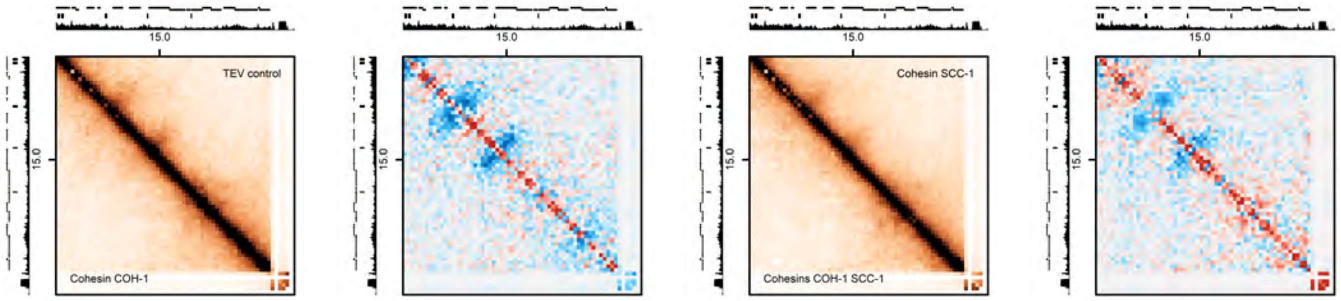

chrII:11229001-11429000

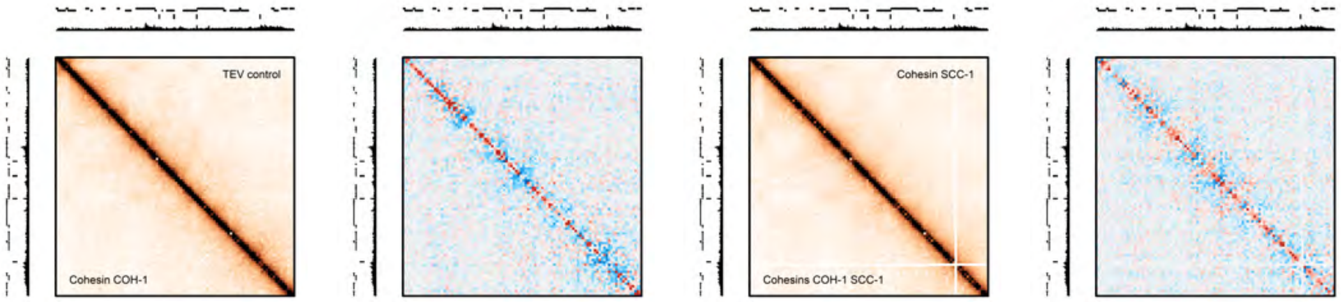

chrII:659001-859000

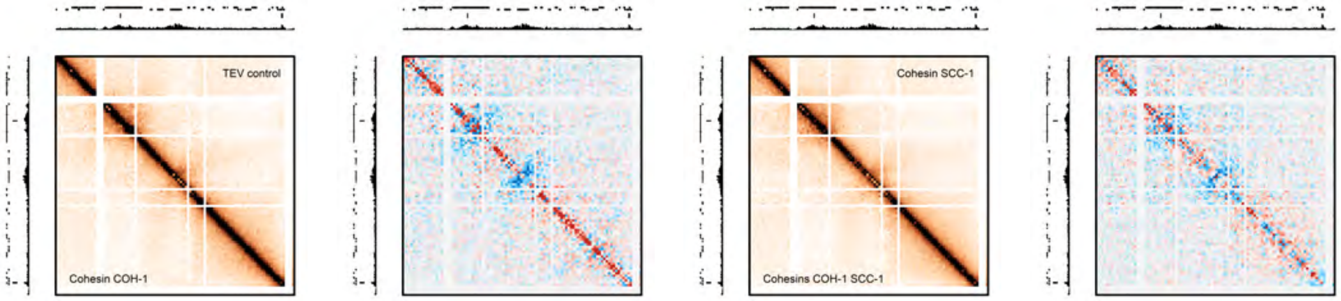

chrV:17807001-18007000

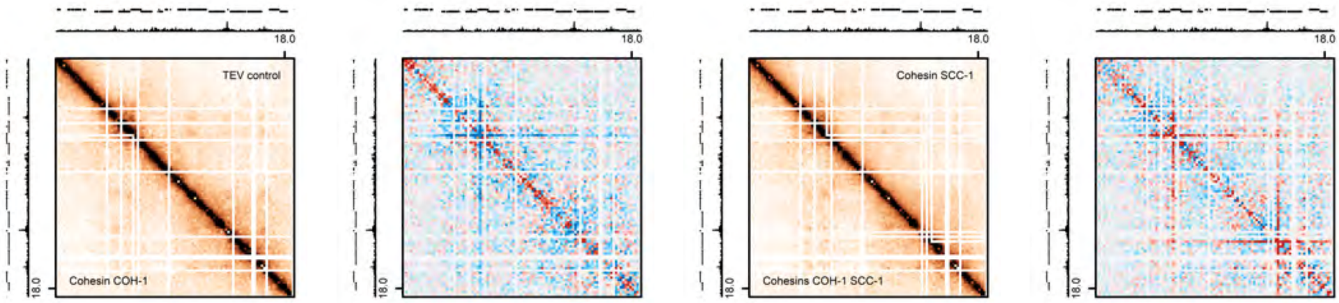

chrI:749001-949000

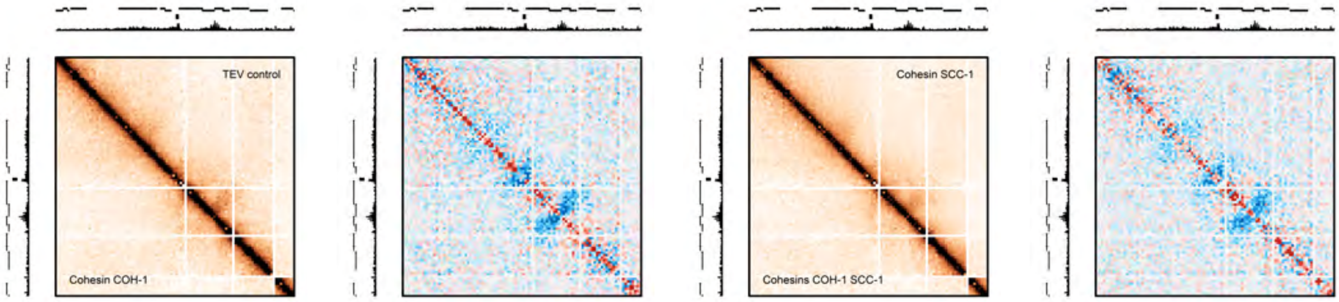

chrX:10805001-11005000

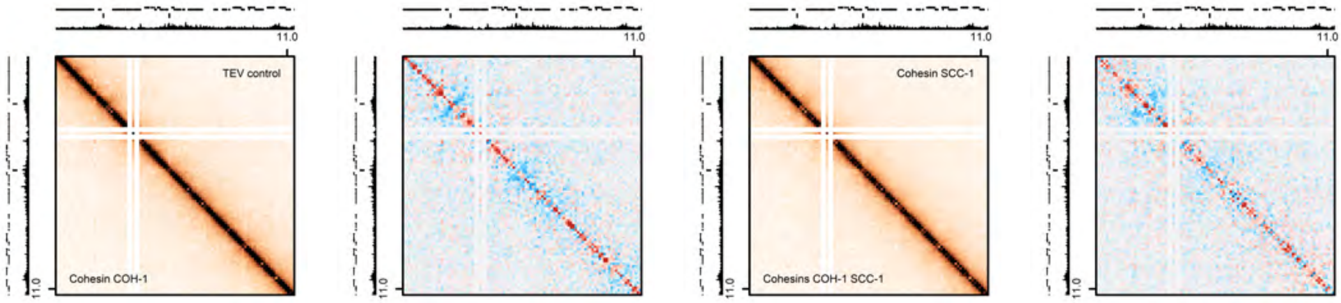

chrX:12775001-12975000

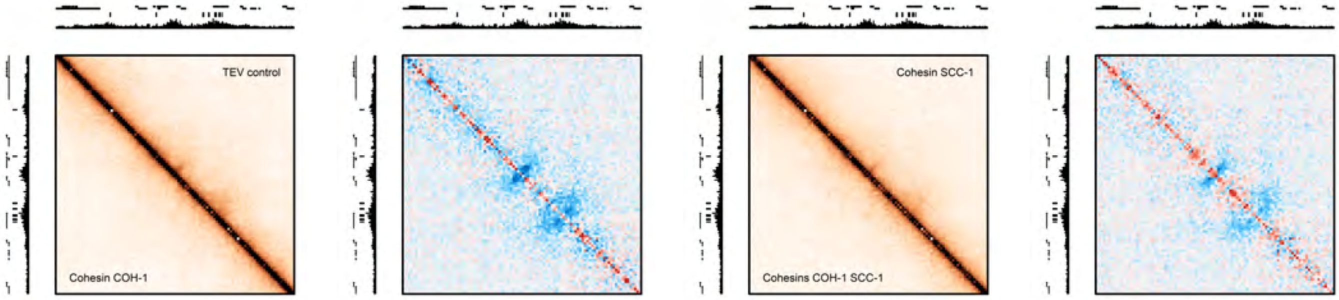

chrV:1245001-1445000

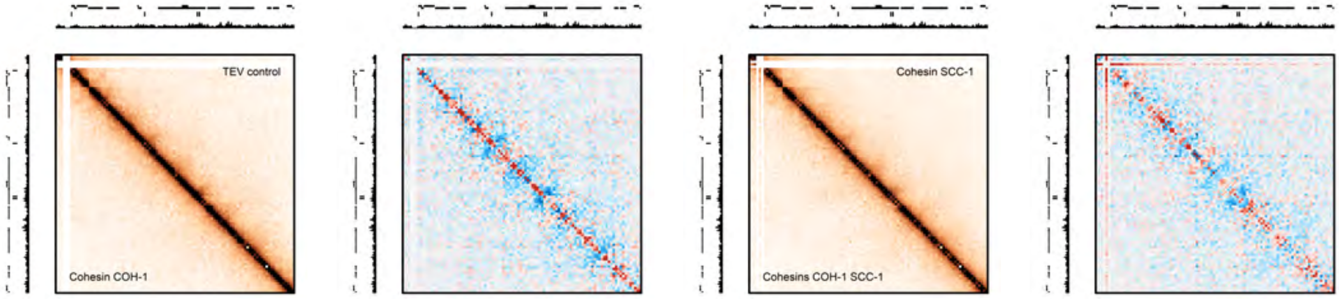

chrX:8465001-8665000

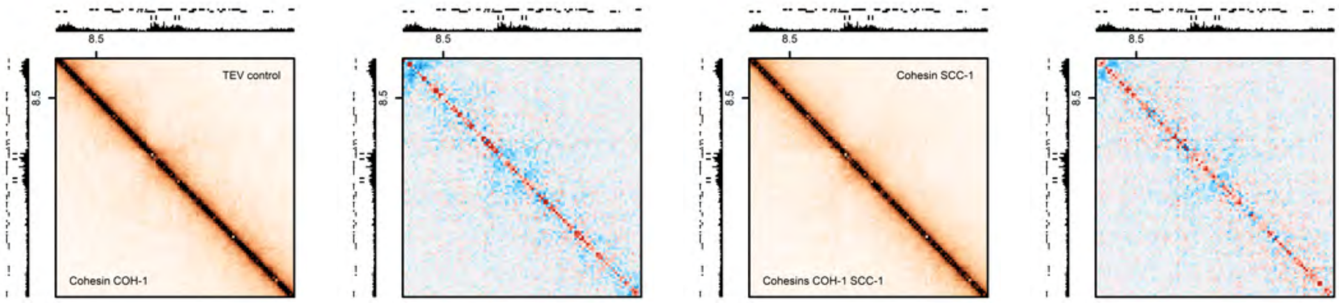

chrIV:9701001-9901000

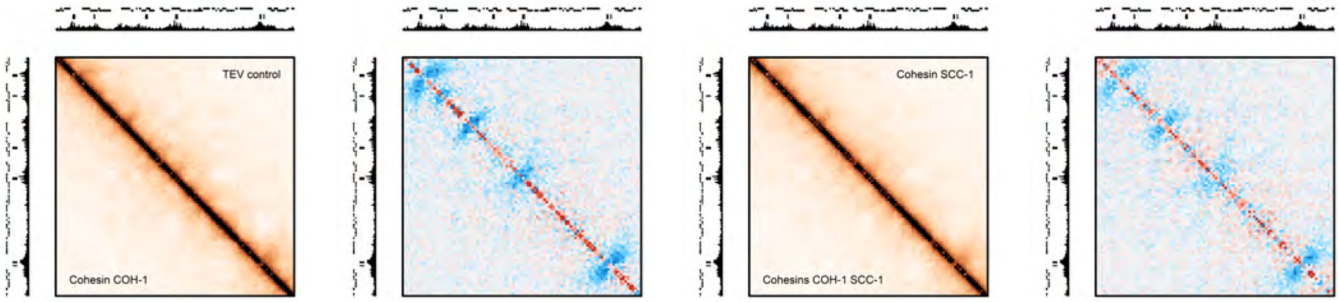

chrI:14039001-14239000

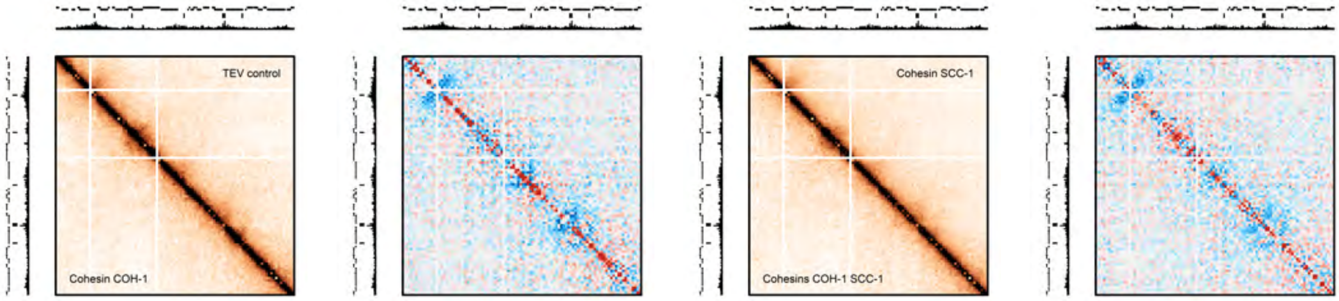

chrIII:4851001-5051000

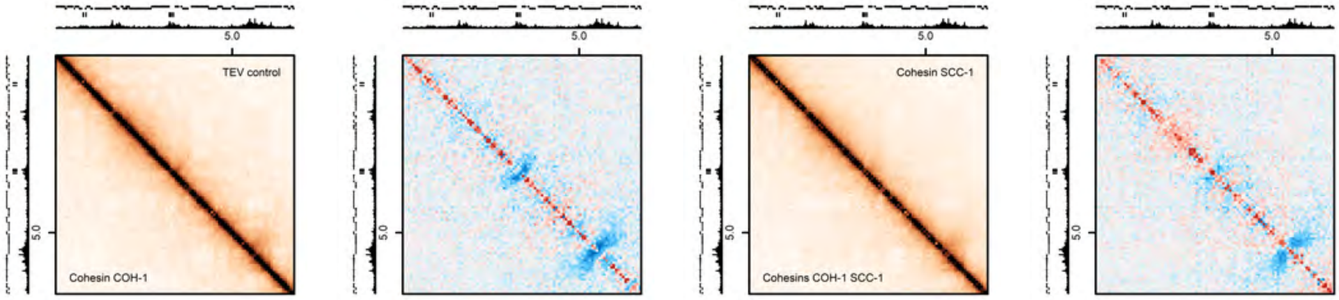

chrII:14973001-15173000

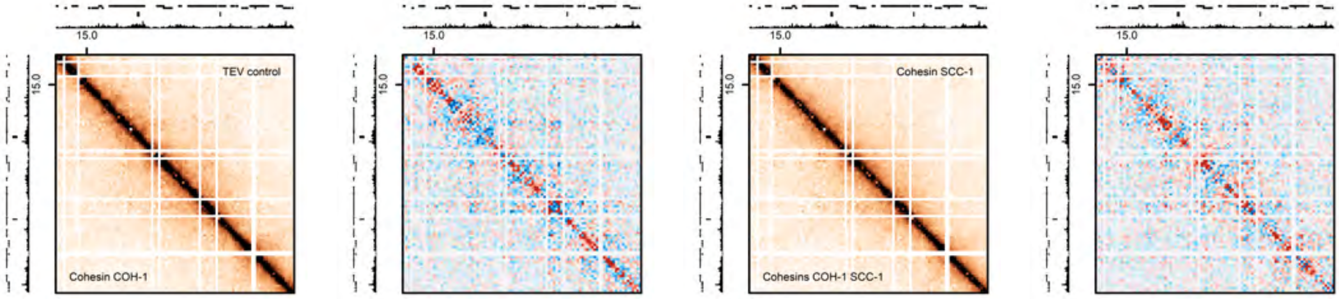

chrX:15089001-15289000

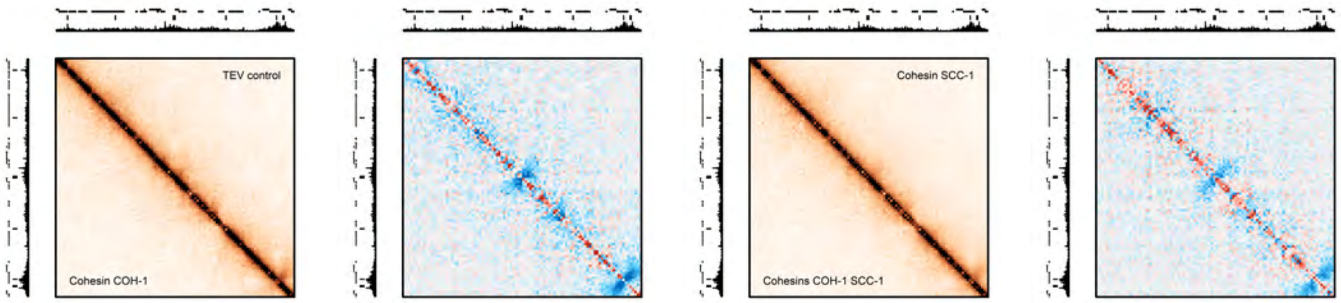

chrII:4705001-4905000

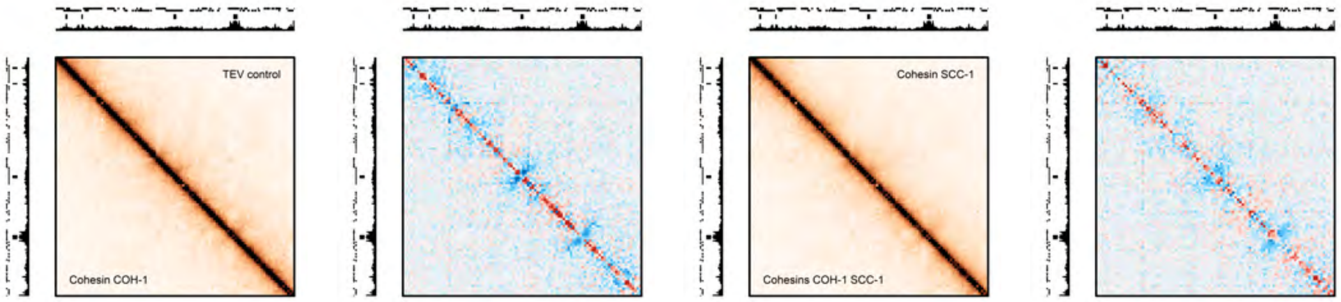

chrX:9741001-9941000

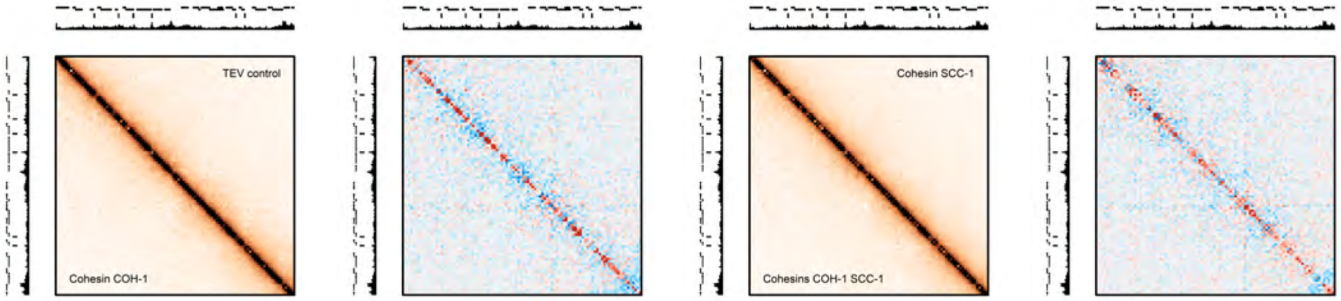

chrII:14569001-14769000

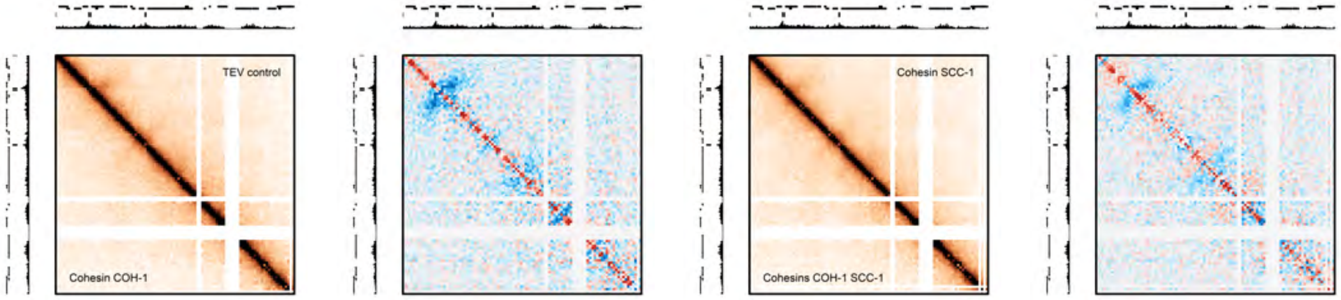

chrIII:645001-845000

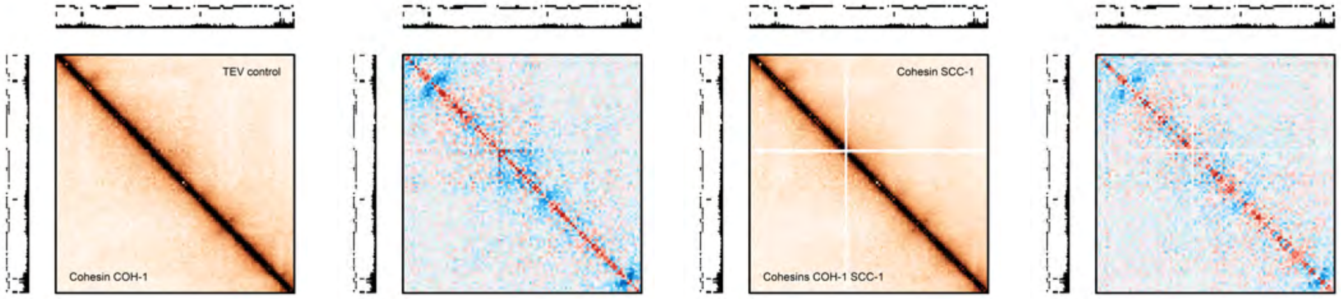

chrI:7849001-8049000

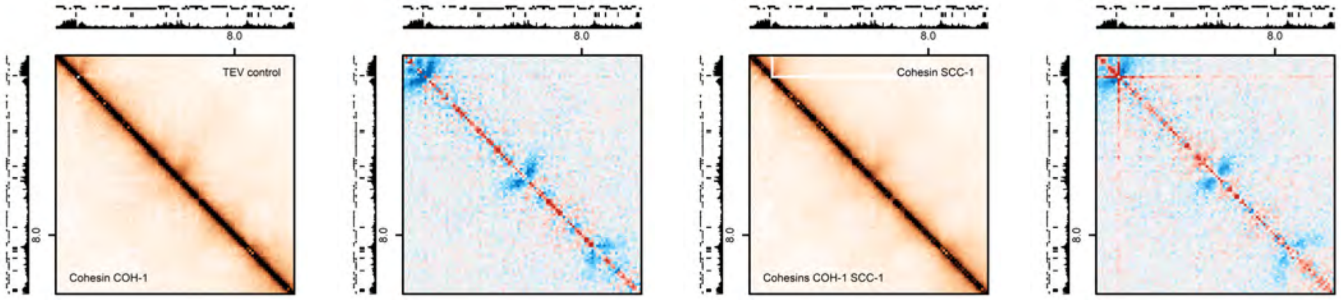

chrIII:11459001-11659000

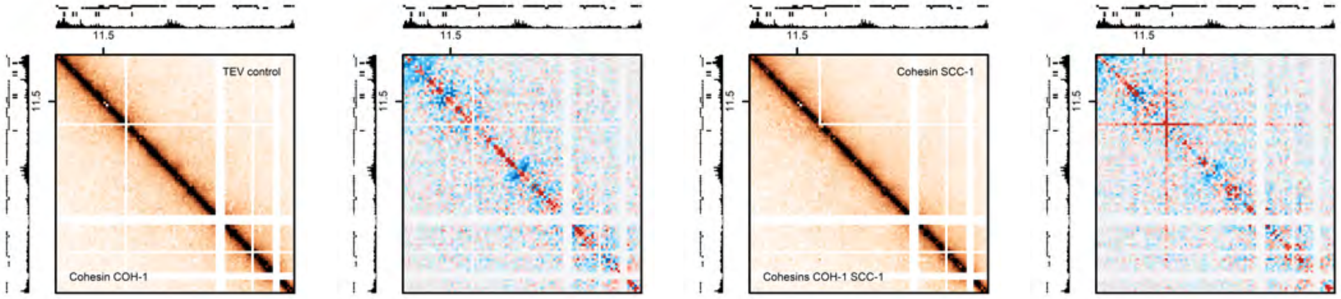

chrX:15607001-15807000

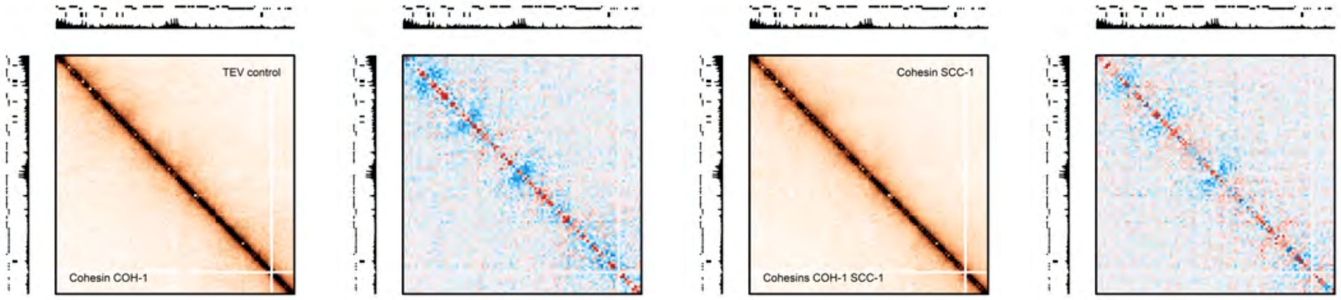

chrIII:9807001-10007000

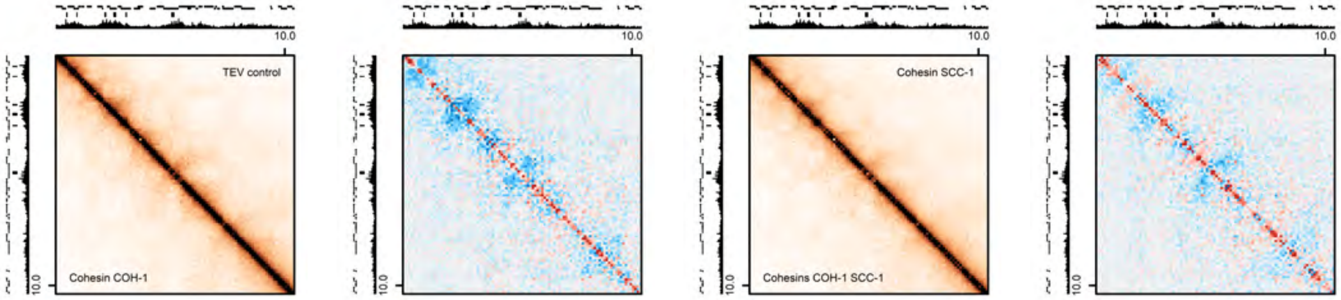

chrIV:5719001-5919000

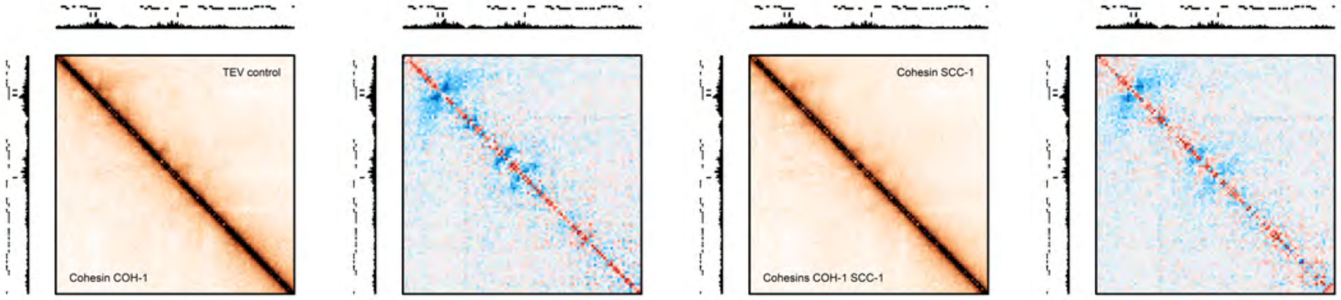

chrX:49001-249000

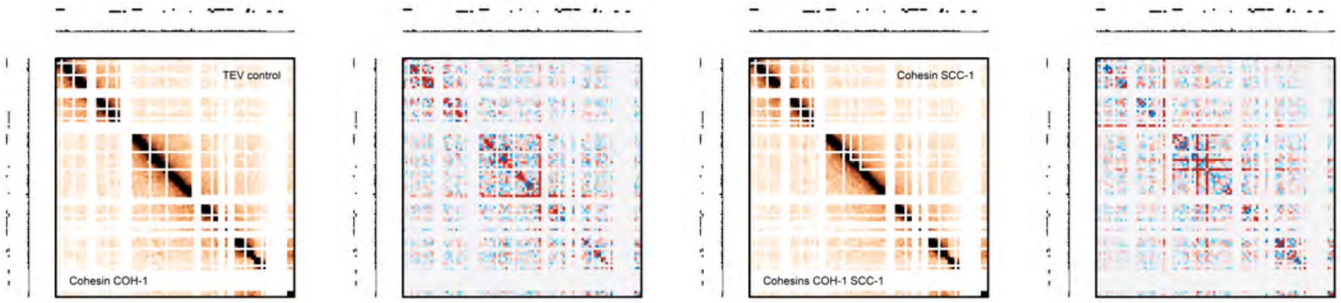

chrII:10901001-11101000

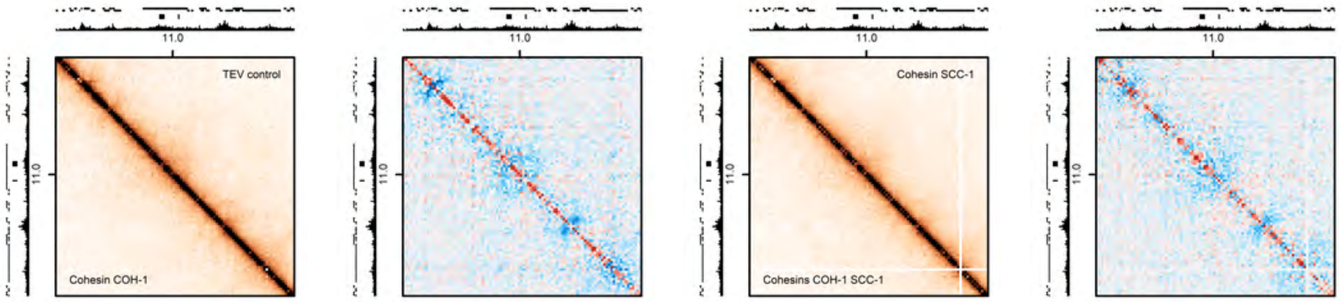

chrX:13151001-13351000

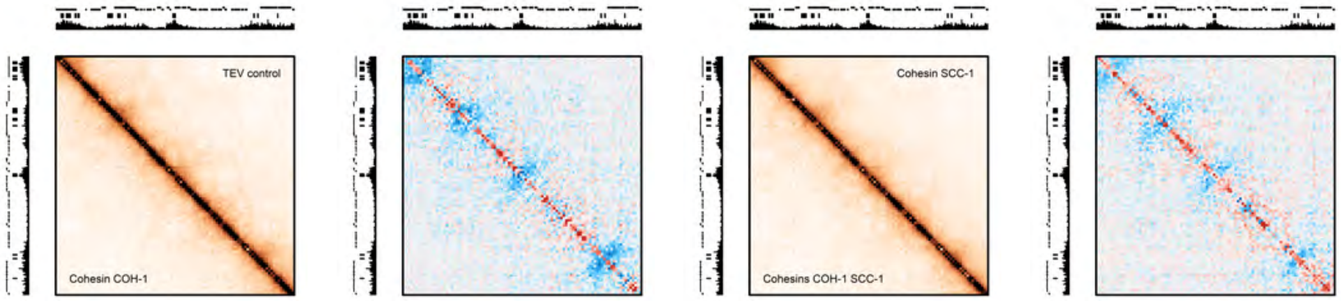

chrX:6421001-6621000

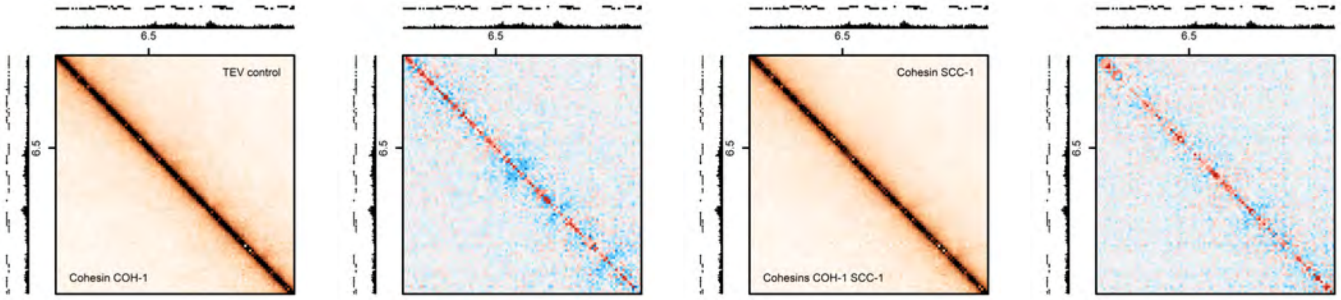

chrX:4711001-4911000

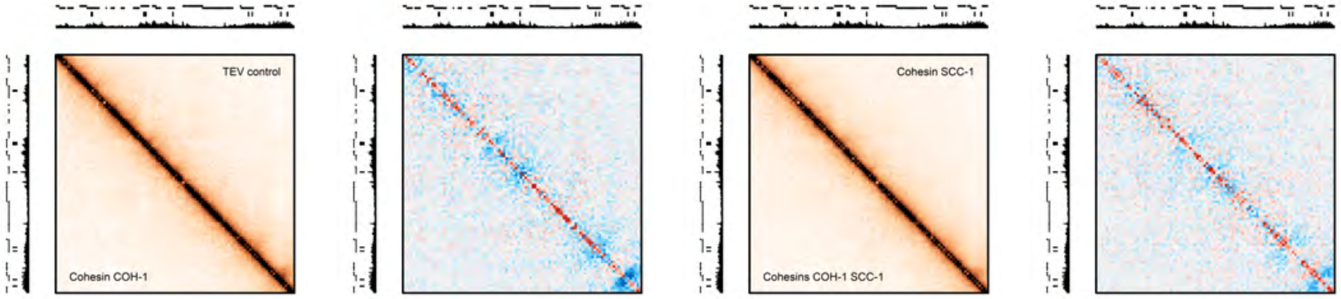

chrIV:8079001-8279000

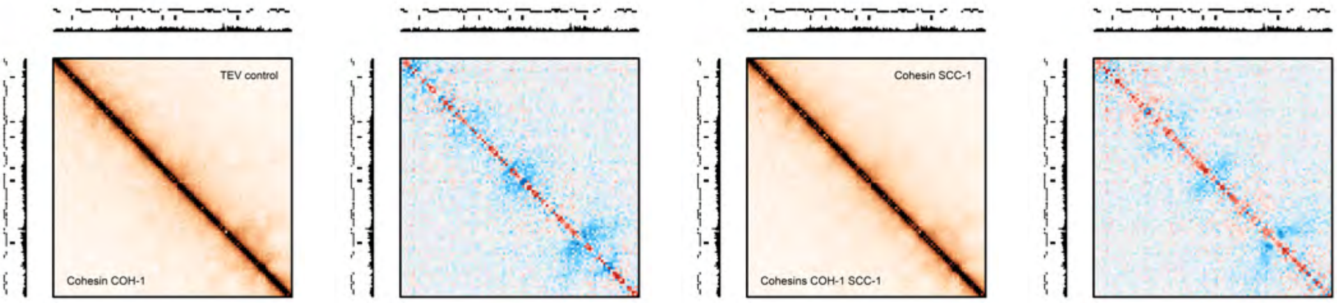

chrII:4293001-4493000

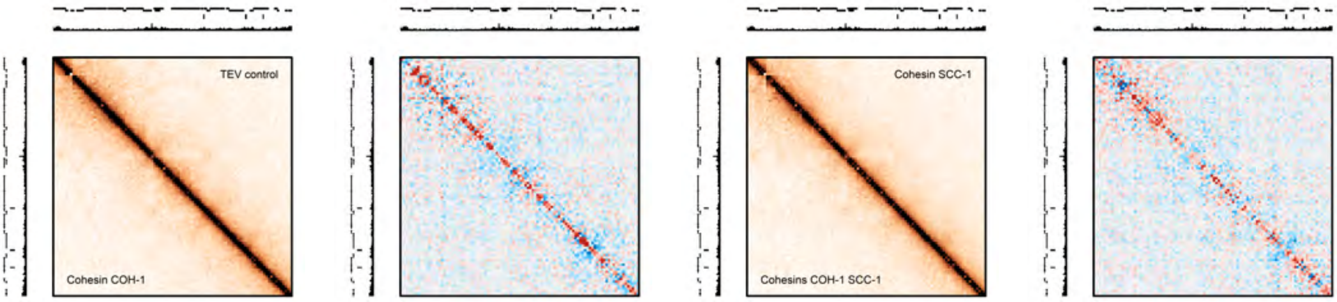

chrIV:10473001-10673000

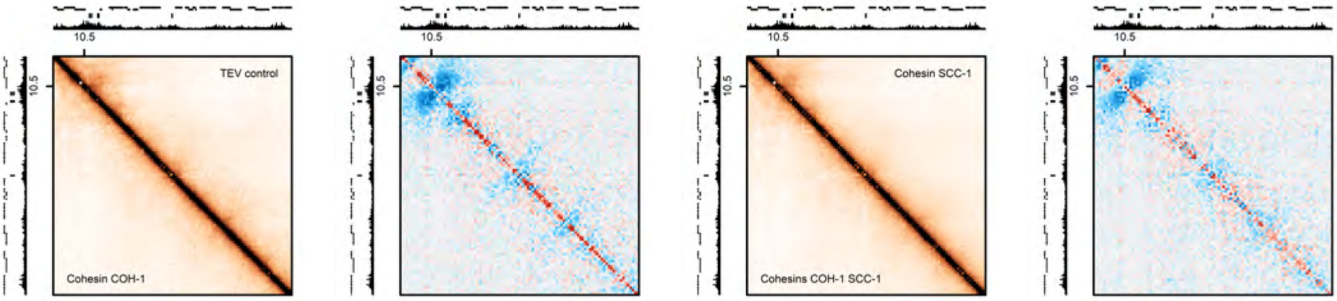

chrII:10829001-11029000

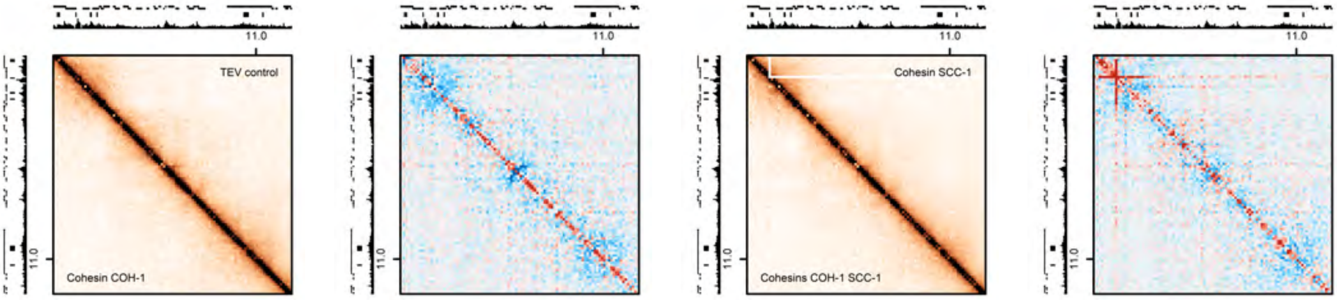

chrI:6185001-6385000

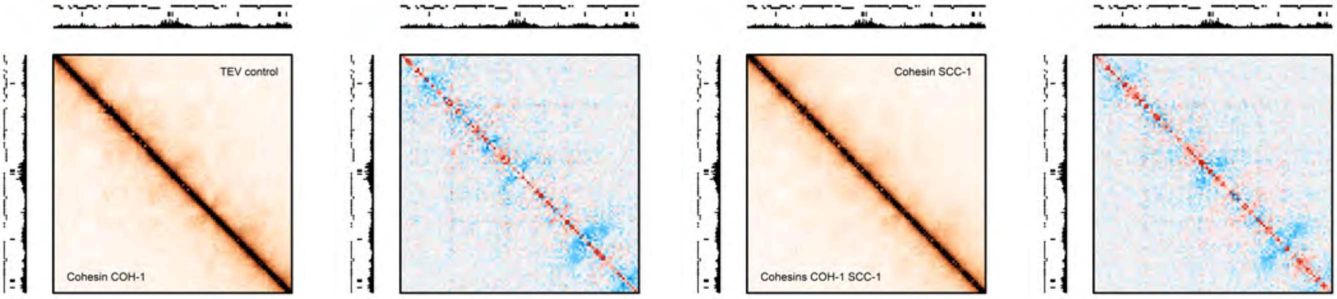

chrV:9351001-9551000

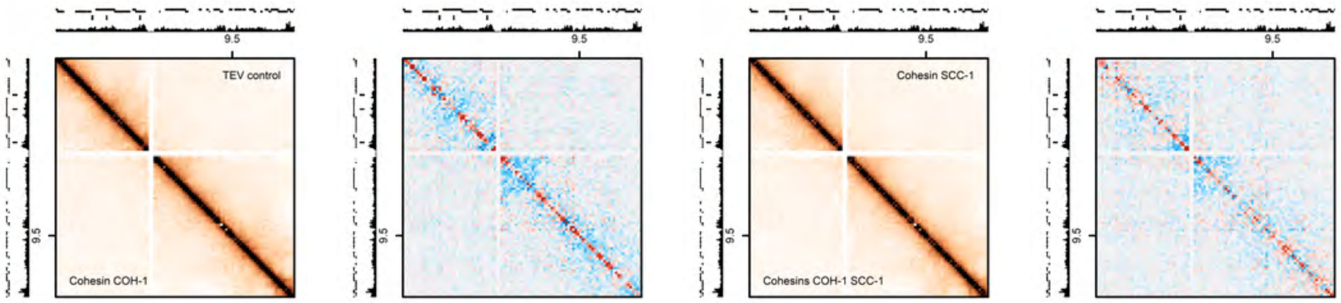

chrIII:6319001-6519000

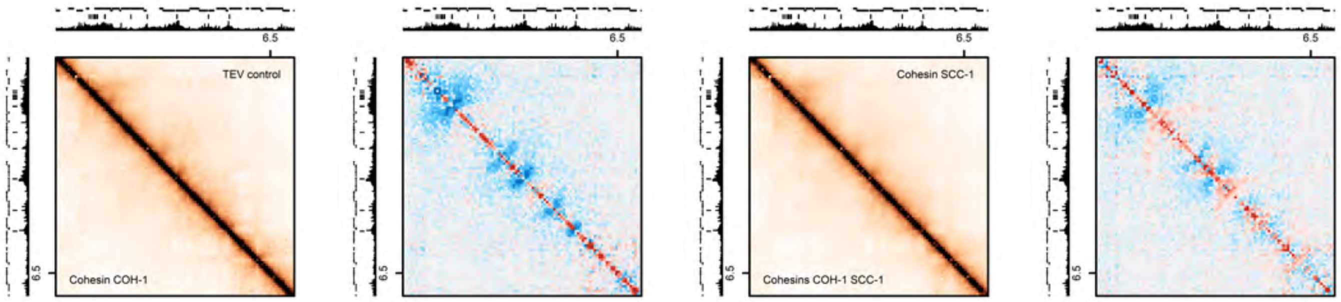

chrI:6277001-6477000

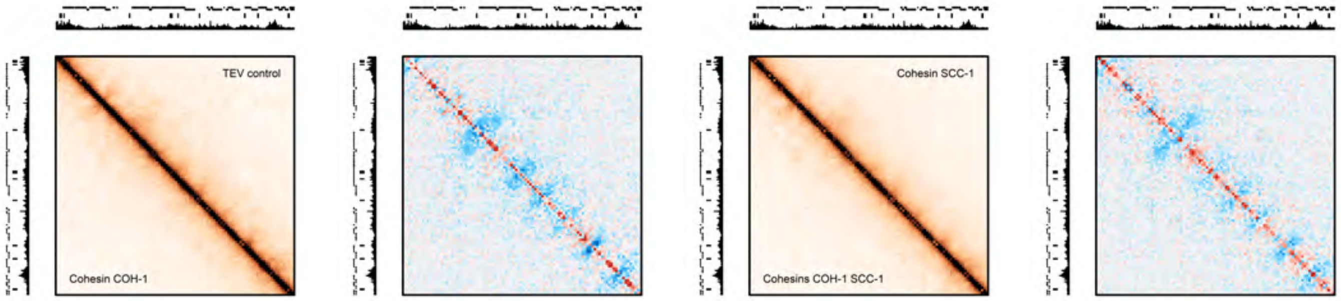

chrIII:8131001-8331000

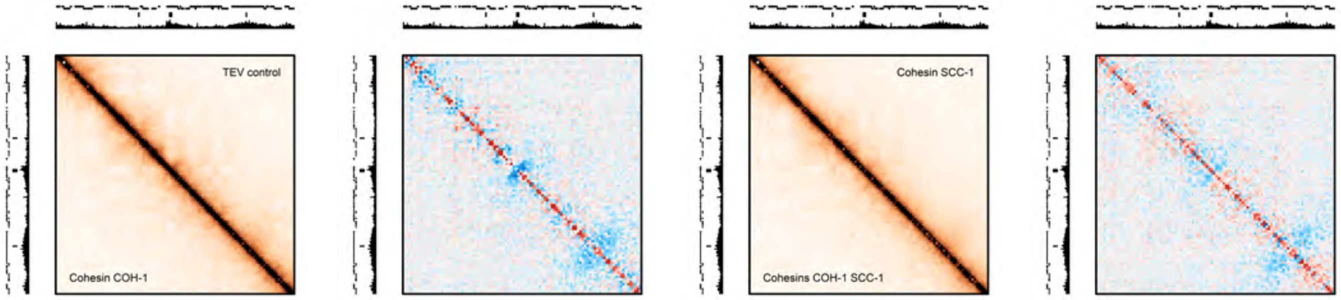

chrV:12715001-12915000

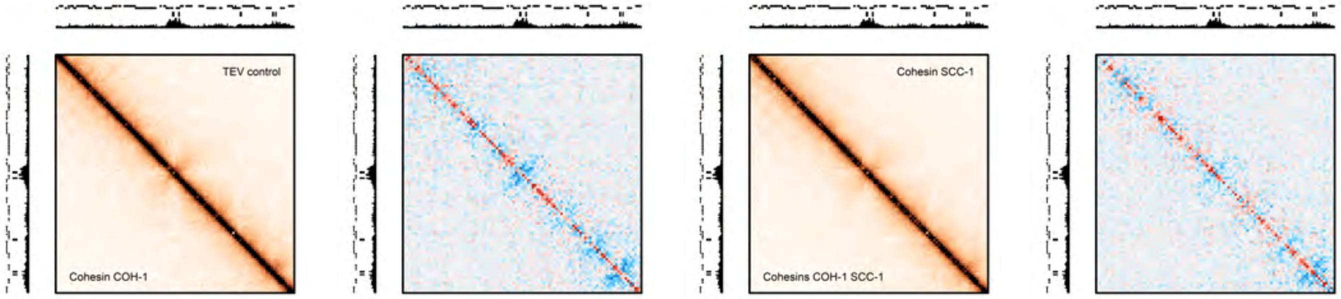

chrII:6079001-6279000

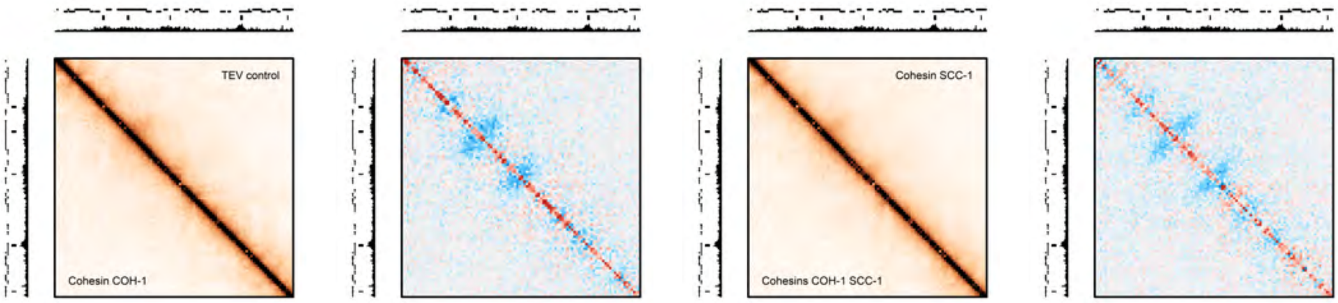

chrIV:1293001-1493000

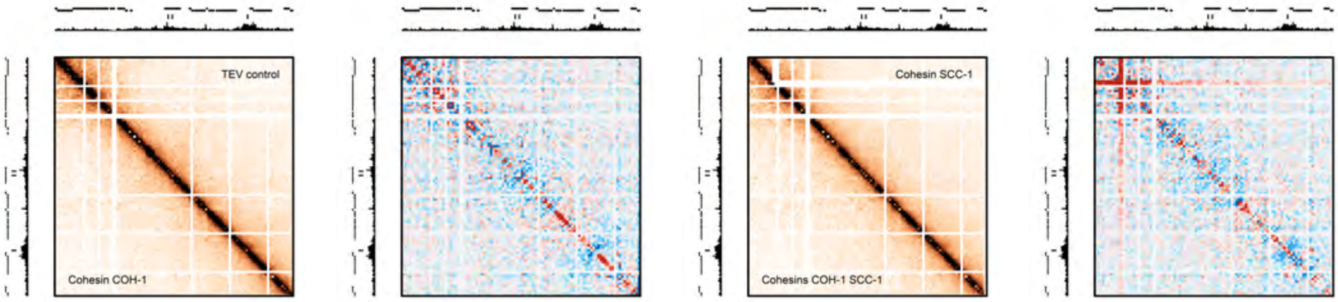

chrIII:-34999-165000

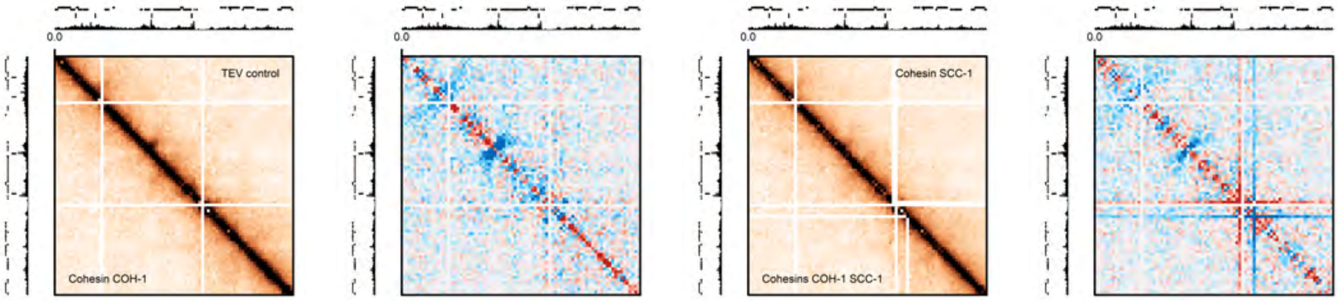

chrIV:11591001-11791000

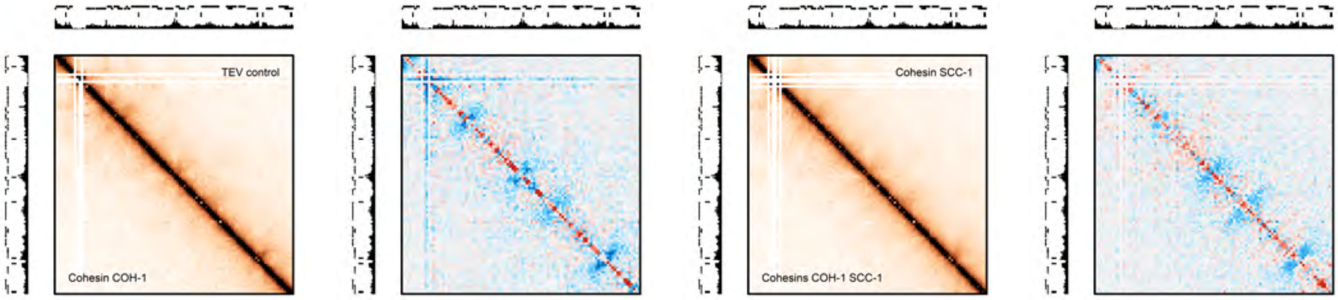

chrI:9069001-9269000

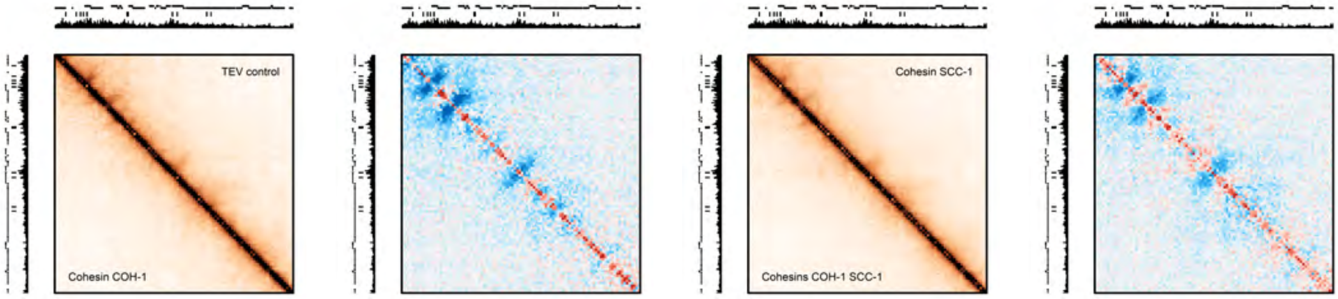

chrX:1505001-1705000

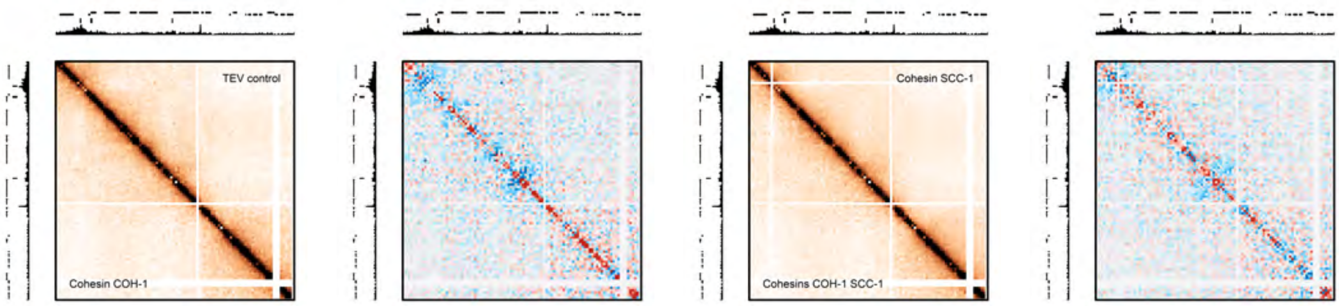

chrV:15087001-15287000

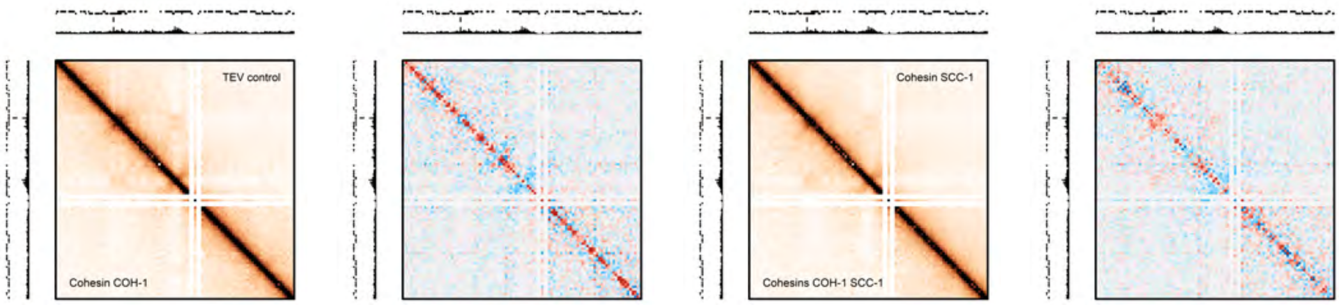

chrIV:1051001-1251000

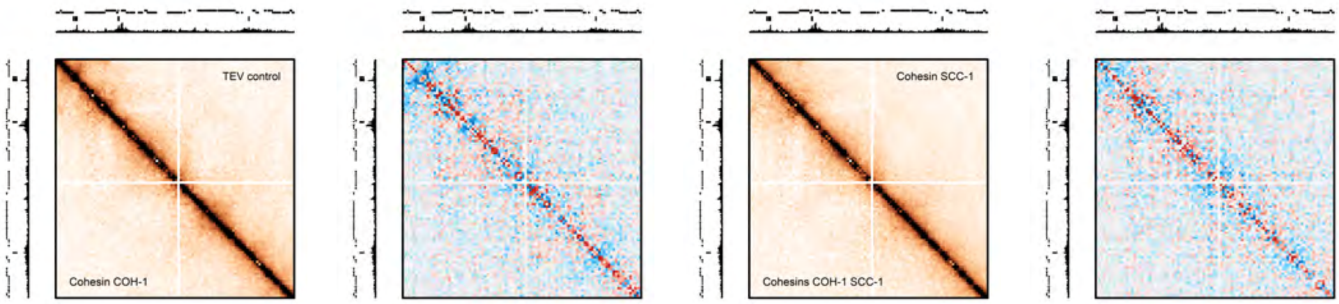

chrI:10133001-10333000

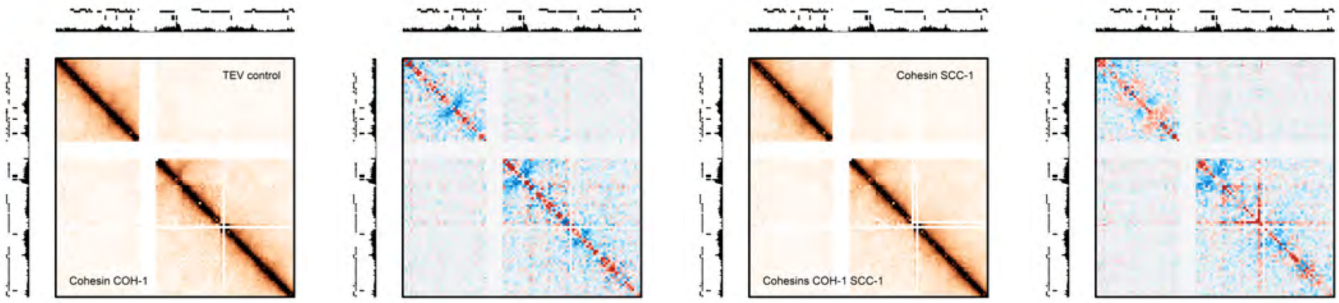

chrI:1169001-1369000

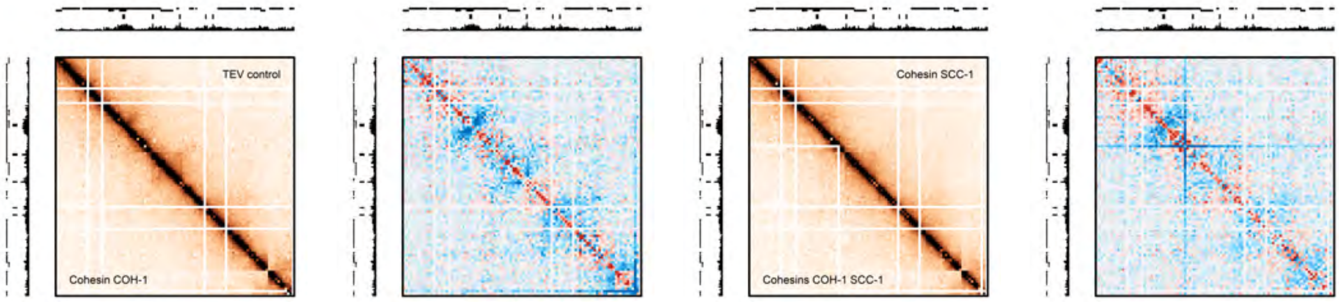

chrII:8545001-8745000

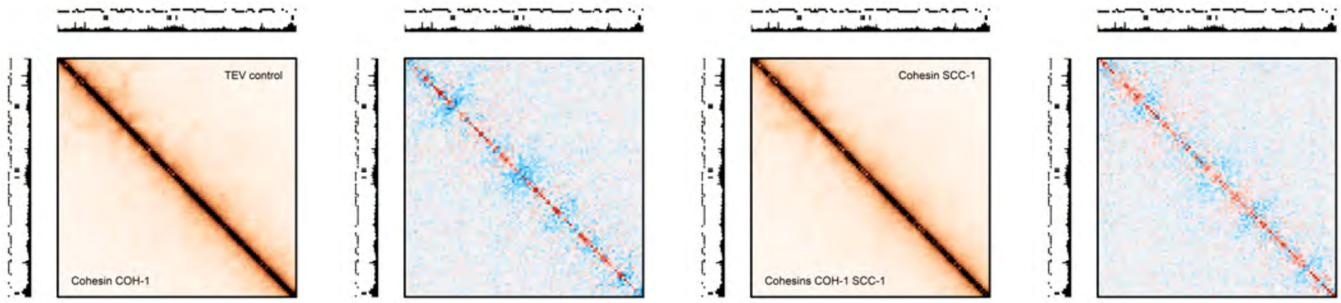

chrIV:6993001-7193000

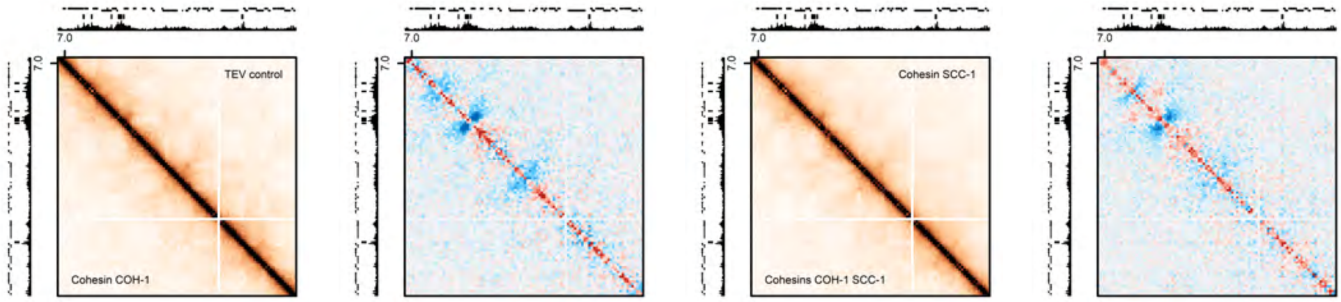

chrIII:12915001-13115000

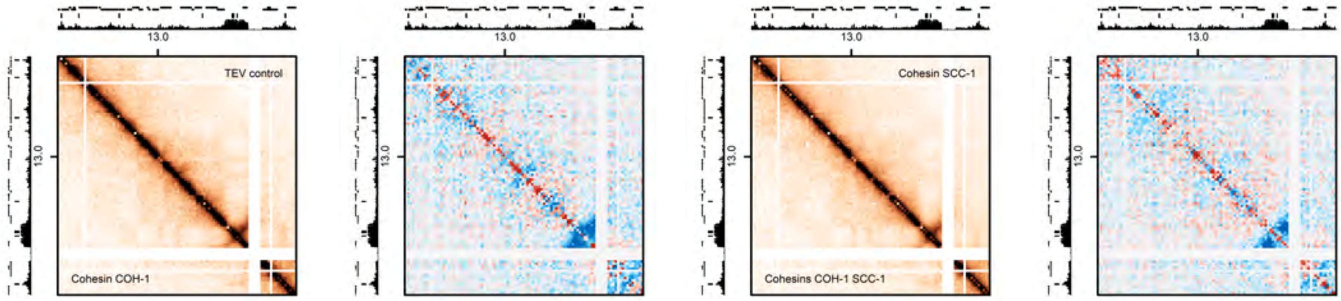

chrX:1975001-2175000

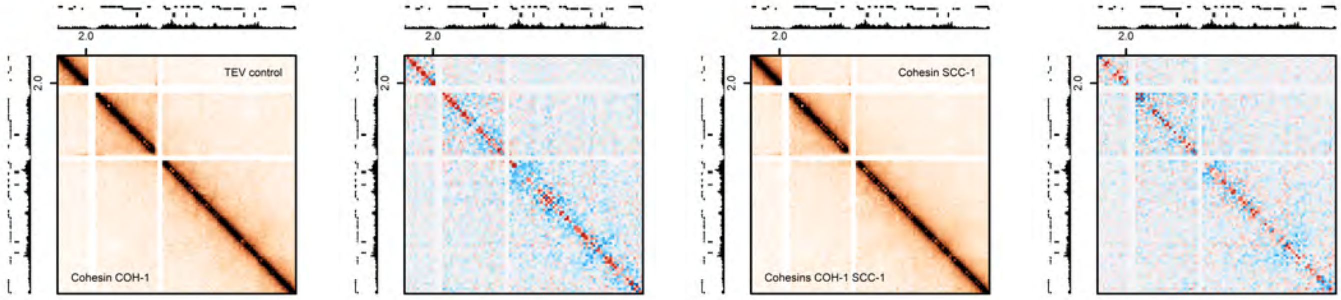

chrX:495001-695000

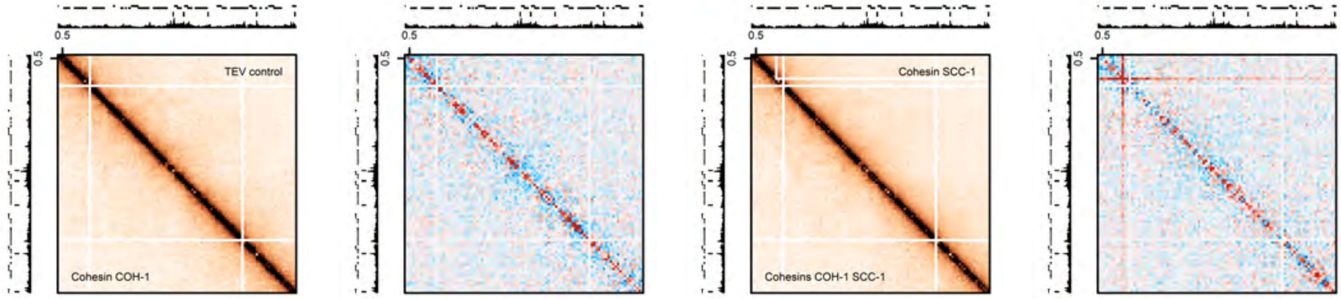

chrX:14195001-14395000

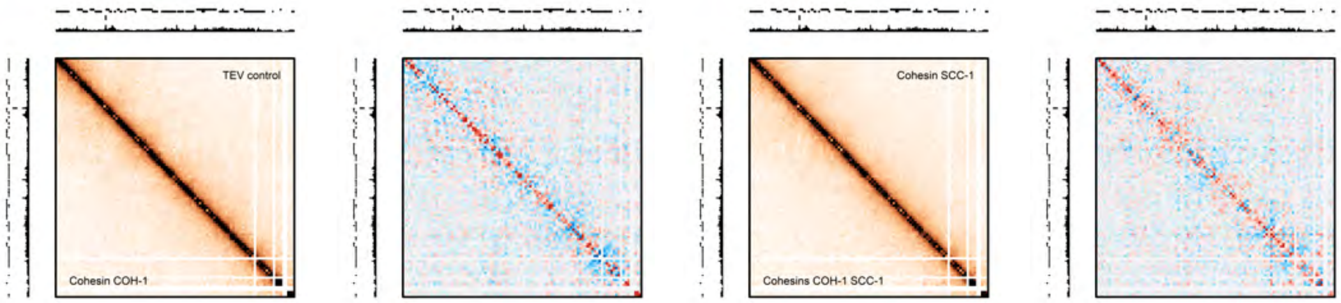

chrIII:4697001-4897000

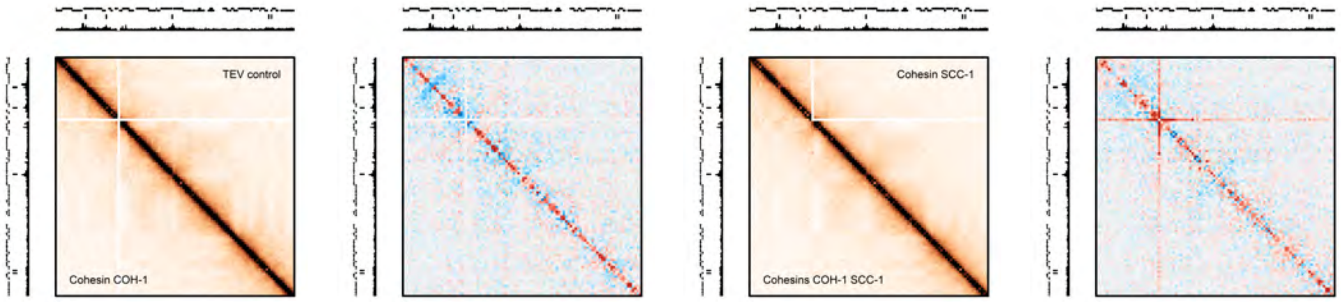

chrX:223001-423000

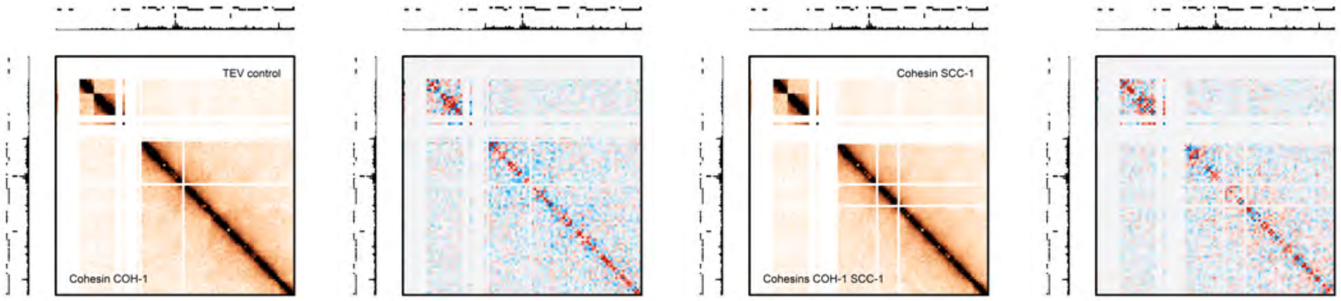

chrIII:12377001-12577000

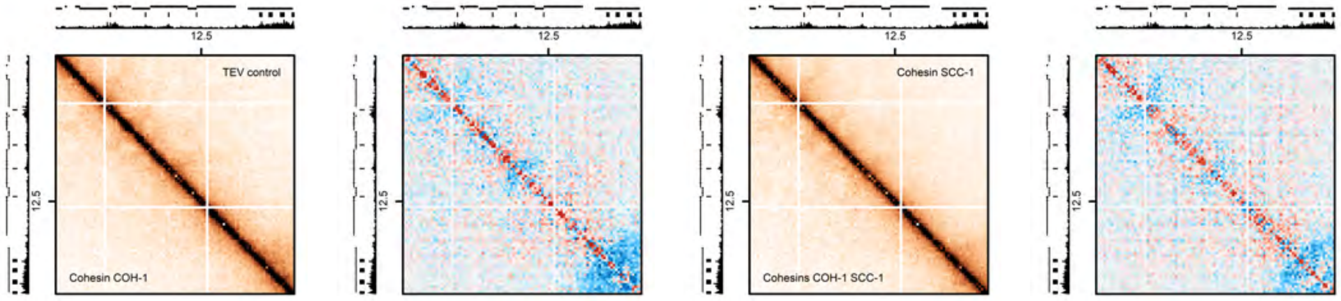

chrV:5201001-5401000

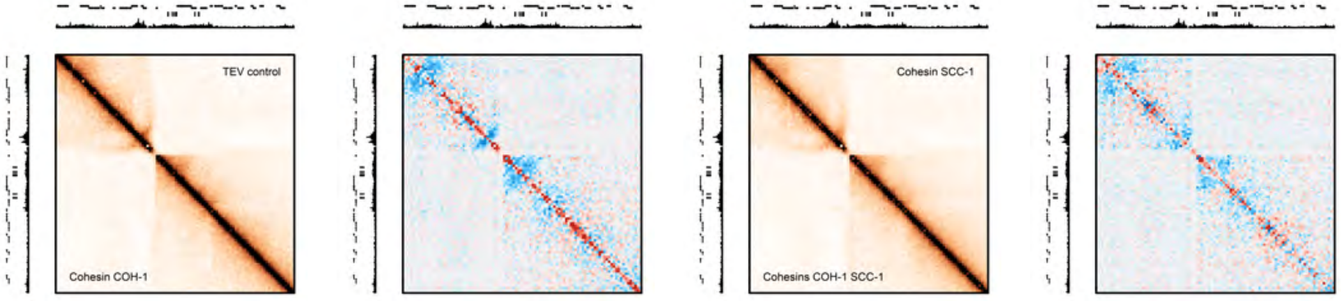

chrIII:1231001-1431000

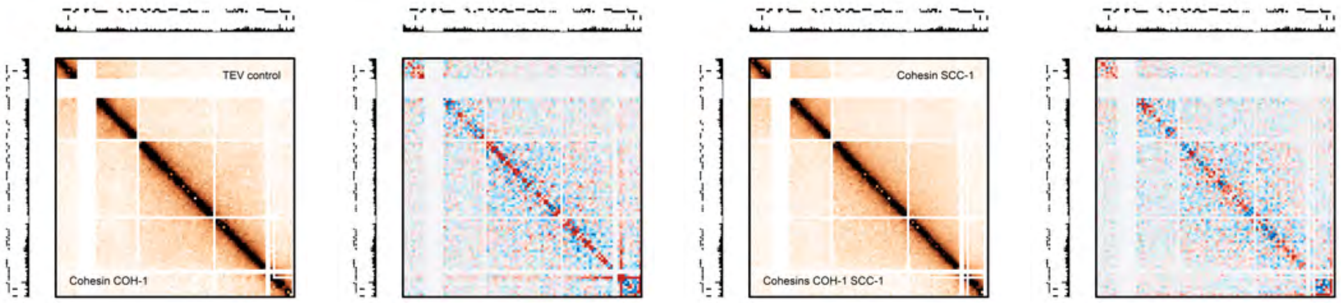

chrV:11789001-11989000

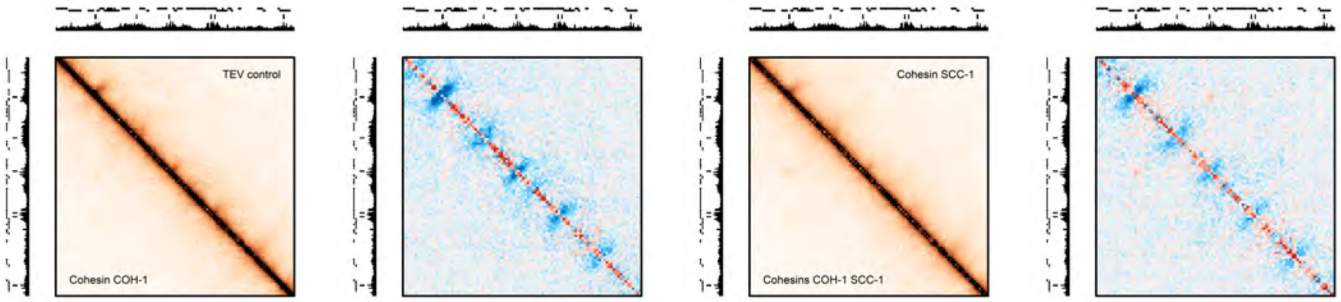

chrII:5473001-5673000

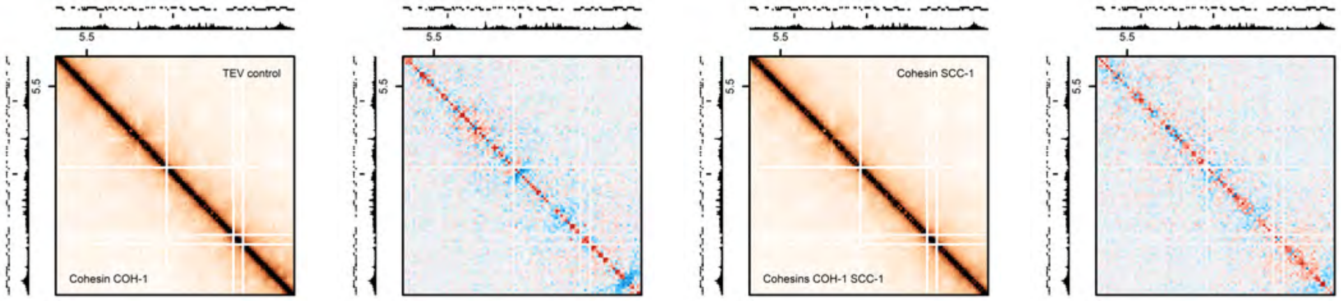

chrII:2953001-3153000

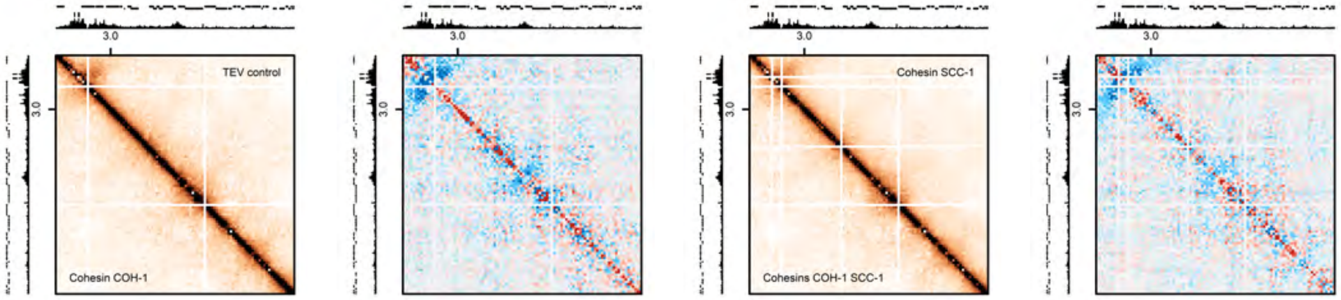

chrX:8621001-8821000

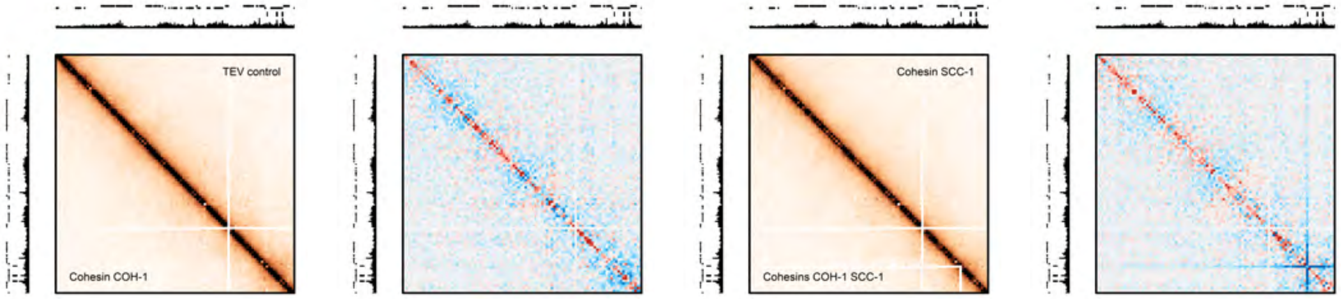

chrX:17379001-17579000

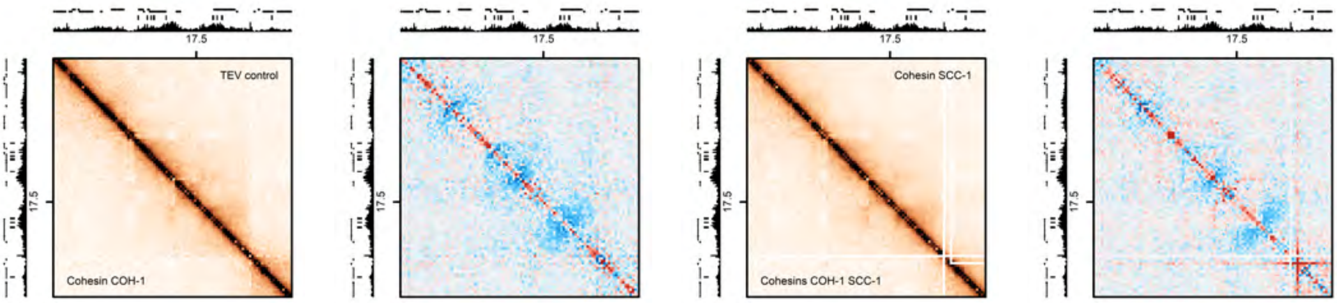

chrV:1897001-2097000

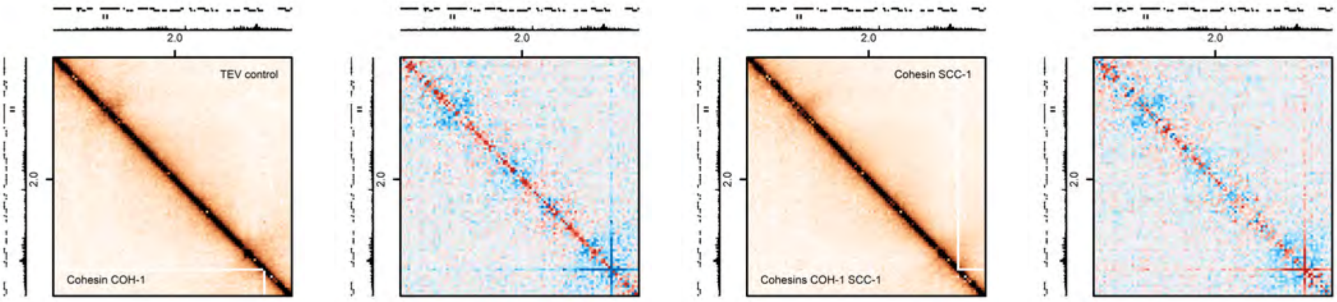

chrII:6391001-6591000

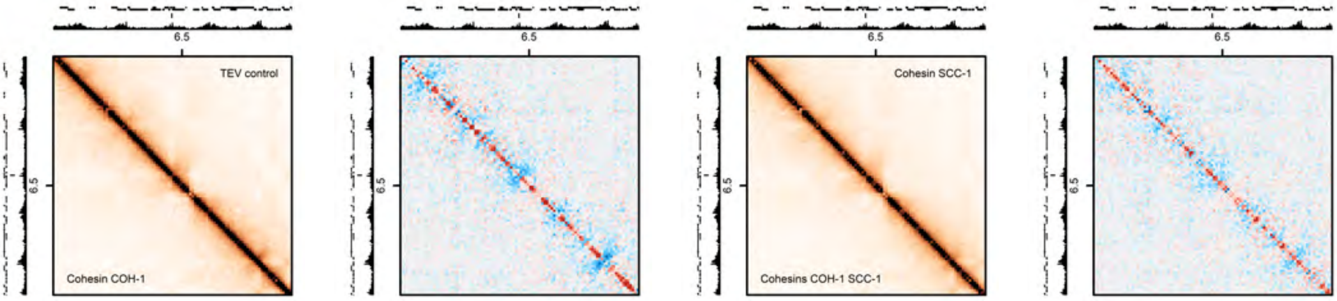

chrIII:4229001-4429000

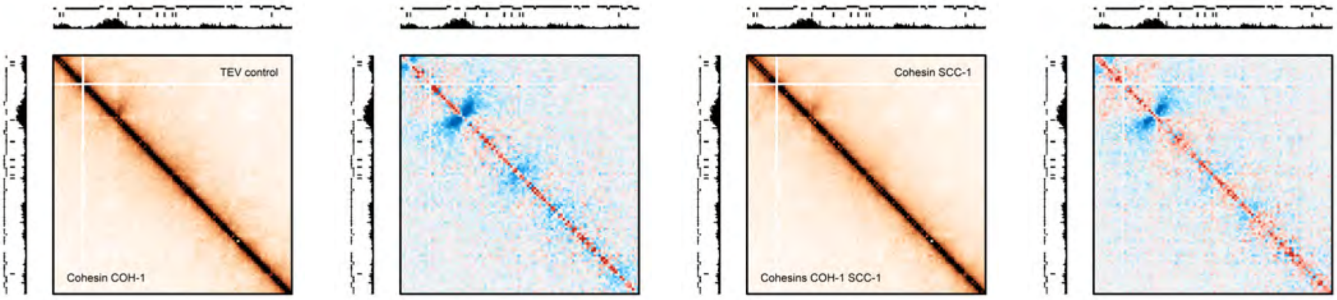

chrV:10233001-10433000

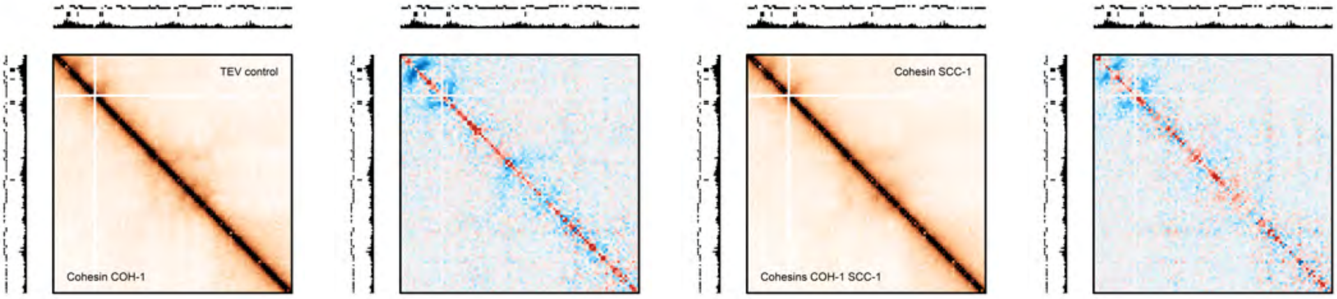

chrIV:10305001-10505000

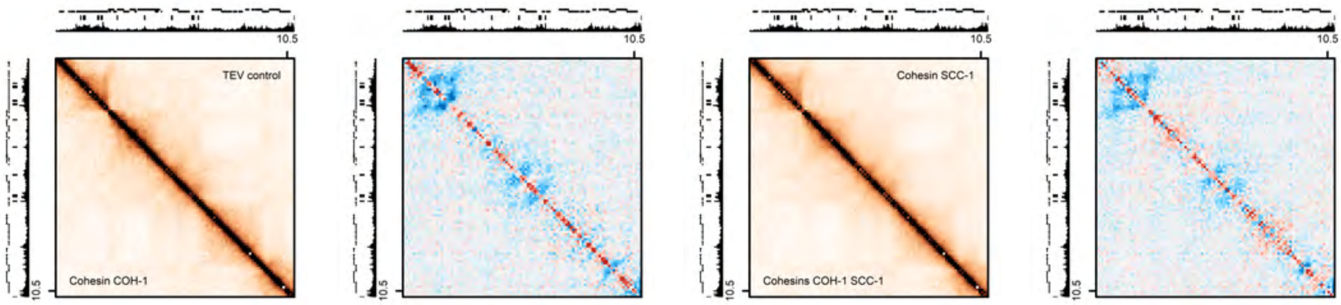

chrI:6359001-6559000

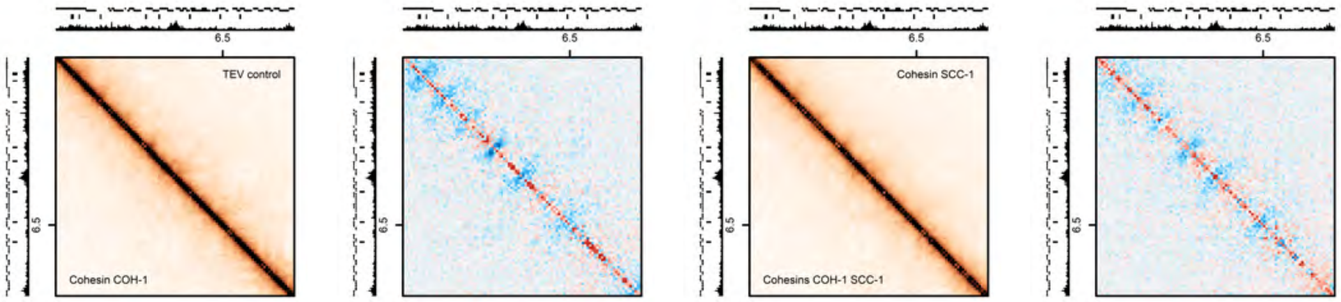

chrI:13085001-13285000

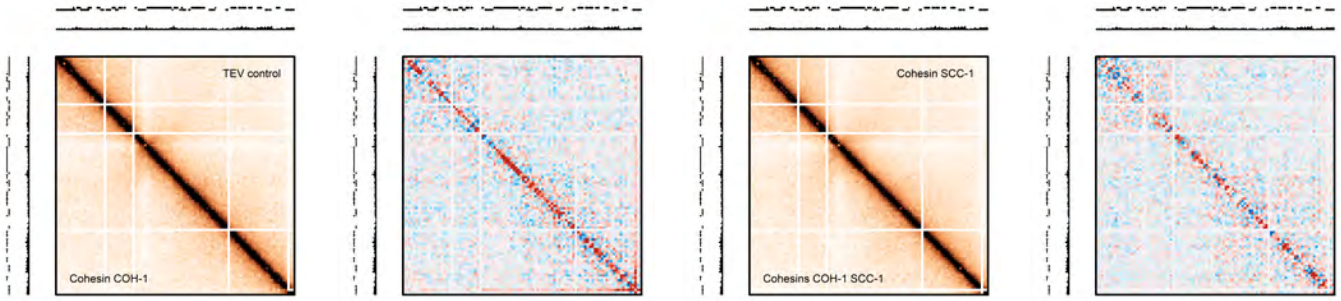

chrII:8241001-8441000

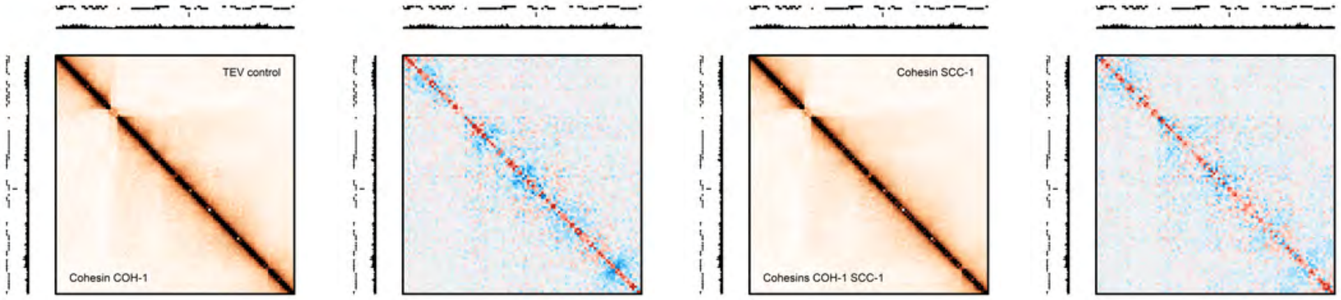

chrX:2605001-2805000

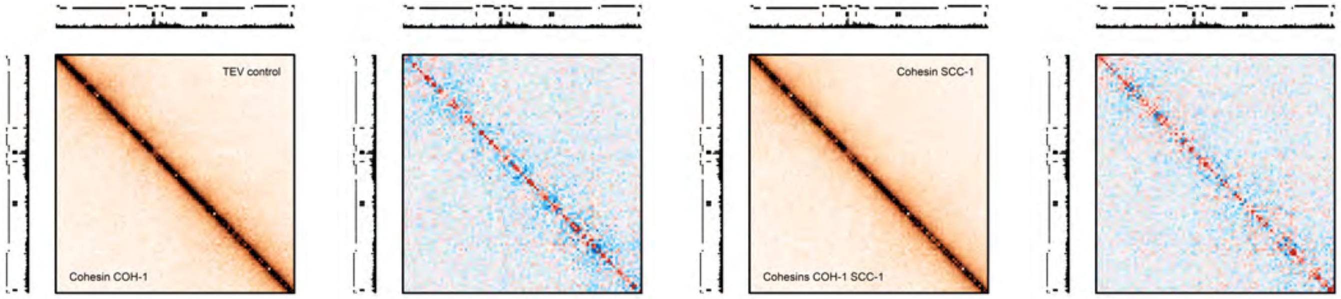

chrX:401001-601000

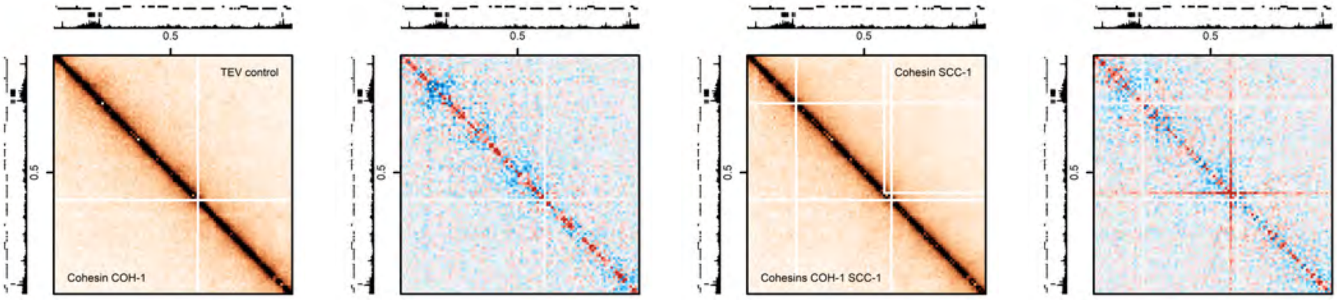

chrIV:5959001-6159000

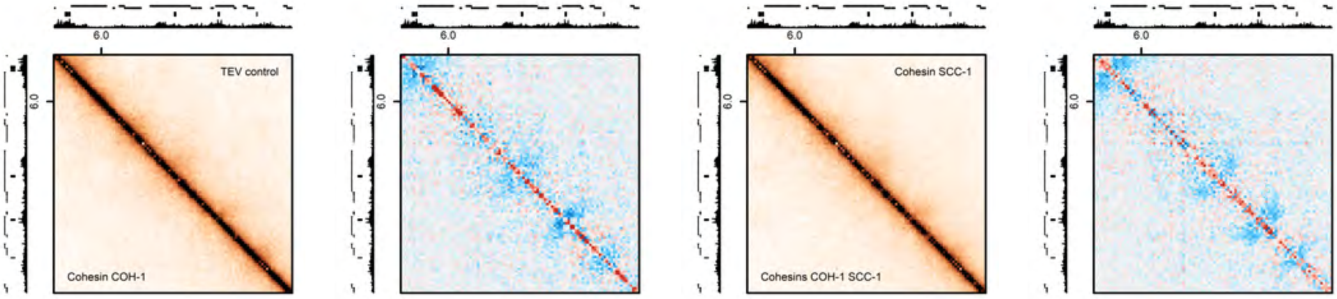

chrIV:13605001-13805000

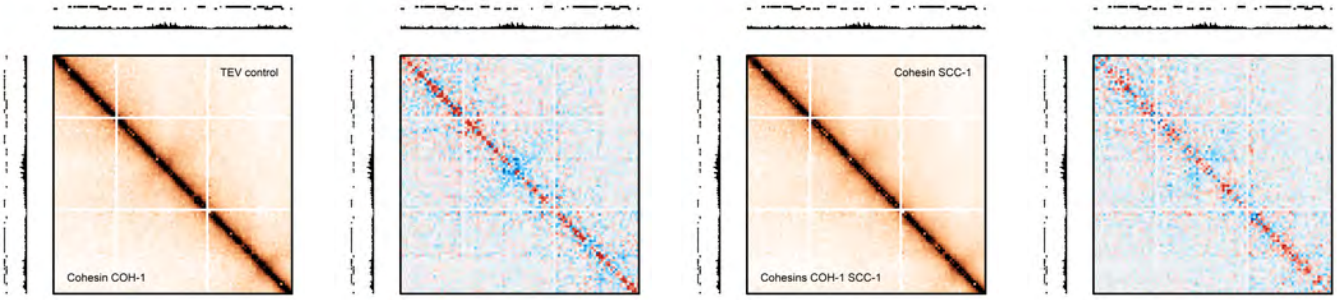

chrX:10295001-10495000

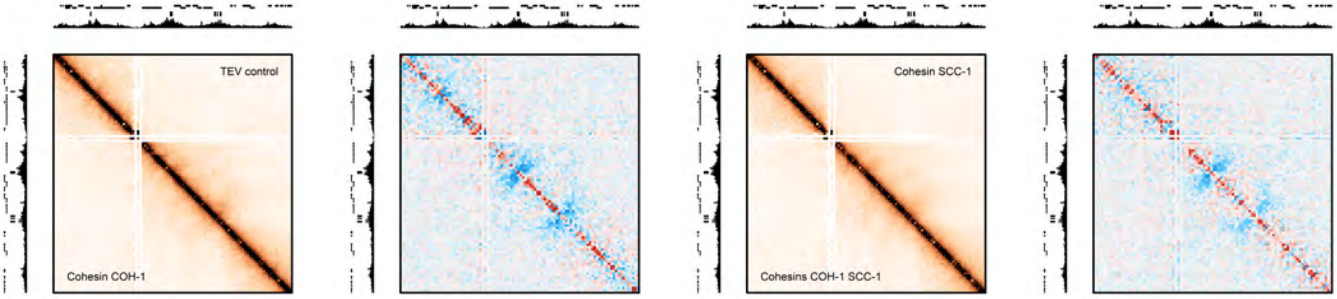

chrIV:1437001-1637000

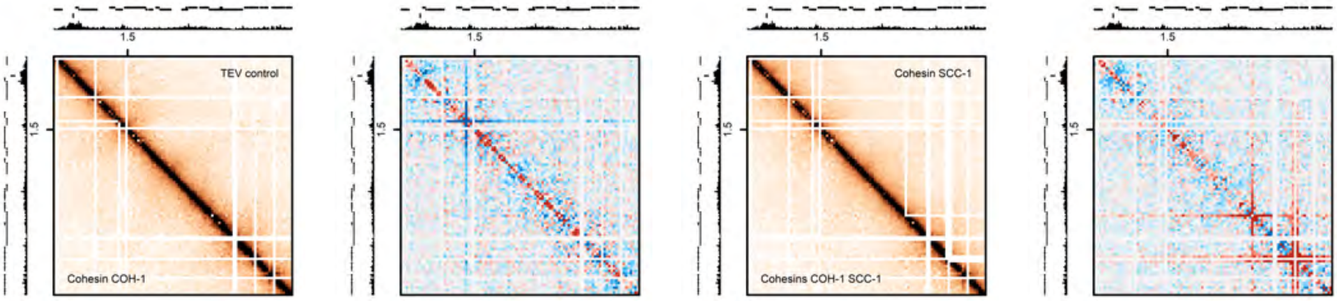

chrII:7435001-7635000

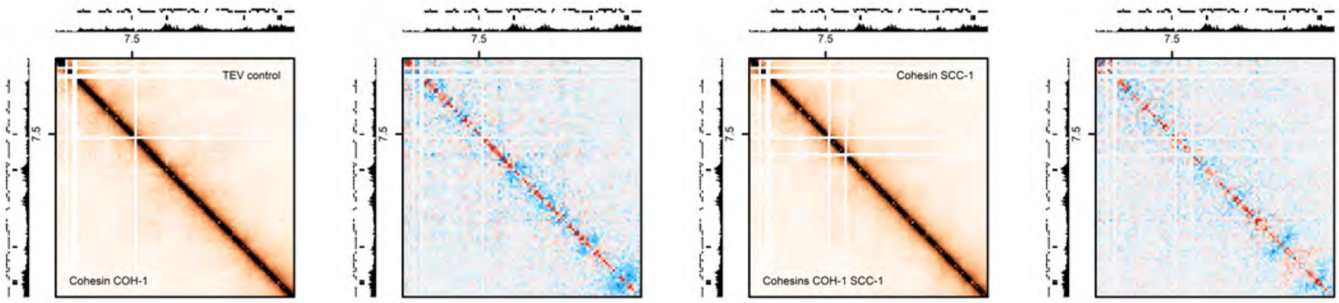

chrI:1981001-2181000

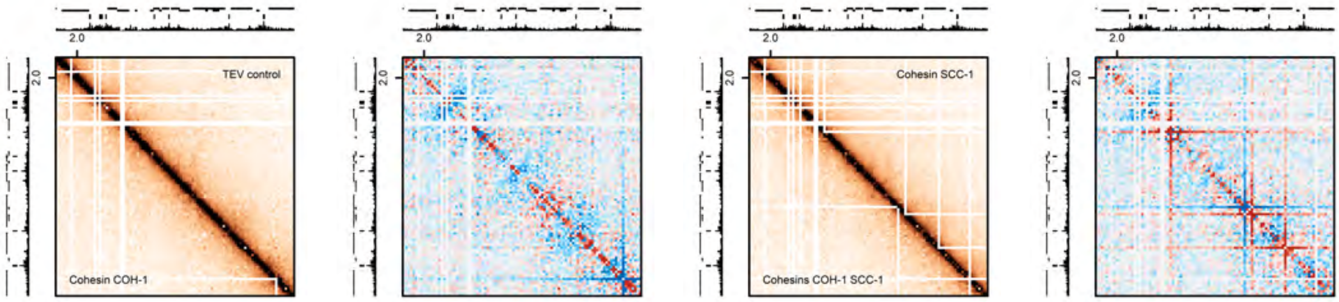

chrIII:6649001-6849000

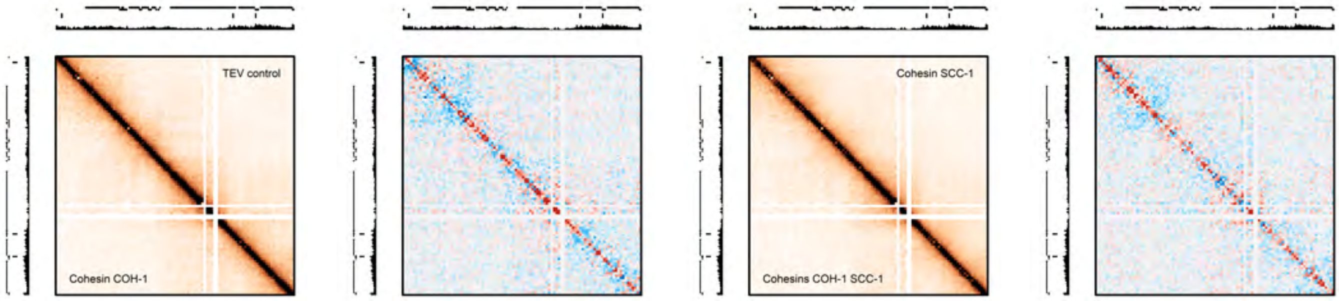

chrIII:1011001-1211000

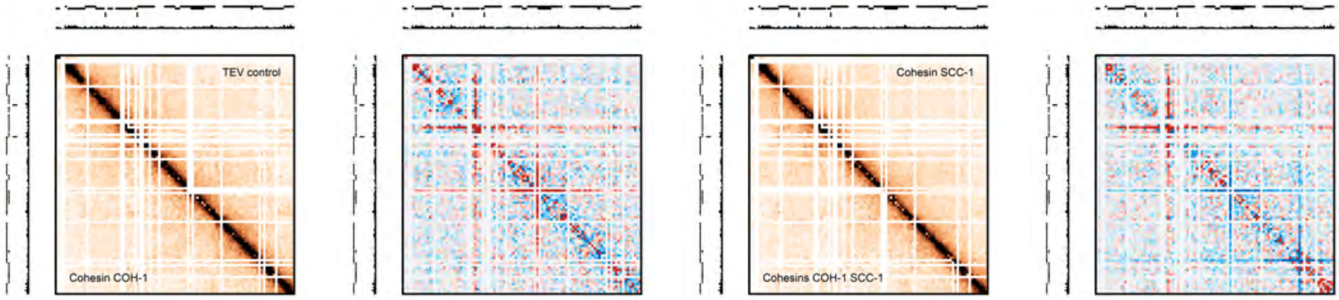

chrIII:2317001-2517000

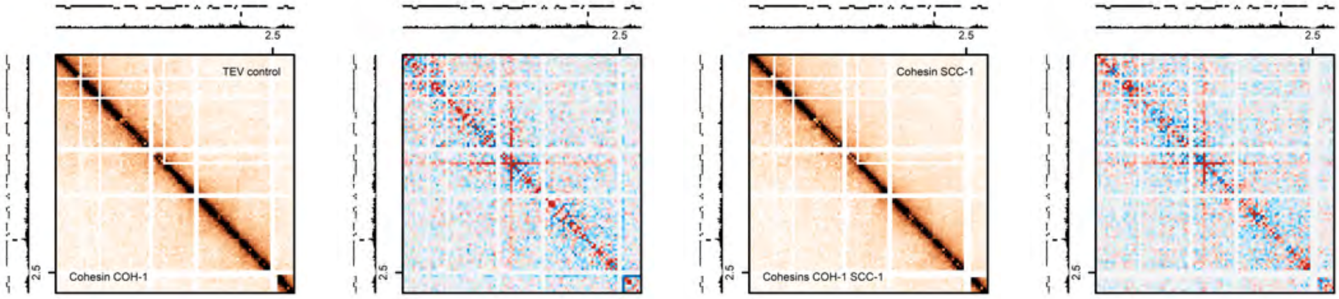

chrX:11457001-11657000

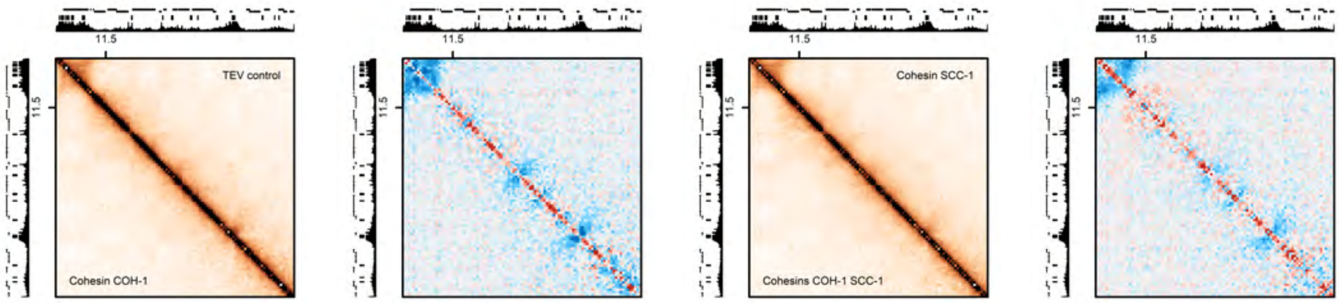

chrII:12601001-12801000

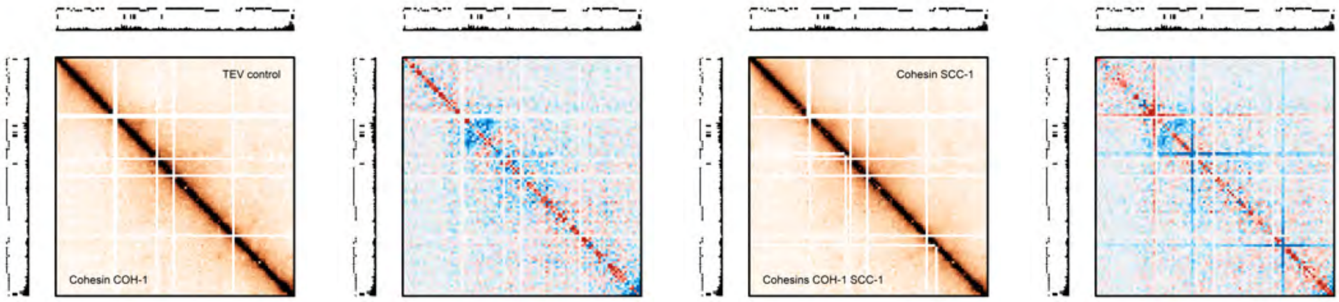

chrIII:11839001-12039000

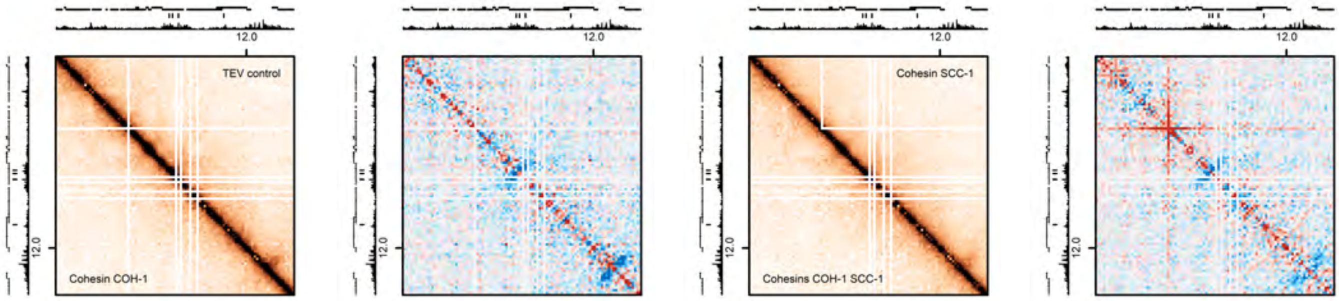

chrV:11213001-11413000

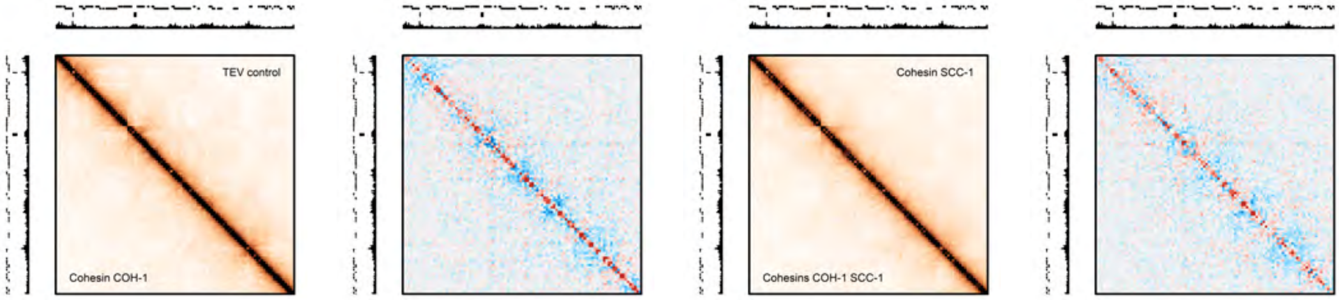

chrI:4877001-5077000

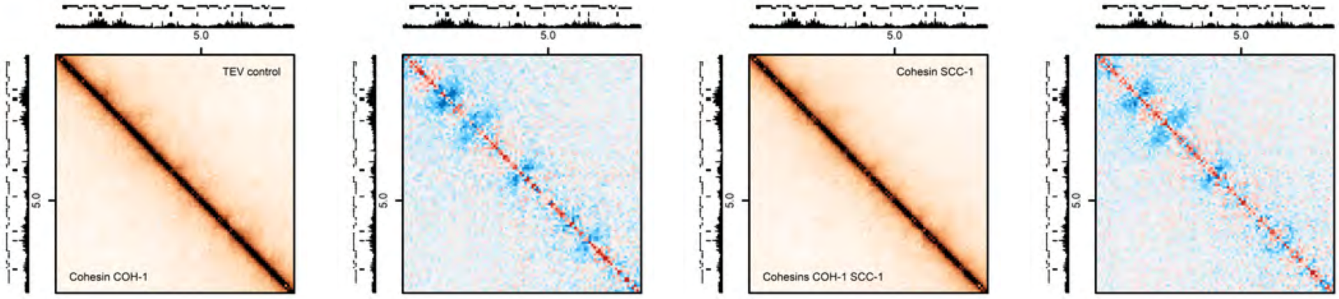

chrV:8639001-8839000

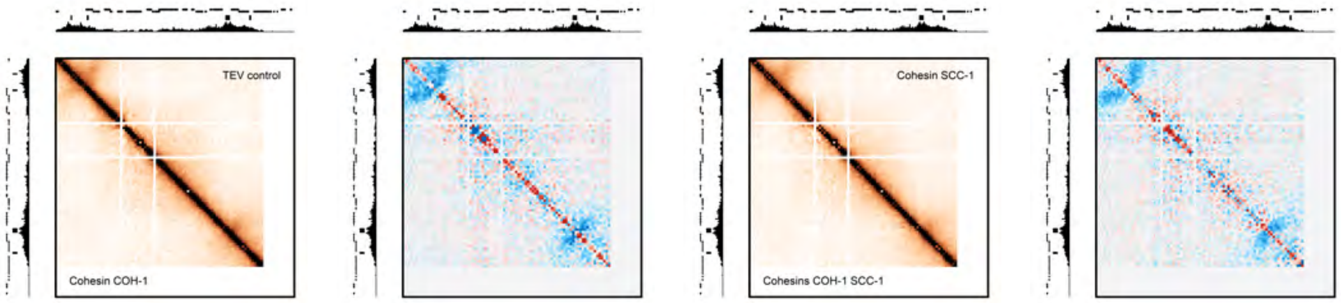

chrII:13803001-14003000

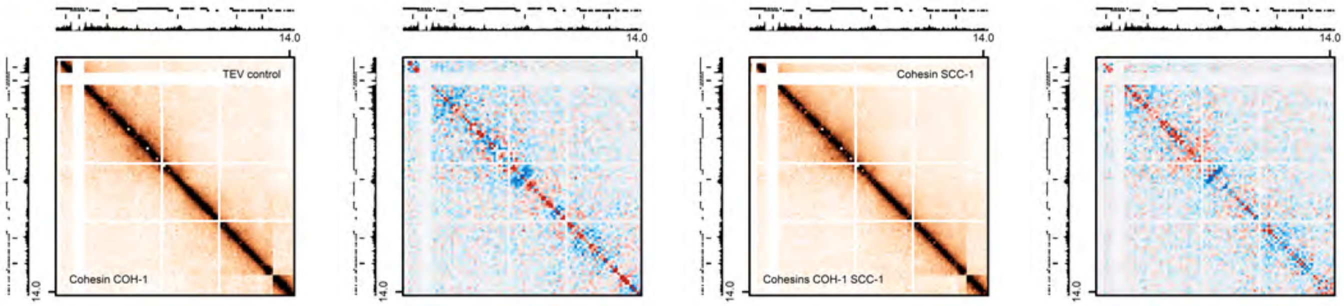

chrIII:11145001-11345000

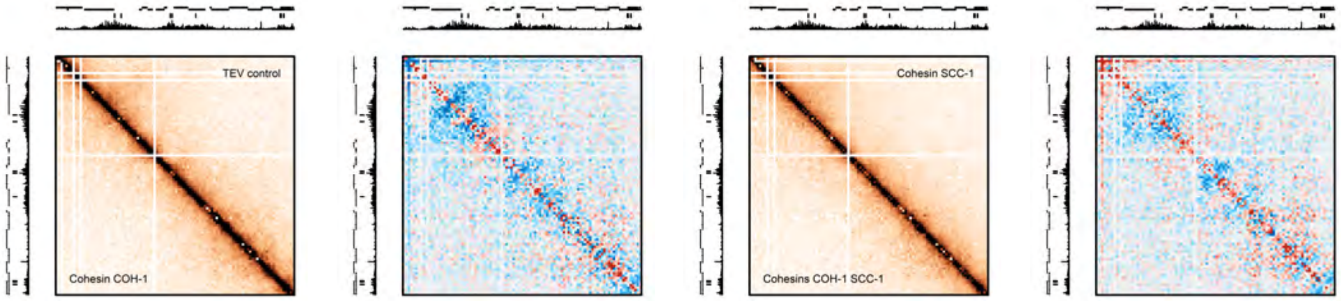

chrV:7879001-8079000

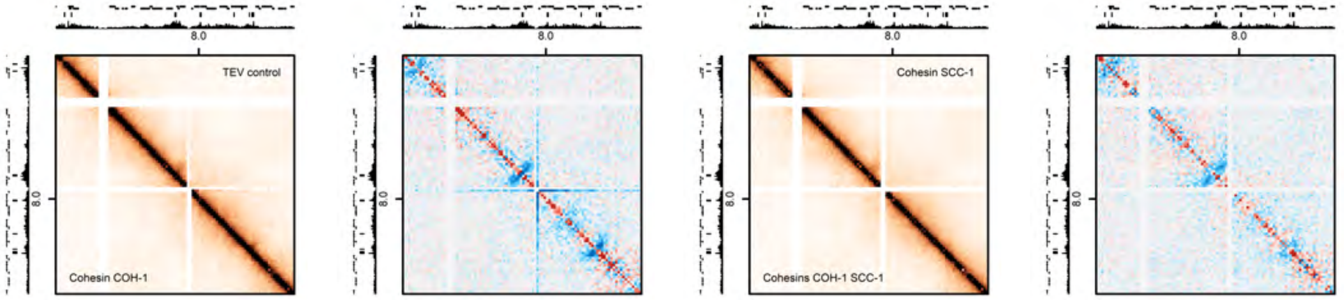

chrII:10583001-10783000

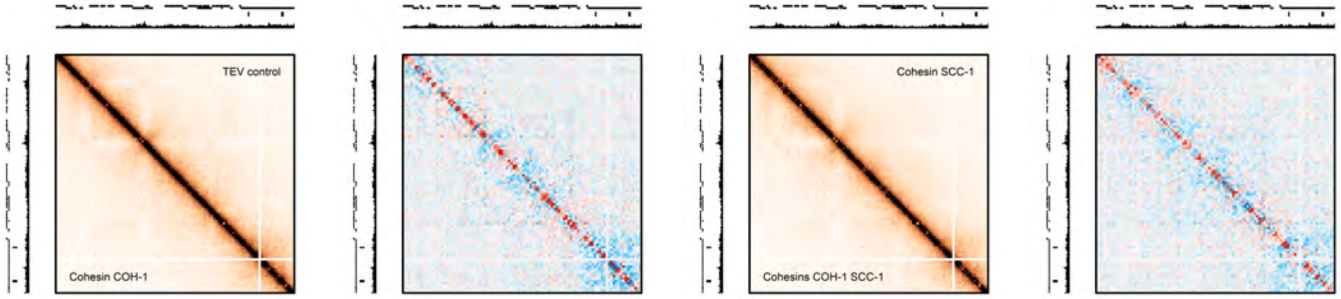

chrI:13221001-13421000

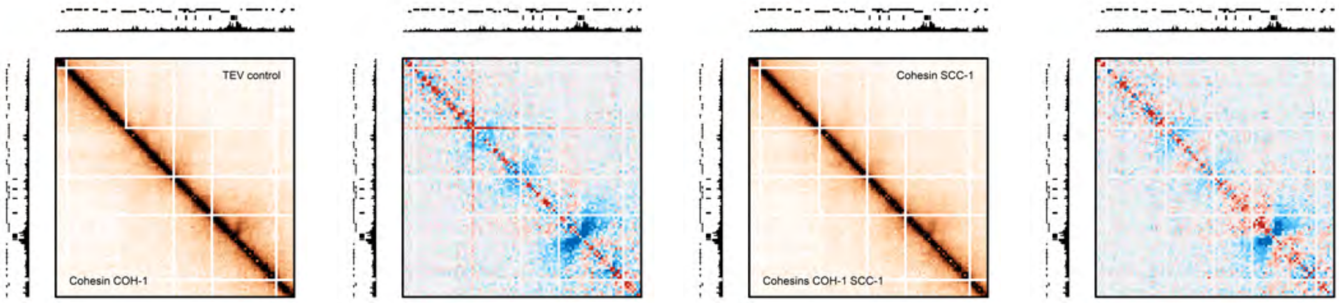

chrV:7445001-7645000

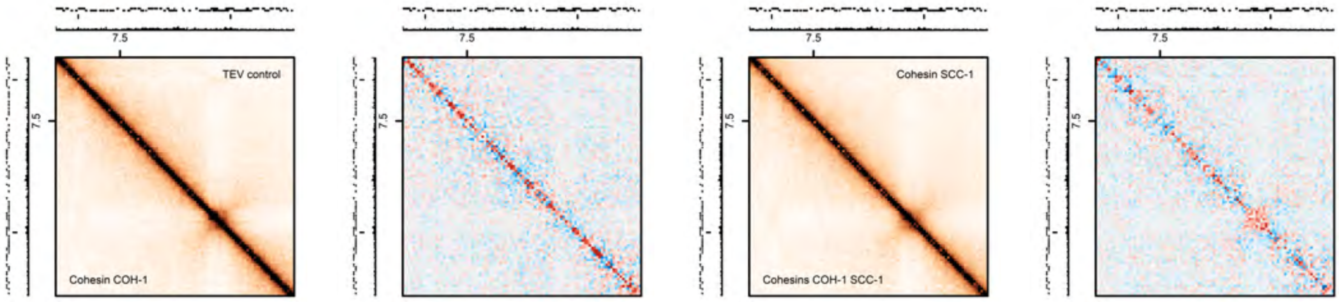

chrIV:14739001-14939000

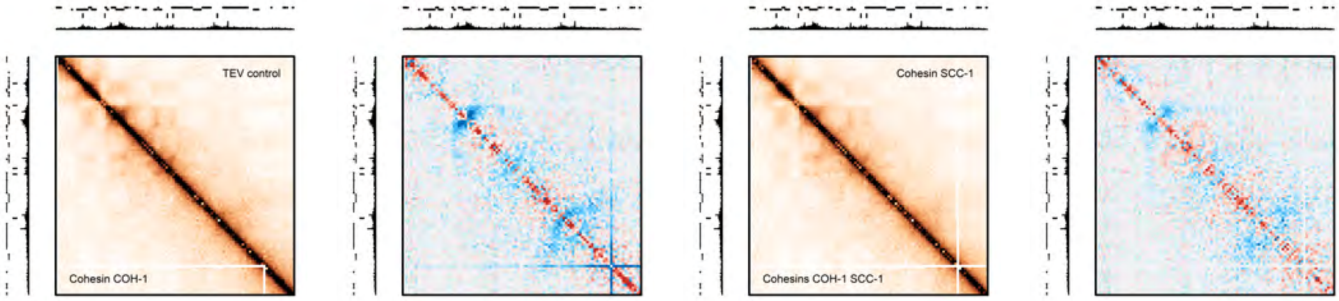

chrIV:5343001-5543000

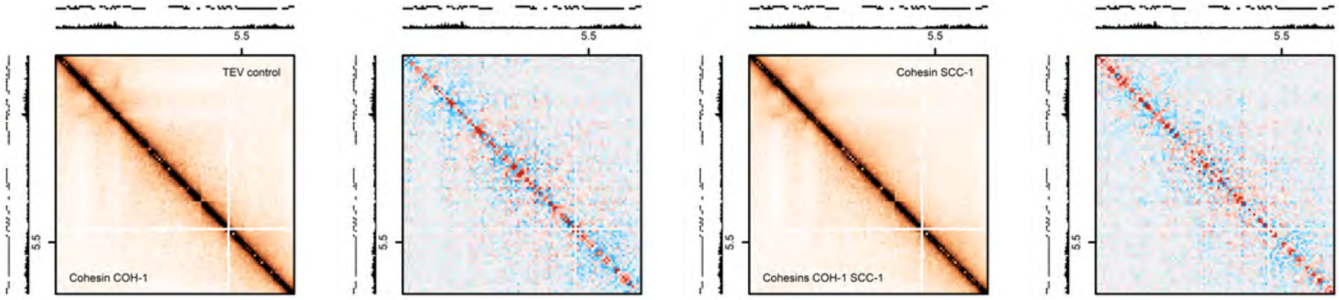

chrX:9983001-10183000

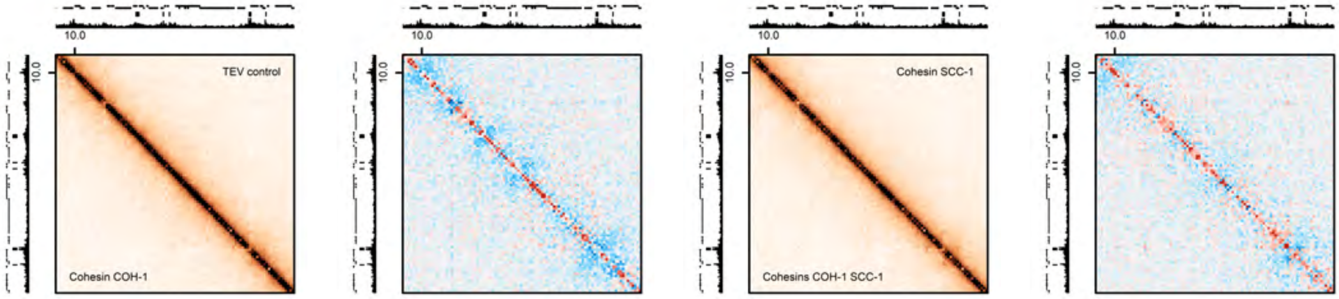

chrIII:201001-401000

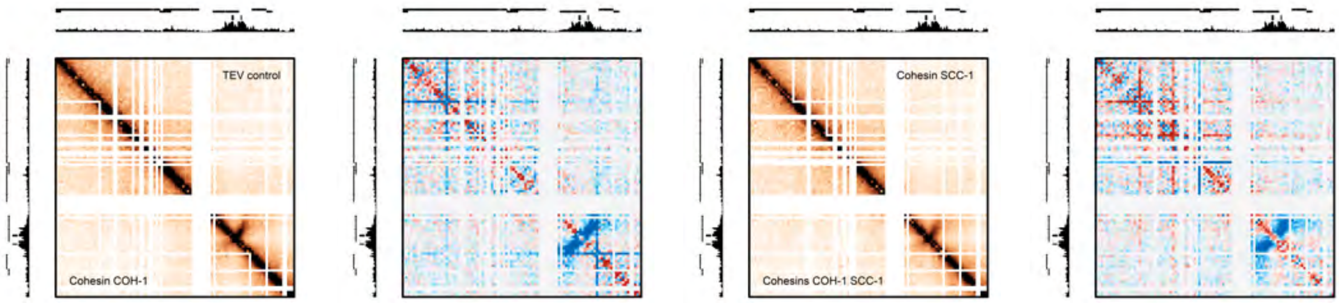

chrII:4095001-4295000

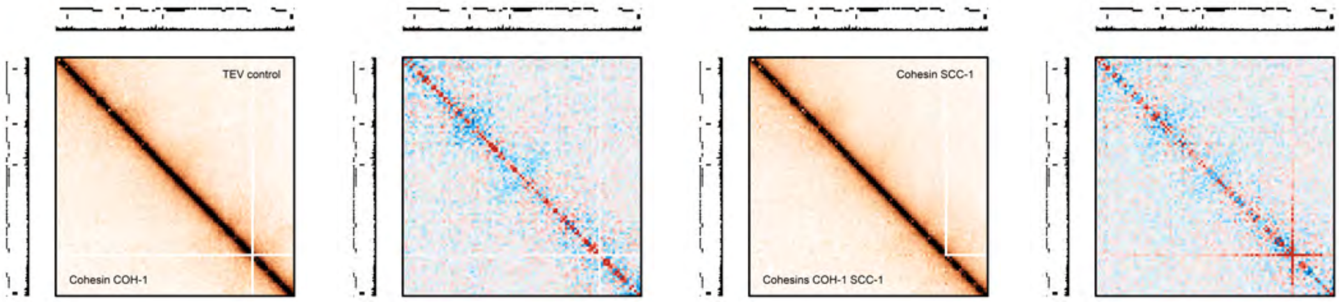

chrX:5249001-5449000

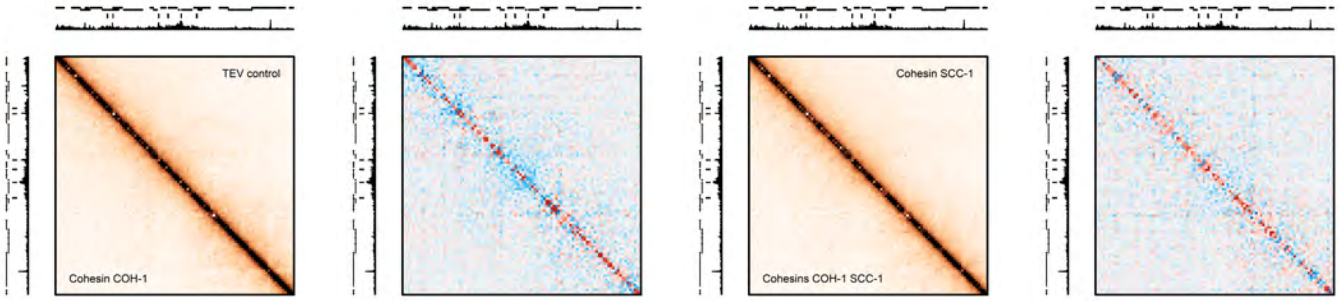

chrII:9323001-9523000

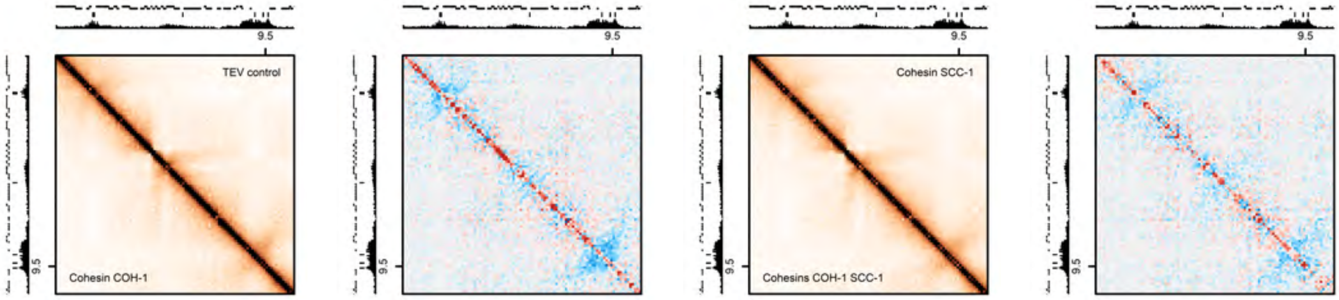

chrV:17903001-18103000

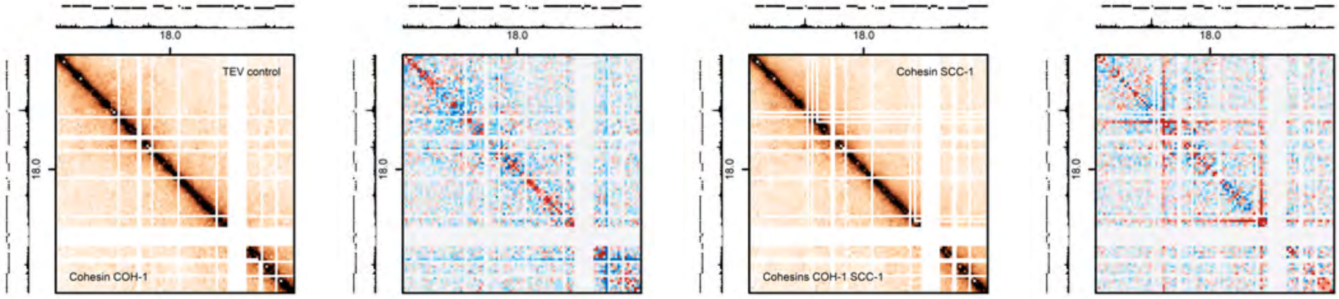

chrIII:2813001-3013000

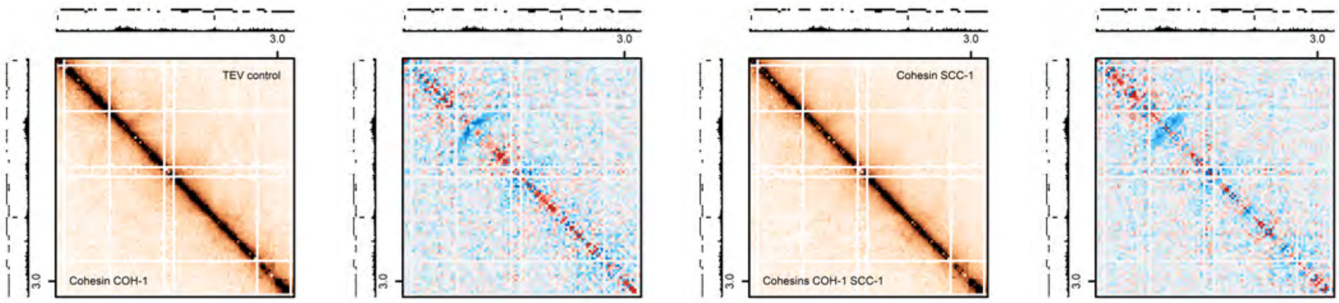

chrV:17655001-17855000

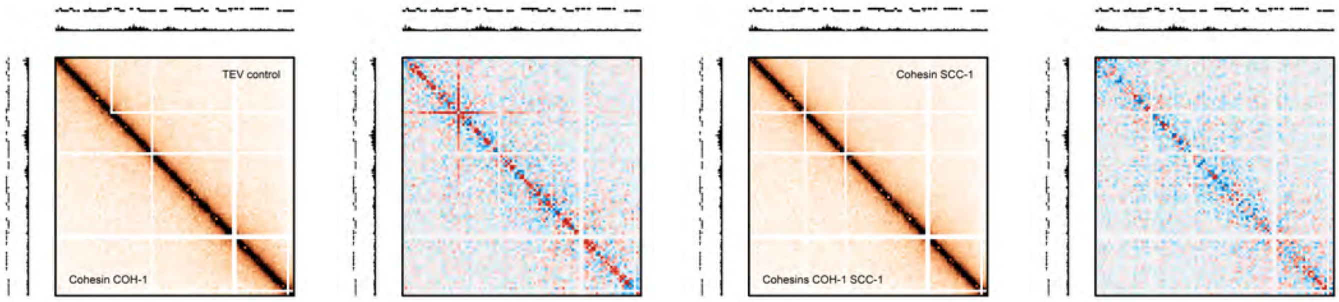

chrX:13105001-13305000

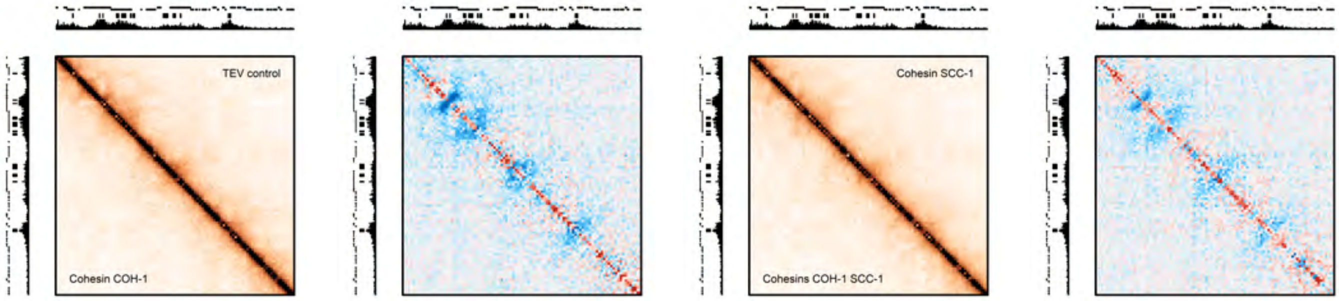

chrIII:9995001-10195000

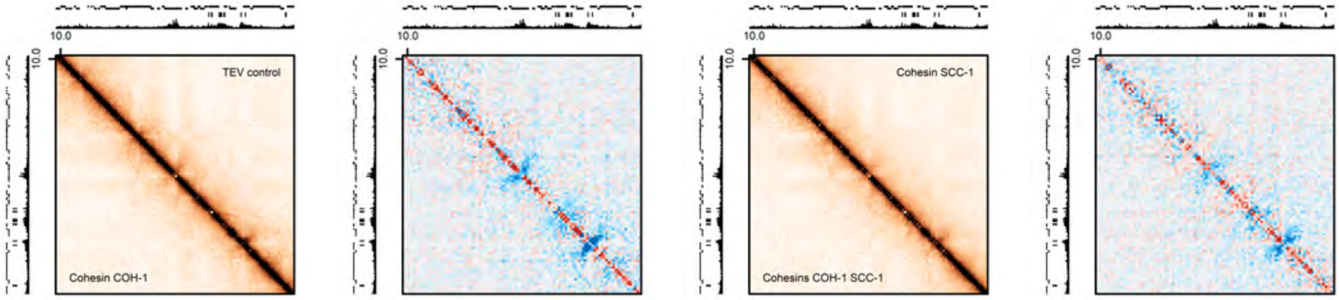

chrV:11995001-12195000

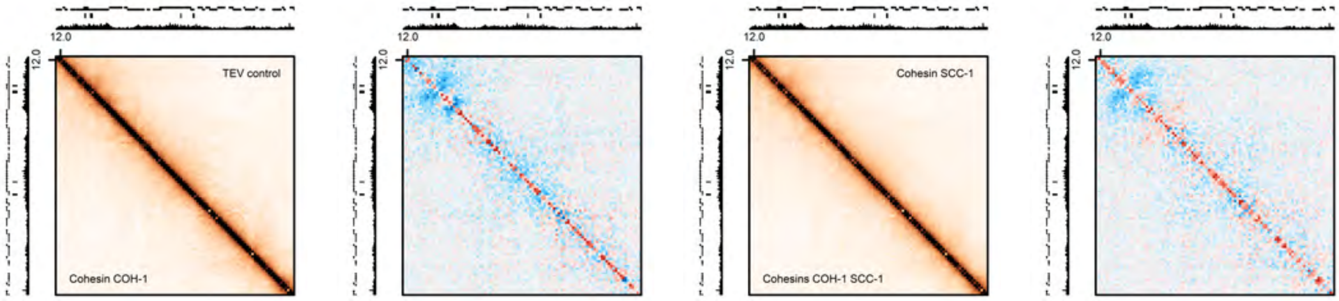

chrIII:2455001-2655000

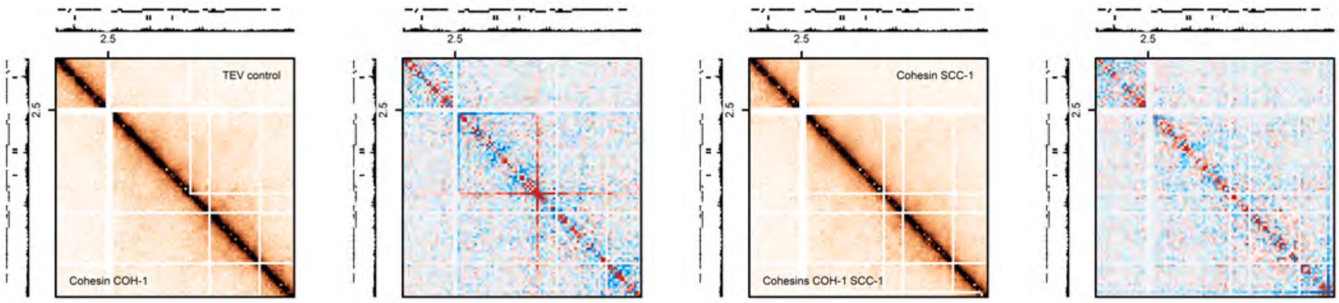

chrI:14709001-14909000

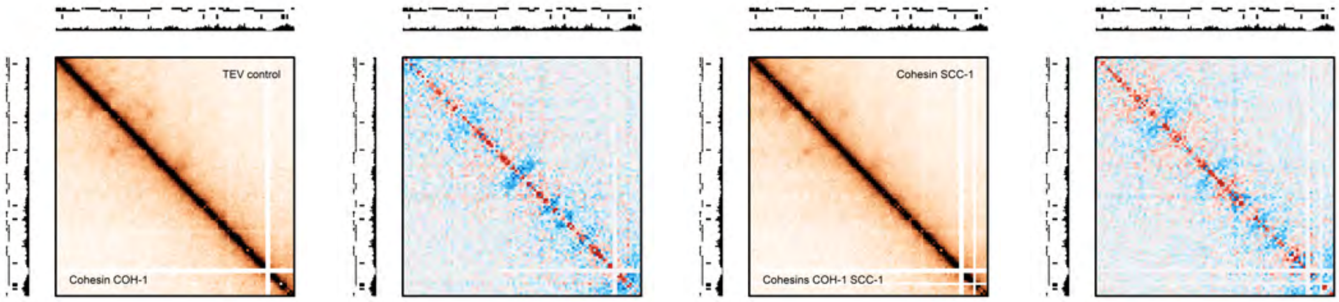

chrII:8685001-8885000

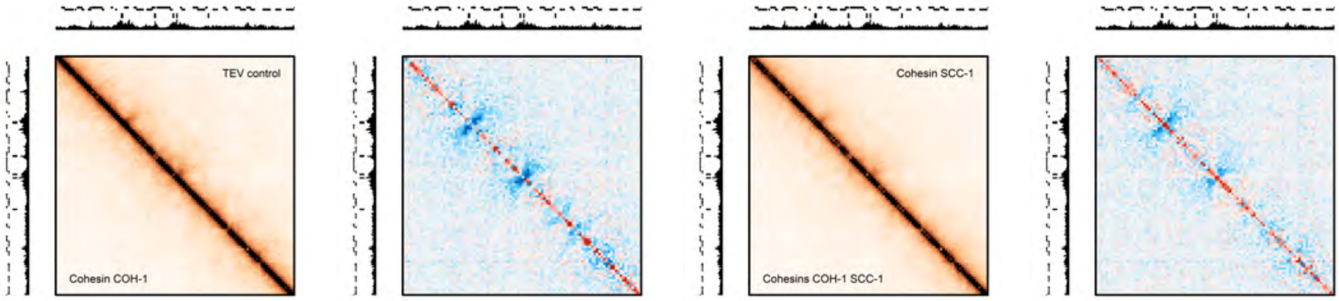

chrV:19567001-19767000

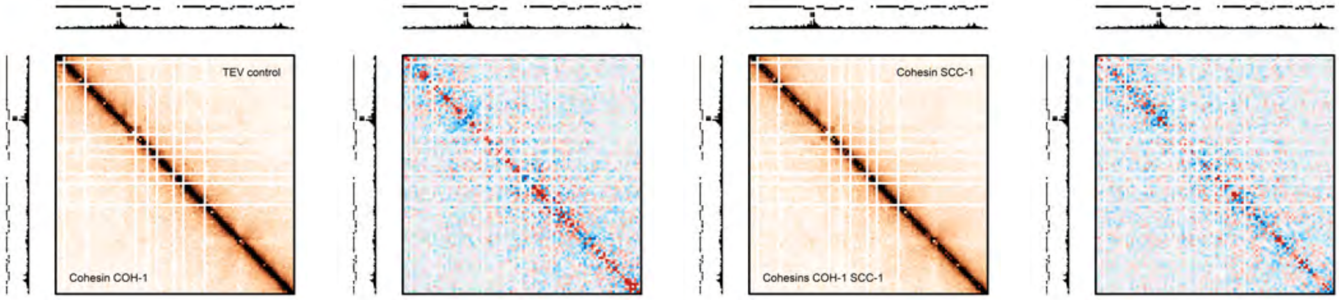

chrX:9835001-10035000

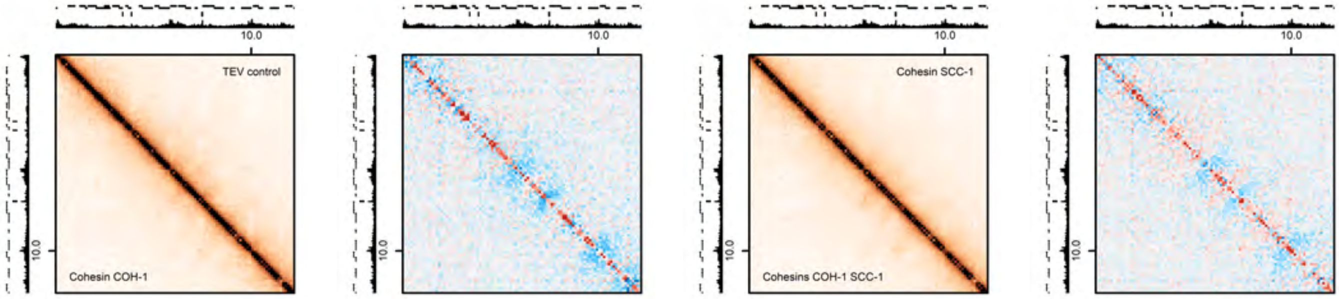

chrIII:4123001-4323000

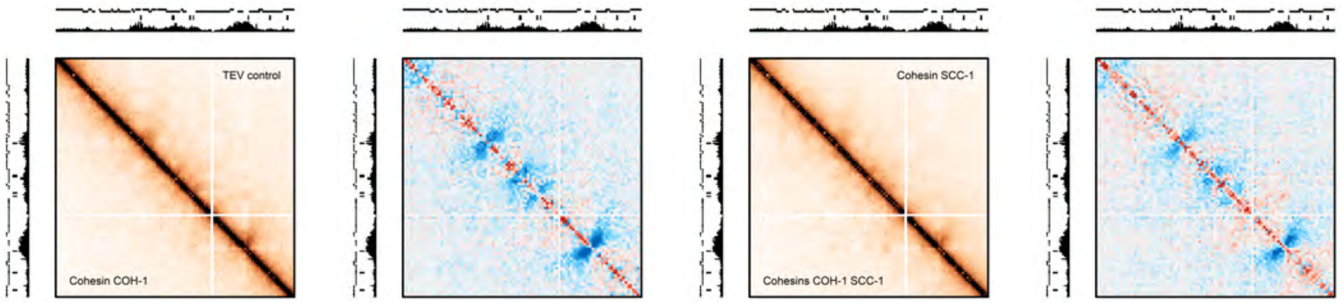

chrII:4653001-4853000

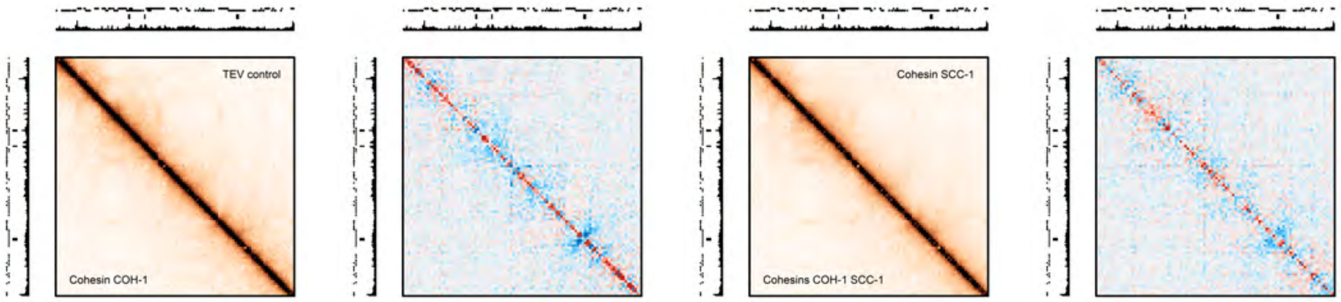

chrII:121001-321000

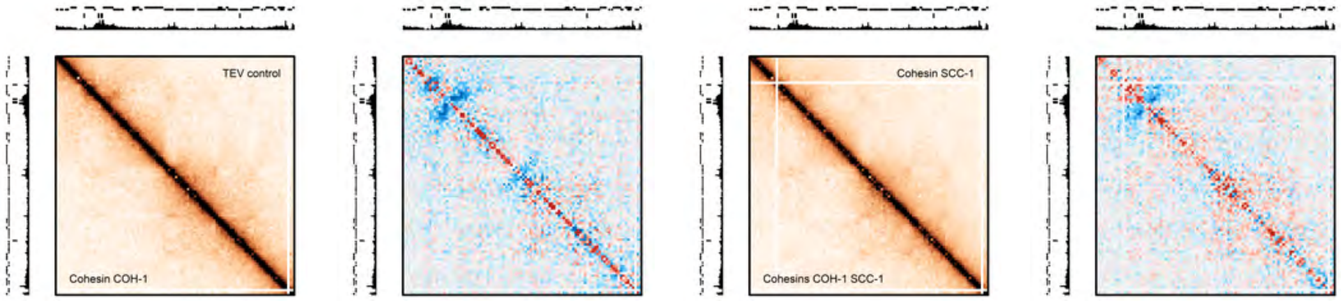

chrIV:9849001-10049000

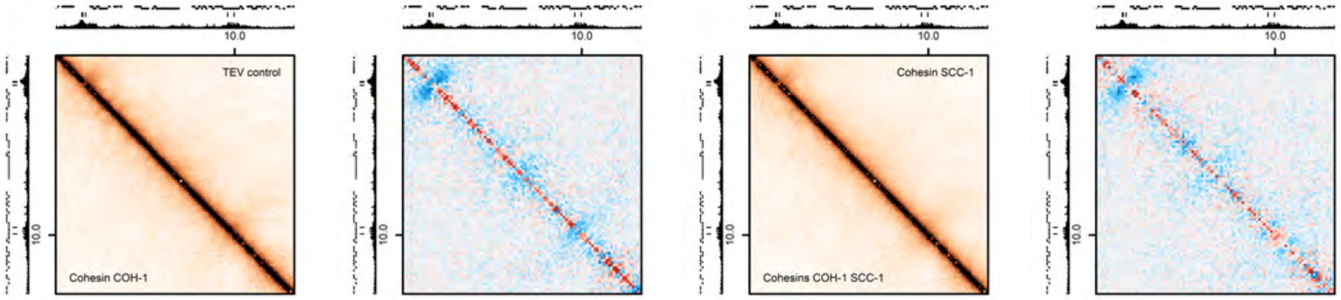

chrII:11297001-11497000

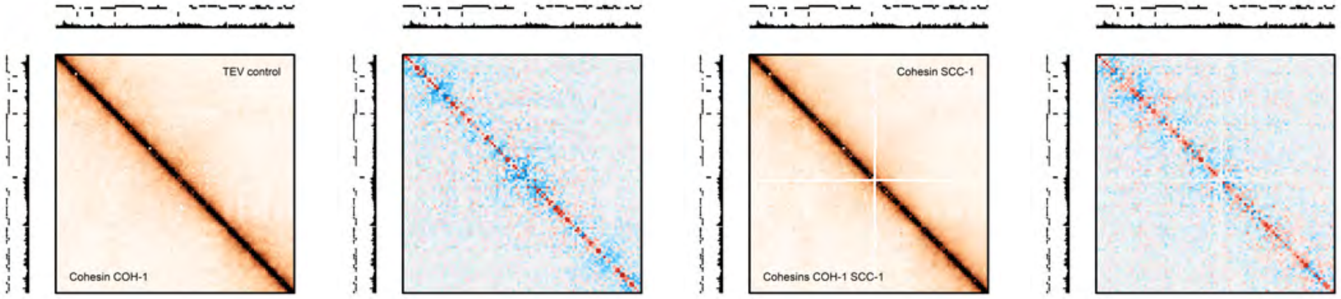

chrX:8567001-8767000

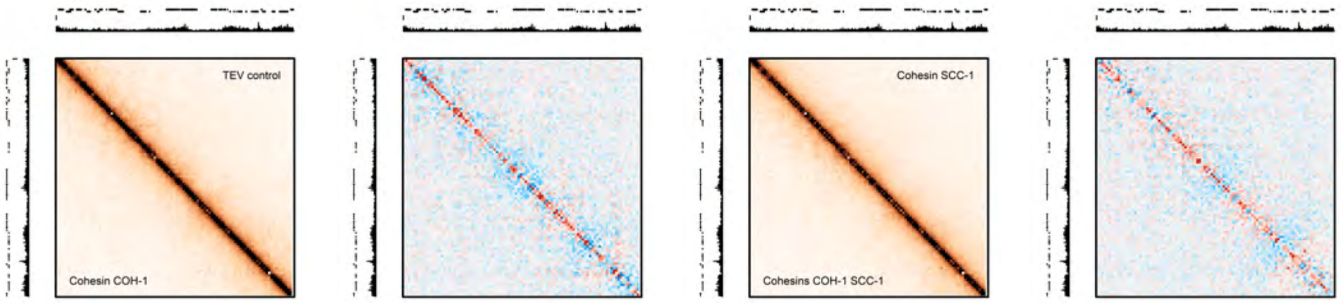

chrX:2805001-3005000

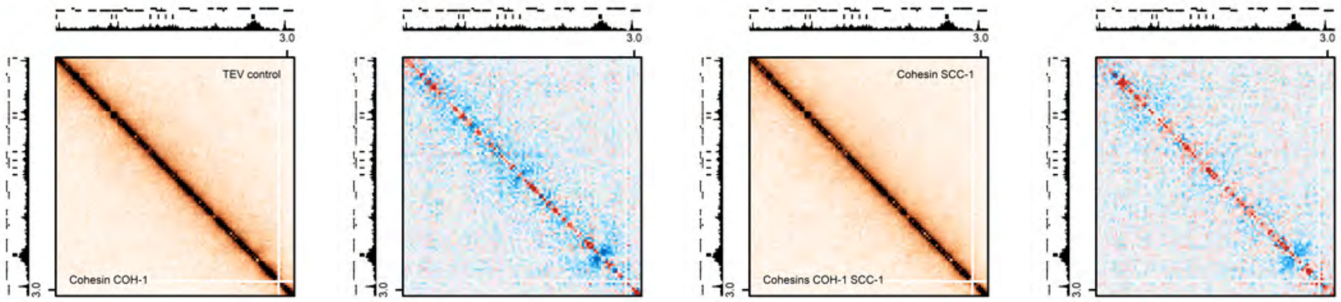

chrIV:13535001-13735000

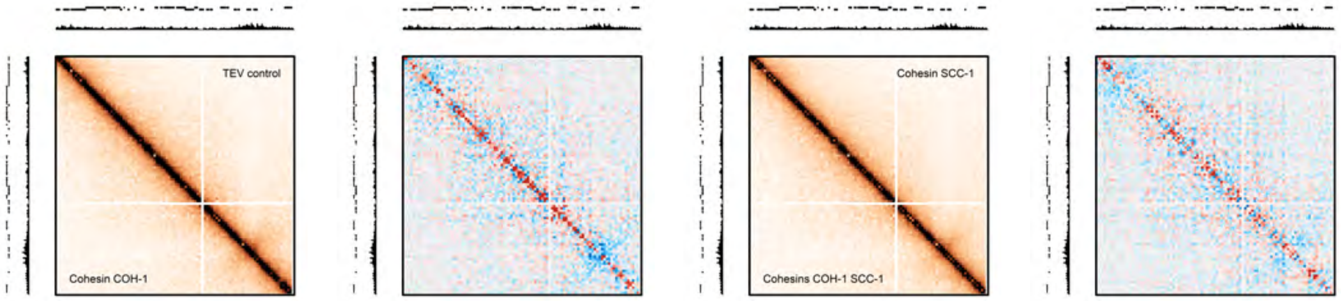

chrII:3207001-3407000

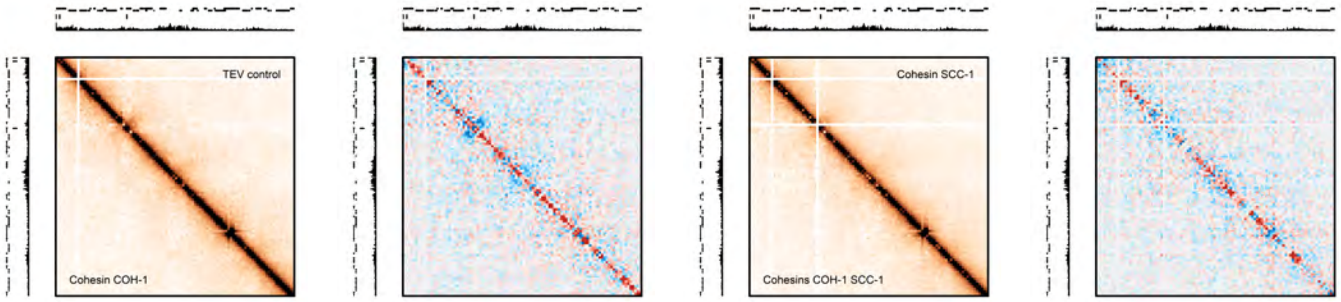

chrIV:275001-475000

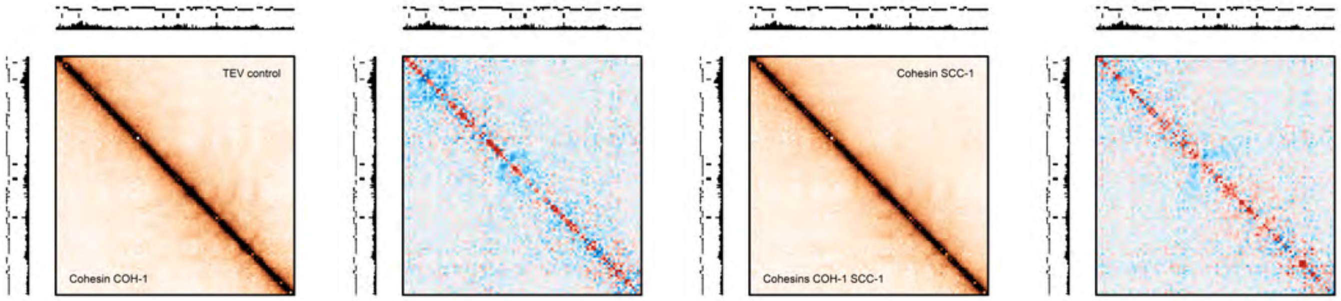

chrV:11757001-11957000

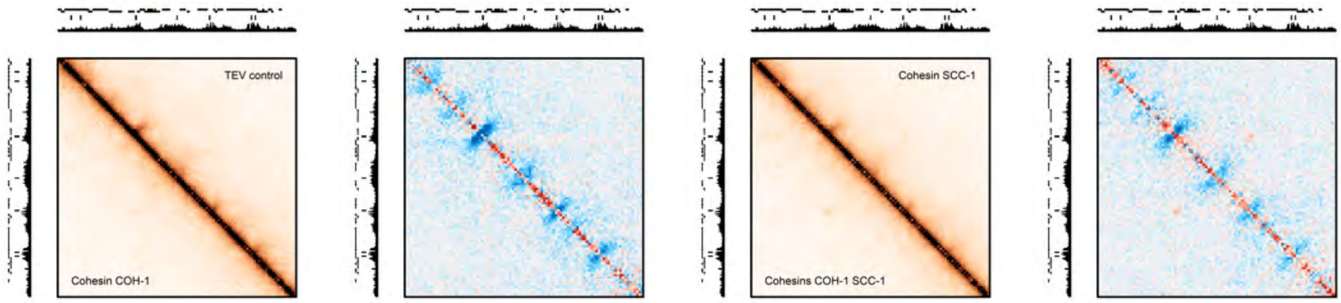

chrIII:1925001-2125000

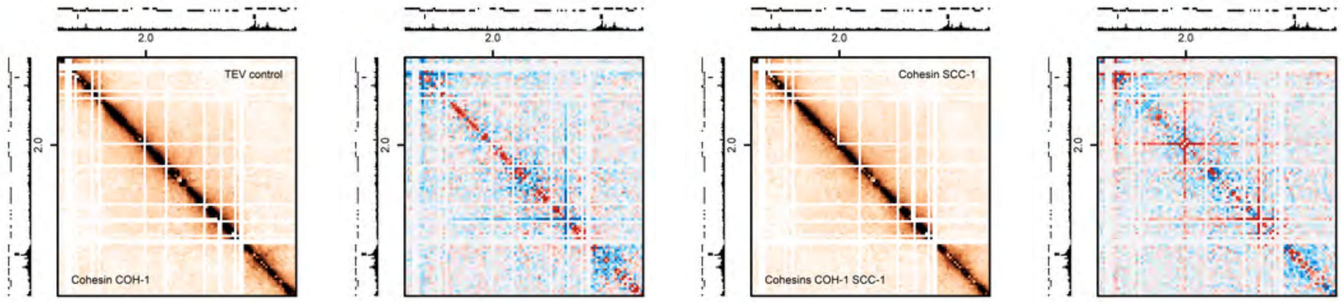

chrII:13855001-14055000

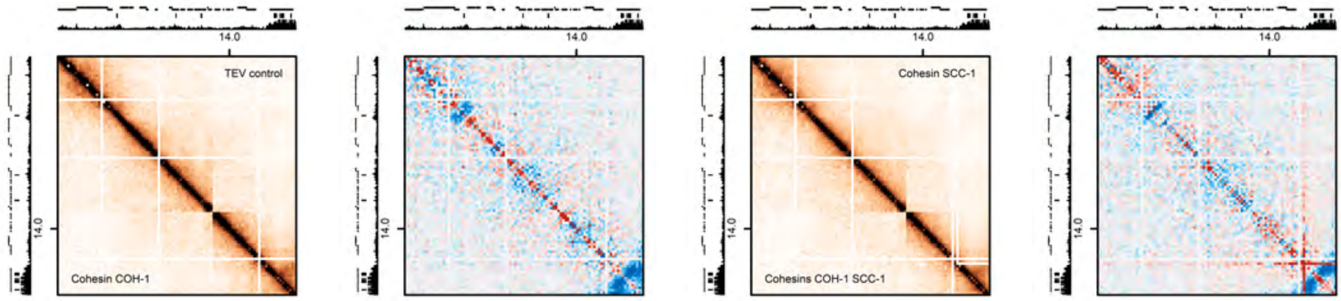

chrX:13533001-13733000

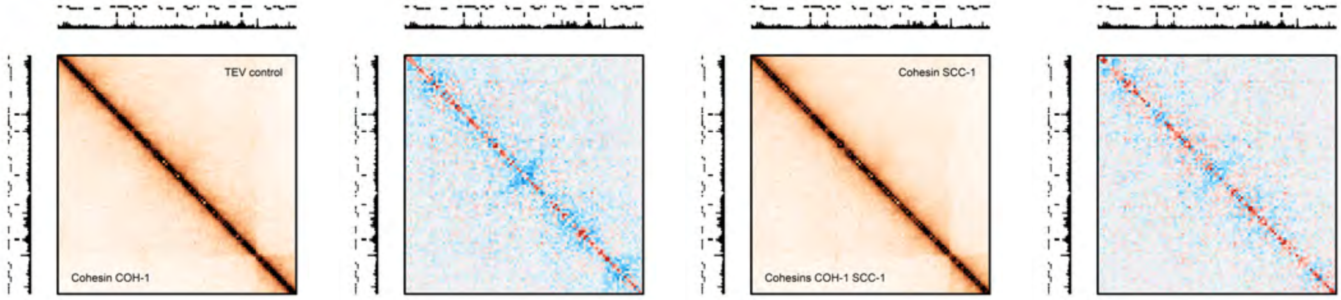

chrX:11723001-11923000

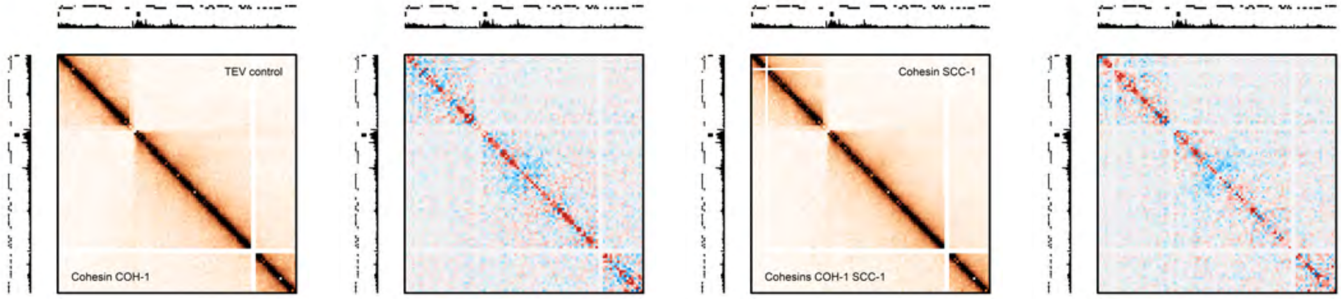

chrV:11335001-11535000

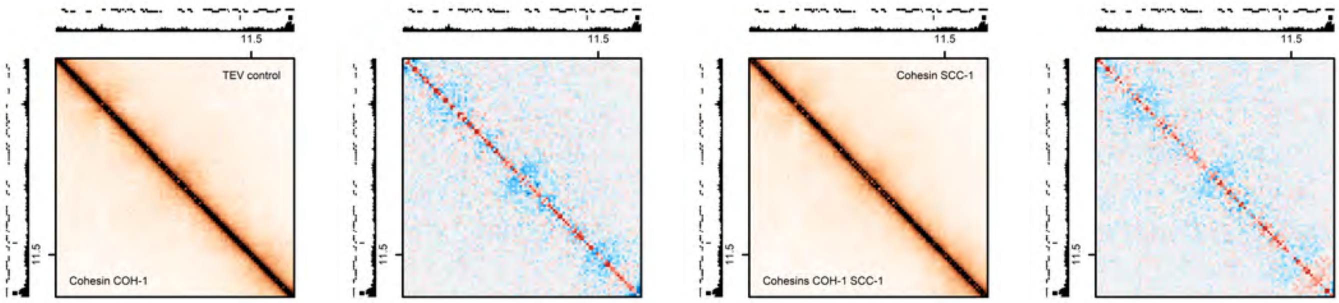

chrIII:11527001-11727000

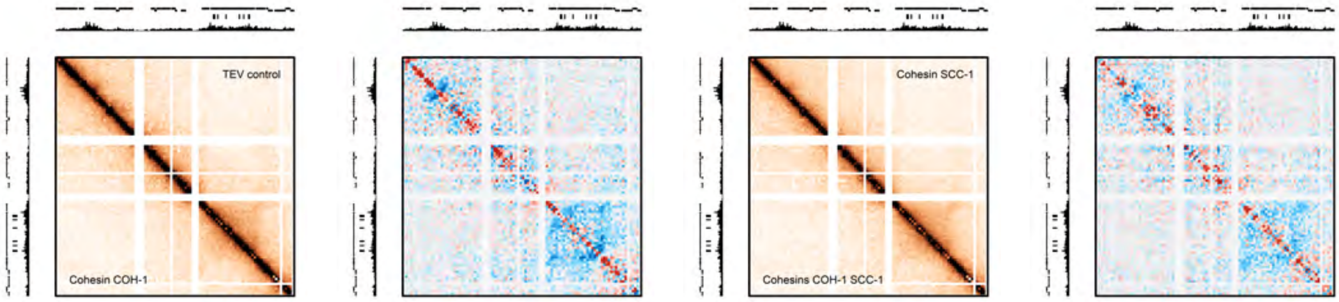

chrI:14665001-14865000

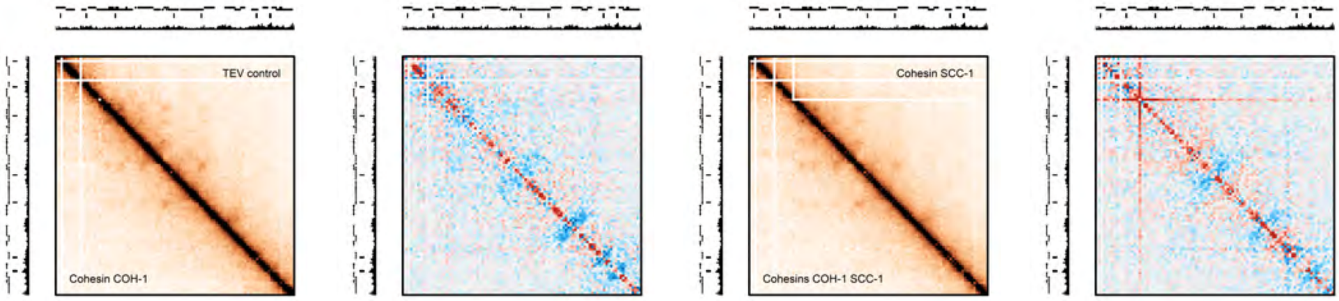

chrIII:519001-719000

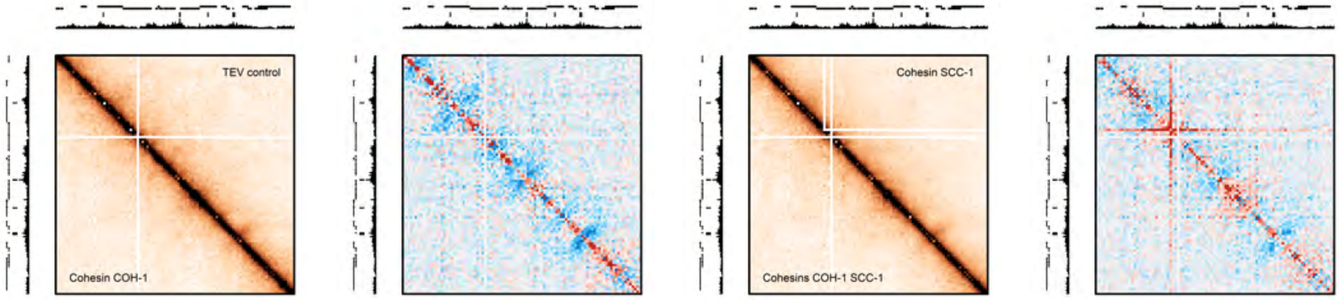

chrII:7621001-7821000

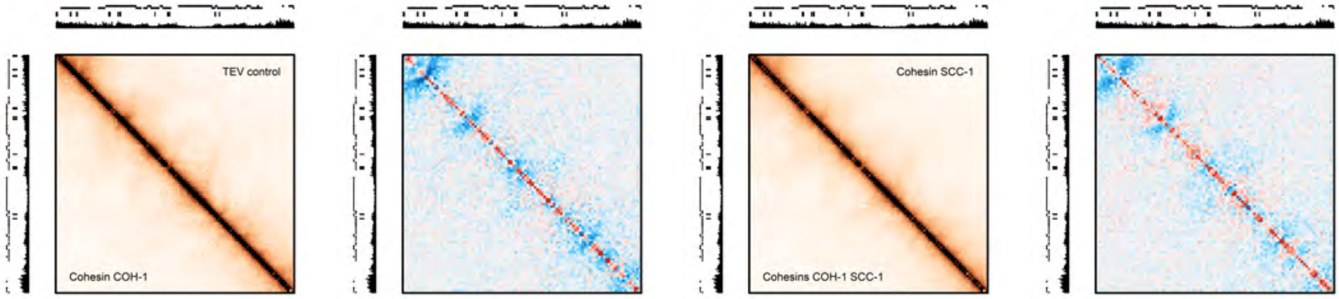

chrIV:4605001-4805000

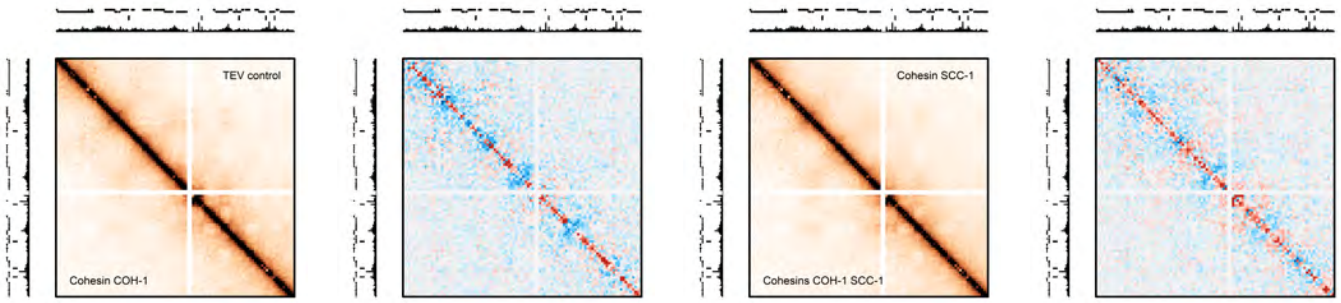

chrIV:14293001-14493000

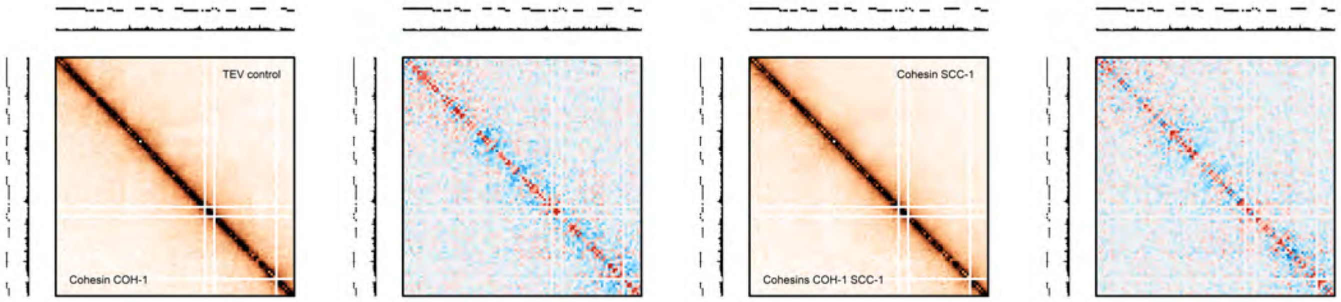

chrIII:2131001-2331000

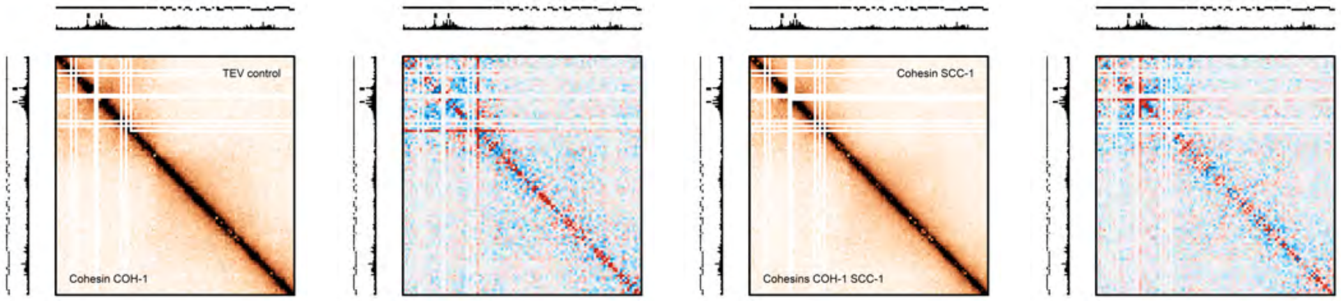

chrV:19727001-19927000

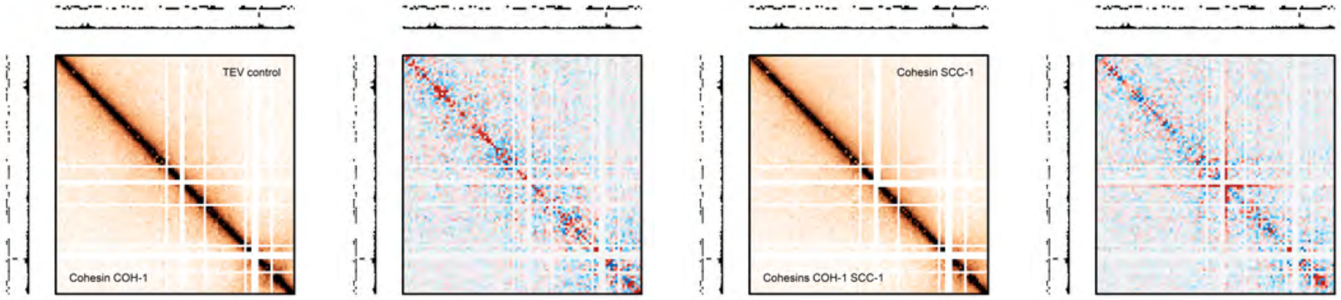

chrIII:5611001-5811000

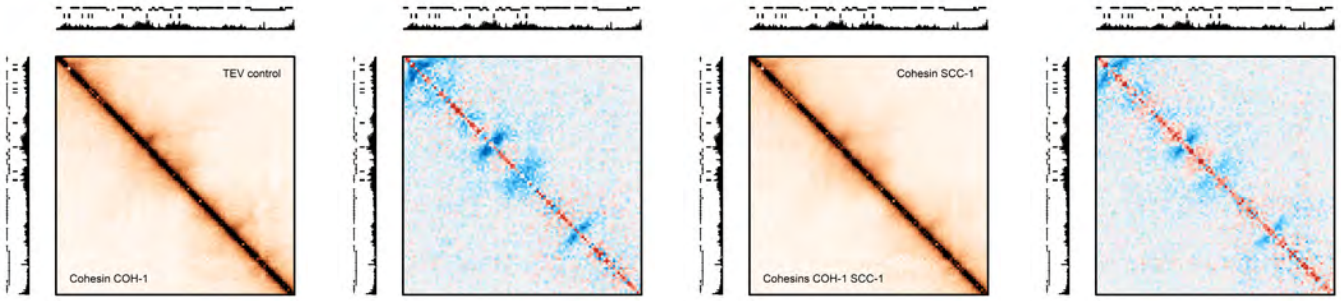

chrV:4053001-4253000

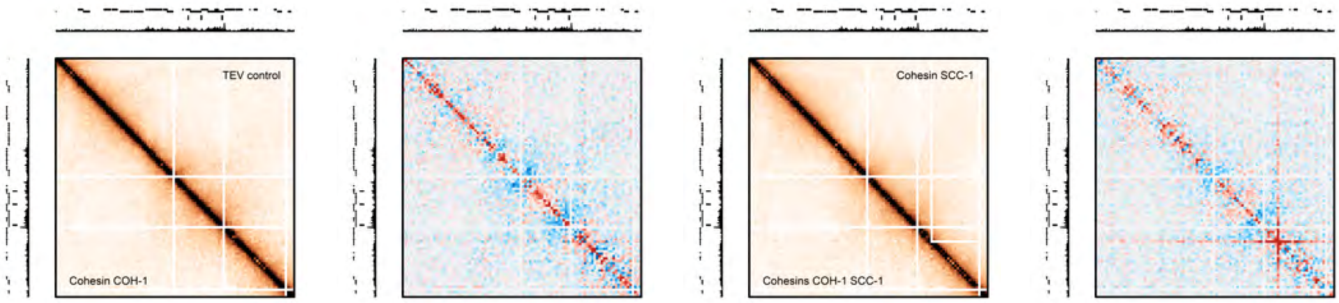

chrV:14593001-14793000

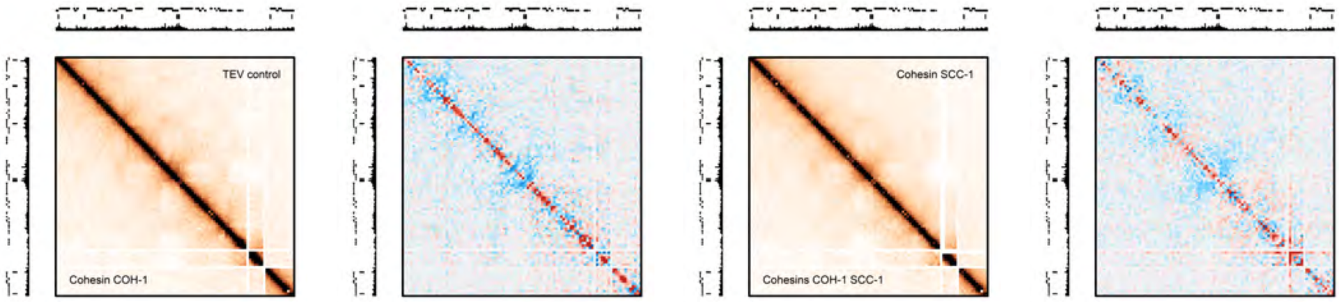

chrX:5159001-5359000

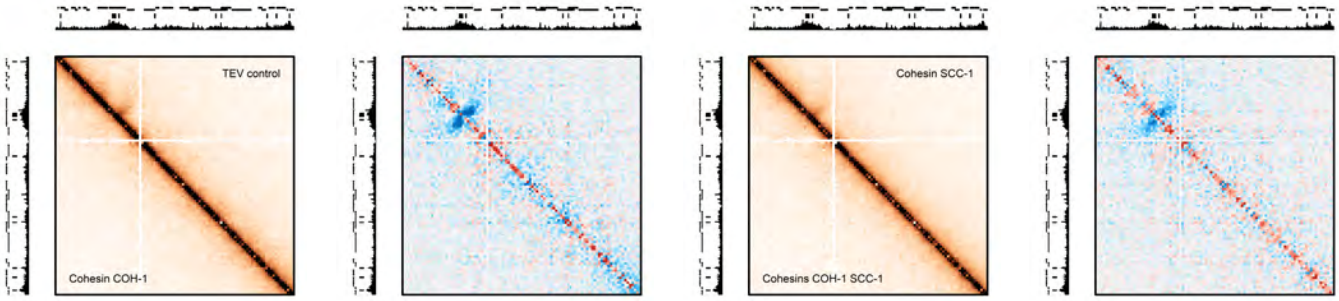

chrII:9473001-9673000

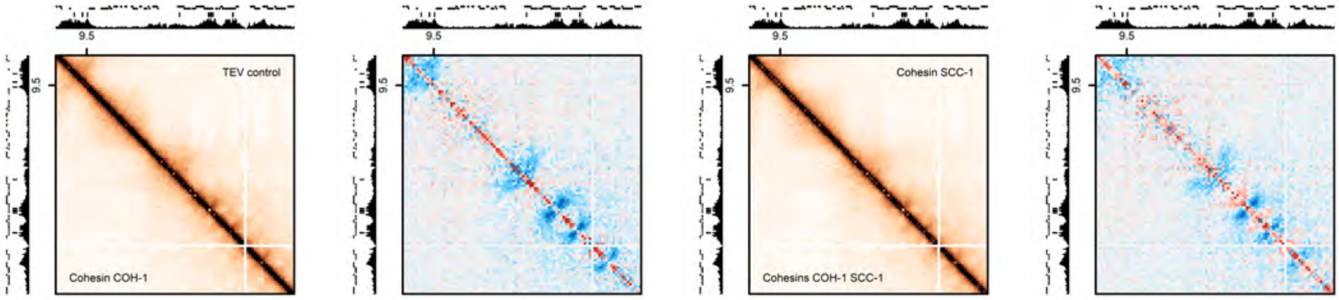

chrX:3215001-3415000

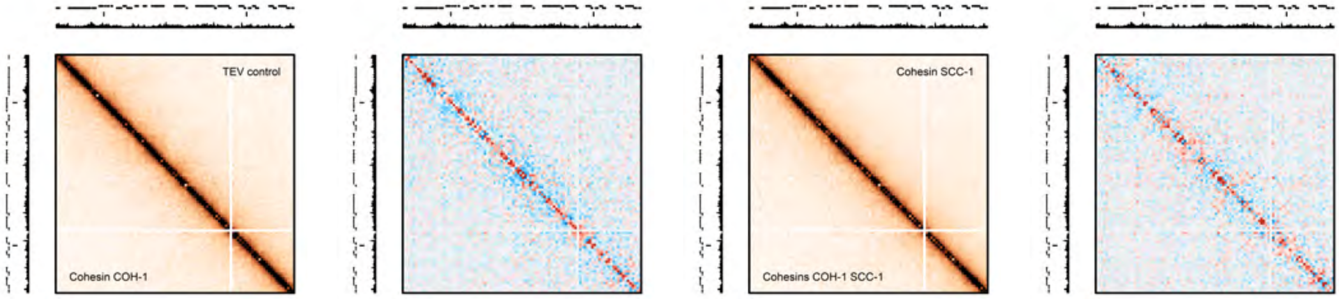

chrV:17155001-17355000

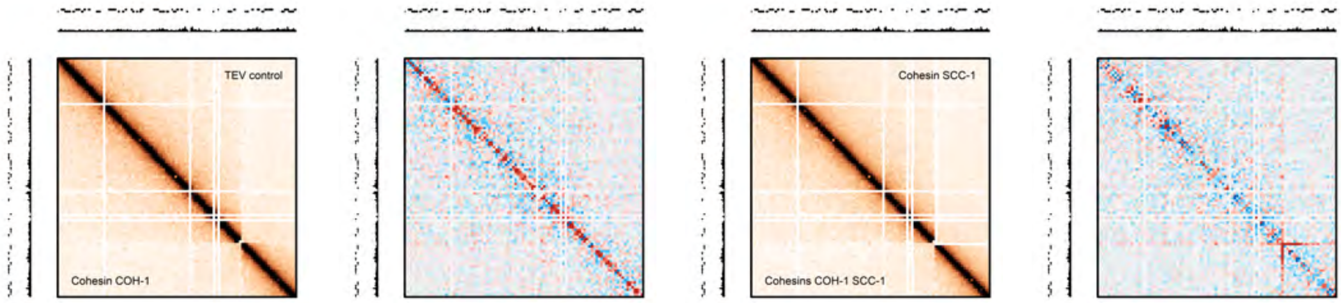

chrIV:17109001-17309000

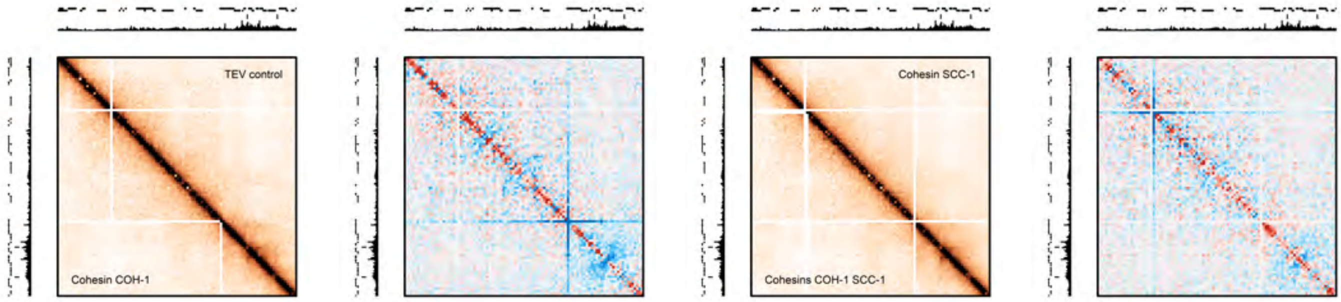

chrI:3583001-3783000

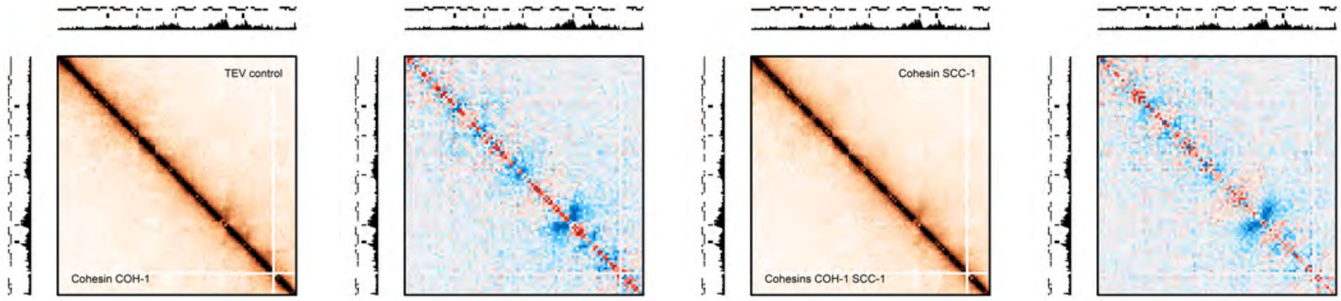

chrX:6309001-6509000

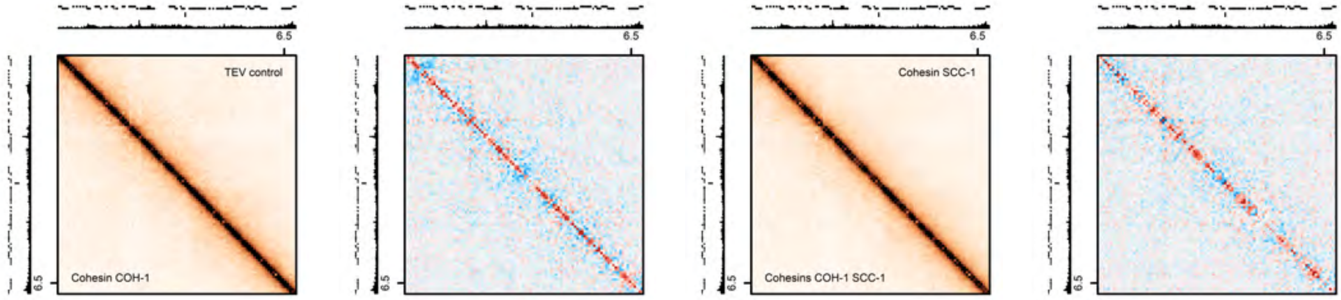

chrIII:5039001-5239000

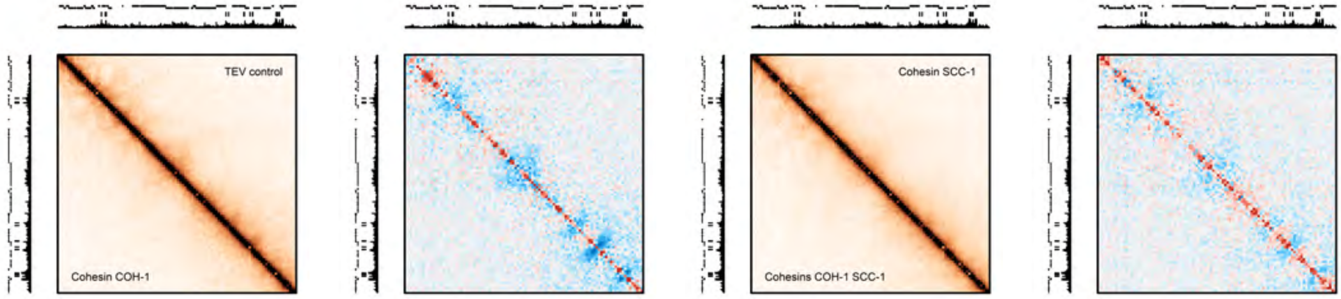

chrV:1573001-1773000

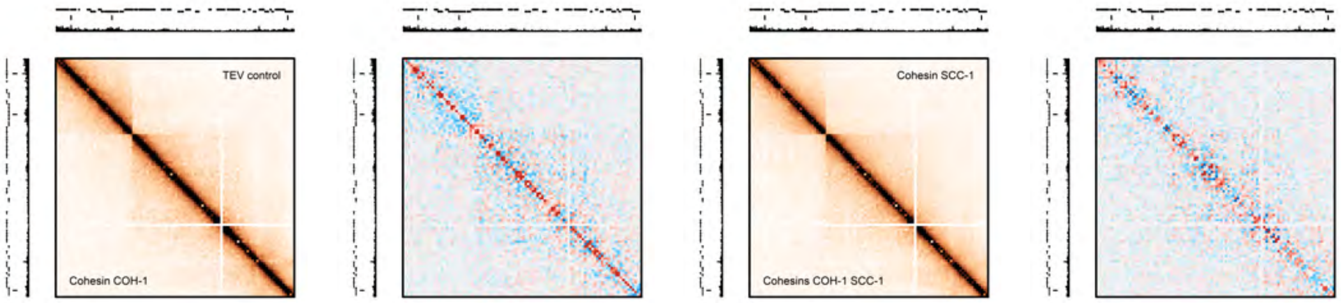

chrII:2493001-2693000

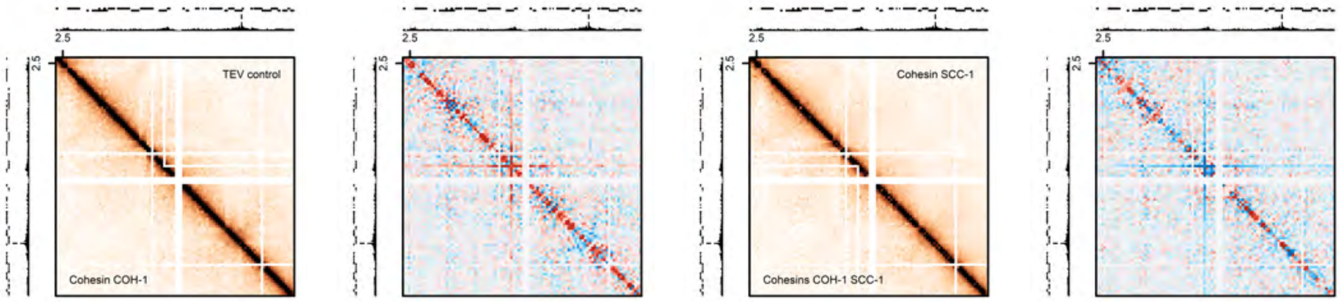

chrII:10107001-10307000

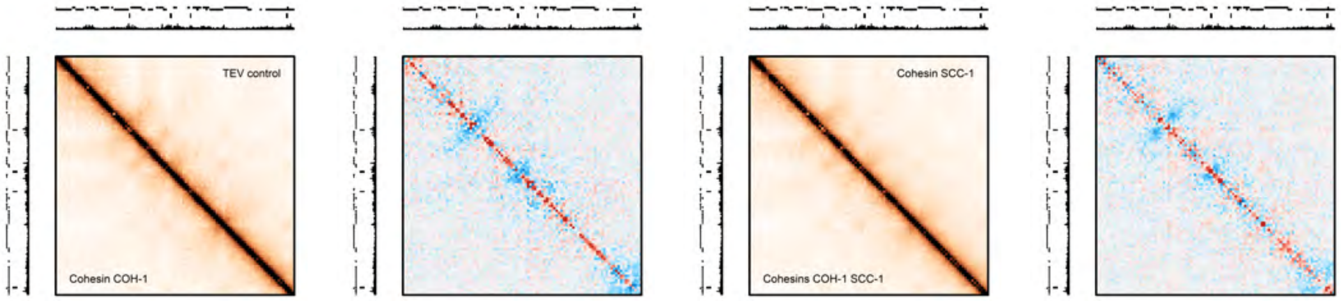

chrX:5999001-6199000

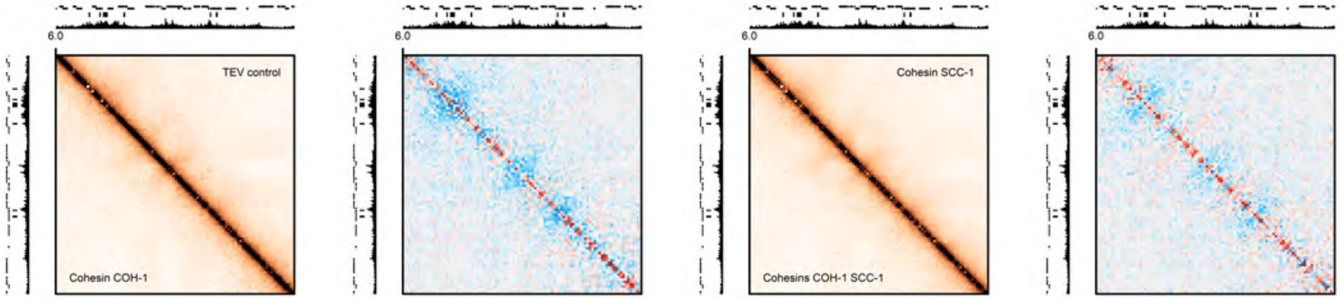

chrIII:3113001-3313000

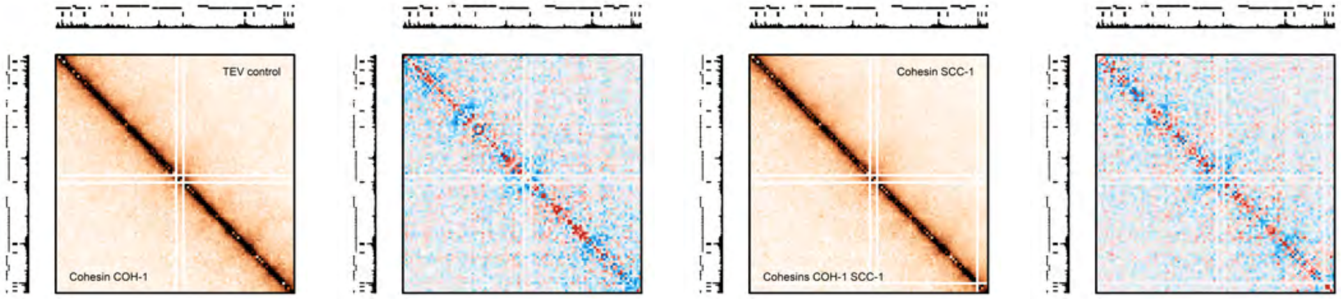

chrX:957001-1157000

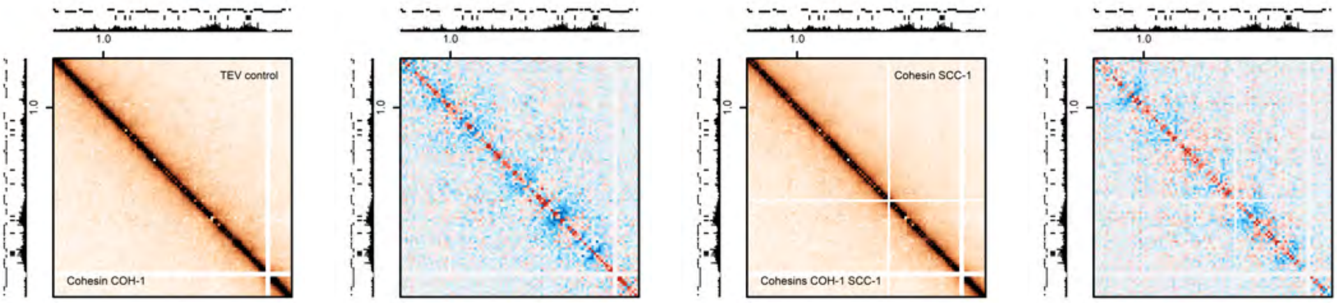

chrII:-38999-161000

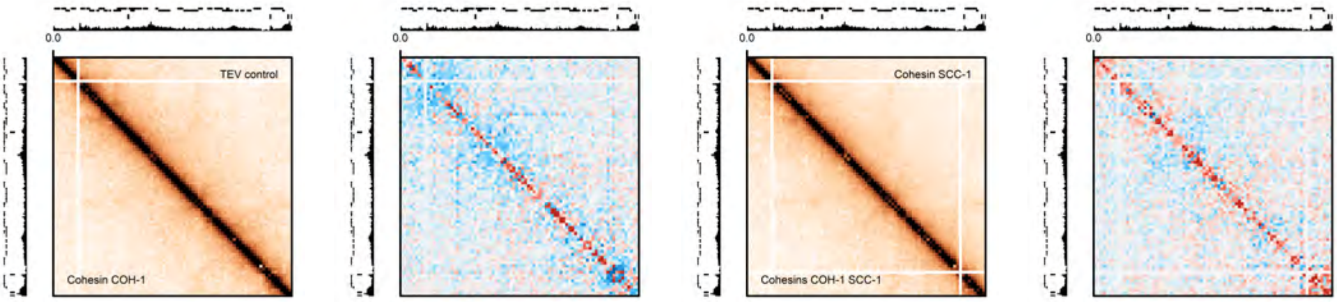

chrV:6041001-6241000

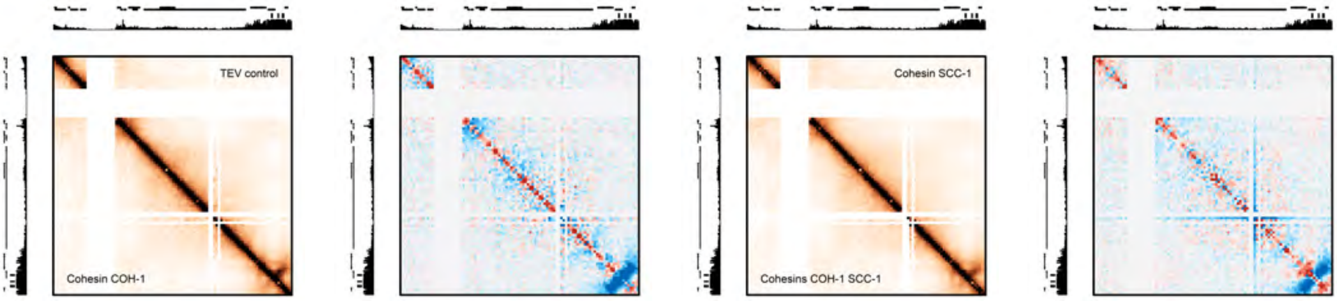

chrII:10403001-10603000

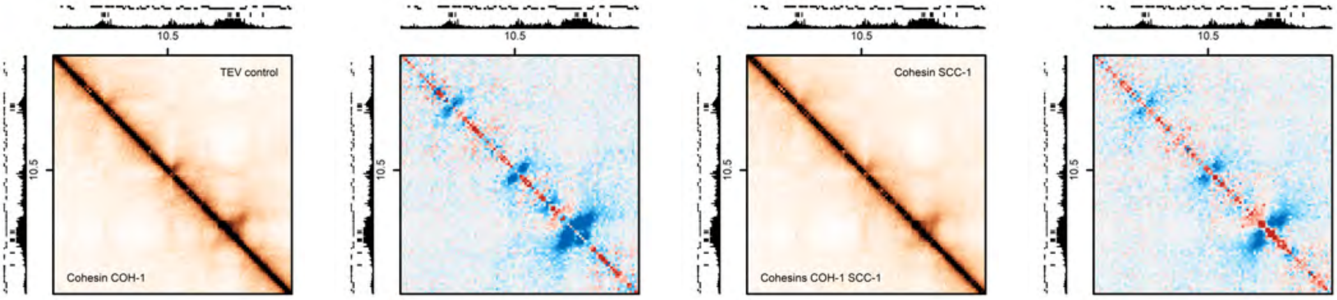

chrV:3927001-4127000

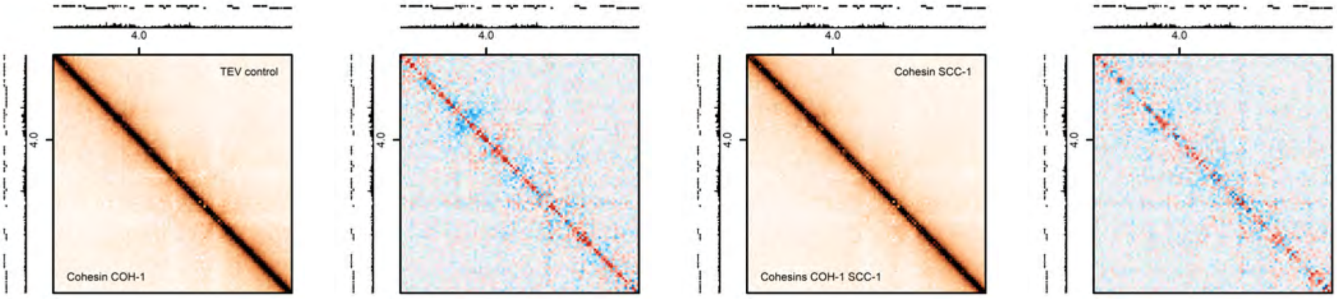

chrII:3113001-3313000

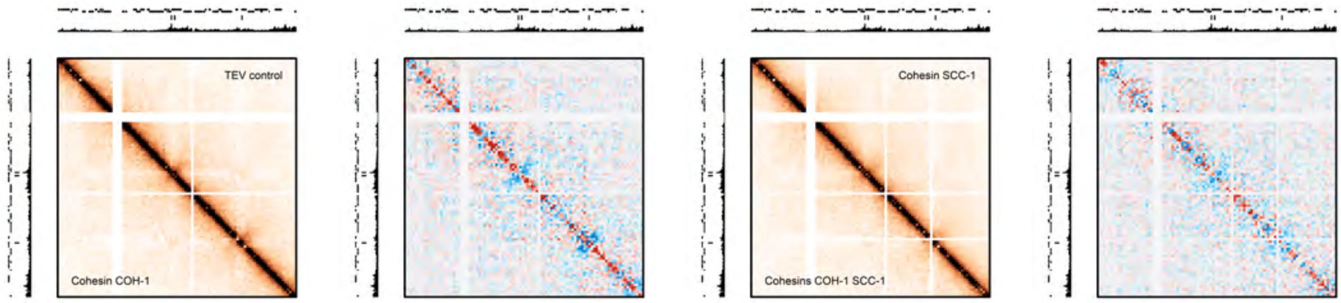

chrIII:6591001-6791000

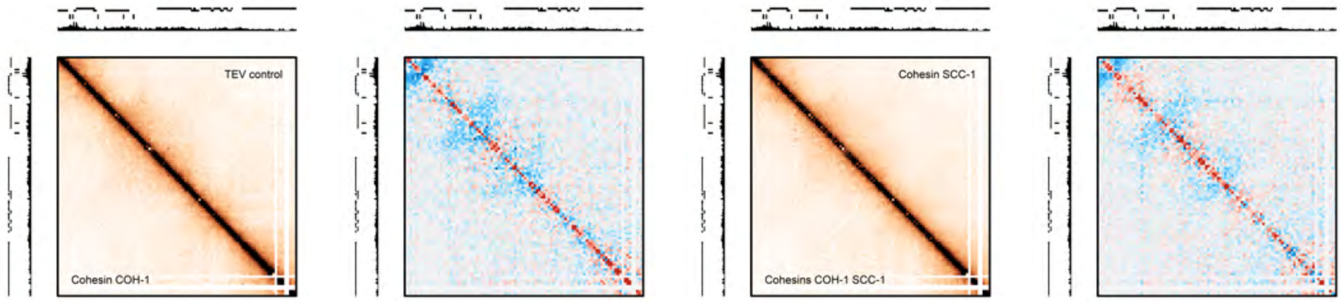

chrI:12009001-12209000

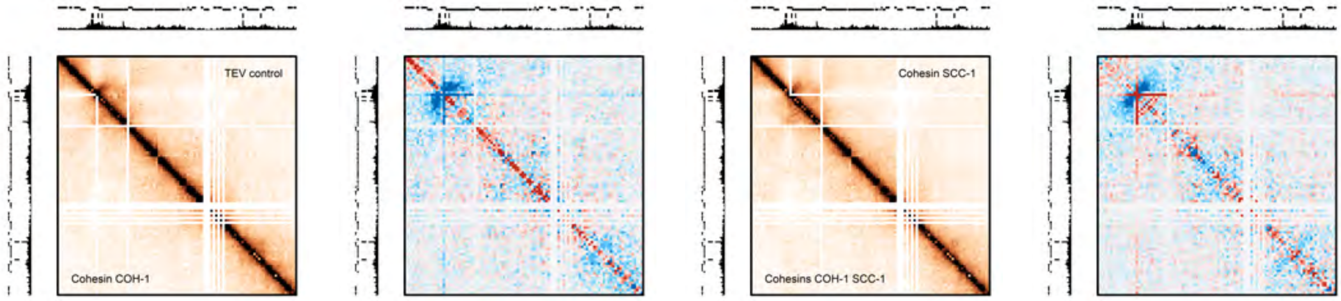

chrX:10471001-10671000

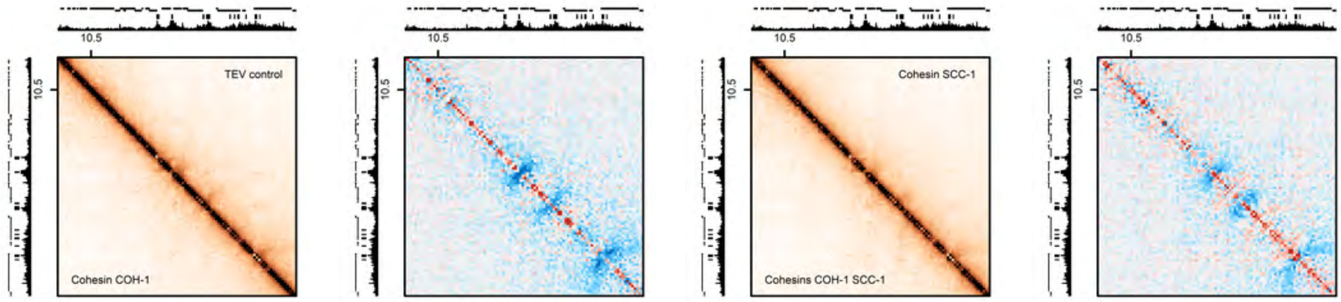

chrX:5409001-5609000

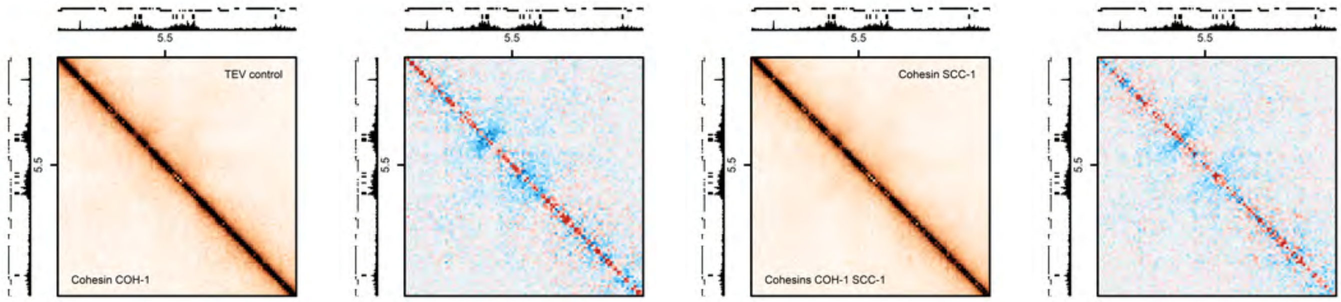

chrII:1551001-1751000

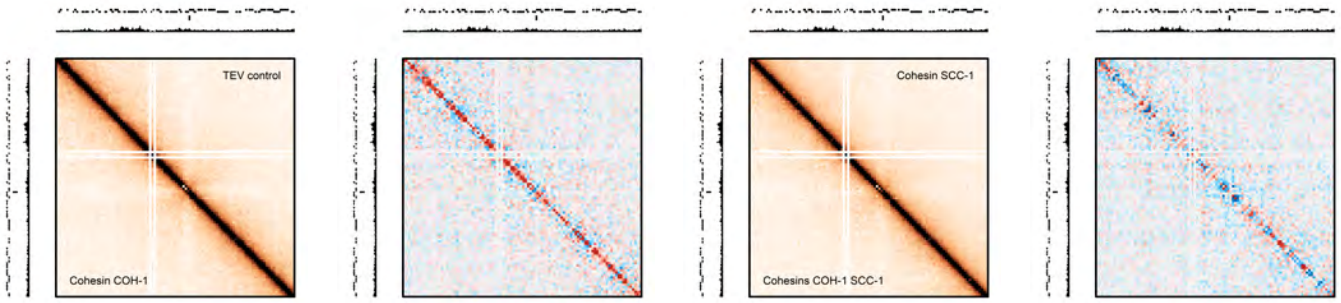

chrX:16349001-16549000

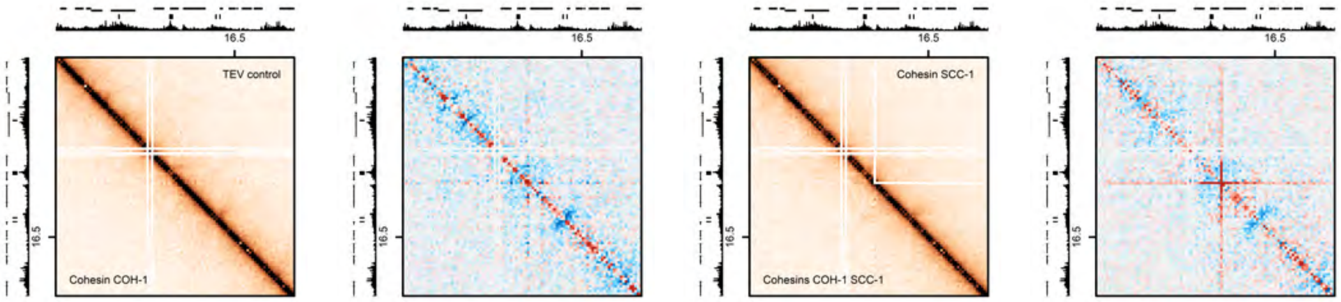

chrV:3351001-3551000

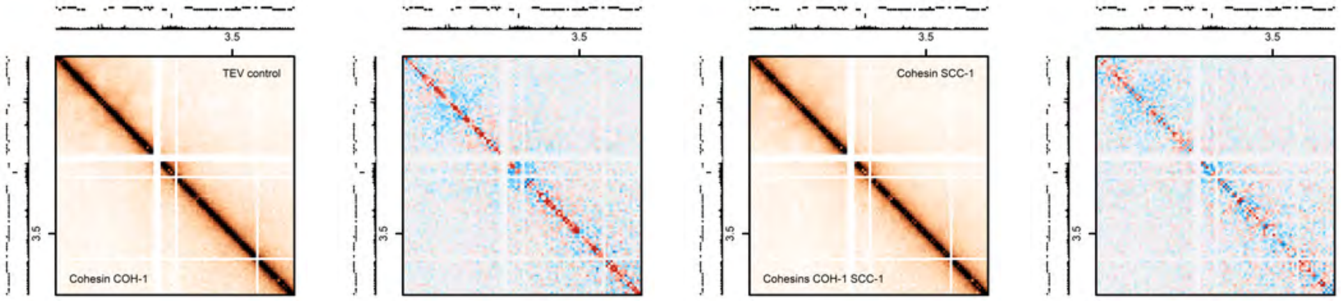

chrX:4563001-4763000

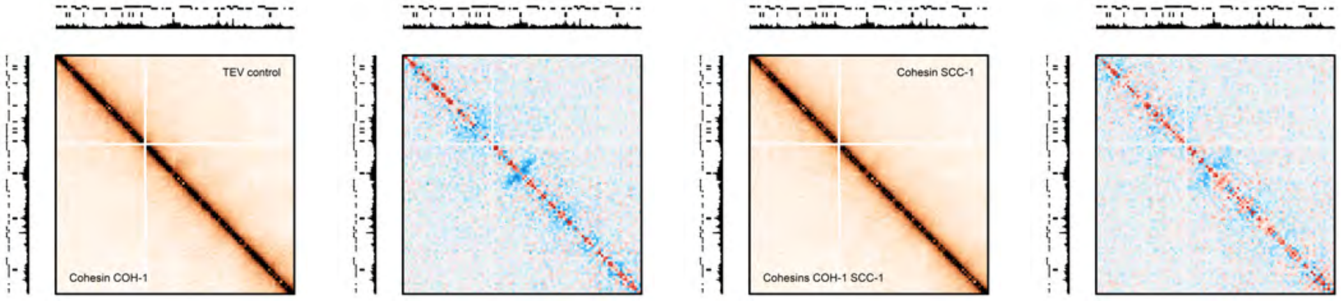

chrIII:1999001-2199000

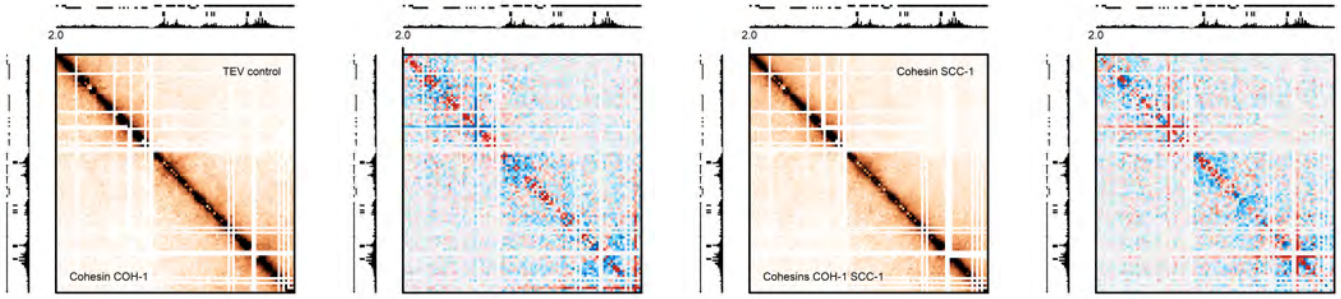

chrIII:7557001-7757000

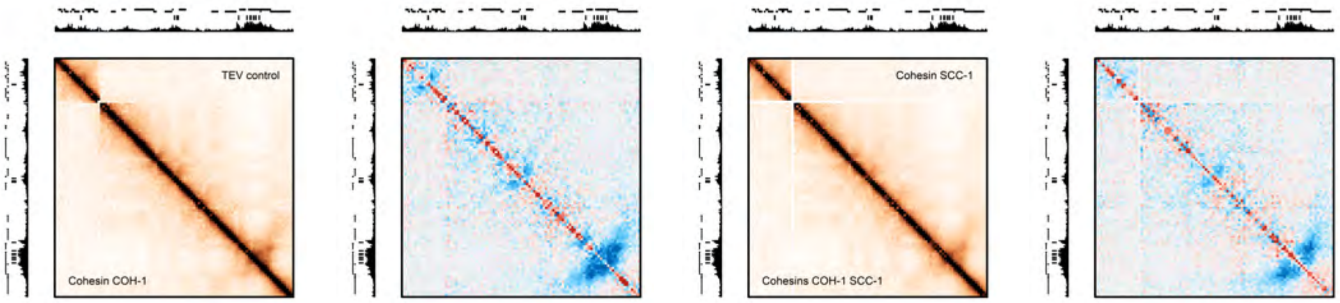

chrX:16387001-16587000

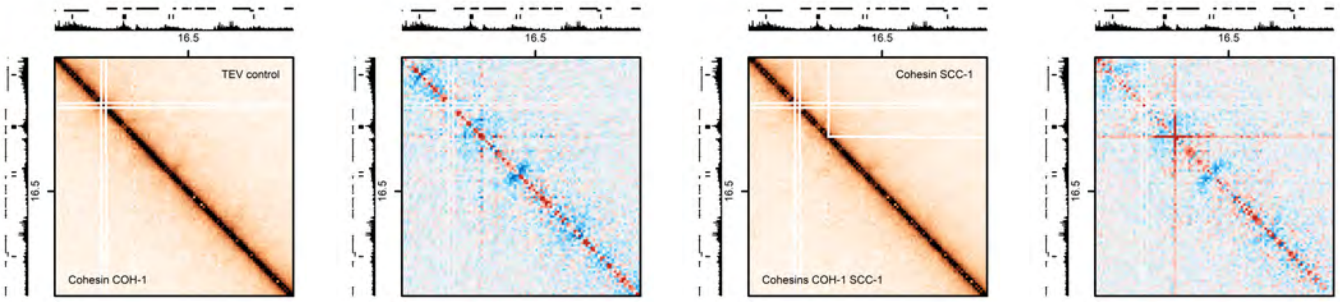

chrV:187001-387000

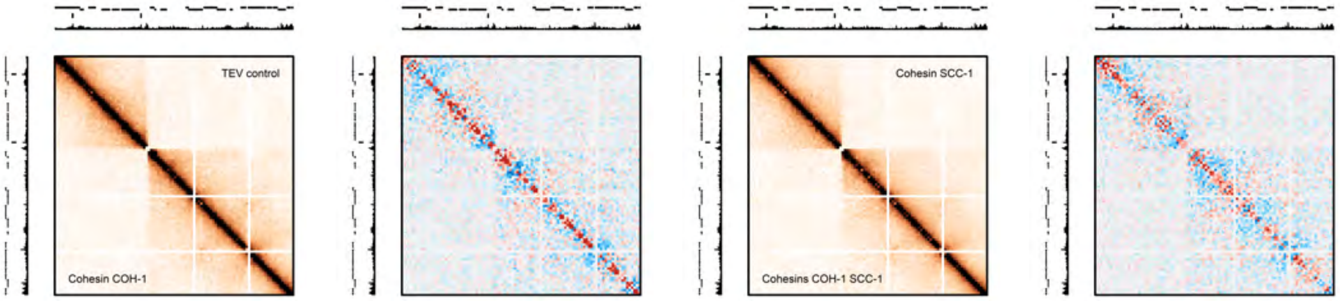

chrIII:10379001-10579000

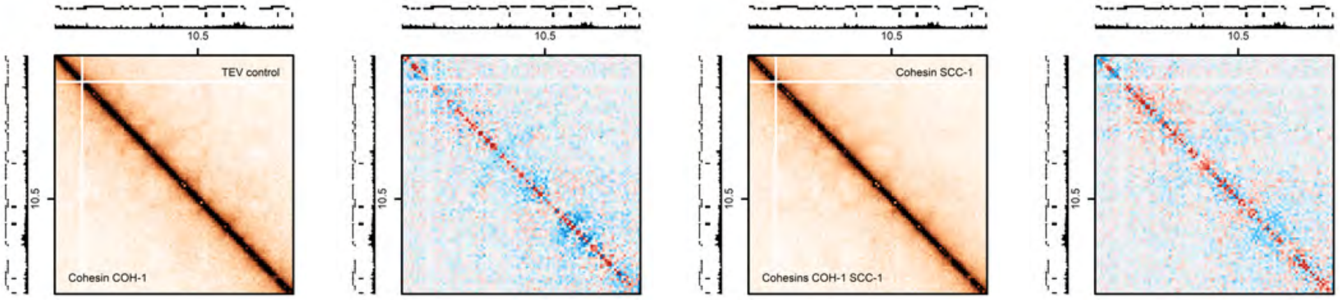

chrIV:6553001-6753000

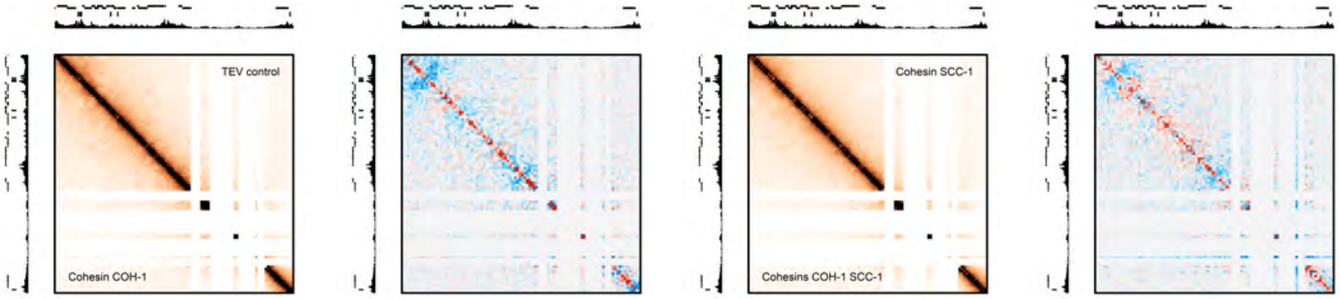

chrI:14177001-14377000

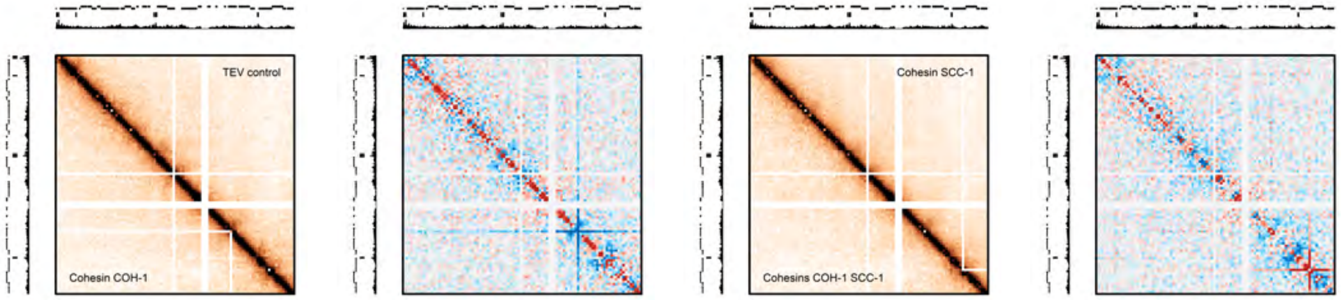

chrIV:77001-277000

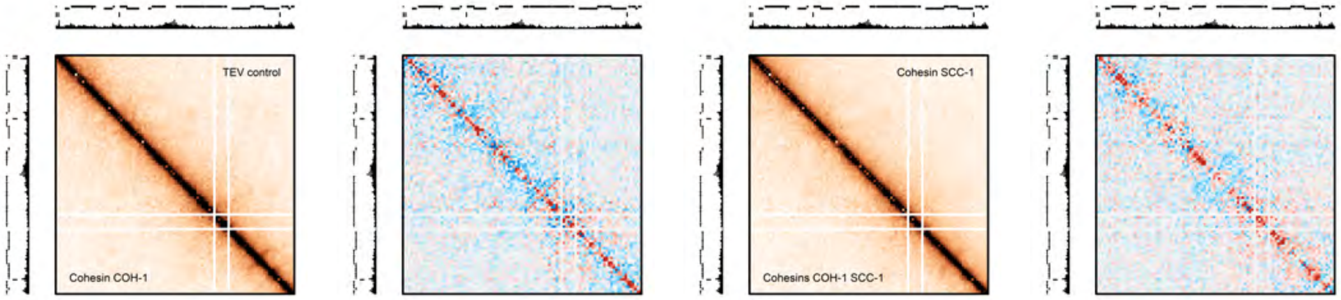

chrV:3535001-3735000

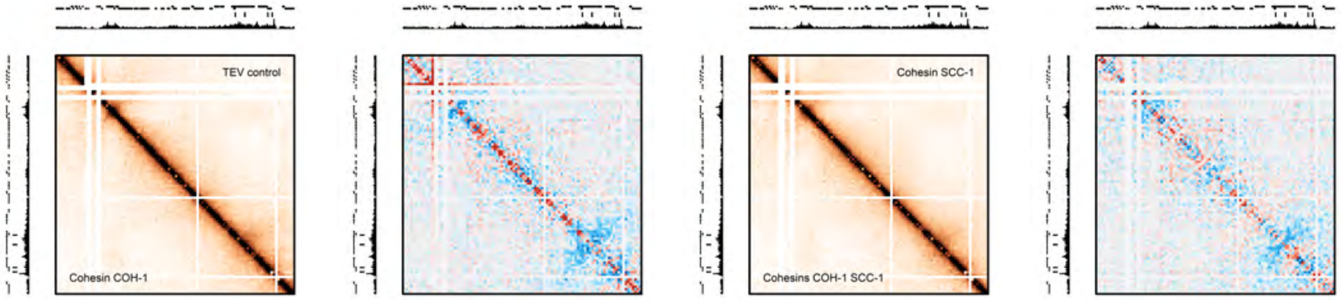

chrII:8321001-8521000

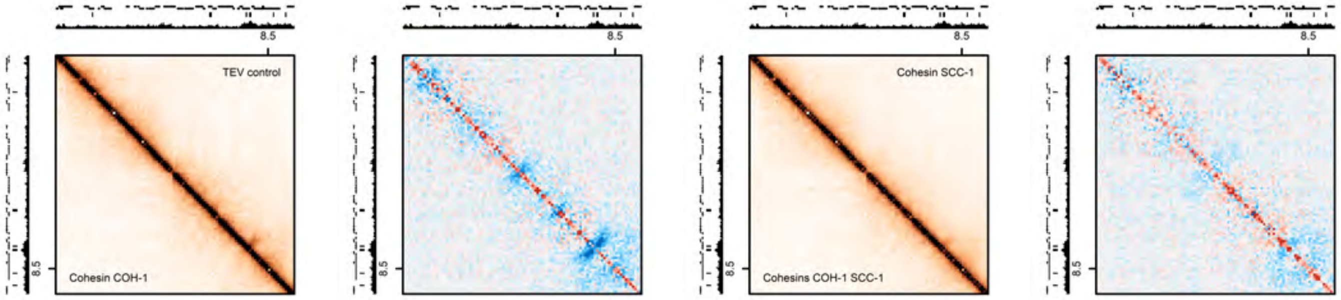

chrX:335001-535000

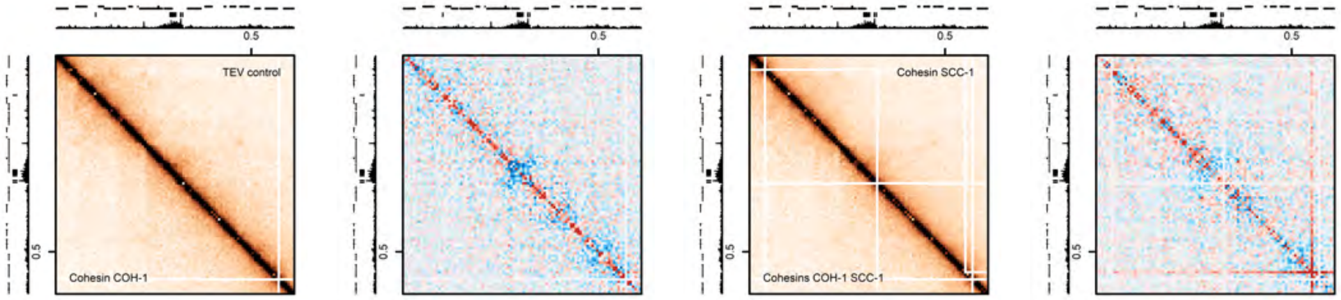

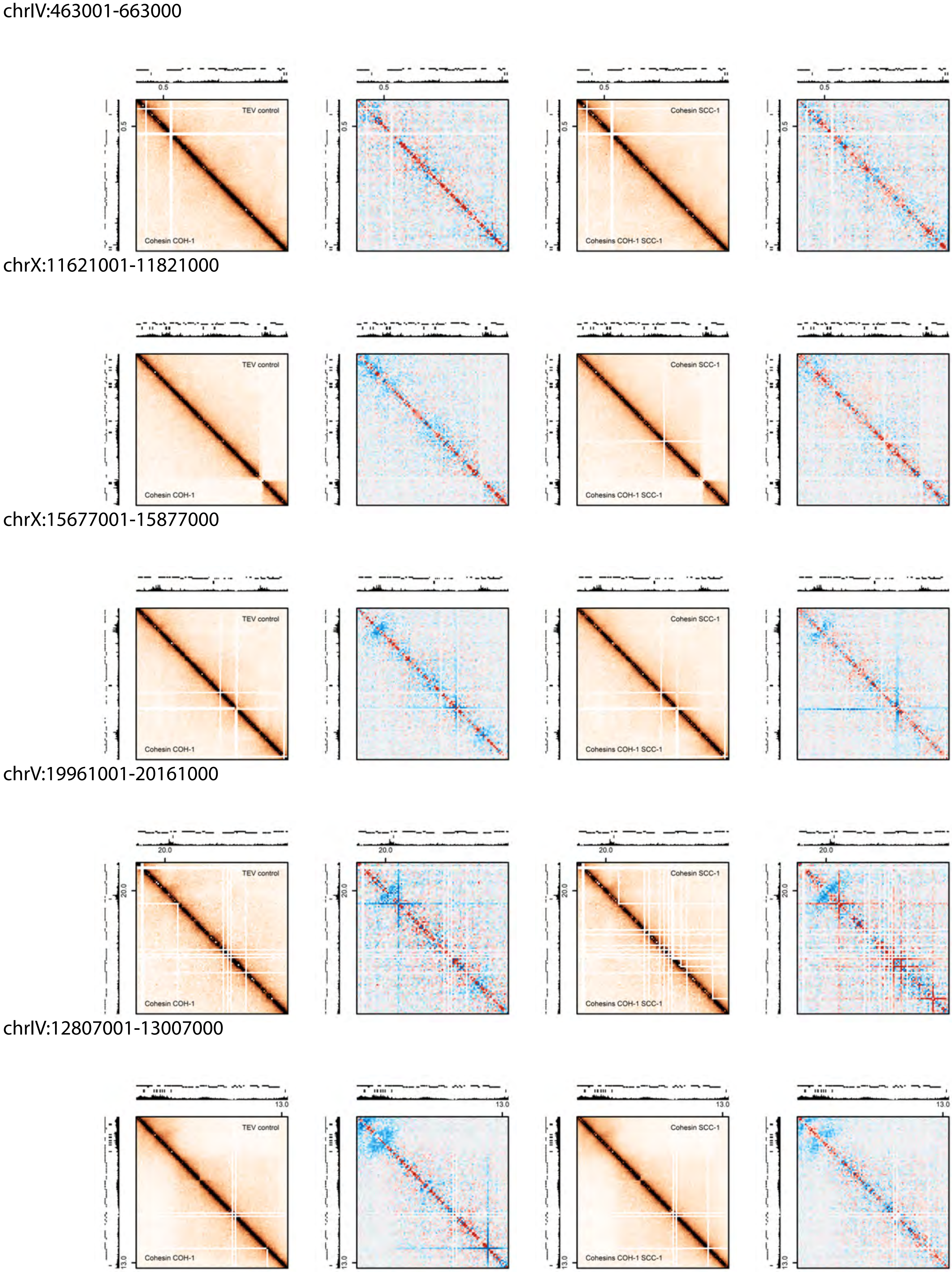
chrIV:463001-663000
TEV control
Cohesin COH-1
Cohesin SCC-1
Cohesins COH-1 SCC-1
0.5
chrX:11621001-11821000
chrX:15677001-15877000
chrV:19961001-20161000
20.0
chrIV:12807001-13007000
13.0

chrIV:12983001-13183000

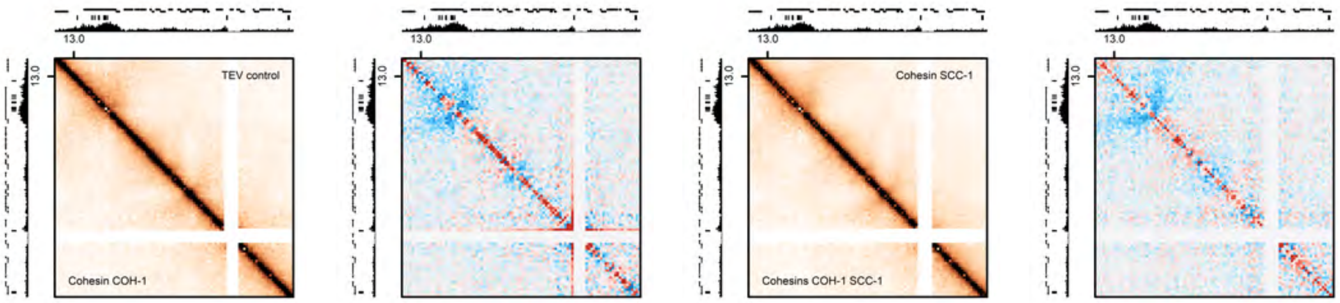

chrV:14913001-15113000

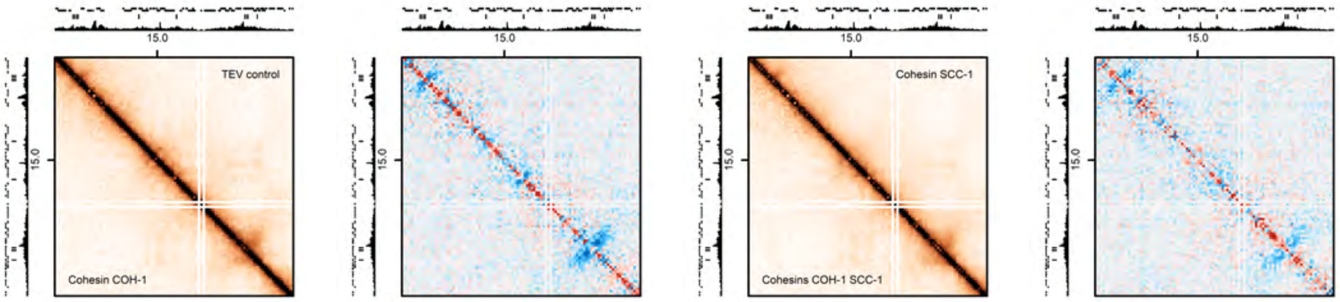

chrV:12113001-12313000

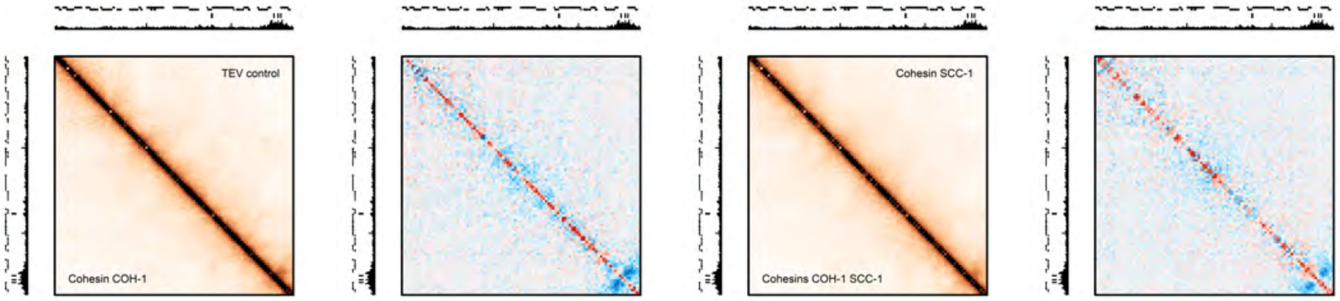

chrII:2733001-2933000

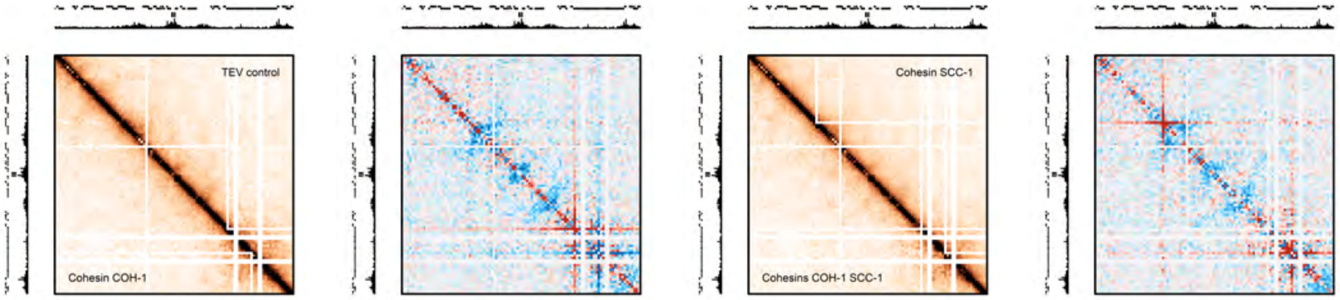

chrIV:7519001-7719000

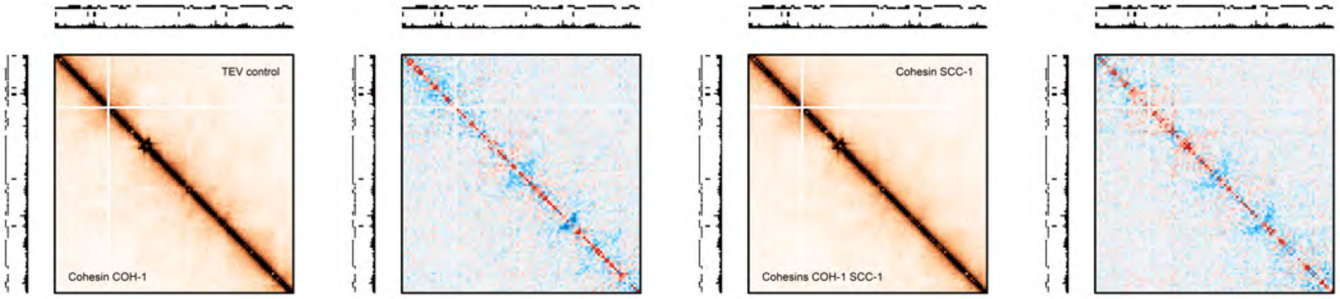

chrIV:2321001-2521000

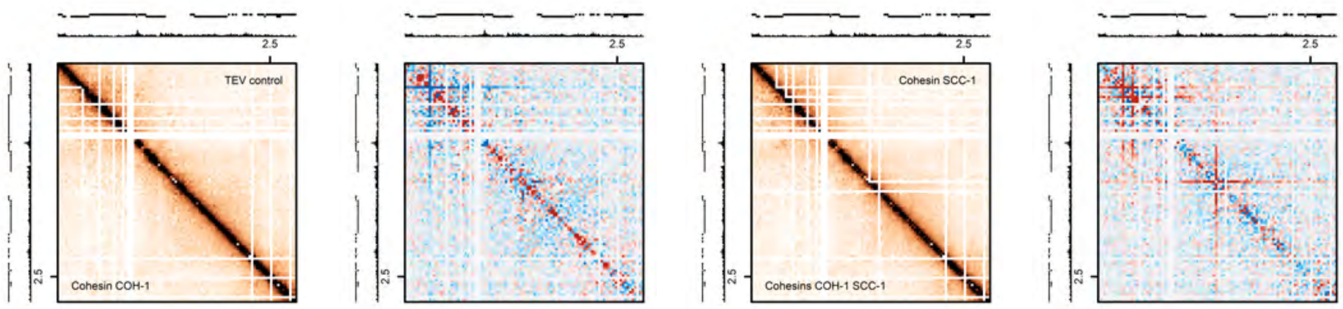

chrV:17757001-17957000

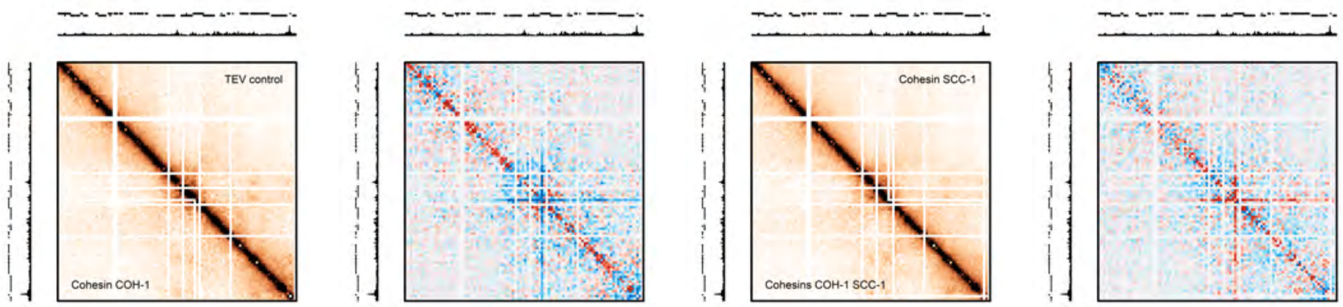

chrIII:10479001-10679000

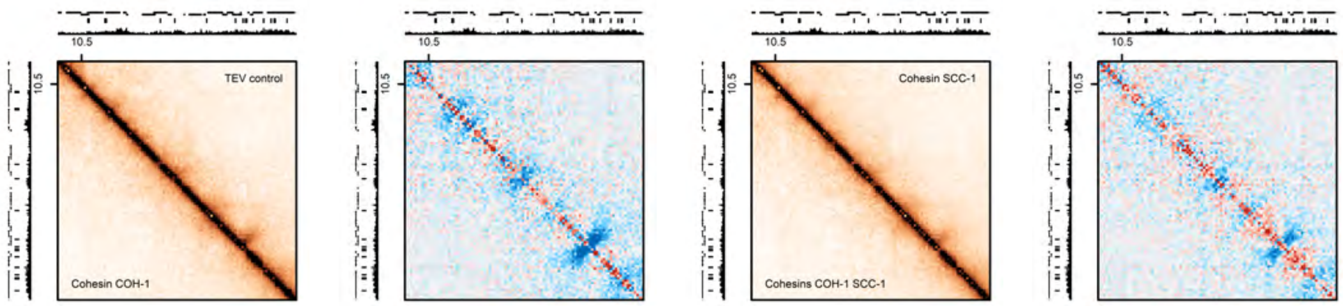

chrX:14639001-14839000

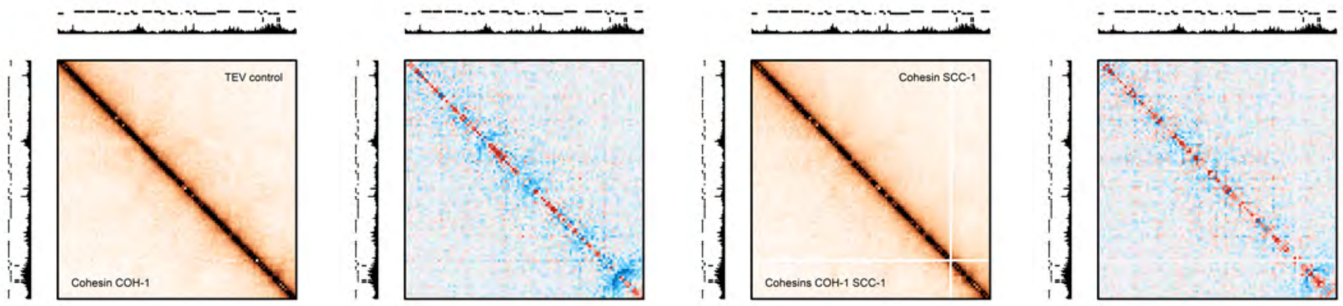

chrX:9273001-9473000

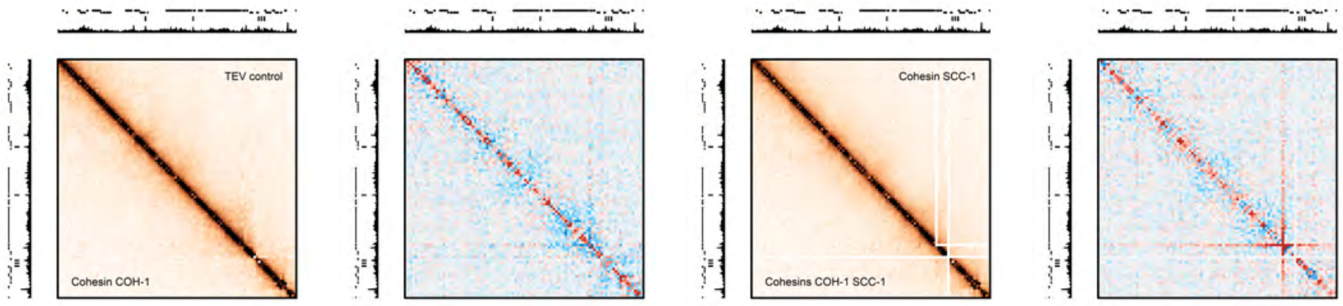

chrIV:11425001-11625000

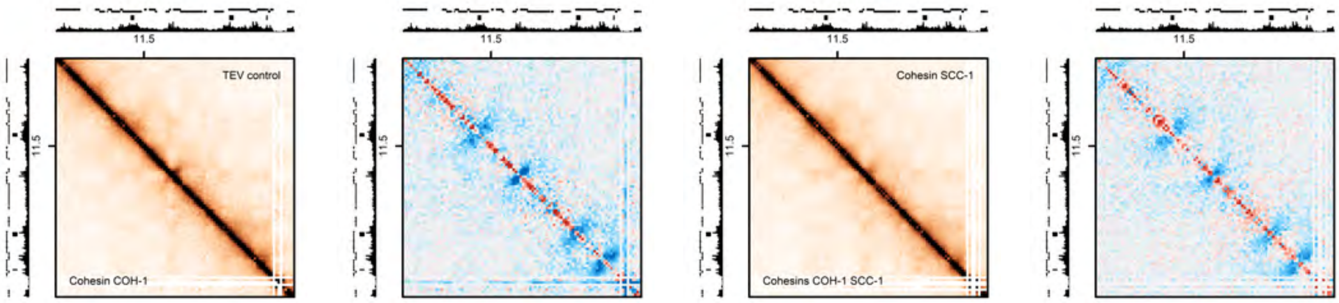

chrII:11741001-11941000

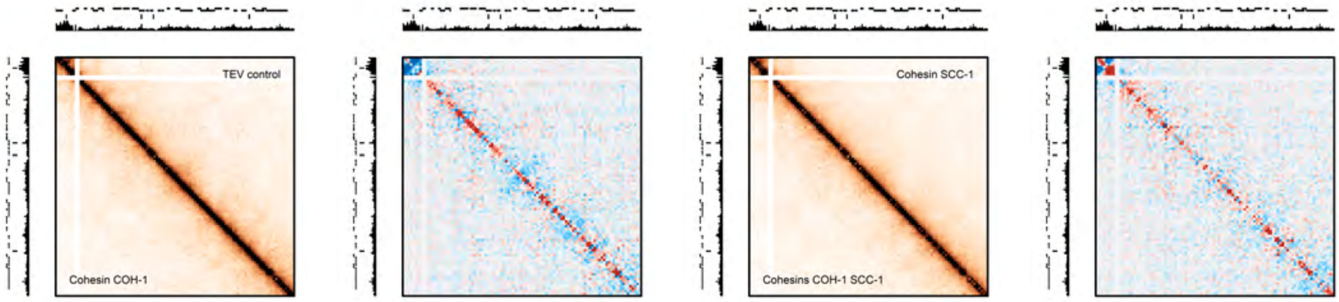

chrX:13581001-13781000

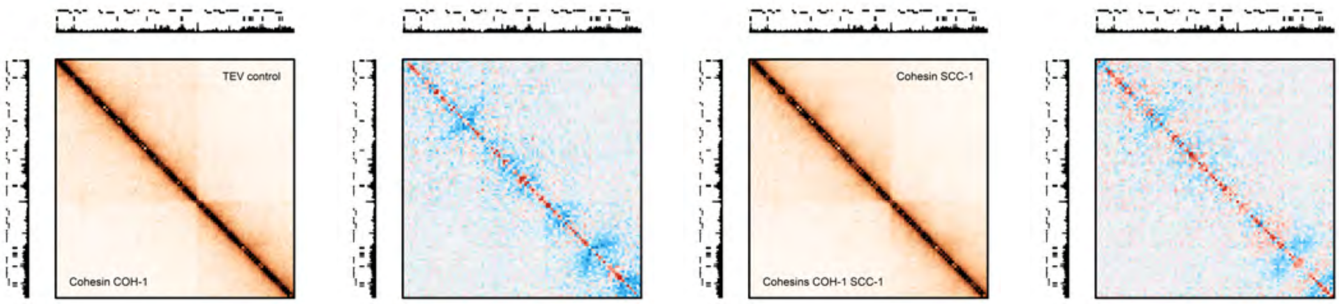

chrX:12349001-12549000

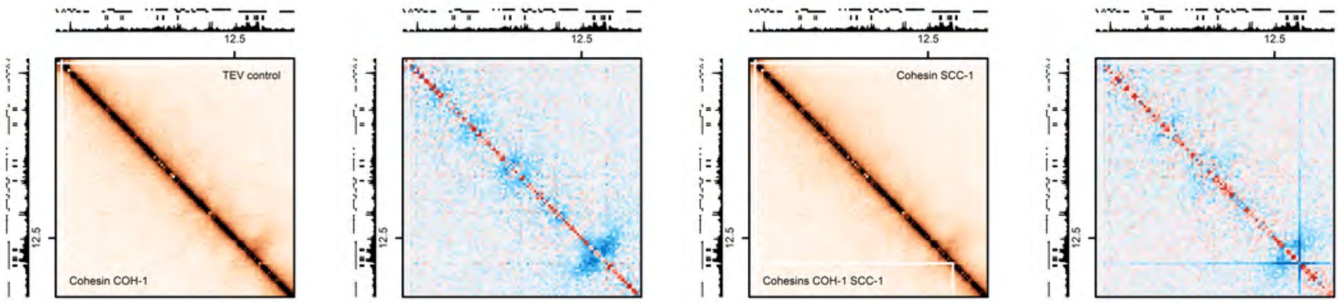

chrIII:8773001-8973000

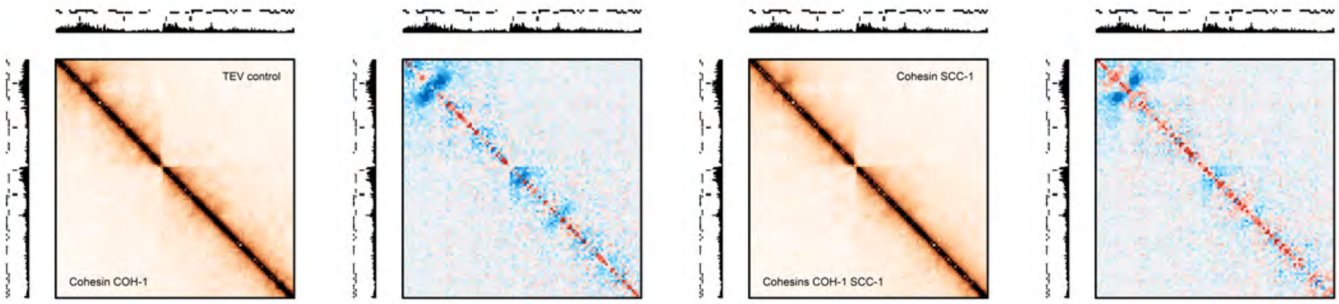

chrX:13223001-13423000

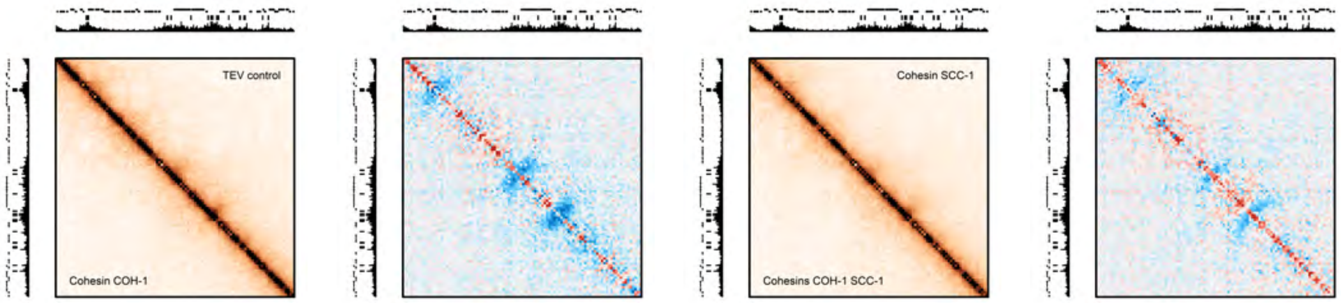

chrIV:6859001-7059000

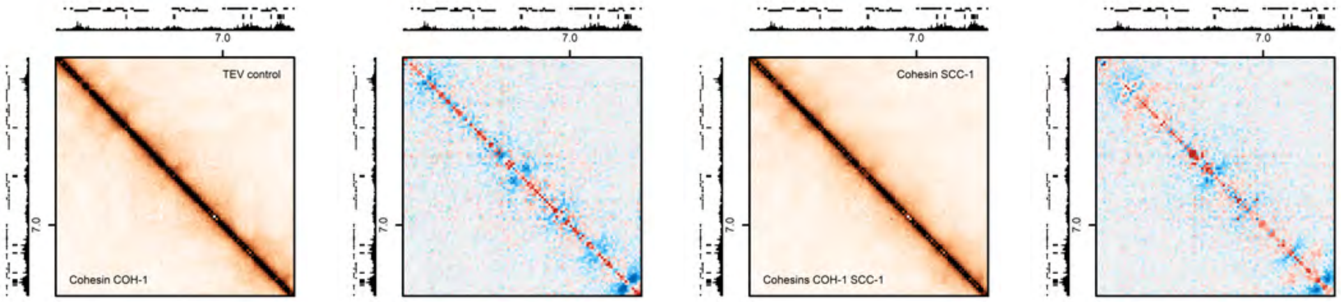

chrII:5785001-5985000

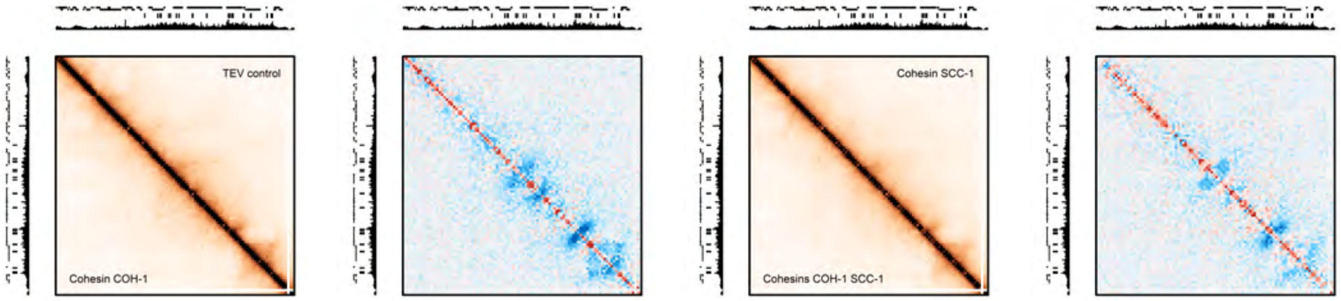

chrI:6737001-6937000

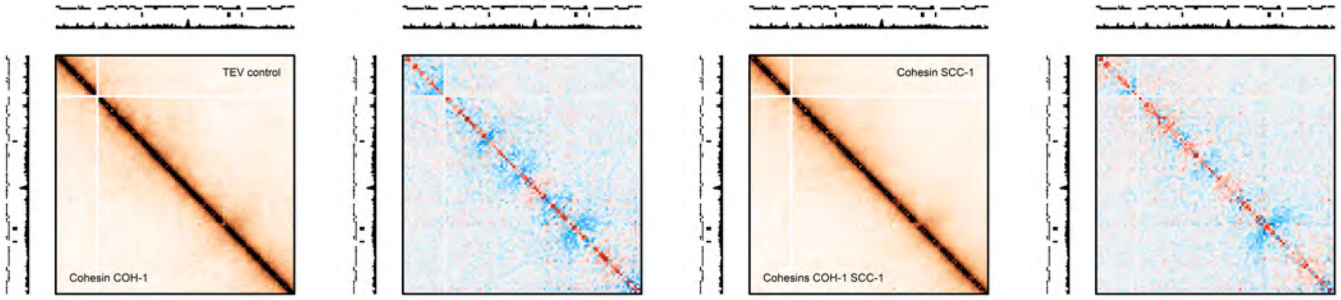

chrI:13853001-14053000

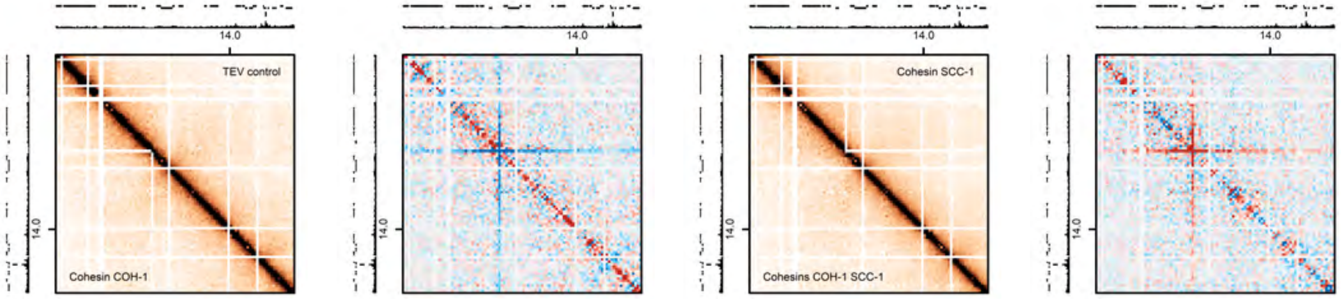

chrIII:13001001-13201000

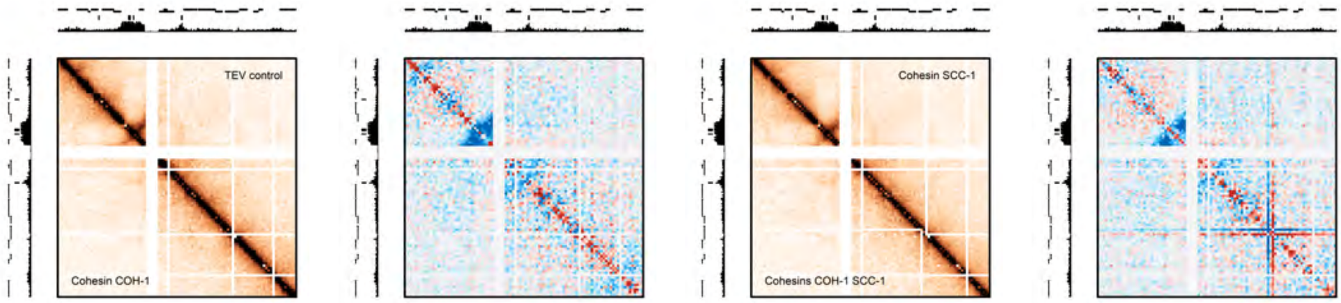

chrIV:10513001-10713000

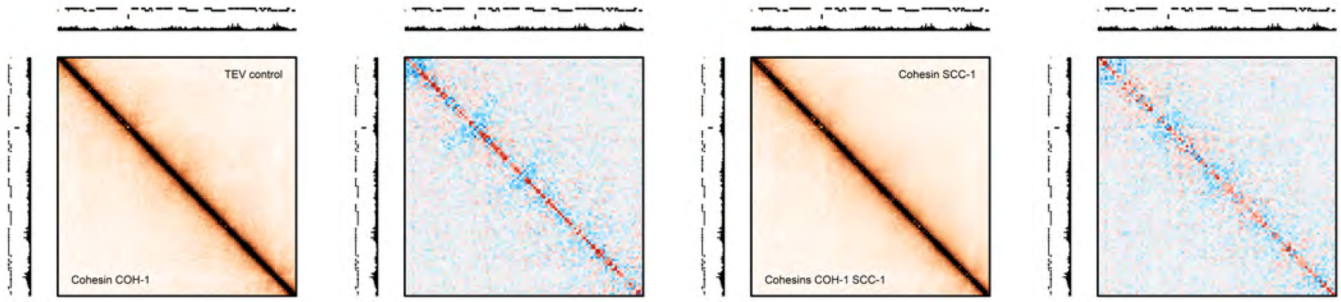

chrX:2219001-2419000

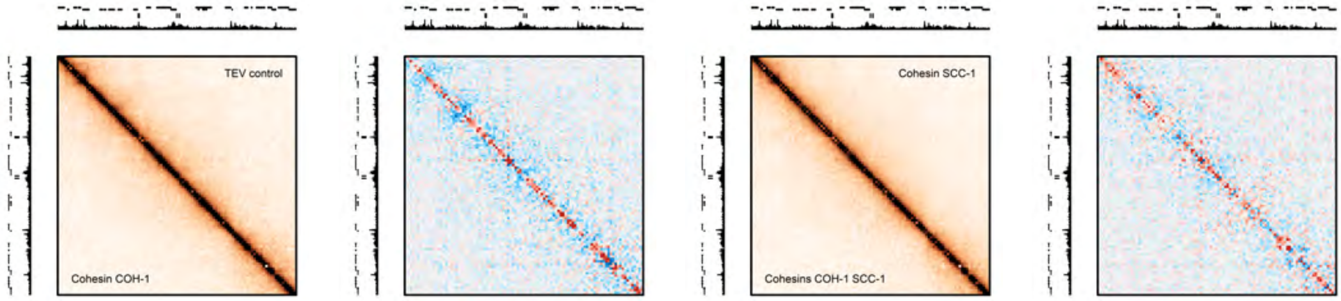

chrIV:4459001-4659000

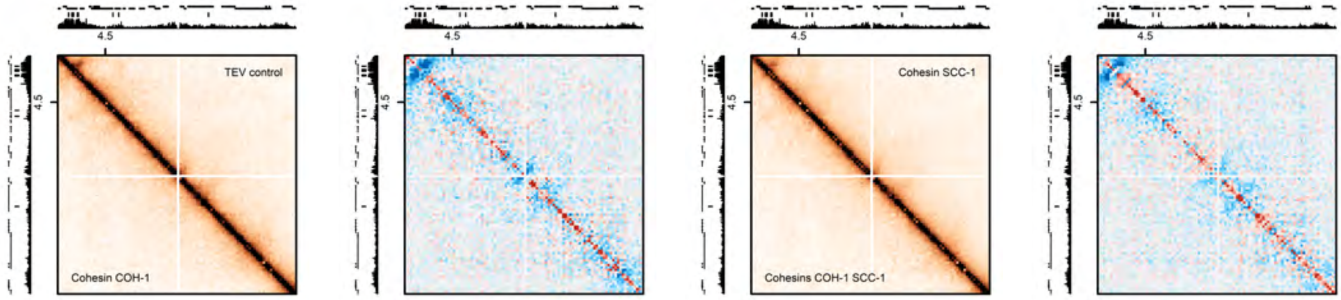

chrIII:4625001-4825000

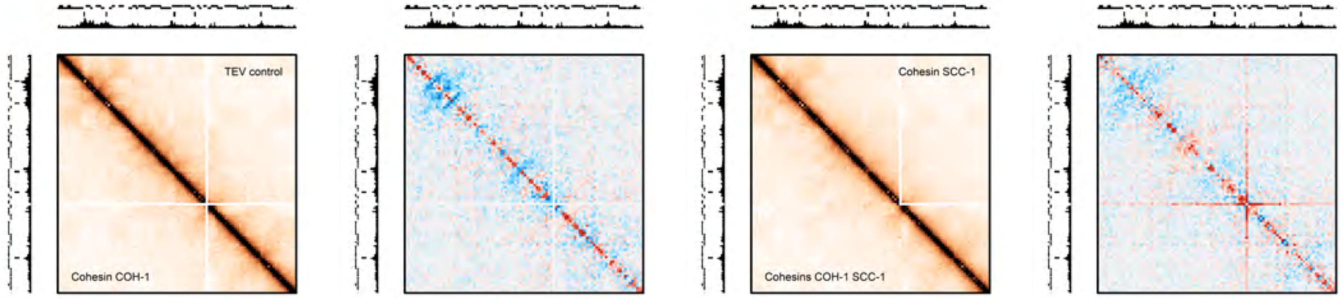

chrV:5423001-5623000

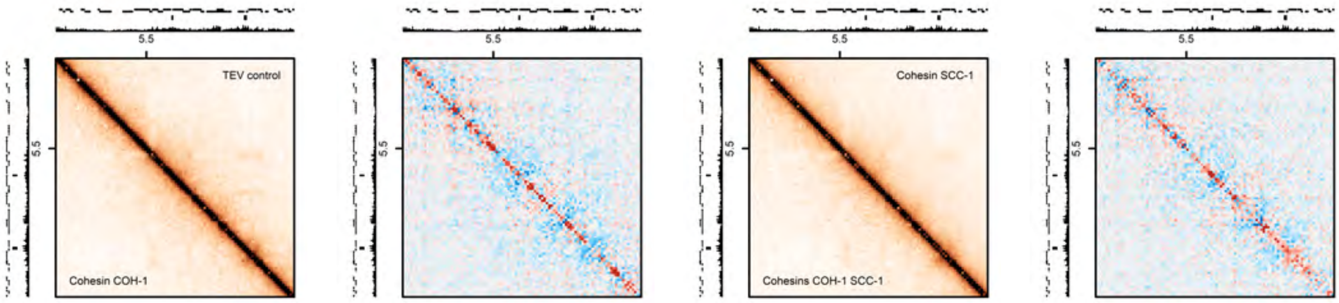

chrI:13589001-13789000

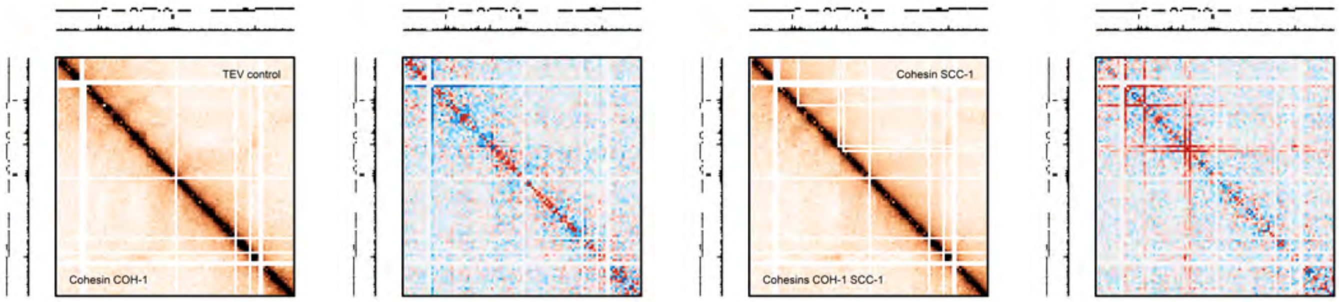

chrIII:889001-1089000

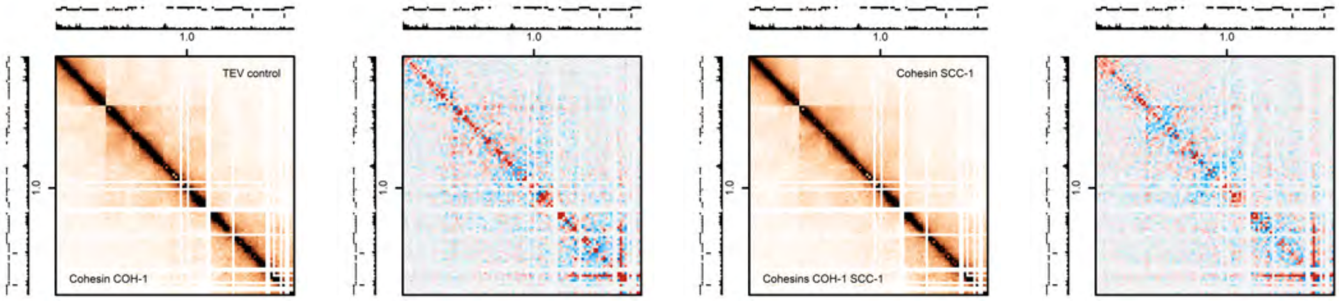

chrX:6495001-6695000

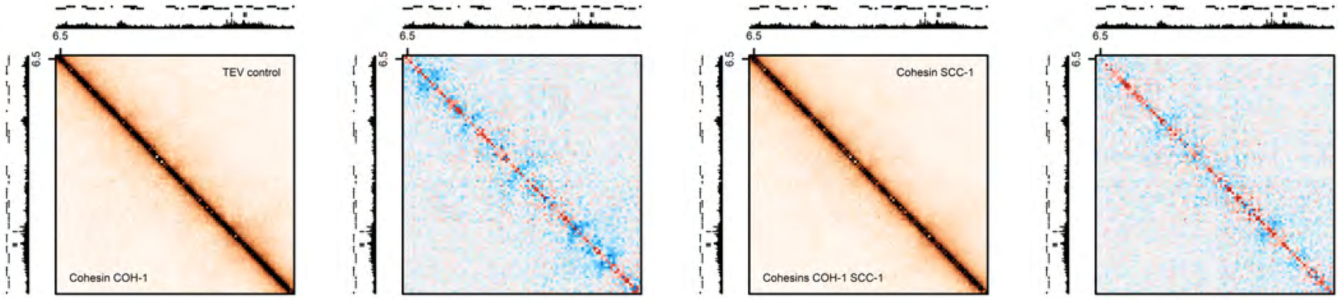

chrI:12247001-12447000

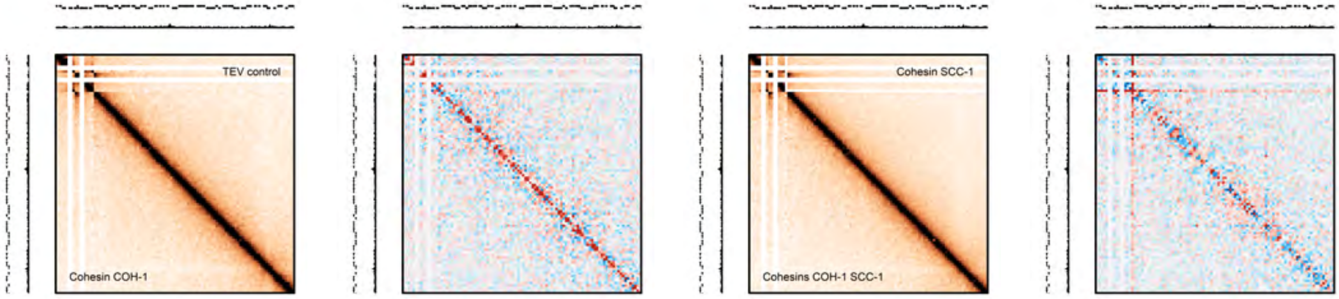

chrX:10747001-10947000

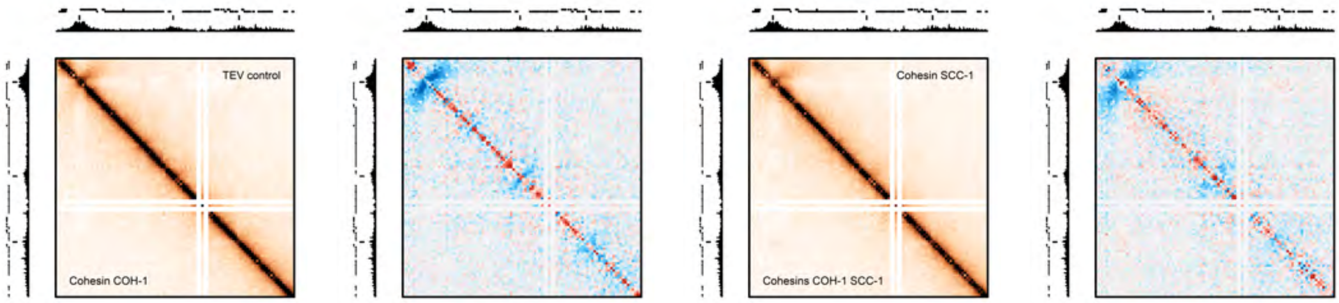

chrII:8123001-8323000

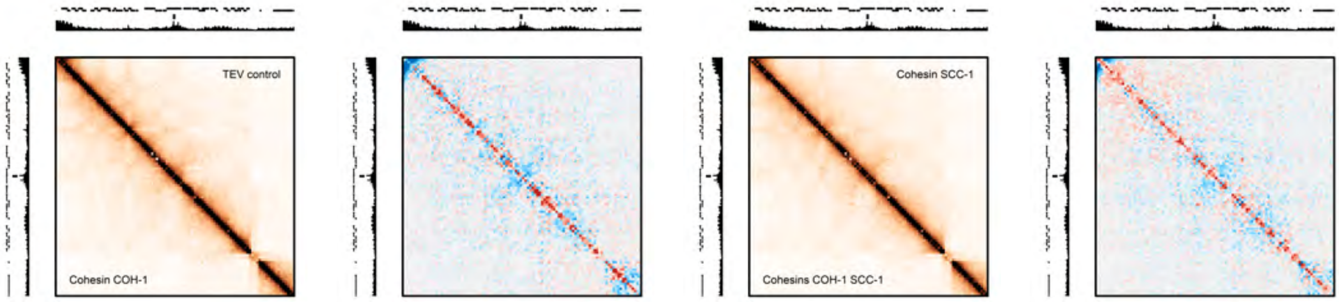

chrII:7885001-8085000

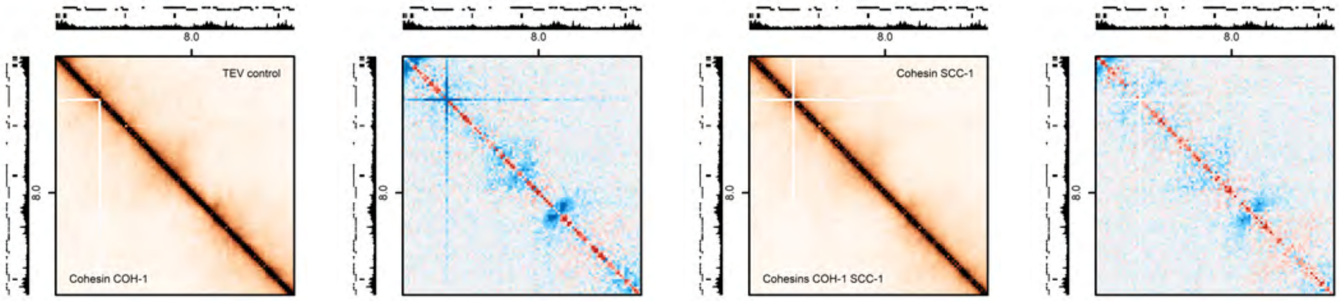

chrIV:6781001-6981000

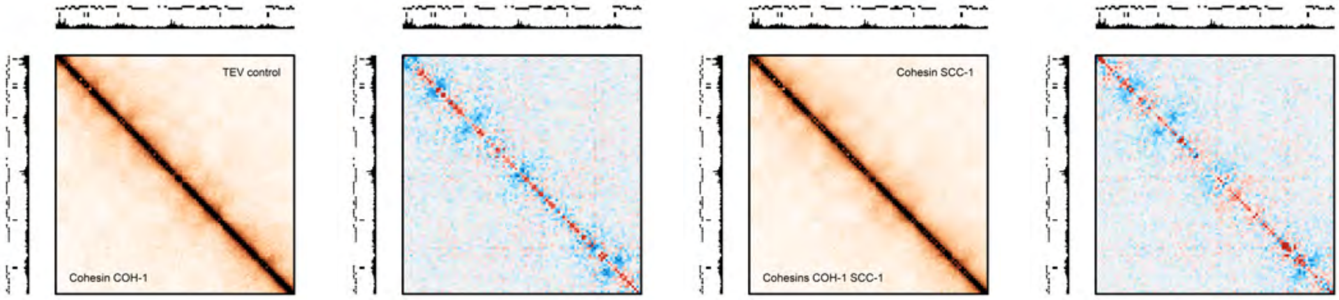

chrII:9791001-9991000

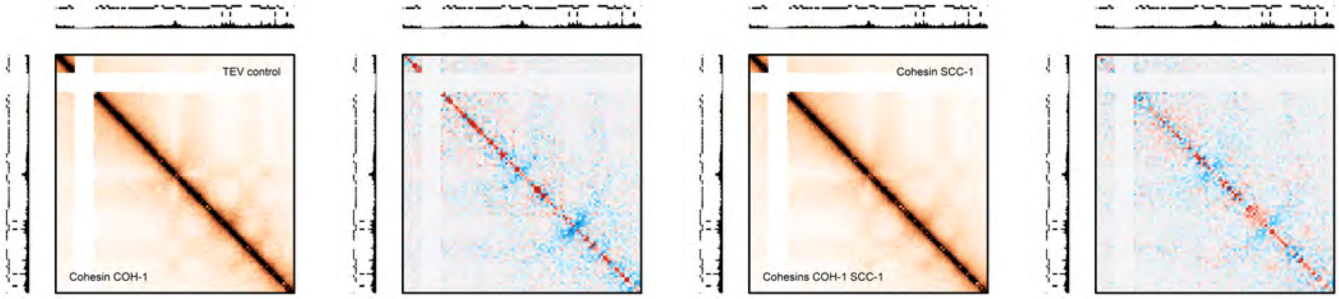

chrII:1683001-1883000

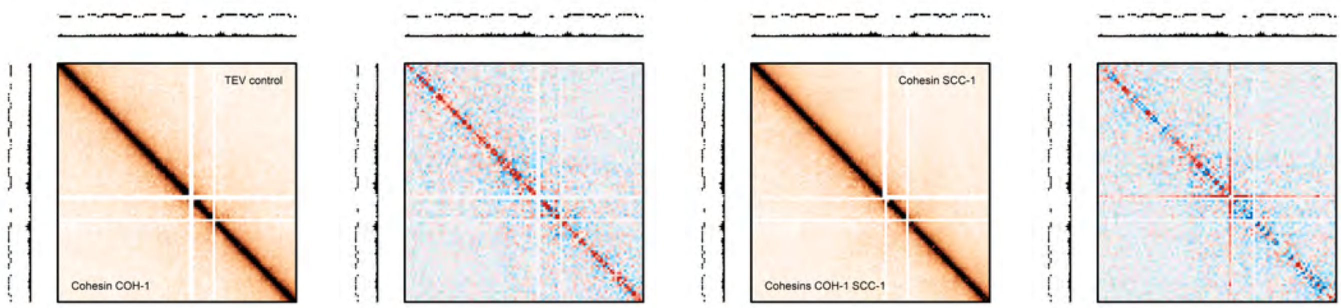

chrV:18899001-19099000

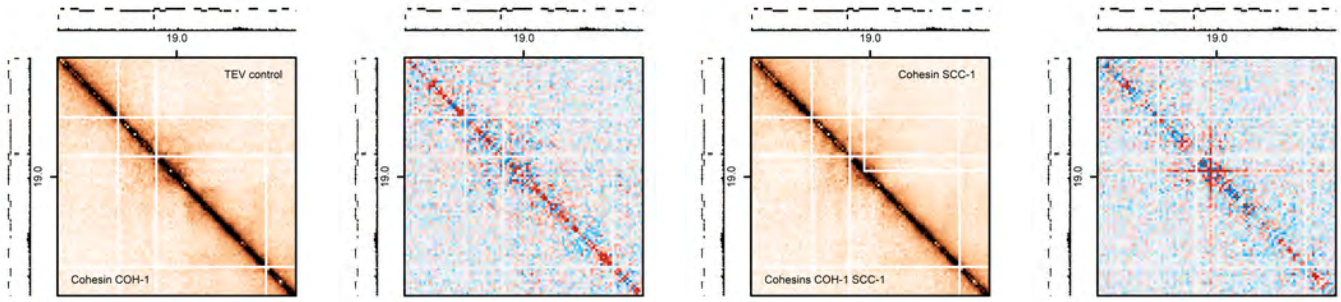

chrIII:7797001-7997000

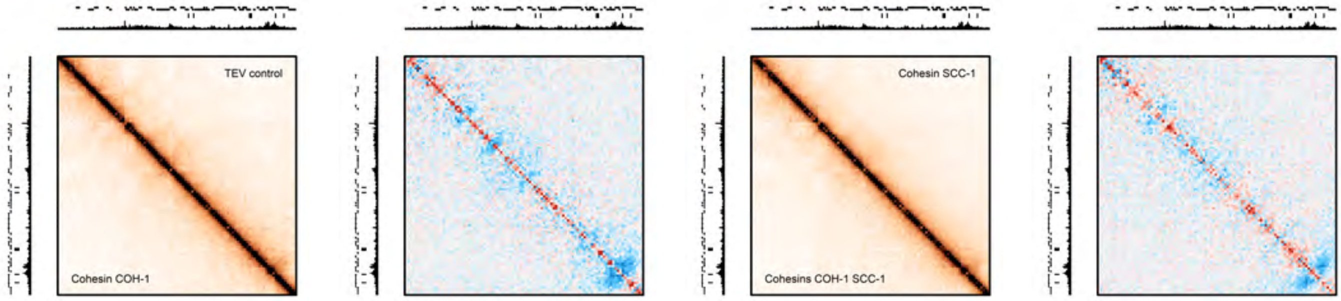

chrII:11837001-12037000

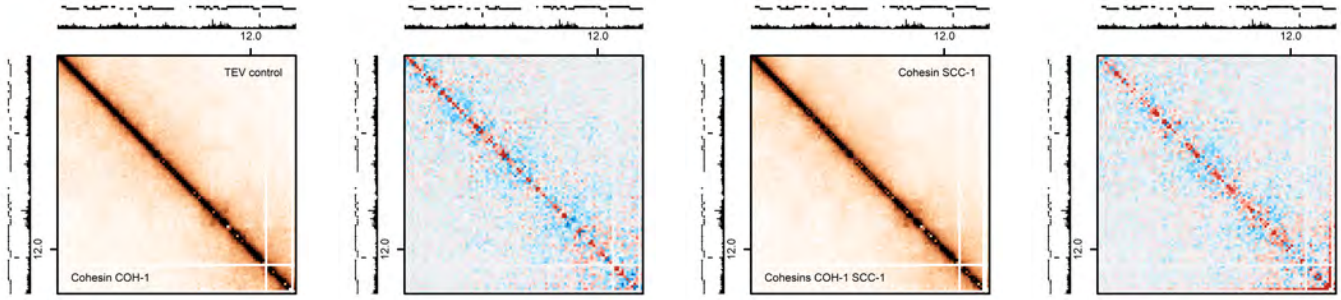

chrV:6367001-6567000

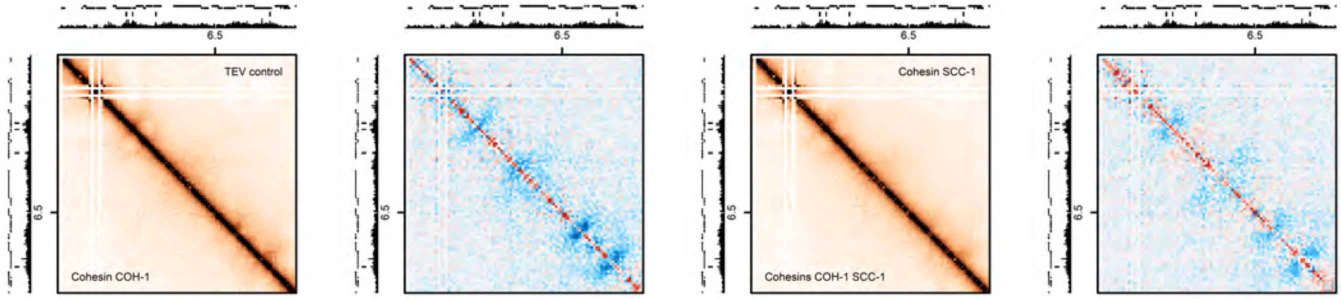

chrIII:10805001-11005000

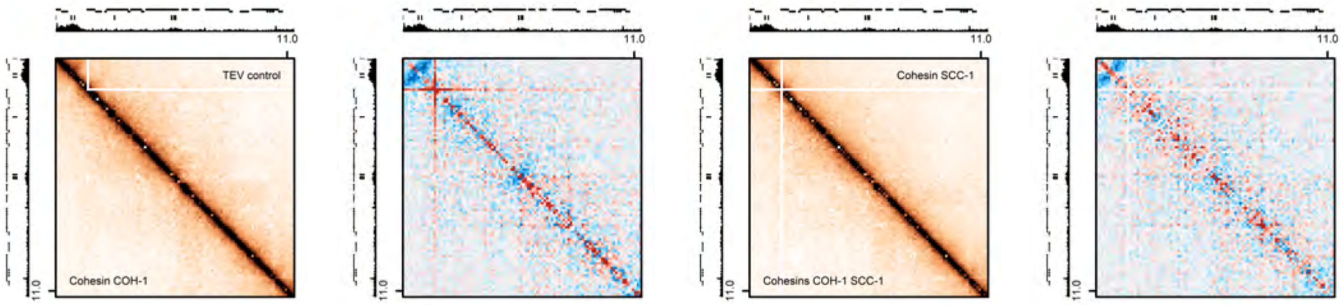

chrV:17345001-17545000

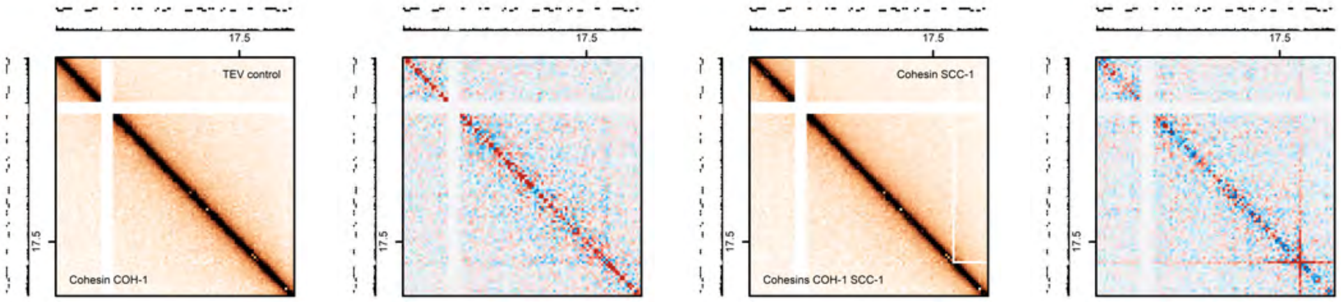

chrV:9411001-9611000

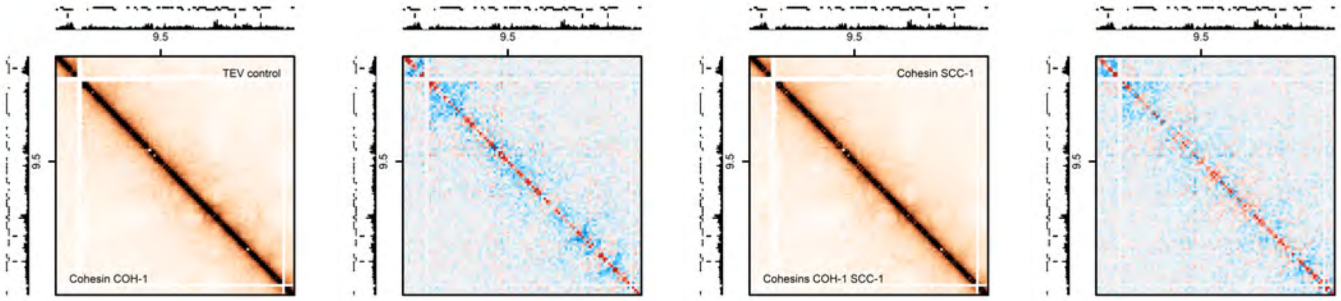

chrIII:8475001-8675000

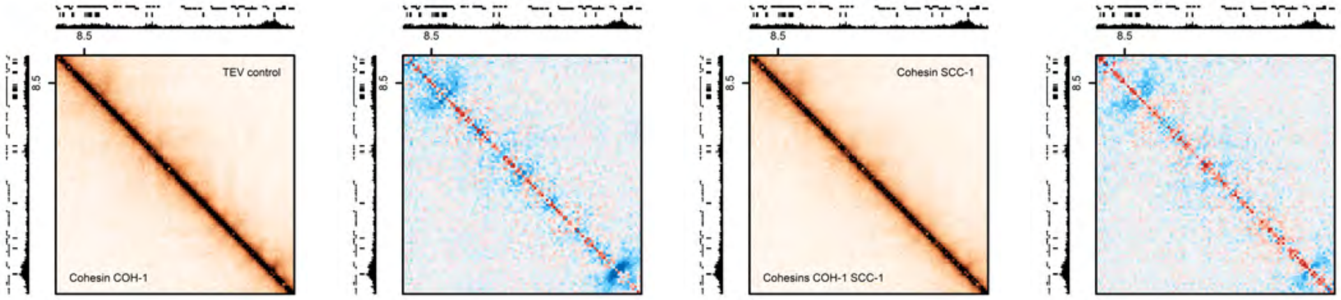

chrV:6599001-6799000

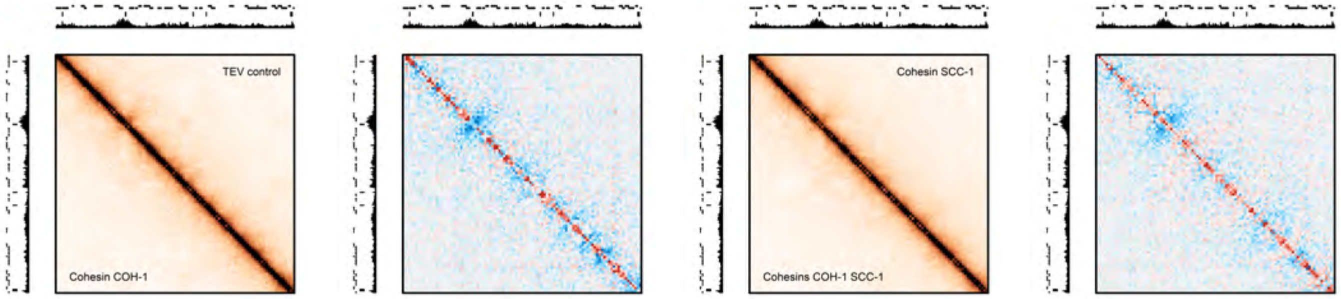

chrV:13369001-13569000

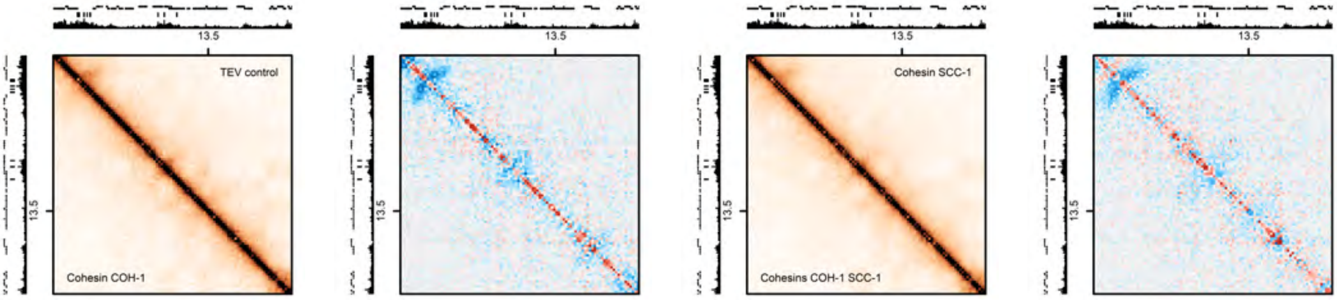

chrII:10943001-11143000

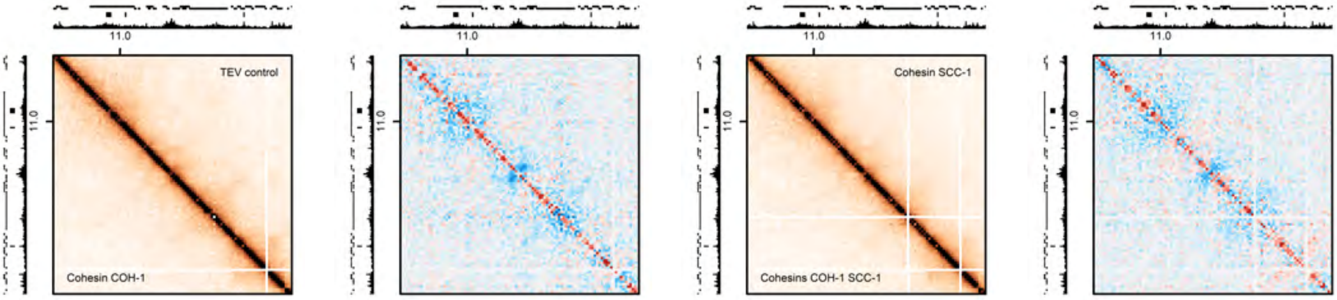

chrIII:2967001-3167000

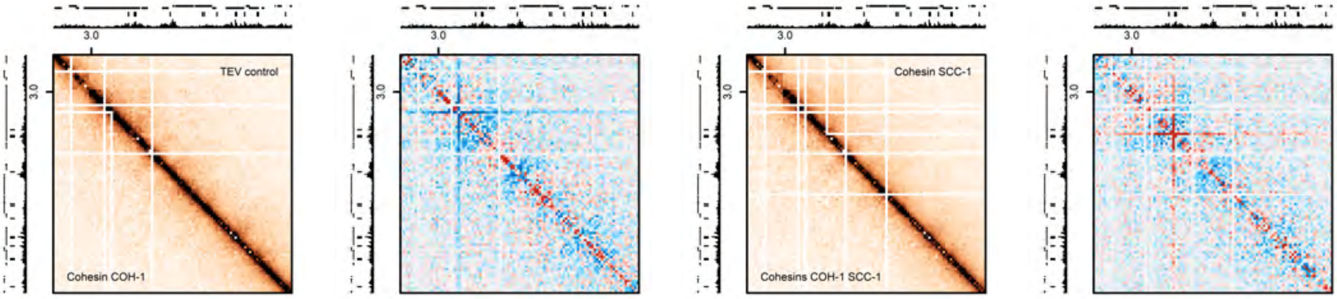

chrX:13777001-13977000

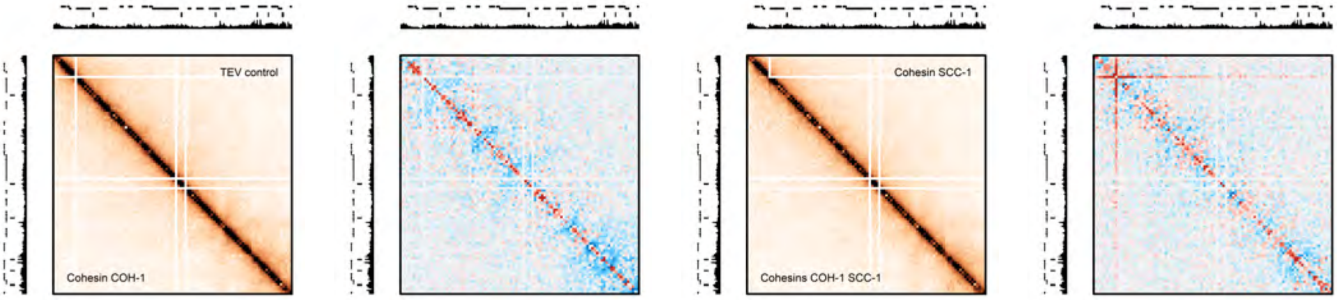

chrI:12871001-13071000

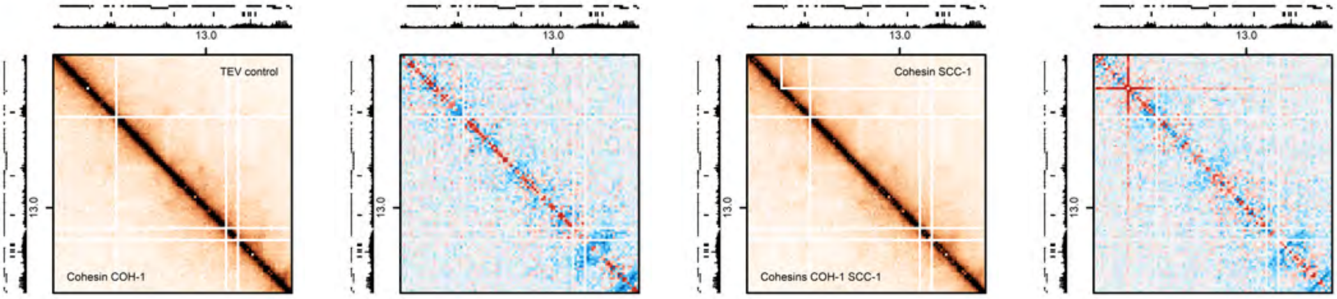

chrIII:5659001-5859000

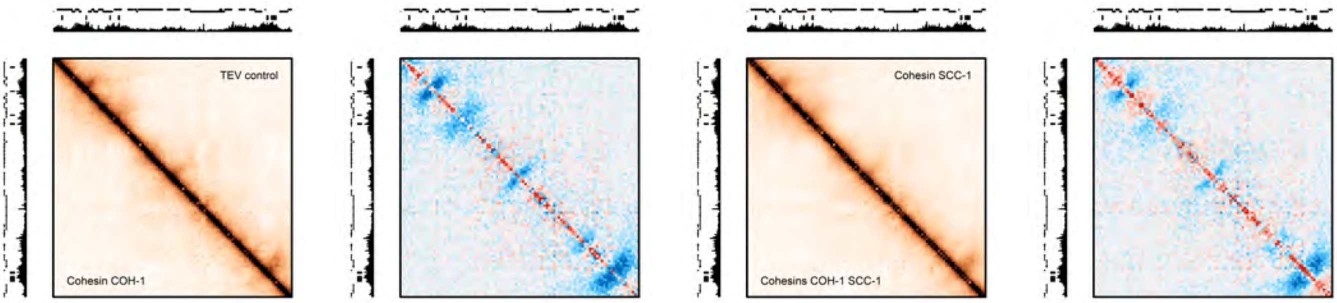

chrII:8485001-8685000

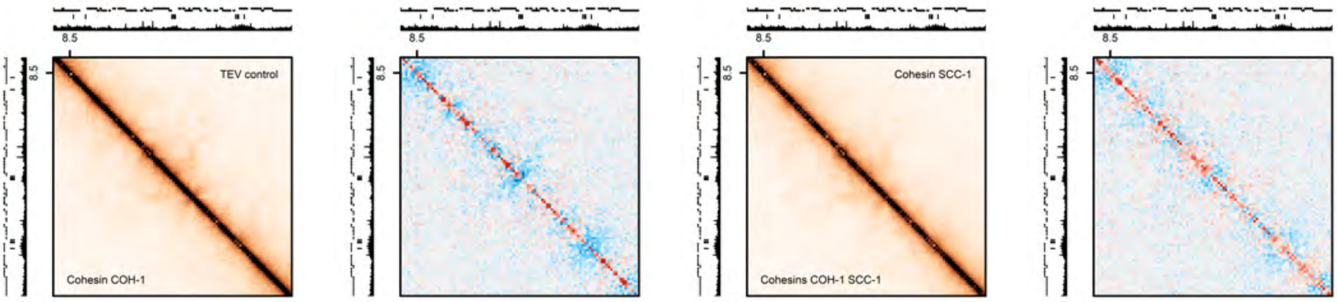

chrIV:11767001-11967000

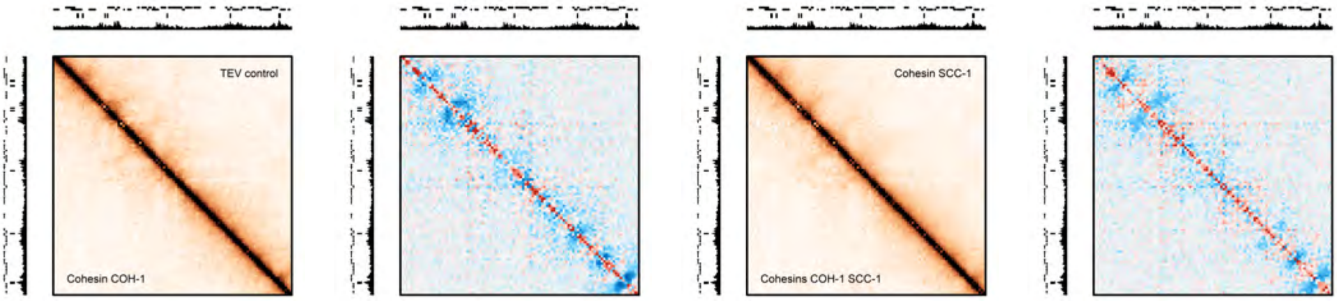

chrIII:3333001-3533000

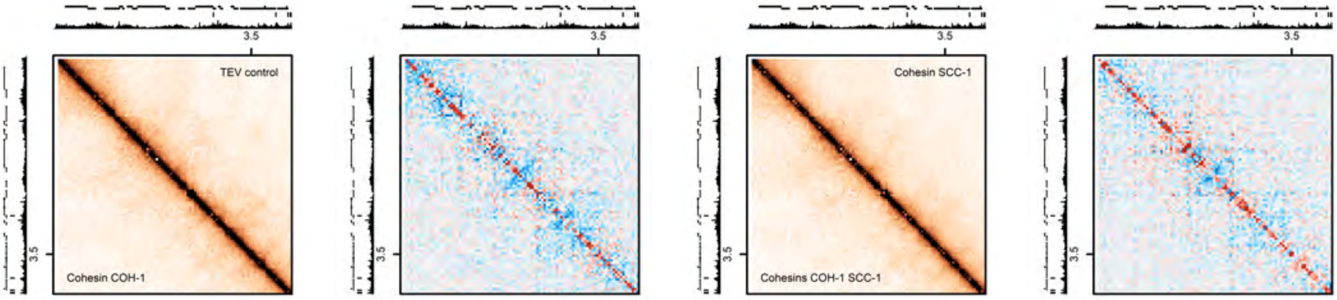

chrX:919001-1119000

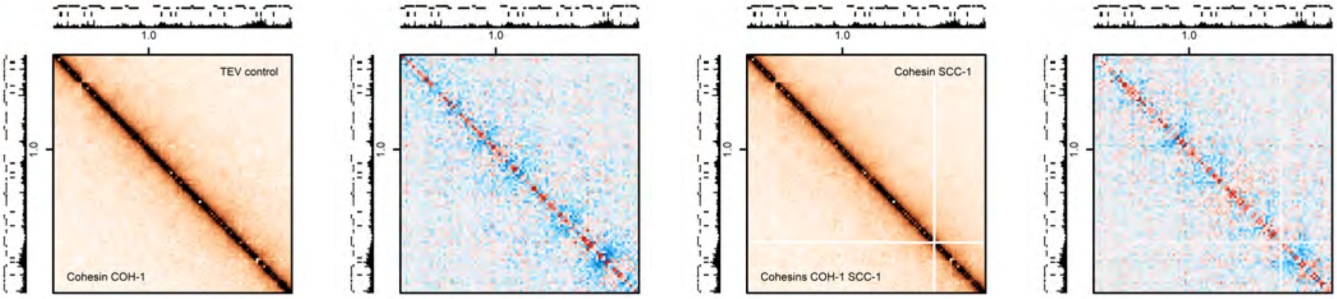

chrV:19317001-19517000

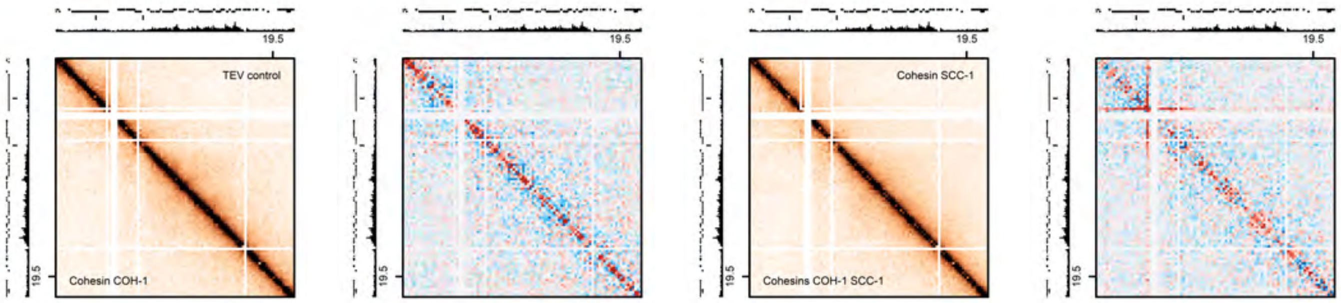

chrV:5171001-5371000

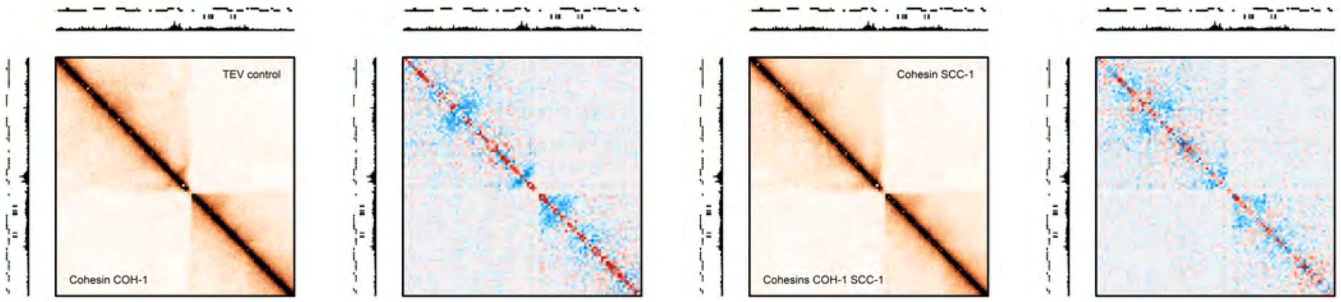

chrV:6007001-6207000

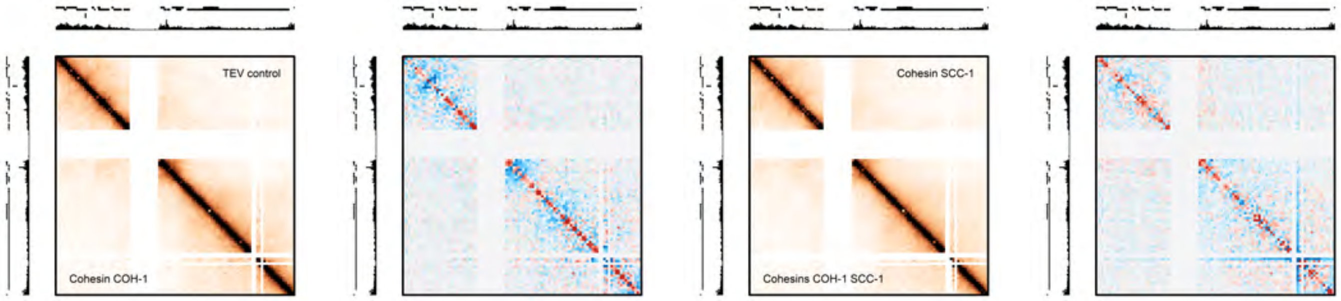

chrV:7631001-7831000

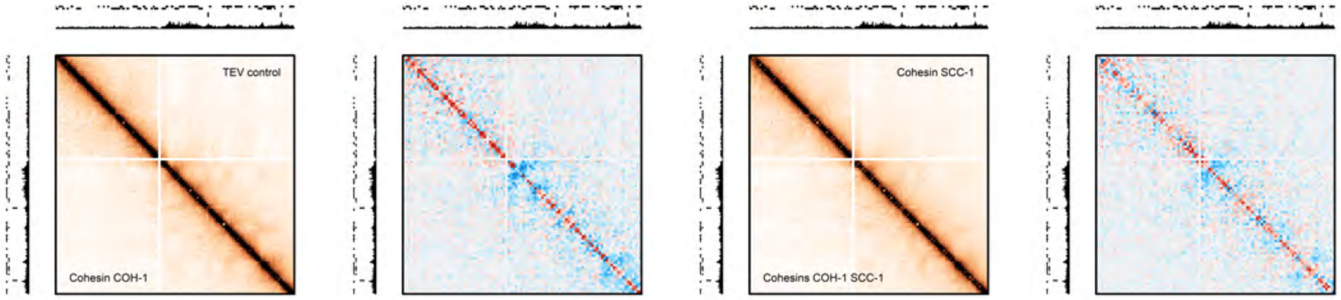

chrX:1403001-1603000

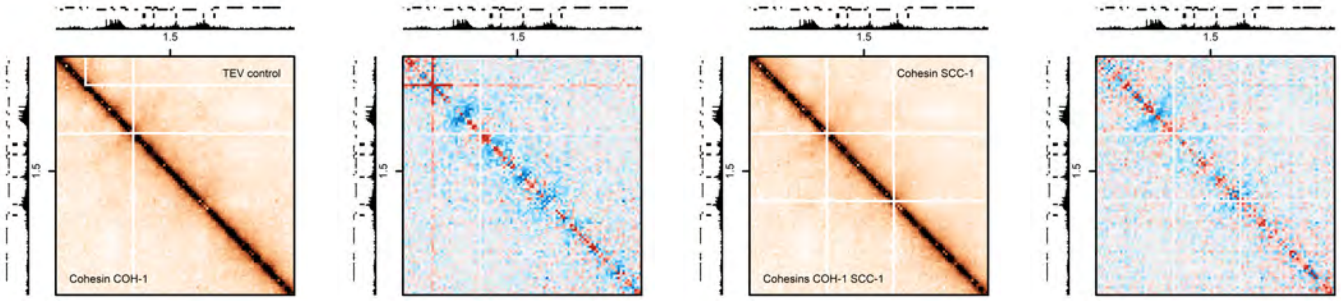

chrIV:12081001-12281000

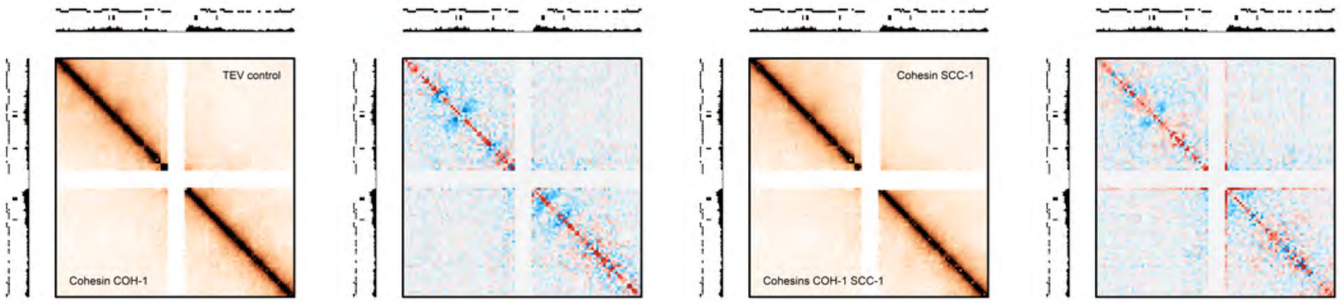

chrII:5279001-5479000

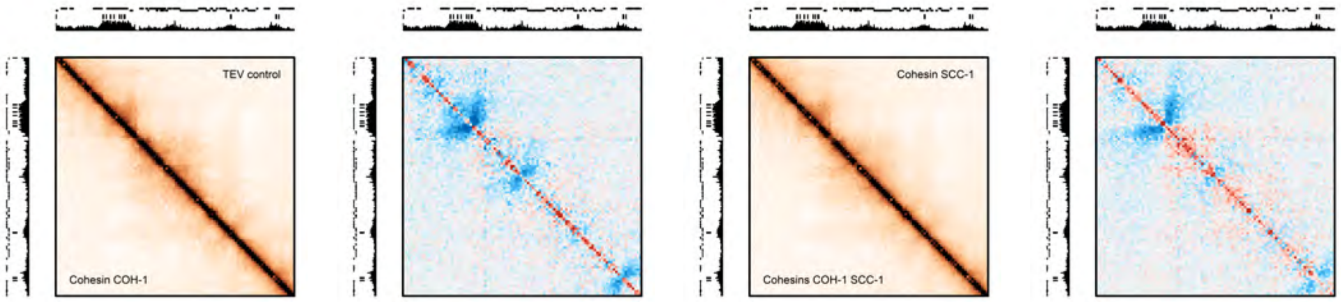

chrI:14873001-15073000

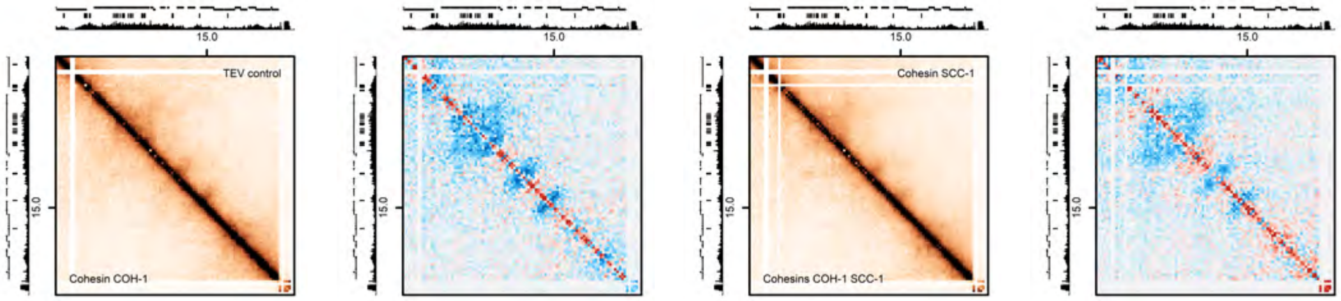

chrX:11985001-12185000

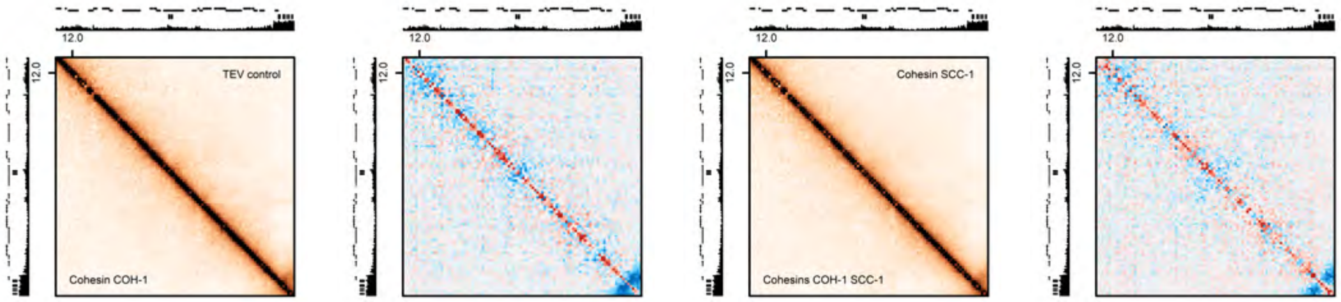

chrII:14943001-15143000

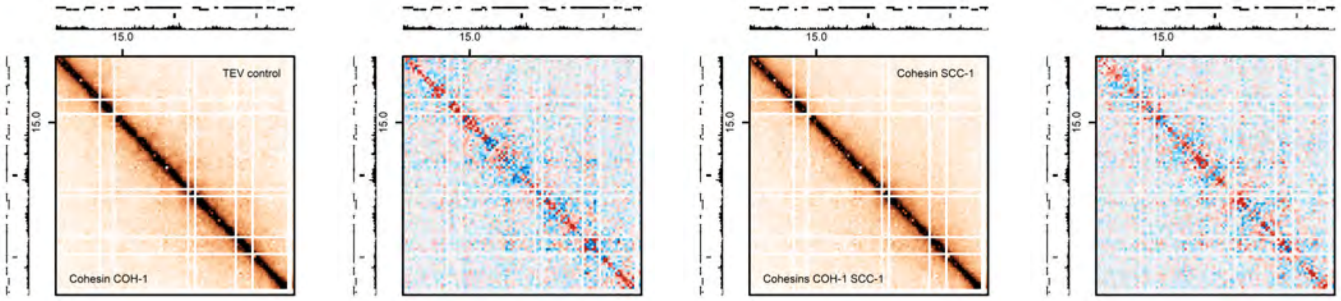

chrIV:16195001-16395000

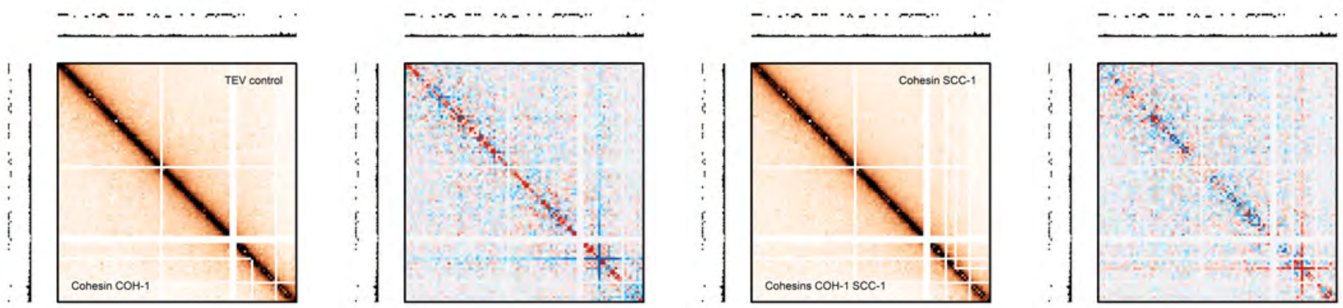

chrX:2947001-3147000

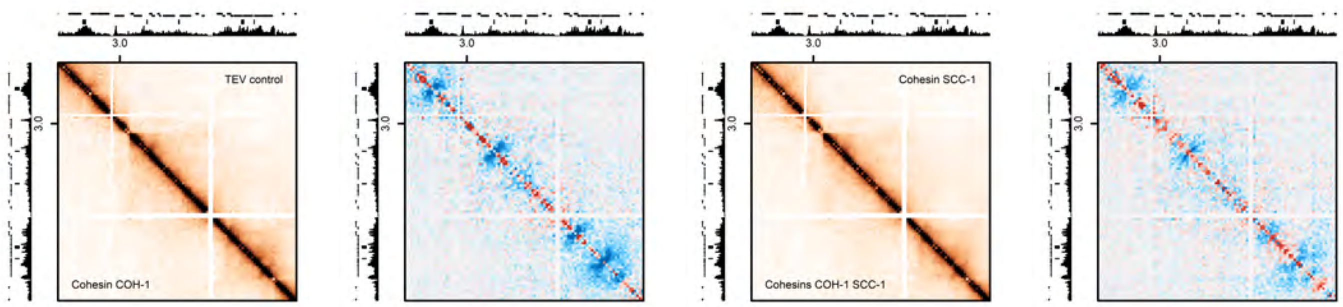

chrIV:9961001-10161000

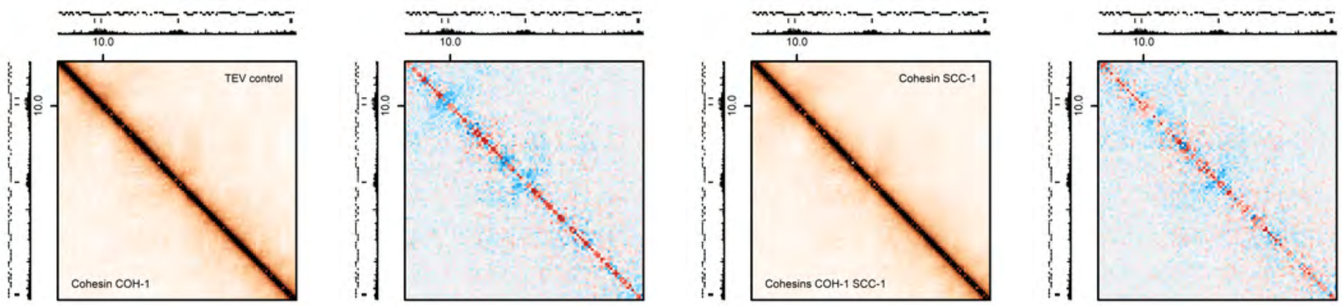

chrII:7129001-7329000

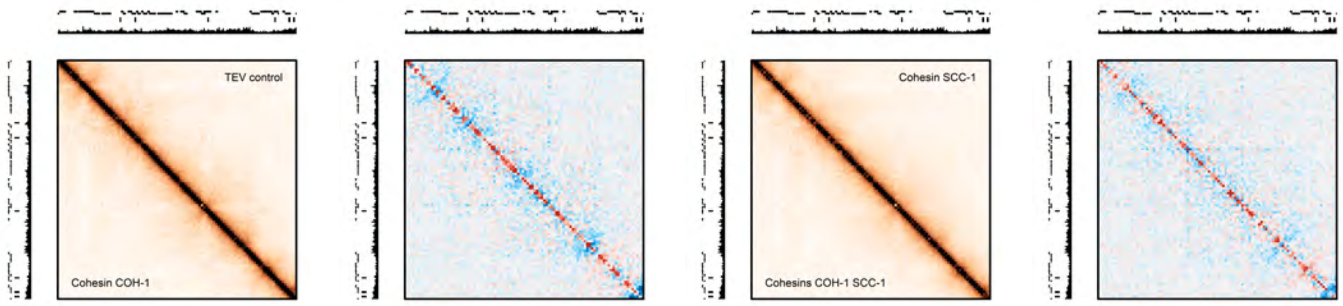

chrI:7027001-7227000

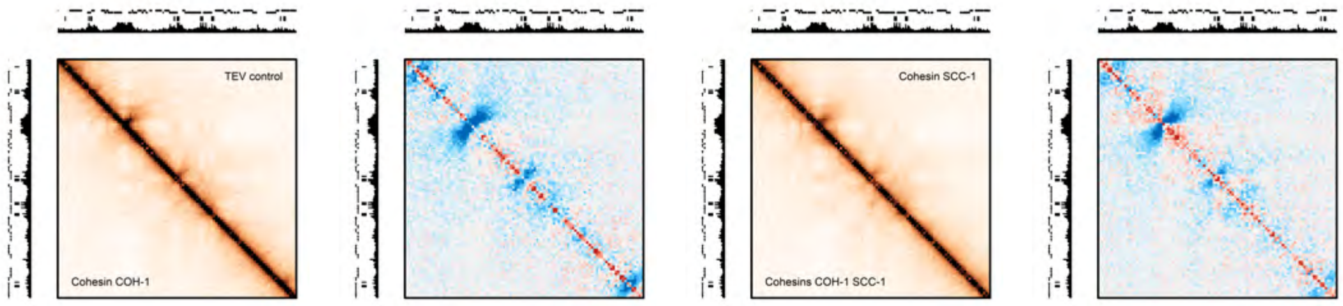

chrIII:6797001-6997000

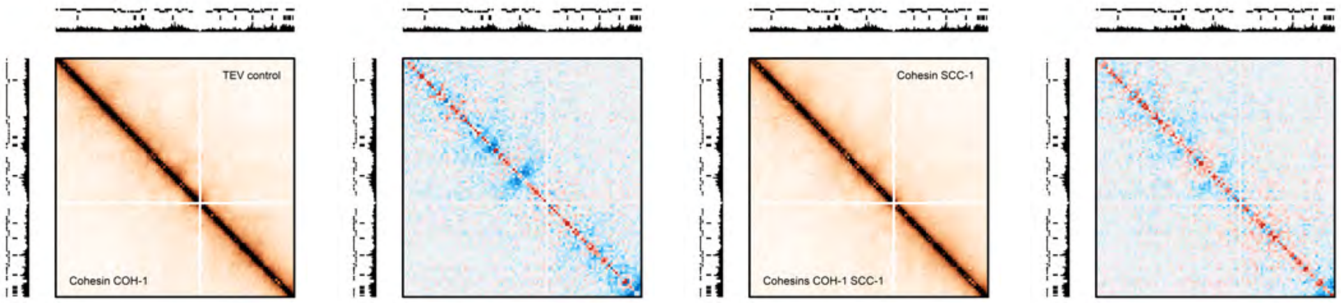

chrI:9445001-9645000

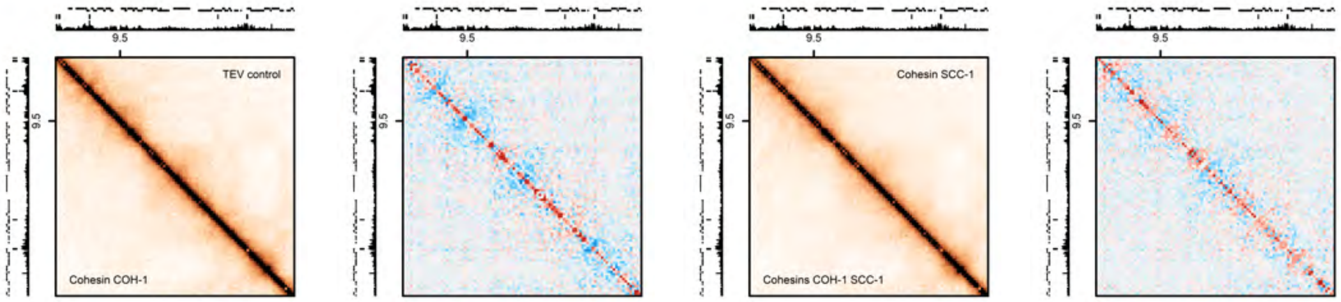

chrX:7765001-7965000

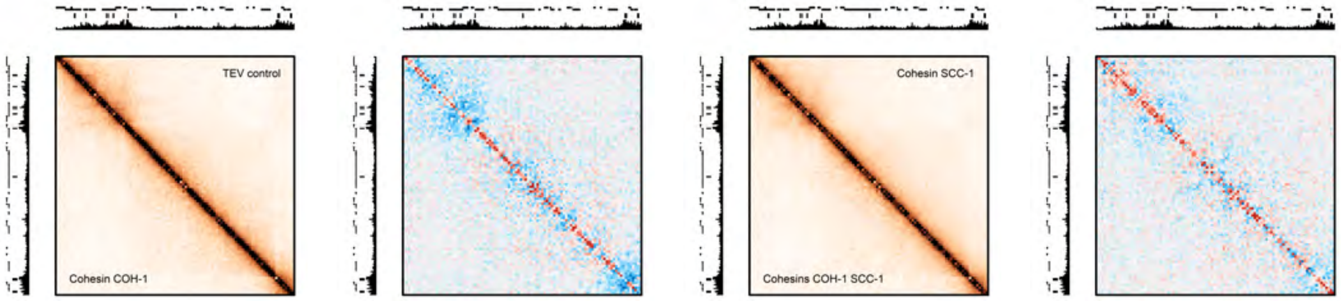

chrV:9753001-9953000

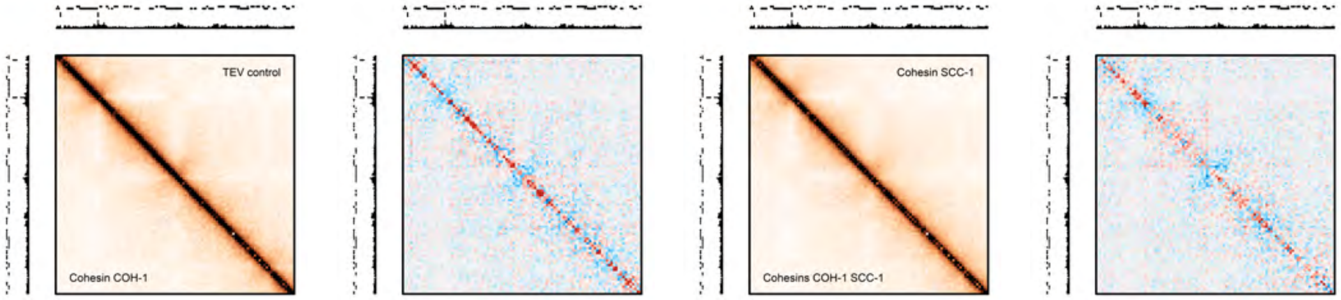

chrV:17223001-17423000

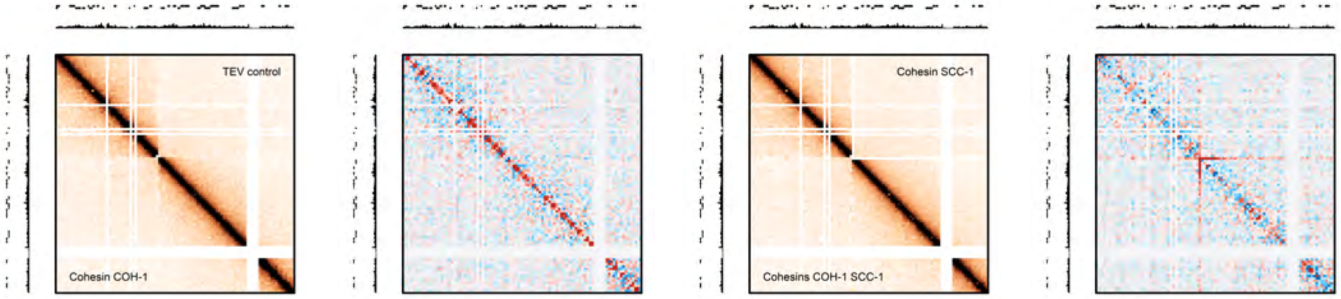

chrX:17229001-17429000

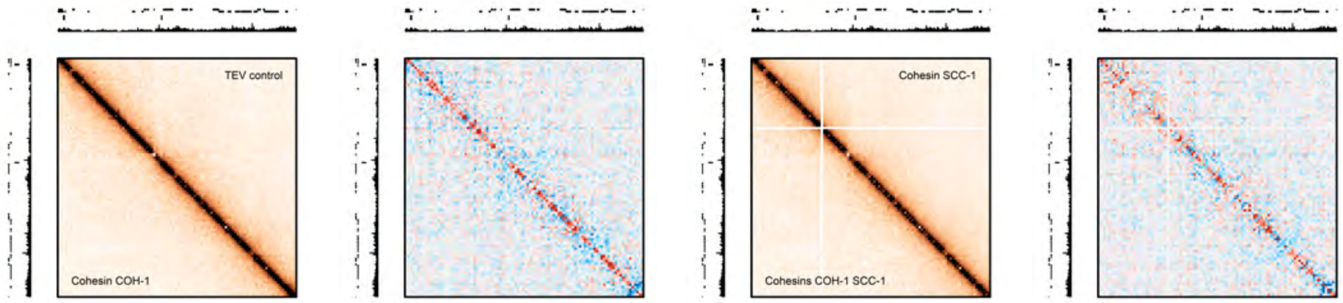

chrII:11783001-11983000

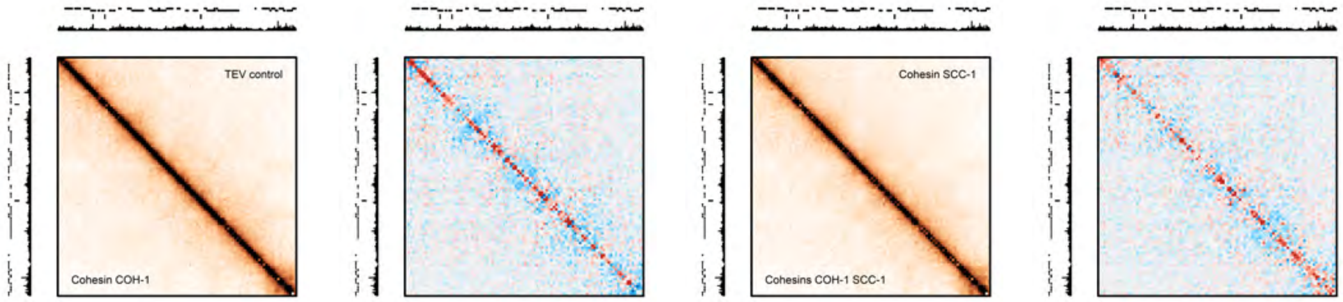

chrX:1295001-1495000

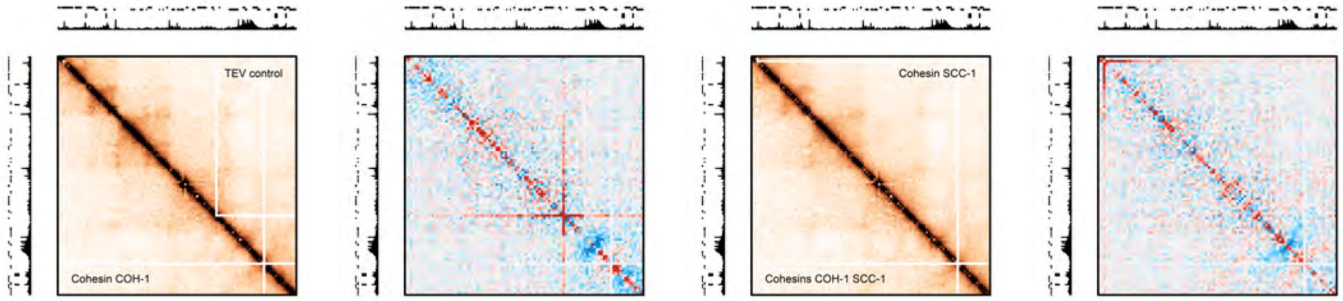

chrX:10051001-10251000

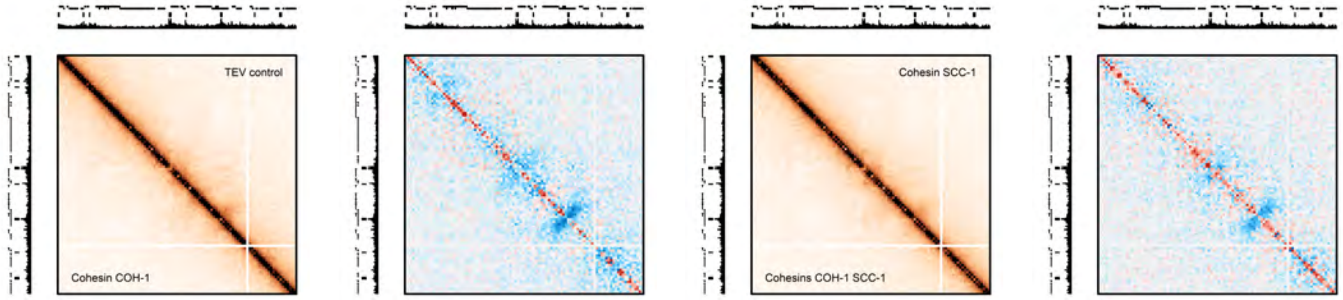

chrI:2083001-2283000

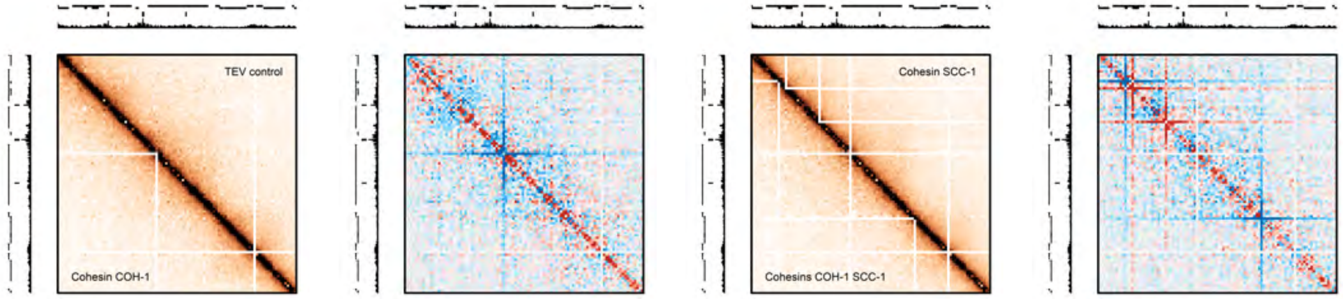

chrV:3723001-3923000

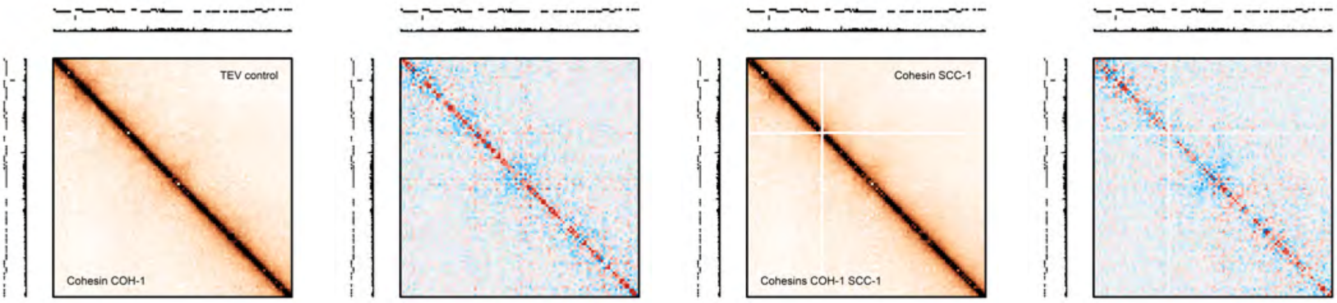

chrX:16253001-16453000

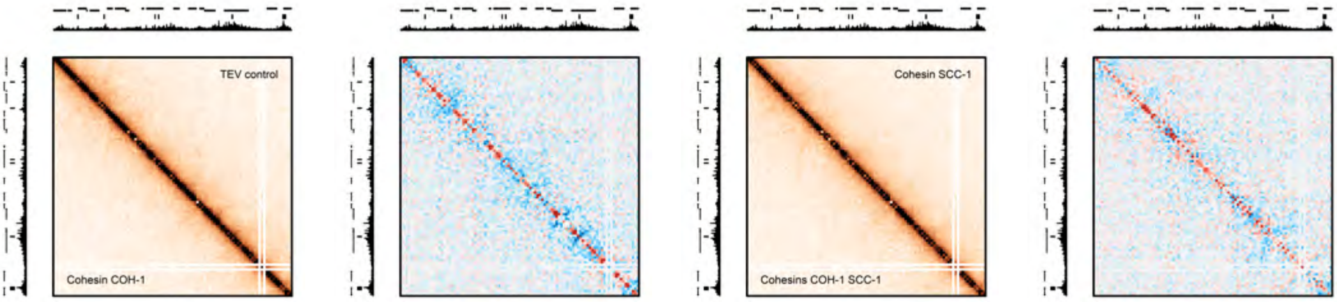

chrIII:7107001-7307000

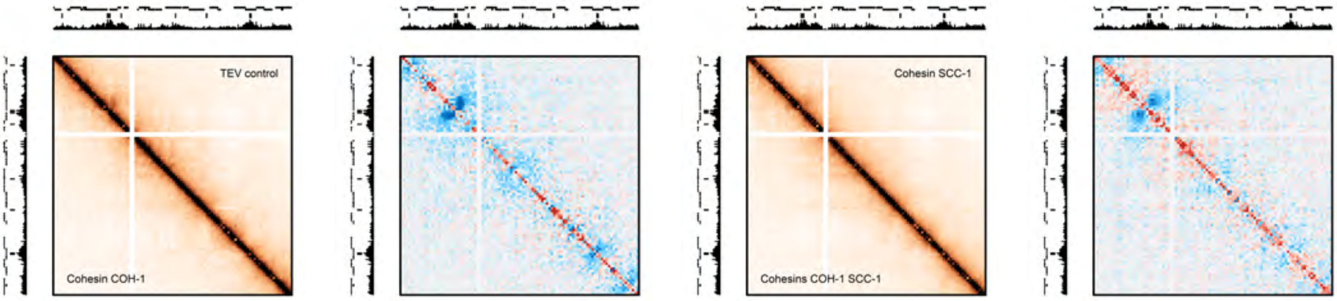

chrX:3123001-3323000

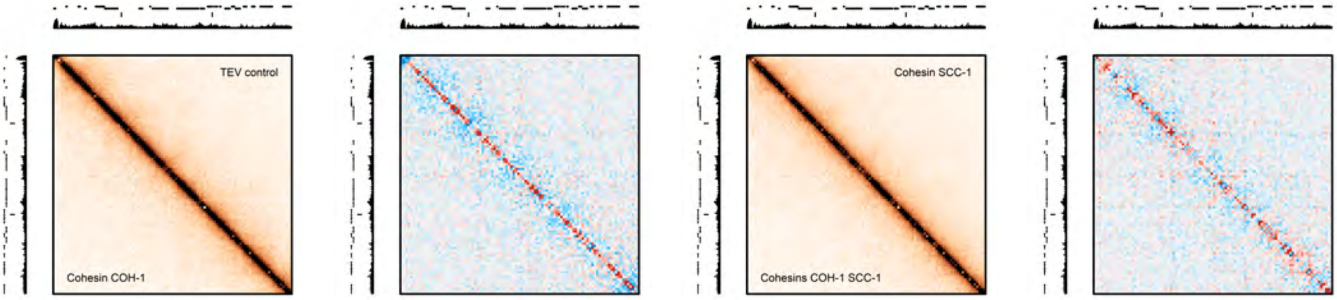

chrX:14879001-15079000

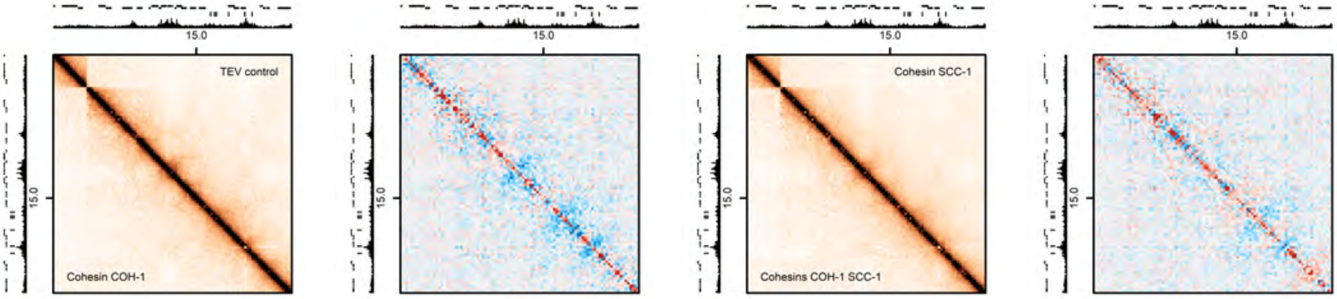

chrX:7033001-7233000

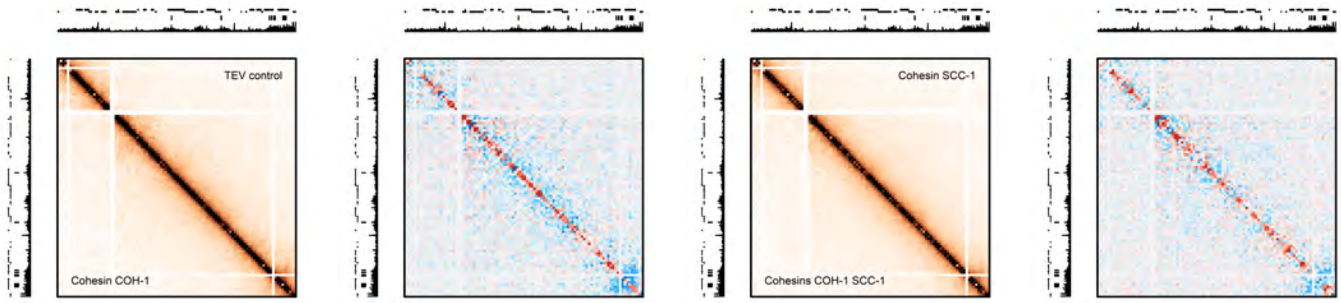

chrI:4553001-4753000

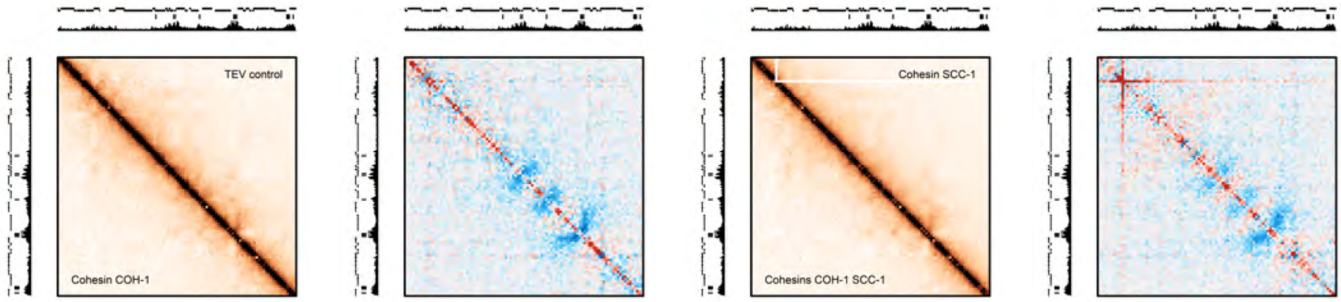

chrX:13615001-13815000

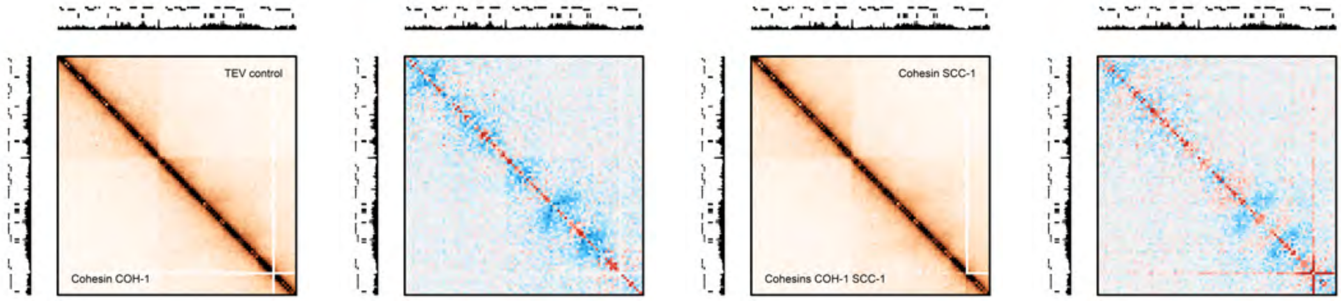

chrX:5595001-5795000

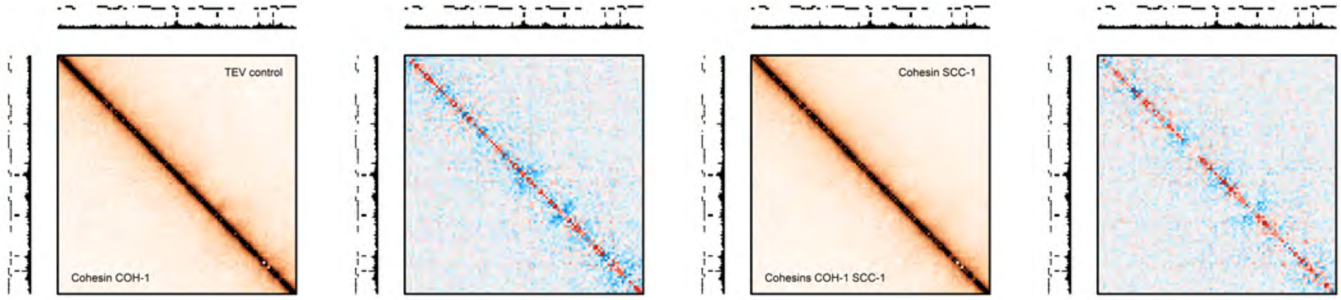

chrI:1773001-1973000

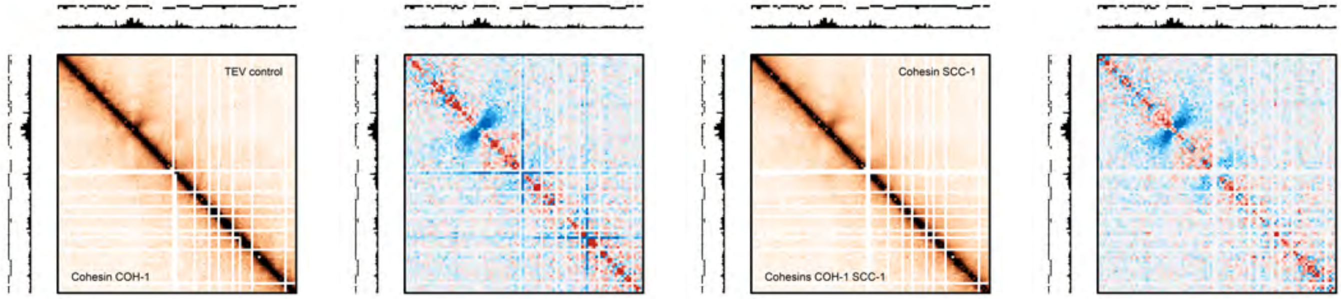

chrII:10451001-10651000

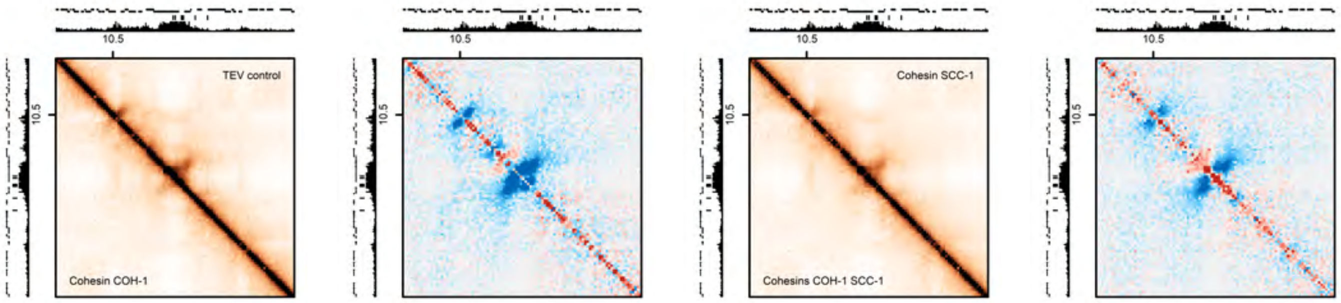

chrI:6983001-7183000

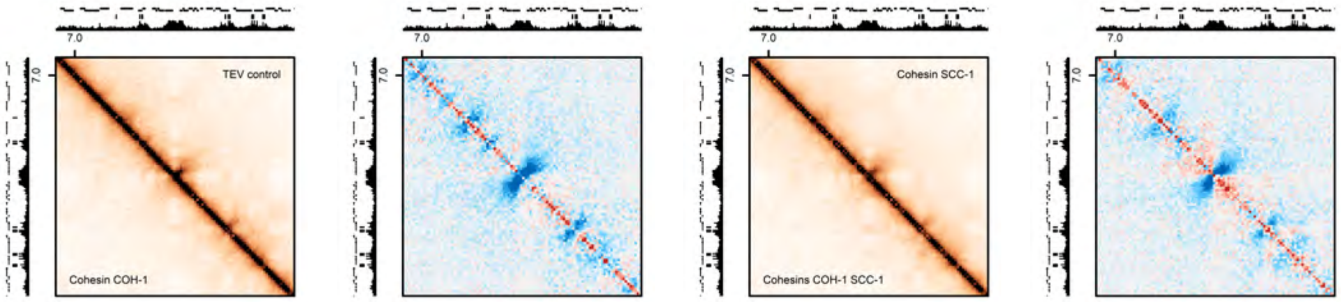

chrV:14035001-14235000

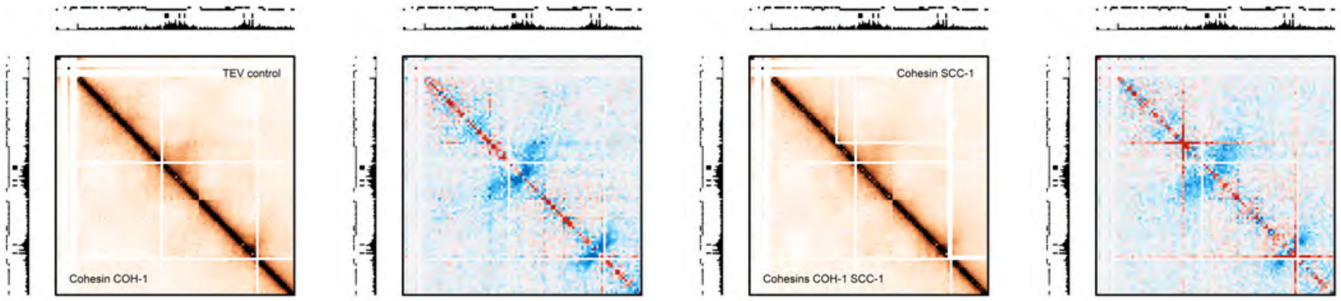

chrIII:5739001-5939000

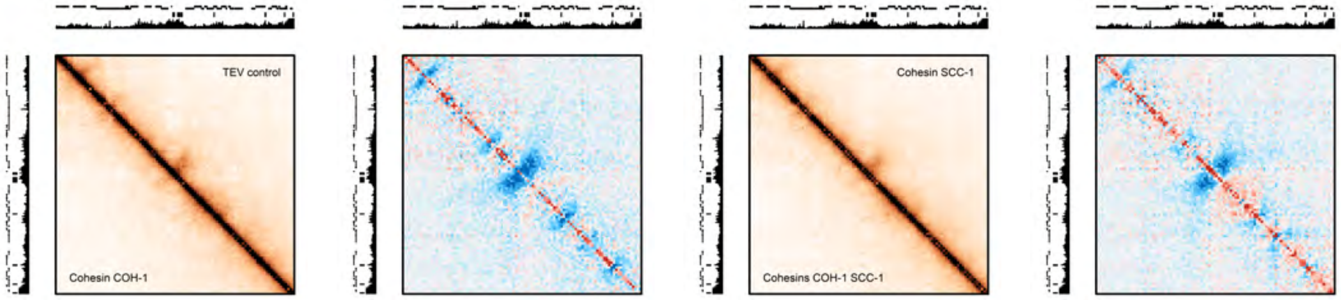

chrIII:6733001-6933000

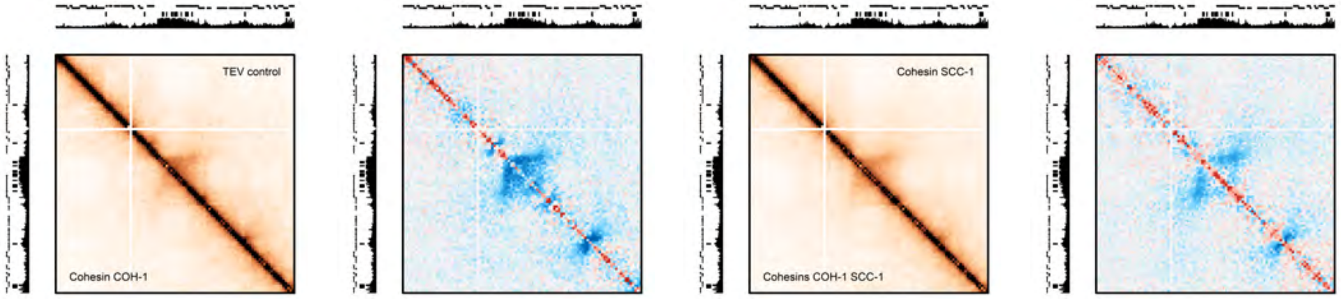

chrX:4043001-4243000

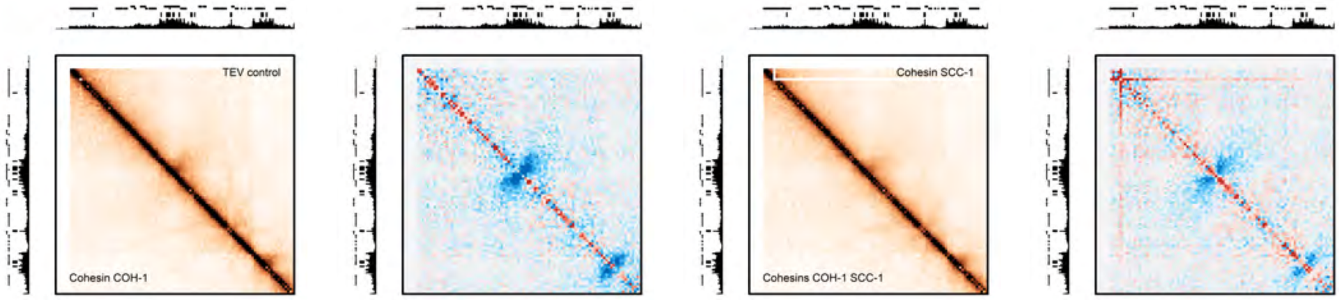

chrI:9013001-9213000

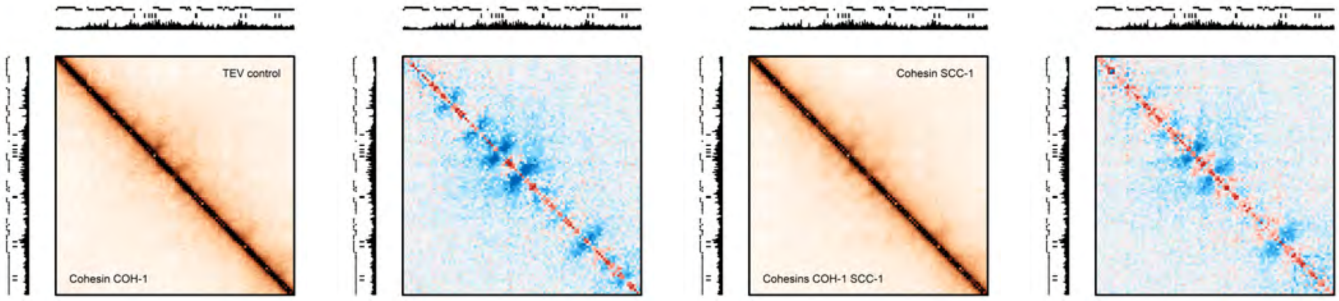

chrIII:12513001-12713000

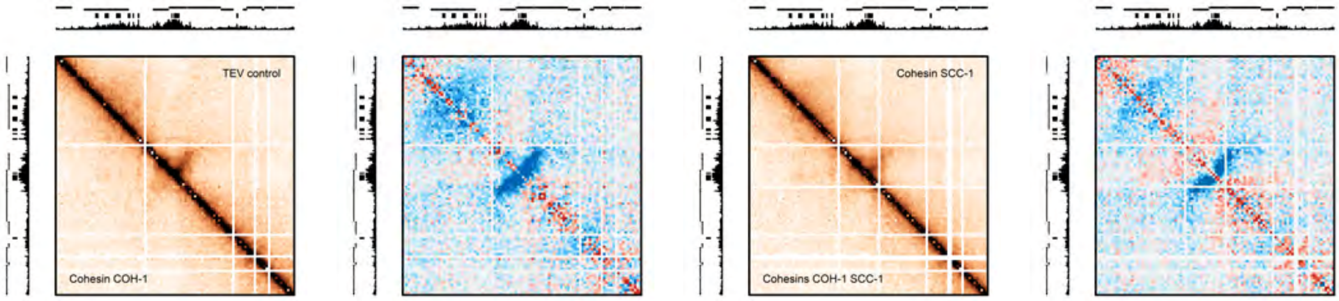

chrI:1597001-1797000

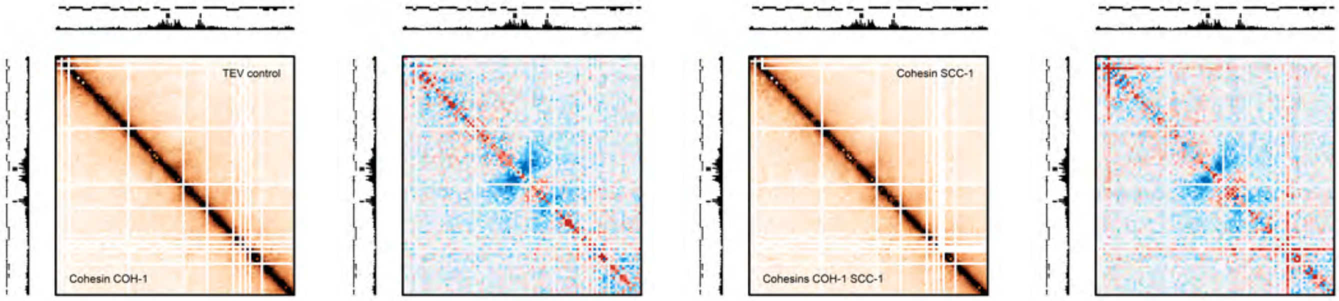

chrIV:4373001-4573000

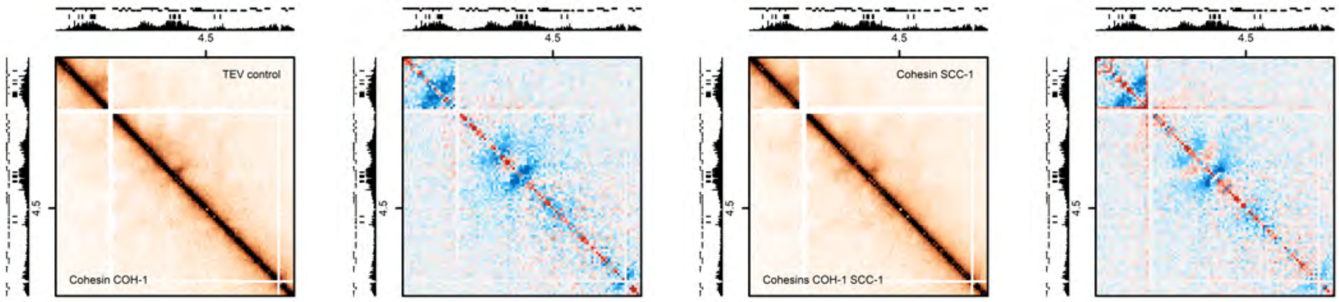

chrII:15109001-15309000

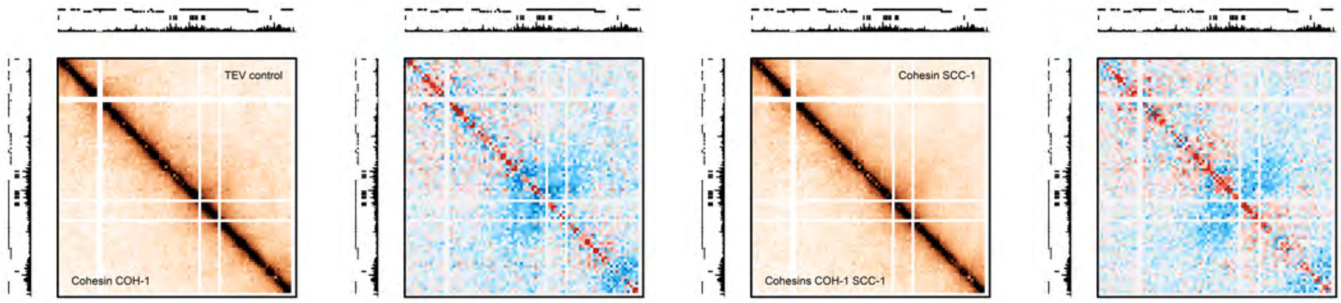

chrII:9181001-9381000

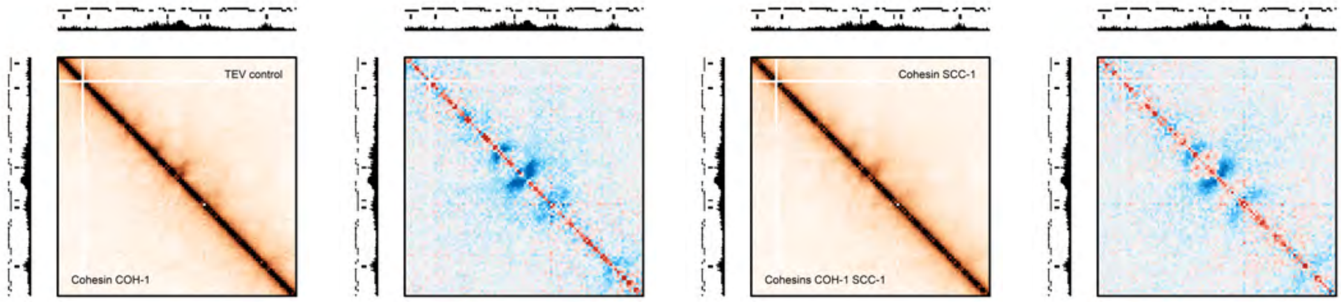

chrV:6127001-6327000

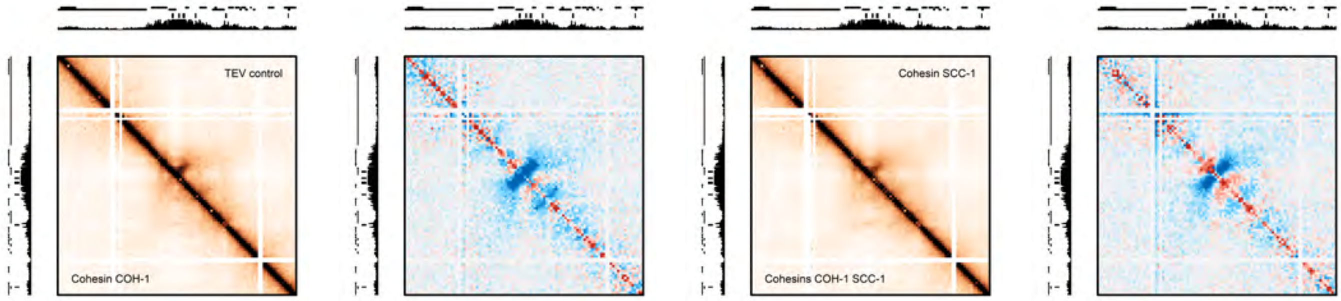

chrI:7393001-7593000

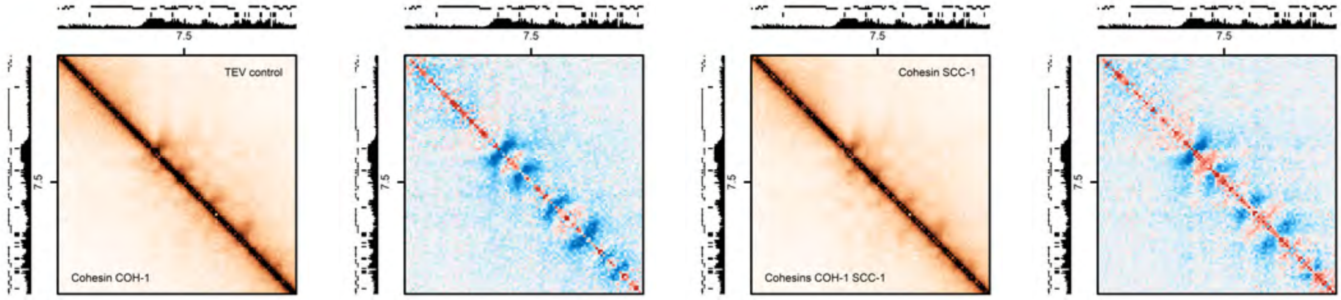

chrV:10769001-10969000

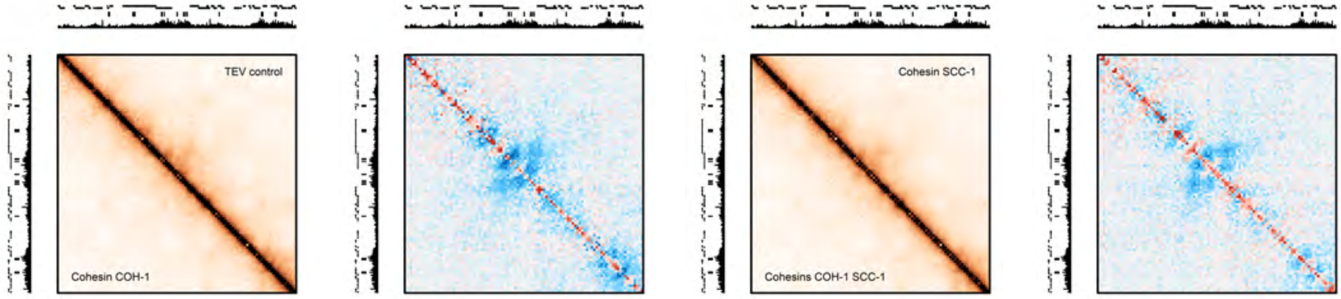

chrV:8837001-9037000

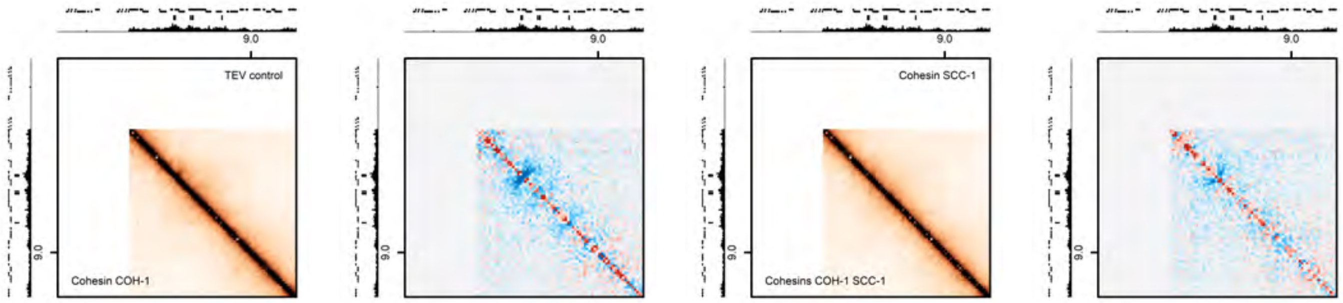

chrIII:8699001-8899000

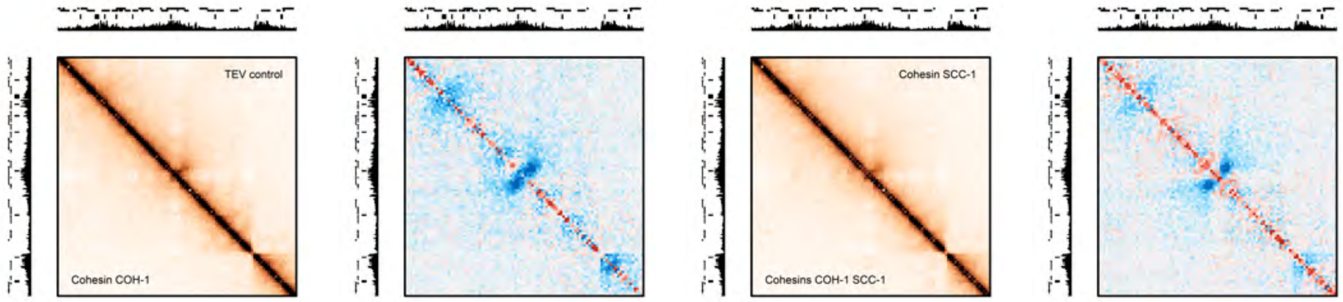

chrX:7919001-8119000

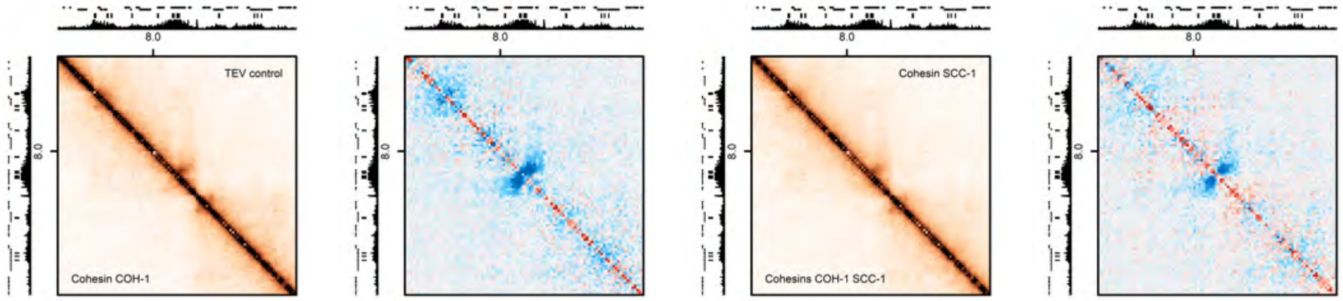

chrV:1053001-1253000

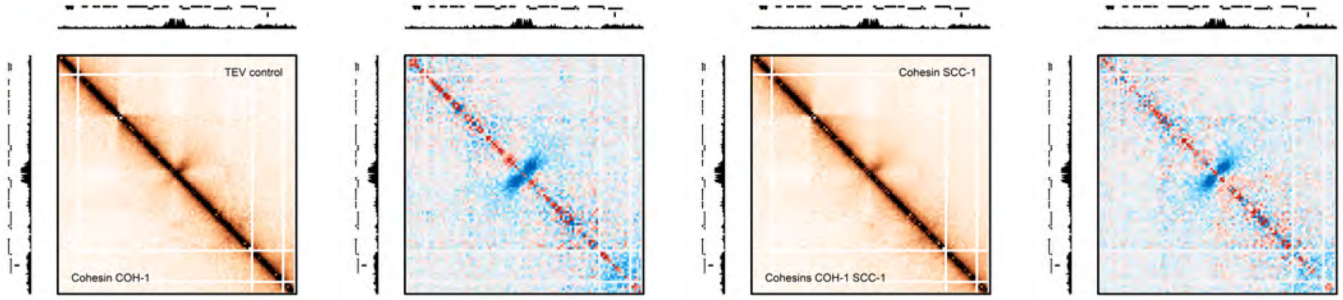

chrIV:3033001-3233000

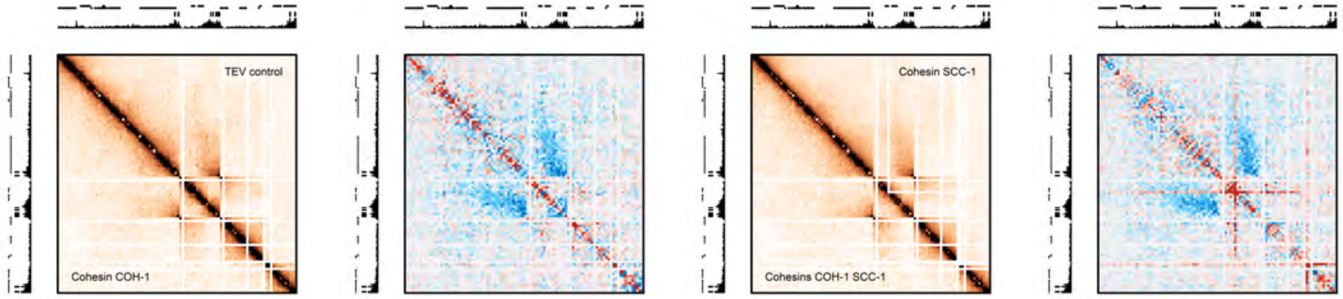

chrI:11943001-12143000

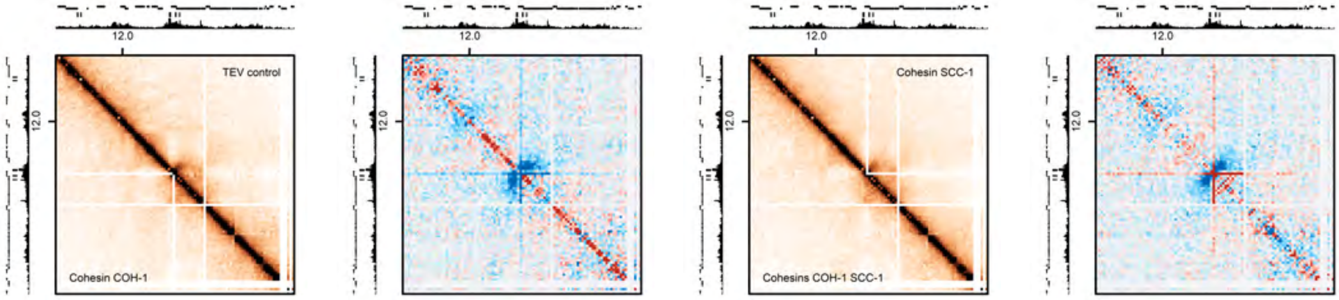

chrII:2875001-3075000

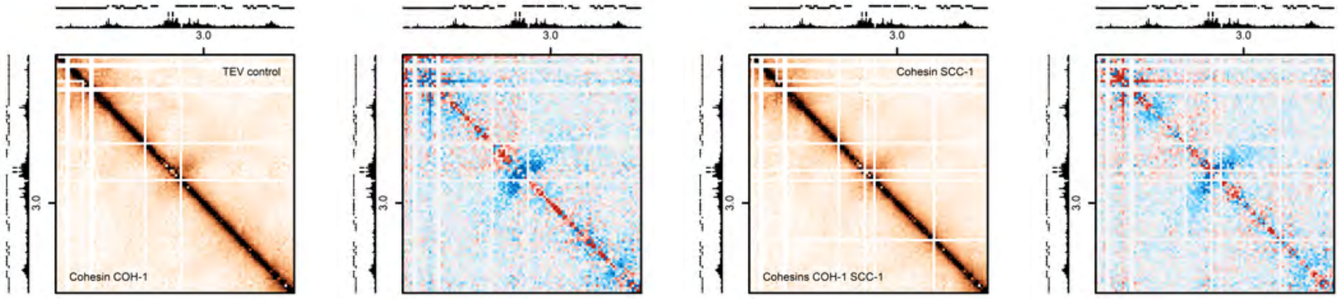

chrII:5233001-5433000

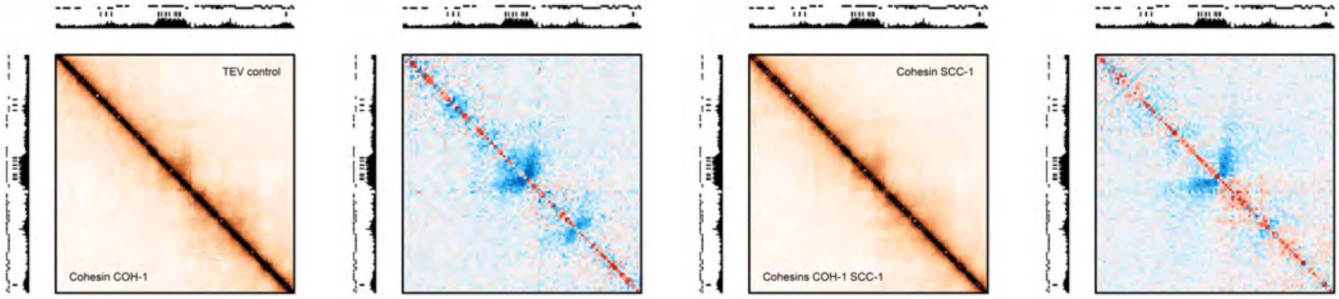

chrI:7767001-7967000

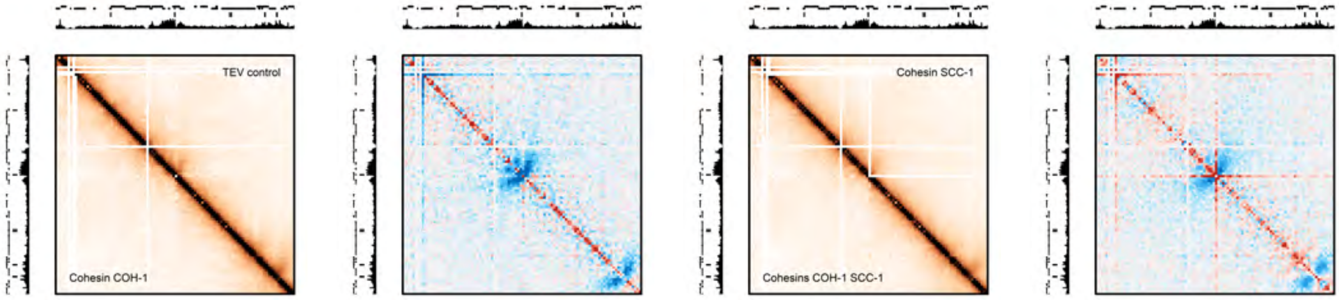

chrI:13271001-13471000

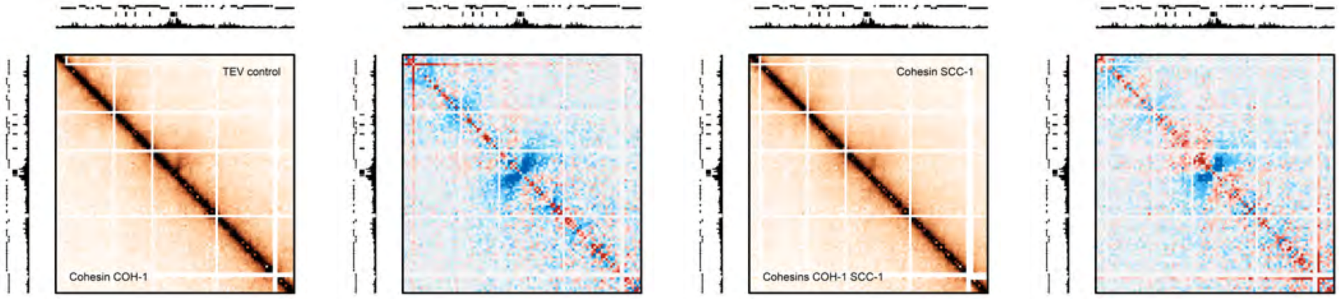

chrI:10657001-10857000

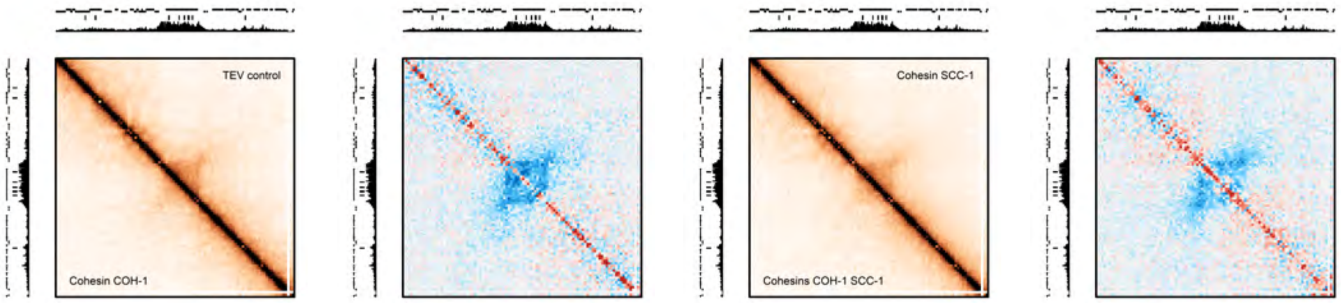

chrIII:3699001-3899000

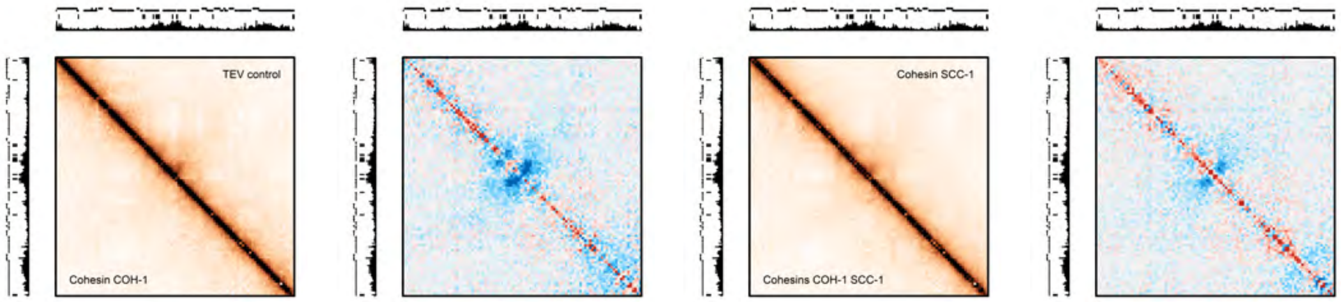

chrX:10541001-10741000

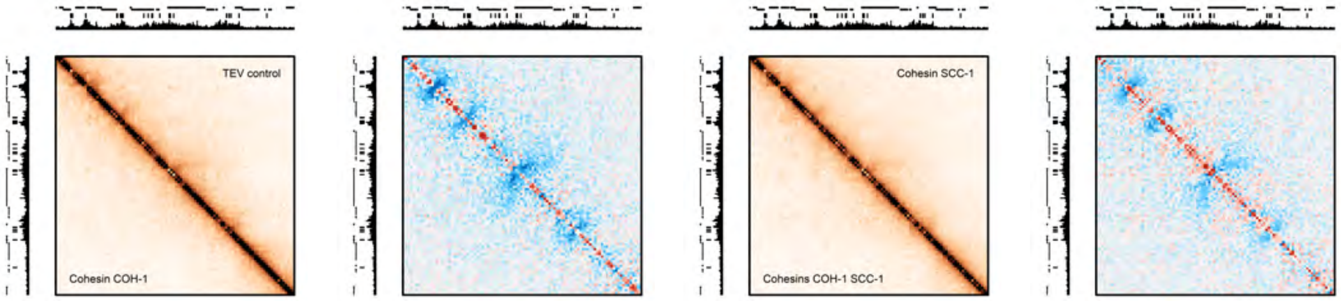

chrII:13363001-13563000

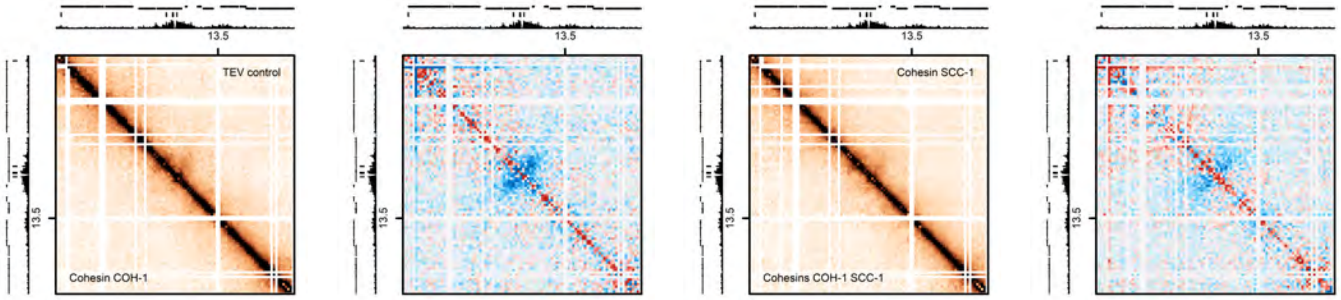

chrIV:10403001-10603000

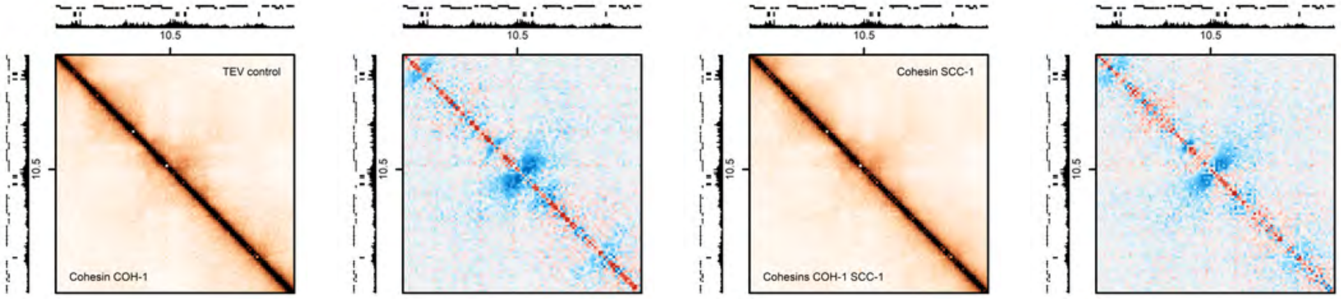

chrX:16833001-17033000

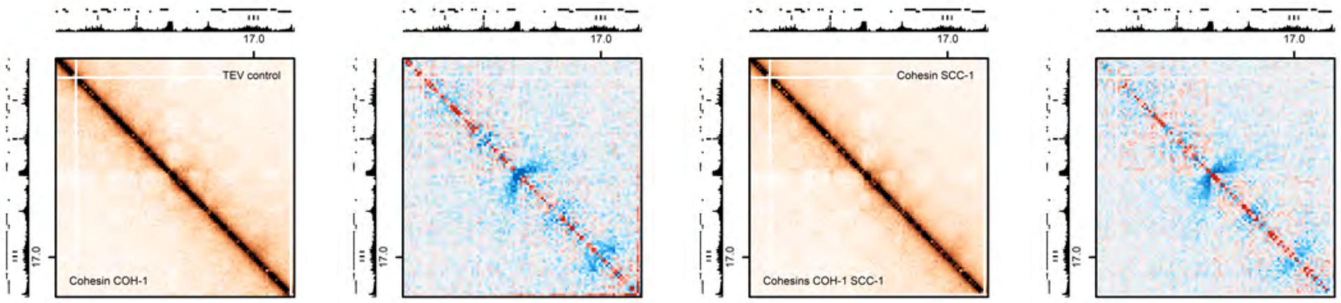

chrI:-62999-137000

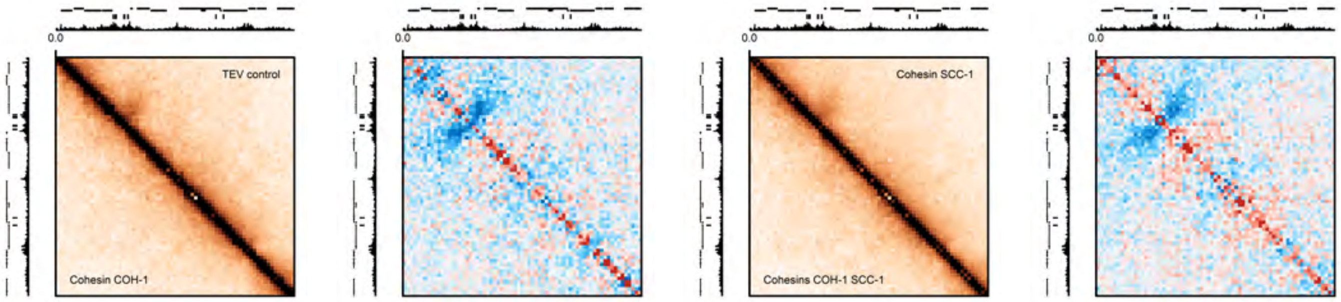

chrIII:7627001-7827000

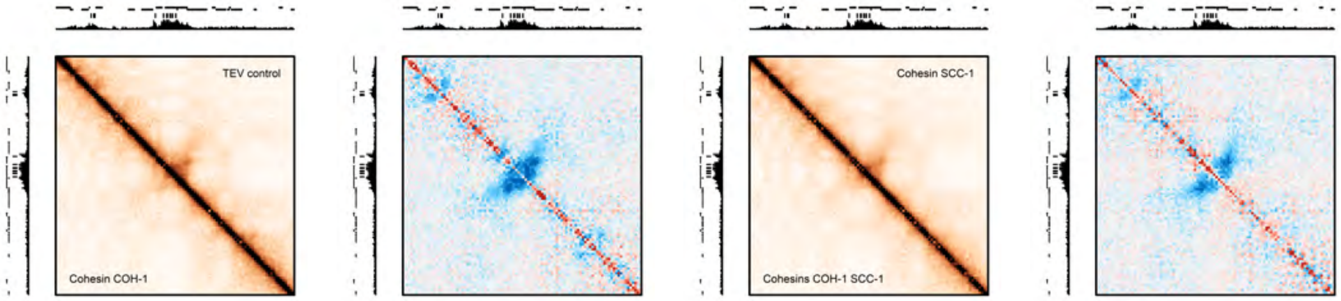

chrI:5281001-5481000

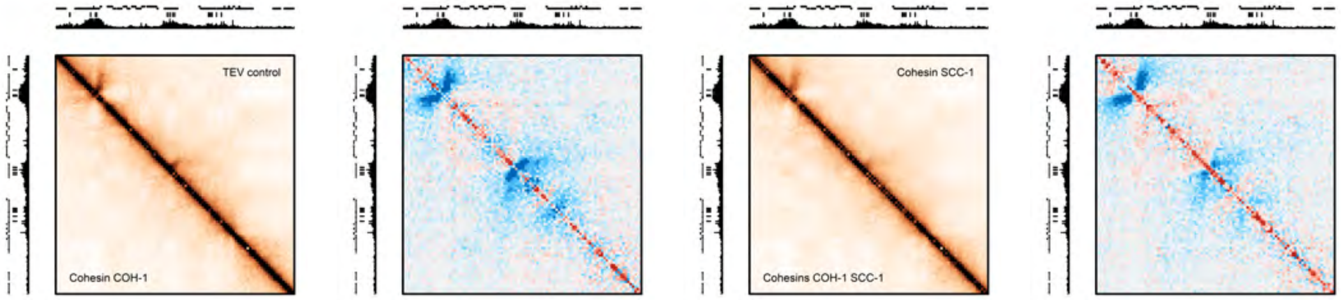

chrIII:9601001-9801000

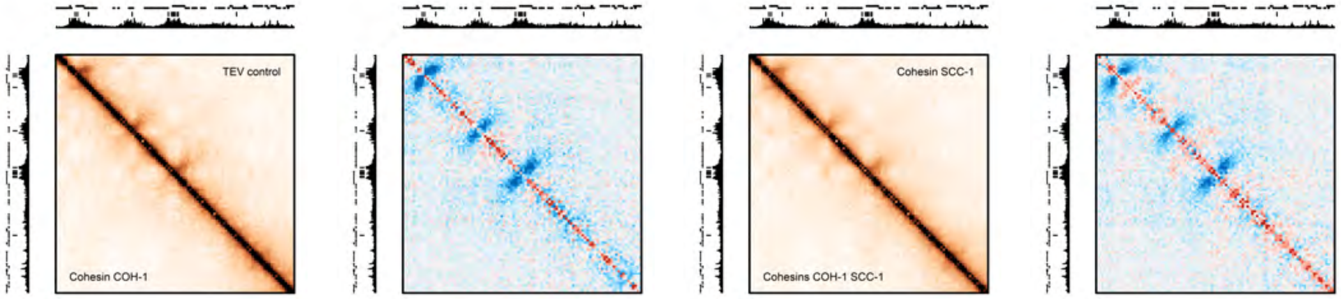

chrIII:6261001-6461000

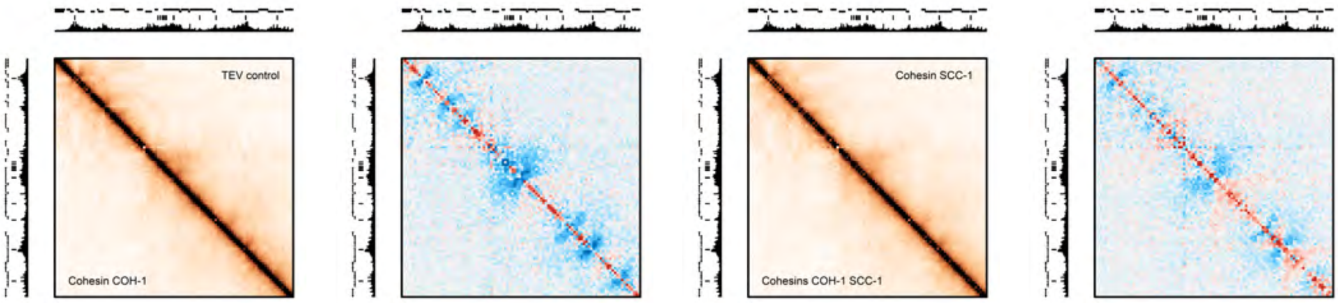

chrIV:2503001-2703000

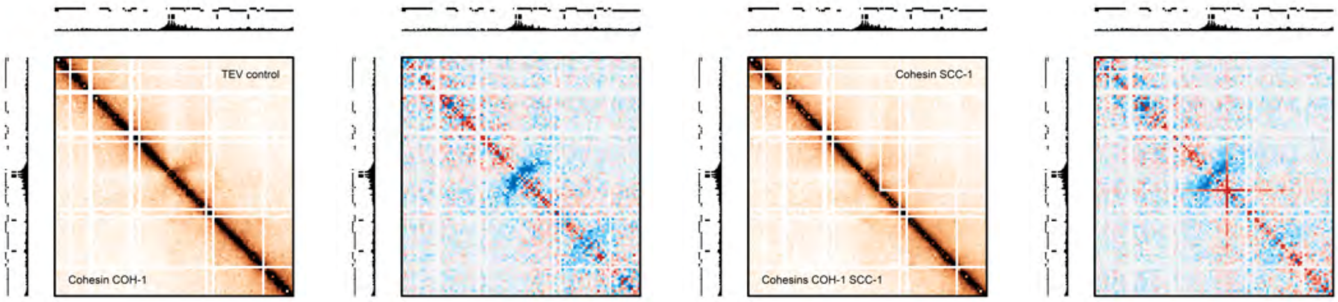

chrIII:251001-451000

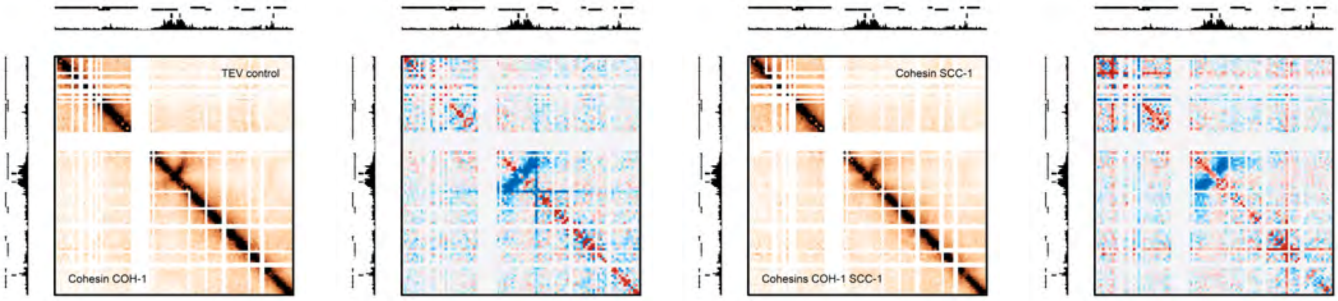

chrIV:1553001-1753000

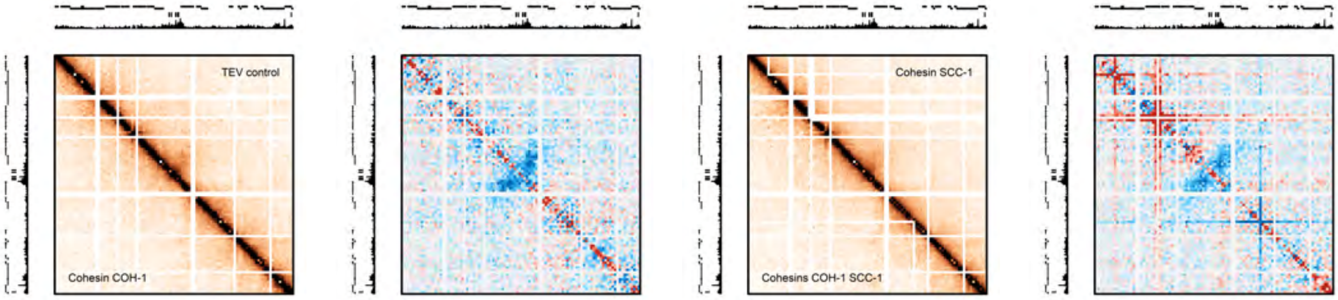

chrX:12413001-12613000

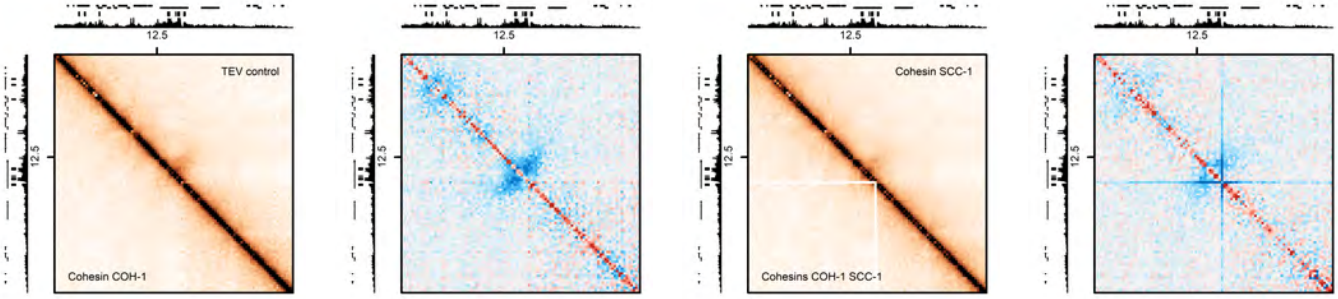

chrV:12987001-13187000

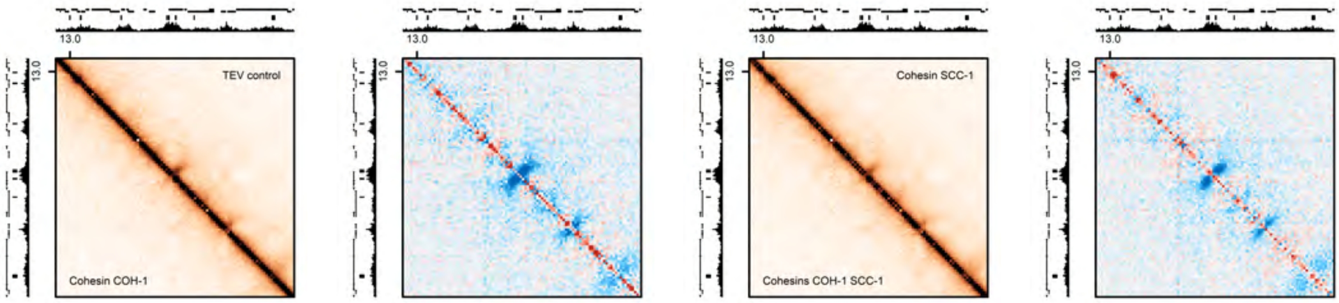

chrI:1737001-1937000

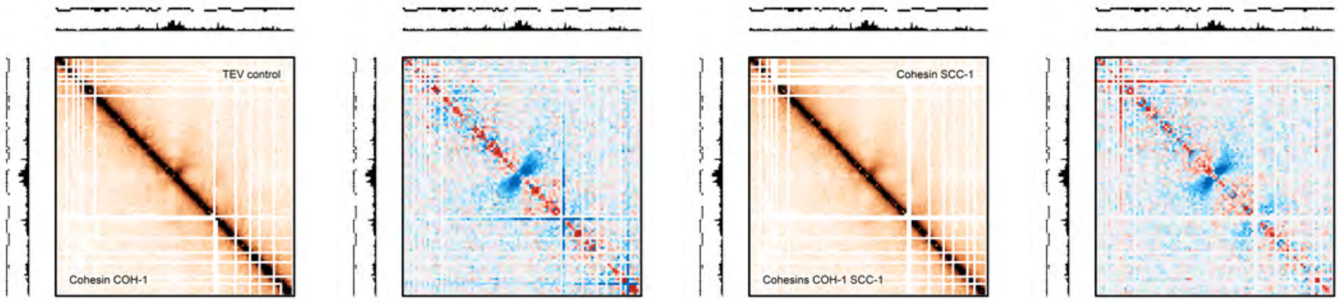

chrII:13943001-14143000

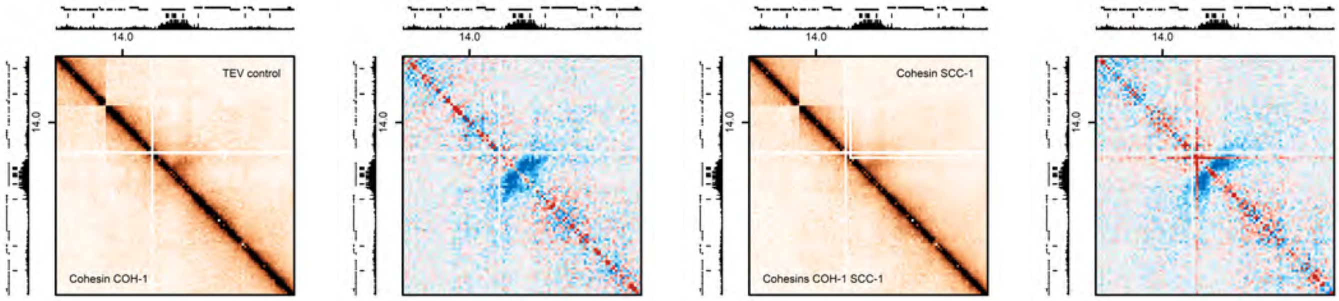

chrIV:7933001-8133000

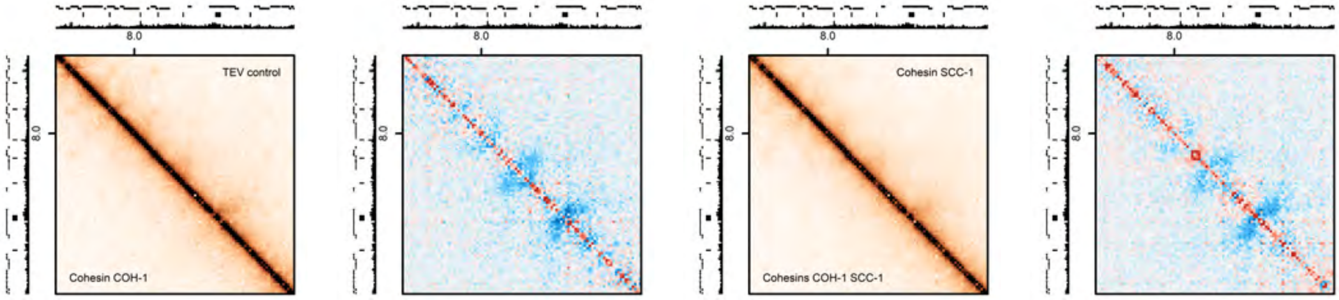

chrII:11533001-11733000

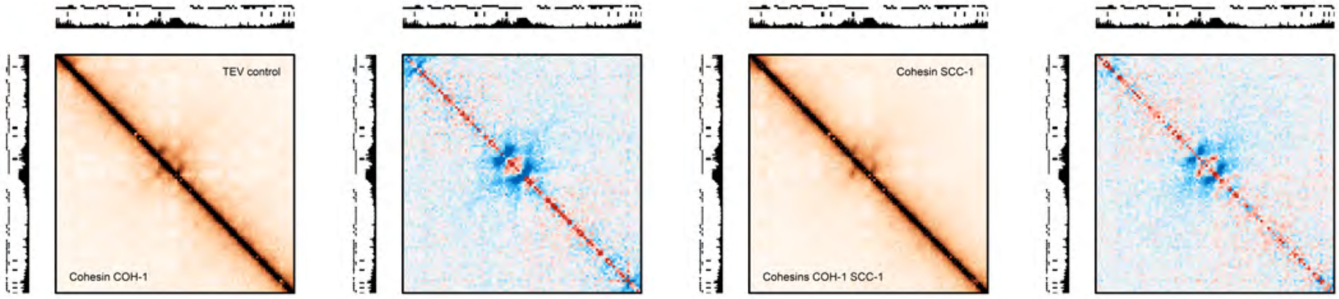

chrIV:14515001-14715000

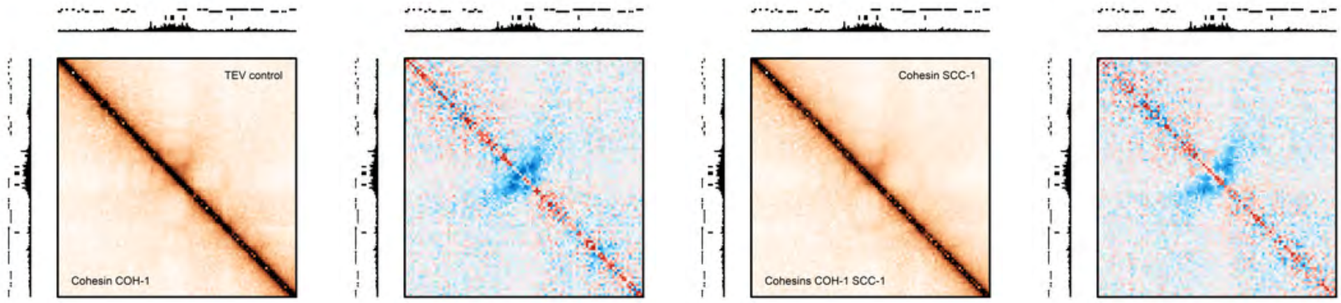

chrII:7793001-7993000

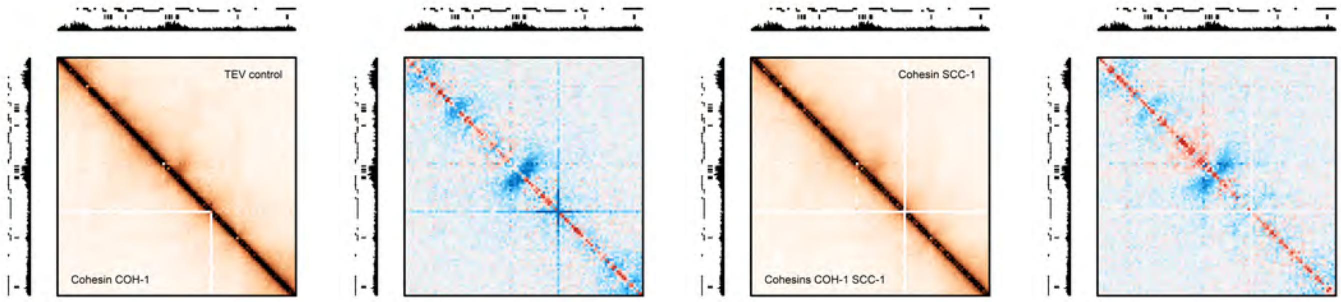

chrX:2495001-2695000

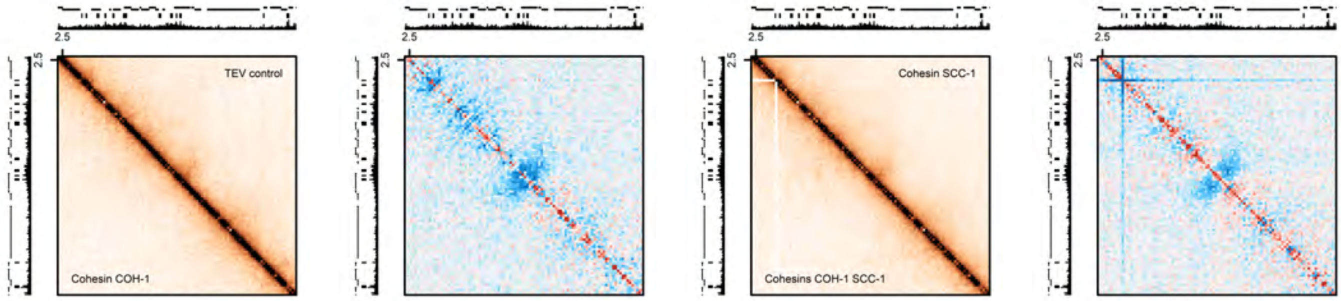

chrV:8325001-8525000

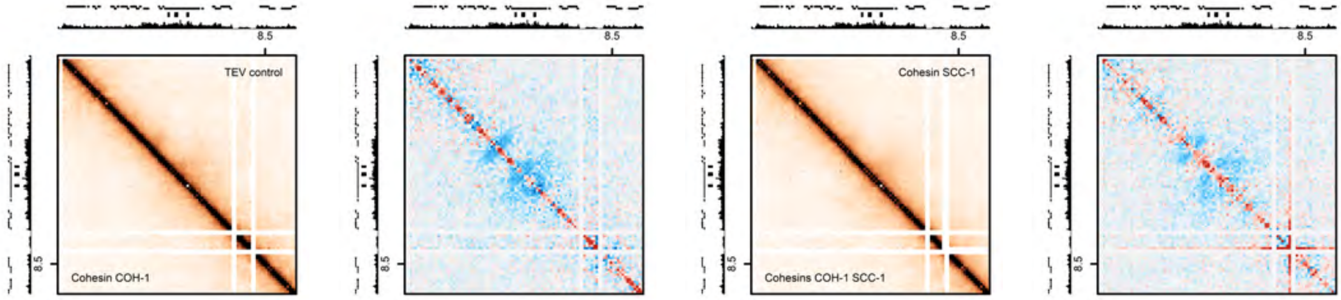

chrIV:3927001-4127000

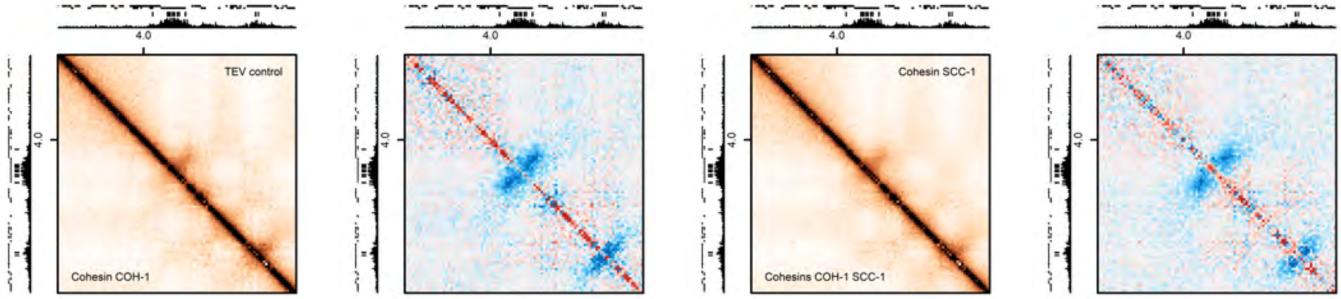

chrX:6153001-6353000

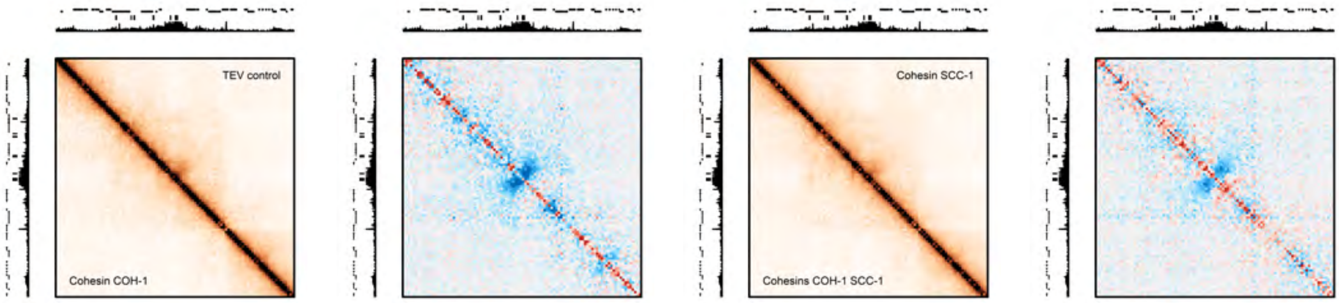

chrIII:1831001-2031000

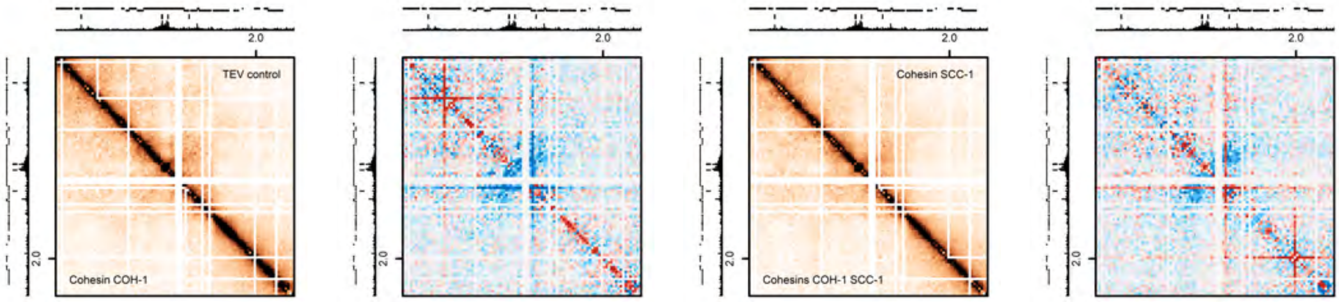

chrI:9281001-9481000

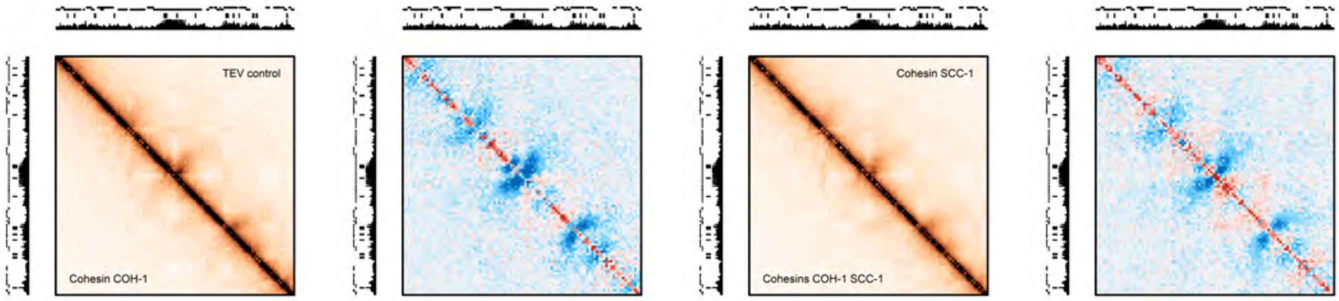

chrV:14749001-14949000

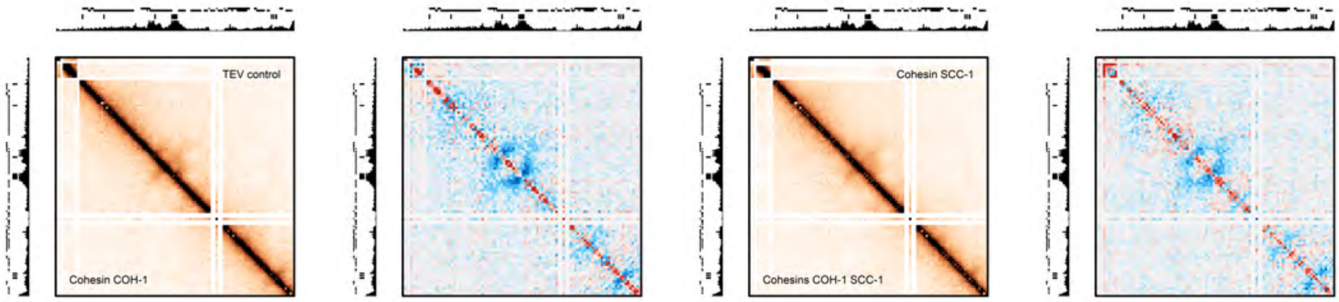

chrII:12697001-12897000

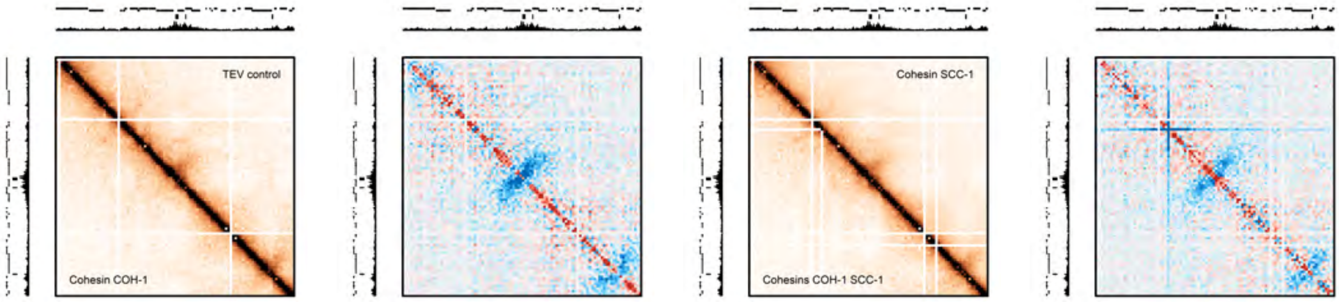

chrV:10369001-10569000

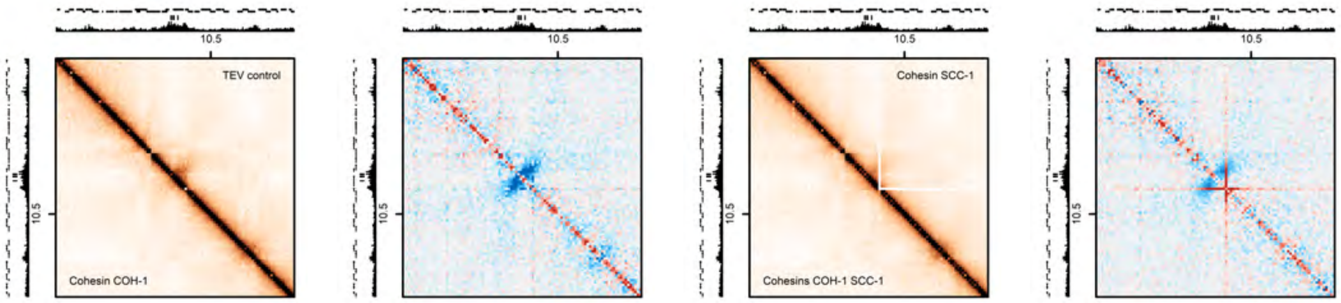

chrX:12077001-12277000

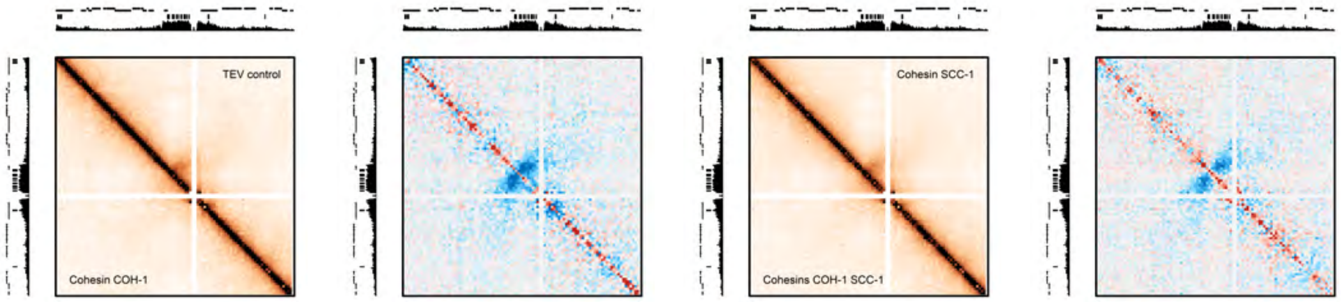

chrI:3621001-3821000

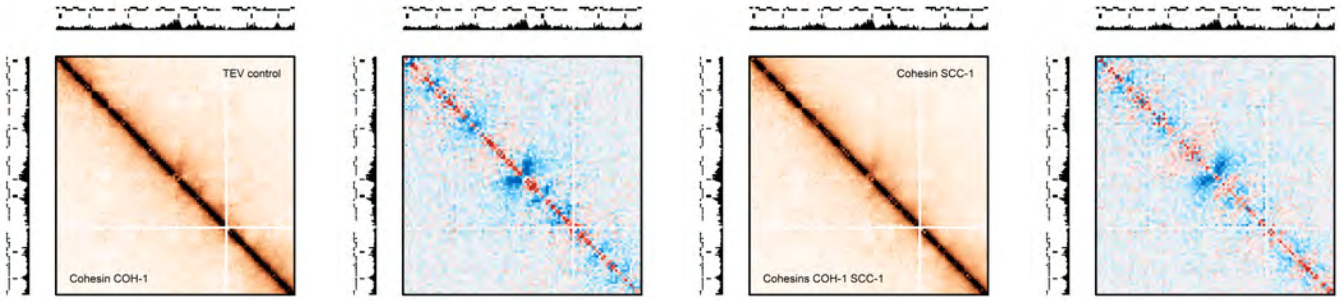

chrIII:4401001-4601000

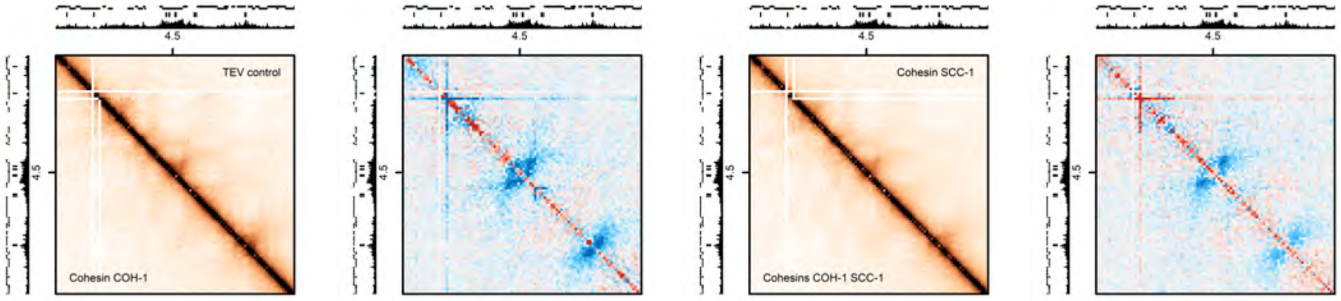

chrV:-84999-115000

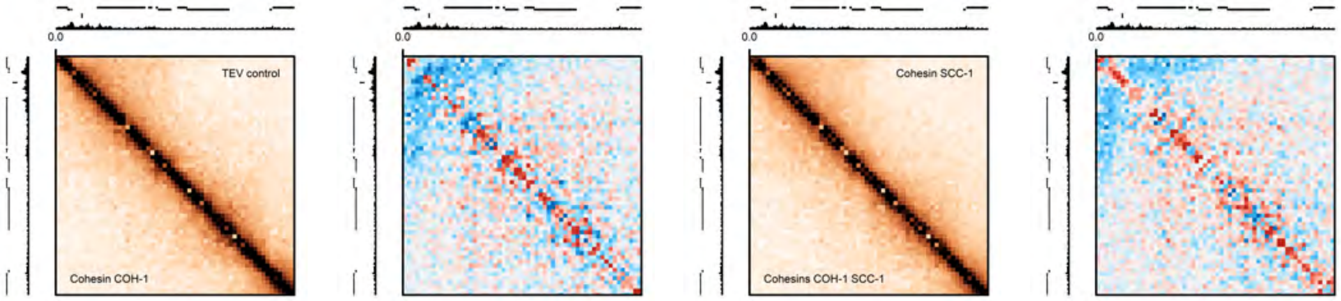

chrIV:16391001-16591000

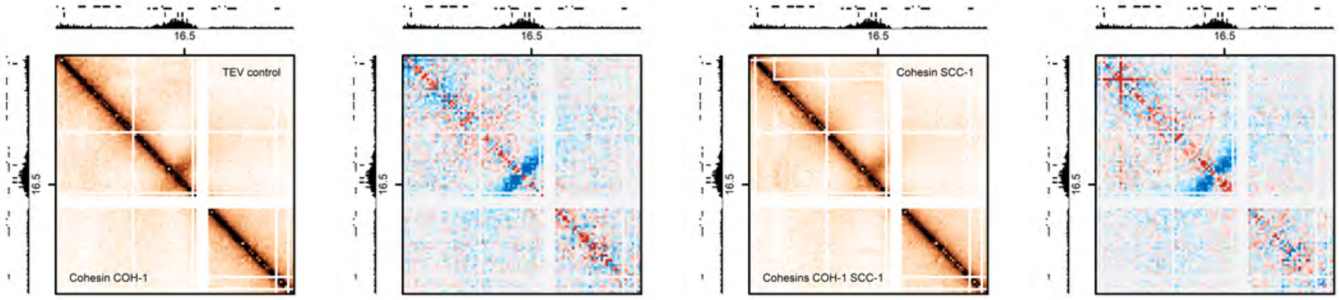

chrIII:7053001-7253000

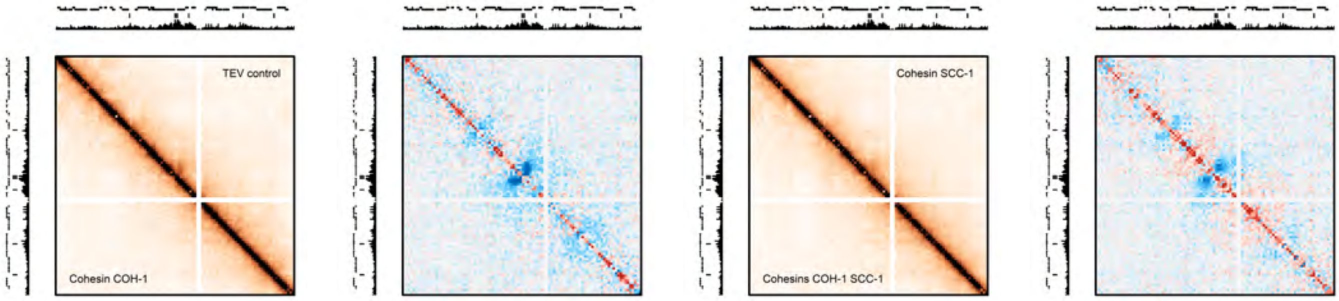

chrI:6479001-6679000

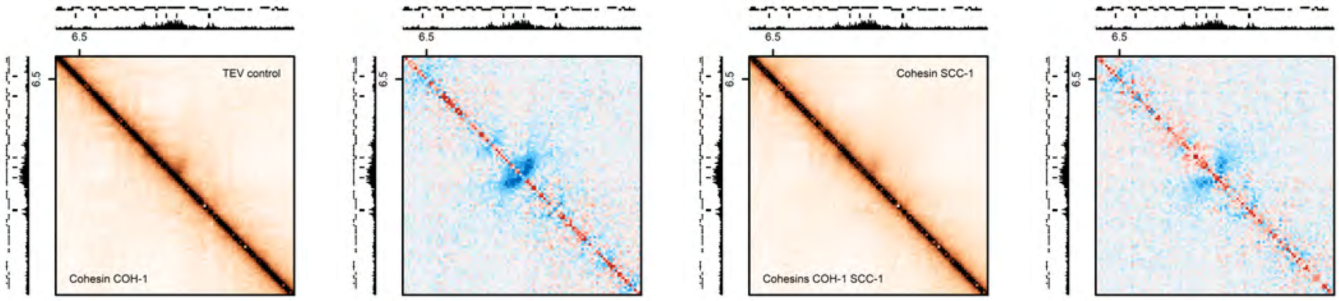

chrX:15177001-15377000

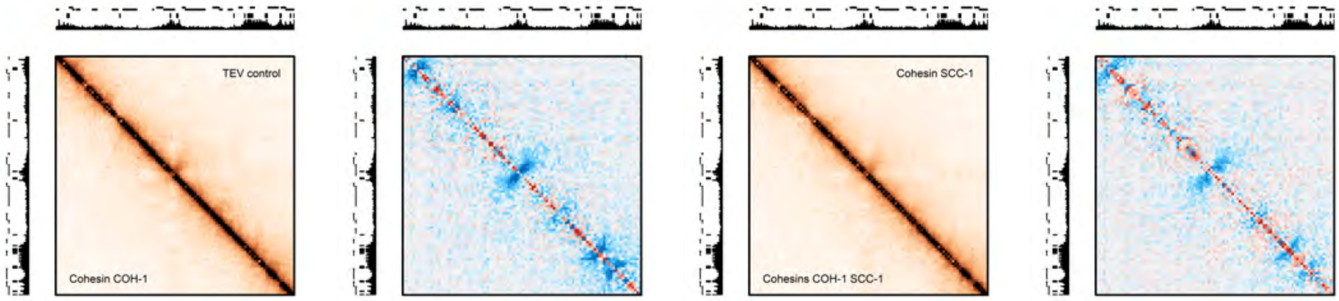

chrIV:12931001-13131000

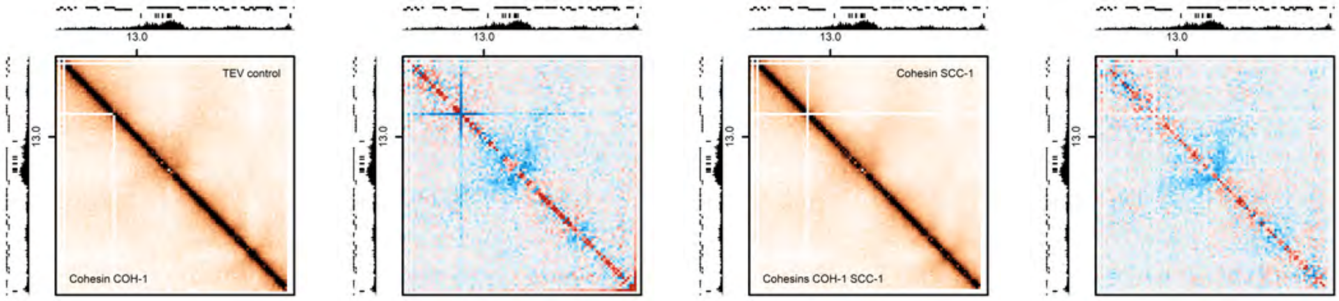

chrII:3859001-4059000

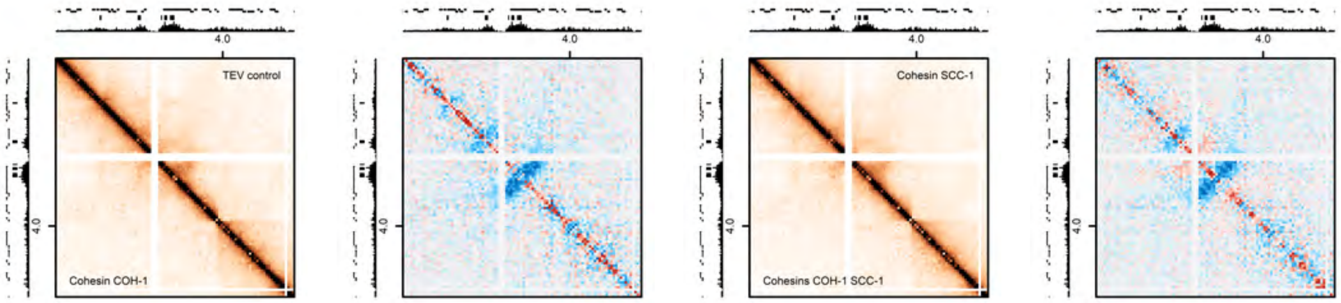

chrV:14403001-14603000

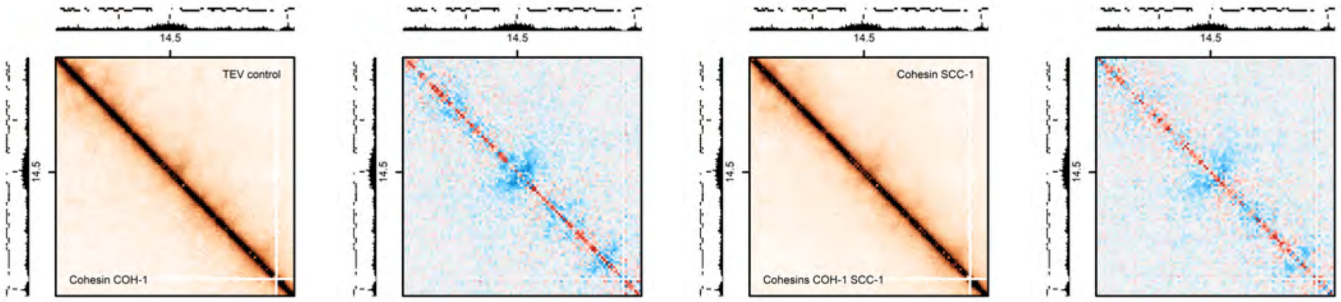

chrX:5717001-5917000

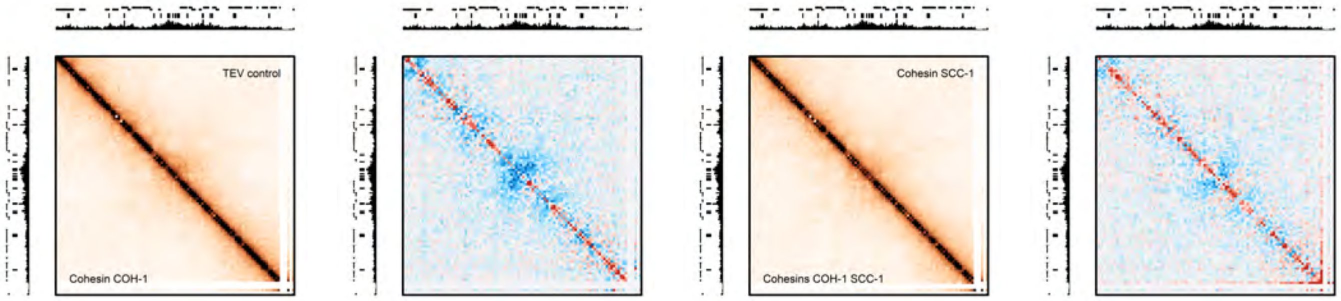

chrIII:11101001-11301000

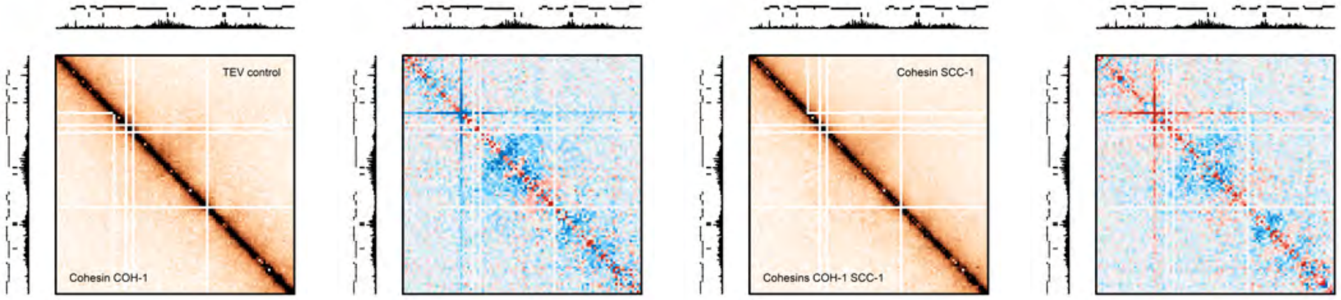

chrIV:13789001-13989000

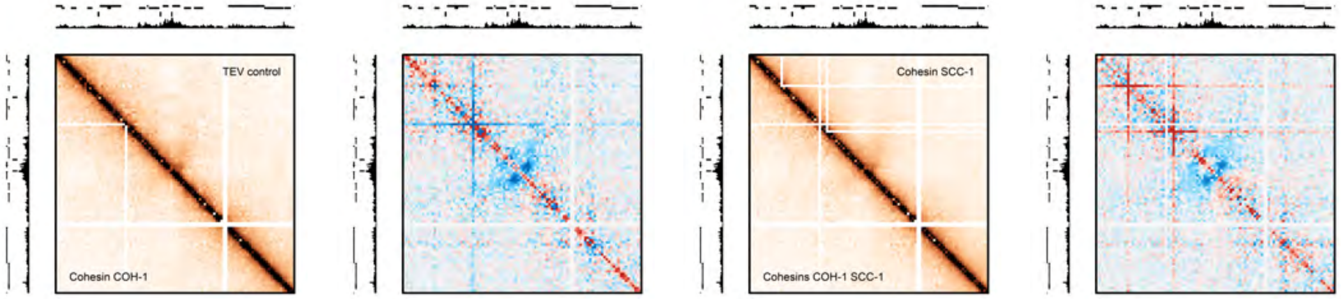

chrX:11375001-11575000

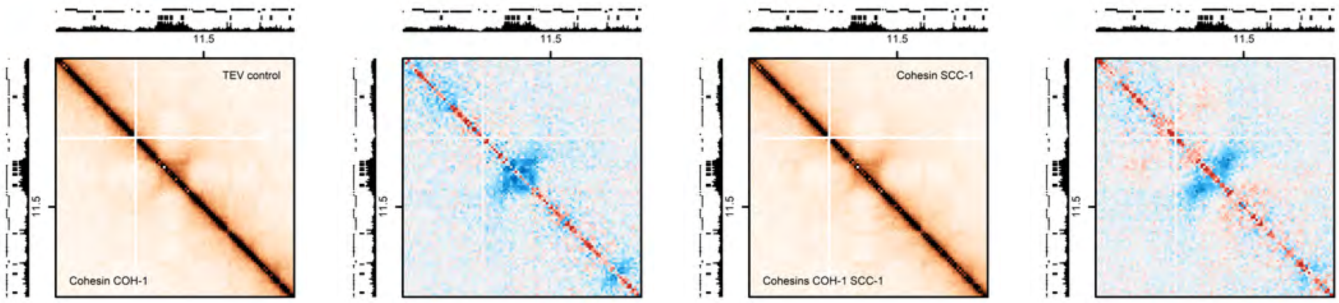

chrIII:10535001-10735000

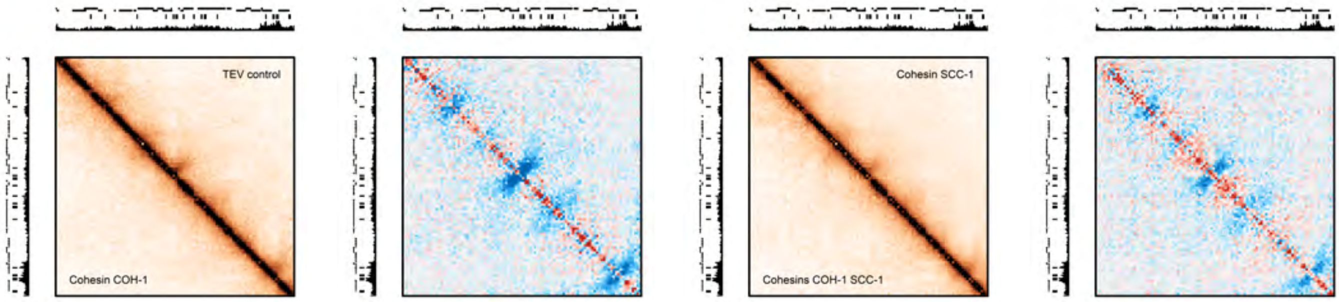

chrI:9625001-9825000

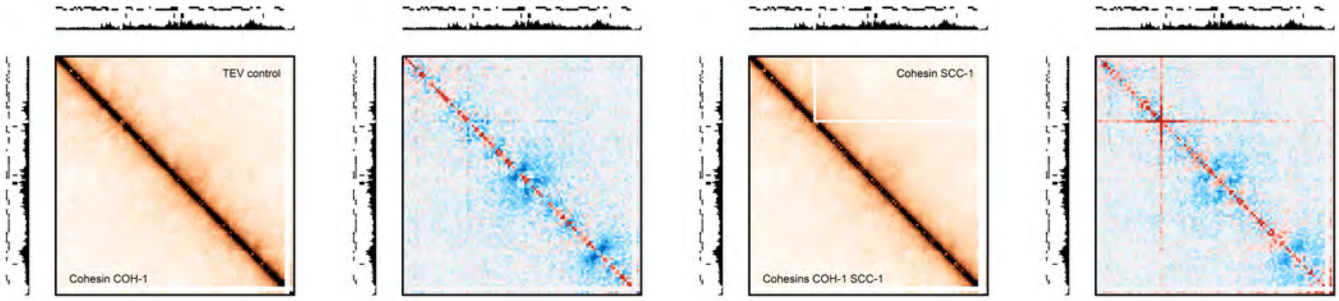

chrIV:5653001-5853000

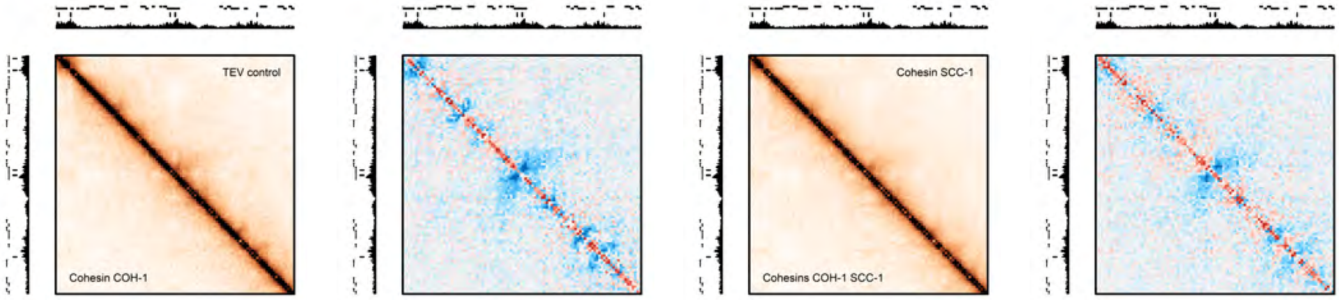

chrI:969001-1169000

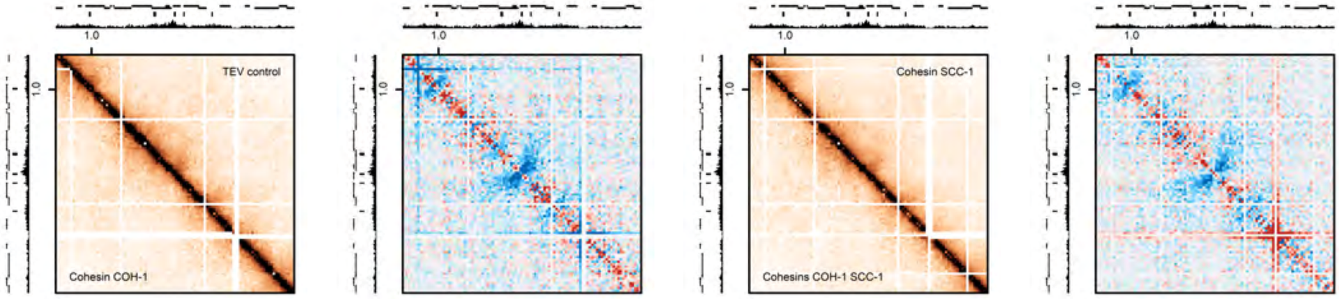

chrV:5745001-5945000

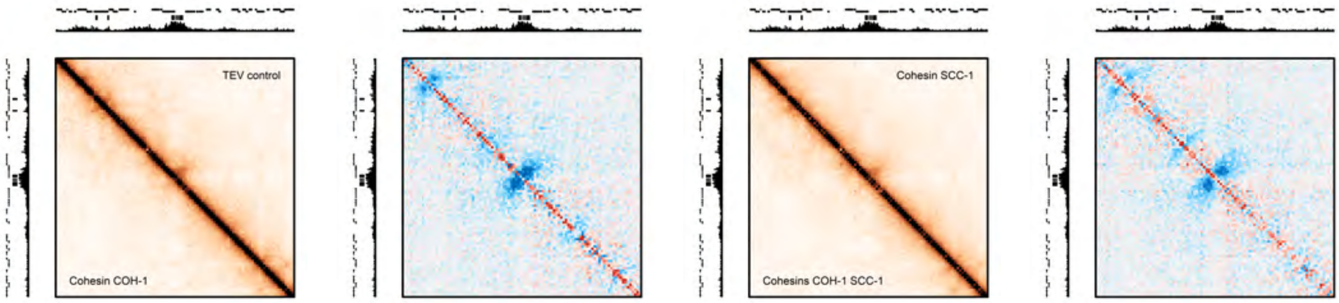

chrII:13191001-13391000

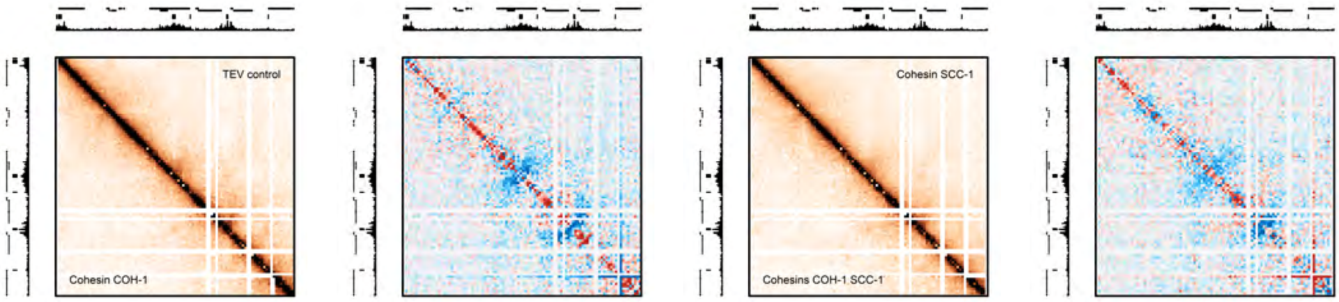

chrIII:11589001-11789000

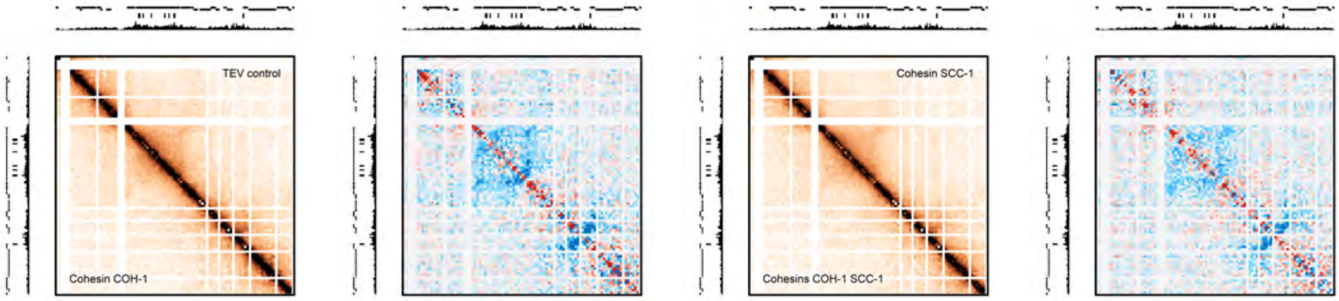

chrX:13065001-13265000

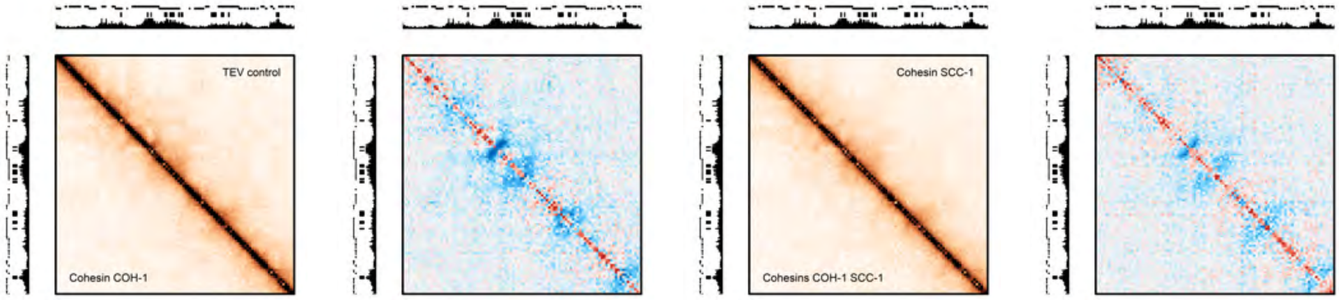

chrI:2487001-2687000

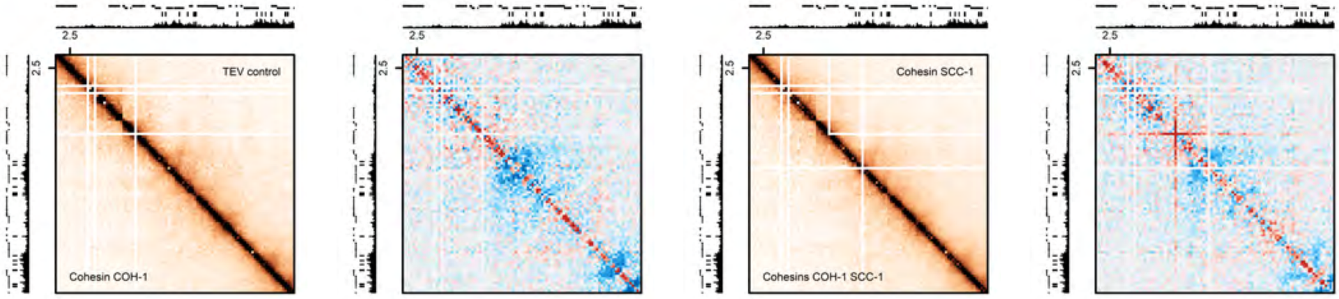

chrIII:8025001-8225000

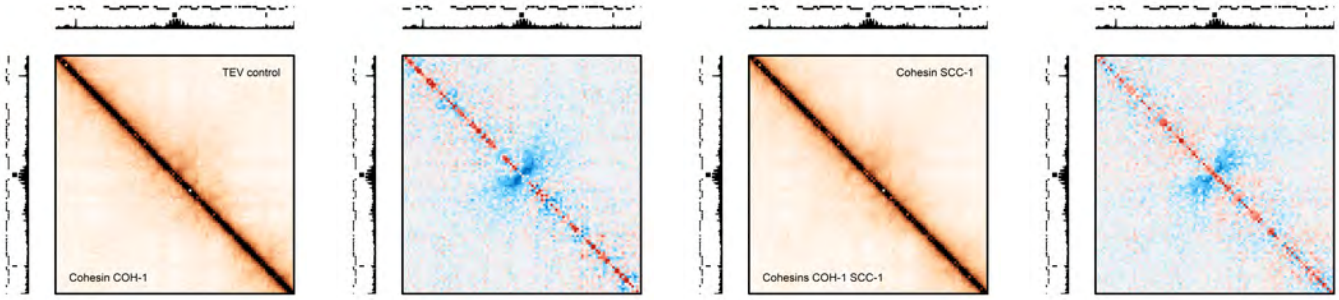

chrV:20679001-20879000

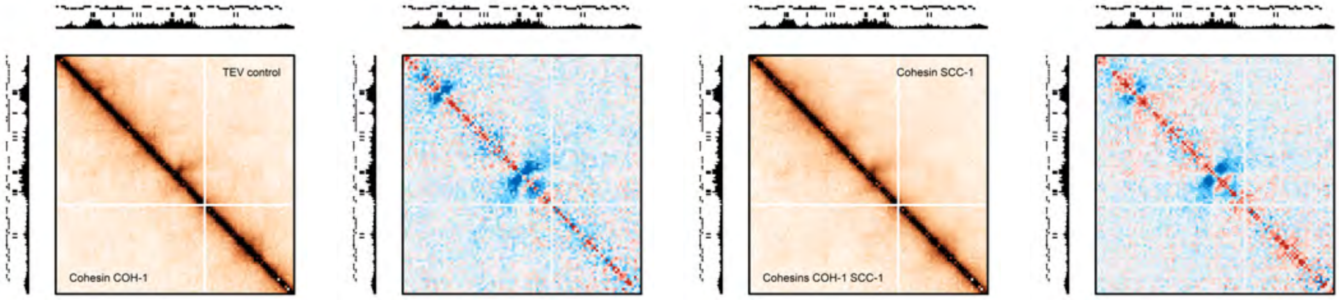

chrI:1921001-2121000

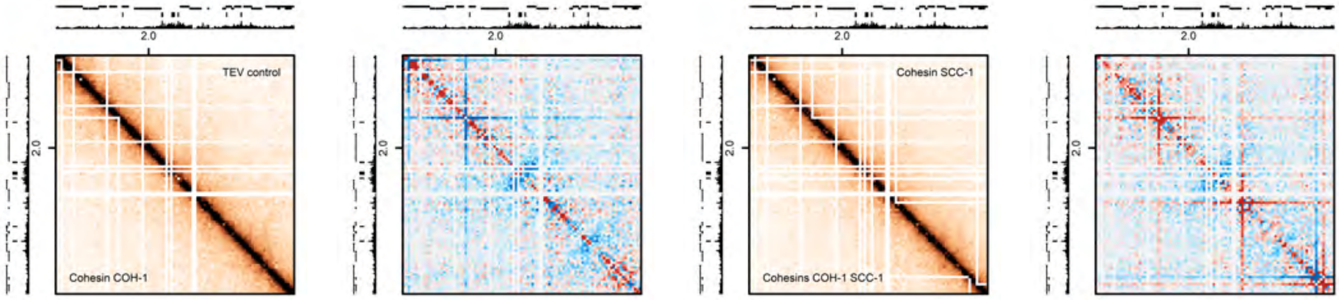

chrX:10087001-10287000

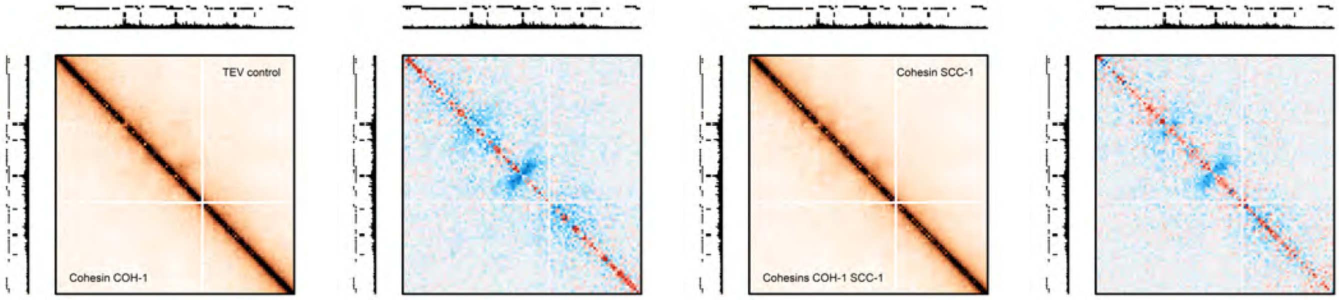

chrII:5835001-6035000

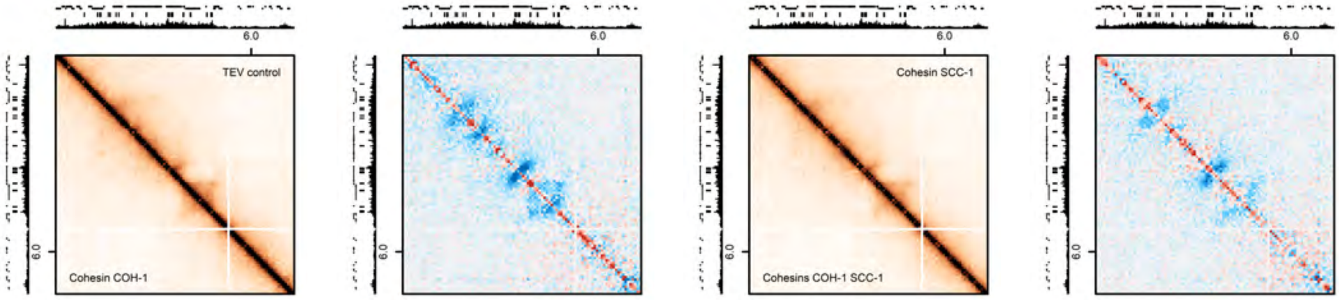

chrIII:6891001-7091000

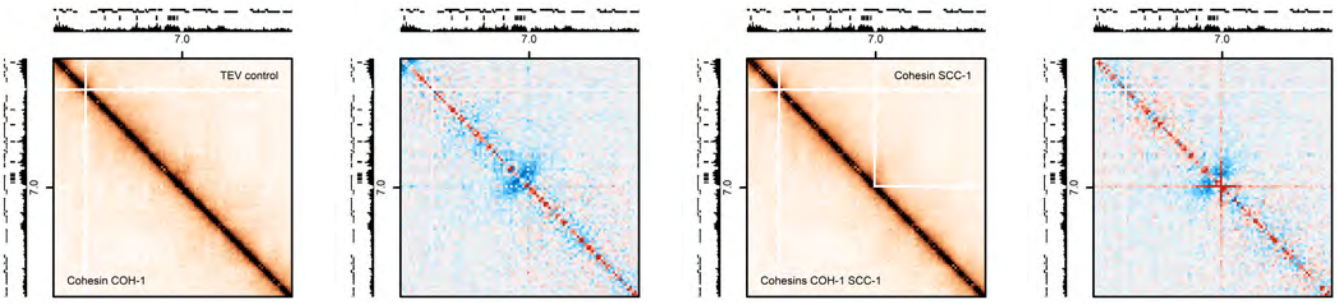

chrI:12719001-12919000

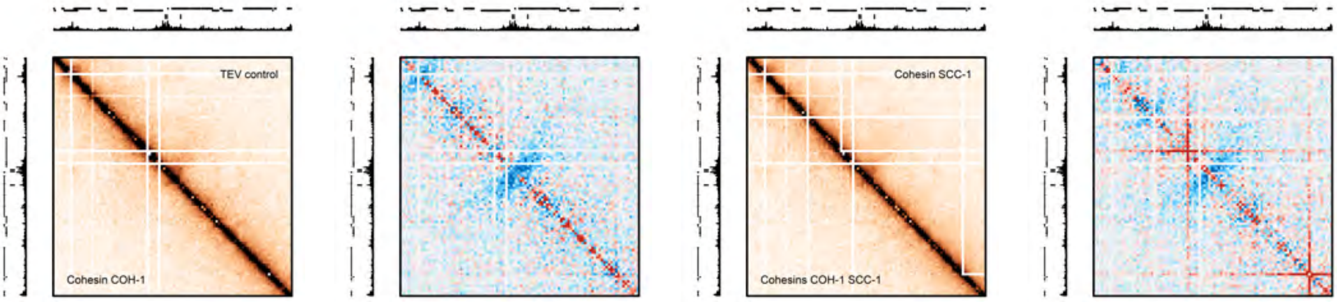

chrII:1139001-1339000

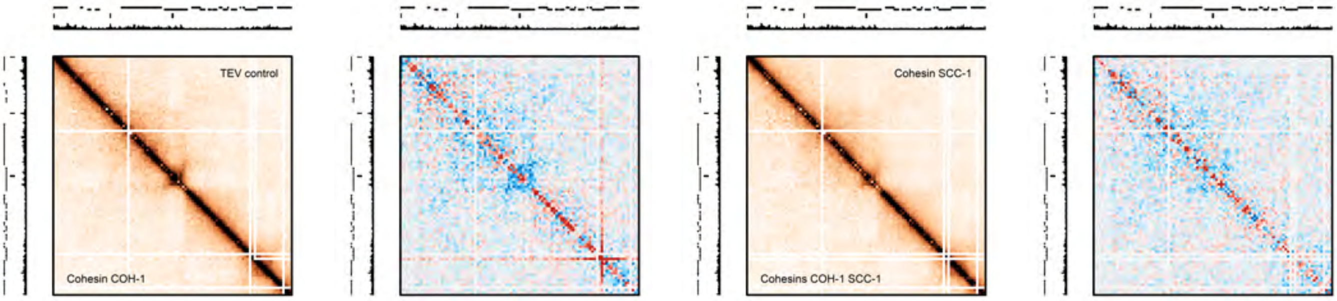

chrV:10619001-10819000

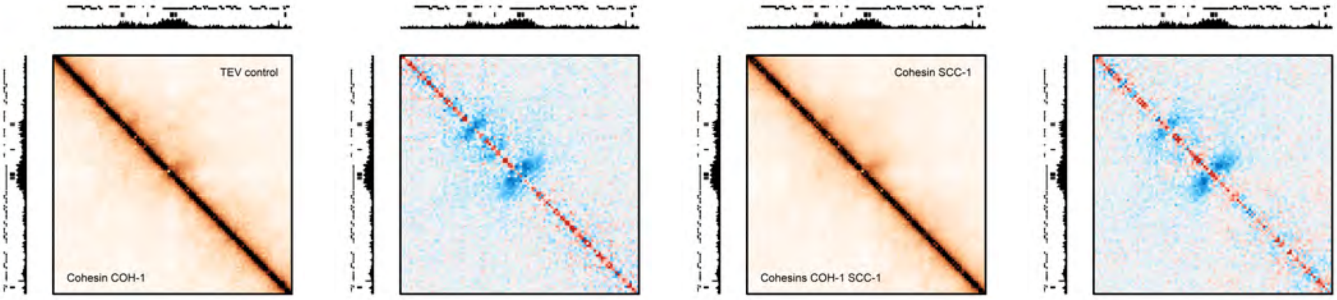

chrIII:6037001-6237000

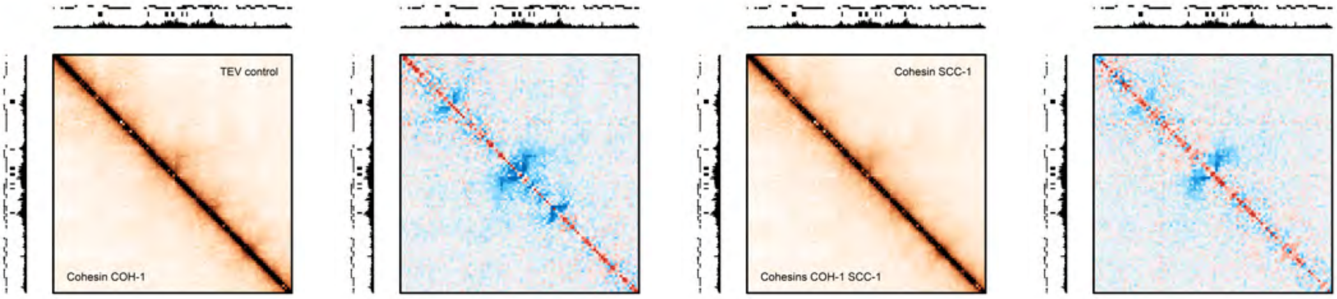

chrV:3595001-3795000

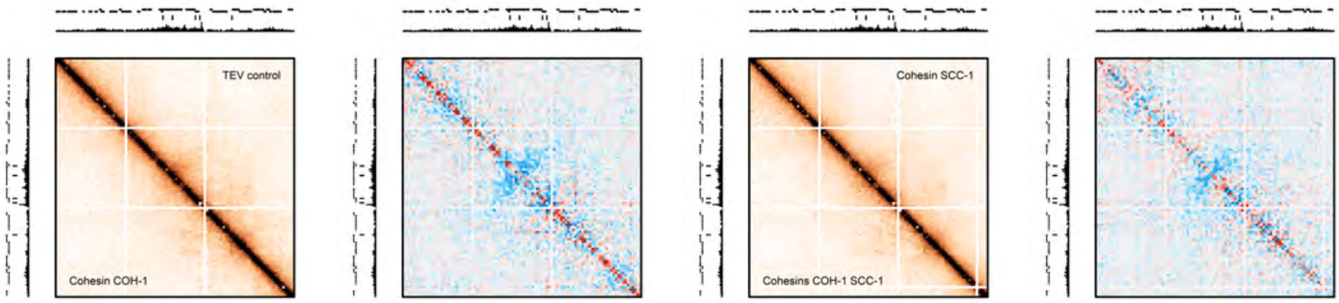

chrIV:2121001-2321000

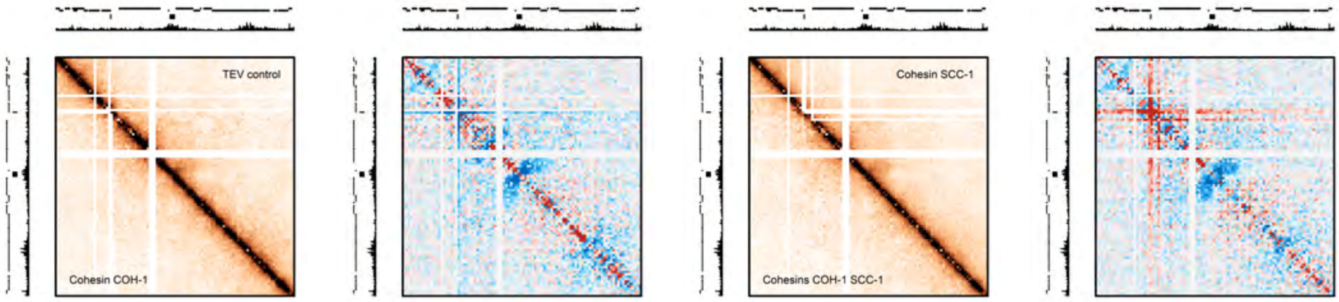

chrII:12925001-13125000

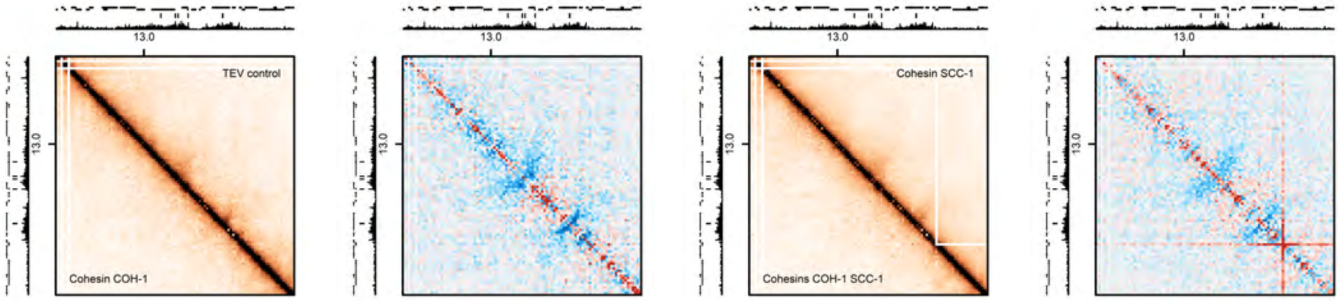

chrII:14503001-14703000

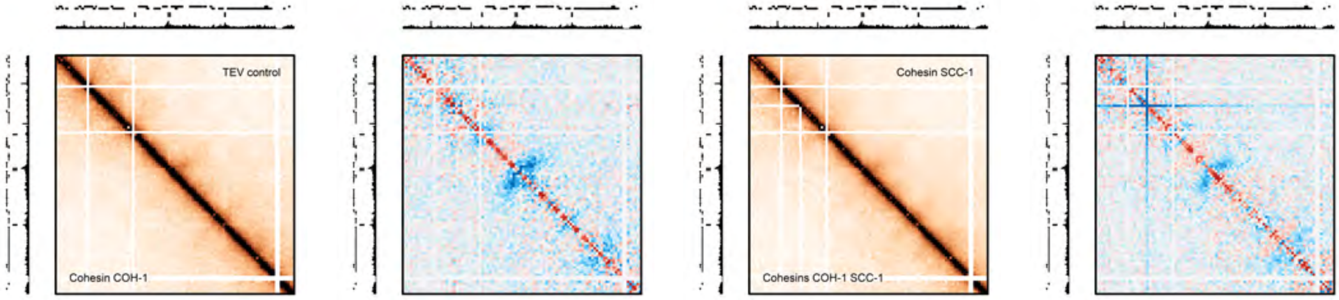

chrIV:10719001-10919000

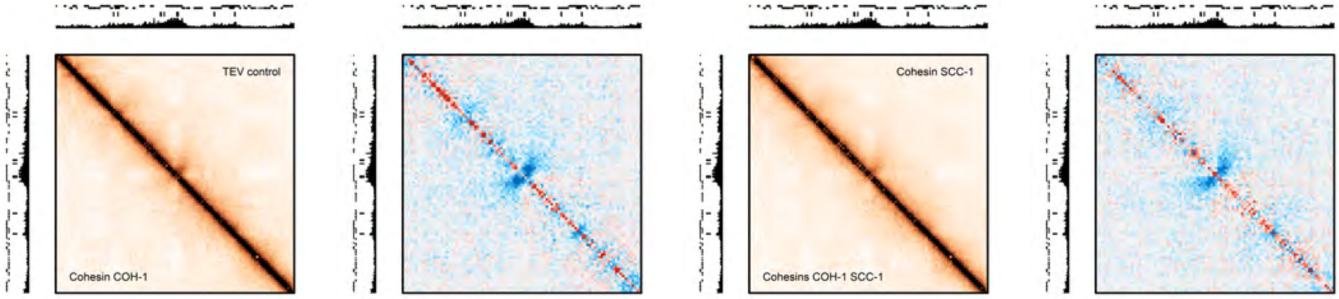

chrIV:2895001-3095000

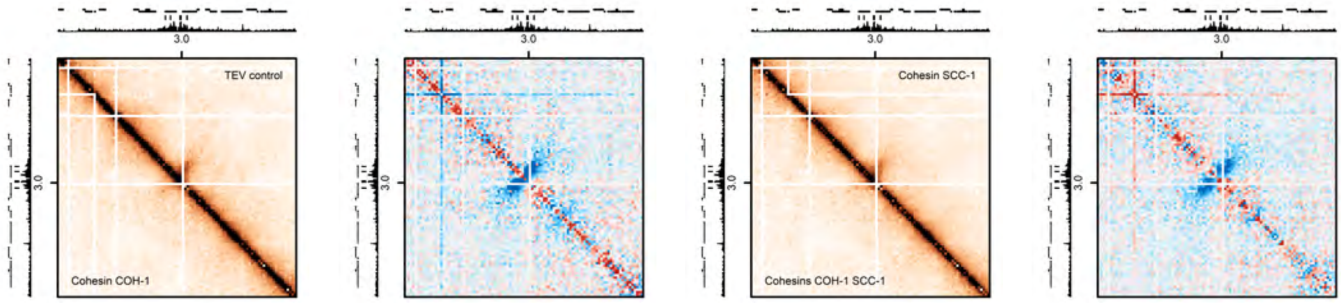

chrII:9505001-9705000

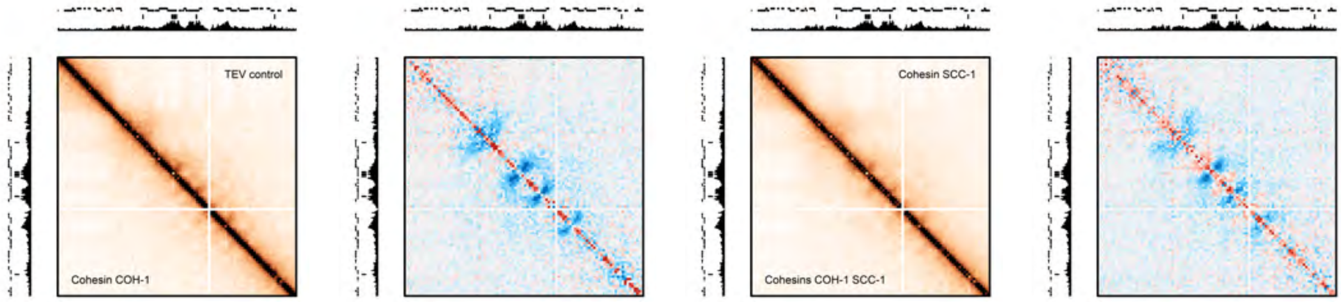

chrV:4405001-4605000

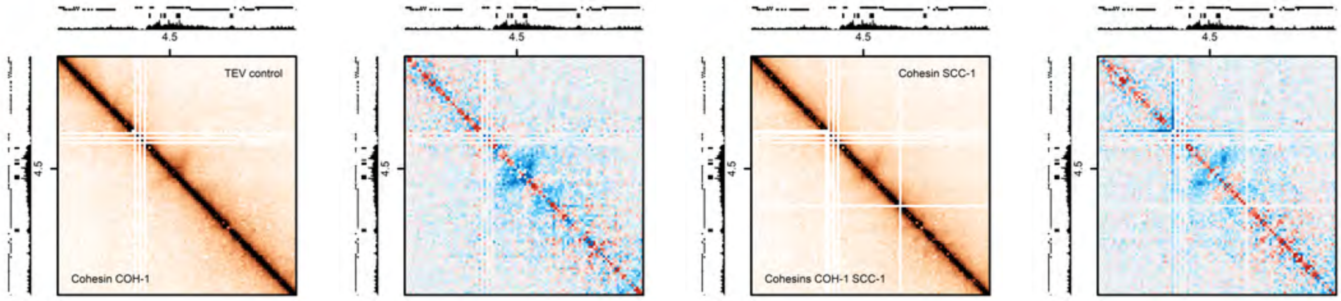

chrI:14837001-15037000

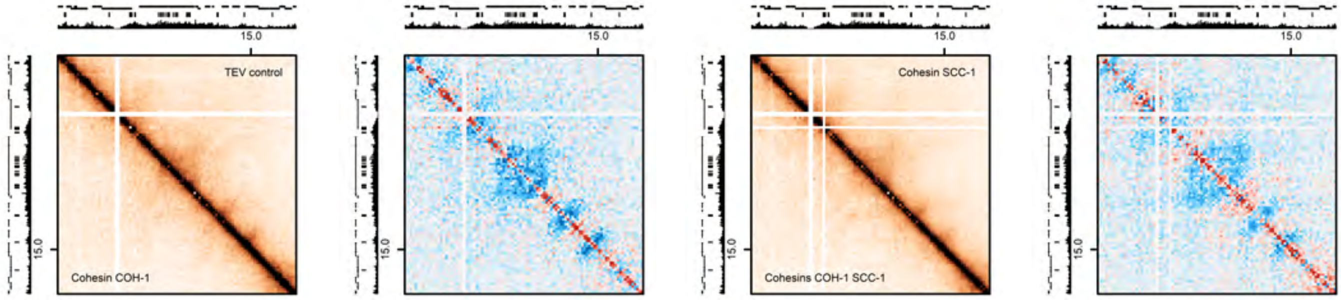

chrV:11723001-11923000

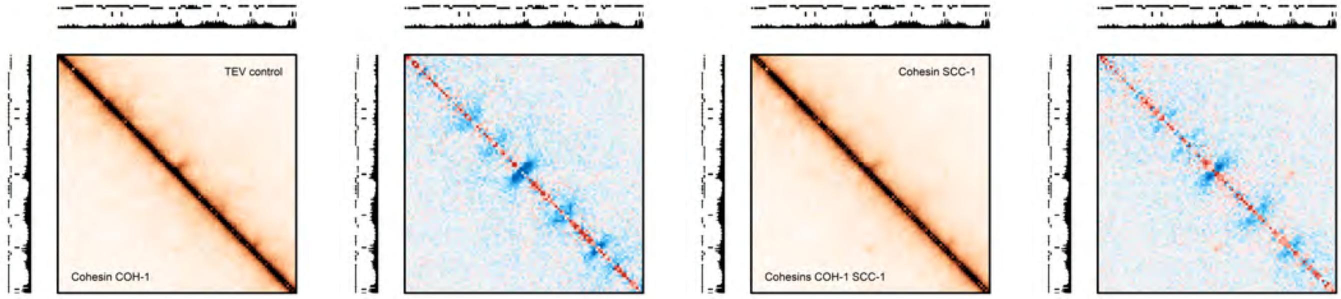

chrV:16255001-16455000

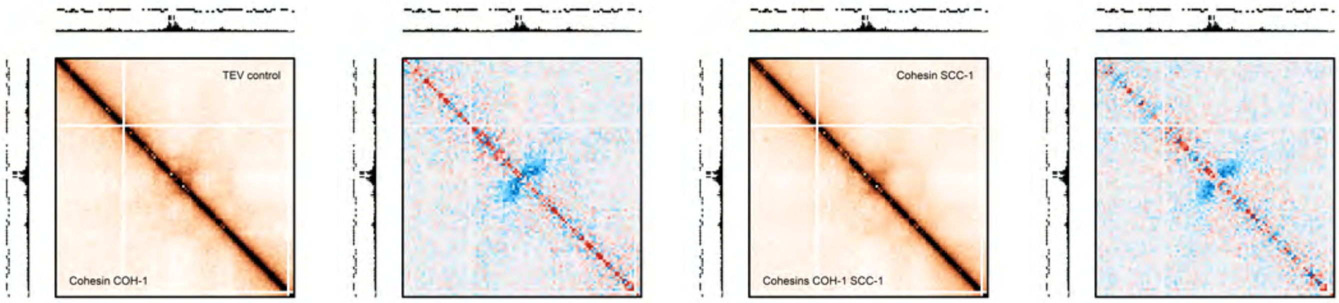

chrI:4815001-5015000

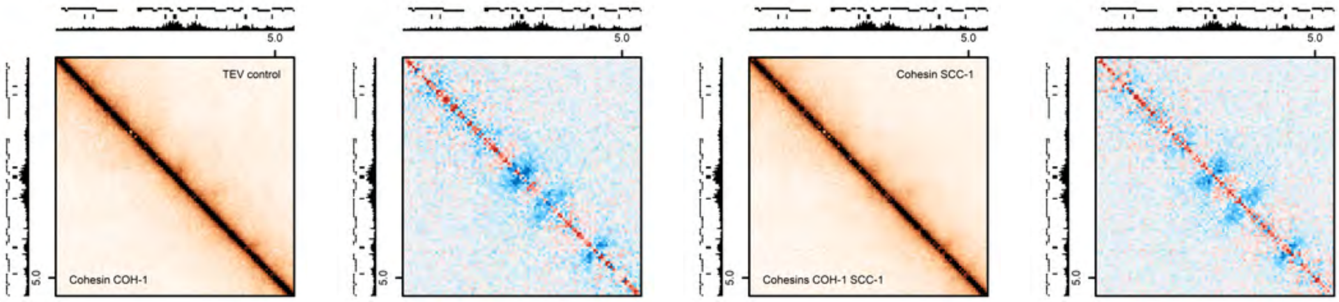

chrII:13637001-13837000

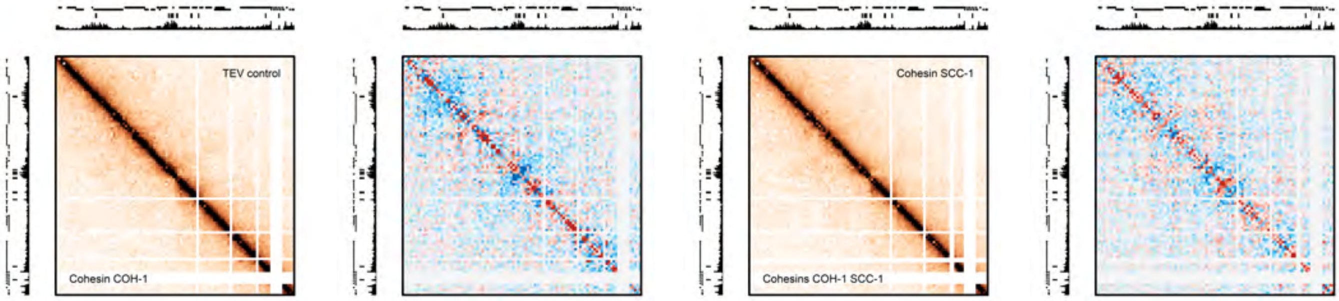

chrIV:9573001-9773000

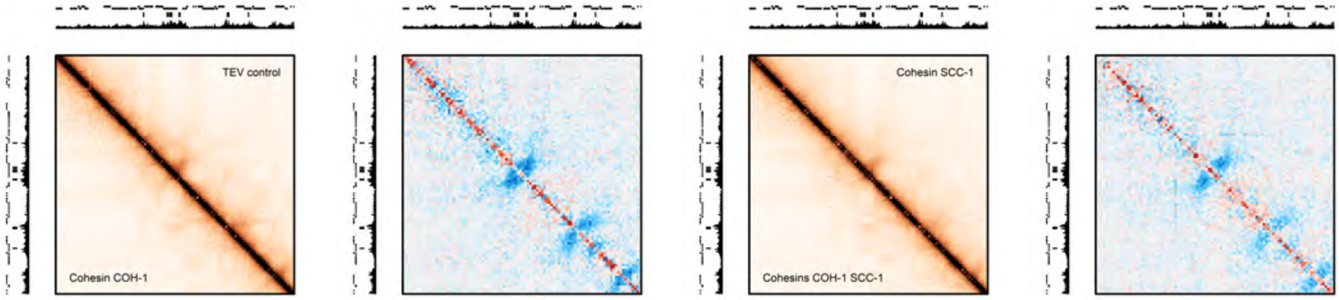

chrX:6643001-6843000

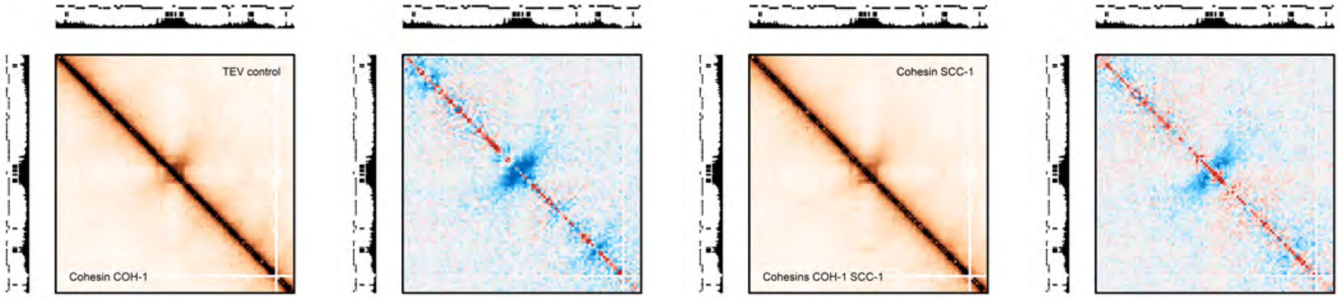

chrX:989001-1189000

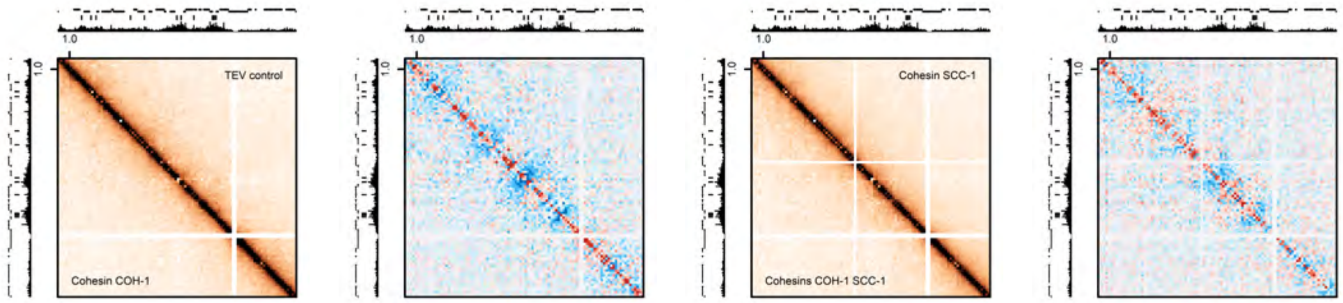

chrIII:13619001-13819000

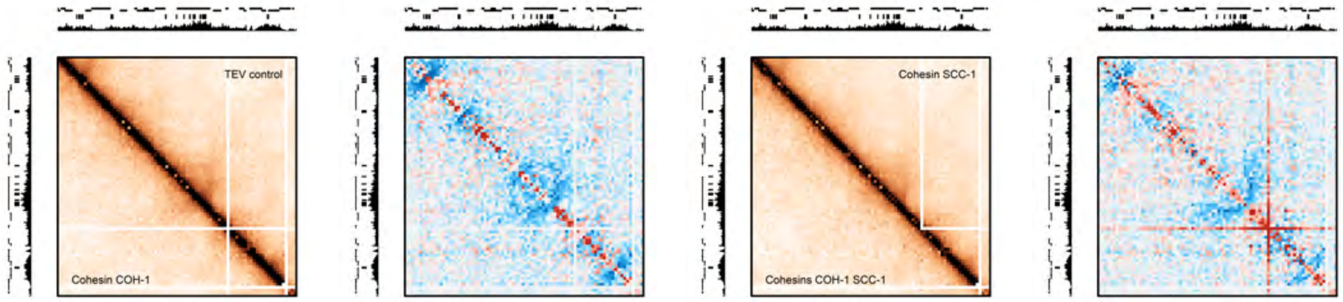

chrIV:6683001-6883000

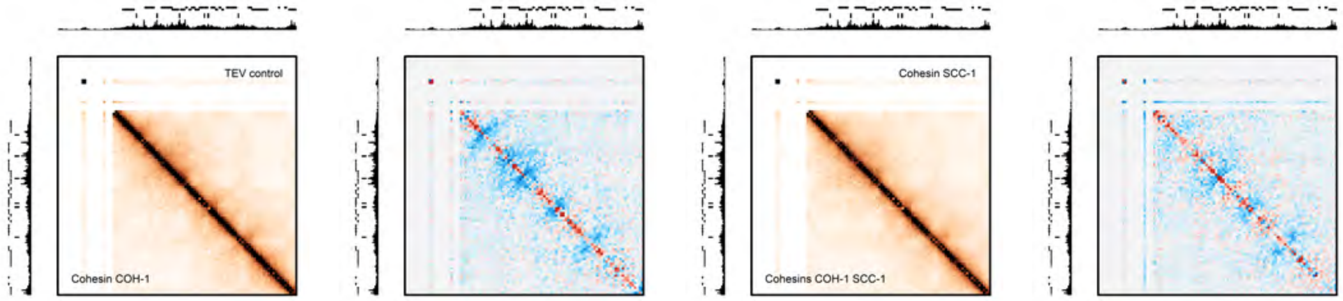

chrIV:11495001-11695000

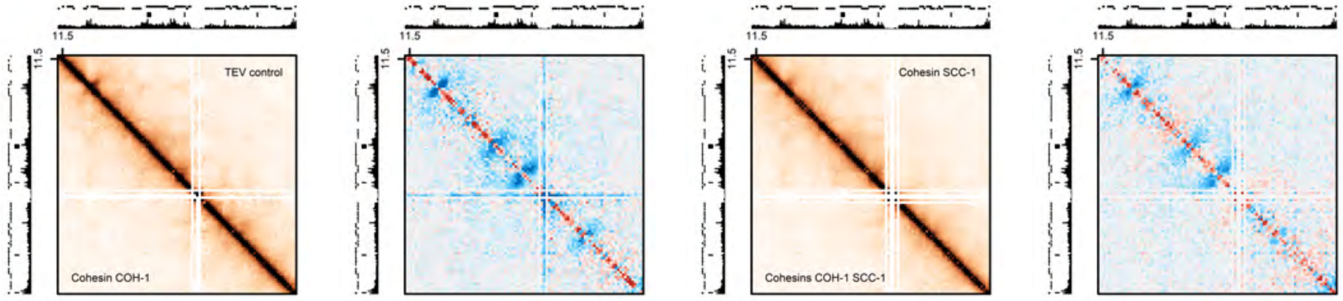

chrII:6245001-6445000

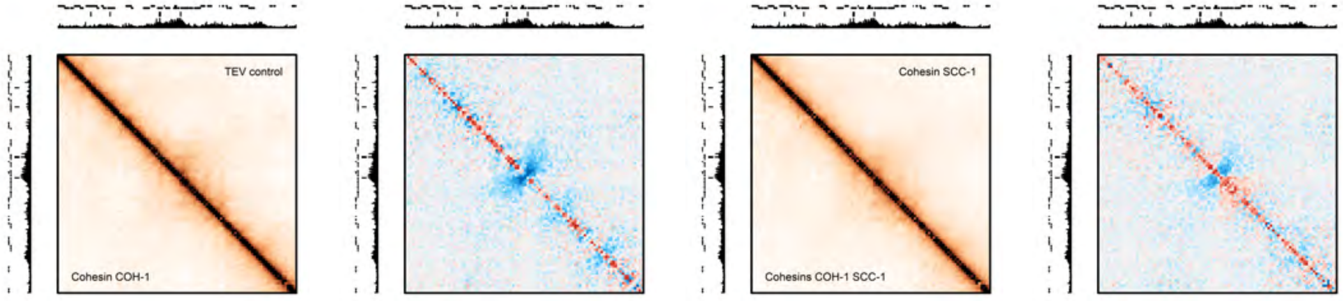

chrV:20317001-20517000

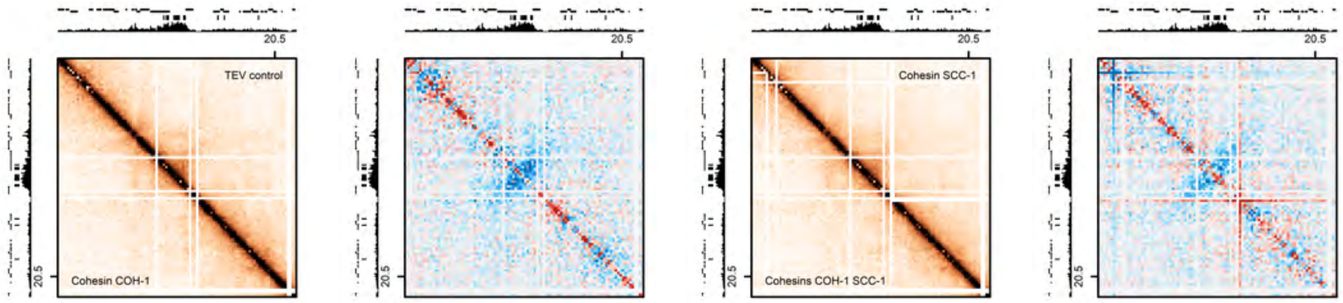

chrX:14721001-14921000

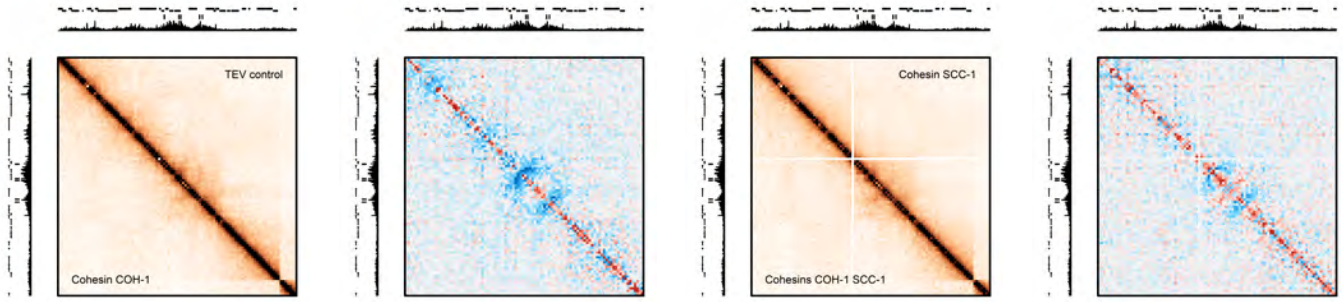

chrV:19425001-19625000

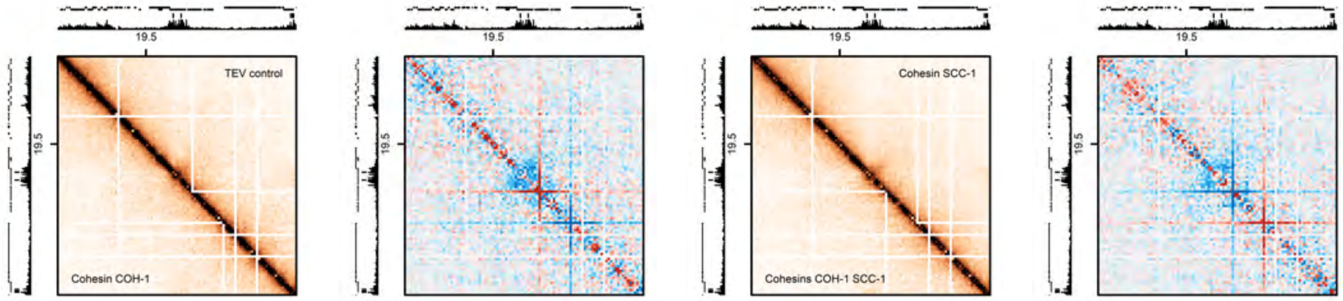

chrIV:16071001-16271000

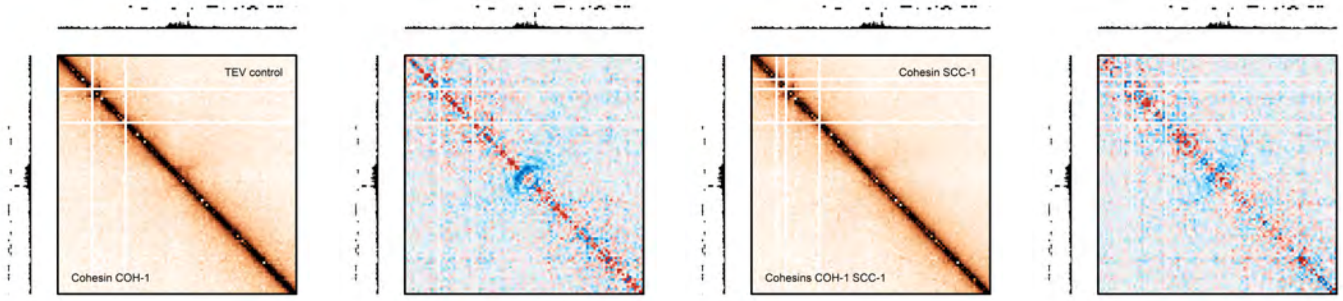

chrIV:12739001-12939000

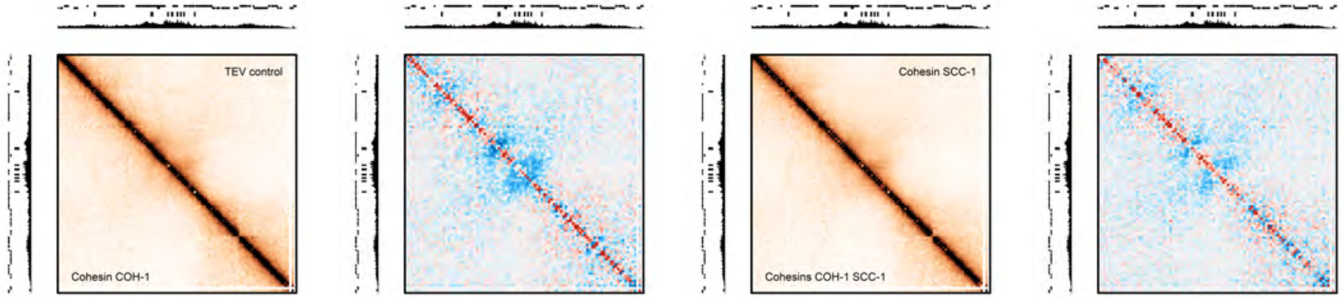

chrI:2761001-2961000

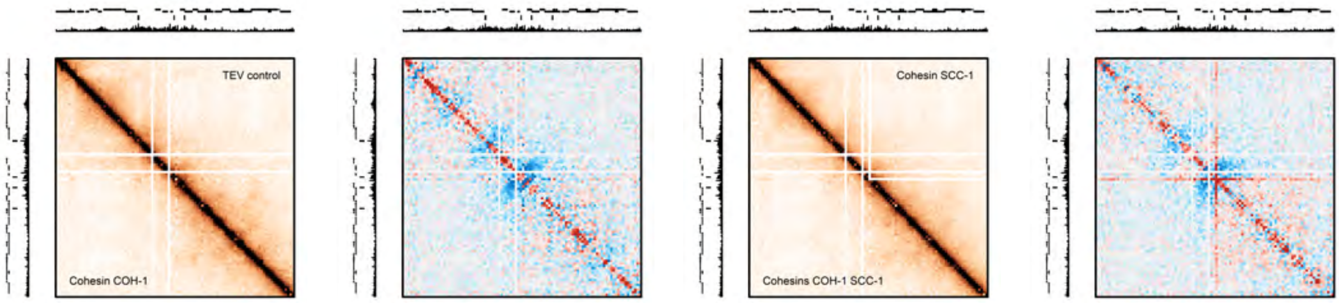

chrX:12809001-13009000

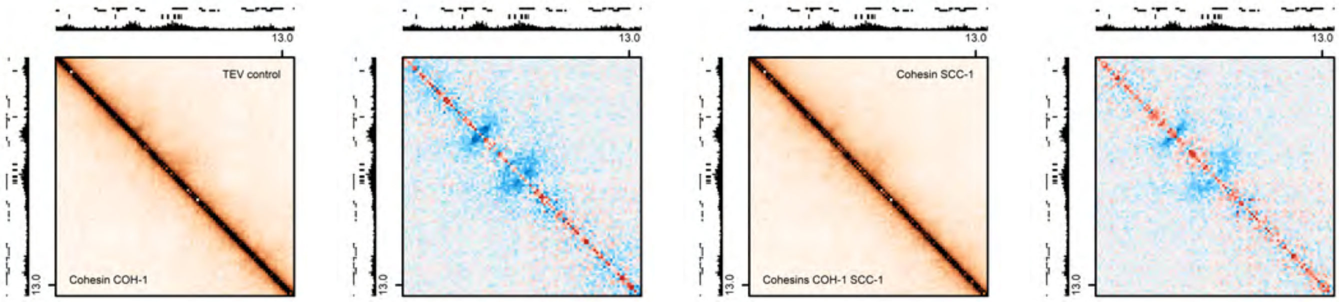

chrIII:4915001-5115000

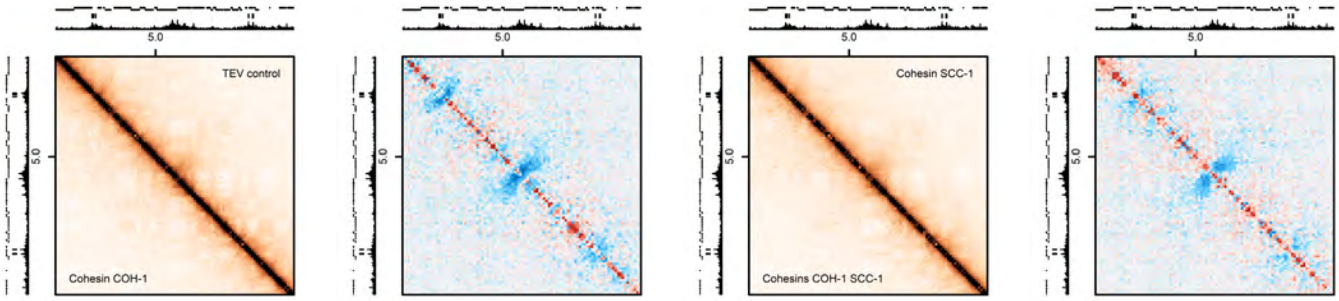

chrV:6781001-6981000

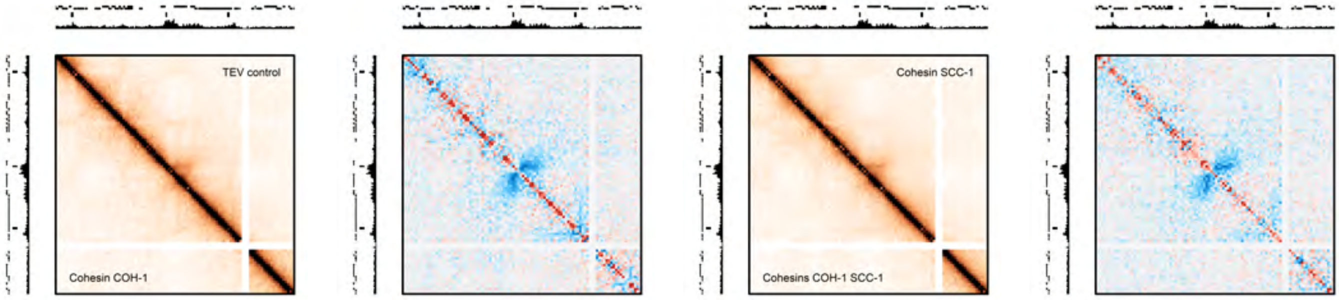

chrX:9451001-9651000

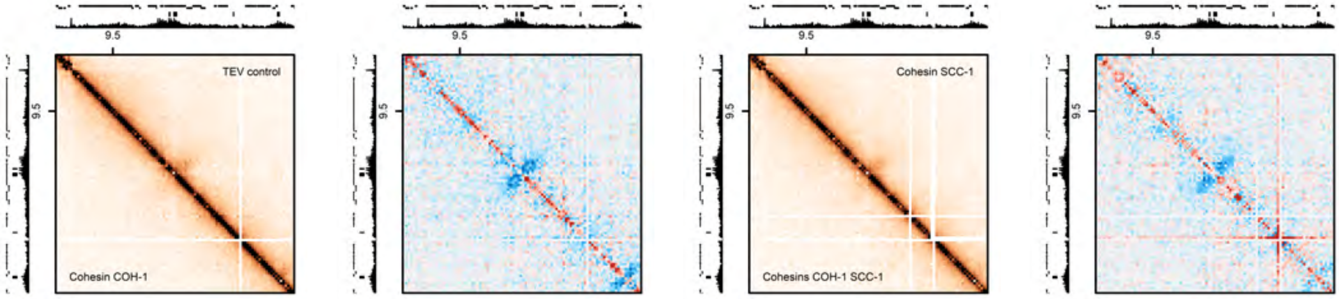

chrIII:4181001-4381000

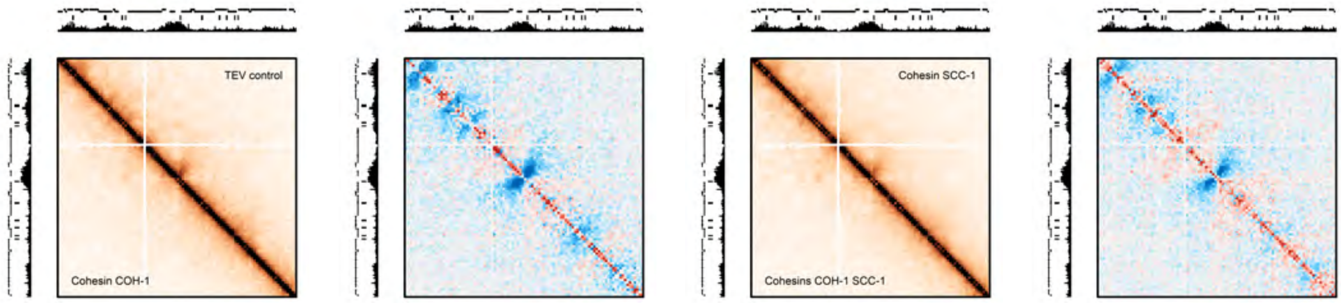

chrV:629001-829000

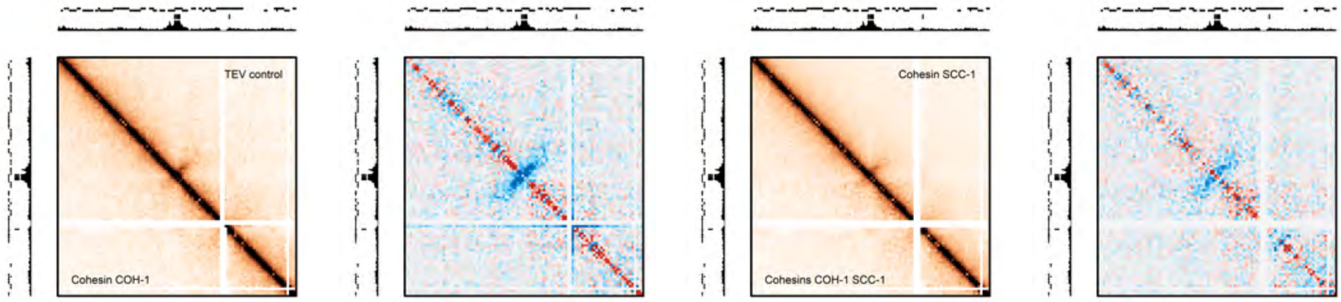

chrX:15509001-15709000

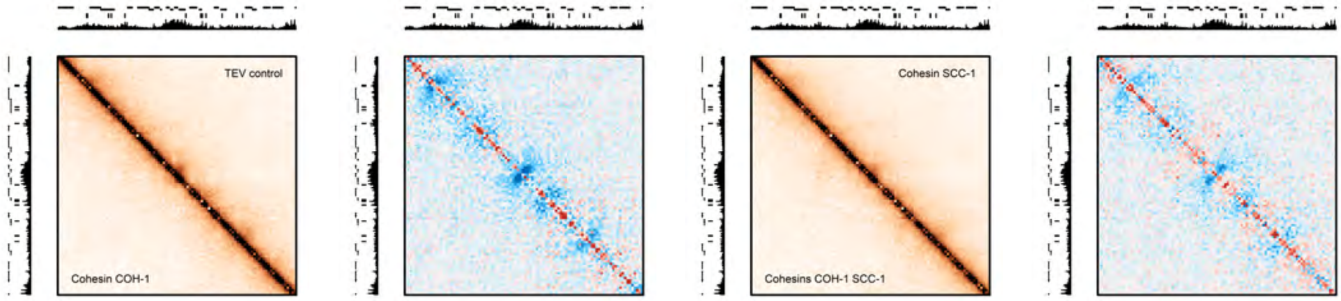

chrIV:15605001-15805000

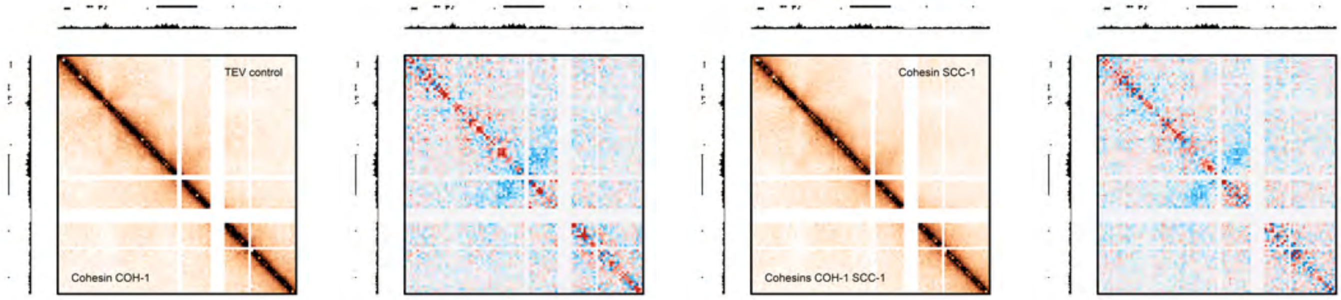

chrII:11647001-11847000

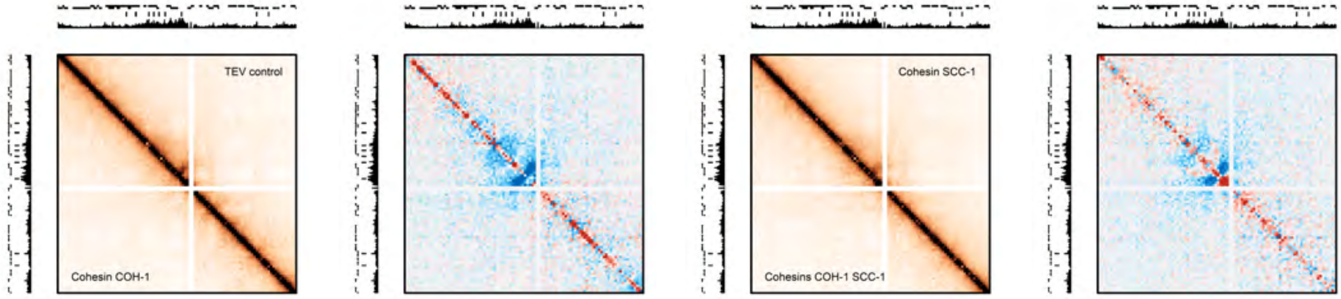

chrII:14717001-14917000

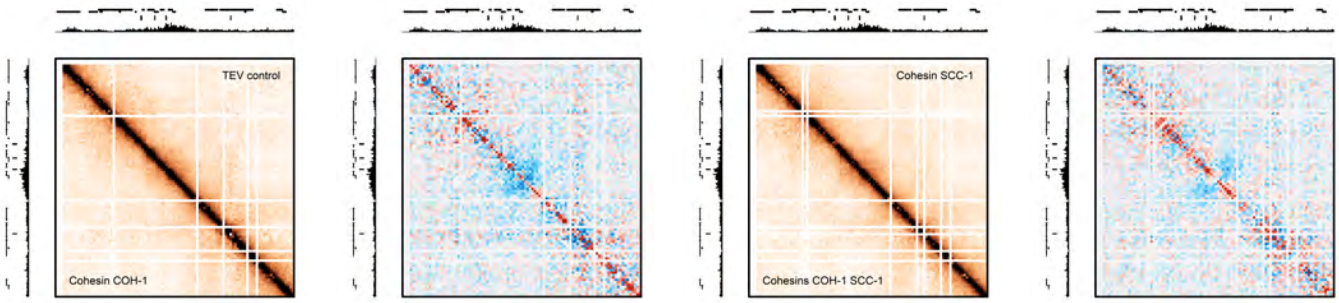

chrV:9941001-10141000

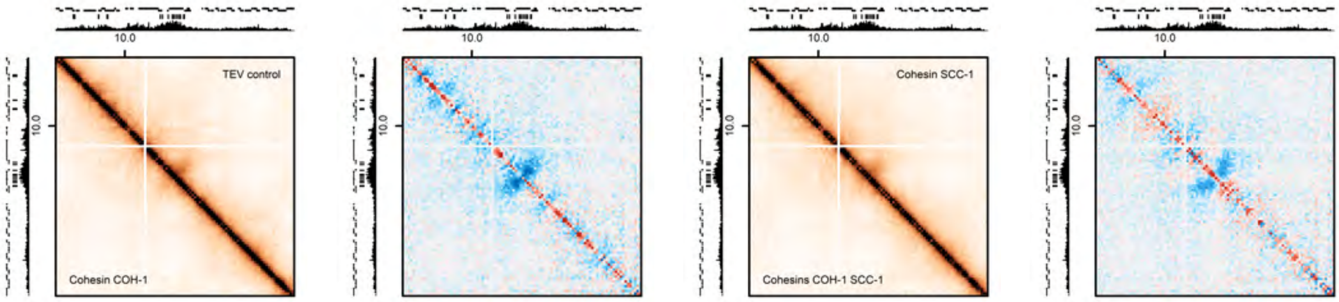

chrX:7135001-7335000

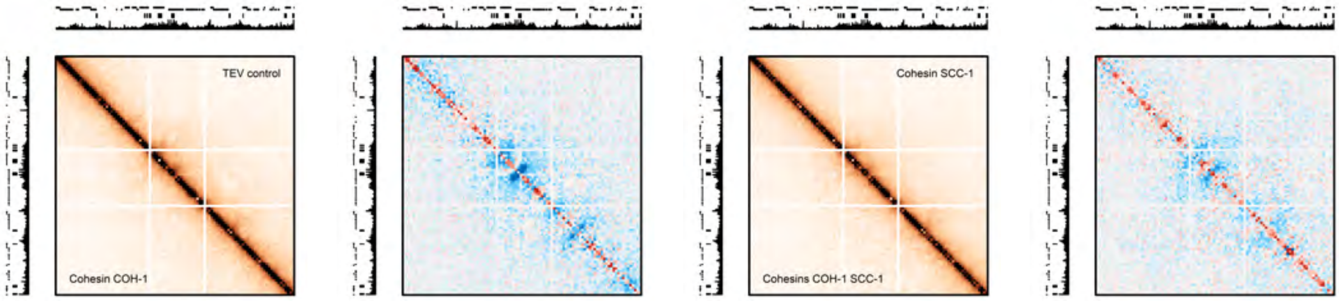

chrI:11101001-11301000

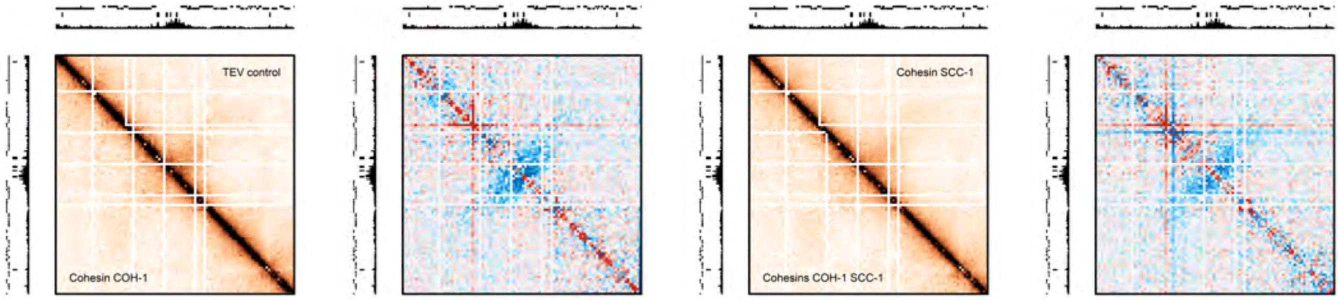

chrIV:3549001-3749000

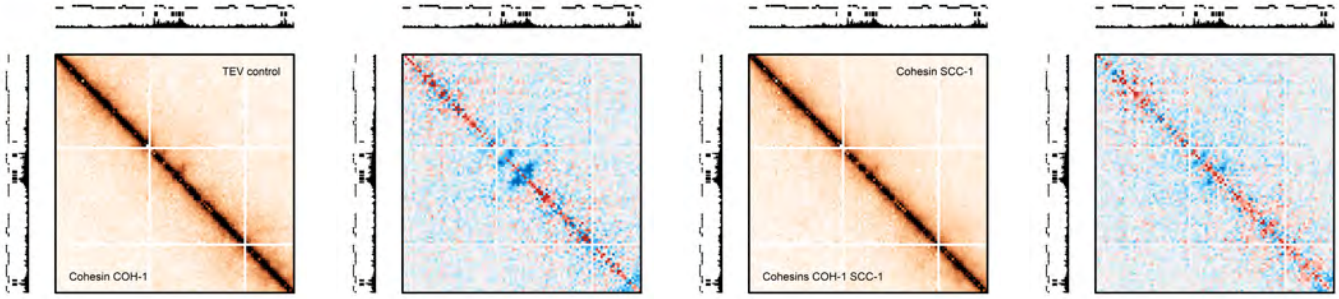

chrIV:9099001-9299000

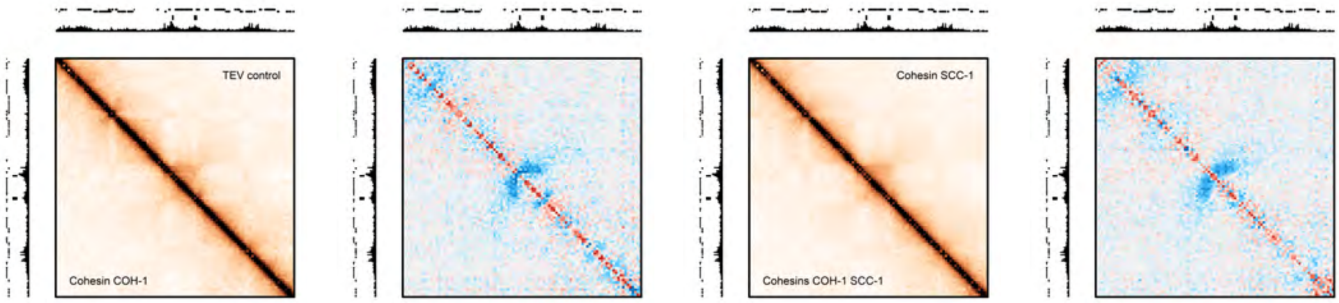

chrIV:13219001-13419000

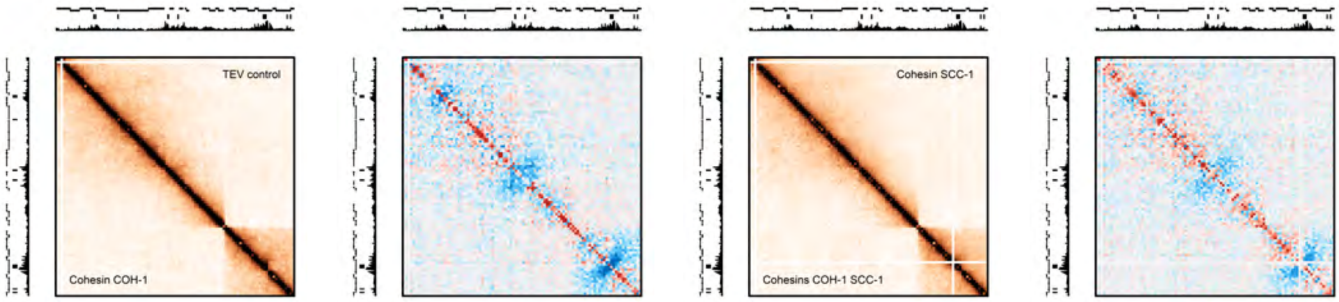

chrI:11571001-11771000

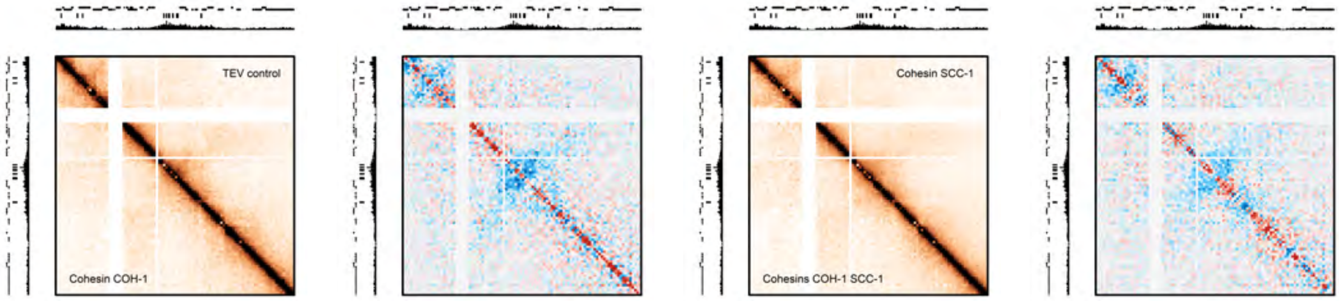

chrV:12437001-12637000

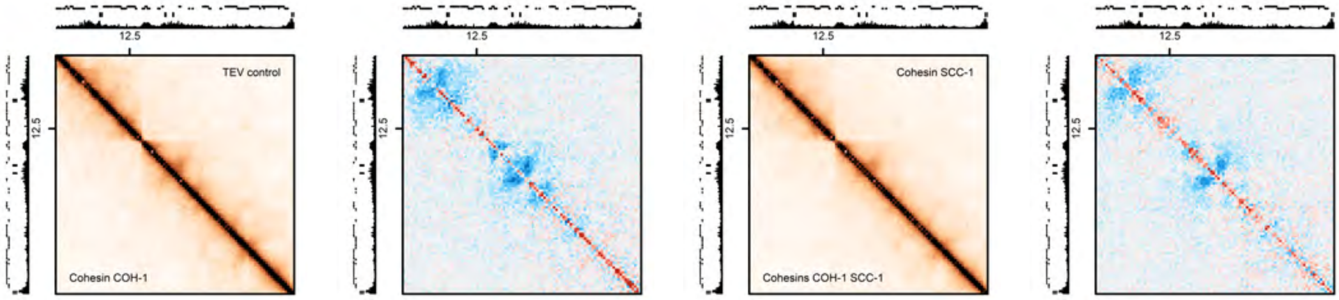

chrV:5903001-6103000

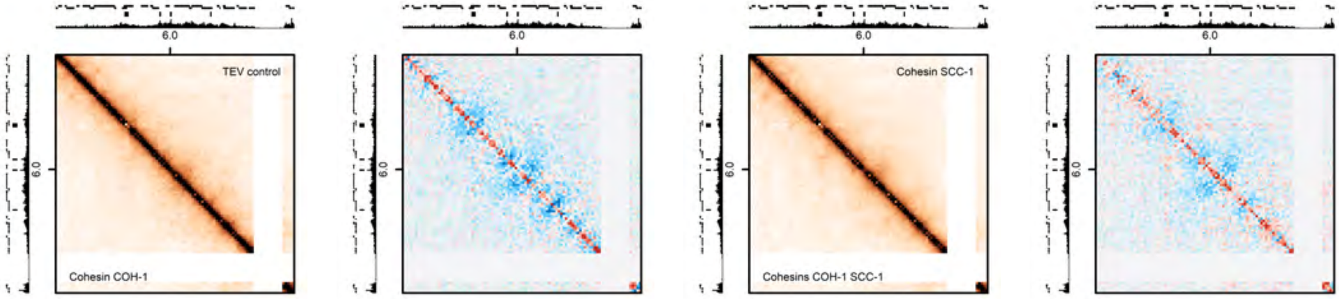

chrX:3725001-3925000

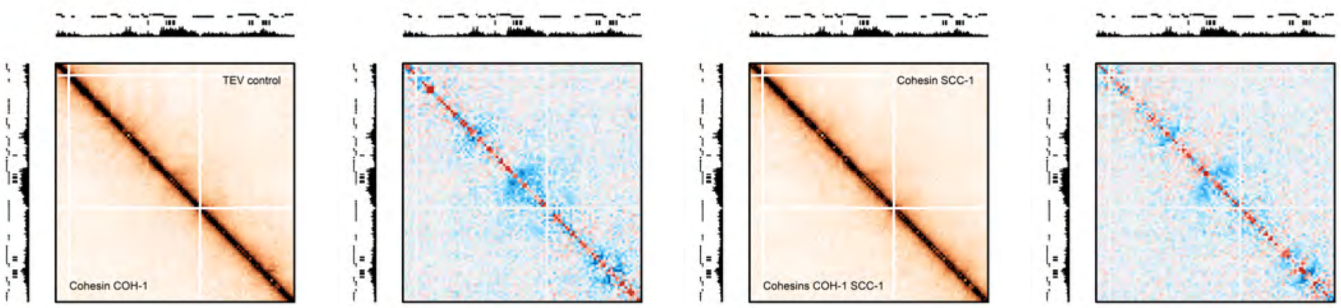

chrII:2355001-2555000

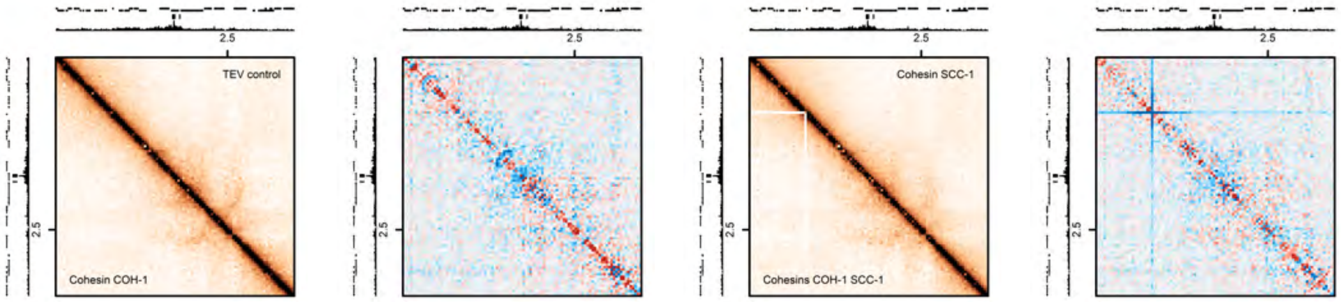

chrII:12123001-12323000

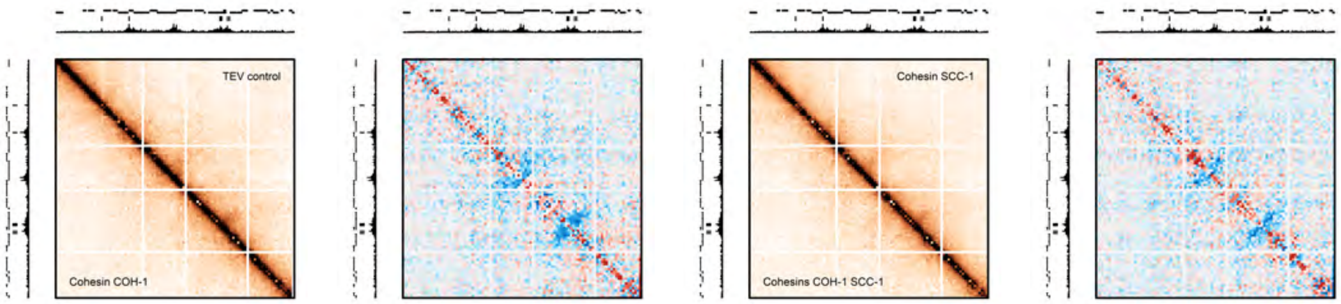

chrII:-78999-121000

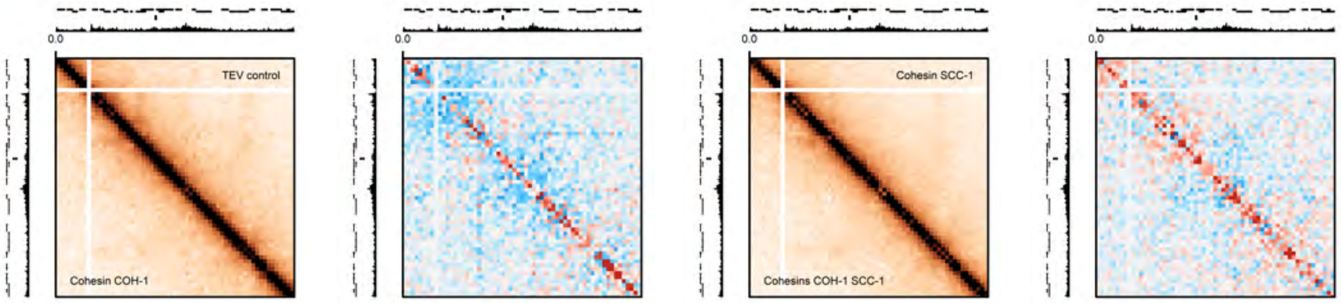

chrIII:11733001-11933000

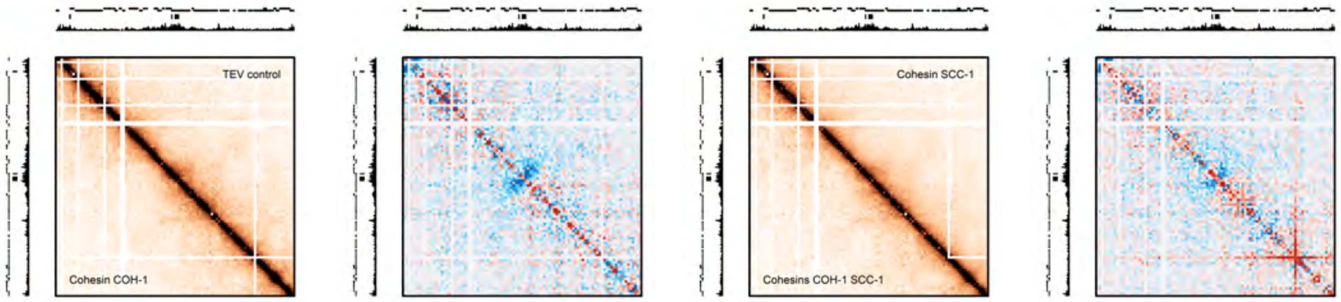

chrIV:3133001-3333000

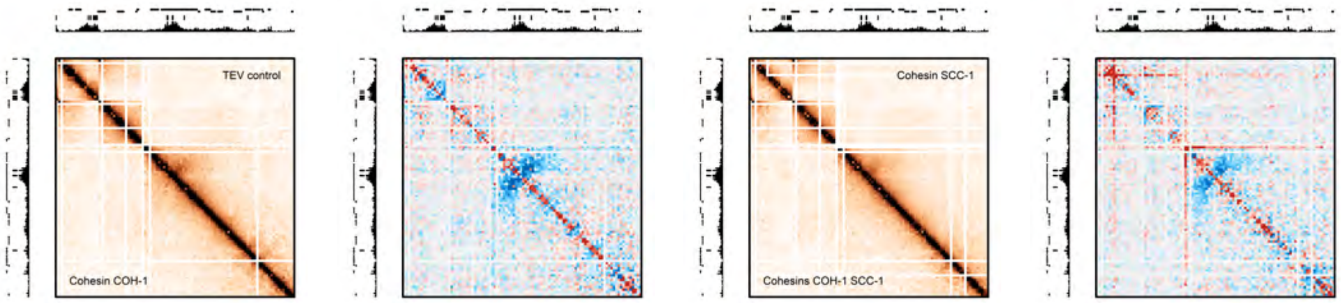

chrIV:5035001-5235000

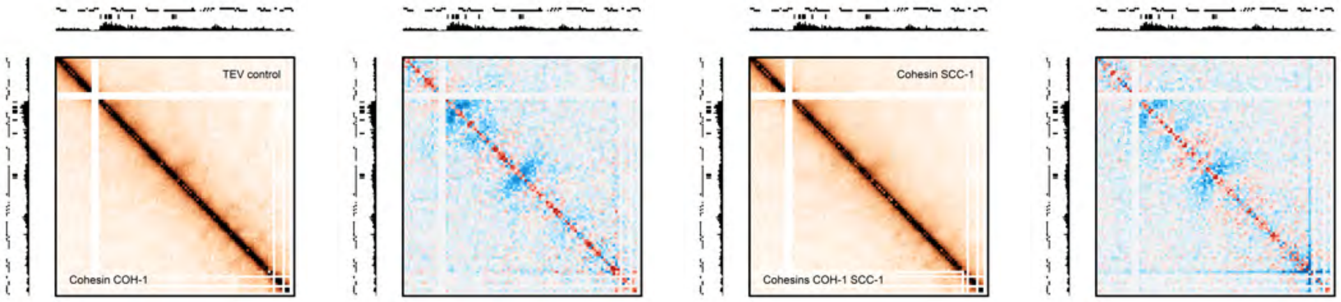

chrI:1203001-1403000

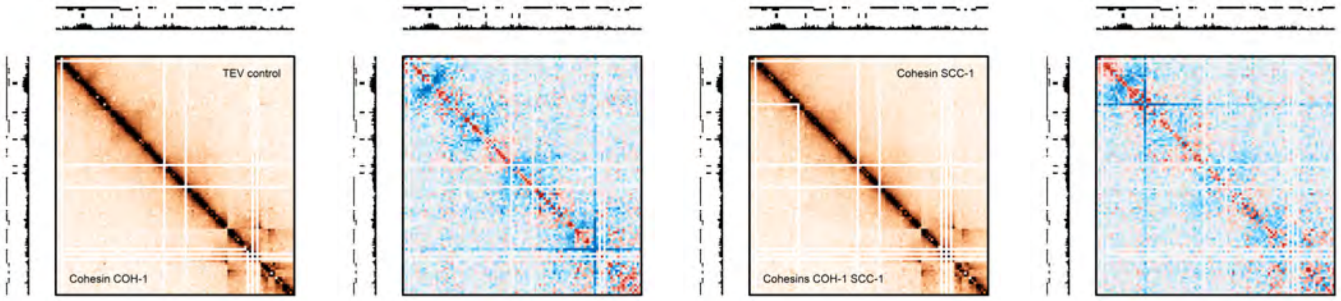

chrI:9999001-10199000

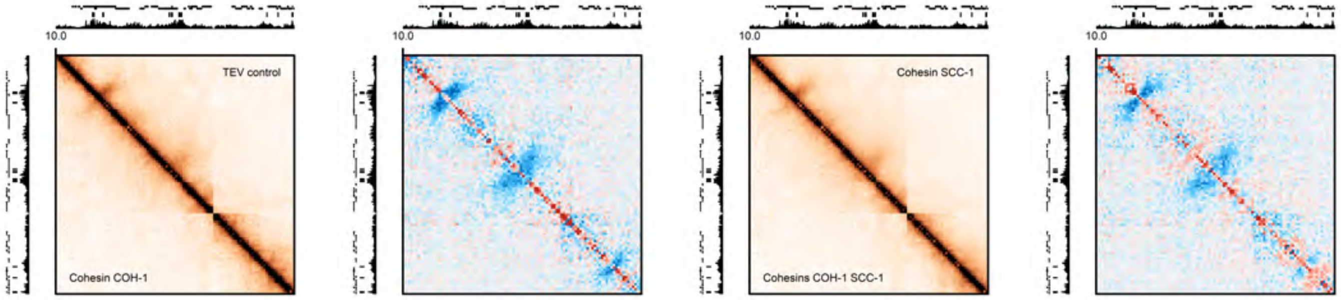

chrI:10993001-11193000

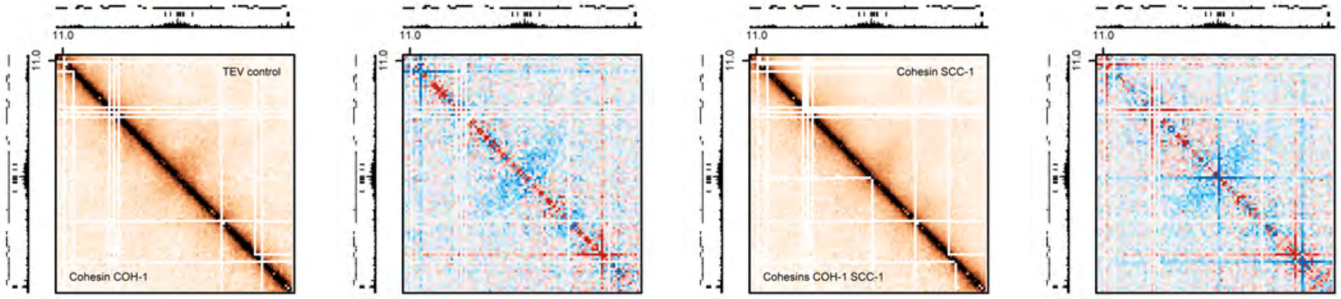

chrIV:-16999-183000

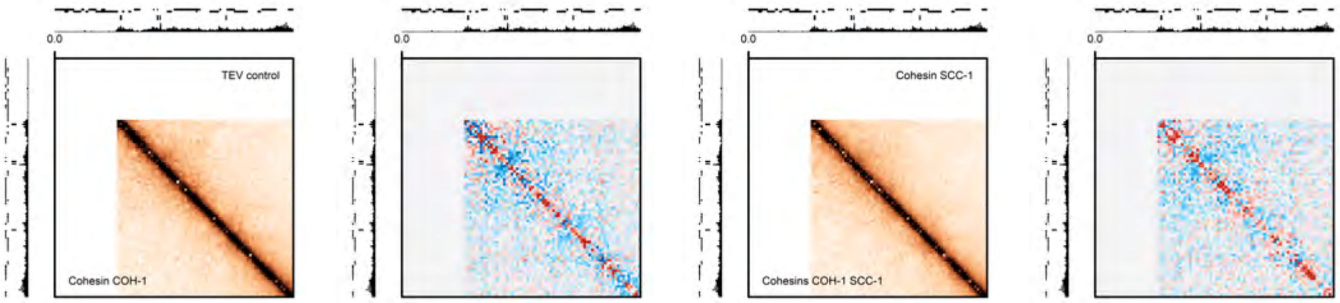

chrI:4599001-4799000

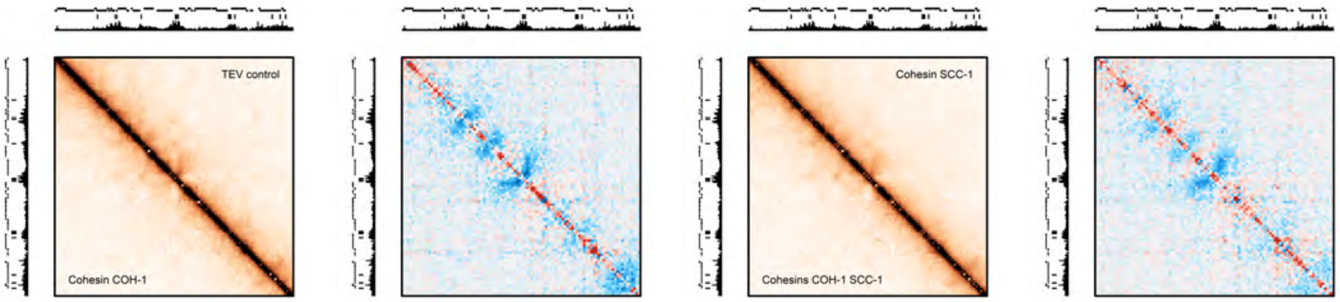

chrX:3015001-3215000

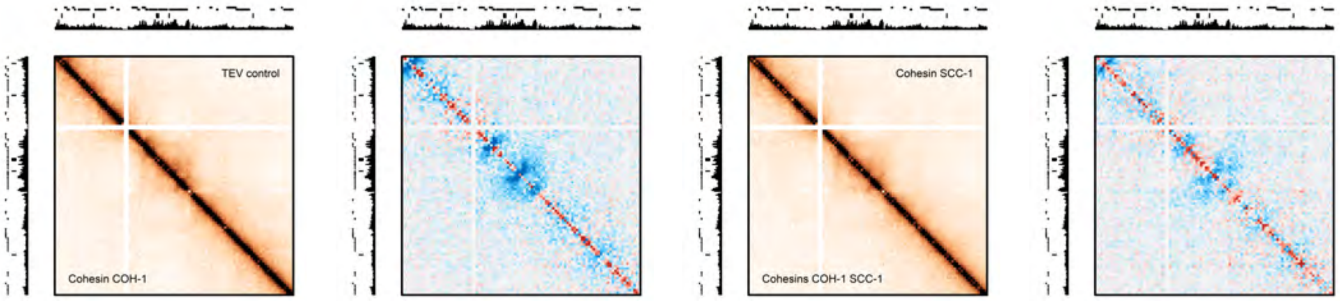

chrI:12473001-12673000

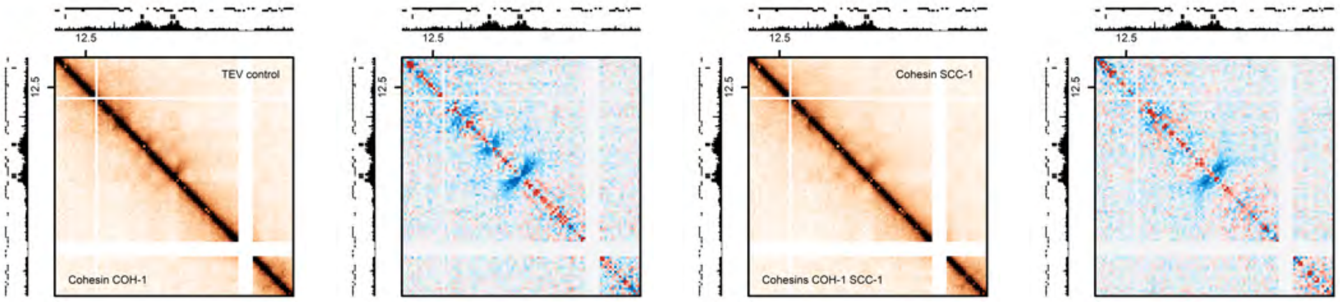

chrX:17419001-17619000

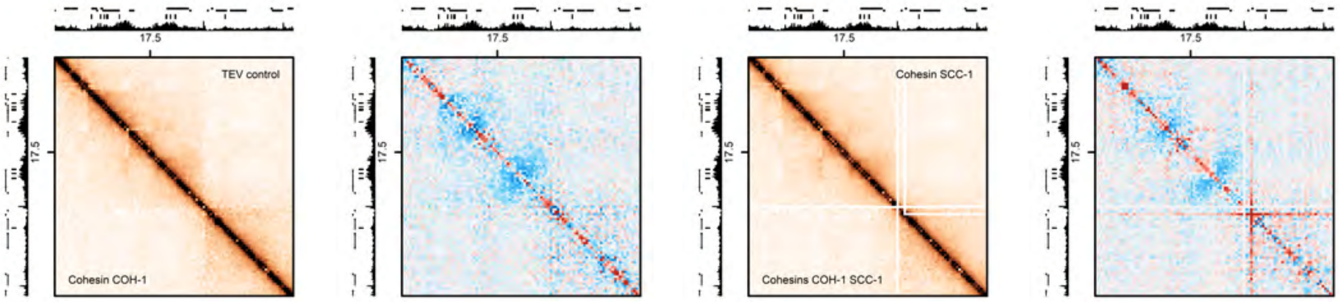

chrII:8029001-8229000

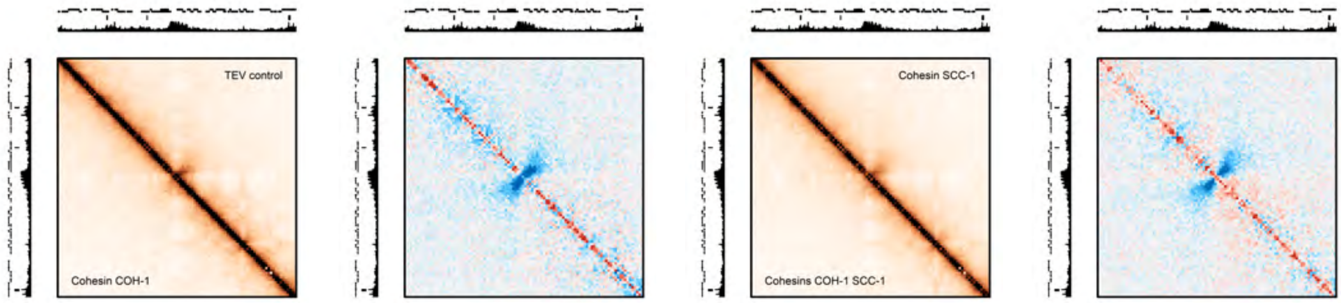

chrIV:1759001-1959000

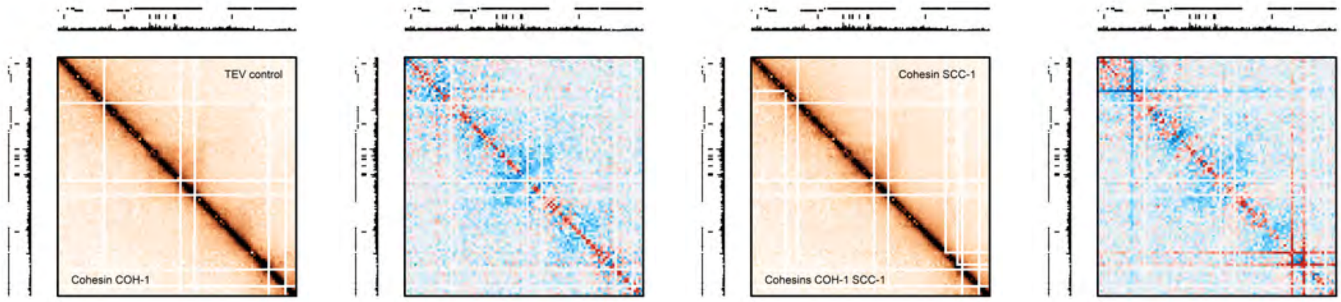

chrIV:4113001-4313000

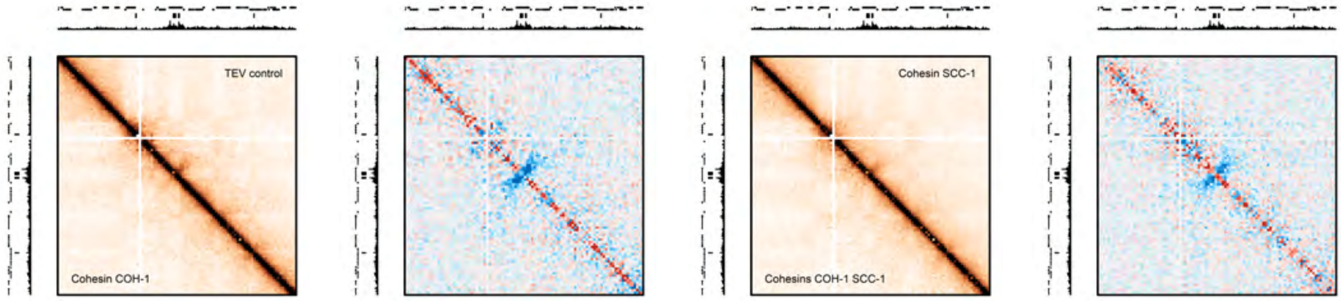

chrIV:5875001-6075000

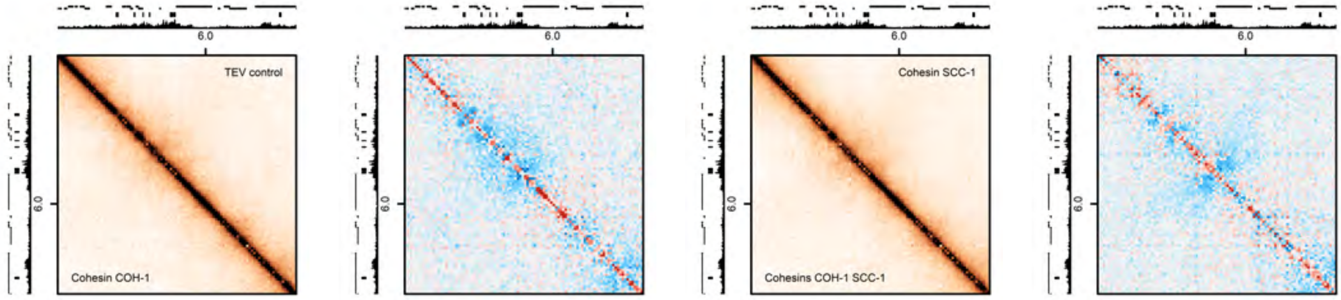

chrV:8687001-8887000

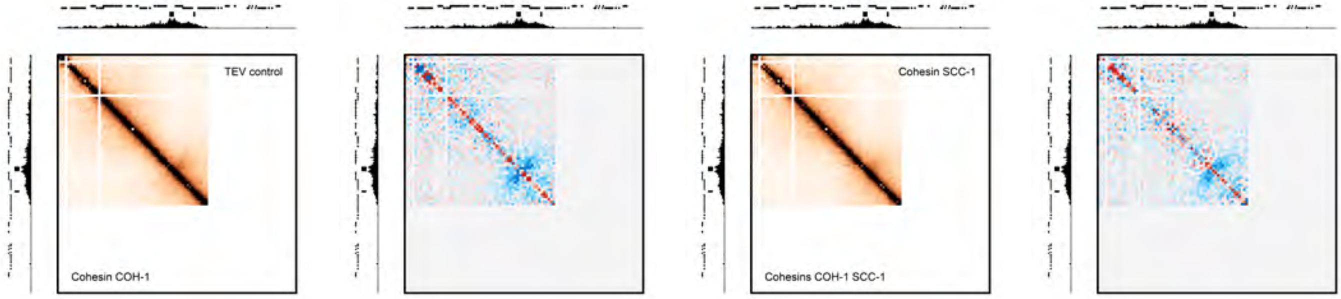

chrV:11433001-11633000

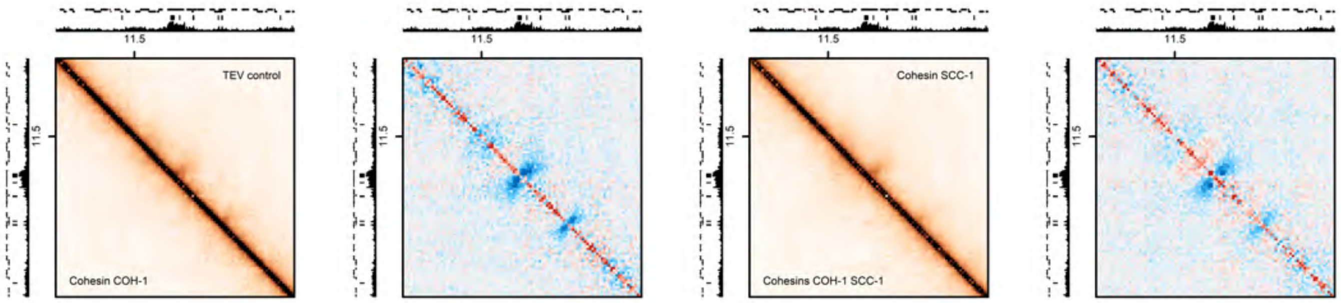

chrV:13557001-13757000

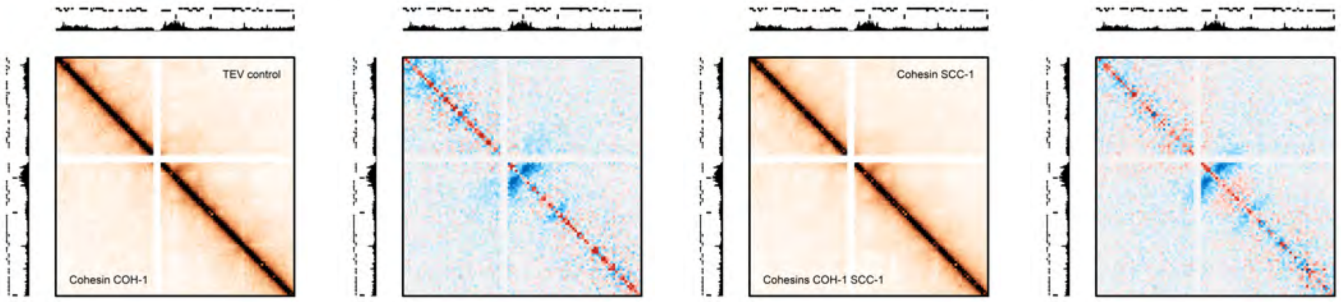

chrI:9757001-9957000

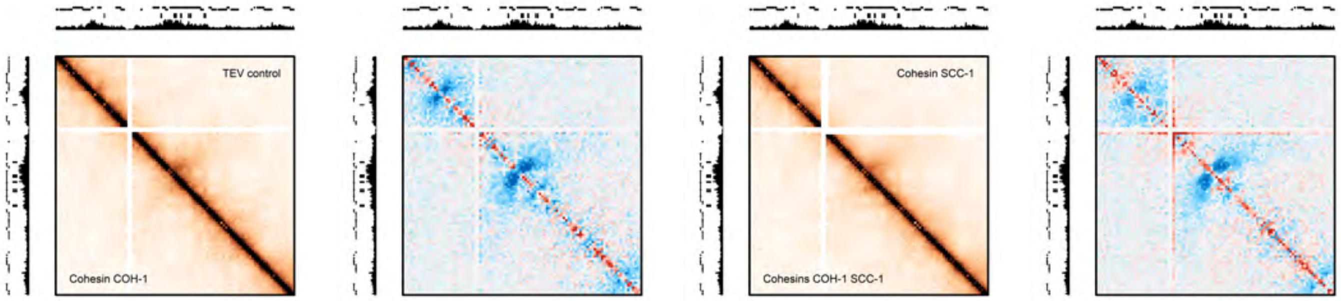

chrV:13689001-13889000

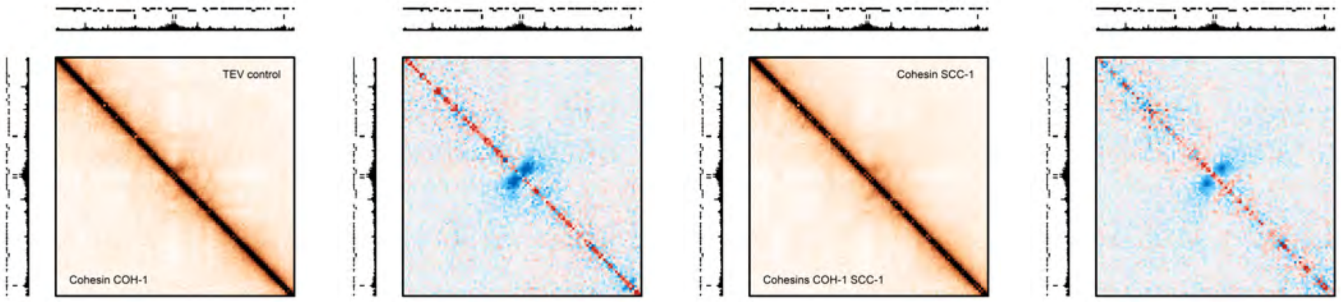

chrIV:14779001-14979000

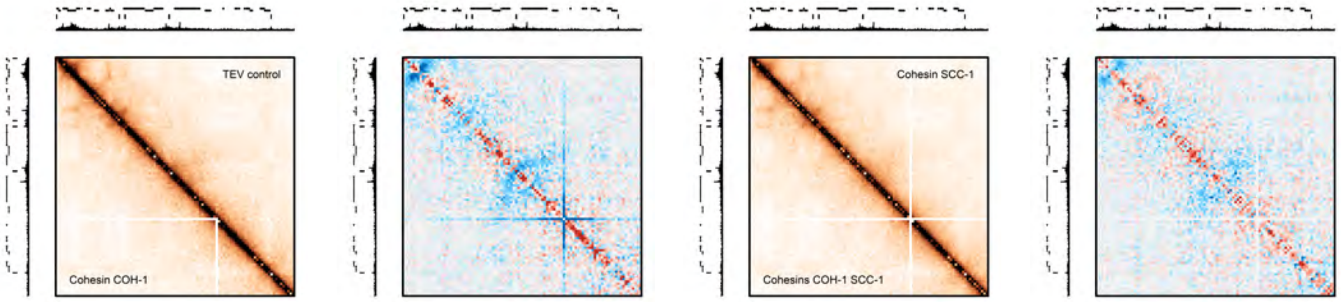

chrIII:735001-935000

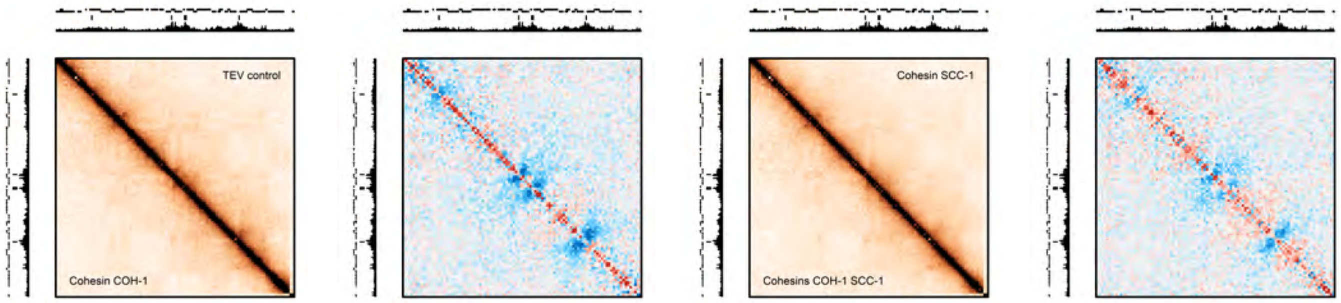

chrV:13903001-14103000

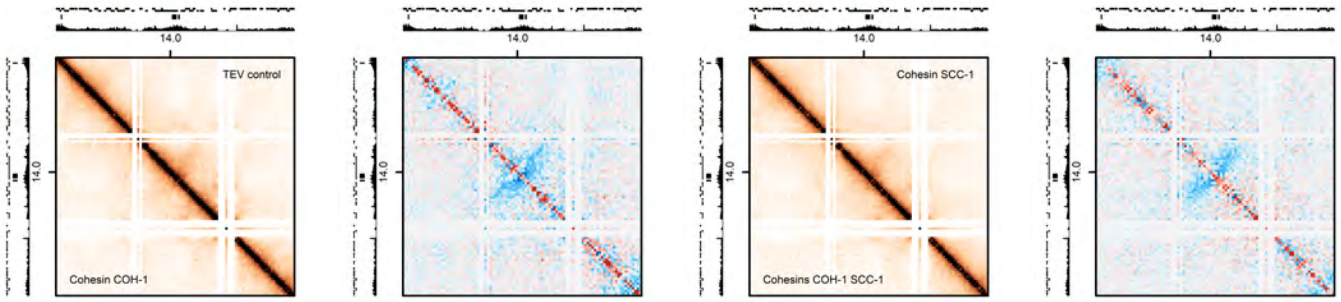

chrI:445001-645000

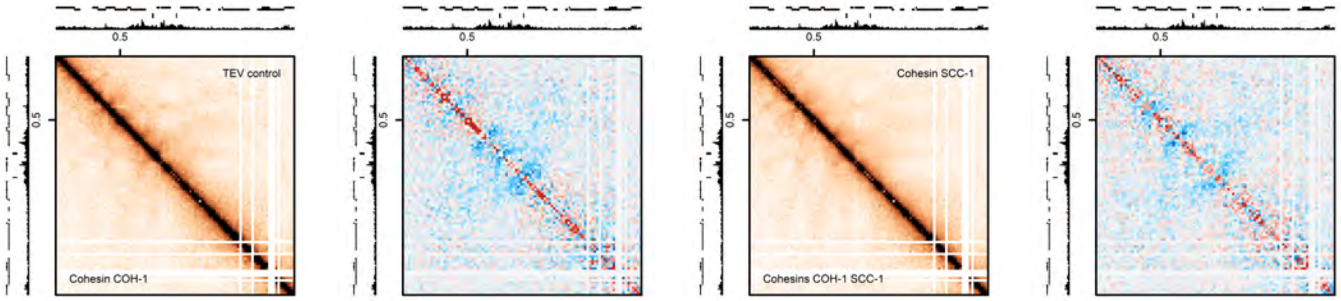

chrIV:13293001-13493000

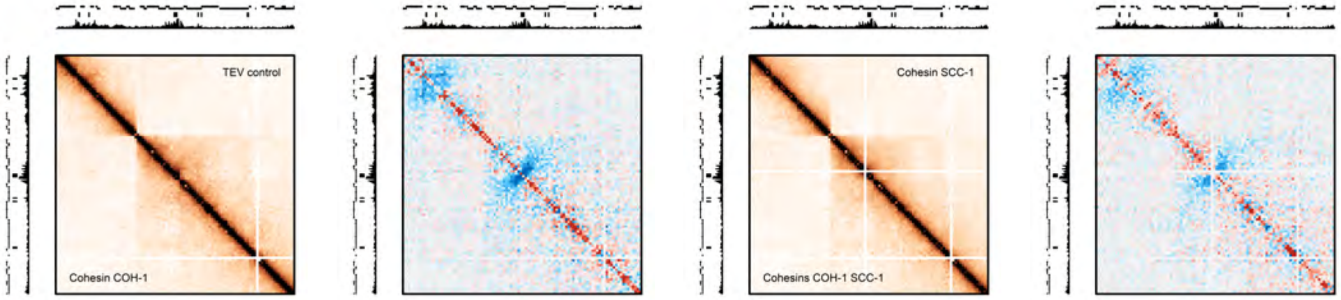

chrIV:8429001-8629000

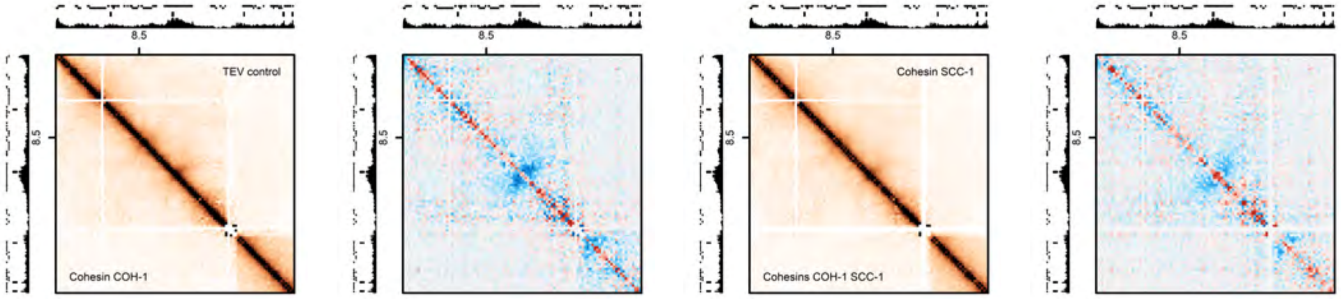

chrV:8561001-8761000

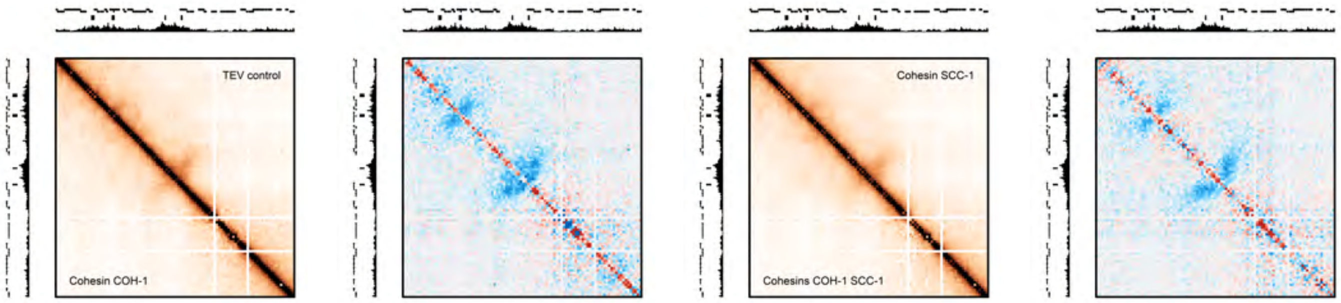

chrIII:12037001-12237000

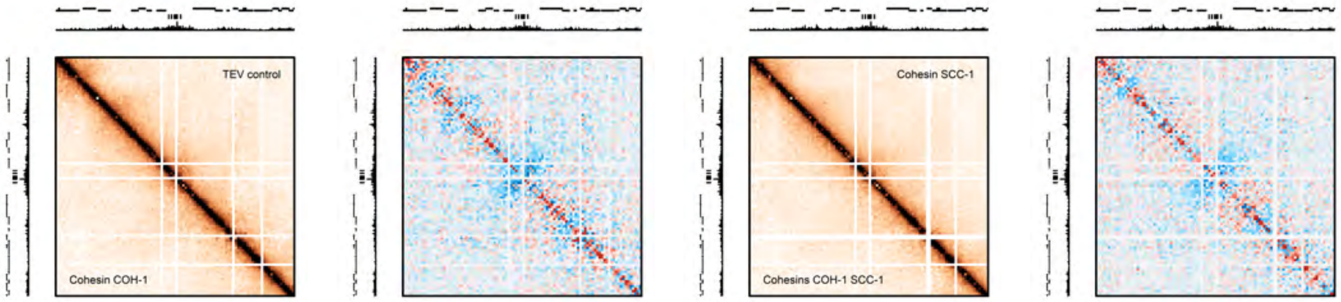

chrII:9889001-10089000

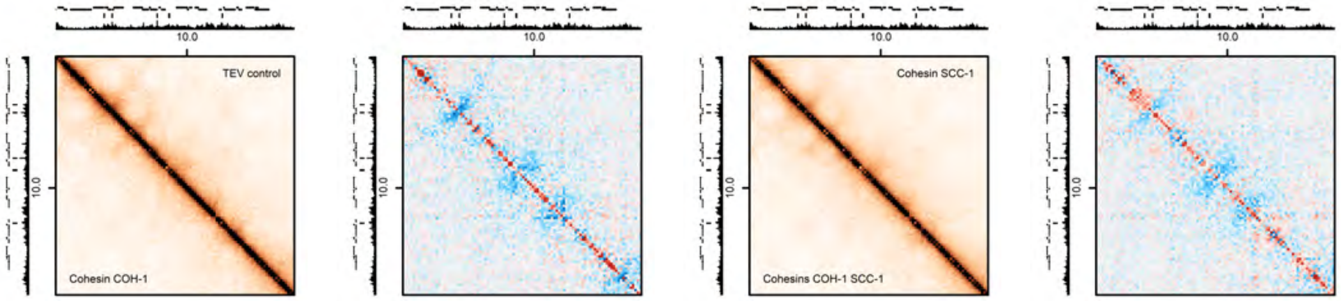

chrIII:1517001-1717000

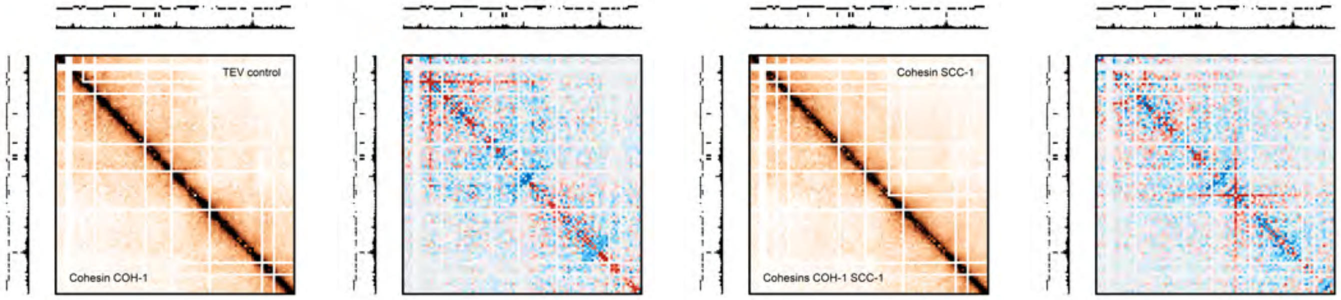

chrII:13093001-13293000

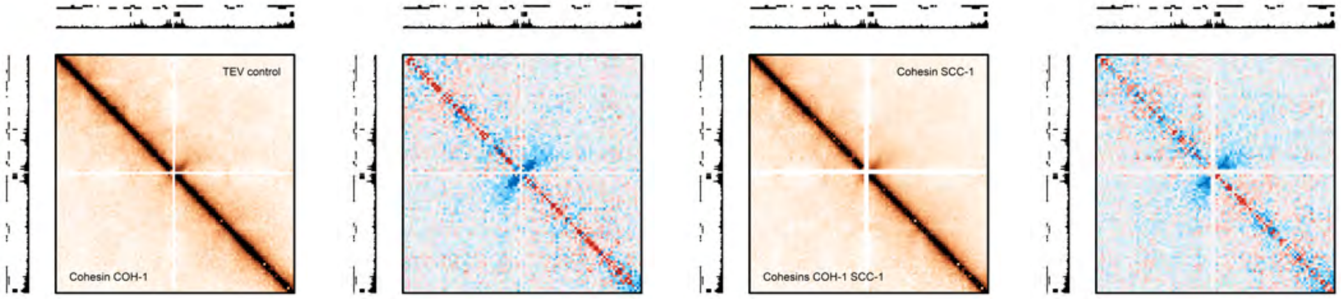

chrIII:12965001-13165000

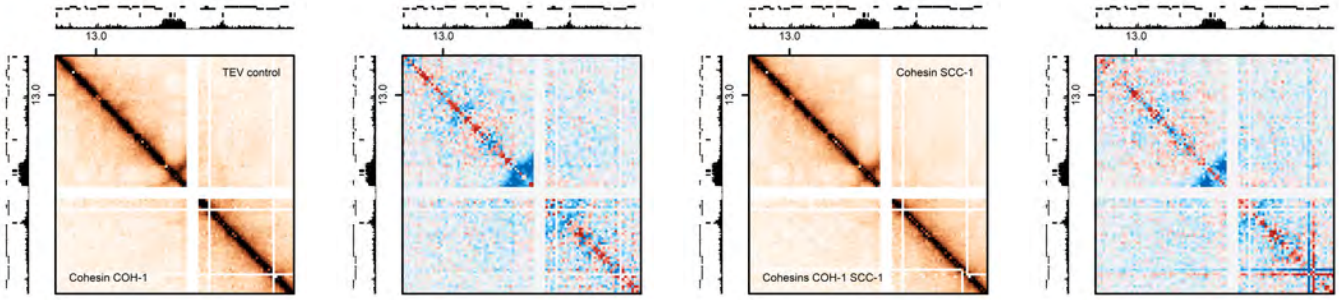

chrV:1787001-1987000

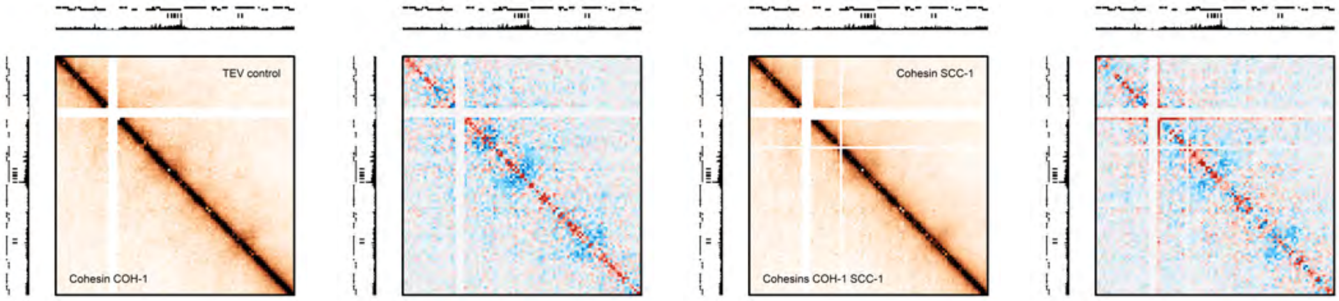

chrX:10669001-10869000

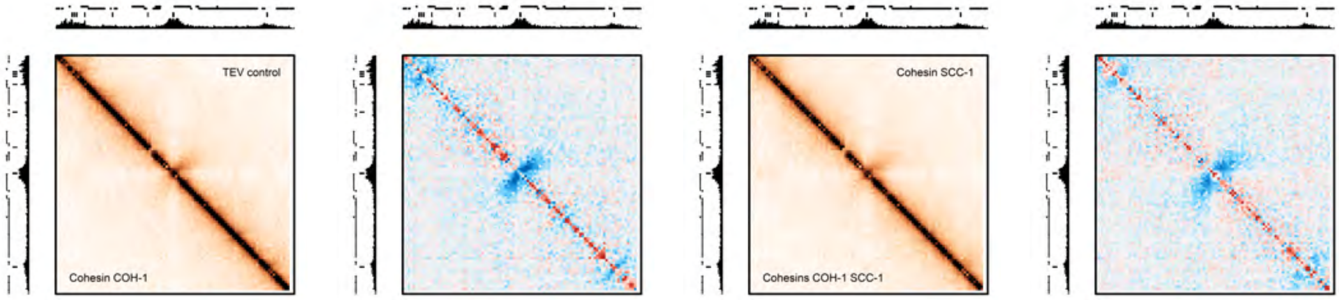

chrX:8155001-8355000

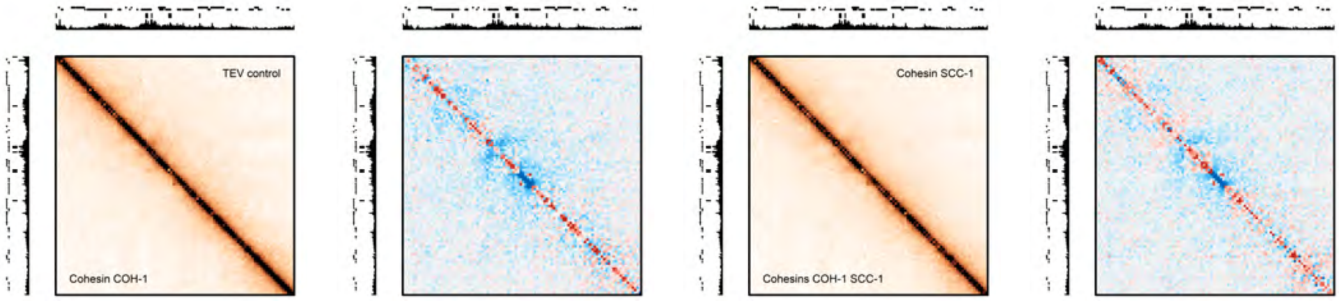

chrX:4425001-4625000

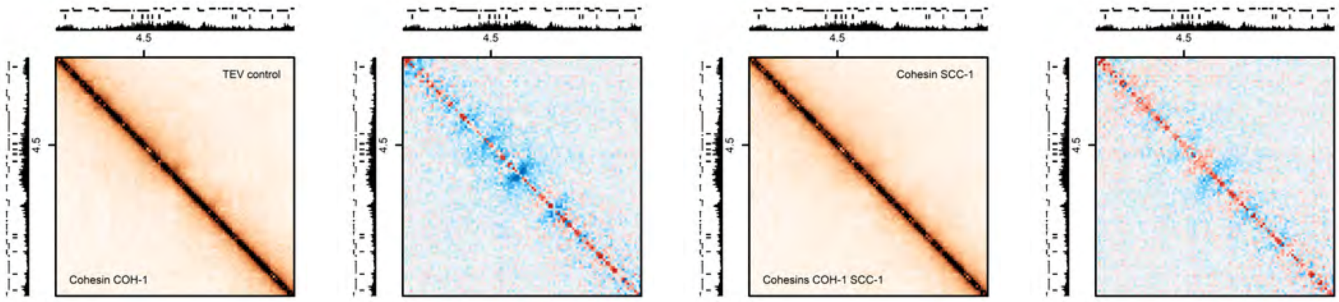

chrV:9171001-9371000

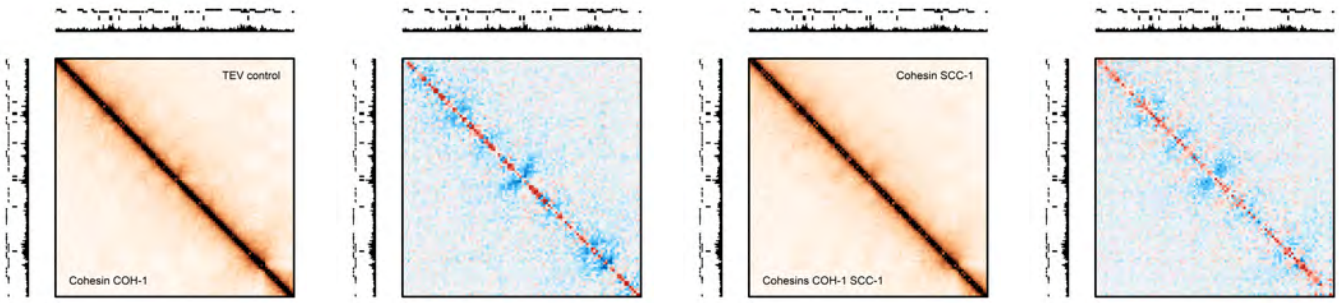

chrV:879001-1079000

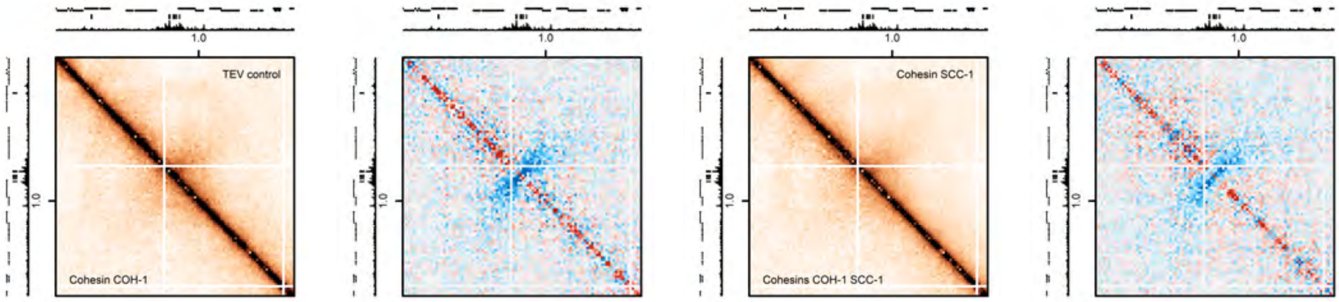

chrV:17851001-18051000

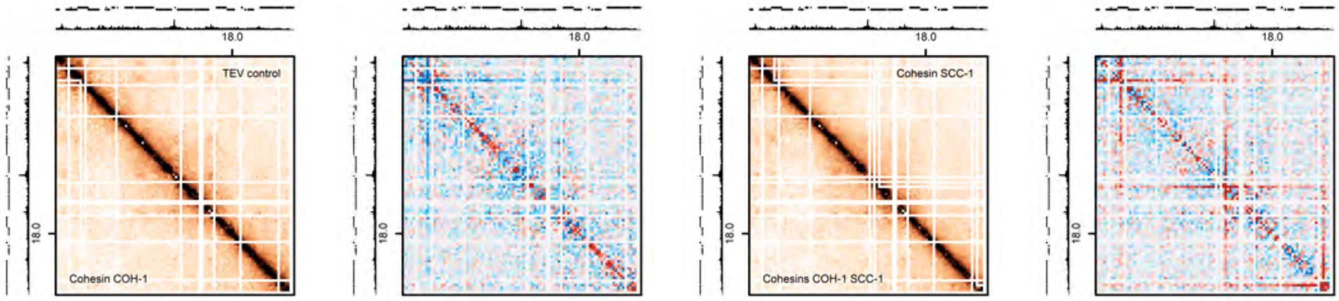

chrX:5019001-5219000

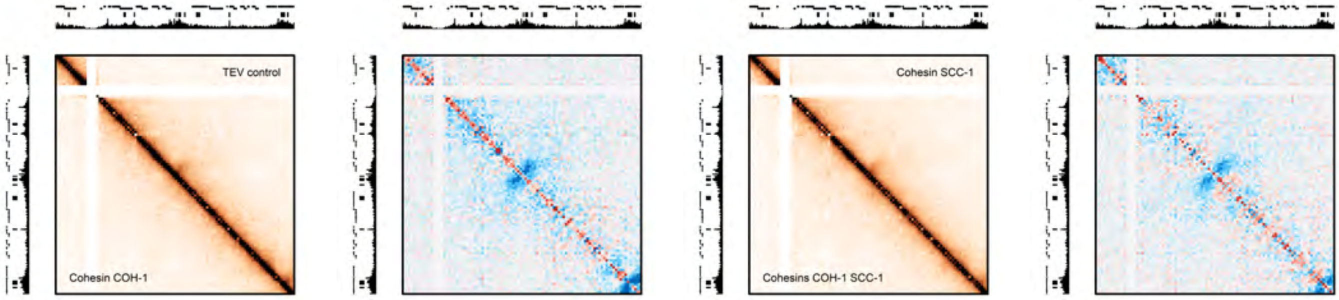

chrIII:2933001-3133000

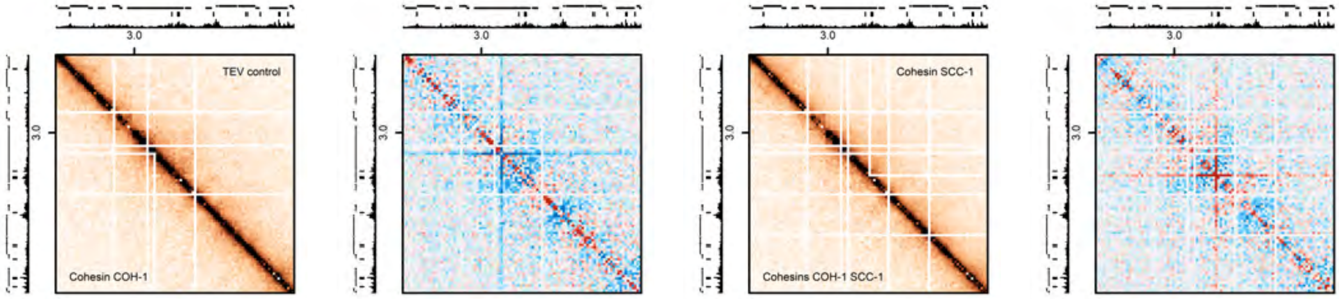

chrI:5213001-5413000

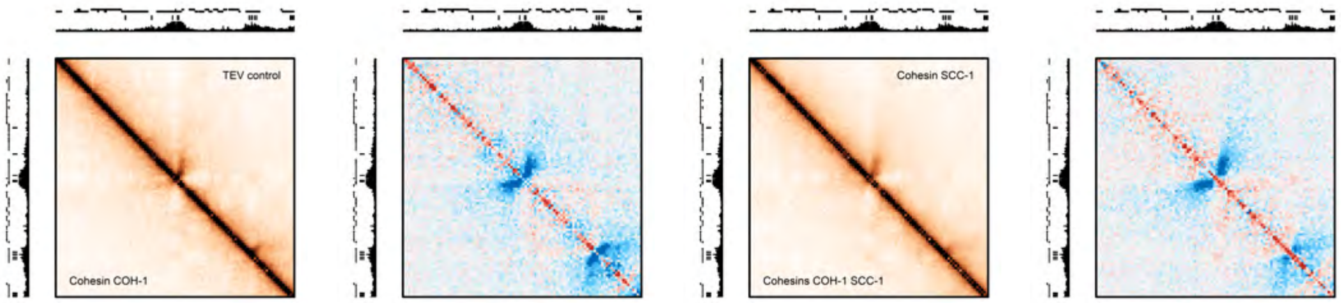

chrX:9001001-9201000

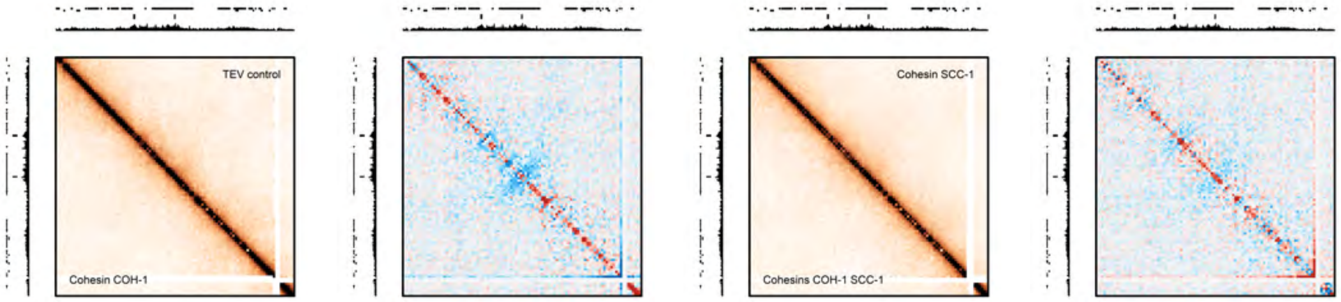

chrII:14147001-14347000

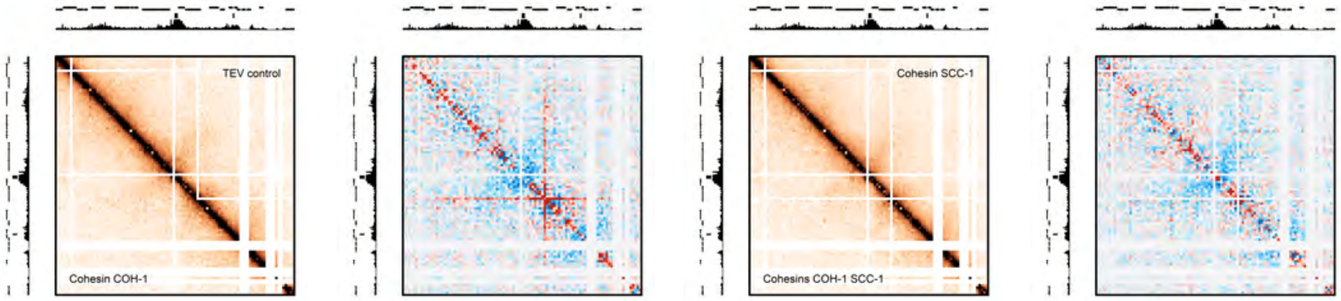

chrII:8963001-9163000

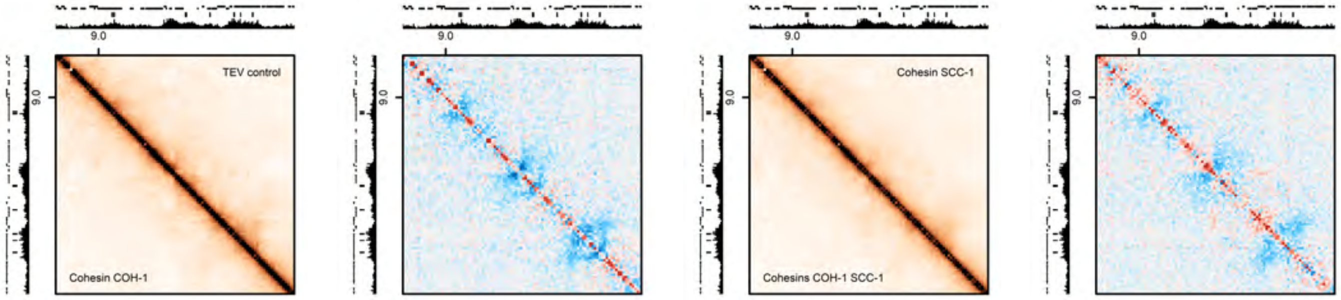

chrII:8383001-8583000

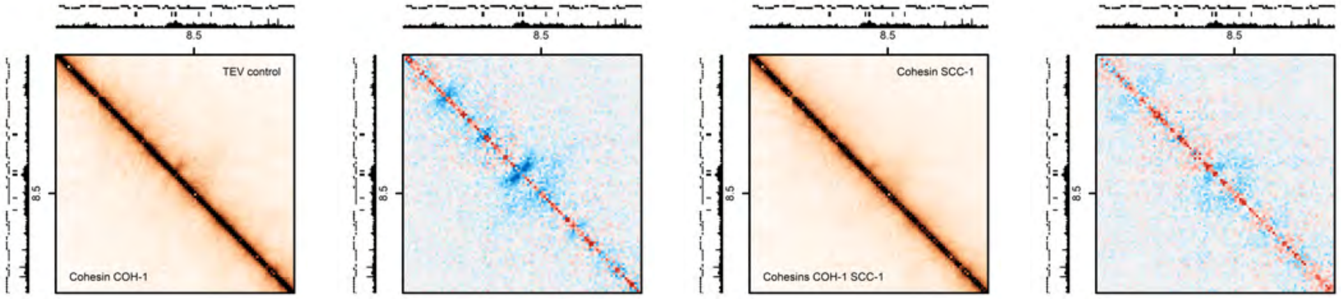

chrIII:5525001-5725000

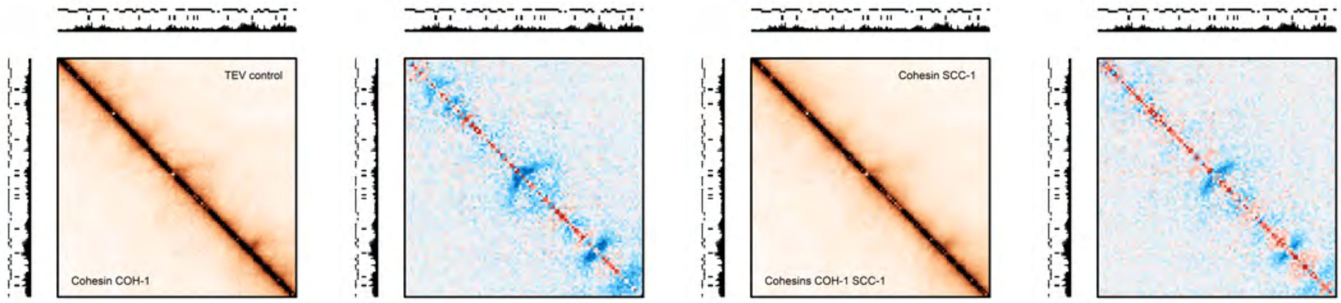

chrII:7241001-7441000

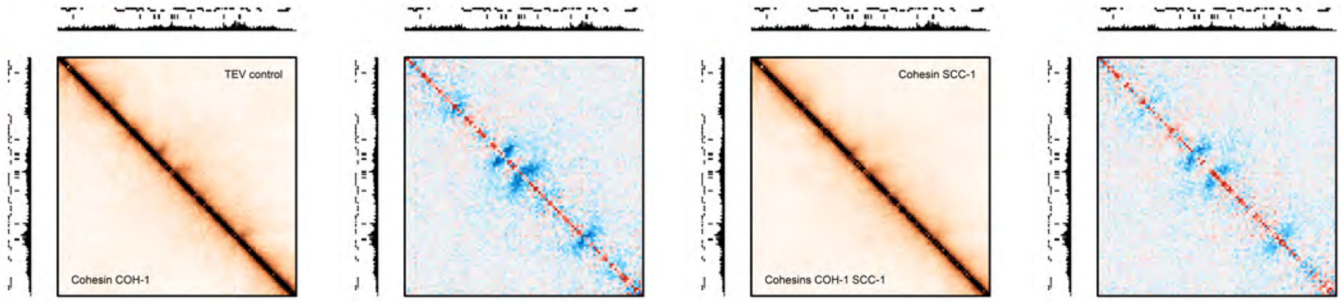

chrX:10935001-11135000

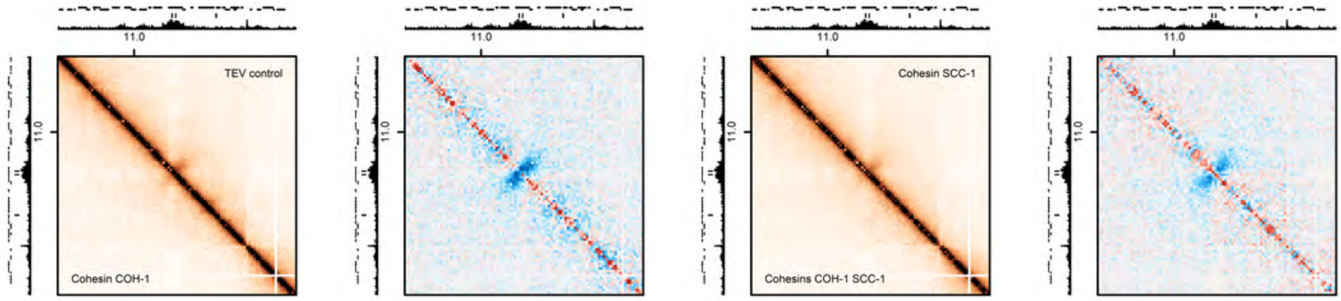

chrX:12569001-12769000

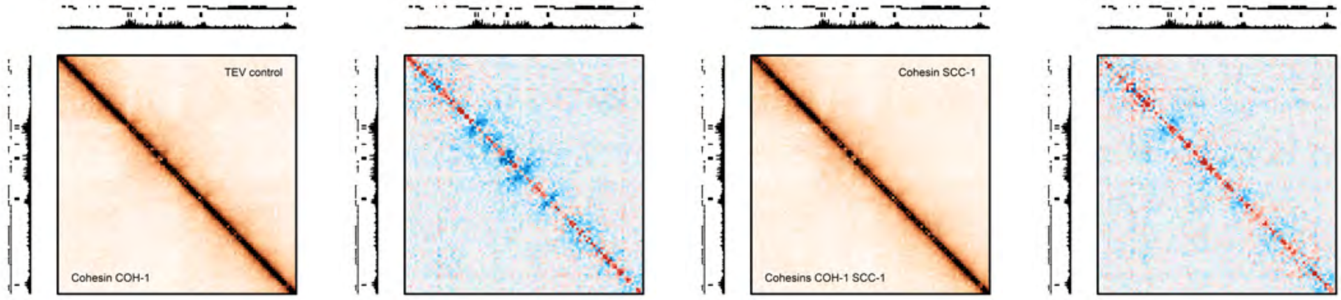

chrX:13645001-13845000

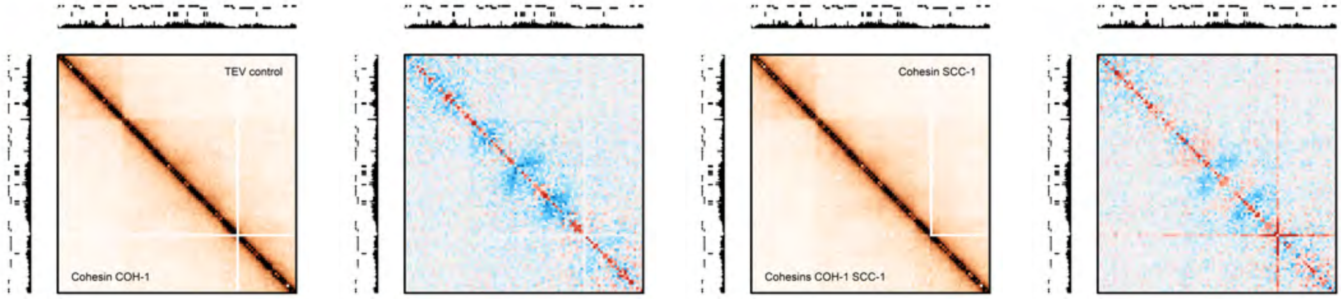

chrIV:6473001-6673000

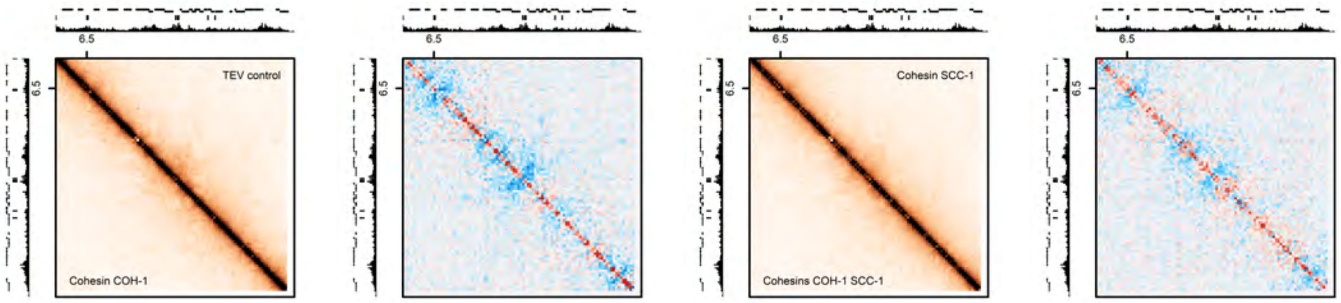

chrV:12201001-12401000

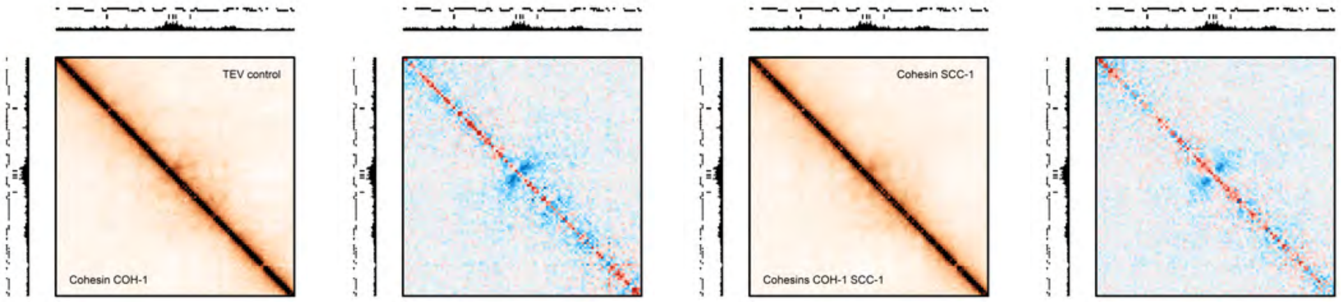

chrI:6021001-6221000

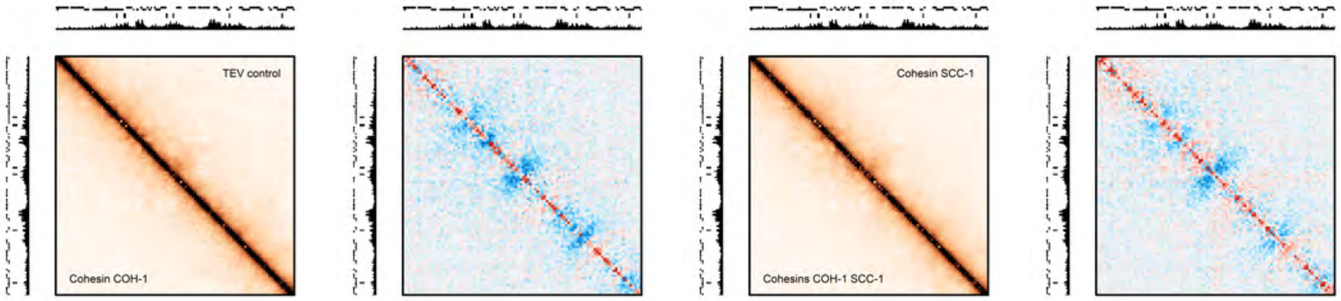

chrX:13255001-13455000

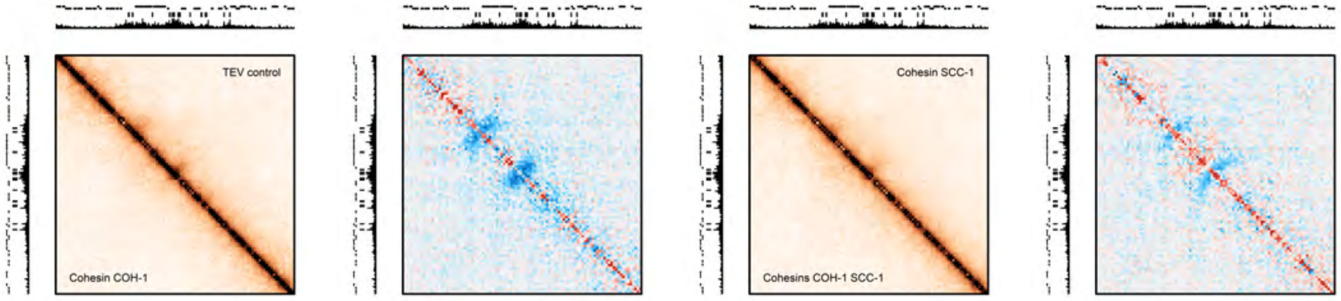

chrIII:1123001-1323000

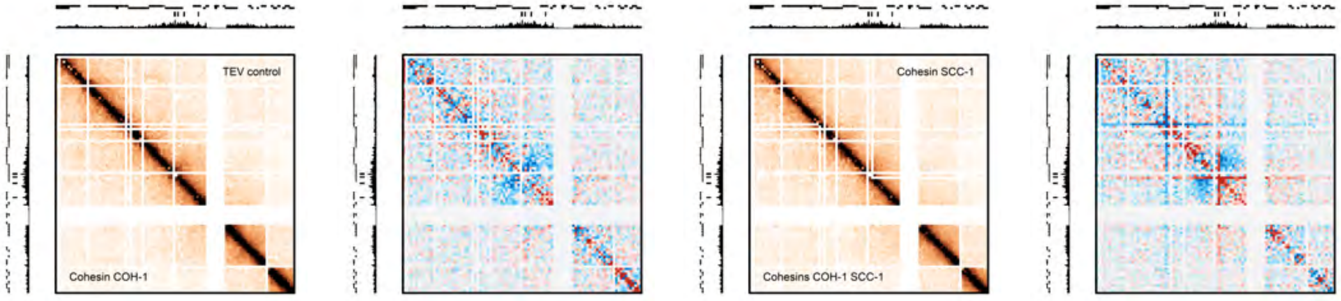

chrV:7747001-7947000

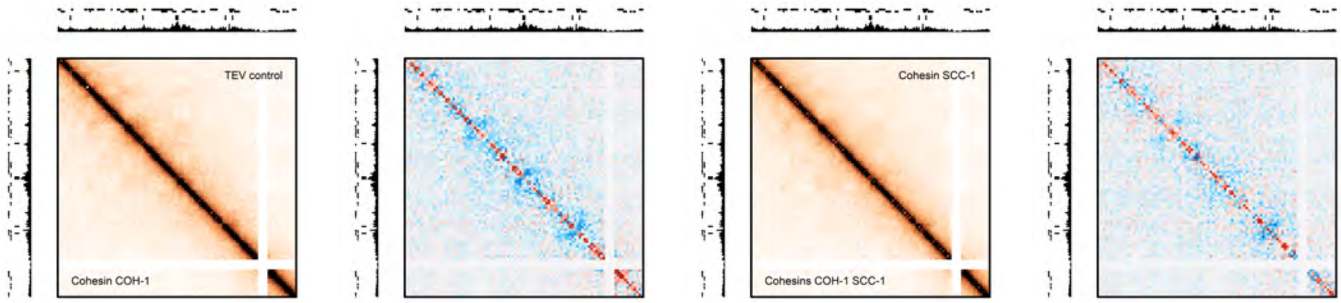

chrI:3209001-3409000

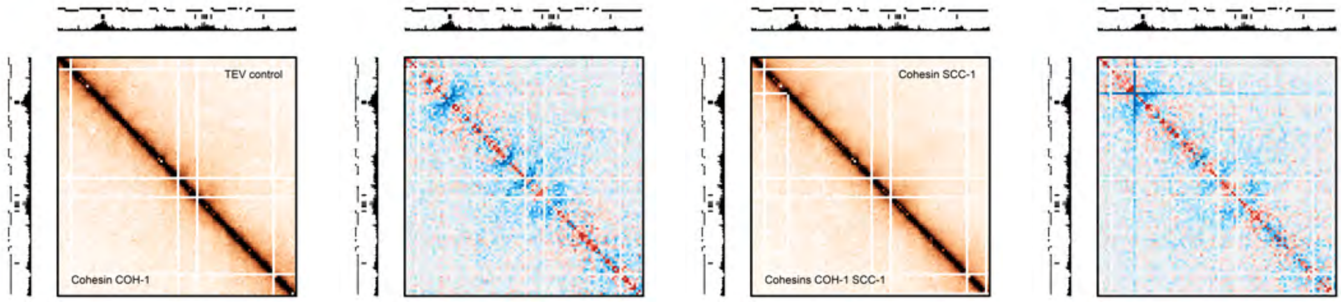

chrI:3727001-3927000

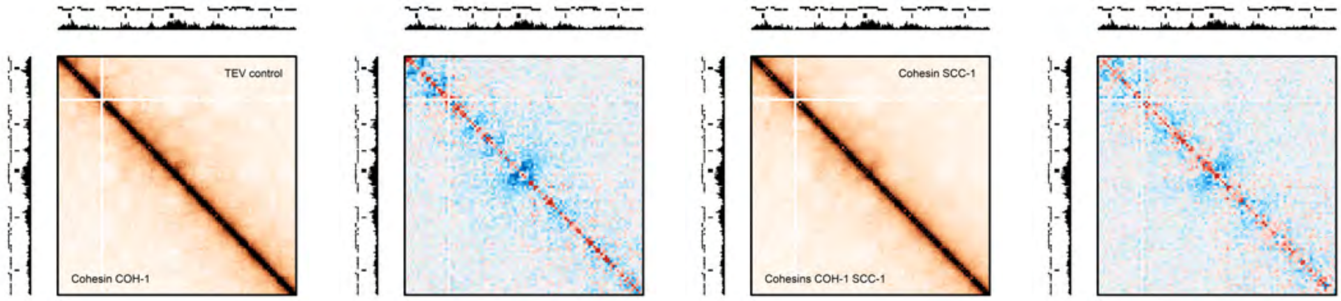

chrIV:731001-931000

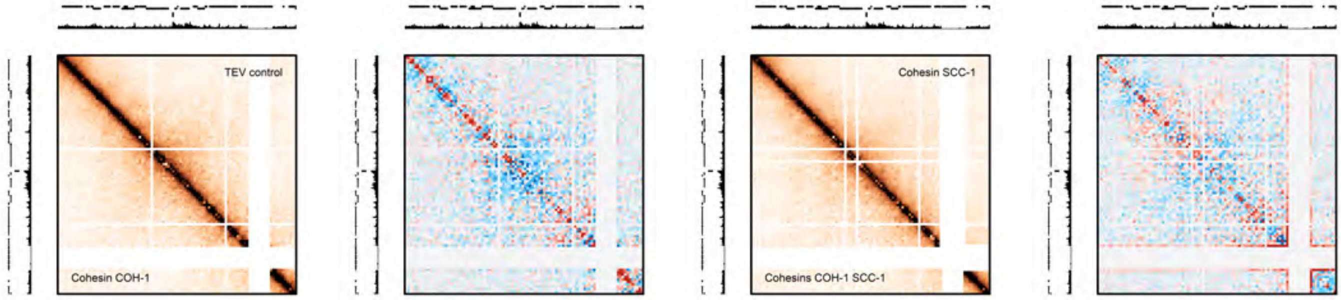

chrII:4807001-5007000

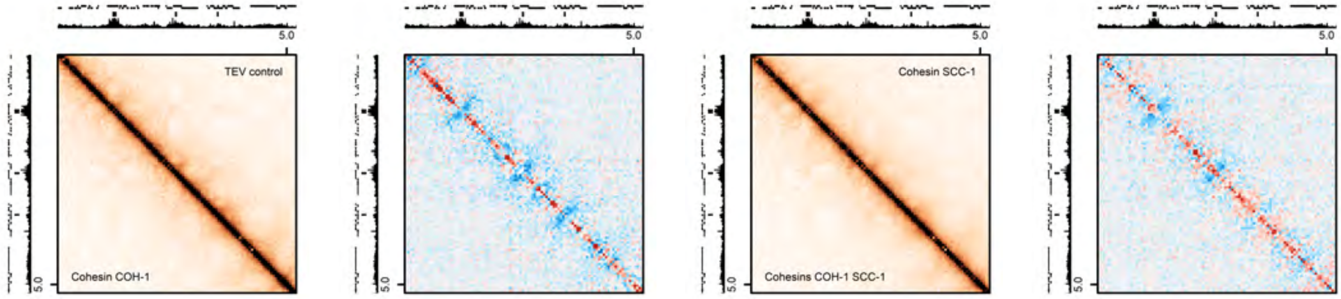

chrI:779001-979000

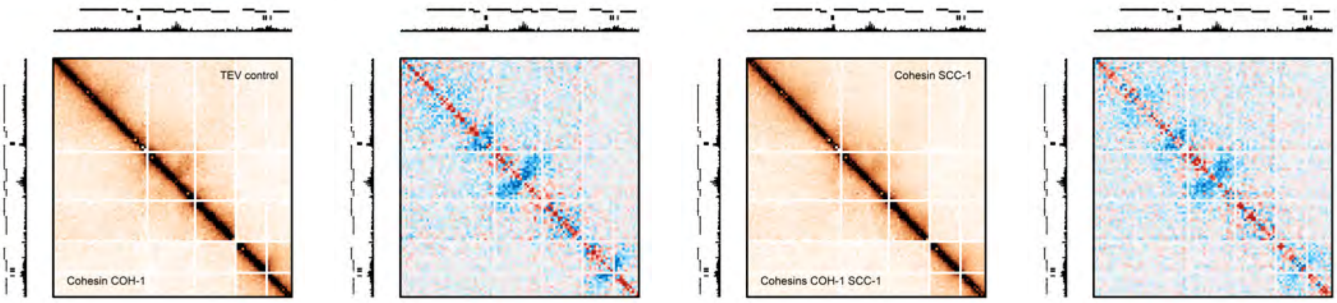

chrIII:6505001-6705000

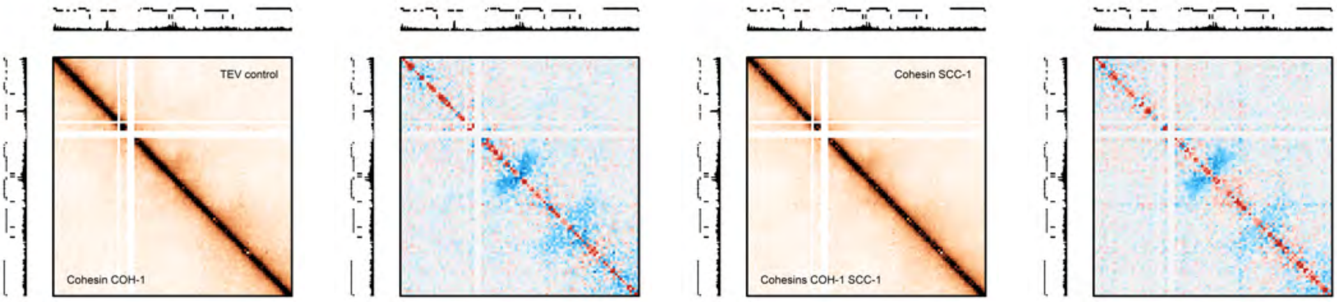

chrI:3909001-4109000

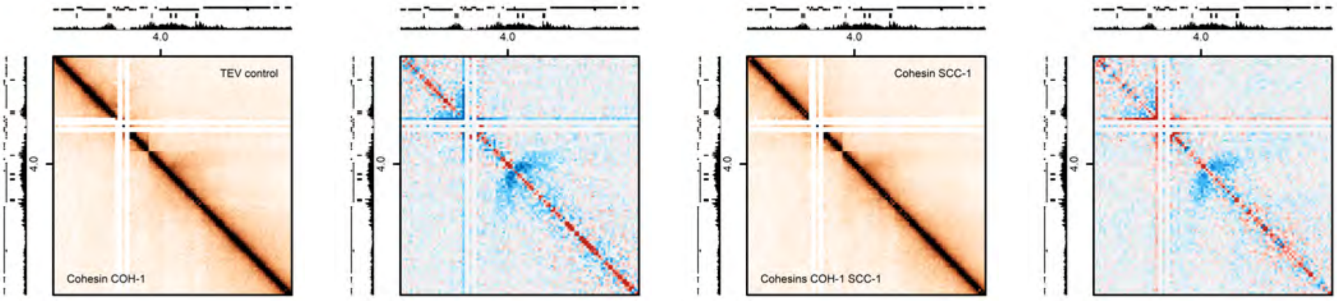

chrIII:-70999-129000

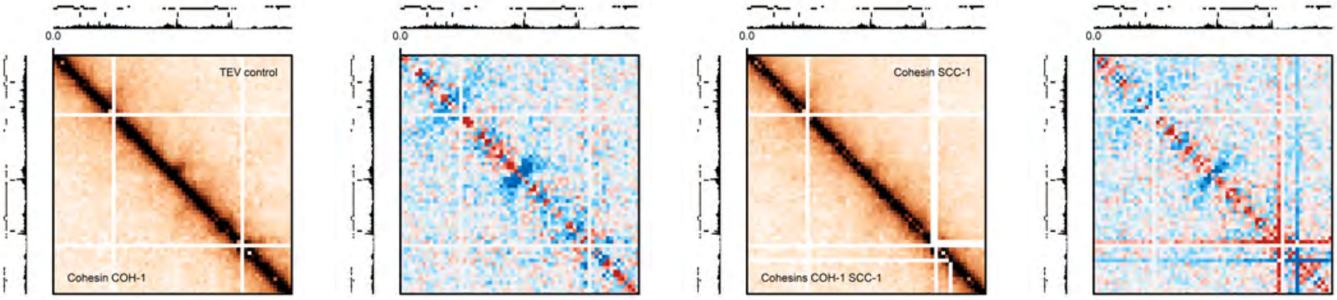

chrI:10463001-10663000

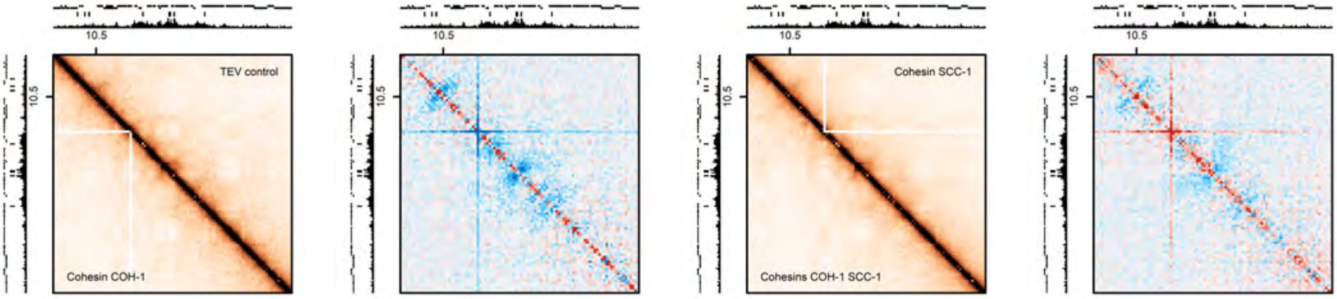

chrIII:7405001-7605000

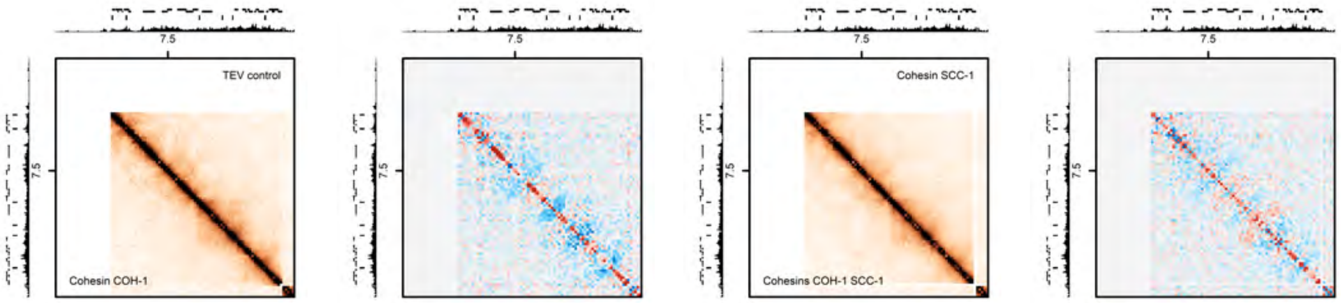

chrIV:8133001-8333000

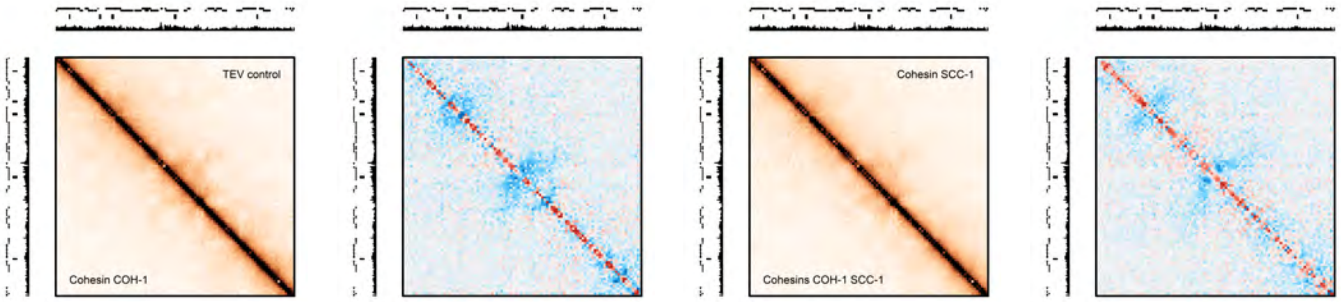

chrII:11941001-12141000

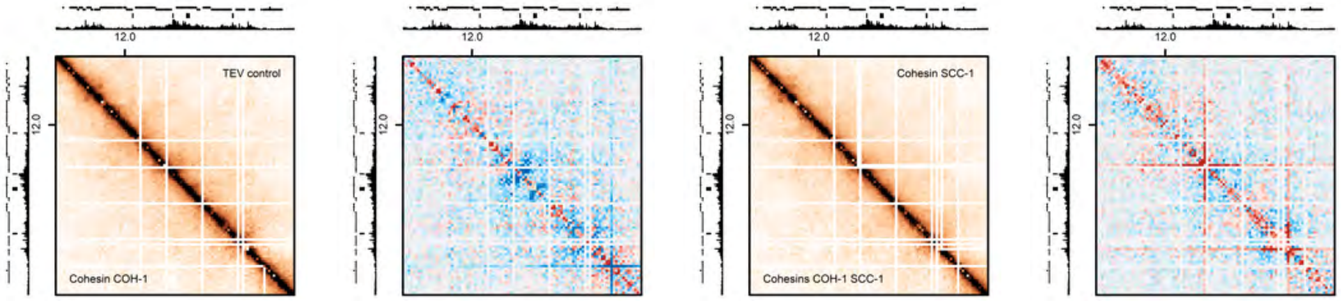

chrIV:13935001-14135000

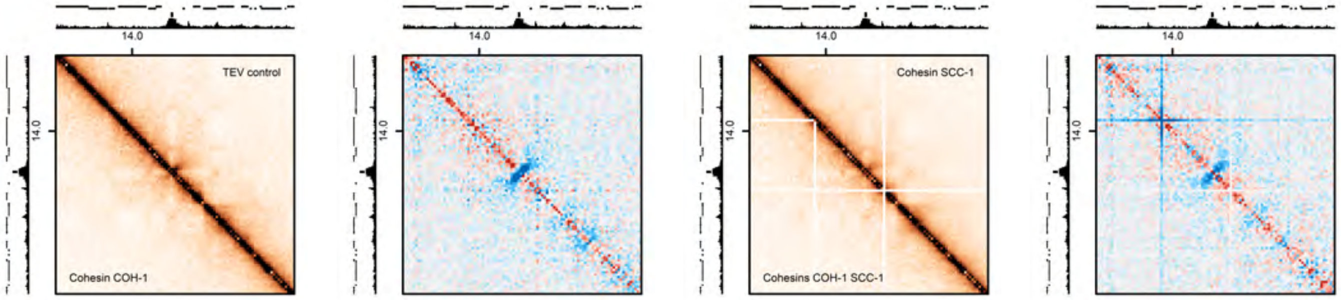

chrIII:3573001-3773000

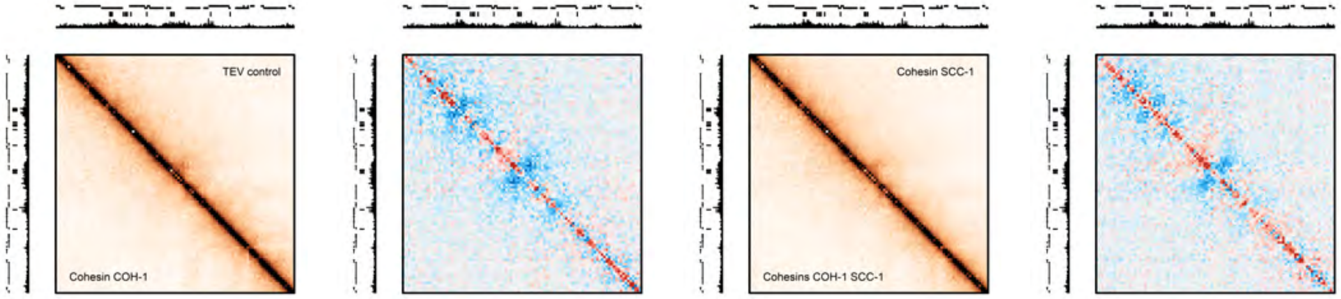

chrIV:12517001-12717000

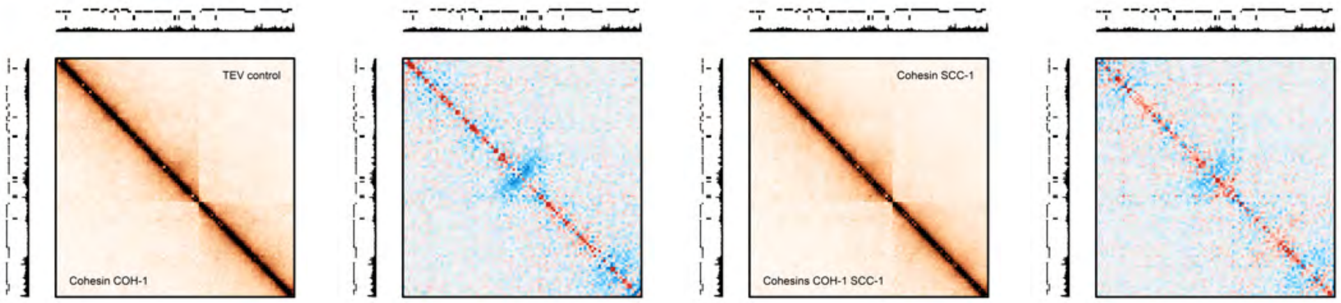

chrIV:9341001-9541000

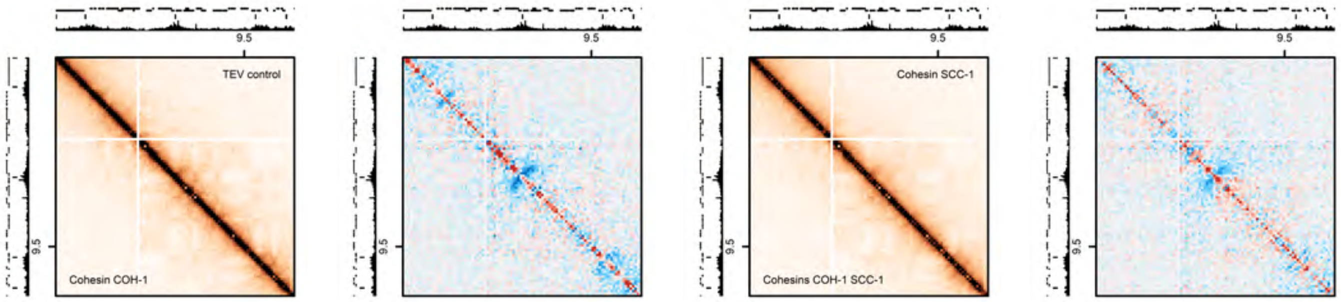

chrIV:2671001-2871000

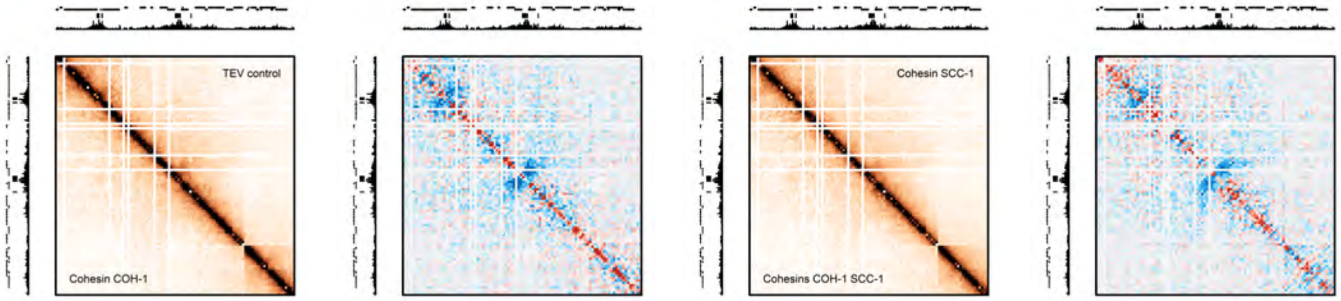

chrV:13291001-13491000

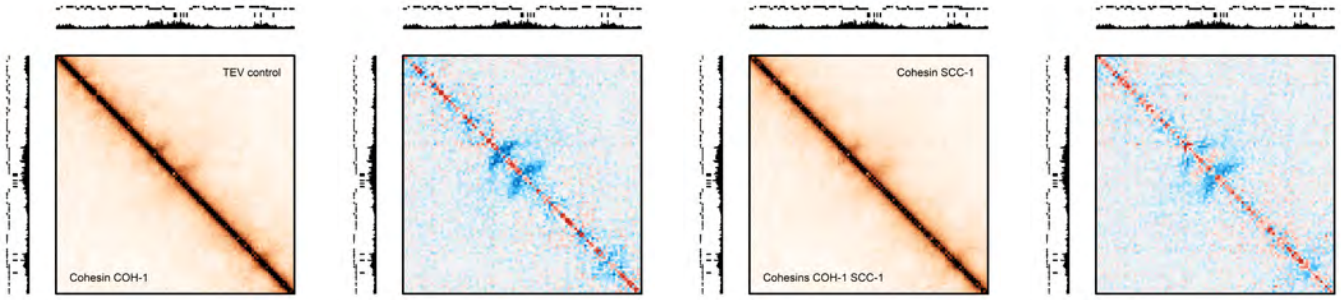

chrIV:7303001-7503000

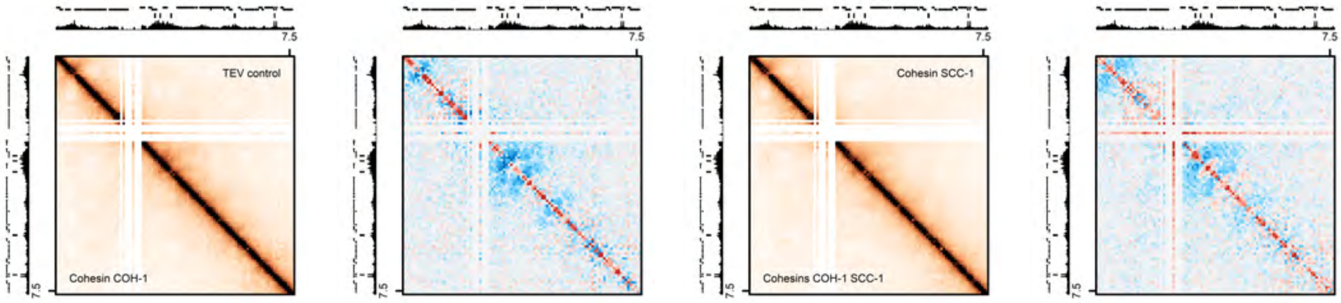

chrI:2583001-2783000

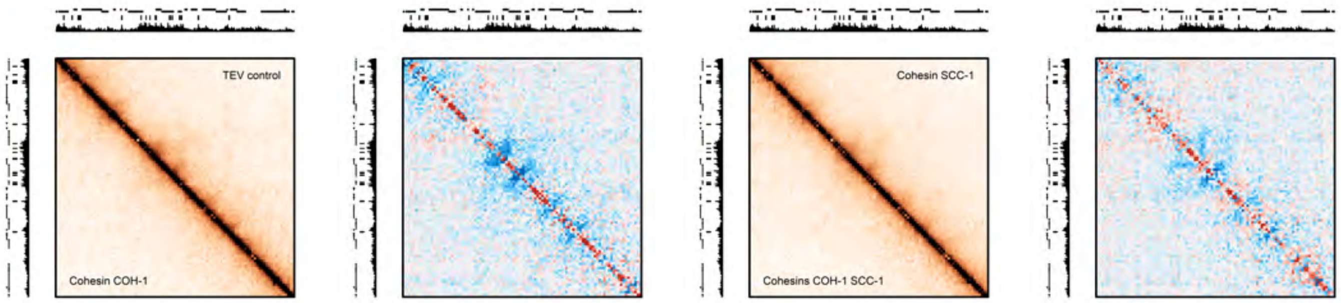

chrX:5941001-6141000

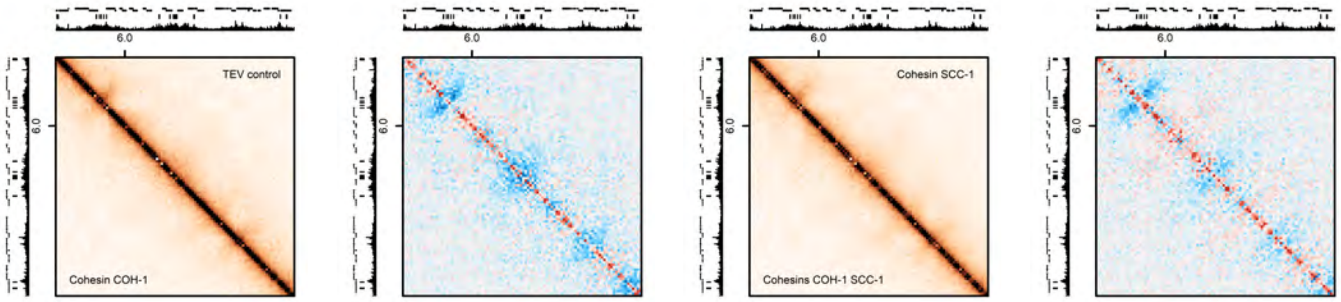

chrV:16497001-16697000

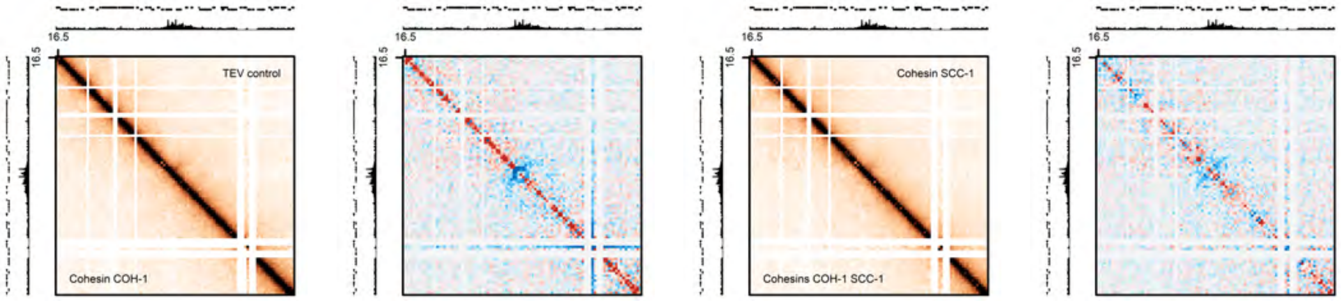

chrIII:3879001-4079000

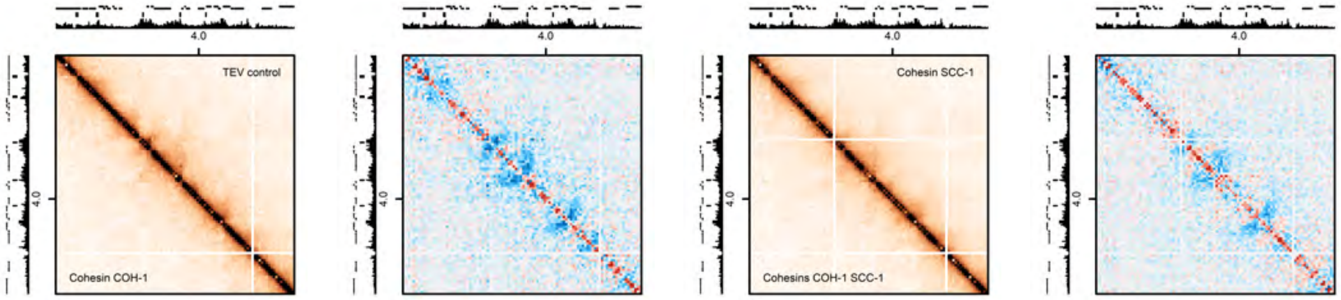

chrIV:5493001-5693000

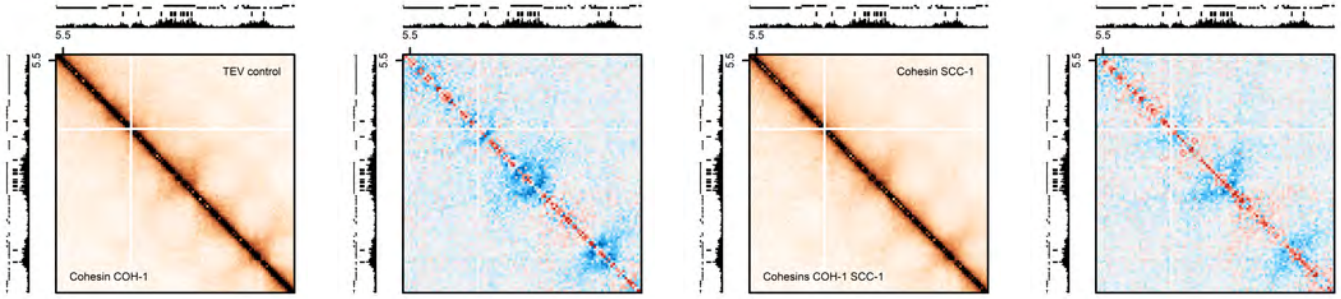

chrIV:8697001-8897000

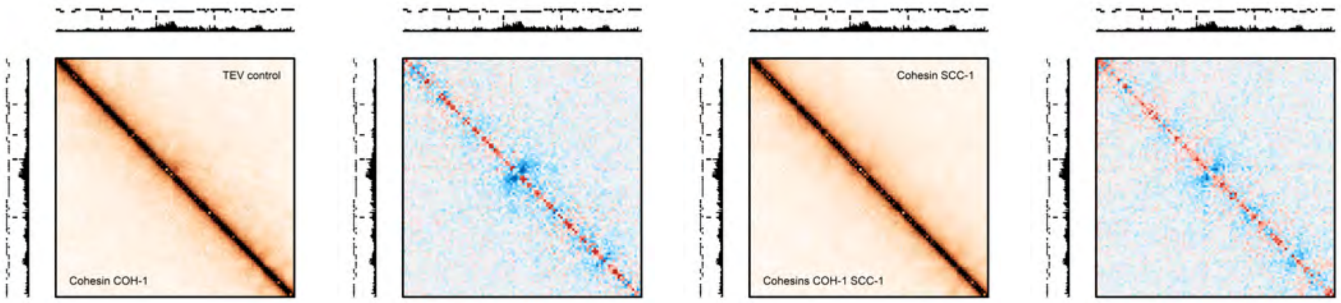

chrV:15561001-15761000

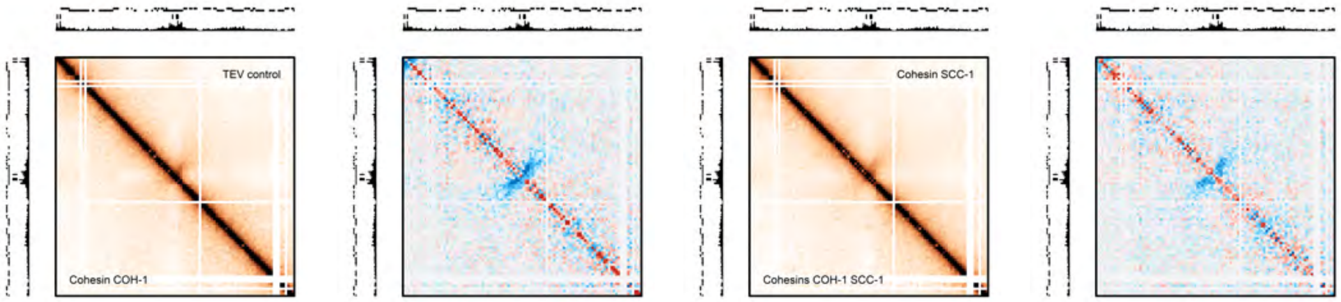

chrII:7539001-7739000

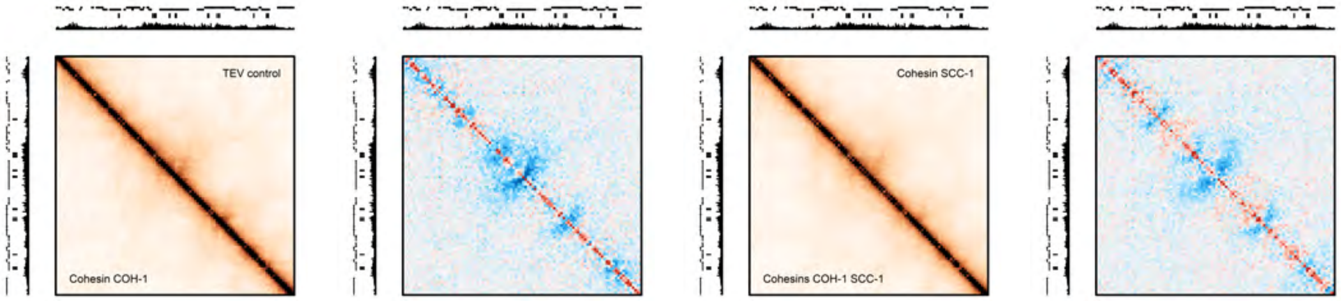

chrV:6415001-6615000

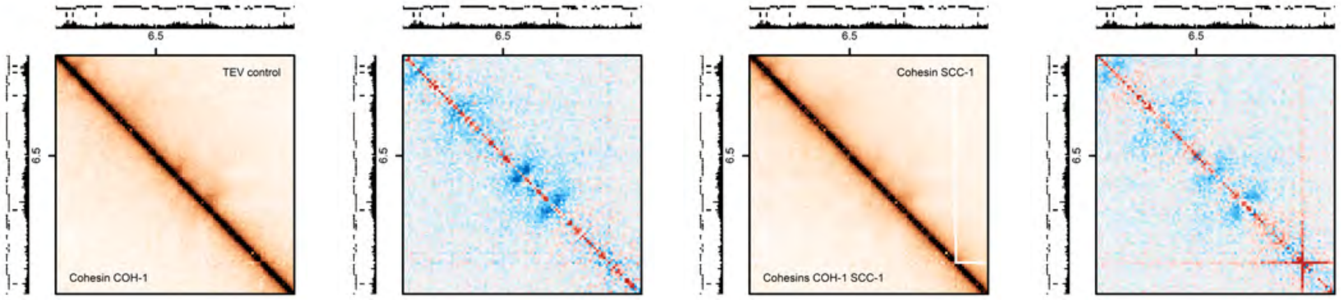

chrI:8205001-8405000

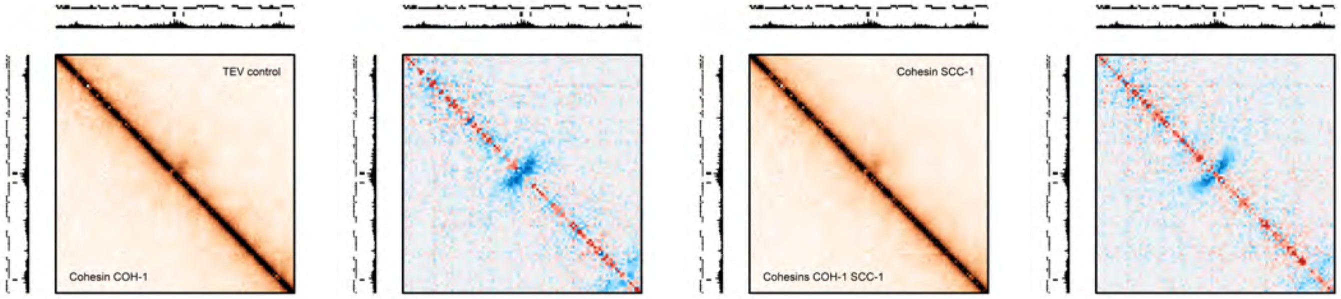

chrV:16681001-16881000

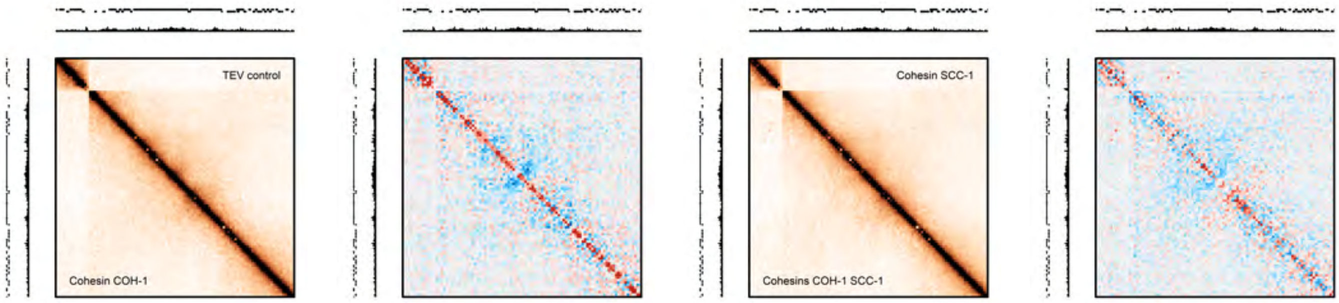

chrI:7561001-7761000

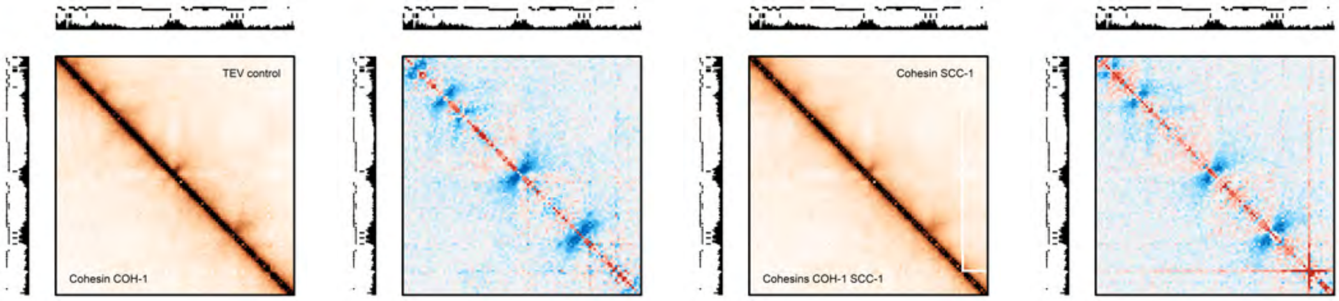

chrI:4393001-4593000

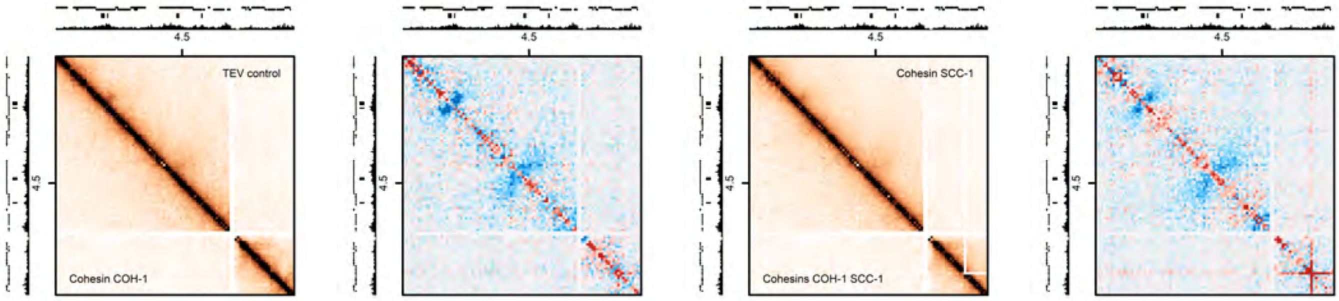

chrIII:11253001-11453000

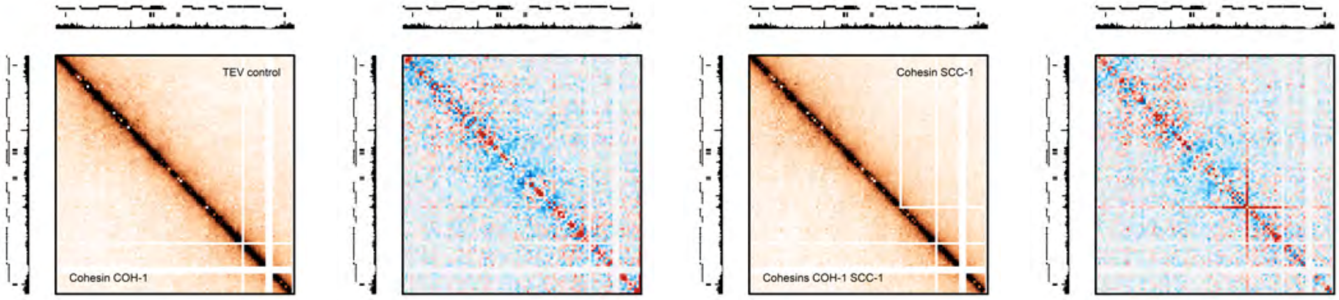

chrIV:6949001-7149000

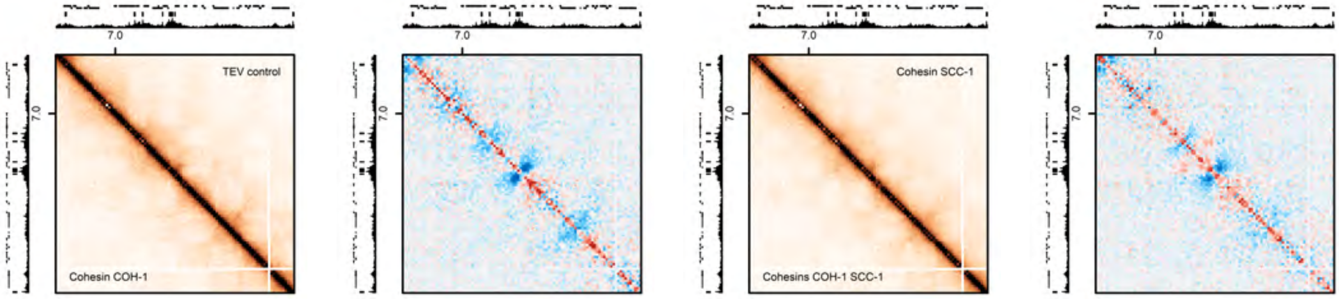

chrV:4539001-4739000

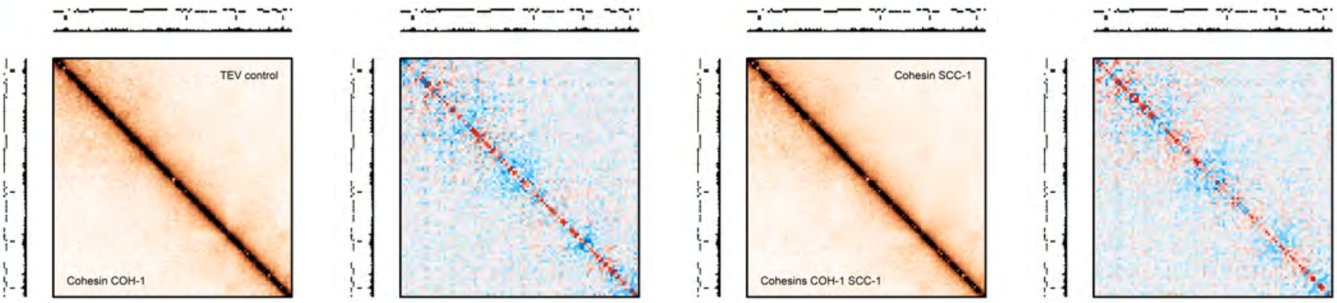

chrII:63001-263000

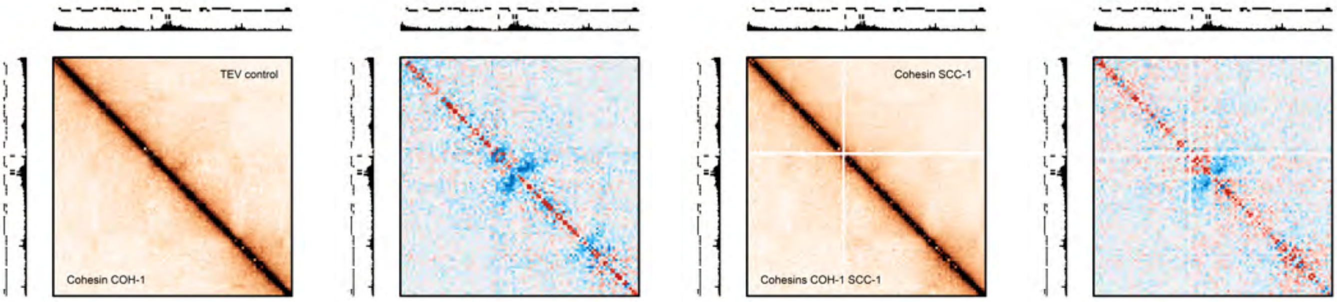

chrX:4119001-4319000

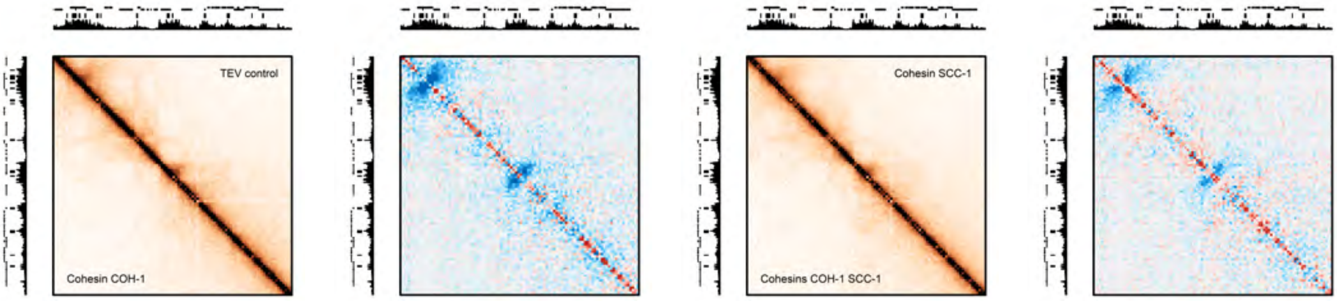

chrV:10977001-11177000

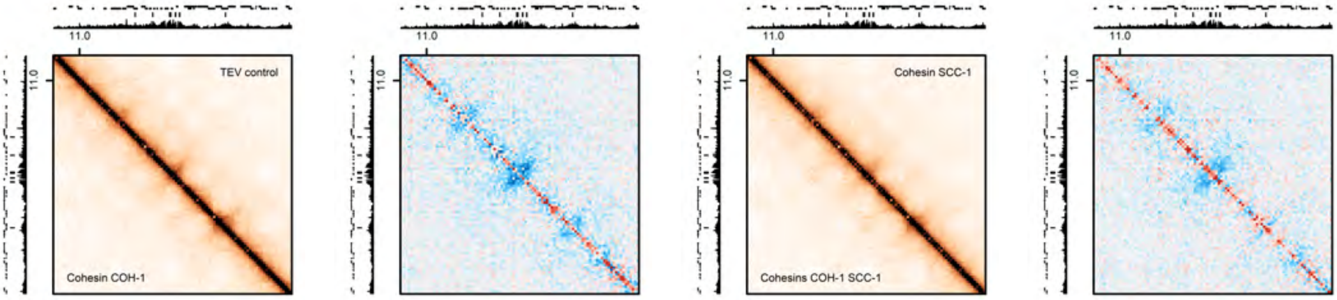

chrI:2193001-2393000

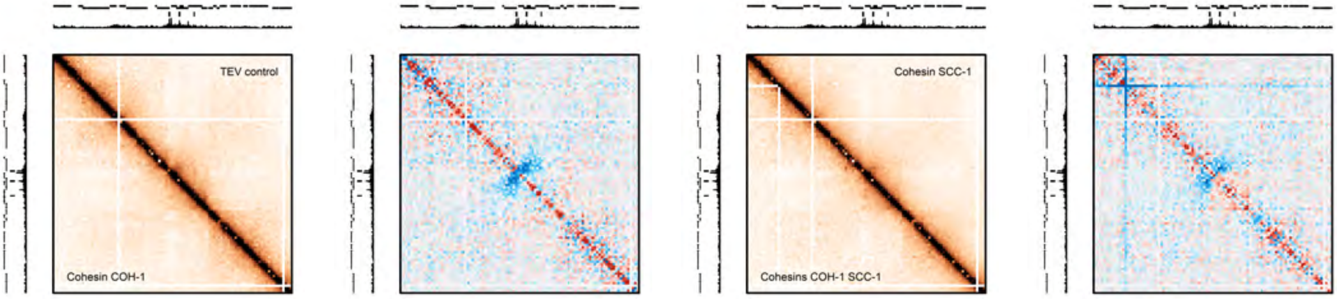

chrX:5111001-5311000

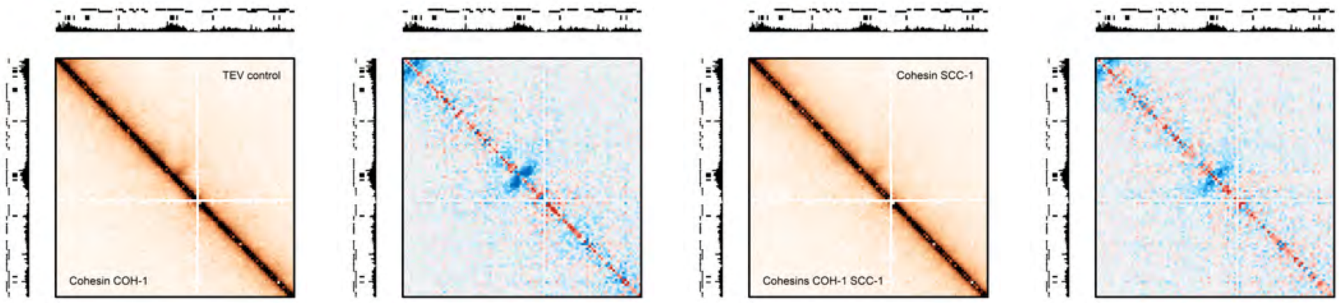

chrI:13971001-14171000

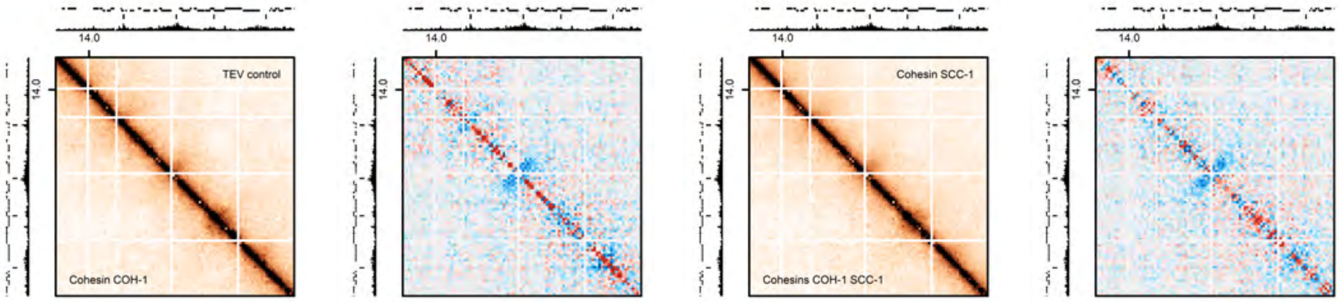

chrIII:10719001-10919000

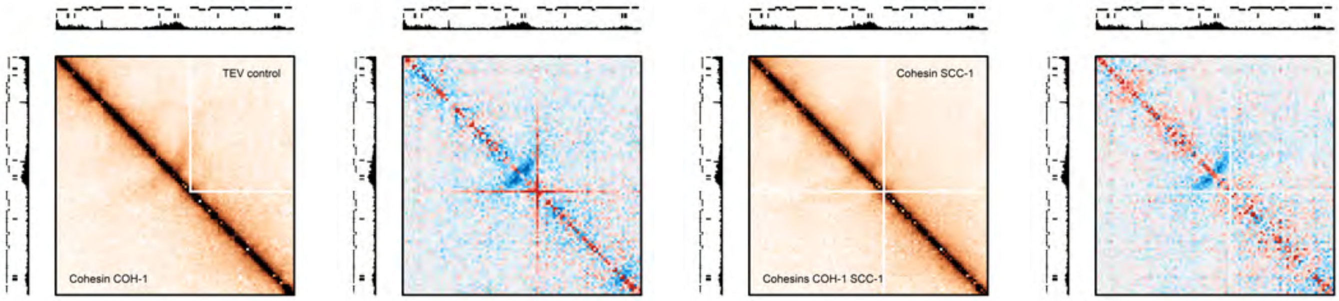

chrV:2577001-2777000

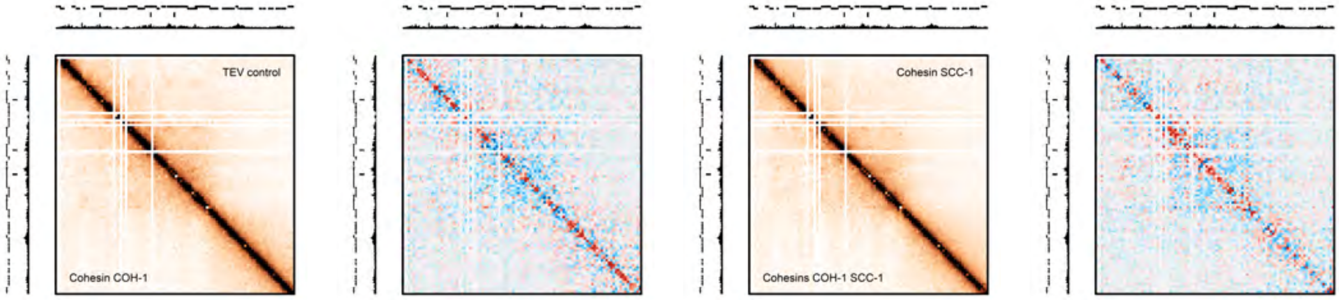

chrI:3001001-3201000

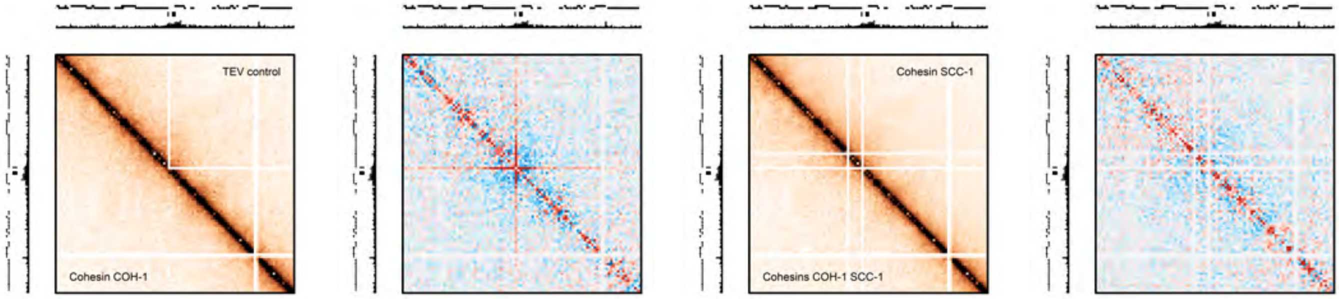

chrIV:17179001-17379000

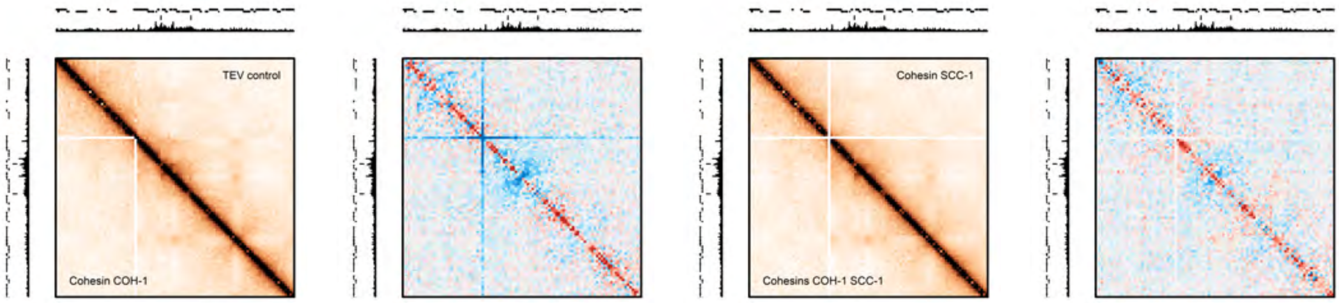

chrIII:7275001-7475000

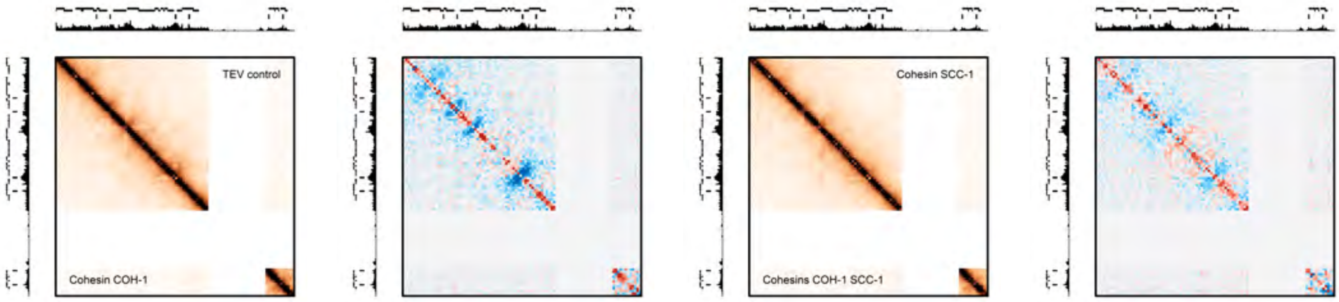

chrIV:967001-1167000

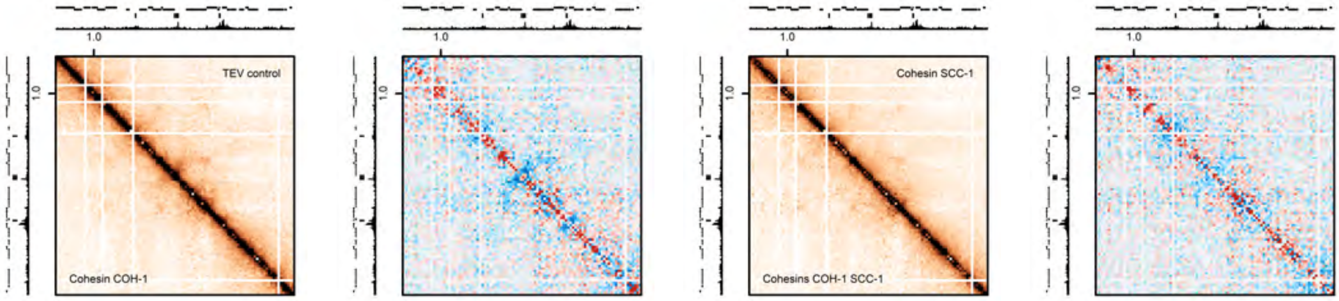

chrV:14267001-14467000

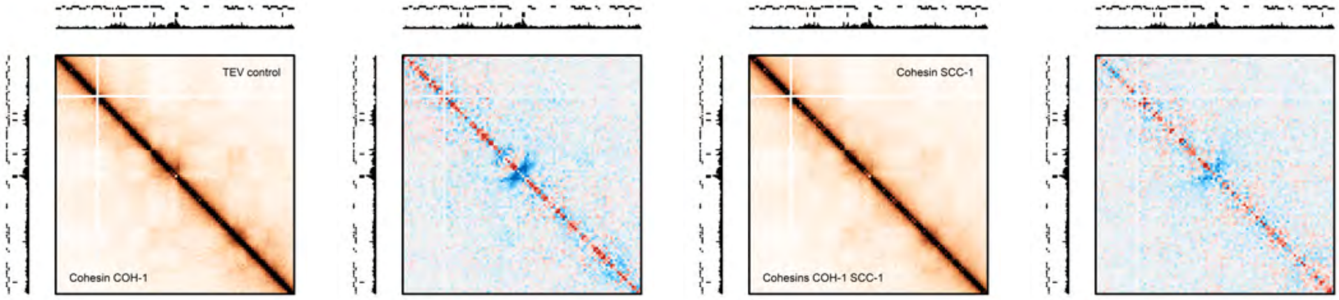

chrV:19905001-20105000

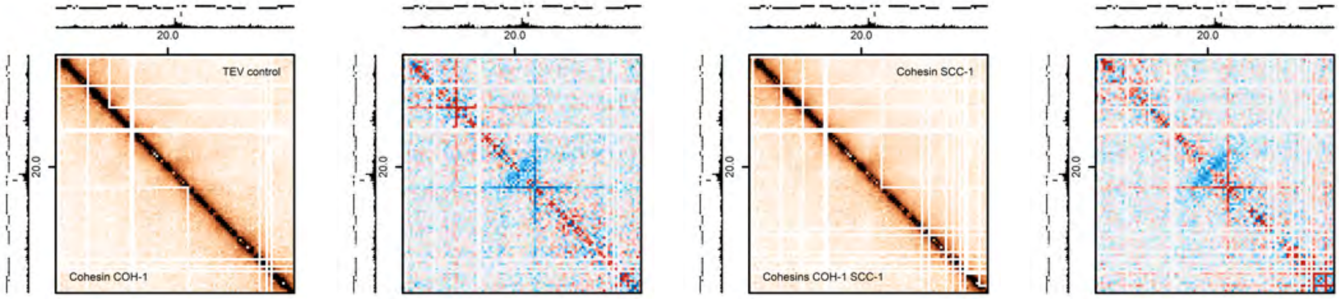

chrX:8377001-8577000

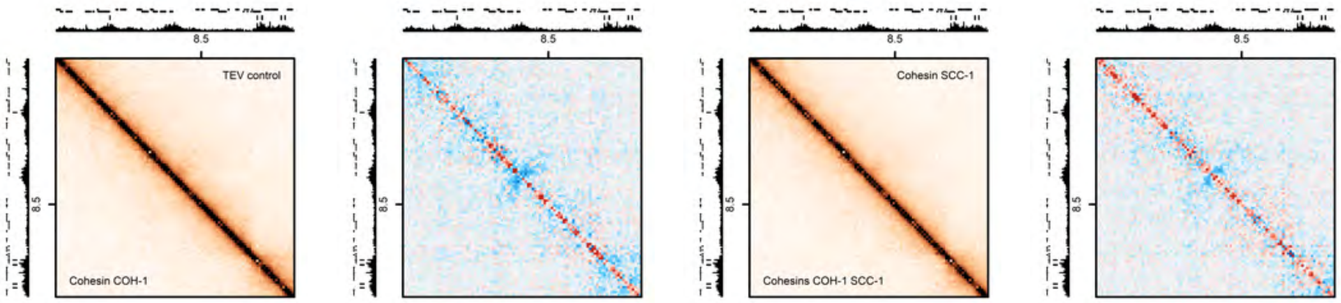

chrI:12935001-13135000

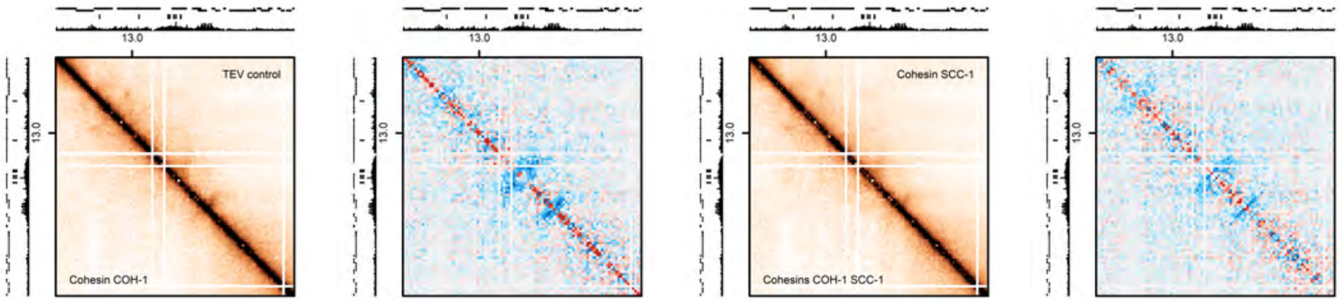

chrV:6993001-7193000

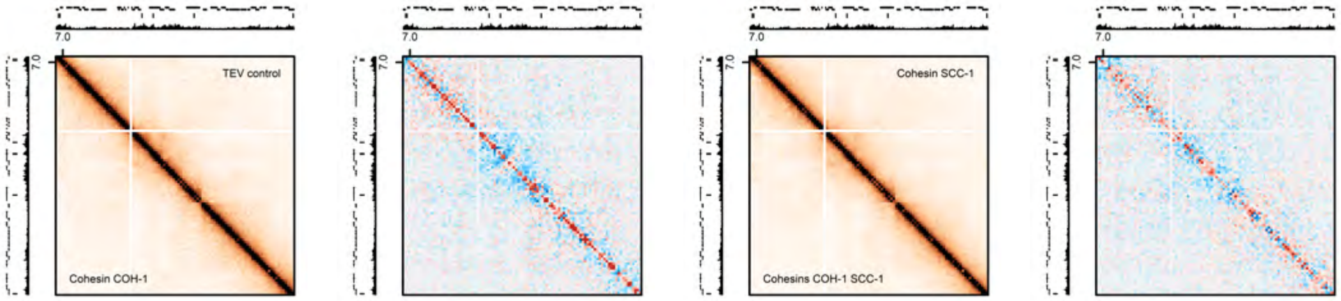

chrV:9631001-9831000

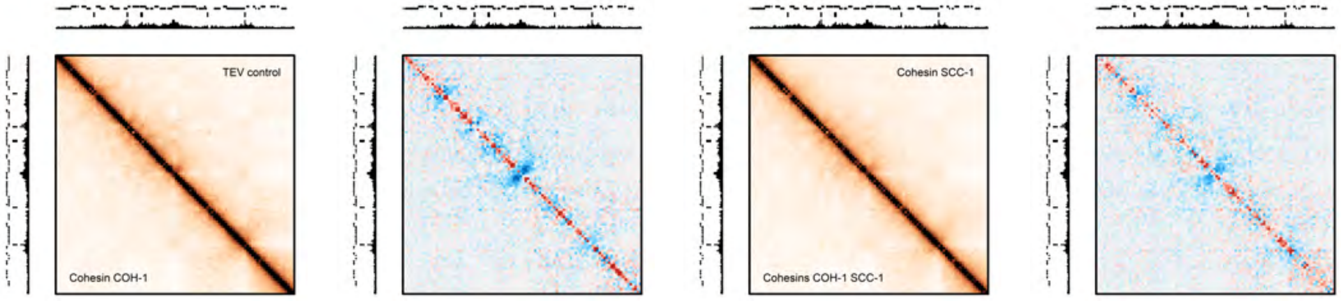

chrIII:465001-665000

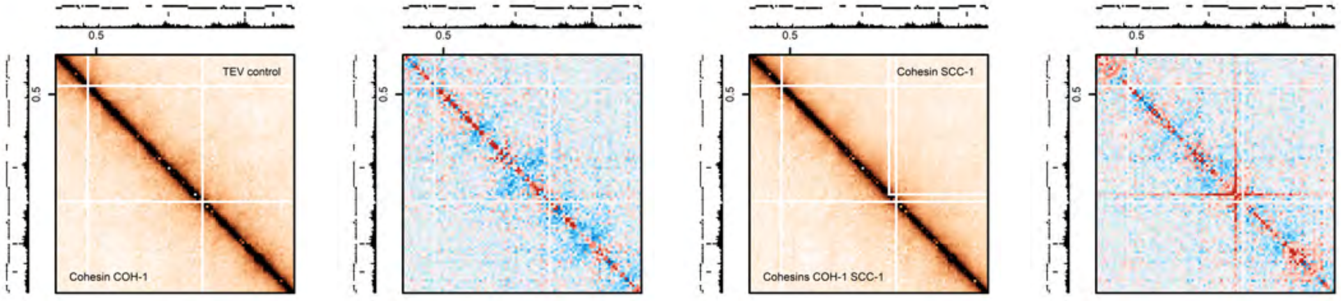

chrIII:5101001-5301000

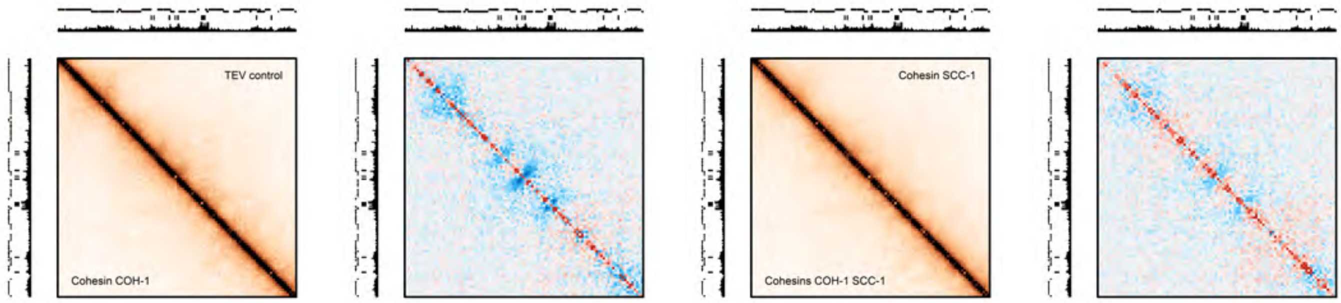

chrIII:7877001-8077000

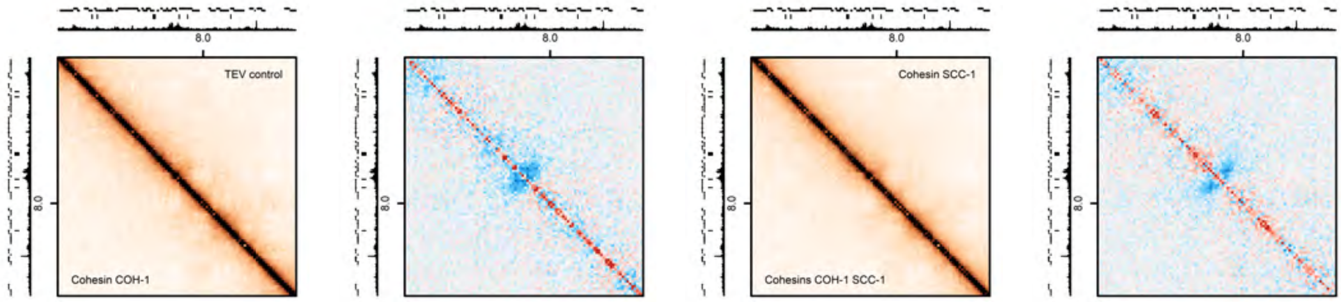

chrV:15465001-15665000

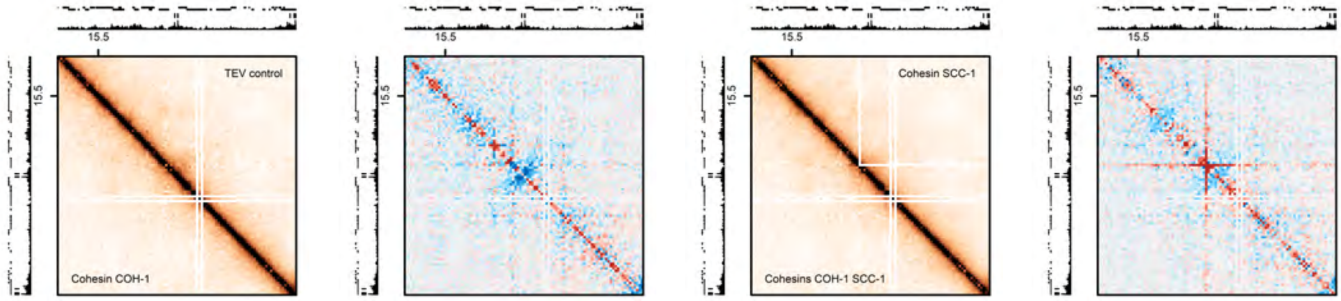

chrIV:10239001-10439000

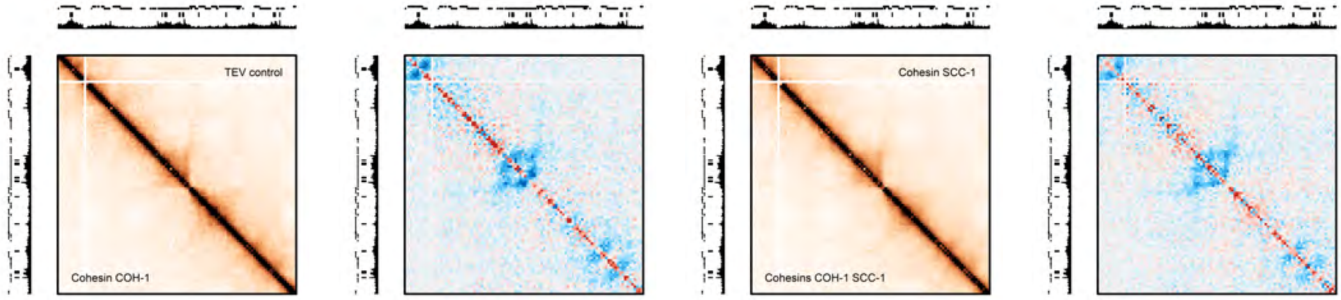

chrX:7561001-7761000

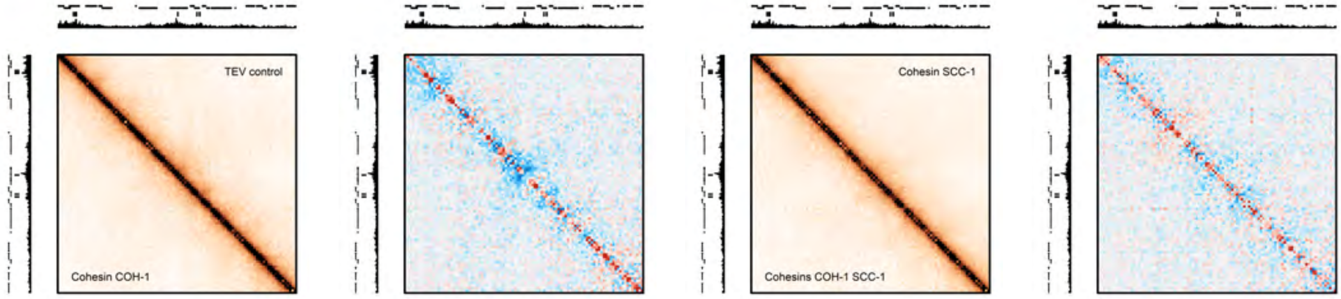

chrI:1463001-1663000

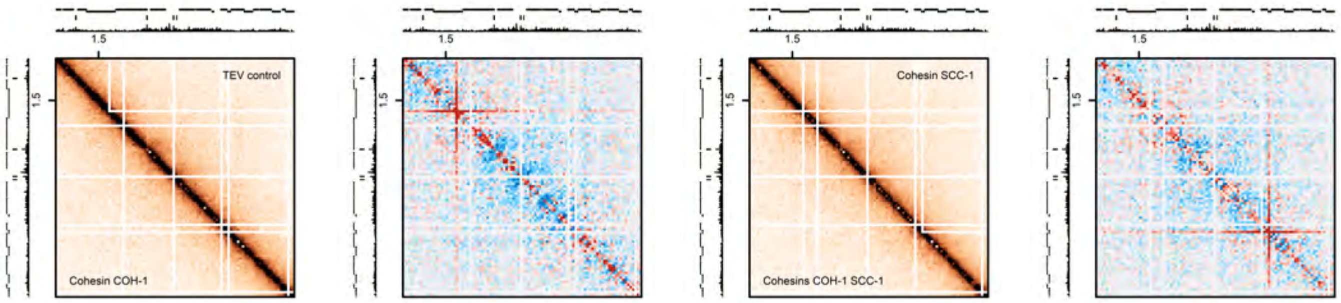

chrII:2703001-2903000

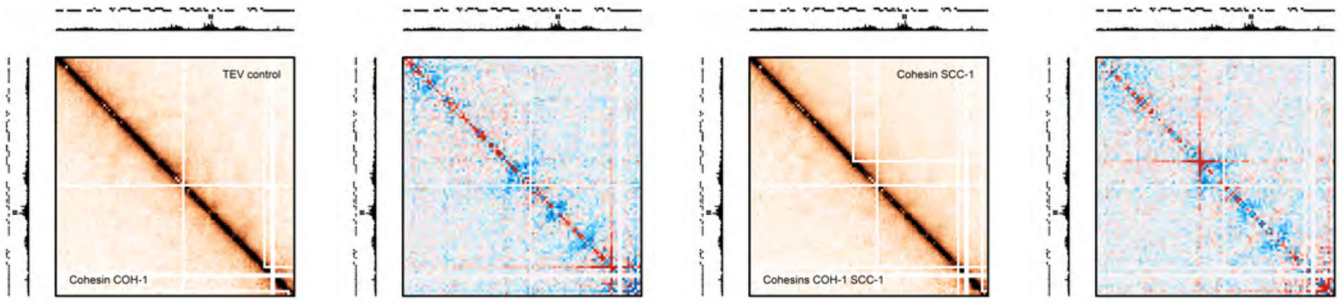

chrII:11059001-11259000

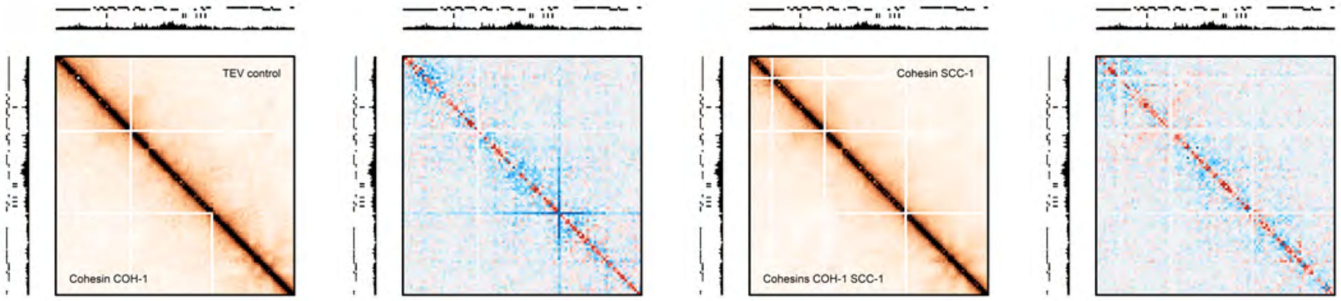

chrI:5779001-5979000

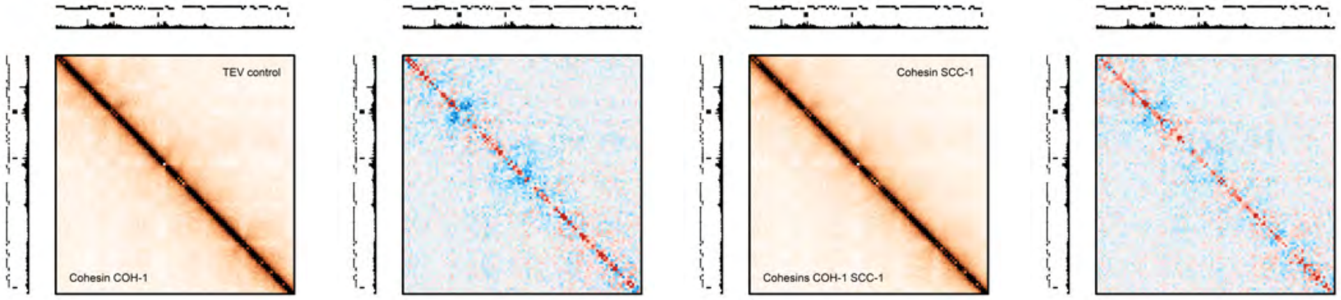

chrV:4087001-4287000

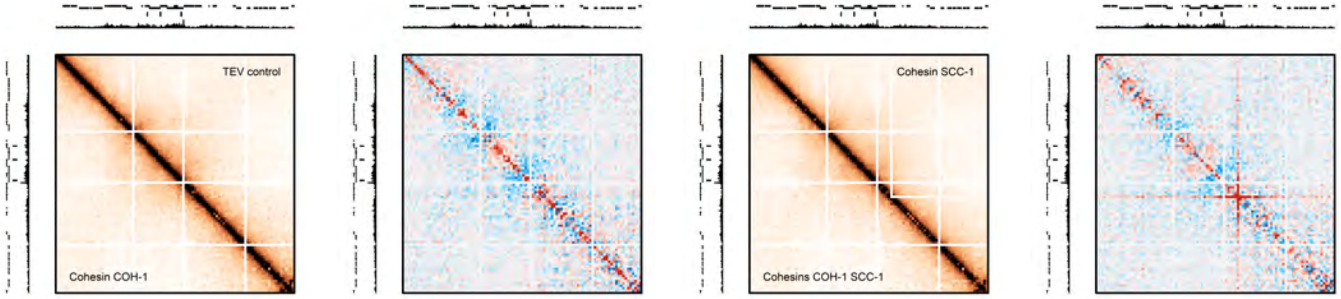

chrI:14509001-14709000

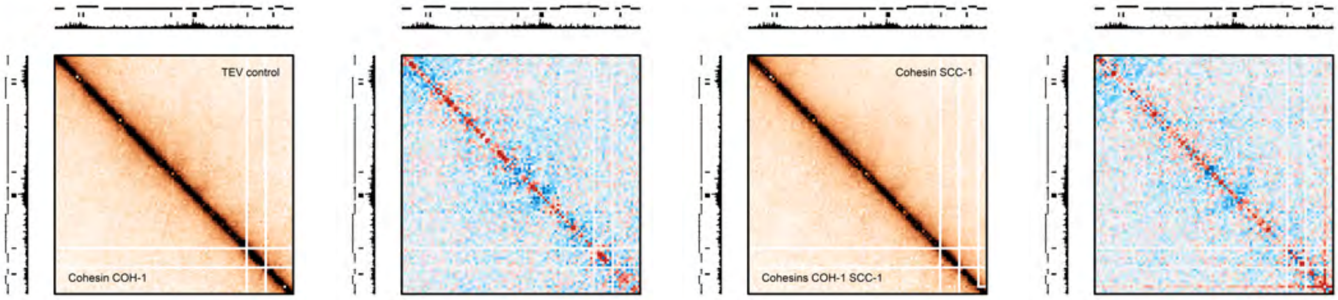

chrX:16697001-16897000

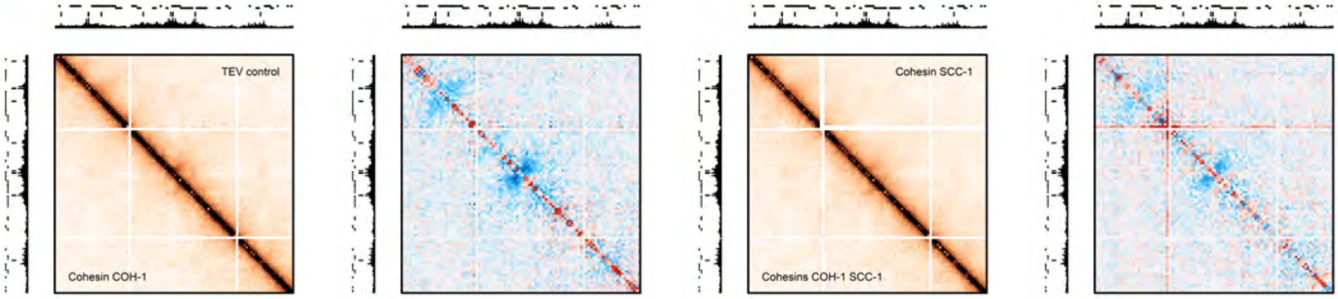

chrX:16513001-16713000

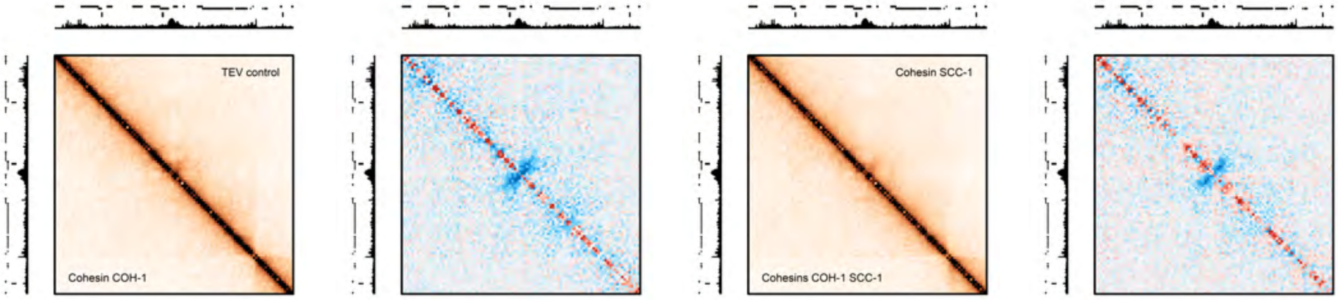

chrIII:8883001-9083000

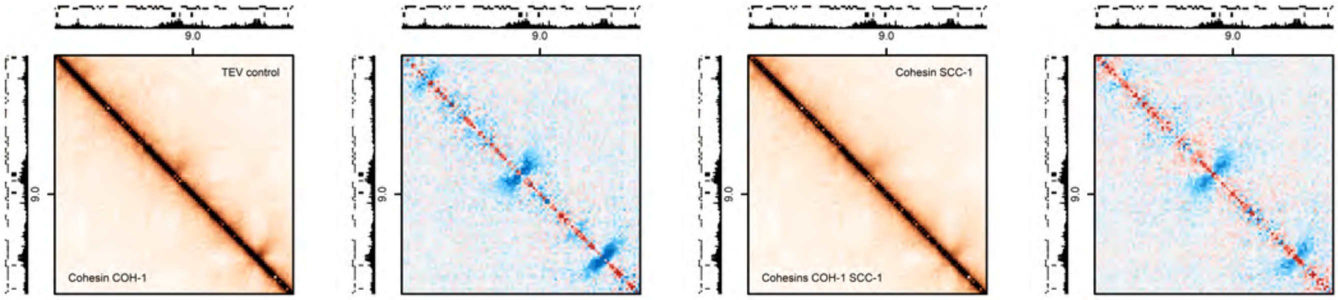

chrIII:11371001-11571000

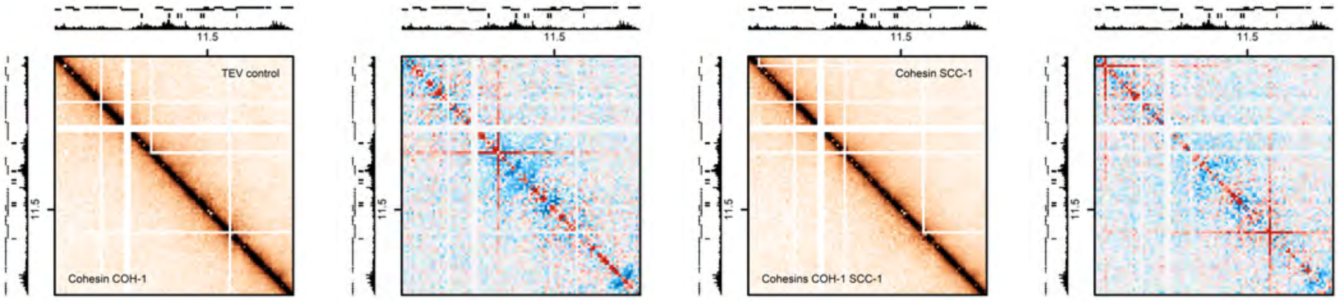

chrV:1973001-2173000

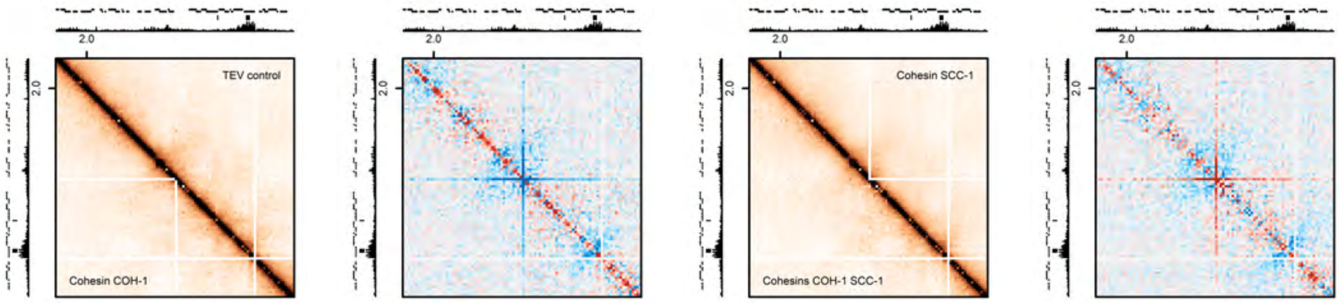

chrII:10287001-10487000

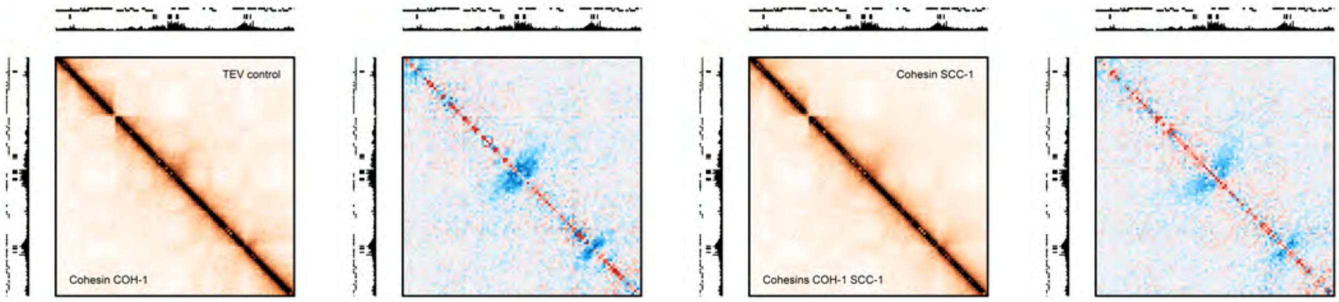

chrV:10149001-10349000

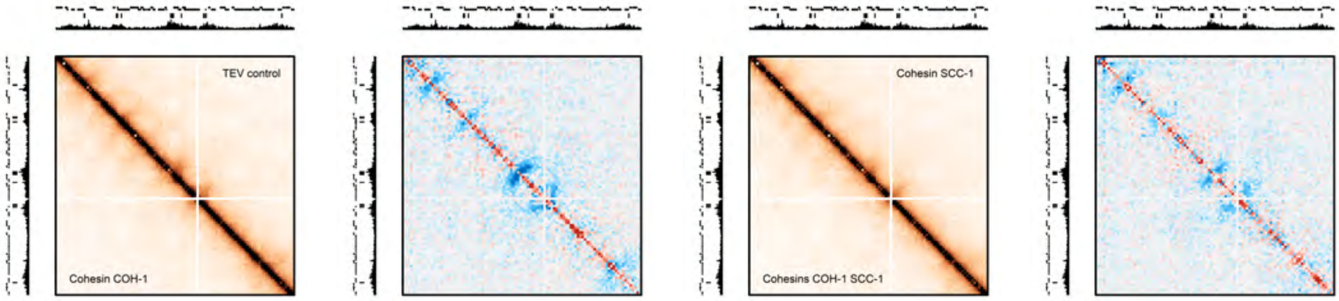

chrX:14405001-14605000

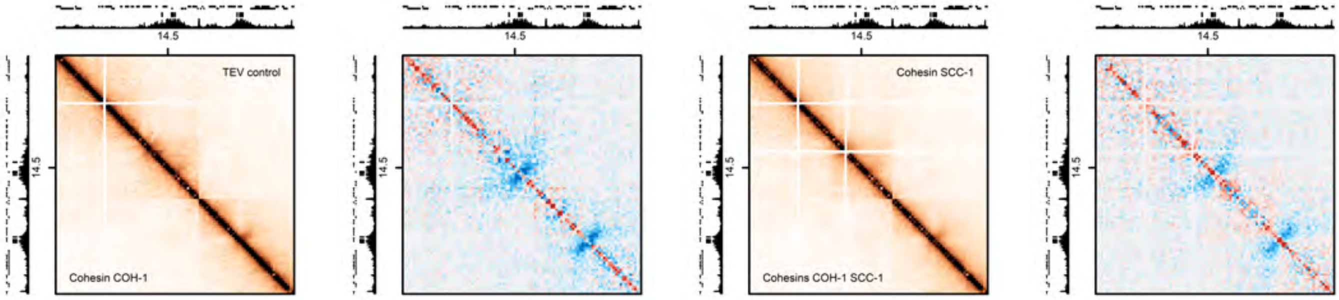

chrX:11865001-12065000

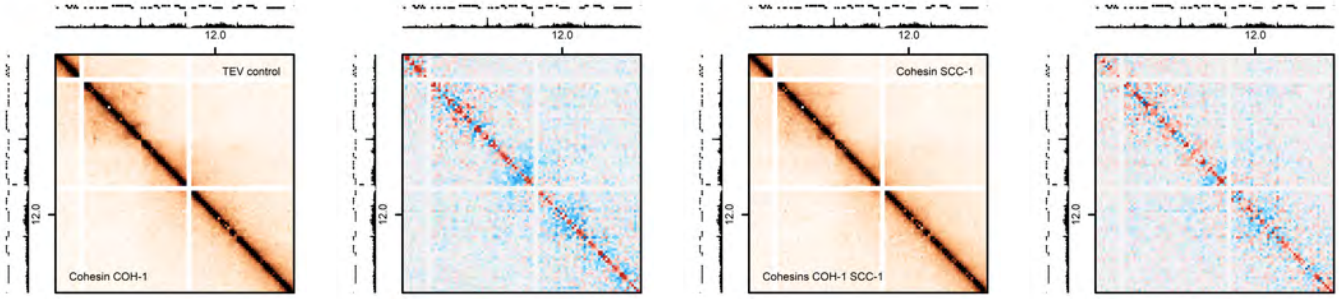

chrII:8645001-8845000

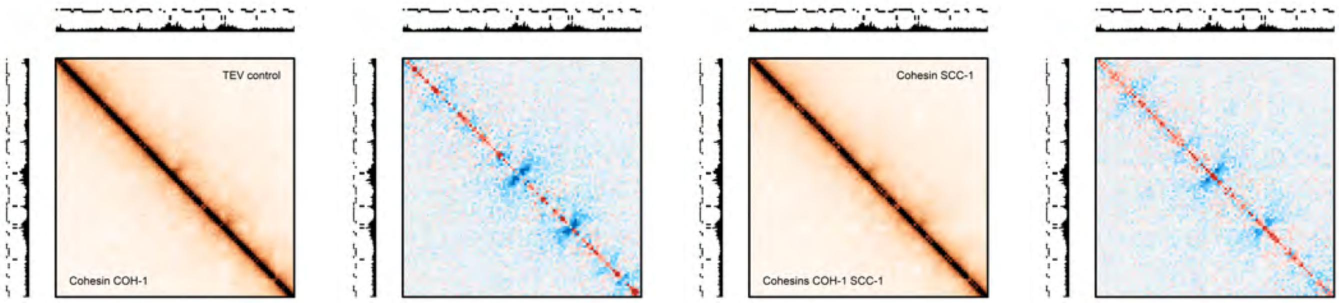

chrIII:12455001-12655000

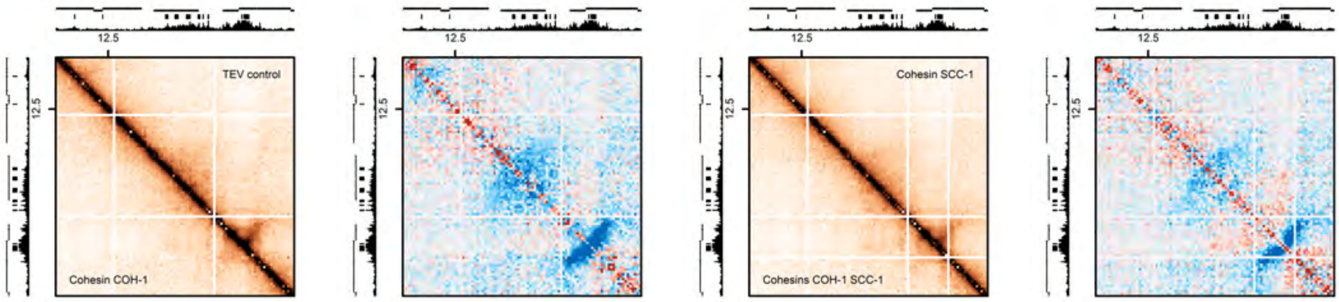

chrV:18683001-18883000

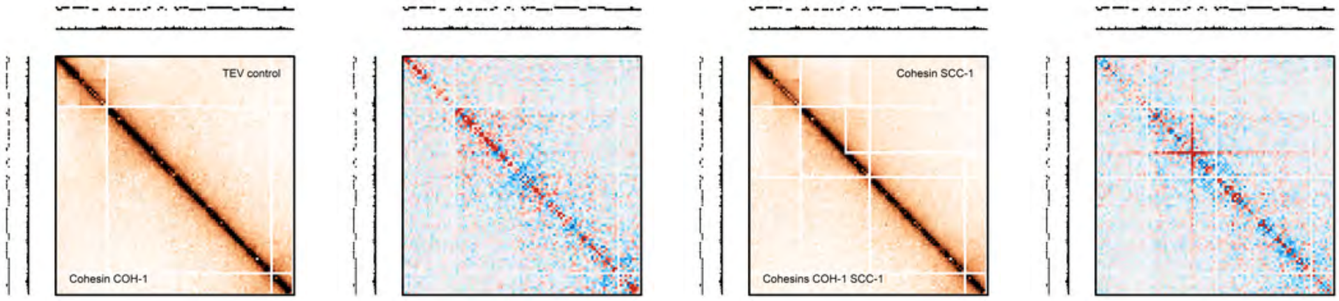

chrII:4193001-4393000

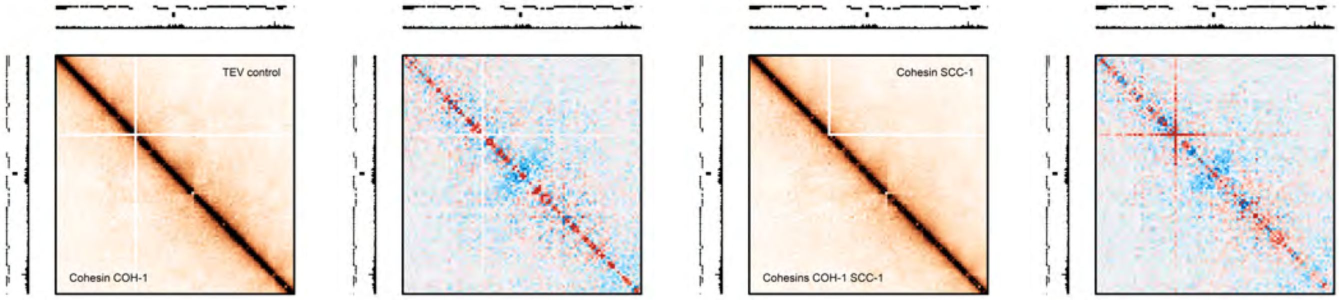

chrX:4863001-5063000

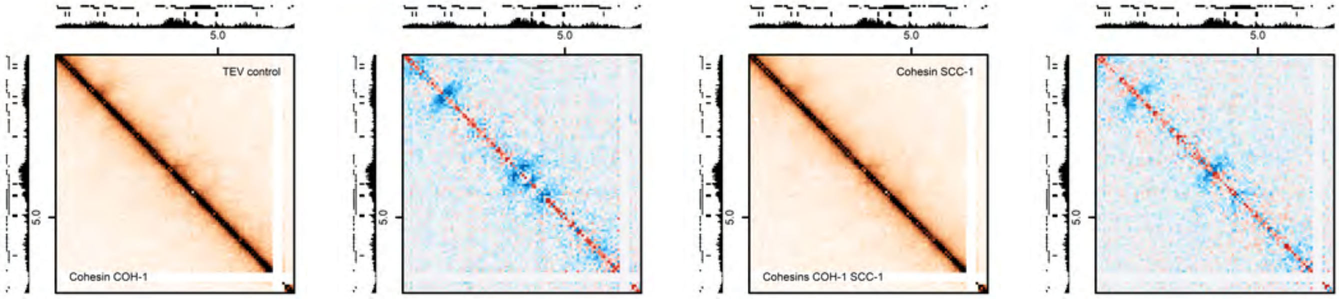

chrIII:8557001-8757000

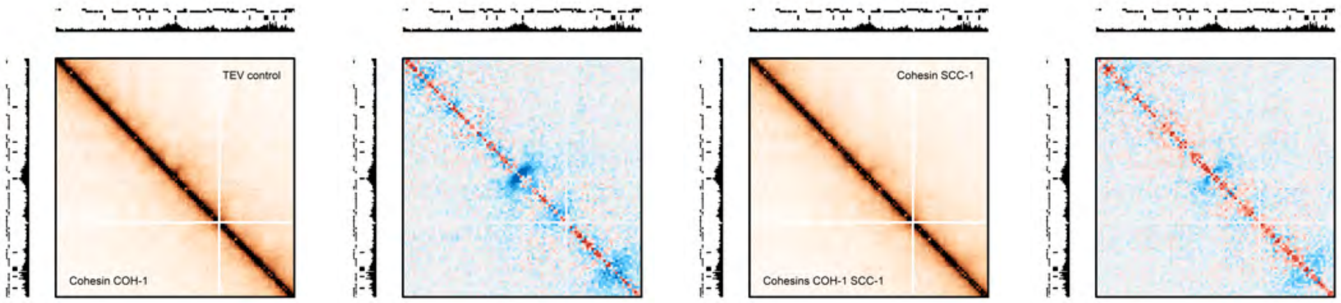

chrV:8987001-9187000

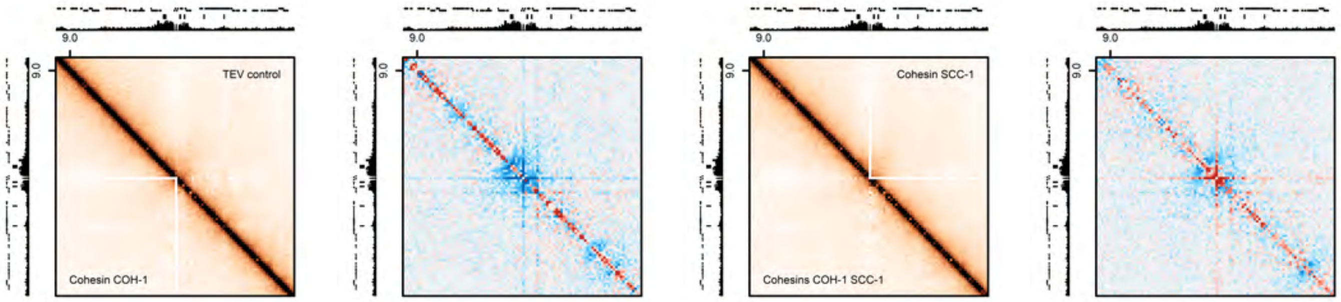

chrI:11477001-11677000

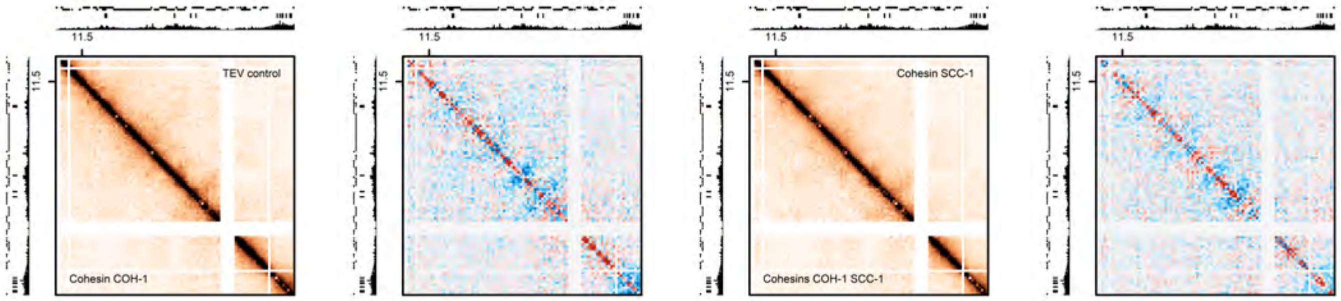

chrIV:8851001-9051000

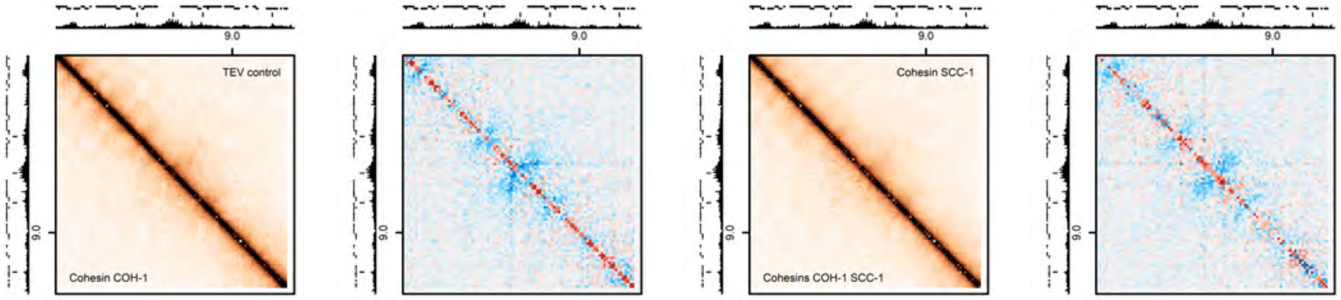

chrIII:9755001-9955000

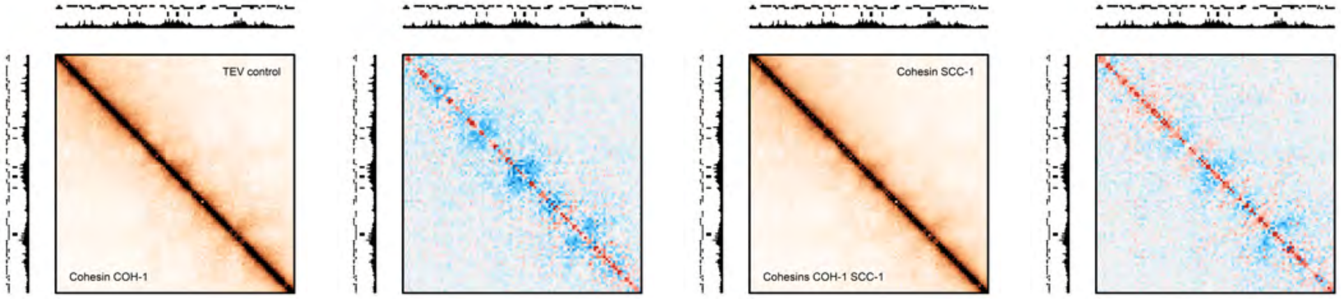

chrIII:8407001-8607000

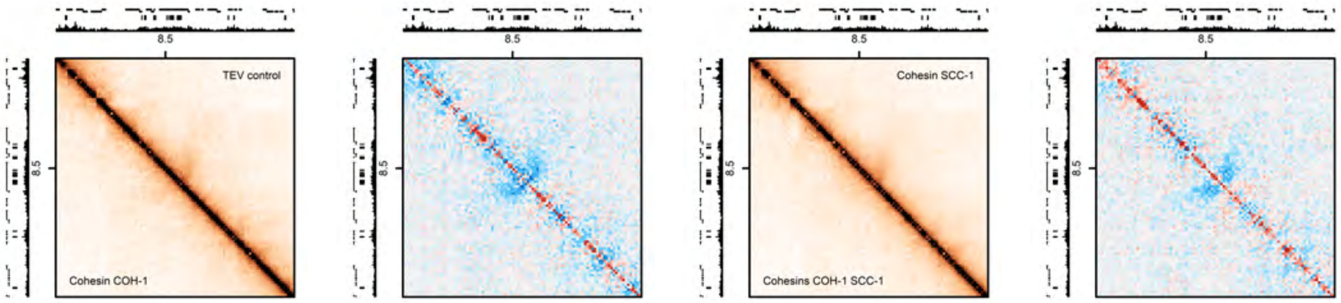

chrIII:8953001-9153000

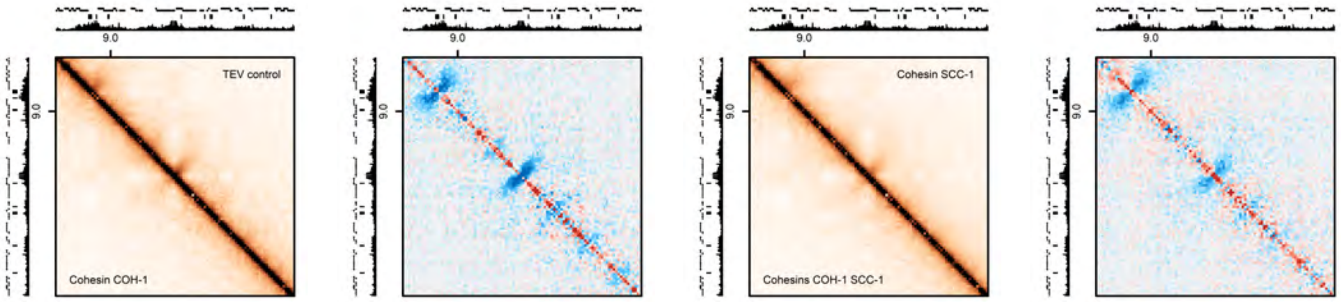

chrIV:16299001-16499000

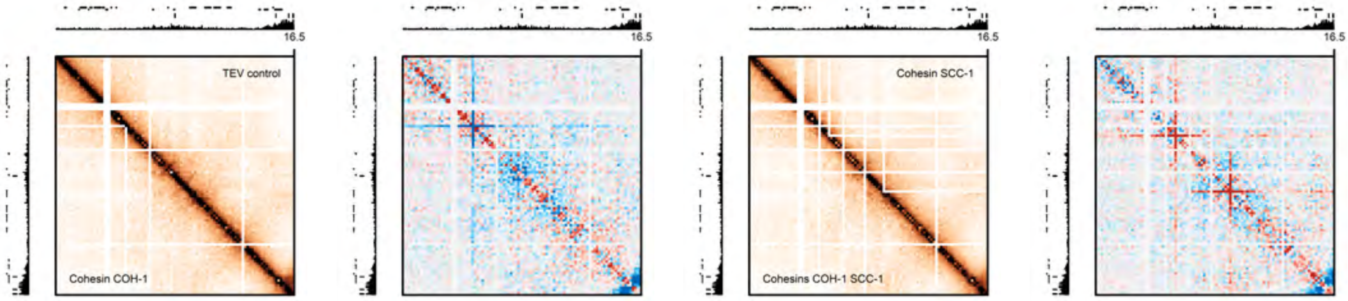

chrIII:10953001-11153000

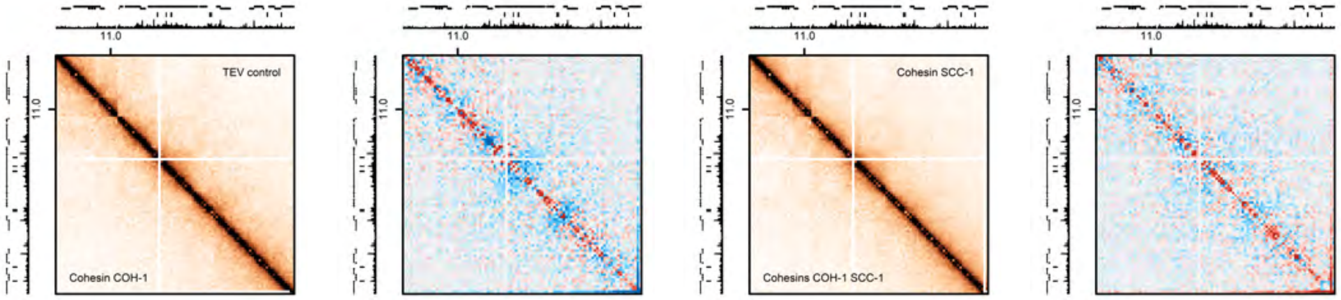

chrII:6515001-6715000

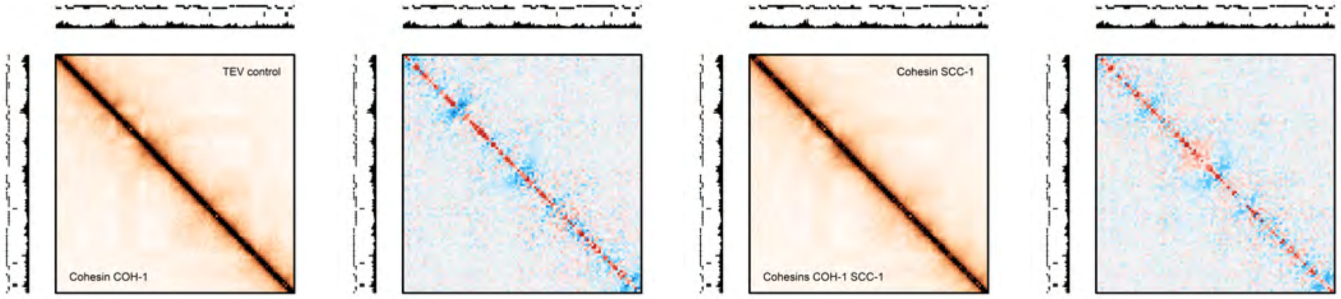

chrIII:4463001-4663000

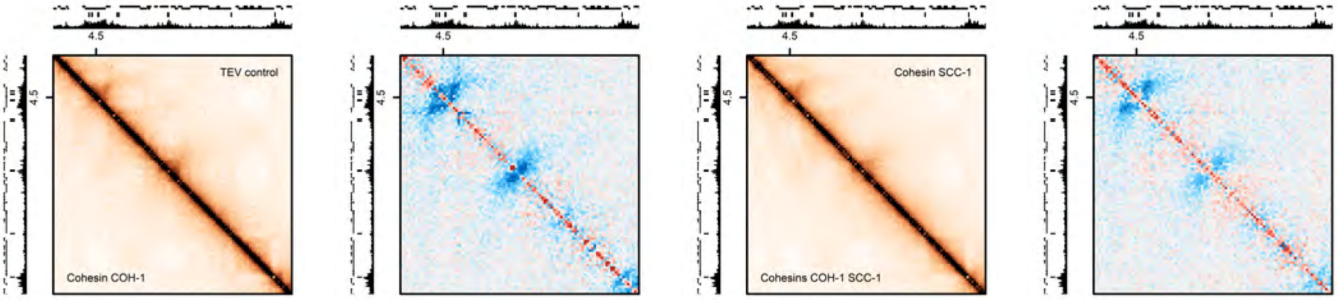

chrV:3293001-3493000

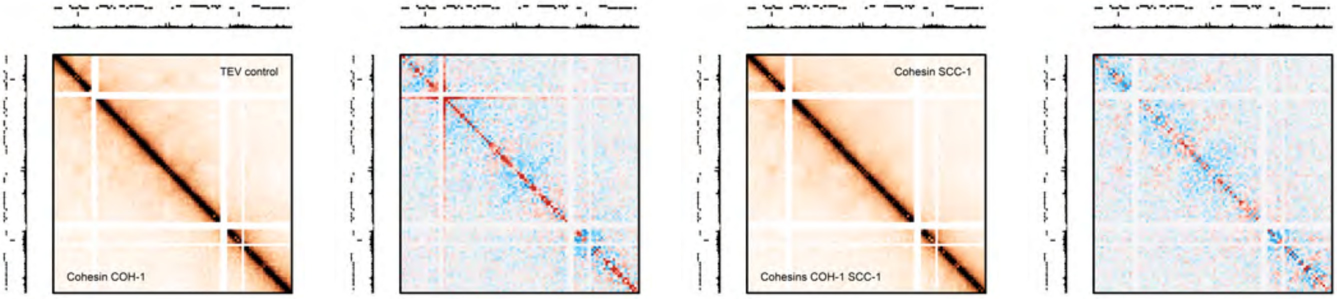

chrIII:2583001-2783000

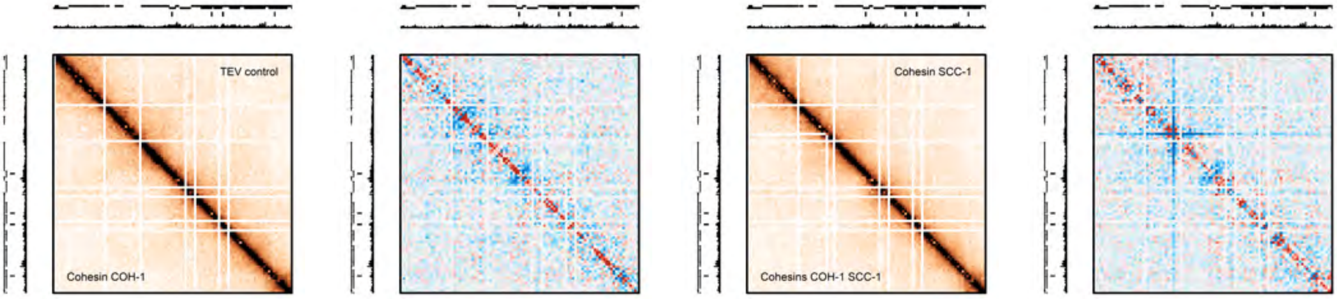

chrIV:2183001-2383000

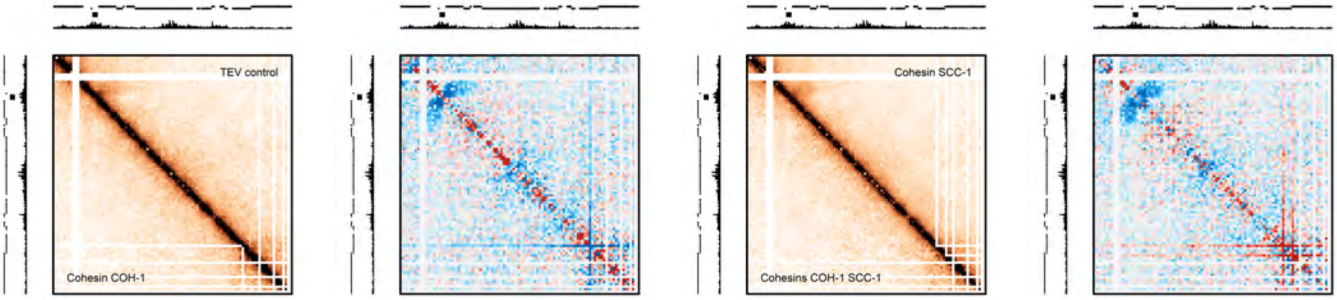

chrI:8687001-8887000

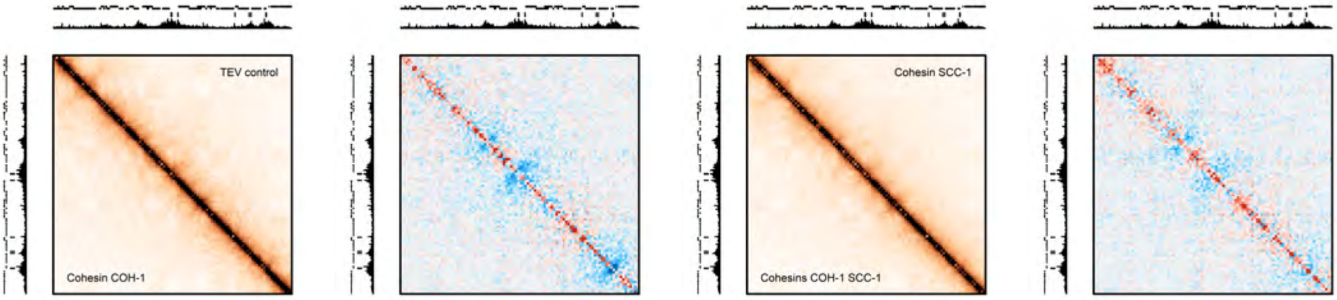

chrX:17533001-17733000

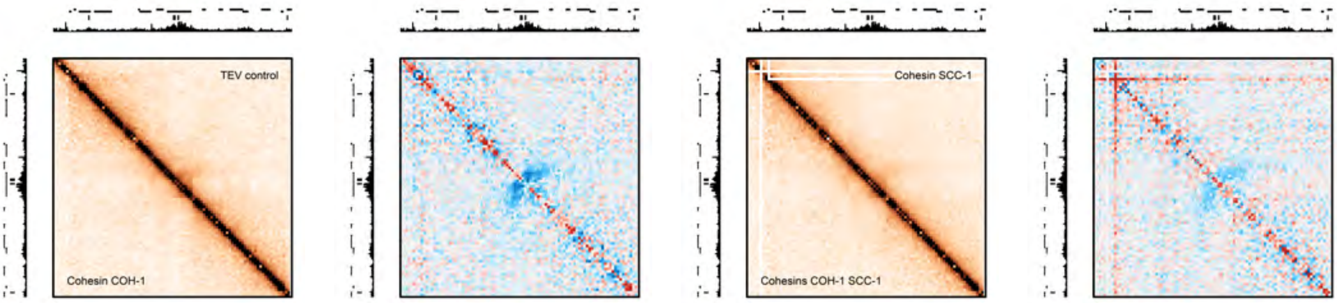

chrII:7917001-8117000

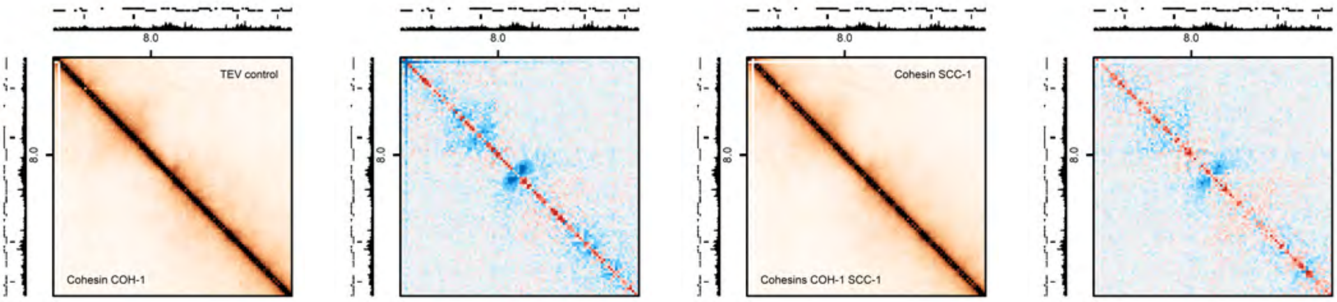

chrII:14305001-14505000

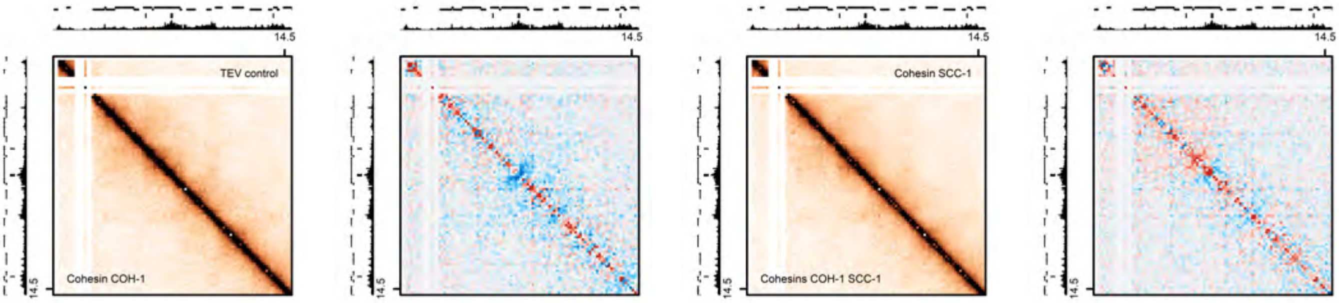

chrV:6251001-6451000

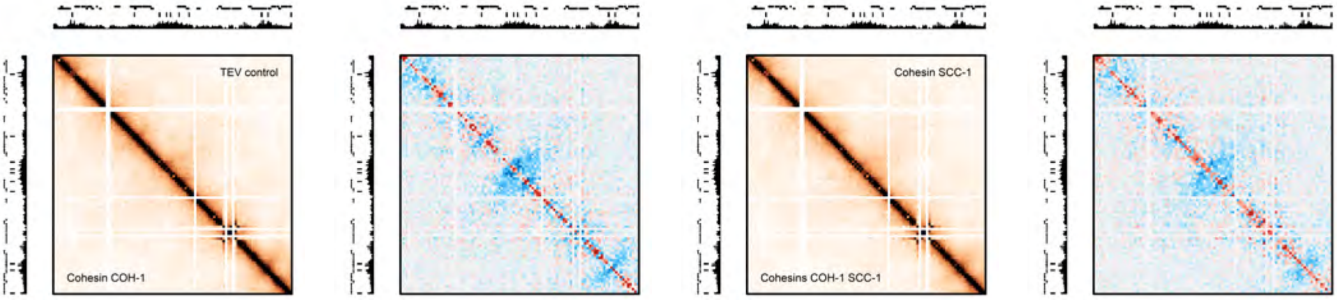

chrIV:15917001-16117000

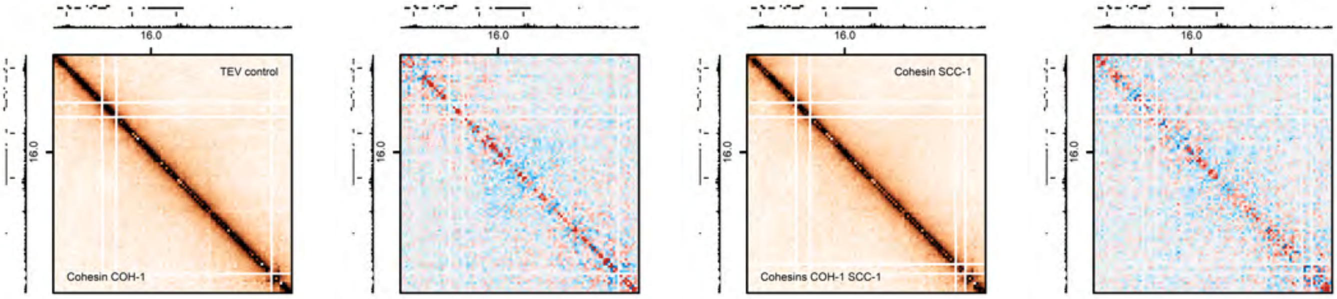

chrX:13849001-14049000

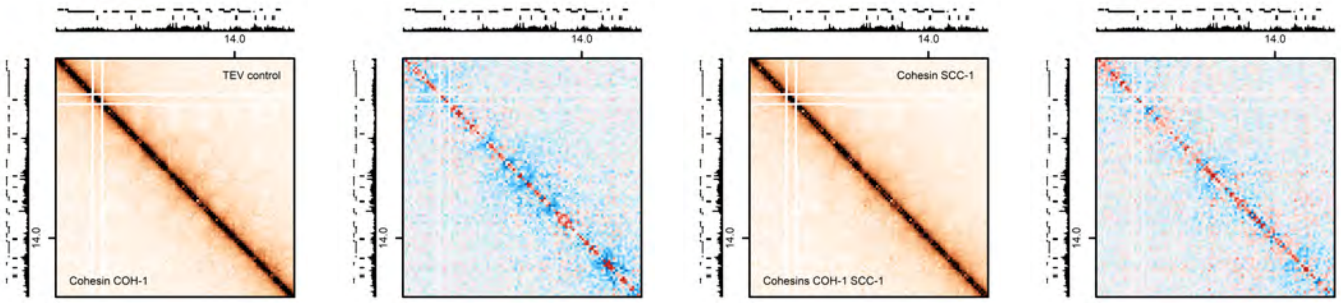

chrIII:5841001-6041000

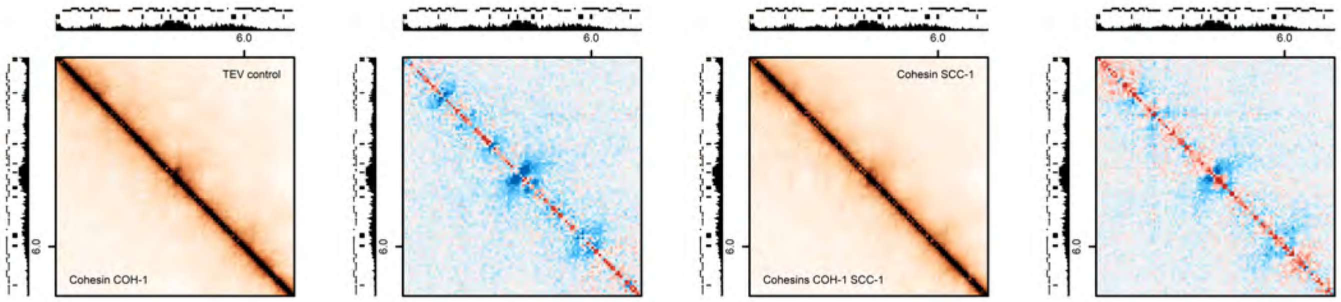

chrIV:7745001-7945000

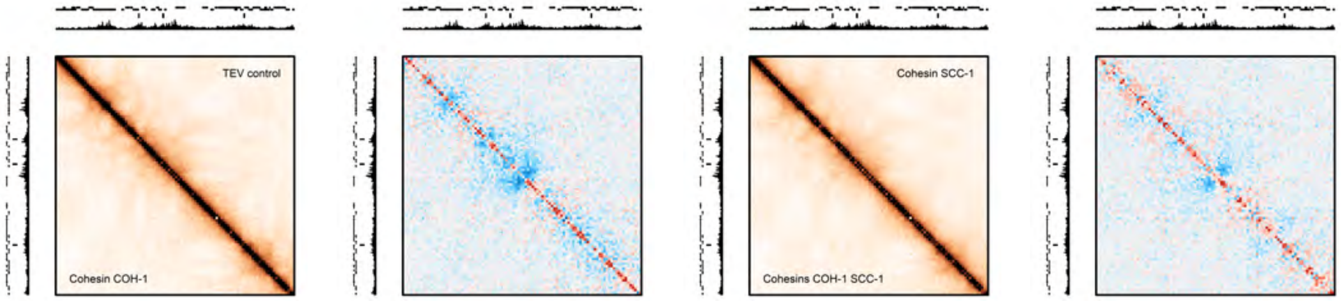

chrIV:201001-401000

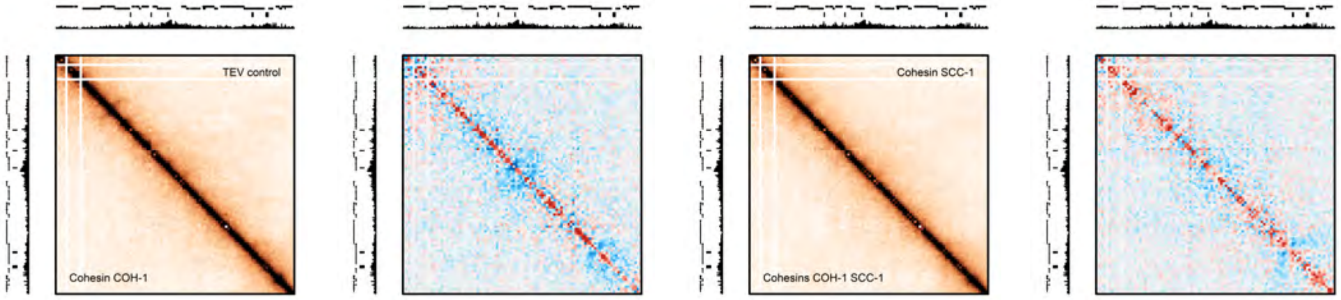

chrX:1355001-1555000

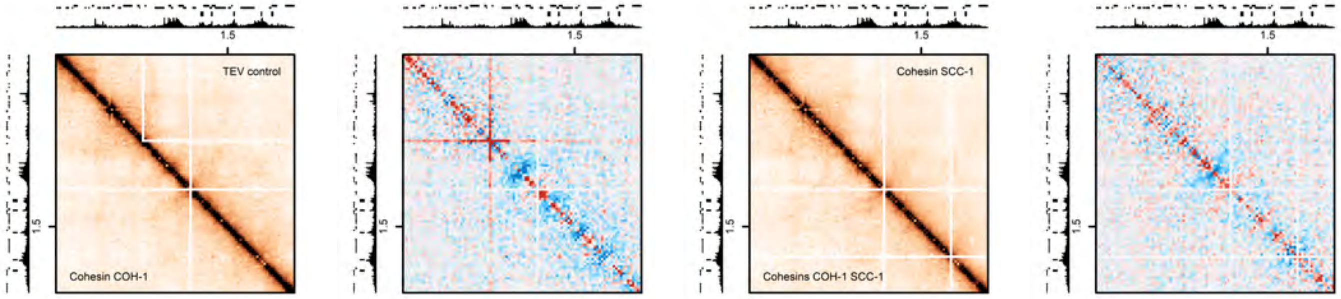

chrIII:8195001-8395000

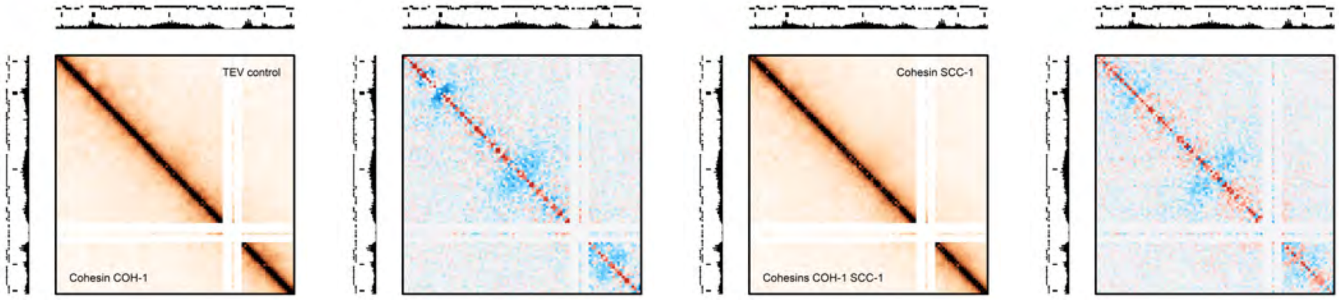

chrIII:12235001-12435000

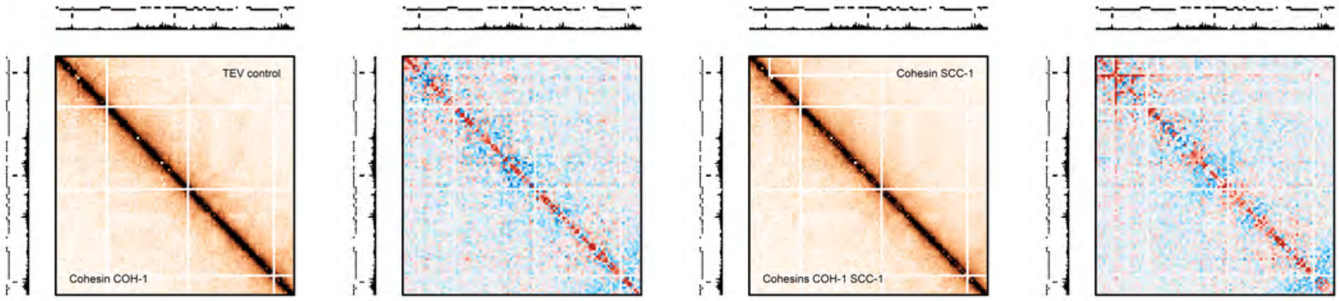

chrV:4961001-5161000

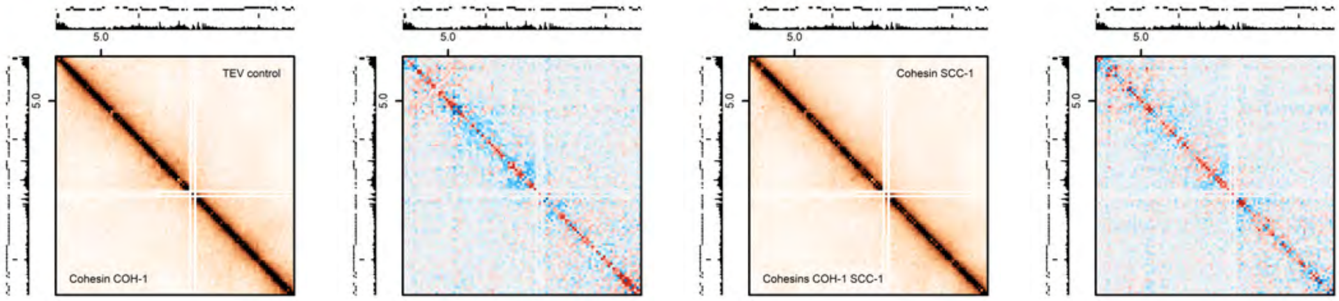

chrV:1373001-1573000

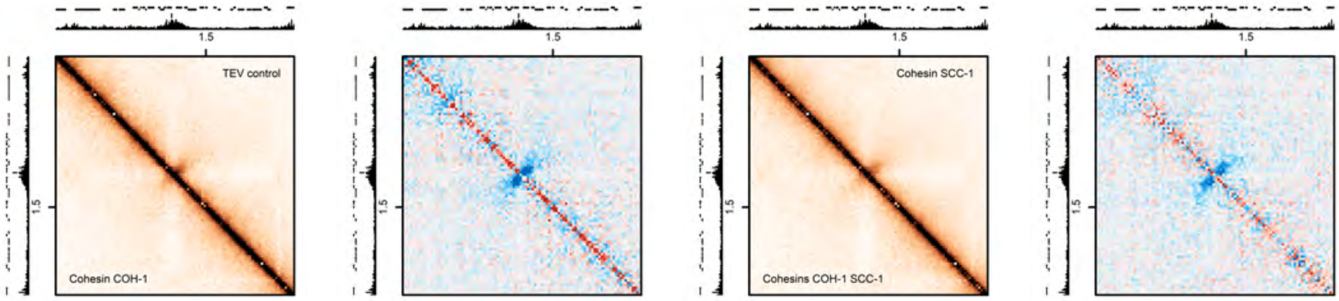

chrV:18477001-18677000

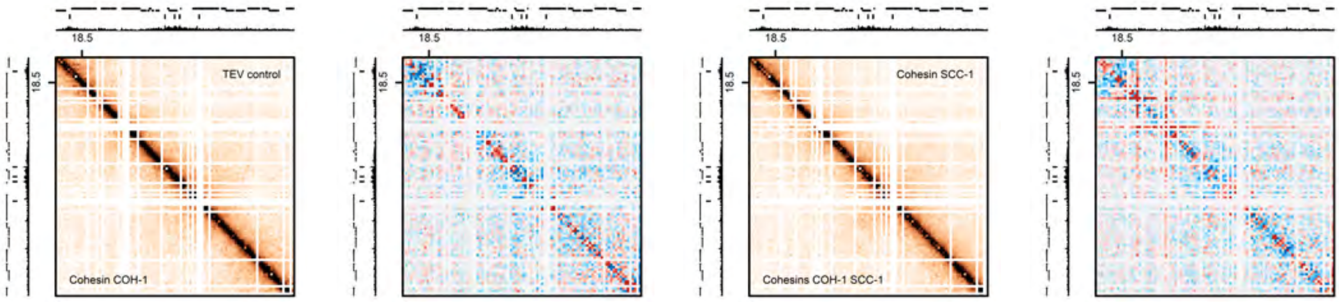

chrI:5591001-5791000

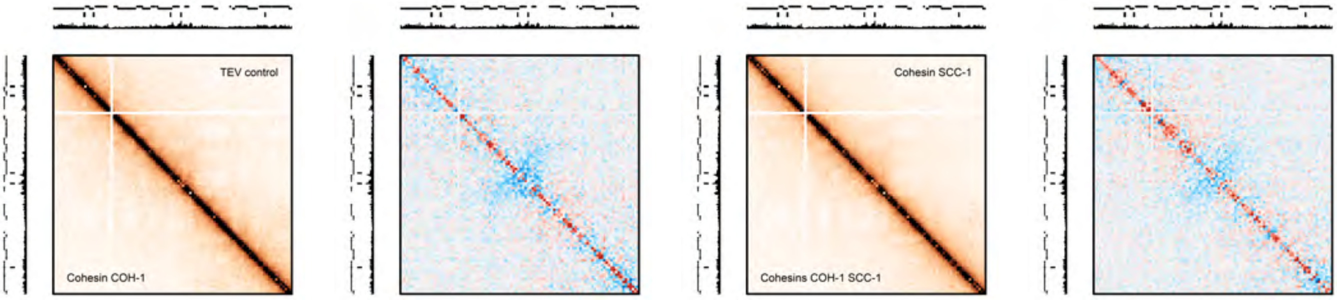

chrIV:8531001-8731000

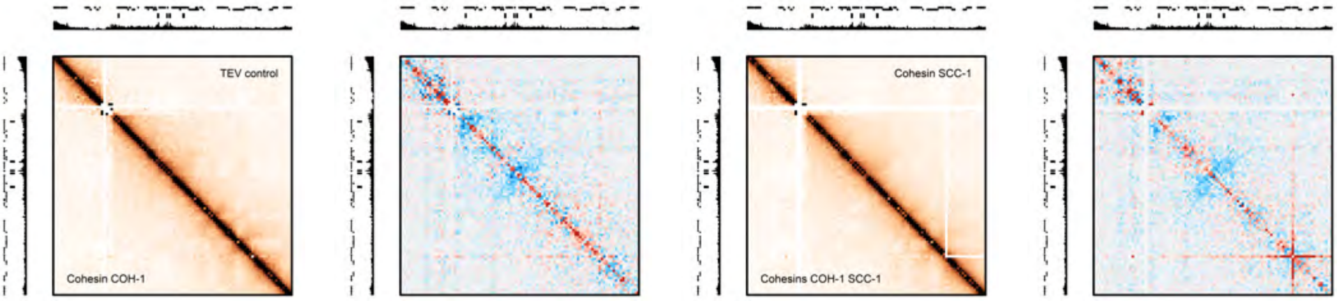

chrX:16051001-16251000

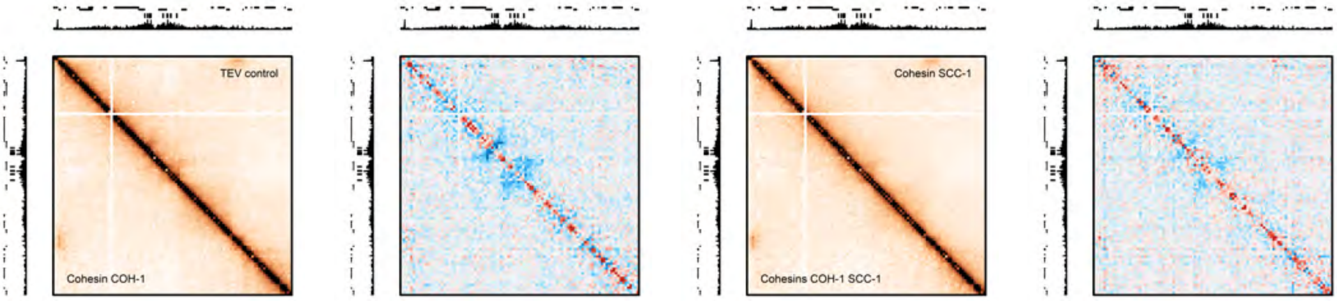

chrIV:8335001-8535000

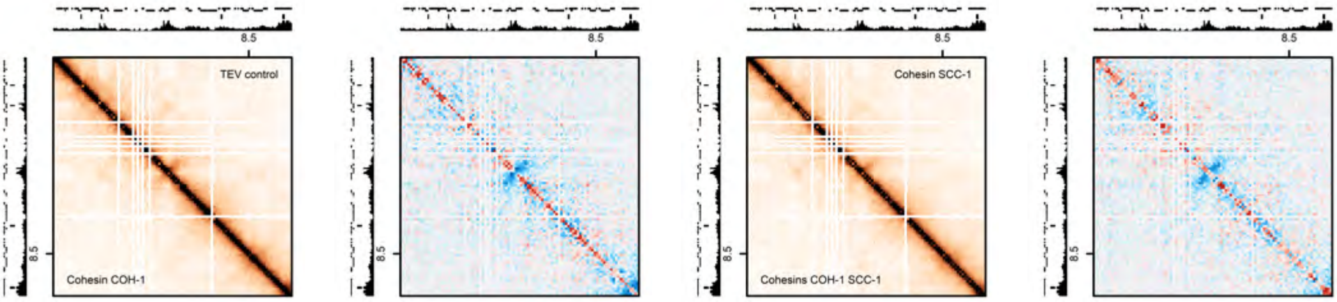

chrIII:13175001-13375000

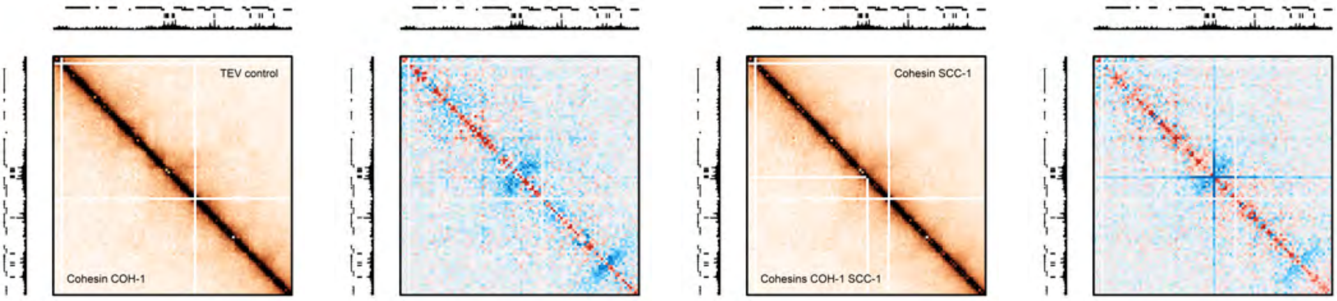

chrIV:9775001-9975000

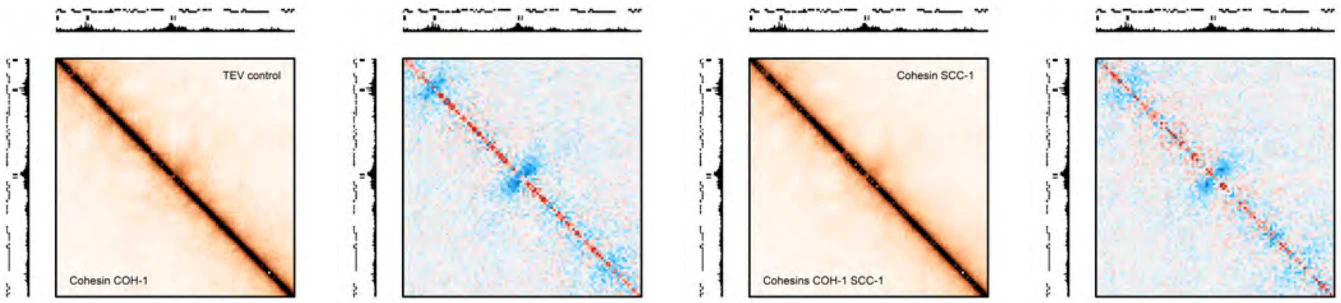

chrV:14973001-15173000

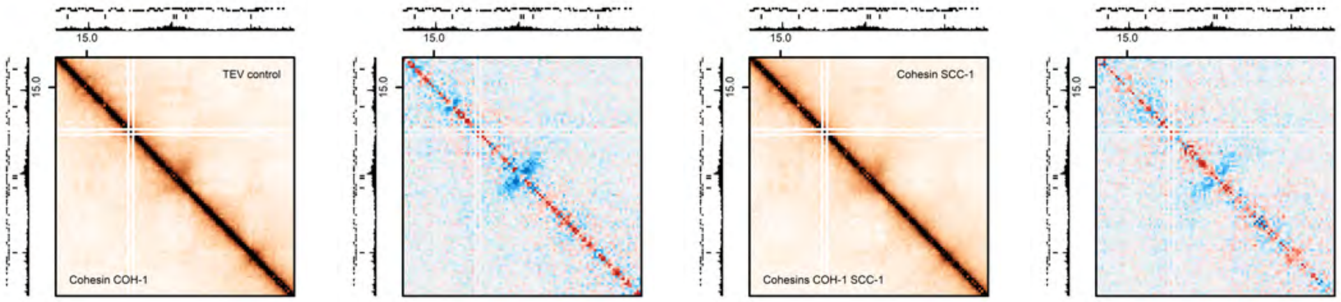

chrII:9389001-9589000

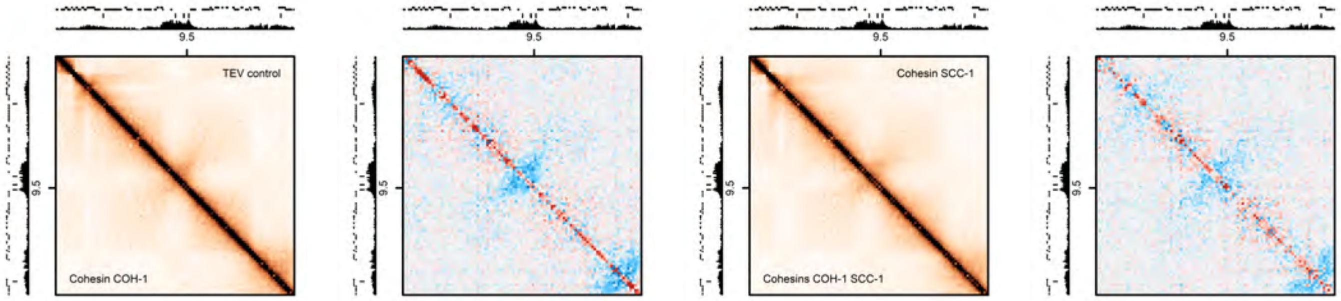

chrII:12565001-12765000

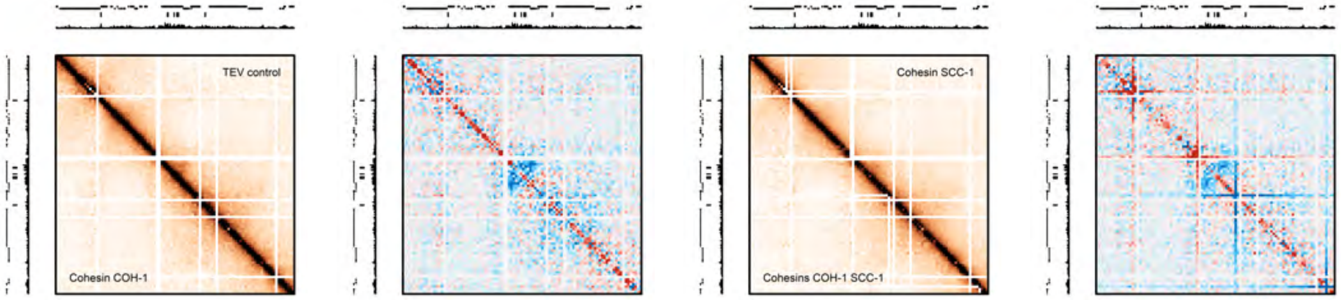

chrI:4215001-4415000

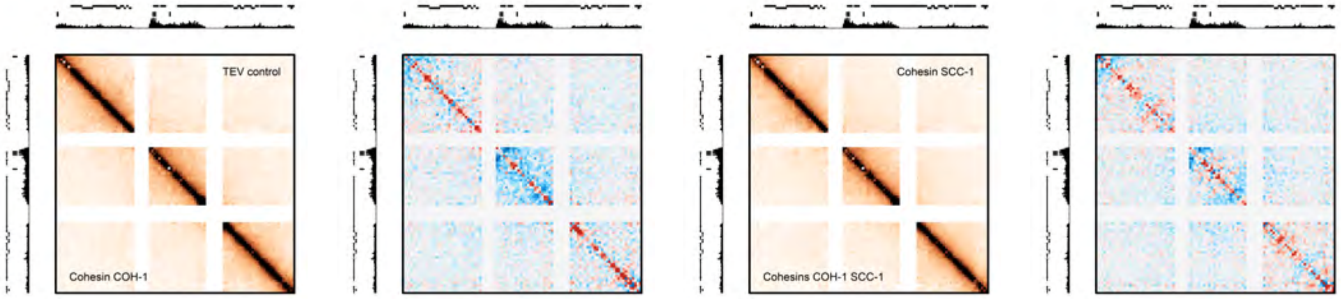

chrIV:4303001-4503000

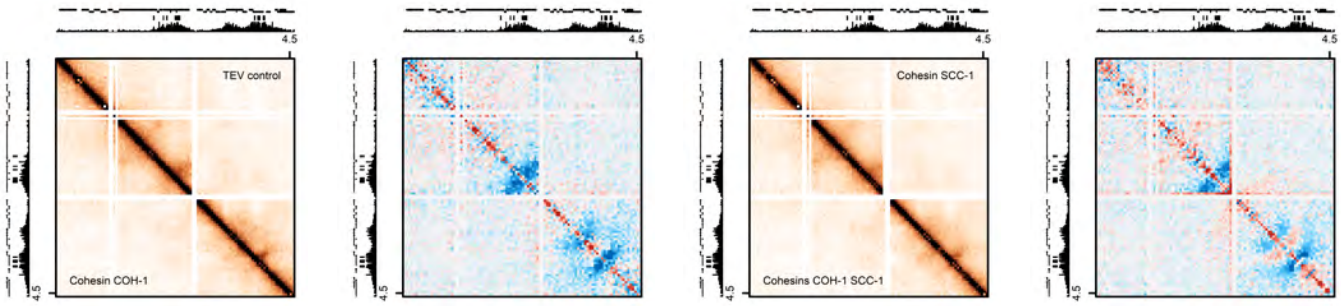

chrI:8765001-8965000

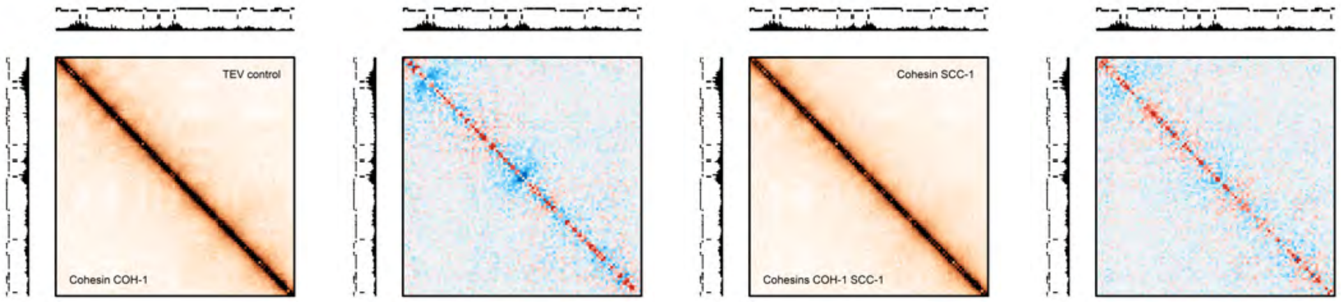

chrIV:12319001-12519000

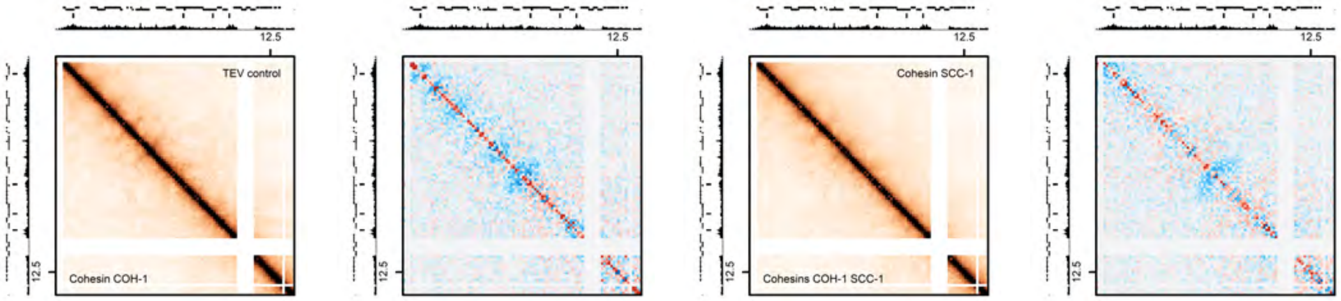

chrI:199001-399000

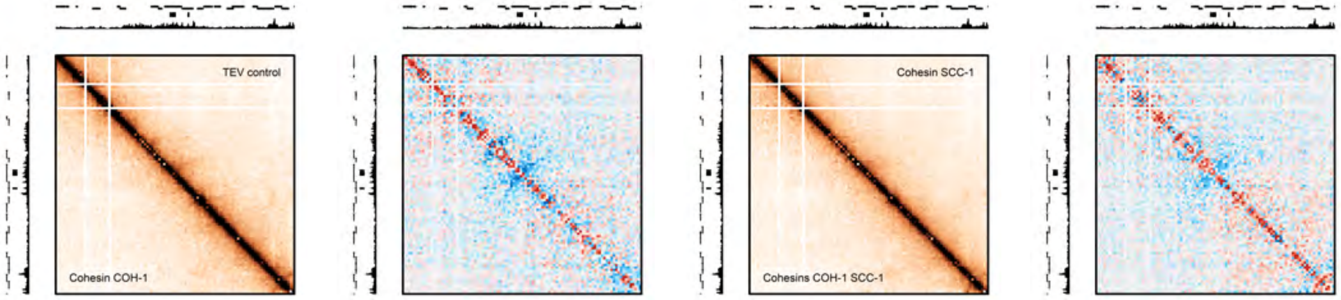

chrX:11143001-11343000

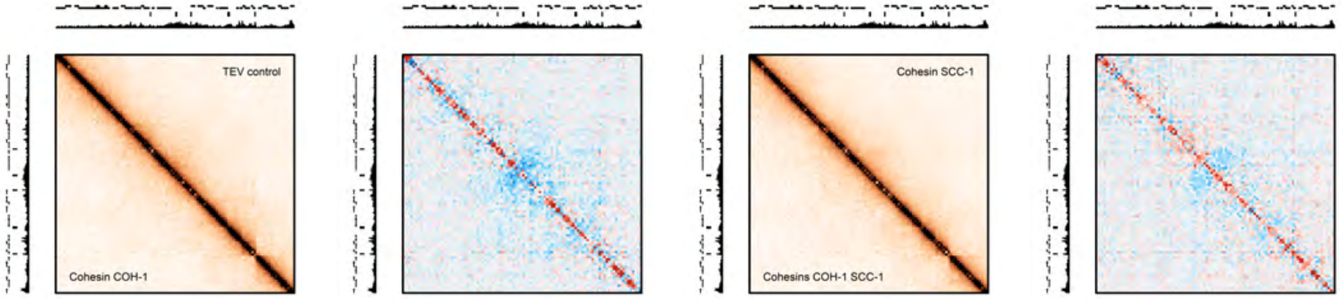

chrII:3169001-3369000

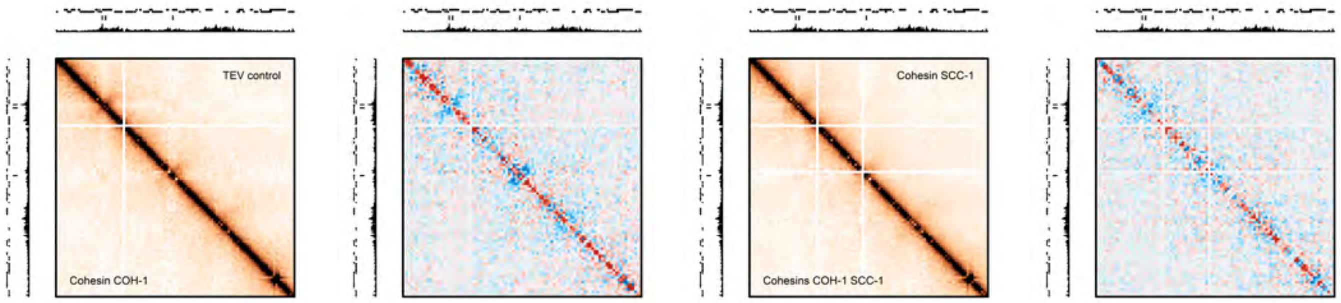

chrII:13571001-13771000

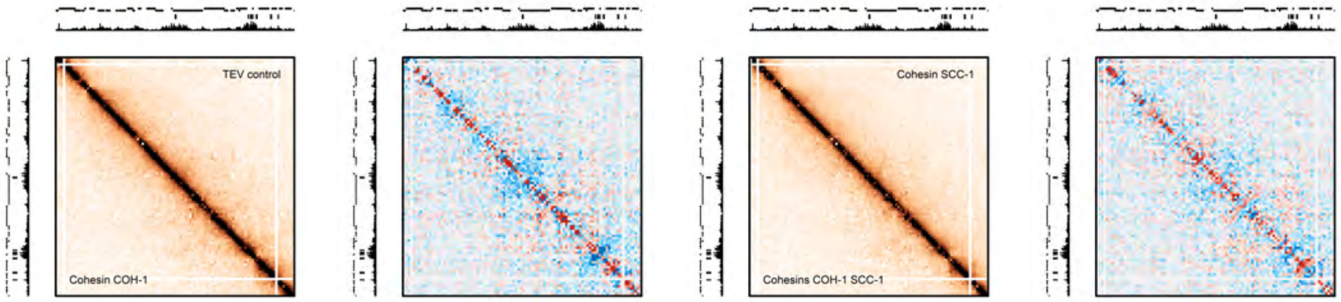

chrV:6561001-6761000

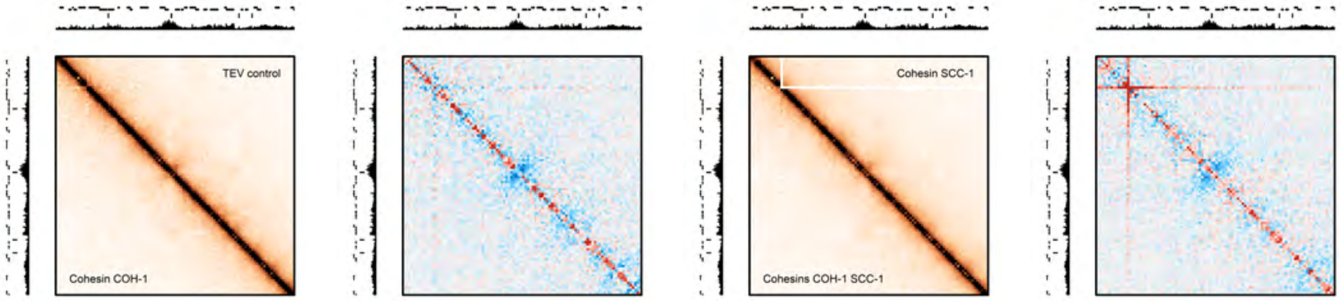

chrIV:15299001-15499000

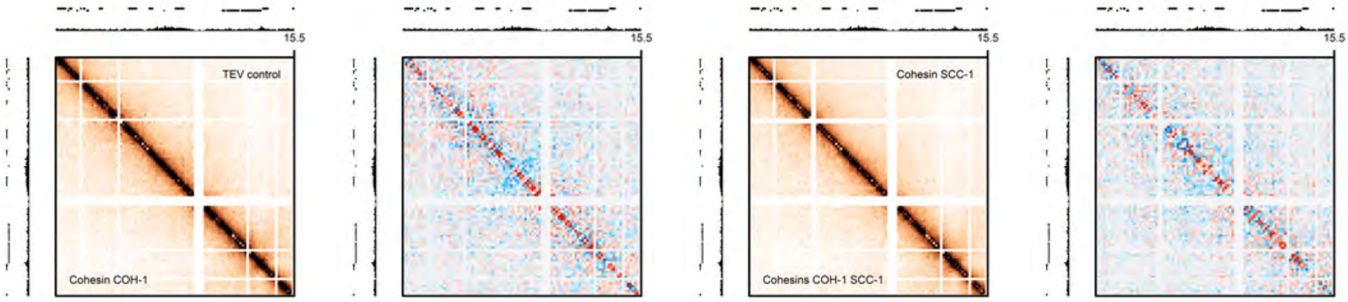

chrII:5603001-5803000

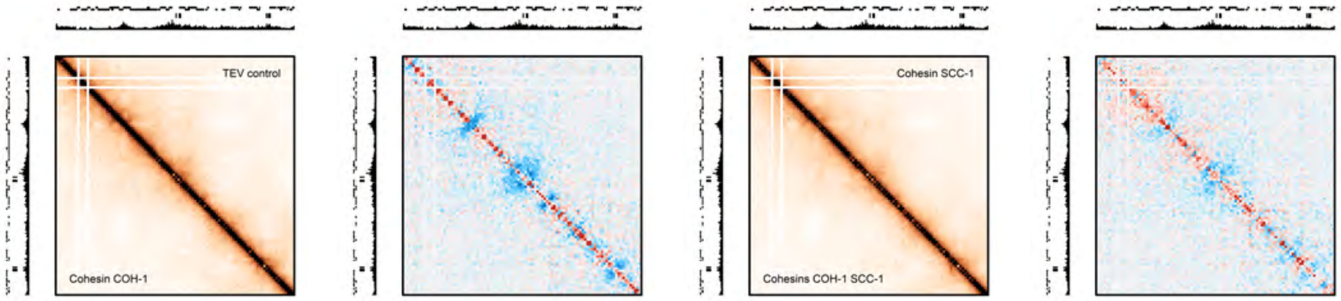

chrIII:11917001-12117000

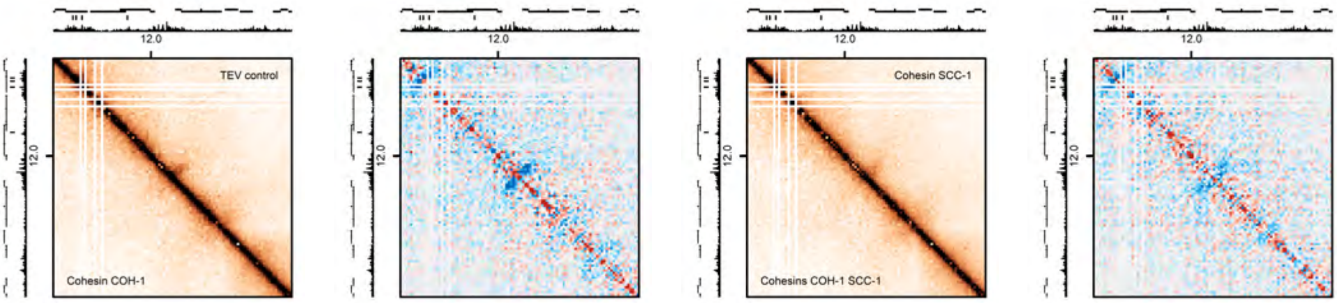

chrIV:1355001-1555000

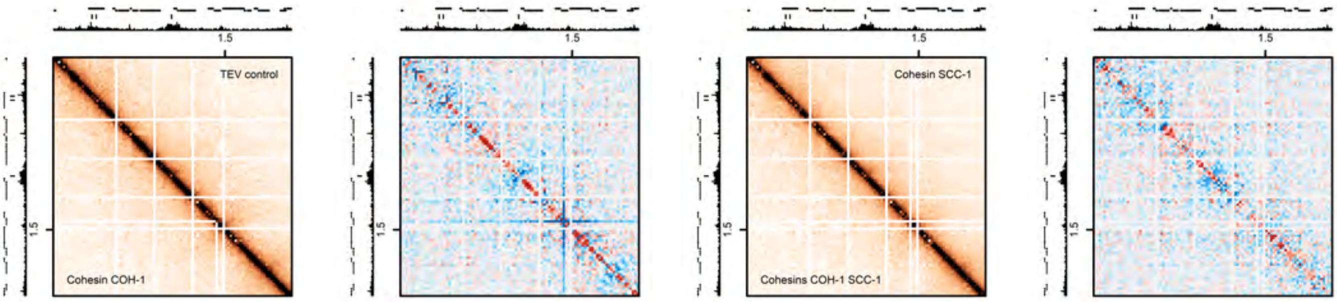

chrIV:10153001-10353000

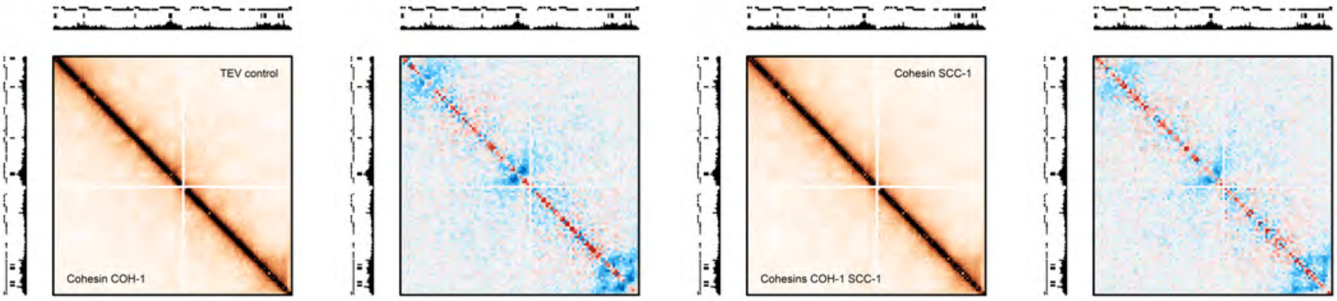

chrX:7715001-7915000

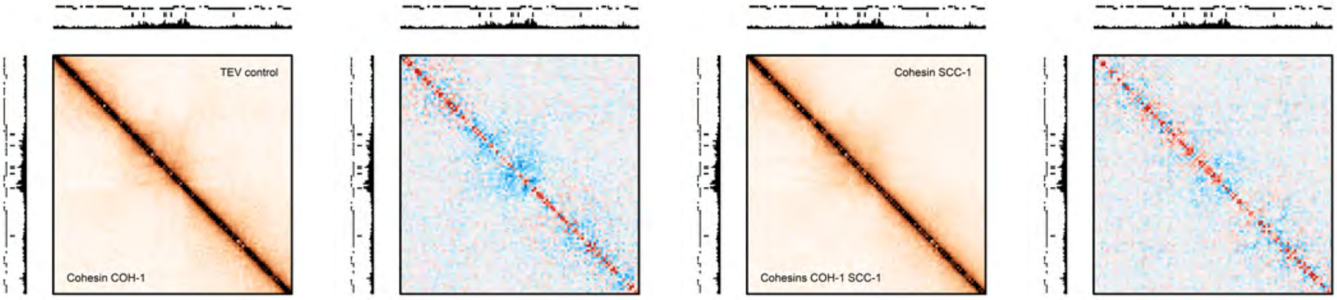

chrIV:16999001-17199000

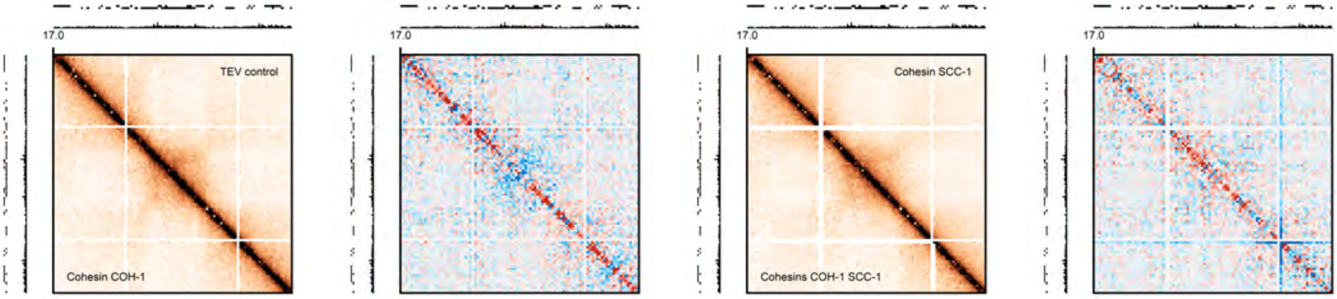

chrI:8031001-8231000

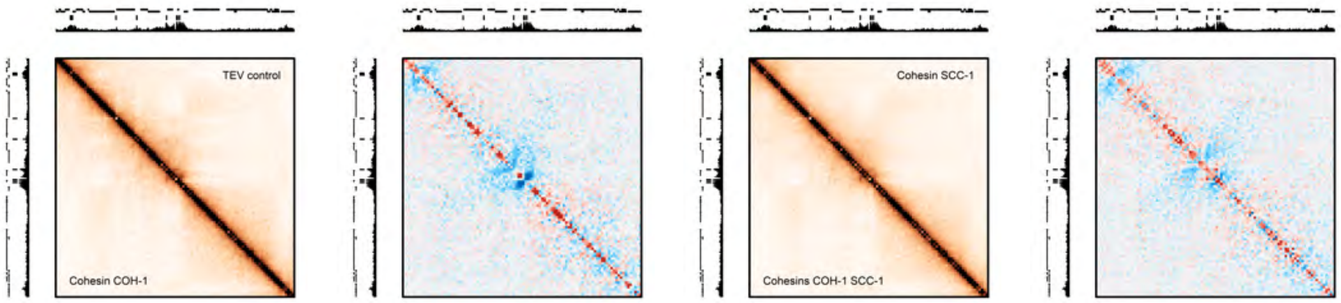

chrII:2095001-2295000

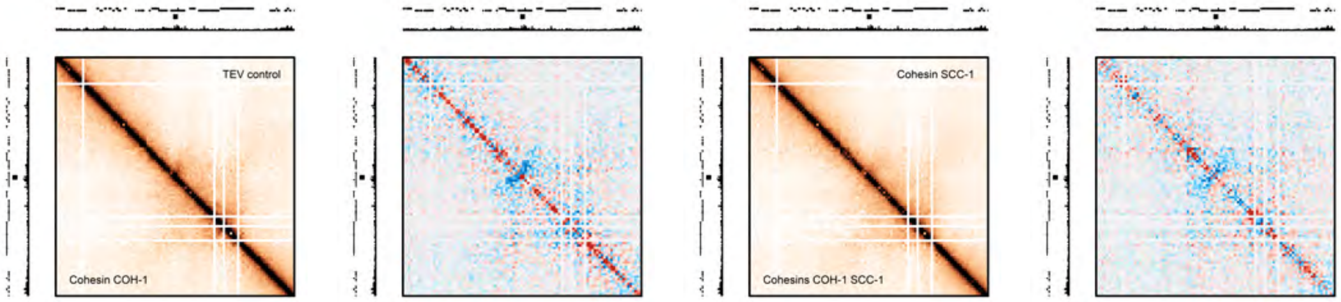

chrIII:10617001-10817000

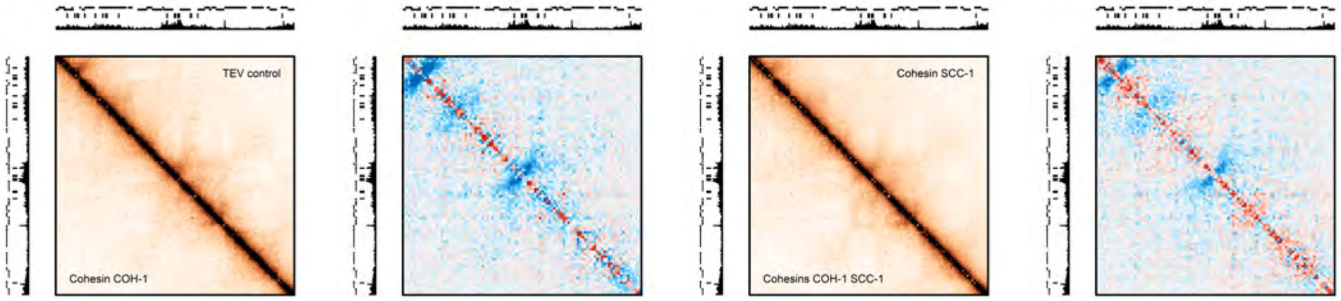

chrI:5899001-6099000

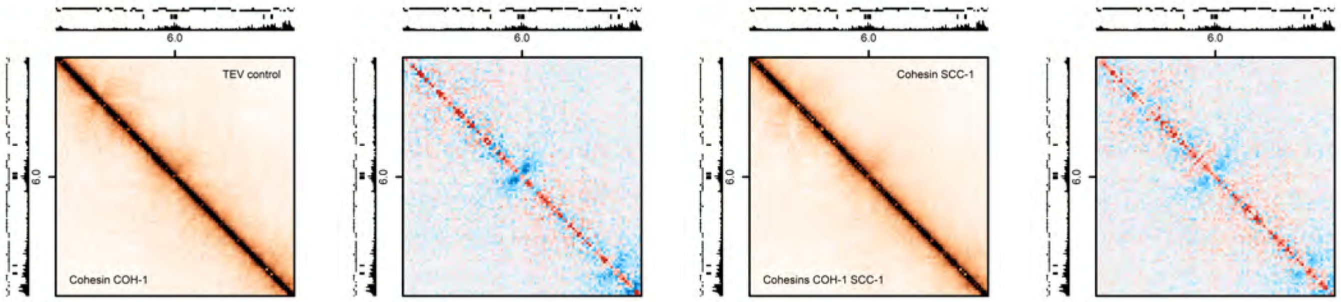

chrIII:3777001-3977000

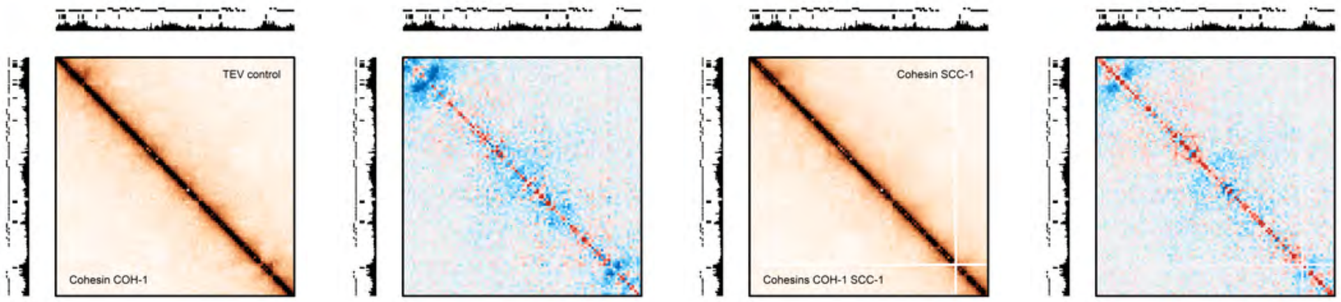

chrIV:3717001-3917000

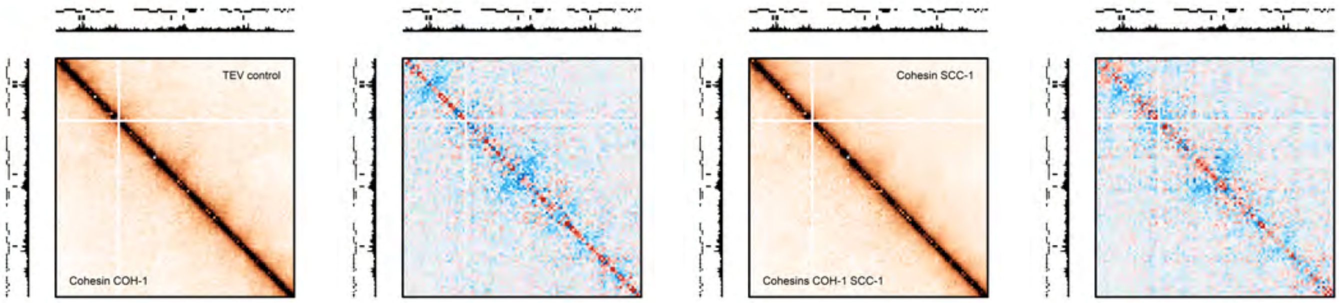

chrI:6245001-6445000

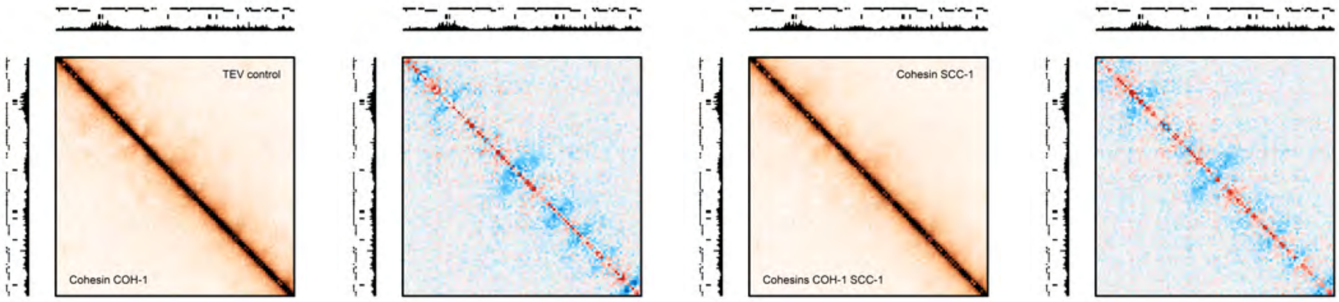

chrIV:1949001-2149000

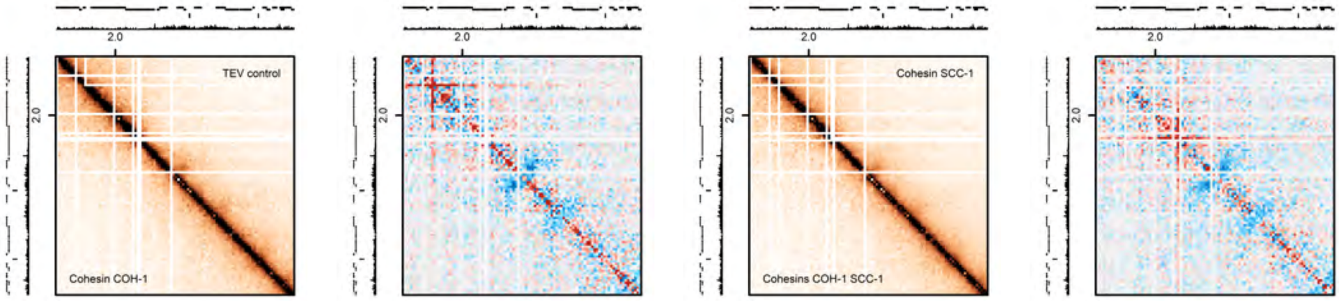

chrIV:11663001-11863000

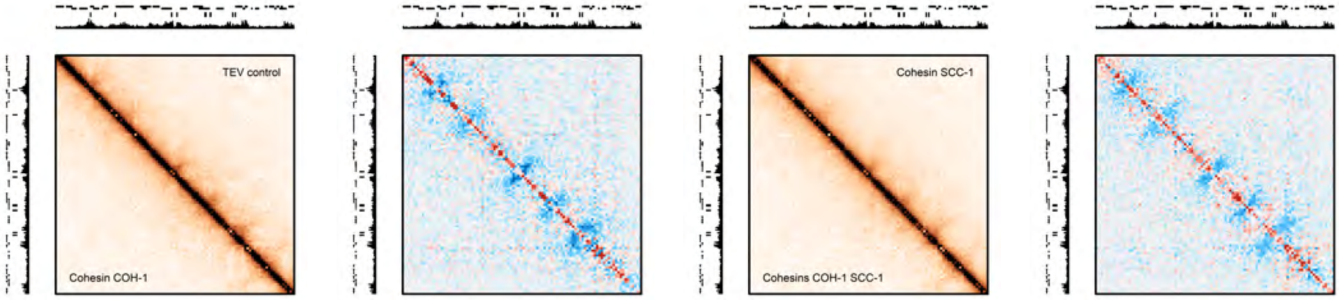

chrII:12251001-12451000

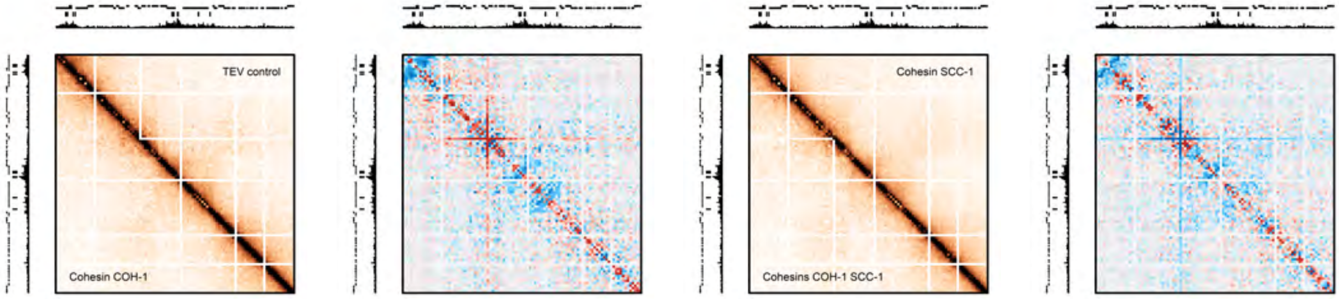

chrI:337001-537000

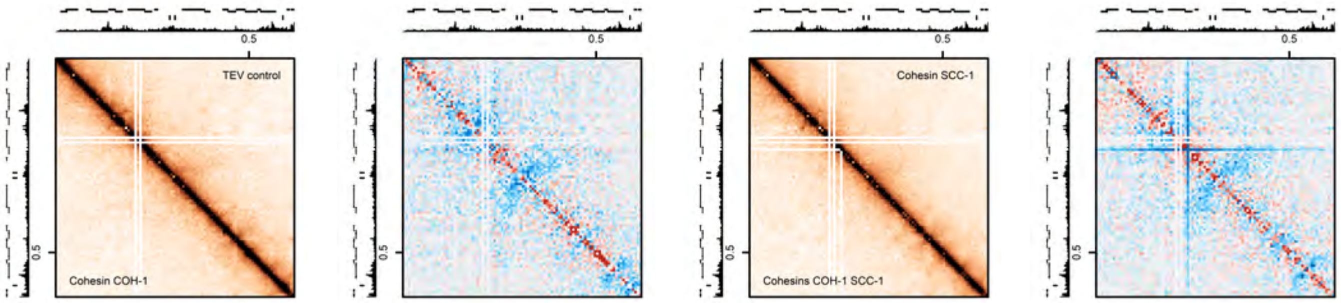

chrI:11773001-11973000

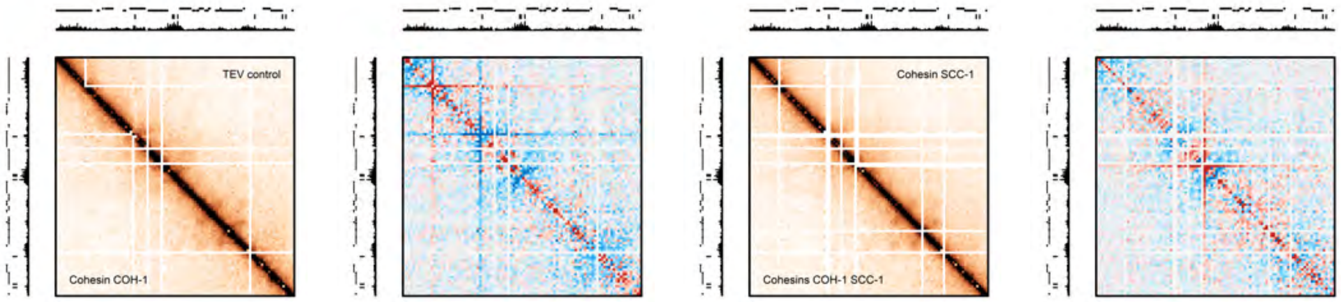

chrIII:11639001-11839000

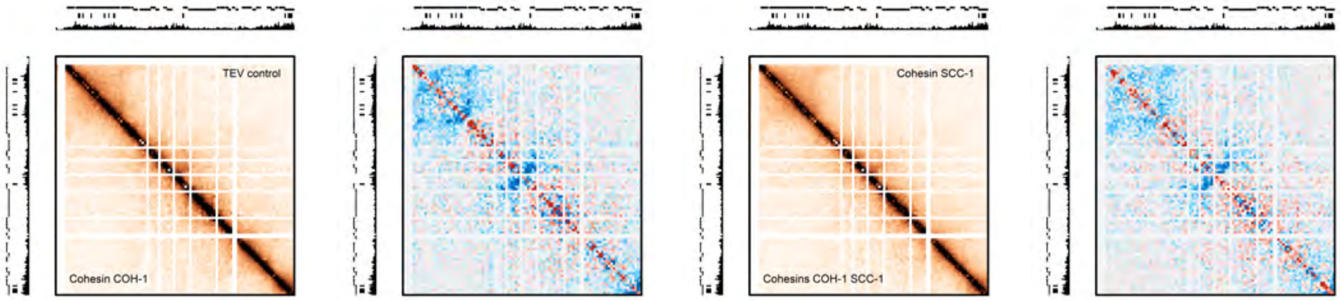

chrI:7915001-8115000

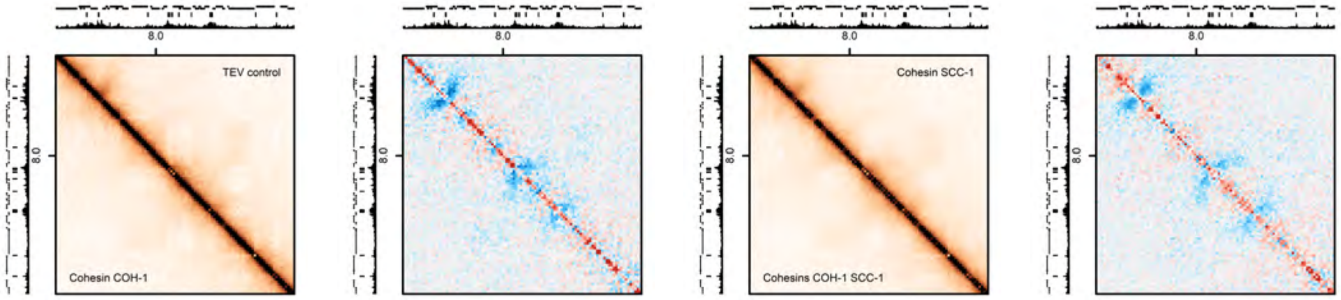

chrX:5379001-5579000

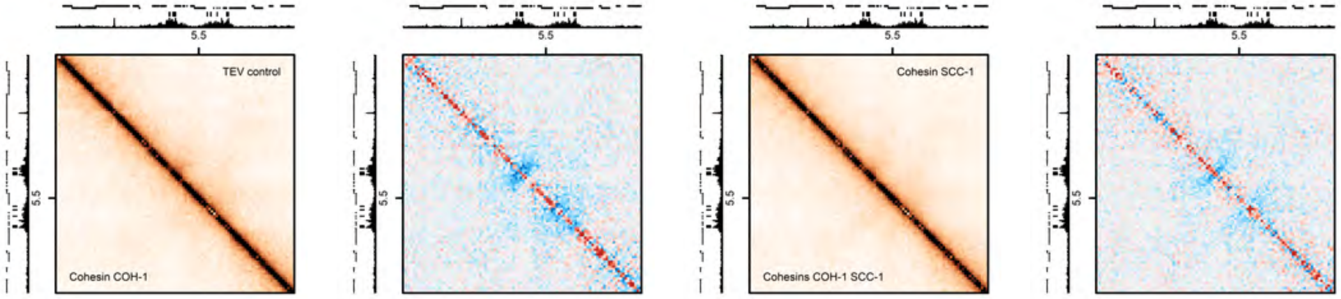

chrII:4477001-4677000

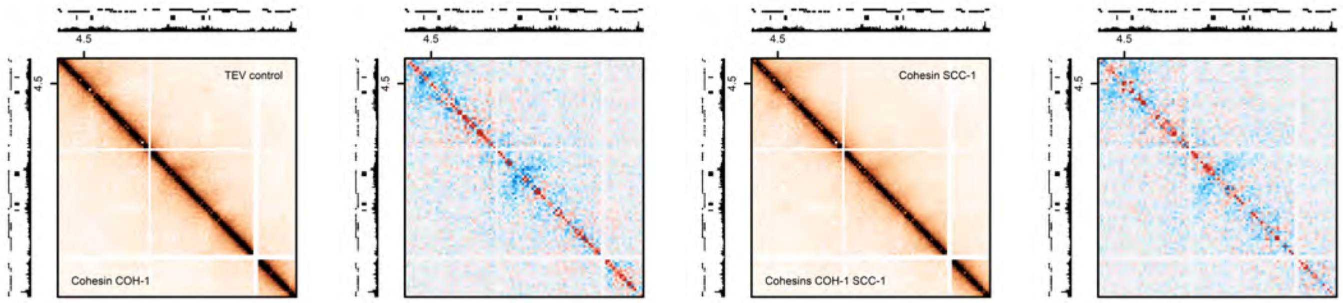

chrI:8311001-8511000

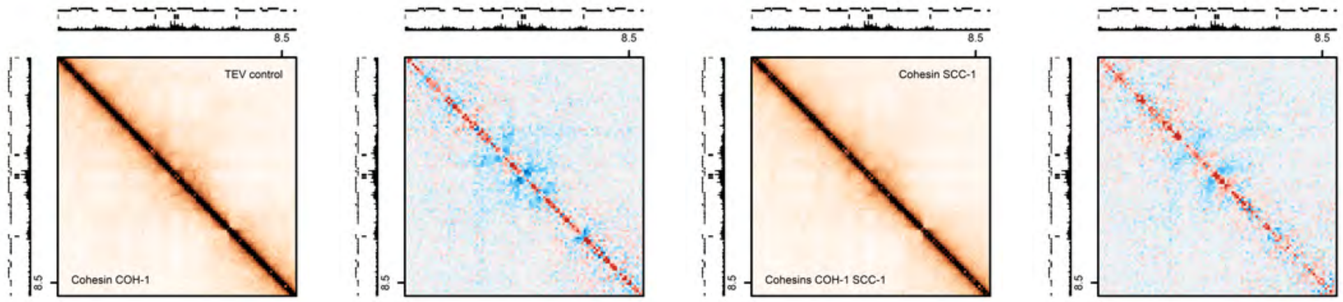

chrIV:10833001-11033000

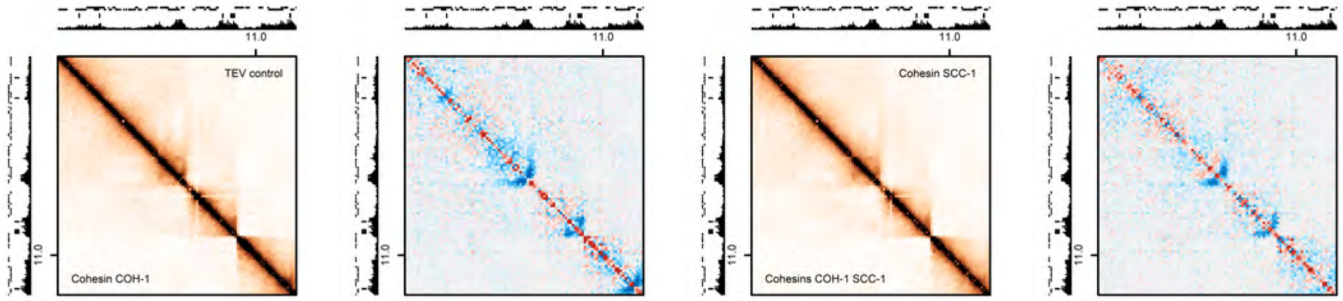

chrIV:16563001-16763000

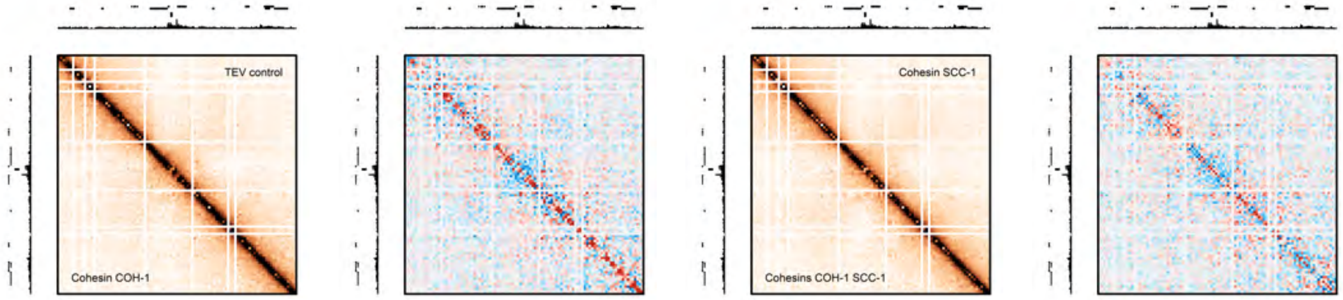

chrIII:13429001-13629000

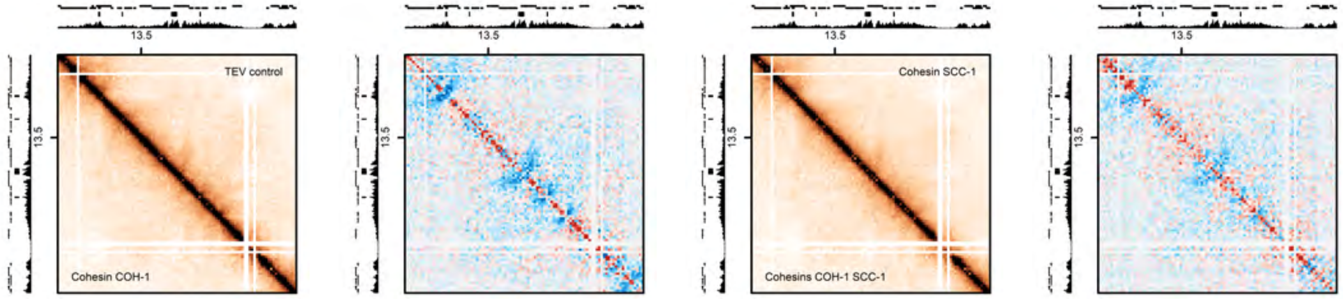

chrIII:3207001-3407000

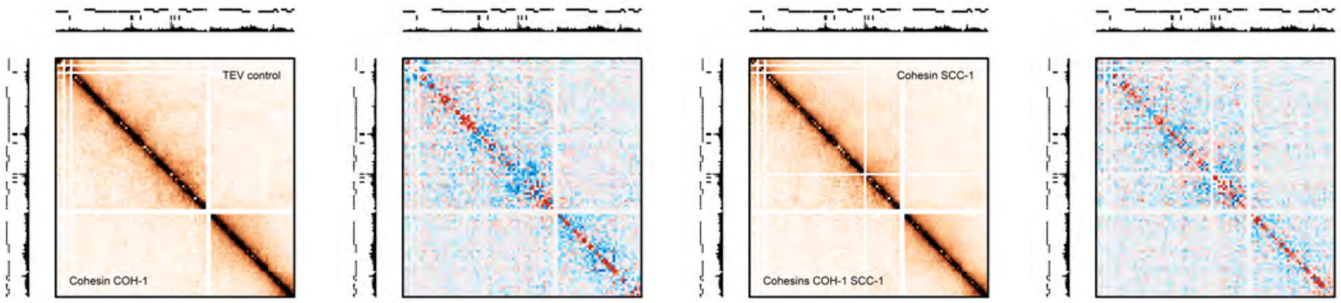

chrIII:6765001-6965000

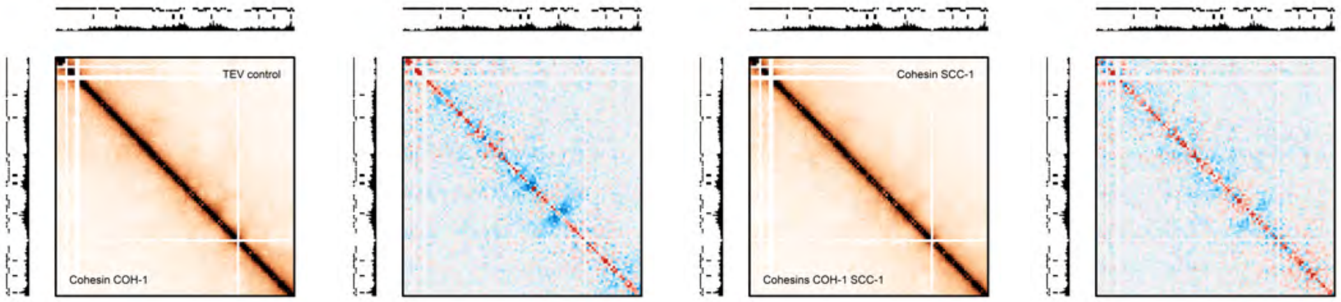

chrIII:2069001-2269000

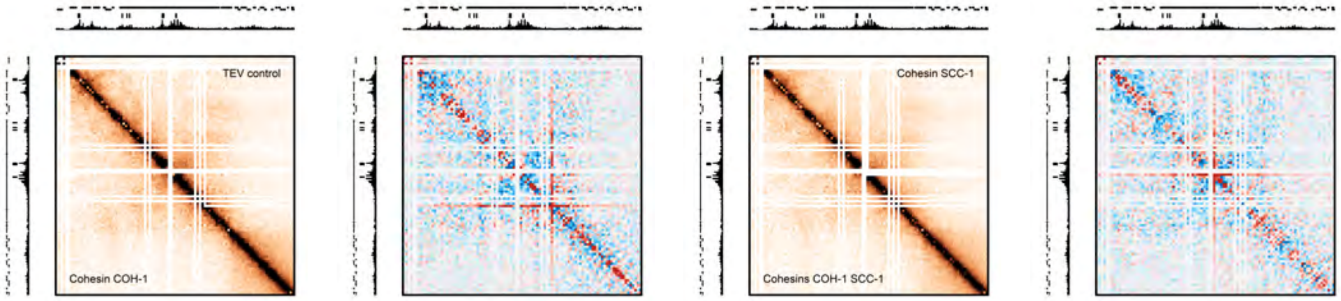

chrII:15161001-15361000

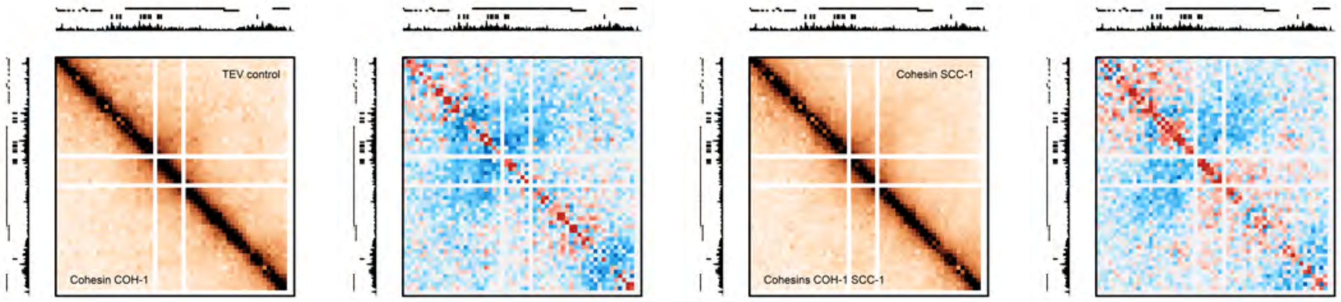

chrI:6785001-6985000

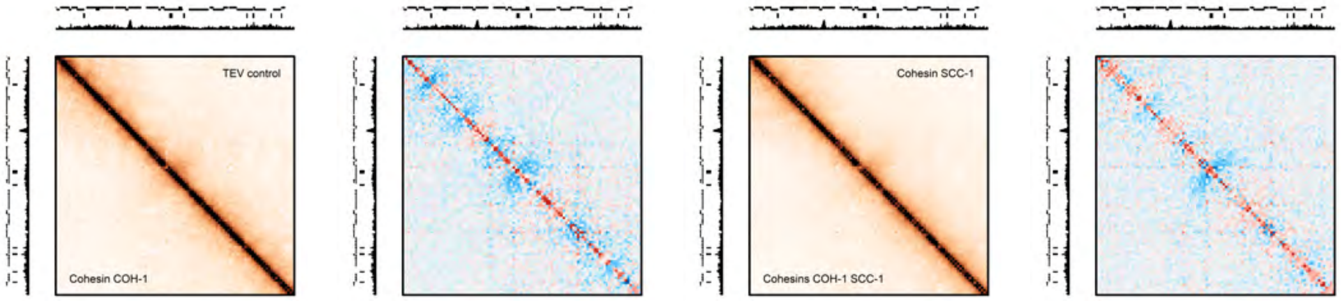

chrIV:6235001-6435000

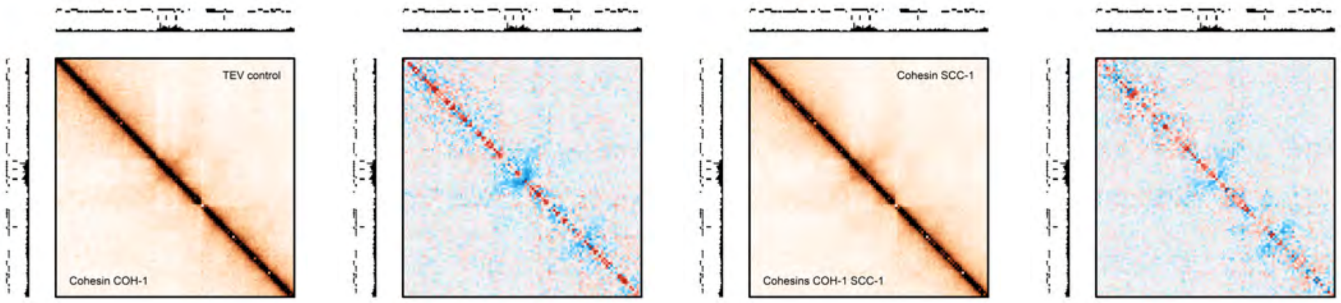

chrX:3845001-4045000

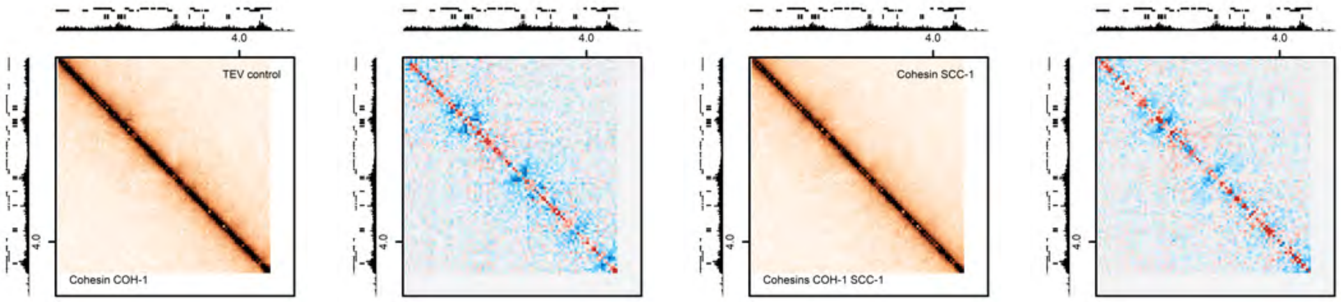

chrV:14099001-14299000

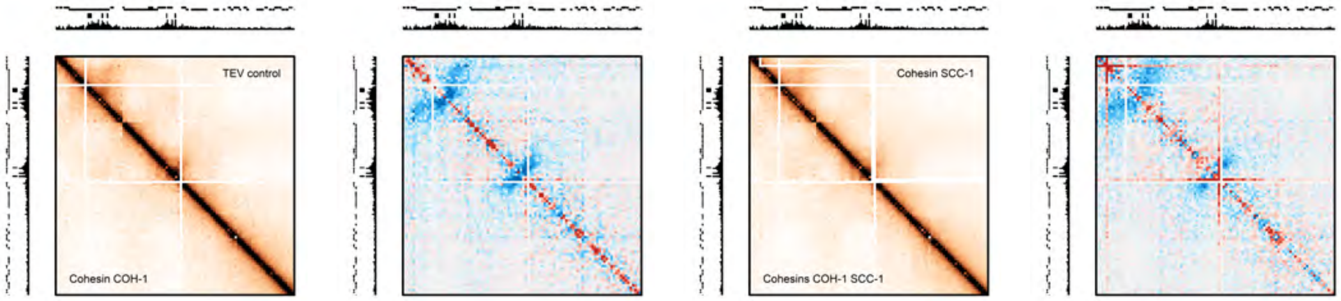

chrX:10331001-10531000

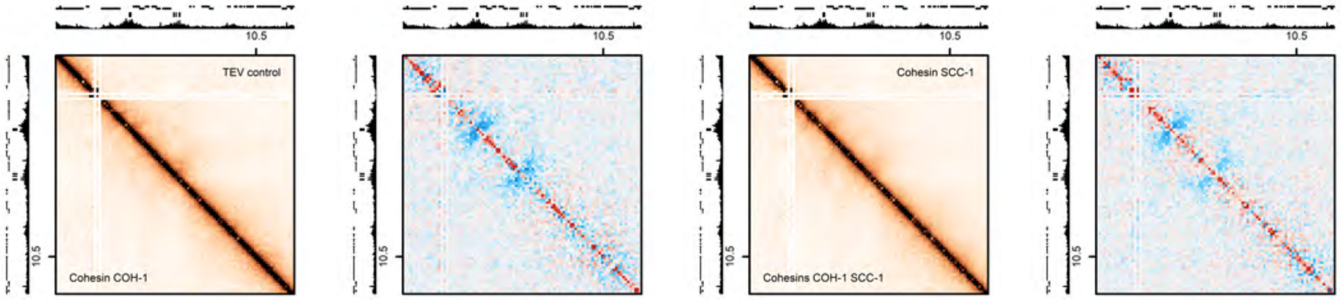

chrIII:10053001-10253000

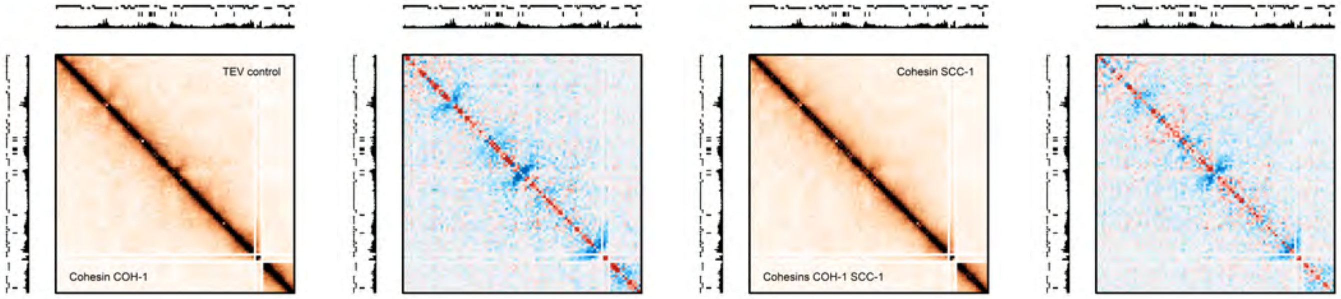

chrI:2029001-2229000

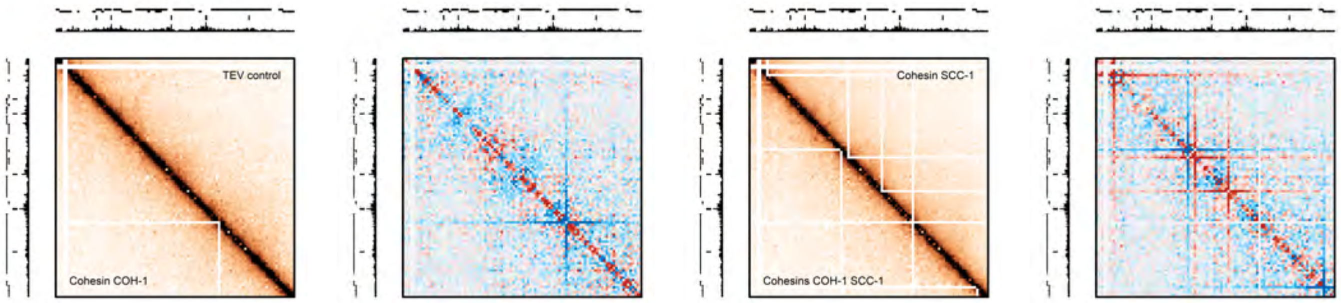

chrIV:5557001-5757000

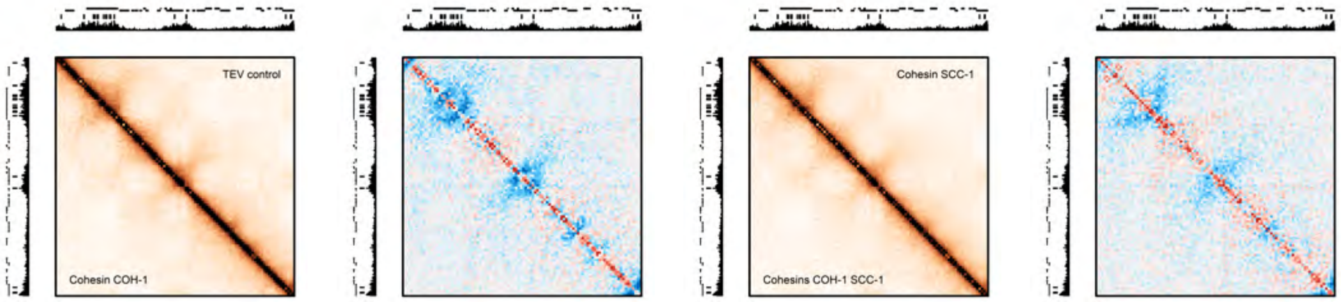

chrV:8113001-8313000

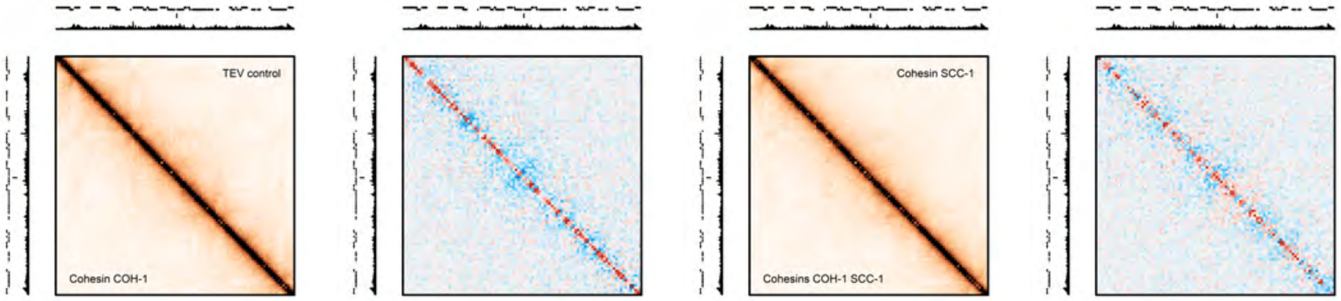

chrIV:10077001-10277000

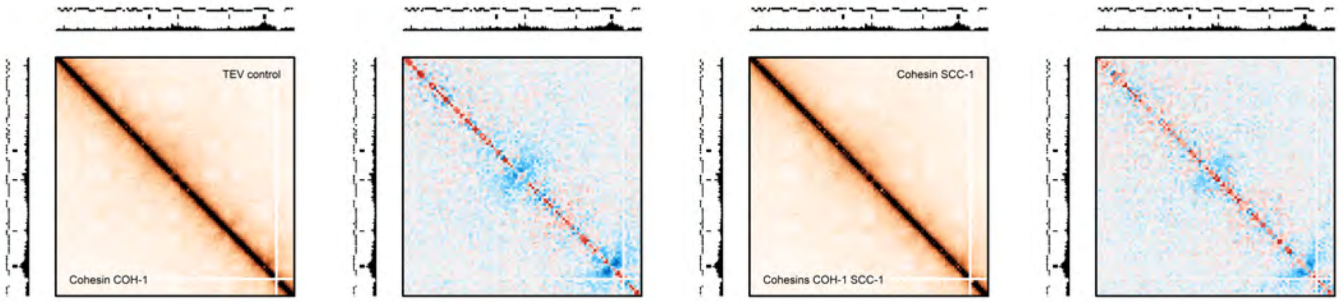

chrV:2141001-2341000

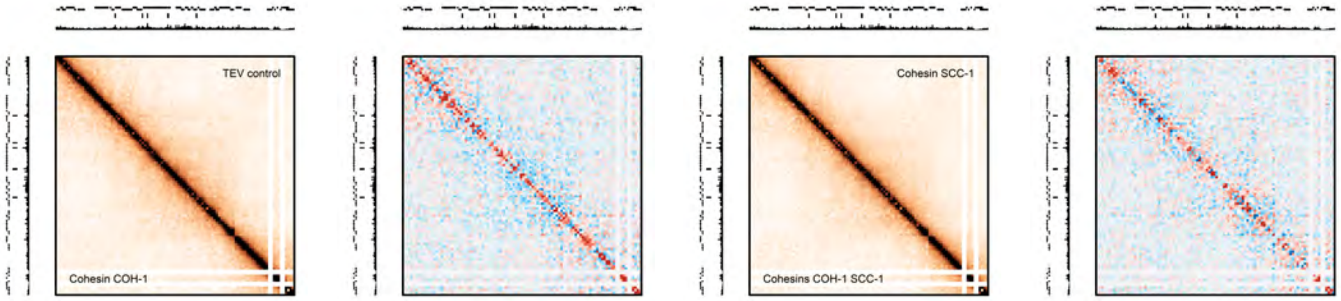

chrV:5121001-5321000

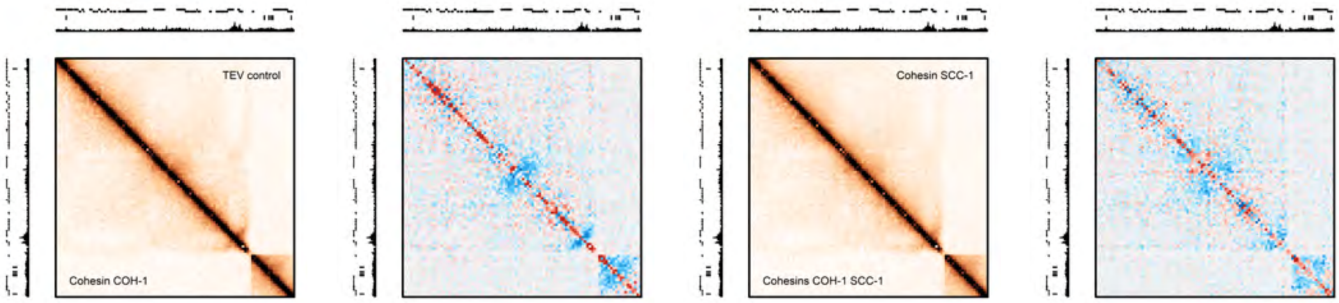

chrII:14841001-15041000

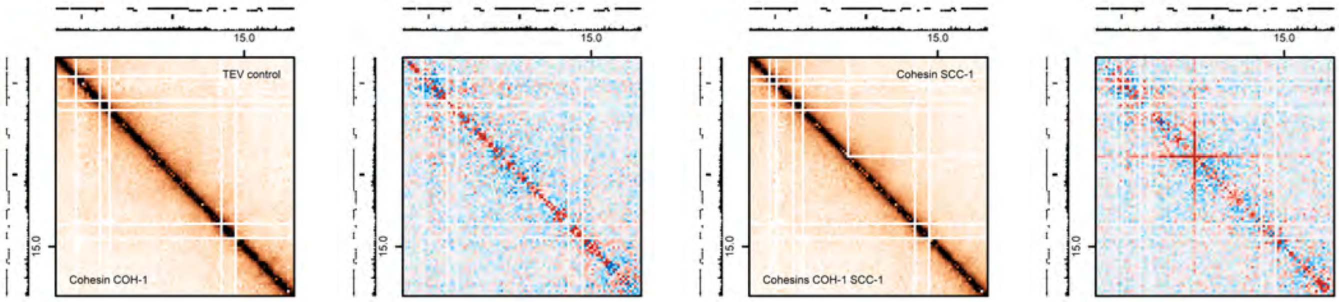

chrI:3303001-3503000

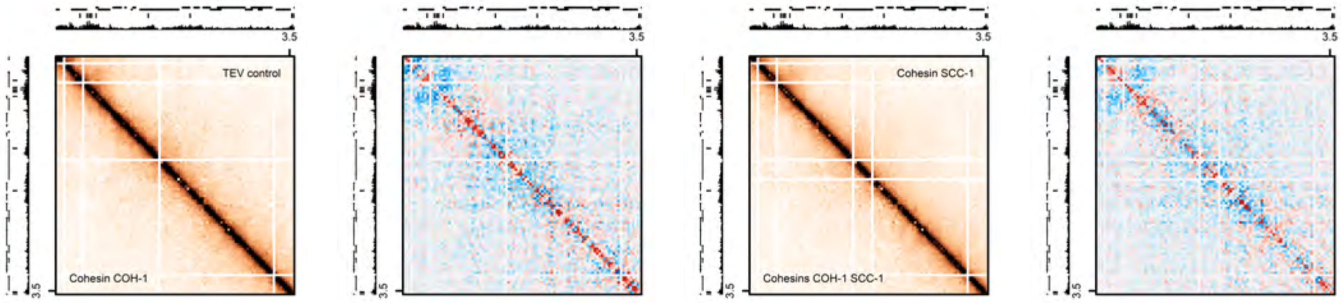

chrII:10067001-10267000

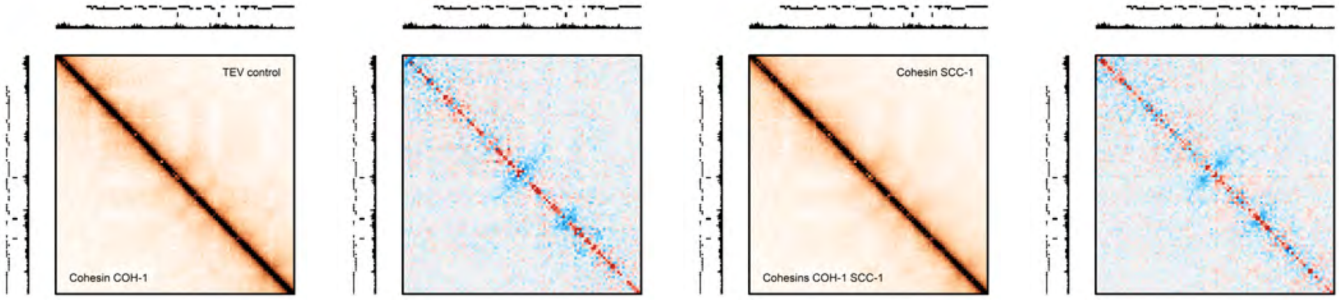

chrIV:11391001-11591000

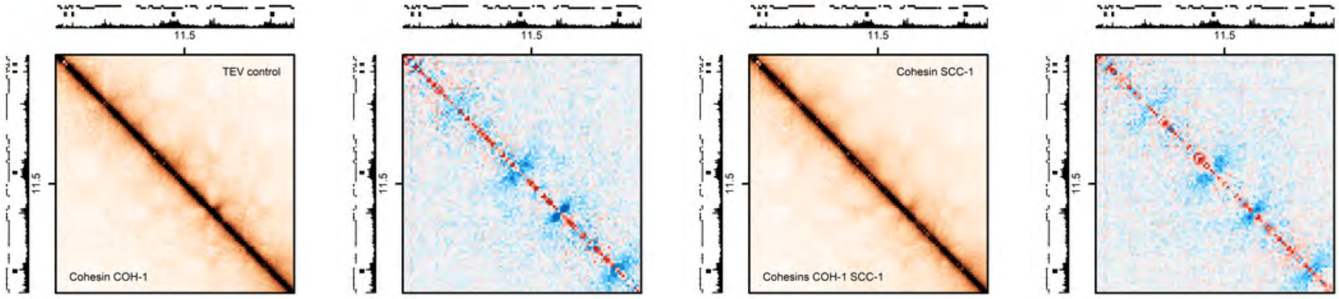

chrIV:5999001-6199000

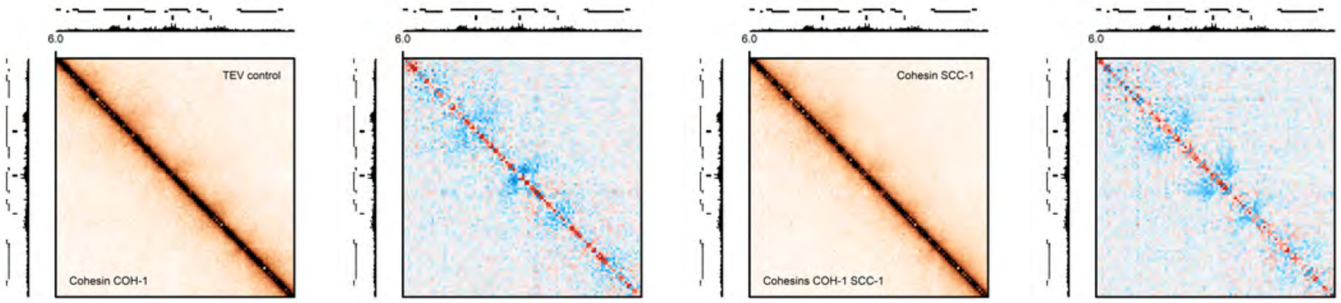

chrIII:4095001-4295000

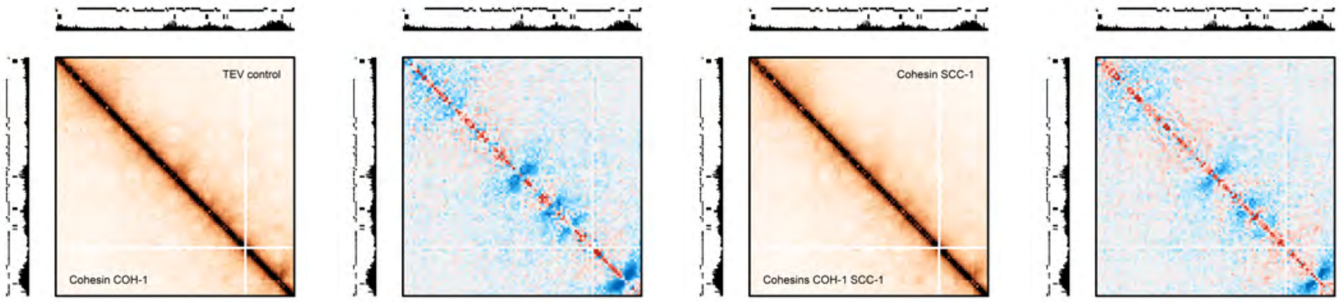

chrIII:9379001-9579000

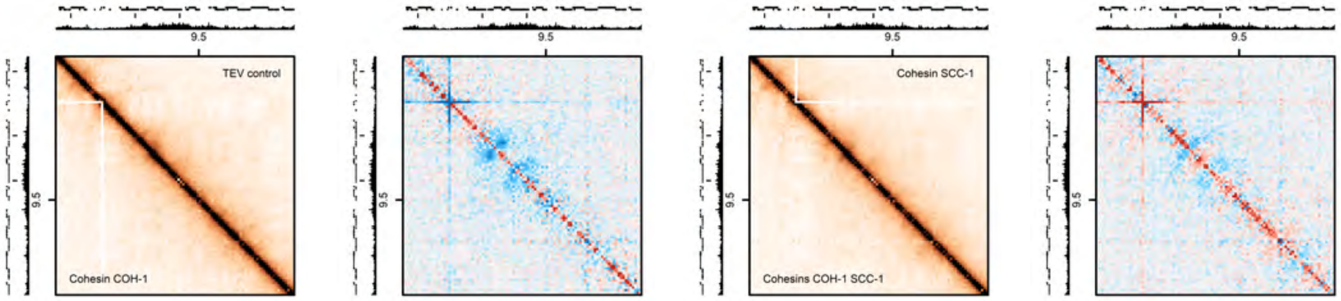

chrI:8479001-8679000

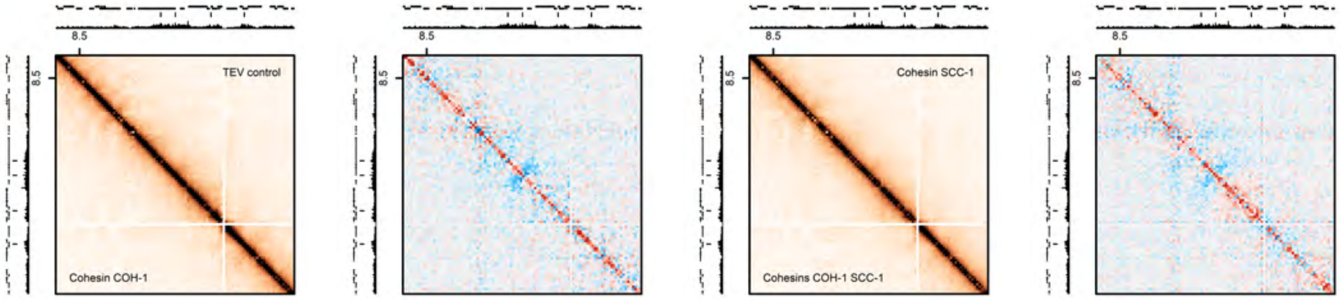

chrII:12777001-12977000

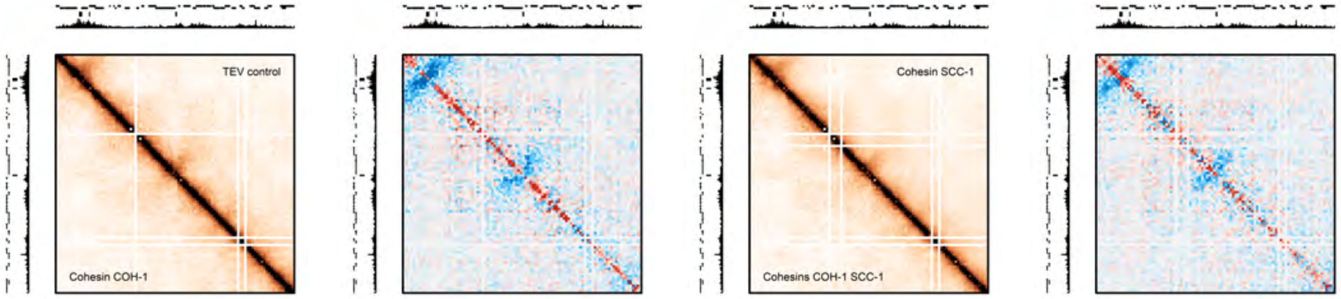

chrX:6563001-6763000

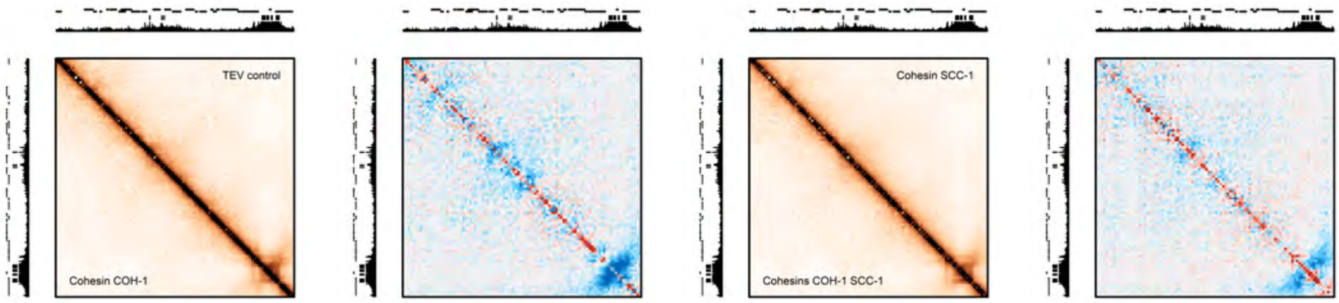

chrX:2871001-3071000

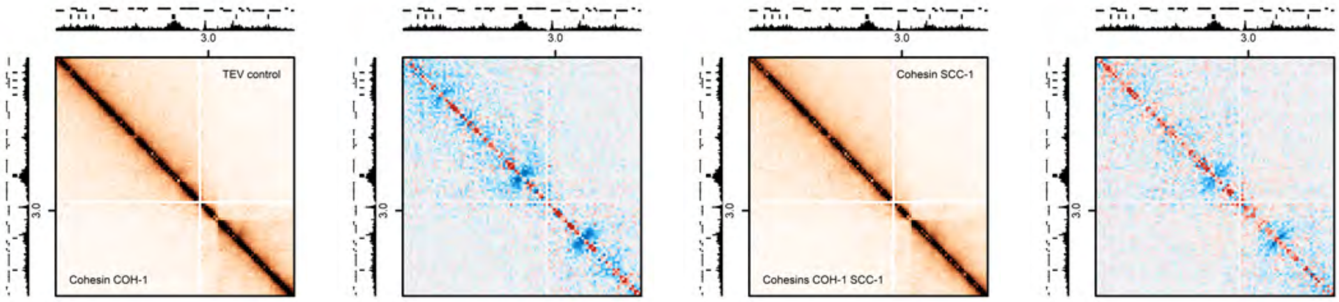

chrII:625001-825000

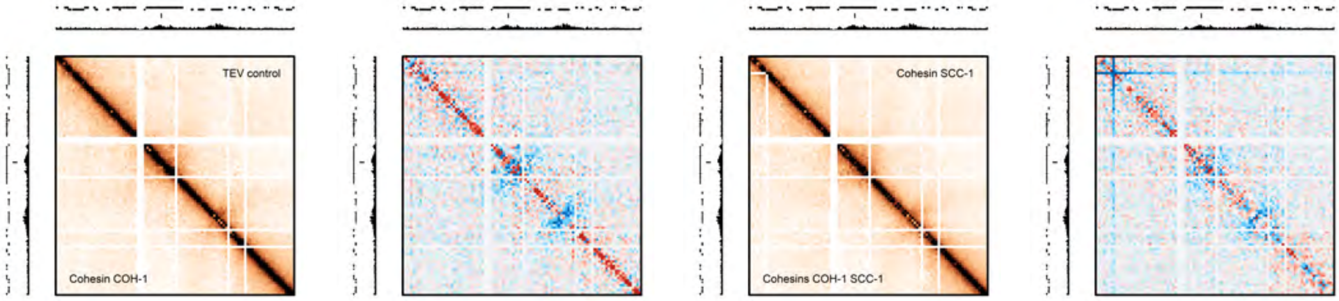

chrV:12367001-12567000

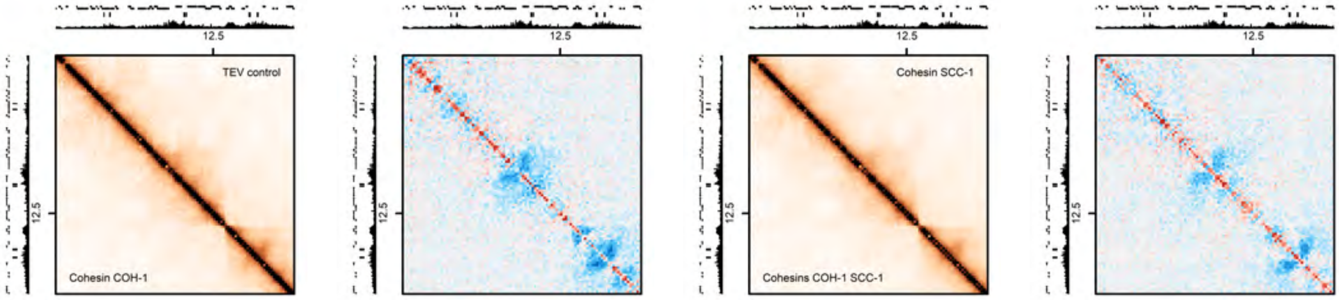

chrV:17561001-17761000

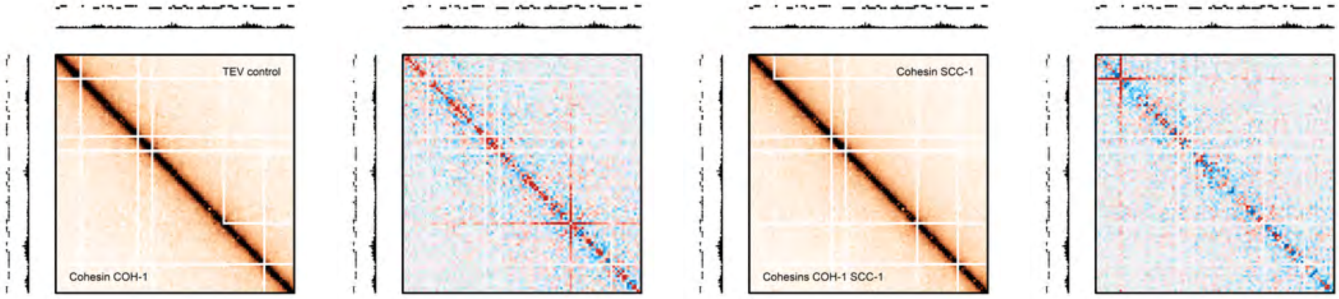

chrV:18131001-18331000

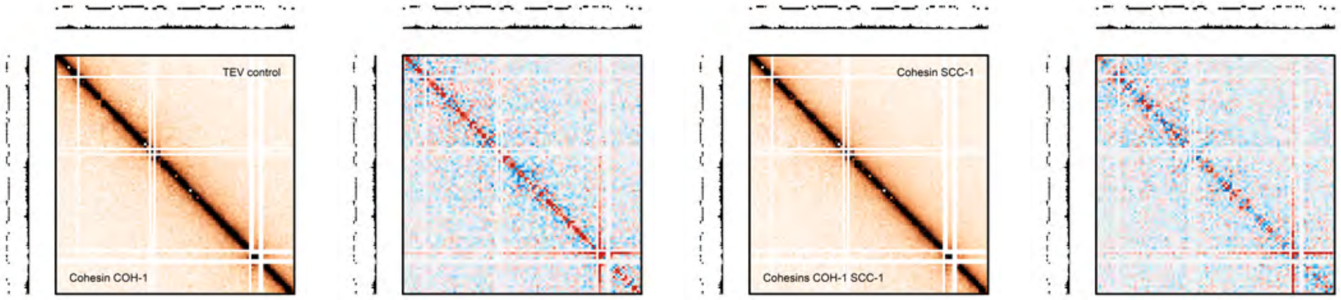

chrX:14461001-14661000

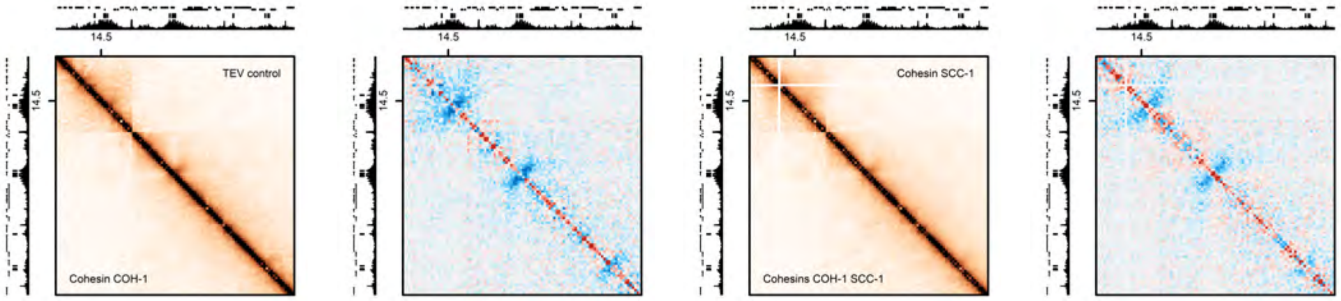

chrIV:3995001-4195000

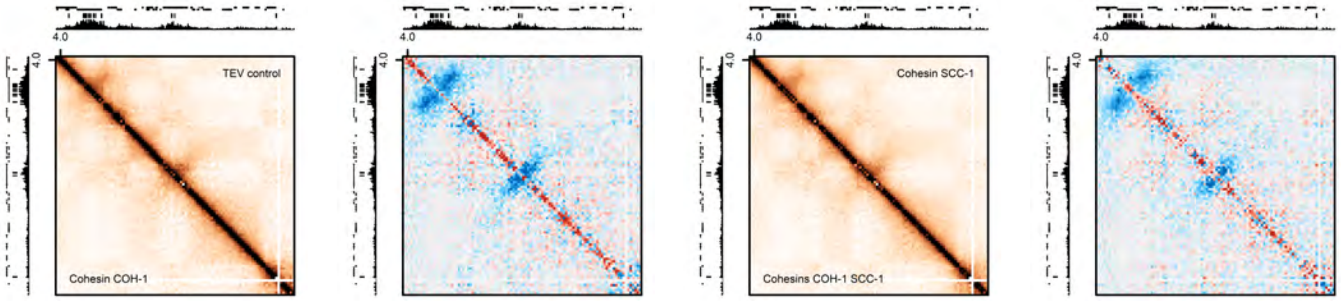

chrII:6045001-6245000

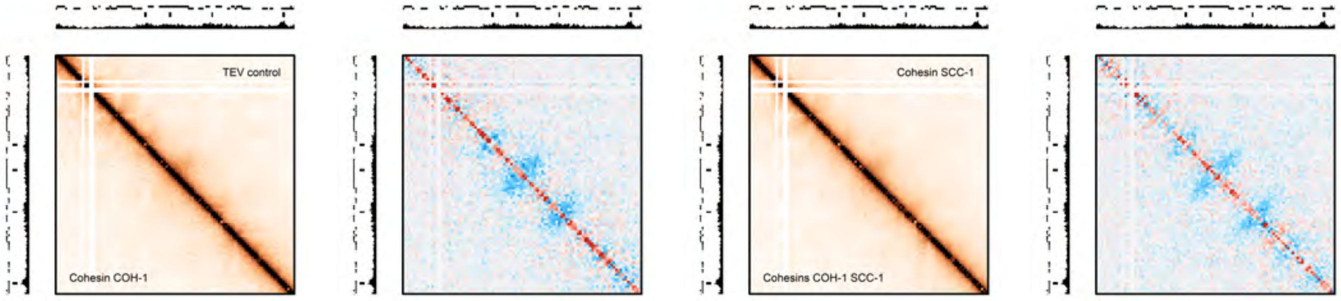

chrIV:9621001-9821000

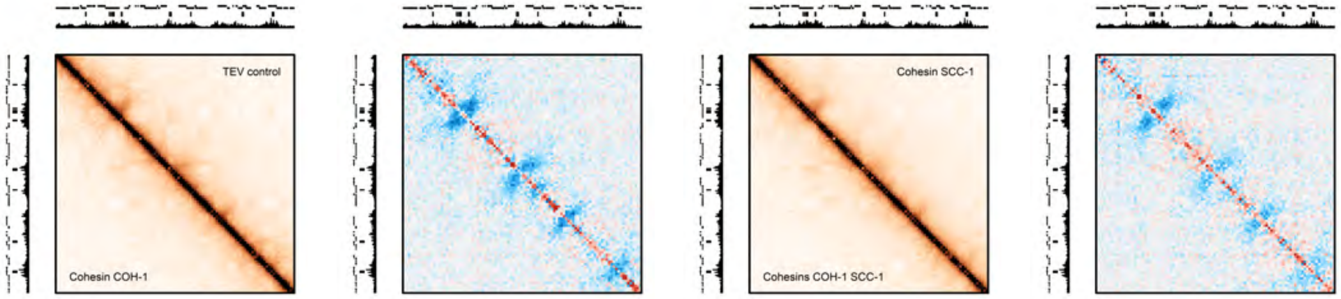

chrI:11683001-11883000

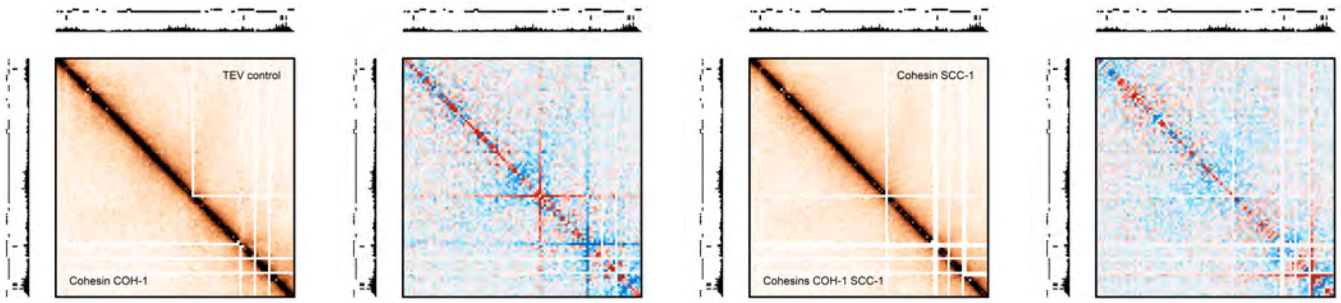

chrI:10185001-10385000

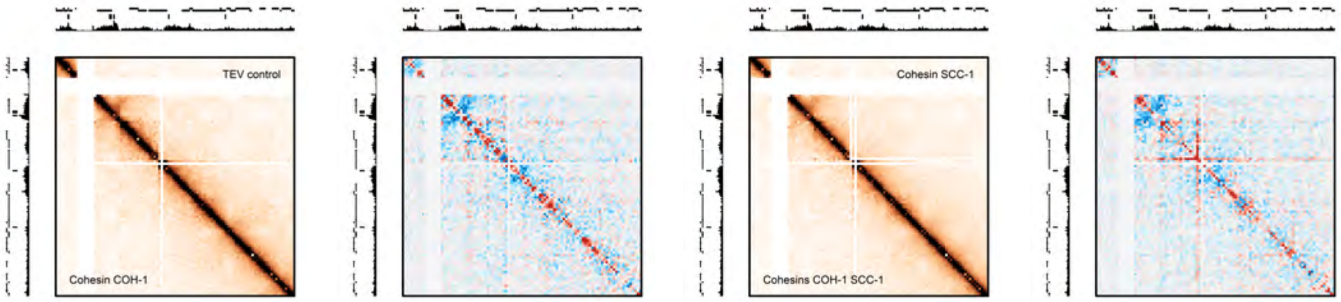

chrIII:1579001-1779000

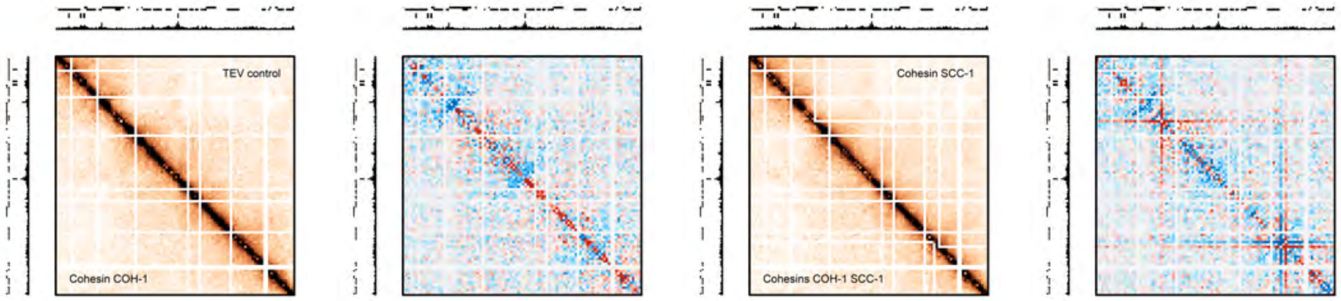

chrIV:11019001-11219000

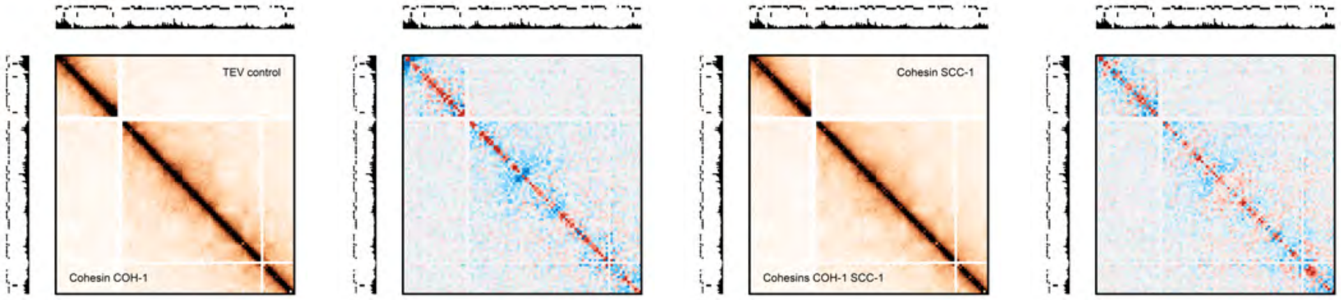

chrII:3427001-3627000

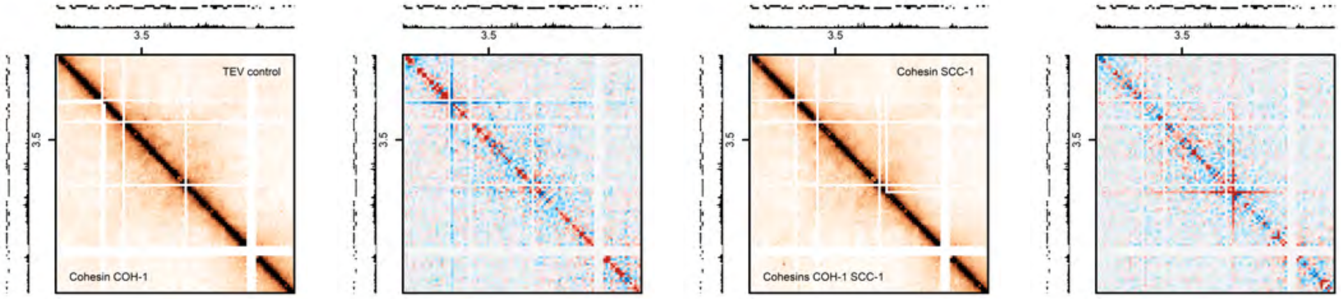

chrX:3367001-3567000

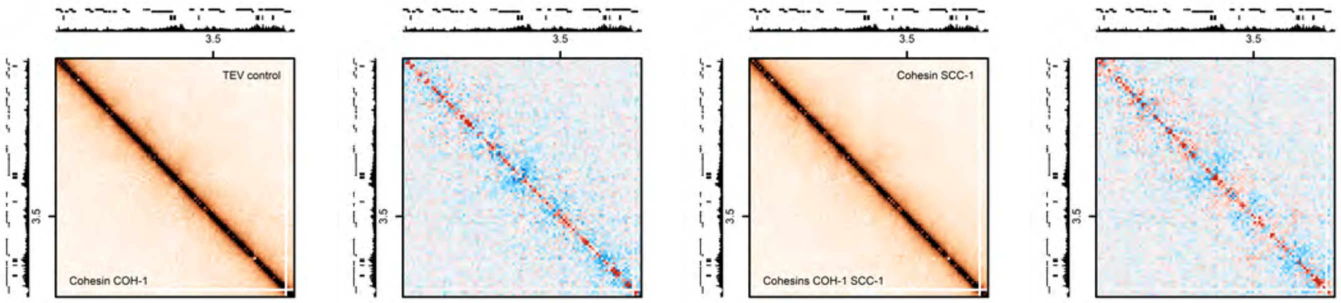

chrIII:5321001-5521000

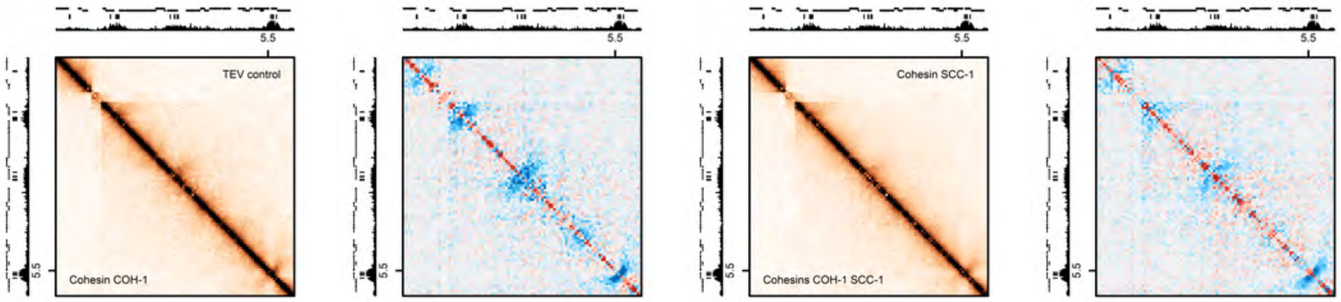

chrV:19105001-19305000

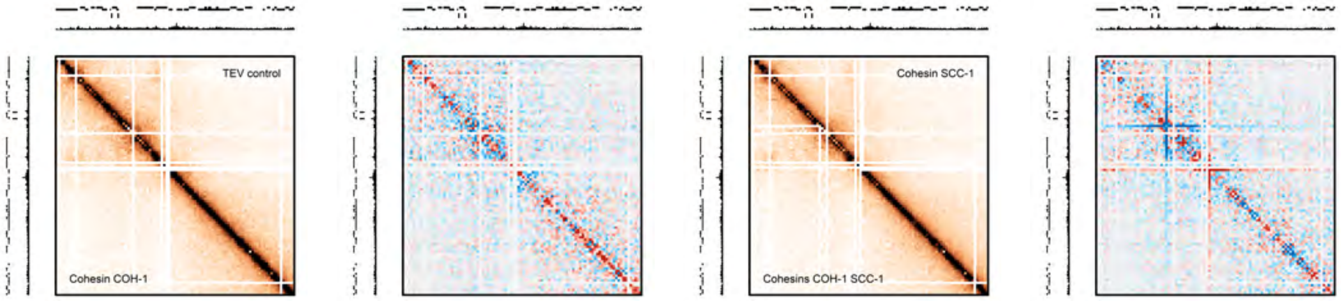

chrV:7335001-7535000

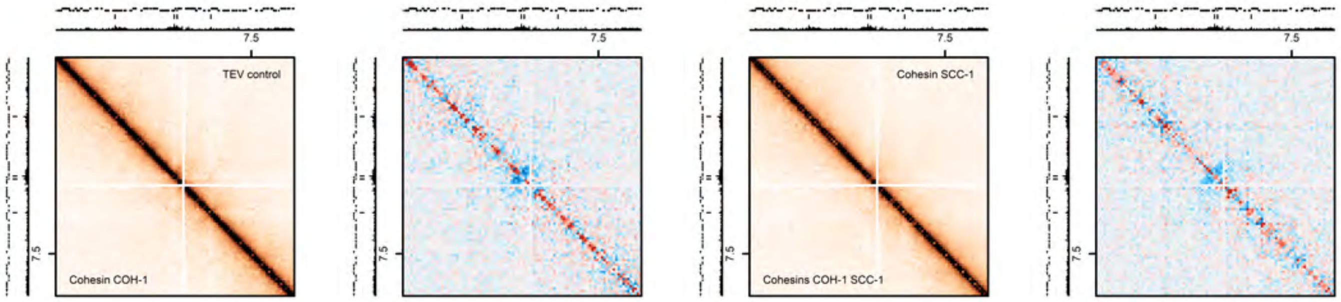

chrI:3147001-3347000

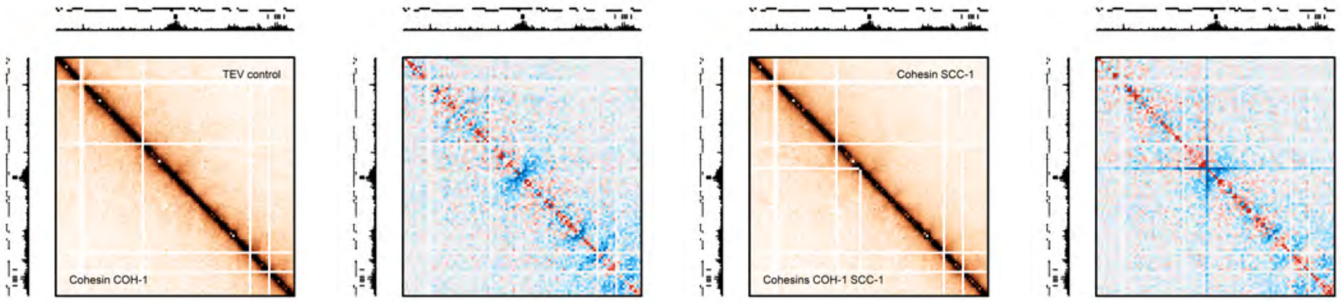

chrV:13181001-13381000

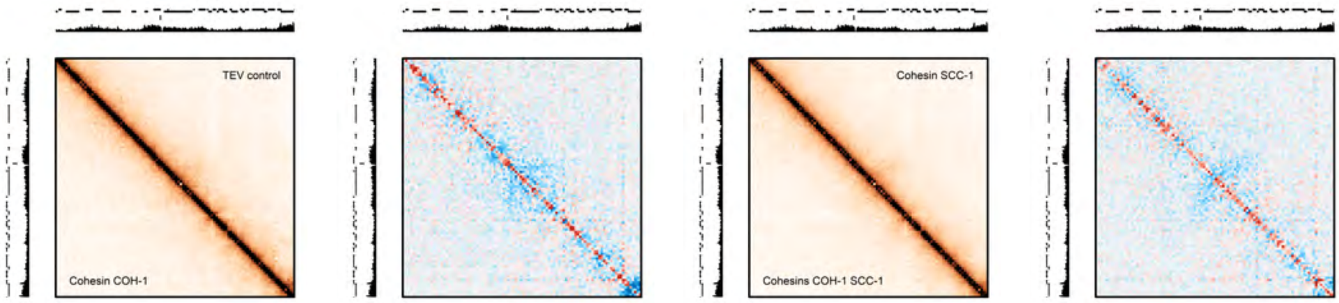

chrI:12075001-12275000

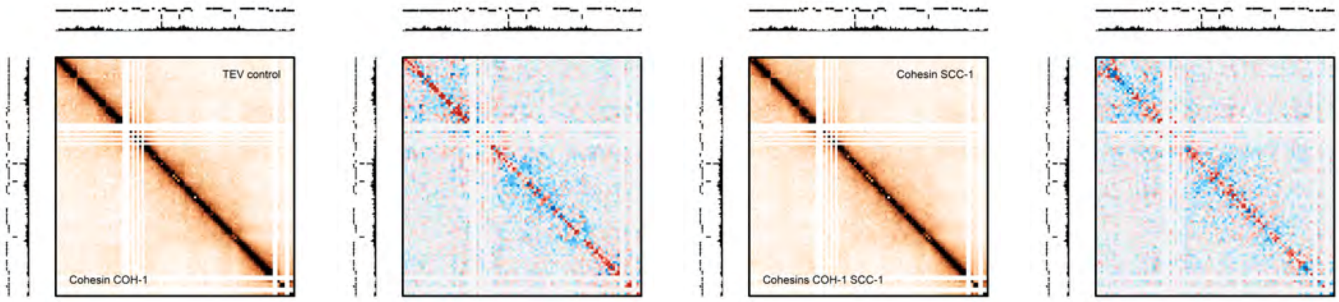

chrII:5365001-5565000

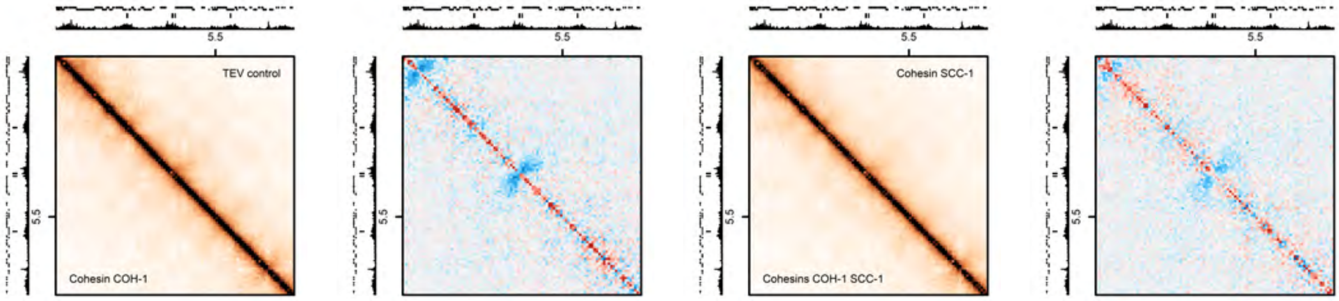

chrI:2317001-2517000

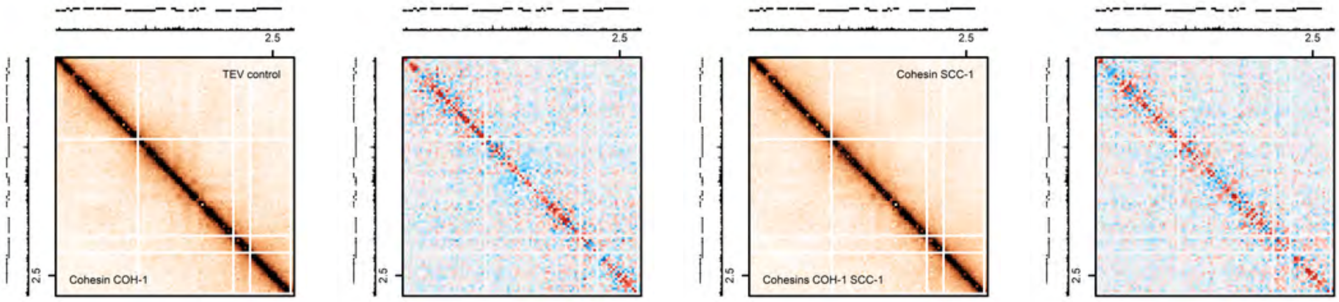

chrX:11505001-11705000

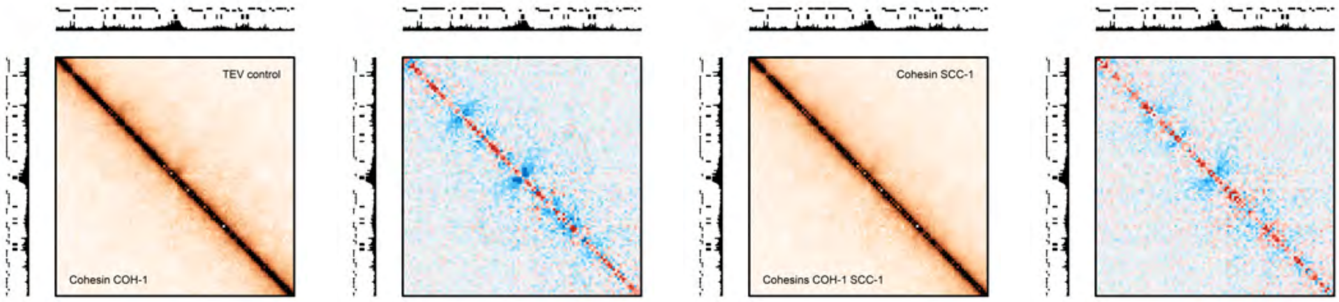

chrIV:9023001-9223000

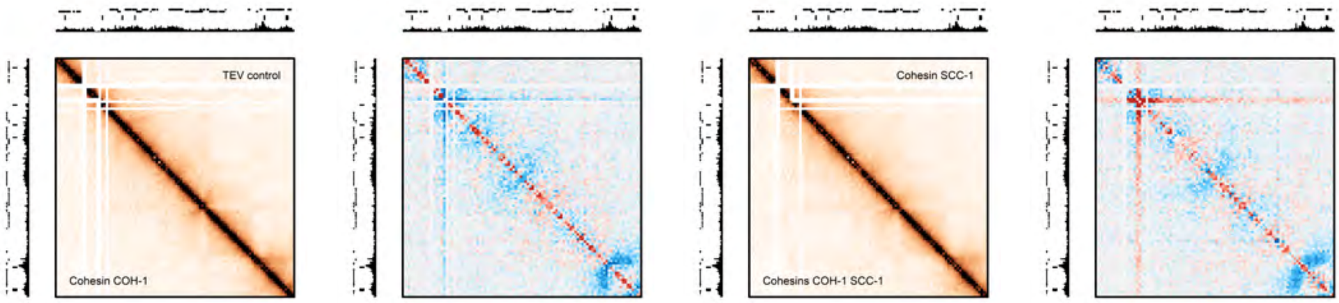

chrIII:13361001-13561000

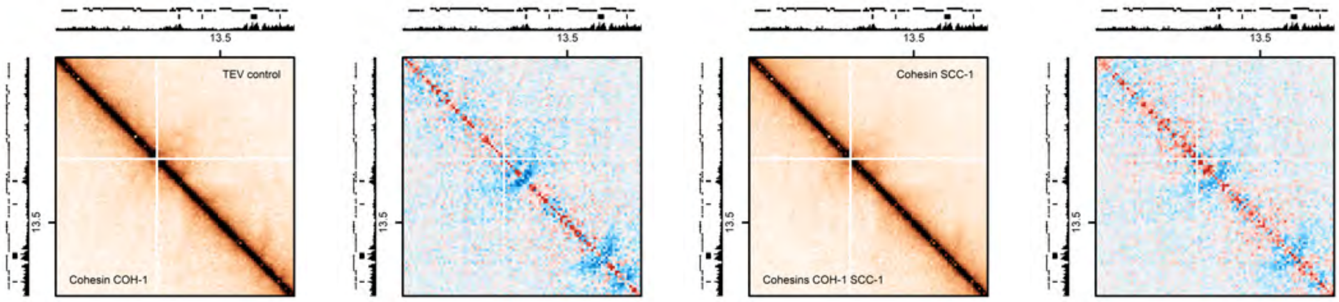

chrI:13735001-13935000

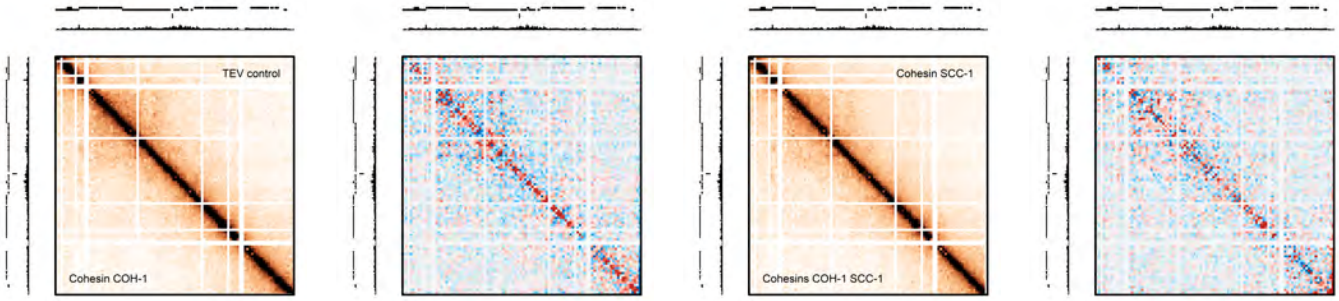

chrIV:1187001-1387000

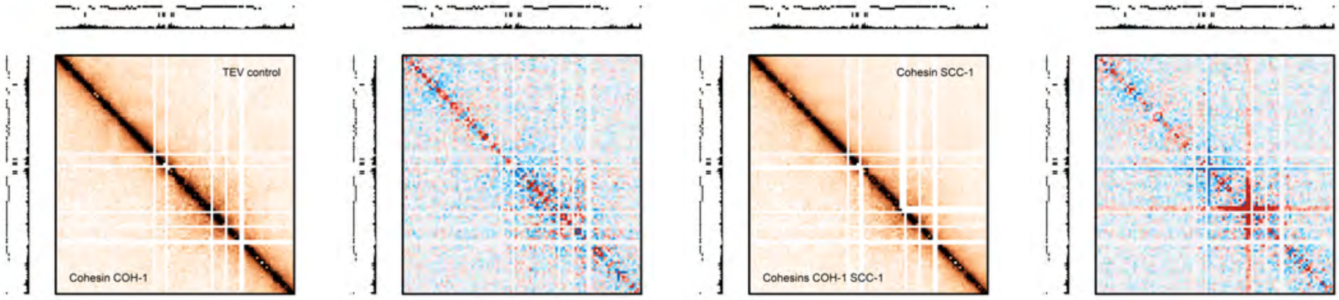

chrX:8703001-8903000

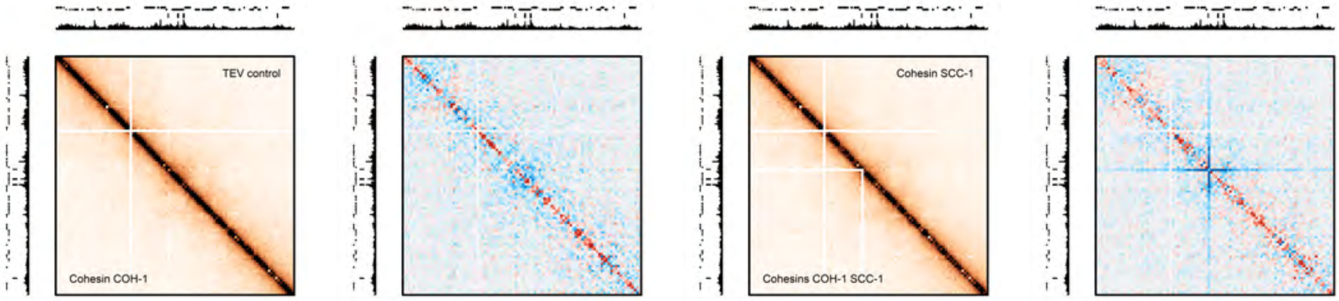

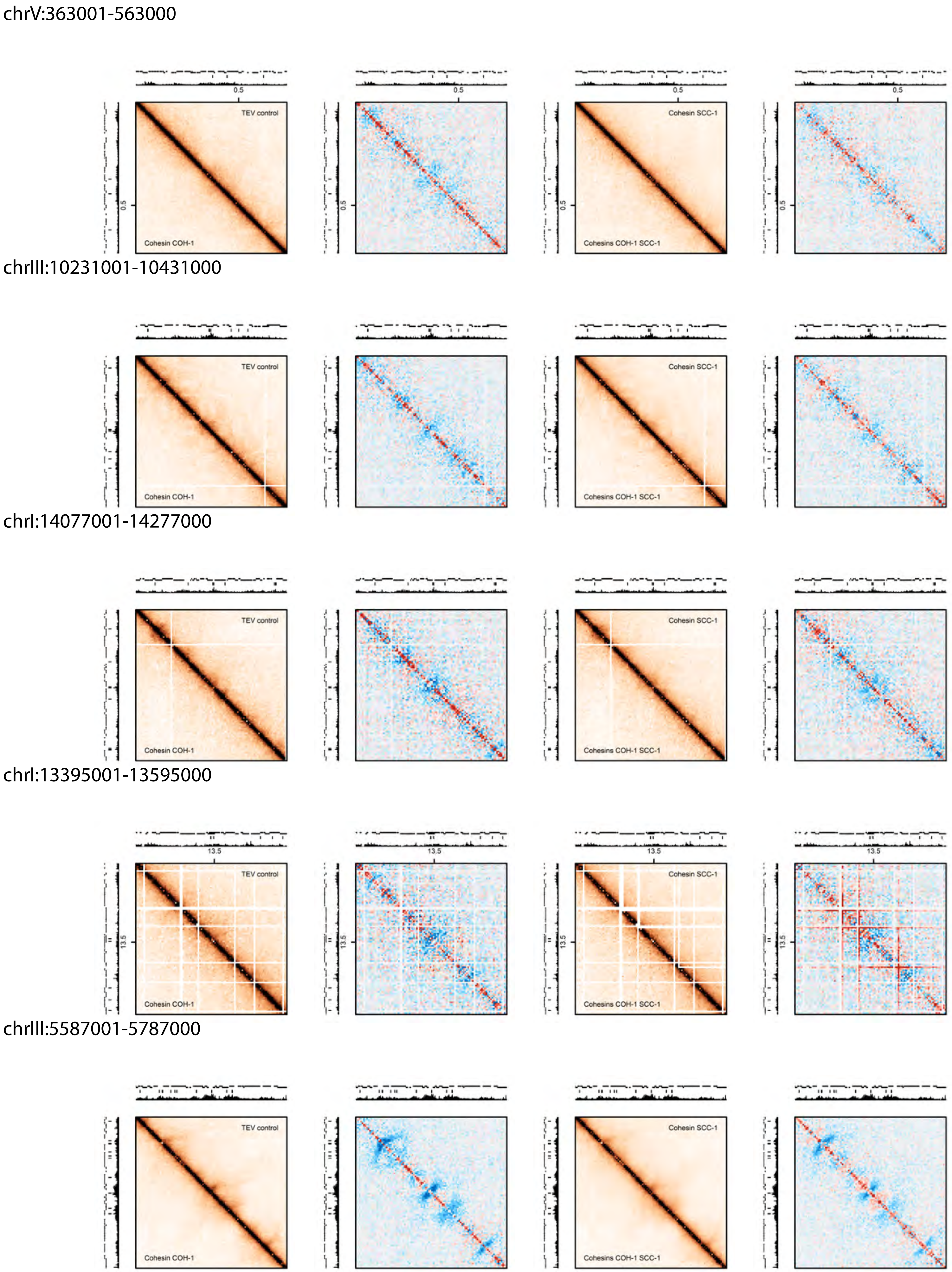
chrV:363001-563000
0.5
TEV control
Cohesin COH-1
Cohesin SCC-1
Cohesins COH-1 SCC-1
chrIII:10231001-10431000
TEV control
Cohesin COH-1
Cohesin SCC-1
Cohesins COH-1 SCC-1
chrI:14077001-14277000
TEV control
Cohesin COH-1
Cohesin SCC-1
Cohesins COH-1 SCC-1
chrI:13395001-13595000
13.5
TEV control
Cohesin COH-1
Cohesin SCC-1
Cohesins COH-1 SCC-1
chrIII:5587001-5787000
TEV control
Cohesin COH-1
Cohesin SCC-1
Cohesins COH-1 SCC-1

chrII:3337001-3537000

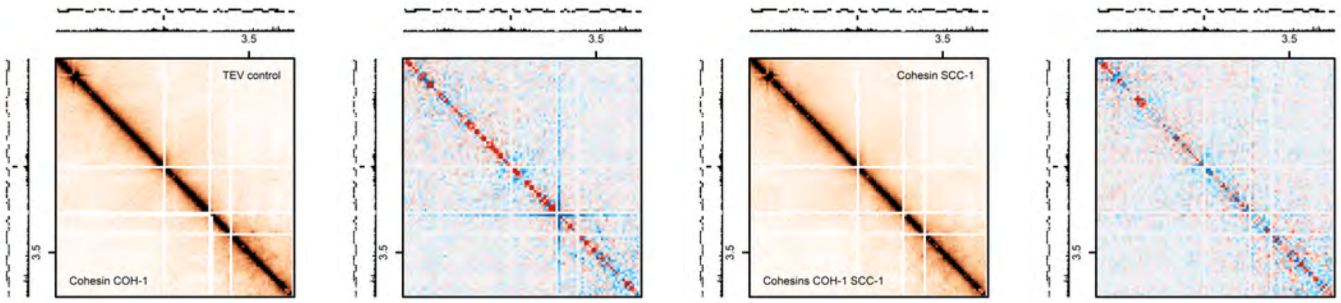

chrV:11537001-11737000

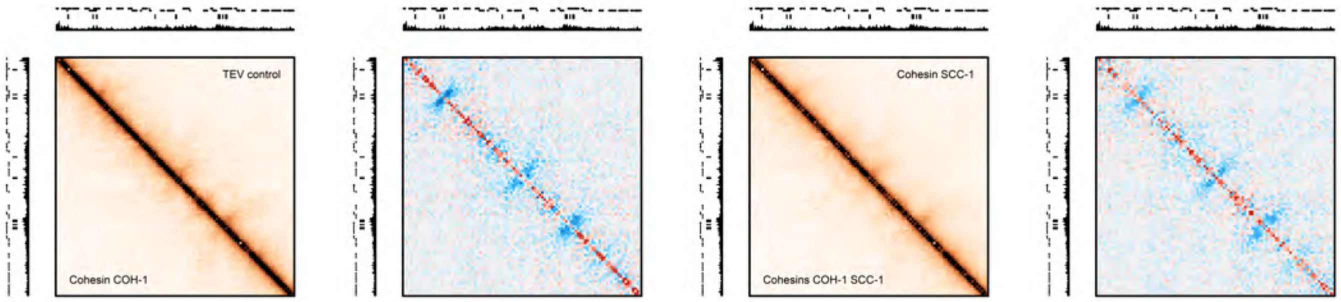

chrV:7919001-8119000

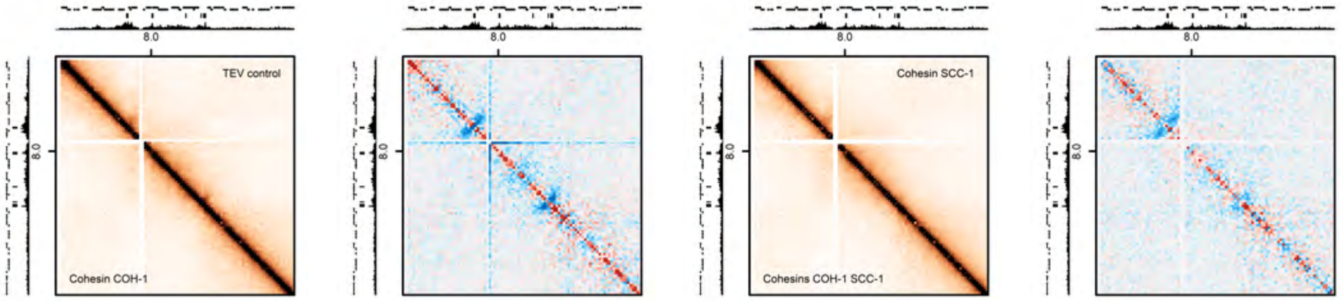

chrIII:2775001-2975000

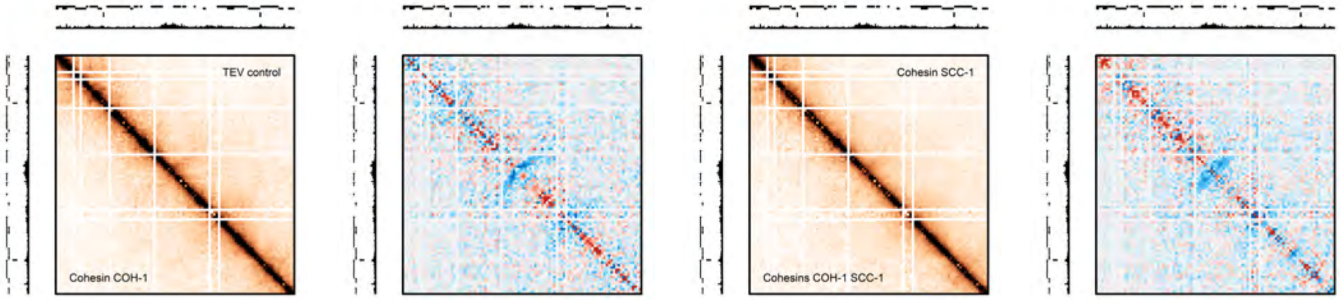

chrII:5121001-5321000

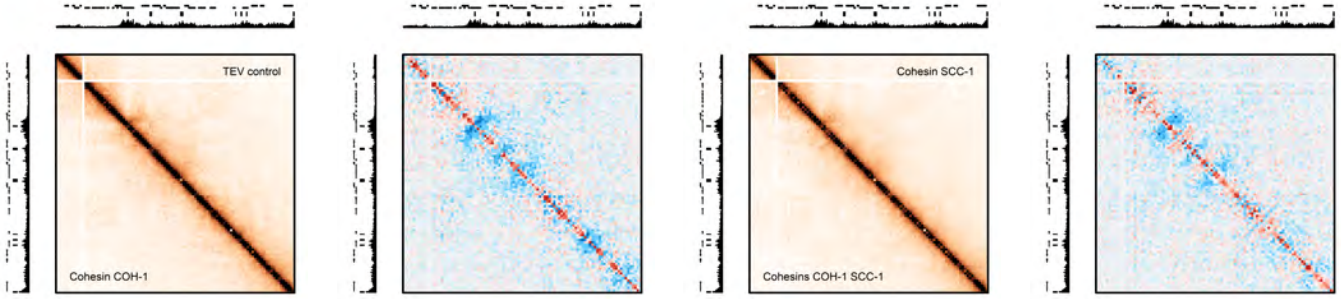

chrII:499001-699000

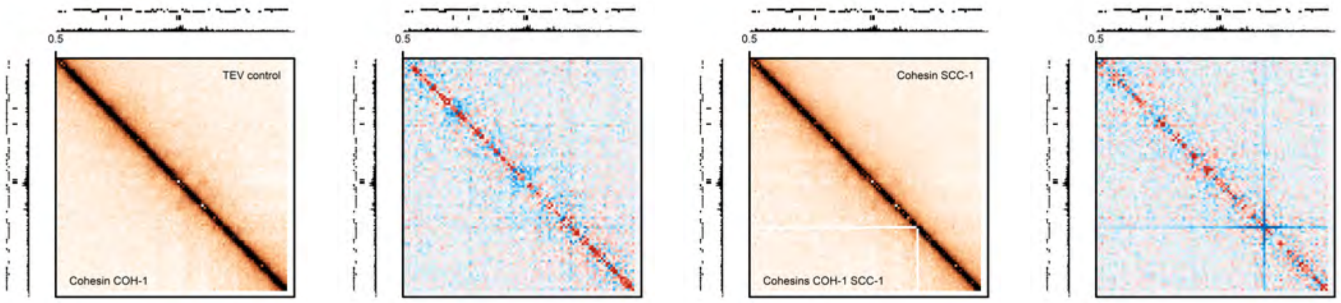

chrI:9329001-9529000

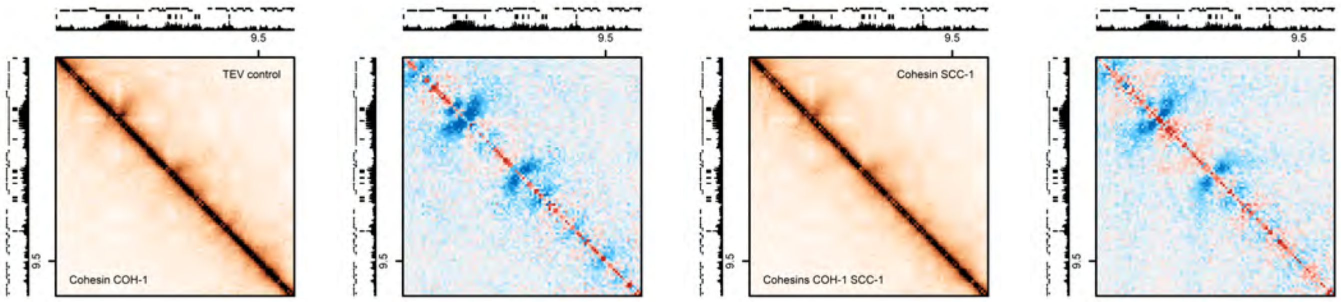

chrV:6897001-7097000

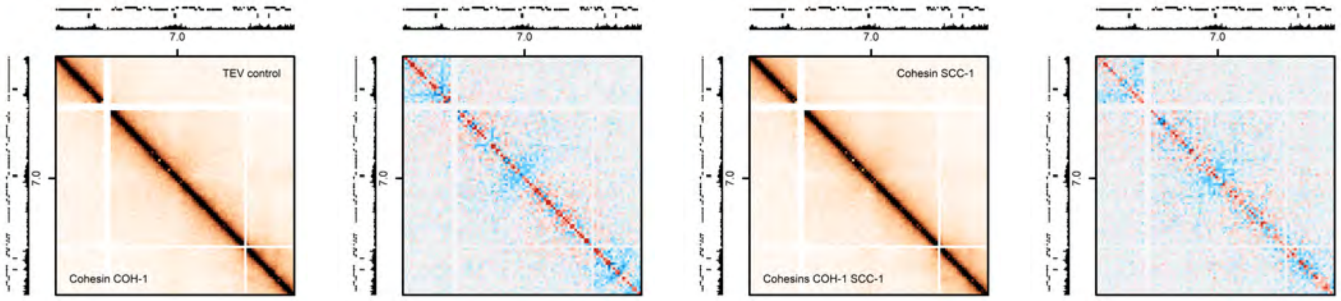

chrV:20779001-20979000

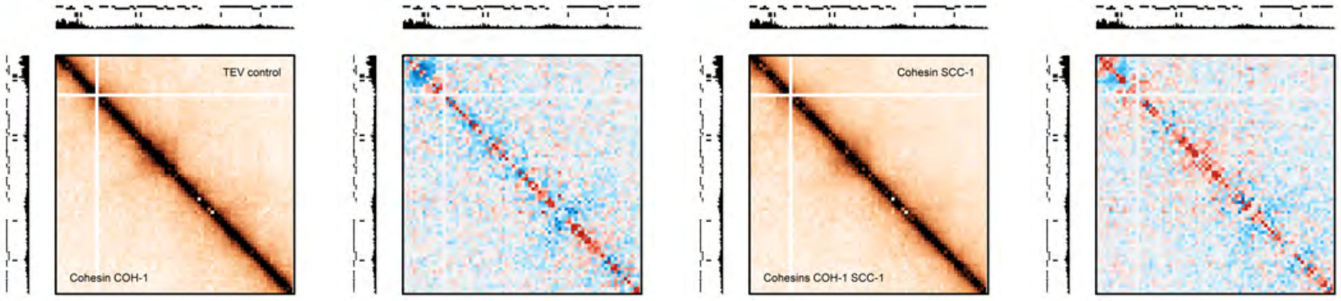

chrIII:1329001-1529000

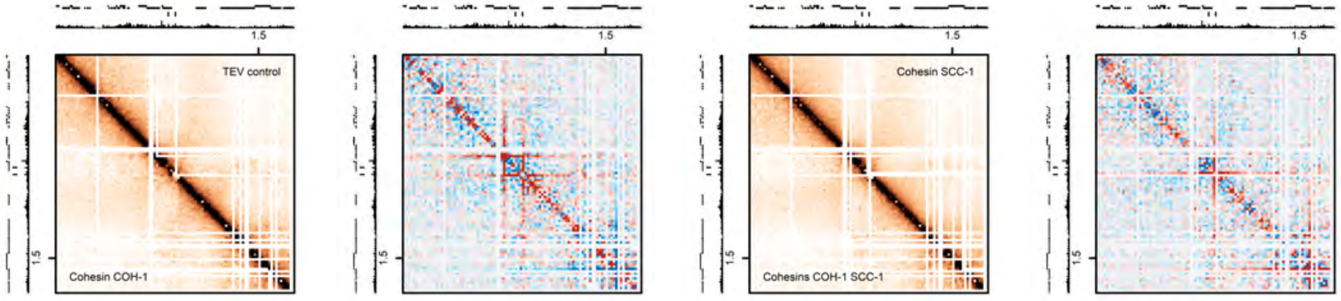

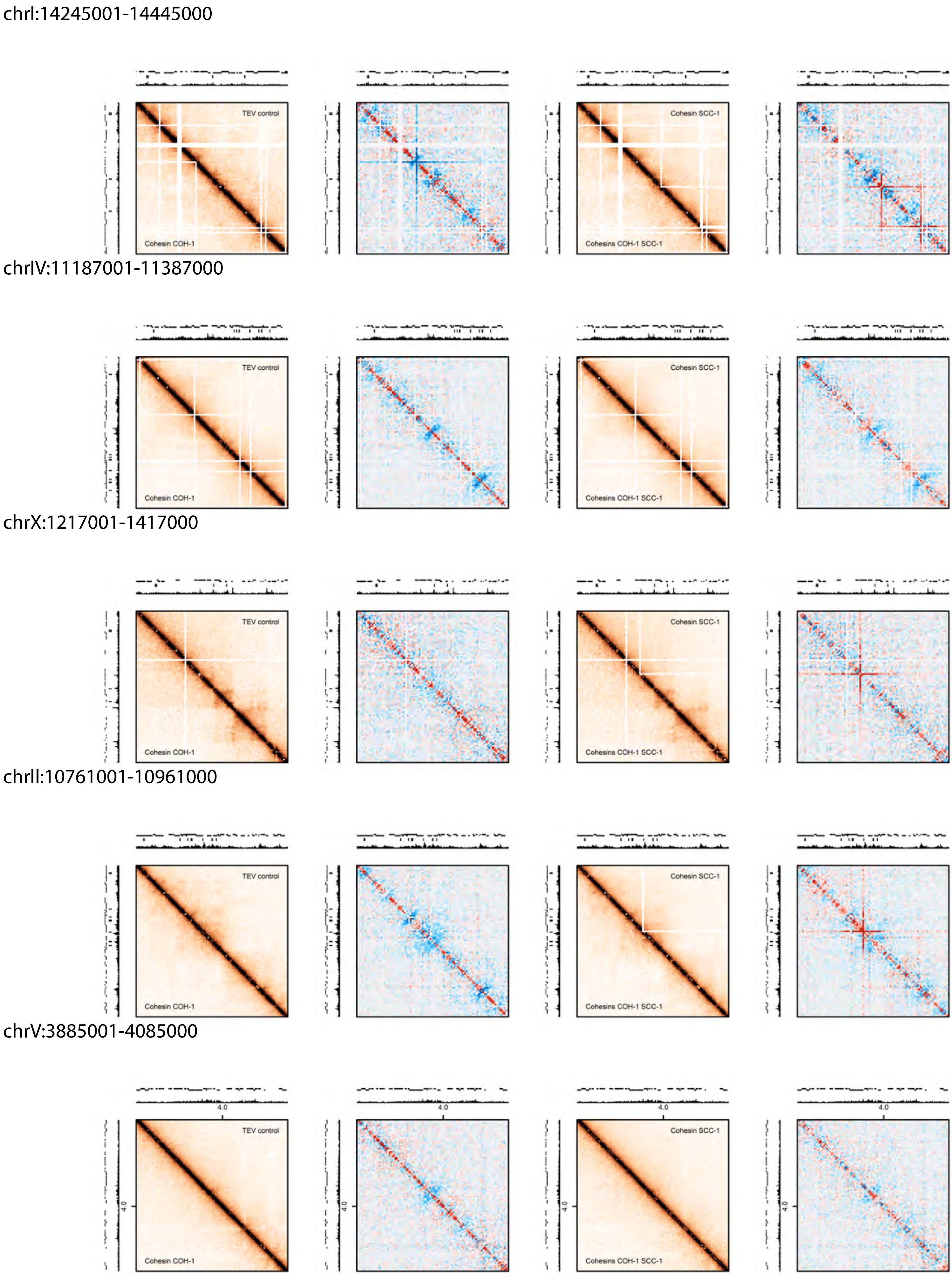
chrI:14245001-14445000
TEV control
Cohesin COH-1
Cohesin SCC-1
Cohesins COH-1 SCC-1
chrIV:11187001-11387000
TEV control
Cohesin COH-1
Cohesin SCC-1
Cohesins COH-1 SCC-1
chrX:1217001-1417000
TEV control
Cohesin COH-1
Cohesin SCC-1
Cohesins COH-1 SCC-1
chrII:10761001-10961000
TEV control
Cohesin COH-1
Cohesin SCC-1
Cohesins COH-1 SCC-1
chrV:3885001-4085000
4.0
TEV control
Cohesin COH-1
Cohesin SCC-1
Cohesins COH-1 SCC-1

chrIII:9139001-9339000

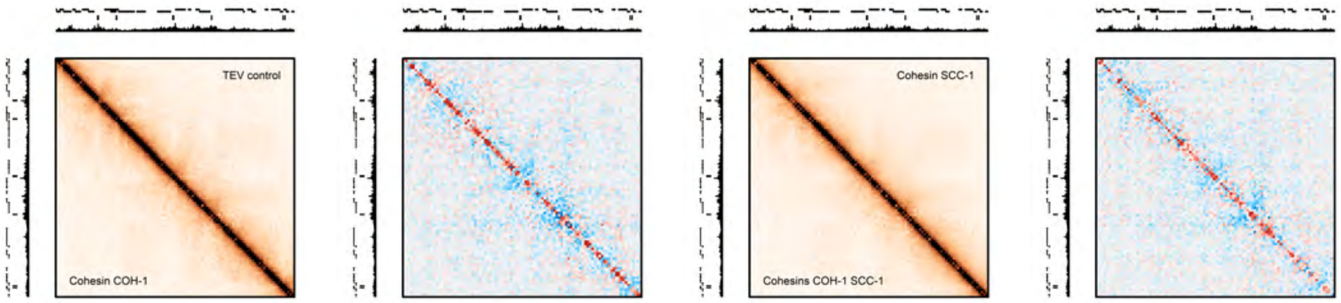

chrV:4723001-4923000

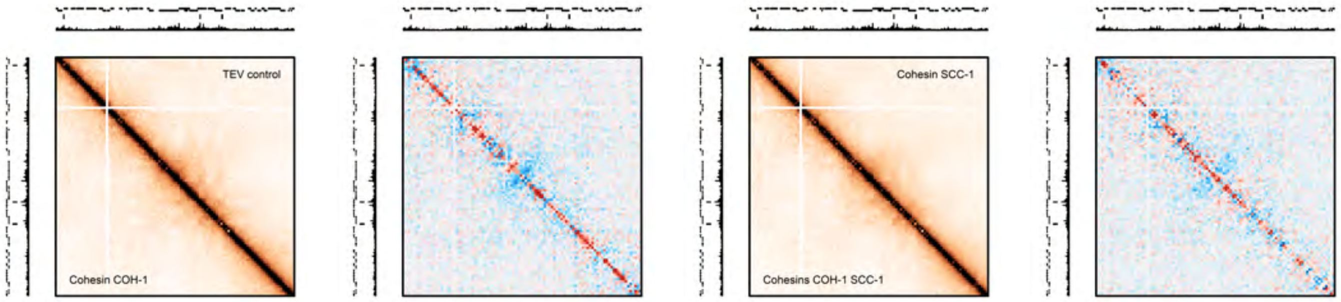

chrII:4399001-4599000

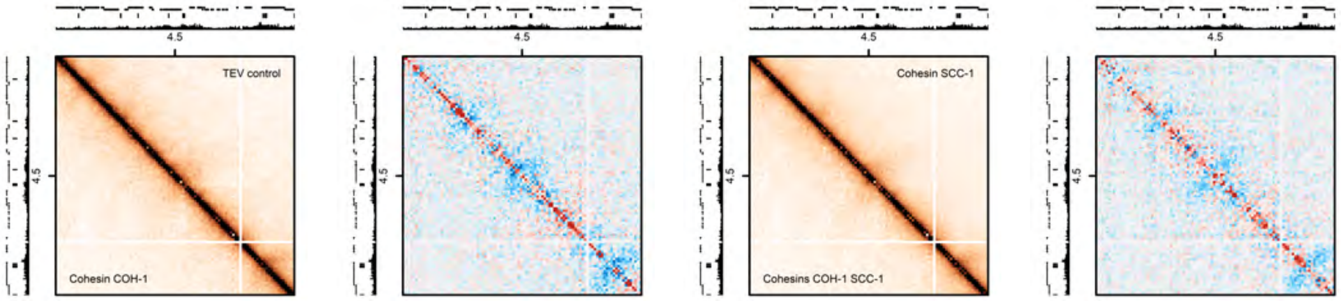

chrI:9691001-9891000

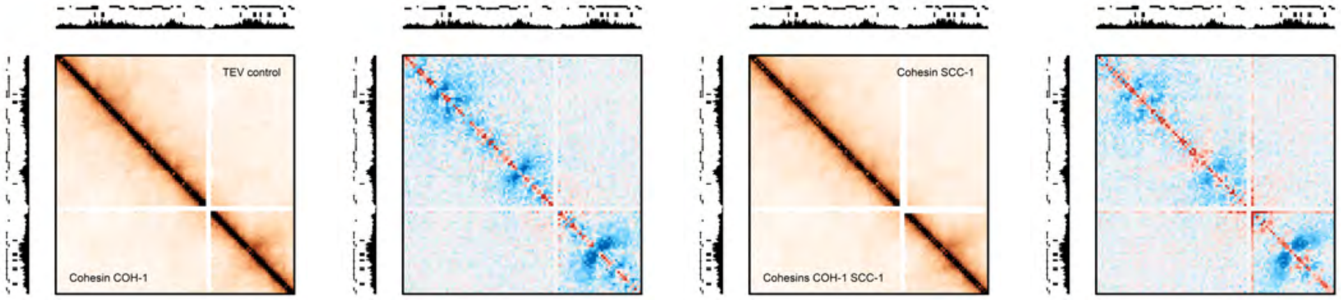

chrIV:10927001-11127000

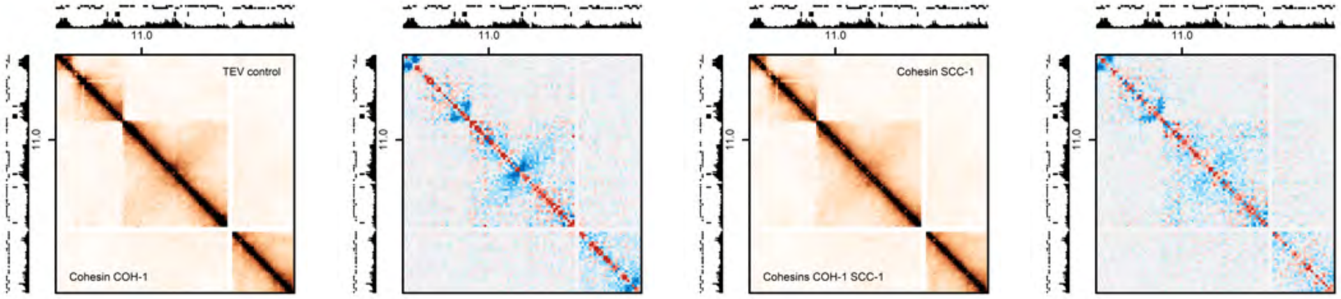

chrII:1403001-1603000

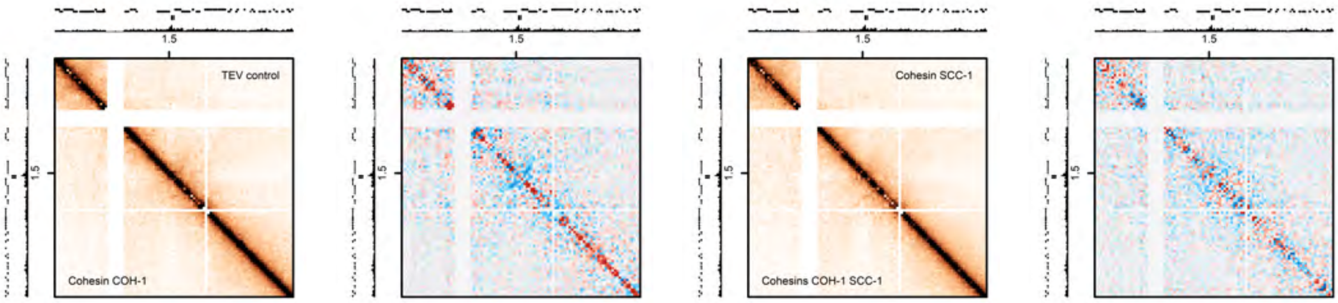

chrX:15243001-15443000

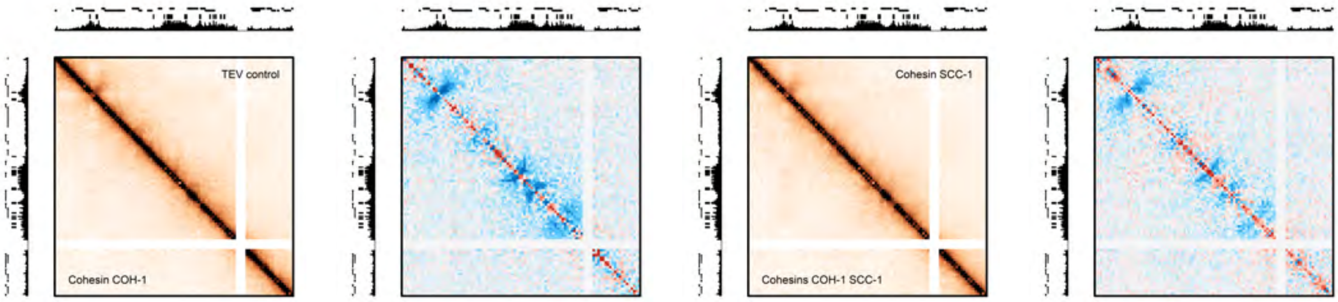

chrIV:7137001-7337000

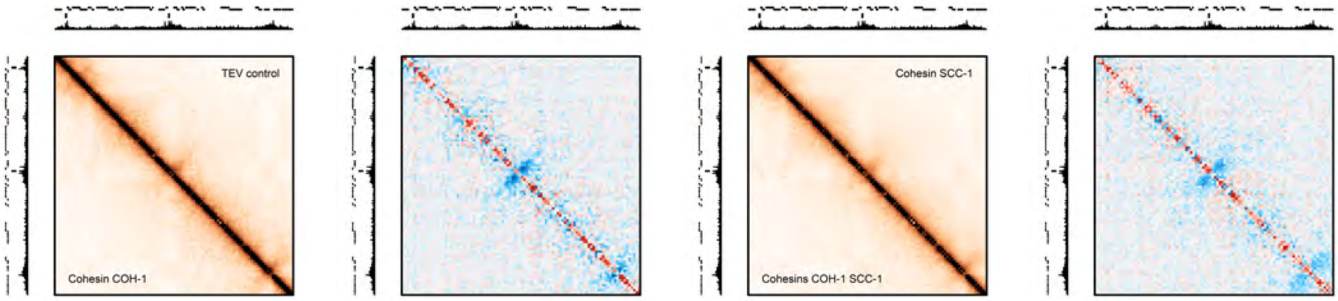

chrV:8509001-8709000

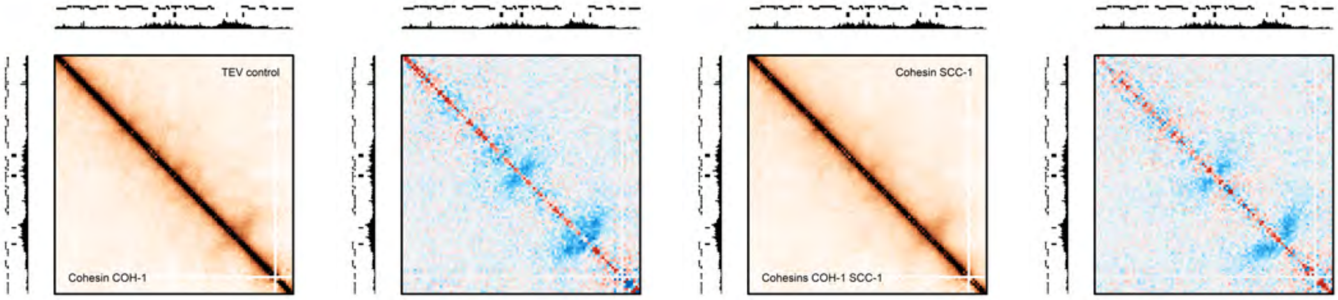

chrI:2861001-3061000

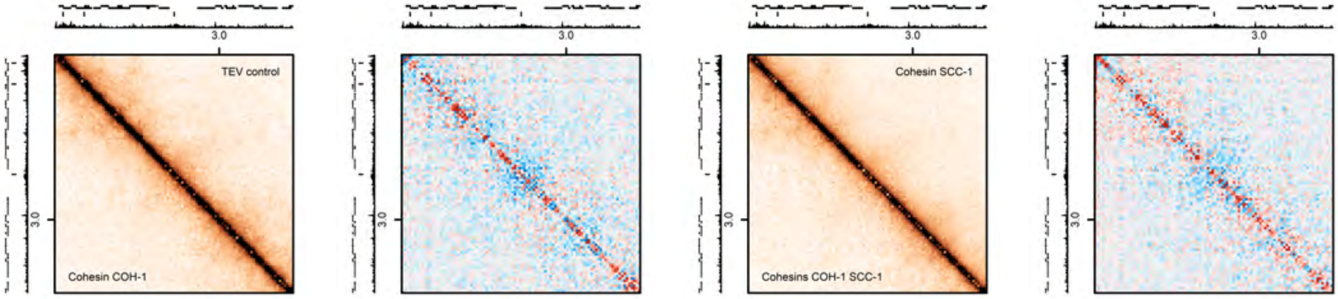

chrIV:2353001-2553000

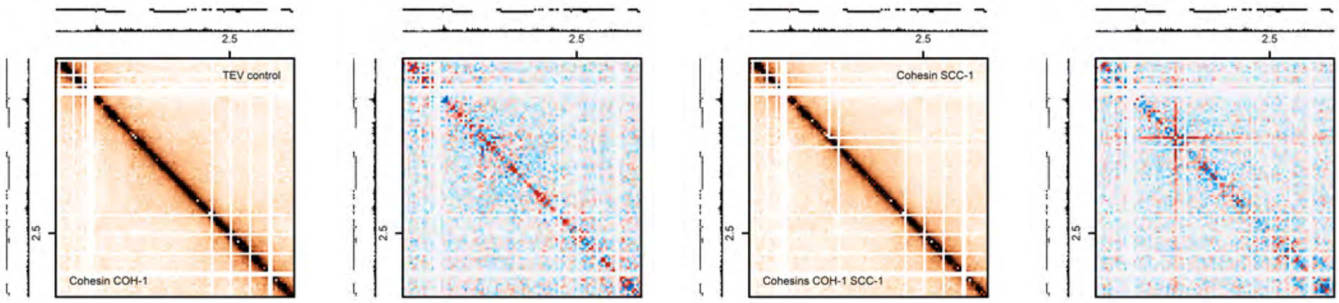

chrV:3481001-3681000

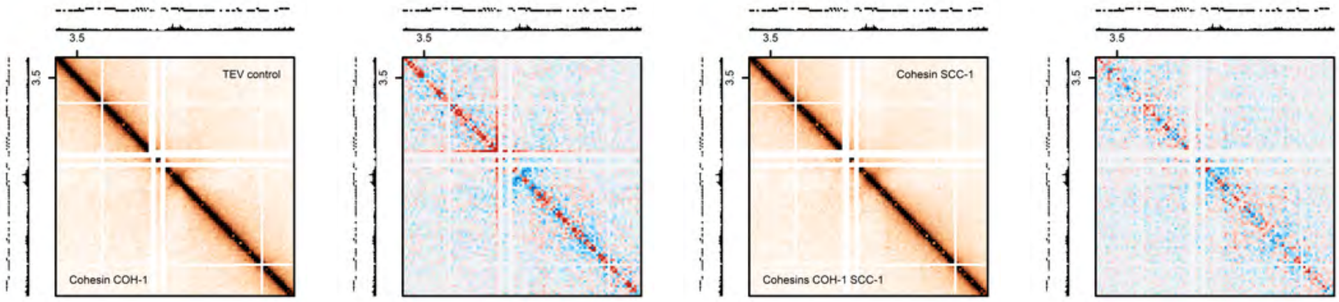

chrI:11837001-12037000

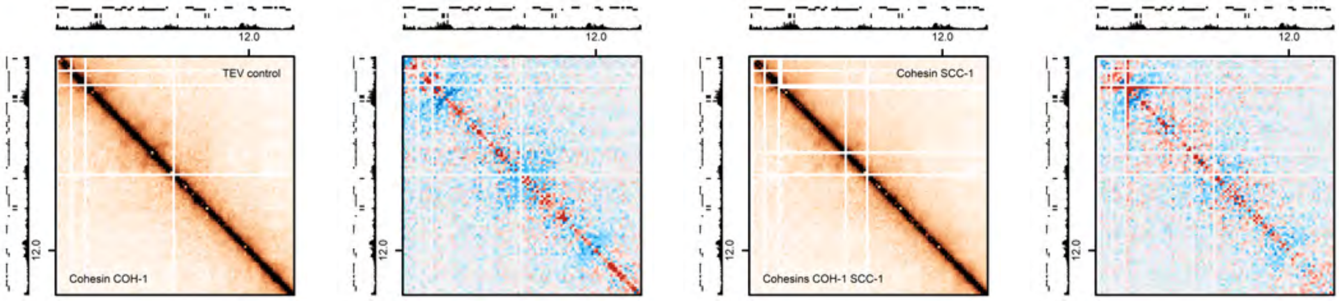

chrI:3413001-3613000

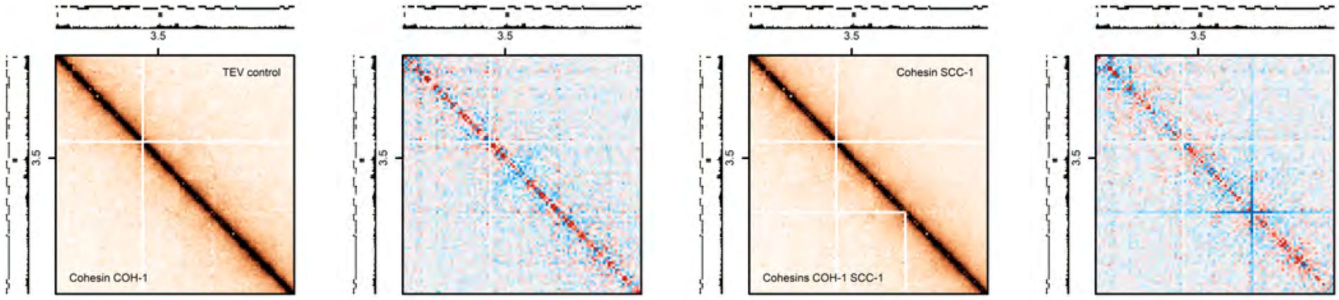

chrIV:563001-763000

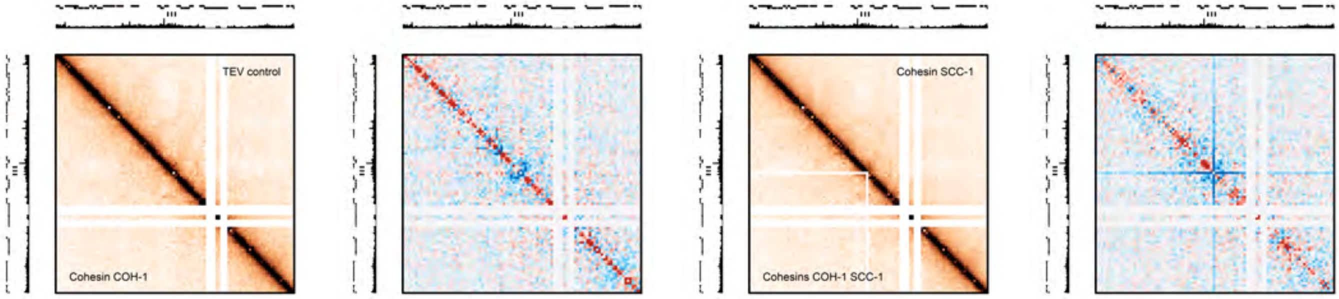

chrIV:4543001-4743000

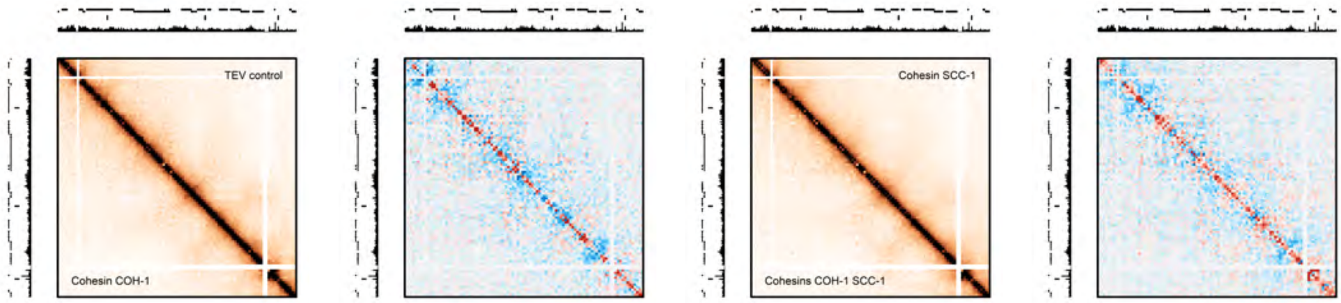

chrI:7611001-7811000

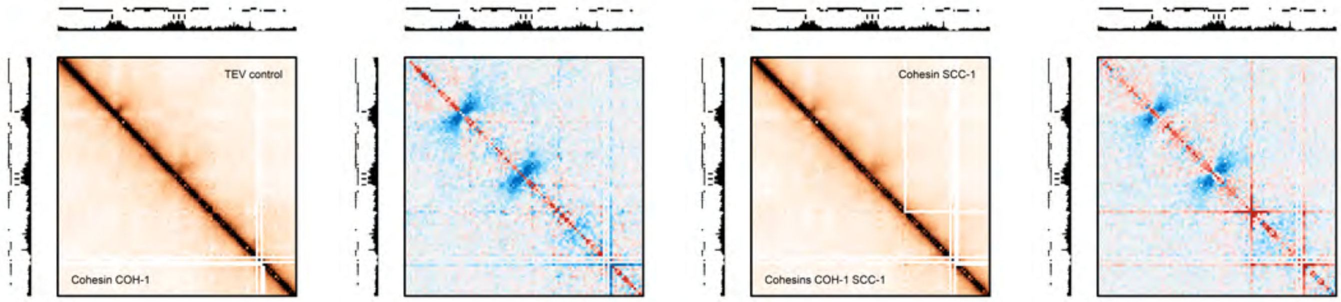

chrX:17117001-17317000

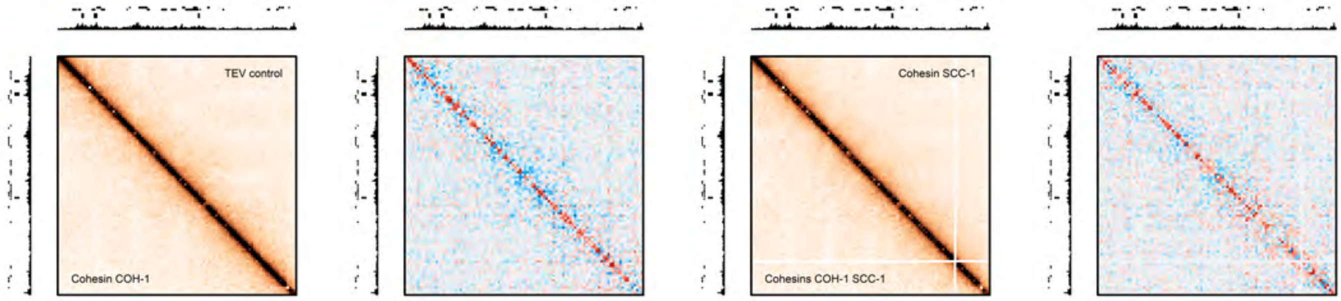

chrI:5493001-5693000

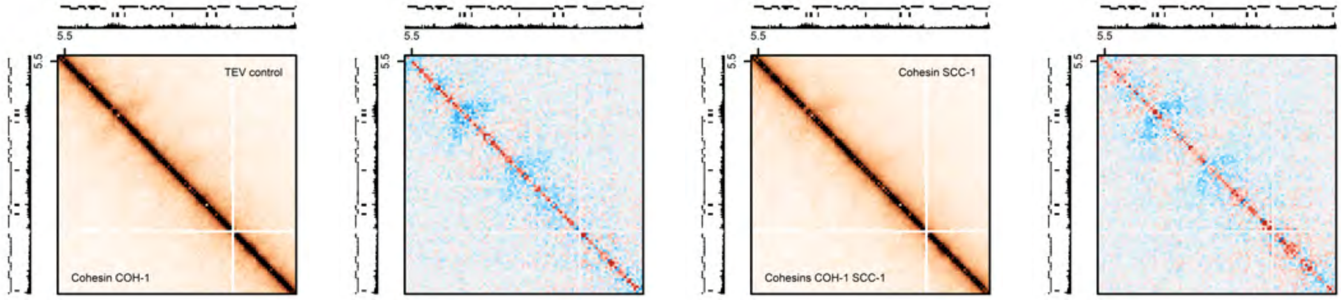

chrII:217001-417000

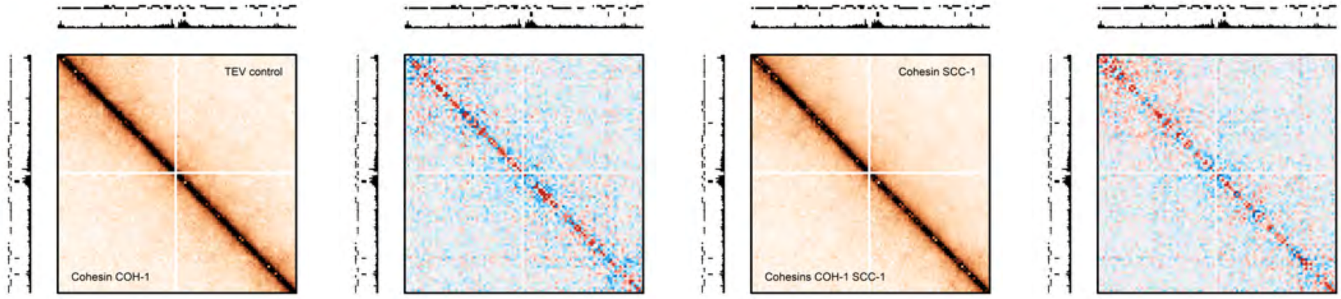

chrX:5879001-6079000

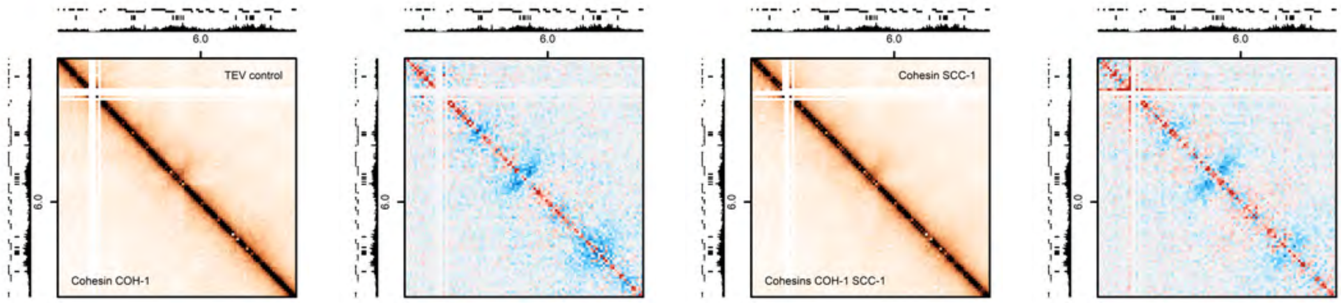

chrIII:3999001-4199000

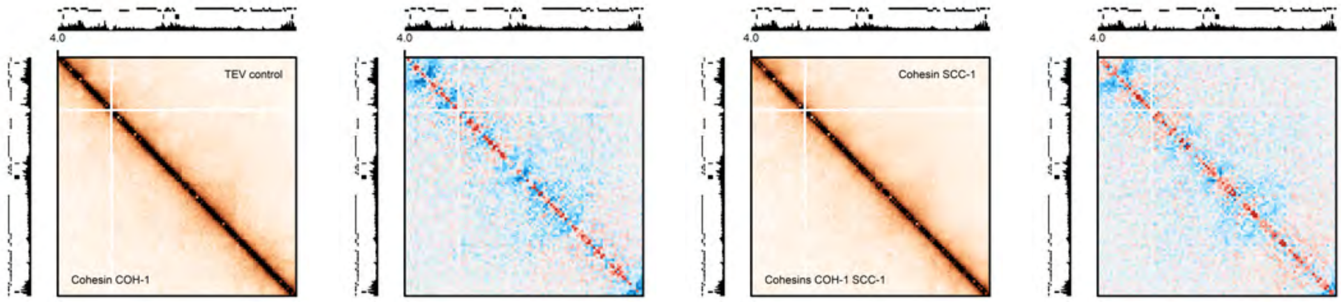

chrV:1673001-1873000

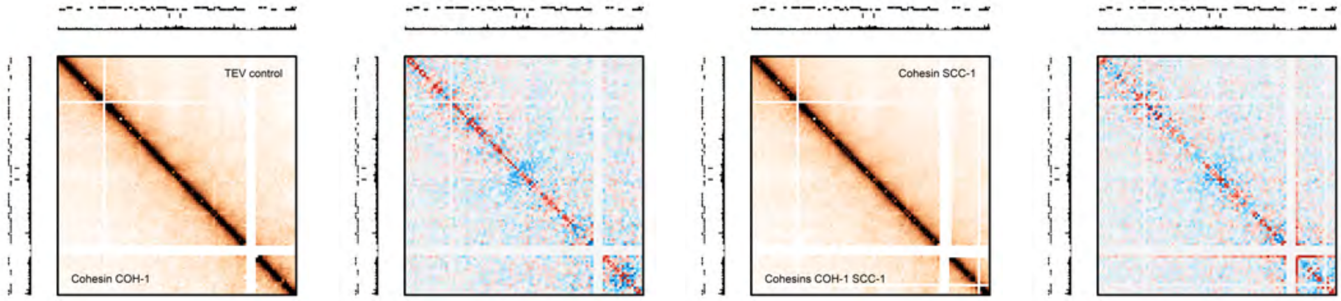

chrIII:4559001-4759000

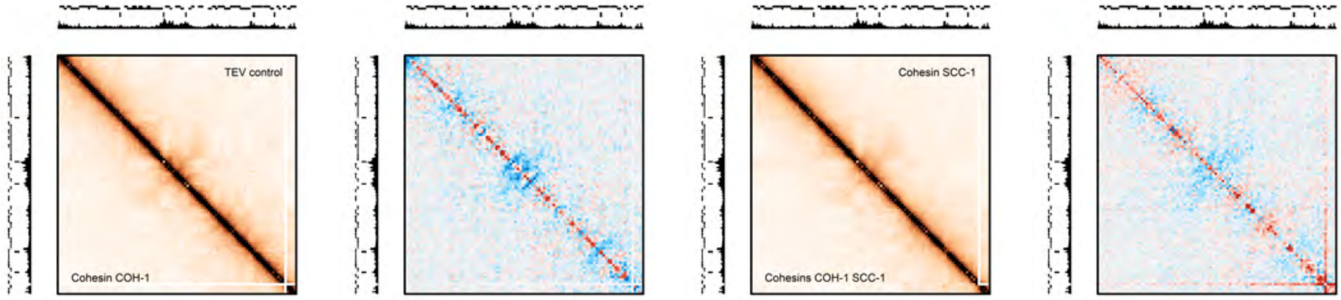

chrIV:5239001-5439000

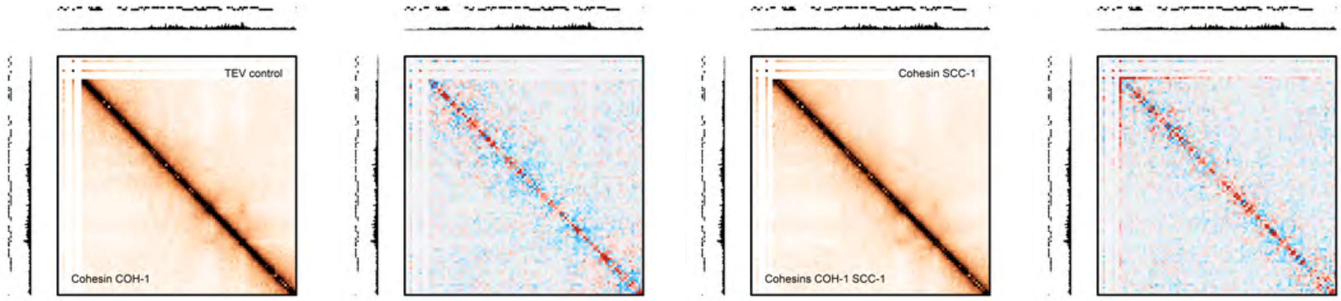

chrIV:855001-1055000

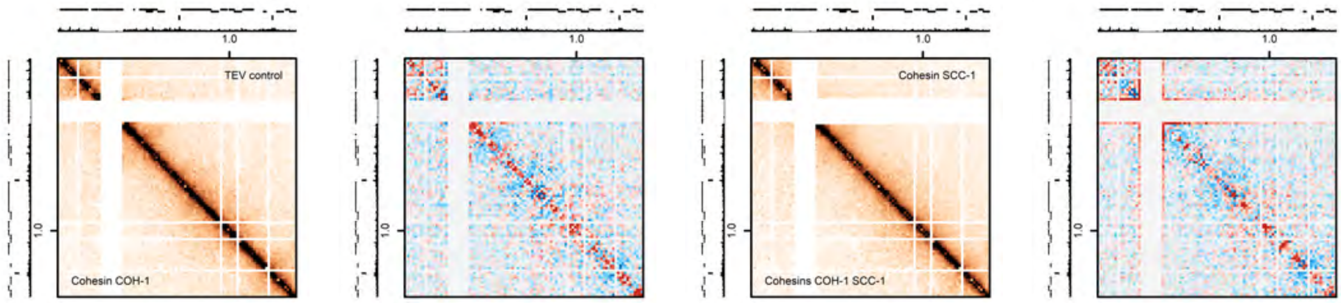

chrII:6611001-6811000

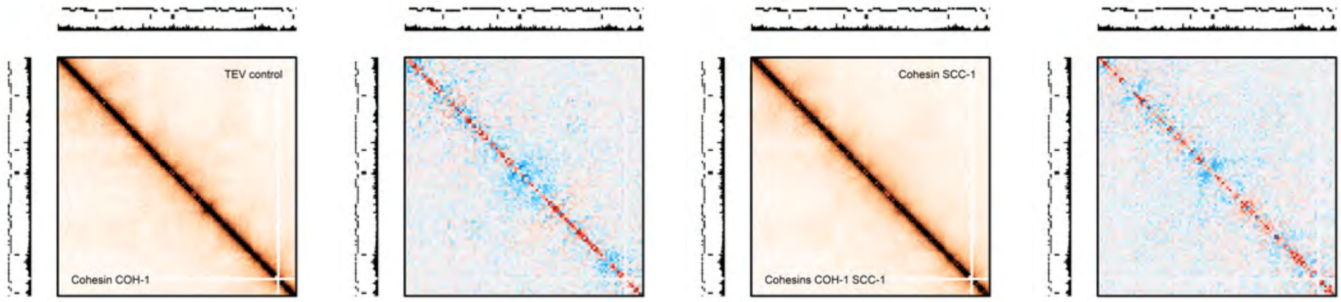

chrI:10307001-10507000

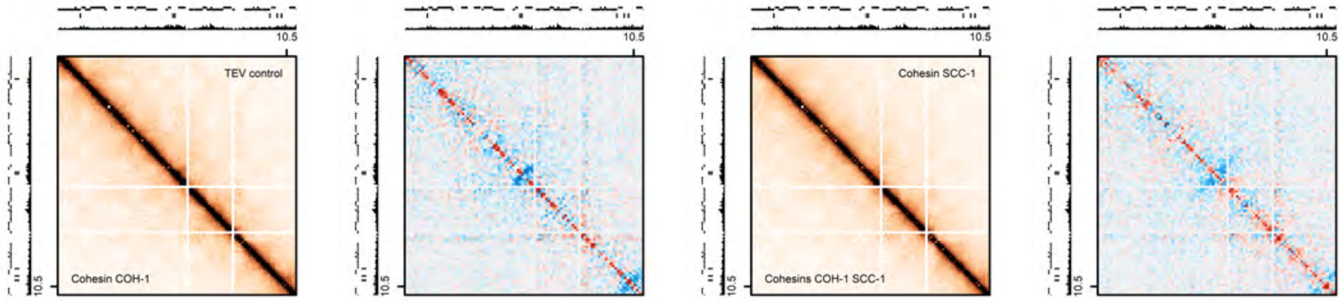

chrI:14425001-14625000

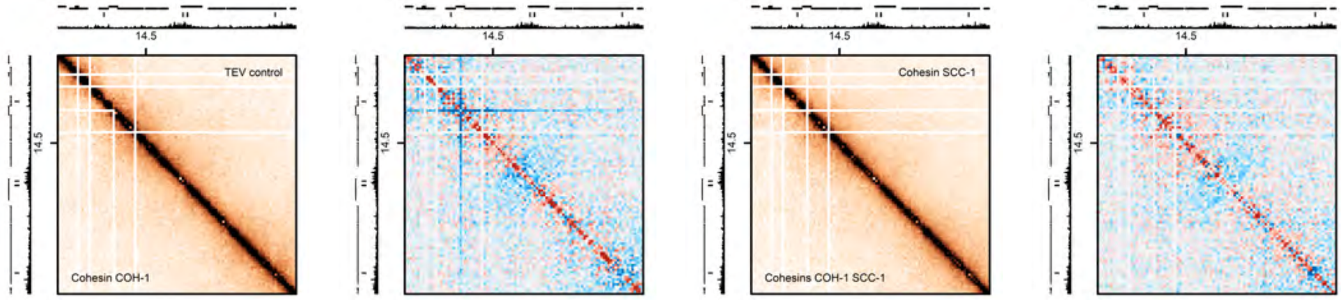

chrV:5457001-5657000

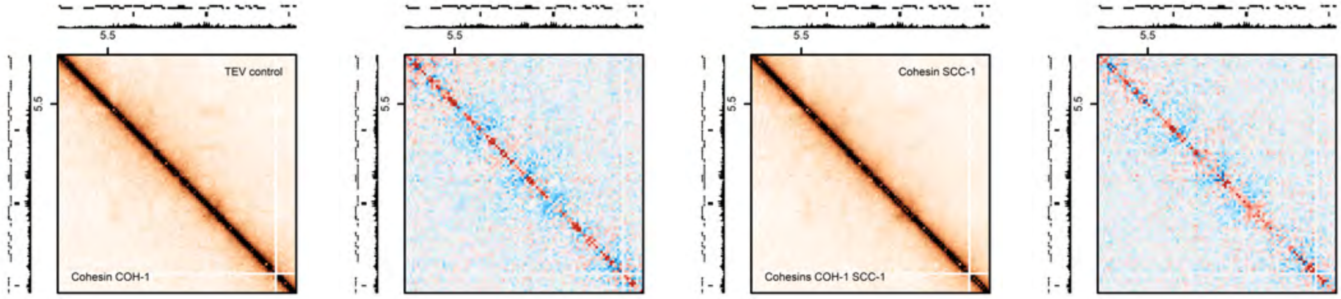

chrV:17023001-17223000

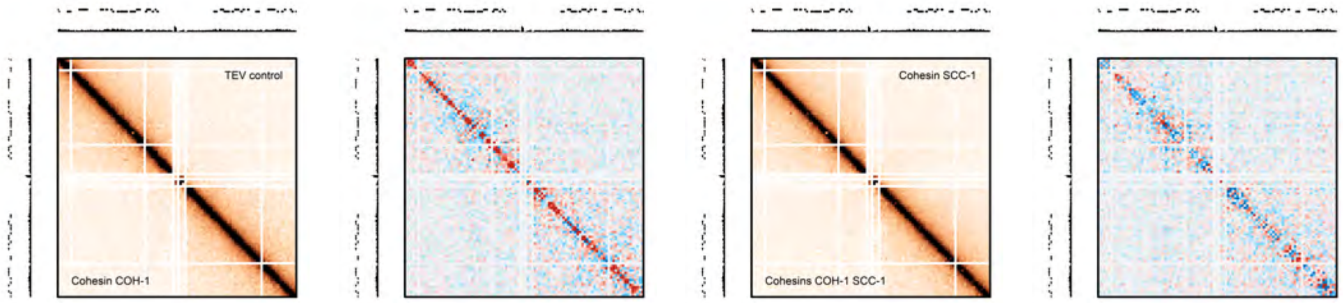

chrIII:12861001-13061000

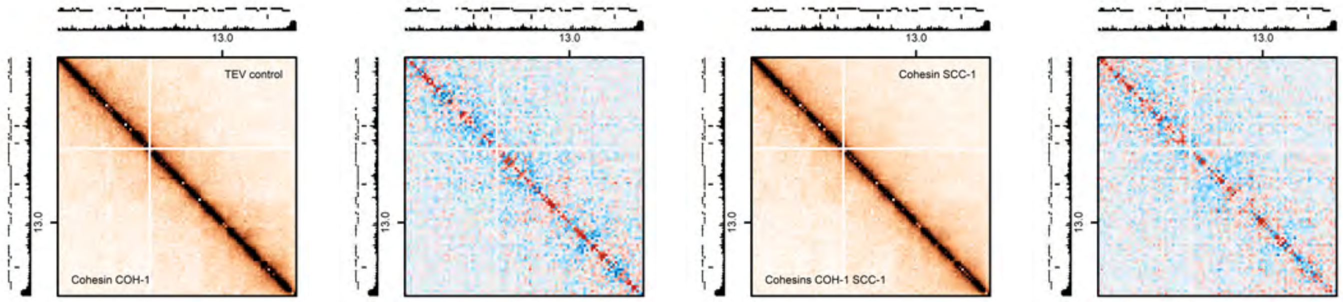

chrIV:1659001-1859000

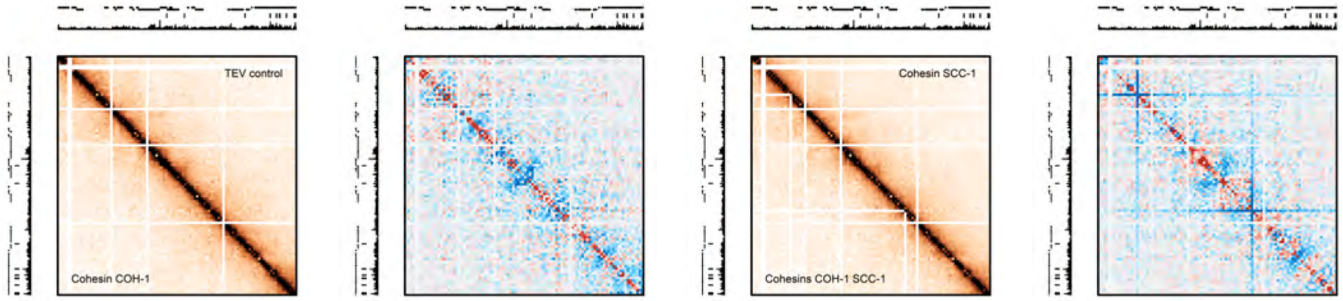

chrIV:3423001-3623000

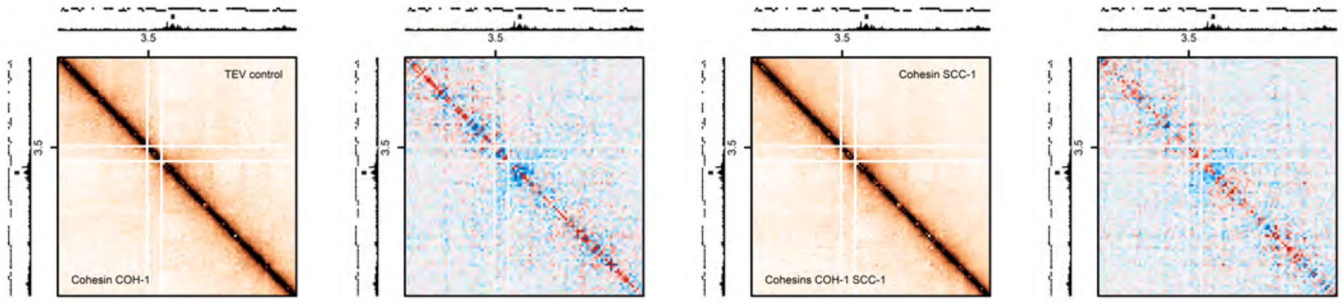

chrX:6707001-6907000

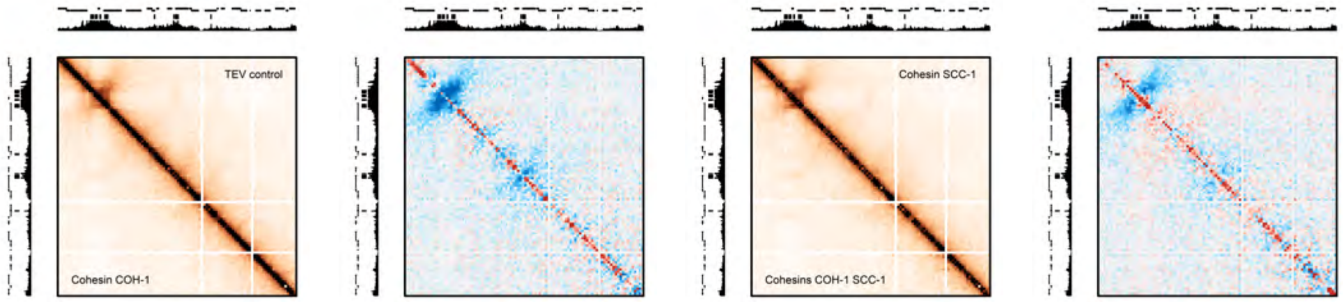

chrII:9017001-9217000

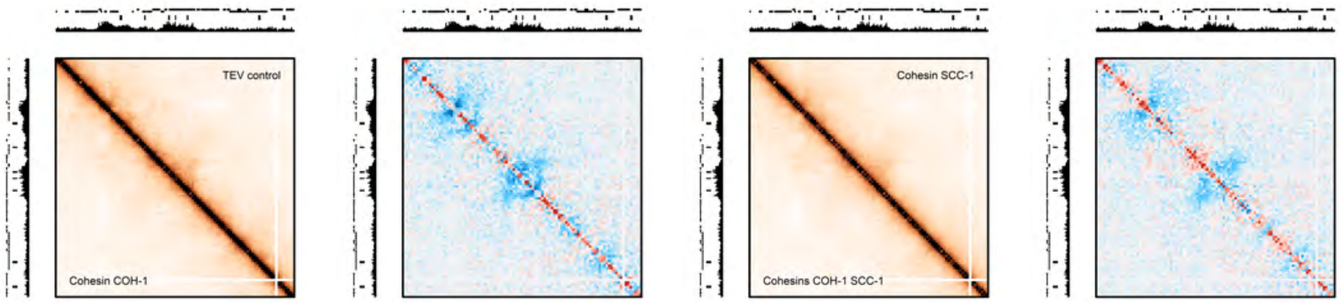

chrIV:12595001-12795000

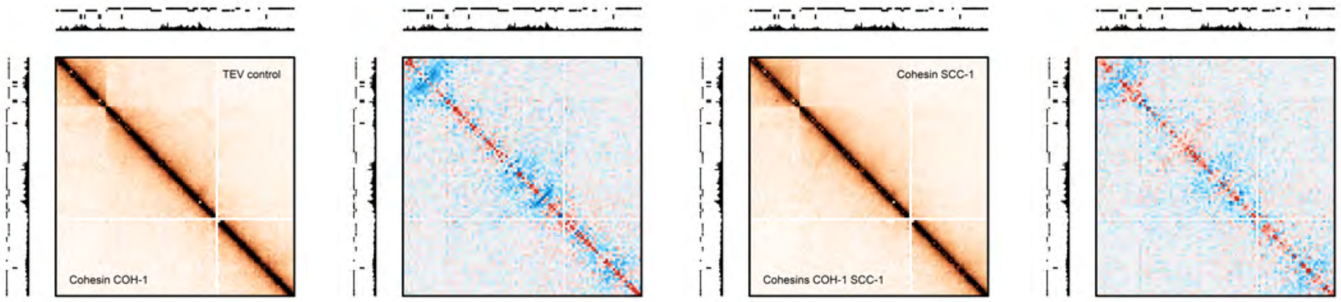

chrII:6841001-7041000

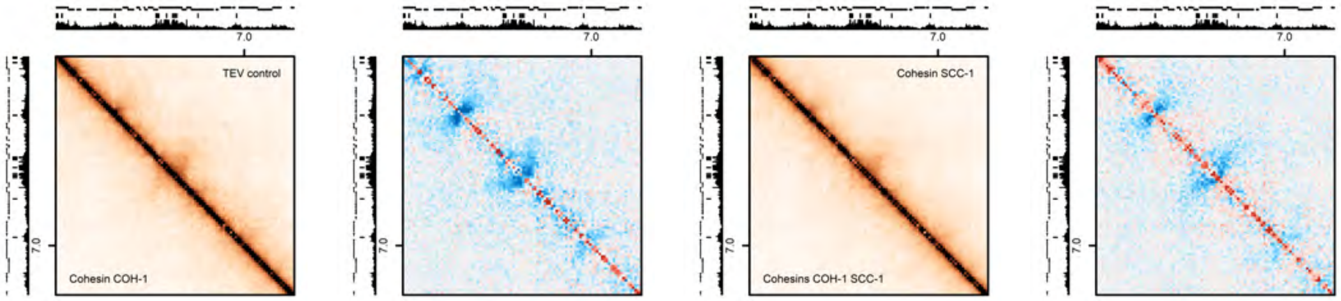

chrIV:3249001-3449000

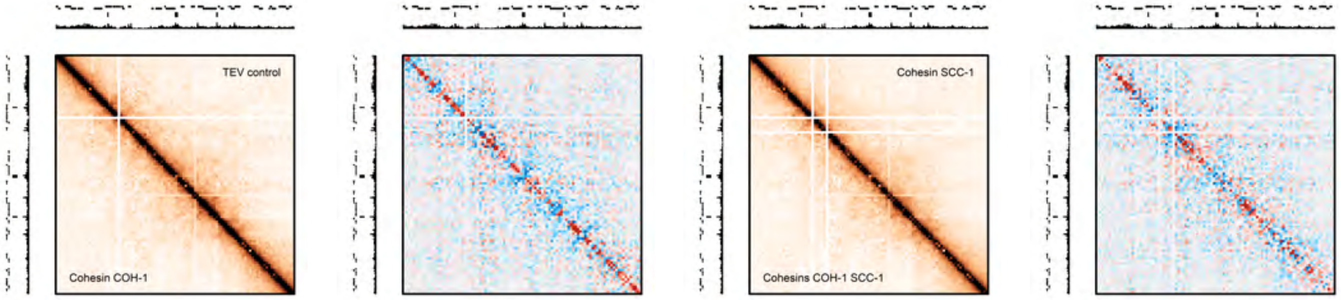

chrII:4053001-4253000

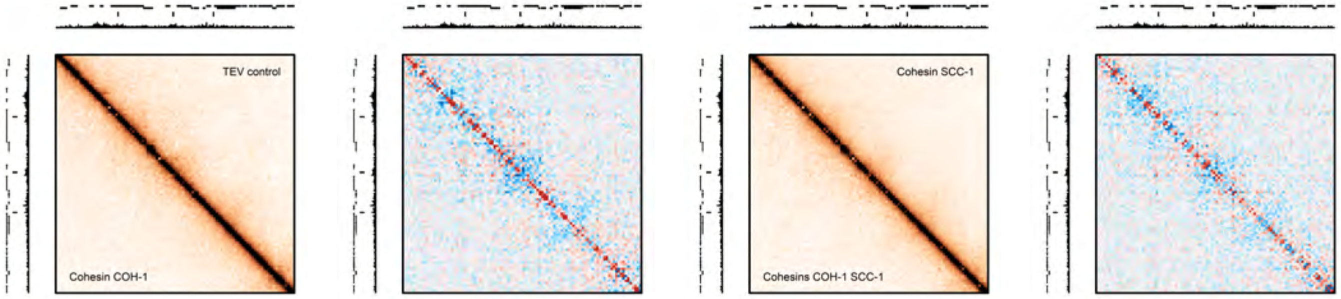

chrIV:13401001-13601000

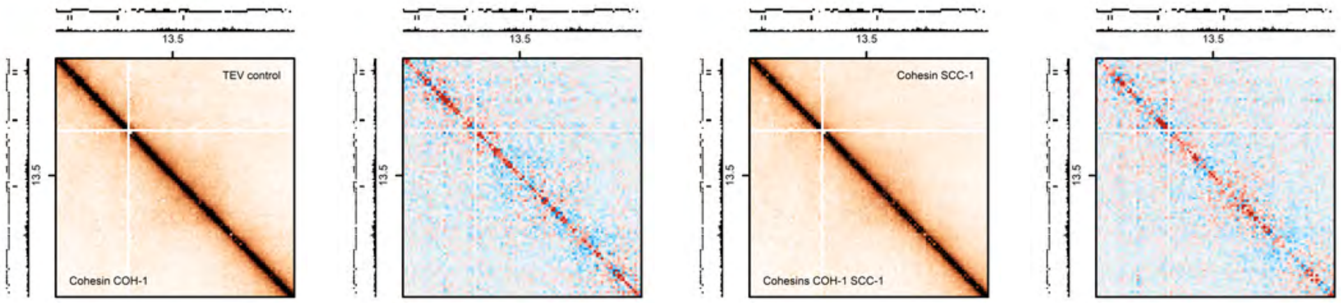

chrIII:12741001-12941000

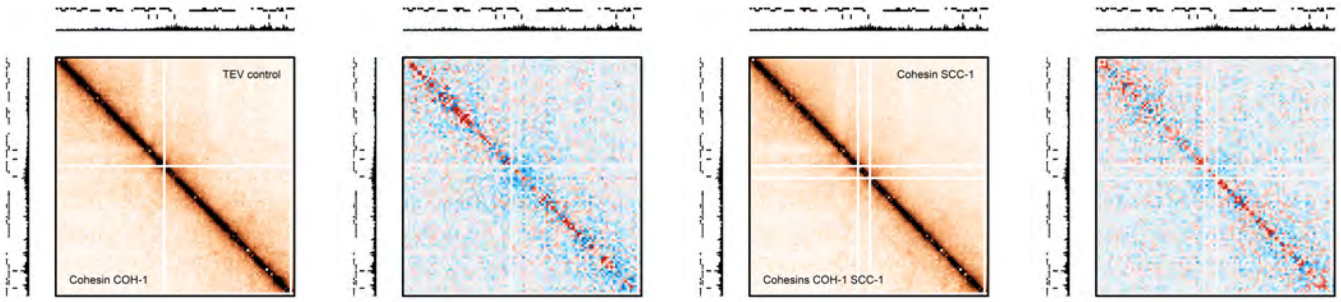

chrII:1851001-2051000

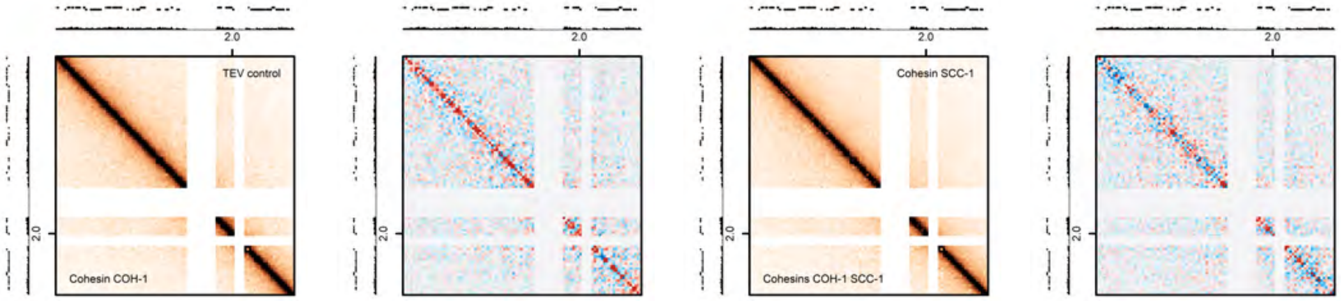

chrIV:14377001-14577000

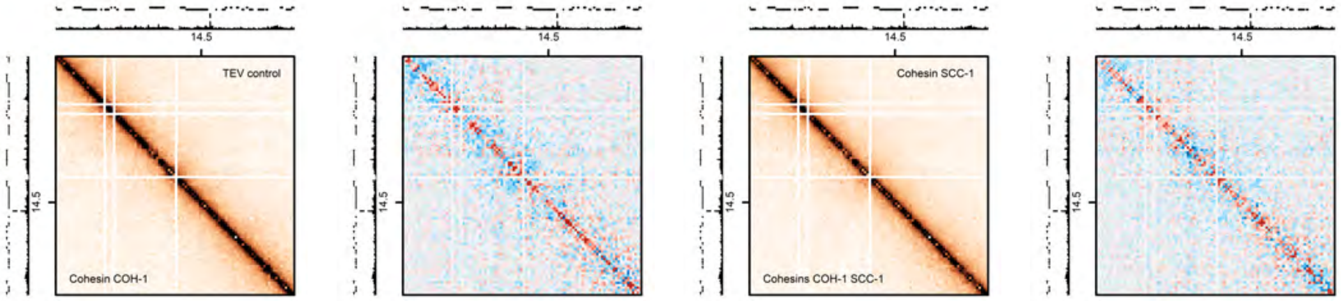

chrIV:11905001-12105000

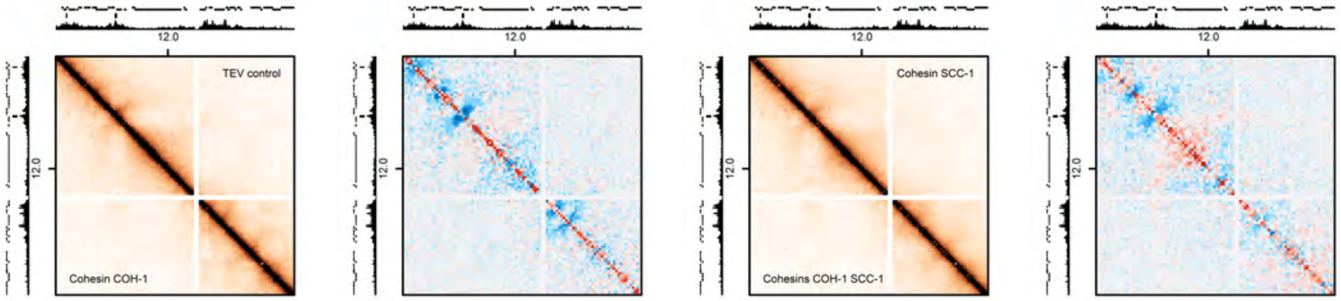

chrV:16813001-17013000

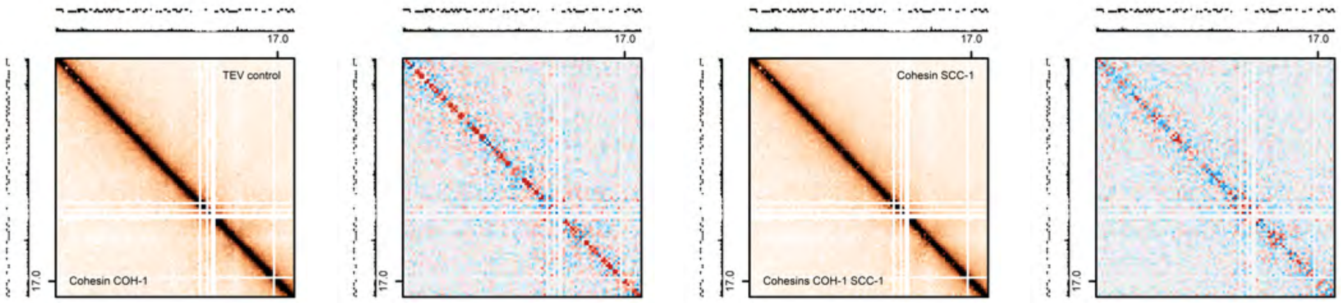

chrI:4335001-4535000

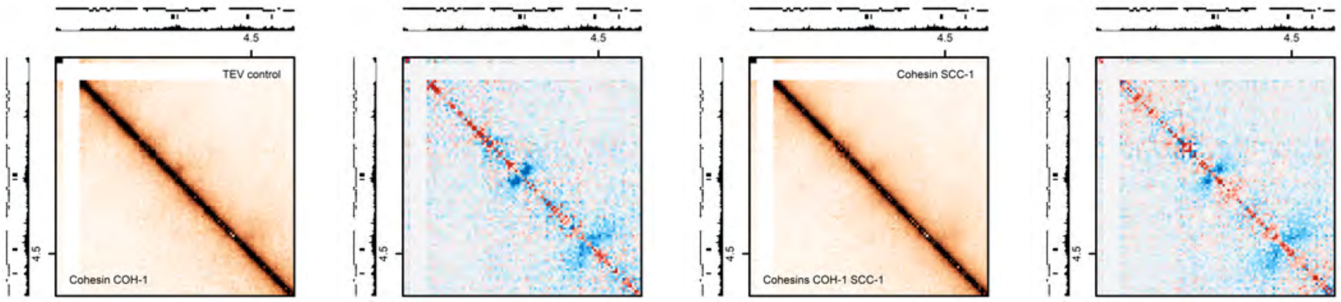

chrIII:8319001-8519000

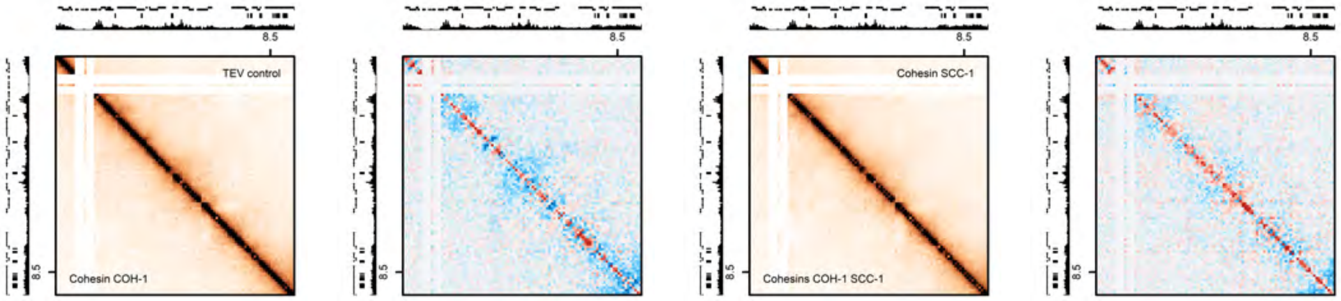

chrX:7487001-7687000

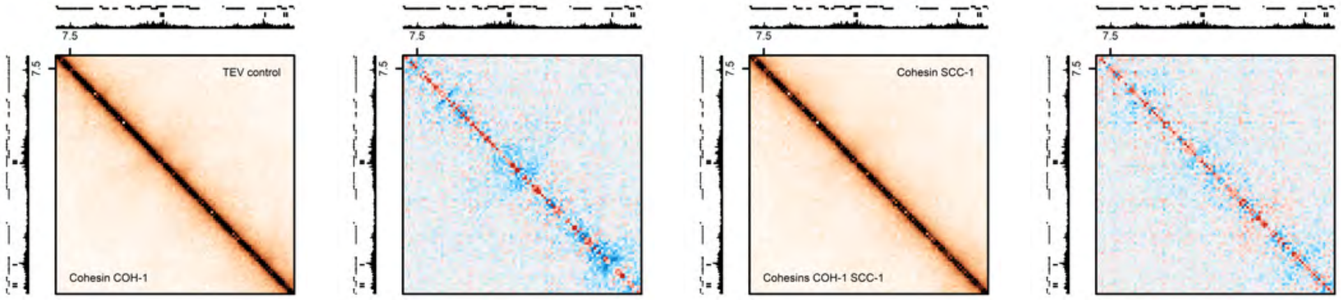

chrII:4921001-5121000

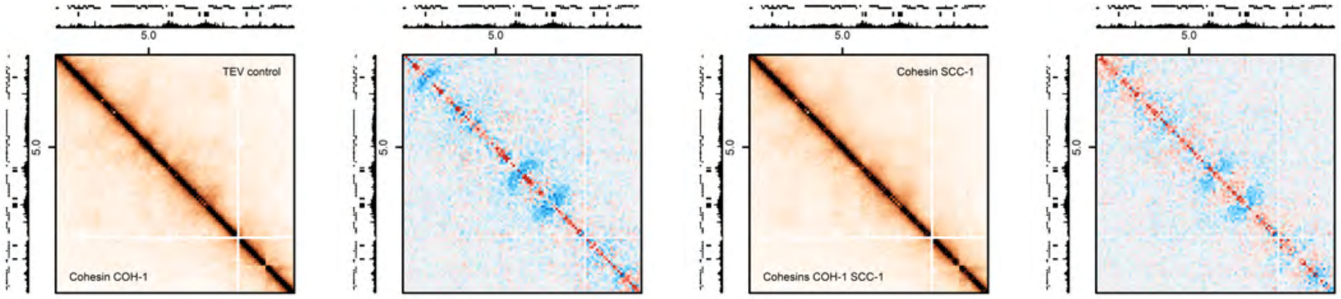

chrX:8835001-9035000

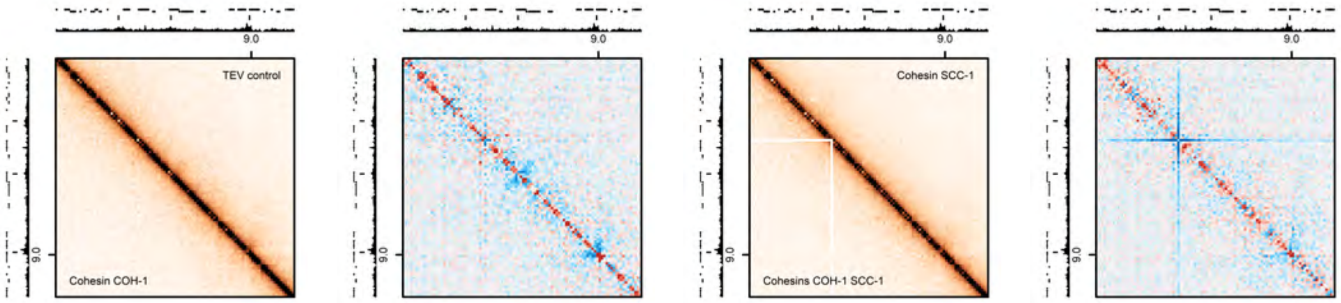

chrI:897001-1097000

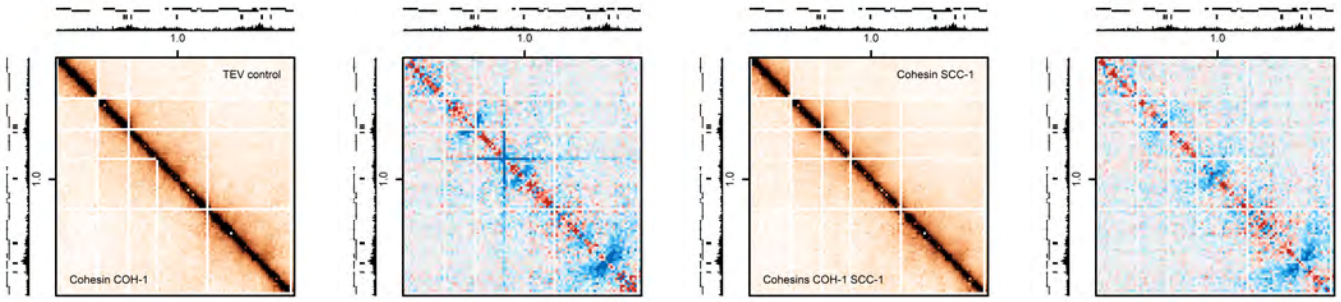

chrIV:11853001-12053000

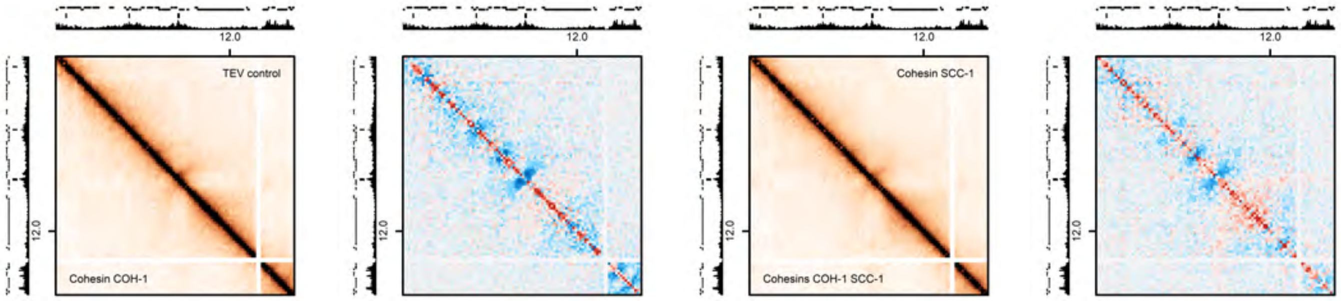

chrII:1243001-1443000

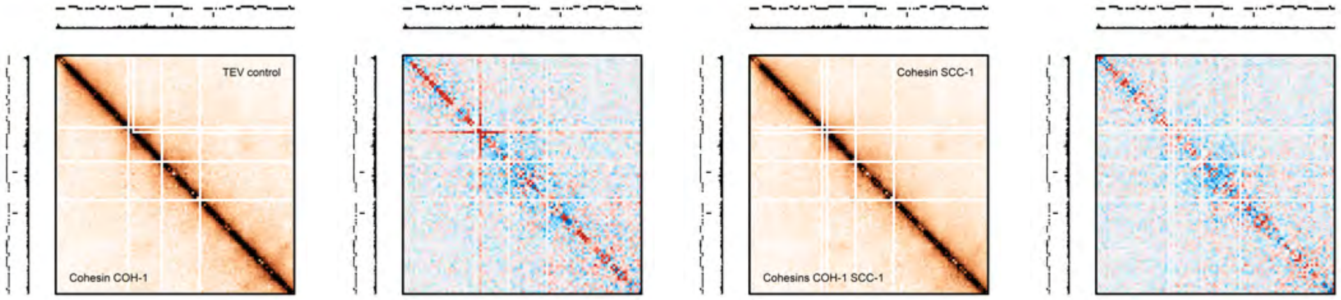

chrX:9903001-10103000

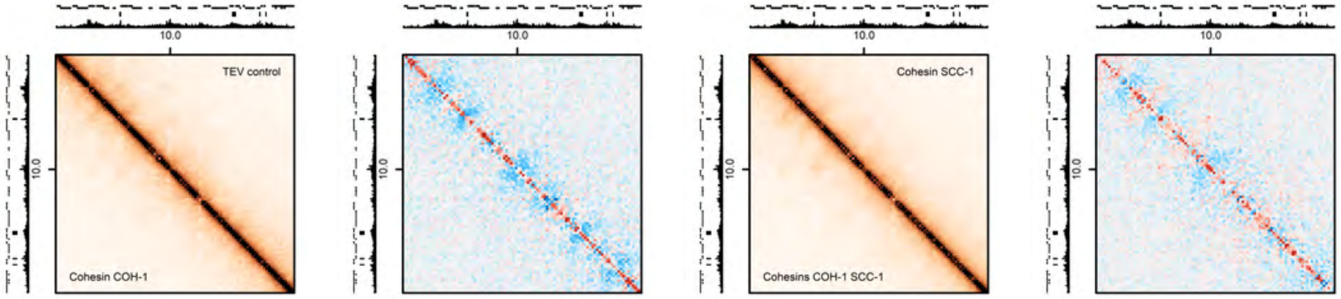

chrI:7445001-7645000

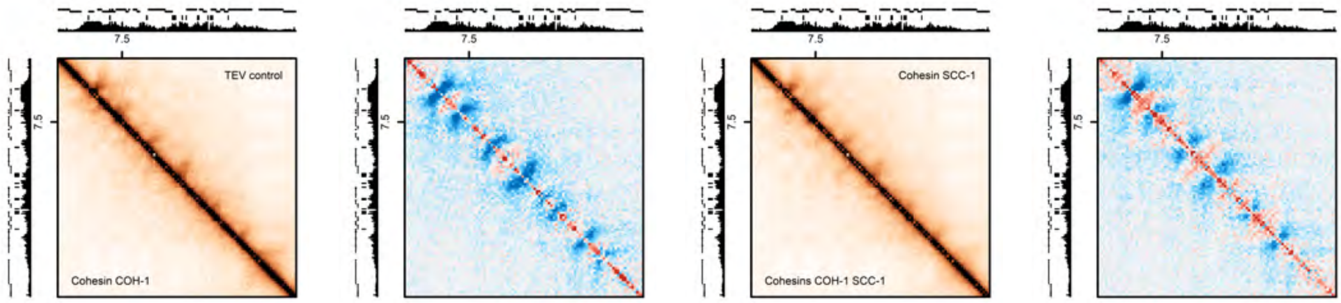

chrIII:9521001-9721000

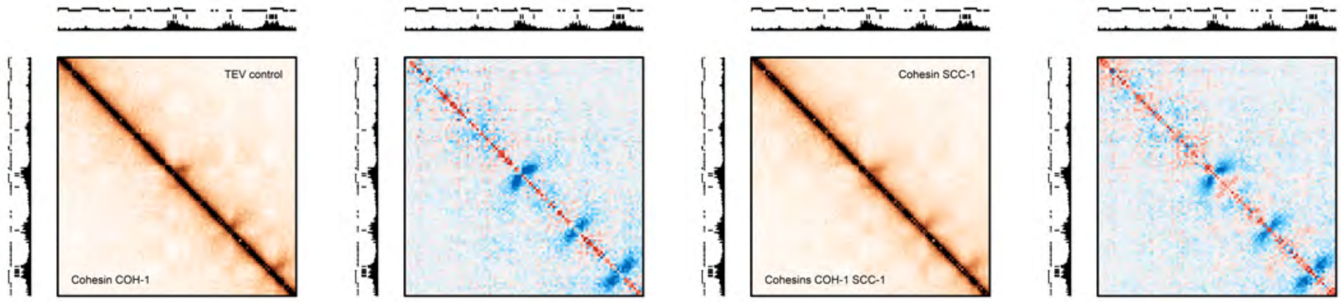

chrII:2241001-2441000

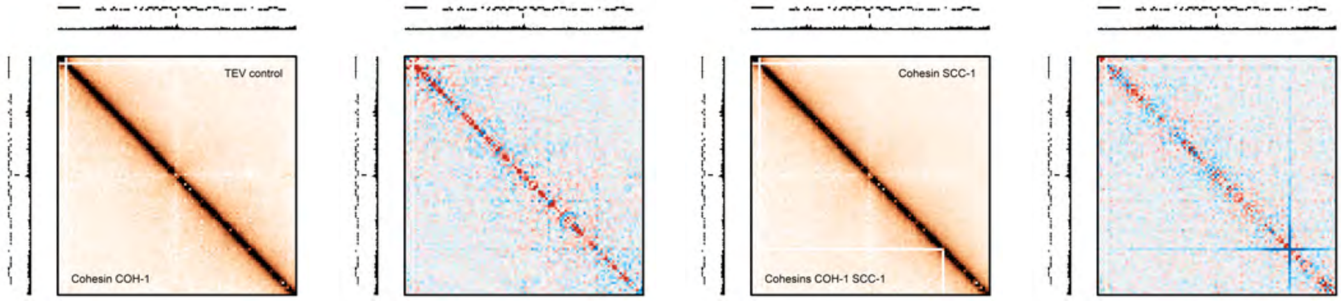

chrIV:14175001-14375000

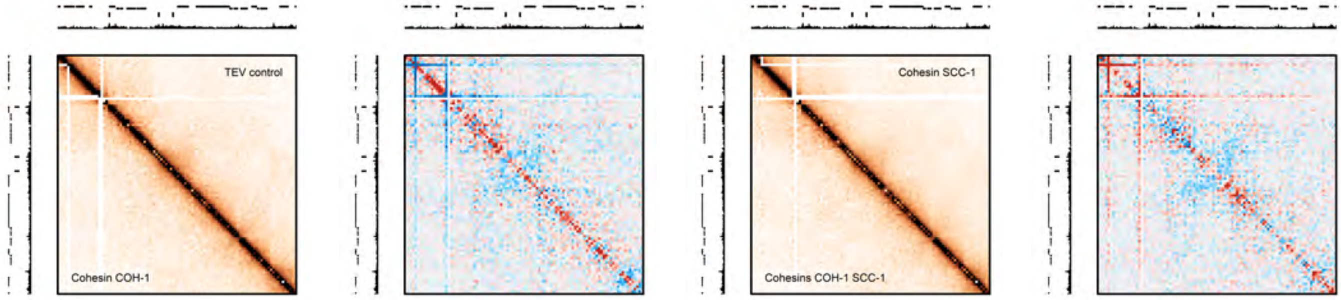

chrV:20169001-20369000

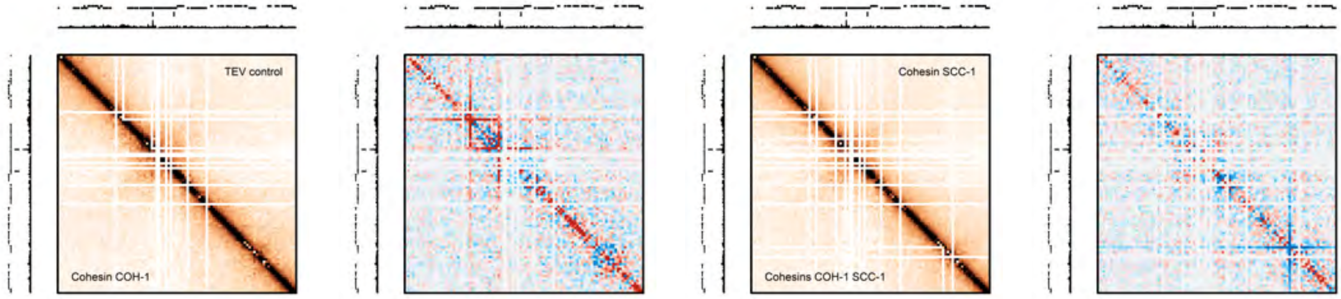

chrIII:13533001-13733000

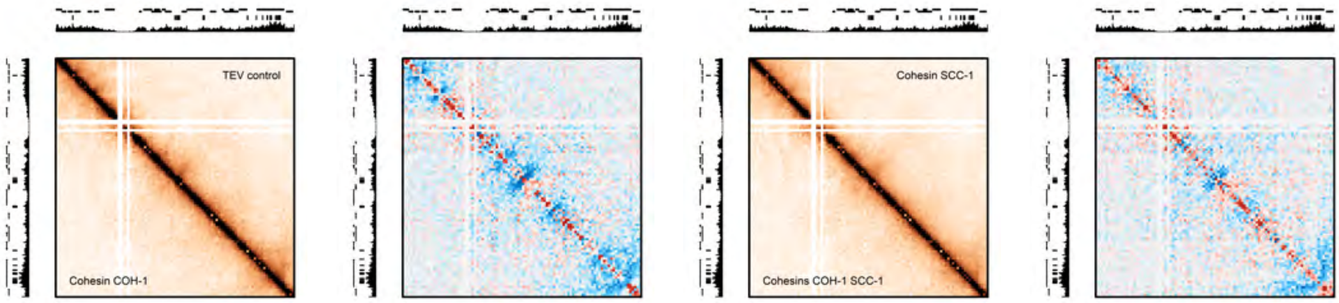

chrII:10197001-10397000

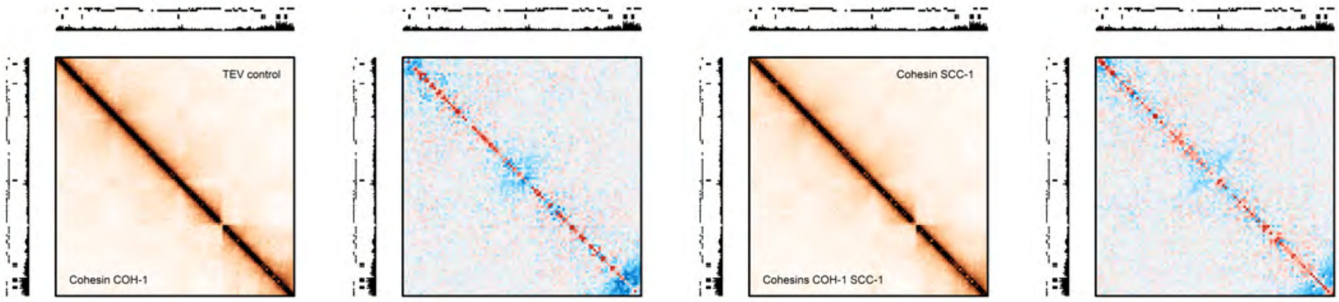

chrI:4079001-4279000

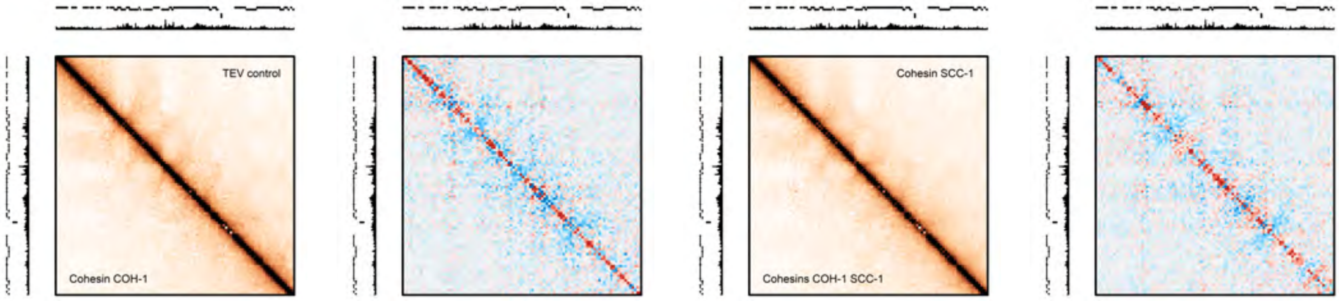

chrI:7117001-7317000

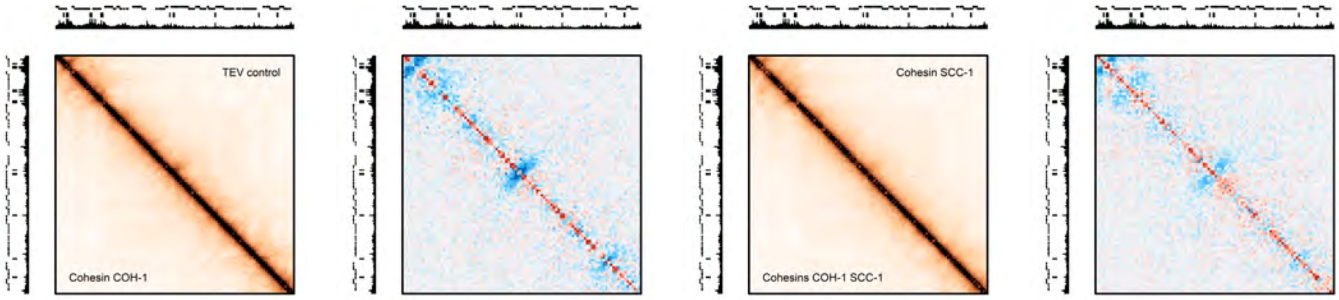

chrV:19251001-19451000

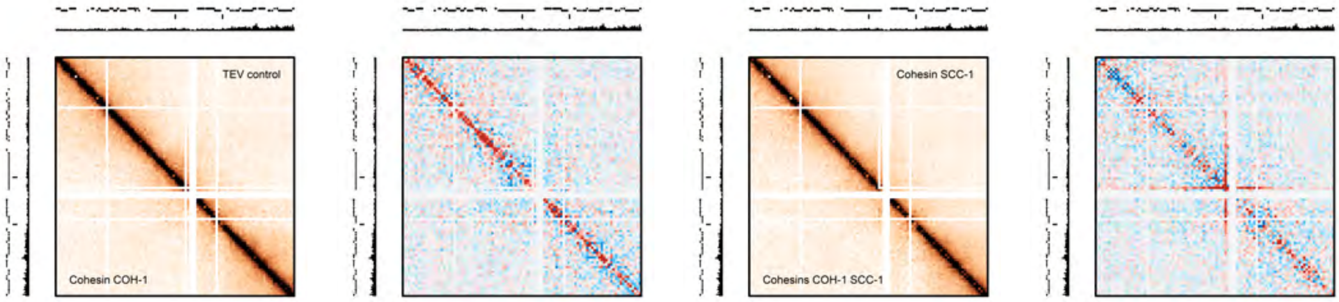

chrV:12537001-12737000

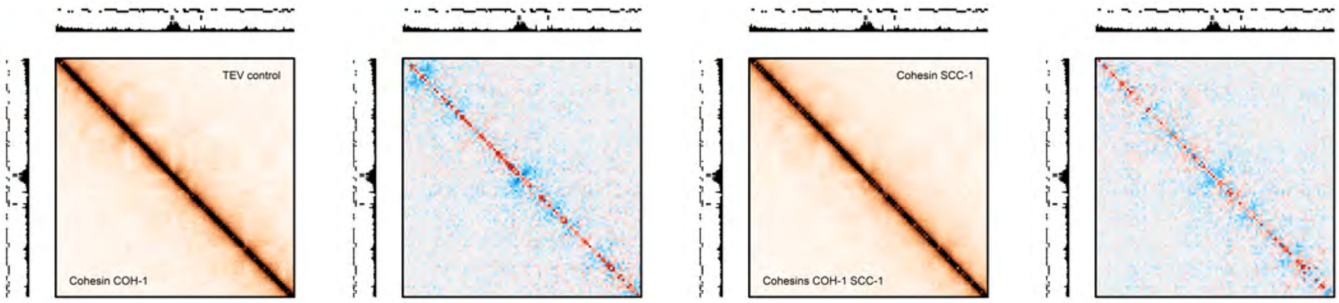

chrI:9937001-10137000

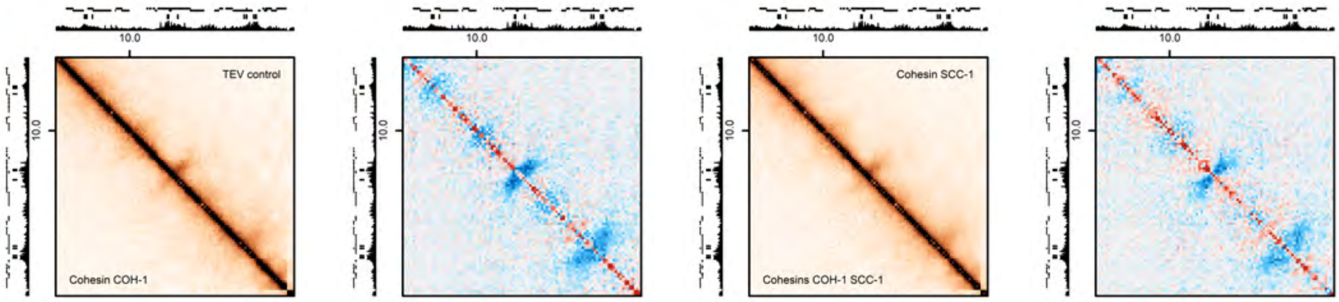

chrIV:16637001-16837000

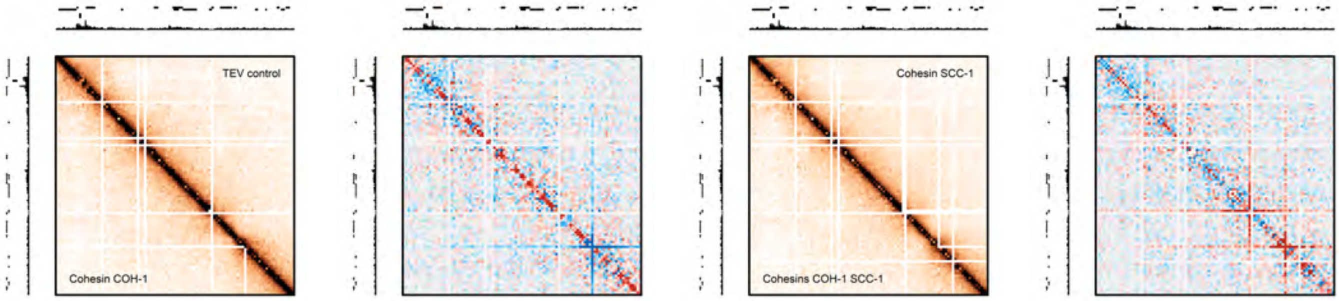

chrX:15941001-16141000

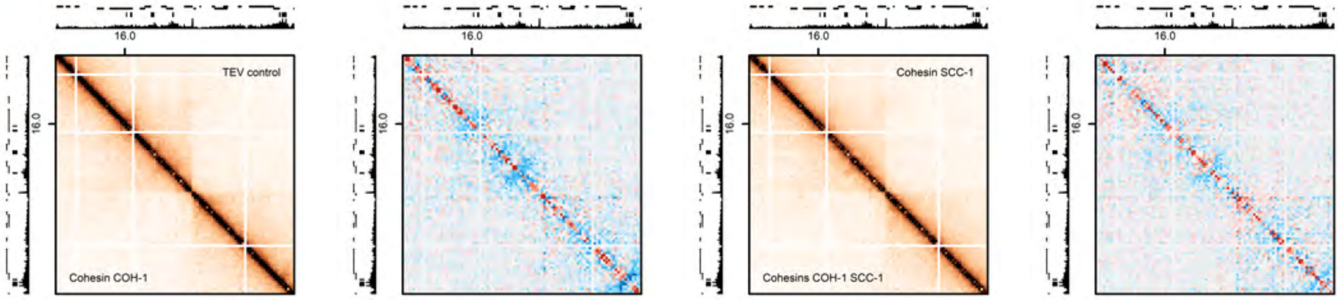

chrX:9539001-9739000

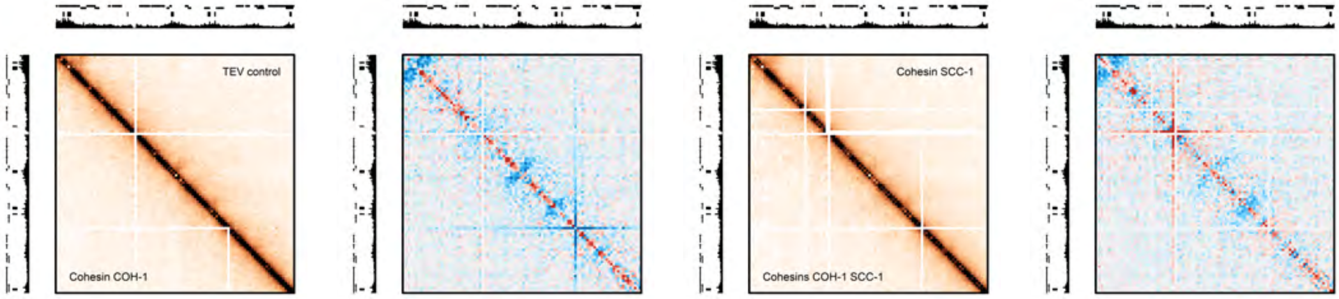

chrIII:6549001-6749000

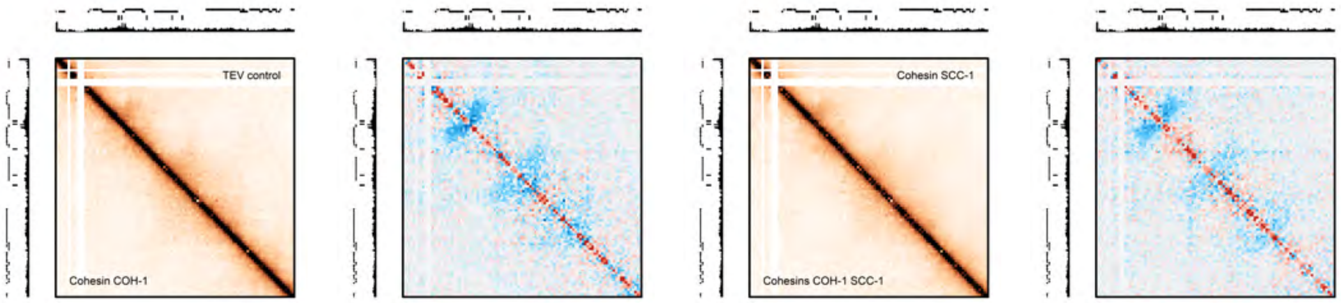

chrIV:7447001-7647000

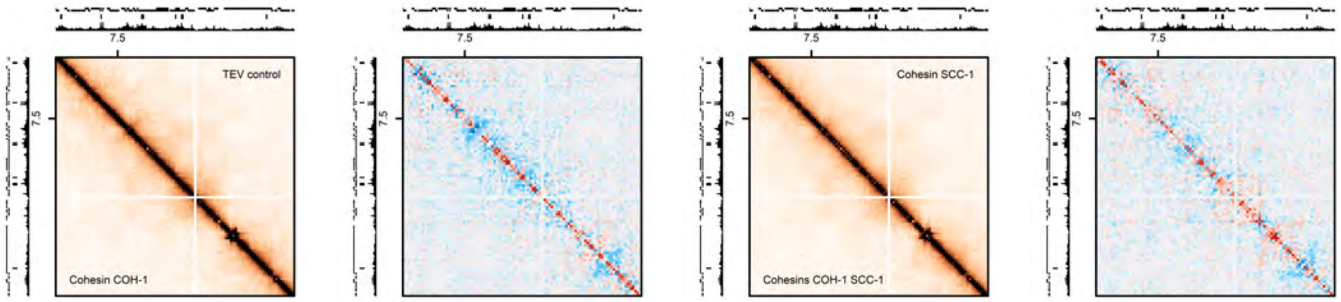

chrIII:955001-1155000

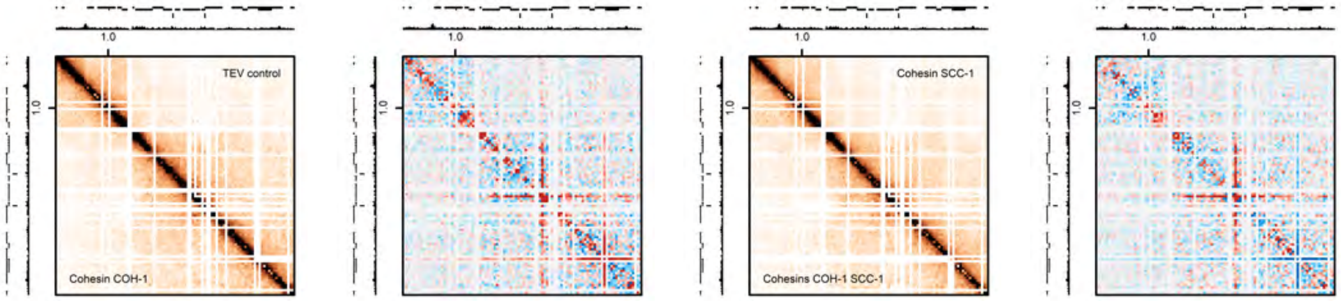

chrI:6631001-6831000

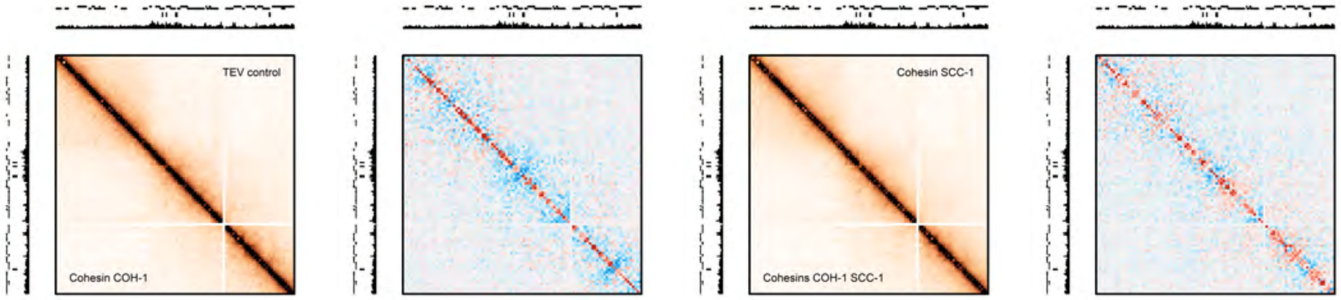

chrV:4865001-5065000

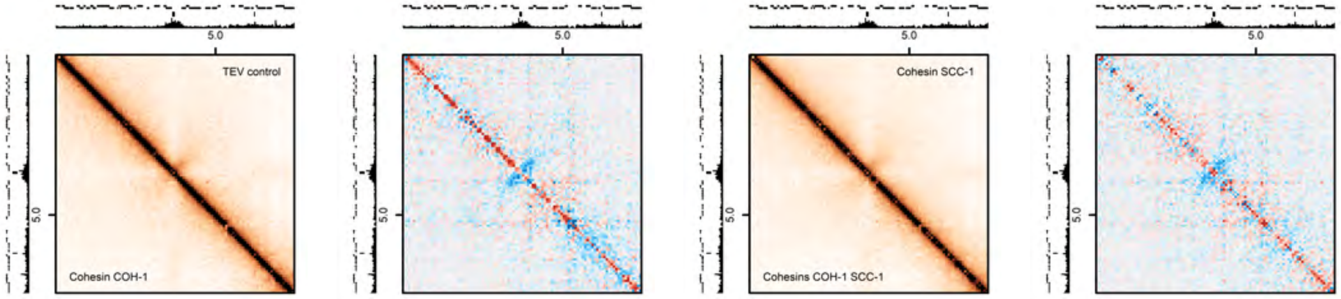

chrIV:17371001-17571000

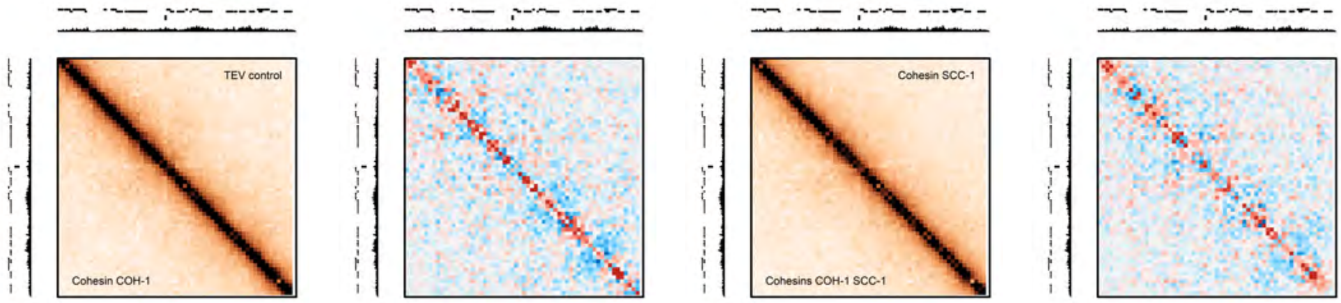

chrIII:3279001-3479000

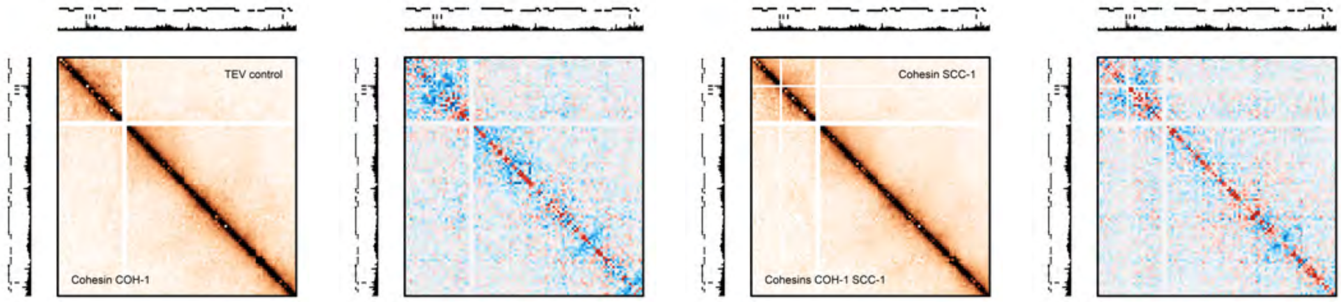

chrV:13807001-14007000

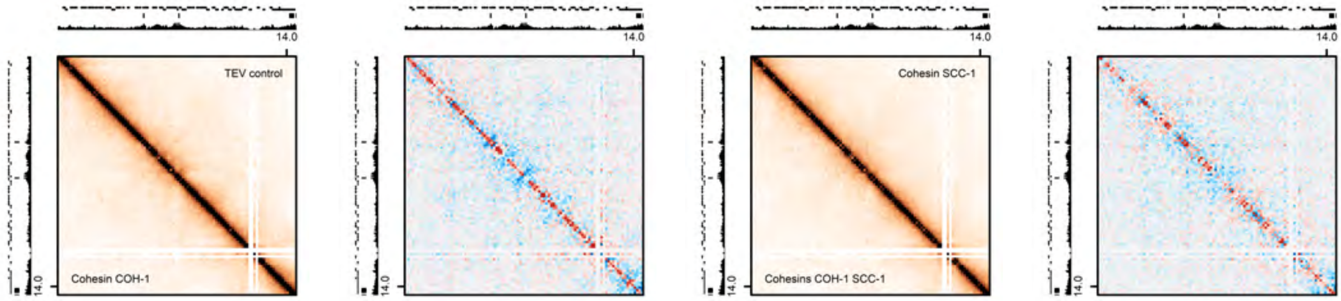

chrI:9241001-9441000

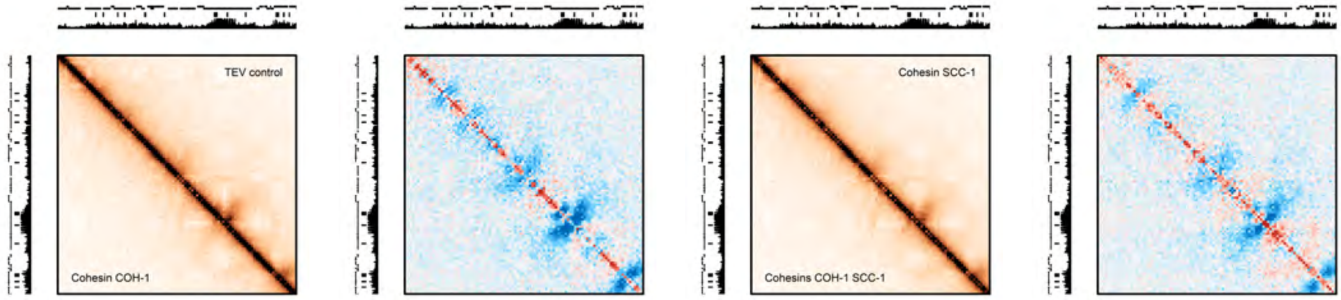

chrV:10843001-11043000

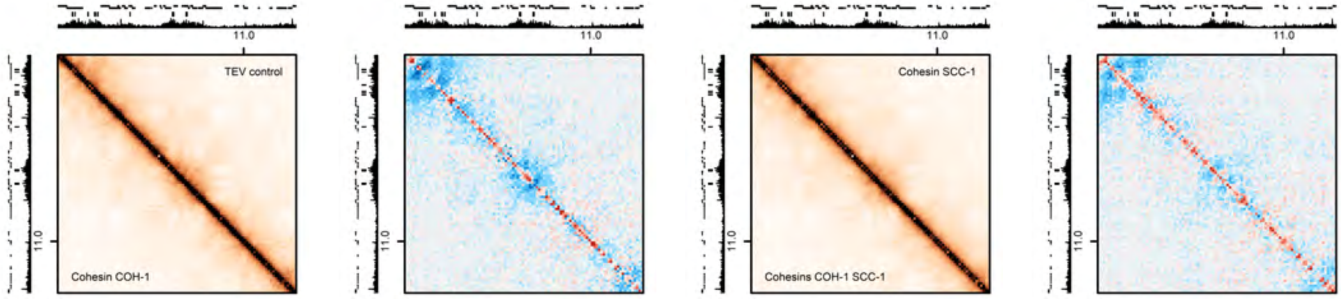

chrIII:3027001-3227000

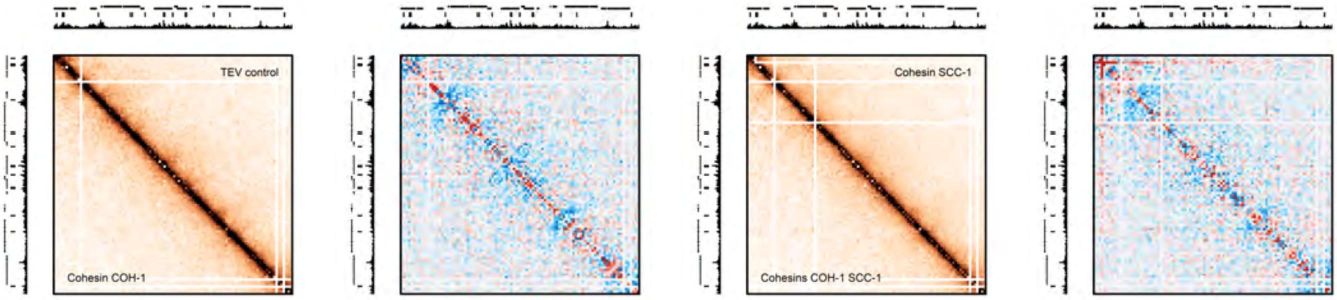

chrI:65001-265000

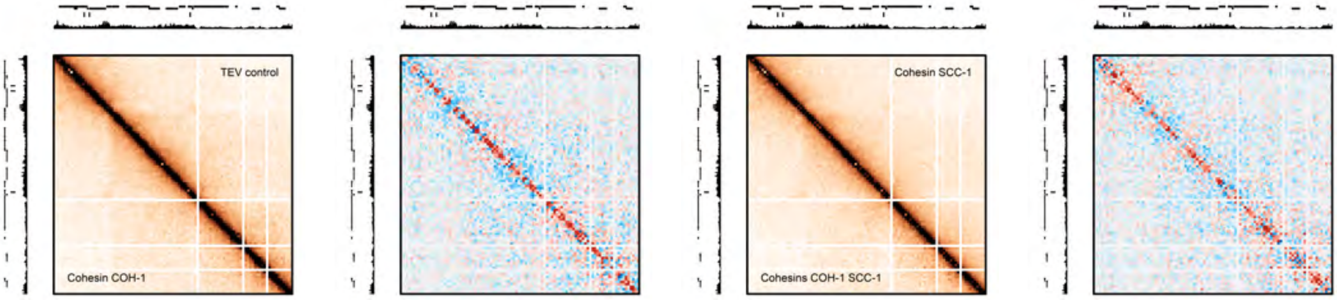

chrIV:1117001-1317000

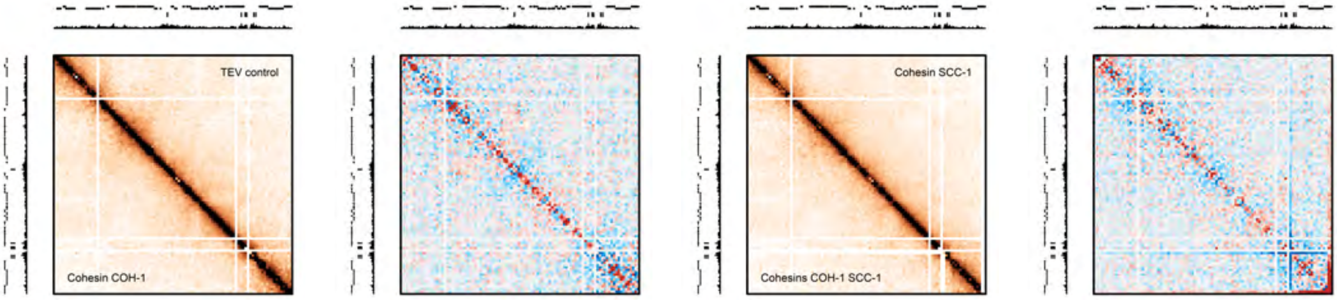

chrIII:787001-987000

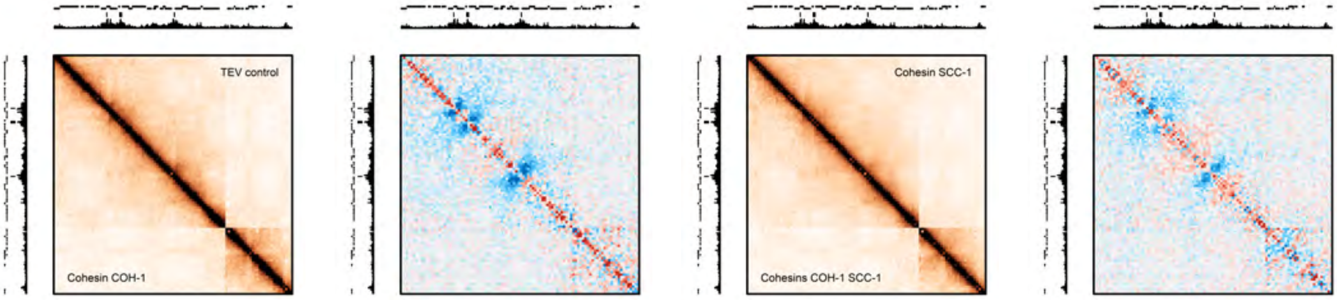

chrV:12797001-12997000

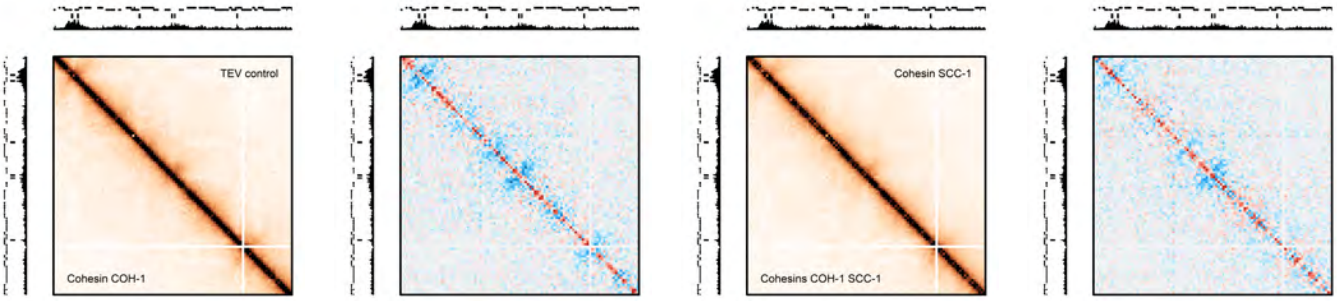

chrX:-56999-143000

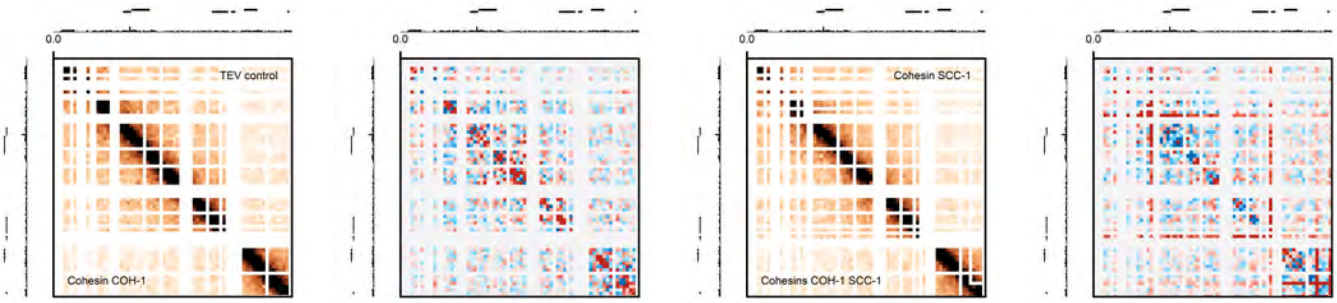

chrII:7677001-7877000

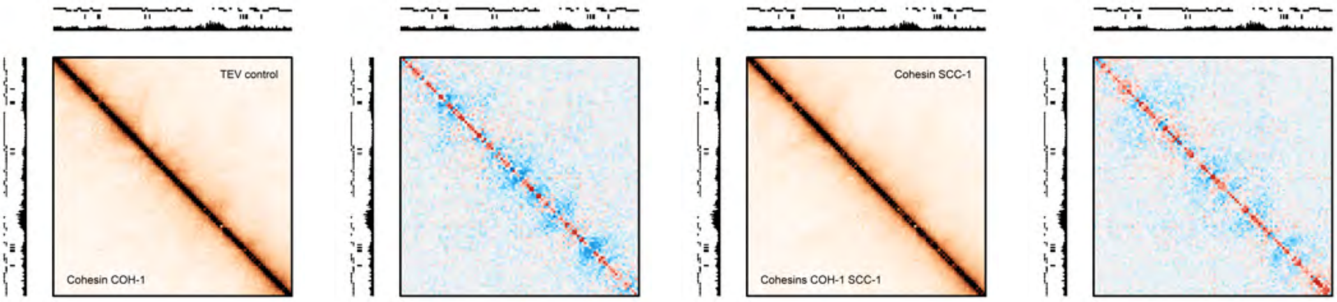

chrI:4685001-4885000

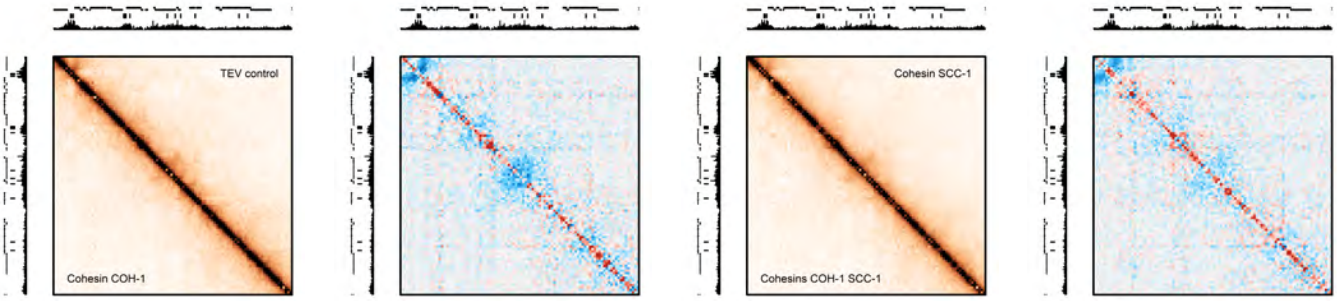

chrV:5595001-5795000

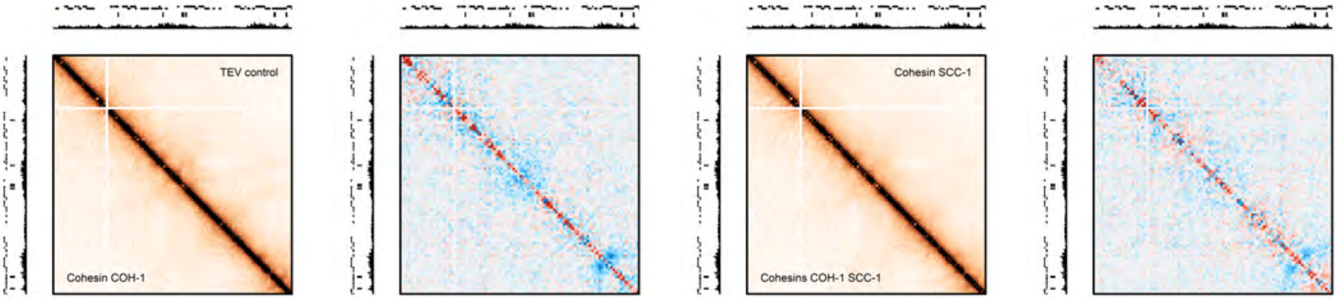

chrV:109001-309000

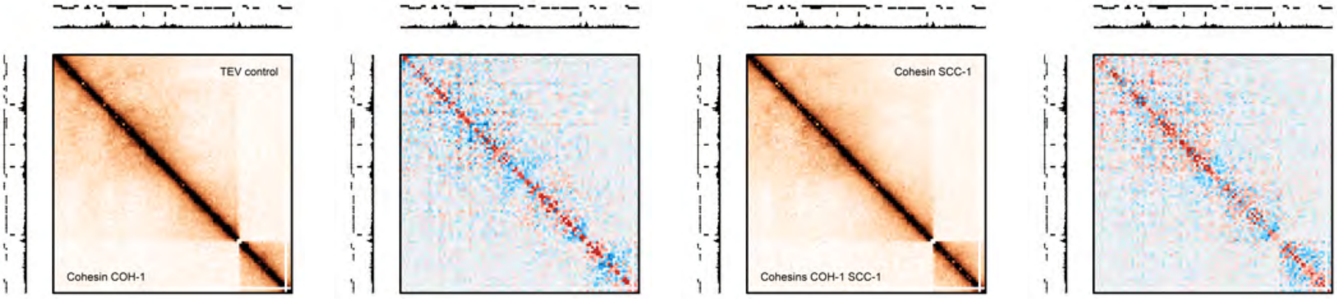

chrIV:2607001-2807000

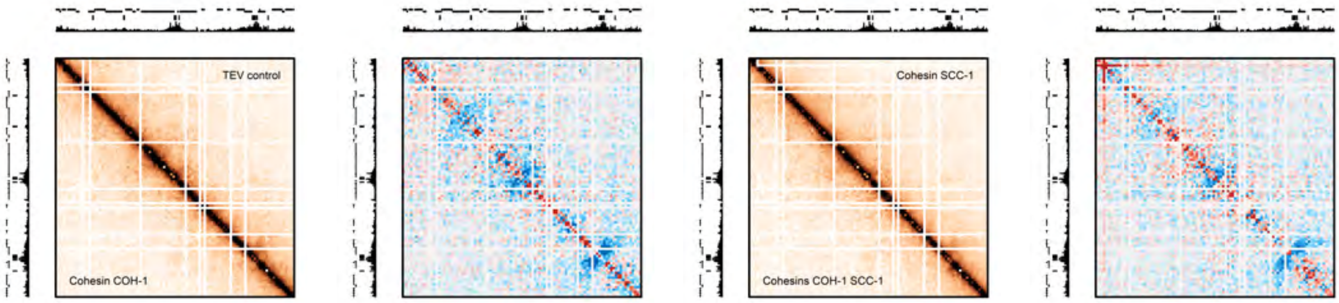

chrII:2627001-2827000

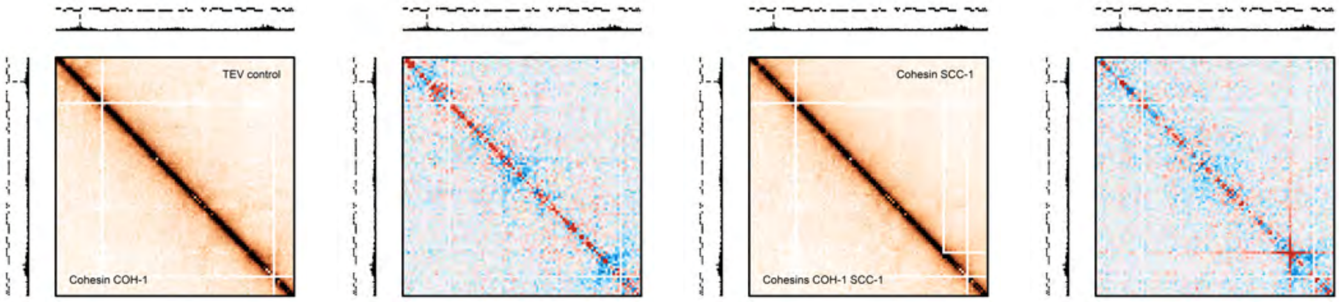

chrIII:3373001-3573000

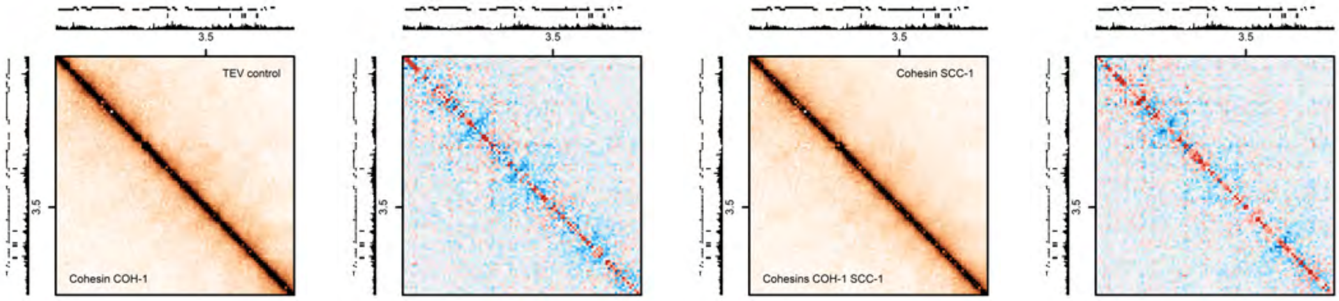

chrI:10077001-10277000

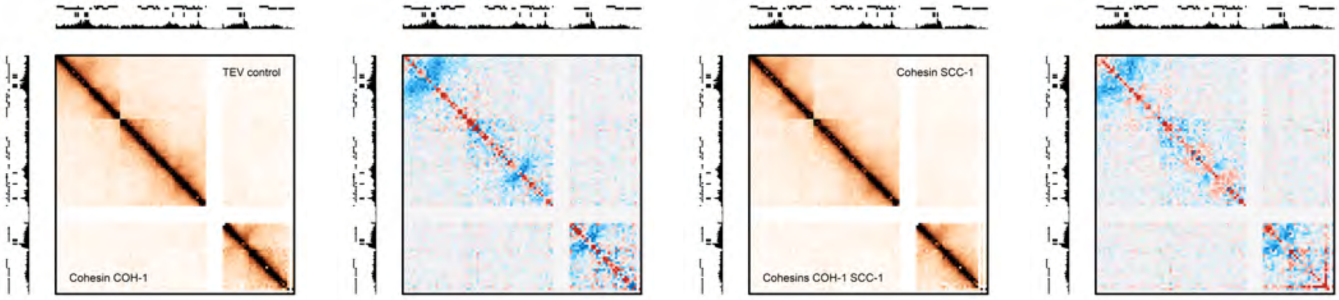

chrX:14049001-14249000

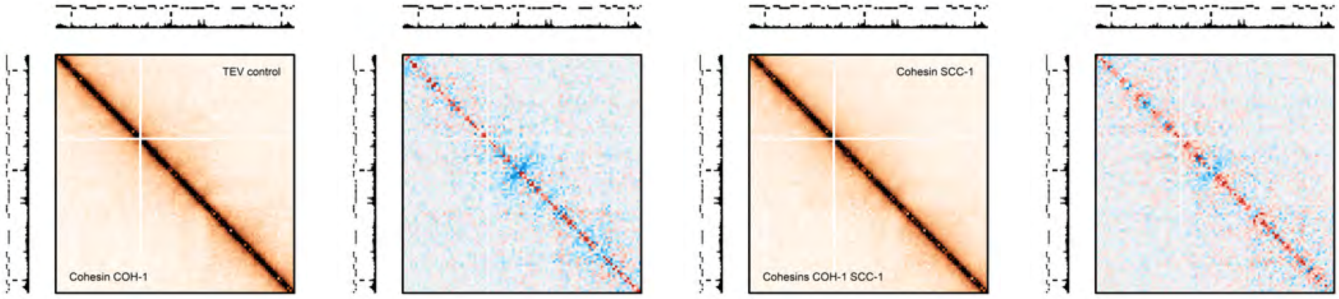

chrX:16633001-16833000

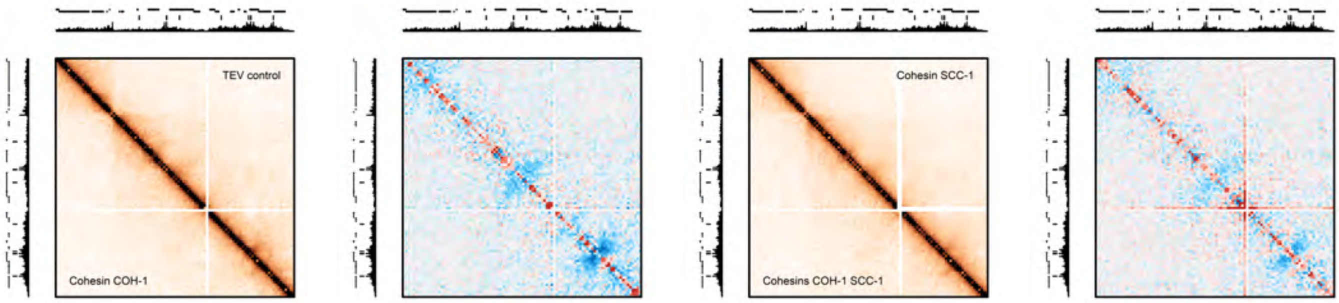

chrV:7115001-7315000

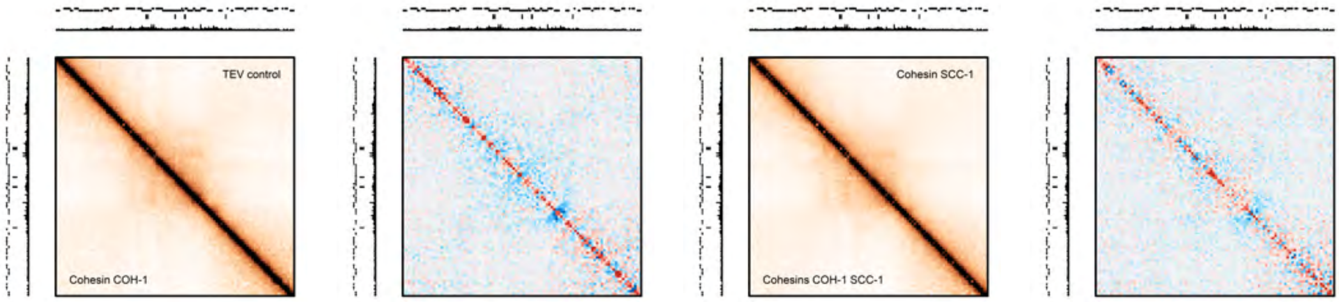

chrII:9631001-9831000

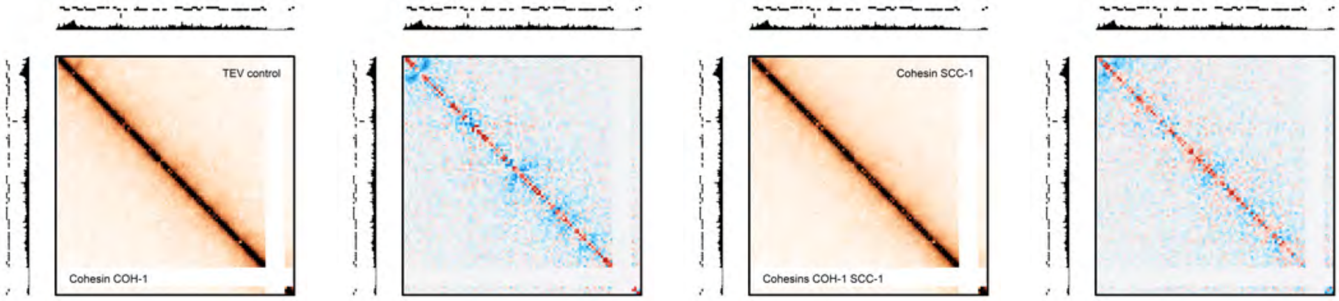

chrII:6789001-6989000

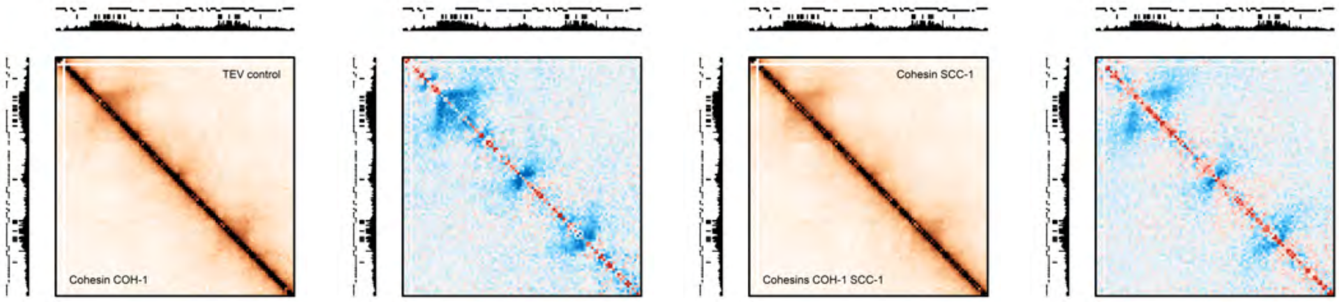

chrV:2805001-3005000

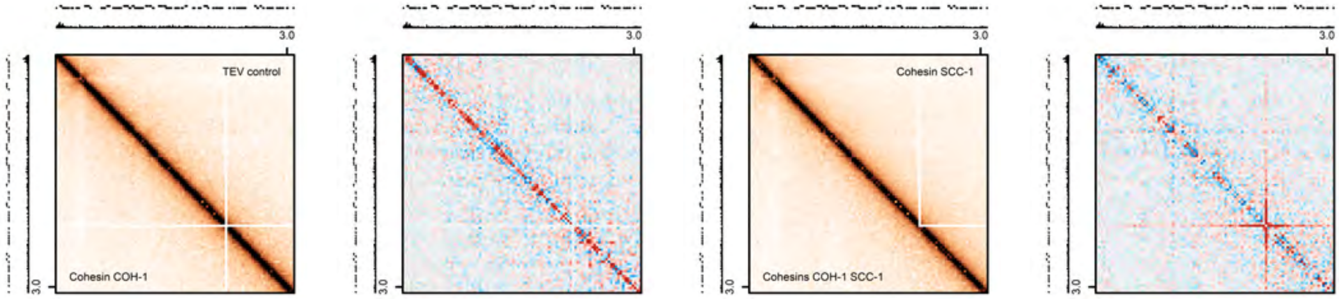

chrIII:5403001-5603000

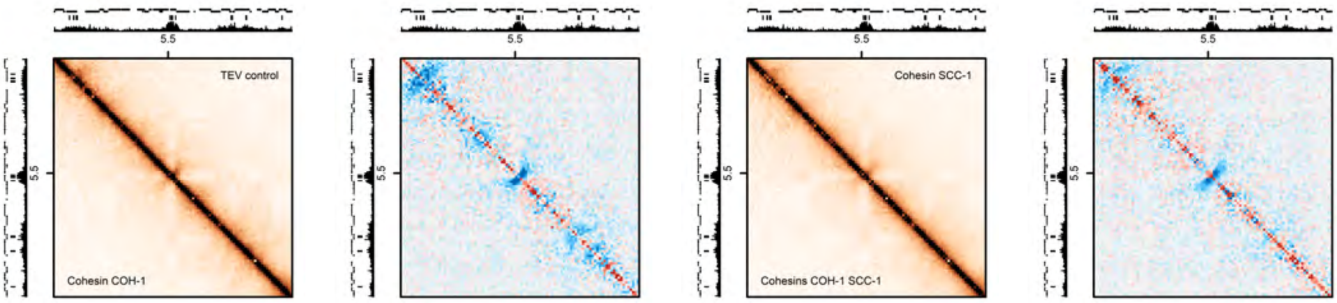

chrII:11875001-12075000

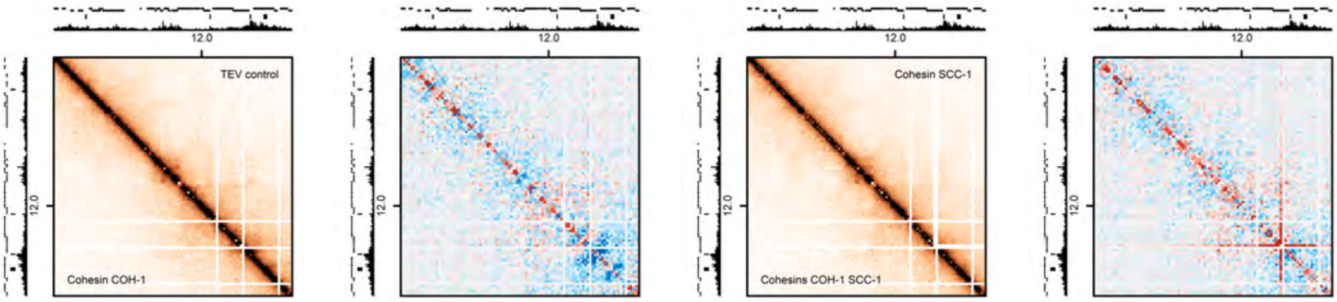

chrV:11111001-11311000

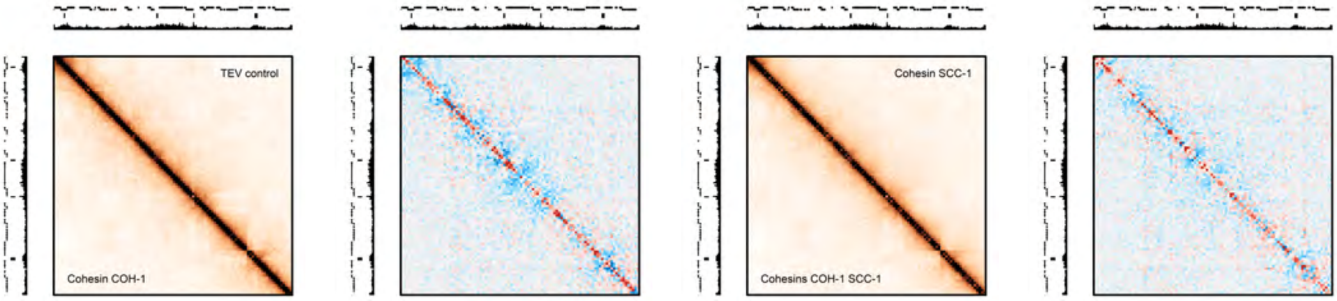

chrX:4801001-5001000

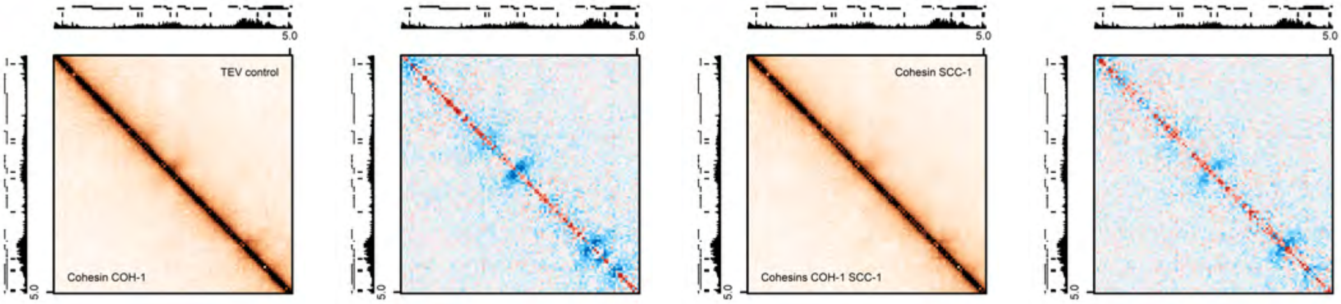

chrI:11353001-11553000

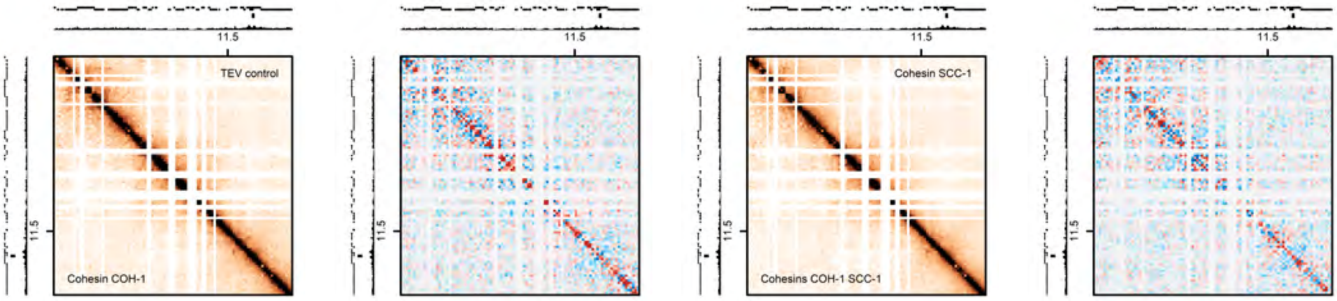

chrIII:5271001-5471000

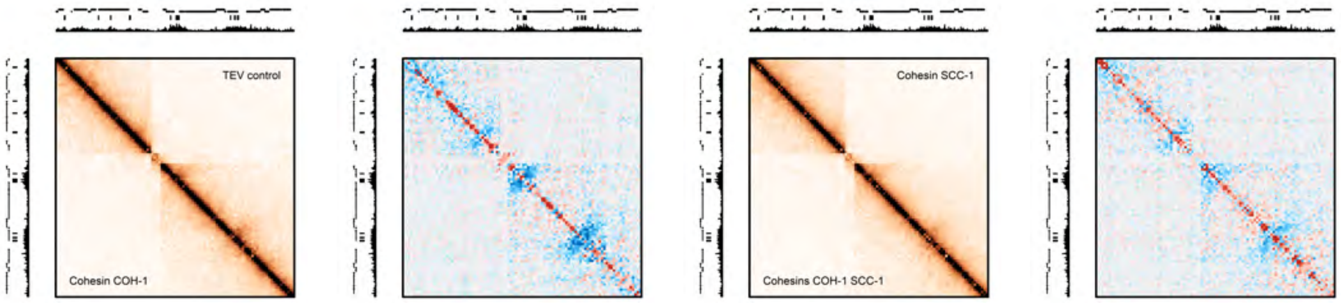

chrV:5667001-5867000

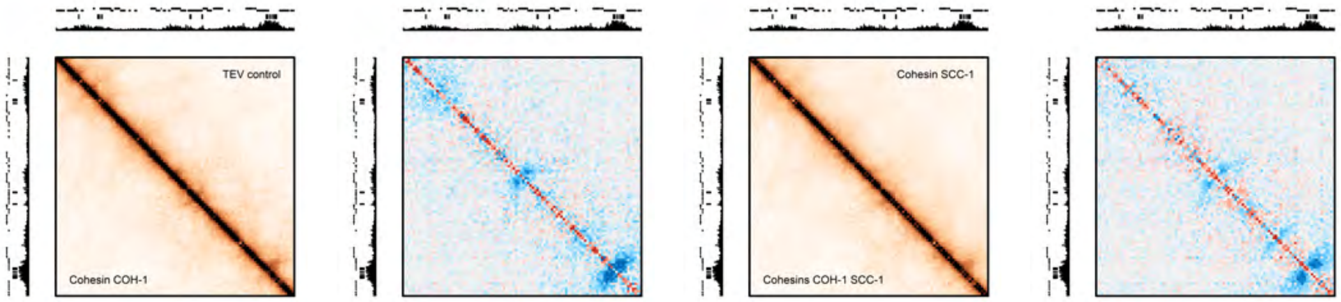

chrV:16001001-16201000

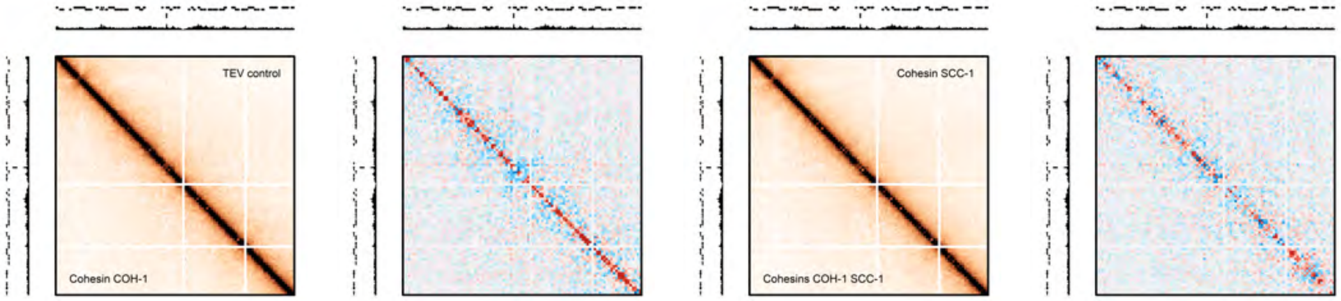

chrV:18393001-18593000

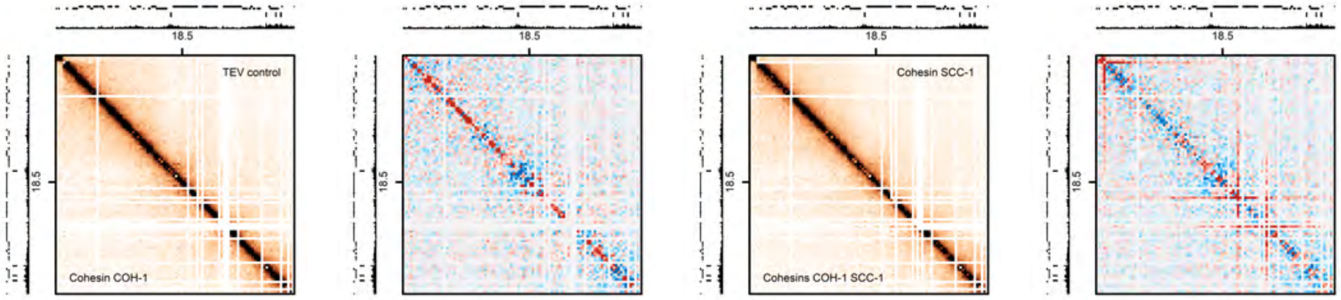

chrX:15745001-15945000

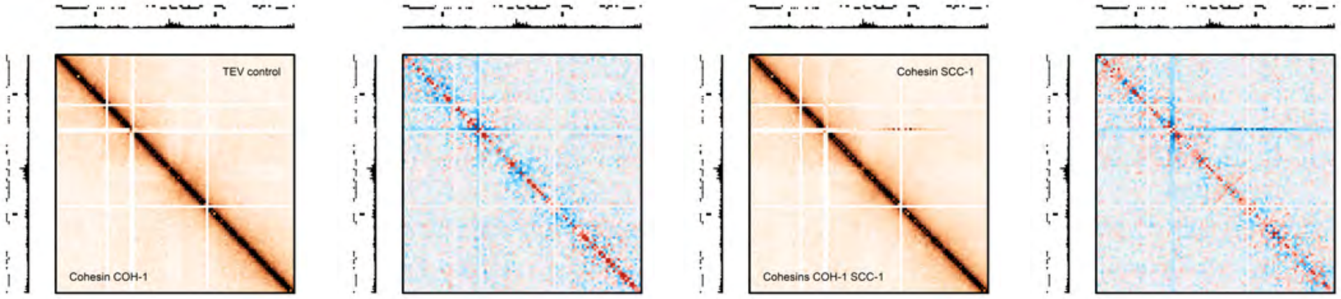

chrII:10349001-10549000

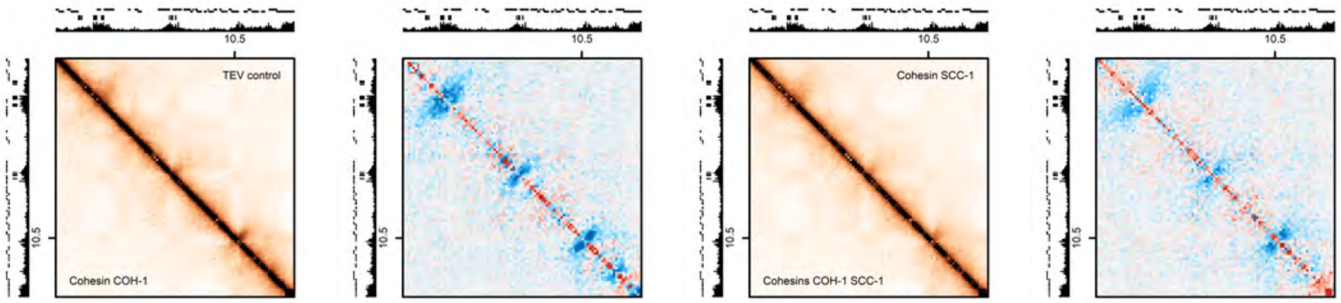

chrIII:1721001-1921000

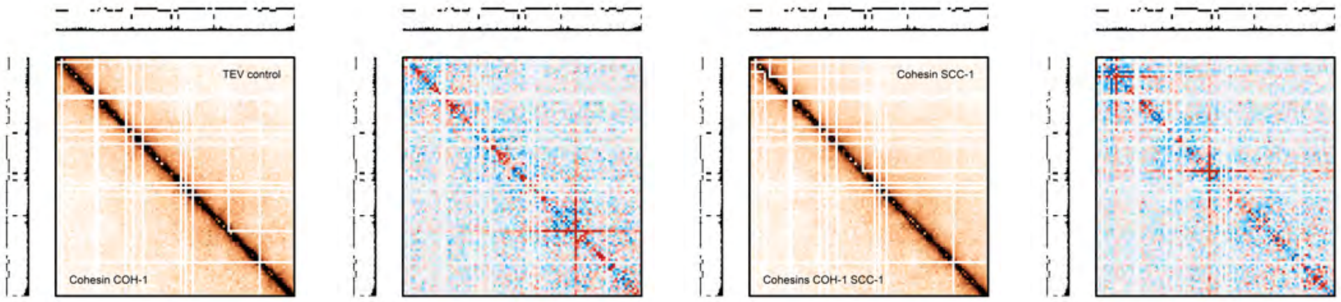

chrI:5035001-5235000

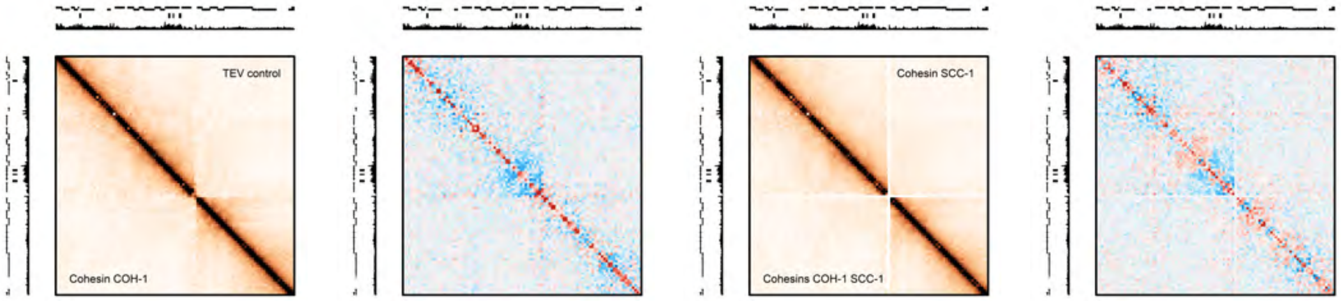

chrII:783001-983000

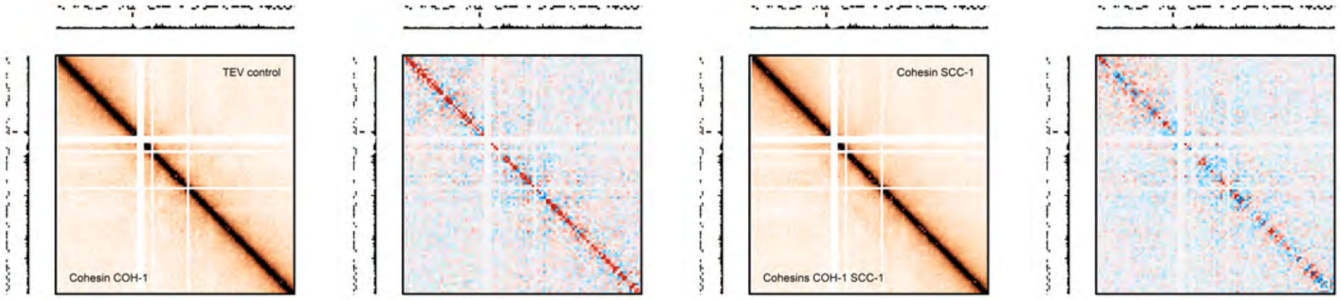

chrIII:3521001-3721000

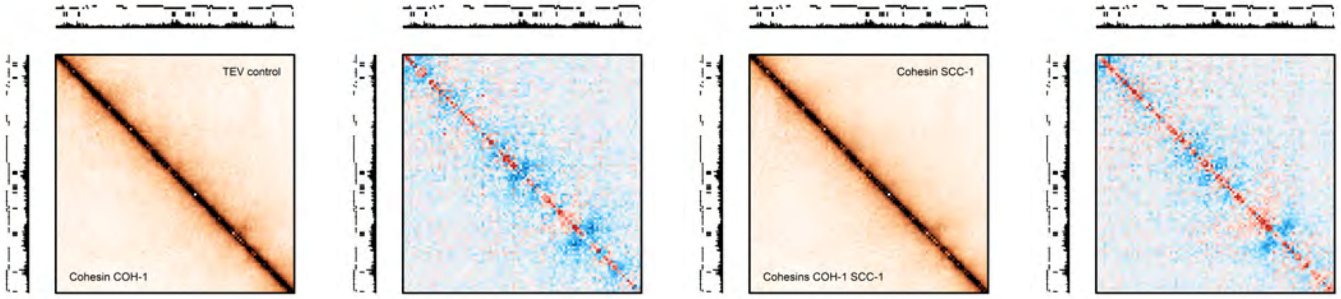

chrI:13447001-13647000

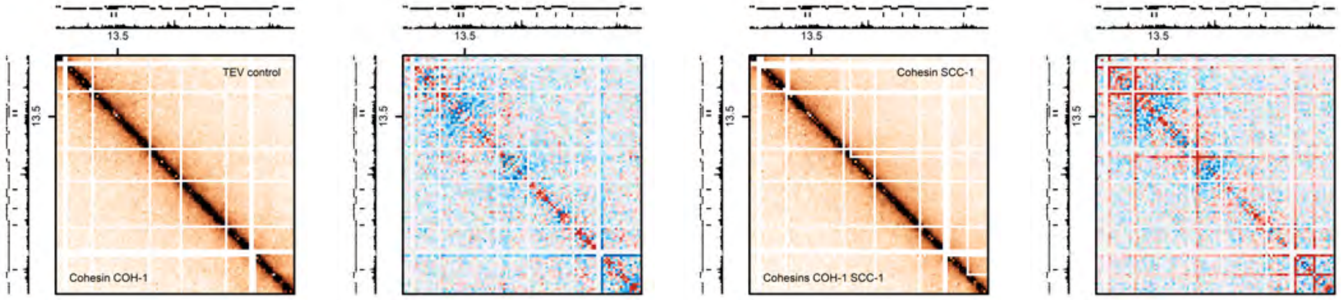

chrIII:7197001-7397000

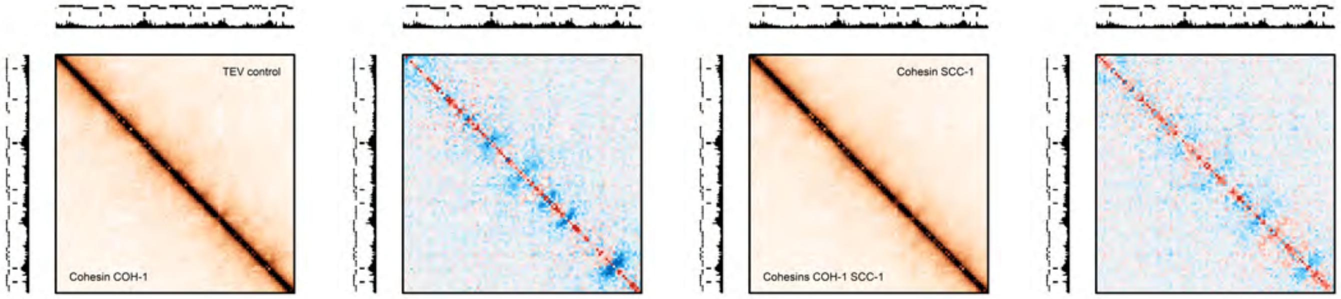

chrI:8883001-9083000

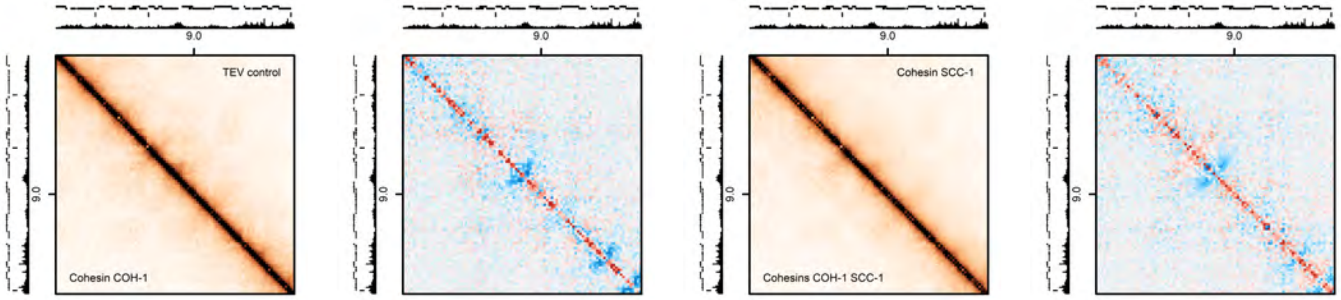

chrIV:6403001-6603000

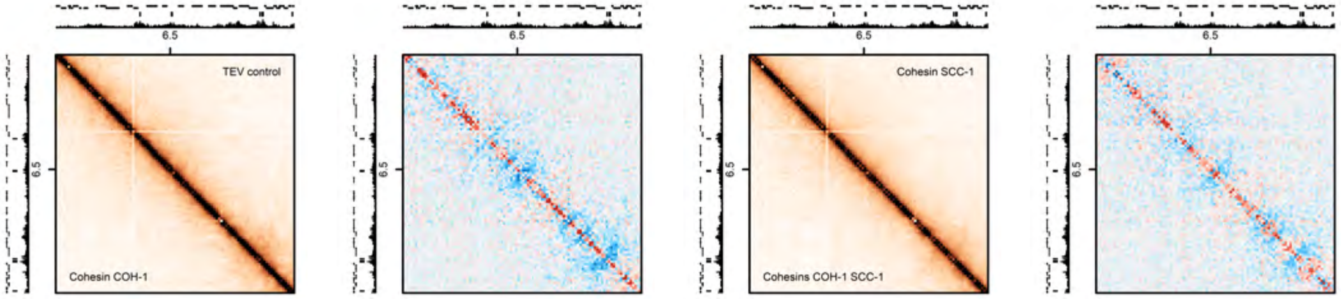

chrIII:1431001-1631000

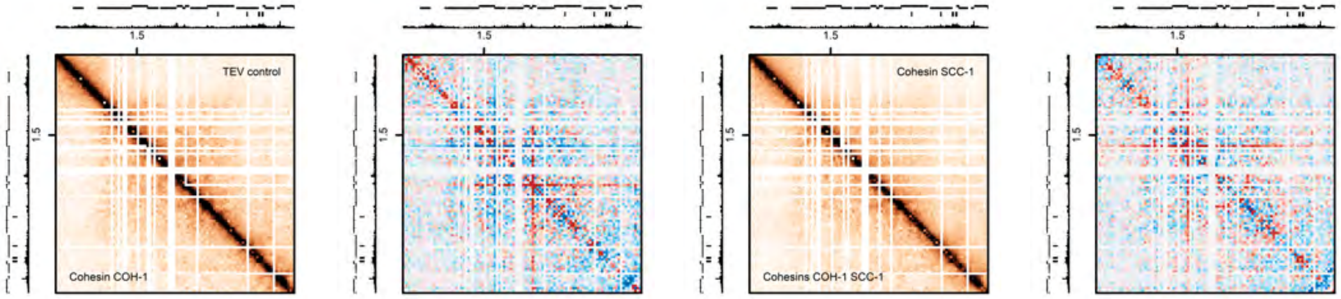

chrV:1135001-1335000

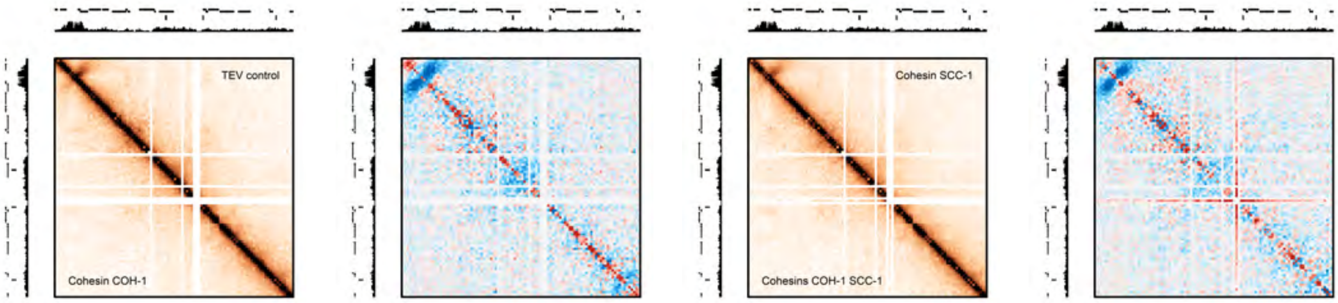

chrIV:6117001-6317000

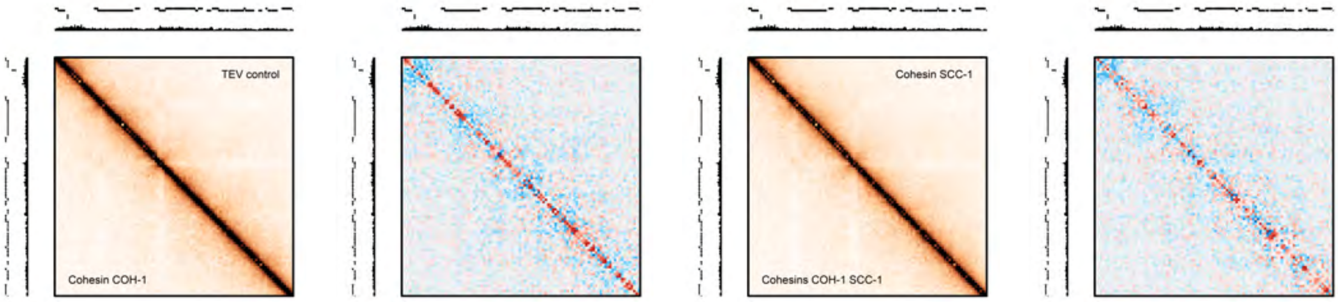

chrIV:2789001-2989000

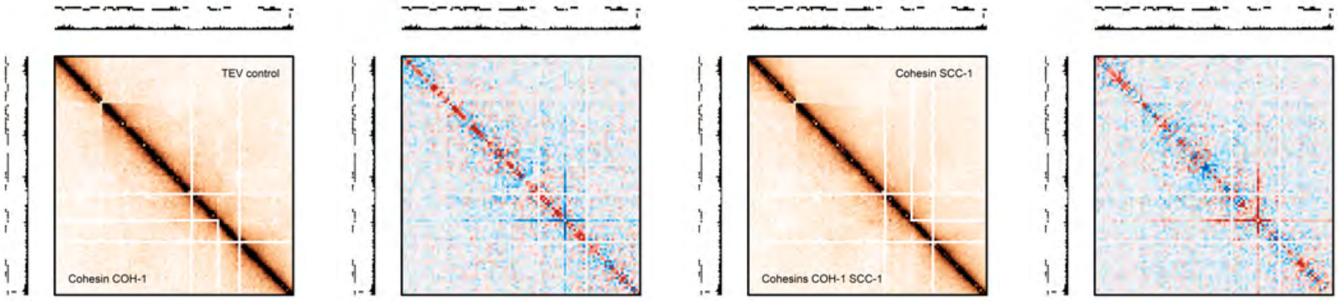

chrX:3581001-3781000

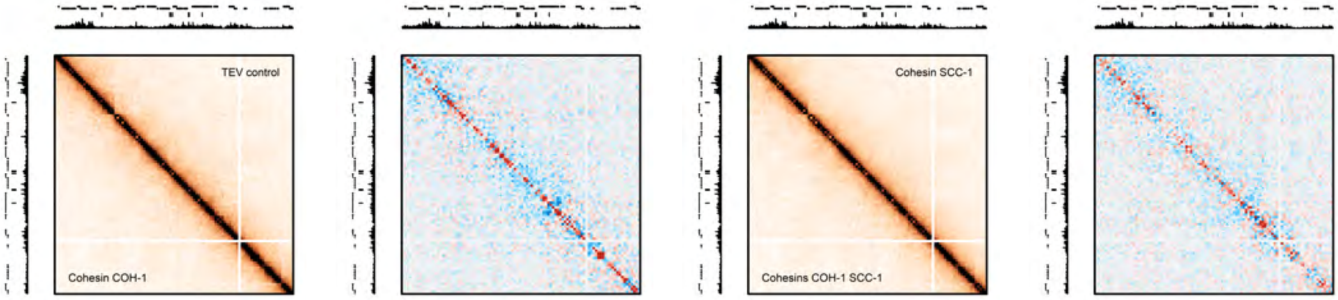

chrIV:9415001-9615000

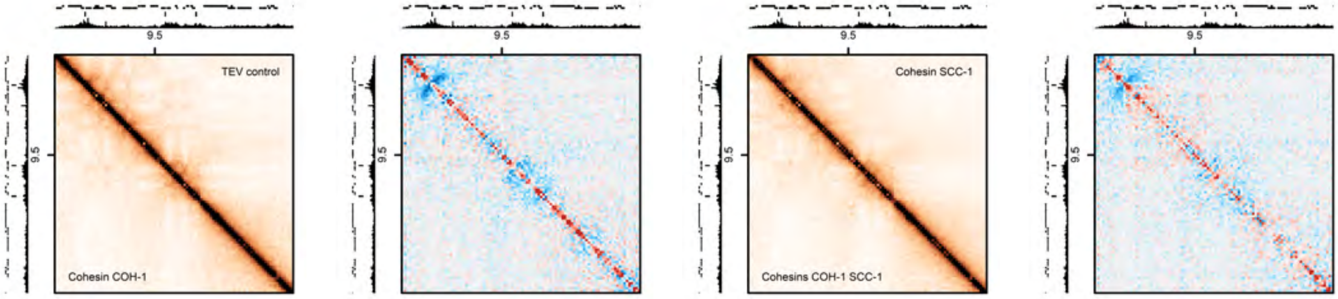

chrII:10981001-11181000

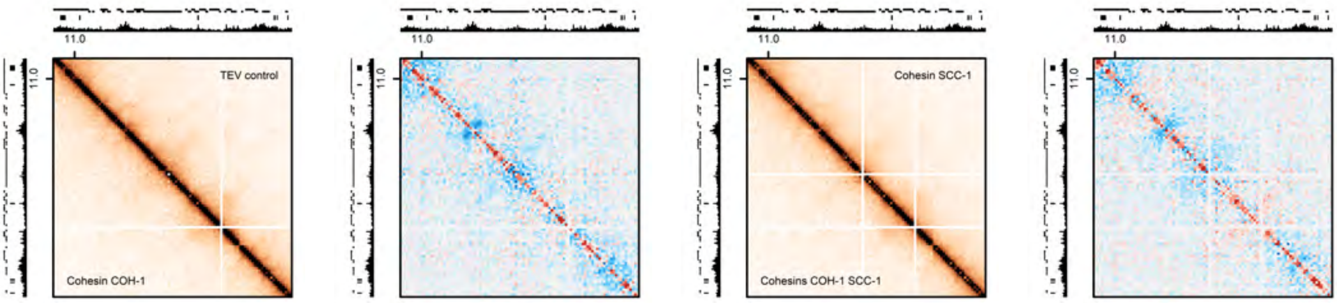

chrX:4527001-4727000

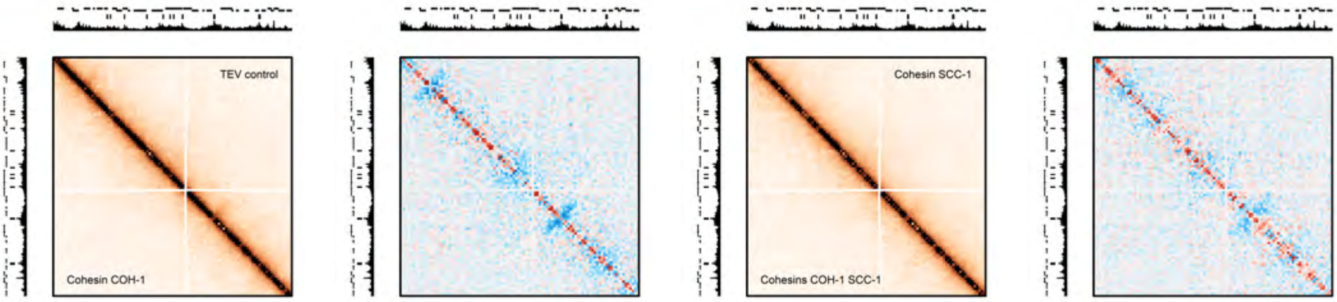

chrI:1247001-1447000

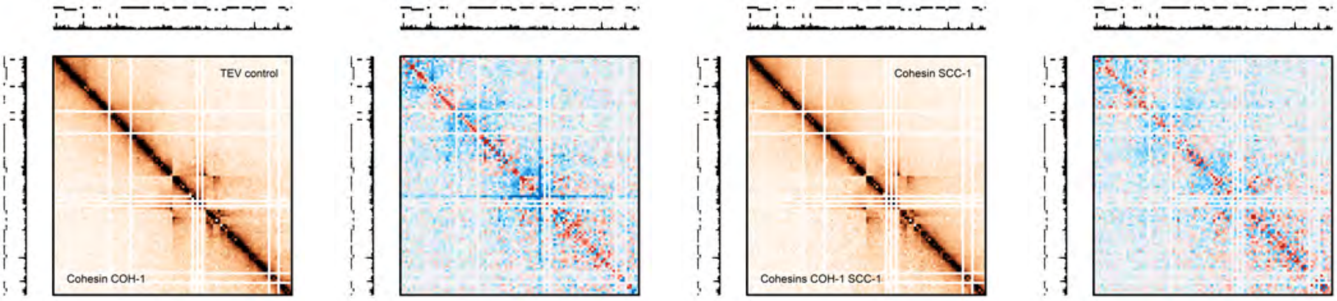

chrV:13477001-13677000

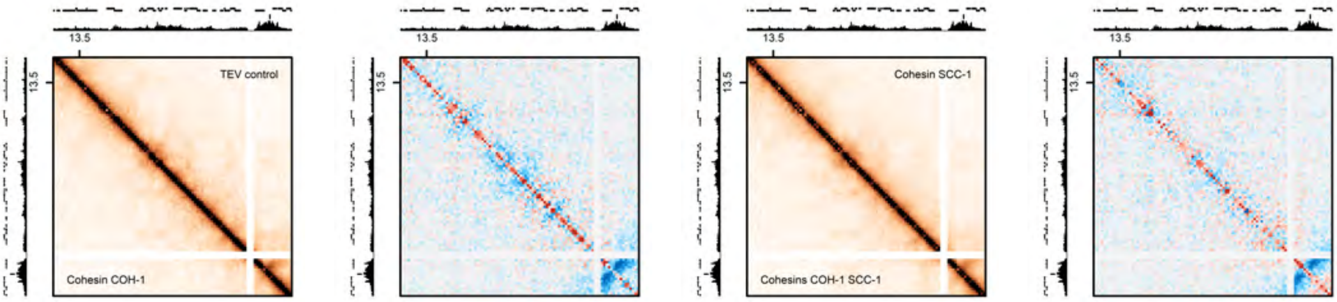

chrX:13427001-13627000

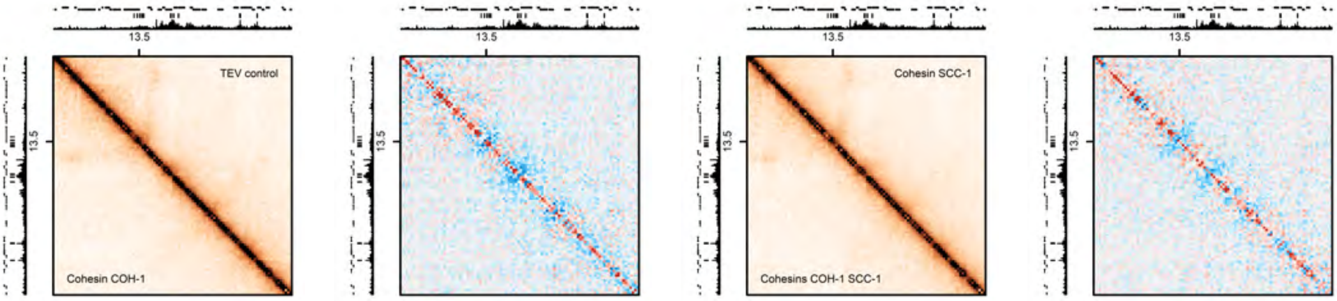

chrX:4285001-4485000

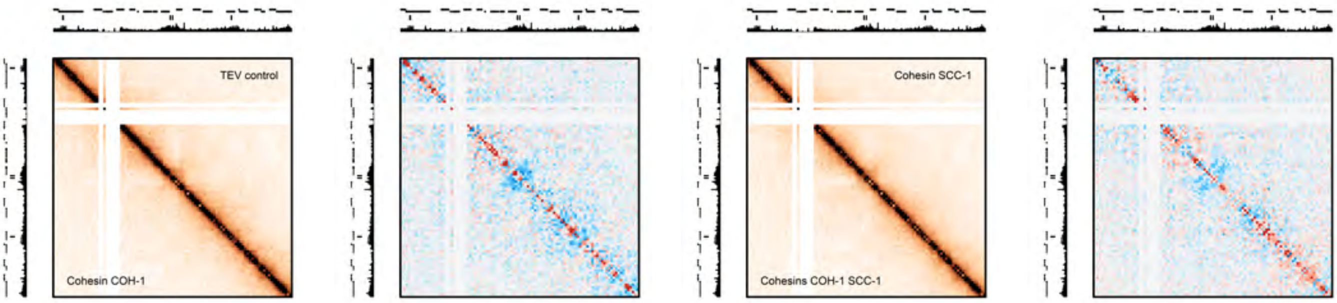

chrX:2333001-2533000

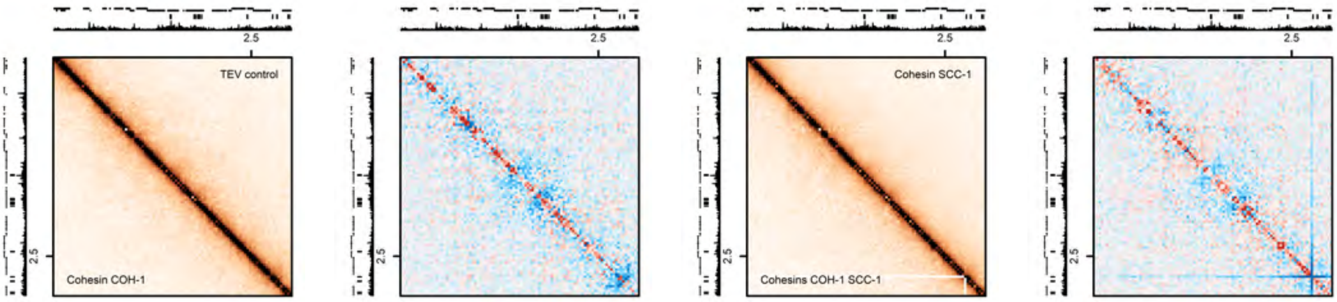

chrII:12967001-13167000

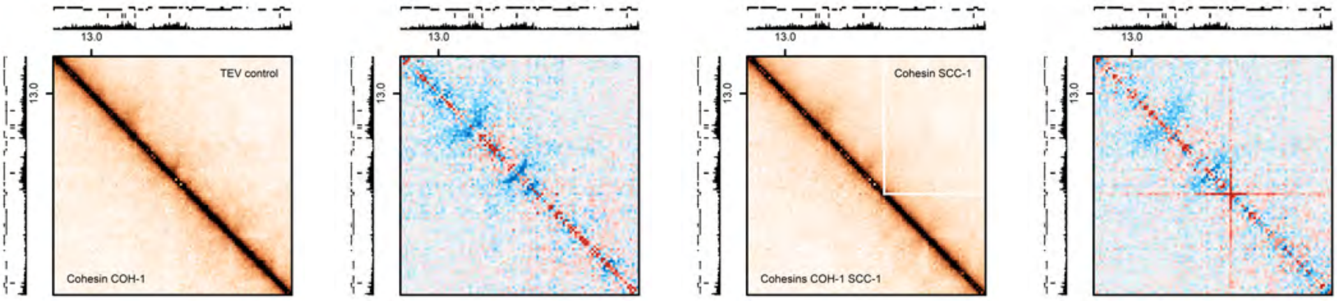

chrIV:14695001-14895000

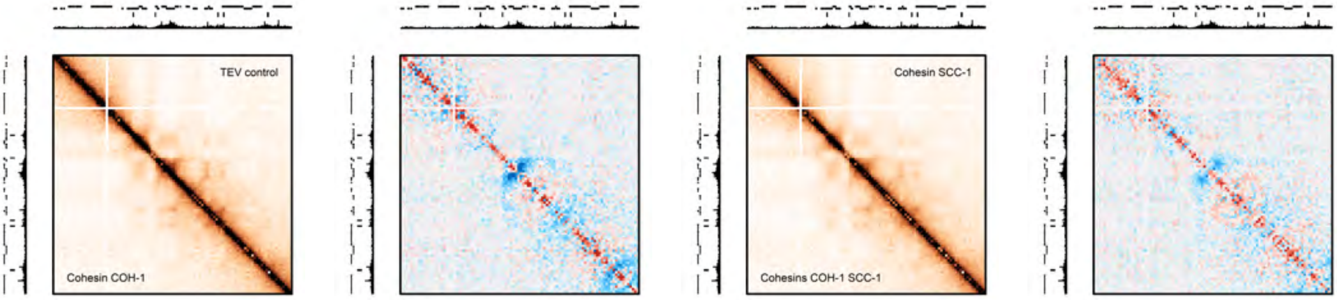

chrX:7359001-7559000

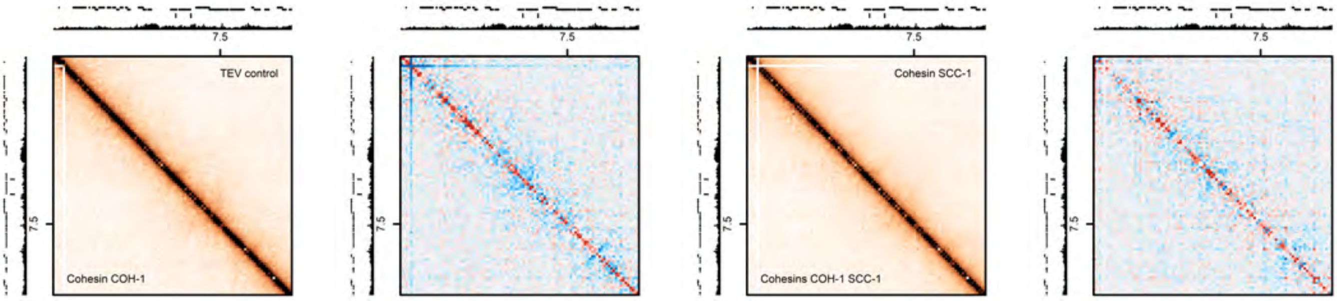

chrIV:15465001-15665000

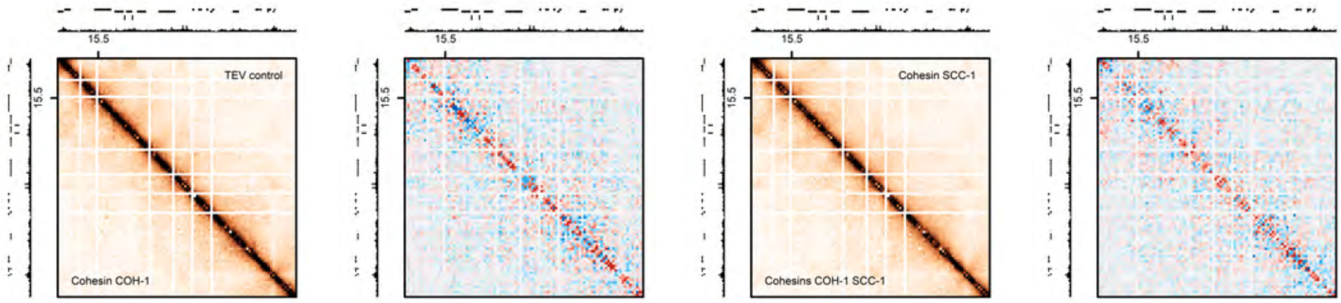

chrX:723001-923000

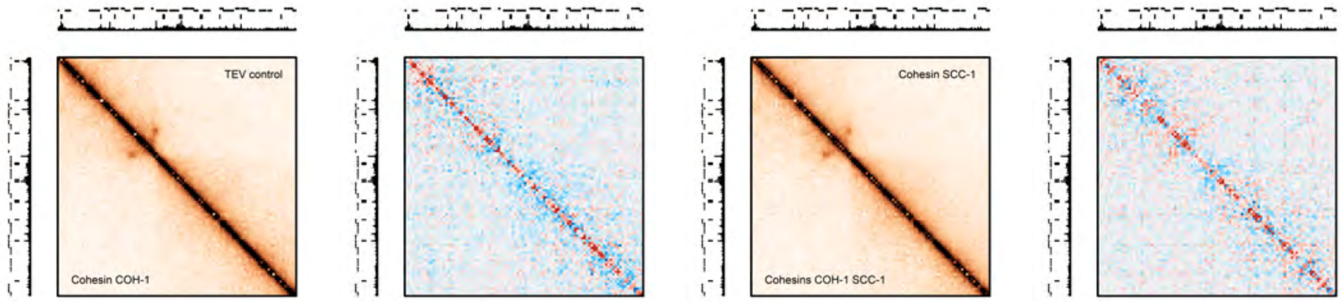

chrIV:9897001-10097000

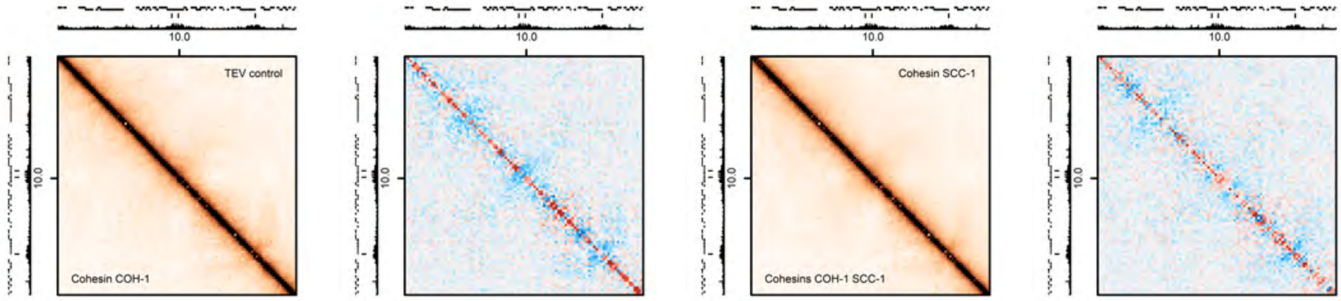

chrV:2515001-2715000

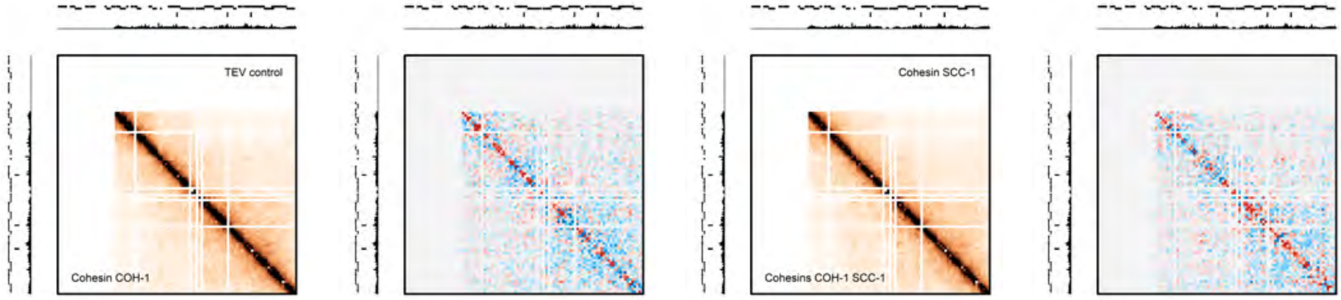

chrII:7293001-7493000

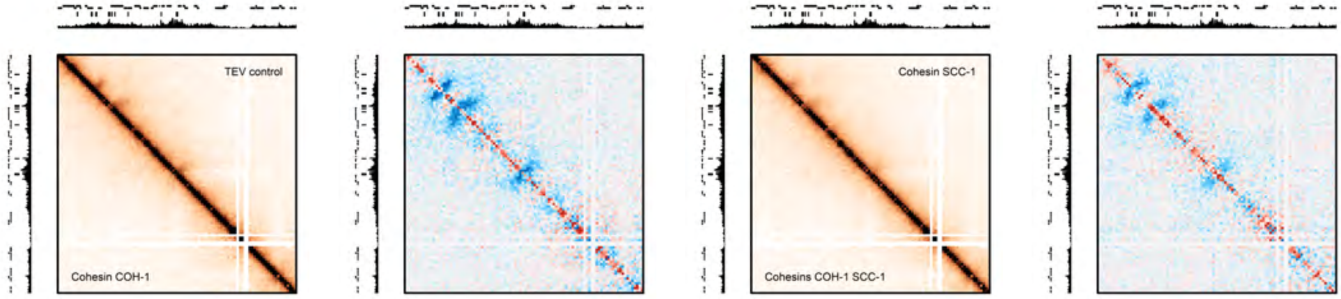

chrIV:7219001-7419000

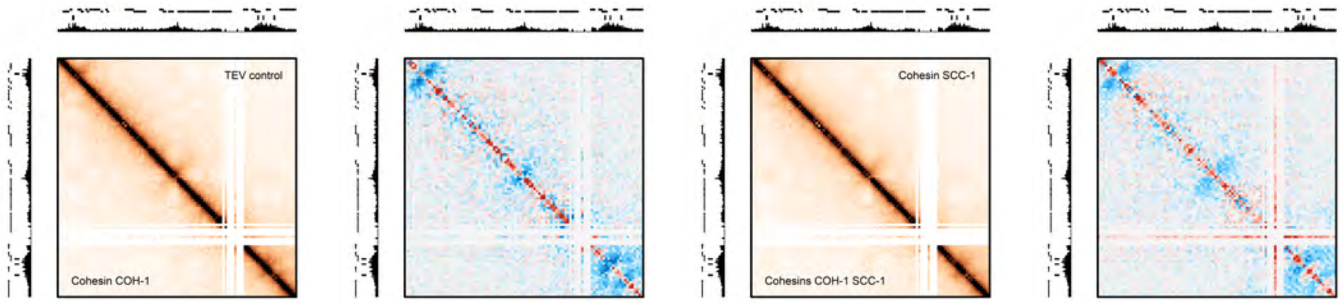

chrV:14521001-14721000

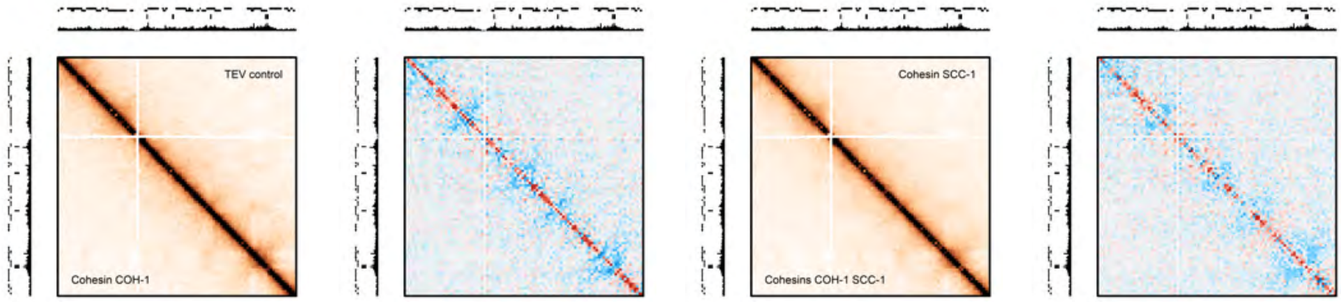

chrIII:13665001-13865000

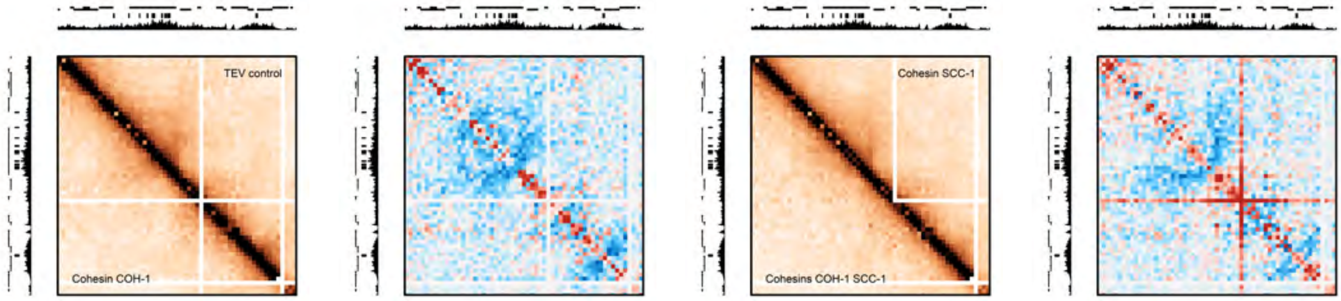

chrI:9843001-10043000

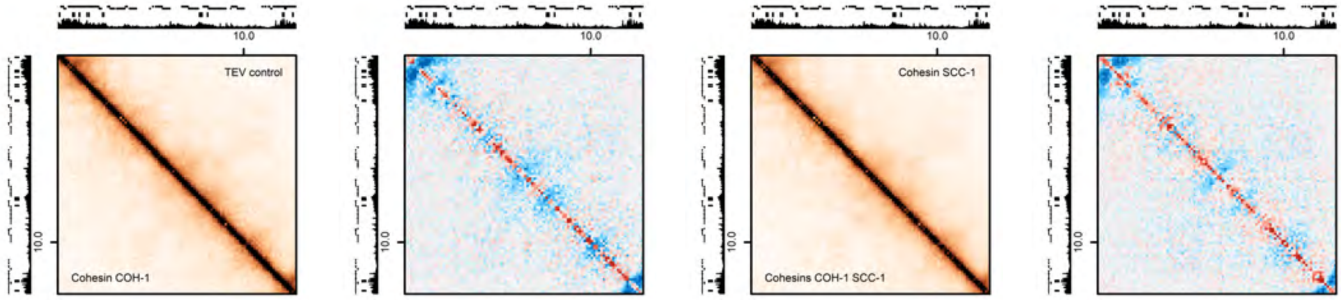

chrX:3799001-3999000

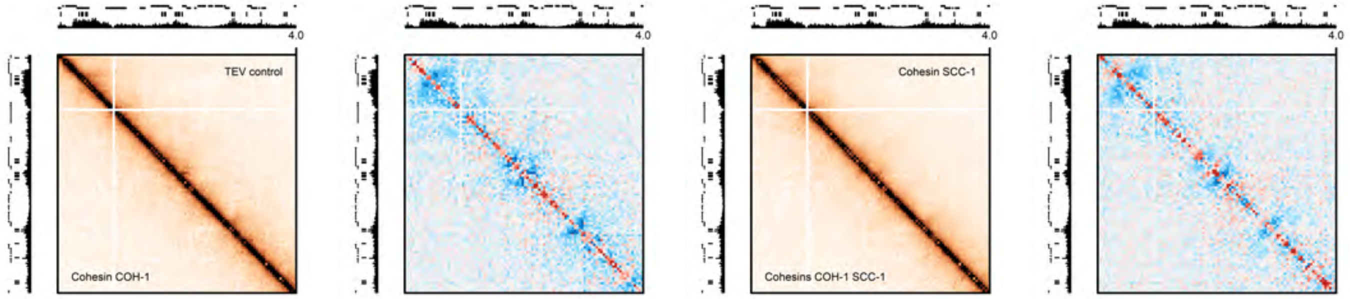

chrV:15781001-15981000

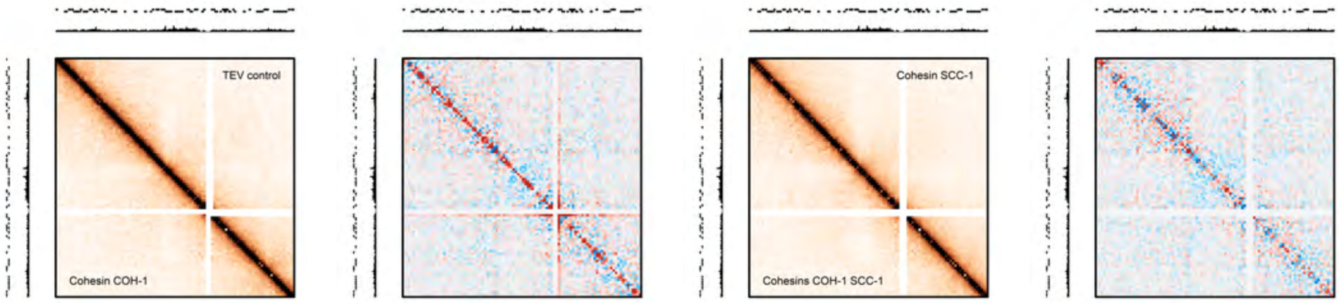

chrV:4257001-4457000

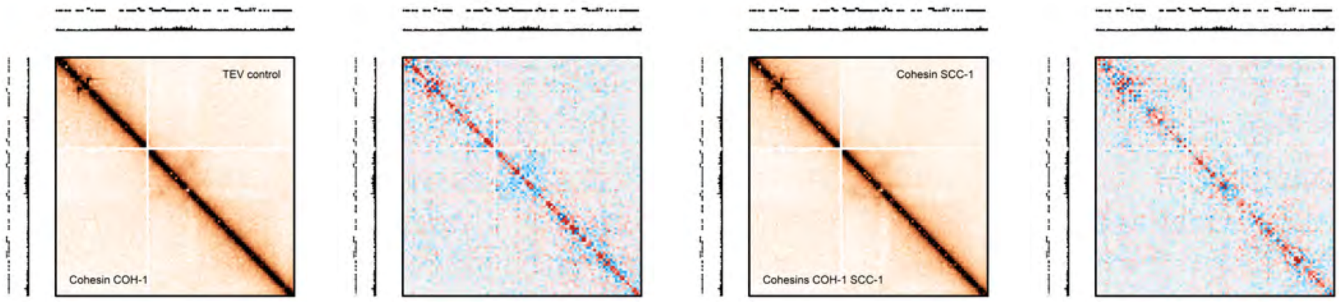

chrV:5307001-5507000

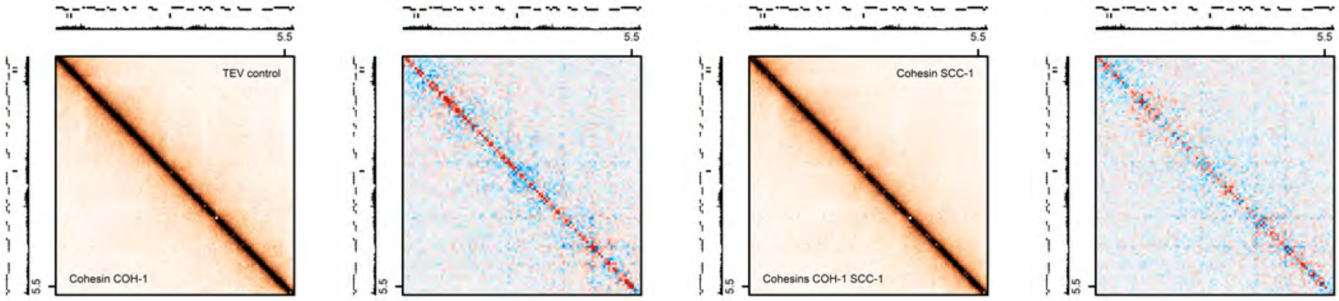

chrIII:2213001-2413000

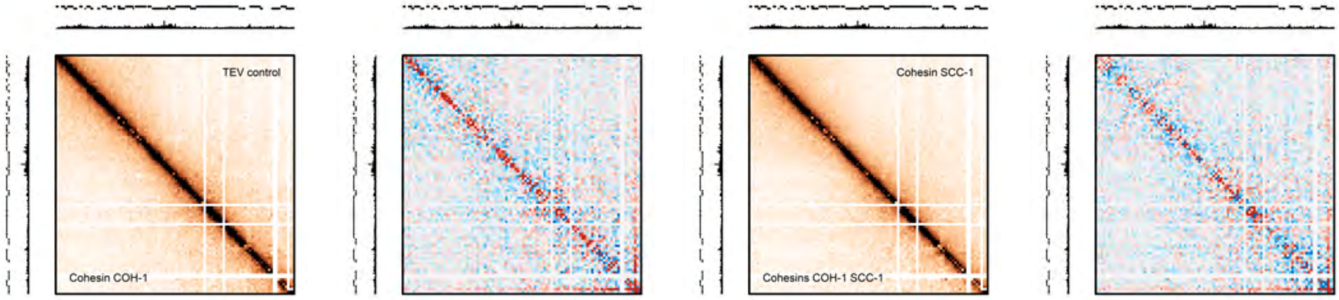

chrV:2701001-2901000

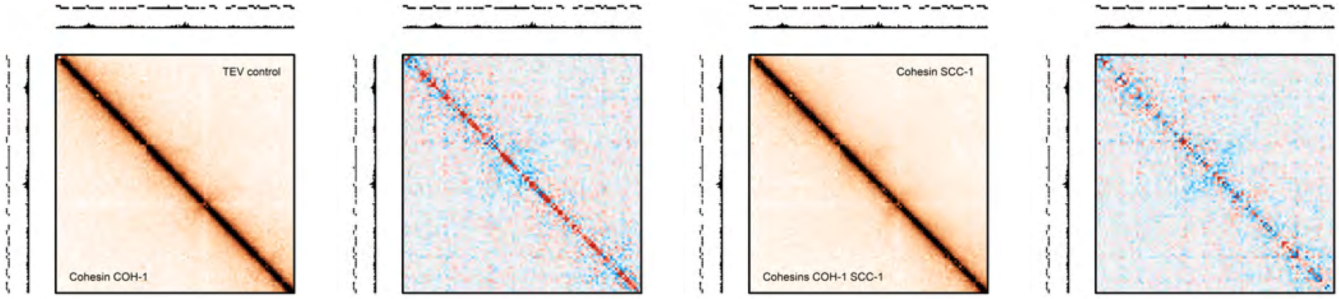

chrIII:13249001-13449000

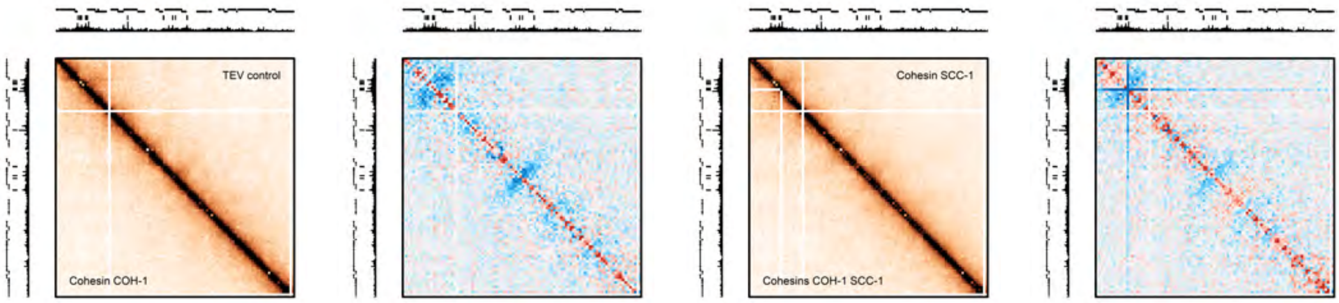

chrI:12637001-12837000

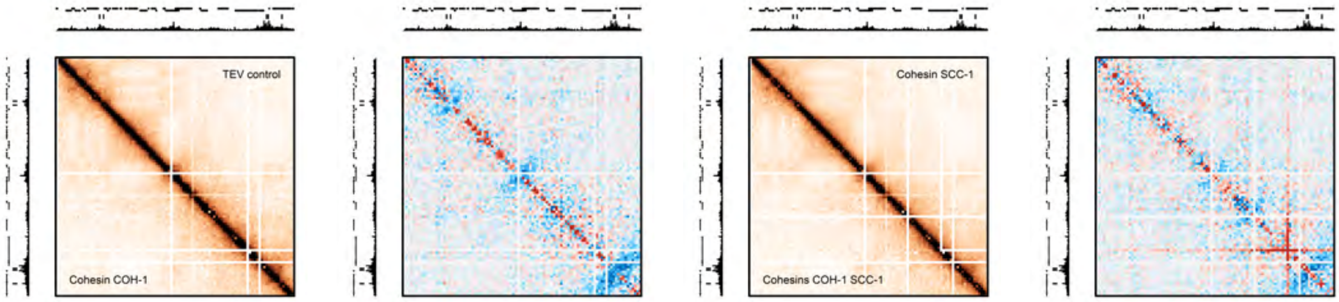

chrIV:7969001-8169000

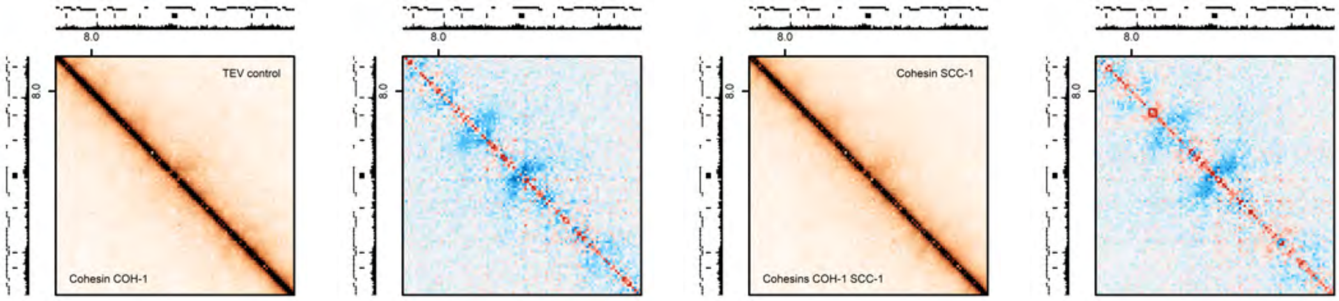

chrV:9231001-9431000

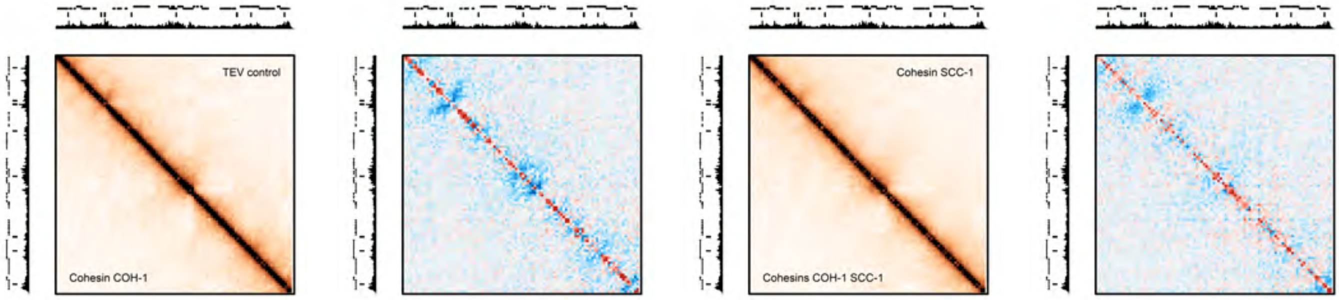

chrIII:327001-527000

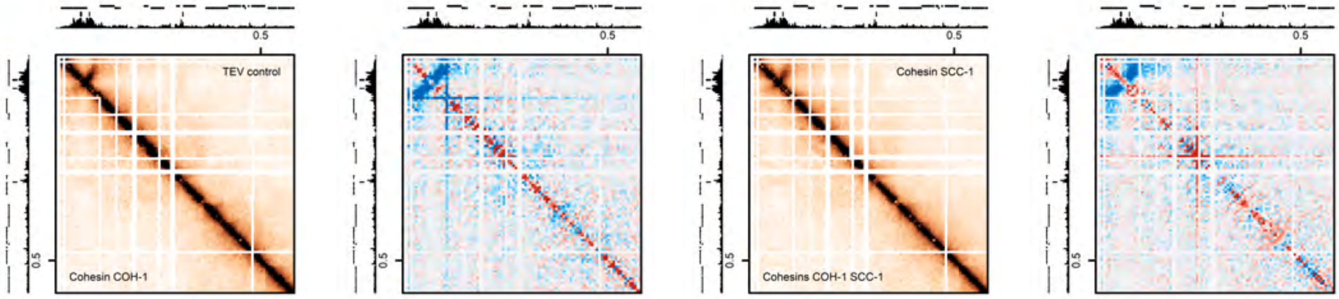

chrI:12579001-12779000

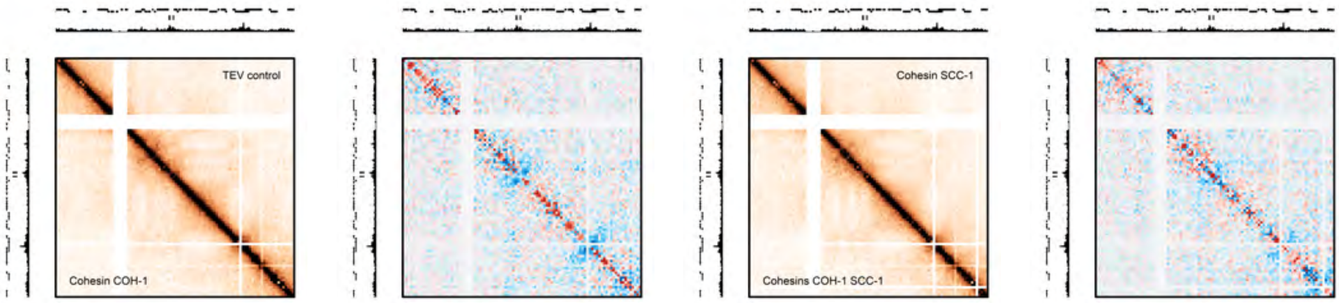

chrIII:12317001-12517000

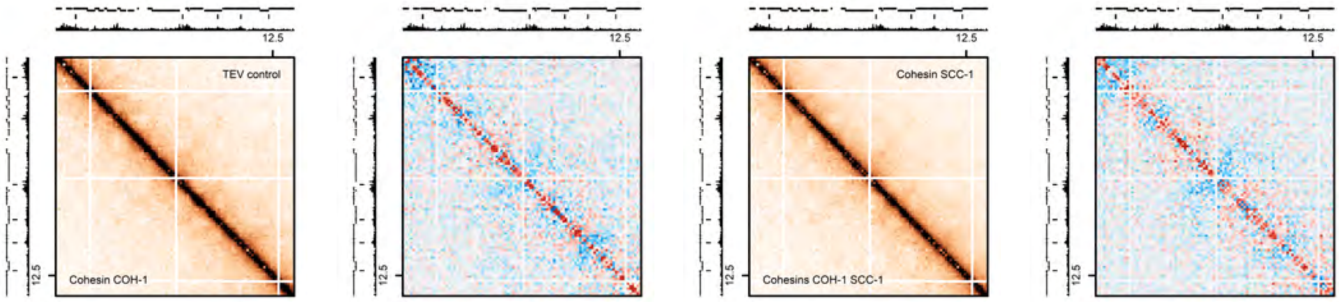

chrV:15231001-15431000

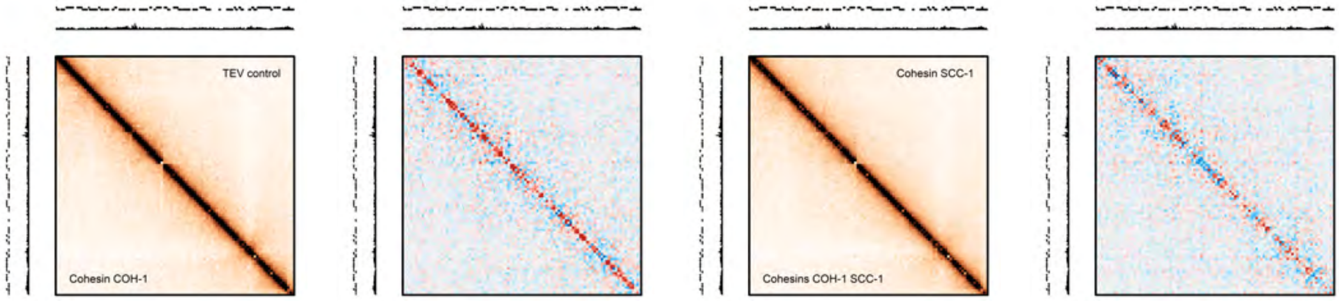

chrII:1043001-1243000

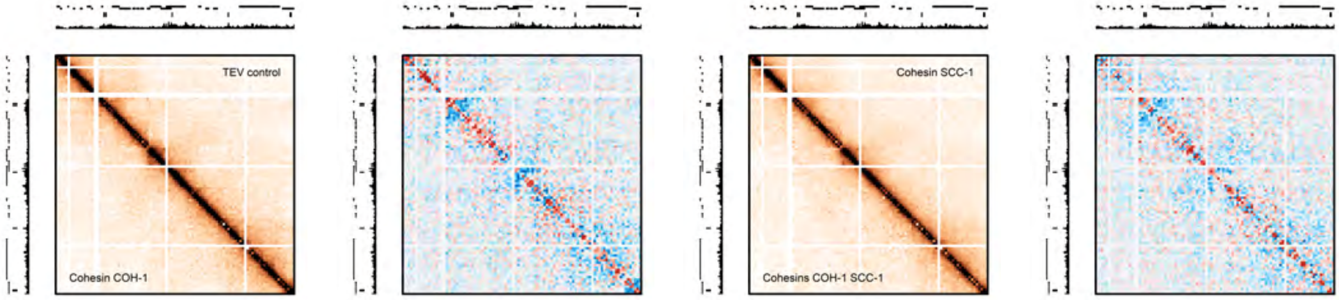

chrX:10227001-10427000

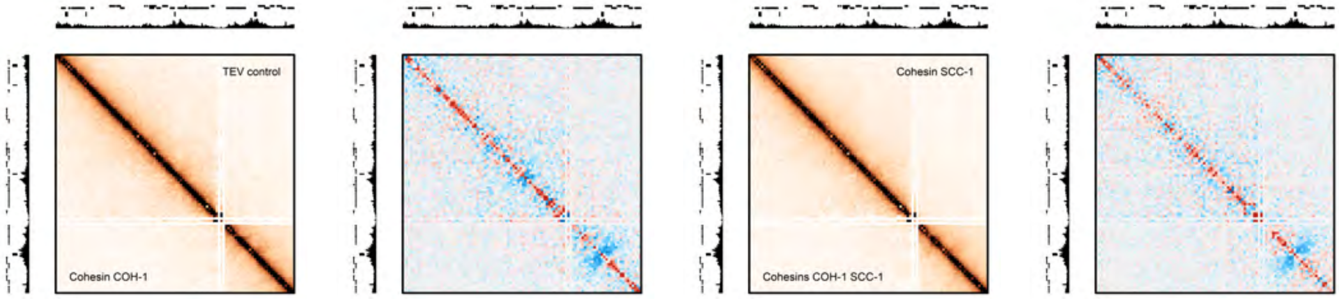

chrV:18953001-19153000

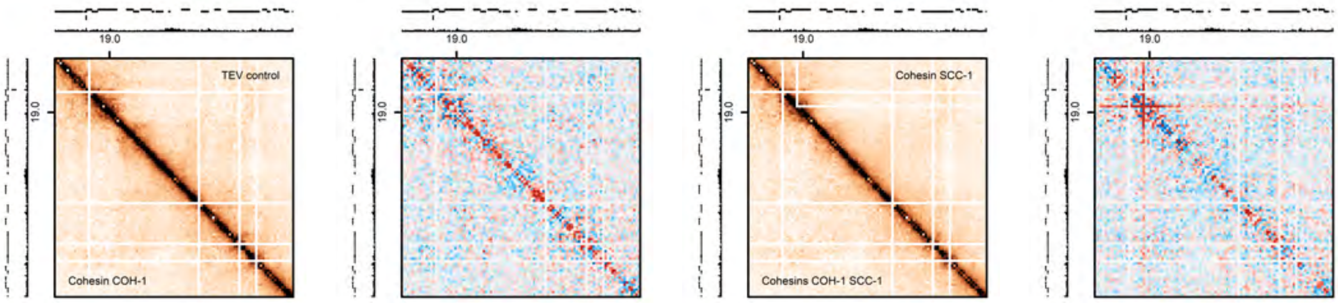

chrV:1479001-1679000

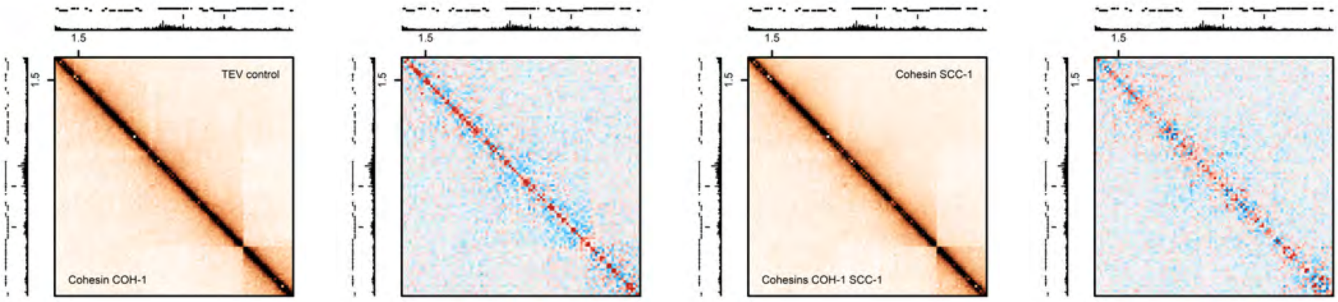

chrIV:11251001-11451000

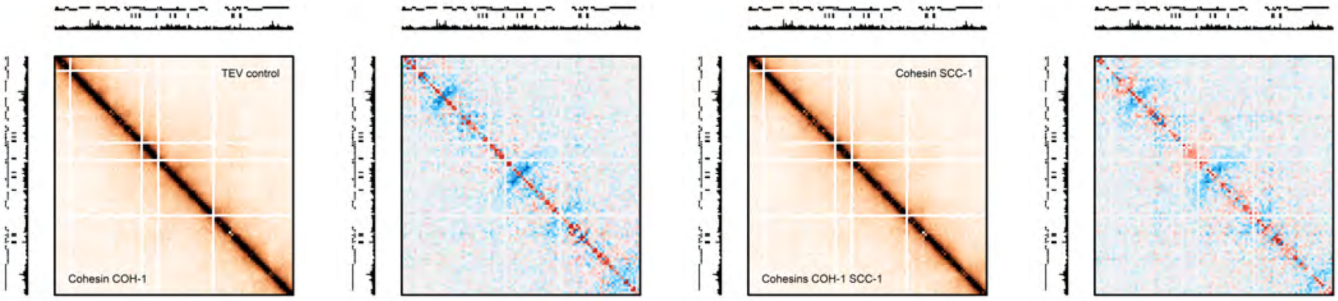

chrII:14203001-14403000

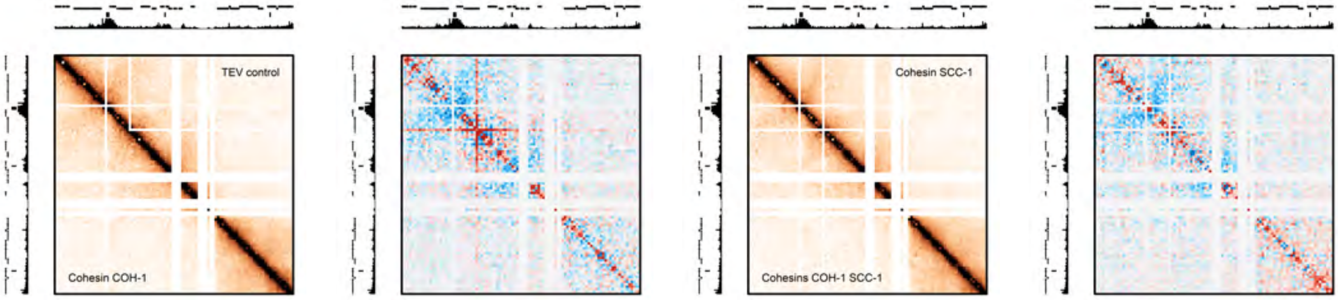

chrIV:15173001-15373000

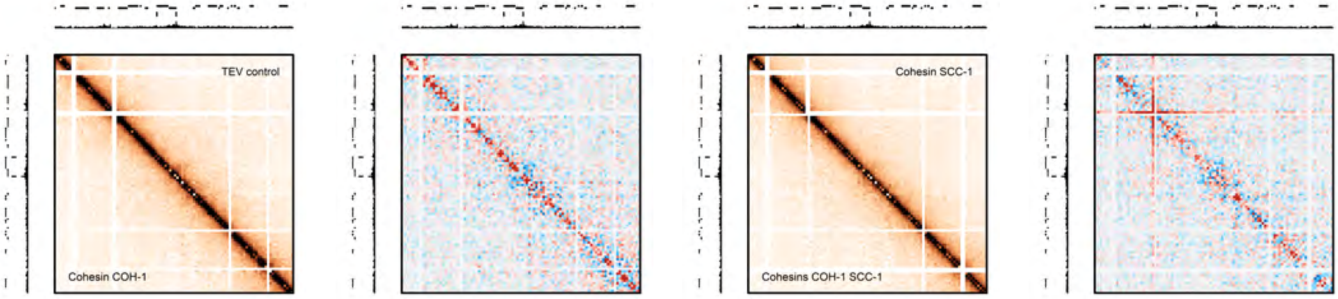

chrII:11445001-11645000

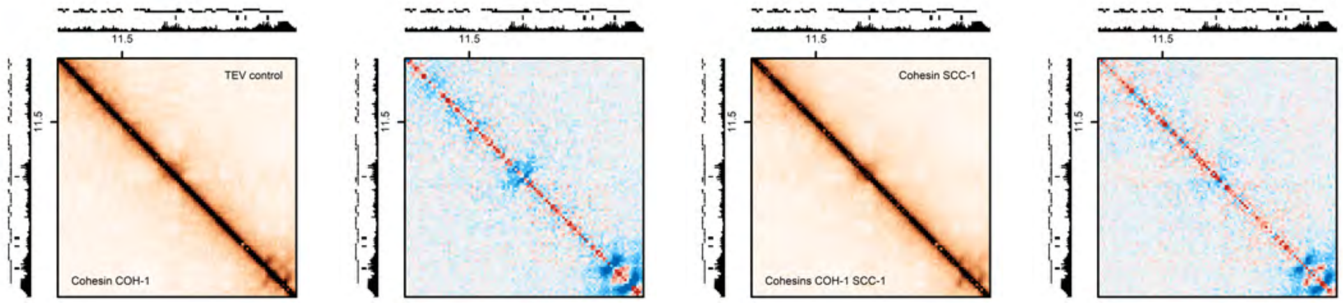

chrV:251001-451000

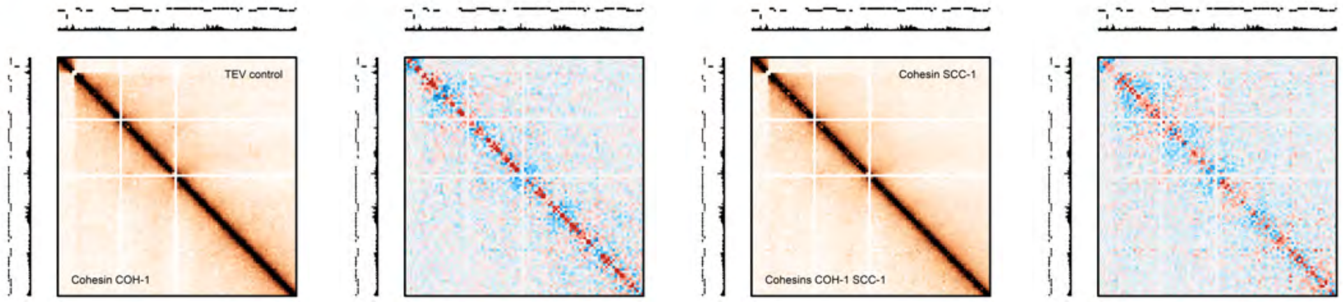

chrIV:9261001-9461000

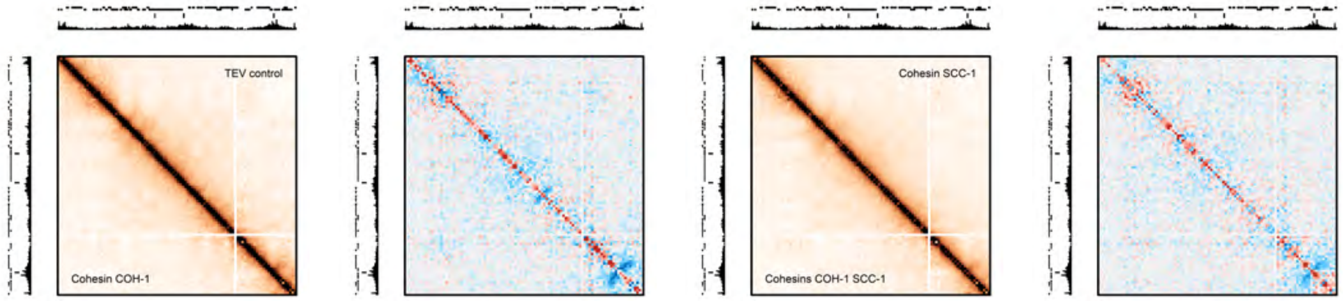

chrX:2115001-2315000

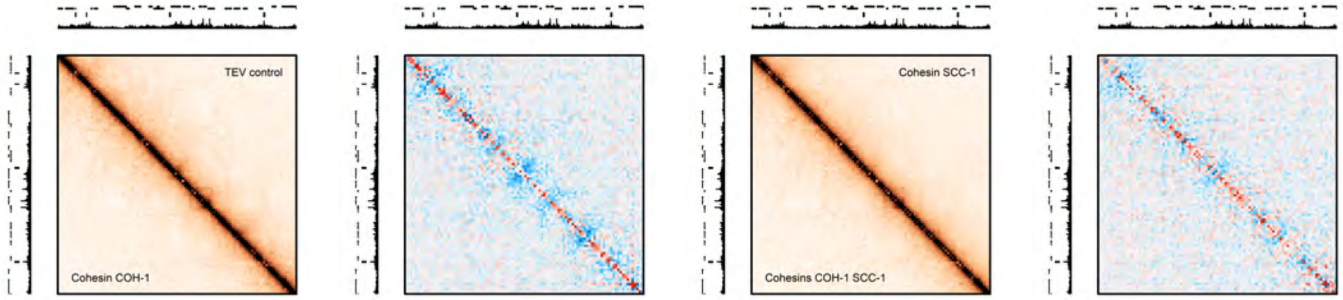

chrIV:2561001-2761000

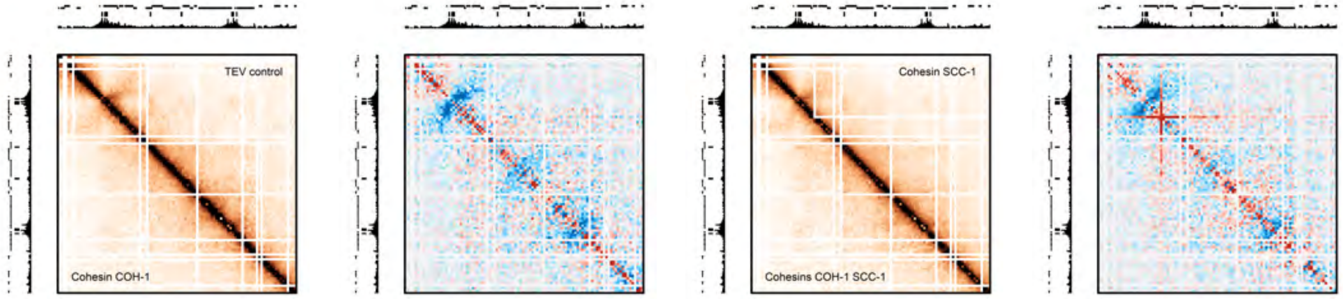

chrI:297001-497000

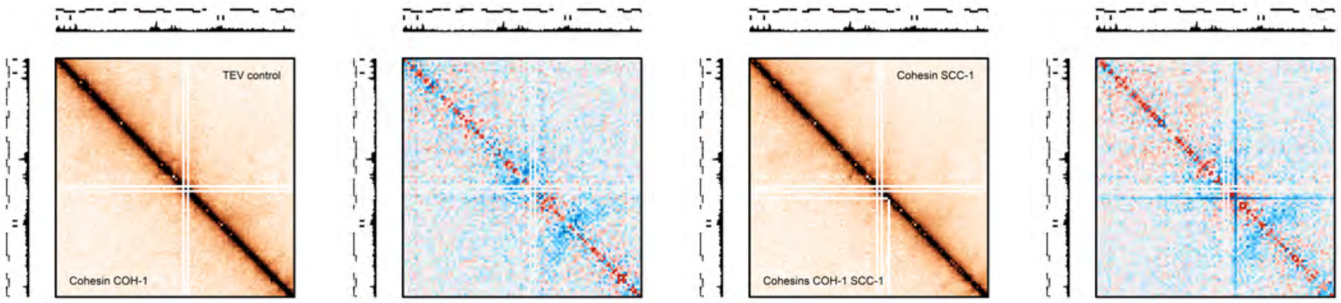

chrV:20021001-20221000

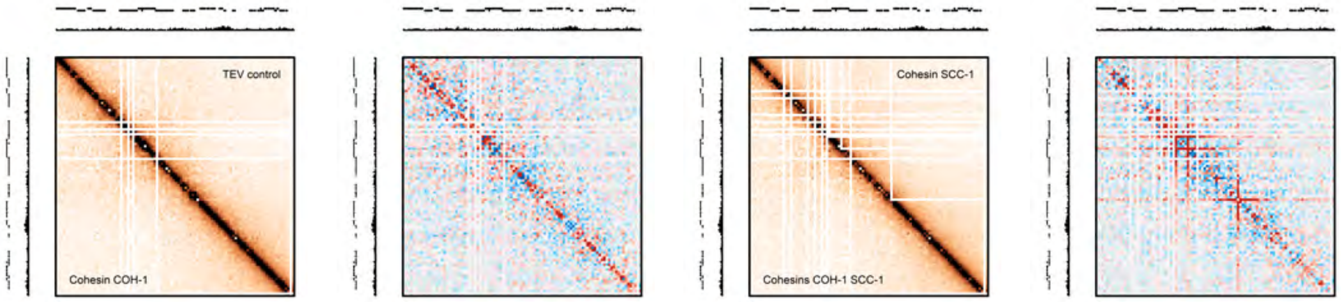

chrIII:10425001-10625000

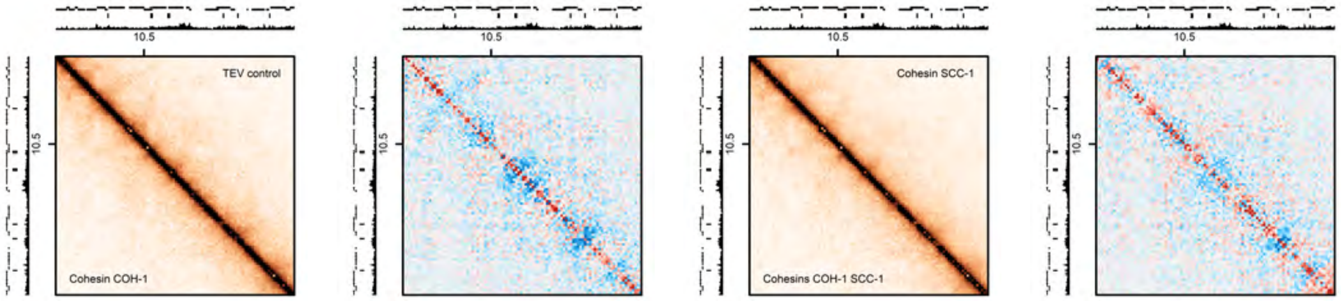

chrIV:12031001-12231000

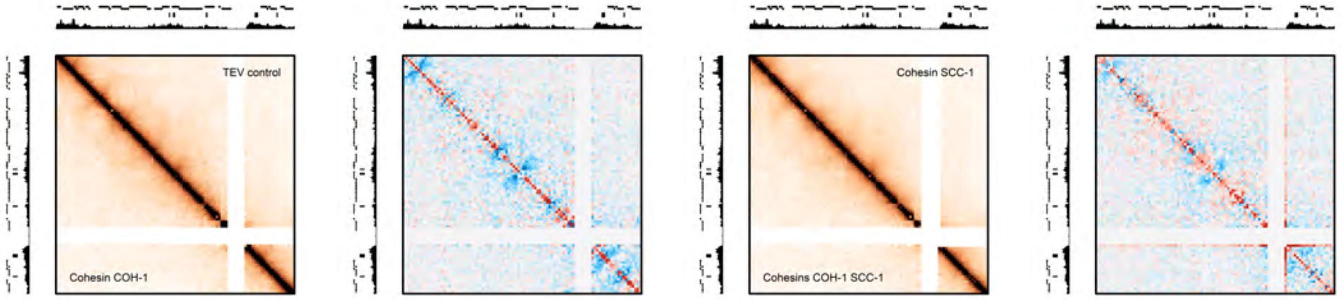

chrII:5945001-6145000

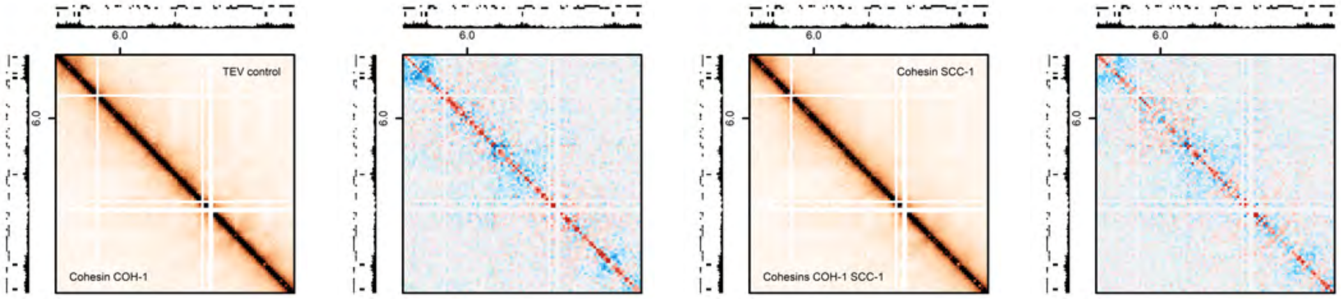

chrX:16899001-17099000

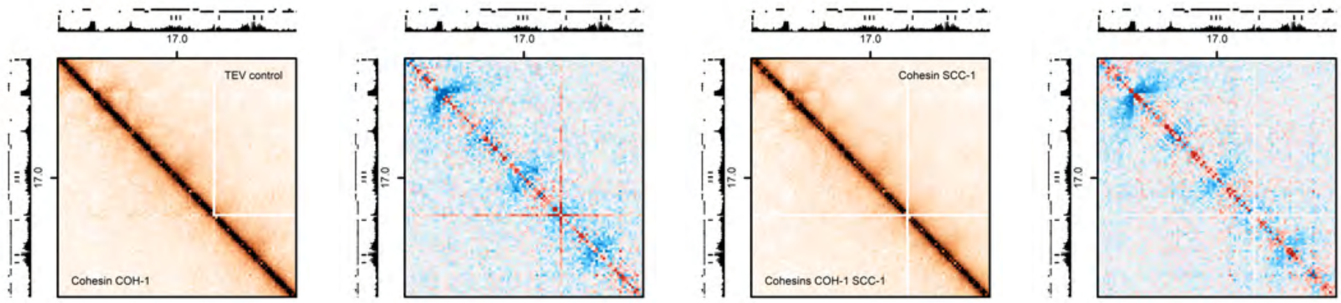

chrII:10657001-10857000

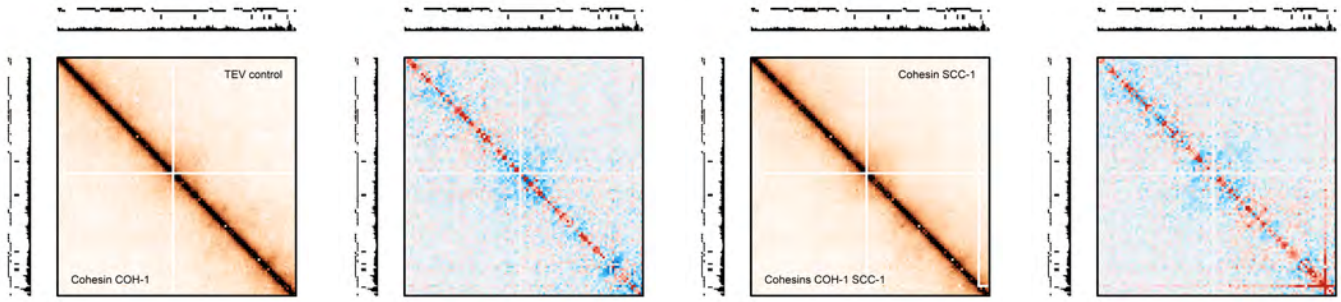

chrV:20613001-20813000

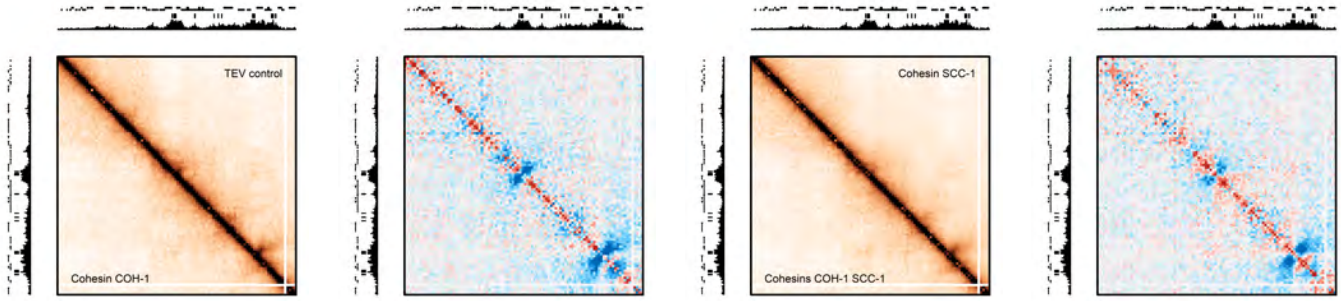

chrX:16959001-17159000

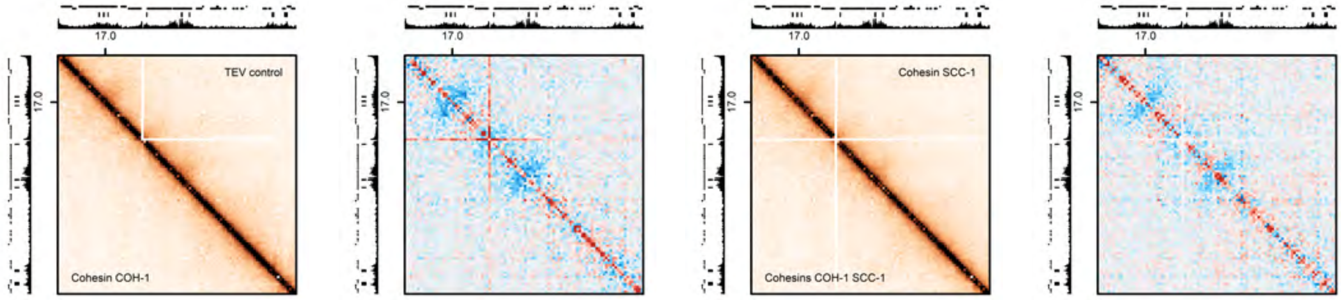

chrI:1385001-1585000

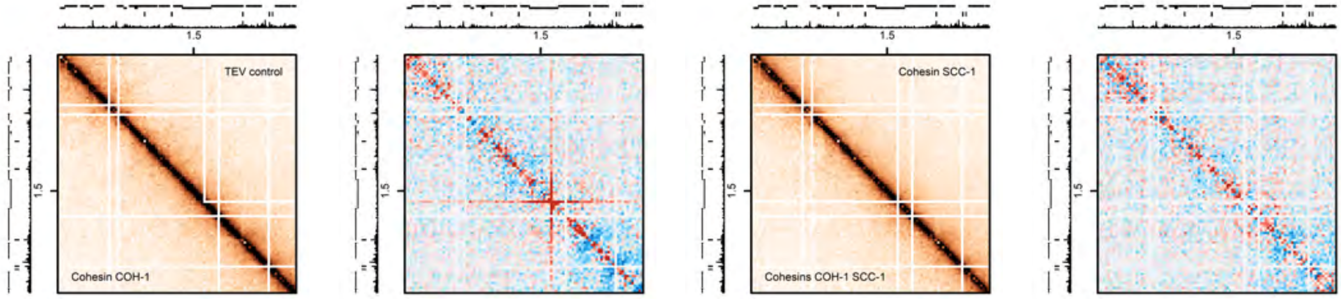

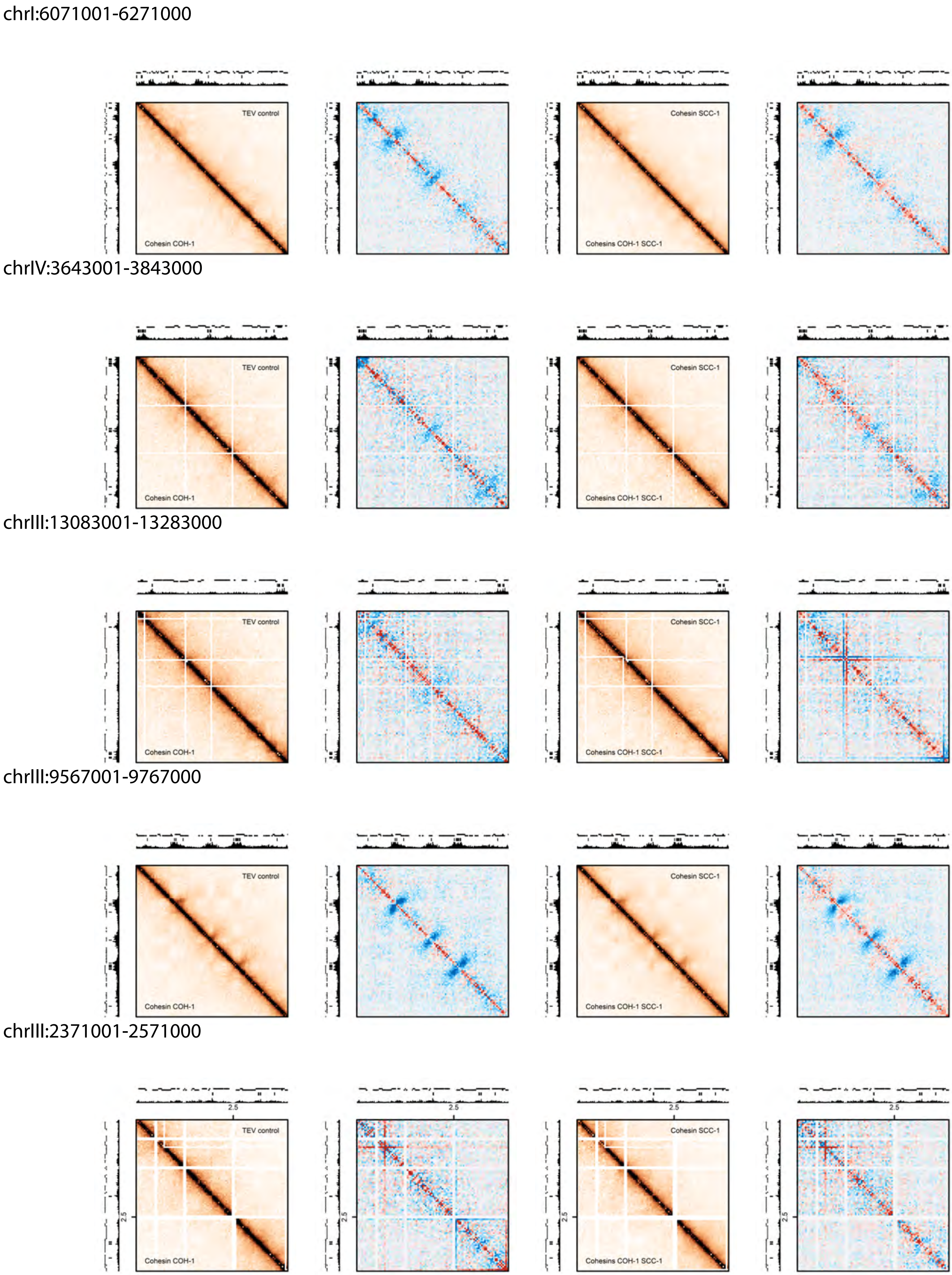
chrI:6071001-6271000
TEV control
Cohesin COH-1
Cohesin SCC-1
Cohesins COH-1 SCC-1
chrIV:3643001-3843000
TEV control
Cohesin COH-1
Cohesin SCC-1
Cohesins COH-1 SCC-1
chrIII:13083001-13283000
TEV control
Cohesin COH-1
Cohesin SCC-1
Cohesins COH-1 SCC-1
chrIII:9567001-9767000
TEV control
Cohesin COH-1
Cohesin SCC-1
Cohesins COH-1 SCC-1
chrIII:2371001-2571000
2.5
TEV control
Cohesin COH-1
Cohesin SCC-1
Cohesins COH-1 SCC-1

chrIV:14883001-15083000

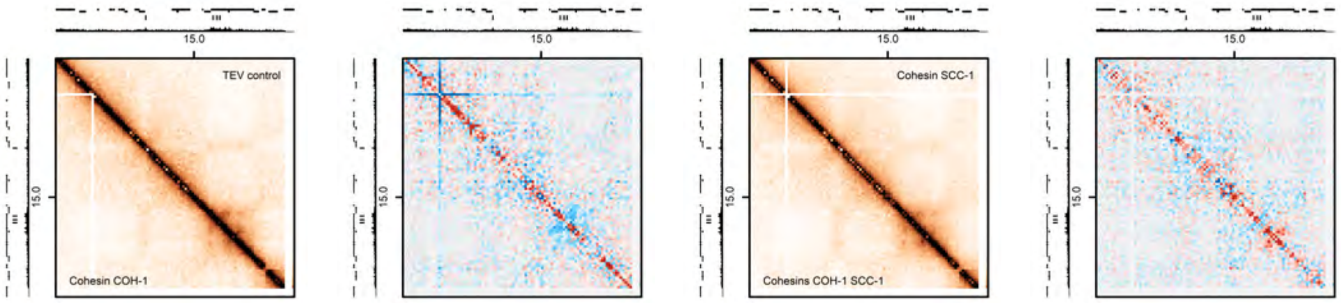

chrIV:7561001-7761000

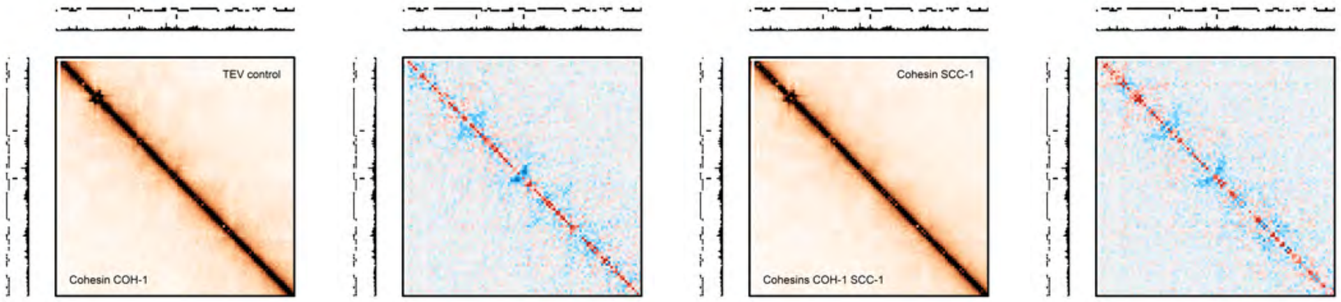

chrX:11311001-11511000

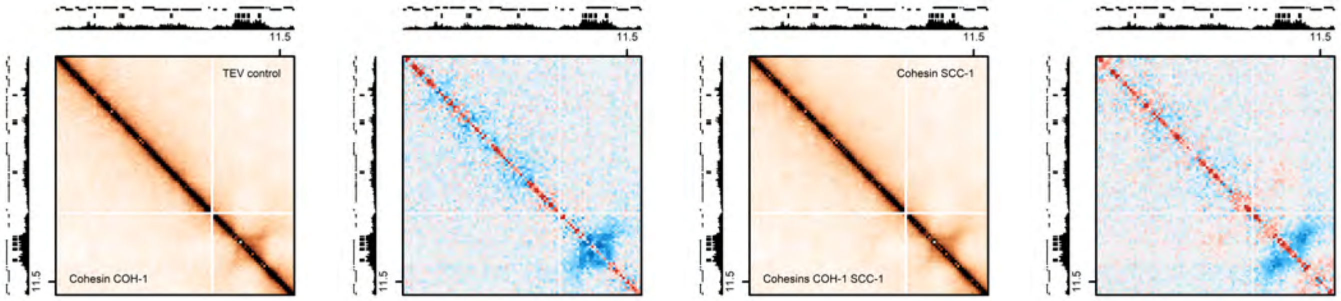

chrV:11923001-12123000

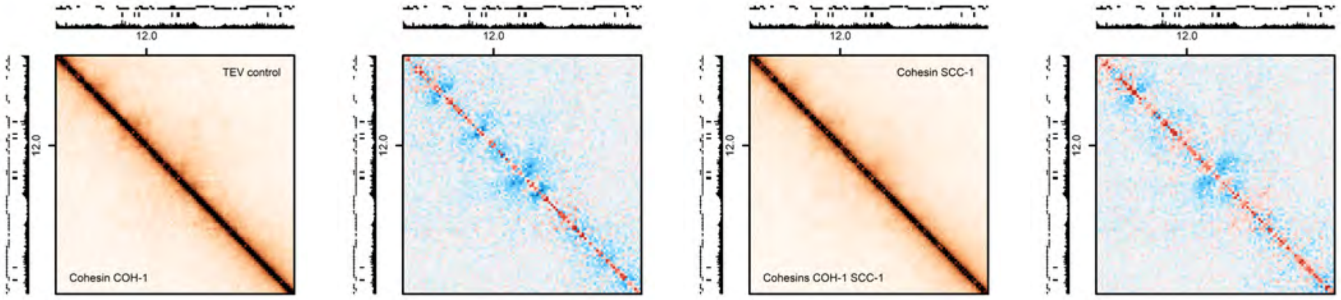

chrII:993001-1193000

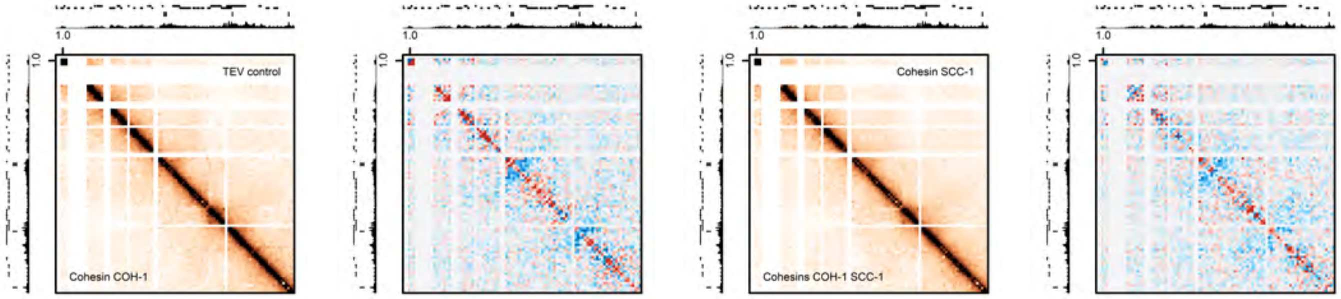

chrII:13233001-13433000

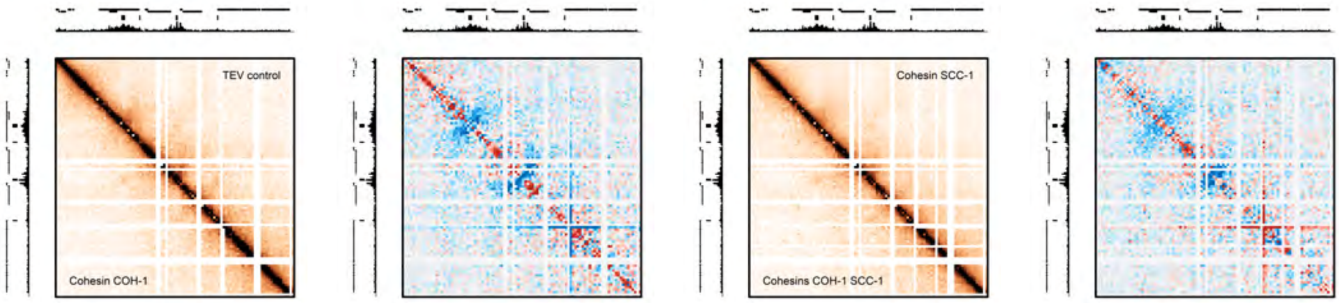

chrII:9975001-10175000

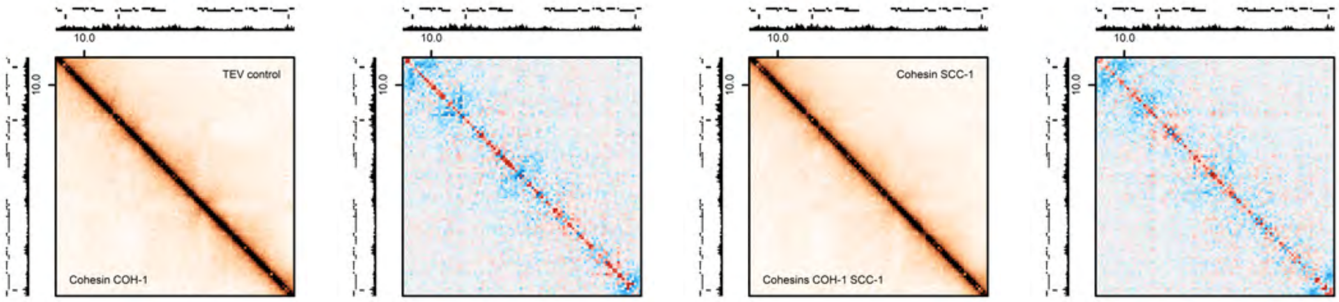

chrX:7855001-8055000

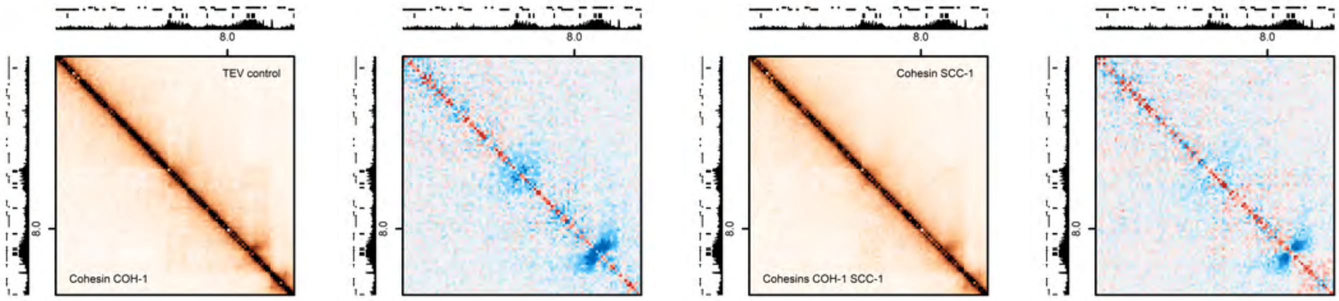

chrI:5445001-5645000

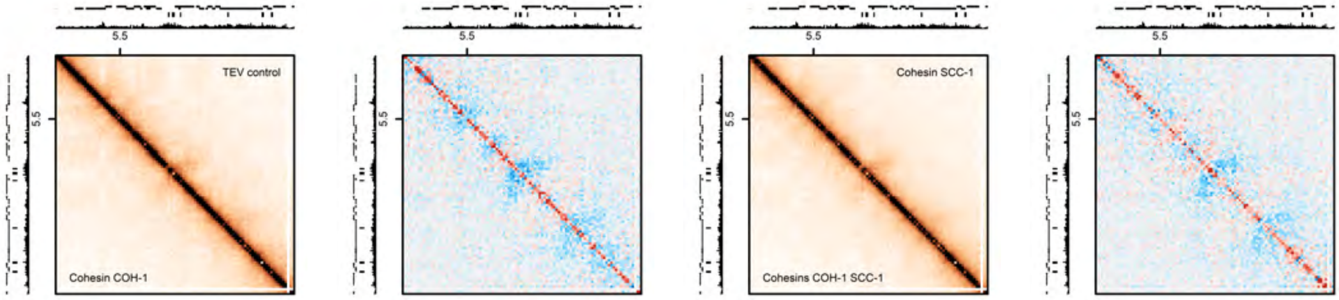

chrX:9121001-9321000

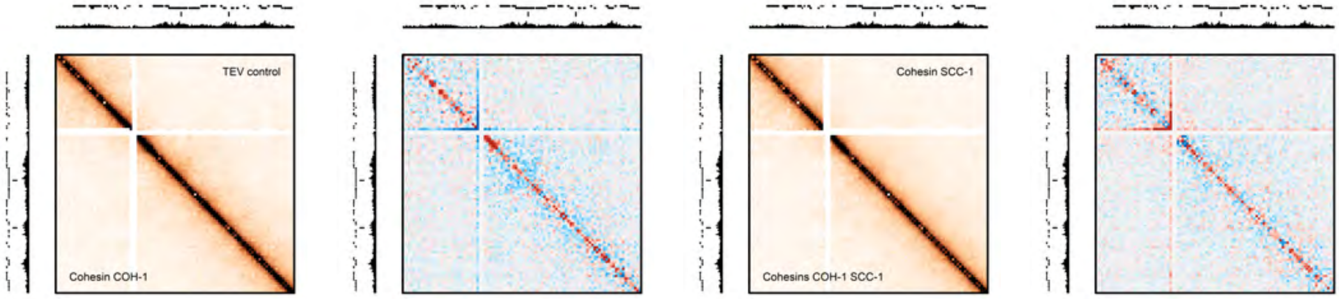

chrIII:3429001-3629000

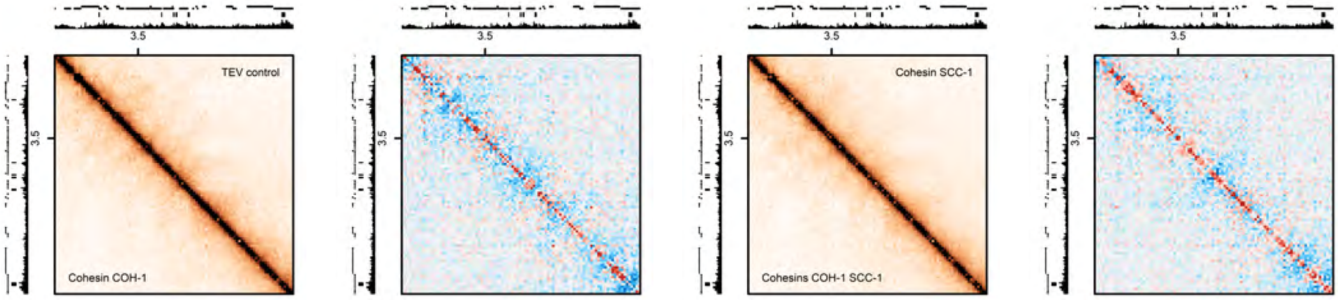

chrII:3715001-3915000

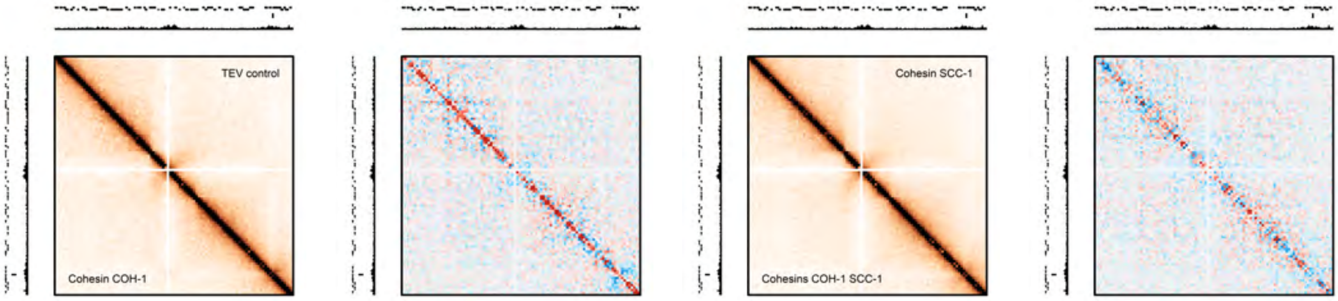

chrII:3993001-4193000

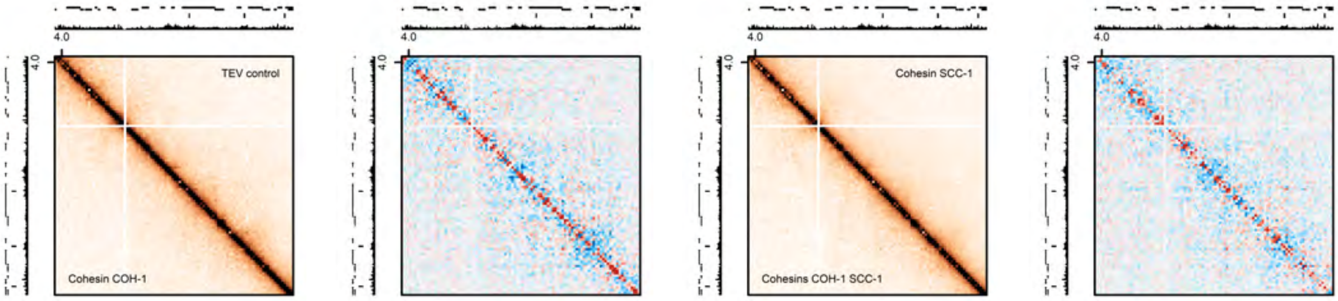

chrIV:4999001-5199000

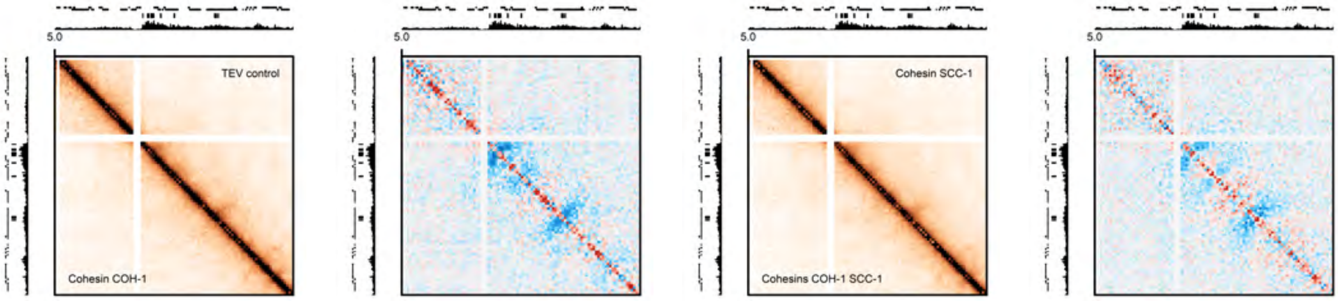

chrX:6859001-7059000

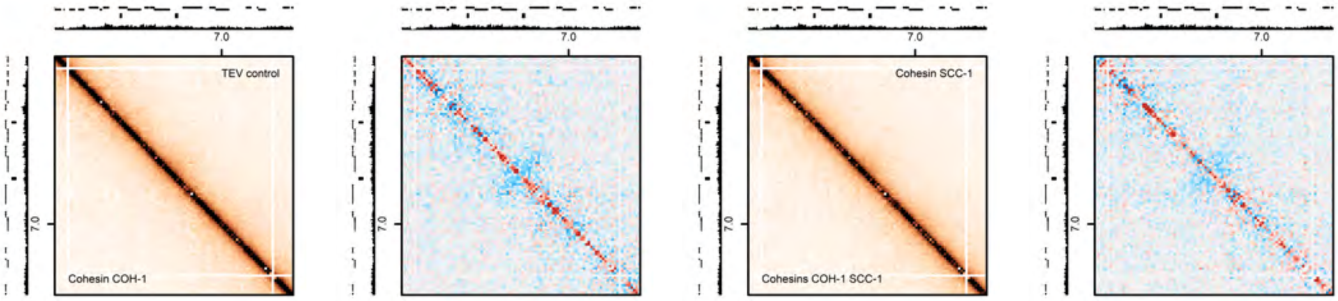

chrII:7183001-7383000

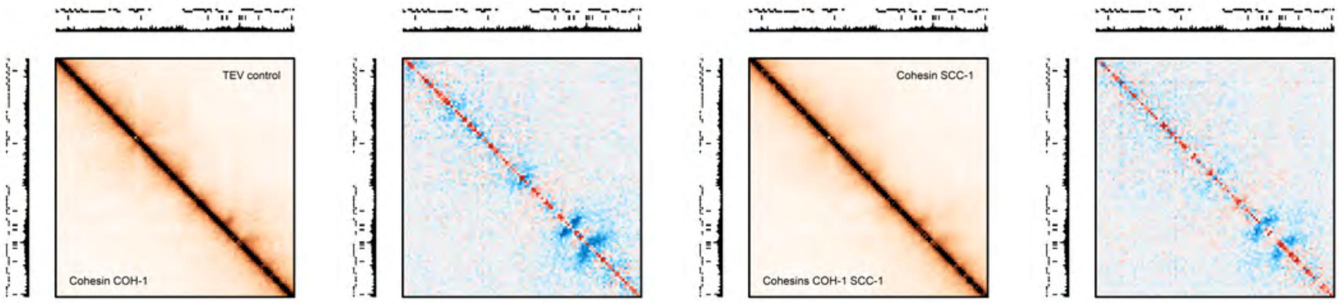

chrII:12163001-12363000

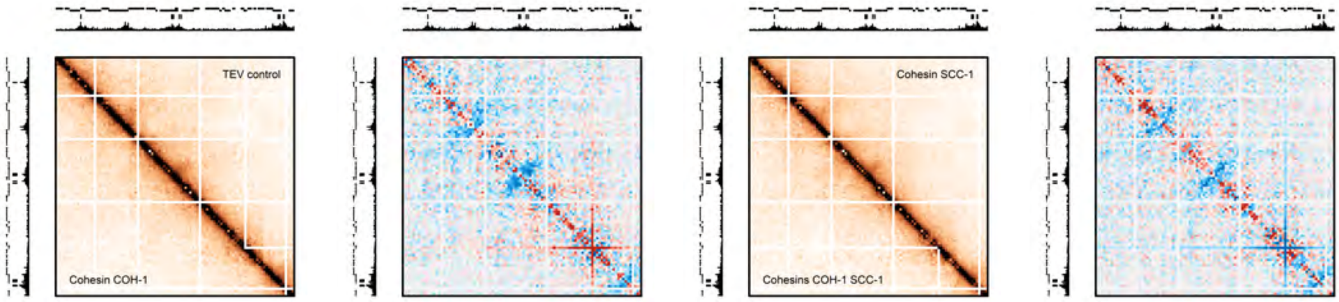

chrV:2031001-2231000

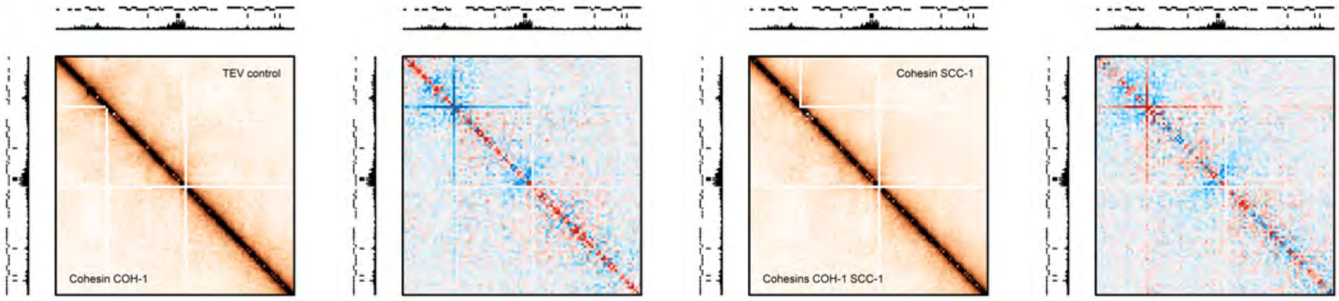

chrIII:5193001-5393000

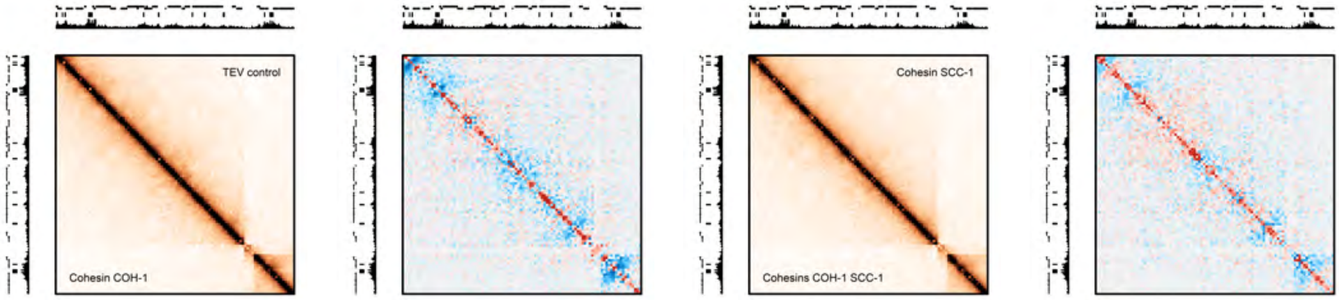

chrI:7499001-7699000

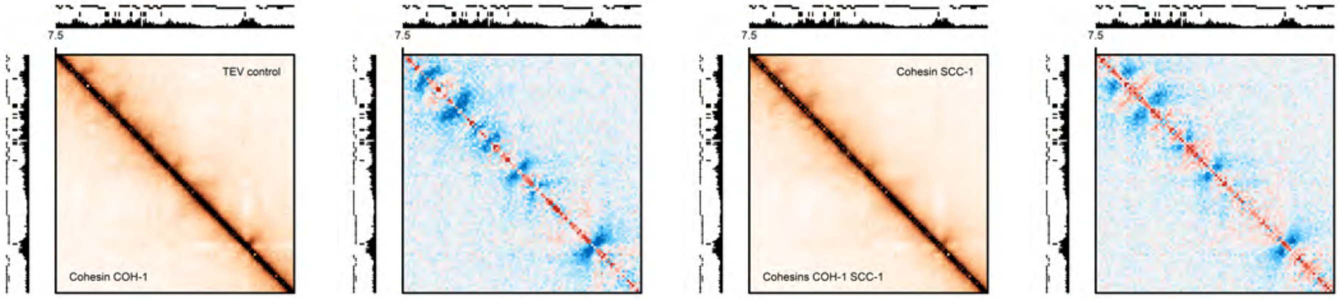

chrIV:11713001-11913000

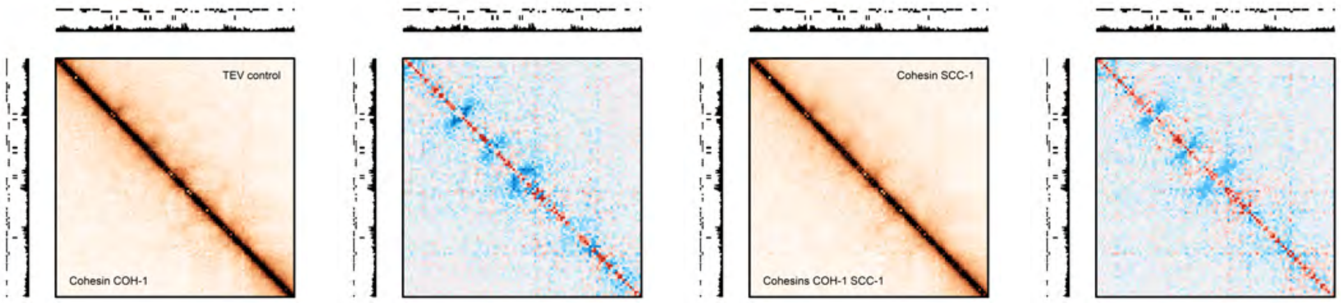

chrIV:13153001-13353000

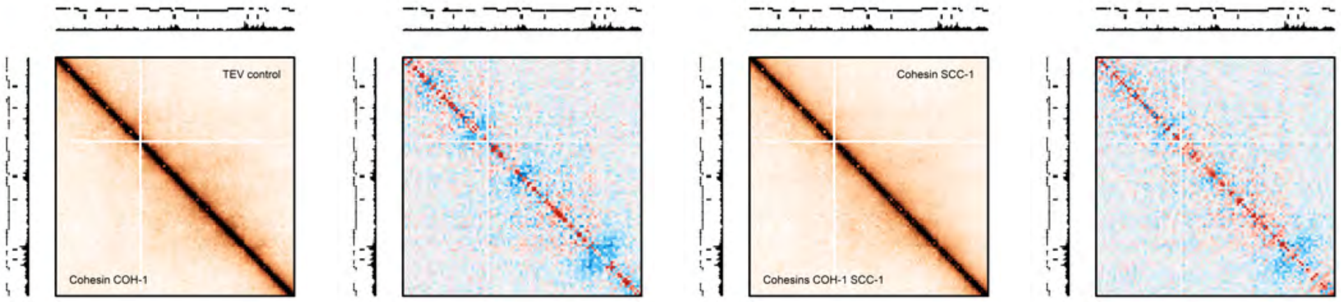

chrII:7003001-7203000

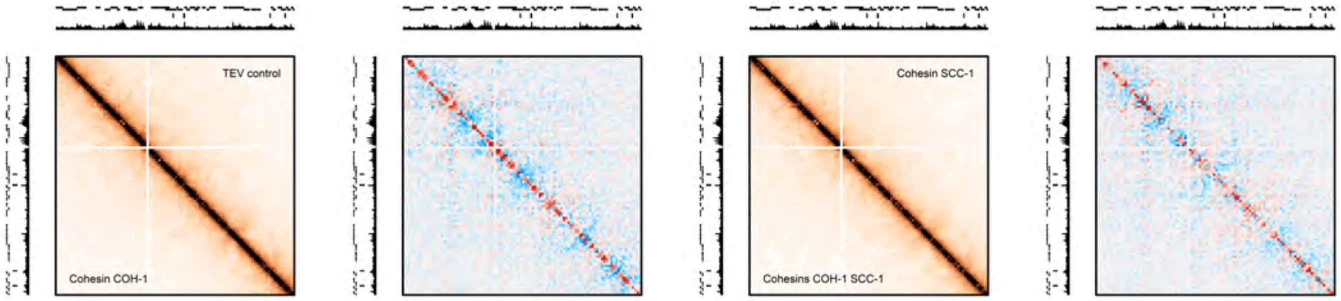

chrI:11275001-11475000

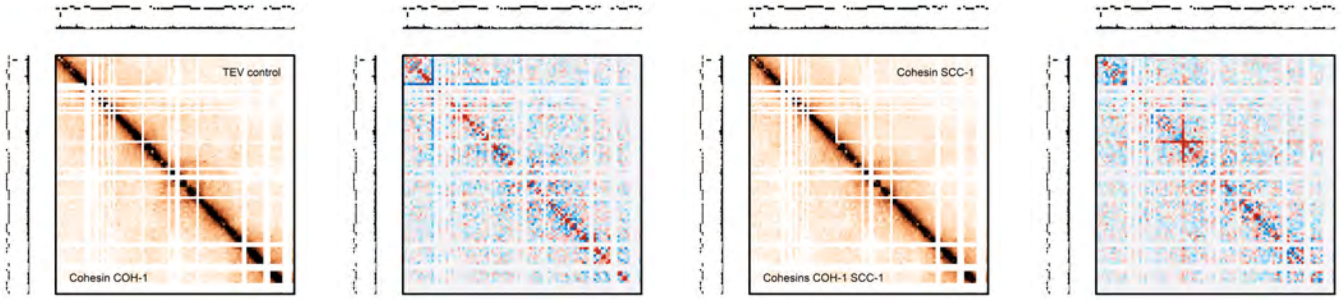

chrX:13925001-14125000

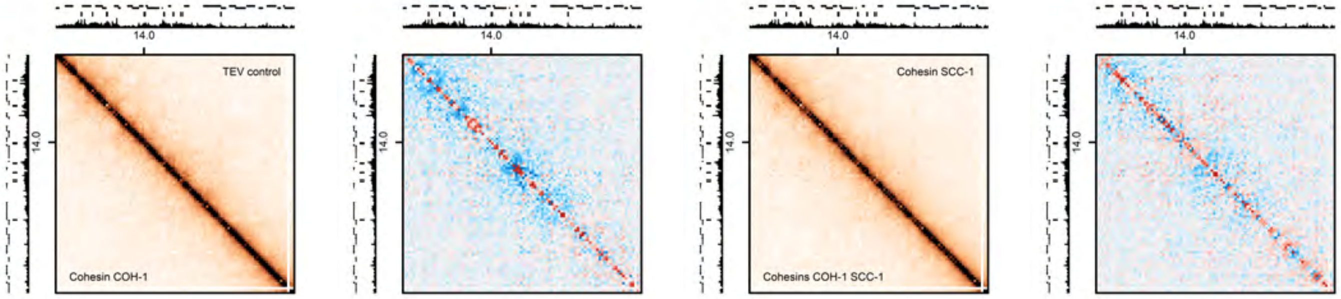

chrIII:8635001-8835000

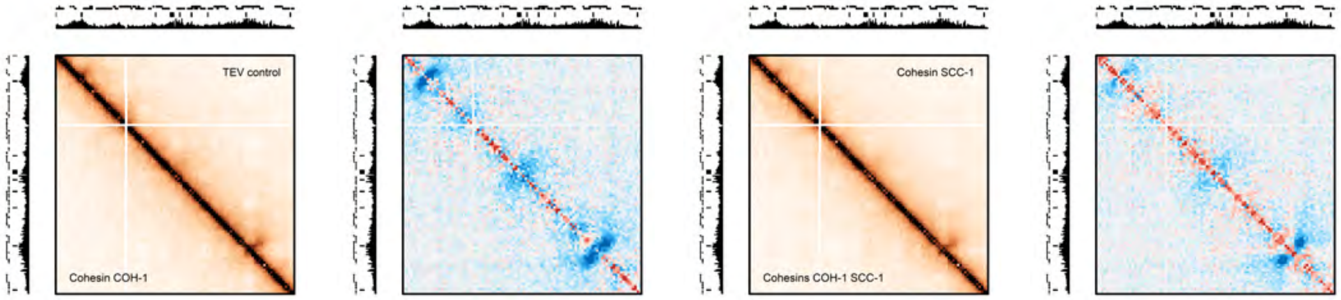

chrI:13543001-13743000

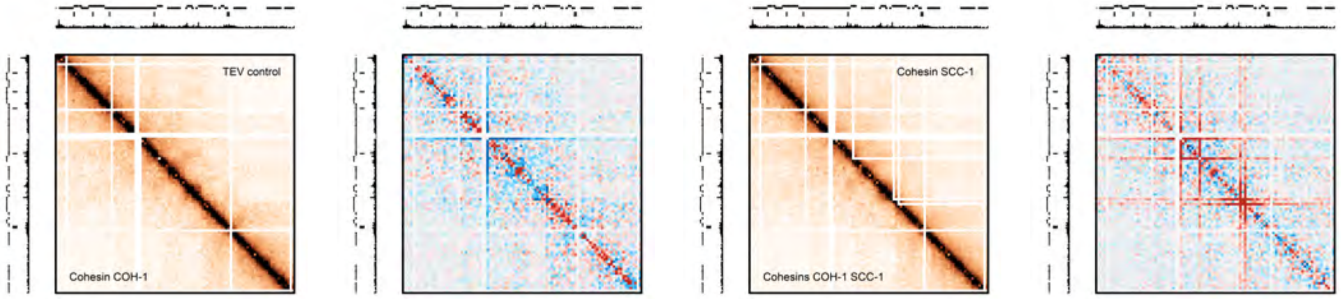

chrX:12907001-13107000

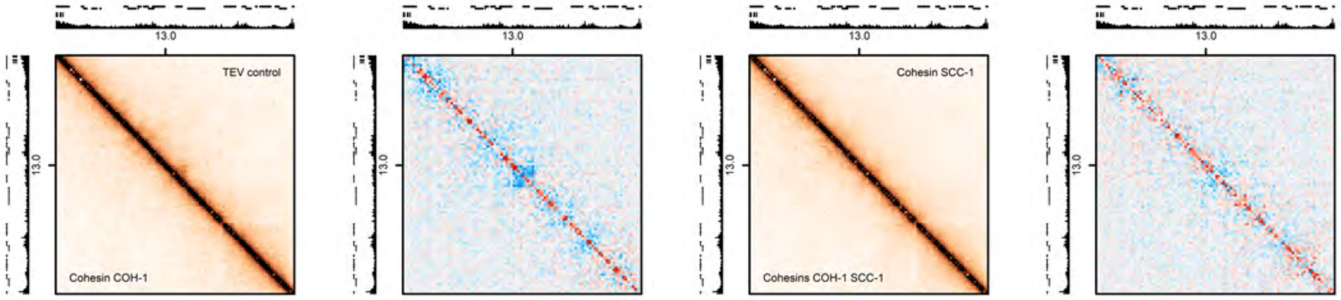

chrI:5727001-5927000

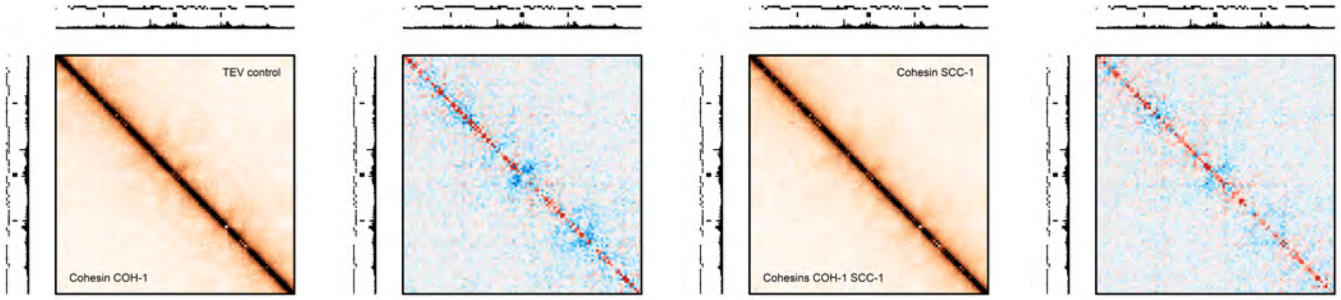

chrX:17325001-17525000

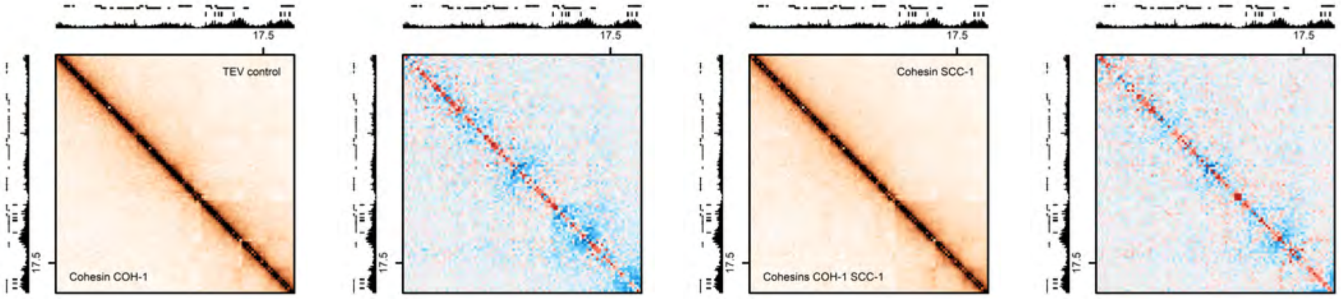

chrI:11897001-12097000

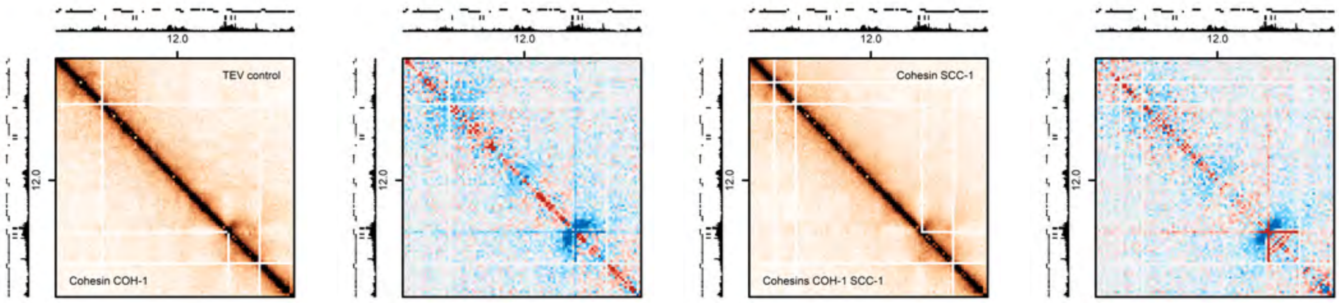

chrII:7739001-7939000

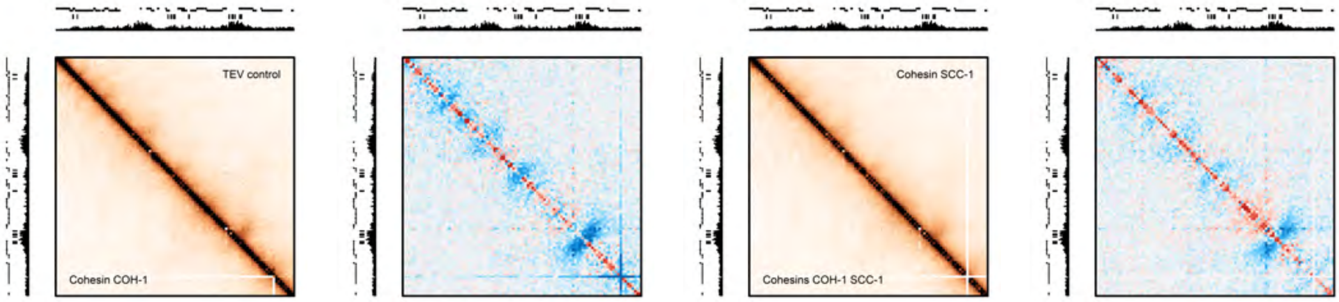

chrV:13067001-13267000

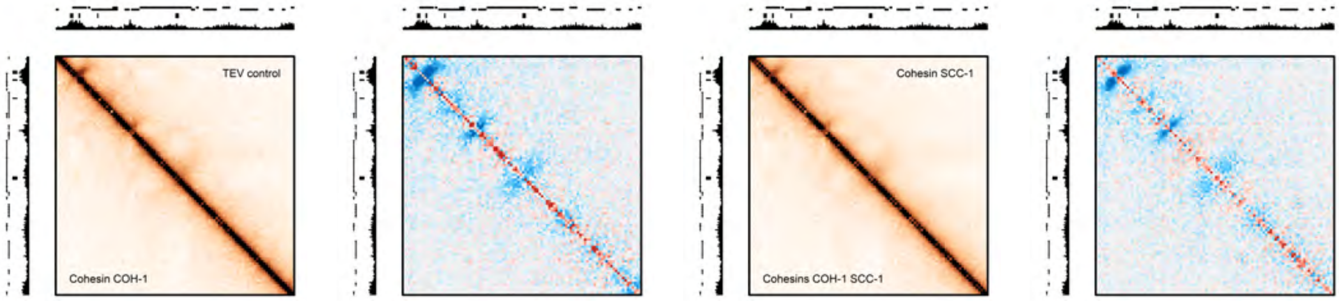

chrIII:1639001-1839000

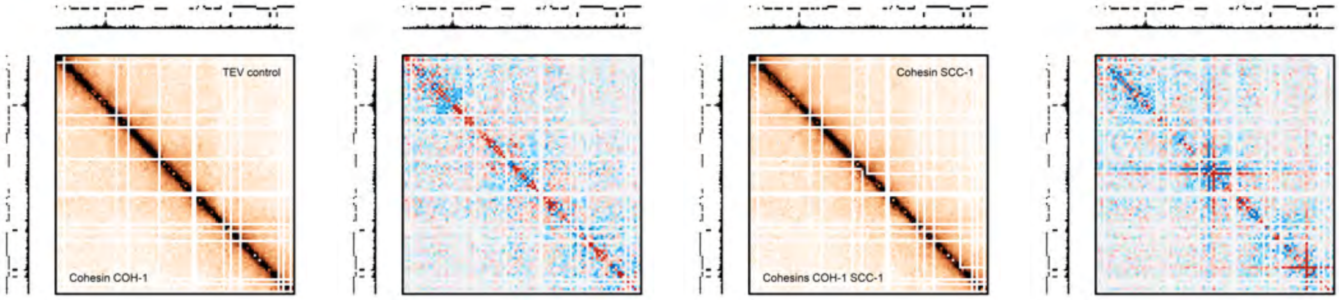

chrX:3499001-3699000

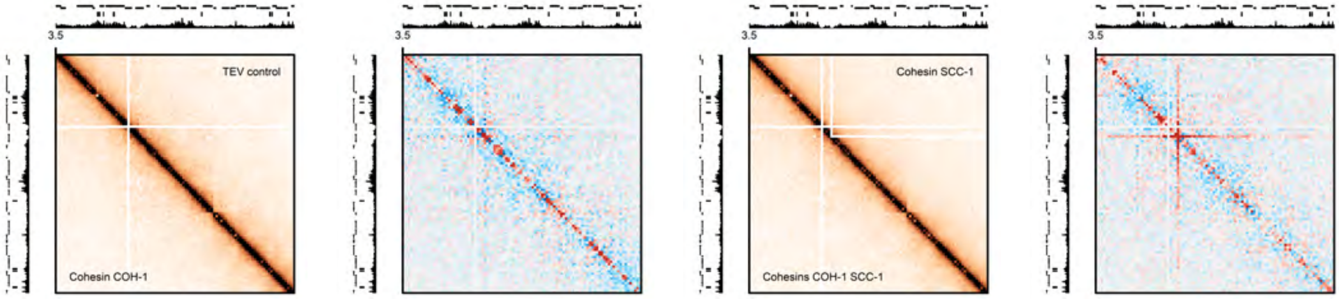

chrIII:4979001-5179000

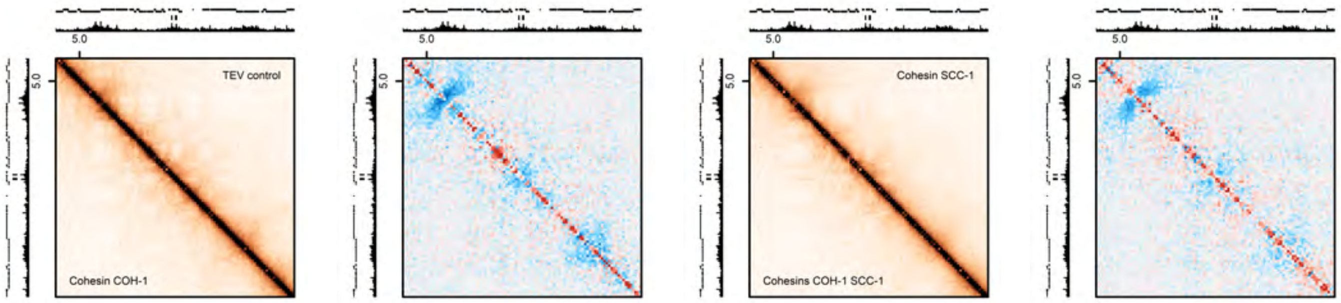

chrX:16305001-16505000

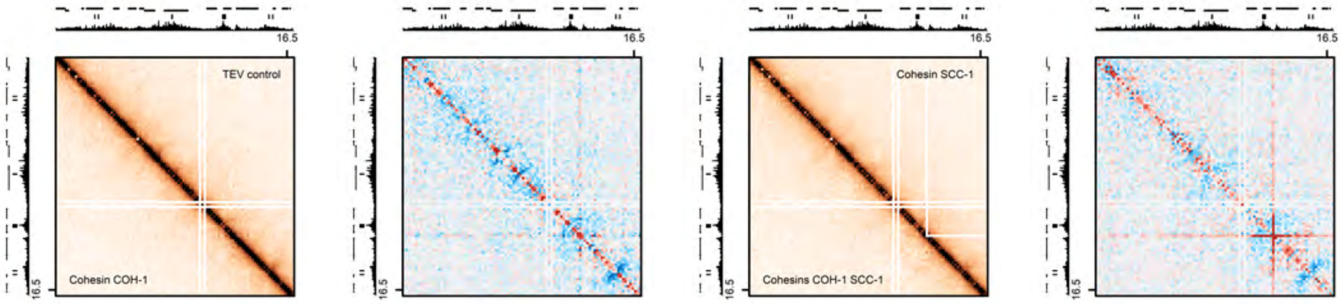

chrI:9507001-9707000

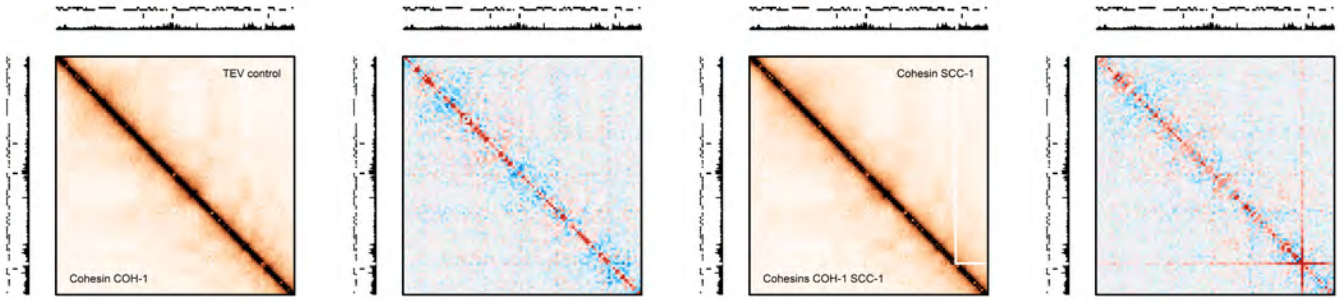

chrV:19521001-19721000

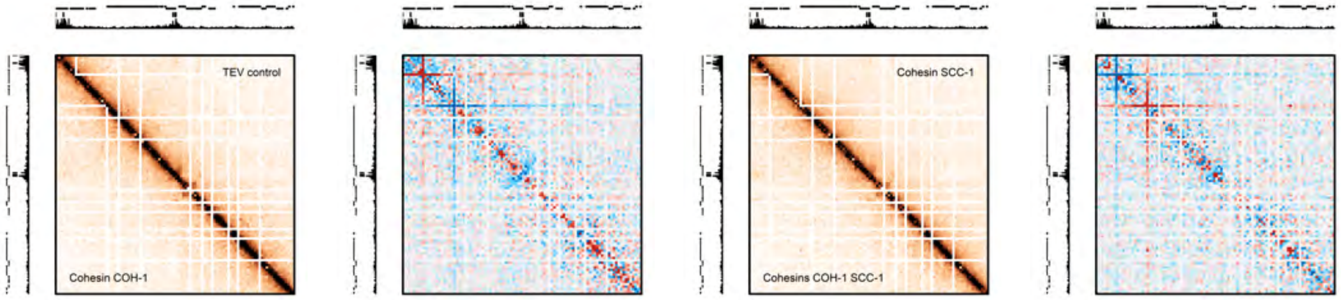

chrIII:571001-771000

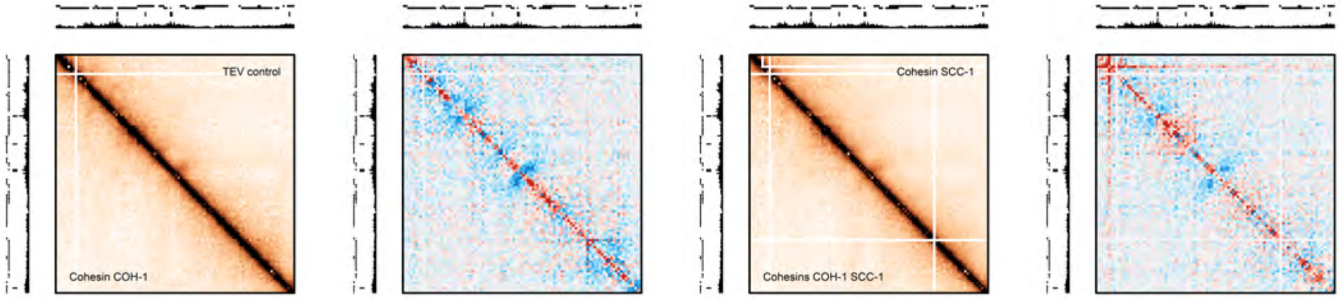

chrIV:7795001-7995000

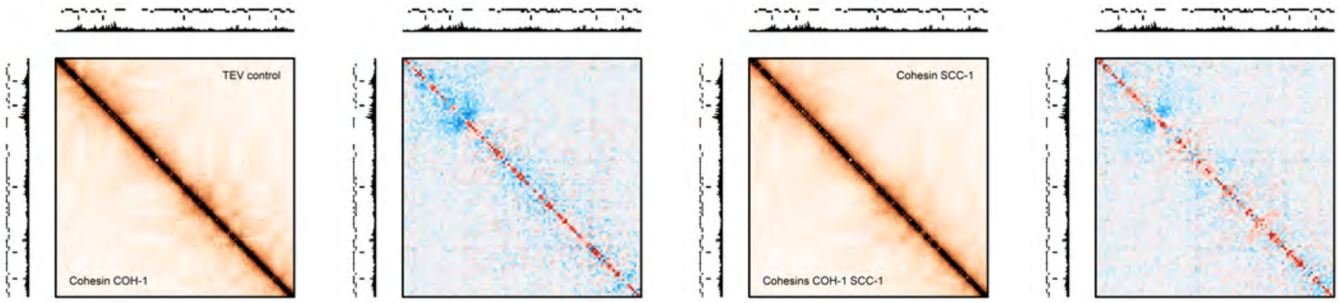

chrV:3179001-3379000

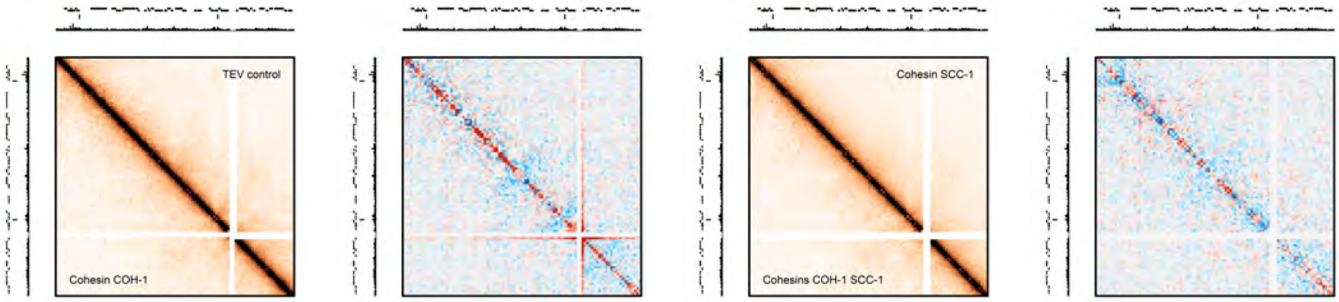

chrII:8831001-9031000

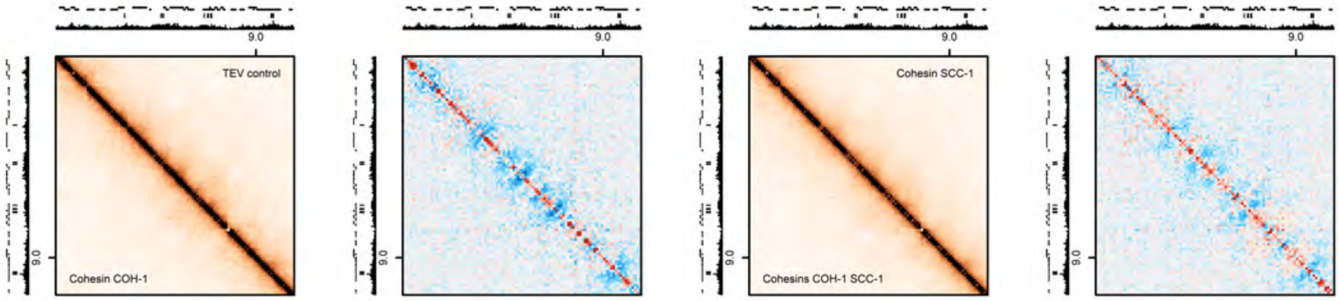

chrI:1129001-1329000

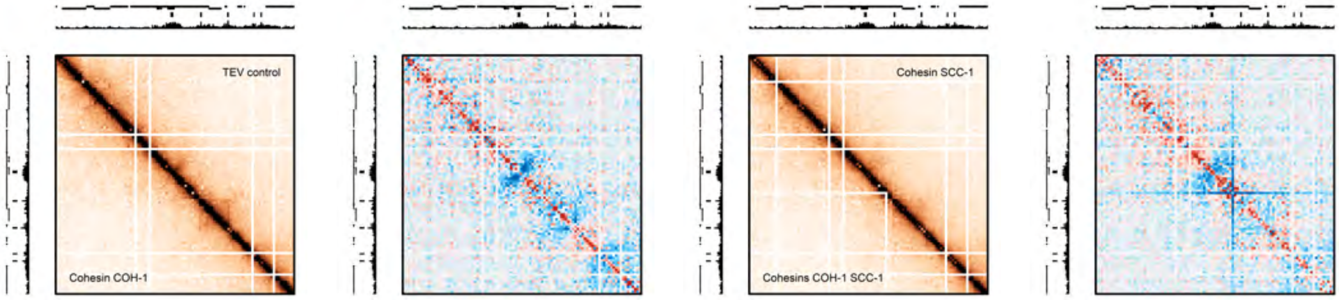

chrII:13723001-13923000

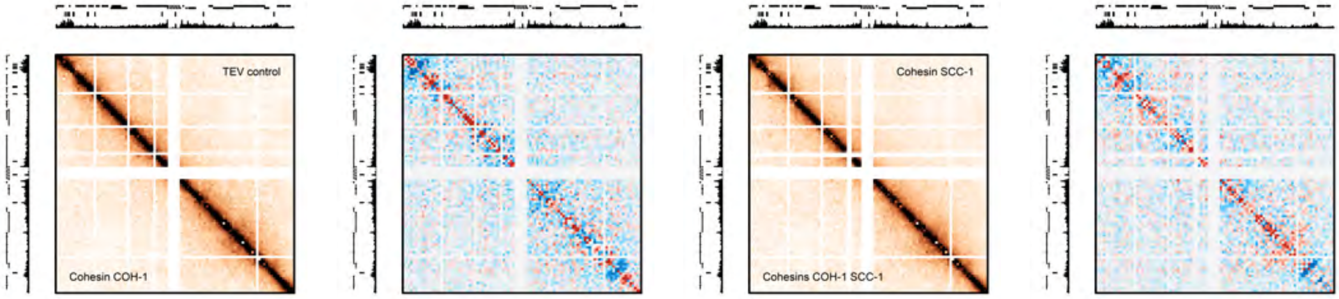

chrII:2823001-3023000

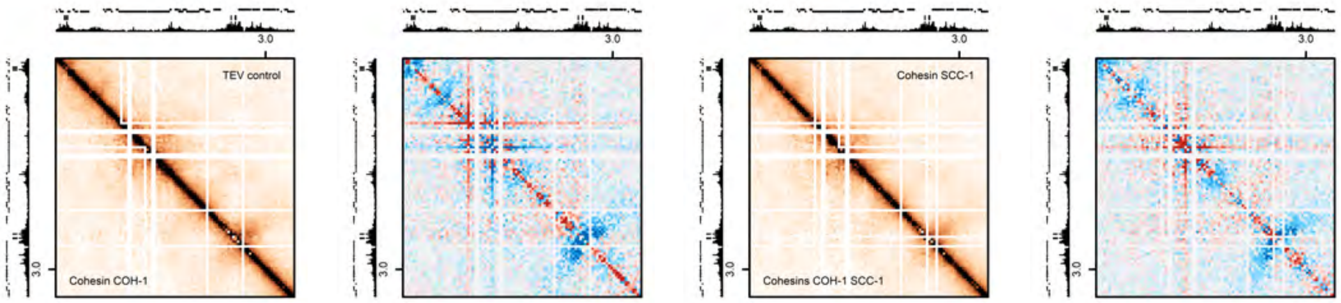

chrI:8583001-8783000

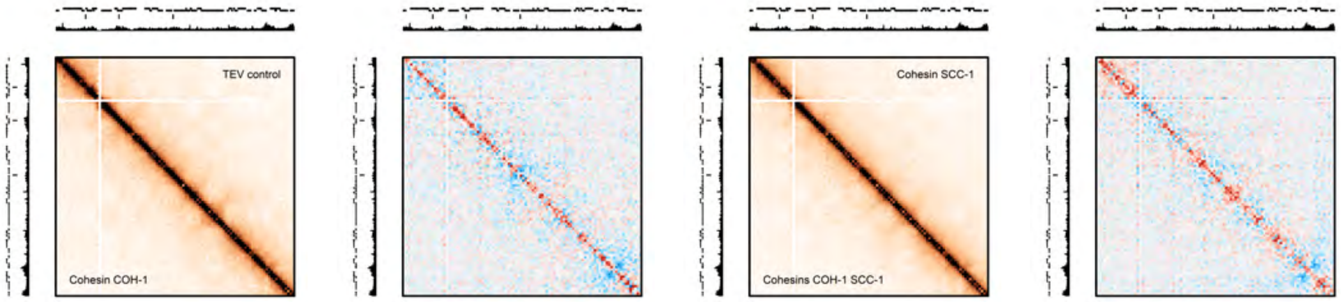

chrI:14943001-15143000

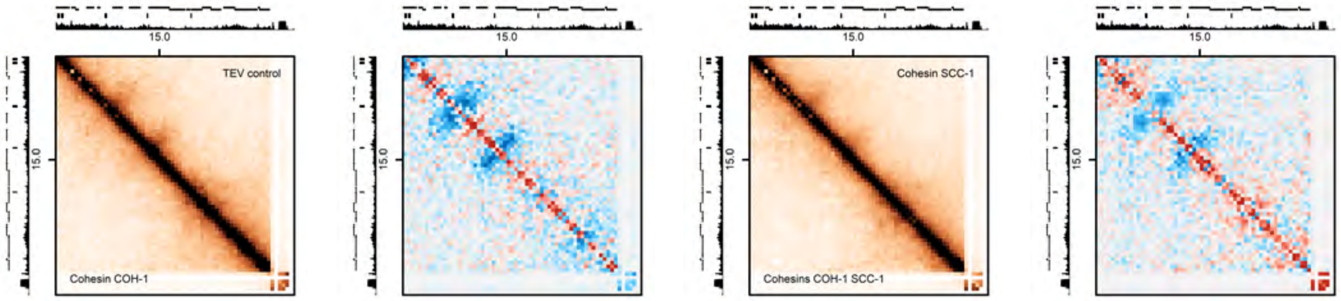

chrII:11229001-11429000

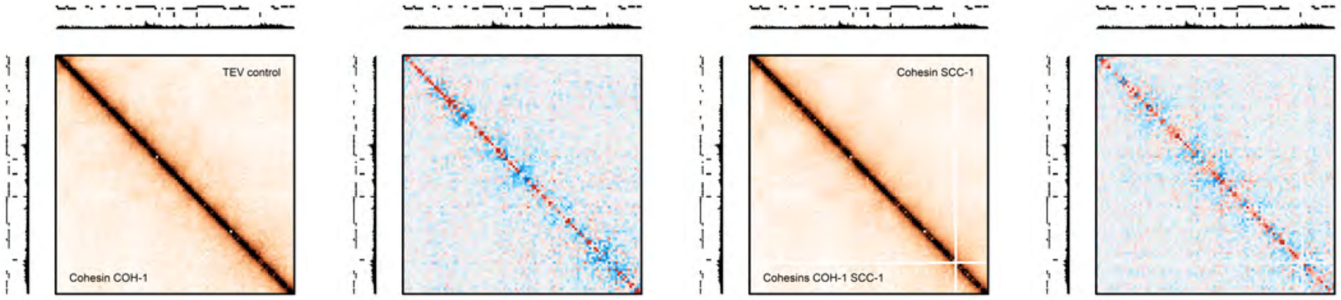

chrII:659001-859000

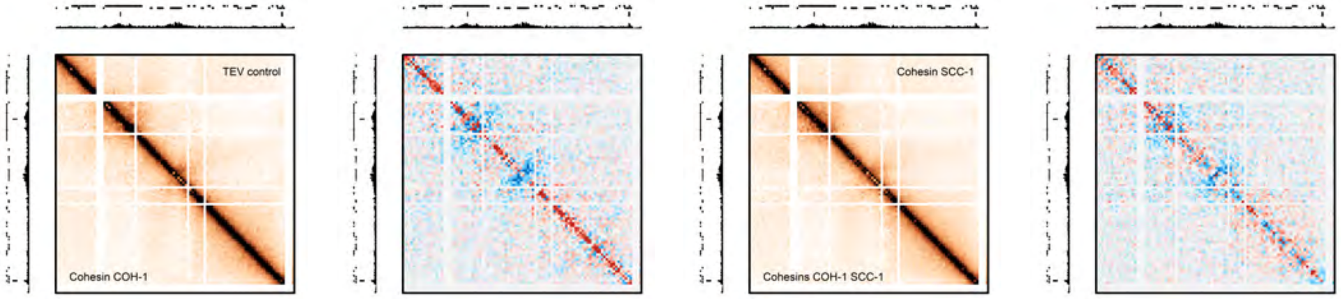

chrV:17807001-18007000

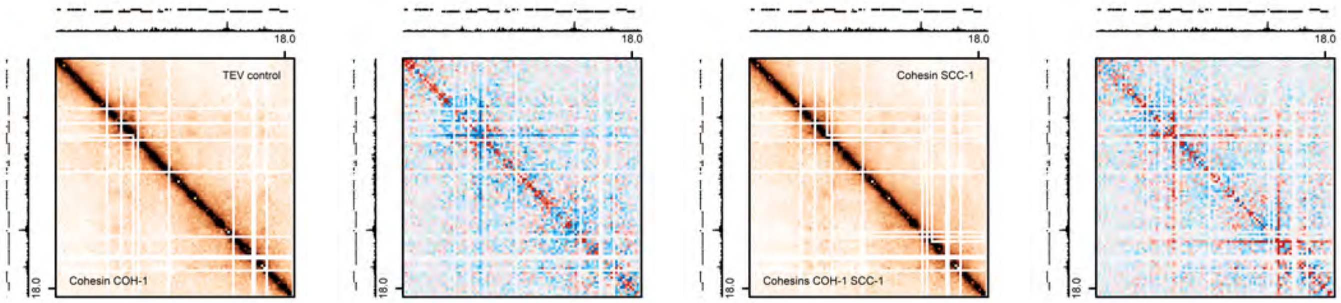

chrI:749001-949000

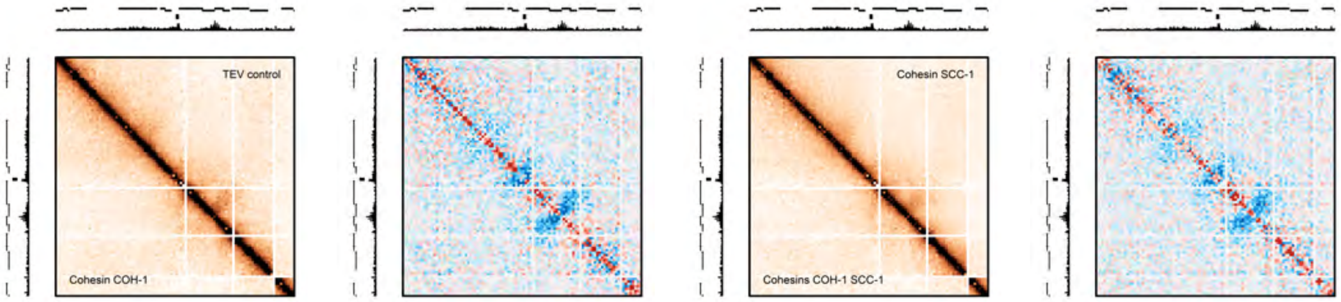

chrX:10805001-11005000

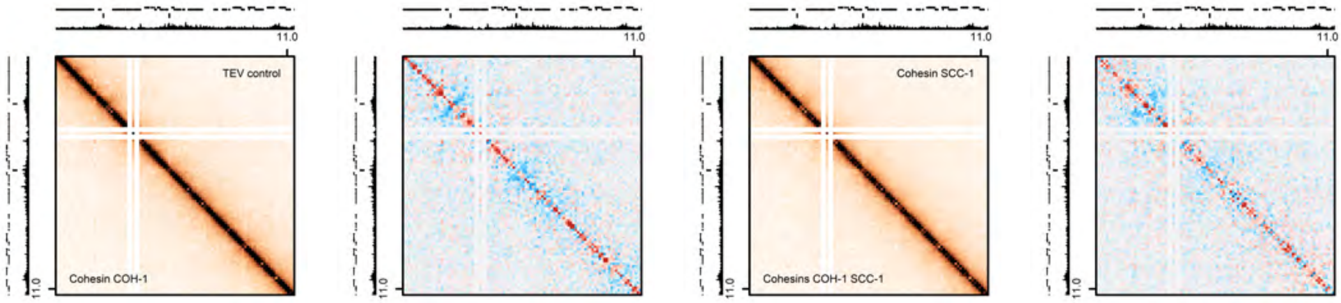

chrX:12775001-12975000

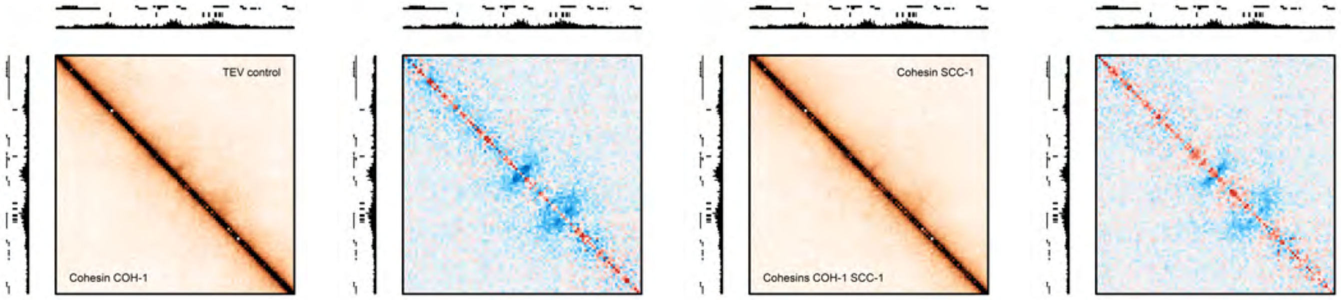

chrV:1245001-1445000

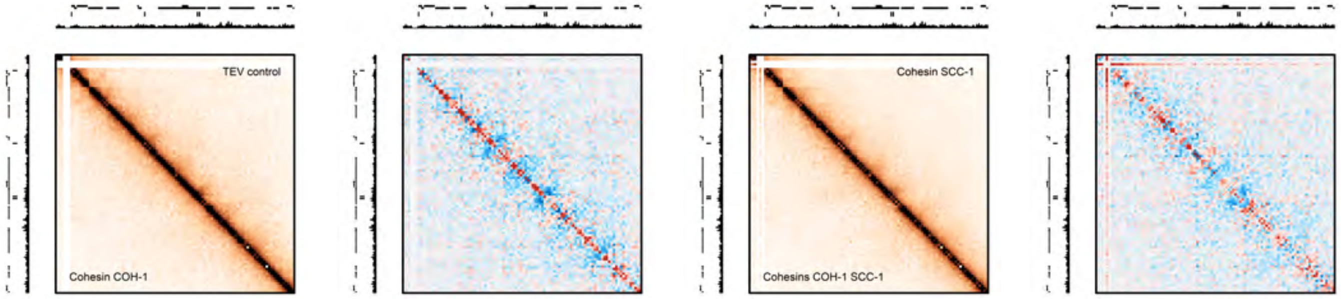

chrX:8465001-8665000

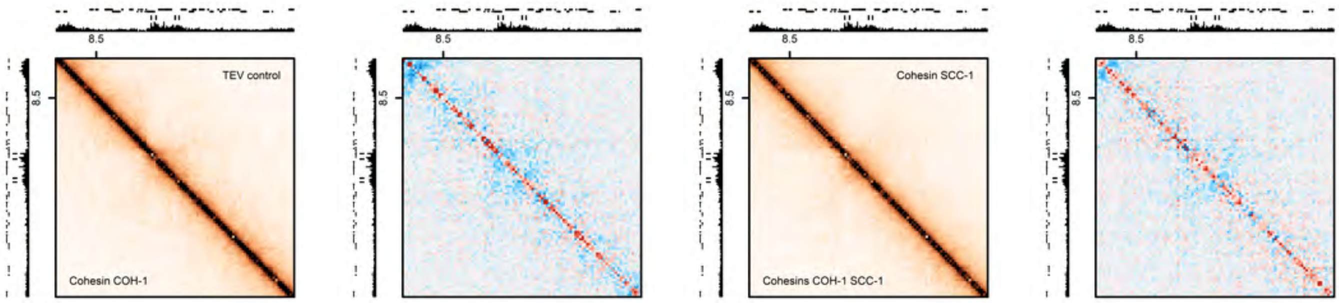

chrIV:9701001-9901000

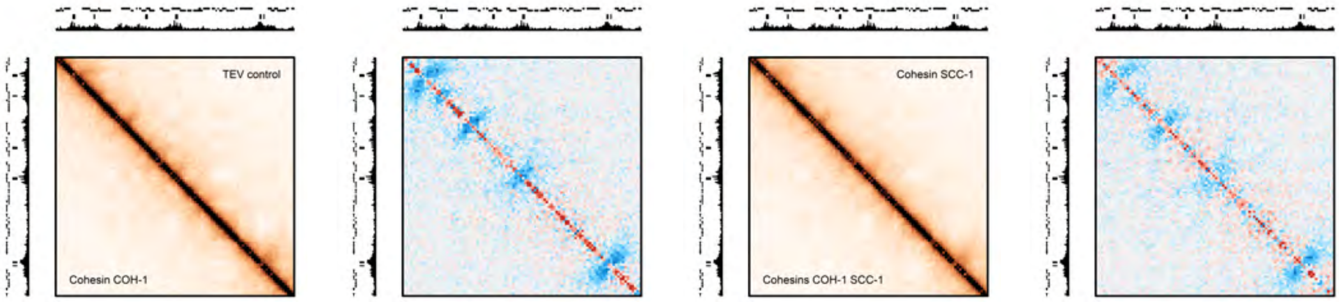

chrI:14039001-14239000

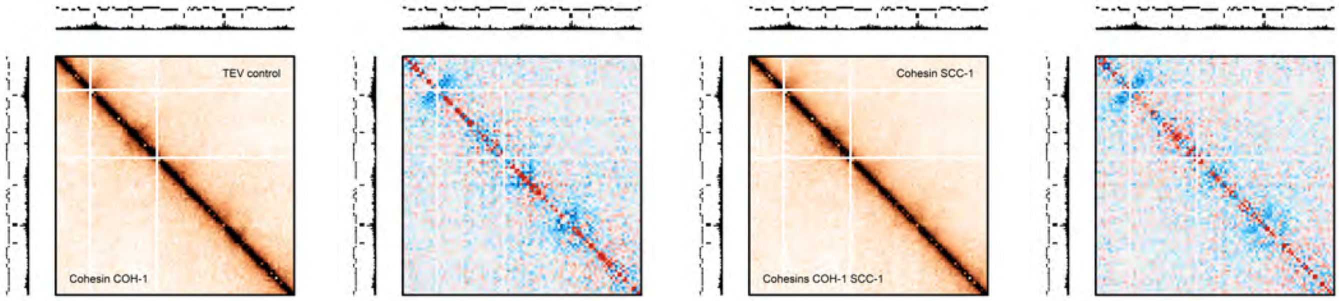

chrIII:4851001-5051000

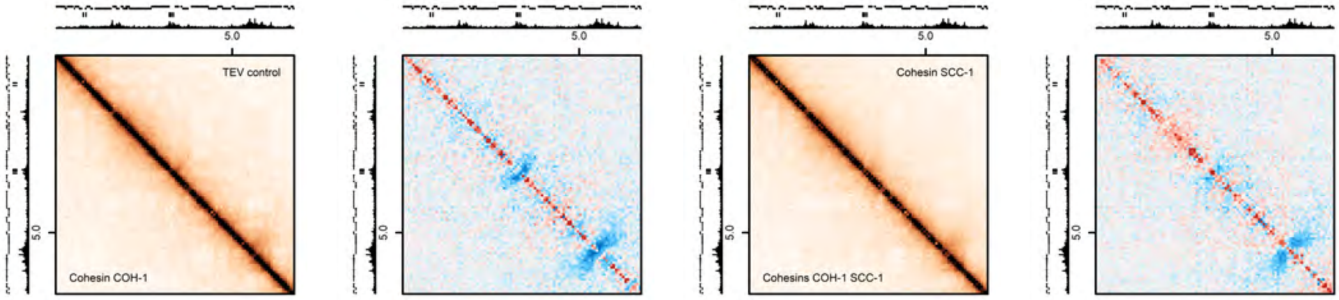

chrII:14973001-15173000

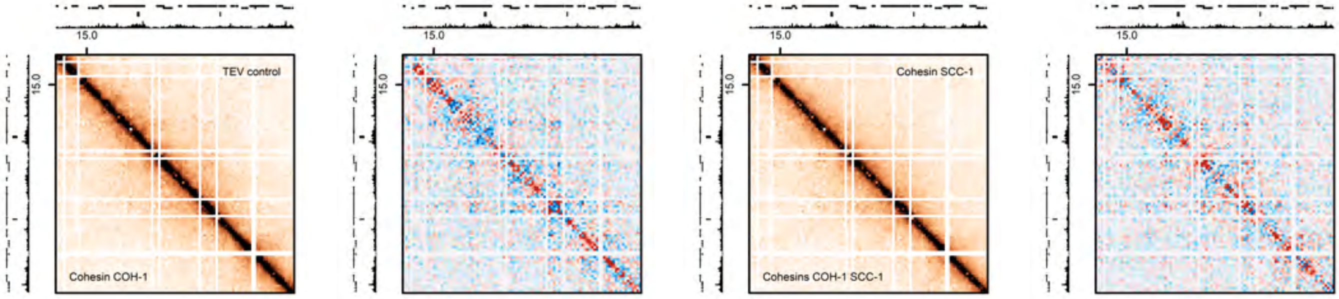

chrX:15089001-15289000

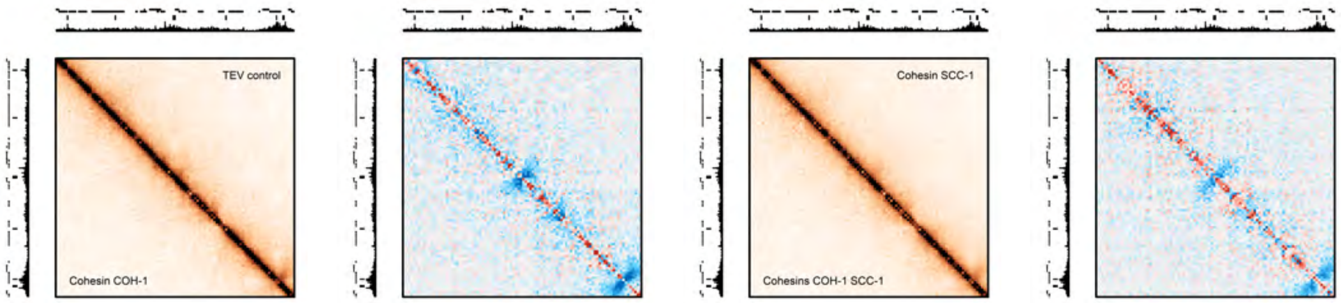

chrII:4705001-4905000

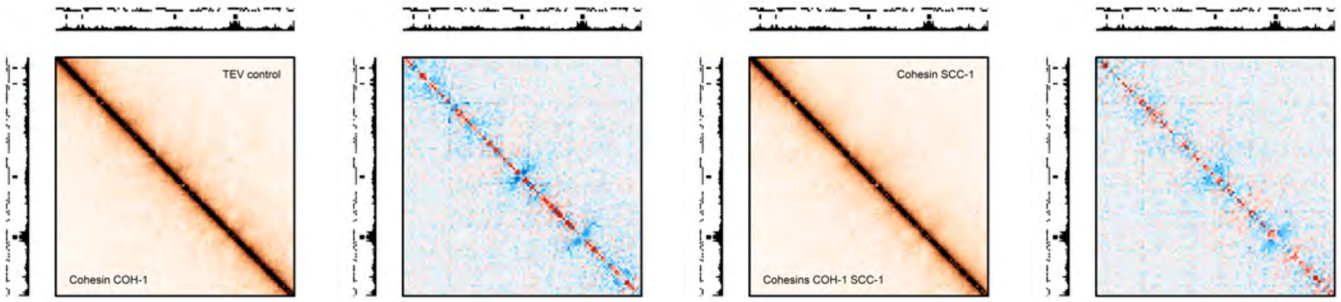

chrX:9741001-9941000

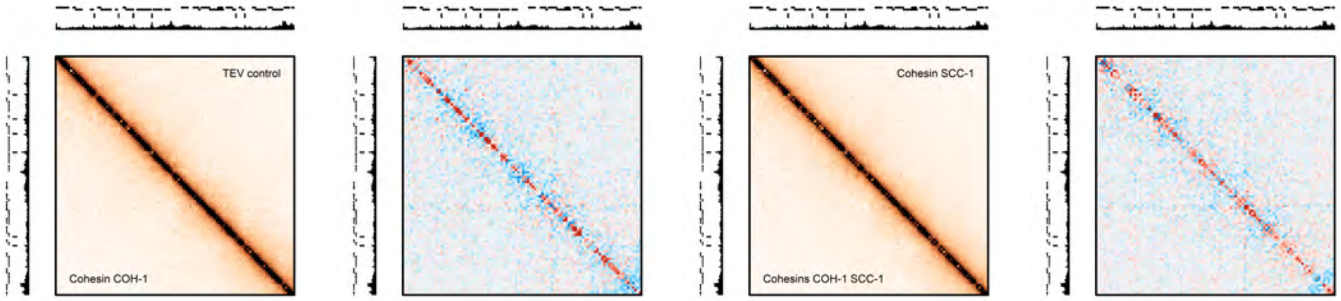

chrII:14569001-14769000

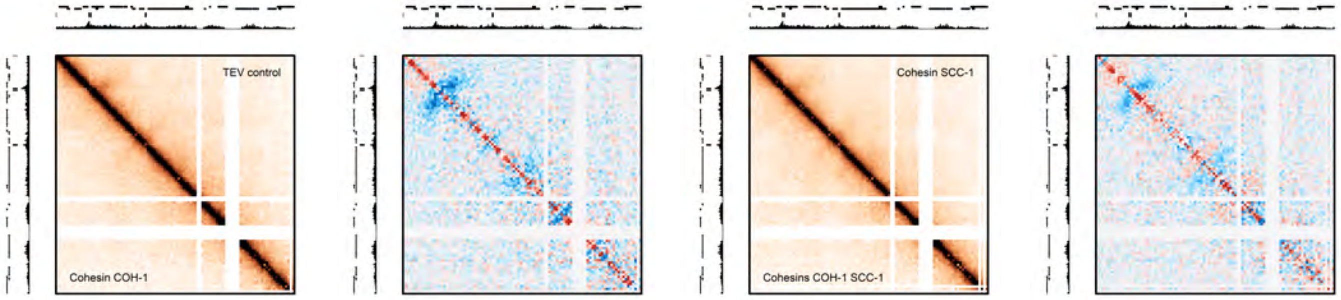

chrIII:645001-845000

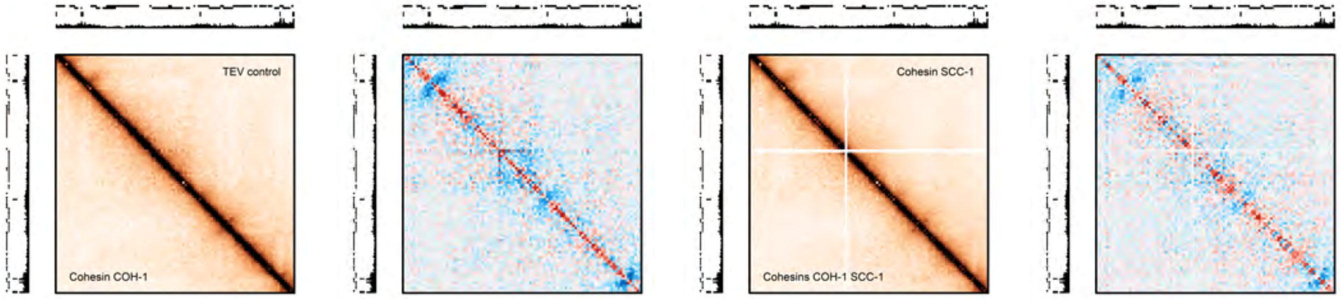

chrI:7849001-8049000

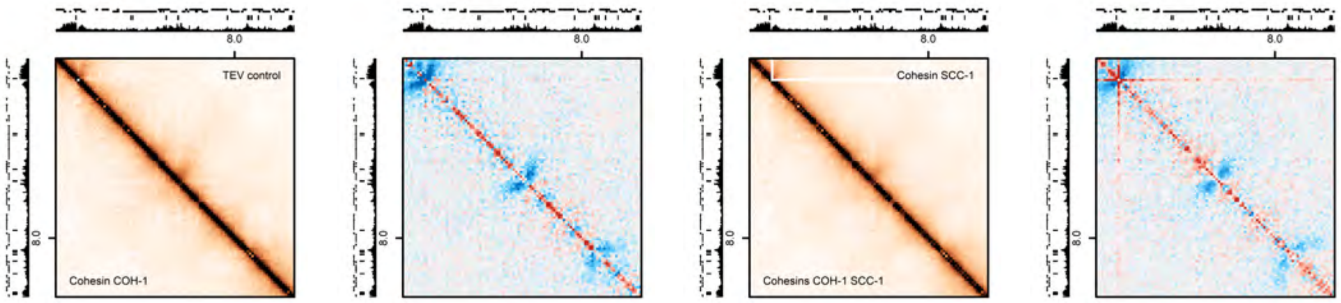

chrIII:11459001-11659000

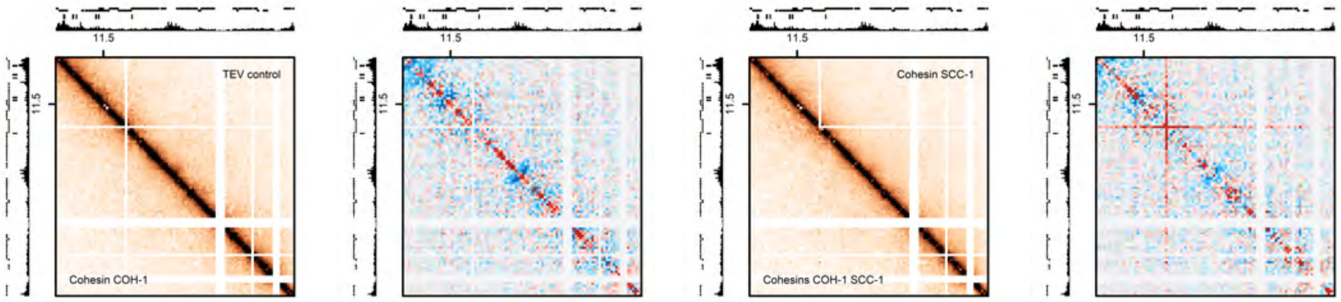

chrX:15607001-15807000

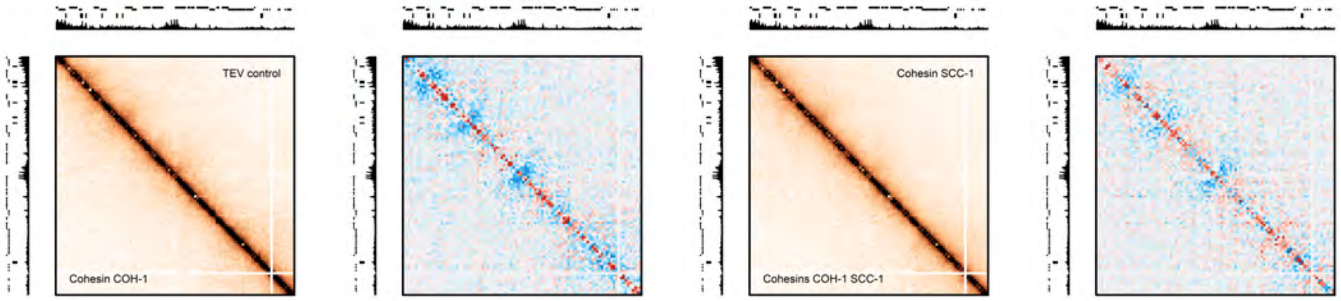

chrIII:9807001-10007000

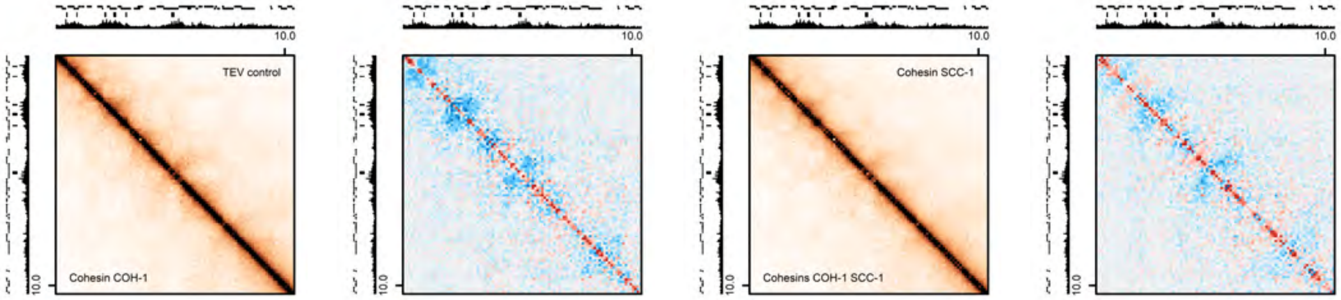

chrIV:5719001-5919000

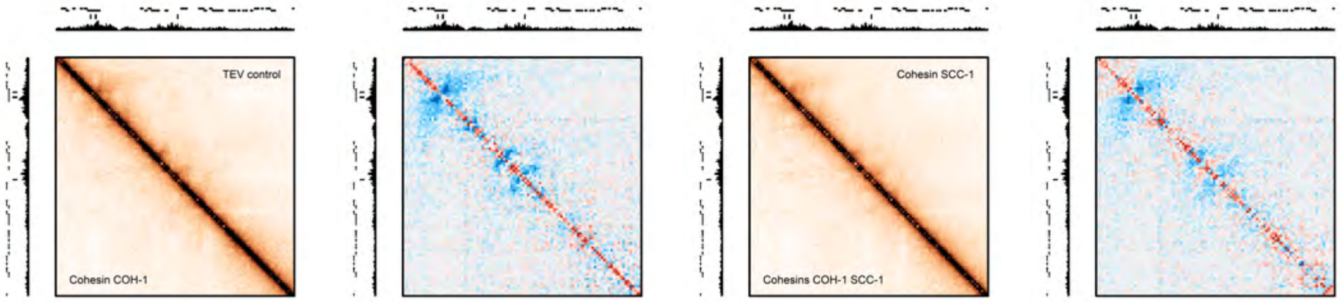

chrX:49001-249000

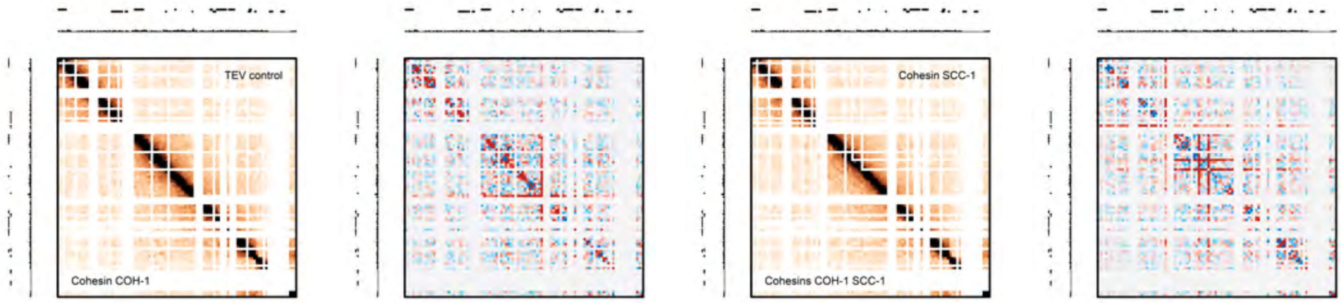

chrII:10901001-11101000

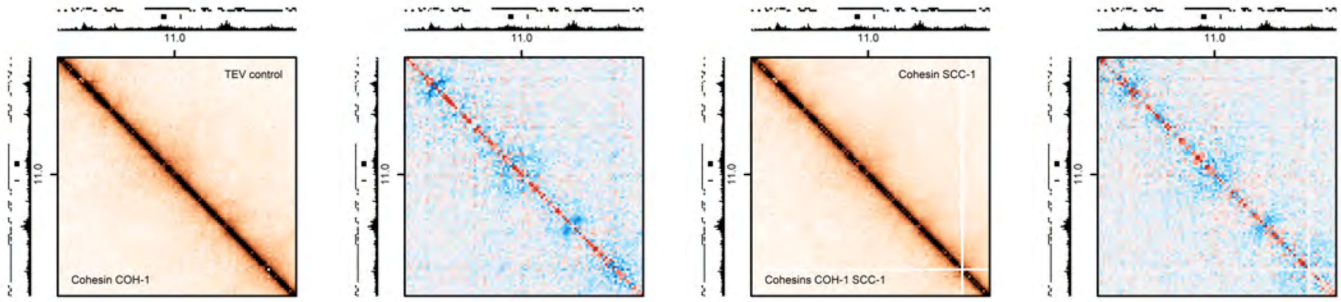

chrX:13151001-13351000

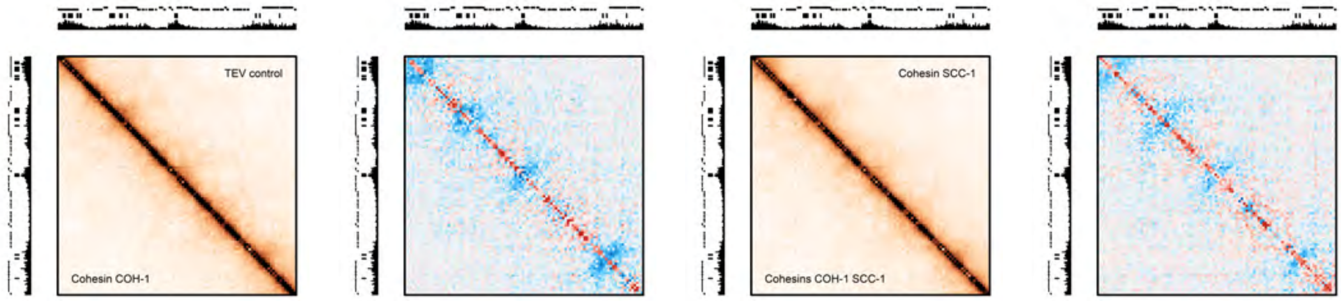

chrX:6421001-6621000

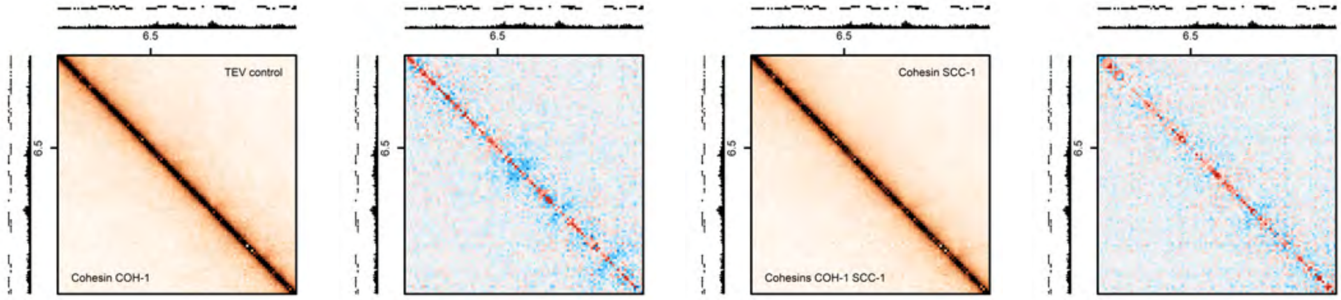

chrX:4711001-4911000

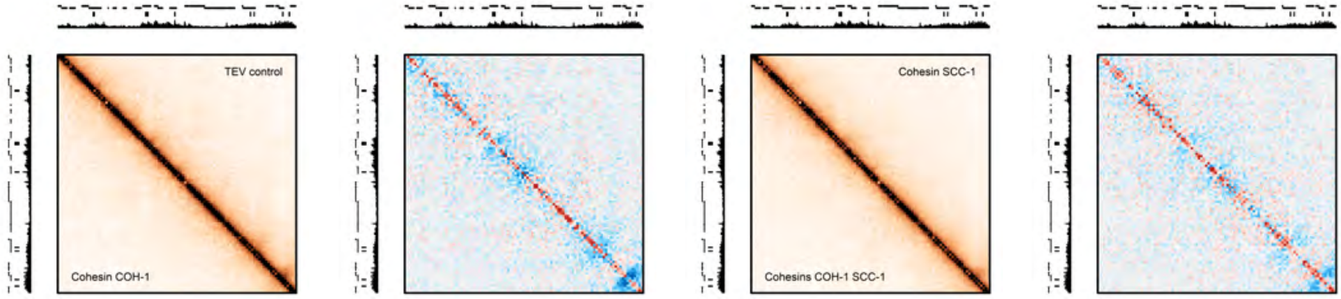

chrIV:8079001-8279000

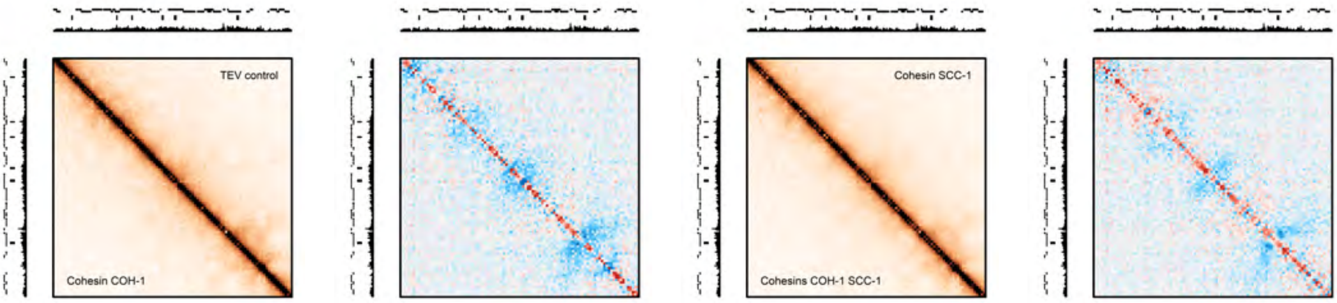

chrII:4293001-4493000

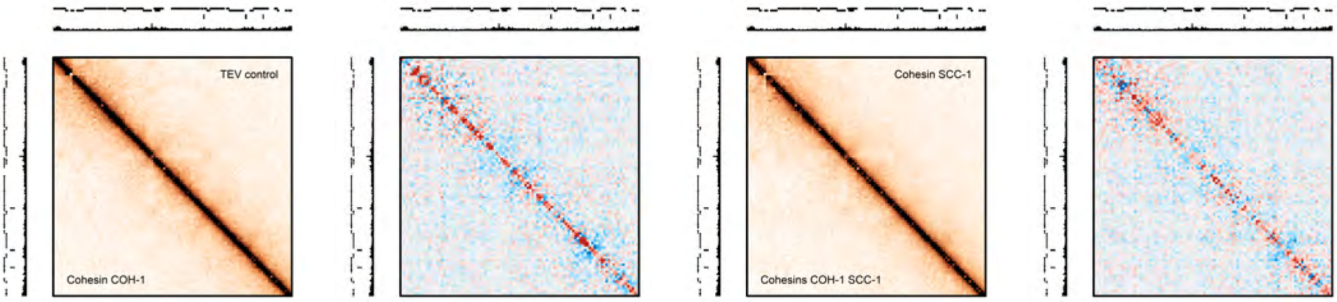

chrIV:10473001-10673000

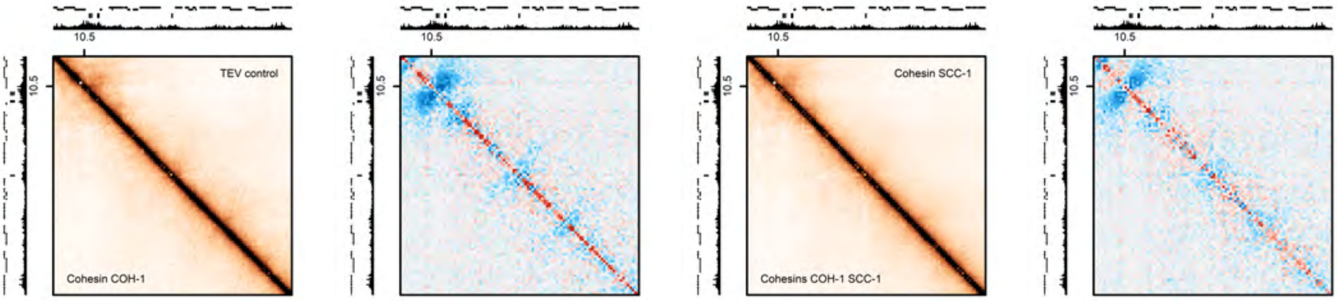

chrII:10829001-11029000

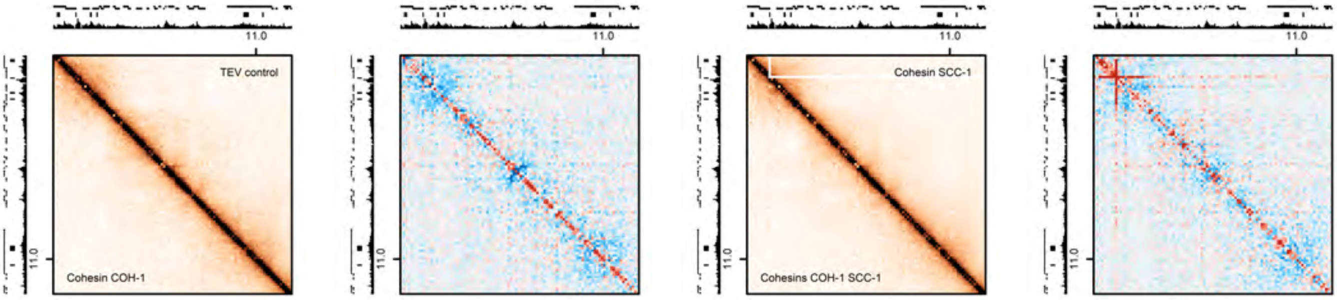

chrI:6185001-6385000

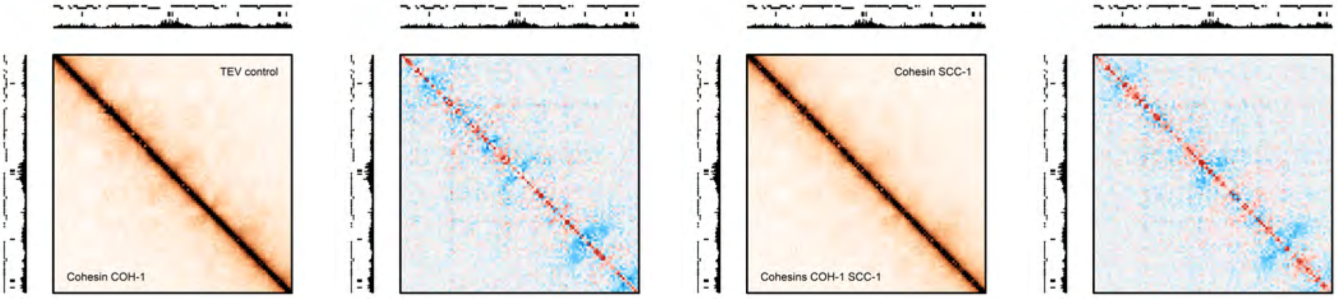

chrV:9351001-9551000

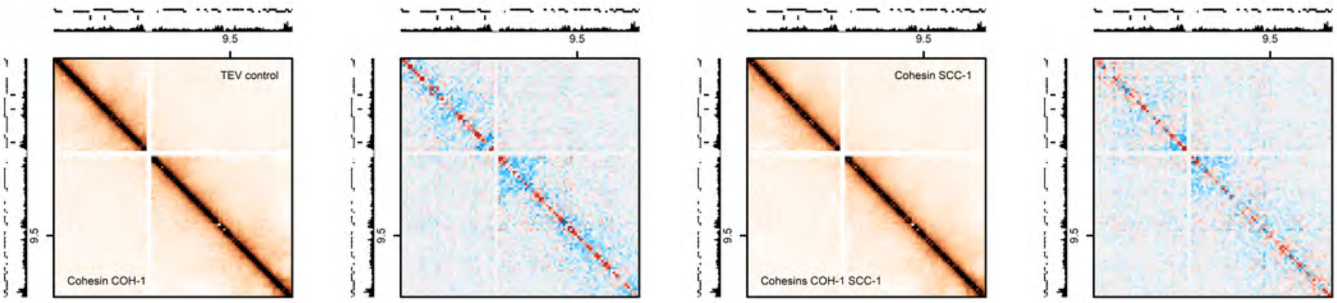

chrIII:6319001-6519000

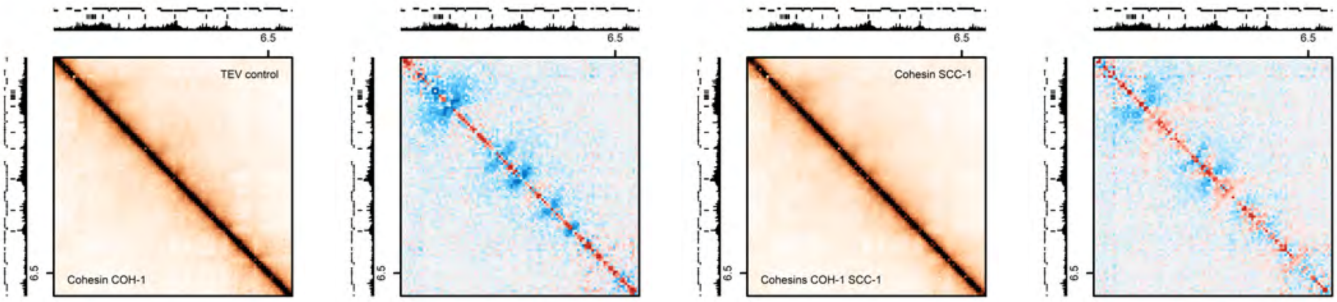

chrI:6277001-6477000

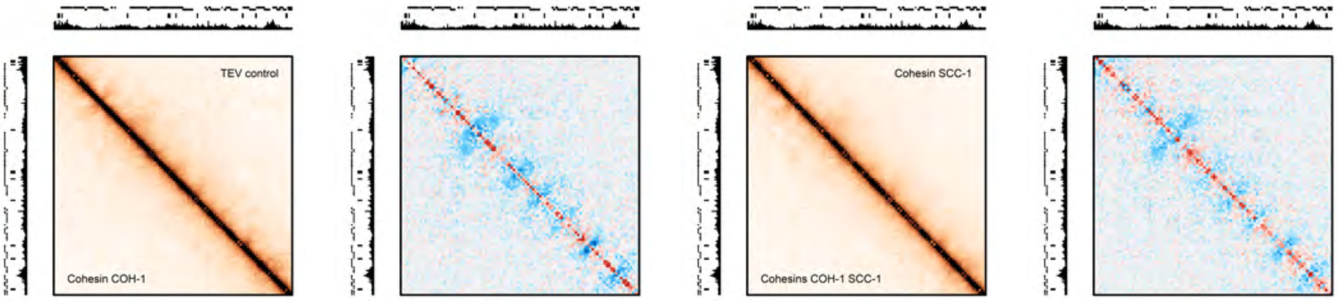

chrIII:8131001-8331000

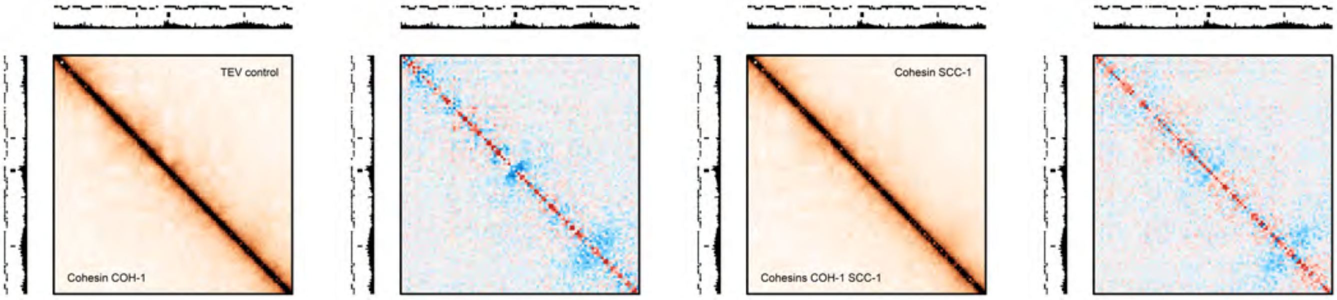

chrV:12715001-12915000

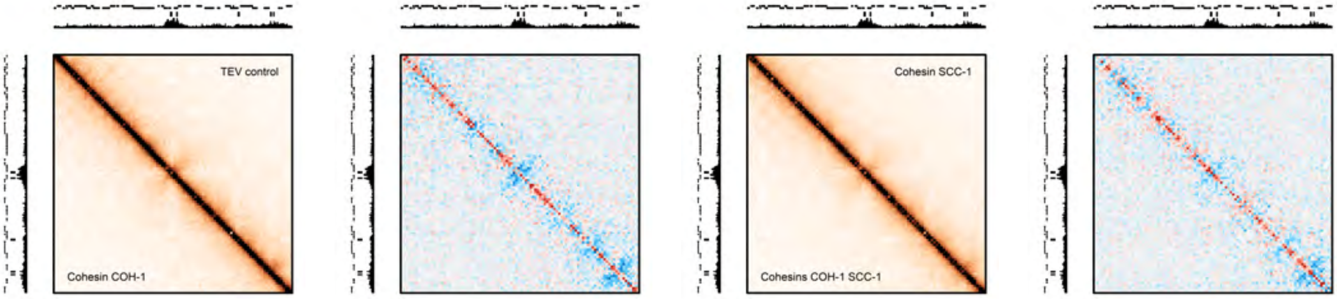

chrII:6079001-6279000

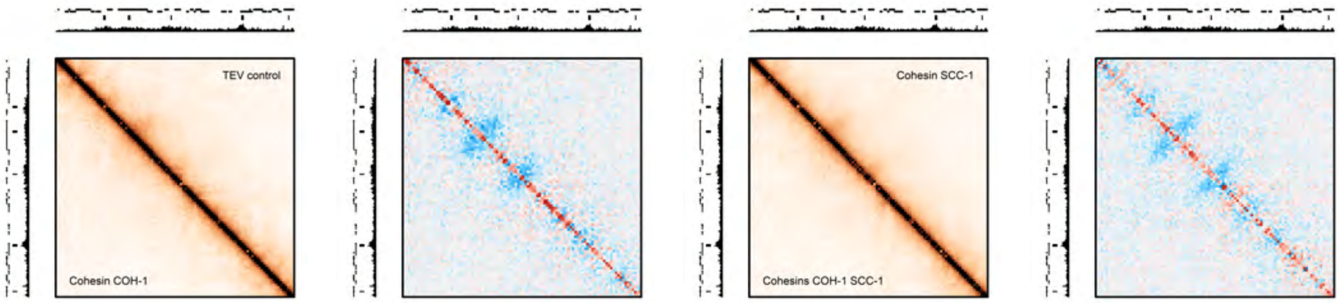

chrIV:1293001-1493000

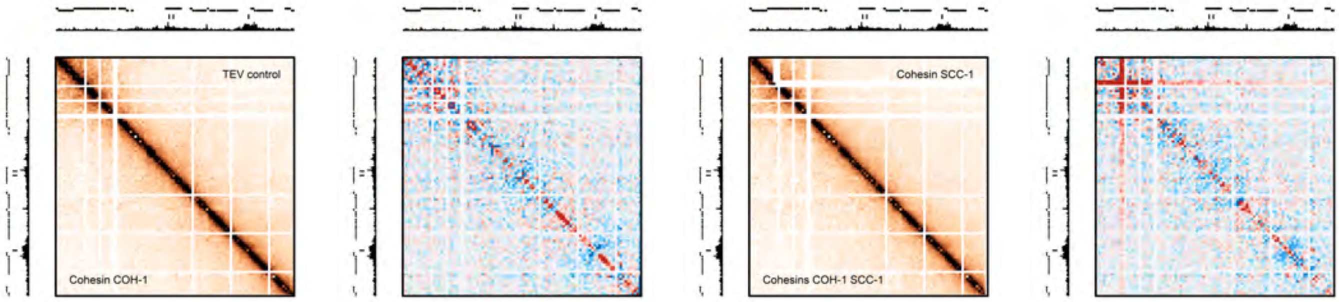

chrIII:-34999-165000

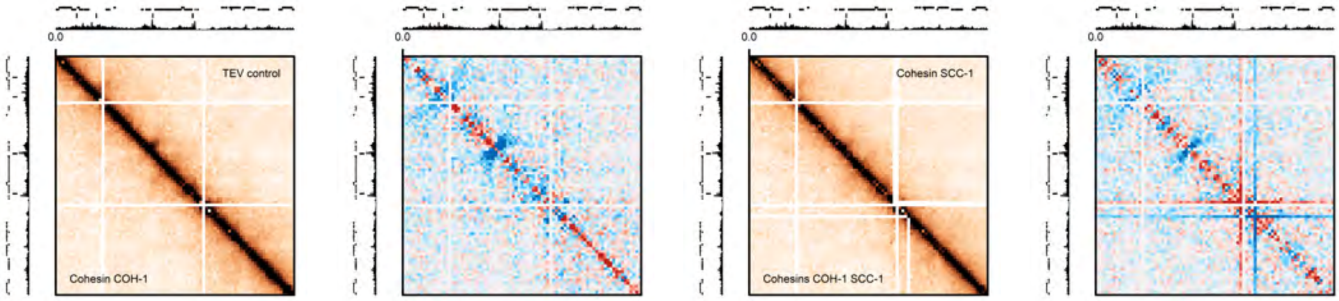

chrIV:11591001-11791000

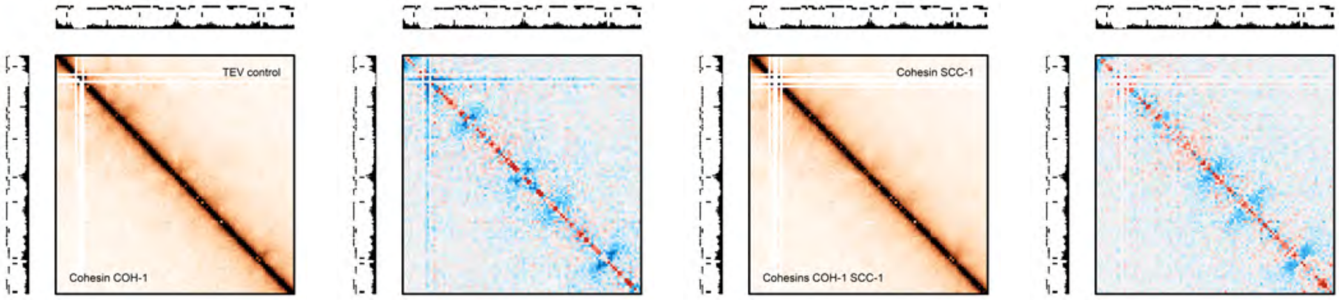

chrI:9069001-9269000

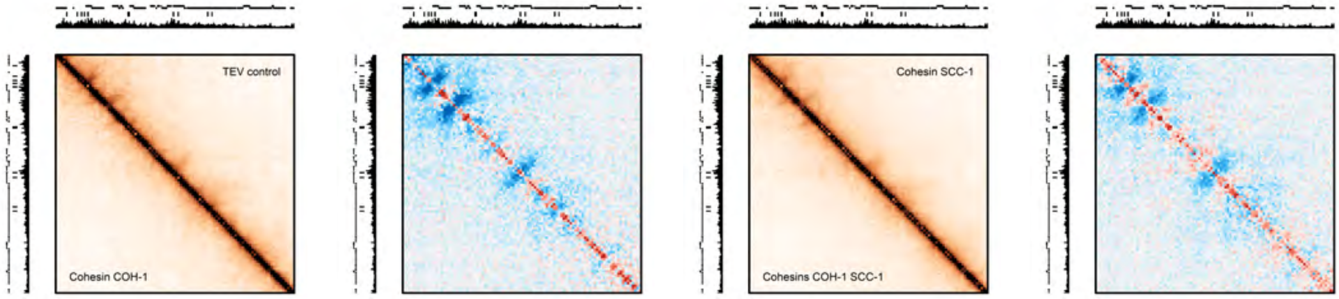

chrX:1505001-1705000

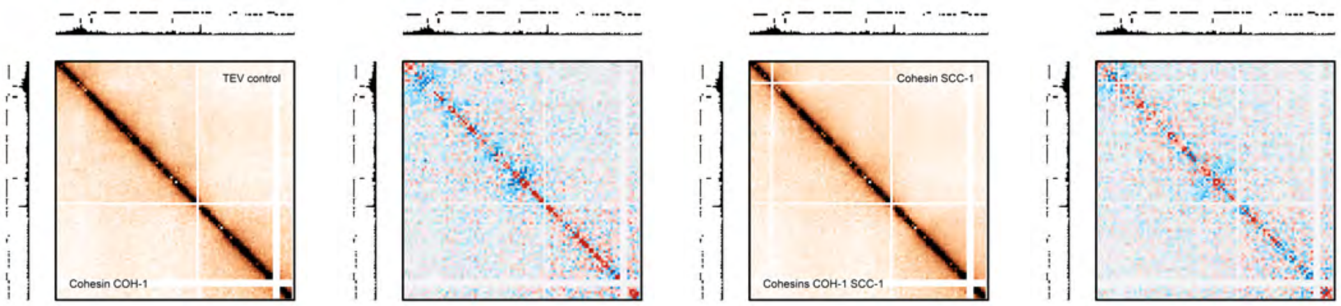

chrV:15087001-15287000

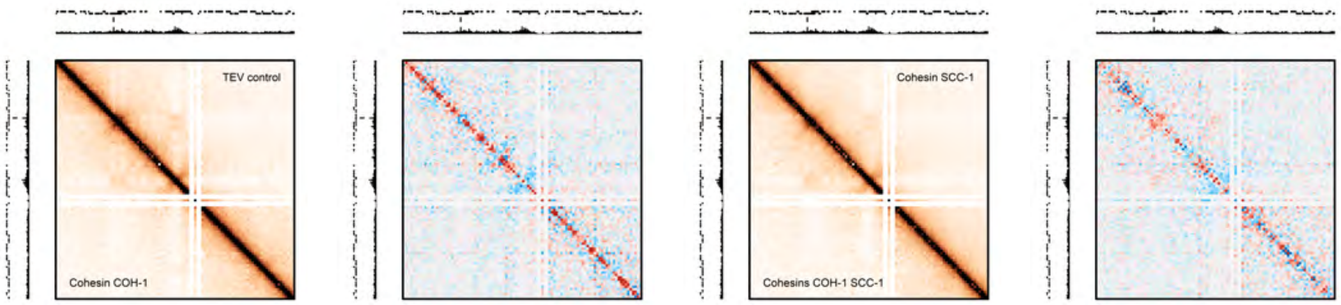

chrIV:1051001-1251000

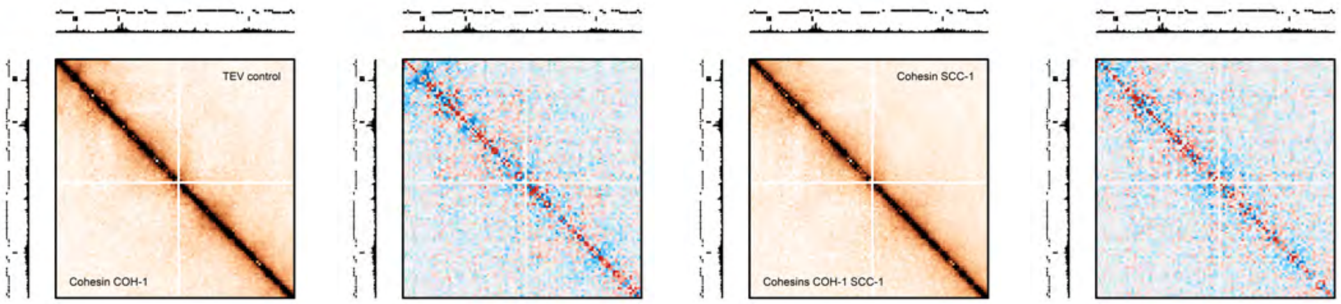

chrI:10133001-10333000

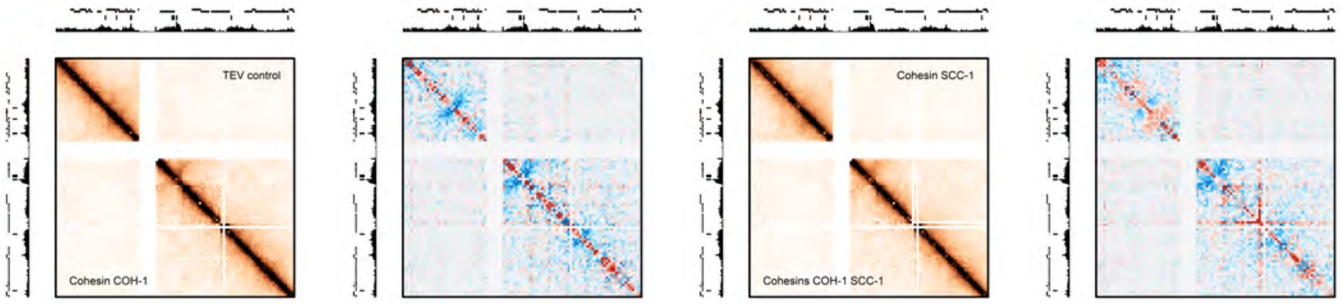

chrI:1169001-1369000

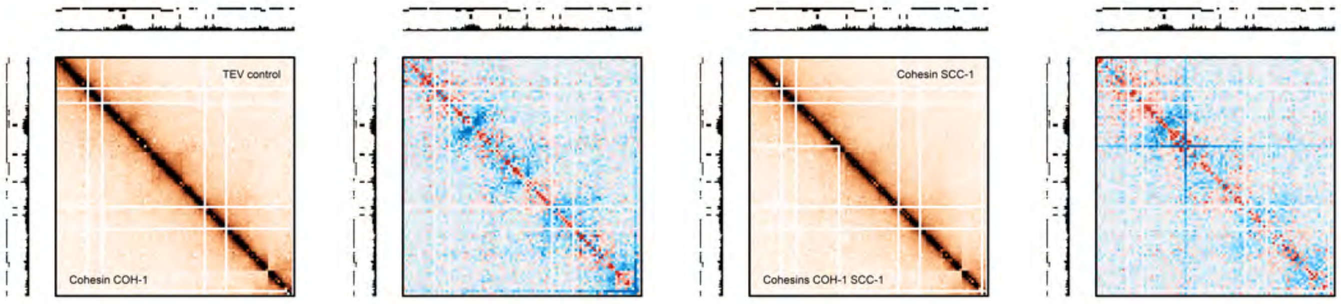

chrII:8545001-8745000

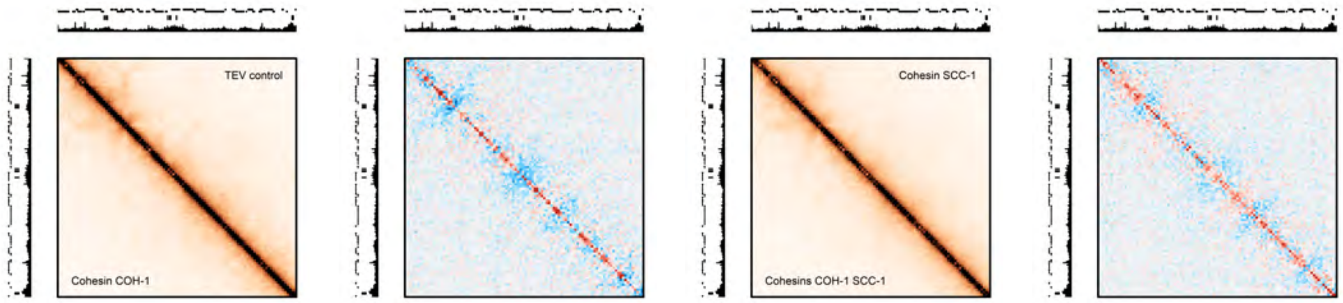

chrIV:6993001-7193000

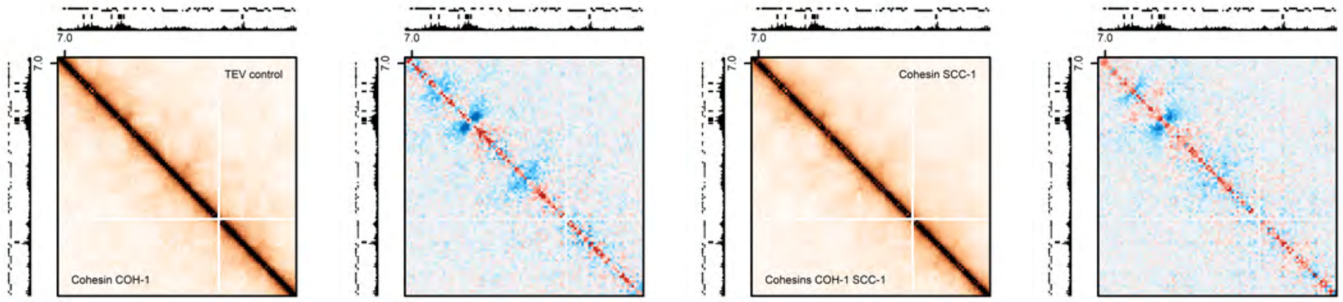

chrIII:12915001-13115000

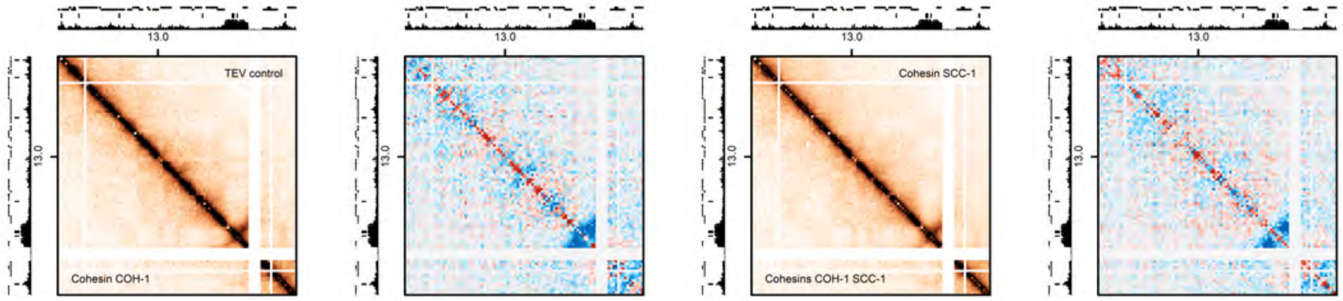

chrX:1975001-2175000

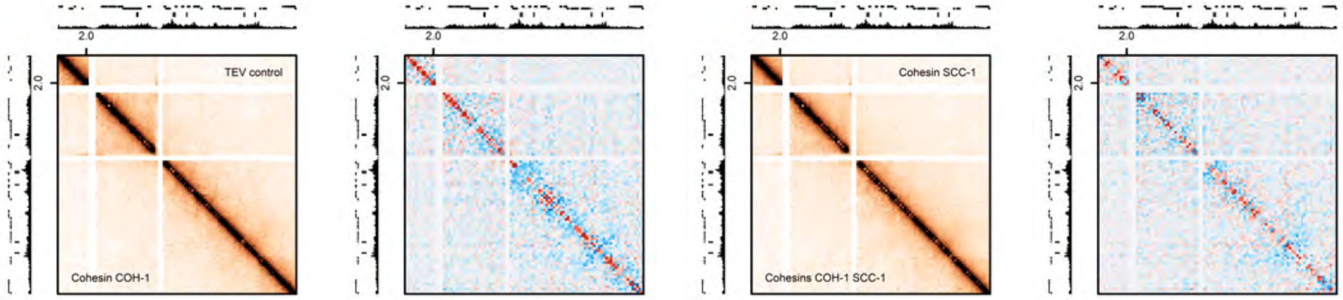

chrX:495001-695000

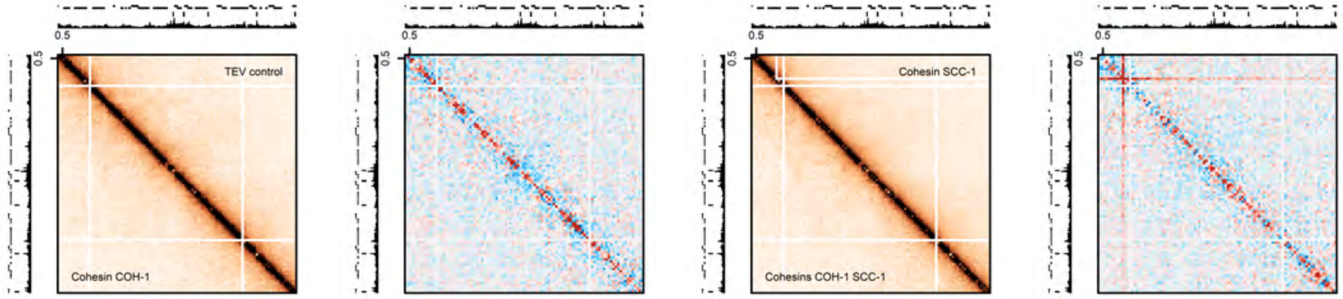

chrX:14195001-14395000

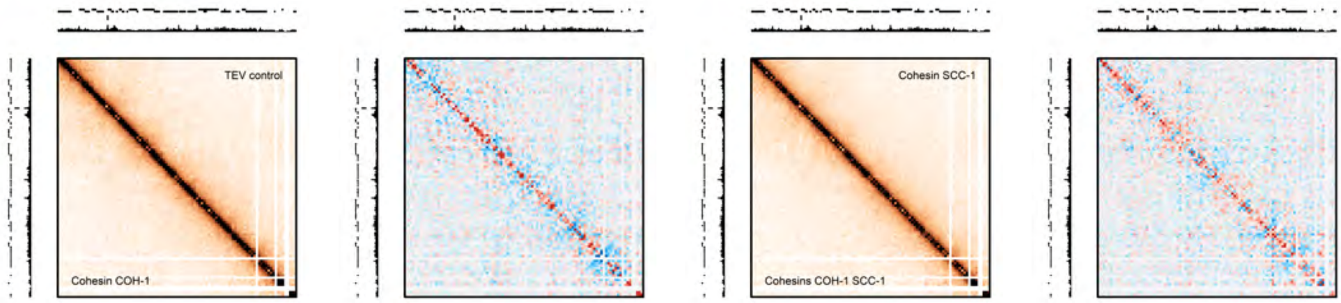

chrIII:4697001-4897000

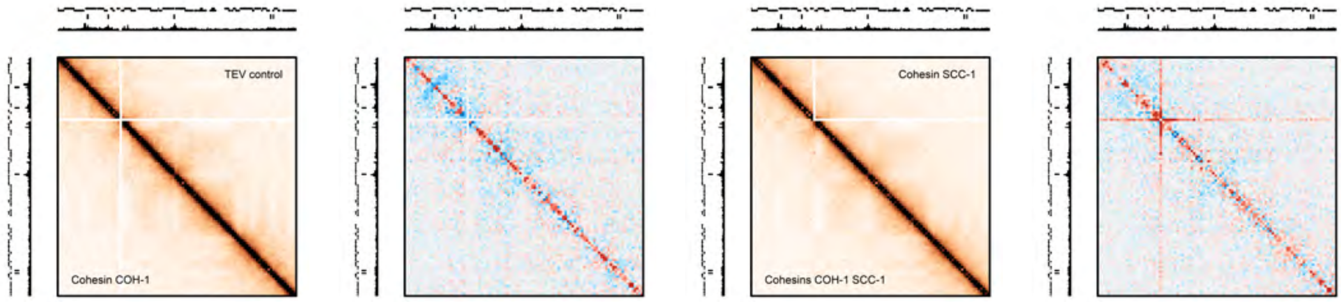

chrX:223001-423000

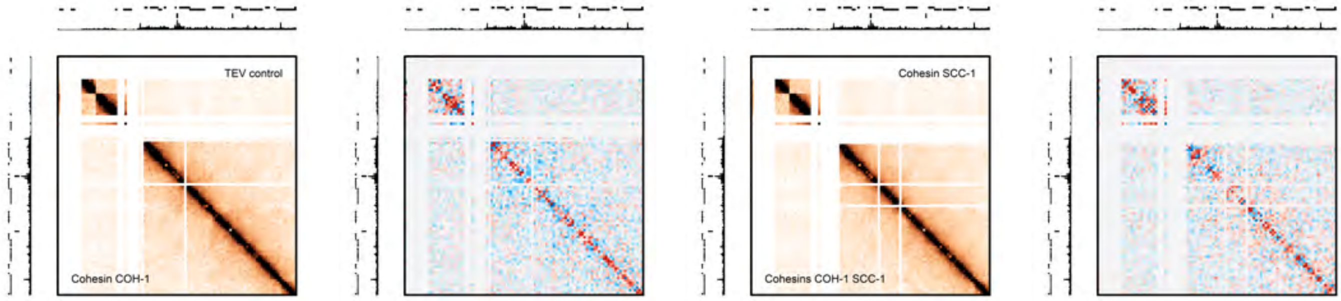

chrIII:12377001-12577000

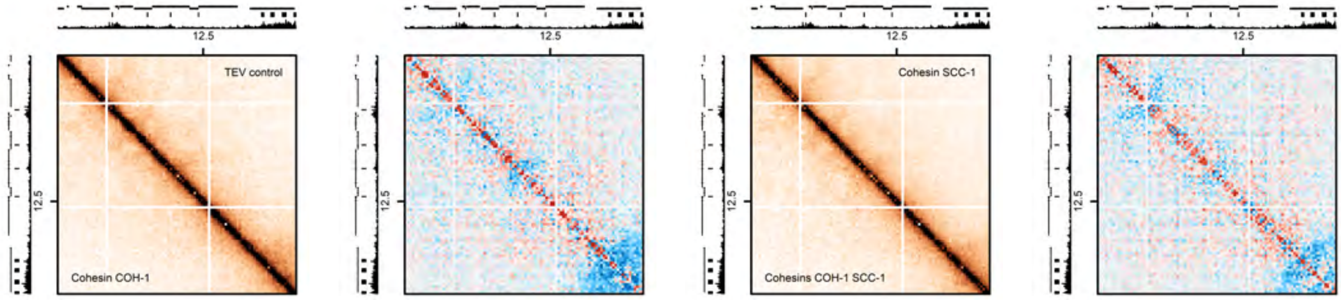

chrV:5201001-5401000

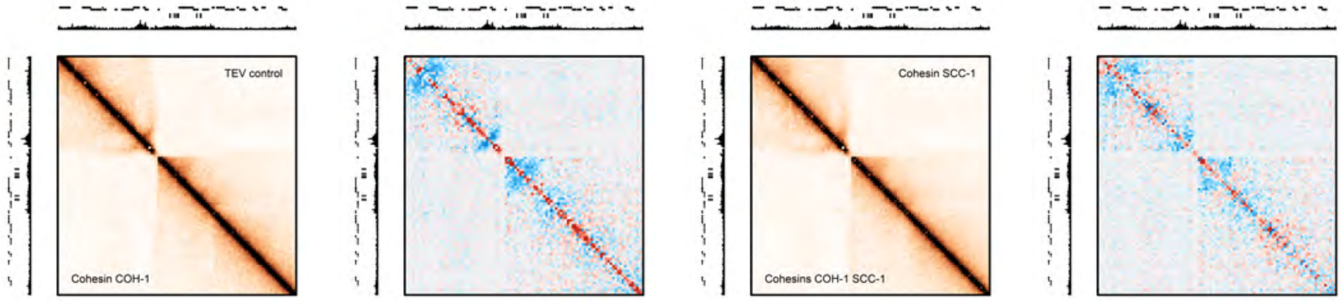

chrIII:1231001-1431000

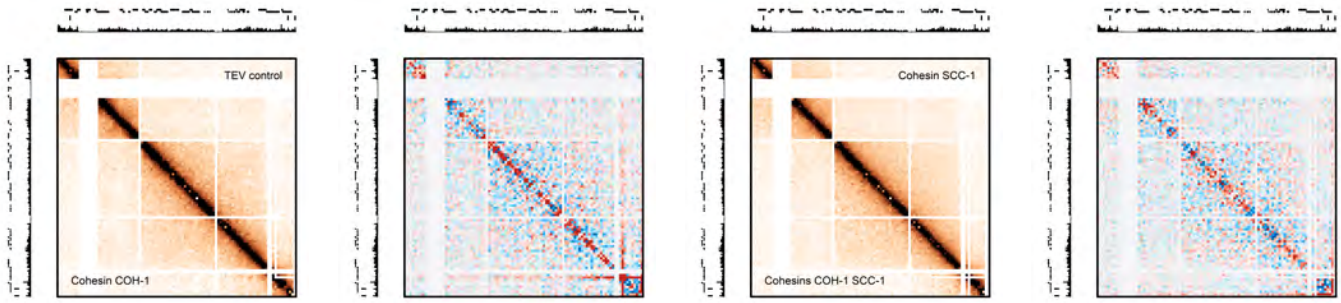

chrV:11789001-11989000

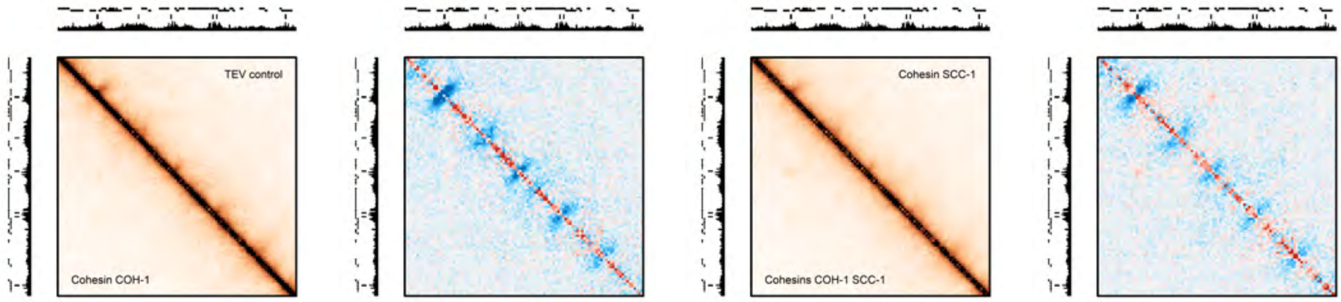

chrII:5473001-5673000

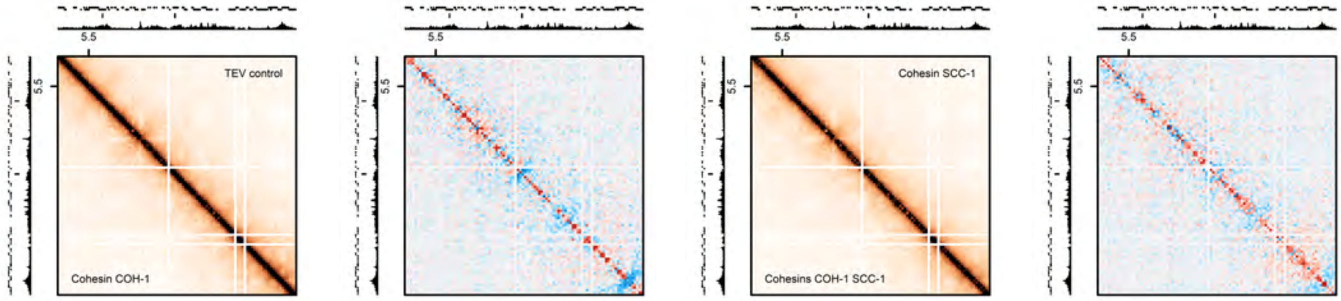

chrII:2953001-3153000

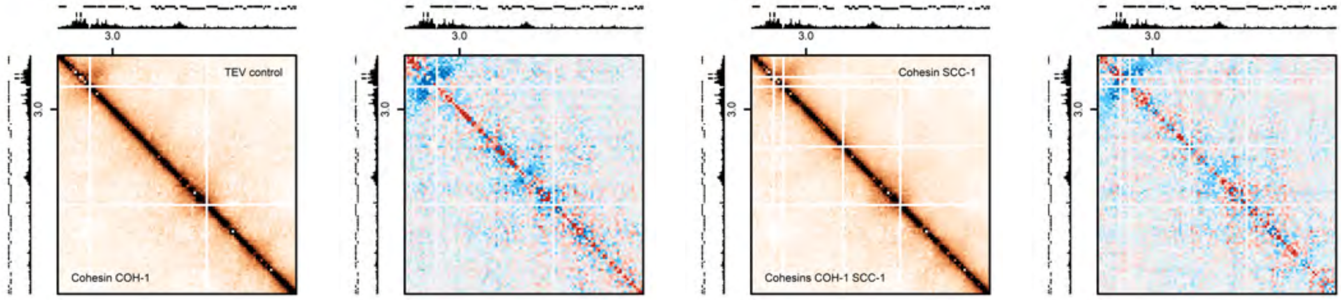

chrX:8621001-8821000

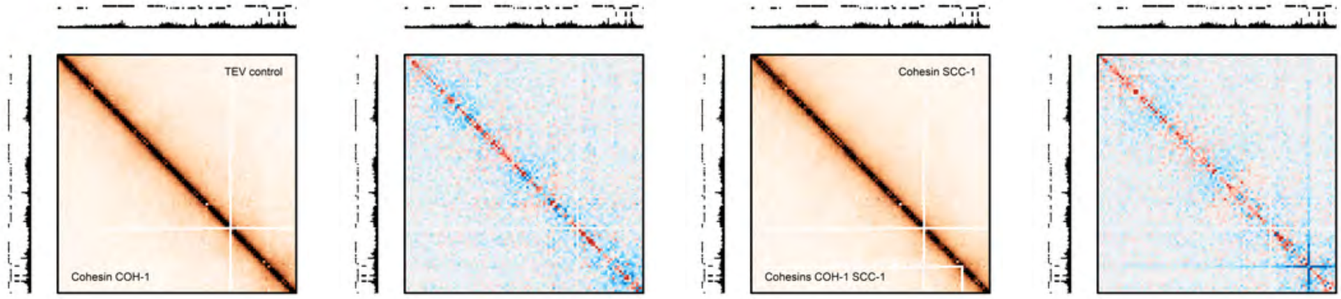

chrX:17379001-17579000

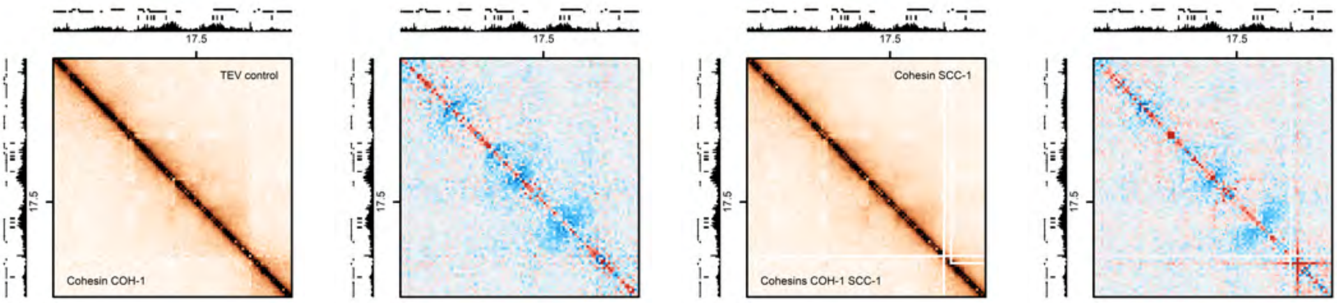

chrV:1897001-2097000

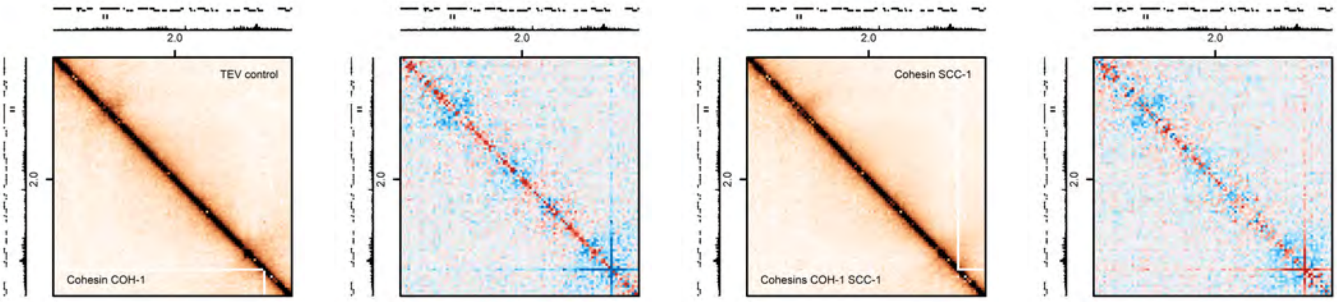

chrII:6391001-6591000

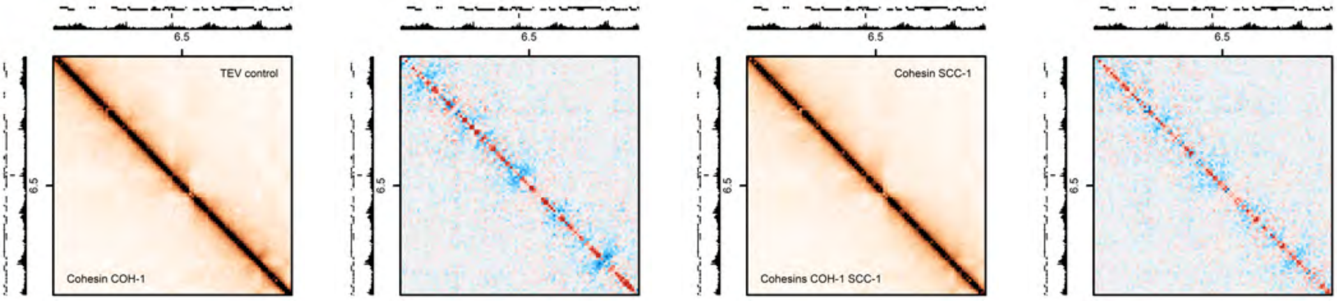

chrIII:4229001-4429000

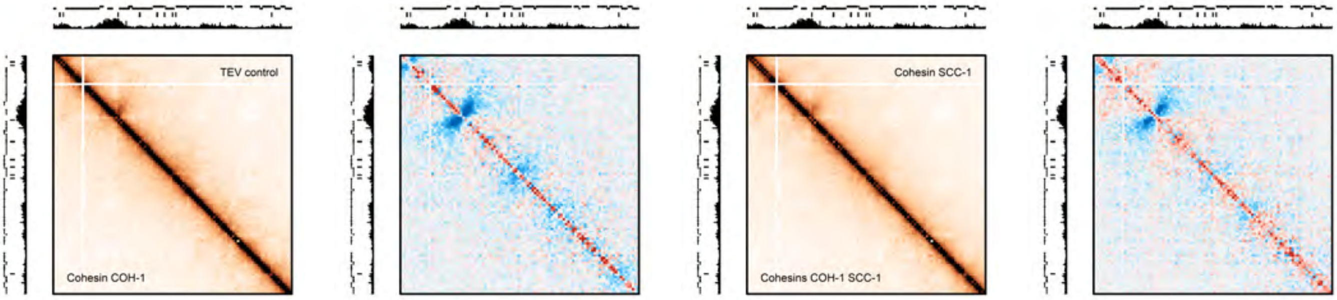

chrV:10233001-10433000

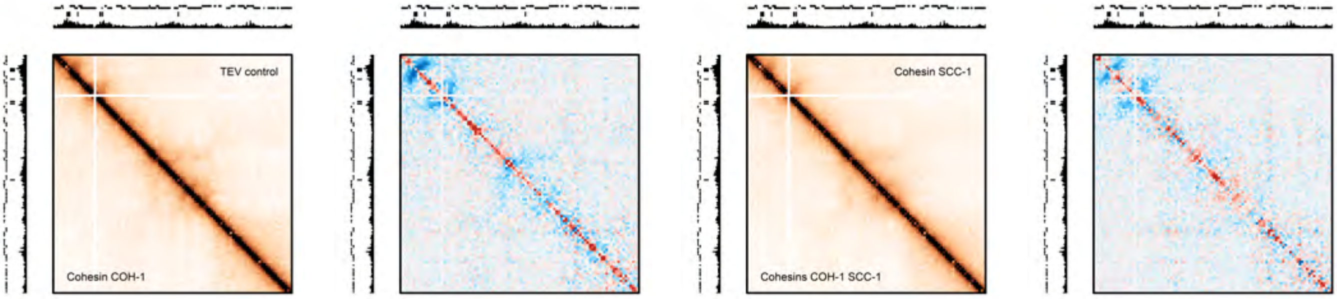

chrIV:10305001-10505000

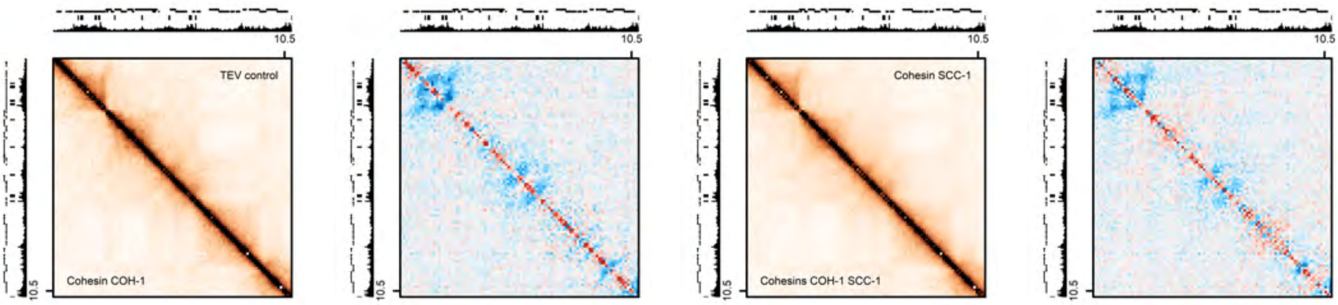

chrI:6359001-6559000

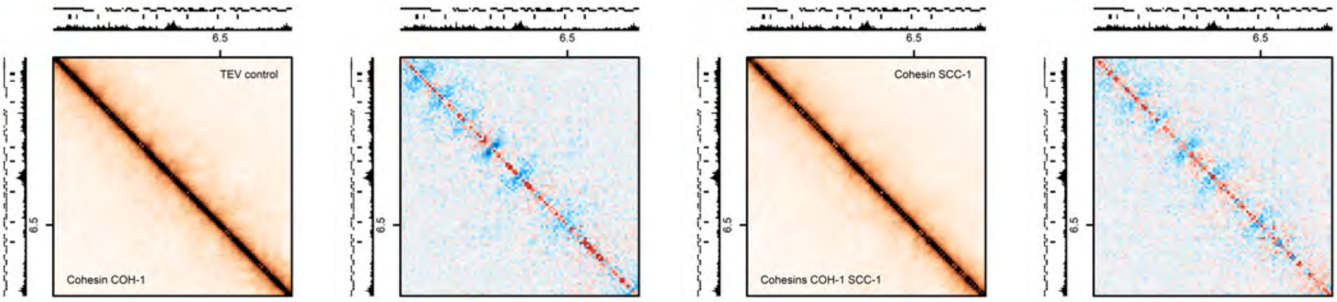

chrI:13085001-13285000

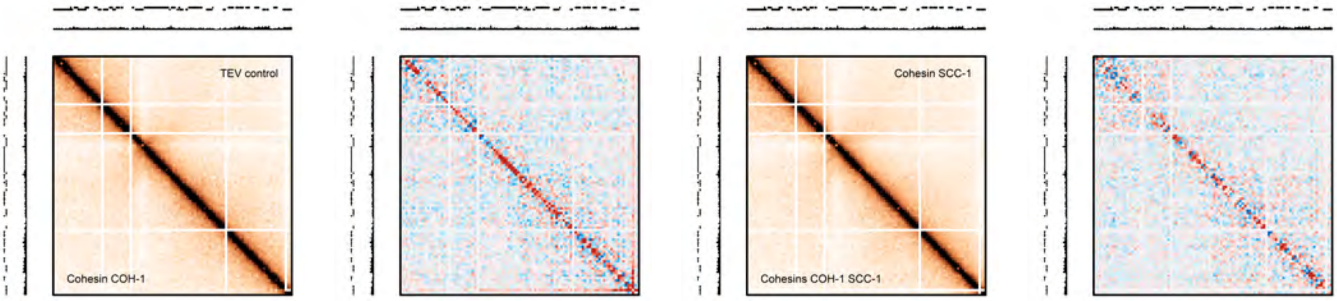

chrII:8241001-8441000

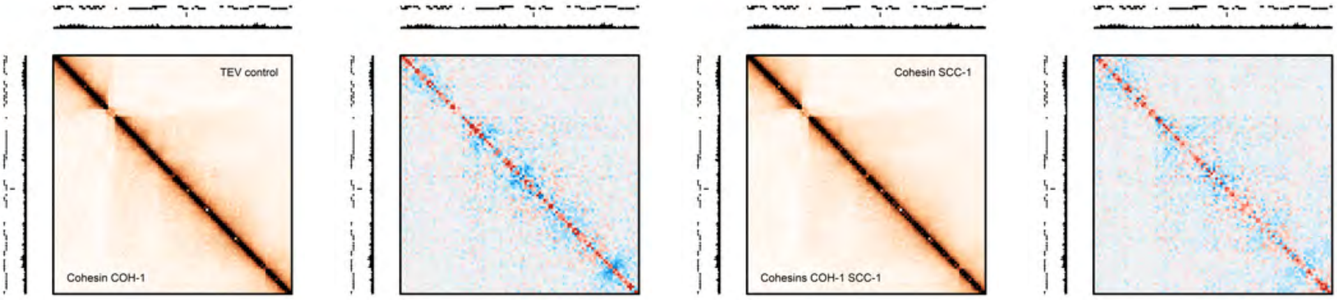

chrX:2605001-2805000

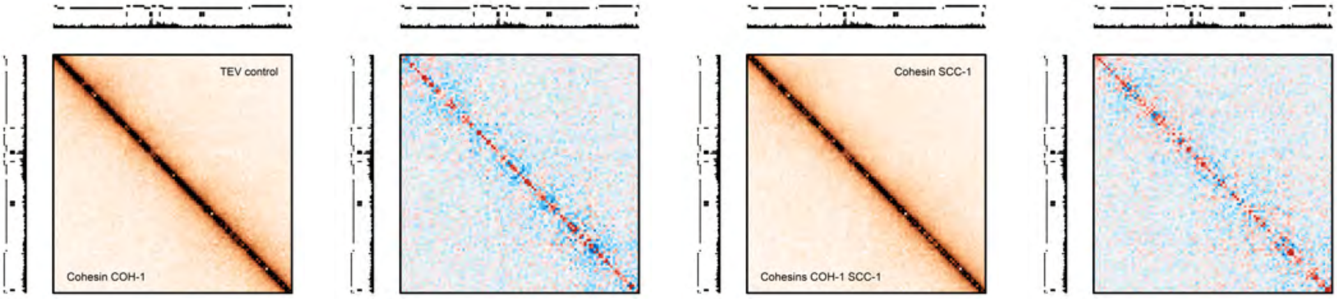

chrX:401001-601000

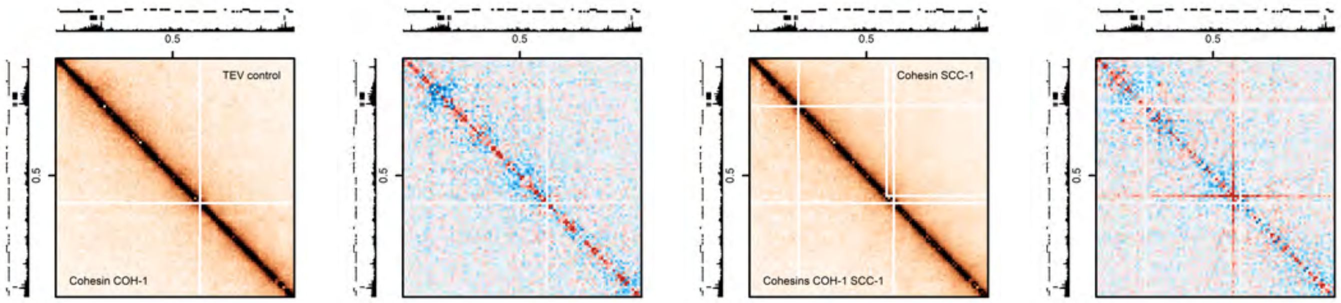

chrIV:5959001-6159000

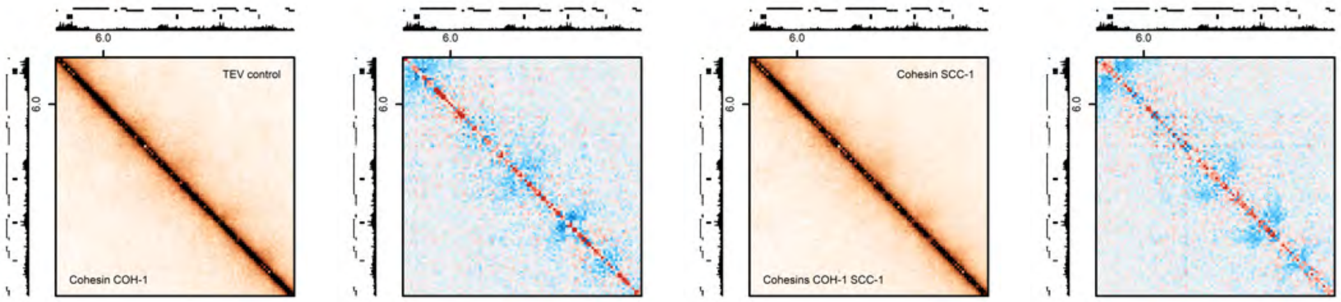

chrIV:13605001-13805000

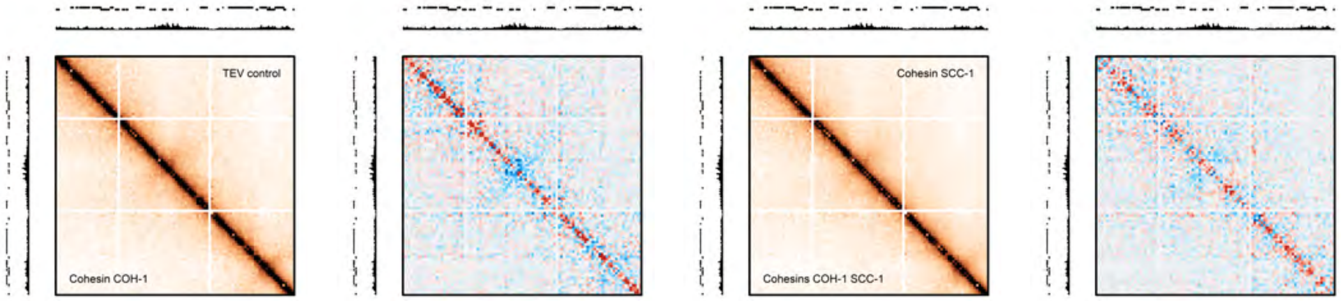

chrX:10295001-10495000

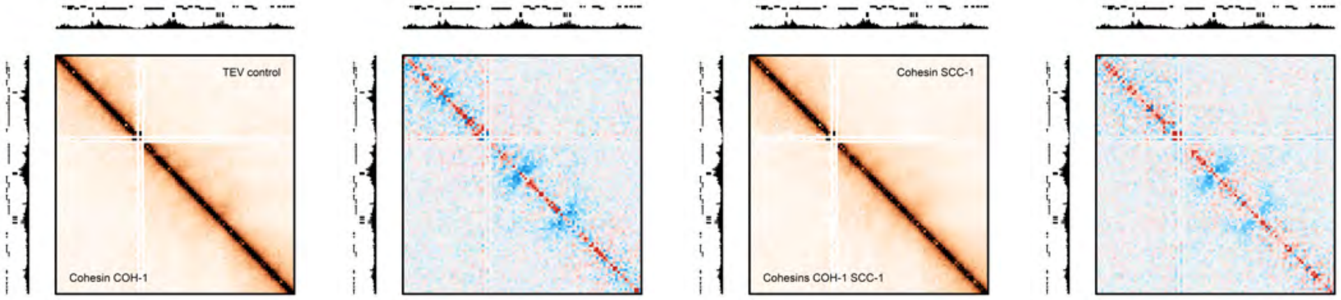

chrIV:1437001-1637000

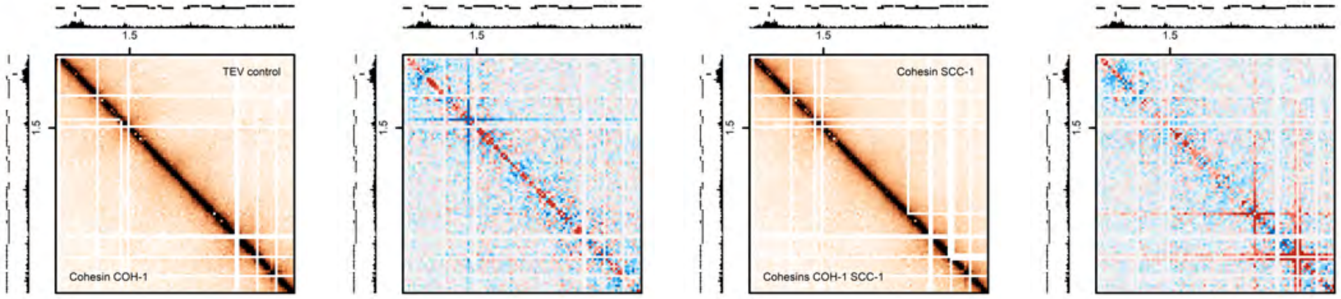

chrII:7435001-7635000

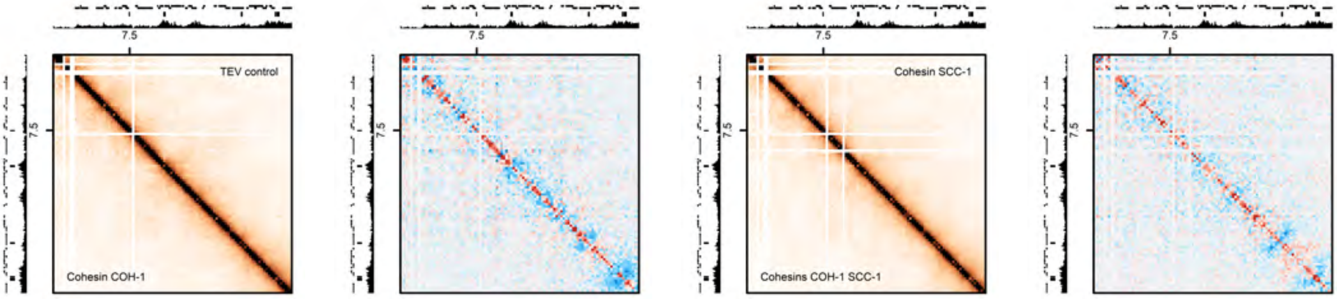

chrI:1981001-2181000

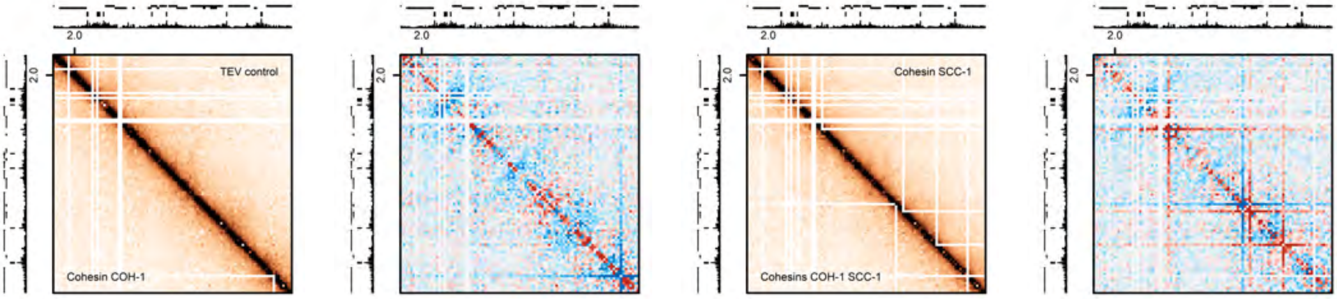

chrIII:6649001-6849000

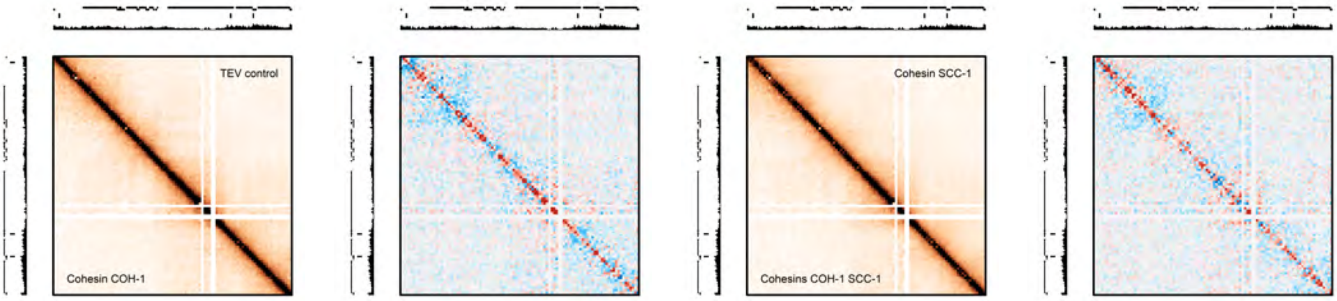

chrIII:1011001-1211000

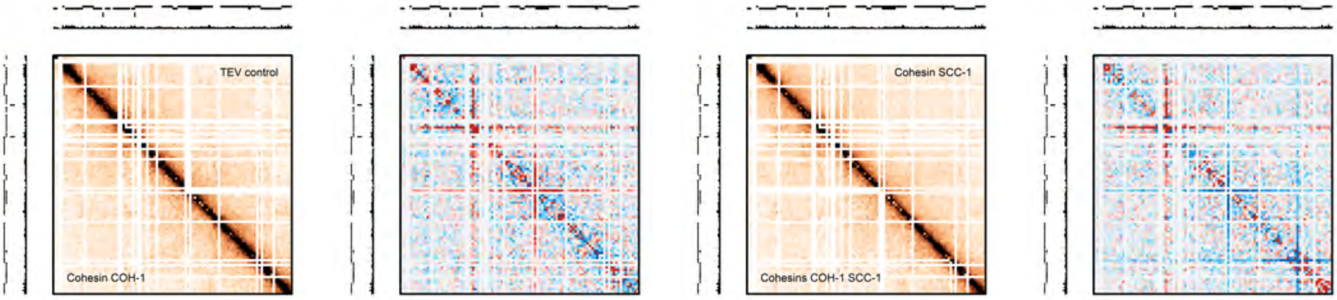

chrIII:2317001-2517000

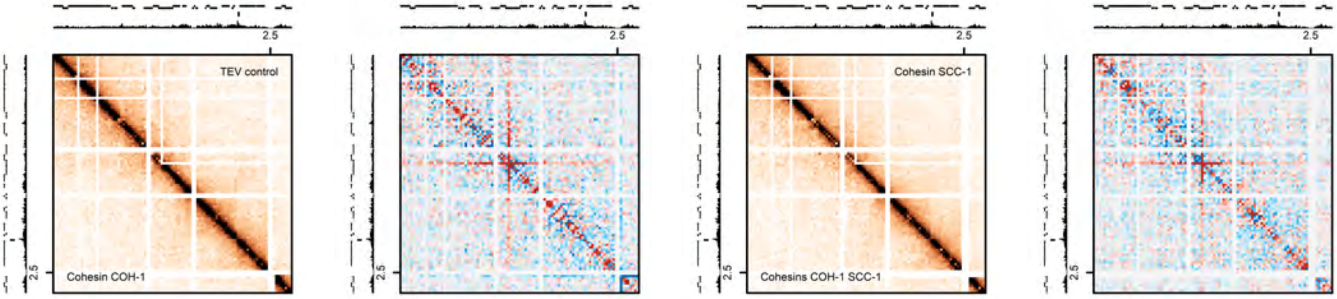

chrX:11457001-11657000

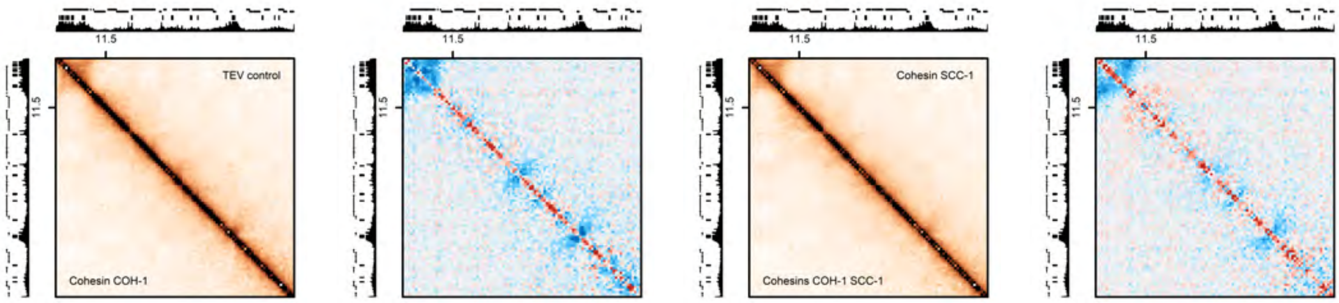

chrII:12601001-12801000

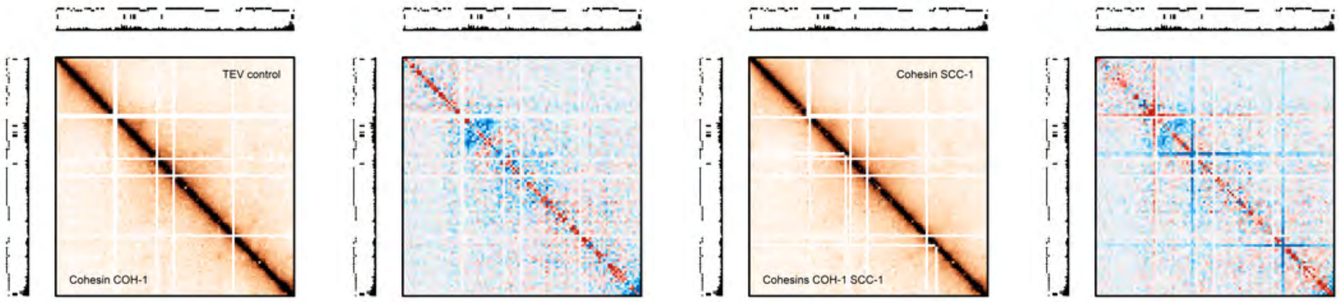

chrIII:11839001-12039000

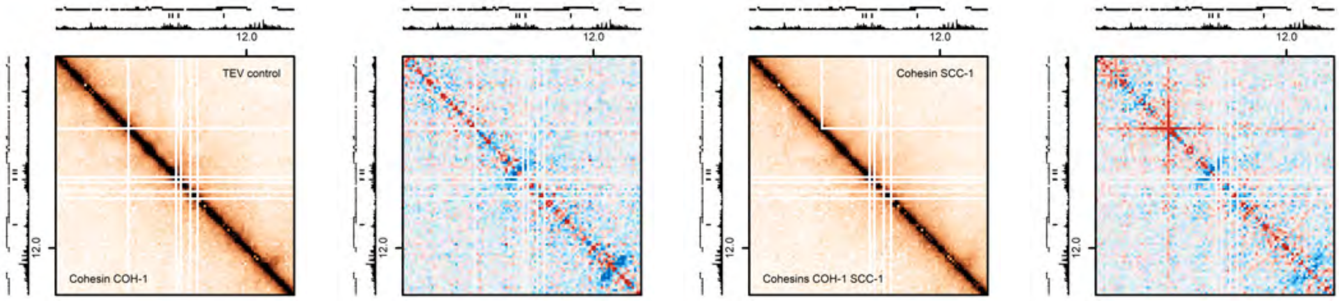

chrV:11213001-11413000

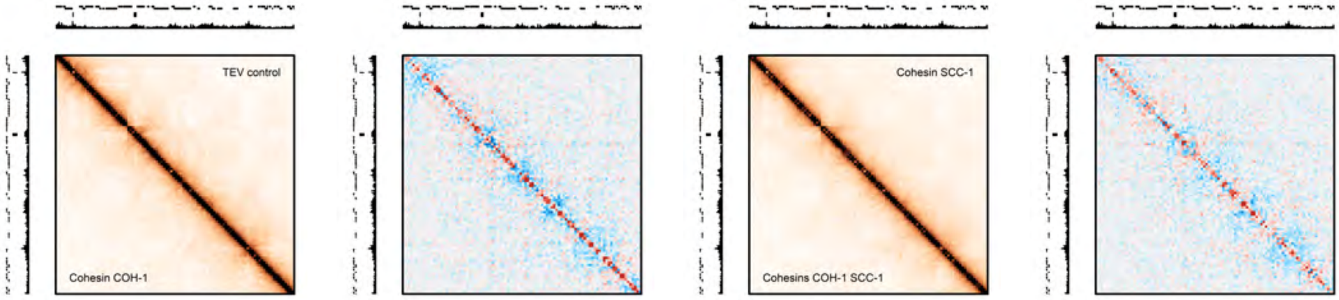

chrI:4877001-5077000

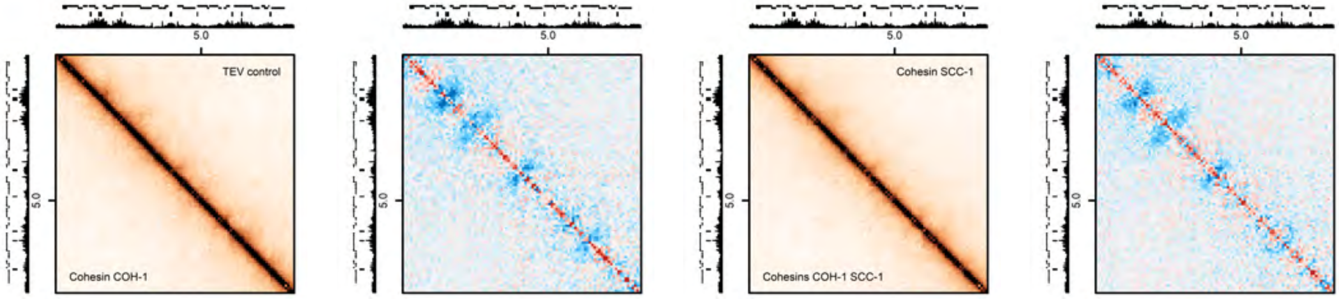

chrV:8639001-8839000

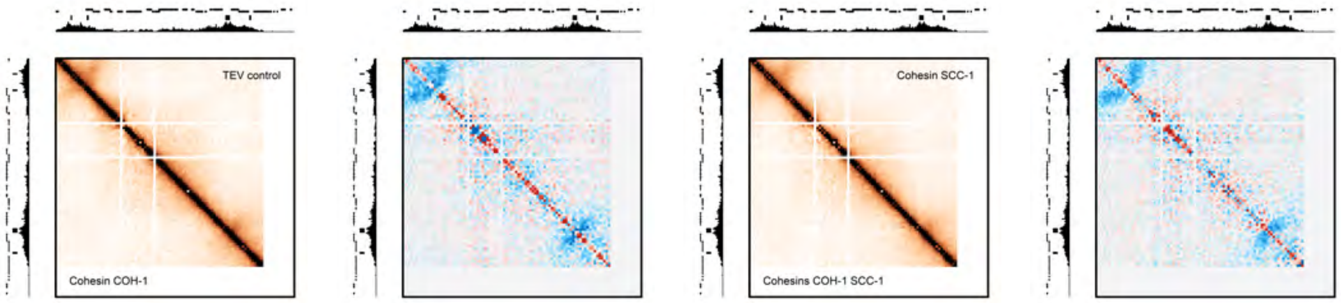

chrII:13803001-14003000

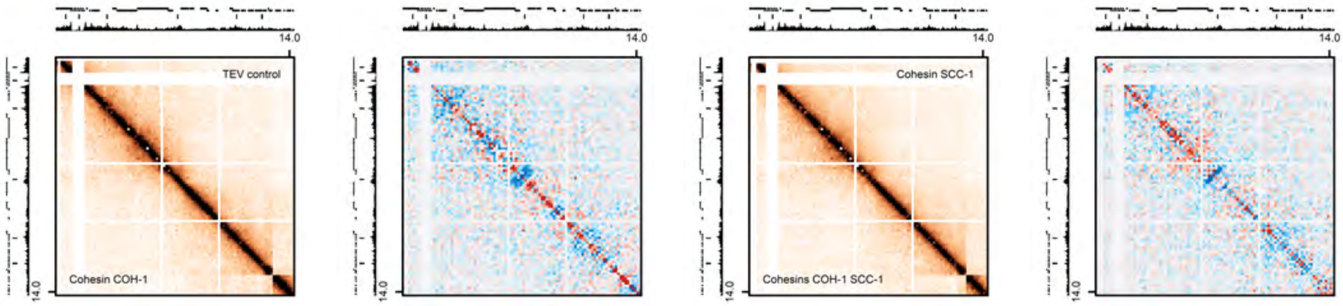

chrIII:11145001-11345000

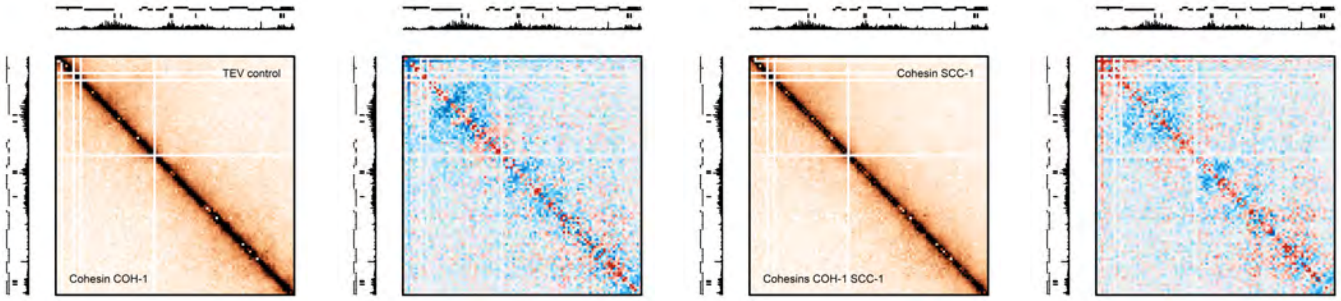

chrV:7879001-8079000

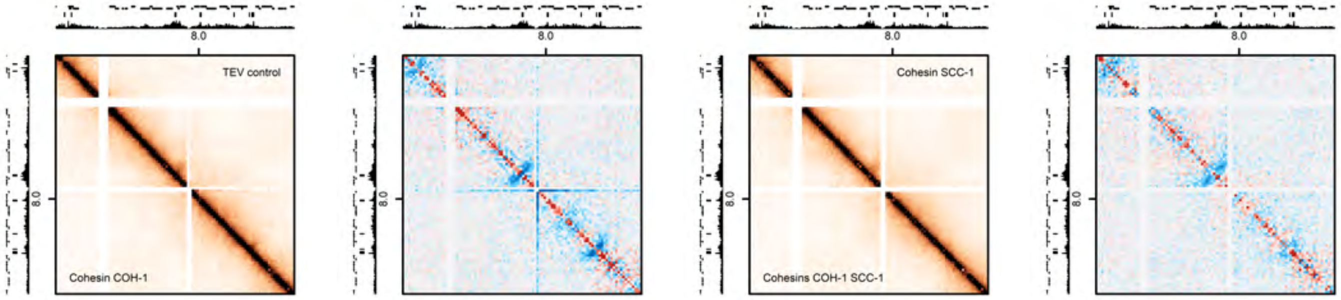

chrII:10583001-10783000

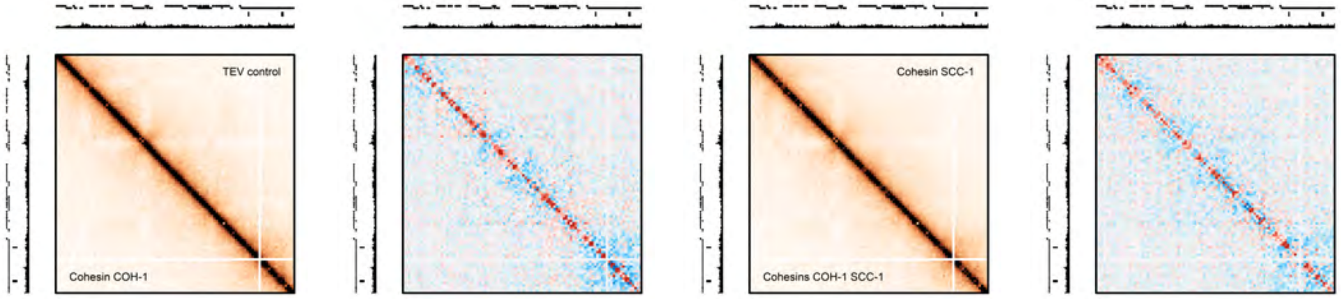

chrI:13221001-13421000

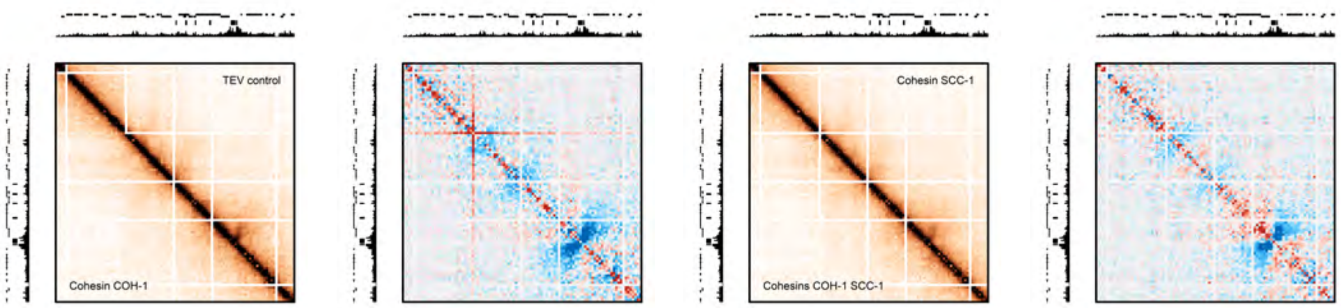

chrV:7445001-7645000

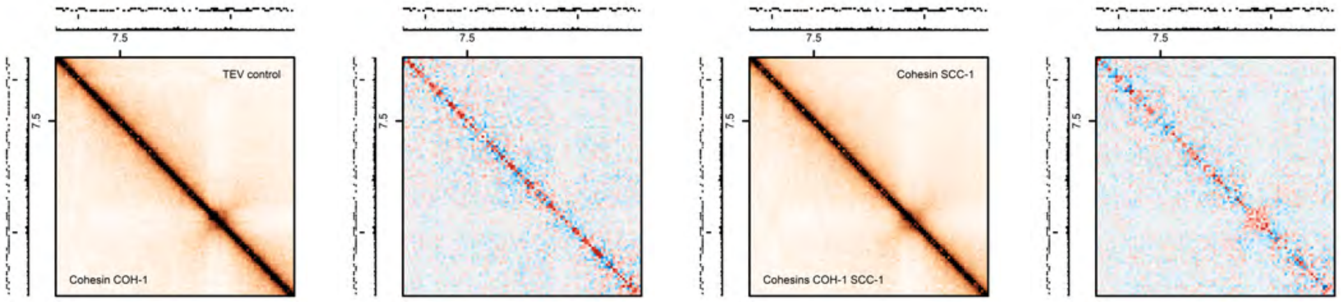

chrIV:14739001-14939000

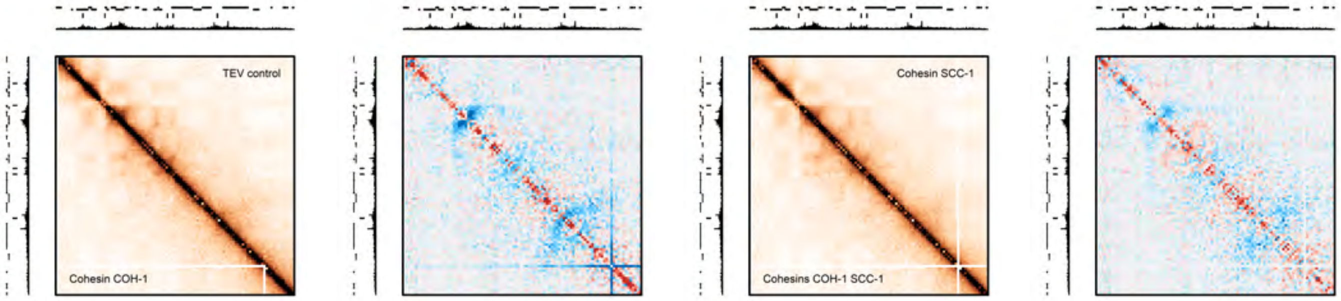

chrIV:5343001-5543000

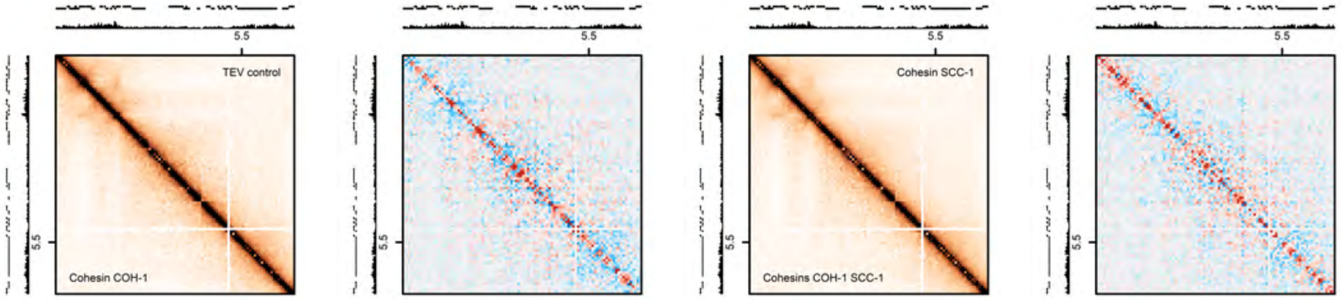

chrX:9983001-10183000

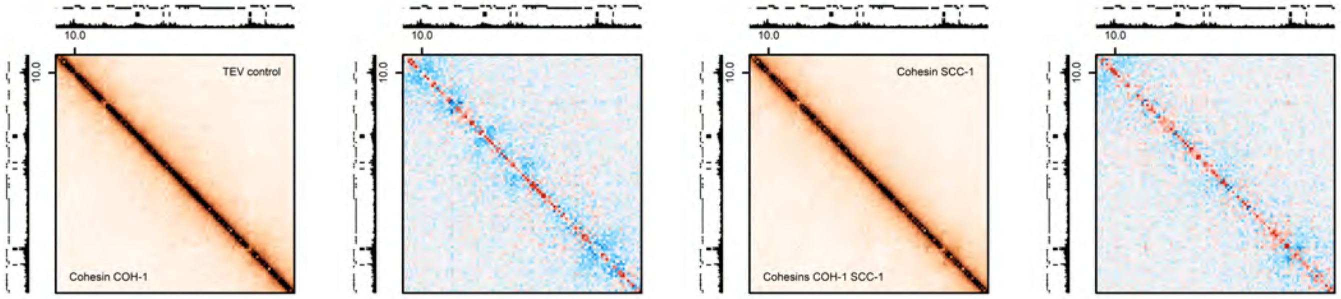

chrIII:201001-401000

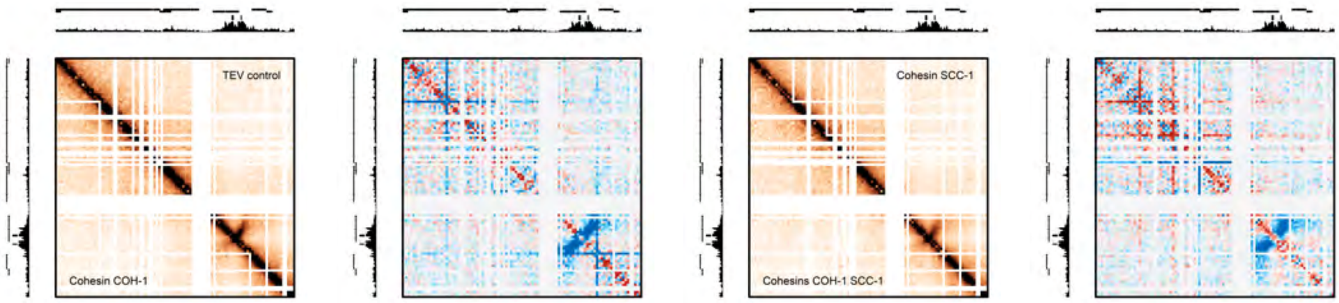

chrII:4095001-4295000

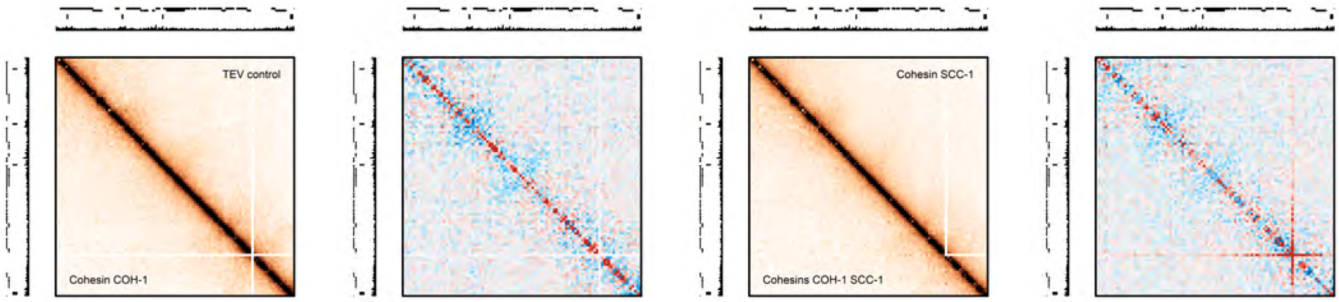

chrX:5249001-5449000

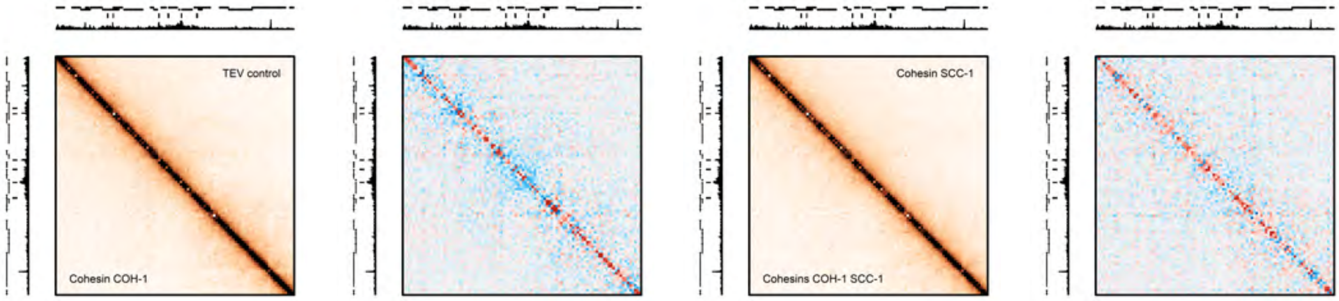

chrII:9323001-9523000

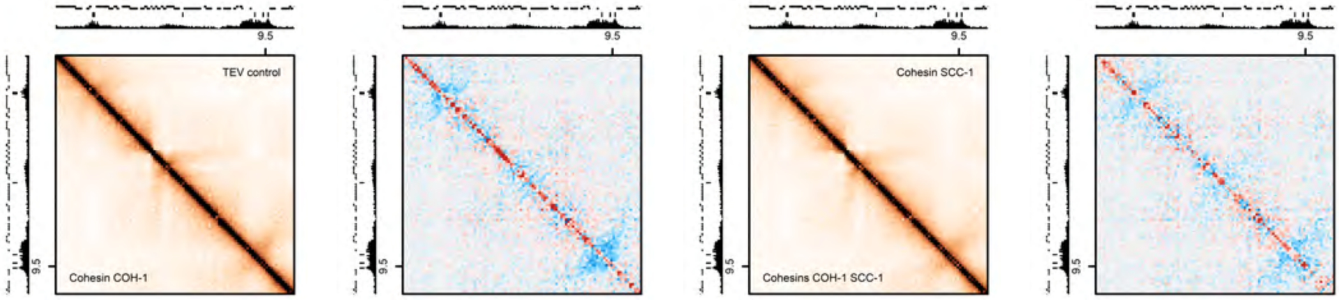

chrV:17903001-18103000

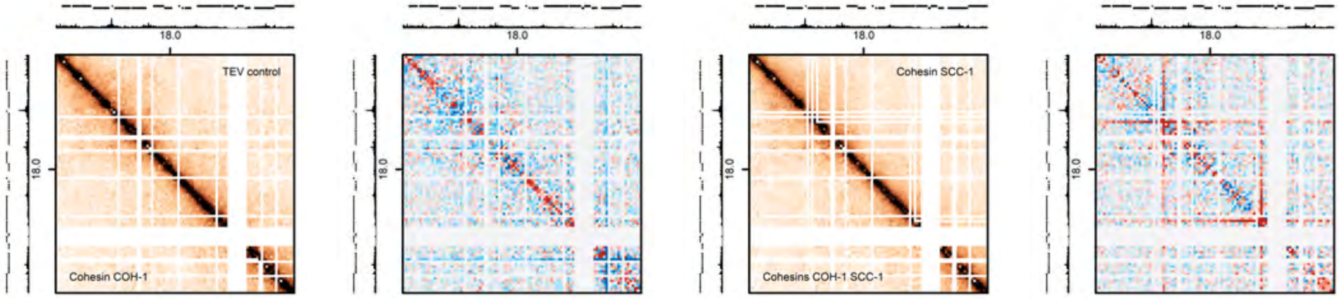

chrIII:2813001-3013000

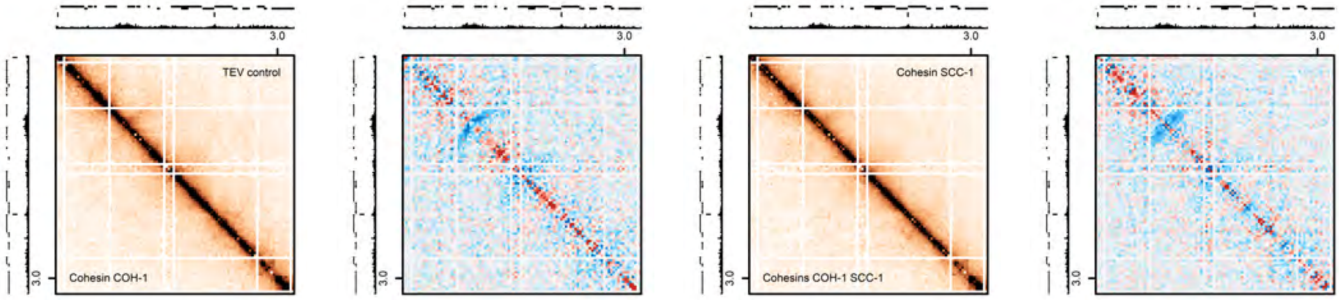

chrV:17655001-17855000

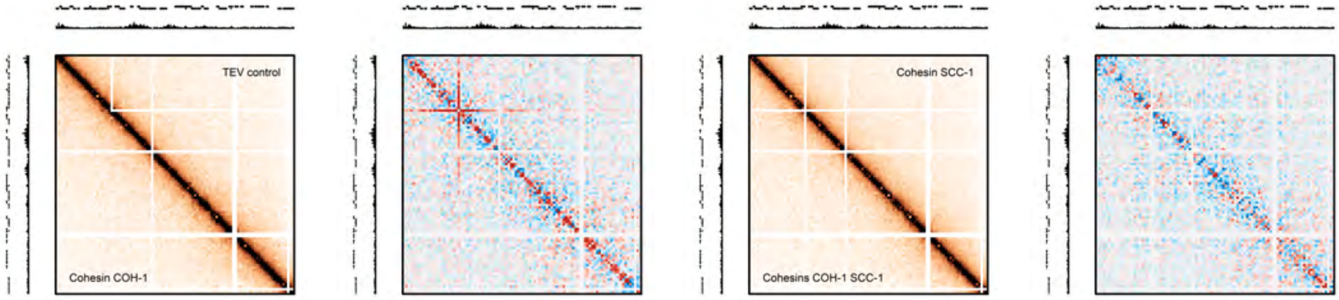

chrX:13105001-13305000

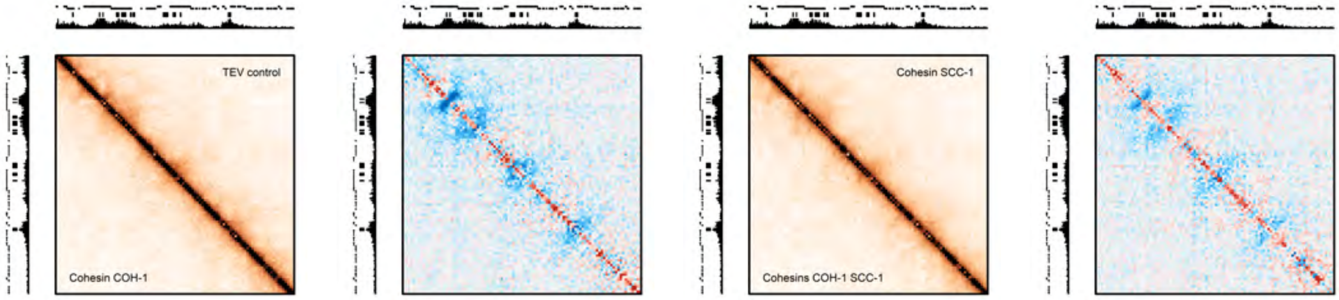

chrIII:9995001-10195000

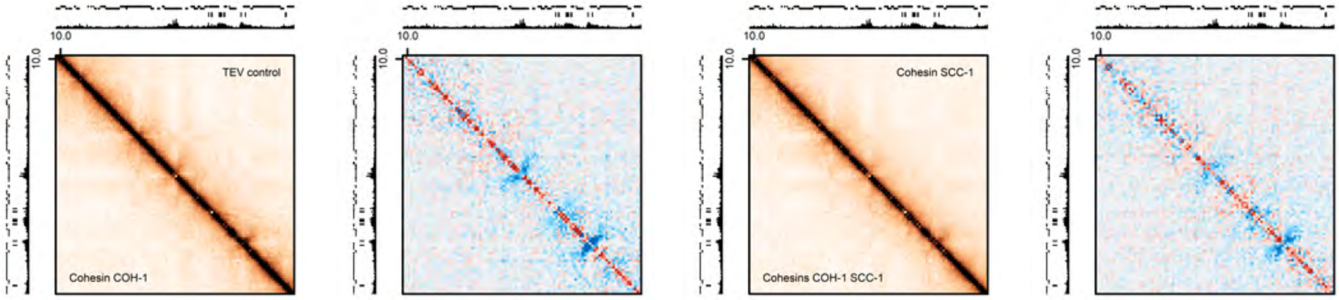

chrV:11995001-12195000

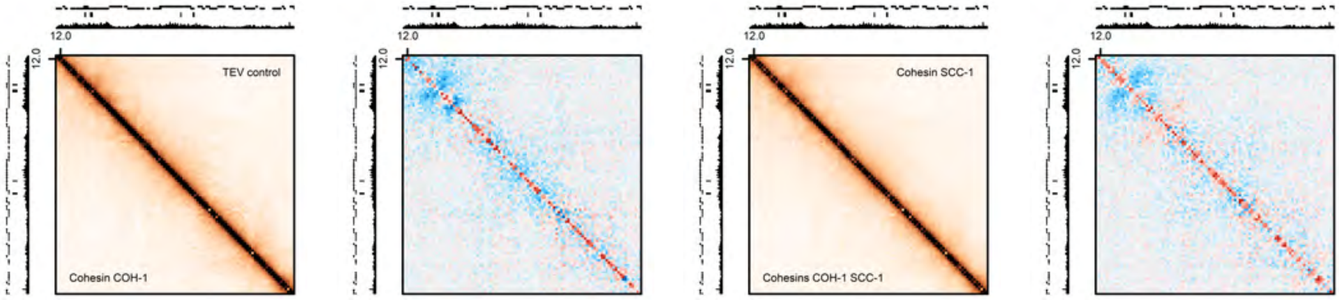

chrIII:2455001-2655000

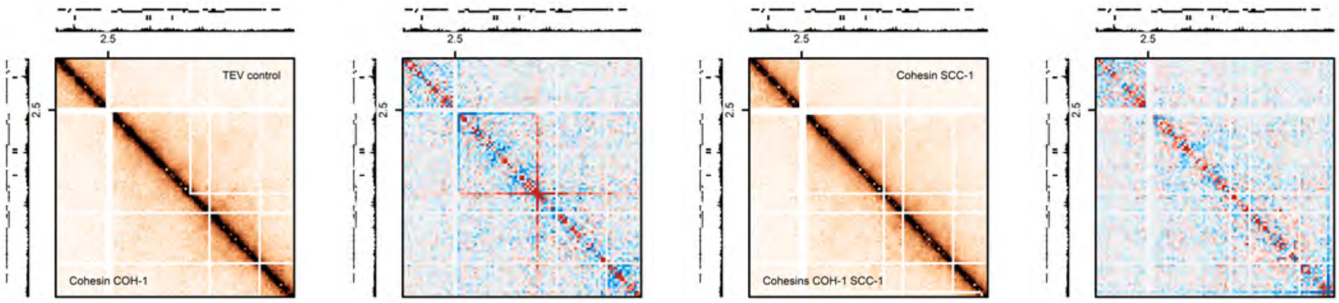

chrI:14709001-14909000

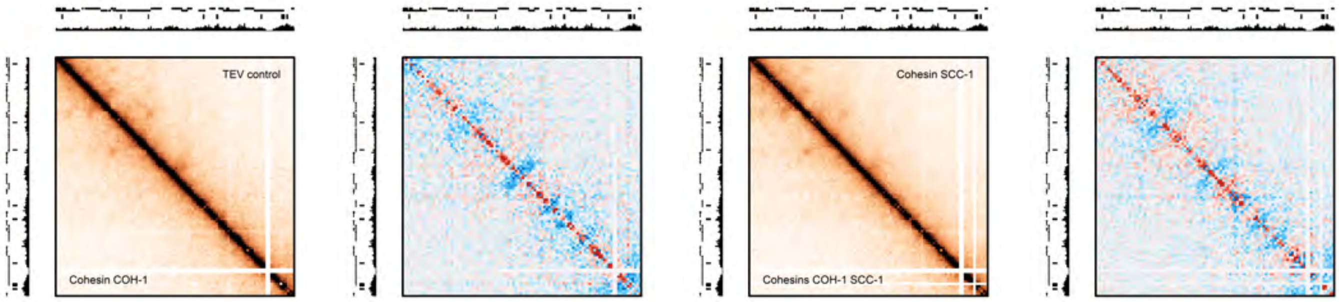

chrII:8685001-8885000

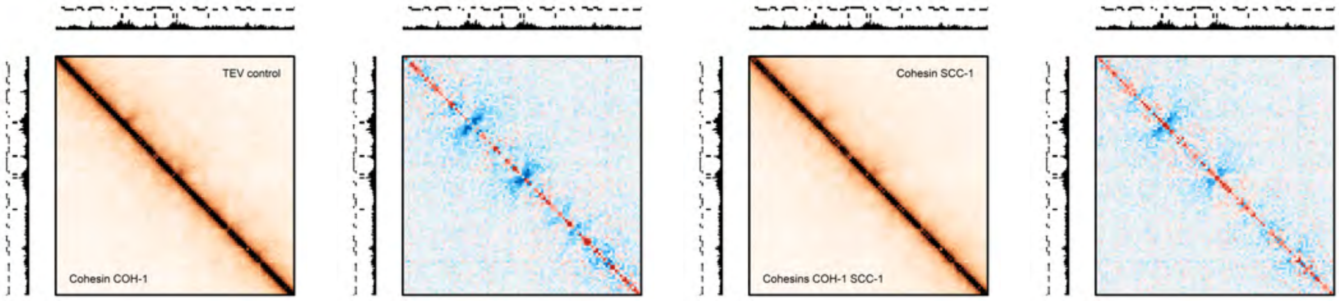

chrV:19567001-19767000

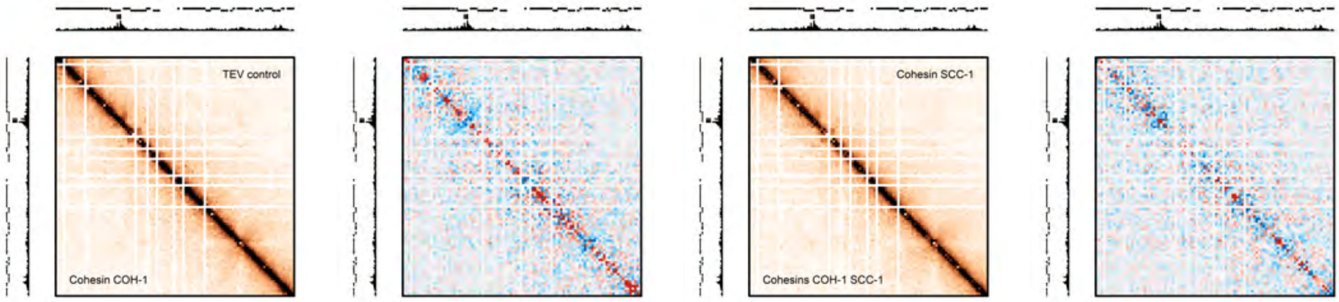

chrX:9835001-10035000

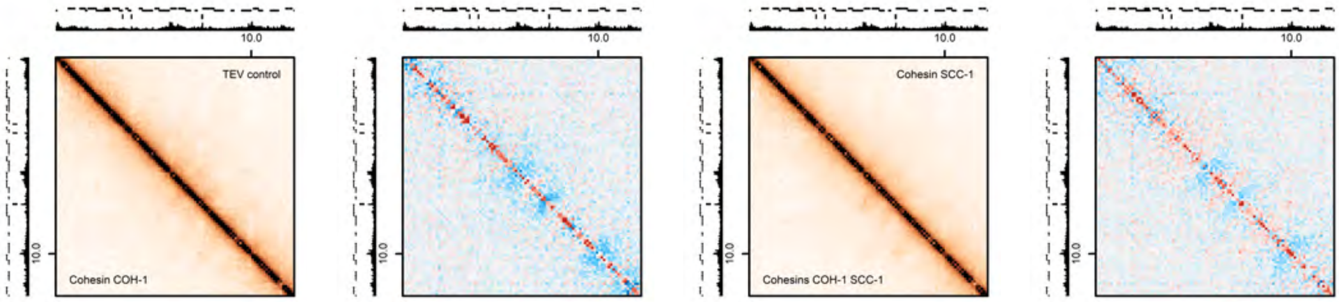

chrIII:4123001-4323000

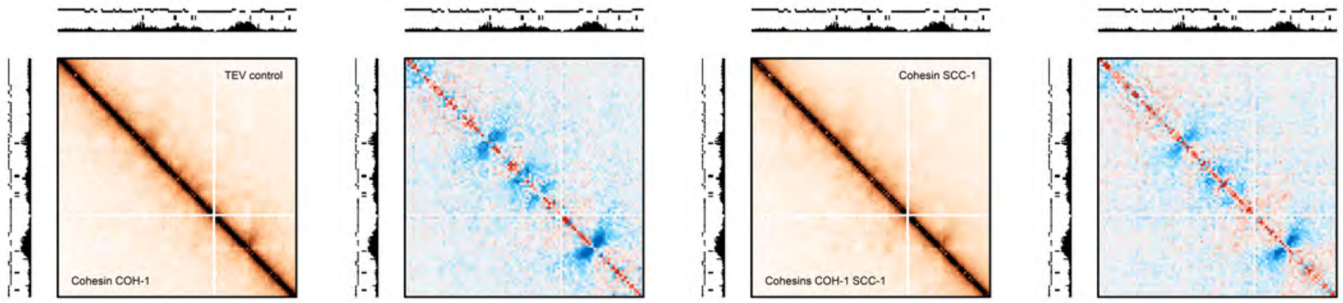

chrII:4653001-4853000

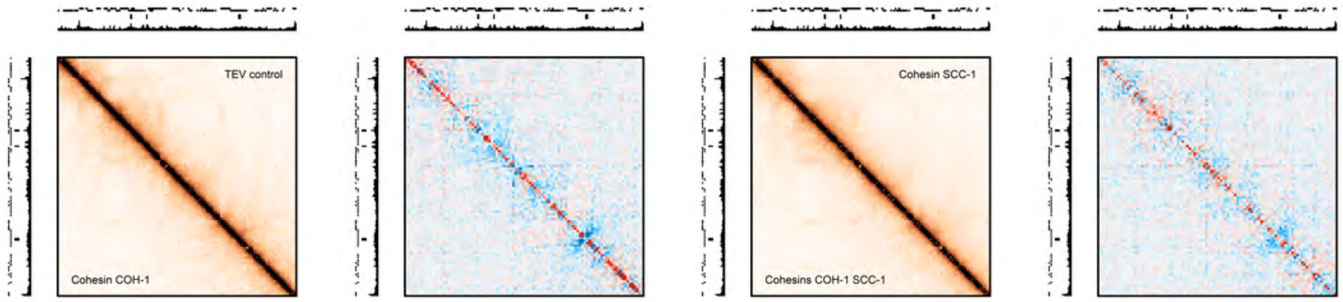

chrII:121001-321000

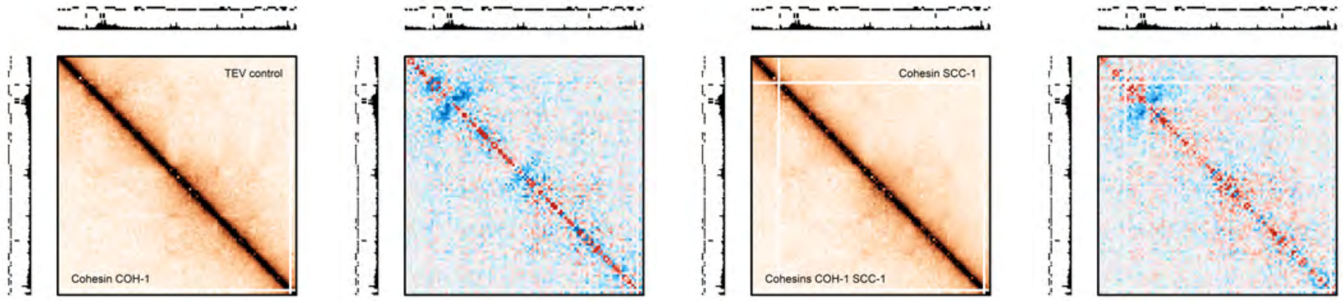

chrIV:9849001-10049000

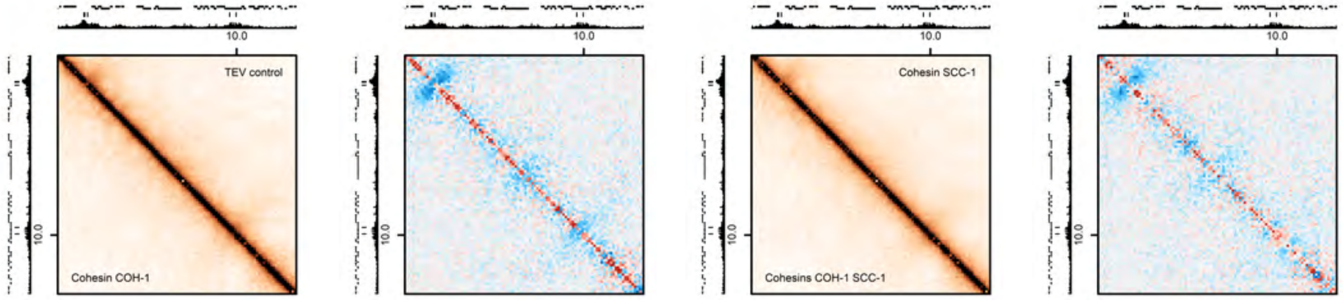

chrII:11297001-11497000

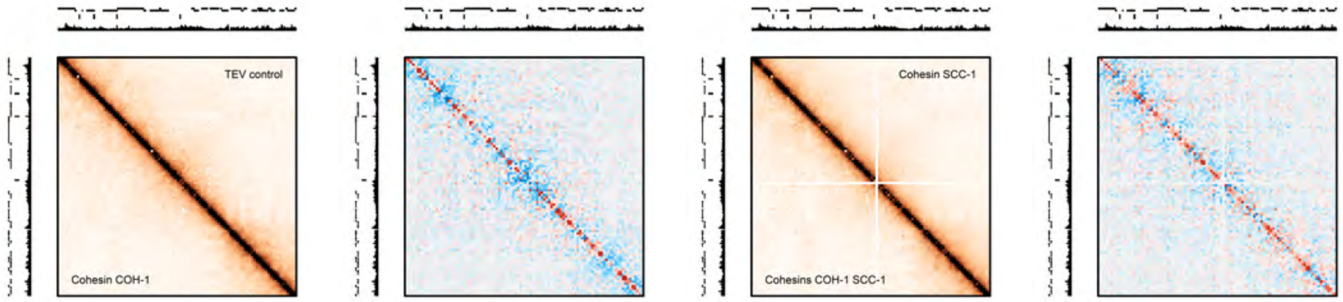

chrX:8567001-8767000

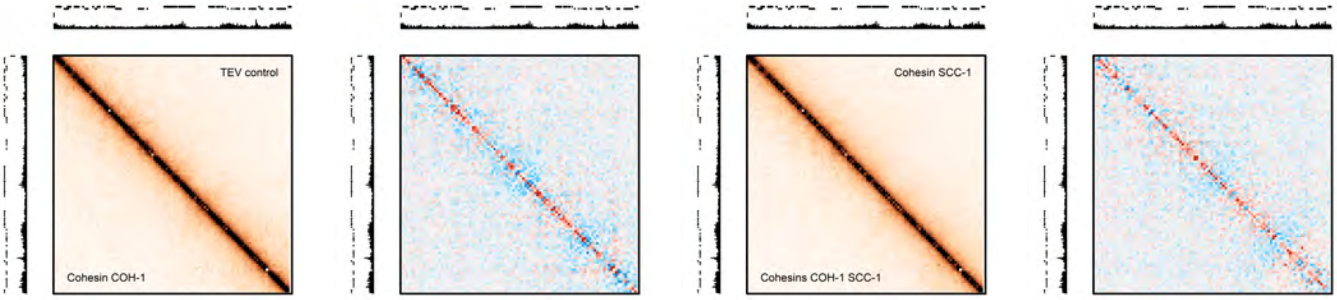

chrX:2805001-3005000

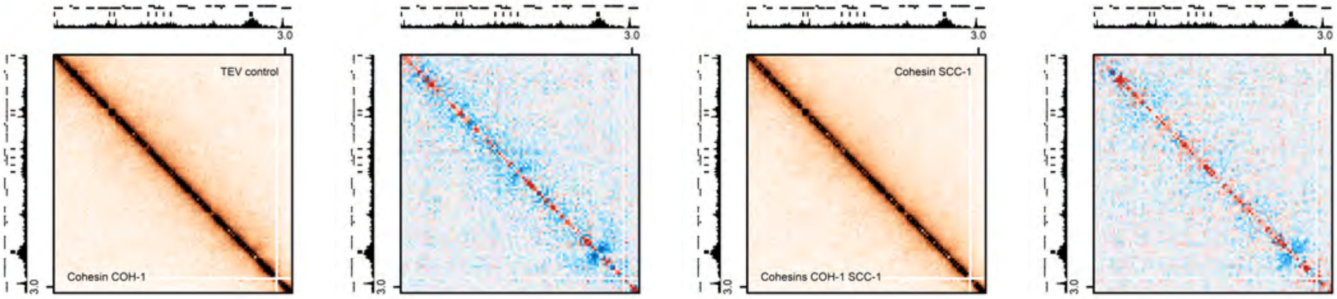

chrIV:13535001-13735000

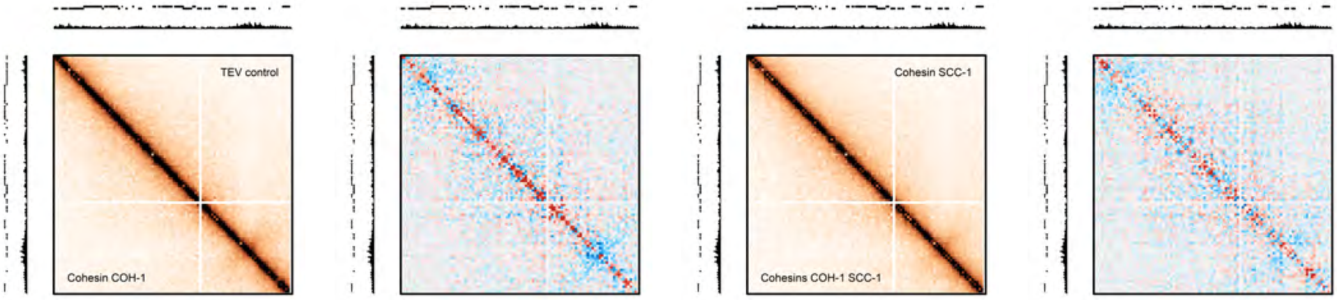

chrII:3207001-3407000

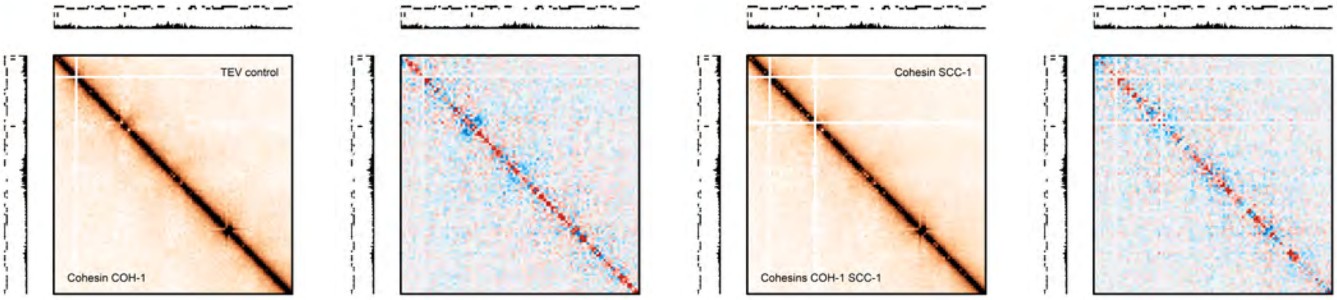

chrIV:275001-475000

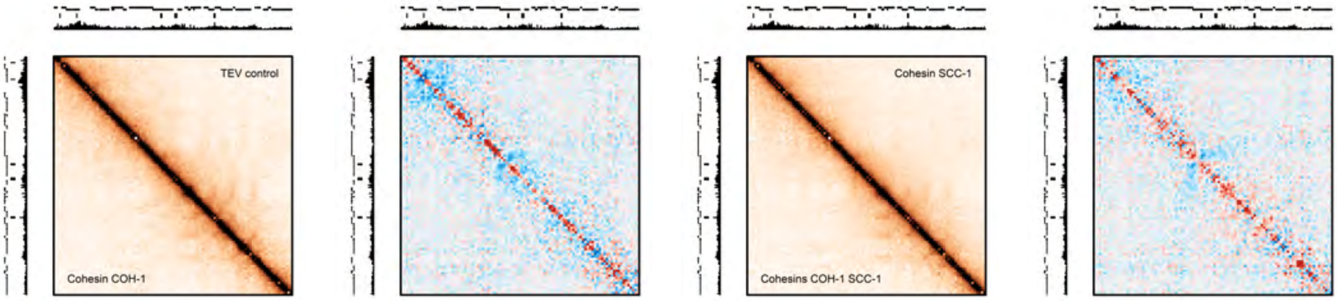

chrV:11757001-11957000

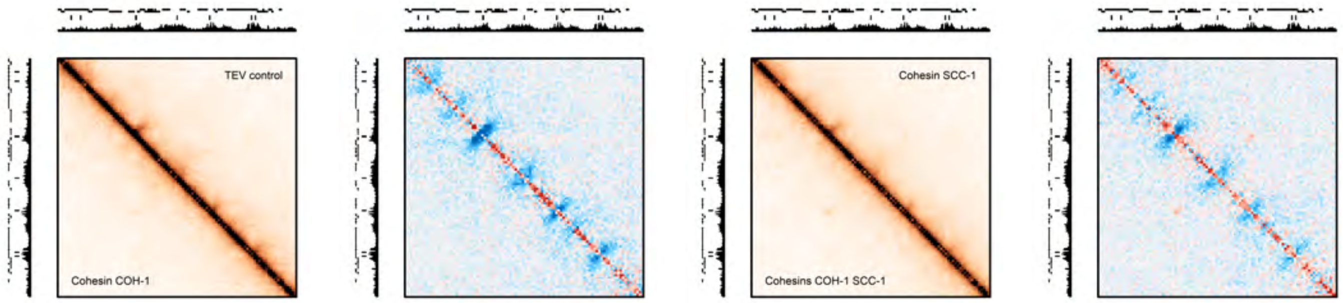

chrIII:1925001-2125000

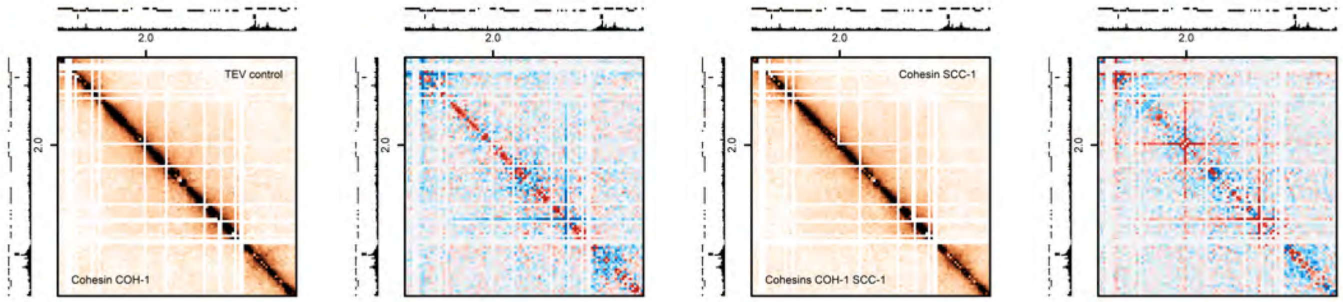

chrII:13855001-14055000

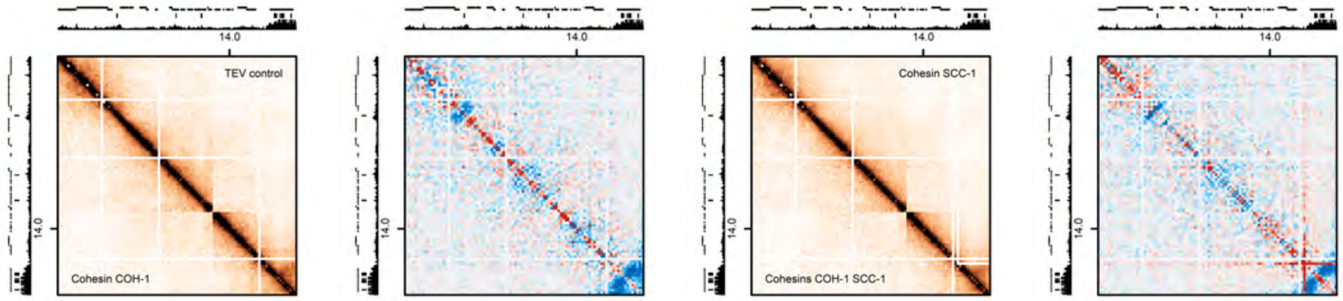

chrX:13533001-13733000

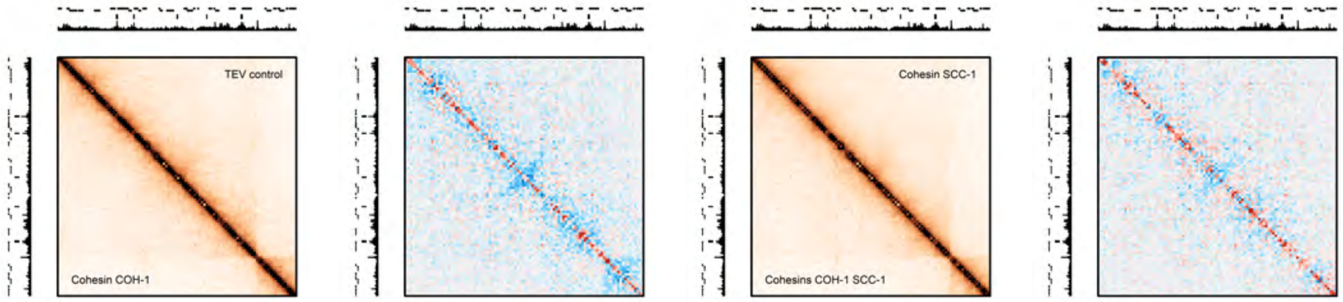

chrX:11723001-11923000

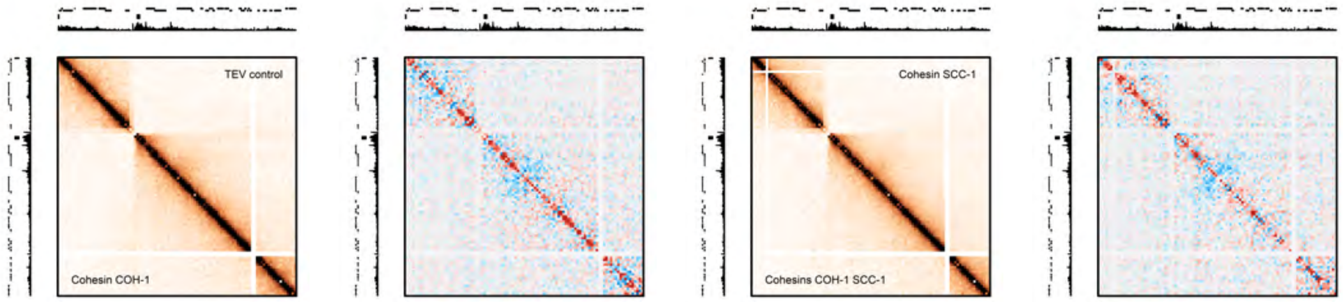

chrV:11335001-11535000

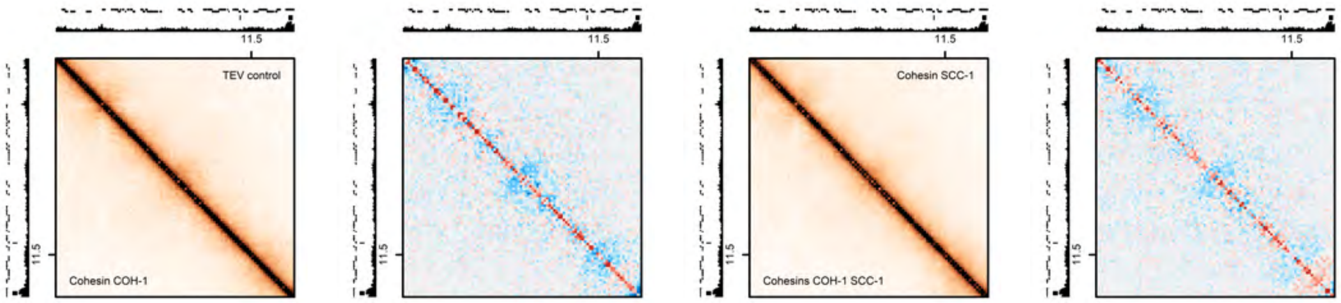

chrIII:11527001-11727000

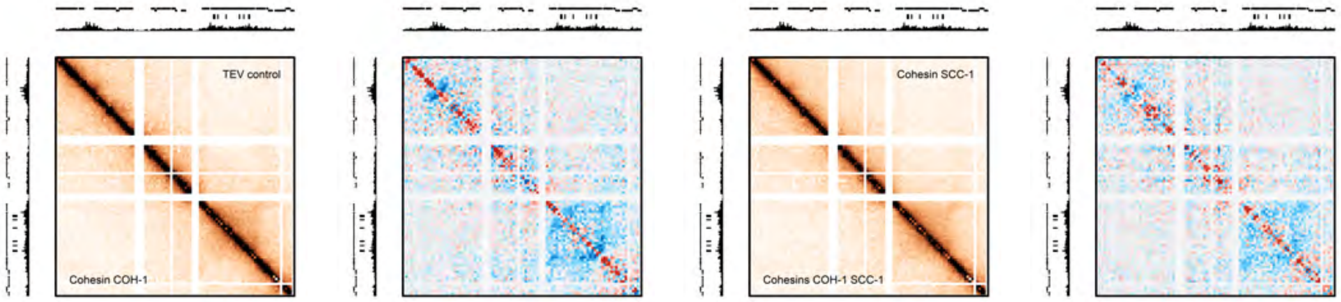

chrI:14665001-14865000

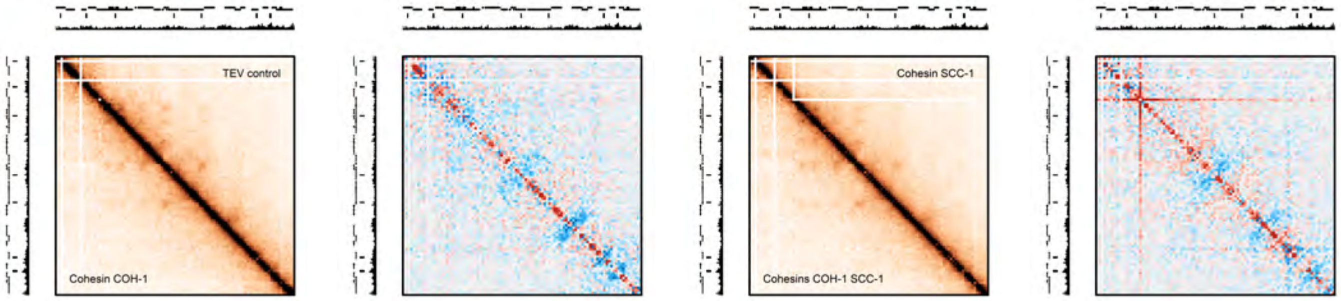

chrIII:519001-719000

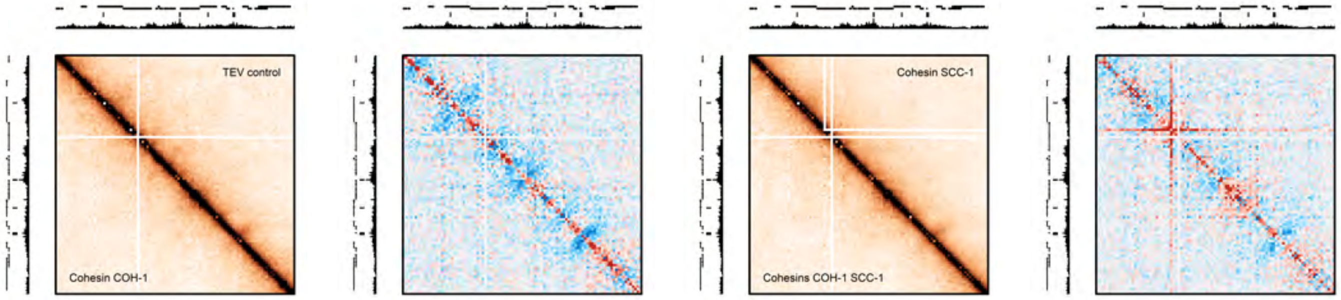

chrII:7621001-7821000

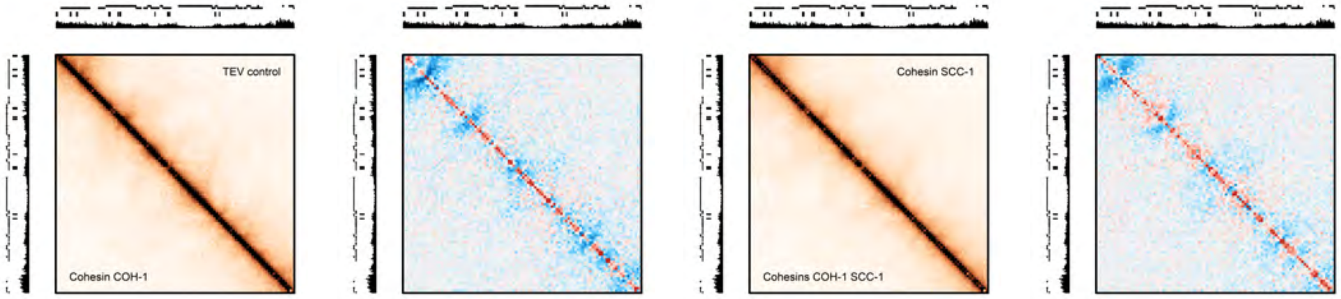

chrIV:4605001-4805000

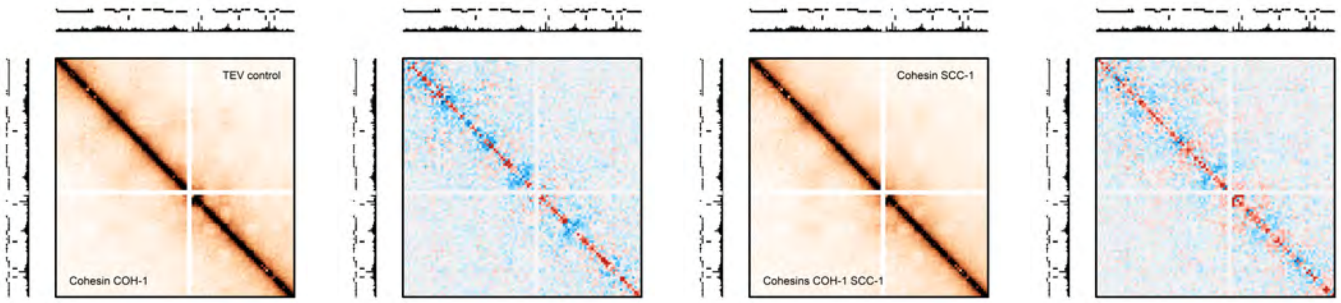

chrIV:14293001-14493000

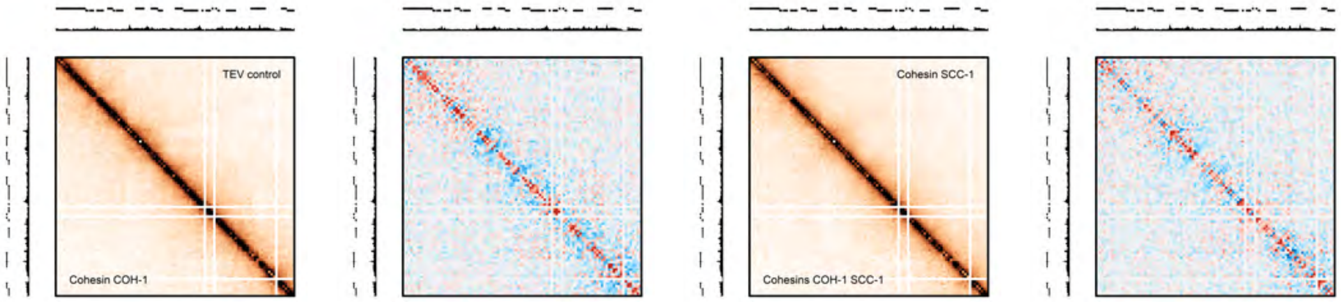

chrIII:2131001-2331000

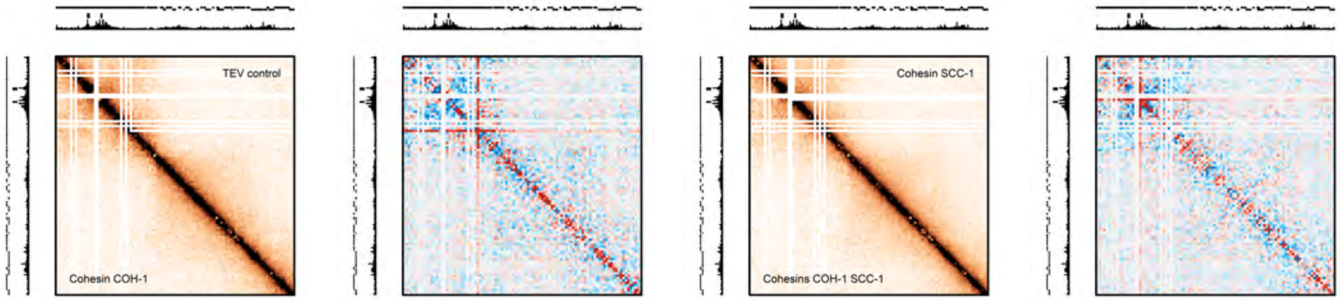

chrV:19727001-19927000

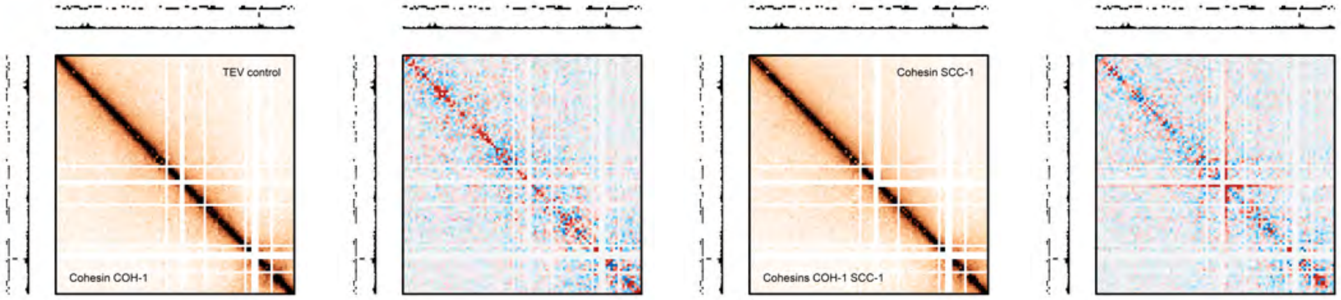

chrIII:5611001-5811000

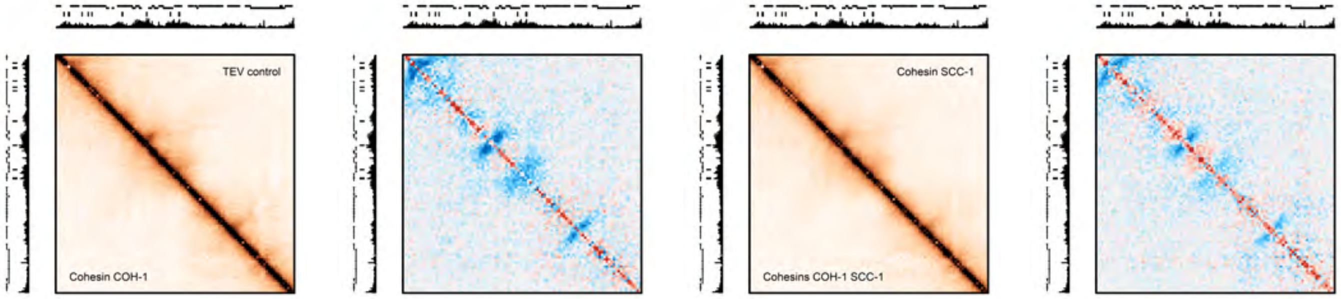

chrV:4053001-4253000

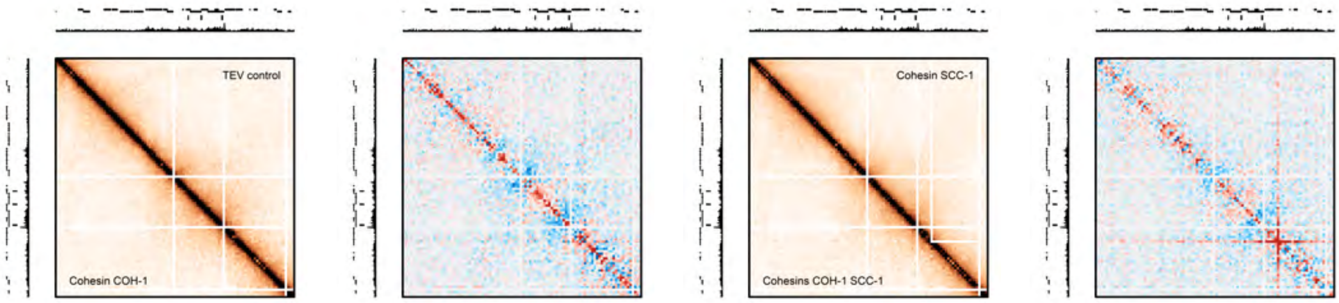

chrV:14593001-14793000

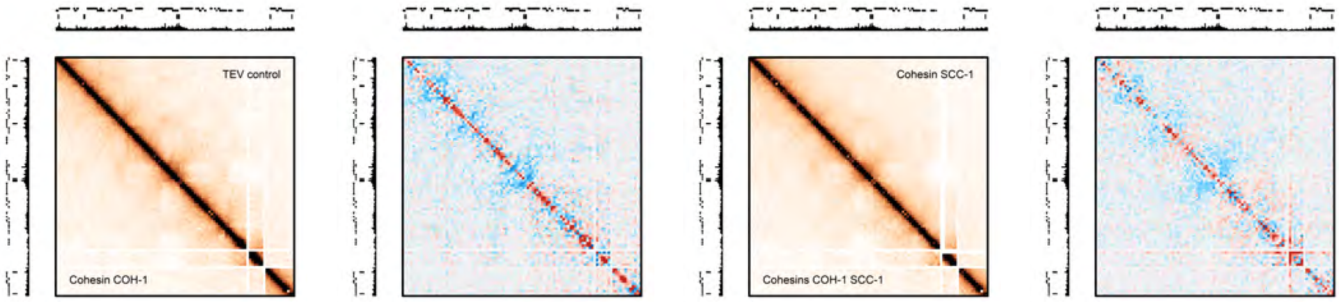

chrX:5159001-5359000

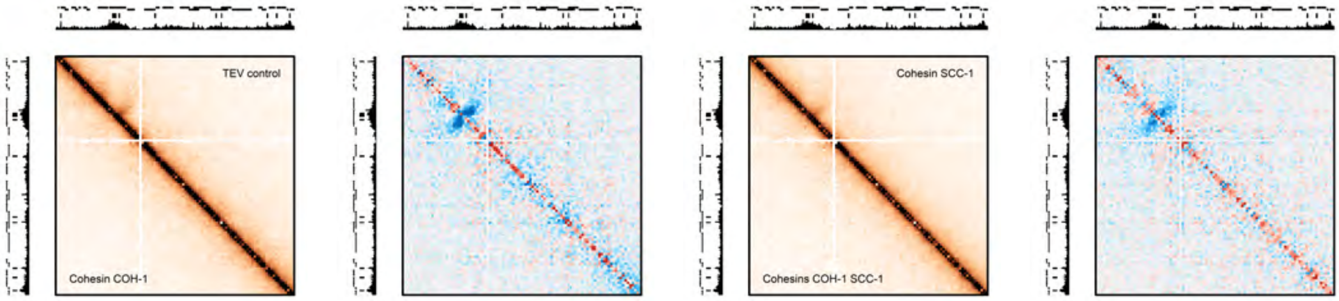

chrII:9473001-9673000

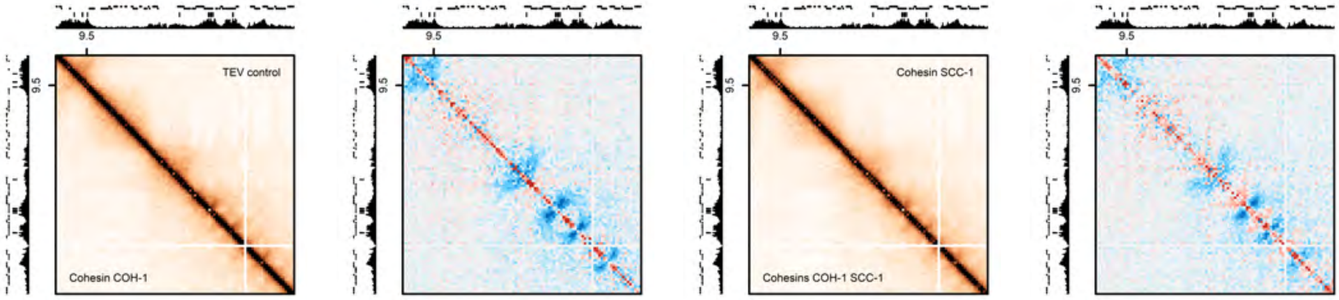

chrX:3215001-3415000

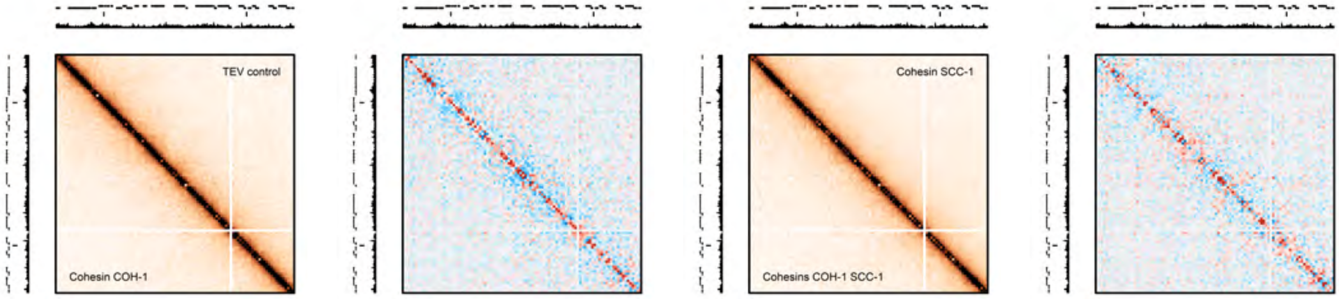

chrV:17155001-17355000

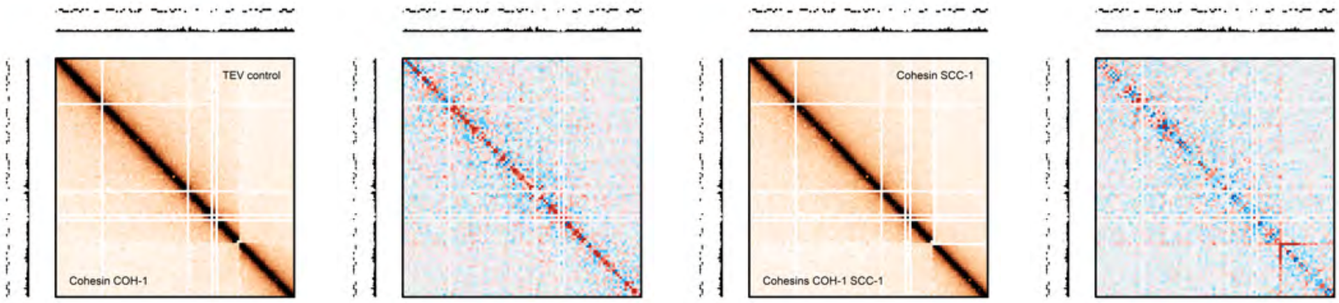

chrIV:17109001-17309000

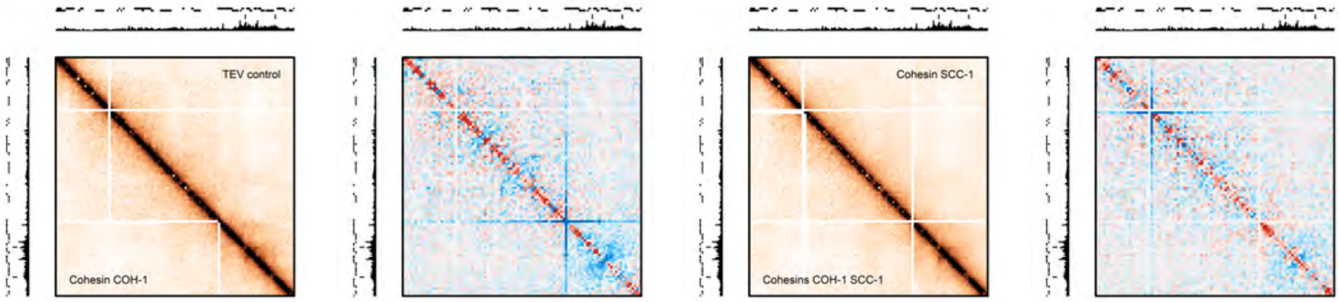

chrI:3583001-3783000

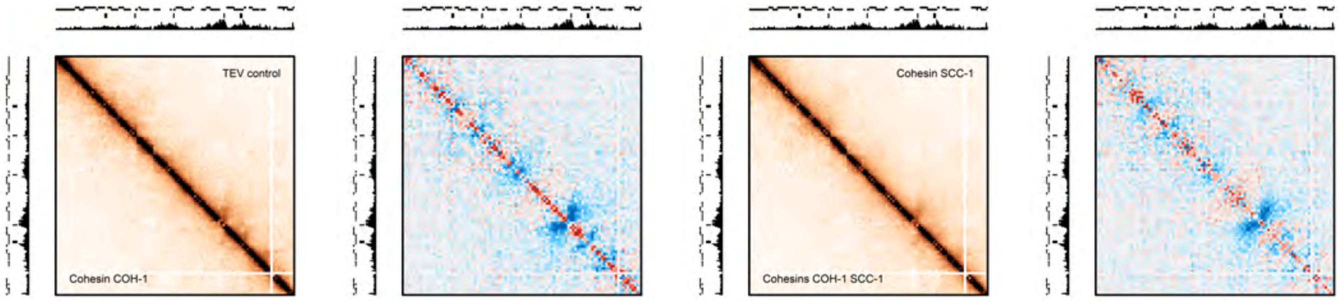

chrX:6309001-6509000

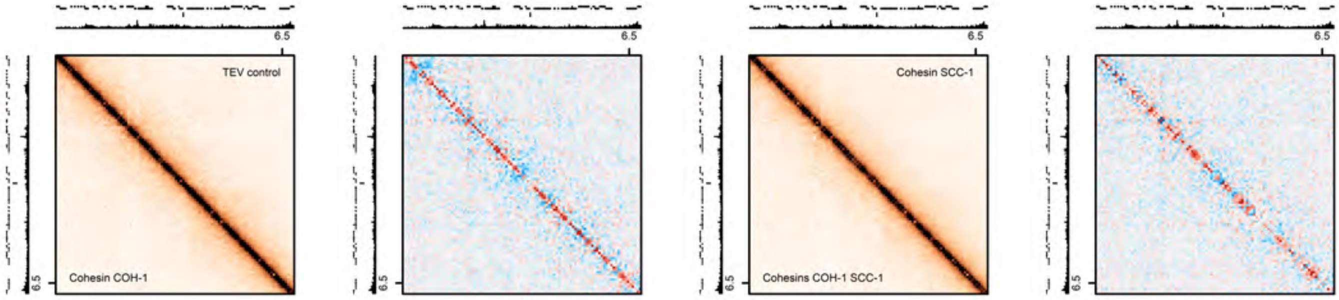

chrIII:5039001-5239000

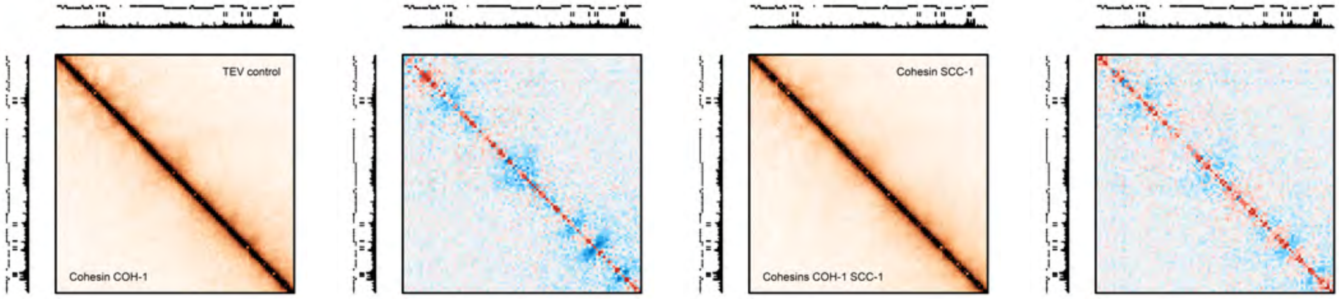

chrV:1573001-1773000

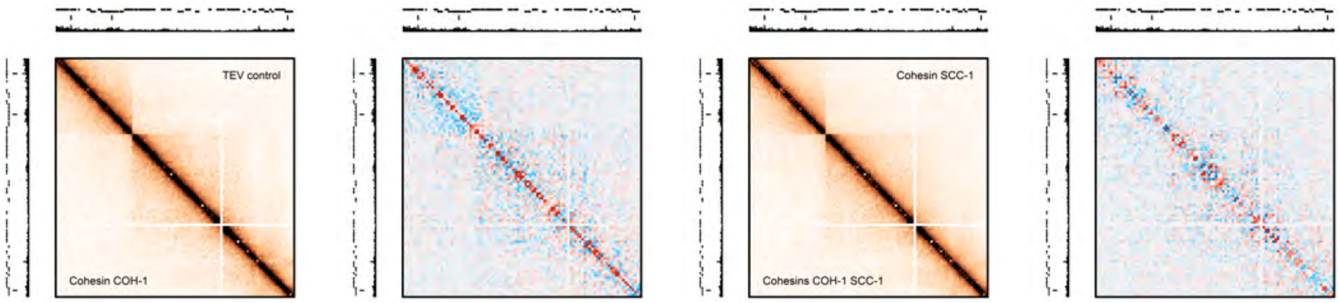

chrII:2493001-2693000

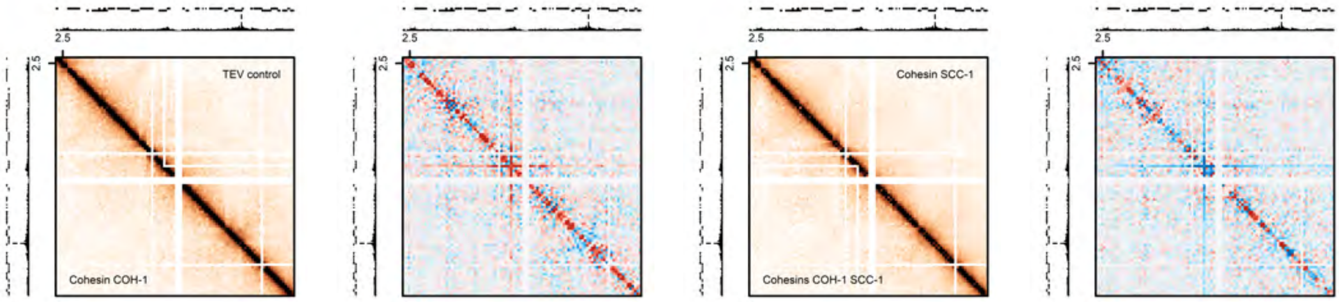

chrII:10107001-10307000

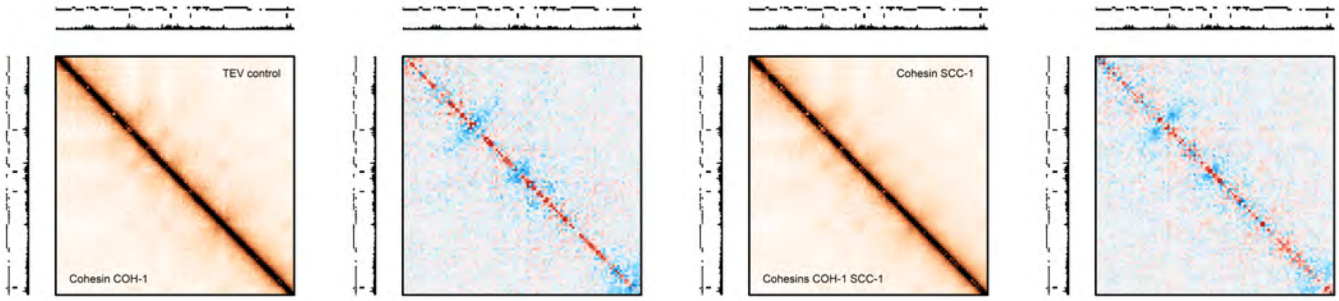

chrX:5999001-6199000

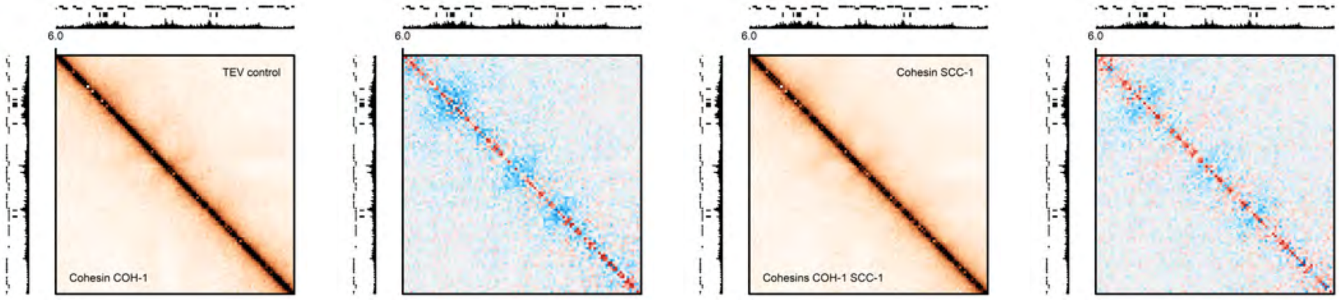

chrIII:3113001-3313000

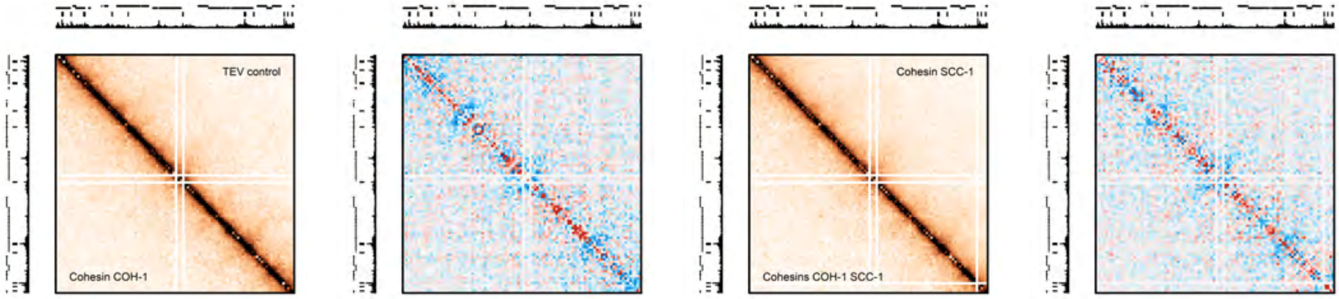

chrX:957001-1157000

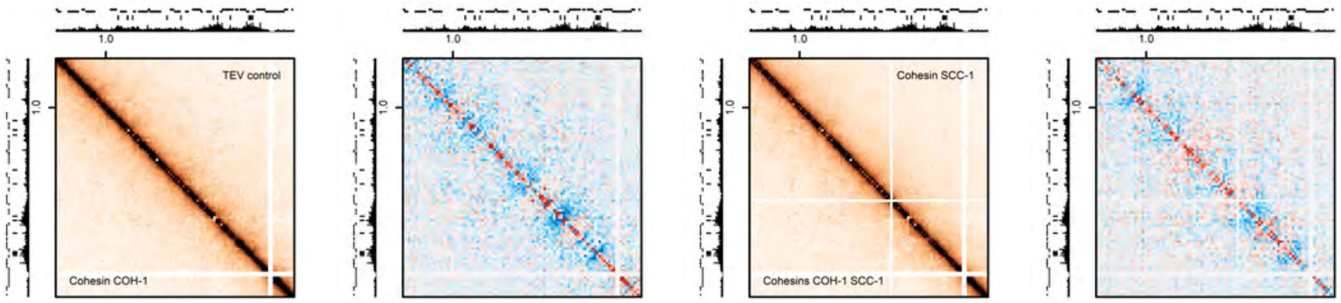

chrII:-38999-161000

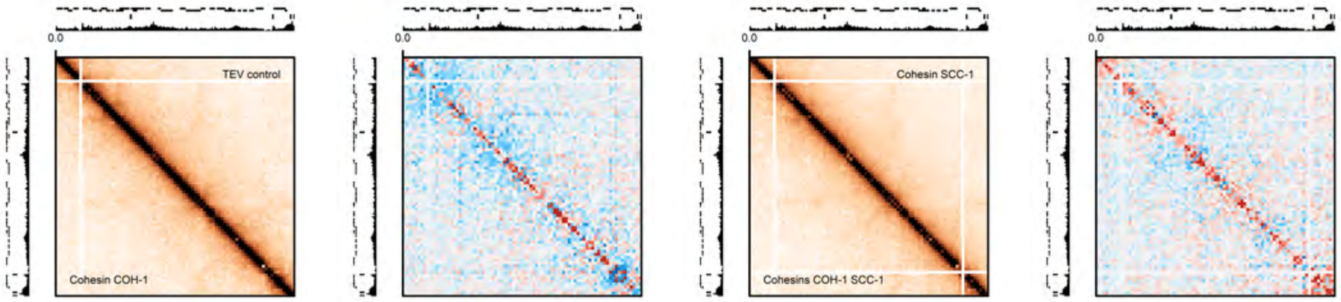

chrV:6041001-6241000

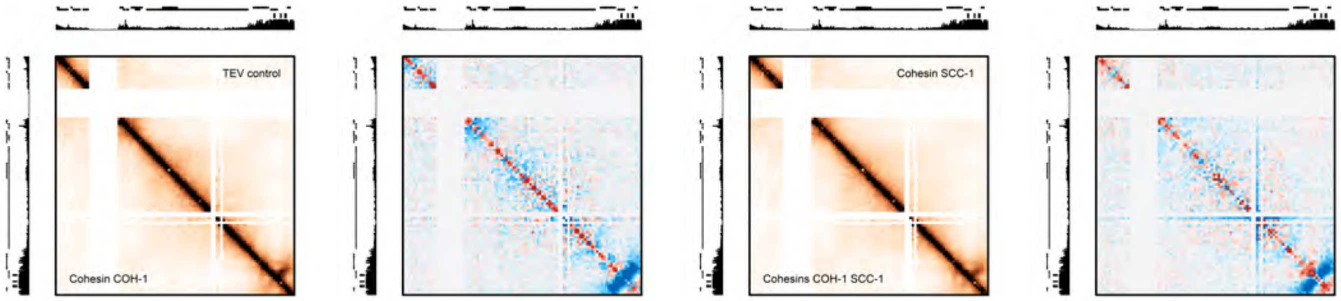

chrII:10403001-10603000

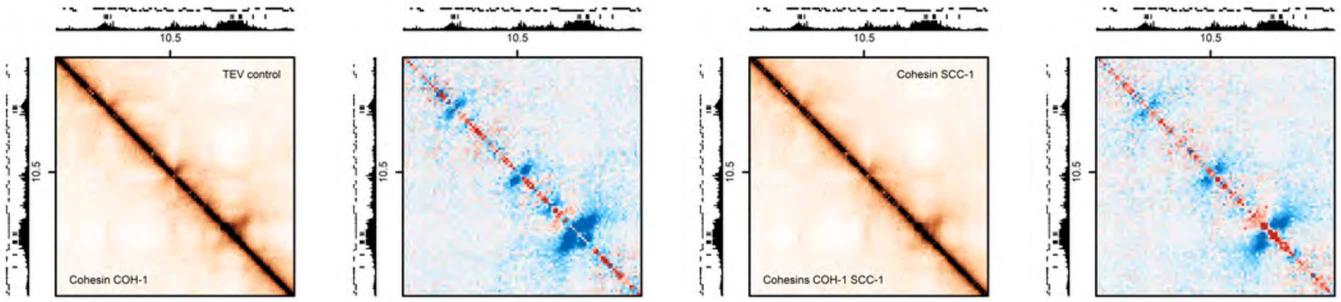

chrV:3927001-4127000

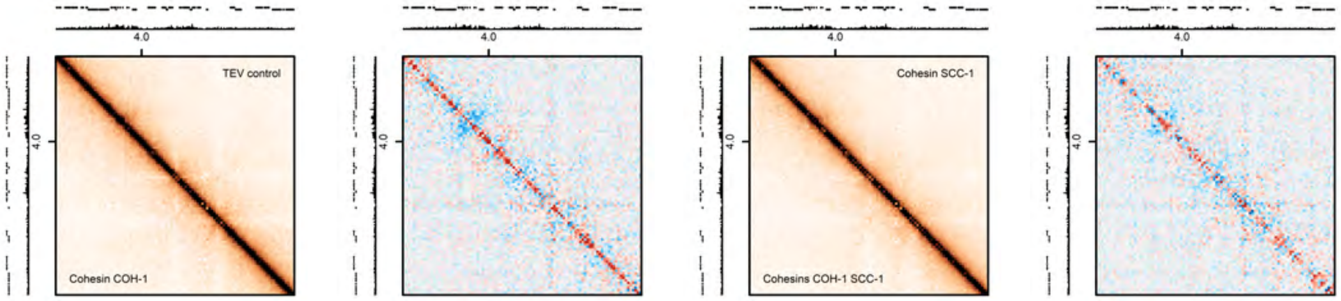

chrII:3113001-3313000

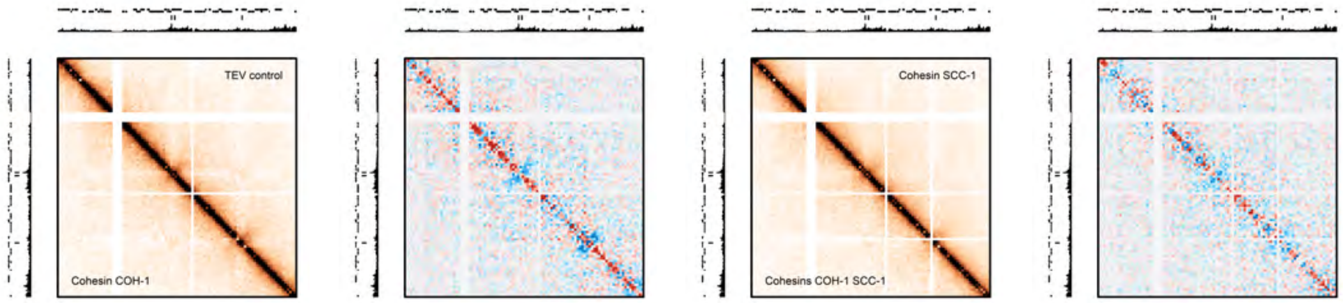

chrIII:6591001-6791000

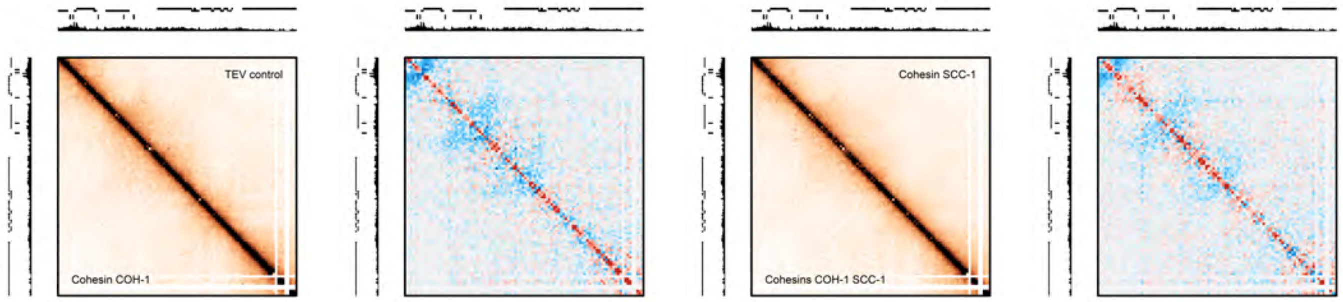

chrI:12009001-12209000

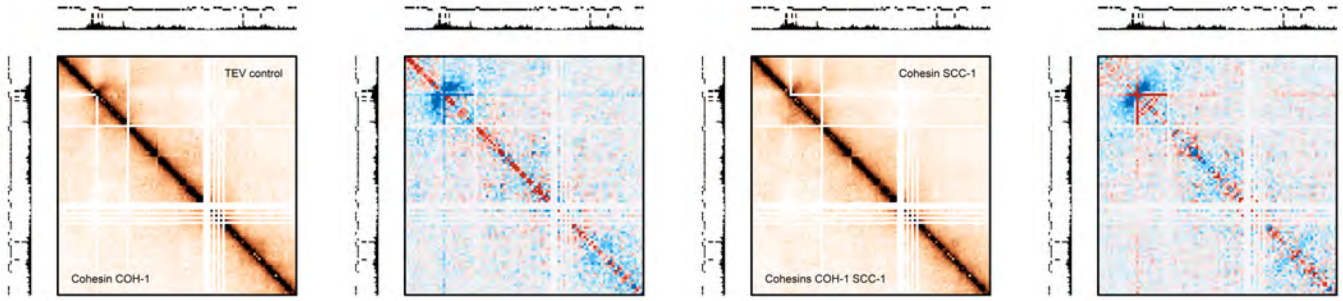

chrX:10471001-10671000

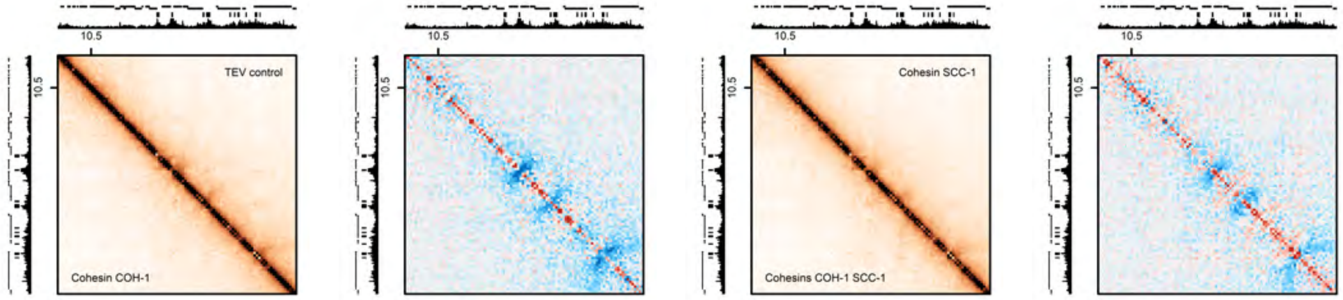

chrX:5409001-5609000

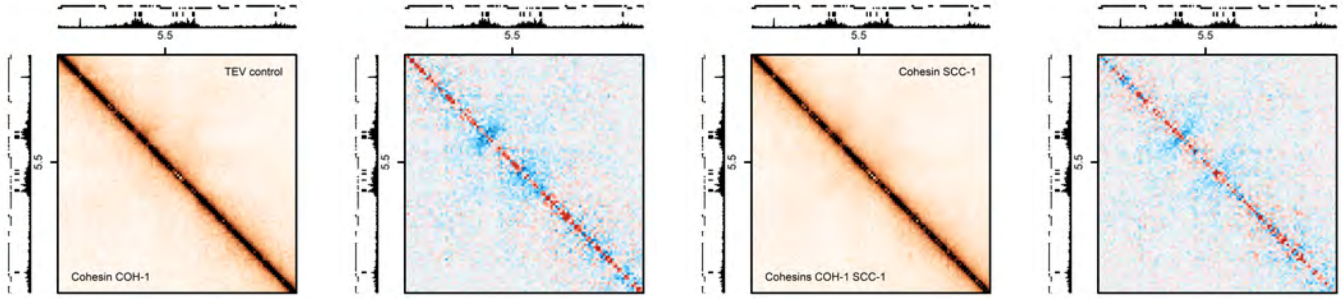

chrII:1551001-1751000

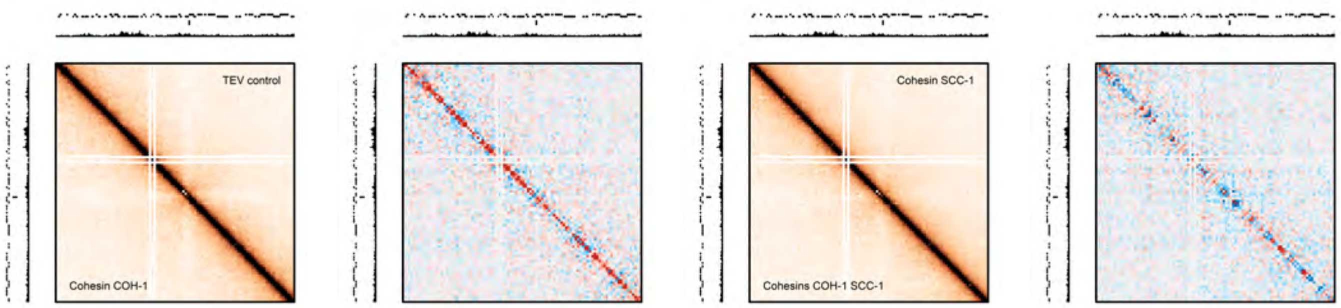

chrX:16349001-16549000

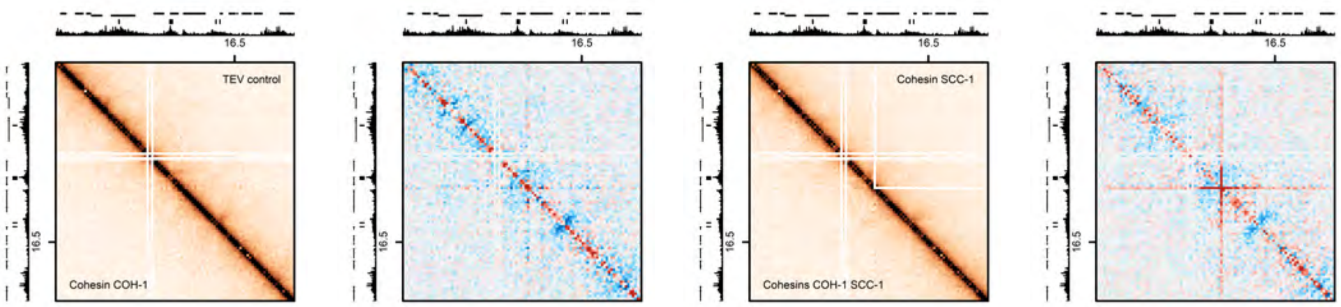

chrV:3351001-3551000

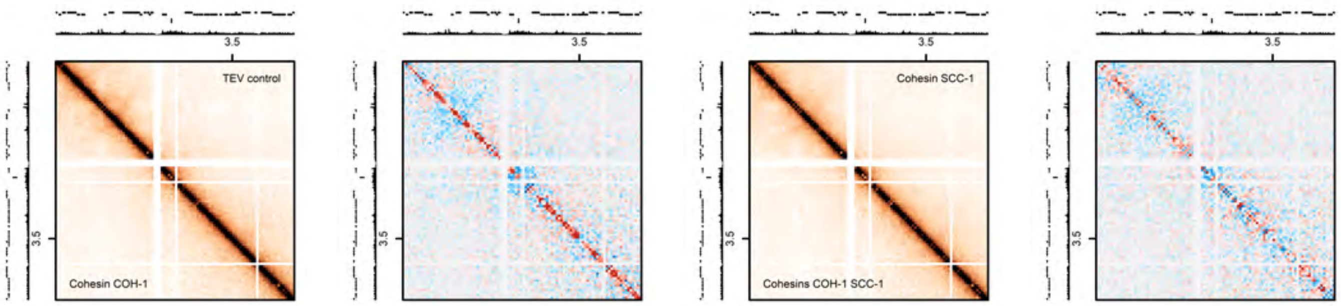

chrX:4563001-4763000

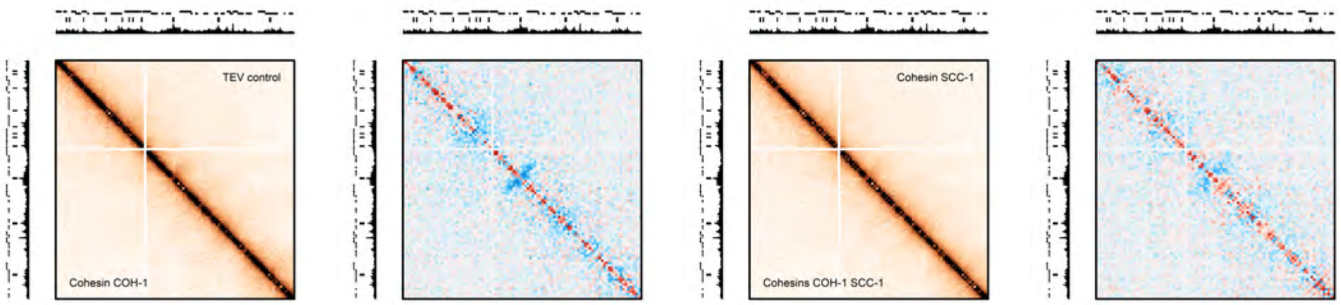

chrIII:1999001-2199000

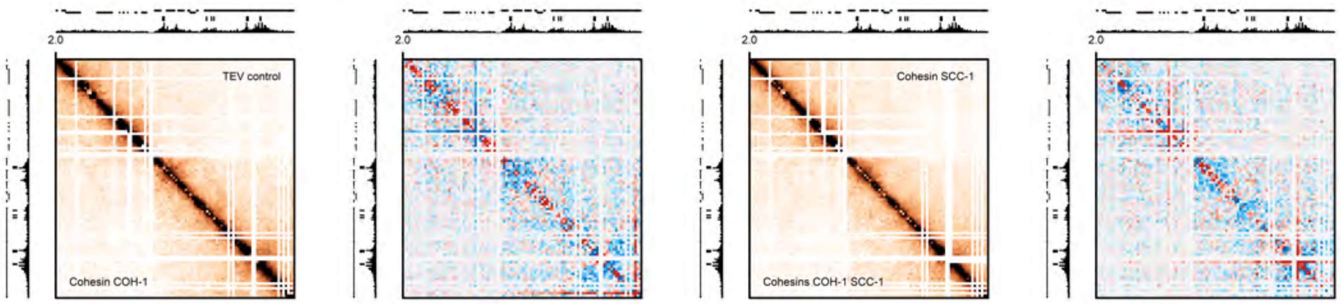

chrIII:7557001-7757000

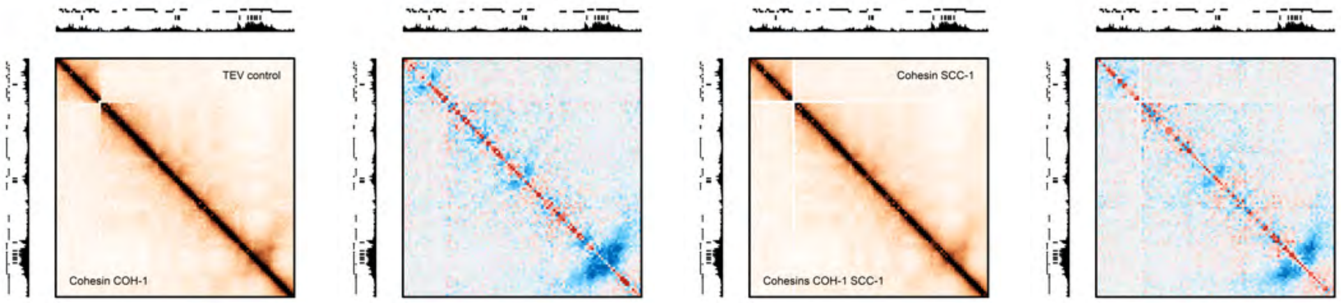

chrX:16387001-16587000

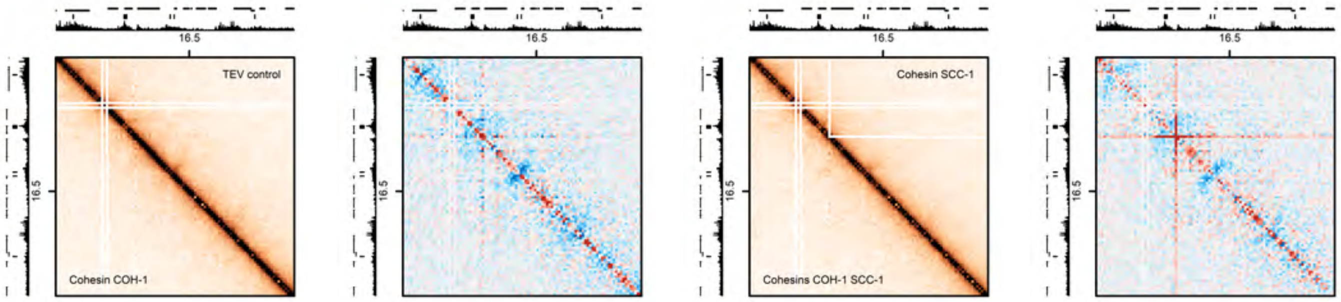

chrV:187001-387000

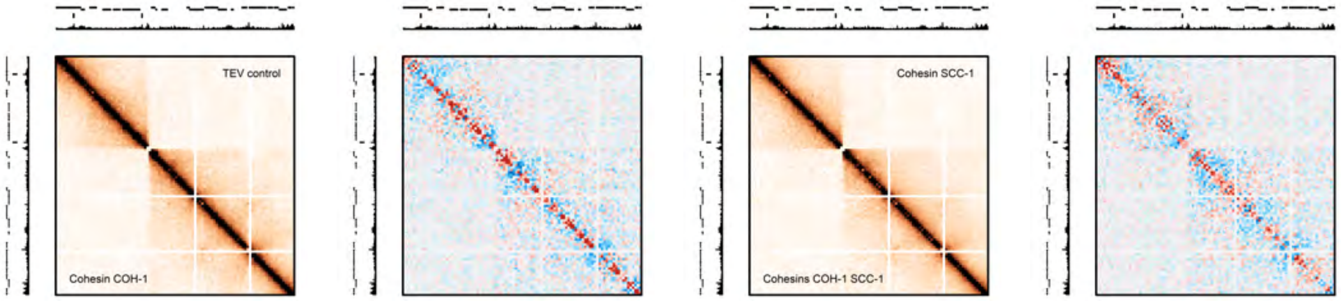

chrIII:10379001-10579000

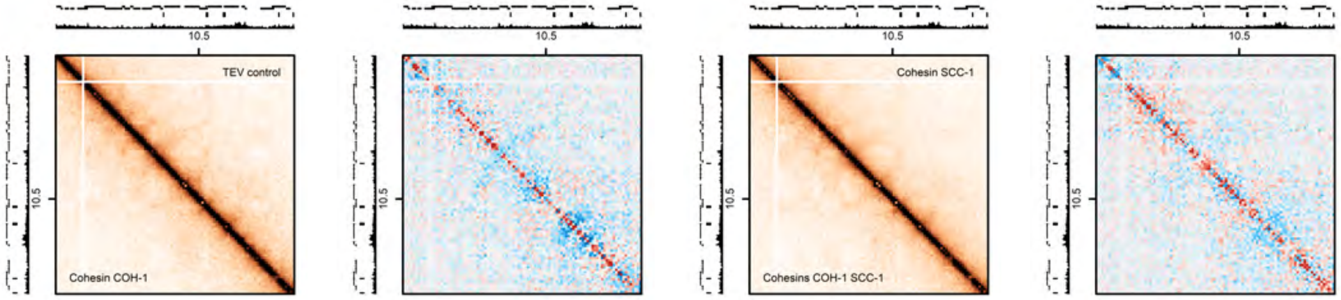

chrIV:6553001-6753000

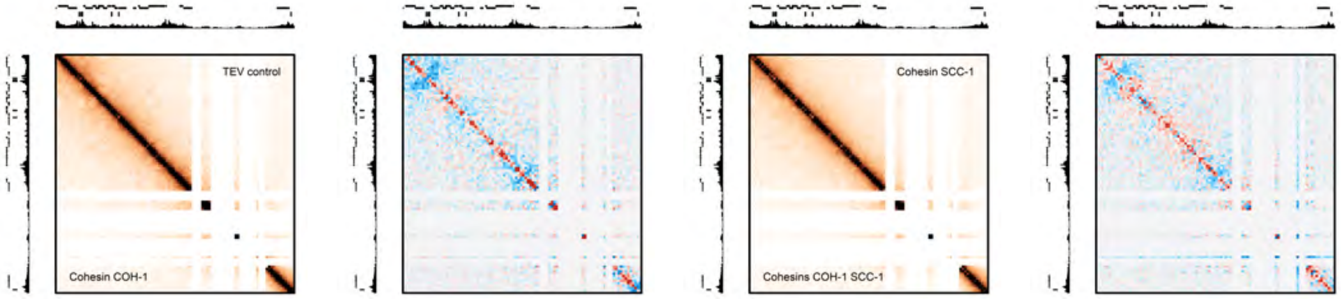

chrI:14177001-14377000

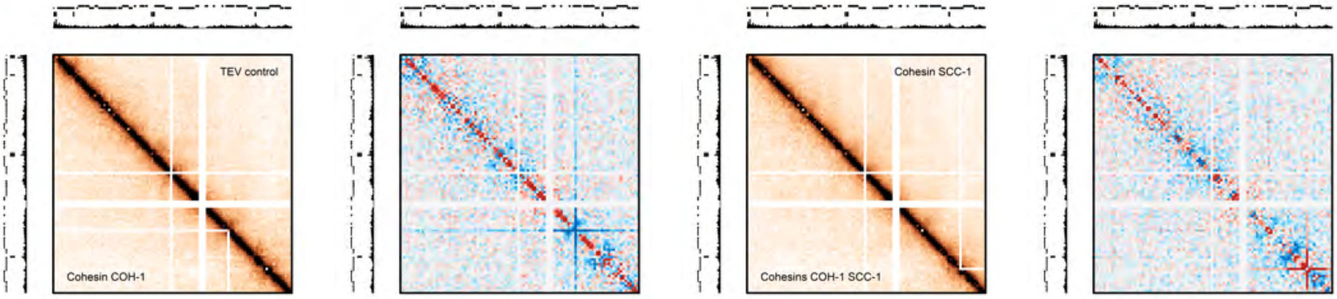

chrIV:77001-277000

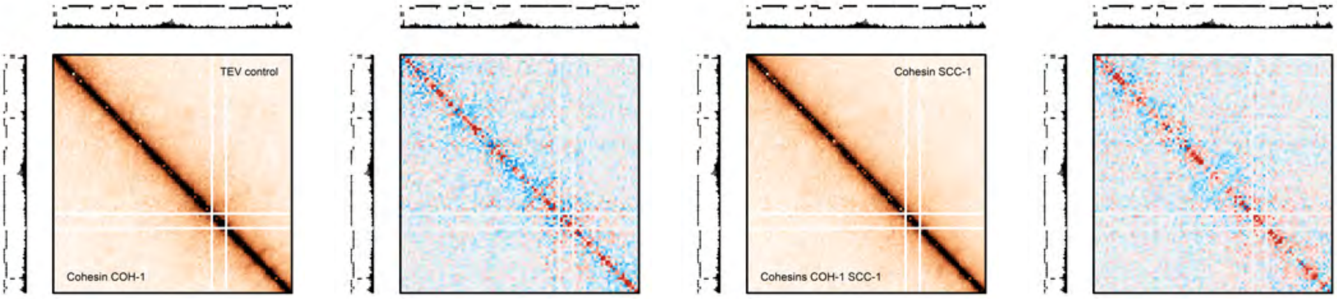

chrV:3535001-3735000

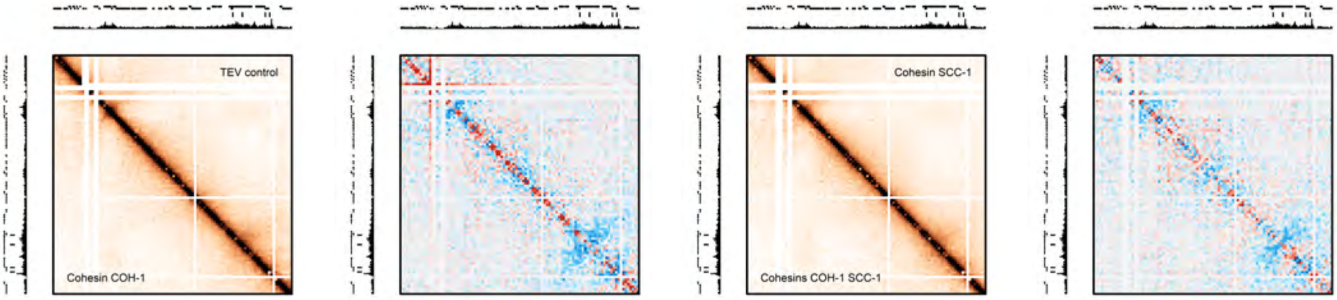

chrII:8321001-8521000

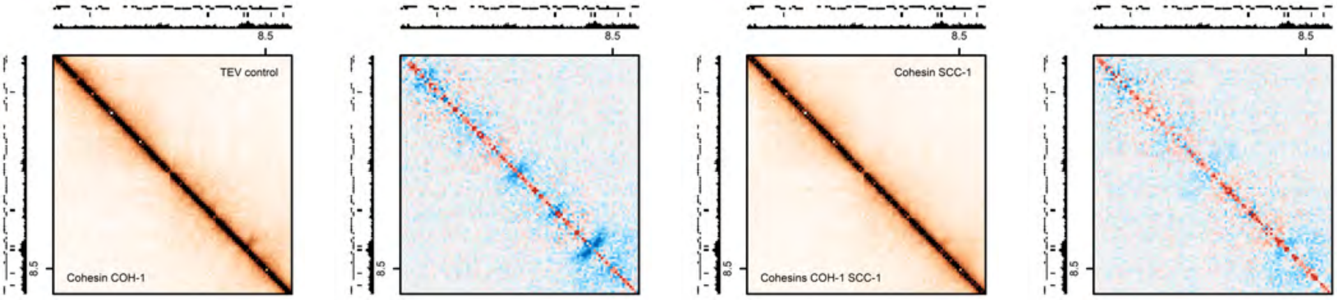

chrX:335001-535000

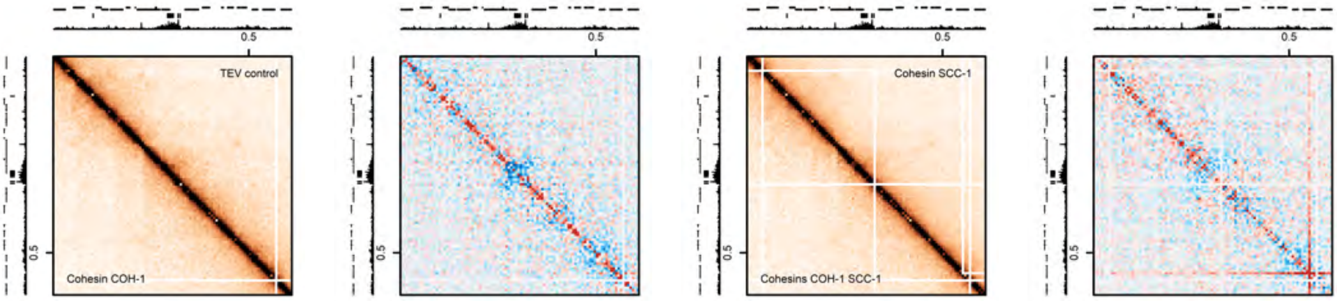

chrIV:463001-663000

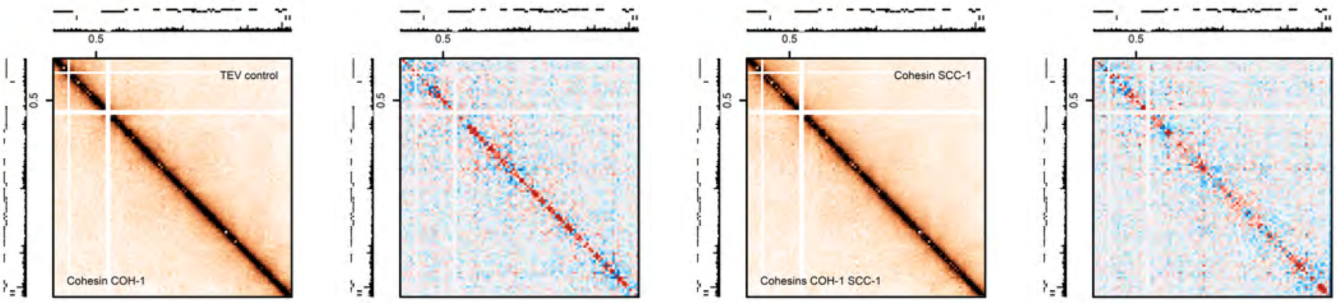

chrX:11621001-11821000

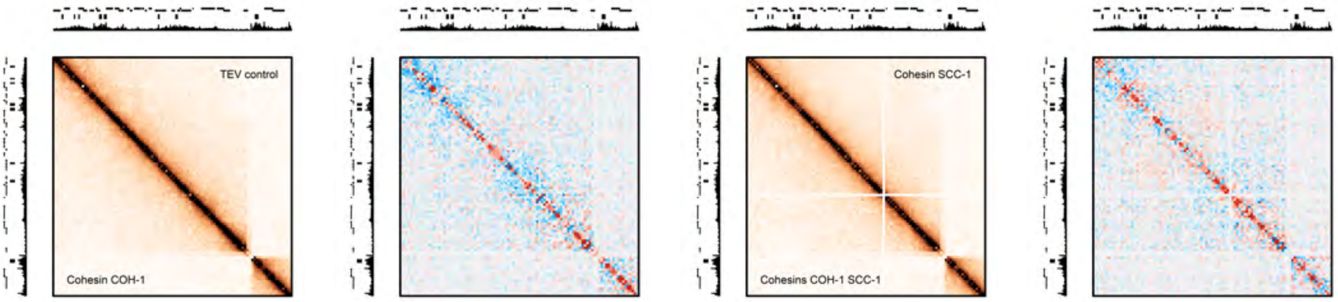

chrX:15677001-15877000

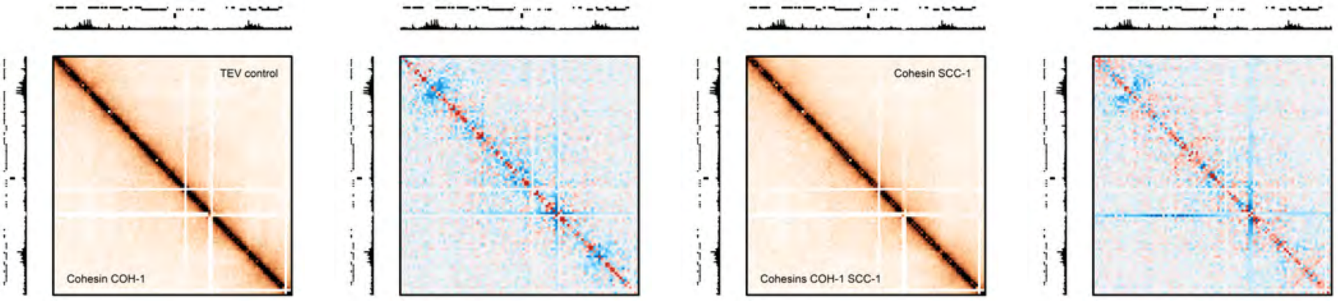

chrV:19961001-20161000

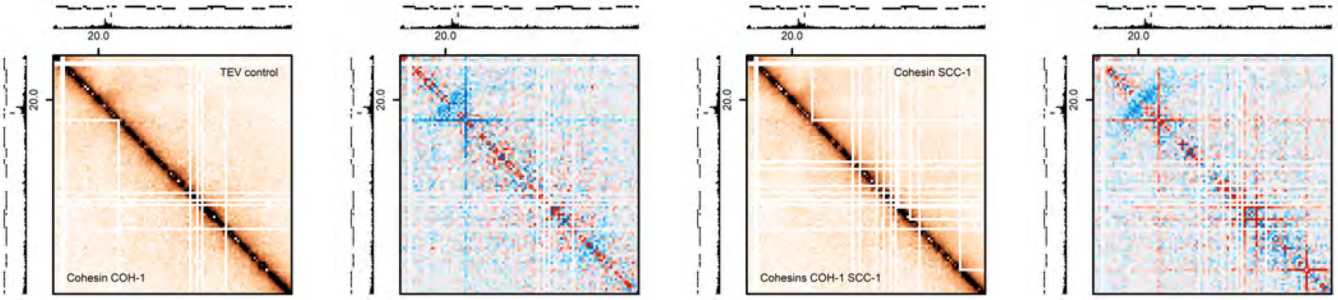

chrIV:12807001-13007000

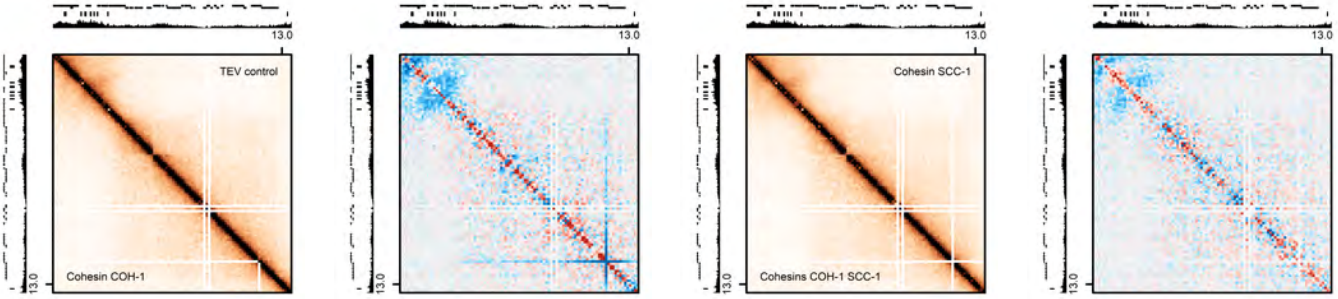

chrIV:12983001-13183000

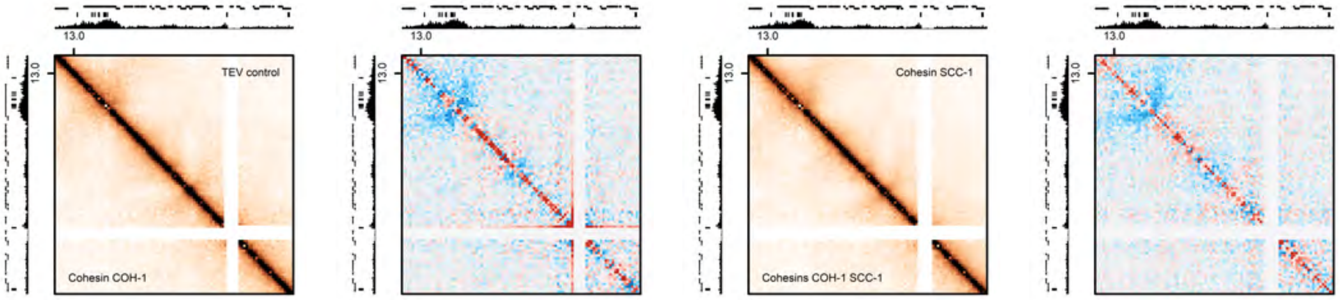

chrV:14913001-15113000

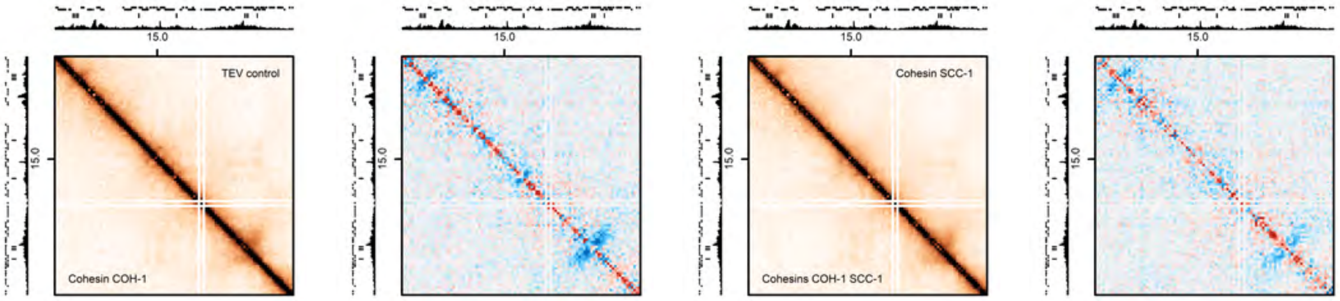

chrV:12113001-12313000

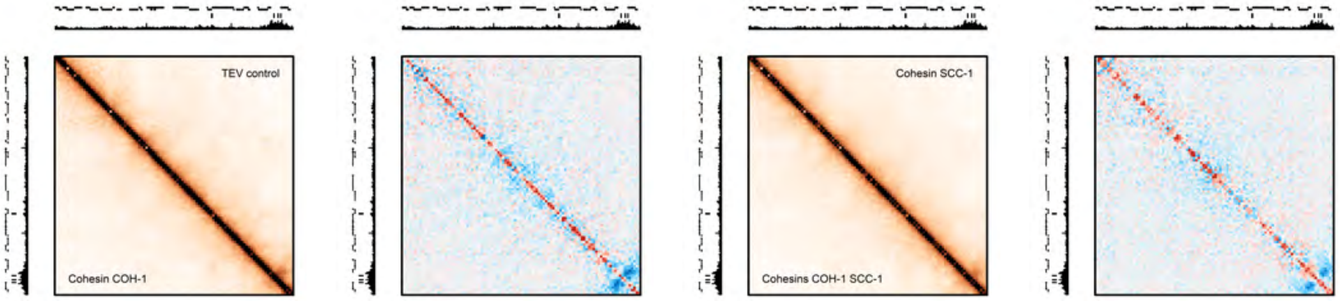

chrII:2733001-2933000

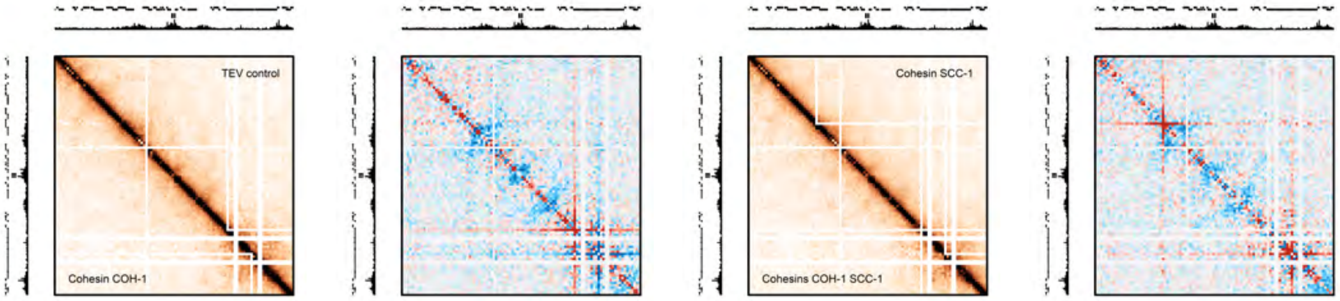

chrIV:7519001-7719000

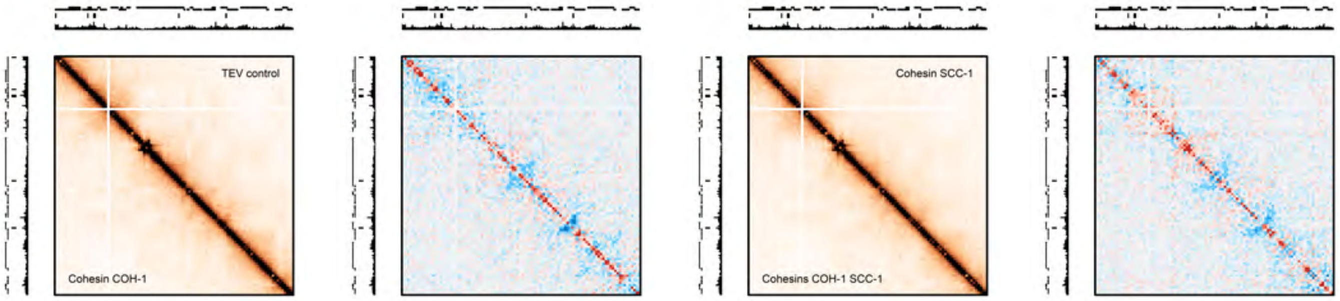

chrIV:2321001-2521000

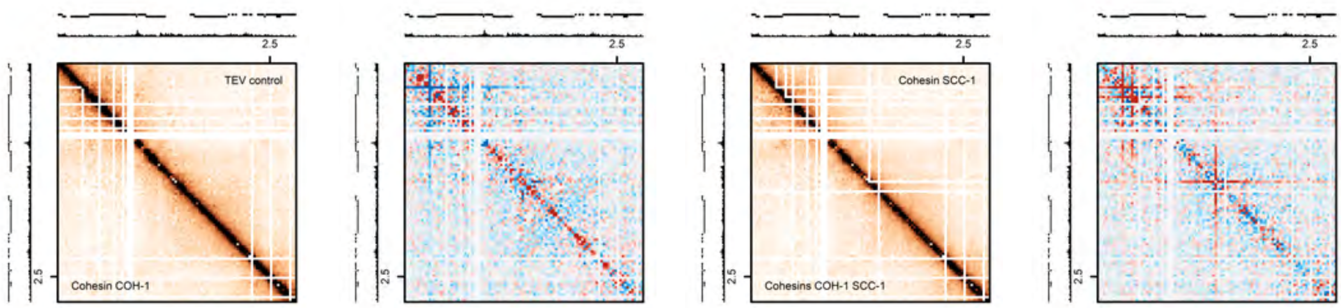

chrV:17757001-17957000

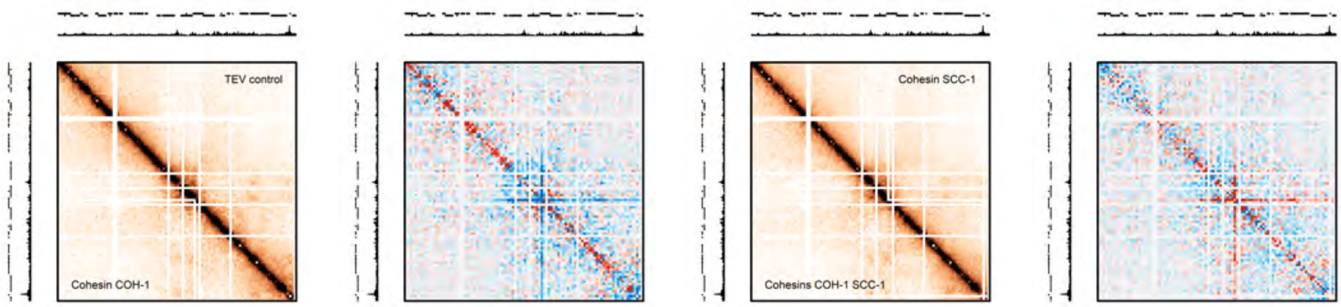

chrIII:10479001-10679000

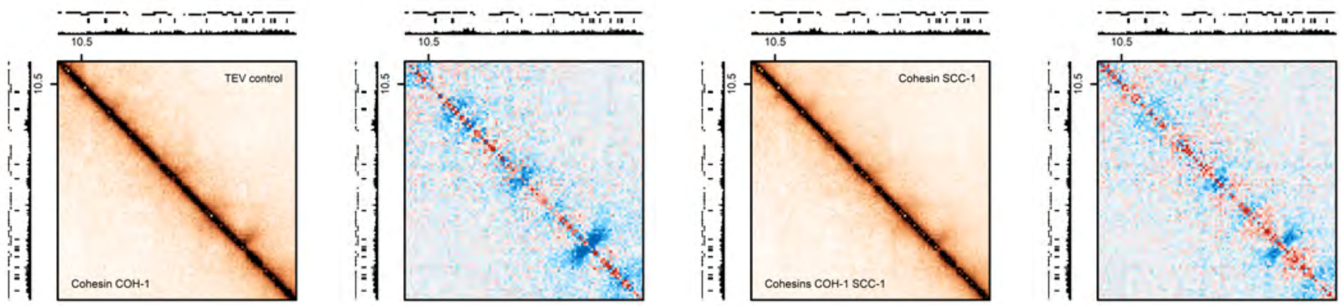

chrX:14639001-14839000

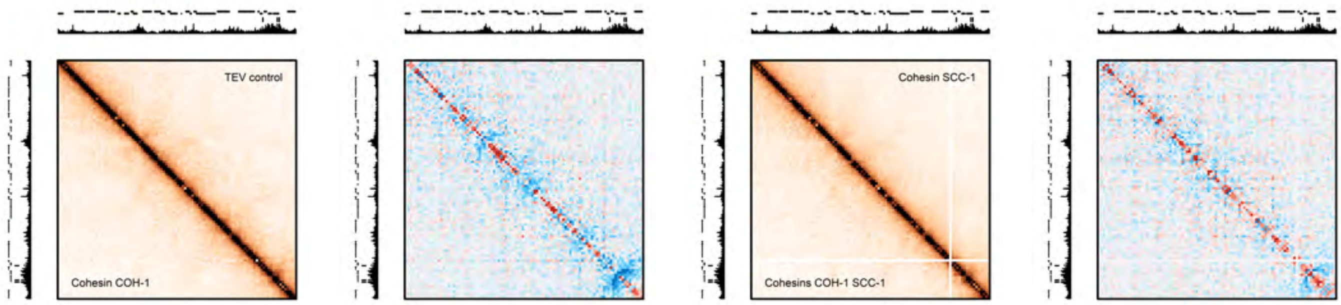

chrX:9273001-9473000

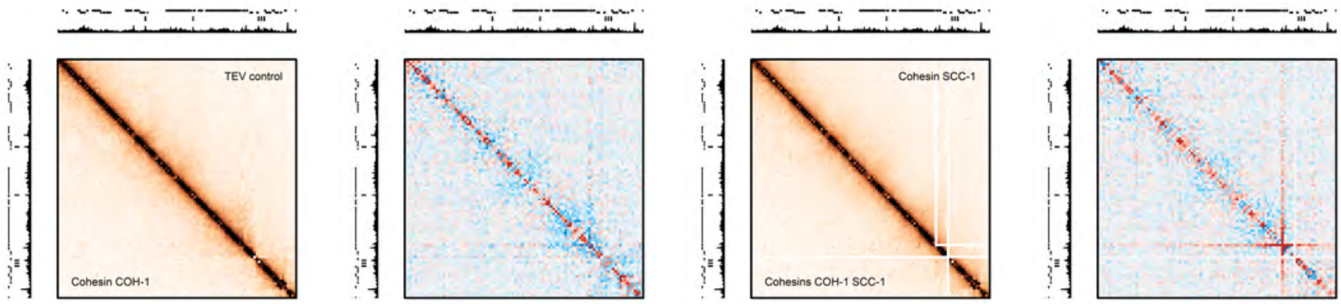

chrIV:11425001-11625000

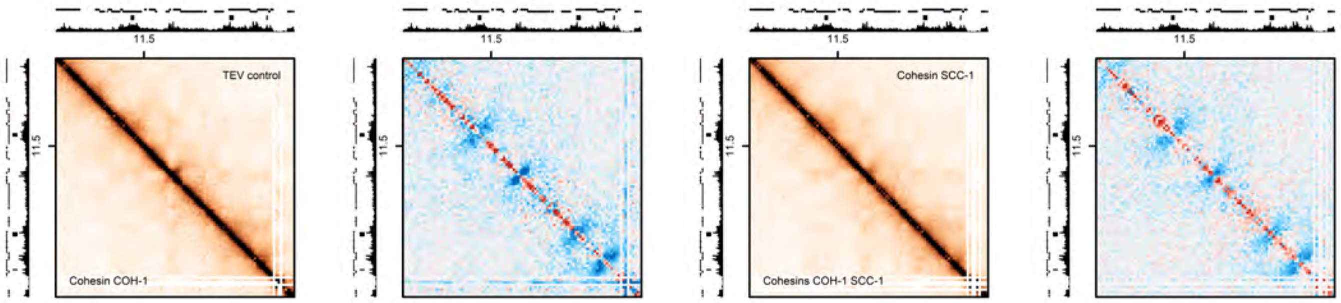

chrII:11741001-11941000

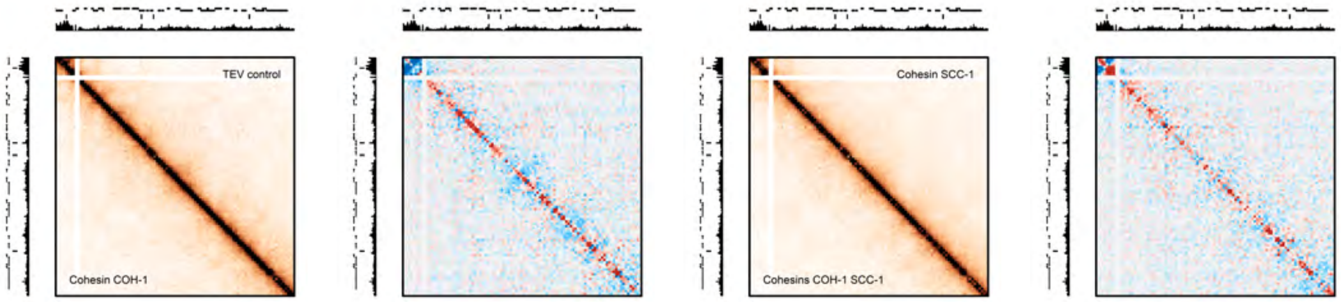

chrX:13581001-13781000

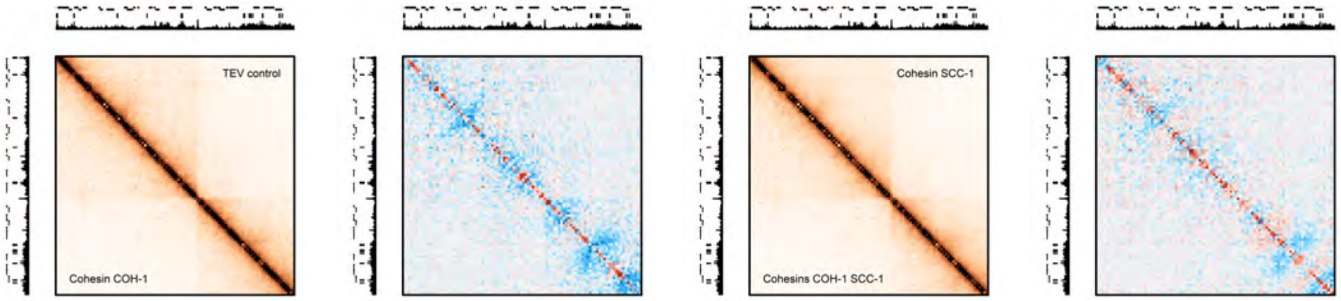

chrX:12349001-12549000

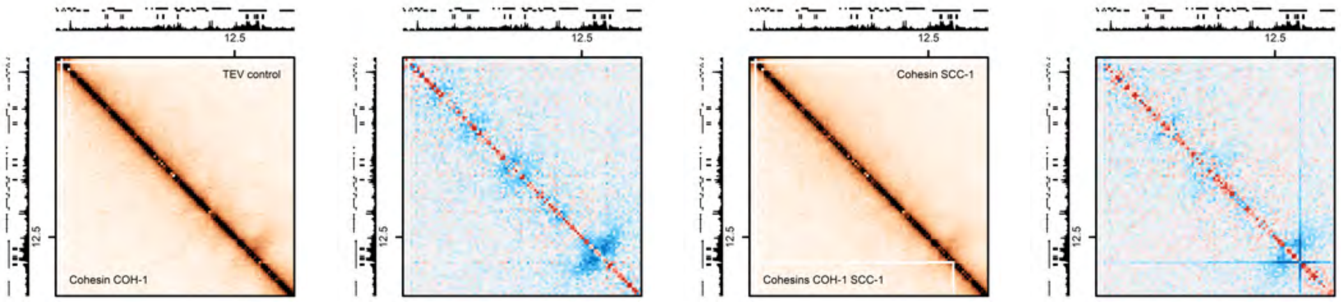

chrIII:8773001-8973000

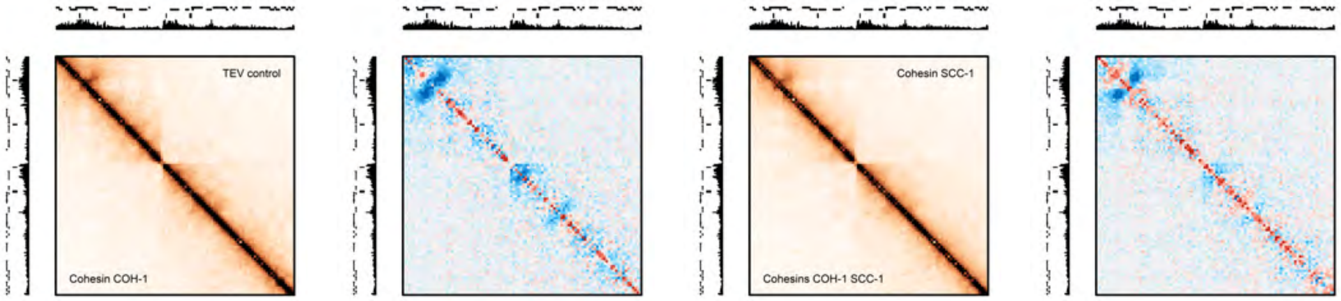

chrX:13223001-13423000

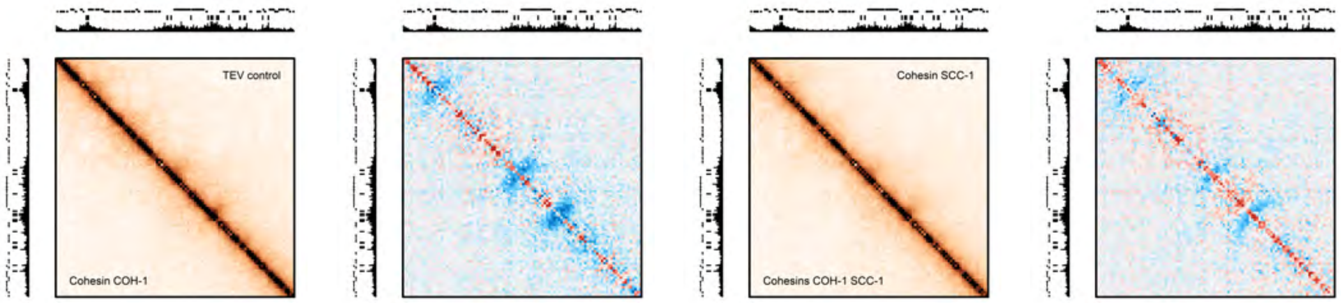

chrIV:6859001-7059000

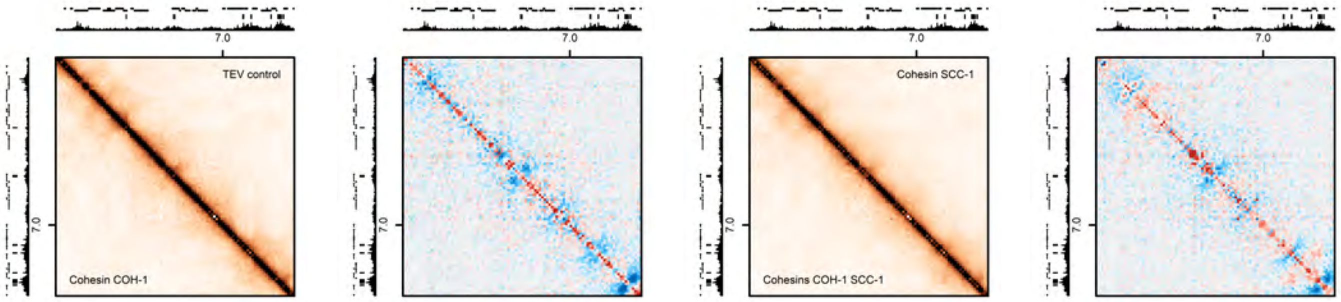

chrII:5785001-5985000

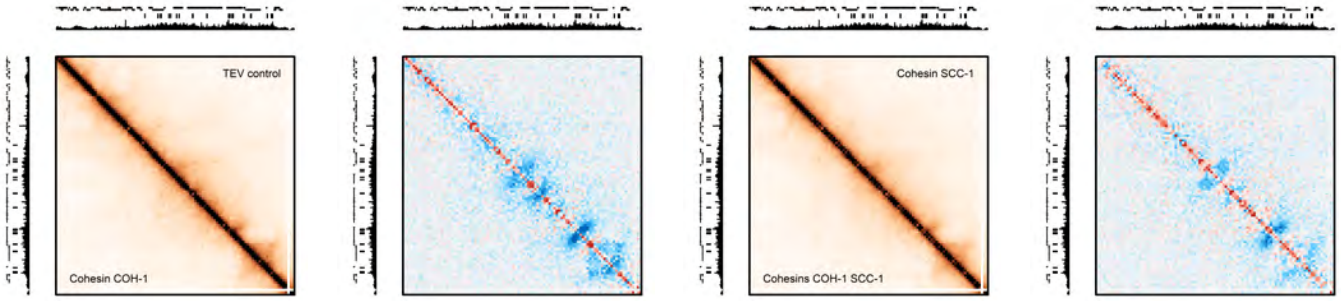

chrI:6737001-6937000

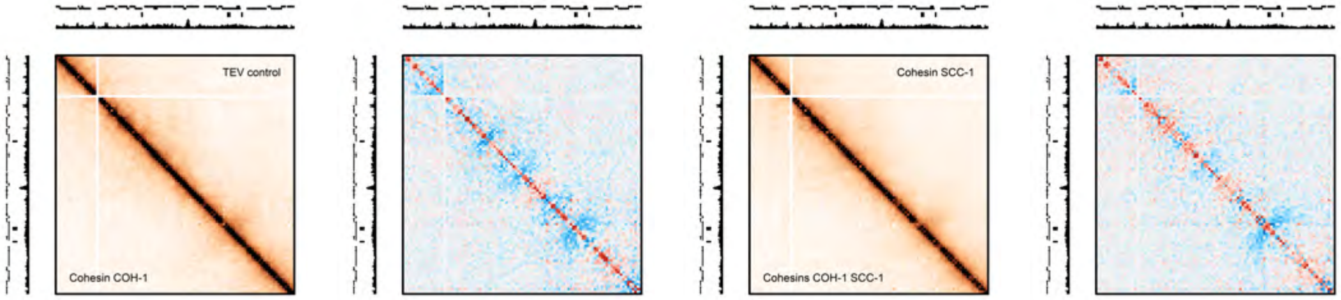

chrI:13853001-14053000

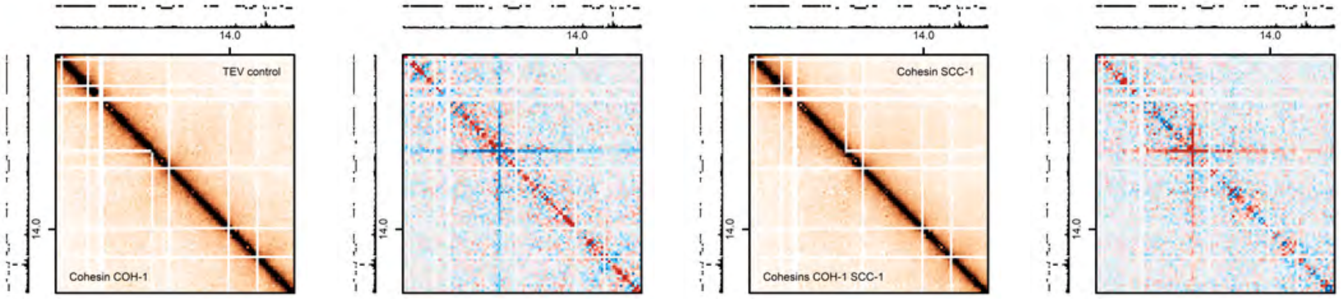

chrIII:13001001-13201000

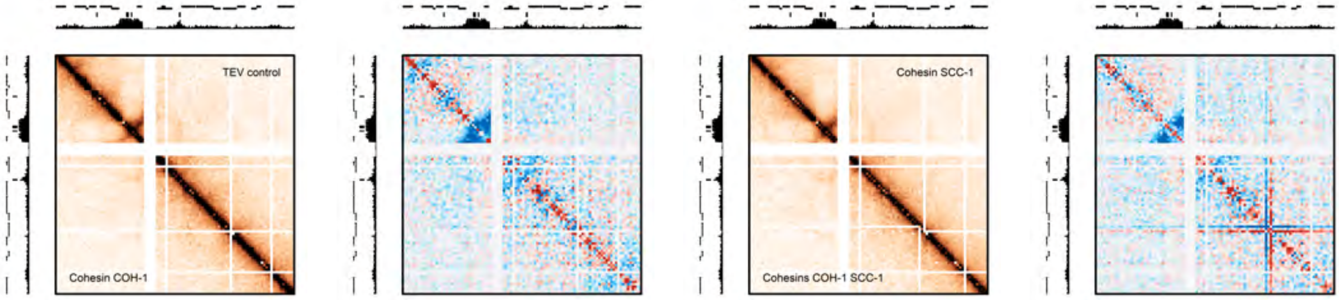

chrIV:10513001-10713000

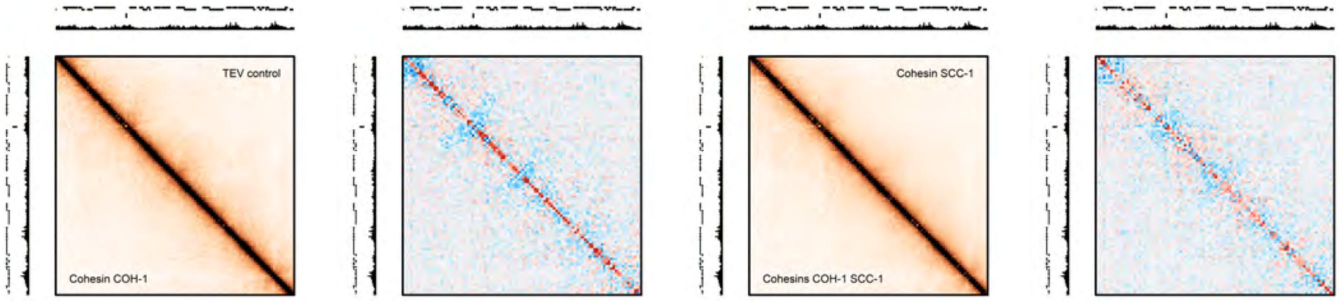

chrX:2219001-2419000

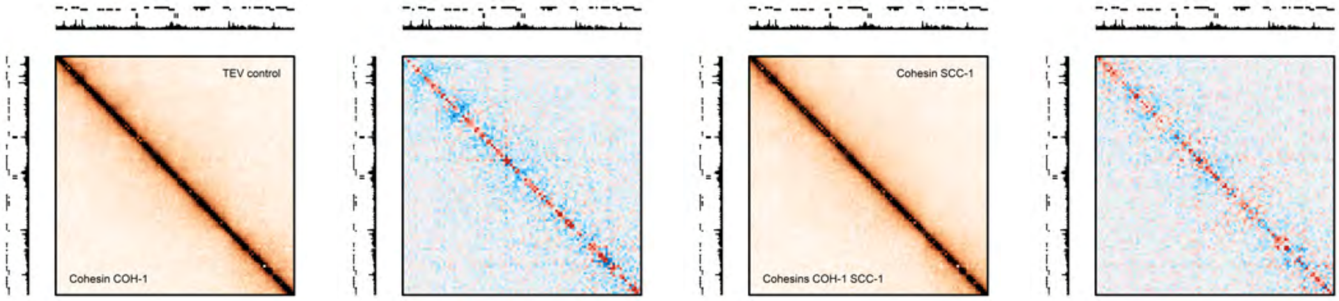

chrIV:4459001-4659000

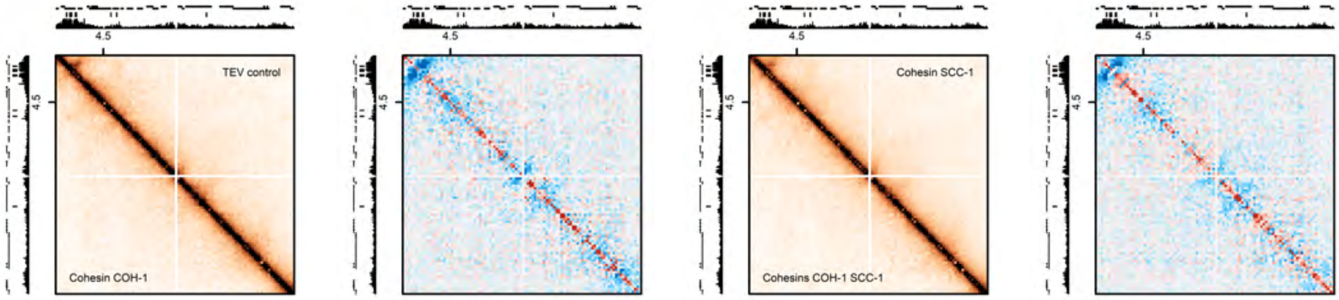

chrIII:4625001-4825000

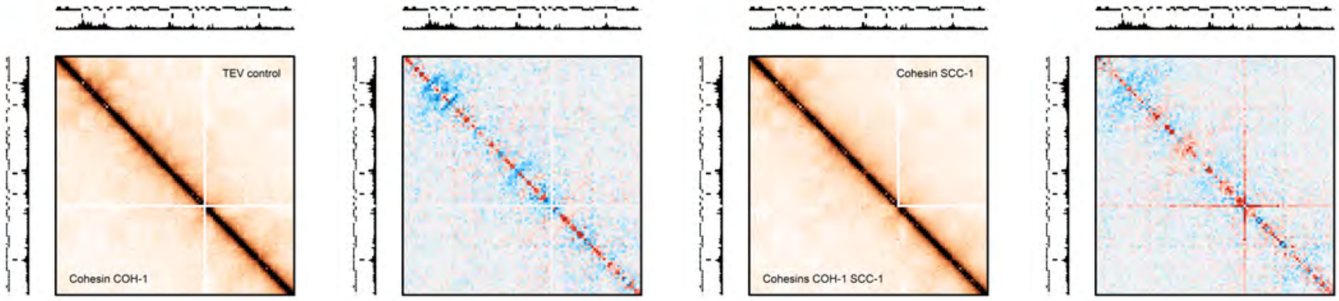

chrV:5423001-5623000

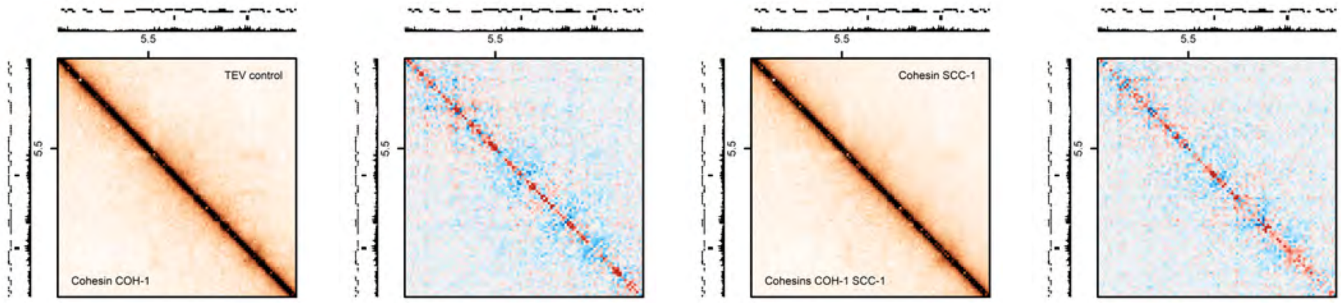

chrI:13589001-13789000

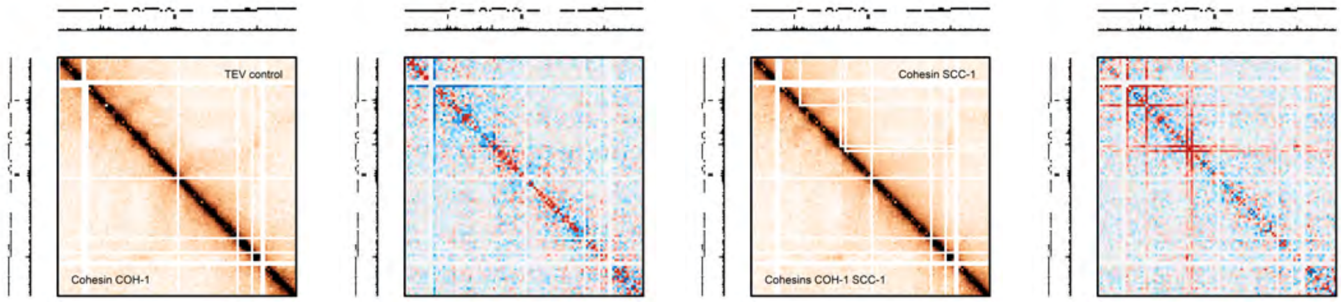

chrIII:889001-1089000

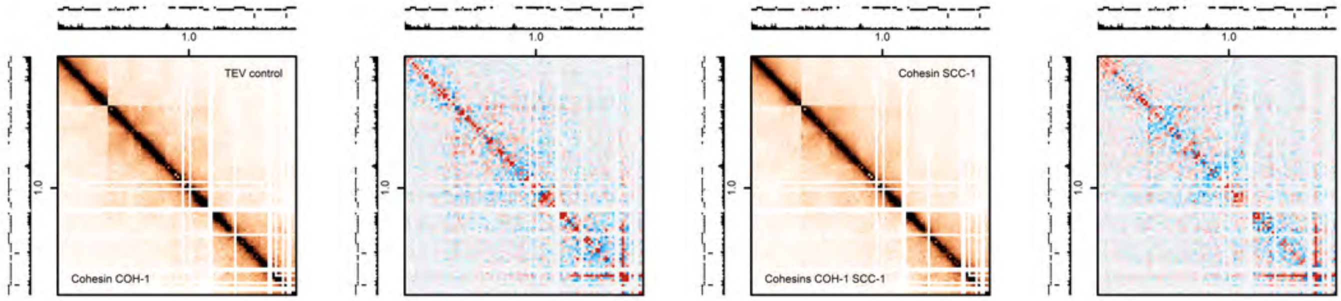

chrX:6495001-6695000

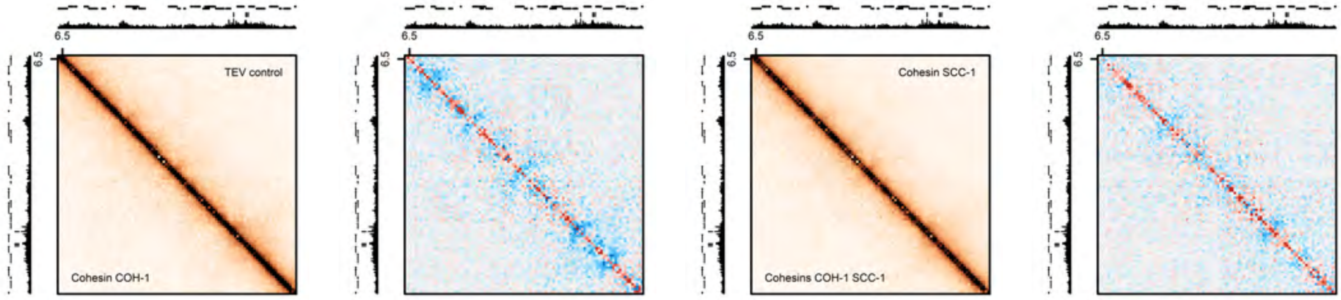

chrI:12247001-12447000

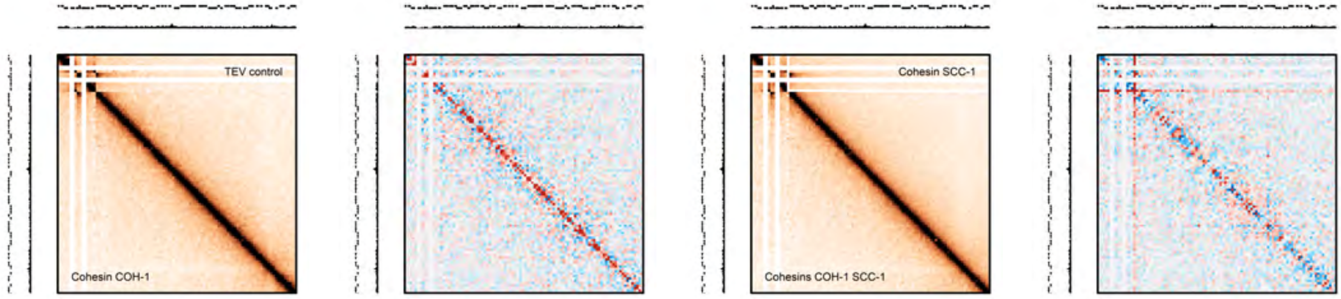

chrX:10747001-10947000

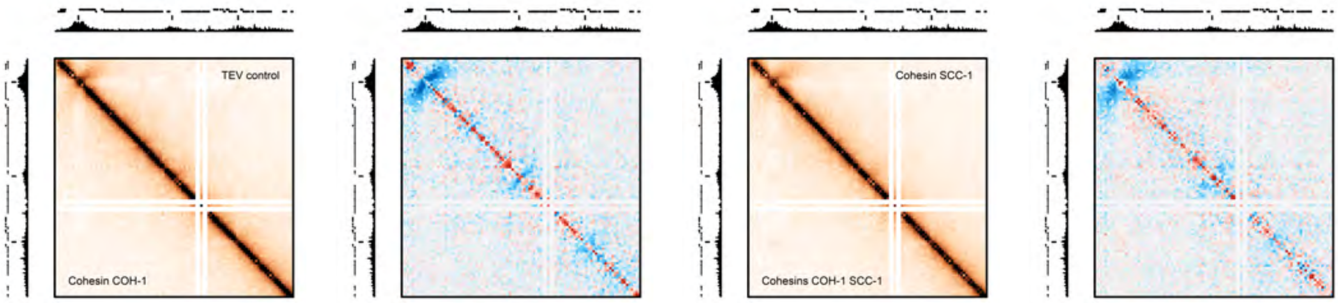

chrII:8123001-8323000

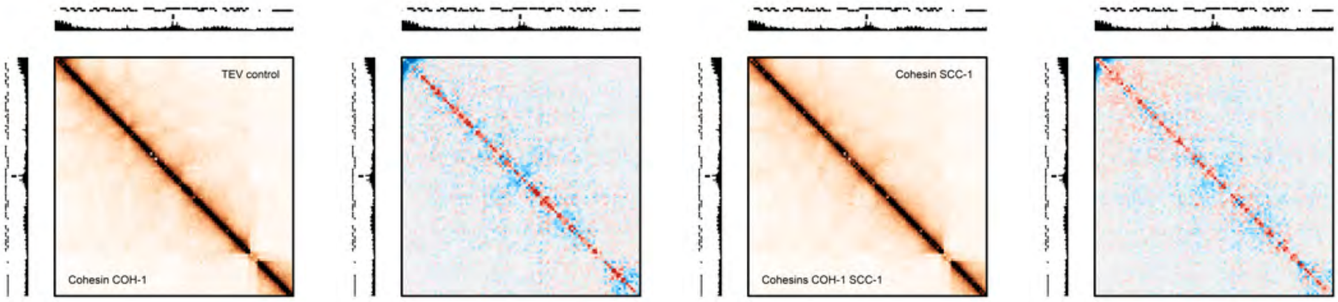

chrII:7885001-8085000

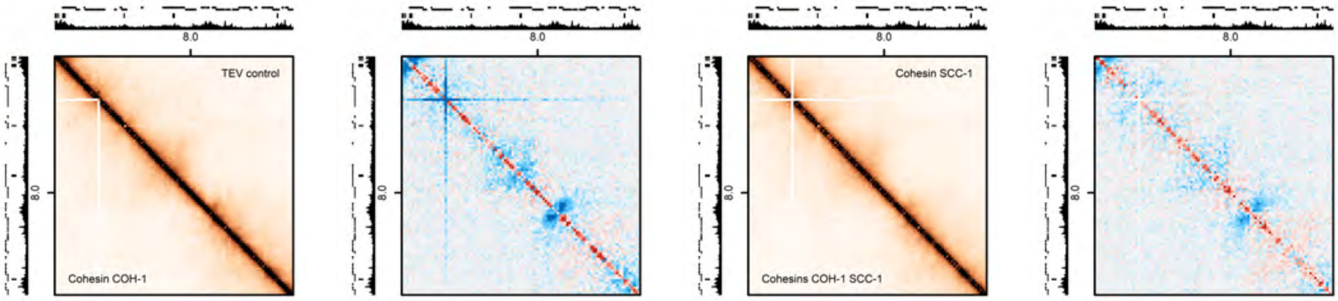

chrIV:6781001-6981000

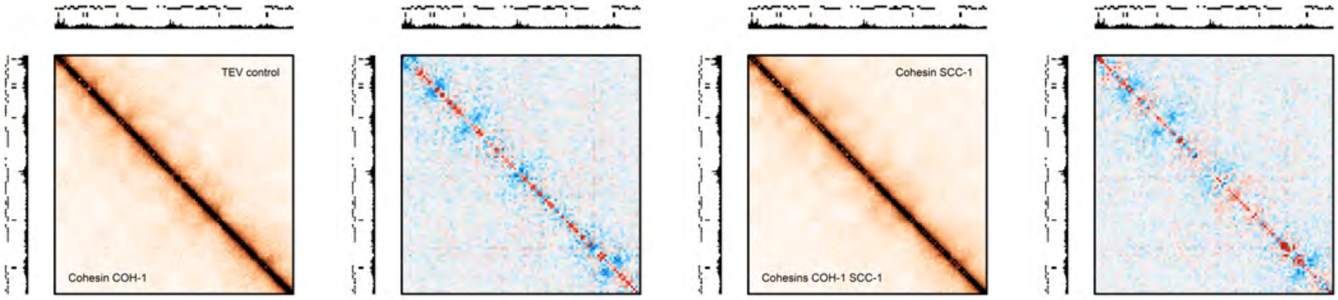

chrII:9791001-9991000

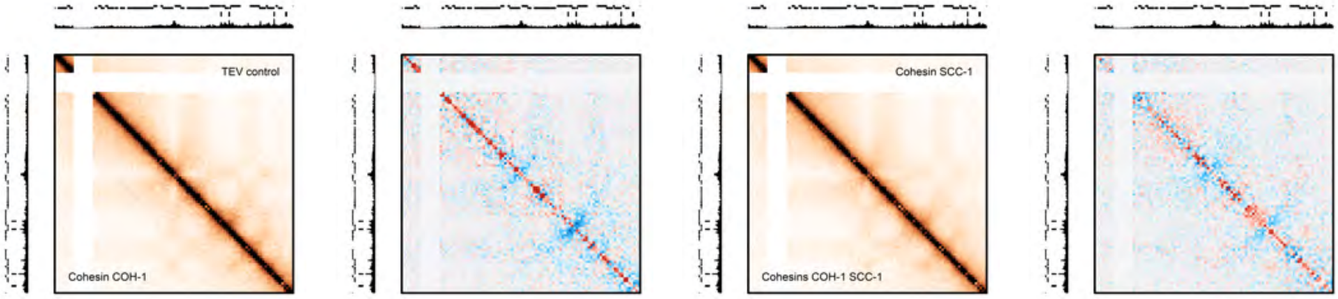

chrII:1683001-1883000

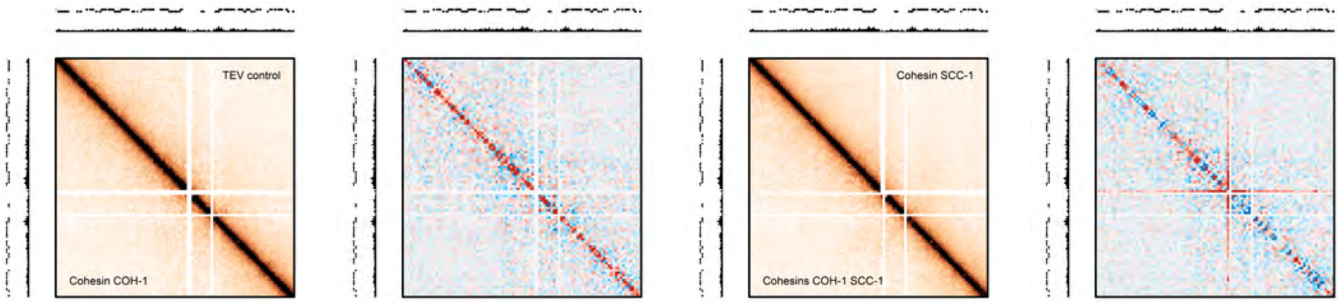

chrV:18899001-19099000

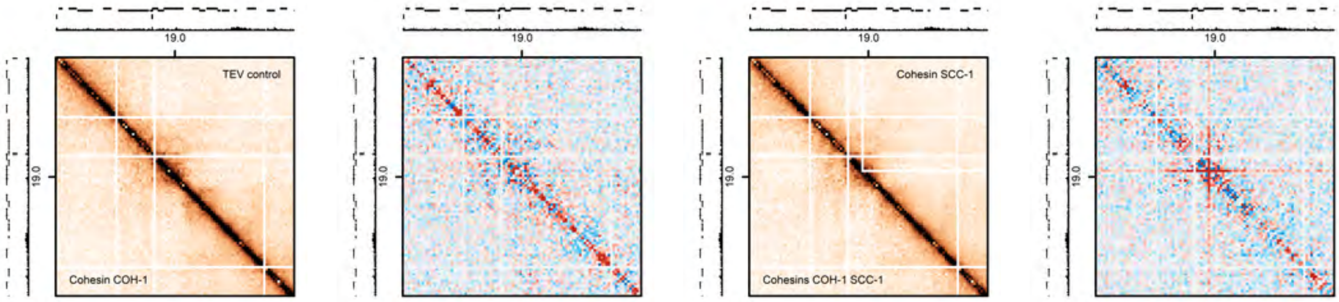

chrIII:7797001-7997000

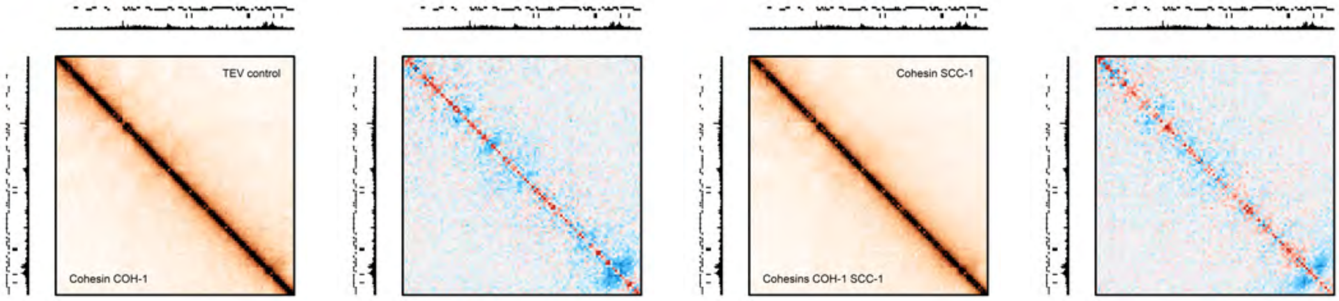

chrII:11837001-12037000

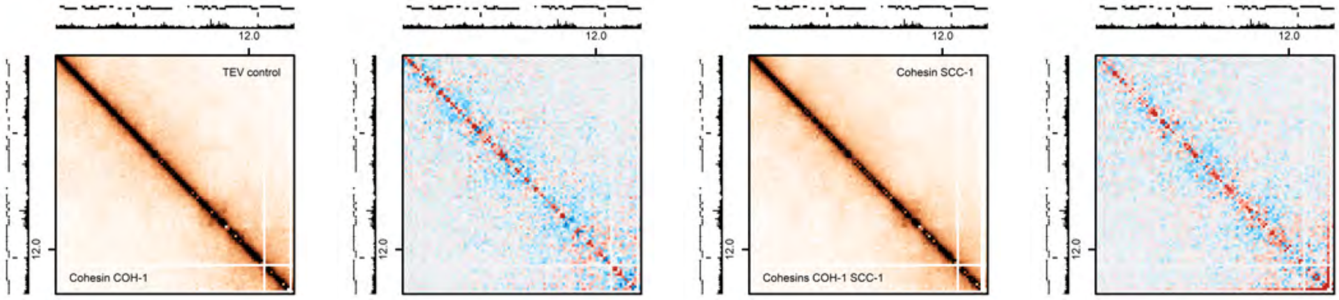

chrV:6367001-6567000

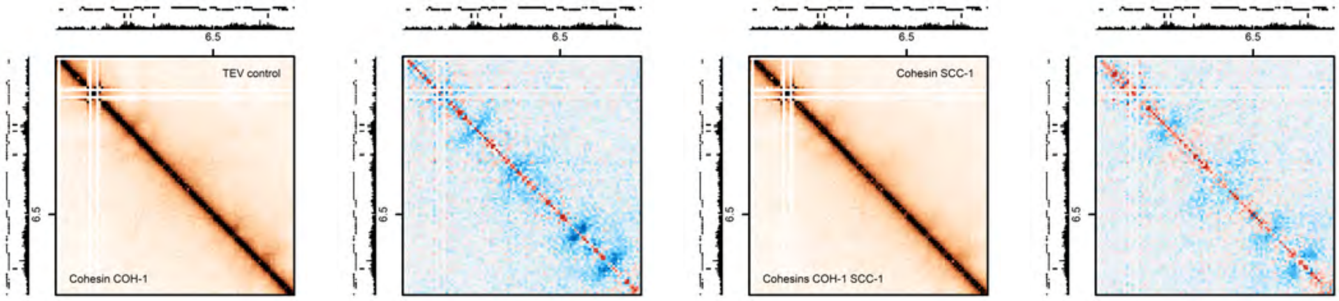

chrIII:10805001-11005000

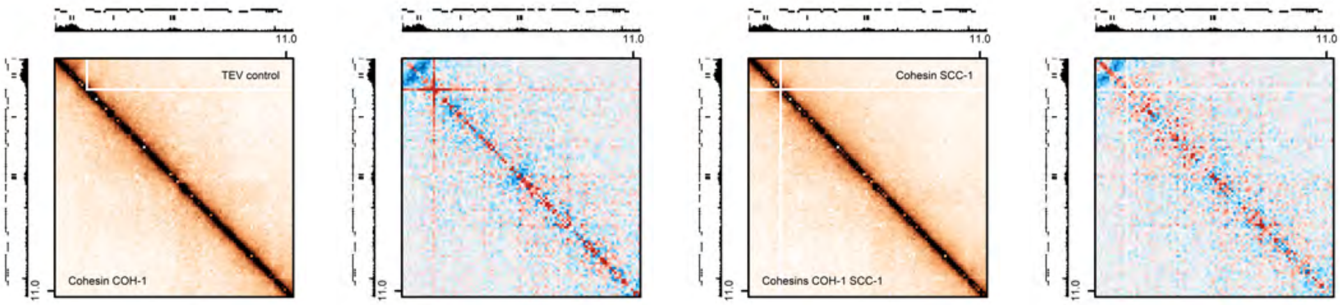

chrV:17345001-17545000

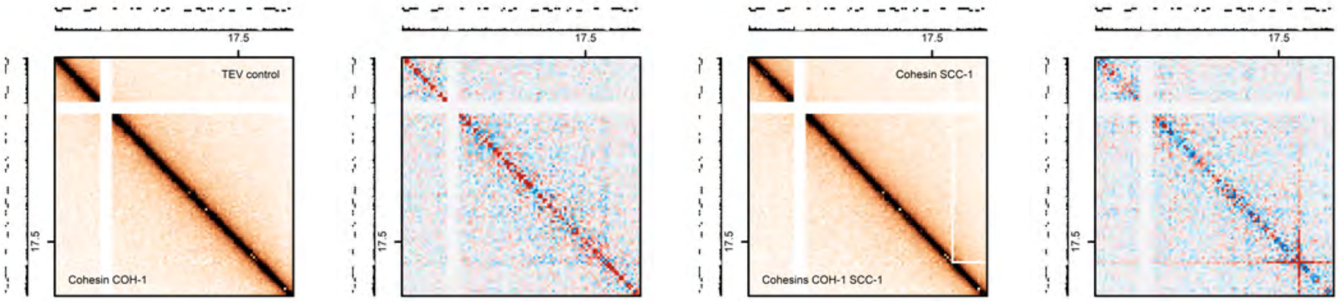

chrV:9411001-9611000

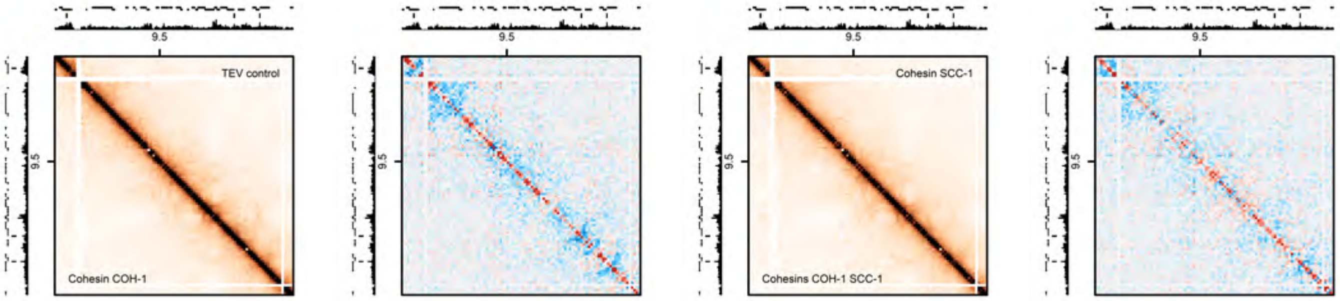

chrIII:8475001-8675000

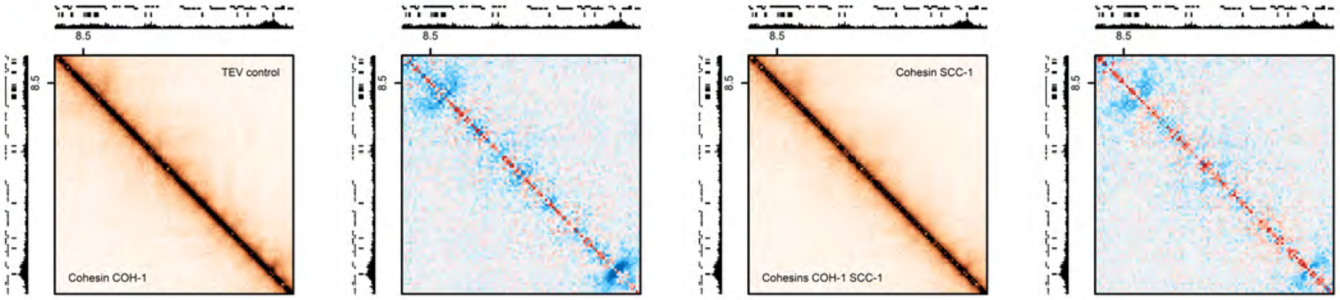

chrV:6599001-6799000

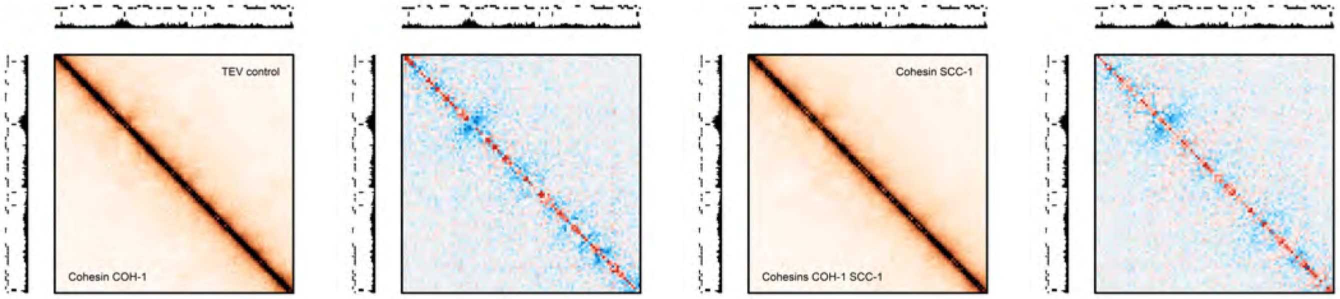

chrV:13369001-13569000

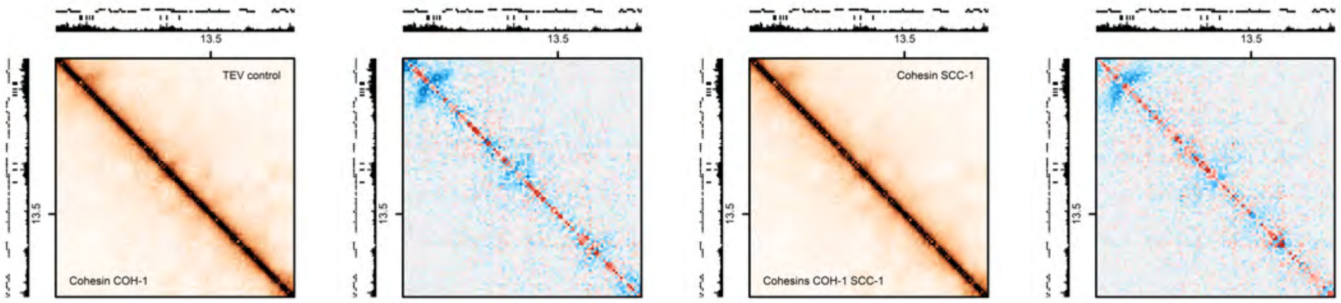

chrII:10943001-11143000

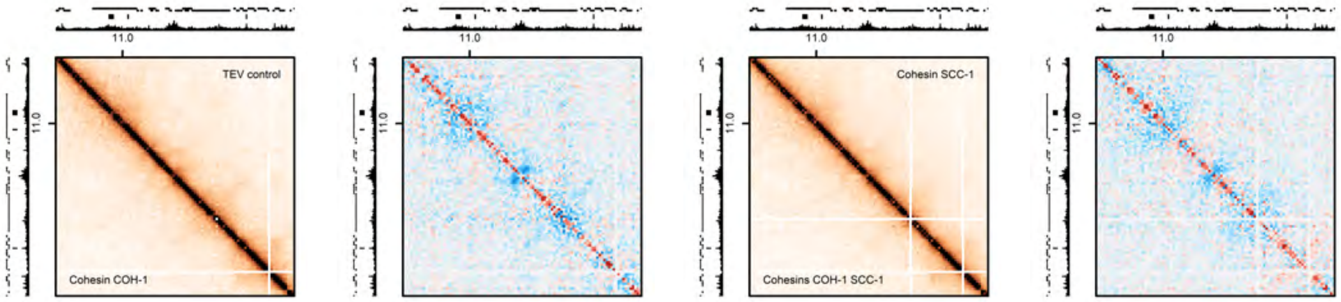

chrIII:2967001-3167000

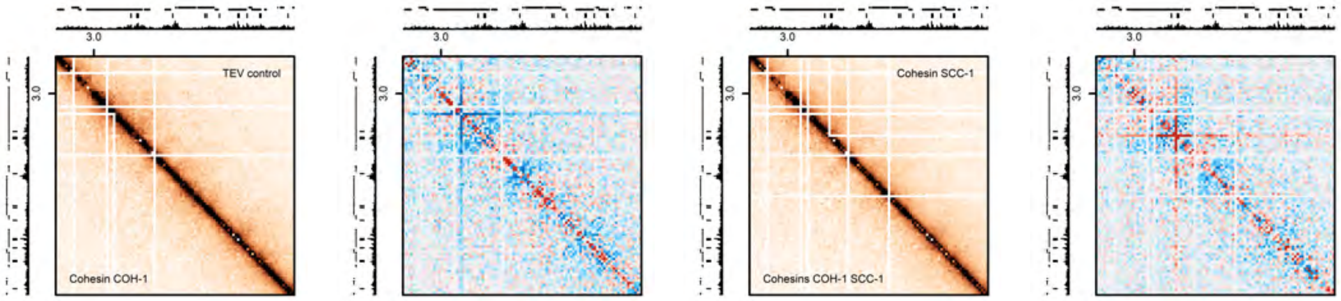

chrX:13777001-13977000

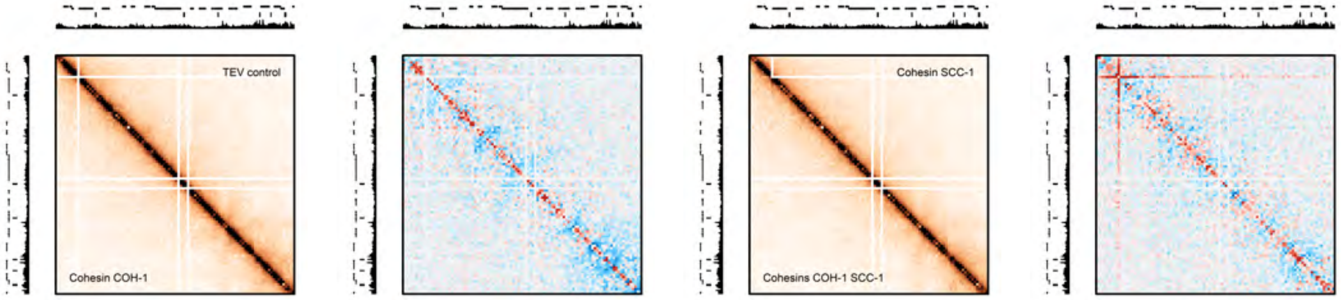

chrI:12871001-13071000

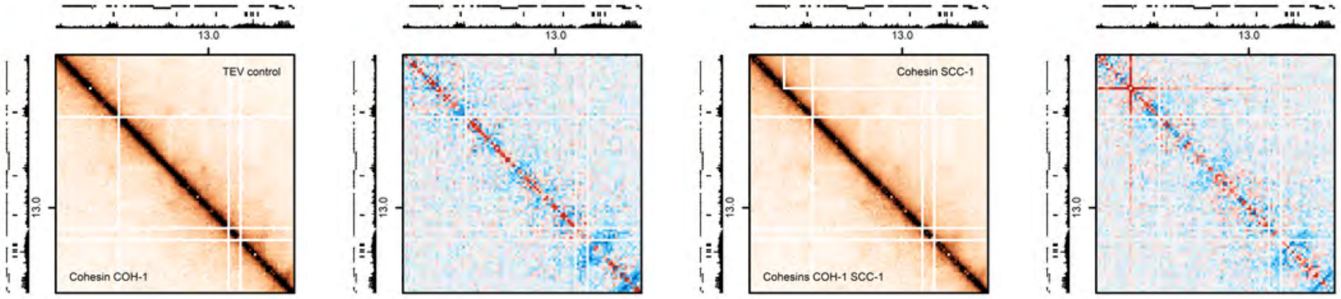

chrIII:5659001-5859000

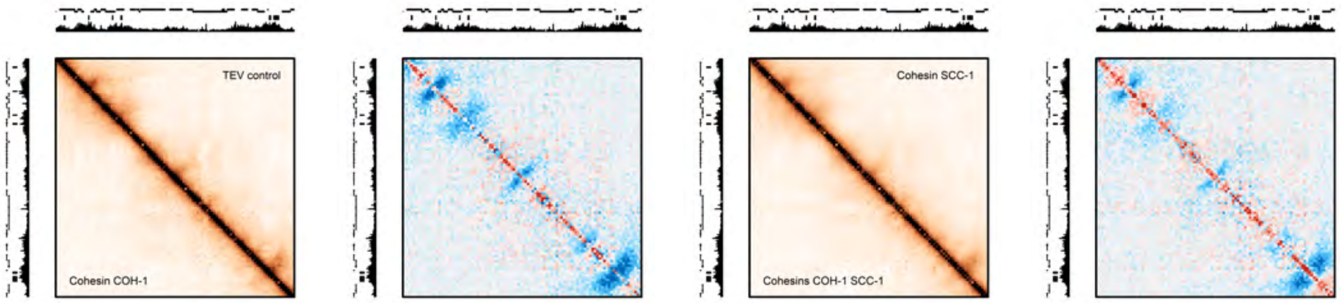

chrII:8485001-8685000

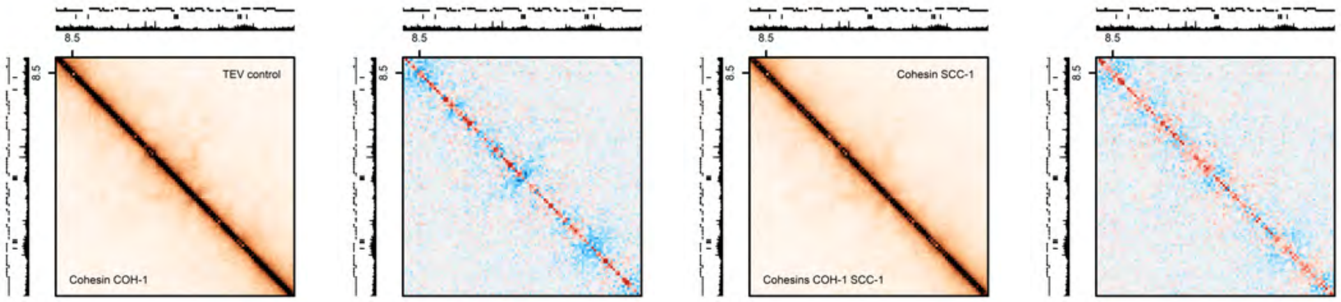

chrIV:11767001-11967000

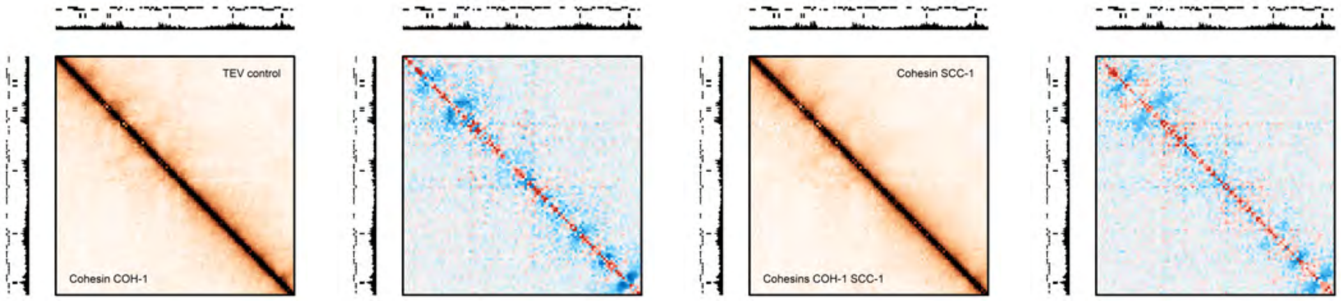

chrIII:3333001-3533000

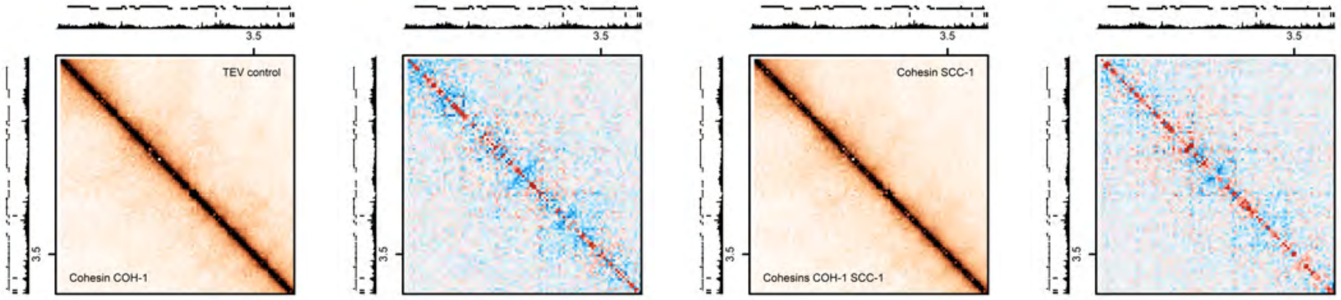

chrX:919001-1119000

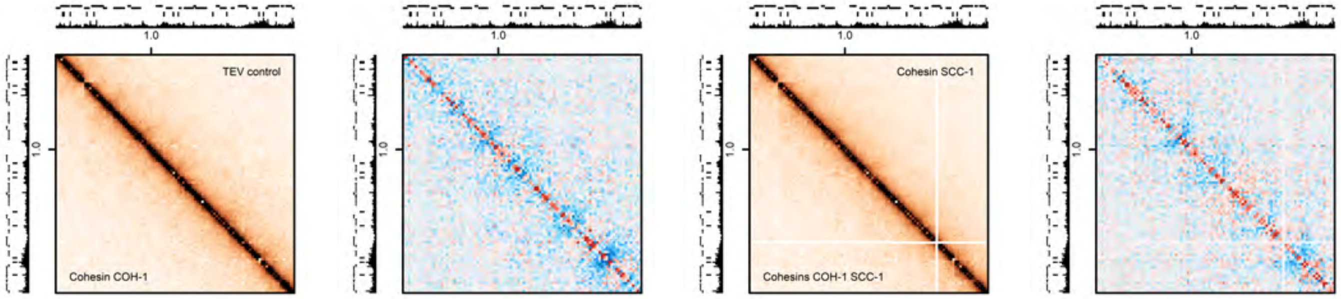

chrV:19317001-19517000

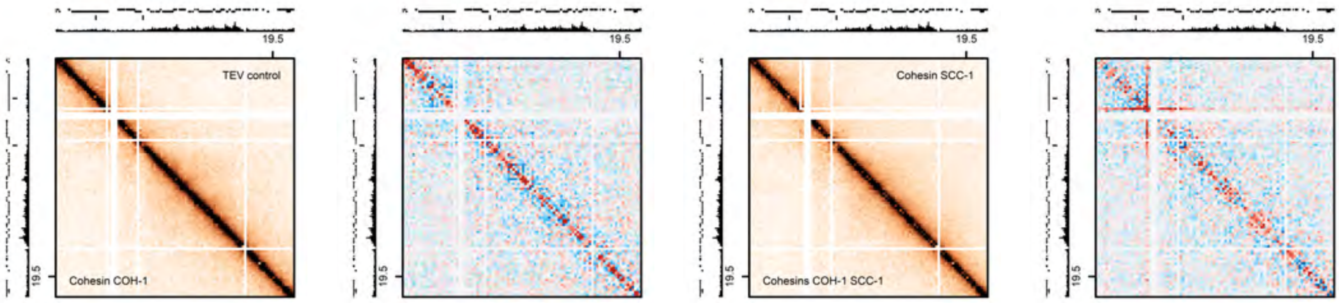

chrV:5171001-5371000

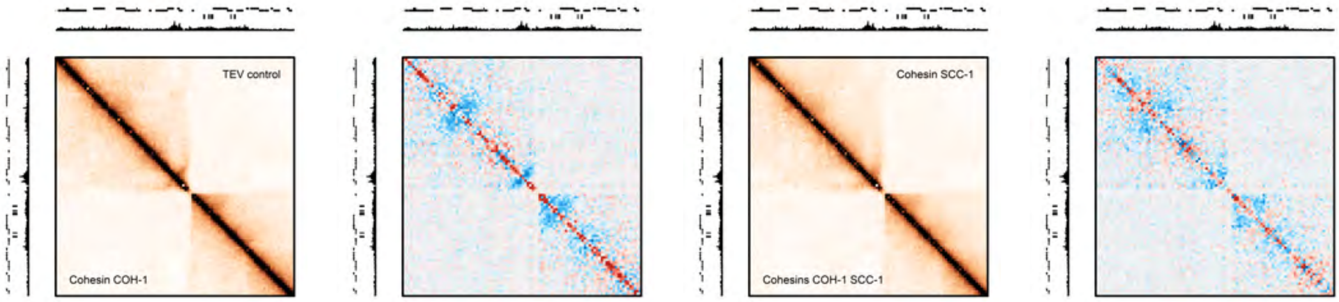

chrV:6007001-6207000

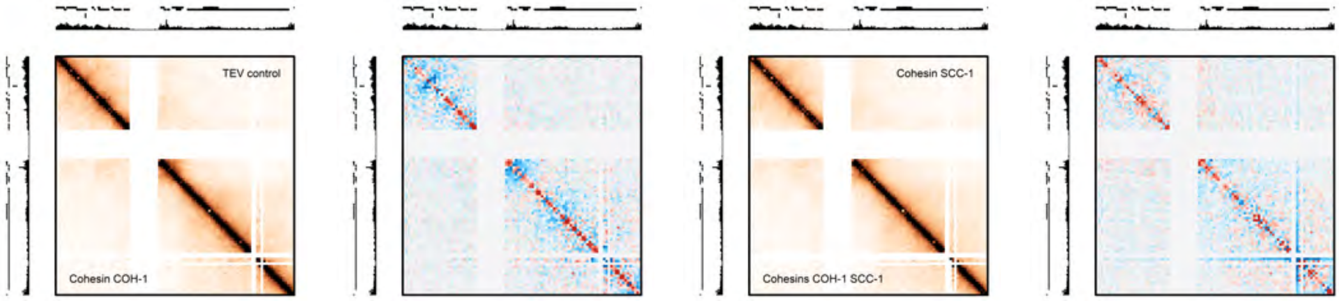

chrV:7631001-7831000

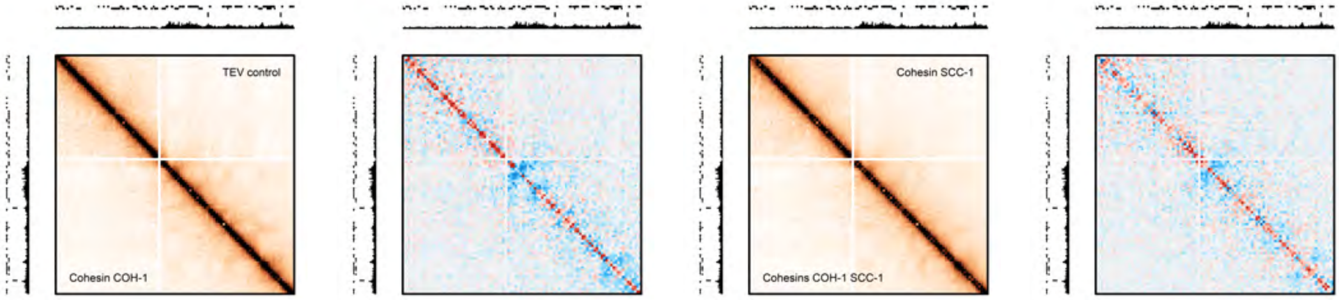

chrX:1403001-1603000

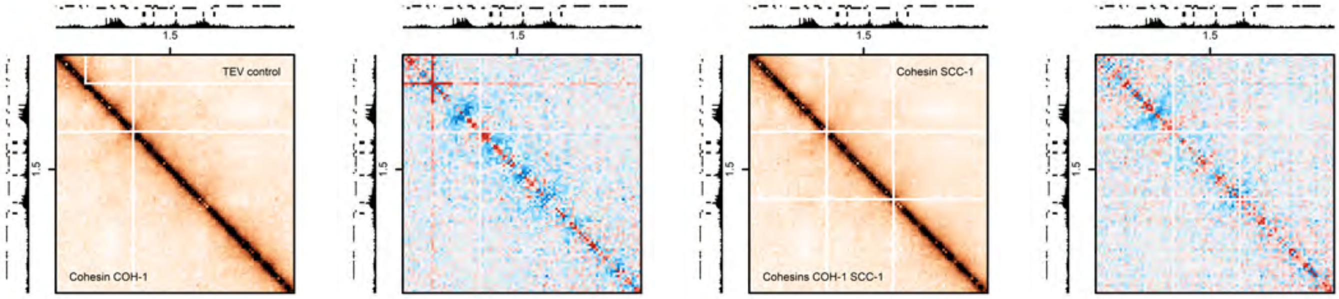

chrIV:12081001-12281000

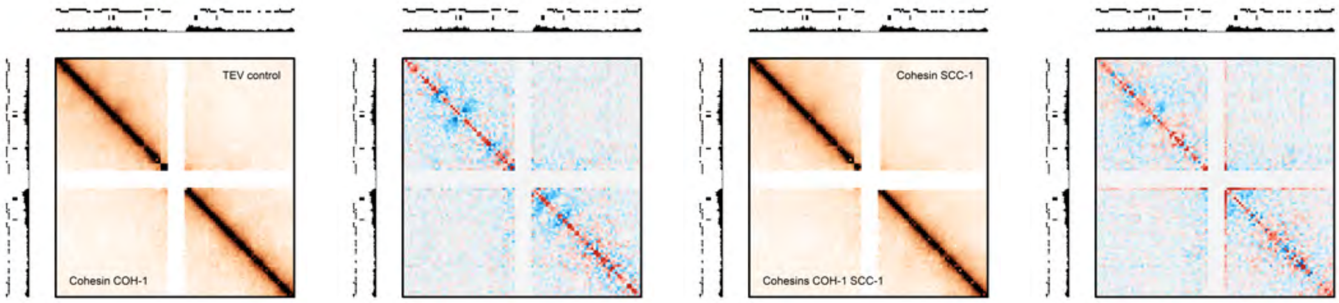

chrII:5279001-5479000

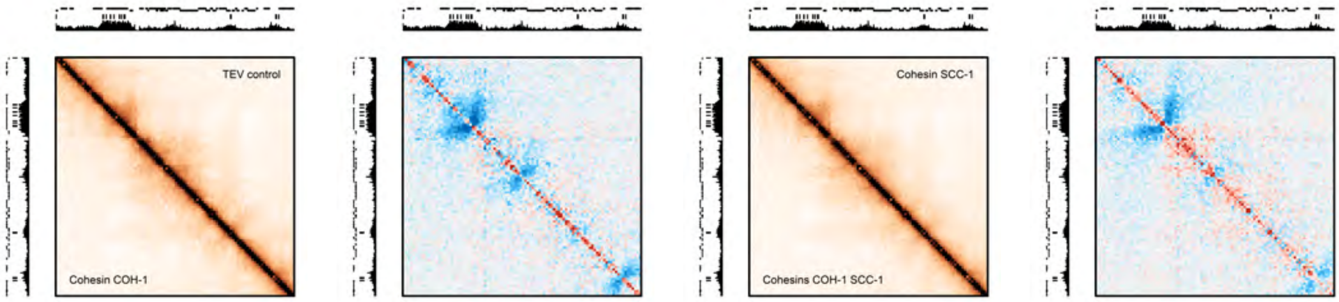

chrI:14873001-15073000

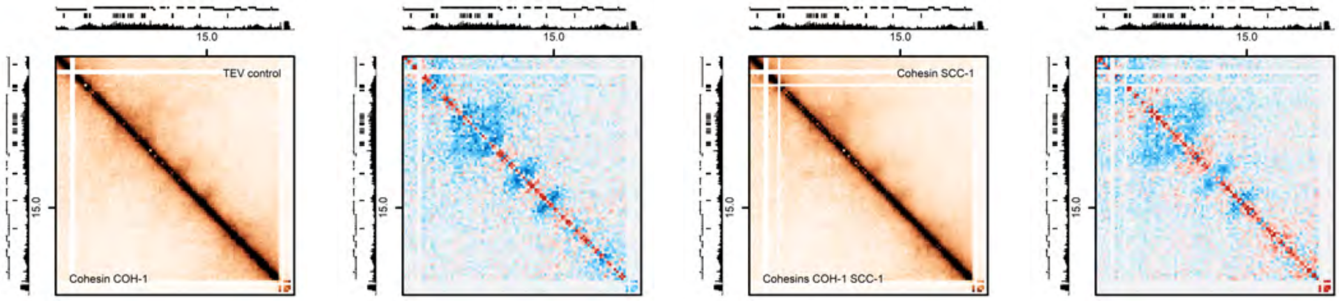

chrX:11985001-12185000

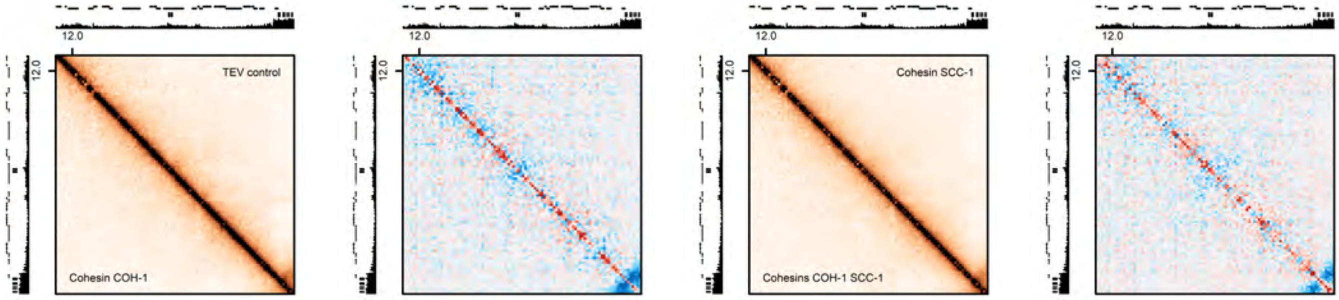

chrII:14943001-15143000

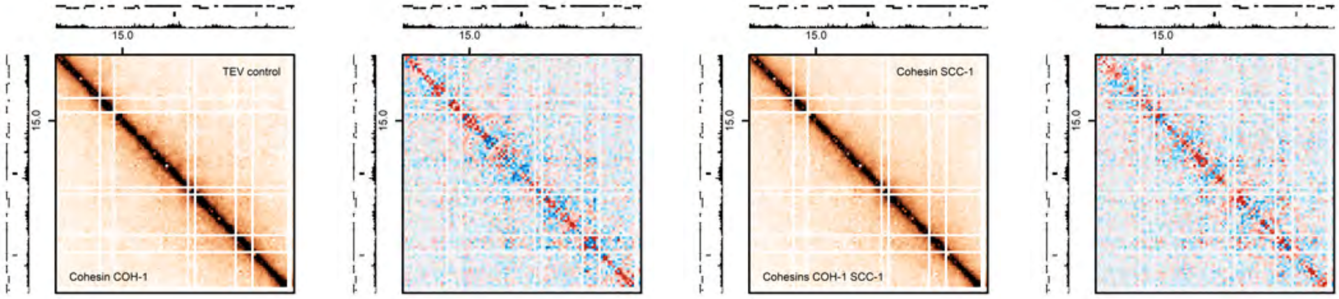

chrIV:16195001-16395000

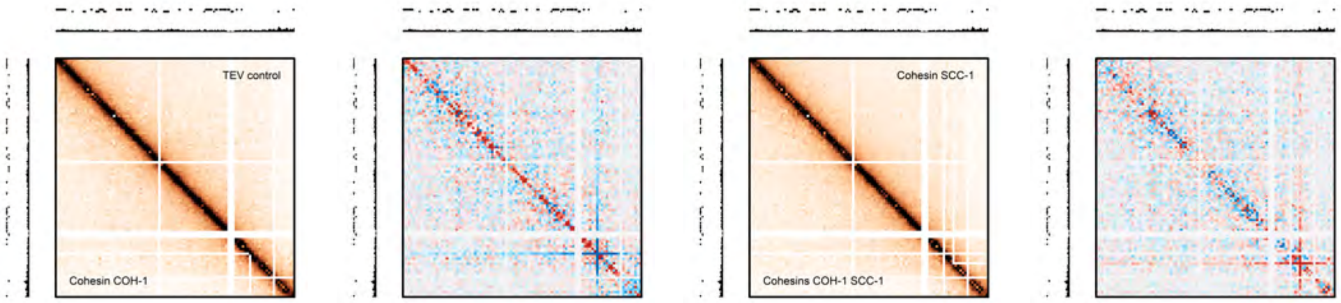

chrX:2947001-3147000

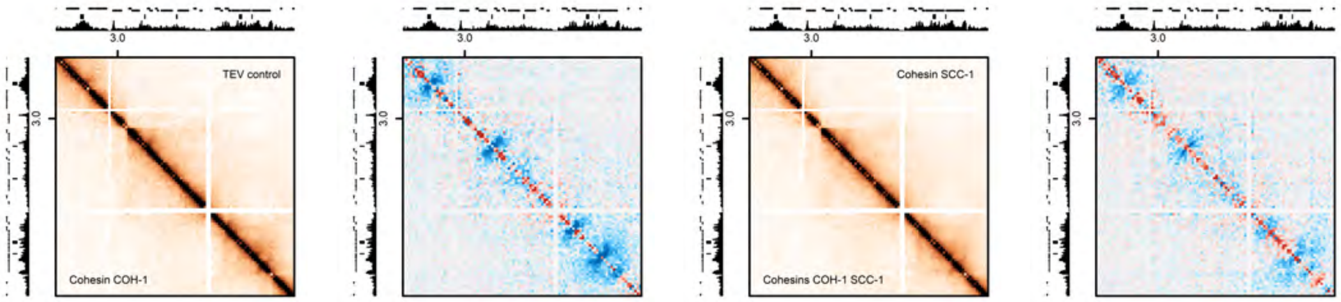

chrIV:9961001-10161000

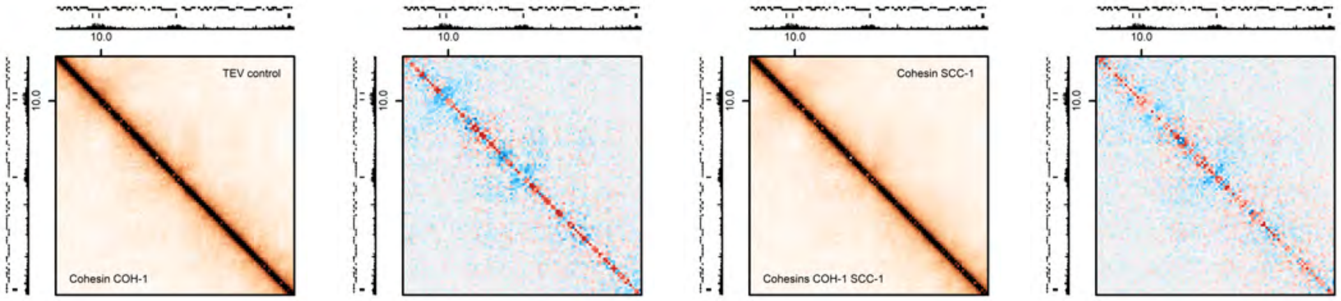

chrII:7129001-7329000

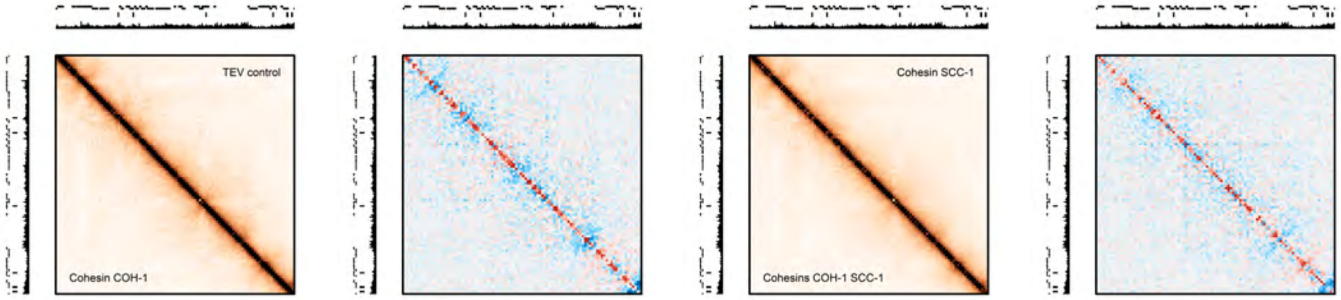

chrI:7027001-7227000

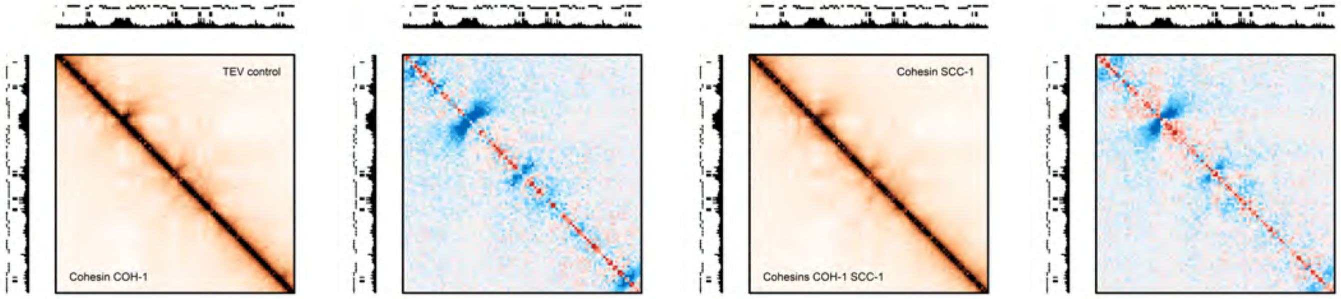

chrIII:6797001-6997000

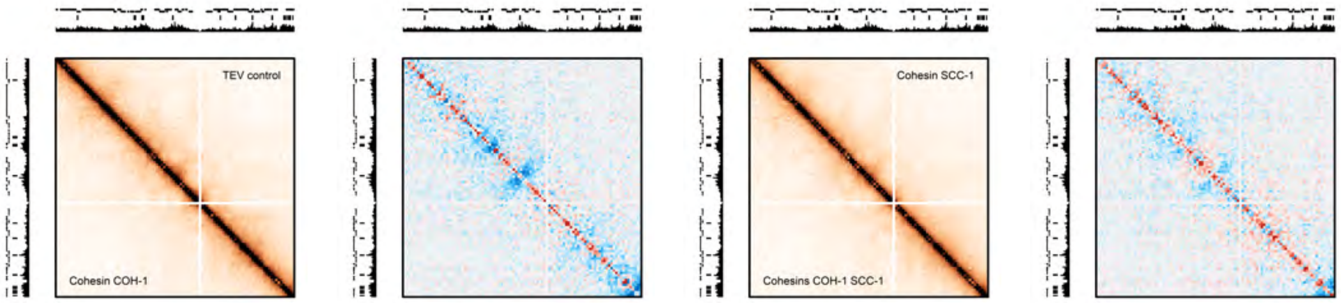

chrI:9445001-9645000

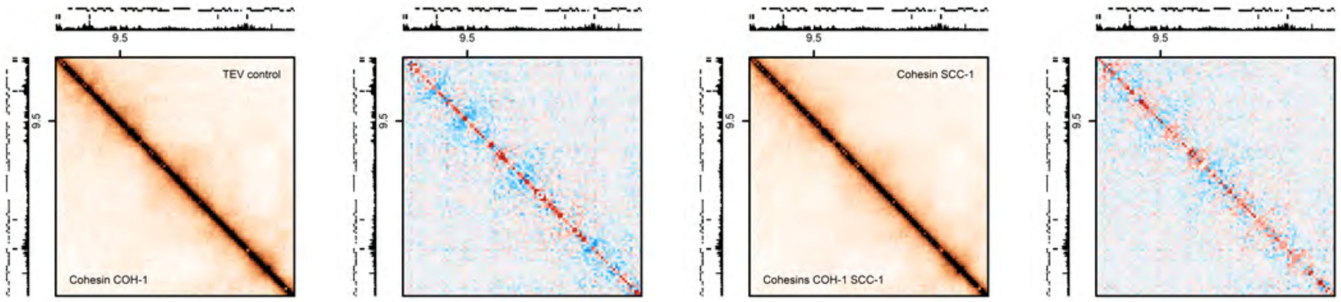

chrX:7765001-7965000

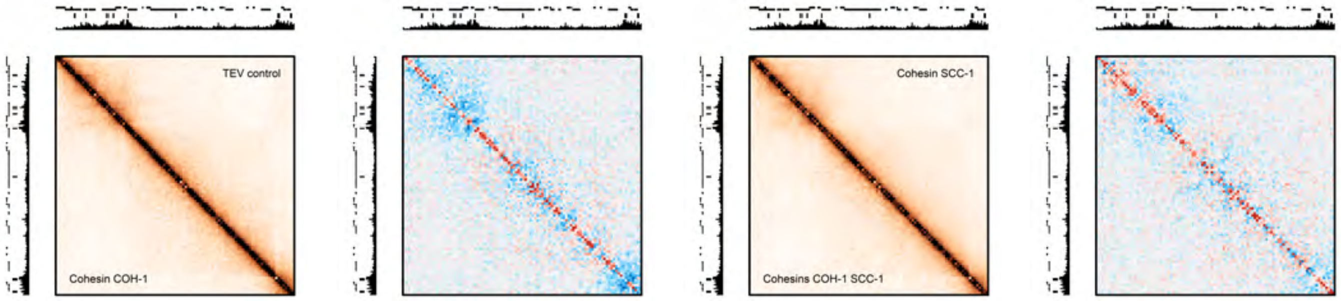

chrV:9753001-9953000

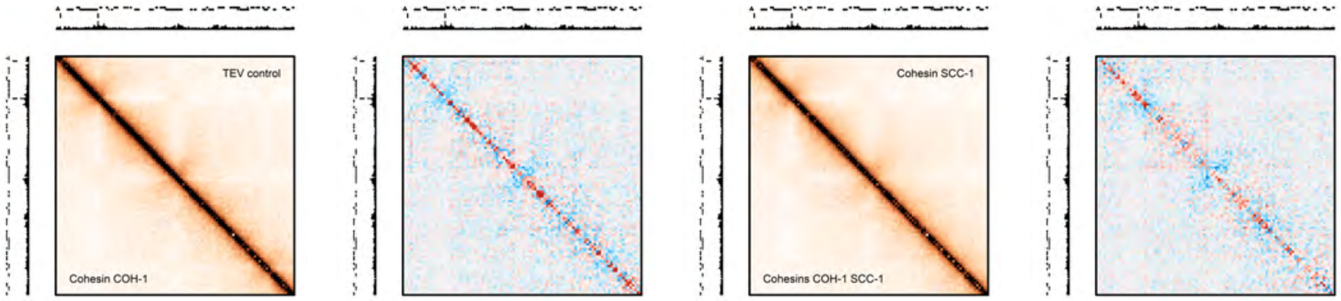

chrV:17223001-17423000

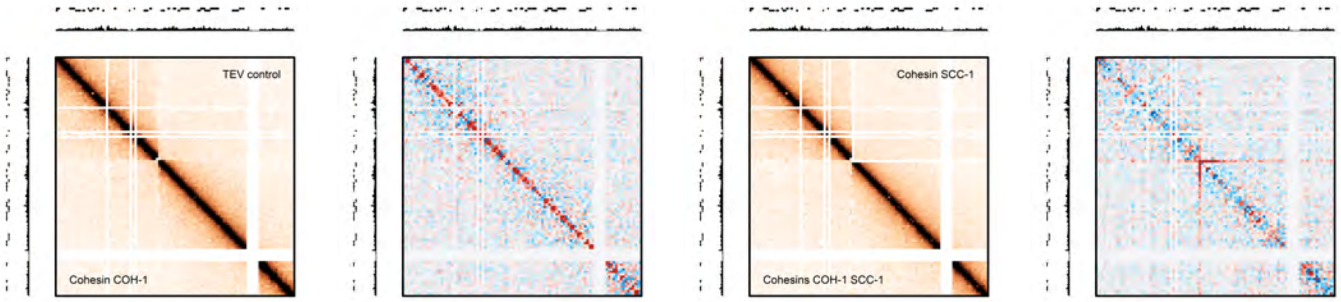

chrX:17229001-17429000

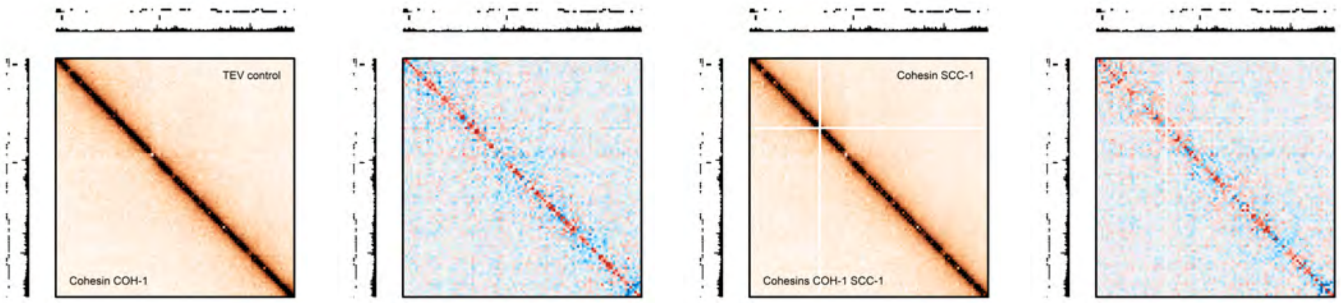

chrII:11783001-11983000

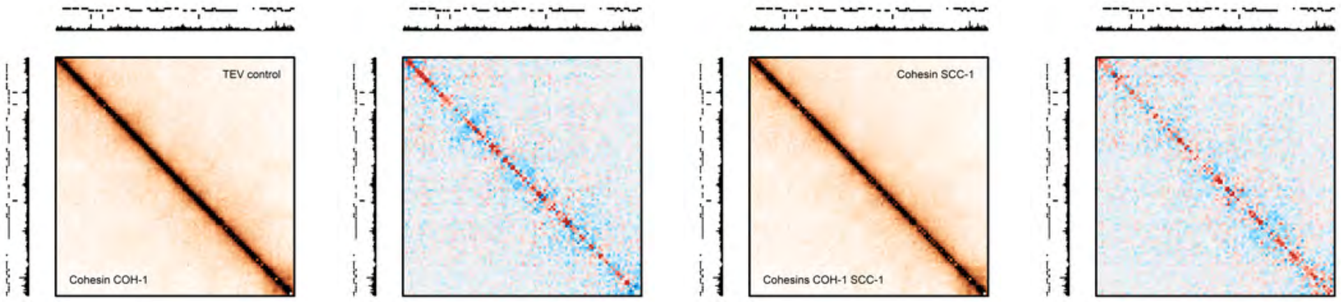

chrX:1295001-1495000

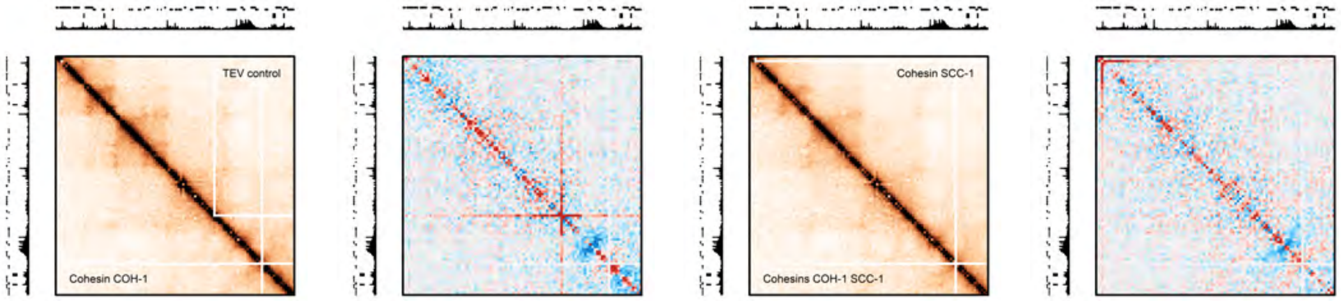

chrX:10051001-10251000

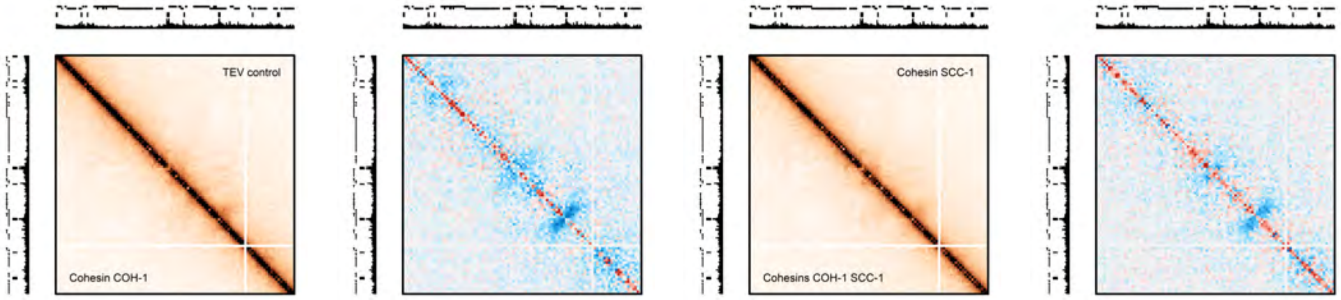

chrI:2083001-2283000

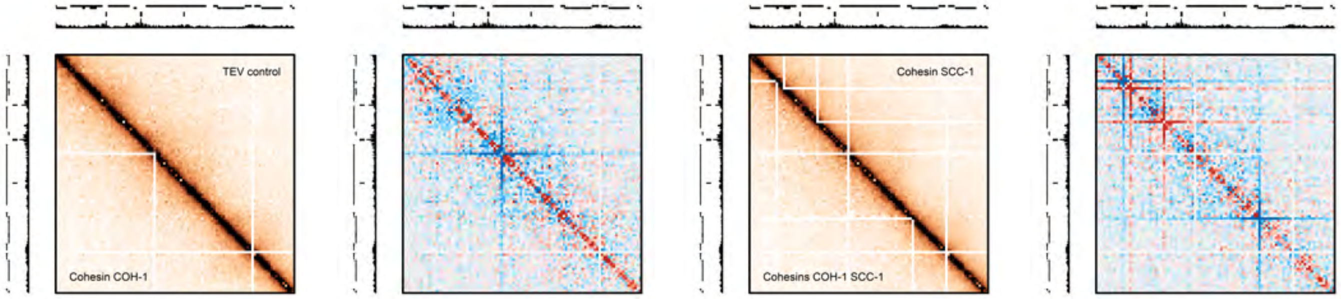

chrV:3723001-3923000

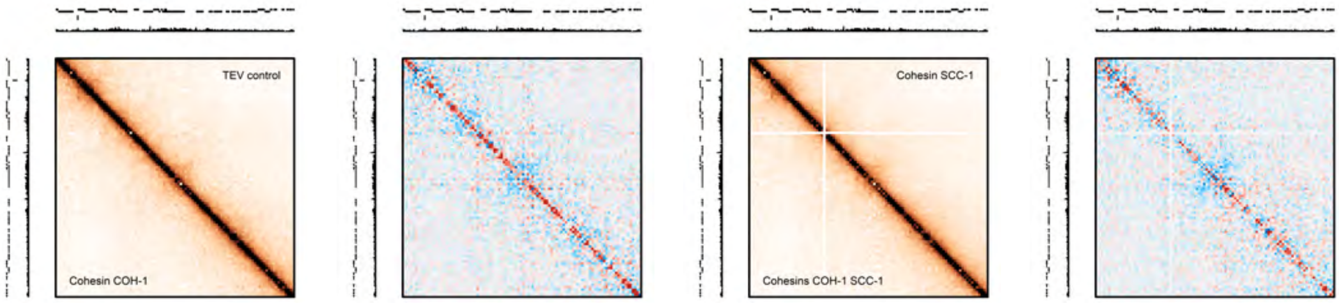

chrX:16253001-16453000

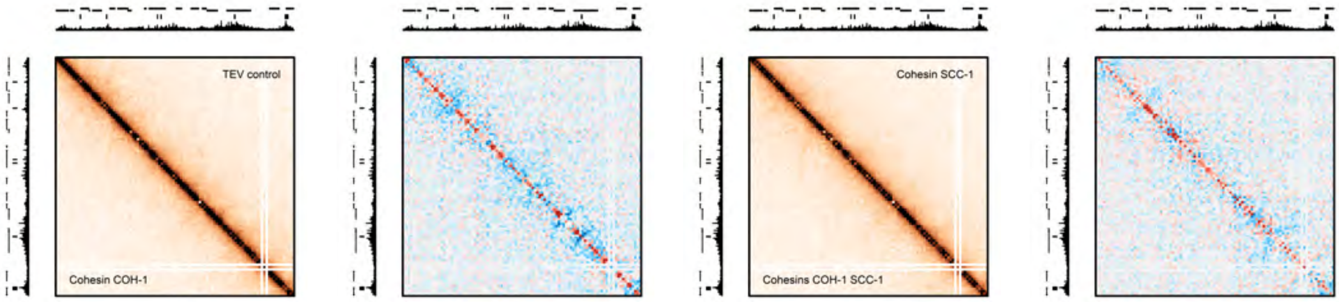

chrIII:7107001-7307000

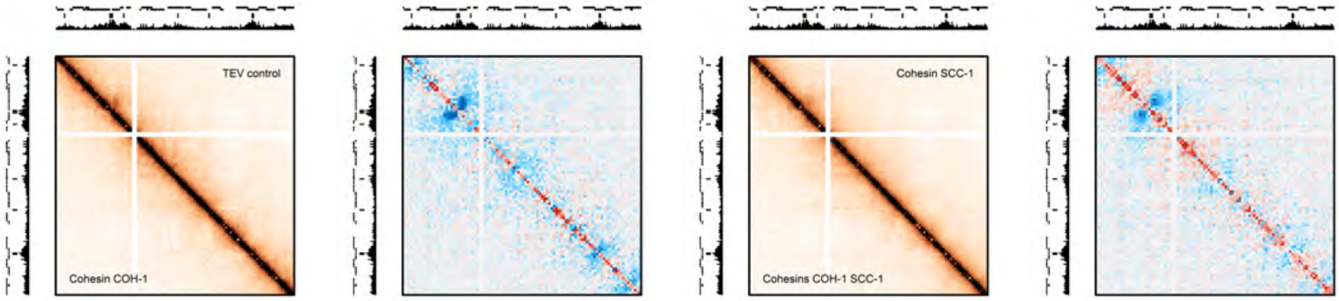

chrX:3123001-3323000

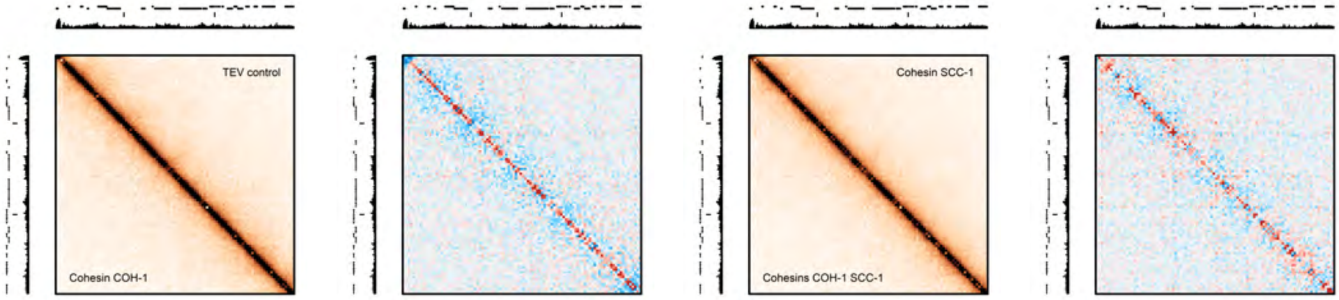

chrX:14879001-15079000

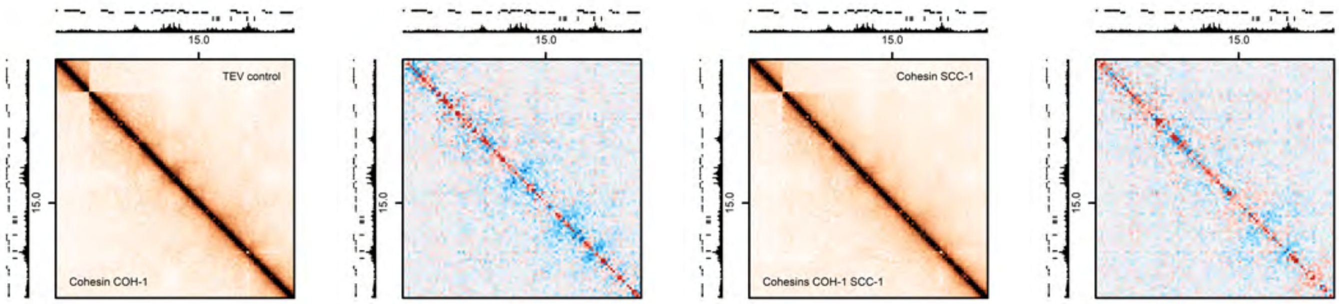

chrX:7033001-7233000

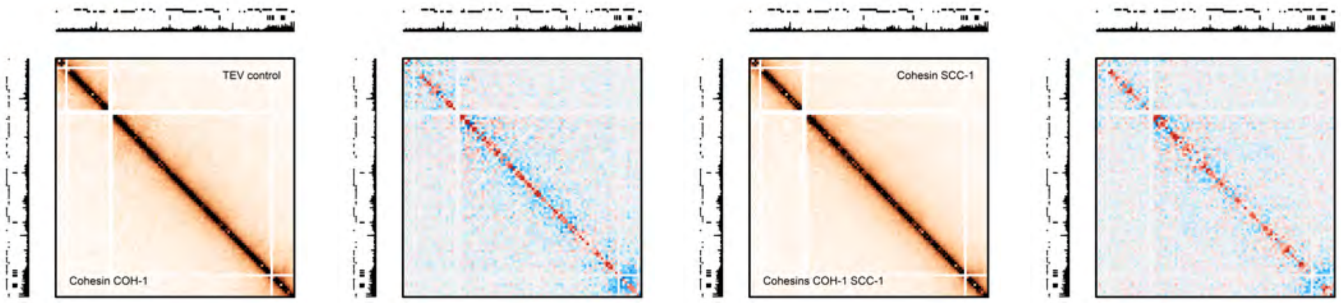

chrI:4553001-4753000

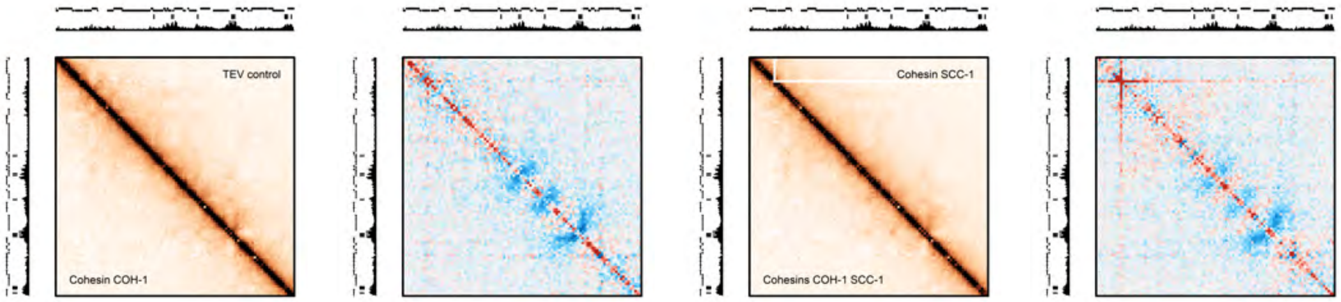

chrX:13615001-13815000

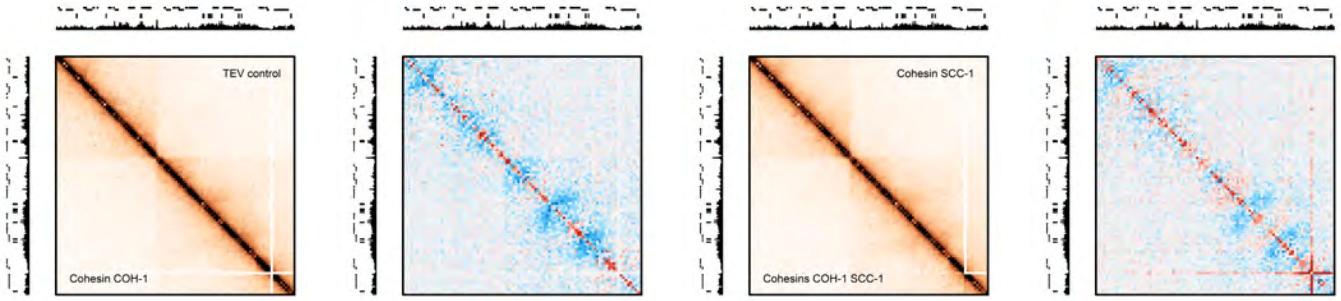

chrX:5595001-5795000

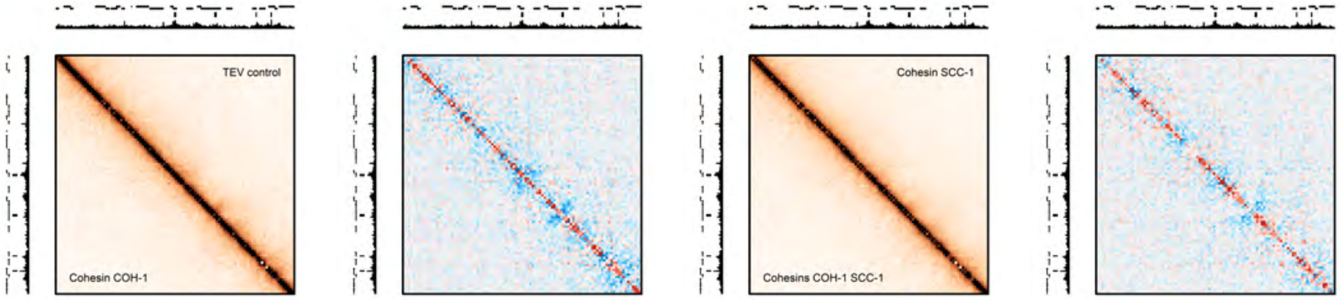

chrI:1773001-1973000

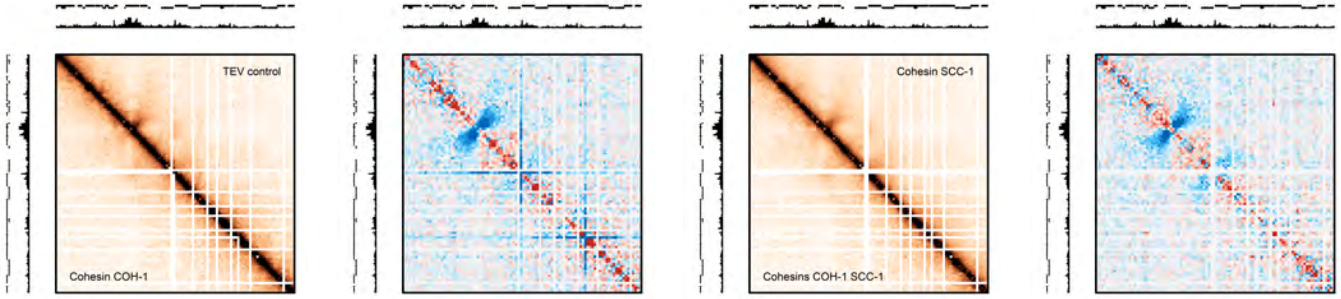

Supplement: Supplementary file 5 — Supplementary Data 3 [file 41467_2025_67302_MOESM5_ESM.pdf]
